# Systems-wide RNAi analysis of *CASP8AP2*/*FLASH *shows transcriptional deregulation of the replication-dependent histone genes and extensive effects on the transcriptome of colorectal cancer cells

**DOI:** 10.1186/1476-4598-11-1

**Published:** 2012-01-04

**Authors:** Amanda B Hummon, Jason J Pitt, Jordi Camps, Georg Emons, Susan B Skube, Konrad Huppi, Tamara L Jones, Tim Beissbarth, Frank Kramer, Marian Grade, Michael J Difilippantonio, Thomas Ried, Natasha J Caplen

**Affiliations:** 1 Cancer Genomics Section, Genetics Branch, Center for Cancer Research, National Cancer Institute, NIH, Bethesda, MD, USA; 2 Gene Silencing Section, Genetics Branch, Center for Cancer Research, National Cancer Institute, NIH, Bethesda, MD, USA; 3 Department of Chemistry and Biochemistry, University of Notre Dame, Notre Dame, IN, USA; 4 Department of Medical Statistics, University Medical Center Göttingen, Göttingen, Germany; 5 Department of General and Visceral Surgery, University Medical Center Göttingen, Göttingen, Germany; 6 Division of Cancer Treatment and Diagnosis, National Cancer Institute, NIH, Bethesda, MD, USA

**Keywords:** *CASP8AP2*, *FLASH*, RNAi screening, RNAi analysis, siRNA, replication-dependent histone transcripts

## Abstract

**Background:**

Colorectal carcinomas (CRC) carry massive genetic and transcriptional alterations that influence multiple cellular pathways. The study of proteins whose loss-of-function (LOF) alters the growth of CRC cells can be used to further understand the cellular processes cancer cells depend upon for survival.

**Results:**

A small-scale RNAi screen of ~400 genes conducted in SW480 CRC cells identified several candidate genes as required for the viability of CRC cells, most prominently *CASP8AP2*/*FLASH*. To understand the function of this gene in maintaining the viability of CRC cells in an unbiased manner, we generated gene specific expression profiles following RNAi. Silencing of *CASP8AP2*/*FLASH *resulted in altered expression of over 2500 genes enriched for genes associated with cellular growth and proliferation. Loss of CASP8AP2/FLASH function was significantly associated with altered transcription of the genes encoding the replication-dependent histone proteins as a result of the expression of the non-canonical polyA variants of these transcripts. Silencing of *CASP8AP2*/*FLASH *also mediated enrichment of changes in the expression of targets of the NFκB and MYC transcription factors. These findings were confirmed by whole transcriptome analysis of *CASP8AP2*/*FLASH *silenced cells at multiple time points. Finally, we identified and validated that CASP8AP2/FLASH LOF increases the expression of neurofilament heavy polypeptide (NEFH), a protein recently linked to regulation of the AKT1/ß-catenin pathway.

**Conclusions:**

We have used unbiased RNAi based approaches to identify and characterize the function of CASP8AP2/FLASH, a protein not previously reported as required for cell survival. This study further defines the role CASP8AP2/FLASH plays in the regulating expression of the replication-dependent histones and shows that its LOF results in broad and reproducible effects on the transcriptome of colorectal cancer cells including the induction of expression of the recently described tumor suppressor gene *NEFH*.

## Background

Cancer cells are characterized by changes in proteins that favor cell survival and proliferation, including down-regulation or de-activation of pro-apoptotic factors and cell cycle regulators, and up-regulation or activation of anti-apoptotic factors including kinases and growth factors. Targeting of specific proteins to overcome or bypass this suppression of cell death and enhancement of proliferation is a major approach for the development of anti-cancer therapies. Like all cancers, colorectal cancer is marked by genomic aberrations and transcriptional deregulation that affect multiple cellular pathways [1]. The characterization of proteins whose function alters the underlying molecular features of CRC has the potential to identify new therapeutic strategies for CRC. Gene-specific loss-of-function (LOF) analysis, through the application of RNA interference (RNAi) based technologies, is increasingly being used to probe the role of a particular protein in a specific cellular context [2].

We have recently used RNAi based LOF approaches to validate the functional dependence of colorectal cancer cells on genes identified as over-expressed in CRC [3]. Loss-of-function analysis via RNAi can also be used to identify proteins required for the survival of CRC cells that show no significant genomic or transcriptional changes. Alterations in apoptosis and related survival mechanisms contribute to both the development of CRC, and the response to treatment [4]. For example, colorectal tumors often show increased expression of members of the anti-apoptotic BCL family including BCL2, mutations in the tumor suppressor TP53, and defects in several pathways related to inflammation including the COX2, TGF-ß, and NFκB pathways. It is likely that many less well-characterized proteins related to cell survival alter the growth of CRC cells. Identification of such genes could though give further insight into the molecular changes underlying CRC, and thus the development of new treatment strategies. To assess the feasibility of identifying proteins whose function has not previously been identified as essential for the survival of colorectal cancer cells we conducted a small-scale RNAi screen of ~400 genes in CRC cells. The gene targets were focused on proteins associated with cell survival, with an emphasis on regulators, and effectors of apoptosis. One of the candidate genes most prominently required for the survival of CRC cells was the gene encoding Caspase-8-Associated Protein 2 or FLICE-associated Huge Protein (*CASP8AP2*/*FLASH*). Subsequent whole transcriptome profiling showed that CASP8AP2/FLASH LOF has wide-ranging, and specific, effects on gene expression including deregulated expression of the replication-dependent histone genes.

## Methods

### Cell Culture and siRNA transfections

SW480 (an aneuploid, mismatch-repair proficient, colon adenocarcinoma cell line), SW837 (an aneuploid, mismatch repair proficient, rectal adenocarcinoma cell line), and SW48 (a mismatch-repair deficient, diploid, colon adenocarcinoma cell line) cells were obtained from ATCC (Manassas, VA) and were maintained in RPMI (Invitrogen, Carlsbad, CA) containing 10% fetal bovine serum (FBS, Invitrogen), supplemented with L-Glutamine and penicillin/streptomycin, at 37°C in a humidified atmosphere containing 5% CO_2_. Cells were passaged every four to five days. The choice of SW480 as the cell line for the RNAi screen was based on characteristics of the spectral karyotype that recapitulate chromosomal aberrations commonly observed in colorectal cancer [3]. The synthetic siRNA based RNAi screen was performed using the Human Apoptosis Set Library (Qiagen, Valencia, CA) arrayed in a total of eleven 96 well plates, one siRNA per well. Gene targets were selected based on searches of the PANTHER (Protein ANalysis THrough Evolutionary Relationships) Classification System, the Gene Ontology database, and PubMed resources (E. Lader, Qiagen, Personnel communication). See Additional file 1, Table S1 for list of genes targeted and siRNA target sequences. Genes targeted included known regulators and effectors of apoptosis, proteins with less defined functions in cell survival and apoptosis and a few proteins with no known direct function linked to apoptosis so all functional groups were represented. We have previously determined successful transfection conditions of siRNAs into SW480 cells using the Oligofectamine transfection reagent (Invitrogen) [3]. We confirmed these conditions for this study by examining the silencing of the *CTNNB1 *gene at an mRNA level (Additional file 2, Figure S1A) and the viability of SW480 cells following silencing of Polo-like kinase 1 (PLK1) (Additional file 2, Figure S1B). The RNAi screen was conducted as follows; transfections were performed by pre-complexing siRNA (2 pmol) with 0.6 μl Oligofectamine lipid transfection reagent (Invitrogen) in 50 μL of serum free RPMI in individual plate wells for 30 min at ambient temperature. Next, SW480 cells (7,000) were added in 50 μL RPMI supplemented with 20% FBS to yield a final concentration of 20 nM siRNA in RPMI, 10% FBS. This final mixture was incubated at ambient temperature for 1 hour before being placed at 37°C in a humidified atmosphere containing 5% CO_2_. After 72 hours cell viability was assayed (Cell Titer Blue Reagent, Promega, Madison, WI). As this was a relatively small-scale siRNA screen, the viability of SW480 cells following siRNA transfection was expressed relative to the average viability for cells transfected with a negative control siRNA - AllStar Negative Control siRNA (siNeg) (Qiagen Inc.) (n = 33) (Additional file 2, Figure S1C). A siRNA corresponding to PLK1 was used as a positive control on every plate (see Additional file 3, Table S2 for sequence); on average this induced a decrease in the viability of SW480 cells of greater than 85% (n = 11) (Additional file 2, Figure S1C). For gene specific analysis SW480, SW48, and SW837 cells were transfected in 96 well plates as above using 15,000 SW48 cells, 10,000 SW837 cells, and 5,000 SW480 cells per well. See Additional file 3, Table S2 for the sequences of gene specific siRNAs. Transfections for whole transcriptome analysis were performed in triplicate in 6-well plates, using 2.1 × 10^5 ^SW480 cells and 1.8 μL Oligofectamine per well.

### RNA assays

#### Quantitative reverse transcription-PCR

Total RNA was isolated from cells using the RNeasy Mini kit (Qiagen) and mRNA reverse transcribed into cDNA using random hexamer primers or oligo dT and reverse transcriptase (Invitrogen SuperScriptIII). Gene expression levels for *CASP8AP2*/*FLASH *and NUP62 were assayed by quantitative reverse transcription-PCR (qRT-PCR) using Power SYBR Green technology (Applied Biosystems, Inc., Foster City, CA) and were normalized to the expression of *YWHAZ*. For each RT-PCR reaction, 300 ng cDNA was used. PCR was performed using default variables of the Applied Biosystems' Prism 7000 sequence detector, except for a total reaction volume of 25 μL. Primers were obtained from Operon Technologies, Inc. (Huntsville, AL) or Invitrogen. Gene expression levels for *HIST1H2BD *were determined by oligo-dT or random hexamer primed synthesis of cDNA (Invitrogen), and real-time PCR amplification in i-cycler (Bio-Rad) based conditions with SYBR green. *HIST1H2BD *expression levels were normalized to the expression of ß-actin. See Additional file 4, Table S3 for all primer sequences.

#### Branched DNA assay

Quantigene Probes (Panomics, Fremont, CA) corresponding to *CASP8AP2/FLASH *were used to directly measure gene specific mRNA levels (normalized to human cyclophilin B (PPIB) expression) in cell lysates as previously described [5].

#### Whole transcriptome analysis

RNA samples were quantified and assessed for quality on a Bioanalyzer (Agilent, Santa Clara, CA) and only samples with an RIN value > 8 were hybridized. For microarray analysis, 700 ng RNA was converted to cRNA using an oligo dT primer, labeled with Cy3, and subjected to mono-channel hybridization onto a 4 × 44K Whole Human Genome Microarray per the manufacturers instructions (Agilent). Microarrays were washed and processed using an Agilent G2565BA scanner. Data were quality controlled and extracted using Agilent Technologies' Feature Extraction (version 9.1). Microarray expression data are available at the Gene Expression Omnibus (GEO) accession GSE29405 and are in accordance with MIAME guidelines.

#### Tumor data: Array CGH and mRNA

The array comparative genomic hybridization (CGH) and transcriptome analysis of primary colon adenocarcinoma tumors and cell lines has been previously described [6,7].

### Caspase 8 and Caspase 3/7 assays

The activation of Caspase 8 and Caspase 3/7 were measured using an Ac-LETD-pNA caspase-8 substrate (Caspase-Glo 8 Assay, Promega) and a DEVD peptide substrate (Caspase-Glo 3/7 Assay, Promega) respectively, following the manufacturers instructions. Plates were measured with a Victor luminometer (Perkin Elmer) at 60 min after addition of the Caspase 8 reagents and 90 min after the addition of the Caspase 3/7 reagents.

### Western blot analysis

Transfections for Western blot analyses were performed in 6-well plates, using 1.8 × 10^5 ^SW480 cells and 10.5 μL RNAiMax per well. Whole cell lysates were harvested from SW480 cells 72 hours post transfection with siCASP8AP2.3 and siCASP8AP2.6 according to the Complete Lysis-M Reagent Kit (Roche, Indianapolis, IN). Reduction of *CASP8AP2*/*FLASH *mRNA was assessed by RT-PCR from replicate samples. Soluble protein fractions were run on a 10% SDS-PAGE gel and transferred to nitrocellulose membrane for 1 hour at 12 V with an additional 30 minutes at 24 V. The membrane was incubated with α-actin clone C4 (1:1000 dilution) (Millipore, Temecula, CA) and α-200 kD Neurofilament Heavy - Neuronal Marker (1:1000 dilution) (Abcam, Cambridge, MA) in 10% milk buffer in 1× PBS on a rocking platform overnight at 4 degrees. The membrane was washed 3 times for 10 minutes in 10% milk buffer in 1× PBS and incubated with HRP α-mouse (1:10,000 dilution) (Jackson ImmunoResearch, West Grove, PA) for 1 hour on a rocking platform at room temperature. The membrane was washed as before, rinsed with DI water and incubated with SuperSignal West Pico Chemiluminescent Substrate (Pierce/Thermo Fisher Scientific Rockford, IL) according the protocol. Kodak BioMax Light Film (Carestream Health, Woodbridge, CT) was used to expose the membrane for varying amounts of time.

### Bioinformatic and statistical analysis

Standard statistical analyses were conducted in Excel. Comparisons of functional effects of gene silencing were assessed using the t-test (unequal variance); a p value of ≤0.05 was considered significant. Comparison of the fold change in different gene expression data sets was assessed using a Pearson's correlation.

#### Gene expression microarray data

Gene expression array signal intensities were Log_2 _transformed and quantile normalized using the R statistical computing software and the package *Limma*. Differential expression between groups was assessed using an empirical Bayes method for the moderated T-statistics; all probes were corrected for multiple testing using Benjamini and Hochberg's *false discovery rate *(FDR) [8-11]. In order to be considered differentially expressed, probes had to have an average Log_2 _fold change of ± 0.6 (approximately 1.5 fold change on a linear scale) and a q-value (FDR) < 0.05. In addition, probes needed to show this significant fold change in the same direction for both siRNAs targeting CASP8AP2/FLASH in the initial analysis and for all three siRNAs in the follow up study.

The time course study also included a comparison of siNeg transfected cells and untransfected cells. Any probes showing a significant differential expression (q-value < 0.05) in the siNeg cells versus untransfected SW480 cells were filtered from data used for analysis of gene ontologies, pathway analysis and gene set enrichment analysis. Of the ~44,000 probes examined this resulted in the removal of just 124 probes from the 24-hour data set, 820 probes at 48 hours, and ~1500 probes at 72 hours.

To determine if any genes exhibiting decreased expression represented interactions between the transfected siRNA and non-targeted transcripts, we assessed the alignment of each siRNA sequence with the 3'UTR sequence of all of the downregulated genes obtained following transfection of each siRNA. The open-access miRanda application http://www.microrna.org was used to perform alignment predictions between each siRNA sequence and the 3'UTR sequence for all genes that showed decreased expression with each siRNA. We then examined in detail the non-targeted genes that were downregulated by two or more siRNAs that showed a potential miRNA-like mismatch alignment with a sequence within the 3'UTR of the non-targeted gene.

One-way hierarchical clustering (average linkage) of the gene profiles was conducted on the expression data using JMP 8.0 (SAS, Cary, NC).

#### Gene expression microarray data analysis: Histone genes

The sequences of the 60 nucleotide probes (Agilent) annotated as corresponding to 79 human histone genes were all reassessed for alignment to the current RNA reference sequence (refseq_rna) database using nBLAST (Megablast, optimized for highly similar sequences) to confirm specificity and annotation. As described in Gertz et al., we confirmed high quality alignments between a probe and a gene as requiring a BLAST score of at least 100 bits [12]. Most probes corresponded to unique histone transcripts and returned scores of 111 bits (the maximum score). Where a probe alignment of 111 bits was returned for more than one gene the annotation was altered to reflect both loci. Probe alignments to genes other than the principal target gene that returned scores of between 100 and 110 bits are noted in Table 1. Probes that did not align to a current RNA reference sequence, but were associated with a significant fold changes in expression, were also aligned against the entire NCBI nucleotide collection; the cDNA clones corresponding to these probes are noted in Table 1. Data corresponding to probes sequences that aligned to transcripts now annotated as a pseudogene were excluded.

**Table 1:** Histone transcripts show altered levels following silencing of *CASP8AP2*/*FLASH*

Ref Seq	Gene Symbol	Chromosomal position	Agilent Probe	CASP8AP2/FLASH SILENCED SW480 cells				Published HeLa data	
				
				Fold change (Log_2_) siCASP8AP2.3	q values siCASP8AP2.3	Fold change (Log_2_) siCASP8AP2.6	q values siCASP8AP2.6	Narita et al	Shepard et al PAS seq^#^
NM_012115	CASP8AP2	chr6:90640368-90640427	A_23_P58898	***-2.06***	6.28E-15	**-1.81**	6.14E-13	-	-

									

NM_003548	HIST2H4A/ HIST2H4B	chr1:146617469-146617528	A_23_P436281	***3.01***	1.21E-20	**2.61**	4.73E-18		chr1 - 148098952-1

NM_003516	HIST2H2AA3/	chr1:146635701-146635760	A_23_P309381	***3.80***	9.00E-09	**4.24**	4.19E-10	Yes*	

	HIST2H2AA4								

NM_003516	HIST2H2AA3/**^1^**	chr1:146636178-146636234	A_23_P103981	***3.83***	3.55E-11	**3.72**	8.65E-11		

	HIST2H2AA4								

NM_003528	HIST2H2BE	chr1:146669546-146669487	A_24_P148321	***3.78***	5.33E-13	**3.63**	1.93E-12		chr1-148122634-6, chr-148124269-1, chr1-148124357-1

NM_003528	HIST2H2BE	chr1:146670196-146670137	A_23_P149545	***2.24***	8.74E-14	**2.39**	7.62E-15		

NM_003528	HIST2H2BE^**2**^	chr1:146671162-146671103	A_24_P156911	***3.52***	6.07E-19	**3.49**	7.82E-19		

NM_003517	HIST2H2AC	chr1:146671911-146671970	A_24_P8721	***3.17***	3.73E-16	**2.97**	3.91E-15		chr1-148125568-4

NM_003517	HIST2H2AC	chr1:146671936-146671995	A_23_P301247	***-1.90***	8.19E-10	**-2.02**	1.36E-10		

NM_175065	HIST2H2AB	chr1:146672317-146672258	A_24_P68631	***3.88***	1.18E-17	**3.77**	3.43E-17		

NM_033445	HIST3H2A	chr1:224952044-224951985	A_23_P149301	***3.10***	1.82E-16	**3.17**	7.19E-17		

NM_175055	HIST3H2BB	chr1:224952760-224952819	A_23_P332992	***3.50***	2.92E-16	**3.42**	6.04E-16		

									

NM_003537	HIST1H3B	chr6:26139947-26139888	A_24_P174924	***1.57***	9.00E-06	**1.12**	1.50E-03		

NM_003537	HIST1H3B	chr6:26139960-26139901	A_23_P93258	***3.48***	1.00E-13	**3.31**	5.69E-13		

NM_003513	HIST1H2AB	chr6:26141499-26141440	A_24_P223384	***1.93***	3.63E-10	**1.95**	2.41E-10		

NM_021062	HIST1H2BB	chr6:26151544-26151485	A_23_P111054	***3.14***	2.36E-15	**2.93**	2.72E-14		

NM_005319	HIST1H1C	chr6:26164088-26164029	A_23_P122443	***3.33***	1.27E-13	**3.40**	5.92E-14	Yes	

NM_003526	HIST1H2BC	chr6:26232021-26231962	A_23_P93180	***3.36***	5.81E-17	**3.30**	1.03E-16		

NM_005321	HIST1H1E	chr6:26264907-26264966	A_23_P7976	***3.35***	1.45E-13	**3.15**	1.18E-12		chr6-26265322-1

NM_021063^**3**^	HIST1H2BD	chr6:26266453-26266512	A_24_P146211	***3.87***	1.47E-19	**3.77**	4.38E-19	Yes	ch6-26279217-1, ch6-26279551-20, chr6-26266834-7

NM_003523	HIST1H2BE	chr6:26292319-26292378	A_23_P30776	***3.42***	2.47E-16	**3.44**	1.72E-16	Yes	

NM_003530	HIST1H3D	chr6:26305256-26305197	A_23_P219045	***3.82***	1.30E-14	**3.83**	1.10E-14		

NM_003530	HIST1H3D	chr6:26305296-26305237	A_24_P217834	***2.99***	3.58E-13	**3.09**	1.01E-13		

NM_021065	HIST1H2AD^**4**^	chr6:26307121-26307062	A_23_P428184	***4.23***	6.78E-16	**4.21**	6.99E-16		

NM_003522	HIST1H2BF	chr6:26308082-26308141	A_23_P42178	***3.44***	3.50E-16	**3.41**	4.47E-16	Yes	

NM_003518	HIST1H2BG	chr6:26324755-26324696	A_23_P167997	***3.93***	1.29E-20	**3.78**	6.51E-20		chr6-26324393-2

NM_021052	HIST1H2AE	chr6:26325533-26325592	A_23_P59045	***4.32***	9.87E-24	**3.90**	7.90E-22	Yes	chr6-26325683-13

NM_005320	HIST1H1D	chr6:26342737-26342678	A_24_P260639	***3.59***	1.39E-13	**3.22**	5.45E-12		

NM_003540	HIST1H4F	chr6:26348797-26348856	A_23_P359540	***2.78***	1.14E-16	**2.44**	1.42E-14		

NM_021018	HIST1H3F	chr6:26358626-26358567	A_23_P30799	***3.36***	2.65E-14	**3.15**	2.41E-13		chr6-26358379-1

NM_003524	HIST1H2BH	chr6:26360201-26360260	A_23_P366216	***3.19***	4.08E-16	**3.04**	2.19E-15	Yes	chr6-26360284-3

BC012185^**5**^	HIST1H3G	chr6:26377826-26377767	A_32_P794894	***3.07***	7.43E-30	**3.54**	8.23E-33		

NM_003534	HIST1H3G	chr6:26379448-26379389	A_23_P42198	***1.15***	1.39E-04	**1.27**	3.34E-05		

NM_003525	HIST1H2BI	chr6:26381354-26381413	A_23_P111041	***3.51***	7.27E-15	**3.69**	1.12E-15	Yes	chr6-26381629-2

NM_003543	HIST1H4H	chr6:26393535-26393476	A_23_P323685	***4.03***	1.13E-21	**3.97**	2.14E-21		

NM_021058	HIST1H2BJ	chr6:27208356-27208297	A_24_P55148	***3.81***	3.69E-20	**3.81**	3.41E-20		

NM_021064	HIST1H2AG	chr6:27209136-27209195	A_24_P303354	***1.30***	2.26E-10	**1.08**	3.92E-08		chr6-27209294-1 ch6-27210796-2 chr6-272111049-7

BC016677^**6**^	HIST1H2AG	chr6:27210269-27210328	A_24_P414658	***4.19***	9.07E-25	**4.05**	4.31E-24		

NM_003495	HIST1H4I	chr6:27215204-27215263	A_24_P20873	***1.75***	1.69E-06	**2.63**	4.38E-11		

NM_080593	HIST1H2BK	chr6:27221956-27221897	A_23_P145238	***2.96***	4.33E-10	**3.34**	1.12E-11	Yes	

NM_080596	HIST1H2AH	chr6:27223225-27223284	A_23_P81859	***1.67***	1.41E-12	**1.51**	3.81E-11		

NM_003519	HIST1H2BL	chr6:27883324-27883265	A_23_P8013	***3.25***	1.72E-14	**3.10**	8.91E-14		chr6-27883235-1

NM_003536	HIST1H3H	chr6:27886003-27886062	A_23_P333484	***4.52***	3.48E-25	**4.05**	4.51E-23	Yes	

NM_021066	HIST1H2AJ/**^7^** HIST1H2AI	chr6:27890225-27890166	A_23_P168014	***2.30***	6.42E-10	**2.11**	6.71E-09		chr6-27890034-2

NM_021066	HIST1H2AJ/ HIST1H2AI	chr6:27890432-27890373	A_24_P394510	***2.44***	5.52E-08	**2.34**	1.62E-07		

NM_003521	HIST1H2BM	chr6:27890982-27891041	A_24_P3783	***3.29***	2.61E-14	**3.39**	8.13E-15		

NM_003510	HIST1H2AK	chr6:27913767-27913708	A_24_P217848	***1.56***	1.98E-13	**1.48**	1.27E-12		

NM_003520	HIST1H2BN^**8**^	chr6:27914524-27914583	A_23_P402081	***3.46***	6.89E-19	**3.45**	7.27E-19		chr6-27927954-1

NM_003511	HIST1H2AL	chr6:27941481-27941540	A_23_P363174	***1.73***	2.13E-11	**1.97**	2.31E-13		chr6-27941554-1

NM_005322	HIST1H1B	chr6:27942667-27942608	A_23_P250385	***-1.35***	8.17E-03	**-1.61**	2.22E-03		

NM_003546	HIST1H4L	chr6:27948987-27948928	A_23_P70480	***-1.46***	3.81E-02	**-1.59**	3.28E-02		

NM_003514	HIST1H2AM	chr6:27968545-27968486	A_32_P221799	***4.10***	3.17E-22	**3.58**	8.93E-20		

NM_003514	HIST1H2AM	chr6:27968615-27968556	A_24_P86389	***2.40***	5.41E-15	**2.35**	1.10E-14		

NM_003527	HIST1H2BO	chr6:27969567-27969626	A_23_P59069	***3.25***	1.97E-17	**3.23**	2.20E-17		

									

^**9**^BC020884	HIST4H4	chr12:14813388-14813329	A_24_P261691	***1.14***	9.21E-09	**1.68**	5.34E-14		

									

NM_002105	H2AFX	chr11:118469860-118469801	A_24_P38895	***0.92***	8.28E-06	**0.82**	6.13E-05		

									

NM_177925	H2AFJ	chr12:14818894-14818953	A_24_P236003	***1.08***	1.95E-12	**0.91**	4.53E-10		

NM_177925	H2AFJ	chr12:14819082-14819141	A_23_P204277	***0.69***	3.92E-06	**0.80**	1.51E-07		

**^1^**Both scores of 106/111 bits, **^2^**HIST2H2BC - score of 100/111 bits, **^3^**Also aligns to NM_138720: HIST1H2BD transcript variant 2, includes polyA, **^4^**HIST1H3D - score of 104/111 bits, **^5^**BC012185/HIST1H3G cDNA clone IMAGE:4563941, includes polyA, **^6^**BC016677/HIST1H2AG cDNA clone IMAGE 4342191, includes poly,**^7^**Both scores of 110/111 bits, **^8^**HIST1H2BL, HIST1H2BK, HIST2H2BF, HIST2H2BF, HIST1H2BI, score of 106 to 100 bits, **^9^**BC020884/HIST4H4 cDNA clone IMAGE:4619662, includes polyA, *annotated as HIST2HAA, **^#^**Genome location-read count

#### Ingenuity Pathway Analysis and Gene Set Enrichment Analysis (GSEA)

Differentially expressed genes (see criteria above) were analyzed through the use of IPA (Ingenuity^® ^Systems, http://www.ingenuity.com). Each dataset was divided into up- and downregulated genes respectively, and then was subjected to gene function (gene ontology) analysis. Enrichment of functional groups within gene sets was calculated using a one-tailed Fisher's exact test. Functional groups with a Benjamini-Hochberg (B-H) corrected p-value of < 0.05 were considered significant. Knowledge-based gene networks were also generated using IPA tools. A maximum network size of 70 molecules was used. In order to condense datasets for Gene Set Enrichment Analysis [13], the median value of each probe (using all siRNA transfections) was normalized to the median value of the same probe in the mock transfections (siNeg). When two or more probes mapped to a single gene, the median normalized expression value was taken so that each gene mapped to a single expression value. GSEA was run using default parameters http://www.broadinstitute.org/gsea/index.jsp. The resulting landscape plots were analyzed for peaks in the tails of the ranked gene lists. FDR q-values < 0.05 were considered significant.

#### Transcription factor target gene datasets

Transcription factor target gene lists were derived from ChIP-Chip, ChiP-PET, or ChIP-seq data curated from peer-reviewed literature (Additional file 5, Table S4). In certain instances, other biological assays and techniques such as gene expression and quantitative ChIP were used in conjunction with ChIP-Chip, ChIP-PET, or ChIP-Seq in order to derive higher-confidence lists. NFκB target genes were taken from http://www.bu.edu/nf-kb/gene-resources/target-genes/ (complied December 2009), which is a compilation of downstream NFkB targets reported in peer-reviewed literature. All gene lists were cross-referenced to the Ingenuity Knowledge Base as well as NCBI Entrez Gene. Genes that could not be mapped to these databases by their published or associated identifiers were excluded.

## Results and Discussion

### RNAi screening in the colorectal cancer cell line SW480 identifies *CASP8AP2/FLASH *as required for cell viability

**Figure 1:**
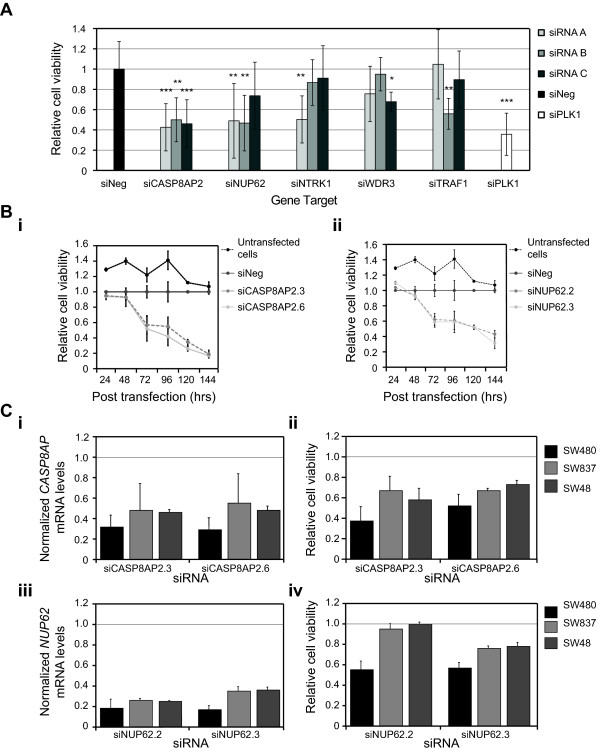
**RNAi identifies *CASP8AP2*/*FLASH *and *NUP62 *as essential for the viability of SW480 cells**. **(A) **The relative viability of SW480 cells following transfection of three different siRNAs targeting each of the genes shown (see Methods for the identity of each siRNA). Data is shown as the mean ± SD of three independent transfections for each siRNA targeting the six genes of interest, normalized to the average viability of SW480 cells transfected with the negative control siRNA (siNeg; eight independent transfections). The normalized data for the positive control siRNA, siPLK1, (six independent transfections) is also shown. The statistical comparison (t-test of unequal variance) of siNeg transfected cells to cells transfected with the siRNAs corresponding to the stated genes is indicated by *** = p ≤ 0.001, ** = p ≤0.01, and * = p ≤0.05. **(B) **The effect of silencing (i) *CASP8AP2/FLASH *and (ii) *NUP62*, over 144 hours, on cell viability and their respective mRNA levels in SW480. The data shown represents the mean and standard deviation for three transfections per condition per time point normalized to the data from cells transfected with the negative control siRNA, siNeg. **(C) **The effect of silencing *CASP8AP2/FLASH *and *NUP62 *on their respective mRNA levels (i and iii) and on cell viability (ii and iv) in three SW480, SW837 and SW48 colorectal cancer cell lines. The data shown represents the mean and standard deviation for three transfections per condition per time point normalized to the data from cells transfected with the negative control siRNA, siNeg.

A siRNA based RNAi screen of 405 genes (two siRNAs/gene), most with annotated functions that include a role related to apoptosis, was conducted in the colorectal cancer (CRC) cell line SW480 (see Additional file 2, Figure S1D for a summary of the screen and Additional file 1, Table S1 for details of genes targeted and the relative cell viability of SW480 cells following RNAi normalized to siNegative control transfected cells). The aim of this screen was to identify genes not previously recognized as required for the survival of CRC cells. The two siRNAs corresponding to 45 genes both induced at least a 25% decrease in cell viability 72 hours post siRNA transfection (Additional file 2, Figure S1E). Of these 45 genes we selected 15 genes that represented a range of reduced viability seen in the initial screen for confirmation using the same siRNAs but with transfections conducted in triplicate (Additional file 2, Figure S1F). These 15 genes included *BIRC5 *(Survivin), silencing of which would be expected to induce a significant effect on the growth of SW480 cells [14] and proliferating cell nuclear antigen (PCNA), an essential protein required for DNA replication. Of the 15 genes followed up here, both siRNAs corresponding to five genes, *CASP8AP2/FLASH, NUP62, NTRK1, WDR3*, and *TRAF1 *reproducibly reduced cell viability by over 50%, levels either comparable or more than that seen following silencing of *BIRC5/*Survivin and PCNA (Additional file 2, Figure S1F). At least one further siRNA corresponding to each of these fives genes was then assessed to confirm the phenotypic effects seen with the siRNAs used in the initial screen (Figure 1A). The silencing of *CASP8AP2/FLASH *and *NUP62 *showing the most consistent effect; an average of 60% reduction in viability was observed in *CASP8AP2*/*FLASH *silenced cells, and an average of 50% reduction in viability was seen in *NUP62 *silenced cells. The numerical values associated with the relative viability of the screens fluctuated slightly, reflecting natural variation in the biological replicate populations of cells and other subtle experimental factors, however, in every screen conducted, the reduction of CASP8AP2/FLASH consistently produced a substantial reduction in viability. The effect of silencing *CASP8AP2*/*FLASH *and *NUP62 *on cell viability was further enhanced when viability was assayed up to 144 hours post siRNA transfection (Figure 1B). We confirmed the silencing of *CASP8AP2/FLASH *and *NUP62 *RNA following RNAi by qRT-PCR (Figure 1C and 1D) and a reduction in viability in two further CRC cell lines. Both siRNAs corresponding to *CASP8AP2/FLASH *reduced the viability of SW837 and SW48 cells, though only one siRNA corresponding to NUP62 induced a reduction in the viability of SW837 and SW48 cells (Figure 1C and 1D).

As the silencing of *CASP8AP2/FLASH *showed the most consistent effect on the survival of CRC cells and has not been studied previously in the context of colorectal cancer we chose to examine this gene in further detail. CASP8AP2/FLASH was initially discovered as involved in the binding of the FAS-associated adaptor protein (FADD), and procaspase-8 [15]. Ligand binding of FAS to the FAS cell surface receptor relays the extrinsic apoptotic death signal through recruitment of FADD, procaspase-8 activation, and formation of the death-inducing signaling complex (DISC). A number of studies have supported the interaction of CASP8AP2/FLASH with FADD and as a regulator for activation of CASPASE 8 [16] and thus apoptosis [17]. However, other studies have challenged the pro-apoptotic role of CASP8AP2/FLASH [18] and there is increasing evidence that the large *CASP8AP2*/*FLASH *protein (222 KDa) has multiple functions. A previous RNAi screen conducted in HeLa cells identified CASP8AP2/FLASH as essential for cell division [19] and other studies have linked it to NFκB signaling [20,21], activation of MYB [22,23], S-phase progression [24-26], and an involvement in histone biology. Evidence for the involvement of CASP8AP2/FLASH in histone biology includes its cellular location in nuclear organelles found adjacent to histone genes and biochemical studies linking it to histone transcript processing [24,27-32].

### Molecular characteristics of *CASP8AP2/FLASH *in colorectal cancer

**Figure 2:**
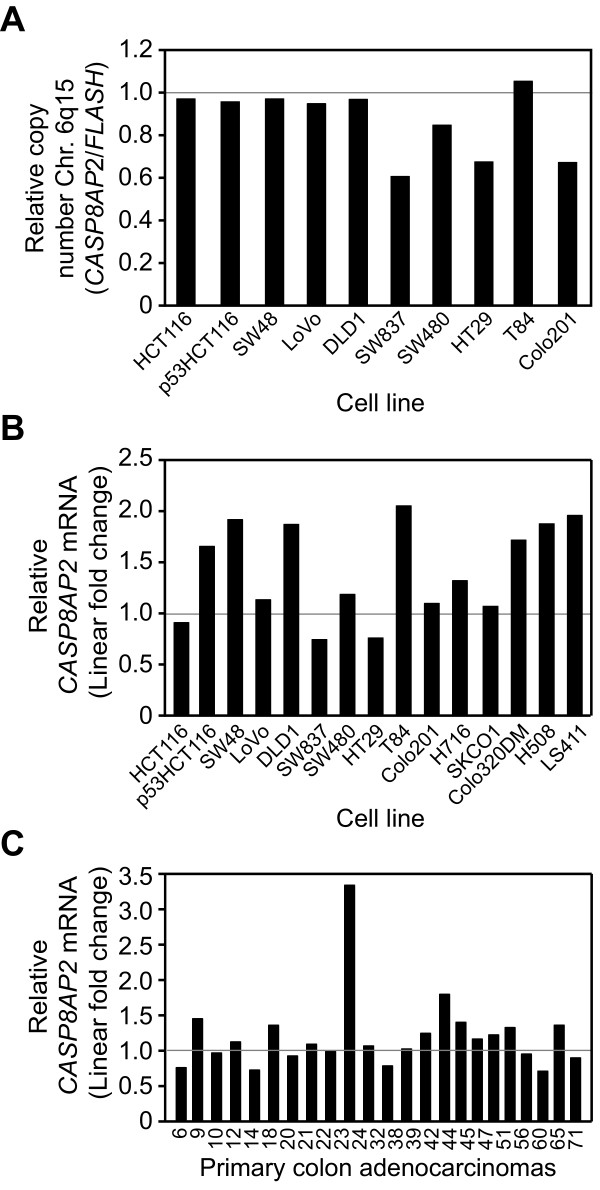
**Molecular genomic characteristics of *CASP8AP2*/*FLASH *in colorectal cancer cells and tumors**. (**A**) The relative copy number of the *CASP8AP2/FLASH *loci and (**B**) the expression of *CASP8AP2/FLASH *mRNA in colorectal cell lines. All data is expressed as a linear fold change from that observed in normal mucosa. (**C**) The expression of *CASP8AP2/FLASH *in 23 primary tumor samples; data is expressed as linear fold change from that observed in normal mucosa.

Specific DNA and RNA based alterations are frequently associated with colorectal cancer. The examination of data obtained from colorectal cancer cell lines and primary tumors showed, however, no overt genomic or expression changes associated with the *CASP8AP2/FLASH *(Figure 2). The Chr. 6q15 region containing *CASP8AP2/FLASH *locus showed a reduction in copy number compared to normal colon mucosa in four out of five microsatellite-stable CRC cell lines (SW837, SW480, HT29, and Colo201) (Figure 2A). Only T84 showed a slight gain and a corresponding increase in mRNA expression compared to normal colon mucosa (~2-fold change) (Figure 2B). *CAPS8AP2*/*FLASH *expression is close to normal expression levels in HCT116 and LoVo cells and slightly increased (1.6 to 1.9-fold change relative to normal colon mucosa) in the other four cell lines (Figure 2B). Gene expression of *CASP8AP2/FLASH *is less than normal colon mucosa (0.7 linear fold change) in HT29 and SW837 cells, close to normal (1.1 to 1.2 linear fold change) in Colo201, SKCO1, and SW480 cells, and higher (> 1.3 linear fold change) in the remaining five cell lines (Figure 2B). CASP8AP2/FLASH protein expression has been shown to be higher in gastric carcinomas compared with normal tissues [33], however, expression of *CASP8AP2/FLASH *in 23 primary colon adenocarcinoma tumors showed levels close to that observed in normal colon mucosa (average linear fold change = 1.103, p = 0.48) (Figure 2C). Only two samples had expression ratios greater than 1.5, of which one had a linear fold change of 3.3 compared to normal mucosa.

### Silencing of *CASP8AP2/FLASH *activates apoptosis associated markers

**Figure 3:**
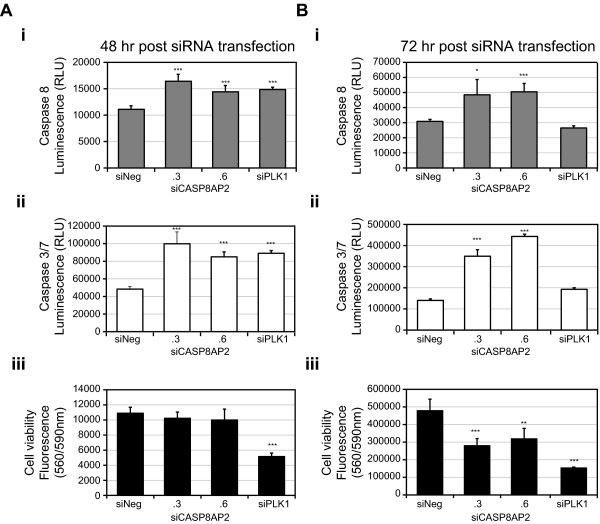
**The silencing of *CASP8AP2*/*FLASH *activates markers of apoptosis**. The effect of silencing *CASP8AP2/FLASH *(two different siRNAs) for (**A**) 48 and (**B**) 72-hours on the levels of (i) activated Caspase 8, (ii) Caspase 3/7 and (iii) cell viability. The viability of cells transfected in parallel is shown for comparison and a siRNA corresponding to *PLK1 *was included as a positive control. Five independent transfections of each siRNA were performed. The statistical comparison (t-test, unequal) of siNeg transfected cells to *CASP8AP2*/*FLASH, NUP62 *or *PLK1 *silenced cells is indicated by *** = p ≤0.001, ** = p ≤0.01, and *. = p ≤0.05. Caspase 8 levels were measured using an Ac-LETD-pNA Caspase-8 substrate (Caspase-Glo 8 Assay, Promega), Caspase 3/7 levels were measured using a DEVD peptide substrate (Caspase-Glo 3/7 Assay, Promega), and cell viability was measured using Cell Titer Blue Reagent, Promega).

To begin to understand the functional processes underlying the decrease in the viability of CRC cells seen following silencing of CASP8AP2/FLASH we first assessed the effect of its LOF had on markers of apoptosis. CASP8AP2/FLASH was originally identified as a pro-apoptotic protein involved in FAS-mediated apoptosis. For example, a previous study showed FAS induced apoptosis was significantly reduced following the silencing of *CASP8AP2/FLASH *in HT1080 cells [34]. We therefore hypothesized that, particularly in the absence of activation of FAS, silencing *CASP8AP2/FLASH *would have minimal effect on established markers of the apoptosis cascade. However, in SW480 cells, 48 hours following initiation of silencing of *CASP8AP2*/*FLASH*, activation of Caspase 8 was observed (Figure 3Ai; siCASP8AP2.3 p = 0.0002, siCASP8AP.6 p = 0.001); a significant activation of Caspase 8 was also observed 72 hours post siRNA transfection (Figure 3Bi siCASP8AP2.3 p = 0.02, siCASP8AP.6 p = 0.001. In addition, an activation of the downstream caspases, Caspase 3 and 7 was observed at both 48 and 72 hours post-siRNA transfection (Figure 3Aii and 3Bii; 48 hours, siCASP8AP2.3 p = 0.0008, siCASP8AP.6 p = 0.00001, 72 hours, siCASP8AP2.3 p = 0.001, siCASP8AP.6 p ≤0.00001). At 48 hours Caspase 8 and Caspase 3/7 levels were similar to that seen following silencing of a positive control, the Polo like kinase 1 gene, *PLK1*, whose inhibition is well known to activate apoptosis [35] and were greater than that seen in *PLK1 *silenced cells 72 hours post siRNA transfection.

The activation of markers of apoptosis can result from alterations in many different cellular processes. Further, because CASP8AP2/FLASH has been linked to multiple functions we chose to use a systems-wide unbiased approach to investigate the principal downstream effects that perturbation of CASP8AP2/FLASH function has on CRC cells.

### The CASP8AP2/FLASH loss-of-function induces changes in the expression of over two thousands genes

**Figure 4:**
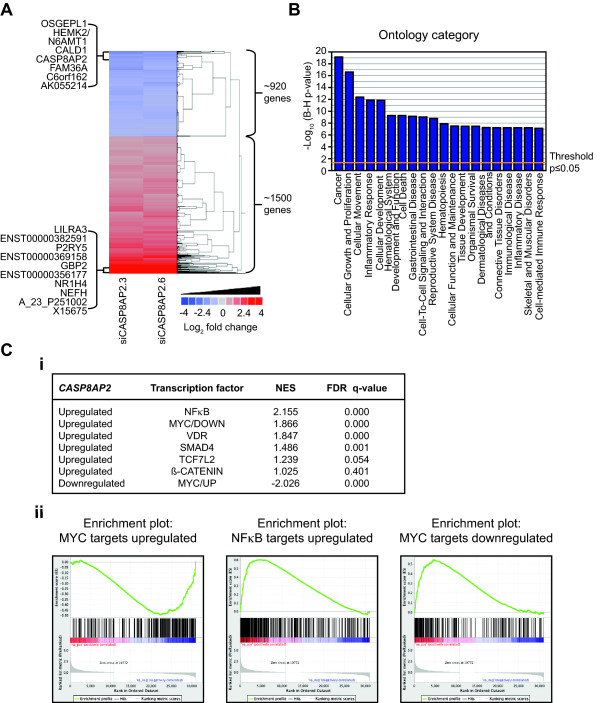
**The *CASP8AP2*/*FLASH *RNAi signature**. **(A) **Heat map representation of those genes showing a significant fold change (> ± 0.6 Log_2 _fold change, p < 0.05) 72 hours post transfection of two different siRNAs corresponding to *CASP8AP2*/*FLASH*. The 10 genes most altered in expression (up and down) following the silencing of *CASP8AP2*/*FLASH *are indicated. Median values are shown where multiple probes corresponding a specific gene were present. The probe corresponding to *CASP8AP2/FLASH *was the fourth most downregulated gene in the *CASP8AP2 *expression profile (ranked by changes mediated by siCASP8AP2.3 and then siCASP8AP2.6). The maximum fold changes in expression seen following the silencing of *CASP8AP2*/*FLASH *ranged from about a 2-fold linear decrease in expression to an over 20-fold increase in expression of over twenty genes. (**B**) The enrichment of functional ontologies for those genes upregulated following silencing of *CASP8AP2*/*FLASH*. No functional ontologies were significantly for those genes downregulated following *CASP8AP2*/*FLASH *loss of function. (**C**) Gene set enrichment analysis (GSEA) of the targets of transcription factors associated with CRC; (**i**) shows the overall statistical analysis for the enrichment of the targets within the *CASP8AP2*/*FLASH*RNAi signature for six transcription factors; NES refers to the normalized enrichment score, (**ii**) shows the enrichment plots for the targets of MYC and NFκB.

To investigate the systems-wide effects of CASP8AP2/FLASH LOF we conducted whole transcriptome microarray analysis of siRNA transfected SW480 cells using two different siRNAs. Differential expression following gene silencing was indicated by probes that showed a fold change in expression of > Log_2 _± 0.6 (corresponding to a linear fold change of ~1.5), with a q-value (FDR) of < 0.05, for both siRNAs targeting *CASP8AP2*/*FLASH*. Overall, there was a high correlation in the fold change seen for those probes showing a significant fold change for both of the siRNAs (siCASP8AP2.3 vs. siCASP8AP2.6 r = 0.97). Only two probes showed a discordant change in the direction of the fold change seen with each siRNA (Additional file 6, Figure S2A), and only one potential off-target interaction within *CASP8AP2/FLASH *the expression profile was identified (Additional file 6, Figure S2B). The final gene expression profile, or RNAi signature, for *CASP8AP2/FLASH *consisted of over 3500 probes (approximately 2500 genes) (Figure 4A and Additional file 7, Table S5).

We first examined genes related to the primary annotated function of CASP8AP2/FLASH in the regulation of the extrinsic apoptotic pathway. While we observed the functional activation of caspase proteins in SW480 cells following the silencing of *CASP8AP2/FLASH*, at a transcriptional level only *CASP7 *expression was increased (~2-fold linear increase). The extrinsic apoptotic associated proteins *FAS *and *FASLG *were both modestly upregulated at transcriptional level, but there was no other obvious link to this function of *CASP8AP2/FLASH*. Because of the lack of a clears association with changes in the expression of proteins associated with the extrinsic apoptotic we next looked for the enrichment of genes associated with specific functional ontologies so as to assess the broad biological processes perturbed as a result of CASP8AP2/FLASH LOF. No ontology categories where enriched within the genes that were downregulated following silencing of *CASP8AP2/FLASH*. Many ontologies were though associated with the genes that were upregulated following CASP8AP2/FLASH LOF, including the cancer, cellular growth and proliferation, cell death and gastrointestinal disease ontologies (Figure 4B and Additional file 8, Table S6). Genes associated with the cancer, cell growth and proliferation, and cell death ontologies, included the upregulation of several cyclin-dependent kinase inhibitors, specifically *CDKN1A *(p21; ~2.5-fold linear increase), *CDKN1C *(p57^Kip2^; ~5-fold linear increase), *CDKN2B *(p15; ~2-fold linear increase), and *CDKN2D *(p19; ~2-fold linear increase). These cyclin-dependent kinase inhibitors are all critical negative regulators of cell cycle. Their up-regulation following silencing of *CASP8AP2/FLASH *could therefore be of relevance to the reduction in cell viability observed in *CASP8AP2/FLASH *silenced CRC cells.

As another approach to identify critical cellular networks perturbed as a result of CASP8AP2/FLASH LOF we used Gene Set Enrichment analysis (GSEA) to identify enrichment for the targets of specific transcription factors associated with CRC within the large *CASP8AP2*/*FLASH *RNAi signature. NFκB targets were significantly enriched within our list of genes upregulated after silencing of *CASP8AP2*/*FLASH*. Activation of NFκB is most frequently associated with an inhibition of apoptosis and constitutive activation of NFκB has been linked to CRC. Previous studies of *CASP8AP2*/*FLASH *function have shown that inhibition of *CASP8AP2/FLASH *expression suppressed TNFα induced activation of NFκB [20,21]. Due to correction for multiple testing no specific canonical pathways were identified as perturbed by LOF of CASP8AP2/FLASH, but dense knowledge-based networks focused on TGFß, GRB2, and TNF were generated from the *CASP8AP2*/*FLASH *RNAi signature (Additional file 9, Figure S3). This TNF-centered network within the *CASP8AP2*/*FLASH *RNAi signature (Additional file 9, Figure S3, network 3) suggests that activation of TNF related signaling following silencing of *CASP8AP2*/*FLASH *may be contributing, at least in part, to the further up-regulation of the expression of NFκB target genes in SW480 cells.

*MYC*, a well-known proto-oncogene, is critical to the proliferation and survival of many cancers [36]. Genomic copy number gains of *MYC *is observed in some 50% of colorectal tumors and as a target of ß-catenin/TCF7L2 MYC expression is frequently deregulated in CRC [1]. Deregulated MYC expression can lead to activation of some genes and repression of other. Interestingly, we observed that after CASP8AP2/FLASH LOF, genes that are activated by MYC were repressed, while conversely genes repressed by MYC were activated (Figure 4C). This indicates that the transcriptional activity of MYC is reduced in the absence of *CASP8AP2/FLASH*. CASP8AP2/FLASH LOF did not directly induce differential expression of *MYC *suggesting that this is a secondary effect perhaps as a result of changes in proteins responsible for the post-translational modification of MYC that also regulate MYC activity [37].

The clearest LOF molecular phenotype that could be related to *CASP8AP2/FLASH *silencing was, however, alterations in the expression of the histone genes.

### The *CASP8AP2/FLASH *RNAi signature shows broad deregulation of histone gene transcription

**Figure 5:**
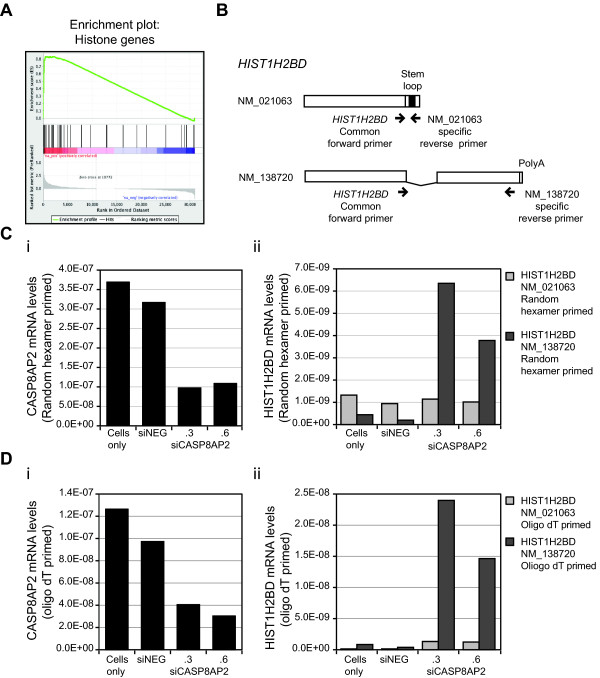
**Loss of *CASP8AP2*/*FLASH *function induces changes in the expression of the replication dependent histone genes**. (**A**) The *CASP8AP2*/*FLASH *RNAi signature was subjected to gene set enrichment analysis for 79 annotated human histone genes. A very high positive correlation was observed indicating a significant enrichment (Normalized enrichment score = 2.53; FDR q-value < 0.001) for deregulation of transcript levels of the human histone genes following silencing of *CASP8AP2*/*FLASH*. (**B**) Diagrammatic representation of the two annotated transcript variants of *HIST1H2BD *and the relative positions of PCR primers used in this study. The primers for the *HIST1H2BD *variant, NM_138720, were designed to flank a known splice site within this transcript so is its expression could be assessed by qRT-PCR without the risk of amplifying genomic DNA. **(C) **RT-PCR quantification of changes in (i) *CASP8AP2/FLASH *and (ii) *HIST1H2BD *expression using cDNA generated using random hexamer priming. **(D) **RT-PCR quantification of changes in *CASP8AP2/FLASH *and *HIST1H2BD *expression following silencing of *CASP8AP2/FLASH*. RNA was harvested 72 hours after silencing of *CASP8AP2/FLASH *and cDNA generated using either (**i**) random hexamers or (**ii**) oligo dT. Data is shown as the median of two independent cDNA syntheses and two independent PCR reactions per cDNA synthesis.

The most intriguing aspect of the *CASP8AP2/FLASH *RNAi signature was the highly significant enrichment (normalized enrichment score = 2.53; FDR q-value < 0.001) for histone transcripts (Figure 5A). Of the approximately eighty genes encoding histone proteins, transcripts corresponding to over half of them showed altered levels compared to siNeg-transfected cells, in some cases exhibiting an over twenty-fold linear change in transcript levels (Table 1). Importantly, all histone transcripts showing a greater than two-fold change in mRNA levels were encoded by a subset of histone genes known as the replication-dependent histone genes. Histone proteins form a critical component of chromosome architecture and structural alterations. Principally, the acetylation and deacetylation of lysine residues on histone tails govern many important cellular functions, in particular transcription. Epigenetic modifications of histone proteins are frequently associated with changes in expression observed in many cancers. The majority of the canonical histone proteins are encoded by a family of over 60 replication-dependent histone genes, most found in clusters on Chromosomes 1 and 6 [38]. The principal transcripts expressed from these genes are uniquely non-polyadenylated, containing instead an evolutionarily conserved 3' stem-loop sequence that is critical for regulating the expression and function of histone proteins. Two further histone transcripts expressed from genes outside of the main gene clusters can also generate non-polyadenylated mRNAs; the *H2AFX *gene that expresses the DNA damage associated histone protein, H2AX, [39] and the *HIST4H4 *gene [38]. Expression of the mRNAs encoding the canonical histone proteins is tightly regulated; their expression is rapidly induced at the beginning of S phase, must remain high throughout S phase, and then is rapidly degraded at the end of S phase [40]. The expression and processing of histone transcripts requires a unique set of proteins including nuclear protein ataxia telangietasia or NPAT protein, the U7 small nuclear RNA associated proteins LMS10 and LSM11, and the stem-loop binding protein, SLBP [40]. A number of recent studies have also implicated CASP8AP2/FLASH in the processing of histone pre-mRNAs. CASP8AP2/FLASH has been detected in Cajal/Histone locus bodies (HLB) associated with NPAT and has been shown to functionally interact with LSM11 [24,27-30]. The most recent of these studies showed that CASP8AP2/FLASH is required for the endonucleolytic cleavage of histone pre-mRNAs [30].

It is important to note that the cRNA used in this study was generated using an oligo dT primer, selective for polyadenylated transcripts, and thus the changes in the levels of the replication dependent histone transcripts would appear to reflect significant alterations in the levels of the less well defined polyadenylated versions of these histone transcripts. The detection of polyadenylated replication-dependent histone transcripts has been noted previously following silencing in HeLa cells of two other replication dependent histone 3' end processing proteins: negative elongation factor (NELF) and the cap binding complex (CBC) [41]. Following silencing of either NELF or CBC, Nariata and co-workers, reported increases in the levels of twelve histone genes by array analysis, also following oligo dT primer selection. Examination of the expression of two of these histone genes, *HIST1HC *and *HIST2H2AA*, in detail confirmed that this result was due to the induction of the expression of polyadenylated transcript variants corresponding to these genes [41]. In SW480 cells we also detected enhanced levels of a putative polyA *HIST1HC *transcript and most of the other histone genes observed by Narita and co-workers to express polyA variants following silencing of *NELF *or *CBC *(Table 1). Further, recent next generation sequencing of RNA polyadenylation has revealed the presence of polyadenylation of over twenty replication dependent histone mRNAs in unperturbed HeLa cells [42]. In an unperturbed state the polyA histone transcripts represented ~4% of the total amount of transcription from the replication dependent histone genes. Of the over twenty histone transcripts with polyA variants detected by sequencing, we detected putative polyA versions corresponding to fifteen of these genes following CASP8AP2/FLASH LOF (Table 1). To confirm that the CASP8AP2/FLASH LOF leads to the induction or enhanced presence of the non-canonical, polyadenylated transcript variants of the replication-dependent histone genes we examined by qRT-PCR the expression of one replication-dependent histone gene *HIST1H2BD*. Unlike most replication-dependent histone genes where only the non-polyA transcript has been annotated as having a consensus reference sequence, two annotated transcript variants have been reported for *HIST1H2BD*; NM_021063, the non-polyA transcript variant and NM_138720 the polyadenylated variant (Figure 5B). Using cDNA primed with either random hexamers (Figure 5C) or oligo dT primers (Figure 5D) and primers specific for the canonical non-polyA and the poly A transcript variants (Figure 5B) we observed that following silencing of *CAPS8AP2*/*FLASH *(Figure 5Ci and 5Di) only the levels of the polyadenylated variant NM_138720 of *HIST1H2BD *were increased. No significant change was seen in the levels of the NM_021603/*HIST1H2BD *non-polyA variant (Figure 5Cii) when primed using random hexamers.

### *CASP8AP2/FLASH *loss-of-function rapidly alters the expression of the transcriptome of SW480 cells

**Figure 6:**
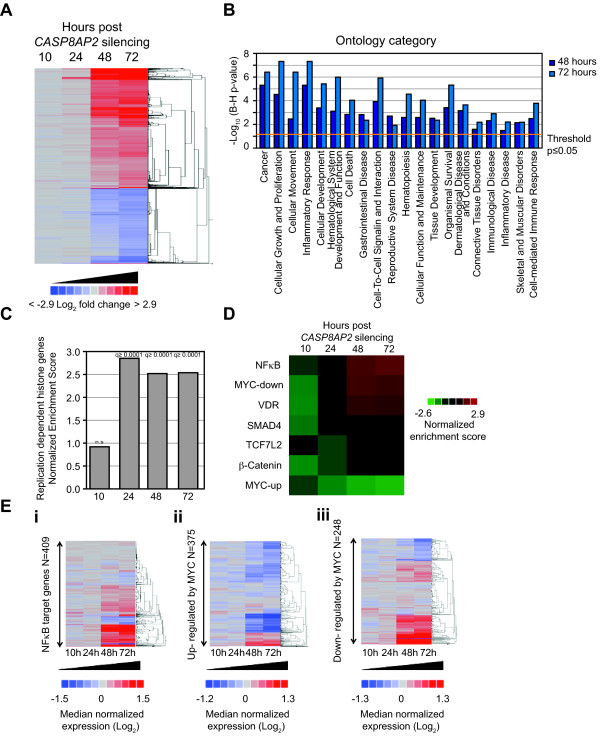
**The loss of *CASP8AP2*/*FLASH *function induces consistent effects on the transcriptome of CRC cells**. (**A**) Heat map representation of the median fold change for those transcripts showing a significant fold change (> ± 0.6 Log_2 _fold change, p < 0.05) at 72 hours post-transfection for all three siRNAs corresponding to *CASP8AP2*/*FLASH *when compared to the expression profiles obtained for siNeg transfected SW480 cells generated at the same time points. Data at earlier time points reflects the fold changes seen for all of these transcripts, though these changes in expression may have not reached significance. (**B**) The enrichment of functional ontologies for upregulated genes with the *CASP8AP2*/*FLASH *RNAi signatures obtained 48 and 72 hours post siRNA transfection. No statistically significant enrichment was seen for genes showing a downregulation in expression within the *CASP8AP2*/*FLASH *RNAi signature. (**C**) Gene set enrichment analysis of the human histone genes at 10, 24, 48 and 72 hours. (**D and E**) Gene set enrichment analysis of the targets of transcription factors associated with CRC. (**D**) Shows the overall statistical analysis for the enrichment of the targets within the *CASP8AP2*/*FLASH*RNAi signature for six transcription factors, (**E**)(**i**) shows the enrichment plots for the targets of NFκB over time, (**ii**) shows the enrichment plots for the upregulated targets of the MYC transcription factor over time, and (**iii**) shows the enrichment plots for the downregulated targets of the MYC transcription factor over time.

To corroborate and extend our study of the very extensive changes in expression seen following CASP8AP2/FLASH LOF we conducted further whole transcriptome expression assays as this platform enabled us to continue to probe in an unbiased and systematic fashion the diverse functional consequence of silencing *CASP8AP2*/*FLASH *we had observed. To further ensure the specificity of the effects seen following *CASP8AP2*/*FLASH *silencing we used an additional siRNA (siCASP8AP2.1) and also included arrays from untransfected SW480 cells. Importantly, to gain additional insight into the temporal effects on the transcriptome following CASP8AP2/FLASH LOF we assessed effects over a time course of 10, 24, 48, and 72 hours post siRNA transfection (see Additional file 10, Figure S4 for further details). At 72 hours all three *CASP8AP2/FLASH *siRNAs induced a significant change (Log_2 _> ± 0.6, q-value < 0.05) in over 2,800 probes (~2300 genes) (Figure 6A, Additional file 11, Table S7). Tracking the expression of these genes over time showed many were also changed at 48 hours, and in some cases even at 24 hours (Figure 6A, Additional file 11, Table S7). In most cases the direction of change seen at the earlier time points were maintained at 72 hours. All of the gene ontology categories enriched in the initial 72 hour *CASP8AP2/FLASH *RNAi signature were also enriched in the follow up study at both the 48 and 72 hour time points (Figure 6B and Additional file 8, Table S6) further confirming the broad but specific effects the CASP8AP2/FLASH LOF has on SW480 cells. Using GSEA we confirmed the significant enrichment for changes in the levels of histone transcripts at 24, 48, and 72 hours (Figure 6C). At just 24 hours after silencing of *CASP8AP2*/*FLASH *the majority of probes indicating a significant change in the expression (q < 0.05) corresponded to the replication dependent histone proteins (Additional file 12 Table S8). At 72 hours post *CASP8AP2*/*FLASH *siRNA transfection we detected almost exactly the same complement of histone genes showing expression of the polyA variant as seen previously (Additional file 12, Table S8). All of the histone genes noted by Narita and co-workers as expressing polyA variants following silencing of *NELF *or *CBC *[41] were again changed following *CASP8PA2*/*FLASH *(Additional file 12, Table S8). Two further polyA variant transcripts noted by Shepard and co-workers [42] were also now detected in *CASP8PA2*/*FLASH *silenced cells (Additional file 12, Table S8). The vast majority of the transcripts corresponding to replication dependent histones showed an increase in levels following silencing of *CASP8AP2*/*FLASH *confirming that the inhibition of the function of proteins required for the canonical processing of histone transcripts predominately leads to the induction and/or accumulation of polyadenylated histone transcripts. Future studies will be required to determine the exact mechanism underlying the generation of these non-canonical transcripts. Interestingly, the probes corresponding to a very limited number of histone transcripts including *HIST1H1B *and *HIST1H4L *consistently detected a decrease in expression (Table 1 and Additional file 12, Table S8) following silencing of *CASP8AP2*/*FLASH*. Further studies will be needed to investigate this, and also the effect these changes in the expression of the replication-dependent histone genes has on the production of the replication-dependent histone proteins and thus on overall chromatin architecture, however our data suggests that CASP8AP2/FLASH LOF leads to highly reproducible and specific changes on the transcriptome.

Complementary to our original data, NFκB transcriptional targets were once again significantly enriched within the upregulated portion of the *CASP8AP2*/*FLASH *signature at 24, 48, and 72 hours post-silencing (GSEA FDR q-values of 0.036, < 0.001, and < 0.001 respectively) (Figure 6D and 6Ei). We had anticipated that the time course data could be used to identify drivers of this transcriptional response, however, though TNF-centered knowledge based networks could be generated from the *CASP8AP2*/*FLASH *RNAi signature (Additional file 13, Figure S5) there was limited overlap between the specific genes that formed these networks and those obtained previously (Additional 8, Figure S3). We also confirmed that gene targets activated by MYC were downregulated at 24, 48, and 72 hours (all FDR q-values of < 0.001), and genes known to be suppressed by MYC were upregulated at the same time points (GSEA FDR q-values of 0.014, < 0.001, and < 0.001 at 24, 48 and 72 hours respectively) (Figure 6D and 6Eii and iii). While it is clear that transcriptional activity of MYC is reduced, we have been unable to identify upstream mediators that link these transcriptional changes to *CASP8AP2*/*FLASH *loss-of-function. Further work will thus be required to identify the specific signaling processes connecting *CASP8AP2*/*FLASH *silencing to alterations in these transcriptional networks.

All of the cyclin dependent kinase inhibitor genes identified as upregulated following *CASP8AP2*/*FLASH *silencing previously were once again upregulated, with *CDKN1C *(p57^Kip2^) once more showing the greatest change. Forty-eight hours post *CASP8AP2*/*FLASH *silencing *CDKN1C*/p57^Kip2 ^mRNA levels increased ~ 4 linear fold, and this was further enhanced by 72 hours post siRNA transfection when its levels were increased by over 6 linear fold. Interestingly, one study of the expression of *CDKN1C*/p57^Kip2 ^observed decreased immunostaining in colorectal carcinomas [43], and in our colorectal cell lines and tumor samples we saw significant reduction in expression (*CDKN1C*/p57^Kip2 ^expression in CRC cell lines versus normal mucosa mean linear = 0.17 (p < 0.00098) and primary colon tumor versus normal mucosa mean linear ratio = 0.15 (p < 0.0026)). Altered expression of *CDKN1C*/p57/Kip2 has been noted in several cancer types, frequently as a result of epigenetic changes leading to speculation that it may act as a tumor suppressor [44]. It will interesting to determine in the future if any of the alterations in histone gene transcription observed following the silencing of *CASP8AP2*/*FLASH *modulates the expression of epigentically regulated genes such as *CDKN1C*/p57^Kip2^. It will also be important in future studies to determine if *CASP8AP2*/*FLASH *LOF induces similar effects in other colorectal cancer cell lines and in other cancer cell types.

### The silencing of CASP8AP2/FLASH enhances the expression of neurofilament heavy polypeptide (NEFH)

**Figure 7:**
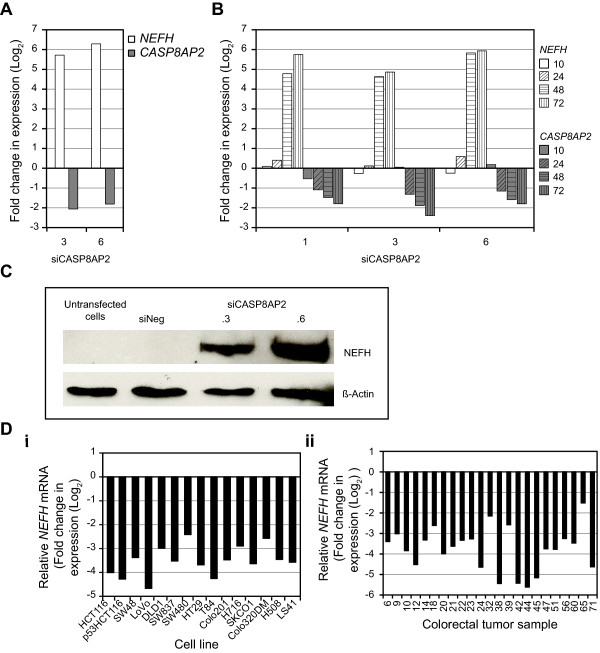
**Loss of function of *CASP8AP2*/*FLASH *induces expression of *NEFH ***. (**A**) The expression of *NEFH *72 hours following silencing of *CASP8AP2*/*FLASH *(2 different siRNAs) as measured by microarray analysis. (**B**) The expression of *NEFH *at 10, 24, 48 and 72 hours following silencing of *CASP8AP2*/*FLASH *(three different siRNAs) as measured by microarray analysis. (**C**) Western blot analysis shows increased abundance of NEFH protein 72 hours after silencing of *CASP8AP2/FLASH*. (**D**) Molecular characteristics of *NEFH *in colorectal cell lines and primary tumors. (**i**) The expression of *NEFH *mRNA in colorectal cell lines. All data is expressed as Log_2 _fold change from that observed in normal mucosa sample. (**ii**) The expression of *NEFH *in 28 primary tumor samples. All data is expressed as Log_2 _fold change from that observed in normal mucosa sample.

The most significantly upregulated annotated gene following *CASP8AP2*/*FLASH *loss-of-function was *NEFH*, which was upregulated more than 40 linear fold following the silencing of *CASP8AP2*/*FLASH*. This gene encodes the neurofilament heavy polypetide (Figure 7A). Recently, it has been shown that the *NEFH *gene is methylated in esophageal squamous cell carcinoma (ESCC) and that the inhibition of *NEFH *expression in ESCC leads to activation of both the AKT and ß-catenin/TCF7L2 pathways [45]. Our time course experiment shows that the up-regulation of *NEFH *occurs between 24 and 48 hours after initiation of the silencing of *CASP8AP2*/*FLASH *(Figure 7B). To assess if CASP8AP2/FLASH LOF increases the level of NEFH at a protein level we performed Western blot analysis following silencing of *CASP8AP2*/*FLASH *(Figure 7C). Duplicate samples were also assessed by qRT-PCR for mRNA reduction: siCASP8AP2.3 and siCASP8AP2.6 reduced *CASP8AP2*/*FLASH *transcript levels by 63% and 77%, respectively. Western blot analysis showed a clear induction of NEFH following inhibition of *CASP8AP2*/*FLASH*. The gene expression data for *NEFH *for CRC cell lines and primary tissues compared to the expression in normal colon mucosa showed this gene is significantly reduced in expression (Figure 7D; p < 2.1 × 10^-13 ^in cell lines and p < 9.6 × 10^-13 ^in tumors). Given that NEFH may function as a tumor suppressor in ESCC [45] it will be interesting to determine if it performs a similar role in colorectal cancer. Ectopic expression of NEFH has been linked to suppression of targets of the ß-catenin/TCF7L2 signaling [45]. While we saw no statistically significant evidence of repression of the ß-catenin pathway within the *CASP8AP2*/*FLASH *RNAi signature (Figures 3C and 4D) it is should be noted that *CTNNB1 *transcript levels were somewhat reduced (~2 linear fold change) at both 48 and 72 hours post *CASP8AP2 *siRNA transfection. Also, one of the most significantly downregulated genes following silencing of *CASP8AP2*/*FLASH *was *NOTUM *(Exp 1: 72 hours, siCASP8AP.3, 3.1 linear fold decrease, p = 0.001; siCASP8AP.6, 3.7 linear fold decrease p = 0.0002; Exp. 2: median fold change 48 hrs: 3.5 linear fold decrease, p = 0.0032; 72 hrs: 4.4 linear fold decrease, p = 0.0002). *NOTUM*, best characterized in *Drosophila *as regulated by *Wingless*, has recently been identified as a direct target of the ß-catenin/TCF7L2 that is overexpressed in hepatocellular carcinoma (HCC) [46]. This same study noted over expression of *NOTUM *in SW480 cells and in pooled sample of three colorectal tumors. We have also observed increased expression of *NOTUM *in SW480 cells (18.3-fold increase relative to normal mucosa) and over 75% of primary colon adenocarcinomas (18/23) showed a 2 linear fold or greater overexpression of *NOTUM*. Further studies will be required, but this may suggest, that *NOTUM*, as in HCC, is a target of the frequently activated ß-catenin/TCF7L2 pathway observed in CRC.

## Conclusions

We have identified Caspase-8-Associated protein 2/FLICE-associated huge protein (CASP8AP2/FLASH) as essential for the survival of colorectal cancer cells. Contrary to what would perhaps be expected given its principle annotated function as a pro-apoptotic factor required for activation of the extrinsic apoptotic pathway, we observed, in the absence of FAS stimulation, significant activation of both Caspase 8 and Caspase 3/7 following inhibition of CASP8AP2/FLASH function. Instead, systems-wide analysis of the LOF of CASP8AP2/FLASH confirmed and extended biochemical studies showing that CASP8AP2/FLASH has a critical role in regulating the expression of the replication-dependent histone genes. The observation that the expression levels CASP8AP2/FLASH are the same in normal and colorectal cancer tissue also suggests a role in normal homeostasis for this protein as would be expected if its primary role is in regulating the expression of the replication-dependent histone genes. Also by using an unbiased, whole transcriptome approach we were able to identify that additional downstream effects of CASP8AP2/FLASH LOF included enhanced expression of genes targeted by NFκB and evidence of decreased MYC activity. Finally, silencing of *CASP8AP2*/*FLASH *led to expression of a recently identified tumor suppressor gene *NEFH*. *NEFH *has been shown to be the subject of epigenetic modification, as too has another gene upregulated following *CASP8AP2*/*FLASH *silencing, *CDKNIC*/p57. It is our current hypothesis that the changes in the expression of these genes following silencing of *CASP8AP2/FLASH *is likely an indirect effect; it could be that these genes are the target of one or more of the transcription factors that we see target enrichment for within the *CASP8AP2/FLASH *RNAi signature, or it may be that changes in the processing of the transcripts corresponding to the replication dependent histone transcripts is altering expression more broadly.

## List of abbreviations

CASP8AP2: Caspase-8-Associated Protein 2; CDKN1C: cyclin-dependent kinase inhibitor 1C (p57, Kip2); CRC: colorectal cancer; DEDs: death-effector domains; DISC: Death-Inducing Signaling Complex; ESCC: esophageal squamous cell carcinoma; FADD: FAS-associated adaptor protein; FDR: False discovery rate; FLASH: FLICE-associated Huge Protein; GSEA: Gene Set Enrichment Analysis; LOF: loss-of-function; NEFH: Neurofilament heavy polypetide; NES: Normalized enrichment score; NUP62: Nucleoporin 62; TRAF1: TNF receptor-associated factor 1; WDR3: WD repeat domain 3; NOTUM: notum pectinacetylesterase homolog (Drosophila); NTRK1: neurotrophic tyrosine kinase, receptor, type 1; qRT-PCR: quantitative reverse transcription PCR; RNAi: RNA interference; siRNA: small interfering RNA.

## Competing interests

The authors declare that they have no competing interests.

## Authors' contributions

**ABH **participated in the design of the experiments, carried out the RNAi screen, the confirmatory experiments, the analysis of Caspase 8 and Caspase 3/7 activation, helped generate the expression profiling data and helped draft the manuscript. **JJP **carried out the bioinformatic analysis, assisted with qRT-PCR analysis of the replication dependent histone genes and helped draft the manuscript. **JC **helped with the extraction of molecular characterization data for *CASP8AP2*/*FLASH *from genomic and transcript CRC data sets and assisted in the generation of the *CASP8AP2*/*FLASH *expression profiles. **TB **and **FK **performed the initial normalization and statistical analysis of the expression array data. **KH **preformed the qRT-PCR analysis of the replication dependent histone genes. **SBS **performed the NEFH western blot analysis. **TLJ and GE **assisted in the generation of the *CASP8AP2*/*FLASH *time course expression profiles. **MG **assisted with the RNAi screen and confirmatory experiments. **MJD and TR **helped define the project concept and edited the manuscript. **NJC **participated in the design of the experiments, helped conduct the data and bioinformatic analysis and drafted the manuscript. All authors read and approved the final manuscript

## Supplementary Material

Additional file 1 **Table S1**. A siRNA based RNAi screen of 418 genes representing different functional groups associated with apoptosis. Data is shown relative to siNegative transfected cells and is ranked alphabetically within the following groups; both siRNAs ≥25% decrease in cell viability, siRNA.1 ≥25% decrease in cell viability, siRNA.2 ≥25% decrease in cell viability, both siRNAs. ≤25% decease in cell viability. Click here for file

Additional file 2 **Figure S1**. Optimization, execution and follow up of a siRNA based RNAi screen of genes associated with apoptosis. (A) Confirmation of Oligofectamine (Invitrogen) transfection conditions using *CTNNB1 *mRNA expression as an end-point. (B) Confirmation of cell line number and transfection conditions using an siRNA corresponding to PLK1 and cell viability as an end-point. (C) Summary of siRNA screening controls. Data is shown as the viability of cells transfected by a negative control siRNA (siNeg; mean and standard deviation of 33 wells) and a positive control siRNA (siPLK1; mean and standard deviation of 11 wells). On average, silencing of *PLK1 *induced an over 85% decrease in cell viability. (D) Summary of screening data with gene targets ranked as shown in Additional File 1, Table S1, both siRNAs ≥25% decrease in cell viability, siRNA.1 ≥25% decrease in cell viability, siRNA.2 ≥25% decrease in cell viability, both siRNAs. ≤25% decease in cell viability. Closed circles indicate data for siRNA and open circles data for siRNA 2. (E) Two different siRNAs corresponding to 45 genes induced a 25% or greater reduction in the viability of SW480 cells compared to siNegative-transfected cells. Data is expressed relative to siNegative-transfected cells; gene targets are ranked alphabetically. Genes chosen for initial validation are marked with *. (F) Reproducibility of the effects of silencing selected genes identified as reducing the viability of SW480 cells. Data is shown as the mean ± SD of three independent transfections for each siRNA targeting the fifteen genes of interest normalized to the values from negative control siRNA transfected cells. Click here for file

Additional file 3 **Table S2**. The sequences of gene specific siRNAs. Click here for file

Additional file 4 **Table S3**. The sequences of gene specific PCR primers. Click here for file

Additional file 5 **Table S4**. Transcription factor data sets used for gene set enrichment analysis. Click here for file

Additional file 6 **Figure S2**. The development of a *CASP8AP2*/*FLASH *RNAi signature in SW480 cells. (**A**) Correlation of the fold change in expression seen following silencing with two different *CASP8AP2/FLASH *siRNAs. Each black circle corresponds to a single probe. A red circle indicates the probe corresponding to *CASP8AP2*/*FLASH*. There was a high concordance between the effects mediated by both siRNAs targeting *CASP8AP2*/*FLASH*, only two probes showed discordant changes with respect to the direction of change in that one siRNA induced an increase in expression, while the second siRNA induced a decrease in expression. (**B**) Within the very large *CASP8AP2/FLASH *expression profile we identified only one transcript (*ABLIM2*) that showed a potential mismatch with both *CASP8AP2 *siRNAs. The probe corresponding to ABLIM2 was ranked as the 349^th ^down-regulate/d probe (out of a total of 1487 downregulated probes; ranked ~Log_2 _-2.0 to Log_2 _-0.6 fold change) within the *CASP8AP2/FLASH *expression profile. As this change is likely to have had only a minimal affect on the *CASP8AP2/FLASH *expression profile as a whole the data for this gene was retained within our subsequent analysis. Click here for file

Additional file 7 **Table S5**. *CASP8AP2*/*FLASH *RNAi signature in SW480 cells. Probes that showed a significant fold change (> Log_2 _± 0.6, q < 0.05) in *CASP8AP2*/*FLASH *silenced cells compared to siNeg transfected SW480 cells are listed, ranked by the fold change seen in siCASP8AP2.3-silenced cells. The probe corresponding to *CASP8AP2*/*FLASH *is highlighted in grey. The columns show: (1) The gene sequence reference, (2) the gene symbol, (3) the chromosomal position of the Agilent array probe, (4) the Agilent array probe identifier, (5) fold change siNeg vs. siCASP8AP2.3 Log_2 _transformed, (6) fold change siNeg vs. siCASP8AP2.6 Log_2 _transformed, (7) FDR (q-value), siNeg vs. siCASP8AP2.3 and (8) FDR (q-value), siNeg vs. siCASP8AP2.6. Click here for file

Additional file 8 **Table S6**. Significant gene ontology categories within *CASP8AP2/FLASH *RNAi signatures. Click here for file

Additional file 9 **Figure S3**. The top three knowledge-based gene networks of genes deregulated as a consequence of silencing *CASP8AP2/FLASH *were generated using Ingenuity pathway analysis tools. A maximum of 70 molecules was used for the generation of networks. Solid edges between gene nodes represent direct interactions and dashed lines represent indirect interactions. Red symbols indicate upregulated genes; Green symbols indicate downregulated genes respectively. The number of molecules in each network is stated, as is a score generated by IPA as an estimate of connectivity. Click here for file

Additional file 10 **Figure S4**. The development of *CASP8AP2*/*FLASH *RNAi signatures in SW480 cells over time. **(A) **Reduction in *CAPS8AP2/FLASH *mRNA measured by (**i**) qRT-PCR and (**ii**) array analysis and cell viability in SW480 cells over time. All data is normalized to the levels of *CAPS8AP2/FLASH *and viability observed in siNeg transfected SW480 cells. Correlation of the fold change in expression seen following silencing with three different *CASP8AP2/FLASH *siRNAs at (**B**) 48 hours and (**C**) 72 hours compared to siNeg transfected SW480 cells. Each black circle corresponds to a single probe. A red circle indicates the probe corresponding to *CASP8AP2/FLASH*. Using the data obtained at 48 and 72 hours we observed no off-target sequence alignments between transcripts showing a decrease in expression for all three siRNAs and the sequences of the three different *CASP8AP2*/*FLASH *siRNAs though nine genes showed off-target alignments with two of three siRNAs (**data not shown**). To assess the reproducibility of our initial findings we examined the expression of genes that previously showed differential expression following transfection of siCASP8AP2.3 and siCASP8AP2.6 siRNAs, with data obtained at the same time point (72 hours) with same siRNAs as part of this follow up study. The data for ~2000 probes were compared. (**D**) Correlation of the fold change in expression seen in two different experiments in which *CASP8AP2*/*FLASH *has been silenced for 72 hours. Each black circle corresponds to a single probe. Click here for file

Additional file 11 **Table S7**. Changes in gene expression over time following silencing of *CASP8AP2*/*FLASH*. Data is shown for the 2820 probes that measured a significant change in expression in SW480 cells transfected with each of the three *CASP8AP2*/*FLASH *siRNAs at 72 hours and the data for the same probes at 48, 24 and 10 hours post siRNA transfection. The median fold change for each probe is shown as well as the data for each of the three siRNAs; data is ranked on the median value at 72 hours. Fold changes ≥ Log_2 _-0.6 and q < 0.05 matched to any probe showing a decreased level of expression are marked in green and fold changes of ≥0.6 and q < 0.05 matched to any probe showing an increased level of expression are marked in red. The probe corresponding to *CASP8AP2*/*FLASH *is marked is highlighted in yellow. Click here for file

Additional file 12 **Table S8**. Changes in histone transcript levels at multiple time points following silencing of *CASP8AP2*/*FLASH*. Click here for file

Additional file 13 **Figure S5**. TNF focused knowledge-based gene networks of genes deregulated as following silencing of *CASP8AP2/FLASH*. TNF network at (**A**) 48-hours, and (**B**) 72-hours generated using Ingenuity pathway analysis tools. A maximum of 70 molecules was used for the generation of networks. Solid edges between gene nodes represent direct interactions and dashed lines represent indirect interactions. Red symbols indicate upregulated genes; Green symbols indicate downregulated genes respectively. The number of molecules in each network is stated, as is a score generated by IPA as an estimate of connectivity. Click here for file
