# Supplementary material for: Systems-wide RNAi analysis of CASP8AP2/FLASH shows transcriptional deregulation of the replication-dependent histone genes and extensive effects on the transcriptome of colorectal cancer cells
Source: Mol Cancer. 2012 Jan 4;11:1. doi: 10.1186/1476-4598-11-1 (PMC3281783; doi:10.1186/1476-4598-11-1)
Supplement: Additional file 1 — Table S1. A siRNA based RNAi screen of 418 genes representing different functional groups associated with apoptosis. Data is shown relative to siNegative transfected cells and is ranked alphabetically within the following groups; both siRNAs ≥25% decrease in cell viability, siRNA.1 ≥25% decrease in cell viability, siRNA.2 ≥25% decrease in cell viability, both siRNAs. ≤25% decease in cell viability. [file 1476-4598-11-1-S1.PDF]

Gene Symbol	Transcript Reference Number	Gene ID	Target siRNA 1	Target siRNA 2	Gene Name	Relative viability (siNegative normalized) siRNA 1	siRNA 2
AKT2	NM_001626	208	ACGGGCTAAAGTGACCATGAA	CAAGCGTGGTGAATACATCAA	v-akt murine thymoma viral oncogene homolog 2	**0.66**	**0.43**
**APAF1**	NM_013229	317	CAGTGAAGGTATGGAATATTA	CCGCATTCTGATGCTTCGCAA	apoptotic protease activating factor	**0.60**	**0.40**
ARID4A	NM_002892	5926	ACGATTGAAGTTGATAGTATT	CTGGTTTGACATTGCTAAGTA	AT rich interactive domain 4A (RBP1-like)	**0.68**	**0.56**
BAG3	NM_004281	9531	CCCAATTCAAGTGATCCGCAA	CCGCAAAGAGGTGGATTCTAA	BCL2-associated athanogene 3	**0.75**	**0.70**
**BIRC5**	NM_001168	332	CTCGGCTGTTCCTGAGAAATA	CTGGCGTAAGATGATGGATTT	baculoviral IAP repeat-containing 5 (survivin)	**0.74**	**0.75**
CAPN11	NM_007058	11131	ATGCGCCTGGTTATTGAGAAA	CACATTCTTTCTAACCATGGA	calpain 11	**0.63**	**0.51**
**CASP8AP2**	NM_012115	9994	AACGAAGTTTCGAGATTCTTA	CAGTCTGATCTCAATAAGGAA	CASP8 associated protein 2	**0.40**	**0.58**
CAV1	NM_001753	857	CAGCCGTGTCTATTCCATCTA	CTGGTCAACCGCGACCCTAAA	caveolin 1, caveolae protein, 22kDa	**0.69**	**0.48**
CLU	NM_001831	1191	CCGGTTTATATGATCTTCATA	GCCGGTTTATATGATCTTCAT	clusterin	**0.72**	**0.45**
DPF2	NM_006268	5977	CTGGACCTGTTGAAAGAGAAA	CTGGATGACCTCGATGATGAA	D4, zinc and double PHD fingers family 2	**0.62**	**0.66**
DUSP7	X93921	1849	CGGCGGCGAGTTCACCTACAA	TACGACTTTGTCAAGAGGAAA	dual specificity phosphatase 7	**0.49**	**0.65**
F2R	NM_001992	2149	CAGTATAGAATAGGCACTTTA	TAGAGTGTGATGTATGTGTAA	coagulation factor II (thrombin) receptor	**0.13**	**0.71**
GPX1	Y00433	2876	AACACCTTGATCTTACAGAAA	GAGGAACACCTTGATCTTACA	glutathione peroxidase 1	**0.73**	**0.71**
GSN	NM_000177	2934	AACGATGCCTTTGTTCTGAAA	CAGCTACATCATTCTGTACAA	gelsolin (amyloidosis, Finnish type)	**0.67**	**0.68**
GZMA	NM_006144	3001	ACACGCGAAGGTGACCTTAAA	ACCCTGTGATTGGAATGAATA	granzyme A	**0.47**	**0.74**
**HSP90B1**	NM_003299	7184	ATGGATTAAATGCATCACAAA	TCGGTCAGAGCTGACGATGAA	heat shock protein 90kDa beta (Grp94), member 1	**0.25**	**0.64**
IGF1R	NM_000875	3480	AACCTTAATGACAACACTTAA	TCGAAGAATCGCATCATCATA	insulin-like growth factor 1 receptor	**0.73**	**0.70**
IL1A	NM_000575	3552	CACGCCTACTTAAGACAATTA	CTGAGGTGATTTATGCCTTAA	interleukin 1, alpha	**0.57**	**0.63**
LGALS1	NM_002305	3956	CACCATCGTGTGCAACAGCAA	CGCCAGCAACCTGAATCTCAA	lectin, galactoside-binding, soluble, 1	**0.40**	**0.65**
MAP2K1	NM_002755	5604	CTGGAAGAATTCCTGAACAAA	CTGGATCAAGTCCTGAAGAAA	mitogen-activated protein kinase kinase 1	**0.75**	**0.53**
MAP3K1	XM_042066	4214	CACGCATGTCAAATTCTCATA	CTCCGGGTGTTTCAACTAGAA	mitogen-activated protein kinase kinase kinase 1	**0.69**	**0.64**
MAPK4	NM_002747	5596	AAGGATCGTTGATCAGCATTA	CGCCTTAAATCTAATCAGCAA	mitogen-activated protein kinase 4	**0.34**	**0.74**
MAPT	NM_016835	4137	CCGCCAGGAGTTCGAAGTGAT	TTAGGCAACATCCATCATAAA	microtubule-associated protein tau	**0.59**	**0.72**
**NFKB2**	X61498	4791	AAGGTGATGGATCTGAGTATA	CTCCTCCATTGTGGAACCCAA	nuclear factor of kappa light polypeptide gene enhancer in B-cells 2	**0.69**	**0.45**
**NLRC4**	AF376061	58484	AACGCTGTTCCATACCTTCTA	CGGACATTACATCCACTTATA	NLR family, CARD domain containing 4	**0.51**	**0.54**
NME5	NM_003551	8382	ATGATATTAGCTAGACATAAA	CATGATATTAGCTAGACATAA	non-metastatic cells 5	**0.55**	**0.39**
**NTRK1**	NM_002529	4914	CACATCATCGAGAACCCACAA	CGAGAGCATCCTGTACCGTAA	neurotrophic tyrosine kinase, receptor, type 1	**0.65**	**0.32**
**NUP62**	NM_016553	23636	ACCGGCTTTGCCTTGAATTTA	CGGCTTTGCCTTGAATTTAAA	nucleoporin 62kDa	**0.59**	**0.52**
NUPR1	NM_012385	26471	CGAGAGGAAACTGGTGACCAA	CTGGATGAATCTGACCTCTAT	nuclear protein, transcriptional regulator, 1	**0.62**	**0.66**
**PCNA**	M15796	5111	TACACTAAGGGCCGAAGATAA	TGCGGATATGGGACACTTAAA	proliferating cell nuclear antigen	**0.69**	**0.74**
PGLYRP1	NM_005091	8993	GAGGTCCAACTATGTGCTCAA	TGGCATCAGCTTCATGGGCAA	peptidoglycan recognition protein 1	**0.61**	**0.33**
PPP1R15A	NM_014330	23645	CCAGTTGTTGATCTTATGCAA	TGGGTTTATATAAGGAATAAA	protein phosphatase 1, regulatory (inhibitor) subunit 15A	**0.73**	**0.74**
RBL1	NM_002895	5933	ACAGGCTAATGTGGAGTATAA	CACCAAGTGACCAACTTATAA	retinoblastoma-like 1 (p107)	**0.64**	**0.60**
**RIPK1**	NM_003804	8737	CCGACATTTCCTGGCATTGAA	TACCACTAGTCTGACGGATAA	receptor (TNFRSF)-interacting serine-threonine kinase 1	**0.23**	**0.61**
**SFRP2**	AF311912	6423	ACCCATTGTAGCTTACCTGTA	ACCGAGGAAGCTCCAAAGGTA	secreted frizzled-related protein 2	**0.36**	**0.44**
TFDP2	NM_006286	7029	AAGGCAGAAATGAACATGAAA	CGCTACTTCTTAAGTACTTAA	transcription factor Dp-2 (E2F dimerization partner 2)	**0.27**	**0.50**
**TNFRSF10A**	NM_003844	8797	ATCAAACTTCATGATCAATCA	CCGGGTCCACAAGACCTTCAA	tumor necrosis factor receptor superfamily, member 10a	**0.33**	**0.73**
TNFRSF18	NM_004195	8784	CAGGAGGGAGAGAGAGACACA	TCGGGATTCTCAGGTCATGAA	tumor necrosis factor receptor superfamily, member 18	**0.57**	**0.59**
TNFSF13B	NM_006573	10673	CACGCCTTACTTCTTGCCTTA	CGGAGGGTAAATGCCAGCAAA	tumor necrosis factor (ligand) superfamily, member 13b	**0.71**	**0.63**
TP53	NM_000546	7157	TTGCAGTTAAGGGTTAGTTTA	TTGGTCGACCTTAGTACCTAA	tumor protein p53 (Li-Fraumeni syndrome)	**0.62**	**0.47**
**TP53BP2**	NM_005426	7159	ACCGTGTATCTCAGTAACAAT	CACGAGCGTCGAAGAGACAAA	tumor protein p53 binding protein, 2	**0.65**	**0.67**
**TRAF1**	NM_005658	7185	AGGGCTCCACTAGGACTTCAA	CAGACTGATCAGGACCTCCAA	TNF receptor-associated factor 1	**0.48**	**0.59**
**WDR3**	NM_006784	10885	CCGGGATGTTATCGGCTTCAA	TCCGCTGATAGGAATGTGAAA	WD repeat domain 3	**0.50**	**0.51**
YWHAZ	M86400	7534	AACCTTGCTTCTAGGAGATAA	CACGTGTATTAAATTCATGTA	tyrosine 3-monooxygenase/tryptophan 5-monooxygenase activation protein, zeta polypeptide	**0.62**	**0.46**
ZNF346	NM_012279	23567	ACGCAAAGAACTTAAAGCTGA	CGCAAAGAACTTAAAGCTGAA	zinc finger protein 346/double-stranded RNA-binding zinc finger protein JAZ	**0.64**	**0.11**
AKT3	NM_181690	10000	AACTGTTGGCTTTGGATTAAA	CAGCAGGCACGTTAACTCGAA	v-akt murine thymoma viral oncogene homolog 3 (protein kinase B, gamma)	0.92	**0.57**
APEX1	NM_001641	328	ACCCTTAATTAAGATCCTCAA	CCGGGTGATTGTGGCTGAATT	APEX nuclease (multifunctional DNA repair enzyme) 1	1.13	**0.70**
APP	NM_000484	351	ACCCAATTAAGTCCTACTTTA	CTGGTCTTCAATTACCAAGAA	amyloid beta (A4) precursor protein (protease nexin-II, Alzheimer disease)	0.82	**0.10**
BAD	AK023420	572	AAGATTTAGGTTAACTTCTCA	CACCTTAAAGGAGTCCACAAA	BCL2-antagonist of cell death	0.90	**0.72**
BAG1	NM_004323	573	CCGTCTCTGGATCGGAATTTA	CTGGATCGGAATTTACCTGAT	BCL2-associated athanogene	1.13	**0.60**
BCL10	NM_003921	8915	CACGTACTGTTTCACGACAAT	GTGCTGAAACTTAGAAATATA	B-cell CLL/lymphoma 10	0.76	**0.50**
BCL11B	NM_022898	64919	CAGAGGTGGGTTAAACTGTAA	CAGCGGATAACAATCTTTCAA	B-cell CLL/lymphoma 11B (zinc finger protein)	0.84	**0.64**
BCL2L10	NM_020396	10017	ACAGATGTGTGAGAACAAGAA	ATGACAGATGTGTGAGAACAA	BCL2-like 10 (apoptosis facilitator)	0.90	**0.51**
BCL2L2	NM_004050	599	AAGGCTGAGTATGGTTCCCAA	TCGCCAGAACGTGGGACCAAA	BCL2-like 2	0.91	**0.73**
BECN1	NM_003766	8678	ACCGACTTGTTCCTTACGGAA	AGGGTCTAAGACGTCCAACAA	beclin 1 (coiled-coil, myosin-like BCL2 interacting protein)	1.15	**0.73**
BIRC7	NM_022161	79444	ATGCTTCTGAATAGAAATAAA	TTGGATGCTTCTGAATAGAAA	baculoviral IAP repeat-containing 7 (livin)	0.93	**0.75**
BOK	NM_032515	666	CAGCCTGTAAACAGAGCGTAA	GCCCTGCACATTCAATCTGAA	BCL2-related ovarian killer	1.05	**0.71**
CAPN9	NM_006615	10753	GAGAACTTCTATGAGATTCTA	GAGGAGTGTAGTTTCCTTGTA	calpain 9 (nCL-4)	1.18	**0.61**
CASP1	NM_033292	834	CCCAGATATACTACAACTCAA	TCGGCAGAGATTTATCCAATA	caspase 1, apoptosis-related cysteine protease (interleukin 1, beta, convertase)	1.25	**0.74**
CASP2	NM_032982	835	TAAGGTGAACGCACTTATCAA	TTGGAAGGTGTCCAAATTTAA	caspase 2, apoptosis-related cysteine protease	0.85	**0.52**
CASP3	NM_004346	836	ACAGCATGTAATGGTATCTTA	CAGCATGTAATGGTATCTTAA	caspase 3, apoptosis-related cysteine protease	1.34	**0.66**
CASP5	NM_004347	838	AAGAATCGCGTGGCTCATCAA	TTCGTGATAAACCACATGCTA	caspase 5, apoptosis-related cysteine protease	1.04	**0.66**
CASP7	NM_001227	840	CAGGCTATTACTCGTGGAGGA	TACCTTGTTAATAGACTTAAT	caspase 7, apoptosis-related cysteine protease	1.14	**0.51**
CD40LG	NM_000074	959	ACACAGCATGATCGAAACATA	TGGGAAACAGCTGACCGTTAA	CD40 ligand	1.00	**0.59**
CUL2	NM_003591	8453	CGGCACAATGCCCTTATTCAA	TACATCGGATGTATACAGATA	cullin 2	0.95	**0.33**
CYC1	NM_001916	1537	CCCATCATGGGAATAAATTAA	TACCATGTCCCAGATAGCCAA	cytochrome c-1	0.87	**0.72**
DAP3	NM_033657	7818	TCGCTTTGTGATGCAGGTGAA	TTGGCTCTGGACCTGCATTAA	death associated protein 3	0.97	**0.61**
DAPK1	NM_004938	1612	AAGCATGTAATGTTAATGTTA	CGGCTATTACTCTGTGGCCAA	death-associated protein kinase 1	0.96	**0.64**
DEDD	NM_004216	9191	CTGAGCCGTAAGACAAATGTA	TGGCTTTATGAGAGCTCTTAA	death effector domain containing	0.77	**0.56**
DFFB	NM_004402	1677	ACGGGTCAGTAGGGATAAGAA	TACGATGTTTATACTCCGTAA	DNA fragmentation factor, 40kDa, beta polypeptide (caspase-activated DNase)	1.23	**0.59**
DIABLO	AK001399	56616	TACCGACAATATACAAGTTTA	TAGCGGGACCGTCACACACAA	diablo homolog (Drosophila)	1.11	**0.45**
DNASE1L3	NM_004944	1776	AAGGGCCTTCACCAACAGCAA	CACGGTGAAGAAGAGCACCAA	deoxyribonuclease I-like 3	0.95	**0.56**
DNASE2	NM_001375	1777	AAGGGCCACCACGTTAGCCAA	GACCTTGATTGTGTGCCTTAA	deoxyribonuclease II, lysosomal	1.31	**0.57**
ERCC6	L04791	2074	ACGGGCATTTCTGATTATTAA	CAGAGCGTTTAGAAAGTGAAA	excision repair cross-complementing rodent repair deficiency, complementation group 6	0.91	**0.66**
FAF1	NM_007051	11124	AAGGACGAGGATGAACGTGAA	CAGGCATGTACTGGCATTGAA	Fas (TNFRSF6) associated factor 1	0.91	**0.68**
FANCA	NM_000135	2175	CAGCATATTCAGGAGGCCTTA	CAGCGTTGAGATATCAAAGAT	Fanconi anemia, complementation group A	0.82	**0.72**
GAB1	NM_002039	2549	ACAGAAGACCTTGGCTCTAAA	CACGAGCATTTCCAAGTGATA	GRB2-associated binding protein 1	1.23	**0.69**
GSTP1	X15480	2950	AAGGATGACTATGTGAAGGCA	CTCCCTCATCTACACCAACTA	glutathione S-transferase pi	0.77	**0.69**
HRK	NM_003806	8739	AGCGATCGTAGAAACACAGAA	CGAGAAGGAAGTGGAGAGTAA	harakiri, BCL2 interacting protein (contains only BH3 domain)	1.20	**0.17**
IGF1	M27544	3479	ATCAATAATGTTCTATAGAAA	CCAGAAGGAAGTACATTTGAA	insulin-like growth factor 1 (somatomedin C)	1.11	**0.29**
IGF2R	NM_000876	3482	ACGGAGTCTCGTACTATATAA	CAGACCAGGCTTGCTCTATAA	insulin-like growth factor 2 receptor	1.04	**0.30**
IGFBP6	NM_002178	3489	CCCACTGGGAGTAGCGGCTAA	CCGCAGAGACCAACAGAGGAA	insulin-like growth factor binding protein 6	1.27	**0.32**
IKBKG	NM_003639	8517	CTCCTCTAGTTCAGAGACATA	TTCGGAAATGCCTCACATATA	inhibitor of kappa light polypeptide gene enhancer in B-cells, kinase gamma	0.83	**0.54**
IL10RB	NM_000628	3588	ATGGACTTATAATGTGCAATA	CAGCTCAGTACCTAAGTTATA	interleukin 10 receptor, beta	1.05	**0.67**
IL24	NM_006850	11009	CAGTGGATGGGTGCTTAGTAA	CTGGCTGAACTTTGTGGTCGA	interleukin 24	0.93	**0.68**
IRF1	NM_002198	3659	CAAGCATGGCTGGGACATCAA	CAGCCGAGATGCTAAGAGCAA	interferon regulatory factor 1	1.14	**0.55**
MAP3K5	NM_005923	4217	CAGCGAGTAGATAATATCGAA	CCGGGAATCTATACTCAATGA	mitogen-activated protein kinase kinase kinase 5	1.13	**0.60**
MAP3K7	NM_003188	6885	CAAGAATATATGAAAGTTCAA	CCCGTGTGAACCATCCTAATA	mitogen-activated protein kinase kinase kinase 7	0.99	**0.60**
MCL1	BC017197	4170	CCCGCCGAATTCATTAATTTA	CTGGTTTGGCATATCTAATAA	myeloid cell leukemia sequence 1 (BCL2-related)	0.82	**0.60**
MYB	NM_005375	4602	CTGGACGAACTGATAATGCTA	TTGGGATATATCATTCCTCAA	v-myb myeloblastosis viral oncogene homolog (avian)	0.98	**0.61**

Gene Symbol	Transcript Reference Number	Gene ID	Target siRNA 1	Target siRNA 2	Gene Name	Relative viability (siNegative normalized)	
						siRNA 1	siRNA 2
NLRP1	NM_014922	22861	CAGCTTCTGCTCGCCAATAAA	GAGGTAAACATTCTAATTGCA	NLR family, pyrin domain containing 1	1.06	**0.68**
NOD1	NM_006092	10392	CAGCCTGACAAGGTCCGCAAA	GCCCGCTCATTTGTTAATAAA	nucleotide-binding oligomerization domain containing 1	0.97	**0.65**
PAK1	NM_002576	5058	TCCACTGATTGCTGCAGCTAA	TTGAAGAGAACTGCAACTGAA	p21/Cdc42/Rac1-activated kinase 1 (STE20 homolog, yeast)	0.98	**0.47**
PDCD1	NM_005018	5133	ATCGGAGAGCTTCGTGCTAAA	CCCATTCCTGAAATTATTTAA	programmed cell death 1	0.95	**0.63**
PDCD2	AK055180	5134	ACGAATGGTGGGACTAACTAA	CAGGCTTATTTCCTTAATCTA	programmed cell death 2	0.89	**0.73**
PHLDA2	NM_003311	7262	CGCGCTCGGCACGACATGAAA	CTGGTTCTTTGTAGTCACATA	pleckstrin homology-like domain, family A, member 2	0.85	**0.26**
PRDX4	NM_006406	10549	AACCTGGTAGTGAAACAATAA	AAGGAGGACTTGGGCCAATAA	peroxiredoxin 4	0.86	**0.41**
PSEN2	NM_000447	5664	AAGCAAGCTATTGGAGCTGAA	CAGGAGAGAAATGAGCCCATA	presenilin 2 (Alzheimer disease 4)	0.76	**0.65**
PTPN13	NM_006264	5783	CGGTCTATTCTTACTAAGAAA	TCCAGGTACATTAAAGATGAA	protein tyrosine phosphatase, non-receptor type 13 (APO-1/CD95 (Fas)-associated	1.09	**0.66**
RAF1	NM_002880	5894	CAGATCTTAGTAAGCTATATA	TGGGAAATAGAAGCCAGTGAA	v-raf-1 murine leukemia viral oncogene homolog 1	0.97	**0.62**
RB1	NM_000321	5925	AAGGTTCAACTACGCGTGTAA	CGCGTGTAAATTCTACTGCAA	retinoblastoma 1	0.93	**0.75**
RBBP4	X74262	5928	CAGCTATCCCTCTATATAATA	CCGATAATGCTATTAGCCCAA	retinoblastoma binding protein 4	1.20	**0.58**
RIPK2	NM_003821	8767	ACGTATGATCTCTCTAATAGA	CACAAGGACATCGACCTGTTA	receptor-interacting serine-threonine kinase 2	1.18	**0.67**
RTEL1	NM_016434	51750	AAGCAGCAAGCCGACATCATA	CCAGATCACGTTTCAGACCAA	regulator of telomere elongation helicase	1.20	**0.38**
SCARB1	NM_005505	949	CCGATCCATGAAGCTAATGTA	TAGGGAGAGGCTCGTCAACAA	scavenger receptor class B, member 1	1.00	**0.31**
SIAH1	NM_003031	6477	CCCATTAATCAGTTCATTAGA	TGGCATTGGAACTACATTCAA	seven in absentia homolog 1 (Drosophila)	1.09	**0.63**
STK17B	NM_004226	9262	AGCATATATGTTGTTAACTCA	CATGTTAATGAAATAATTCAA	serine/threonine kinase 17b (apoptosis-inducing)	1.00	**0.74**
TAX1BP1	NM_006024	8887	CAGATCAATCAGCTAATAATA	CAGTCTTTGGCTTATCAATAA	Tax1 (human T-cell leukemia virus type I) binding protein 1	1.14	**0.70**
TNFAIP8	NM_014350	25816	CCGAGTACATGTGAGCGGTAA	TAGCATTGATGGAGAAATTTA	tumor necrosis factor, alpha-induced protein 8	0.91	**0.60**
TNFRSF10B	AF016266	8795	CCGACTTCACTTGATACTATA	CTGGACAACCTTACAAGTATA	tumor necrosis factor receptor superfamily, member 10b	1.03	**0.20**
TNFRSF10C	NM_003841	8794	ACCAACGCTTCCAACAATGAA	ATCGTAGGGATCATAGTTCTA	tumor necrosis factor receptor superfamily, member 10c, decoy without an intracellular domain	0.94	**0.58**
TNFRSF10D	NM_003840	8793	CCGGAGTGACATCAAGTGCAA	TAGAACGGGATTATCTTGTTA	tumor necrosis factor receptor superfamily, member 10d, decoy with truncated death domain	0.97	**0.39**
TNFRSF11A	NM_003839	8792	ACCCGCTAGGTGGTTAATTTA	CCCGCTAGGTGGTTAATTTAT	tumor necrosis factor receptor superfamily, member 11a, activator of NFKB	0.95	**0.71**
TNFRSF25	NM_003790	8718	CACCGTCCAGTTGGTGGGTAA	CGCGGTATTAAATCTGTGAAA	tumor necrosis factor receptor superfamily, member 25	0.87	**0.36**
TNFRSF6B	NM_003823	8771	AAGGAGGTGGCATGTCGGTCA	CTGCTCCAGCAAGGACCATGA	tumor necrosis factor receptor superfamily, member 6b, decoy	0.81	**0.27**
TNFSF10	NM_003810	8743	AACACAAAGAACGACAAACAA	ATGGTCCAATATATTTACAAA	tumor necrosis factor (ligand) superfamily, member 10	0.79	**0.57**
TNFSF11	NM_003701	8600	CAGATGGATCCTAATAGAATA	CAGCGTCGCCCTGTTCTTCTA	tumor necrosis factor (ligand) superfamily, member 11	1.06	**0.56**
TNFSF4	NM_003326	7292	AACGTTTATGACTATAGGTAA	CAGGCCAAGATTCGAGAGGAA	tumor necrosis factor (ligand) superfamily, member 4	0.96	**0.59**
TP53I3	NM_004881	9540	AAGCTGATATGAGGAAATAAA	CAGAGCCGTTTAAAGCTGATA	tumor protein p53 inducible protein 3	1.17	**0.46**
TRAF2	NM_021138	7186	CACGAGGGCATATATGAAGAA	CTGGACCAAGACAAGATTGAA	TNF receptor-associated factor 2	1.03	**0.72**
TRAIP	NM_005879	10293	CCGTGATGATATTGATCTCAA	CGGGACCAGCCTGAGGTGTAA	TRAF interacting protein	1.02	**0.58**
VEGFB	NM_003377	7423	CAGTGTGAATGCAGACCTAAA	CCGGATGCAGATCCTCATGAT	vascular endothelial growth factor B	0.91	**0.62**
XIAP	NM_001167	331	CACGTACTTGTGCGAATTATT	CTGTTGTTAAGCAACAGTATA	X-linked inhibitor of apoptosis	0.89	**0.57**
YWHAG	NM_012479	7532	ACCAGTGTTAGCTTAATCTTA	CCGATTAGGCCTGGCTCTTAA	tyrosine 3-monooxygenase/tryptophan 5-monooxygenase activation protein, gamma	1.02	**0.10**
YWHAH	NM_003405	7533	CAGGCTACAGTTGATATTTAA	TTCCATCAGCTTTATAATAAA	tyrosine 3-monooxygenase/tryptophan 5-monooxygenase activation protein, eta polypeptide	1.23	**0.67**
ZRANB1	AJ252060	54764	CAAGGGTGAAATCTTCGTATA	CAGATCTGTAATGACCCTAAA	zinc finger, RAN-binding domain containing 1	0.92	**0.47**
ADAM17	NM_003183	6868	CTGCAGTAAACAATCAATCTA	TCCCATGAAGAACACGTGTAA	a disintegrin and metalloproteinase domain 17 (tumor necrosis factor, alpha, converting	**0.73**	1.09
APR-3	NM_016085	51374	AAGCCAAGACTTCATGAACTA	CTGGAATACTATCACCTCTTA	apoptosis related protein APR-3	**0.48**	0.86
ATM	NM_000051	472	AACCATGAGTCTAGTACTTAA	TTGGCTTATACGCGCAGTGTA	ataxia telangiectasia mutated (includes complementation groups A, C and D)	**0.17**	0.92
BAX	NM_004324	581	ATCATCAGATGTGGTCTATAA	CAGCTCTGAGCAGATCATGAA	BCL2-associated X protein	**0.72**	1.06
BCL2	NM_000633	596	CAGGCTTAAGATTTGGAATAA	CTGTGGCATTATTGCATTATA	B-cell CLL/lymphoma 2	**0.71**	1.19
BMF	AK024472	90427	CAGACGCTAGAAACTAAATTA	GACGCTAGAAACTAAATTATA	Bcl2 modifying factor	**0.23**	1.03
BNIP1	NM_013979	662	ACGAACGATCCTGGATGCAAA	CACGTCCGGATCTGTAACCAA	BCL2/adenovirus E1B 19kDa interacting protein 1	**0.50**	0.94
BTK	NM_000061	695	CAGCTCGAAACTGTTTGGTAA	CCCTTTATGATTACATGCCAA	Bruton agammaglobulinemia tyrosine kinase	**0.64**	0.91
CAPN1	NM_005186	823	CAGGGACTTGTGTACTGGTTA	TCCCACGGAACTGCTGTCAAA	calpain 1, (mu/I) large subunit	**0.57**	1.09
CAPN6	NM_014289	827	AAGGGTGGTCCAACTGCCAAA	CAAGGTCATTATGTCACTGCA	calpain 6	**0.72**	0.89
CARD10	AY028896	29775	CACAGGGAGTGTGACACTTAA	CTGCACATTATGGAGCATTAA	caspase recruitment domain family, member 10	**0.73**	0.77
CARD14	NM_024110	79092	CACCTGGATTATGAGCTCCTA	CCGCATCGTCAGTATGGACAA	caspase recruitment domain family, member 14	**0.38**	0.79
CARD18	NM_021571	59082	AAGATATTCTCGTGAAAGAAA	CCAAGTCTTGCTGCAAATTTA	caspase recruitment domain family, member 18	**0.74**	1.21
CARD8	NM_014959	22900	CCCTGATATTCTACTTATTTA	CCGGATAGGTGTGTTTATGAA	caspase recruitment domain family, member 8	**0.48**	1.16
CARD9	NM_052813	64170	ACGTAAGGACTCCAAGATGTA	CCGCGTCTTCTCCATGATCAT	Homo sapiens caspase recruitment domain family, member 9 (CARD9)	**0.73**	1.03
CASP4	NM_001225	837	CTGGACTATAGTGTAGATGTA	TCGGGTCATGGCAGACTCTAT	caspase 4, apoptosis-related cysteine protease	**0.66**	1.28
CAV2	NM_001233	858	CAGACTTCTCGGAATTCATAA	TACGCTAATAAGTGACAAATA	caveolin 2	**0.70**	1.07
CDKN1A	NM_000389	1026	CAGTTTGTGTGTCTTAATTAT	CTGGCATTAGAATTATTTAAA	cyclin-dependent kinase inhibitor 1A (p21, Cip1)	**0.74**	1.02
CDKN2A	NM_000077	1029	CACGCCCTAAGCGCACATTCA	CAGGTGTGCCACATTCGCTAA	cyclin-dependent kinase inhibitor 2A (melanoma, p16, inhibits CDK4)	**0.00**	1.08
CIDEB	NM_014430	27141	CAGGCTAGATAACCCACCCAA	CCGATTCACCTTTGACGTGTA	cell death-inducing DFFA-like effector b	**0.53**	1.28
CNR2	NM_001841	1269	AACCCTGTCATCTATGCTCTA	TTCCGGAATCATCTACACCTA	cannabinoid receptor 2 (macrophage)	**0.68**	0.98
CTNNAL1	NM_003798	8727	AAGCTTGGGAATCCCAAATTA	ACGCTTATTAATCATAAAGAT	catenin (cadherin-associated protein), alpha-like 1	**0.67**	0.99
CTSD	NM_001909	1509	CCCAGAGGACTACACGCTCAA	TCCCGAGGTGCTCAAGAACTA	cathepsin D (lysosomal aspartyl protease)	**0.56**	1.23
CUL1	NM_003592	8454	AACGTAGTTATCAGCGATTCA	ACCGACAGCACTCAAATTAAA	cullin 1	**0.50**	0.78
DAPK3	NM_001348	1613	CCCAGAGATTGTGAACTATGA	CCGGCAGAAGGGCACGGGCAA	death-associated protein kinase 3	**0.72**	1.30
DDIT3	S40706	1649	AAGGAAGTGTATCTTCATACA	CAGCTTGTATATAGAGATTGT	DNA-damage-inducible transcript 3	**0.28**	1.23
DFFA	NM_004401	1676	CCGGAGCATCTCAGCAAGCAA	CTGGCGAGATCCGGACTCTAA	DNA fragmentation factor, 45kDa, alpha polypeptide	**0.71**	1.43
DNASE1	AJ298844	1773	ACCTGTGCTTACAGAAAGAAA	TACCTGGATGTCCAAGAGAAA	deoxyribonuclease I	**0.49**	0.84
ENC1	NM_003633	8507	ACGGCATGTATCACAGATGTA	CAGCGGCTCCATTAACATCTA	ectodermal-neural cortex (with BTB-like domain)	**0.49**	1.23
FADD	NM_003824	8772	AGCGGGATCTCGTATCTTTAA	CAGCGGGATCTCGTATCTTTA	Fas (TNFRSF6)-associated via death domain	**0.38**	0.87
FASTK	NM_006712	10922	CAGCAGCAAGGTGGTACAGAA	CTGGTGGTTCAGGAAACGCAA	FAST kinase	**0.75**	0.94
FOXO3	NM_001455	2309	CTGAATGATGGGCTGACTGAA	CTGGTTAGAAACTAGAATATT	forkhead box O3	**0.67**	1.27
GADD45B	NM_015675	4616	GAGGATGACATCGCCCTGCAA	TCCCAGTTTGCGAATTAATAA	growth arrest and DNA-damage-inducible, beta	**0.55**	0.89
GADD45G	NM_006705	10912	CGGCGTCTACGAGTCAGCCAA	CTAAAGGATGCTGCTGTTGAA	growth arrest and DNA-damage-inducible, gamma	**0.66**	1.13
GDNF	NM_000514	2668	CGCGCTGAGCAGTGACTCAAA	CTGGAGTTAATGTCCAACCTA	glial cell derived neurotrophic factor	**0.36**	0.92
GSK3B	NM_002093	2932	AACACTGGTCACGTTTGGAAA	CTGCATTTATCGTTAACCTAA	glycogen synthase kinase 3 beta	**0.30**	1.17
GSR	X15722	2936	ATGGTCTGTGCTAACAAGGAA	CGGCATGATAAGGTACTTAGA	glutathione reductase	**0.61**	0.89
HSPA5	NM_005347	3309	TAGGGTGTGTGTTCACCTTCA	TGGGATAAGGAAACACTTCTA	heat shock 70kDa protein 5 (glucose-regulated protein, 78kDa)	**0.56**	0.98
HTRA2	NM_013247	27429	CCCGATGTTCAGCATGGTGTA	CCGCGAGGTCCCTATCTCGAA	HtrA serine peptidase 2	**0.60**	0.89
IGF2	M29645	3481	CAGCAGCATCTTCAAACATGT	CAGCATCTTCAAACATGTACA	insulin-like growth factor 2 (somatomedin A)	**0.54**	0.88
IGFBP4	NM_001552	3487	CGGGATGAACCTATCCTTCCA	CTGAATGTGCCTAATGGAGAA	insulin-like growth factor binding protein 4	**0.48**	0.93
JUN	NM_002228	3725	AAGAAGTGTCCGAGAACTAAA	CTGCCCAGTGTTGTTTGTAAA	v-jun sarcoma virus 17 oncogene homolog (avian)	**0.71**	1.02
JUND	NM_005354	3727	GAGGATGGAAACACCCTTCTA	GCGCCTGGAAGAGAAAGTGAA	jun D proto-oncogene	**0.61**	0.95
LTBR	NM_002342	4055	CCGCCACACGGTCACCTGCAA	CCGGCGGGTCTATGACTATCA	lymphotoxin beta receptor (TNFR superfamily, member 3)	**0.28**	1.18
MALT1	NM_006785	10892	CACAGTTGATACATAGTTGTA	CTGGTAATCCAAGTAATGTTA	mucosa associated lymphoid tissue lymphoma translocation gene 1	**0.55**	0.77
MAP3K3	NM_002401	4215	CAGGAATACTCAGATCGGGAA	CCACGTGTCTGTGCACCACAA	mitogen-activated protein kinase kinase kinase 3	**0.67**	1.12
MAPK10	NM_002753	5602	CCGCATGTGTCTGTATTCATA	TCCGAGCACAATAAACTCAAA	mitogen-activated protein kinase 10	**0.63**	1.43
MAPK12	NM_002969	6300	CTGGACGTATTCACTCCTGAT	TGGAAGCGTGTTACTTACAAA	mitogen-activated protein kinase 12	**0.25**	0.99
MAPK14	NM_001315	1432	CAGAGAACTGCGGTTACTTAA	CTCAGTGATACGTACAGCCAA	mitogen-activated protein kinase 14	**0.54**	1.05
MDM4	NM_002393	4194	AAAGAGGTCATGCACTATTTA	TCAGAGCTTCTCCGTAAAGAA	Mdm4, transformed 3T3 cell double minute 4, p53 binding protein	**0.59**	1.23
NCKAP1	NM_013436	10787	GAGGCTATAATAAACGTATTA	TTCACTGAGATTATTCCTATA	NCK-associated protein 1	**0.68**	0.92
NFKBIA	NM_020529	4792	AAGGGTGTACTTATATCCACA	CTGAAAGAACATGGACTTGTA	nuclear factor of kappa light polypeptide gene enhancer in B-cells inhibitor, alpha	**0.52**	0.87
NGFR	NM_002507	4804	AACGTTAAGTGATGAACATTA	ACGTTAAGTGATGAACATTAA	nerve growth factor receptor (TNFR superfamily, member 16)	**0.54**	0.91
NGFRAP1	NM_014380	27018	ATGGCAAATATTCACCAGGAA	CTGGGAAAGAATCCCGGGAAA	nerve growth factor receptor (TNFRSF16) associated protein 1	**0.55**	0.98
PDCD6	NM_013232	10016	CACGGGTGTGTGGAAGTACAT	GACGGATATATTCAGACGTTA	programmed cell death 6	**0.60**	1.09

Gene Symbol	Transcript Reference Number	Gene ID	Target siRNA 1	Target siRNA 2	Gene Name	Relative viability (siNegative normalized)	
						siRNA 1	siRNA 2
PRDX5	NM_012094	25824	CACCTGCAGCCTGGCACCCAA	CTGAGTGTTAATGATGCCTTT	peroxiredoxin 5	**0.56**	0.77
PTGS2	NM_000963	5743	CCCGCAGTACAGAAAGTATCA	TCGTATTGCTGCTGAATTTAA	prostaglandin-endoperoxide synthase 2 (prostaglandin G/H synthase and cyclooxygenase)	**0.25**	1.20
PYCARD	NM_013258	29108	CAGCTTCTACCTGGAGACCTA	CGCGAGGGTCACAAACGTTGA	PYD and CARD domain containing	**0.74**	1.31
RELB	NM_006509	5971	CCAGATTGCCATTGTGTTCAA	GCGGATTTGCCGAATTAACAA	v-rel reticuloendotheliosis viral oncogene homolog B, nuclear factor of kappa light polypeptide	**0.68**	0.91
RNF7	NM_014245	9616	CCAGTGAACAGGGTAAATAAA	GGCCCAGATGATATAATTTAA	ring finger protein 7	**0.60**	0.98
SERPINB2	NM_002575	5055	CAGAAGGGTAGTTATCCTGAT	CTGGAAAGTGAAATAACCTAT	serine (or cysteine) proteinase inhibitor, clade B (ovalbumin), member 2	**0.75**	1.06
STK3	NM_006281	6788	CCGGCGCCTAAGAGTAAACTA	CGGCGCCTAAGAGTAAACTAA	serine/threonine kinase 3 (STE20 homolog, yeast)	**0.70**	0.99
TANK	NM_004180	10010	ATCCTTTATAGTGATGCTACA	CAGACTATTATTGACAAGCTA	TRAF family member-associated NFKB activator	**0.53**	1.14
TGFB1	NM_000660	7040	CACGTGGAGCTGTACCAGAAA	CAGCATATATATGTTCTTCAA	transforming growth factor, beta 1 (Camurati-Engelmann disease)	**0.04**	1.07
TMBIM6	NM_003217	7009	AAGGCCGAACATGGAGATCAA	CTGATTATGTGTATCAAGGTA	transmembrane BAX inhibitor motif containing 6	**0.16**	0.93
TNFAIP1	NM_021137	7126	ACCCATGTCTTTCTACCCTAA	TGCCCTCTTGAGGTCCTTTAA	tumor necrosis factor, alpha-induced protein 1 (endothelial)	**0.46**	0.82
TNFAIP2	NM_006291	7127	CAGGGTGATGCTGAAGATGAT	CCGGATGTCCATGGAGCAGAA	tumor necrosis factor, alpha-induced protein 2	**0.54**	0.78
TNFAIP3	NM_006290	7128	CAGCCTTTACTCATACTATTA	CCGAGCTGTTCCACTTGTTAA	tumor necrosis factor, alpha-induced protein 3	**0.56**	0.93
TNFRSF11B	NM_002546	4982	CACAATGTACAAATTGTATCA	TACCTTCATTATGACGAAGAA	tumor necrosis factor receptor superfamily, member 11b	**0.56**	0.90
TNFRSF9	NM_001561	3604	CAAGTTGGACCTTGATATTTA	CTGAGTTATGGTAATACGTGA	tumor necrosis factor receptor superfamily, member 9	**0.51**	1.23
TNFSF18	NM_005092	8995	ATGGCTAAGTTTGGACCATTA	CACCATAGACTTGATATTCAA	tumor necrosis factor (ligand) superfamily, member 18	**0.52**	1.06
TRAF3	NM_003300	7187	AGAGAGCATCGTTAAAGATAA	TCCGATGATCGCGCTGCAGAA	TNF receptor-associated factor 3	**0.65**	0.78
TRAF4	NM_004295	9618	CACCAGGACATTCGAAAGCGA	CCGGAGCTGGAAGTACAAGTA	TNF receptor-associated factor 4	**0.74**	0.83
VEGFA	AF022375	7422	ATAGAGAATTCTACATACTAA	*GAGGGTTTCAATATACATCTA*	vascular endothelial growth factor A	**0.65**	1.20
AATF	NM_012138	26574	TCGAGAACTCATAGAACGGAA	TTGGAAGGAAGGATCAAACTA	apoptosis antagonizing transcription factor	0.85	0.83
AATK	AB014541	9625	CTCCAACGTGTCAGCCAACAA	TCCGCTGAGATCAGAAGGCAA	apoptosis-associated tyrosine kinase	0.81	0.83
AGTR2	NM_000686	186	CTGCATGAGTGTTGATAGGTA	TGGAGGTTTATAGTTAATCTA	angiotensin II receptor, type 2	0.81	0.99
AIF1	NM_004847	199	CACCTAGCAGTTGGTTGGCAA	CAGAGTCAACAAATTAAATAA	allograft inflammatory factor 1	1.12	0.98
AIFM1	NM_004208	9131	AGGGTACTGATTGTATCTGAA	CAGCGATGGCATGTTCCTCTA	apoptosis-inducing factor, mitochondrion-associated, 1	1.25	0.97
AKT1	NM_005163	207	ACGCTACTTCCTCCTCAAGAA	CACGCTTGGTCCCGAGGCCAA	v-akt murine thymoma viral oncogene homolog 1	0.94	1.18
APG5L	NM_004849	9474	CTAGGAGATCTCCTCAAAGAA	TCCAACTTGTTTCACGCTATA	APG5 autophagy 5-like (S. cerevisiae)	0.97	1.18
API5	NM_006595	8539	AAGAGTAATGGTCAATCTTAA	CAAGTTTGCTATTCAAATCAA	apoptosis inhibitor 5	1.01	0.84
ARFIP2	NM_012402	23647	AAAGTTTGACATCGTCAAGAA	CAGGACCCAACCTCAATGAAA	ADP-ribosylation factor interacting protein 2	0.82	0.85
BAG2	NM_004282	9532	CAGGCGAAGATCAACGCTAAA	TCAGGCGAAGATCAACGCTAA	BCL2-associated athanogene 2	1.00	1.00
BAG4	NM_004874	9530	CAACATATACTTCATGTGTAA	CTGGATATAATCTGAGACAAA	BCL2-associated athanogene 4	1.20	0.91
BAG5	NM_004873	9529	AAGGAGATATTCAGCAAGCTA	CCGGATTATTCTACTCCTGCA	BCL2-associated athanogene 5	0.93	0.87
BAK1	NM_001188	578	AACCCATTCACTACAGGTGAA	CAGGATTCAGCTATTCTGGAA	BCL2-antagonist/killer 1	1.17	0.78
BARD1	NM_000465	580	CTGAATATTATACCAGATGAA	GACCATCAATACAGTCGCATA	BRCA1 associated RING domain 1	0.86	0.96
BBC3	AF332558	27113	CAGCCTGTAAGATACTGTATA	CCCAGCCTGTAAGATACTGTA	BCL2 binding component 3	0.98	0.91
BCL2A1	NM_004049	597	AGGACACTCCATATTGTGAAA	TGCGGAGTTCATAATGAATAA	BCL2-related protein A1	1.15	1.05
BCL2L1	NM_001191	598	AAGGCGGATTTGAATCTCTTT	CTGCTTGGGATAAAGATGCAA	BCL2-like 1	0.88	1.09
BCL2L14	NM_030766	79370	ATGGCCTGCATTTGAGCTAAA	TCCGTTTCTAGTACTATTTAT	BCL2-like 14 (apoptosis facilitator)	0.77	0.98
BCL3	NM_005178	602	ACGGATCACGATGTAAATTAT	CACGATGTAAATTATTAAGCA	B-cell CLL/lymphoma 3	0.84	1.21
BCL6	NM_001706	604	AACCTGCATTTCCGTCACAAA	AACCTTAGTGTGATCAATCTA	B-cell CLL/lymphoma 6	0.76	1.07
BCL7B	NM_001707	9275	CACGTCCCTGAGGATATTTAA	CTCAGGTTACTTGAACTTGAA	B-cell CLL/lymphoma 7B	1.15	0.92
BDNF	X91251	627	CGGGACGGTCACAGTCCTTGA	CTGGCGATTCATAAGGATAGA	brain-derived neurotrophic factor	0.90	1.19
BFAR	NM_016561	51283	CCGGGACGAGTGGAATGATTA	TCCGGTGTGCTCACAGCTTTA	bifunctional apoptosis regulator	1.11	0.95
BID	NM_001196	637	AAAGACAATGTTAAACTTATA	CAGGGATGAGTGCATCACAAA	BH3 interacting domain death agonist	1.17	1.00
BIK	NM_001197	638	CCGAGGAGCAGGAGTGCTCAA	TCGGCAAAGAATCTACTGGAA	BCL2-interacting killer (apoptosis-inducing)	1.08	1.04
BIRC2	NM_001166	329	TAGGCGTTTCTGATAACACTA	TCCCAGGTCCCTCGTATCAAA	baculoviral IAP repeat-containing 2	0.98	0.94
BIRC3	AF070674	330	CACAGACGTCTTTACAAGCAA	GCCGGAATTATTAATGAACAA	baculoviral IAP repeat-containing 3	0.88	0.94
BIRC6	NM_016252	57448	ACGGATGGAACTGACAGAATA	CCCGATATATTGGATAATCGA	baculoviral IAP repeat-containing 6 (apollon)	0.93	1.02
BMP4	NM_001202	652	CACGAGACTGGTCCACCACAA	CCCGATGGGATTCCCGTCCAA	bone morphogenetic protein 4	1.05	1.00
BNIP2	NM_004330	663	AAGGCAATTGAACCCTATAAA	CAGGGTTGATATGAAGGCAAT	BCL2/adenovirus E1B 19kDa interacting protein 2	1.12	0.82
BNIP3	NM_004052	664	ATGAATAACATTGATAATAAA	ATGCTAATTGTTCATAATCAA	BCL2/adenovirus E1B 19kDa interacting protein 3	1.24	1.00
BNIP3L	AL132665	665	AAGCTTTACACCTGTTTACAA	GACATCGATATTGACTATTTA	BCL2/adenovirus E1B 19kDa interacting protein 3-like	1.05	1.02
BRAF	NM_004333	673	ACACGACATGTGAATATCCTA	TTGCTTATATGTTAAATTGAA	v-raf murine sarcoma viral oncogene homolog B1	0.77	1.28
BRE	NM_004899	9577	AAGAAATAAACTCACAAATTA	CTCGCTGTTCTTCCTCTGAAA	brain and reproductive organ-expressed (TNFRSF1A modulator)	0.94	1.07
CARD11	NM_032415	84433	CAACTCGAGATCGATCAGCTA	CACAGTACTCGCAGTGCTTAA	caspase recruitment domain family, member 11	1.15	0.89
CARD6	NM_032587	84674	AACCTTCTCCATGCAAATCTA	CCCAATTTGCTTGAATGGGAA	caspase recruitment domain family, member 6	1.21	0.91
CASP10	NM_001230	843	CACGTTCTTAGCCTAGATTGA	CAGTGGTGCCTGCATTTATAA	caspase 10, apoptosis-related cysteine protease	1.20	1.05
CASP14	NM_012114	23581	CACAGATGCCTTGCACGTTTA	GAGGAGAAATATGATATGTCA	caspase 14, apoptosis-related cysteine protease	1.24	1.29
CASP6	NM_001226	839	CAGGAATTAAATCATCCCTTA	GCGCAGATAGAGACAATCTTA	caspase 6, apoptosis-related cysteine protease	1.18	1.31
CASP8	NM_001228	841	AAGATAATCAACGACTATGAA	CGGAGGGTCGATCATCTATTA	caspase 8, apoptosis-related cysteine protease	1.16	1.31
CASP9	NM_001229	842	CAGGGATTAACCTCTGCACTA	CTGGACTTTCCTGCAACTCAA	caspase 9, apoptosis-related cysteine protease	1.20	1.35
CBL	NM_005188	867	CCGTACTATCTTGTCAAGATA	TGCGGTTGTGTCAGAACCCAA	Cas-Br-M (murine) ecotropic retroviral transforming sequence	1.09	1.15
CBX4	NM_003655	8535	AAGCCCTTCTTTGGGAATATA	GAGGCTGGTCGCCCAAATATA	chromobox homolog 4	0.93	0.98
CD14	NM_000591	929	CAGCCTAGACCTGTCTGACAA	CTCAGAGGTTCGGAAGACTTA	CD14 antigen	0.98	1.05
CD180	NM_005582	4064	AAGCCTGAACTTCAATGGCAA	TGGGACTATCACGAAGACCAA	CD180 molecule	0.84	1.08
CD27	NM_001242	939	AAGGTTGGCTTGCCACCTGAA	ACCGAGTGTGATCCTCTTCCA	CD27 molecule	1.12	0.77
CD38	NM_001775	952	CTCAATGGATCCCGCAGTAAA	CTGGAATCGATTATAAGCAAA	CD38 antigen (p45)	0.97	1.26
CD40	NM_001250	958	CCCAGTCGGCTTCTTCTCCAA	TTGGTGCTGGTCTTTATCAAA	CD40 molecule, TNF receptor superfamily member 5	0.89	1.25
CD70	NM_001252	970	CAGCTACGTATCCATCGTGAT	CTGCGTCTCAGCTTCCACCAA	CD70 molecule	1.29	0.83
CDC25A	NM_001789	993	CAGCTTAGCTAGCATTACTAA	CTGGCCAAATAGCAAAGACAA	cell division cycle 25A	1.00	0.80
CDKN2D	NM_001800	1032	ACCCAAGGGCAGAGCATTTAA	ATGAGTTATGAGTTATTCATA	cyclin-dependent kinase inhibitor 2D (p19, inhibits CDK4)	0.86	1.01
CGRRF1	NM_006568	10668	CCGGGAAATTTATGATATTAT	CGGGAAATTTATGATATTATT	cell growth regulator with ring finger domain 1	0.95	1.10
CHUK	NM_001278	1147	CAGGAGAAGTTCGGTTTAGTA	TTCCATAAGCTTGGTGACAAA	conserved helix-loop-helix ubiquitous kinase	1.05	1.22
CIDEA	NM_001279	1149	CGGGTGCTGGATGACAAGGAA	GAGAGTCACCTTCGACTTGTA	cell death-inducing DFFA-like effector a	0.98	1.27
CLDN5	NM_003277	7122	CACCGGCGACTACGACAAGAA	CACCGGGCACATGCAGTGCAA	claudin 5 (transmembrane protein deleted in velocardiofacial syndrome)	0.96	1.17
CLN3	NM_000086	1201	CACACTCGTCATCAAATTGTT	CCGCTGGTACCAGATGCTGTA	ceroid-lipofuscinosis, neuronal 3, juvenile (Batten, Spielmeyer-Vogt disease)	1.13	0.81
CNR1	NM_016083	1268	CACGTTAAAGTAGGACTATCA	TTCCATAGTTTAGGTACTCAA	cannabinoid receptor 1 (brain)	1.05	0.98
CNTF	NM_000614	1270	ACCAGTATAGACAGAAGTAAA	GACCAGTATAGACAGAAGTAA	ciliary neurotrophic factor	0.93	0.77
CRADD	NM_003805	8738	AGGCAGGTGTCTCATATGTAA	CAGGGTTTCCACTAGACATTA	CASP2 and RIPK1 domain containing adaptor with death domain	1.13	0.89
CTNNB1	NM_001904	1499	ATGGGTAGGGTAAATCAGTAA	CTCGGGATGTTCACAACCGAA	catenin (cadherin-associated protein), beta 1, 88kDa	1.21	1.21
CUL3	NM_003590	8452	AACAACTTTCTTCAAACGCTA	GACGTTAAGAATGGCAGATAA	cullin 3	1.49	0.95
DAD1	NM_001344	1603	CAGATTTGACACTTACTGCTA	TTGCCTGAGAATACAGATCAA	defender against cell death 1	1.05	1.08
DAP	NM_004394	1611	ACGGACCAGGGTGGAGGAATA	CGAAGGGAAACTAGAGACTAA	death-associated protein	0.97	0.85
DAPK2	NM_014326	23604	CGGAATTTGTTGCTCCAGAAA	CTGGTTAAAGAGACCCGGAAA	death-associated protein kinase 2	0.76	0.81
DAXX	NM_001350	1616	CACCCGCTACCCAGAGGTTAA	CACGGTTGAAGCGTAAGCTGA	death-associated protein 6	1.16	0.99
DCC	NM_005215	1630	CACCTGTGTCCAAGAACTCTA	CAGCATACCAATTACCCATAA	deleted in colorectal carcinoma	0.84	1.04
DDX41	NM_016222	51428	CCGAGCATTGATGTCAGTGAA	CCGCCTGTACTCATCTTTGCA	DEAD (Asp-Glu-Ala-Asp) box polypeptide 41	1.02	0.95
DOCK1	NM_001380	1793	ACGATCGTCAACGATGTGAAA	CACGCTGGTTTGAGACTGTTA	dedicator of cytokinesis 1	1.22	1.02
DUSP6	NM_001946	1848	TACGGACACTATTATCACTAA	TGCGGAATTGGTTAATACTAA	dual specificity phosphatase 6	0.79	0.95
E2F3	NM_001949	1871	TACAGTATTTGCGTTACTTTA	TTGCGTTACTTTAAGTACTAA	E2F transcription factor 3	1.21	1.02
E2F5	NM_001951	1875	AAGGACTCATTACTTGTCTTA	GACGATTCCATTAATAATAGA	E2F transcription factor 5, p130-binding	1.26	1.02
EI24	NM_004879	9538	AACCACGTATTGTTAGTAGAA	ATCGTTGGTTCAATAAAGGAA	etoposide induced 2.4 mRNA	1.07	1.07
EIF2AK2	NM_002759	5610	ACGGAAAGACTTACGTTATTA	CGGAAAGACTTACGTTATTAA	eukaryotic translation initiation factor 2-alpha kinase 2	0.99	0.84

Gene Symbol	Transcript Reference Number	Gene ID	Target siRNA 1	Target siRNA 2	Gene Name	Relative viability (siNegative normalized)	
						siRNA 1	siRNA 2
ERCC3	NM_000122	2071	ACCGTTCTGAATAAACAGAAT	CCGGGAATATGTGGCAATCAA	excision repair cross-complementing rodent repair deficiency, complementation group 3	1.11	0.98
F2	NM_000506	2147	AAGGTACGAGCGAAACATTGA	CCGCACAAGGTACGAGCGAAA	coagulation factor II (thrombin)	1.03	1.12
FAIM3	NM_005449	9214	CAGGGTTGGGTCTTACACAAA	CCGAGTTACTCTGAAGCAATA	Fas apoptotic inhibitory molecule 3	0.86	1.33
FAS	NM_000043	355	AAGGACATTACTAGTGACTCA	TCAGTGTATGTTAGTACAAAT	Fas (TNF receptor superfamily, member 6)	1.15	0.84
FASLG	NM_000639	356	ATCGGTGAAACTAACAGATAA	GAGGCTTGCATAATAAGCTAA	Fas ligand (TNF superfamily, member 6)	1.29	1.00
FIGF	NM_004469	2277	CAGGTTGTAAGTGCTTGCCAA	TTCTATGACATTGAAACACTA	c-fos induced growth factor (vascular endothelial growth factor D)	0.77	1.02
FOXK2	NM_004514	3607	CCGAGCACAAACATCAAGATA	CTCGGTGACCATCGGCCGCAA	forkhead box K2	1.10	1.07
FOXO1	NM_002015	2308	AACCAAGTAGCCTGTTATCAA	CCCGAGTTTAGTAACAGTGCA	forkhead box O1	0.92	0.97
FXR1	NM_005087	8087	ACTGAAGAAGTTCCTAGTTTA	CAGCTAAGAATGGAACGCCTA	fragile X mental retardation, autosomal homolog 1	1.17	1.14
GADD45A	L24498	1647	ACCAAATATGTTAAAGTTTAA	CTCCCGTGACTAATTCCTTTA	growth arrest and DNA-damage-inducible, alpha	0.89	0.91
GAS2	NM_005256	2620	ACGTGTCTATTTGAATCGGAA	TACAGAGTATTCACAATAGTA	growth arrest-specific 2	1.14	1.10
GPX4	NM_002085	2879	AGGGAGTAACGAAGAGATCAA	GTGGATGAAGATCCAACCCAA	glutathione peroxidase 4 (phospholipid hydroperoxidase)	1.28	1.01
GRB2	NM_002086	2885	AGGCTTTAAGCTCCAGCTTTA	CCCGTGAACCGGAACGTCTAA	growth factor receptor-bound protein 2	1.08	0.91
GSTA1	M25627	2938	TTGCAACAATAAAGTACTTTA	TTGCAATACCAATGTTCTAAA	glutathione S-transferase A1	0.93	0.77
GSTM1	X08020	2944	CTGCTACAATCCAGAATTTGA	TCCAGAATTTGAGAAACTGAA	glutathione S-transferase M1	1.33	0.82
GSTO1	NM_004832	9446	CAGGAGTCAGCAATAAAGCTA	CAGGCATGAAGTCATCAATAT	glutathione S-transferase omega 1	0.93	0.89
GSTT2	BC002415	2953	AAGCTCGGCCATCCTGATTTA	CAAGGAGTTCTTGCAGATCAA	glutathione S-transferase theta 2	0.81	0.99
GULP1	NM_016315	51454	ACCCAGTTACTGCTCAGTTTA	CTCCAGTACCTAGTAGATCTA	GULP, engulfment adaptor PTB domain containing 1	1.45	1.21
GZMB	NM_004131	3002	CTGGAGCCAAGTCCAGATTTA	TACGCCATTATTACGACAGTA	granzyme B	1.18	0.89
HSPE1	NM_002157	3336	AGCGTGAAAGTTGGAGATAAA	CTGAAATAAGTCACTATTGAA	heat shock 10kDa protein 1 (chaperonin 10)	0.97	0.89
HTATIP2	NM_006410	10553	AACCGGCAGAGTGCTCTTAAA	ATCGGTAATTTCAGGGTCTAA	HIV-1 Tat interactive protein 2, 30kDa	0.90	0.97
HTT	NM_002111	3064	CAGGGTAGAATTGTTTGGCAA	CAGGTGTTTATTGGCTTTGTA	huntingtin	1.09	0.89
IGFALS	NM_004970	3483	CAGCTTGAGGTGCTCACGCTA	CCGACTCTTCCTCAAGGACAA	insulin-like growth factor binding protein, acid labile subunit	0.96	1.02
IGFBP2	NM_000597	3485	ACAGTGCAAGATGTCTCTGAA	CACACGTATTTATATTTGGAA	insulin-like growth factor binding protein 2, 36kDa	1.25	1.16
IL1B	NM_000576	3553	CAAGATAGAAATCAATAACAA	CAGCCAATCTTCATTGCTCAA	interleukin 1, beta	0.79	1.00
IL2	NM_000586	3558	CACCAGGATGCTCACATTTAA	CTGGAGGAAGTGCTAAATTTA	interleukin 2	1.03	1.06
IL2RA	NM_000417	3559	CCACCCTATATGTAGTATAAA	CTCCACCCTATATGTAGTATA	interleukin 2 receptor, alpha	0.98	1.00
IL3	NM_000588	3562	AGCAATTGAGAGCATTCTTAA	CTGCTCTAACATGATCGATGA	interleukin 3 (colony-stimulating factor, multiple)	1.07	0.99
IL3RA	M74782	3563	ACCCACCAATCACGAACCTAA	CACAGATAAGTTTGTCGTCTT	interleukin 3 receptor, alpha (low affinity)	0.85	1.10
JUNB	NM_002229	3726	AAACACGCACTTAGTCTCTAA	CTCGATTCATATTGAATATAA	jun B proto-oncogene	0.97	0.87
KALRN	NM_007064	11139	CAGGACATCAATCAAGTCTTA	CCCATTGAGTATCAACGGAAA	kalirin, RhoGEF kinase	1.06	0.80
LITAF	NM_004862	9516	GCCAATGTAGTCTCACTTAAA	TGCCAATGTAGTCTCACTTAA	lipopolysaccharide-induced TNF factor	1.13	0.95
LTA	NM_000595	4049	ACCCTCGATGAAGCCCAATAA	CCGGAGCTTTCAAAGAAGGAA	lymphotoxin alpha (TNF superfamily, member 1)	0.97	0.97
LTB	NM_002341	4050	CAGGATCAGGGAGGACTGGTA	CCCGCAGGACGGCCTCTATTA	lymphotoxin beta (TNF superfamily, member 3)	1.19	0.86
MADD	NM_003682	8567	CAGACCCACTACTATAGTAAA	CAGGAGGGCGTTAGTGGATCA	MAP-kinase activating death domain	1.01	1.18
MAEA	NM_005882	10296	TAGAGTCTAATTGCTATCCAT	TTCCATAACTGTAGAGTCTAA	macrophage erythroblast attacher	0.99	0.92
MAP2K4	NM_003010	6416	AGGGAGAATGGTGCTGTTTAA	AGGGTGTATAGTGTTCACAAA	mitogen-activated protein kinase kinase 4	1.12	0.91
MAP2K5	NM_002757	5607	AAGACGTATGTTGGAACAAAT	CAAGACGTATGTTGGAACAAA	mitogen-activated protein kinase kinase 5	1.38	1.21
MAP2K6	NM_002758	5608	AAGGCTTGCATTTCTATTGGA	TAGACCTATGATAAATAACCA	mitogen-activated protein kinase kinase 6	1.28	1.26
MAP3K14	NM_003954	9020	CACATGCATGTGACTCCTCAA	TACCTAGTGCATGCTCTGCAA	mitogen-activated protein kinase kinase kinase 14	0.82	1.28
MAPK7	NM_002749	5598	CAGACCCACCTTTCAGCCTTA	TACGAGATCATCGAGACCATA	mitogen-activated protein kinase 7	1.21	0.92
MAPK8	NM_002750	5599	AAGCCCAGTAATATAGTAGTA	TCGGGACTTAAAGCCCAGTAA	mitogen-activated protein kinase 8	1.14	1.06
MDM2	NM_002392	4193	CCGGATCTTGATGCTGGTGTA	GCCCAGTATGTAGACAACCAA	Mdm2, transformed 3T3 cell double minute 2, p53 binding protein	0.87	1.09
MMP9	NM_004994	4318	AACCTTTGAGGGCGACCTCAA	ACGGCTTGCCCTGGTGCAGTA	matrix metalloproteinase 9	0.90	0.98
MPO	NM_000250	4353	AGGCATCACCACCGTGTCTAA	CTCATGTATGTGCGAAGTATA	myeloperoxidase	1.05	1.07
MTL5	NM_004923	9633	CCACACTGAGTTTAAATCTAA	CCCAAATTATGTGTTCTTCTA	metallothionein-like 5, testis-specific	0.87	1.08
MX1	NM_002462	4599	TAAGATCAAGACACTCATCAA	TCCGTTAGCCGTGGTGATTTA	myxovirus (influenza virus) resistance 1, interferon-inducible protein p78	1.00	1.31
MYBL2	NM_002466	4605	CCAGGGCTTCTTGCAGGCCAA	CCGGAAGTCTCTGGCTCTTGA	v-myb myeloblastosis viral oncogene homolog (avian)-like 2	1.18	0.86
MYC	NM_002467	4609	CCCAAGGTAGTTATCCTTAAA	CTCGGTGCAGCCGTATTTCTA	v-myc myelocytomatosis viral oncogene homolog (avian)	0.88	0.98
MYCN	BC002712	4613	TACCTAAGTACTGTAATAATA	TAGTATGACGCTGATACATAA	v-myc myelocytomatosis viral related oncogene, neuroblastoma derived	0.94	0.79
NAIP	NM_004536	4671	CGTGGTGAACTTTGTGAATTA	TCGAAATTCATGATACTACTA	NLR family, apoptosis inhibitory protein	1.37	0.89
NFKB1	NM_003998	4790	CTGGGTATACTTCATGTGACA	GACGCCATCTATGACAGTAAA	nuclear factor of kappa light polypeptide gene enhancer in B-cells 1 (p105)	0.89	1.24
NFKBIB	AK057862	4793	CACGTGGCCGTTATCCACAAA	CAGGATGAATACGACGACATT	nuclear factor of kappa light polypeptide gene enhancer in B-cells inhibitor, beta	1.00	0.85
NFKBIE	NM_004556	4794	CTGAGTGAGAAGAGACTGAAA	CTGGCTGTACATCTGGACCAA	nuclear factor of kappa light polypeptide gene enhancer in B-cells inhibitor, epsilon	0.77	1.13
NME3	NM_002513	4832	ACGCACCTTCCTGGCCGTGAA	CTGCATCGAGGTTGGCAAGAA	non-metastatic cells 3, protein expressed in	0.83	0.82
NME6	NM_005793	10201	CAGACTTCTCCTAGACATCTA	CAGGATCTAGCCTTCTATCTA	non-metastatic cells 6, protein expressed in (nucleoside-diphosphate kinase)	1.03	0.93
NOD2	NM_022162	64127	CAGTTATGGATTGAAAGTTTA	TAGGCCGTTCCTTCAAAGCAA	nucleotide-binding oligomerization domain containing 2	1.20	0.85
NOS1	NM_000620	4842	CAGAGAATAGTTCACATCTAT	CAGCGGCAATTTGATATCCAA	nitric oxide synthase 1 (neuronal)	0.96	1.02
NOS2A	NM_000625	4843	ATCGAATTTGTCAACCAATAT	CTGGGCCGTGCAAACCTTCAA	nitric oxide synthase 2A (inducible, hepatocytes)	0.76	0.89
NOS3	M95296	4846	CAGGAAGAAGACCTTTAAAGA	CCGGGACTTCATCAACCAGTA	nitric oxide synthase 3 (endothelial cell)	1.07	1.15
NRG2	NM_013982	9542	ACCCGTGGTGGTGGAGGGCAA	CCGAGACATTCGCATCAAATA	neuregulin 2	0.78	0.81
NTF3	NM_002527	4908	AACGGGCAACTCTCCCGTCAA	CAAGCAGATGGTGGACGTTAA	neurotrophin 3	1.18	0.88
P2RX1	NM_002558	5023	ACCATAGGTGACTAAATTAAA	CCGCATGGTGCTGGTGCGTAA	purinergic receptor P2X, ligand-gated ion channel, 1	1.01	1.28
PAK2	NM_002577	5062	CCGCGACCGGATCATACGAAA	CCGGATCATACGAAATCAATT	p21 (CDKN1A)-activated kinase 2	0.89	1.20
PARP1	NM_001618	142	CCCGGTGGCTGTGGTATGAAT	CTTGAAATATGTAGTATTAAA	poly (ADP-ribose) polymerase 1	0.99	0.89
PAWR	NM_002583	5074	CAGAATGAAGCTGTAAACTTA	CGAGAAGATGCAATTACACAA	PRKC, apoptosis, WT1, regulator	1.30	0.96
PAX7	NM_002584	5081	CAGGTTTAGTGAGTTCGATTA	TAGCCGCGTGTCAGAATCAA	paired box gene 7	1.19	0.76
PDCD10	NM_007217	11235	ACGCCTTAATGTGTCATTATA	TACGGATTCAGTTCCAGTTTA	programmed cell death 10	1.32	1.18
PDCD4	NM_014456	27250	CACCATGAGCTTGTATATGAA	CTCAACGTCTGTGCTAATTTA	programmed cell death 4 (neoplastic transformation inhibitor)	1.09	1.03
PDCD5	NM_004708	9141	AAGGTTTAATAGAAATCCTTA	CAGAACAAGGTTTAATAGAAA	programmed cell death 5	1.27	1.00
PDCD6IP	AB037796	10015	CCAGCTTGTTTGCAATTTATA	GAGGTACTTTATACTAACATA	programmed cell death 6 interacting protein	0.90	0.93
PERP	NM_022121	64065	CACGTGAAACTTAACACTTTA	TAGAATGGACTCGGTCTGTTA	PERP, TP53 apoptosis effector	0.92	1.06
PGLYRP2	NM_052890	114770	CACCTGGACAATCTCGTGCTA	CACGTCTATTCAGGAAACCTA	peptidoglycan recognition protein 2	1.13	1.04
PIK3C2G	NM_004570	5288	CCAGATCAAGAAATTCGTAAA	CCCGTAGAAATGATAACTCCA	phosphoinositide-3-kinase, class 2, gamma polypeptide	1.05	0.78
PMAIP1	NM_021127	5366	TGGGCTATATACAGTCCTCAA	TTCGGTCACTACACAACGTAA	phorbol-12-myristate-13-acetate-induced protein 1	0.80	0.92
PML	NM_033238	5371	CAGGGTTGTCCGCTCTGATTA	TGCGGTGAACCGGGAAAGCAA	promyelocytic leukemia	1.04	0.77
POR	S90469	5447	CCGGCTGAAGAGCTACGAGAA	GAGGAACATCATCGTGTTCTA	P450 (cytochrome) oxidoreductase	1.33	0.96
PRF1	NM_005041	5551	CACCGTGTGGGACAATAACAA	CACGGTGGAGTGCCGCTTCTA	perforin 1 (pore forming protein)	1.00	0.87
PRKCZ	NM_002744	5590	CGGAAGCATGACAGCATTAAA	GACCAAATTTACGCCATGAAA	protein kinase C, zeta	1.09	1.13
PSEN1	NM_007318	5663	CAGGCATATCTCATTATGATT	CTGAGGTTGCTTTATCCTAAA	presenilin 1 (Alzheimer disease 3)	0.92	1.01
PTEN	NM_000314	5728	AAGATTTATGATGCACTTATT	CAATTGAGATTCTACAGTAA	phosphatase and tensin homolog (mutated in multiple advanced cancers 1)	0.98	0.99
PTGS1	M59979	5742	CACGCCATTGGTTGGAAGCTA	GAGGTTTGGCATGAAACCCTA	prostaglandin-endoperoxide synthase 1 (prostaglandin G/H synthase and cyclooxygenase)	0.85	0.97
PTK2	NM_005607	5747	CACCTGGGTACTGGTATGGAA	CCGGTCGAATGATAAGGTGTA	PTK2 protein tyrosine kinase 2	0.81	0.89
PTK2B	NM_173176	2185	AAGCTGATCGGCATCATTGAA	CAGGAGAACTTAAAGCCCAAA	PTK2B protein tyrosine kinase 2 beta	0.86	1.01
PTPN6	NM_002831	5777	CCGGAACAAATGCGTCCCATA	TAGGCCCTGATGAGAACGCTA	protein tyrosine phosphatase, non-receptor type 6	0.78	1.16
RAD23B	NM_002874	5887	CTGGGATGACTTGGGCTCATA	TAGGTGTCTAATTACTGTTTA	RAD23 homolog B (S. cerevisiae)	1.31	0.80
RBBP5	NM_005057	5929	ACGGCAGATCGAATAATCAGA	TCCATTTAAACCGAAACTCTA	retinoblastoma binding protein 5	1.33	0.96
RBBP6	AF352051	5930	ACCATATGACATGAAAGCATA	ATAGTGGTTCTTCGTATTCAA	retinoblastoma binding protein 6	0.80	0.79
RBL2	X74594	5934	ACCCTATATTAGGAAACTTTA	CAGTGTTCTAATCGTAAAGAA	retinoblastoma-like 2 (p130)	0.93	1.19
RELA	NM_021975	5970	CCGGATTGAGGAGAAACGTAA	CGGATTGAGGAGAAACGTAAA	v-rel reticuloendotheliosis viral oncogene homolog A, nuclear factor of kappa light polypeptide	1.24	0.95
RHOA	NM_001664	387	ACCTAAGATTACAAATCAGAA	CAAGCTAGACGTGGGAAGAAA	ras homolog gene family, member A	1.03	0.91

Gene Symbol	Transcript Reference Number	Gene ID	Target siRNA 1	Target siRNA 2	Gene Name	Relative viability (siNegative normalized)	
						siRNA 1	siRNA 2
SEMA4D	NM_006378	10507	ACGGATAGCAAGAGTGTGCAA	CCGGCACATCCTGCGAACCAA	sema domain, immunoglobulin domain (Ig), transmembrane domain (TM) and short	0.97	1.17
SFRP1	NM_003012	6422	AAGGGCCATTTAGATTAGGAA	CAGCCTGCAGGGAATAACATA	secreted frizzled-related protein 1	1.24	0.98
SFRP5	NM_003015	6425	CCGCTGGGACAAGAAGAATAA	CTGCGAGGAGTACGACTACTA	secreted frizzled-related protein 5	0.81	1.00
SNCA	NM_000345	6622	CATGCTTATAAGCAACATGAA	CCCAGTCATGACATTTCTCAA	synuclein, alpha (non A4 component of amyloid precursor)	0.77	1.00
SOD1	NM_000454	6647	ATGGCACTTATTATGAGGCTA	CCAGTGCAGGTCCTCACTTTA	superoxide dismutase 1, soluble (amyotrophic lateral sclerosis 1 (adult))	0.96	1.07
STK17A	NM_004760	9263	AACCAGGATATTTAACAGGTA	TCCATTGTAACCGAAGAGTTA	serine/threonine kinase 17a (apoptosis-inducing)	0.78	1.20
TDP2	NM_016614	51567	ACCGAAATAGTTAATATTTGA	CGGAACGAATGAATCAGTTAA	tyrosyl-DNA phosphodiesterase 2	1.12	1.42
TFDP1	NM_007111	7027	CCGGATCTGGTAACATGGCAA	CGGCGCGTCTACGATGCCTTA	transcription factor Dp-1	1.03	1.23
TIA1	NM_022173	7072	AACGATTTGGGAGGTAGTGAA	CTGGGCTAACAGAACAACTAA	TIA1 cytotoxic granule-associated RNA binding protein	0.81	0.86
TIAF1	NM_004740	9220	AGGAGTGTCATTTGTCCCTAA	TACCCGATGTATGGAAATAAA	TGFB1-induced anti-apoptotic factor 1	1.01	0.76
TNF	NM_000594	7124	CCGACTCAGCGCTGAGATCAA	TAGGGTCGGAACCCAAGCTTA	tumor necrosis factor (TNF superfamily, member 2)	0.93	1.07
TNFAIP6	NM_007115	7130	AAGGCGGTGTGTGAATTTGAA	TGGCGTCTTTACAGATCCAAA	tumor necrosis factor, alpha-induced protein 6	1.16	1.14
TNFRSF14	NM_003820	8764	CACCTACATTGCCCACCTCAA	CTGCTGGAGTTCATCCTGCTA	tumor necrosis factor receptor superfamily, member 14	0.80	0.77
TNFRSF17	NM_001192	608	AACCATTAAAGGACGAGTTTA	CTCGAGCAGTGCCACTTTAAA	tumor necrosis factor receptor superfamily, member 17	1.18	1.01
TNFRSF1A	NM_001065	7132	ACCGGCATTATTGGAGTGAAA	CCCGGTGACTGTCCCAACTTT	tumor necrosis factor receptor superfamily, member 1A	1.02	1.02
TNFRSF1B	NM_001066	7133	AGGGTGATAAATTGTTGATAA	CAGCCTTGGGTCTACTAATAA	tumor necrosis factor receptor superfamily, member 1B	1.07	0.95
TNFRSF4	NM_003327	7293	ACGGGAGTCTCCGACCGGCAA	CAGCAATAGCTCGGACGCAAT	tumor necrosis factor receptor superfamily, member 4	0.97	0.91
TNFRSF8	NM_001243	943	AAGGACTCTCTCACACAGGAA	ACCCATATCAAGGGTGACTAA	tumor necrosis factor receptor superfamily, member 8	1.14	0.97
TNFSF12	NM_003809	8742	CAGGGCATTGTGTTCACTGTA	TTAAATAGAATAAGTCATAAA	tumor necrosis factor (ligand) superfamily, member 12	0.99	0.88
TNFSF13	NM_003808	8741	AGCGCAGGTGTCTTCCATTTA	CCGGGCAAGGGCGAAACTTAA	tumor necrosis factor (ligand) superfamily, member 13	1.05	0.90
TNFSF14	NM_003807	8740	CAGAAAGTCCTCAGTCGATAT	CAGCCCAGGAGTGTTGAGCAA	tumor necrosis factor (ligand) superfamily, member 14	1.19	1.22
TNFSF15	NM_005118	9966	ACCGAATGAACTATACCAACA	TCGGGTCAGGATTGAAAGAGA	tumor necrosis factor (ligand) superfamily, member 15	0.84	0.99
TNFSF8	NM_001244	944	CACGGAGCACACCAATAACAA	TCCGGCAGGGATGATTGTCAA	tumor necrosis factor (ligand) superfamily, member 8	0.88	1.07
TNFSF9	NM_003811	8744	ATCCGCTATCACAGCTTTCAA	TCCGTGGGTTCTGTCATGTAA	tumor necrosis factor (ligand) superfamily, member 9	1.03	1.07
TP53I11	AK054815	9537	CCCGTTAGTCCCTGATCCCAA	TAGGCAATGAAATCAAGTTTA	tumor protein p53 inducible protein 11	0.90	1.01
TP73	NM_005427	7161	ATCGAGTATTTCACCTCCCAA	CCCGGGATGCTCAACAACCAT	tumor protein p73	0.95	1.35
TRADD	NM_003789	8717	CCCGAATGTTAAGCAATGATA	CCGAATGTTAAGCAATGATAA	TNFRSF1A-associated via death domain	1.12	0.94
TRAF5	NM_004619	7188	AGGGTACTTGCTATAATGGAA	CTGGAGGGTACTTGCTATAAT	TNF receptor-associated factor 5	0.92	1.10
TRAF6	NM_004620	7189	AGGGATGAGTTTAAACCCTAA	CAGGGATGAGTTTAAACCCTA	TNF receptor-associated factor 6	0.99	0.79
TXNL1	NM_004786	9352	ACAGAGGATGATATTAAAGAA	CTGACAGAGGATGATATTAAA	thioredoxin-like 1	1.22	1.32
VEGFC	NM_005429	7424	ACCCTTGTGGGTCCATGCTAA	TTGCTGCAGCACATTATAATA	vascular endothelial growth factor C	0.88	1.48
YWHAB	NM_003404	7529	CAGCAATATGTTCACTATGTT	CTGGATAATACCTTTAAGAAT	tyrosine 3-monooxygenase/tryptophan 5-monooxygenase activation protein, beta polypeptide	0.84	0.96
YWHAE	NM_006761	7531	AGAGAGGTTAATCACACTATA	CTGAAGCAGGTTAGCGTTGAA	tyrosine 3-monooxygenase/tryptophan 5-monooxygenase activation protein, epsilon	0.80	1.05
YWHAQ	NM_006826	10971	CAAACGATAGATAATTCCCAA	CTGGAATTGTTGGATAAATAT	tyrosine 3-monooxygenase/tryptophan 5-monooxygenase activation protein, theta polypeptide	1.23	0.79
ZNF443	NM_005815	10224	TCGGTCATTATGATAACTTAA	TTCGGTCATTATGATAACTTA	zinc finger protein 443	0.83	1.08
