# Supplementary figures and images for: Systems-wide RNAi analysis of CASP8AP2/FLASH shows transcriptional deregulation of the replication-dependent histone genes and extensive effects on the transcriptome of colorectal cancer cells
Source: Mol Cancer. 2012 Jan 4;11:1. doi: 10.1186/1476-4598-11-1 (PMC3281783; doi:10.1186/1476-4598-11-1)

**A**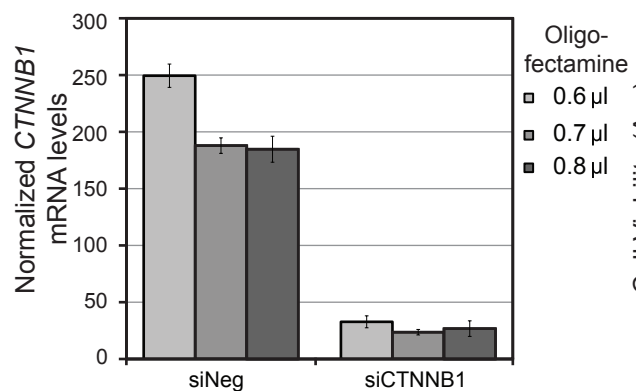**B**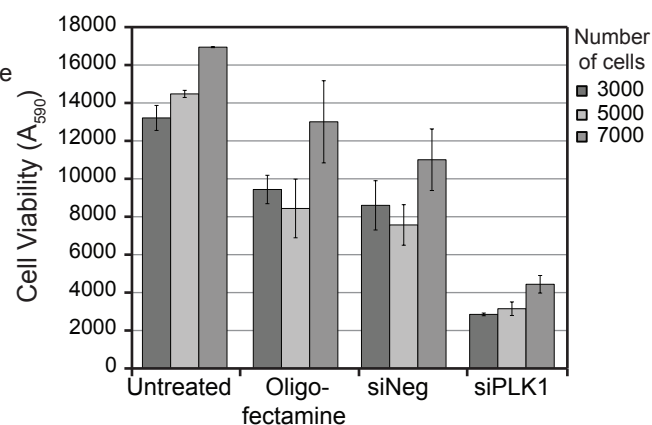**C**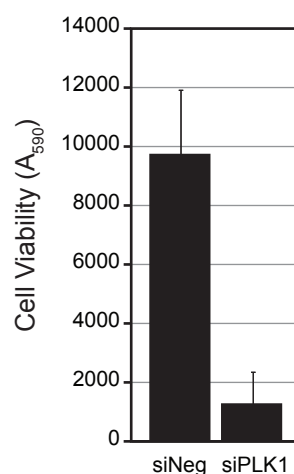**D**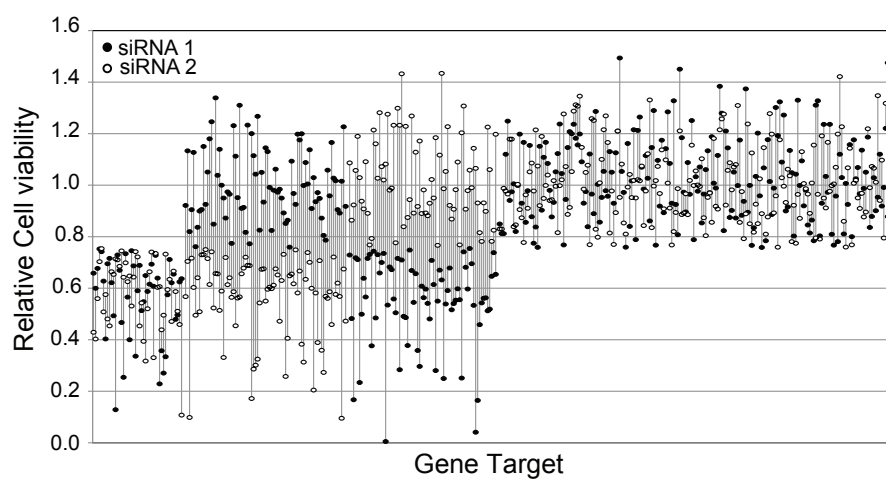**E**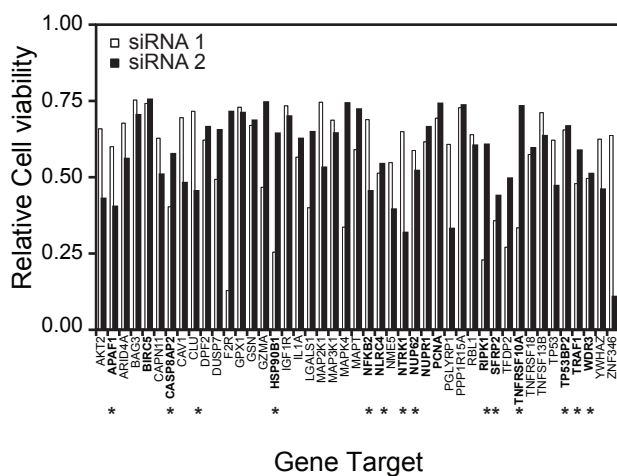

Supplement: Additional file 2 — Figure S1. Optimization, execution and follow up of a siRNA based RNAi screen of genes associated with apoptosis. (A) Confirmation of Oligofectamine (Invitrogen) transfection conditions using CTNNB1 mRNA expression as an end-point. (B) Confirmation of cell line number and transfection conditions using an siRNA corresponding to PLK1 and cell viability as an end-point. (C) Summary of siRNA screening controls. Data is shown as the viability of cells transfected by a negative control siRNA (siNeg; mean and standard deviation of 33 wells) and a positive control siRNA (siPLK1; mean and standard deviation of 11 wells). On average, silencing of PLK1 induced an over 85% decrease in cell viability. (D) Summary of screening data with gene targets ranked as shown in Additional File 1, Table S1, both siRNAs ≥25% decrease in cell viability, siRNA.1 ≥25% decrease in cell viability, siRNA.2 ≥25% decrease in cell viability, both siRNAs. ≤25% decease in cell viability. Closed circles indicate data for siRNA and open circles data for siRNA 2. (E) Two different siRNAs corresponding to 45 genes induced a 25% or greater reduction in the viability of SW480 cells compared to siNegative-transfected cells. Data is expressed relative to siNegative-transfected cells; gene targets are ranked alphabetically. Genes chosen for initial validation are marked with *. (F) Reproducibility of the effects of silencing selected genes identified as reducing the viability of SW480 cells. Data is shown as the mean ± SD of three independent transfections for each siRNA targeting the fifteen genes of interest normalized to the values from negative control siRNA transfected cells. [file 1476-4598-11-1-S2.PDF]

**A**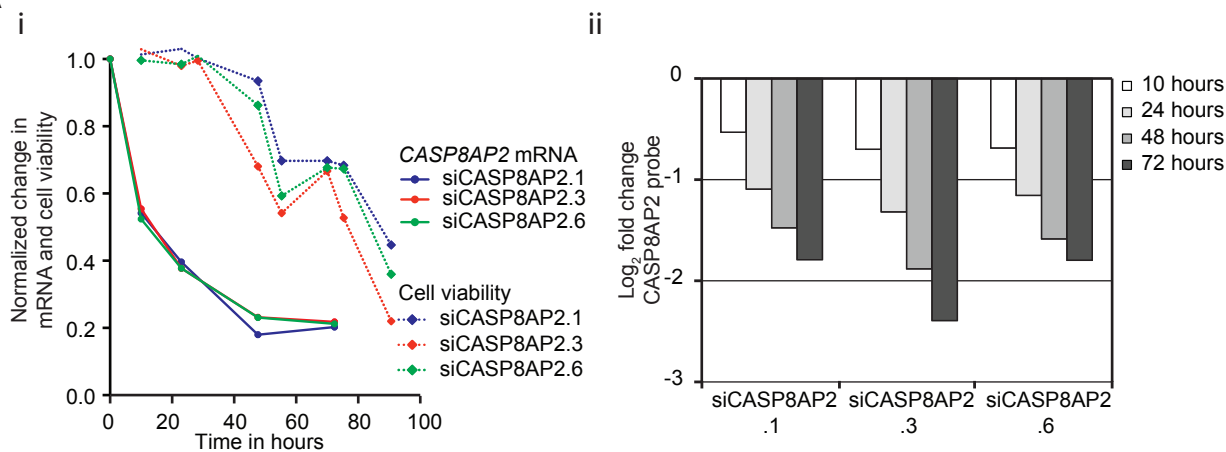**B**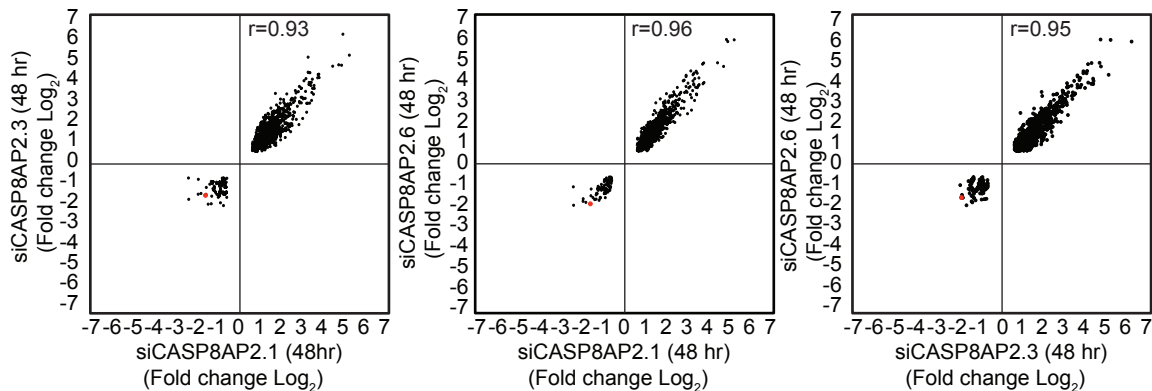**C**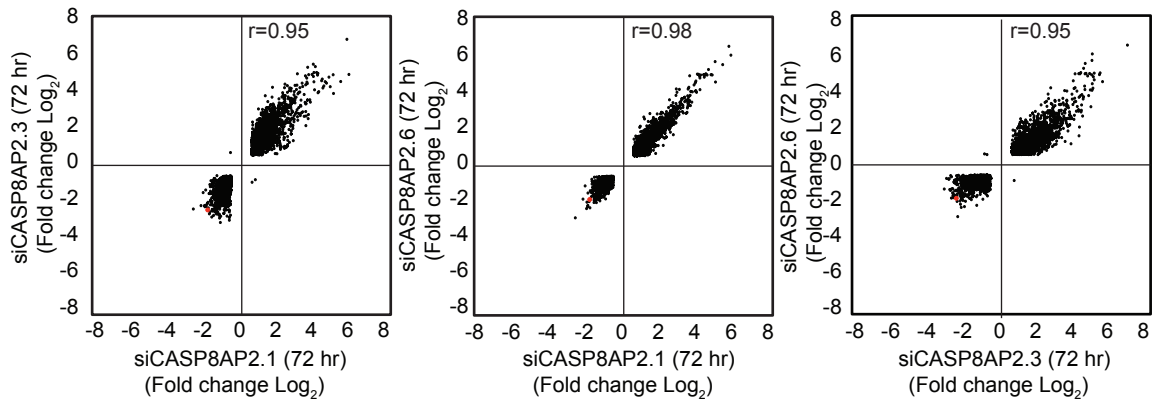**D**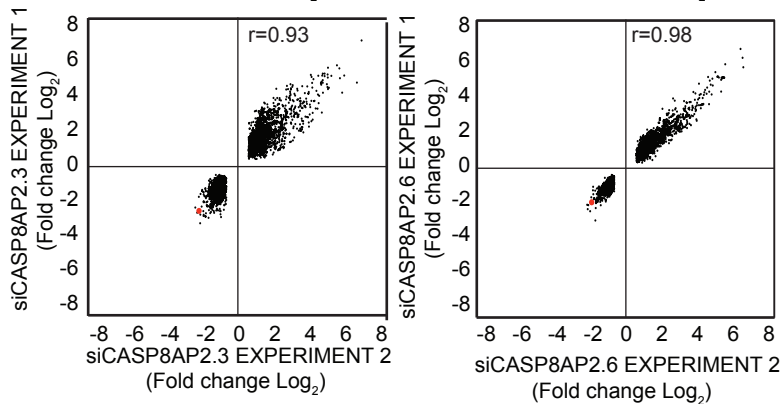

Supplement: Additional file 10 — Figure S4. The development of CASP8AP2/FLASH RNAi signatures in SW480 cells over time. (A) Reduction in CAPS8AP2/FLASH mRNA measured by (i) qRT-PCR and (ii) array analysis and cell viability in SW480 cells over time. All data is normalized to the levels of CAPS8AP2/FLASH and viability observed in siNeg transfected SW480 cells. Correlation of the fold change in expression seen following silencing with three different CASP8AP2/FLASH siRNAs at (B) 48 hours and (C) 72 hours compared to siNeg transfected SW480 cells. Each black circle corresponds to a single probe. A red circle indicates the probe corresponding to CASP8AP2/FLASH. Using the data obtained at 48 and 72 hours we observed no off-target sequence alignments between transcripts showing a decrease in expression for all three siRNAs and the sequences of the three different CASP8AP2/FLASH siRNAs though nine genes showed off-target alignments with two of three siRNAs (data not shown). To assess the reproducibility of our initial findings we examined the expression of genes that previously showed differential expression following transfection of siCASP8AP2.3 and siCASP8AP2.6 siRNAs, with data obtained at the same time point (72 hours) with same siRNAs as part of this follow up study. The data for ~2000 probes were compared. (D) Correlation of the fold change in expression seen in two different experiments in which CASP8AP2/FLASH has been silenced for 72 hours. Each black circle corresponds to a single probe. [file 1476-4598-11-1-S10.PDF]
