# Supplementary material for: Systems-wide RNAi analysis of CASP8AP2/FLASH shows transcriptional deregulation of the replication-dependent histone genes and extensive effects on the transcriptome of colorectal cancer cells
Source: Mol Cancer. 2012 Jan 4;11:1. doi: 10.1186/1476-4598-11-1 (PMC3281783; doi:10.1186/1476-4598-11-1)
Supplement: Additional file 3 — Table S2. The sequences of gene specific siRNAs. [file 1476-4598-11-1-S3.PDF]

Additional file 3, Table S2

Gene/siRNA	Target sequence 5' – 3'	Additional information
CAPS8AP2/FLASH		
siCASP8AP2.1:	AACGAAGTTTCGAGATTCTTA	Fig. 1 siRNA B<br>Screen target siRNA.1
siCASP8AP2.3:	CAGCTGATGTGCGGAAGTCAA	Fig. 1 siRNA A<br>Screen siRNA 2
siCASP8AP2.6	CACATACGTAGATCTAACGAA	Fig. 1 siRNA C
NUP62		
siNUP62.2	CTGGAGAGCCTGATCAACAAA	Fig. 1 siRNA A
siNUP62.3	CCGCGAGGTGGAGAAGGTGAA	Fig. 1 siRNA B
siNUP62.5	ACCGGCTTTGCCTTGAATTTA	Fig. 1 siRNAC<br>Screen siRNA 1
NTRK1		
siNTRK1.1	CGAGAGCATCCTGTACCGTAA	Fig. 1 siRNA A<br>Screen siRNA 2
siNTRK1.5	ACATCATCGAGAACCCACAA	Fig. 1 siRNA B<br>Overlap with screen siRNA 1
siNTRK1.6	CTGGGAGTGGTTAGCCGGAAT	Fig. 1 siRN C
WDR3		
siWDR3.1	CCGGGATGTTATCGGCTTCAA	Fig. 1 siRNA A<br>Screen siRNA 1
siWDR3.4<br><br>siWDR3.5	CCAGCGGGTGACTAATATAAA<br><br>TCCGCTGATAGGAATGTGAAA	Screen siRNA 2<br>Fig. 1 siRNA B<br>Fig. 1 siRNA C
TRAF1		
siTRAF1.6<br><br>siTRAF1.7<br>si.TRAF1.4	CAGACTGATCAGGACCTCCAA<br><br>CCCGAGGAATGGCGAGGATCA<br>ATCGTTAGGGTTGCTTCTATA	Fig. 1 siRNA B<br>Screen siRNA 2<br>Fig. 1 siRNA C<br>Fig. 1 siRNA A
PLK1		
siPLK1.7:	CGCGGGCAAGATTGTGCCTAA	
