# Supplementary material for: Systems-wide RNAi analysis of CASP8AP2/FLASH shows transcriptional deregulation of the replication-dependent histone genes and extensive effects on the transcriptome of colorectal cancer cells
Source: Mol Cancer. 2012 Jan 4;11:1. doi: 10.1186/1476-4598-11-1 (PMC3281783; doi:10.1186/1476-4598-11-1)
Supplement: Additional file 4 — Table S3. The sequences of gene specific PCR primers. [file 1476-4598-11-1-S4.PDF]

Additional file 4, Table S3

**Gene**	**Primer Forward 5' – 3'**	**Primer Reverse 5' – 3'**
CAPS8AP2/FLASH	AGTGAGGCTAAAAGTGAAGGTA	TGAACTGGGAGATTCTGTGG
NUP62	CCTCGGCTGAAGAAAGAA	TGTAGCAGGTGTGGTTGTTG
YWHAZ	ACTTTTGGTACATTGTGGCTTCAA	CCGCCAGGACAAACCAGTAT
HIST1H2BD<br>non-polyA *HIST1H2BD* variant, NM_021063<br>polyA *HIST1H2BD* variant, NM_138720	ACCATCACCTCCAGGGAGA	 TTTATTGAAAACATGCGTGGCTC<br>GAGACTTCCTAAGTTCTATGTATA
ß-actin	CAATGAGCTGCGTGTGGCT	GAGTCCATCACGATGCCAGT
