# Supplementary material for: Systems-wide RNAi analysis of CASP8AP2/FLASH shows transcriptional deregulation of the replication-dependent histone genes and extensive effects on the transcriptome of colorectal cancer cells
Source: Mol Cancer. 2012 Jan 4;11:1. doi: 10.1186/1476-4598-11-1 (PMC3281783; doi:10.1186/1476-4598-11-1)
Supplement: Additional file 5 — Table S4. Transcription factor data sets used for gene set enrichment analysis. [file 1476-4598-11-1-S5.PDF]

Additional file 5, Table S4

Transcription Factor	Method	Tissue Type	Number of Target Genes	Reference
VDR	Gene expression; TFBS; gel shift: ChIP	Tongue squamous tumor (SCC25)	576	Wang TT, Tavera-Mendoza LE, Laperriere D, Libby E, MacLeod NB, Nagai Y, Bourdeau V, Konstorum A, Lallemant B, Zhang R, Mader S, White JH: **Large-scale in silico and microarray-based identification of direct 1,25-dihydroxyvitamin D3 target genes.** *Mol Endocrinol* 2005, **19:**2685-95.
SMAD4	ChIP-chip	Human keratinocytes (HaCaT)	396	Koinuma D, Tsutsumi S, Kamimura N, Imamura T, Aburatani H, Miyazono K: **Promoter-wide analysis of Smad4 binding sites in human epithelial cells.** *Cancer Sci* 2009 **100:**2133-42
CTNNB1	ChIP-Seq	Colorectal cancer (HCT-116)	985	Bottomly D, Kyler SL, McWeeney SK, Yochum GS: **Identification of {beta}-catenin binding regions in colon cancer cells using ChIP-Seq.** *Nucleic Acids Res* 2010 **38:** 5735-45
TCF7L2	ChIP-Chip	Colorectal cancer (LS174T)	2119	Hatzis P, van der Flier LG, van Driel MA, Guryev V, Nielsen F, Denissov S, Nijman IJ, Koster J, Santo EE, Welboren W, Versteeg R, Cuppen E, van de Wetering M, Clevers H, Stunnenberg HG: **Genome-wide pattern of TCF7L2/TCF4 chromatin occupancy in colorectal cancer cells.** *Mol Cell Biol*, 2008, **28**: 2732-44
MYC (up-regulated)	ChIP-PET; gene expression	Human B lymphoid tumor	985	Zeller KI, Zhao X, Lee CW, Chiu KP, Yao F, Yustein JT, Ooi HS, Orlov YL, Shahab A, Yong HC, Fu Y, Weng Z, Kuznetsov VA, Sung WK, Ruan Y, Dang CV, Wei CL. **Global mapping of c-Myc binding sites and target gene networks in human B cells.** *Proc Natl Acad Sci USA*, 2006 **103**: 17834-9
MYC (down-regulated)	ChIP-PET; gene expression	Human B lymphoid tumor	1431	
NF-kB	Literature curated	Various	421	http://people.bu.edu/gilmore/nf-kb/target/index.html
