# Supplementary material for: Systems-wide RNAi analysis of CASP8AP2/FLASH shows transcriptional deregulation of the replication-dependent histone genes and extensive effects on the transcriptome of colorectal cancer cells
Source: Mol Cancer. 2012 Jan 4;11:1. doi: 10.1186/1476-4598-11-1 (PMC3281783; doi:10.1186/1476-4598-11-1)
Supplement: Additional file 6 — Figure S2. The development of a CASP8AP2/FLASH RNAi signature in SW480 cells. (A) Correlation of the fold change in expression seen following silencing with two different CASP8AP2/FLASH siRNAs. Each black circle corresponds to a single probe. A red circle indicates the probe corresponding to CASP8AP2/FLASH. There was a high concordance between the effects mediated by both siRNAs targeting CASP8AP2/FLASH, only two probes showed discordant changes with respect to the direction of change in that one siRNA induced an increase in expression, while the second siRNA induced a decrease in expression. (B) Within the very large CASP8AP2/FLASH expression profile we identified only one transcript (ABLIM2) that showed a potential mismatch with both CASP8AP2 siRNAs. The probe corresponding to ABLIM2 was ranked as the 349th down-regulate/d probe (out of a total of 1487 downregulated probes; ranked ~Log2 -2.0 to Log2 -0.6 fold change) within the CASP8AP2/FLASH expression profile. As this change is likely to have had only a minimal affect on the CASP8AP2/FLASH expression profile as a whole the data for this gene was retained within our subsequent analysis. [file 1476-4598-11-1-S6.PDF]

A

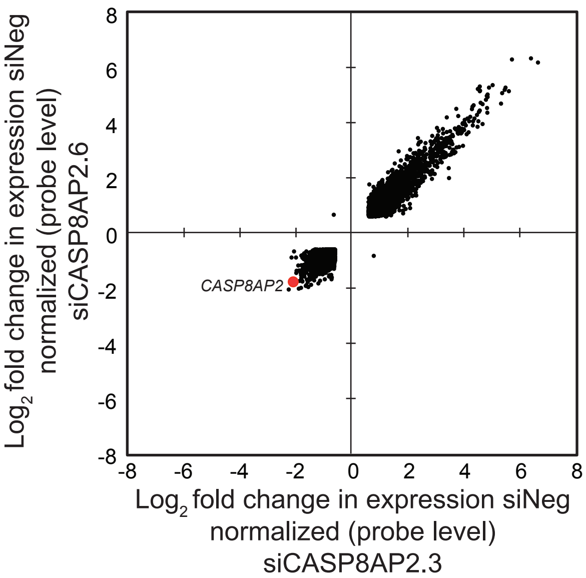

B

<table>
<tr><th>Gene</th><th>siRNA</th><th>M</th><th>q</th><th>Accession</th><th>Alignment Length</th><th>Alignment</th><th>Identity</th><th>Energy</th><th>Score</th><th>3' Start</th><th>3' Stop</th></tr>
<tr><td>ABLIM2</td><td>siCASP8AP2.3</td><td>-1.06</td><td>0.001</td><td>NM_001130088</td><td>20</td><td>gTCGACTACACGCCTTCAGTT
 :|||| | |:||||||||:
gGGCTGCCGGGTGGAAGTCAG</td><td>70</td><td>-25.9</td><td>99</td><td>295</td><td>315</td></tr>
<tr><td>ABLIM2</td><td>siCASP8AP2.6</td><td>-1.01</td><td>0.002</td><td>NM_001130088</td><td>19</td><td>gtGTATGCATCTAGATTGCTT
  || ::||: |||||||:
gcCA-GTGTG---CTAACGAG</td><td>57.9</td><td>-14.19</td><td>74</td><td>185</td><td>201</td></tr>
</table>
