# Supplementary material for: Systems-wide RNAi analysis of CASP8AP2/FLASH shows transcriptional deregulation of the replication-dependent histone genes and extensive effects on the transcriptome of colorectal cancer cells
Source: Mol Cancer. 2012 Jan 4;11:1. doi: 10.1186/1476-4598-11-1 (PMC3281783; doi:10.1186/1476-4598-11-1)
Supplement: Additional file 7 — Table S5. CASP8AP2/FLASH RNAi signature in SW480 cells. Probes that showed a significant fold change (> Log2 ± 0.6, q < 0.05) in CASP8AP2/FLASH silenced cells compared to siNeg transfected SW480 cells are listed, ranked by the fold change seen in siCASP8AP2.3-silenced cells. The probe corresponding to CASP8AP2/FLASH is highlighted in grey. The columns show: (1) The gene sequence reference, (2) the gene symbol, (3) the chromosomal position of the Agilent array probe, (4) the Agilent array probe identifier, (5) fold change siNeg vs. siCASP8AP2.3 Log2 transformed, (6) fold change siNeg vs. siCASP8AP2.6 Log2 transformed, (7) FDR (q-value), siNeg vs. siCASP8AP2.3 and (8) FDR (q-value), siNeg vs. siCASP8AP2.6. [file 1476-4598-11-1-S7.PDF]

Gene	Symbol	Chromosomal position	Probe	siCASP8AP2.3	CASP8AP2.6	siCASP8AP2.3	CASP8AP2.6
				M	M	Q	Q
AK055441	OSGEPL1	chr2:190437014-190436955	A_24_P302574	-2.26	-2.05	8.37E-14	2.16E-12
NM_013240	HEMK2	chr21:29172476-29172417	A_23_P80086	-2.16	-0.89	2.58E-19	8.32E-07
NM_033138	CALD1	chr7:134110580-134110639	A_23_P42575	-2.07	-0.67	1.34E-14	2.74E-03
**NM_012115**	**CASP8AP2**	**chr6:90640368-90640427**	**A_23_P58898**	**-2.06**	**-1.81**	**6.28E-15**	**6.14E-13**
NM_198076	FAM36A	chr1:241331562-241332403	A_32_P106646	-2.00	-0.89	6.38E-20	6.40E-08
NM_198076	FAM36A	chr1:241333642-241333701	A_24_P79712	-1.99	-1.45	9.44E-15	3.76E-10
NM_020425	C6orf162	chr6:88107988-88108047	A_24_P388940	-1.98	-1.37	9.11E-17	3.49E-11
AK094603	AK094603	chr4:186882459-186882398	A_24_P912136	-1.92	-1.18	1.18E-07	7.86E-04
NM_003517	HIST2H2AC	chr1:146671936-146671995	A_23_P301247	-1.90	-2.02	8.19E-10	1.36E-10
AK055214	AK055214	chr3:190090520-190090579	A_24_P778741	-1.89	-1.36	5.65E-09	1.07E-05
NM_022353	OSGEPL1	chr2:190444225-190444166	A_23_P210081	-1.87	-1.74	1.06E-07	5.88E-07
NM_024641	MANEA	chr6:96163497-96163556	A_23_P255663	-1.83	-1.44	5.48E-09	1.74E-06
NM_080650	ATPBD4	chr15:33461322-33453050	A_24_P56557	-1.82	-1.99	3.05E-11	1.53E-12
NM_006105	RAPGEF3	chr12:46419194-46418747	A_23_P151307	-1.82	-1.53	4.28E-07	1.49E-05
NM_152641	ARID2	chr12:44587680-44587739	A_32_P74955	-1.82	-1.47	5.09E-17	1.40E-13
BX647543	BX647543	chr11:128296150-128296209	A_32_P90812	-1.81	-1.63	1.07E-06	9.65E-06
BC004287	BC004287	chr8:81703765-81703706	A_32_P229493	-1.81	-1.43	1.59E-06	1.18E-04
NM_017421	COQ3	chr6:99930589-99926178	A_23_P111228	-1.81	-1.41	1.54E-06	1.51E-04
NM_005096	ZMYM3	chrX:70242506-70242477	A_23_P137073	-1.81	-1.17	3.54E-06	2.74E-03
NM_015655	ZNF337	chr20:25603624-25603565	A_24_P318939	-1.81	-0.82	2.85E-08	9.77E-03
ENST00000369183	ENST00000369183	chr10:120055518-120055459	A_32_P10123	-1.79	-0.82	1.32E-10	1.17E-03
NM_052862	RCSD1	chr1:164406639-164406698	A_23_P23279	-1.78	-1.74	4.16E-04	7.13E-04
NM_032900	ARHGAP19	chr10:98972775-98972716	A_23_P1387	-1.77	-1.76	6.20E-11	7.80E-11
NM_016835	MAPT	chr17:41461336-41461395	A_24_P224488	-1.77	-1.36	2.30E-10	2.35E-07
NM_014020	LR8	chr7:149926856-149926797	A_23_P157007	-1.77	-1.31	5.99E-06	7.80E-04
NM_001001552	LEMD1	chr1:202082480-202082421	A_24_P696761	-1.77	-0.90	2.47E-07	7.43E-03
AK022110	AK022110	chr5:54498809-54498868	A_24_P876522	-1.73	-1.16	1.94E-06	1.16E-03
ENST00000260257	ENST00000260257	chr11:111251783-111251724	A_23_P362261	-1.72	-1.46	4.53E-11	5.09E-09
THC2315901	THC2315901	chr3:159745548-159745607	A_23_P124313	-1.72	-0.95	1.15E-07	2.49E-03
A_32_P171043	A_32_P171043	chr18:055249306-055249247	A_32_P171043	-1.71	-1.35	3.95E-04	6.47E-03
NM_001948	DUT	chr19:057646905-057646964	A_24_P160874	-1.71	-0.90	4.93E-08	2.87E-03
ENST00000333926	ENST00000333926	chr10:59718642-59718701	A_24_P38754	-1.71	-0.74	7.98E-10	4.13E-03
NM_175605	IFT88	chr13:20135644-20135703	A_23_P48339	-1.70	-1.58	4.66E-14	5.76E-13
NM_001483	GBAS	chr7:55841456-55841515	A_23_P82674	-1.69	-1.47	1.64E-08	4.68E-07
NM_014744	TBC1D5	chr3:17175042-17174983	A_23_P355455	-1.69	-1.37	4.79E-15	7.35E-12
NM_016371	HSD17B7	chr1:159504977-159505767	A_32_P52282	-1.68	-1.21	2.22E-05	2.50E-03
NM_153234	LIX1	chr5:96453411-96453352	A_32_P116606	-1.68	-1.15	3.55E-05	5.19E-03
NM_005328	HAS2	chr8:122695327-122695268	A_23_P10206	-1.67	-1.26	2.72E-10	4.68E-07
NM_003262	TLOC1	chr3:171198708-171198767	A_23_P357856	-1.66	-0.82	6.06E-07	1.38E-02
A_24_P213336	A_24_P213336	chr15:075058637-075058578	A_24_P213336	-1.65	-1.92	1.24E-02	5.18E-03
AY173948	GNPDA2	chr4:44545045-44544986	A_32_P19608	-1.65	-0.96	8.30E-12	8.82E-06
AK055214	AK055214	chr3:190089354-190089413	A_32_P39049	-1.64	-1.03	1.79E-05	8.23E-03
BC035691	GSR	chr8:30655867-30655808	A_32_P31618	-1.64	-0.98	2.21E-04	3.75E-02
NM_178493	NOTUM	chr17:77507817-77506653	A_23_P101007	-1.63	-1.89	1.18E-03	2.32E-04
NM_152891	PRSS33	chr16:2774313-2774254	A_23_P89003	-1.63	-1.44	5.97E-04	3.02E-03
AV749257	AV749257	chr12:20598082-20598023	A_32_P16625	-1.63	-0.67	4.26E-07	4.32E-02
BX648681	MGC40579	chr3:143387476-143387282	A_24_P362805	-1.62	-1.03	5.16E-11	4.71E-06
AF034174	AF034174	chr6:34362966-34362907	A_24_P123632	-1.62	-0.96	5.72E-06	7.98E-03
NM_001049	SSTR1	chr14:37751725-37751784	A_24_P244706	-1.61	-1.69	5.93E-03	5.36E-03
ENST00000366932	ENST00000366932	chr1:214899497-214899556	A_32_P221822	-1.61	-1.05	9.25E-12	1.07E-06
A_24_P706752	A_24_P706752	chr4:110989040-110988981	A_24_P706752	-1.61	-0.95	5.90E-07	2.77E-03
NM_021069	SORBS2	chr4:186890189-186886030	A_23_P121795	-1.60	-1.10	1.14E-06	6.75E-04
NM_014744	TBC1D5	chr3:17177221-17177162	A_24_P289029	-1.58	-1.78	3.24E-10	8.07E-12
NM_017617	NOTCH1	chr9:136665660-136665601	A_23_P60387	-1.57	-0.99	2.76E-05	9.59E-03
NM_018487	HCA112	chr7:149939676-149939735	A_23_P252082	-1.56	-1.96	1.04E-03	5.74E-05
NM_176815	DHFRL1	chr3:95259894-95259835	A_24_P186065	-1.55	-1.33	2.04E-09	1.10E-07

Gene	Symbol	Chromosomal position	Probe	siCASP8AP2.3	CASP8AP2.6	siCASP8AP2.3	CASP8AP2.6
				M	M	Q	Q
NM_004613	TGM2	chr20:36191326-36191267	A_32_P86763	-1.55	-1.31	3.08E-03	1.69E-02
CR624291	CR624291	chr6:34355721-34355662	A_24_P726215	-1.55	-1.30	3.57E-13	1.19E-10
THC2312748	THC2312748	chr2:39617675-39617734	A_32_P156564	-1.54	-1.17	5.69E-11	1.14E-07
BC048201	BC048201	chr21:14438397-14438456	A_32_P134427	-1.54	-0.91	2.10E-05	1.41E-02
NM_003130	SRI	chr7:87479989-87479930	A_23_P59718	-1.53	-1.52	7.25E-10	9.45E-10
NM_020425	C6orf162	chr6:88106555-88106614	A_23_P59255	-1.53	-1.23	1.47E-11	7.75E-09
ENST00000382327	ENST00000382327	chr5:14758088-14758029	A_24_P303145	-1.53	-1.17	9.04E-08	2.30E-05
NM_014186	COMMD9	chr11:36252399-36252340	A_23_P87323	-1.53	-1.06	2.94E-08	5.53E-05
AB002330	SR140	chr3:144261358-144261417	A_24_P943106	-1.53	-1.04	2.36E-13	3.06E-08
THC2233630	THC2233630	chr3:180803154-180803213	A_23_P425492	-1.53	-0.72	1.47E-08	5.73E-03
NM_018369	DEPDC1B	chr5:59929057-59928998	A_23_P361419	-1.52	-1.33	6.24E-09	1.85E-07
NM_145011	ZNF25	chr10:38279844-38279785	A_24_P69691	-1.52	-0.83	2.88E-15	3.14E-07
BC035184	BC035184	chr18:35041667-35041608	A_32_P72181	-1.51	-1.62	1.94E-06	5.14E-07
BX107298	BX107298	chr11:43844957-43845016	A_32_P115258	-1.51	-1.25	3.24E-12	1.20E-09
NM_005578	LPP	chr3:190078444-190078503	A_24_P114551	-1.51	-1.16	1.37E-08	6.05E-06
NM_017617	NOTCH1	chr9:136665660-136665601	A_23_P60387	-1.51	-1.10	6.16E-05	3.99E-03
NM_012393	PFAS	chr17:8113762-8113821	A_23_P402610	-1.51	-1.07	1.54E-10	1.00E-06
ENST00000328681	ENST00000328681	chr1:161552634-161552693	A_24_P173823	-1.51	-0.96	1.75E-08	1.70E-04
AL711212	AL711212	chr15:99213721-99213780	A_32_P3527	-1.50	-1.14	2.10E-07	4.86E-05
AK074383	LOC200169	chr1:232037617-232037558	A_32_P305020	-1.50	-0.70	4.61E-06	3.86E-02
NM_005687	FARSLB	chr2:223314566-223314507	A_23_P165355	-1.49	-1.69	5.72E-06	4.96E-07
NM_016371	HSD17B7	chr1:159504957-159505747	A_23_P11859	-1.49	-1.18	3.33E-05	1.07E-03
AK093643	AK093643	chr13:46249338-46249279	A_24_P841662	-1.49	-1.00	1.61E-10	3.56E-06
NM_003838	FPGT	chr1:74384646-74384705	A_23_P200030	-1.48	-1.24	1.47E-07	6.40E-06
AB037774	ZMYM6	chr1:35122041-35121982	A_24_P25326	-1.48	-0.91	3.60E-10	2.83E-05
NM_004923	MTL5	chr11:68231586-68231527	A_23_P161507	-1.48	-0.78	7.79E-07	8.85E-03
NM_022553	VPS52	chr6:33327376-33327317	A_23_P70583	-1.48	-0.73	3.86E-09	2.08E-03
NM_000318	PXMP3	chr8:78058387-78058328	A_23_P31702	-1.47	-1.40	7.69E-09	2.80E-08
NM_078474	TM2D3	chr15:100007737-100004574	A_24_P193498	-1.47	-1.22	6.60E-05	1.09E-03
NM_015202	KIAA0556	chr16:27698597-27698656	A_23_P381203	-1.47	-0.85	3.45E-07	2.42E-03
NM_015039	NMNAT2	chr1:179949497-179949438	A_23_P354908	-1.47	-0.84	2.26E-05	1.78E-02
U79275	HSU79275	chr12:46414781-46414722	A_32_P393316	-1.46	-1.88	1.47E-04	2.25E-06
NM_003546	HIST1H4L	chr6:27948987-27948928	A_23_P70480	-1.46	-1.59	3.81E-02	3.28E-02
NM_016147	PPME1	chr11:73642648-73642707	A_23_P64567	-1.46	-1.11	2.40E-06	2.85E-04
AB040888	ODZ3	chr4:184096905-184096964	A_23_P121722	-1.46	-1.10	8.92E-07	1.68E-04
NM_001006636	GTDC1	chr2:144537419-144537360	A_23_P153945	-1.45	-1.29	9.71E-06	8.35E-05
NM_001013398	IGFBP3	chr7:45725907-45725848	A_23_P215634	-1.45	-1.26	6.64E-03	2.64E-02
AK056534	LOC145757	chr15:99221222-99221281	A_32_P145039	-1.45	-1.21	1.39E-06	5.02E-05
NM_024772	ZMYM1	chr1:35249693-35249752	A_24_P53985	-1.45	-1.19	6.03E-09	6.82E-07
NM_057180	VPS29	chr12:109396555-109393716	A_24_P175989	-1.45	-1.14	3.07E-07	4.08E-05
NM_024772	ZMYM1	chr1:35250128-35250187	A_23_P161091	-1.45	-1.10	1.74E-15	2.72E-11
NM_003634	NIPSNAP1	chr22:28281310-28279471	A_24_P237278	-1.45	-1.07	1.02E-04	5.10E-03
NM_001951	E2F5	chr8:86313451-86313510	A_23_P31721	-1.44	-1.65	7.37E-10	1.34E-11
AK001173	AQR	chr15:32935085-32935042	A_24_P316305	-1.44	-1.40	3.06E-08	6.13E-08
AK096154	AK096154	chr5:114567741-114567682	A_32_P61145	-1.44	-1.11	3.84E-07	6.21E-05
NM_017617	NOTCH1	chr9:136665660-136665601	A_23_P60387	-1.44	-0.97	1.11E-04	1.13E-02
NM_001013398	IGFBP3	chr7:45725907-45725848	A_23_P215634	-1.43	-1.26	6.11E-03	2.19E-02
BX648207	BX648207	chr12:42399496-42399437	A_32_P15156	-1.43	-0.99	6.37E-12	1.84E-07
AK098787	PLEKHA2	chr8:38947831-38947890	A_32_P150130	-1.43	-0.87	1.40E-04	2.66E-02
NM_145858	CRYZL1	chr21:33891562-33891503	A_23_P218731	-1.42	-1.35	1.68E-09	6.59E-09
NM_017915	C12orf48	chr12:101093619-101093678	A_23_P87769	-1.42	-1.24	2.91E-07	5.26E-06
THC2386589	THC2386589	chr8:121606727-121606668	A_32_P196233	-1.42	-1.01	1.91E-10	1.31E-06
NM_017617	NOTCH1	chr9:136665660-136665601	A_23_P60387	-1.42	-0.95	1.40E-04	1.45E-02
AL049980	DKFZP564C152	chr11:43837399-43837458	A_23_P336728	-1.42	-0.81	1.45E-07	2.04E-03
NM_015340	LARS2	chr3:45565074-45565133	A_23_P212397	-1.42	-0.66	8.14E-14	4.10E-05
NM_000693	ALDH1A3	chr15:99274026-99274085	A_23_P205959	-1.41	-1.58	2.51E-06	2.54E-07

Gene	Symbol	Chromosomal position	Probe	siCASP8AP2.3	CASP8AP2.6	siCASP8AP2.3	CASP8AP2.6
				M	M	Q	Q
NM_002274	KRT13	chr17:36914835-36913446	A_24_P228149	-1.41	-1.45	1.29E-03	1.31E-03
NM_173827	COX18	chr4:74287218-74287159	A_23_P321485	-1.41	-1.38	5.12E-10	8.50E-10
BC013792	BC013792	chr12:107679582-107679523	A_23_P333802	-1.41	-1.23	3.78E-10	1.65E-08
NM_022344	C17orf75	chr17:27686485-27685943	A_23_P15639	-1.41	-1.14	2.21E-07	1.99E-05
NM_017617	NOTCH1	chr9:136665660-136665601	A_23_P60387	-1.41	-1.09	9.85E-05	2.99E-03
NM_005117	FGF19	chr11:69222940-69222881	A_23_P52714	-1.41	-0.86	1.44E-05	9.18E-03
NM_018660	ZNF395	chr8:28266027-28265203	A_23_P157460	-1.41	-0.77	8.00E-05	4.26E-02
NM_001361	DHODH	chr16:70615789-70615848	A_23_P15202	-1.40	-1.16	6.58E-08	4.45E-06
NM_017617	NOTCH1	chr9:136665660-136665601	A_23_P60387	-1.40	-1.06	1.50E-04	5.16E-03
NM_173830	C6orf182	chr6:109587269-109587328	A_24_P189112	-1.40	-1.02	1.47E-11	9.17E-08
NM_017617	NOTCH1	chr9:136665660-136665601	A_23_P60387	-1.40	-0.96	3.67E-04	1.93E-02
NM_017617	NOTCH1	chr9:136665660-136665601	A_23_P60387	-1.40	-0.90	1.40E-04	1.93E-02
ENST00000333310	ENST00000333310	chr12:120682613-120682672	A_24_P119131	-1.40	-0.84	1.83E-04	3.43E-02
NM_007371	BRD3	chr9:133927878-133927819	A_23_P216689	-1.40	-0.75	2.66E-05	3.04E-02
NM_024641	MANEA	chr6:96161690-96161749	A_24_P69274	-1.39	-1.42	1.35E-04	1.23E-04
BC041772	LOC124976	chr17:4388996-4389055	A_24_P8371	-1.39	-1.17	7.29E-04	5.99E-03
NM_024060	AHNAK	chr11:61957718-61957659	A_23_P21363	-1.39	-1.15	2.17E-03	1.53E-02
BC008299	C14orf139	chr14:94943501-94943442	A_23_P14302	-1.39	-1.13	6.74E-05	1.31E-03
NM_175623	RAB3IP	chr12:68499991-68500050	A_32_P180920	-1.39	-0.97	4.14E-10	2.91E-06
ENST00000361080	ENST00000361080	chr9:105238889-105238948	A_24_P479510	-1.39	-0.97	9.99E-05	7.90E-03
NM_016025	METTL9	chr16:21575892-21575951	A_23_P206347	-1.39	-0.91	3.93E-07	6.59E-04
NM_020236	MRPL1	chr4:79161761-79161819	A_23_P84821	-1.39	-0.88	1.42E-04	2.02E-02
THC2276742	THC2276742	chr3:126427425-126427366	A_32_P131050	-1.39	-0.70	3.20E-07	8.90E-03
NM_002949	MRPL12	chr17:77284406-77284465	A_23_P170352	-1.39	-0.63	1.94E-09	3.60E-03
NM_032273	TMEM126A	chr11:85045062-85045121	A_23_P13524	-1.38	-1.49	1.98E-07	3.06E-08
NM_080664	C14orf126	chr14:30984988-30984978	A_23_P37144	-1.38	-1.41	6.03E-06	4.35E-06
NM_145243	OMA1	chr1:58658847-58658788	A_23_P138137	-1.37	-1.57	2.32E-10	2.64E-12
NM_001013398	IGFBP3	chr7:45725907-45725848	A_23_P215634	-1.37	-1.42	1.12E-02	1.25E-02
NM_152757	FLJ30313	chr20:60553082-60553023	A_23_P432506	-1.37	-1.16	3.39E-03	1.87E-02
ENST00000312275	ENST00000312275	chr3:158743974-158753753	A_32_P516818	-1.37	-1.05	2.52E-07	4.69E-05
NM_001512	GSTA4	chr6:52951156-52951097	A_23_P110941	-1.37	-0.98	7.54E-04	2.21E-02
AK054572	AK054572	chr2:24138562-24138621	A_32_P3317	-1.37	-0.76	6.50E-09	5.83E-04
NM_018371	ChGn	chr8:19306294-19306235	A_23_P134835	-1.37	-0.66	1.41E-08	4.22E-03
NM_015522	DYNC2LI1	chr2:43928506-43933300	A_23_P502174	-1.36	-1.42	3.17E-08	1.00E-08
NM_001013398	IGFBP3	chr7:45725907-45725848	A_23_P215634	-1.36	-1.37	9.85E-03	1.29E-02
THC2408010	THC2408010	chr2:99229402-99229461	A_24_P482189	-1.36	-1.32	1.01E-05	2.01E-05
NM_032023	RASSF4	chr10:44809774-44809833	A_24_P295590	-1.36	-0.97	8.84E-10	3.31E-06
NM_017617	NOTCH1	chr9:136665660-136665601	A_23_P60387	-1.36	-0.91	2.61E-04	1.91E-02
BC009111	LOC552889	chr12:73221102-73221161	A_24_P263956	-1.36	-0.80	4.11E-05	2.07E-02
NM_032637	SKP2	chr5:36206229-36206288	A_23_P156310	-1.35	-1.72	1.86E-04	3.87E-06
NM_005322	HIST1H1B	chr6:27942667-27942608	A_23_P250385	-1.35	-1.61	8.17E-03	2.22E-03
BC066344	SWS1	chr17:15822153-15822094	A_23_P164421	-1.35	-1.54	2.18E-15	1.24E-17
NM_001013398	IGFBP3	chr7:45725907-45725848	A_23_P215634	-1.35	-1.43	1.15E-02	1.04E-02
NM_001013398	IGFBP3	chr7:45725907-45725848	A_23_P215634	-1.35	-1.20	9.46E-03	3.05E-02
NM_006443	C6orf108	chr6:43305091-43305032	A_24_P414733	-1.35	-1.18	3.71E-05	3.09E-04
BX113895	BX113895	chr8:33338121-33338062	A_32_P69076	-1.35	-1.13	1.59E-08	1.11E-06
AL833119	DKFZp313A2432	chr11:22800312-22800253	A_24_P111242	-1.35	-1.10	4.41E-07	2.90E-05
NM_015979	CRSP3	chr6:131936970-131936911	A_23_P330999	-1.35	-1.02	2.03E-12	7.71E-09
NM_016625	RSRC1	chr3:159744700-159744759	A_32_P34589	-1.34	-1.16	7.94E-13	6.63E-11
CR607569	CR607569	chr2:70348408-70348349	A_24_P405430	-1.34	-1.05	1.18E-12	2.40E-09
NM_183050	BCKDHB	chr6:81112559-81112618	A_24_P239664	-1.34	-0.97	1.30E-08	1.44E-05
NM_153234	LIX1	chr5:96458343-96458284	A_24_P226241	-1.33	-1.50	2.13E-03	6.92E-04
AK091439	TMEM48	chr1:53944064-53944005	A_24_P222184	-1.33	-1.34	2.55E-09	2.07E-09
CR591289	CR591289	chr1:40392068-40392009	A_32_P39431	-1.33	-0.96	7.57E-06	1.12E-03
NM_031455	CCDC3	chr10:12978694-12978635	A_24_P369232	-1.33	-0.67	5.59E-09	2.05E-03
THC2405550	THC2405550	chr7:098912800-098912859	A_32_P223327	-1.33	-0.60	5.41E-08	1.23E-02

Gene	Symbol	Chromosomal position	Probe	siCASP8AP2.3	CASP8AP2.6	siCASP8AP2.3	CASP8AP2.6
				M	M	Q	Q
ENST00000379426	ENST00000379426	chr6:11691559-11691618	A_32_P178966	-1.32	-1.50	2.18E-08	6.02E-10
NM_001013398	IGFBP3	chr7:45725907-45725848	A_23_P215634	-1.32	-1.32	1.32E-02	1.89E-02
NM_001013398	IGFBP3	chr7:45725907-45725848	A_23_P215634	-1.32	-1.29	1.01E-02	1.70E-02
NM_018303	EXOC2	chr6:431031-430972	A_23_P214354	-1.32	-1.04	2.49E-09	8.19E-07
A_24_P850187	A_24_P850187	chr2:038370809-038371298	A_24_P850187	-1.32	-0.99	3.48E-03	4.13E-02
AV708150	AV708150	chr6:34363641-34363700	A_32_P70491	-1.32	-0.94	2.84E-05	3.03E-03
AK091439	TMEM48	chr1:53944632-53944573	A_24_P222192	-1.31	-1.38	2.75E-06	1.00E-06
NM_031942	CDCA7	chr2:174058400-174058459	A_24_P171549	-1.31	-1.24	3.55E-10	1.97E-09
CR605444	CR605444	chr6:99423527-99423468	A_32_P67610	-1.31	-1.20	1.21E-06	7.13E-06
NM_182734	PLCB1	chr20:8812945-8813004	A_24_P941643	-1.31	-1.19	2.96E-04	1.25E-03
NM_005829	AP3S2	chr15:88178782-88178723	A_24_P287691	-1.31	-1.07	4.75E-08	4.68E-06
NM_000522	HOXA13	chr7:27009973-27009914	A_23_P389281	-1.31	-1.07	2.26E-07	1.39E-05
BC012758	RNF187	chr1:224989101-224989160	A_24_P23995	-1.31	-1.00	7.90E-04	1.42E-02
NM_025152	NUBPL	chr14:31398683-31398742	A_23_P170908	-1.31	-0.79	3.59E-08	4.91E-04
BQ926066	BQ926066	chr1:170604139-170604197	A_32_P6682	-1.31	-0.66	2.88E-06	2.01E-02
AK055407	C6orf206	chr6:43747466-43747525	A_24_P52189	-1.31	-0.62	6.30E-08	8.59E-03
BX641069	AYTL1	chr16:54177658-54177717	A_24_P364807	-1.30	-1.10	2.39E-04	2.38E-03
NM_000890	KCNJ5	chr11:128292646-128292705	A_23_P202927	-1.30	-1.02	2.92E-04	5.79E-03
BE703537	BE703537	chr5:31589783-31589724	A_32_P104300	-1.30	-0.89	8.77E-08	1.30E-04
NM_006606	RBBP9	chr20:18415660-18415601	A_23_P257538	-1.30	-0.87	2.67E-09	2.45E-05
NM_145213	MRPL30	chr2:99263182-99269734	A_24_P257201	-1.30	-0.76	2.05E-04	4.30E-02
NM_145243	OMA1	chr1:58683812-58683753	A_23_P138139	-1.29	-1.58	3.54E-09	8.01E-12
NM_033419	PERLD1	chr17:35081125-35081066	A_24_P275828	-1.29	-1.54	9.27E-13	1.24E-15
NM_001013398	IGFBP3	chr7:45725907-45725848	A_23_P215634	-1.29	-1.21	1.23E-02	2.68E-02
AK021664	AK021664	chr15:46214619-46214560	A_32_P31744	-1.29	-1.02	3.16E-10	1.51E-07
A_32_P36835	A_32_P36835	chr3:047507537-047507478	A_32_P36835	-1.29	-0.95	1.10E-08	1.11E-05
AL133028	KIAA1211	chr4:57036427-57036486	A_24_P331882	-1.29	-0.93	2.05E-06	5.00E-04
NM_006049	SNAPC5	chr15:64574743-64573907	A_23_P383977	-1.29	-0.89	4.63E-08	8.11E-05
NM_016025	METTL9	chr16:21575516-21575575	A_24_P202139	-1.29	-0.89	1.65E-07	2.14E-04
XM_932180	LOC644424	chr13:018332293-018332352	A_32_P12065	-1.29	-0.80	2.51E-08	2.80E-04
BC031266	RNF36	chr15:42811637-42811696	A_24_P50543	-1.29	-0.76	3.04E-07	2.11E-03
A_24_P127121	A_24_P127121	chrX:106181577-106181638	A_24_P127121	-1.29	-0.75	4.53E-05	2.25E-02
A_24_P400970	A_24_P400970	chr7:063338886-063338949	A_24_P400970	-1.29	-0.70	4.12E-05	3.47E-02
BC032648	SOLH	chr16:529804-529863	A_24_P928639	-1.29	-0.60	2.55E-06	3.37E-02
NM_014059	RGC32	chr13:40940937-40942635	A_24_P10137	-1.28	-1.39	3.75E-03	2.31E-03
NM_001007255	KARCA1	chr1:157883109-157883168	A_23_P86100	-1.28	-1.25	9.10E-10	1.92E-09
NM_020748	INTS2	chr17:57298052-57297993	A_23_P420269	-1.28	-0.98	5.44E-06	4.51E-04
NM_017810	ZNF434	chr16:3372834-3372775	A_23_P218282	-1.28	-0.83	3.75E-09	4.36E-05
THC2279352	THC2279352	chr15:55368664-55368723	A_24_P13663	-1.28	-0.79	2.33E-10	2.25E-05
NM_052845	MMAB	chr12:108456407-108456348	A_24_P56221	-1.27	-1.15	2.99E-07	2.51E-06
NM_019021	C11orf71	chr11:113775602-113775543	A_23_P13150	-1.27	-1.13	1.24E-06	1.53E-05
NM_031942	CDCA7	chr2:174058997-174059056	A_23_P251421	-1.27	-1.04	1.53E-08	1.97E-06
NM_032826	SLC35B4	chr7:133433105-133433046	A_24_P367561	-1.27	-0.72	1.52E-10	6.26E-05
AI267321	AI267321	chr12:19562932-19562873	A_32_P209104	-1.27	-0.71	5.42E-08	1.61E-03
NM_005687	FARSLB	chr2:223262047-223261988	A_23_P165346	-1.26	-1.27	4.82E-08	4.26E-08
BC022538	FBXO36	chr2:230703070-230703129	A_23_P422981	-1.26	-1.13	1.54E-11	4.72E-10
NM_001346	DGKG	chr3:187349860-187349801	A_23_P40926	-1.26	-1.06	2.48E-06	6.18E-05
NM_145011	ZNF25	chr10:38279074-38279015	A_23_P381577	-1.26	-0.74	1.08E-08	3.98E-04
NM_005247	FGF3	chr11:69334189-69334130	A_23_P113204	-1.25	-1.51	9.91E-07	1.21E-08
NM_002643	PIGF	chr2:46731348-46731289	A_23_P131653	-1.25	-1.36	3.48E-10	3.01E-11
NM_152417	TMEM68	chr8:56814923-56814864	A_23_P136172	-1.25	-1.03	1.89E-13	1.02E-10
THC2433670	THC2433670	chr8:1698467-1698408	A_32_P186725	-1.25	-1.03	9.57E-05	1.44E-03
NM_020234	DTWD1	chr15:47704841-47704900	A_24_P70117	-1.25	-1.01	8.83E-10	2.23E-07
CR624880	CR624880	chr12:65999712-65999771	A_32_P150418	-1.25	-0.89	5.58E-11	5.12E-07
CR591264	CR591264	chr17:30360430-30360489	A_32_P204624	-1.25	-0.87	5.41E-08	8.65E-05
NM_005117	FGF19	chr11:69223151-69223092	A_23_P427587	-1.25	-0.80	1.14E-04	1.73E-02

Gene	Symbol	Chromosomal position	Probe	siCASP8AP2.3	CASP8AP2.6	siCASP8AP2.3	CASP8AP2.6
				M	M	Q	Q
NM_032013	NDRG3	chr20:34714313-34714254	A_24_P185604	-1.25	-0.74	2.01E-04	3.84E-02
NM_145212	MRPL30	chr2:99270731-99270790	A_23_P218619	-1.25	-0.73	1.06E-04	3.08E-02
NM_052934	SLC26A9	chr1:202614081-202614022	A_23_P201248	-1.25	-0.72	1.53E-06	5.31E-03
THC2304800	THC2304800	chr17:77529398-77529351	A_24_P928947	-1.24	-1.11	1.85E-03	7.29E-03
NM_003317	TITF1	chr14:36056741-36056682	A_24_P184692	-1.24	-1.07	1.51E-04	1.27E-03
NM_183050	BCKDHB	chr6:81111949-81112008	A_24_P914513	-1.24	-0.90	9.07E-11	4.70E-07
THC2311626	THC2311626	chr12:37009215-37009274	A_32_P89679	-1.24	-0.89	2.14E-08	2.55E-05
NM_004956	ETV1	chr7:13704336-13704277	A_32_P78491	-1.24	-0.79	1.22E-08	1.28E-04
NM_016353	ZDHHC2	chr8:17123203-17123262	A_23_P9086	-1.24	-0.60	5.56E-15	5.99E-06
NM_003130	SRI	chr7:87483312-87482480	A_23_P358928	-1.23	-1.26	6.58E-11	3.03E-11
NM_057180	VPS29	chr12:109392583-109392524	A_23_P128384	-1.23	-1.23	2.08E-08	1.91E-08
NM_001013398	IGFBP3	chr7:45725907-45725848	A_23_P215634	-1.23	-1.23	2.11E-02	2.98E-02
BC067300	BC067300	chr2:234167112-234167053	A_23_P343594	-1.23	-1.08	9.24E-09	2.28E-07
NM_177444	PPFIBP1	chr12:27622403-27677584	A_23_P337917	-1.23	-1.04	1.18E-02	4.83E-02
THC2279790	THC2279790	chr10:6820845-6820904	A_32_P15466	-1.23	-0.93	2.65E-03	3.32E-02
NM_032483	PPAPDC1B	chr8:38241553-38241494	A_23_P329971	-1.23	-0.89	6.81E-06	1.14E-03
AK023647	AK023647	chr21:41575616-41575675	A_24_P889070	-1.23	-0.88	1.17E-07	9.07E-05
NM_017882	CLN6	chr15:66287062-66287003	A_23_P117797	-1.23	-0.88	5.59E-07	2.50E-04
NM_152834	TMEM18	chr2:658758-658699	A_24_P385585	-1.23	-0.86	1.27E-11	2.31E-07
NM_206808	CLYBL	chr13:99315165-99316552	A_23_P376591	-1.22	-1.33	1.26E-06	2.10E-07
THC2303869	THC2303869	chr2:44025502-44025443	A_24_P674924	-1.22	-1.18	5.10E-04	1.03E-03
NM_004365	CETN3	chr5:89730993-89730934	A_23_P7732	-1.22	-1.01	2.63E-06	8.77E-05
NM_005434	MALL	chr2:110200447-110200388	A_23_P102551	-1.22	-1.01	3.12E-03	1.99E-02
NM_031482	ATG10	chr5:81496083-81510094	A_23_P92824	-1.22	-1.00	3.46E-09	5.09E-07
NM_014039	C11orf54	chr11:93135711-93135770	A_24_P201404	-1.22	-0.98	1.31E-13	1.81E-10
NM_032276	RHBDD1	chr2:227686552-227686611	A_24_P134834	-1.22	-0.86	1.08E-07	1.07E-04
NM_080737	SYTL4	chrX:99736002-99735943	A_24_P122337	-1.22	-0.83	1.61E-05	3.84E-03
AK055101	AK055101	chr6:99827748-99827689	A_32_P74964	-1.22	-0.81	3.88E-05	7.21E-03
NM_182760	SUMF1	chr3:4378152-4378094	A_23_P69242	-1.22	-0.71	1.70E-12	3.92E-06
NM_018984	SSH1	chr12:107683782-107683723	A_23_P429560	-1.22	-0.67	7.75E-12	2.67E-05
AJ420450	AJ420450	chr9:100148542-100148601	A_32_P144326	-1.21	-1.21	6.14E-09	7.41E-09
BC032946	AQP5	chr12:48645622-48645681	A_23_P117104	-1.21	-1.05	1.54E-03	8.51E-03
NM_002311	LIG3	chr17:30352504-30353068	A_23_P501134	-1.21	-0.90	3.67E-06	4.94E-04
NM_002957	RXRA	chr9:134554548-134554607	A_23_P219176	-1.21	-0.74	1.03E-07	7.41E-04
ENST00000354271	ENST00000354271	chr17:4872193-4872252	A_32_P149288	-1.21	-0.69	3.62E-11	2.90E-05
NM_130797	DPP6	chr7:154122615-154122674	A_23_P170888	-1.20	-1.83	1.10E-02	1.60E-04
NM_005482	PIGK	chr1:77269544-77269485	A_23_P34307	-1.20	-1.12	1.80E-10	1.17E-09
BC030122	BC030122	chr5:87767932-87767991	A_32_P215556	-1.20	-1.02	3.47E-05	4.31E-04
NM_005240	ETV3	chr1:153916396-153916337	A_23_P400945	-1.20	-0.89	6.00E-07	1.64E-04
NM_144988	ALG14	chr1:95160667-95160608	A_23_P257423	-1.20	-0.85	2.59E-06	7.10E-04
THC2377297	THC2377297	chr6:142587113-142587172	A_32_P190316	-1.20	-0.80	1.63E-11	8.15E-07
NM_017676	FLJ20125	chr5:102451565-102451506	A_23_P110611	-1.19	-1.33	1.79E-07	1.31E-08
NM_145729	MRPL24	chr1:153520586-153520527	A_23_P137848	-1.19	-1.23	3.43E-13	1.07E-13
AF165514	HSD17B7P2	chr10:38694524-38694863	A_32_P53183	-1.19	-0.98	5.96E-05	1.04E-03
AI024778	AI024778	chr2:178233321-178233262	A_32_P216507	-1.19	-0.98	2.70E-04	3.28E-03
THC2437580	THC2437580	chr8:109568346-109568405	A_23_P59999	-1.19	-0.85	5.14E-07	2.49E-04
AK095738	AK095738	chr17:36034898-36034839	A_32_P20221	-1.19	-0.80	1.73E-08	6.09E-05
NM_012236	SCMH1	chr1:41162685-41162626	A_23_P12477	-1.19	-0.79	5.62E-05	8.98E-03
NM_013341	PTD004	chr2:174763042-174762983	A_24_P114438	-1.19	-0.62	2.99E-09	9.10E-04
BC015836	BC015836	chr3:156491482-156491330	A_32_P167017	-1.18	-1.39	1.16E-03	1.83E-04
NM_005482	PIGK	chr1:77266997-77266938	A_24_P379512	-1.18	-1.35	8.58E-09	1.73E-10
AK057056	C20orf117	chr20:34839793-34839734	A_32_P32179	-1.18	-1.03	1.38E-04	1.07E-03
NM_004928	C21orf2	chr21:44573807-44573748	A_23_P211167	-1.18	-0.84	2.35E-06	7.38E-04
AK023791	SEC15L2	chr2:72315478-72315419	A_32_P16258	-1.18	-0.62	1.22E-06	1.07E-02
NM_017845	COMMD8	chr4:47293953-47293894	A_23_P44257	-1.17	-1.44	8.72E-07	7.13E-09
ENST00000305820	ENST00000305820	chr6:7461811-7461752	A_24_P246591	-1.17	-1.38	2.89E-04	2.86E-05

Gene	Symbol	Chromosomal position	Probe	siCASP8AP2.3	CASP8AP2.6	siCASP8AP2.3	CASP8AP2.6
				M	M	Q	Q
ENST00000314720	ENST00000314720	chrX:13442451-13442510	A_23_P320159	-1.17	-1.16	3.16E-11	4.27E-11
AK098124	TBC1D8	chr2:101098841-101098900	A_24_P576445	-1.17	-1.13	1.21E-03	2.24E-03
AK000383	DUSP22	chr6:295182-295241	A_23_P167905	-1.17	-0.88	6.22E-09	4.45E-06
NM_199005	ZNF322B	chr6:26745035-26744976	A_32_P163533	-1.17	-0.87	3.06E-03	3.99E-02
NM_006055	LANCL1	chr2:211122250-211122191	A_24_P259922	-1.17	-0.74	1.72E-05	6.97E-03
NM_080670	SLC35A4	chr5:139928407-139928466	A_23_P69840	-1.17	-0.65	1.09E-08	7.82E-04
XM_926634	LOC653256	chr3_random:14298-14357	A_32_P221452	-1.16	-1.09	2.47E-11	1.61E-10
NM_014962	BTBD3	chr20:11852376-11852435	A_23_P102759	-1.16	-0.99	1.04E-06	2.18E-05
NM_007047	BTN3A2	chr6:26486236-26486295	A_24_P252078	-1.16	-0.98	1.09E-02	4.63E-02
ENST00000368192	ENST00000368192	chr1:153904482-153904423	A_24_P849245	-1.16	-0.97	1.90E-04	2.30E-03
NM_033284	TBL1Y	chrY:6998754-6998813	A_23_P114466	-1.16	-0.93	7.66E-04	9.60E-03
NM_032138	KBTBD7	chr13:40664213-40664154	A_23_P25605	-1.16	-0.91	3.57E-08	7.24E-06
NM_016063	HDDC2	chr6:125638484-125638425	A_23_P122796	-1.16	-0.89	5.33E-09	2.84E-06
NM_014035	SNX24	chr5:122371487-122371546	A_23_P19095	-1.16	-0.88	1.52E-06	2.12E-04
AK021744	AK021744	chr12:103913803-103913862	A_32_P167459	-1.16	-0.88	1.09E-05	8.02E-04
AV742170	AV742170	chr4:140668153-140668094	A_32_P40667	-1.16	-0.69	2.83E-07	1.61E-03
NM_007220	CA5B	chrX:15564243-15564302	A_24_P941268	-1.16	-0.68	2.00E-07	1.69E-03
NM_007107	SSR3	chr3:157741829-157741770	A_24_P319942	-1.16	-0.67	4.59E-05	2.42E-02
NM_018660	ZNF395	chr8:28259369-28259310	A_23_P146077	-1.16	-0.66	5.23E-07	3.87E-03
NM_203438	C10orf4	chr10:95420523-95419563	A_24_P189464	-1.15	-1.49	6.27E-06	1.89E-08
NM_015533	DAK	chr11:60869597-60869913	A_23_P36129	-1.15	-1.25	1.05E-05	2.24E-06
NM_017842	FLJ20489	chr12:46461578-46461637	A_23_P204511	-1.15	-1.14	3.27E-03	4.90E-03
NM_015385	SORBS1	chr10:97062364-97062305	A_24_P326511	-1.15	-1.08	3.96E-04	1.08E-03
ENST00000368847	ENST00000368847	chr6:111698929-111698988	A_32_P197870	-1.15	-1.02	3.84E-05	2.52E-04
NM_024641	MANEA	chr6:96141312-96141371	A_24_P187921	-1.15	-1.02	2.58E-04	1.60E-03
NM_032026	TATDN1	chr8:125590095-125590036	A_23_P254978	-1.15	-0.99	1.94E-07	4.73E-06
THC2414638	THC2414638	chr3:13430459-13430400	A_24_P479793	-1.15	-0.97	7.32E-04	6.17E-03
ENST00000373644	ENST00000373644	chr10:70123522-70123581	A_23_P322756	-1.15	-0.91	1.22E-04	2.82E-03
ENST00000354937	ENST00000354937	chr3:197191068-197191009	A_24_P315873	-1.15	-0.88	2.70E-04	6.42E-03
THC2313538	THC2313538	chr2:038923497-038923556	A_32_P135601	-1.15	-0.87	1.34E-04	4.45E-03
NM_000532	PCCB	chr3:137518505-137518564	A_24_P302802	-1.15	-0.84	2.88E-05	2.40E-03
AJ272267	CHDH	chr3:53826549-53826490	A_23_P69293	-1.15	-0.77	7.83E-07	7.47E-04
AL831999	AL831999	chr13:52172550-52172609	A_32_P161455	-1.15	-0.76	5.51E-06	2.81E-03
NM_001007094	ZNF37A	chr10:38443805-38444177	A_23_P23941	-1.15	-0.63	1.72E-11	3.46E-05
AK097893	LOC257396	chr5:52446527-52446586	A_32_P92563	-1.14	-1.73	1.53E-04	5.49E-08
NM_020211	RGMA	chr15:91387839-91387780	A_23_P372308	-1.14	-1.32	2.16E-02	1.04E-02
NM_014962	BTBD3	chr20:11854389-11854448	A_24_P134356	-1.14	-1.30	2.79E-09	5.03E-11
NM_145814	CACNG6	chr19:59207607-59207666	A_23_P501933	-1.14	-1.25	2.13E-02	1.57E-02
THC2435127	THC2435127	chr2:178233897-178233956	A_32_P27706	-1.14	-1.18	1.20E-07	6.24E-08
AJ243950	SERGEF	chr11:17937626-17856342	A_23_P139207	-1.14	-1.10	2.91E-04	5.58E-04
NM_052845	MMAB	chr12:108456524-108456465	A_23_P2537	-1.14	-1.08	1.33E-06	3.99E-06
NM_022717	U1SNRNPBP	chr12:122475425-122475484	A_23_P159101	-1.14	-1.07	7.98E-10	4.71E-09
NM_138477	CDAN1	chr15:40803592-40803533	A_23_P349127	-1.14	-1.06	6.80E-06	2.83E-05
NM_000671	ADH5	chr4:100349988-100349929	A_24_P260346	-1.14	-0.88	1.03E-03	1.56E-02
AK056172	LOC554203	chrX:72947451-72947510	A_32_P223935	-1.14	-0.80	1.06E-04	8.13E-03
NM_003631	PARG	chr10:50697154-50697095	A_23_P97810	-1.14	-0.73	4.13E-05	1.07E-02
NM_001012410	SGOL1	chr3:20177369-20177310	A_23_P29723	-1.14	-0.70	1.68E-09	6.41E-05
ENST00000329536	ENST00000329536	chr3:75873462-75873162	A_23_P6980	-1.14	-0.69	1.33E-09	8.05E-05
NM_004866	SCAMP1	chr5:77810559-77810618	A_24_P149023	-1.14	-0.67	1.07E-09	1.16E-04
BE091362	BE091362	chr5:61722478-61722537	A_32_P132276	-1.14	-0.60	3.58E-09	1.04E-03
BC013295	BC013295	chr2:74972458-74972399	A_24_P170874	-1.13	-1.18	8.11E-09	2.81E-09
NM_000890	KCNJ5	chr11:128292324-128292383	A_24_P309521	-1.13	-1.02	2.05E-04	9.65E-04
AK024346	LOC153346	chr5:149214015-149214074	A_32_P211188	-1.13	-0.99	1.08E-04	8.57E-04
NM_023934	FUNDC2	chrX:153825401-153825460	A_23_P171314	-1.13	-0.92	5.77E-10	1.63E-07
NM_006726	LRBA	chr4:151543855-151543796	A_24_P360078	-1.13	-0.91	6.83E-07	4.56E-05
NM_138408	C6orf51	chr6:111395596-111395655	A_23_P400465	-1.13	-0.87	8.38E-07	1.16E-04

Gene	Symbol	Chromosomal position	Probe	siCASP8AP2.3	CASP8AP2.6	siCASP8AP2.3	CASP8AP2.6
				M	M	Q	Q
NM_003685	KHSRP	chr19:6367594-6367535	A_24_P134235	-1.13	-0.76	8.73E-04	3.50E-02
NM_023074	ZNF649	chr19:57084420-57084361	A_23_P208208	-1.13	-0.74	2.70E-09	3.15E-05
BC041926	BC041926	chr7:138704225-138704166	A_24_P681011	-1.13	-0.74	3.20E-05	7.40E-03
NM_001008397	LOC493869	chr5:54496090-54496149	A_23_P404259	-1.12	-1.33	2.90E-05	1.37E-06
NM_002012	FHIT	chr3:59712919-59712860	A_23_P125164	-1.12	-1.33	1.11E-02	3.29E-03
NM_005053	RAD23A	chr19:12920341-12920533	A_24_P22887	-1.12	-1.26	2.91E-03	1.05E-03
G36631	G36631	chr16:19619624-19619565	A_32_P178537	-1.12	-1.06	4.33E-03	9.47E-03
THC2303284	THC2303284	chr8:130921265-130921206	A_24_P12660	-1.12	-0.95	4.48E-06	1.00E-04
BX537532	LOC550643	chrX:56726994-56727053	A_24_P867868	-1.12	-0.95	2.62E-05	3.68E-04
NM_022766	CERK	chr22:45401846-45401787	A_24_P62237	-1.12	-0.91	6.19E-04	6.90E-03
BC036246	FLJ32549	chr12:62867130-62867071	A_24_P68294	-1.12	-0.89	3.16E-09	9.87E-07
THC2372489	THC2372489	chr14:59041310-59041369	A_23_P205584	-1.12	-0.79	4.28E-07	2.90E-04
NM_052909	KIAA1909	chr5:237724-237783	A_32_P129269	-1.12	-0.79	1.97E-04	1.18E-02
NM_032376	TMEM101	chr17:39444478-39444419	A_23_P15516	-1.12	-0.73	1.28E-08	9.03E-05
NM_003486	SLC7A5	chr16:86425579-86424132	A_23_P3792	-1.12	-0.70	4.37E-04	3.79E-02
NM_001380	DOCK1	chr10:129140451-129140510	A_23_P45059	-1.11	-1.22	7.59E-05	1.78E-05
NM_005897	IPP	chr1:45833867-45833808	A_23_P9662	-1.11	-1.16	7.87E-06	3.76E-06
NM_018356	C5orf22	chr5:31589914-31589973	A_23_P7761	-1.11	-1.08	2.55E-08	5.17E-08
NM_002861	PCYT2	chr17_random:1962309-1962127	A_24_P404245	-1.11	-0.96	3.10E-05	3.43E-04
BC042557	LOC120376	chr11:110684475-110684534	A_23_P116173	-1.11	-0.94	1.01E-02	4.10E-02
NM_182758	WDR72	chr15:51595237-51595178	A_24_P263330	-1.11	-0.91	1.57E-03	1.37E-02
AF085871	AF085871	chr3:171634300-171634241	A_32_P23272	-1.11	-0.84	6.55E-09	4.31E-06
NM_020960	GPR107	chr9:129981710-129981769	A_24_P295379	-1.11	-0.78	1.68E-06	6.22E-04
NM_005915	MCM6	chr2:136432008-136431949	A_23_P90612	-1.11	-0.78	2.79E-04	1.38E-02
NM_002942	ROBO2	chr3:77778406-77778465	A_24_P180151	-1.11	-0.68	2.05E-04	3.05E-02
THC2378571	THC2378571	chr9:103934507-103934448	A_32_P2766	-1.11	-0.66	6.30E-06	7.99E-03
A_32_P109495	A_32_P109495	chr5:056251958-056252017	A_32_P109495	-1.11	-0.63	2.42E-07	2.56E-03
NM_018122	DARS2	chr1:170558782-170558841	A_23_P148984	-1.10	-1.17	2.55E-04	1.33E-04
NM_014391	ANKRD1	chr10:92662601-92662542	A_23_P161218	-1.10	-1.17	2.87E-02	2.71E-02
AB033060	AHRR	chr5:491030-491089	A_23_P358709	-1.10	-1.14	4.19E-02	4.73E-02
NM_014039	C11orf54	chr11:93134442-93134501	A_23_P202750	-1.10	-1.09	1.05E-05	1.45E-05
AK057088	AK057088	chr6:34360429-34360370	A_24_P554156	-1.10	-1.04	8.28E-09	3.57E-08
AK093982	AK093982	chr1:194205794-194205735	A_32_P204330	-1.10	-1.03	5.62E-06	2.29E-05
NM_032088	PCDHGA8	chr5:140872578-140872637	A_23_P354734	-1.10	-0.98	6.59E-05	4.24E-04
A_24_P900721	A_24_P900721	chr7:135856547-135856741	A_24_P900721	-1.10	-0.98	1.06E-02	3.16E-02
NM_003825	SNAP23	chr15:40611186-40611245	A_23_P206177	-1.10	-0.96	4.71E-03	1.83E-02
NM_173536	GABRG1	chr4:45879113-45879054	A_32_P89899	-1.10	-0.94	1.94E-03	1.13E-02
NM_145309	LRRC51	chr11:71485383-71485442	A_24_P184388	-1.10	-0.88	1.04E-06	7.48E-05
AV645774	AV645774	chr7:129399254-129399313	A_32_P112034	-1.10	-0.82	7.70E-11	2.07E-07
NM_176853	THEM4	chr1:148659606-148659547	A_24_P928510	-1.10	-0.82	6.31E-05	3.32E-03
NM_182503	DEADC1	chr6:143790029-143789970	A_24_P186204	-1.10	-0.80	1.65E-04	7.80E-03
NM_006116	MAP3K7IP1	chr22:38151822-38151881	A_23_P80342	-1.10	-0.78	5.73E-06	1.21E-03
ENST00000326140	ENST00000326140	chr4:25607086-25607145	A_24_P341222	-1.10	-0.77	9.73E-10	4.63E-06
NM_024325	ZNF343	chr20:2410907-2410848	A_23_P218706	-1.10	-0.75	6.69E-09	2.72E-05
A_32_P148407	A_32_P148407	chr2:135045744-135045798	A_32_P148407	-1.10	-0.65	5.57E-06	7.81E-03
NM_024622	FASTKD1	chr2:170219304-170219245	A_23_P28590	-1.10	-0.65	1.83E-04	3.64E-02
NM_153824	PYCR1	chr17:77484533-77484474	A_24_P204358	-1.09	-1.08	4.73E-03	6.95E-03
NM_013407	DHPS	chr19:12651482-12651344	A_23_P501887	-1.09	-0.91	6.23E-05	9.66E-04
NM_145261	DNAJC19	chr3:182184769-182184710	A_23_P121396	-1.09	-0.81	5.08E-06	6.56E-04
CR602075	CR602075	chr2:3073271-3073215	A_32_P5800	-1.09	-0.73	2.38E-06	1.26E-03
NM_012140	SLC25A10	chr17:77297930-77297989	A_23_P146830	-1.09	-0.71	1.37E-04	1.69E-02
CR749275	CTNND1	chr11:57341609-57341669	A_24_P881527	-1.09	-0.63	2.31E-05	1.77E-02
NM_024325	ZNF343	chr20:2422457-2422198	A_23_P357248	-1.08	-1.11	9.62E-08	4.96E-08
NM_182503	DEADC1	chr6:143791267-143791208	A_23_P134014	-1.08	-1.10	6.35E-06	4.65E-06
NM_022114	PRDM16	chr1:3378219-3378278	A_32_P225816	-1.08	-1.01	1.37E-03	3.72E-03
NM_181713	UBXD4	chr2:24134328-24134387	A_23_P131240	-1.08	-0.93	1.10E-03	6.56E-03

Gene	Symbol	Chromosomal position	Probe	siCASP8AP2.3	CASP8AP2.6	siCASP8AP2.3	CASP8AP2.6
				M	M	Q	Q
NM_032211	LOXL4	chr10:99997795-99997736	A_24_P406754	-1.08	-0.85	3.29E-03	2.93E-02
AK001846	AK001846	chr1:23377236-23377177	A_24_P724984	-1.08	-0.83	5.71E-08	1.67E-05
NM_024092	TMEM109	chr11:60443882-60443941	A_23_P203364	-1.08	-0.82	4.26E-05	2.06E-03
NM_018083	ZNF358	chr19:7491214-7491273	A_24_P19175	-1.08	-0.76	2.90E-04	1.36E-02
CR611166	CR611166	chr17:53433448-53433389	A_24_P921933	-1.08	-0.72	6.47E-07	7.23E-04
NM_024066	PRNPIP	chr1:44443132-44419671	A_32_P155416	-1.08	-0.71	3.73E-05	8.03E-03
NM_173562	KCTD20	chr6:36565595-36565654	A_23_P374351	-1.08	-0.65	6.15E-09	1.84E-04
NM_001024594	C1orf53	chr1:194607999-194608058	A_32_P210572	-1.07	-1.17	4.31E-04	1.57E-04
NM_001003803	ATP5S	chr14:49860559-49862110	A_24_P118231	-1.07	-1.05	3.17E-07	5.74E-07
NM_017890	VPS13B	chr8:100958888-100958947	A_24_P98251	-1.07	-1.01	2.37E-07	1.10E-06
NM_032437	KIAA1799	chr1:63748798-63750170	A_32_P137266	-1.07	-0.99	1.76E-06	1.03E-05
NM_138730	HMGN3	chr6:79970060-79968811	A_24_P132099	-1.07	-0.97	4.14E-07	3.48E-06
NM_015469	NIPSNAP3A	chr9:104601329-104601388	A_23_P20606	-1.07	-0.97	5.59E-07	4.38E-06
NM_002857	PEX19	chr1:157061503-157061444	A_23_P160188	-1.07	-0.94	1.86E-06	2.36E-05
NM_015464	SOSTDC1	chr7:16275292-16275233	A_23_P145841	-1.07	-0.93	1.06E-02	3.80E-02
A_32_P73580	A_32_P73580	chr7:004822023-004822082	A_32_P73580	-1.07	-0.92	1.05E-05	1.53E-04
THC2314901	THC2314901	chr2:99274290-99274349	A_32_P185029	-1.07	-0.89	3.62E-05	6.26E-04
NM_020381	PDSS2	chr6:107580846-107580787	A_23_P134167	-1.07	-0.79	8.94E-04	1.91E-02
NM_199227	MAP1D	chr2:172771026-172771085	A_23_P90790	-1.07	-0.73	9.46E-08	1.78E-04
AK124299	AK124299	chr14:59835063-59835122	A_24_P688133	-1.07	-0.64	1.07E-08	3.05E-04
NM_022090	LOC63920	chr5:159753488-159753429	A_23_P259103	-1.06	-1.37	1.10E-06	2.27E-09
AK000276	LOC283859	chr16:49227801-49227860	A_24_P304881	-1.06	-1.08	1.51E-04	1.54E-04
NM_003920	TIMELESS	chr12:55097271-55097212	A_23_P53276	-1.06	-1.06	7.24E-09	7.36E-09
NM_000532	PCCB	chr3:137528364-137528423	A_24_P302797	-1.06	-1.06	6.15E-06	6.77E-06
NM_025114	CEP290	chr12:86945362-86945303	A_23_P36865	-1.06	-1.02	1.47E-05	3.45E-05
NM_032432	ABLIM2	chr4:8085206-8085147	A_23_P255672	-1.06	-1.01	1.03E-03	2.21E-03
NM_032437	KIAA1799	chr1:63739477-63739536	A_24_P118608	-1.06	-0.99	5.66E-07	2.60E-06
AK026192	AK026192	chr4:111325089-111325030	A_32_P80597	-1.06	-0.96	1.18E-04	5.44E-04
THC2338051	THC2338051	chr4:104304836-104304777	A_23_P81094	-1.06	-0.96	3.57E-04	1.67E-03
NM_001010862	SPIN3	chrX:56886156-56886097	A_24_P136725	-1.06	-0.92	4.34E-06	5.65E-05
BC032027	BC032027	chr5:168371797-168371738	A_24_P93931	-1.06	-0.92	8.53E-03	3.22E-02
A_24_P332595	A_24_P332595	chr15:087304397-087304456	A_24_P332595	-1.06	-0.88	3.15E-06	1.02E-04
ENST00000371327	ENST00000371327	chr10:96362812-96362871	A_32_P200934	-1.06	-0.79	2.59E-05	1.91E-03
THC2426708	THC2426708	chr22:44794274-44794333	A_24_P934679	-1.06	-0.77	3.53E-07	1.66E-04
NM_022081	HPS4	chr22:25173204-25173145	A_23_P109446	-1.06	-0.76	3.85E-10	1.93E-06
BC060766	SLC2A14	chr12:7856529-7856470	A_32_P47754	-1.06	-0.76	2.12E-03	3.80E-02
NM_022369	STRA6	chr15:72259178-72259119	A_23_P65779	-1.06	-0.73	1.51E-03	4.04E-02
NM_176794	MRPL43	chr10:102731191-102731132	A_23_P382154	-1.06	-0.72	1.24E-05	3.39E-03
THC2373712	THC2373712	chr12:58469273-58469332	A_32_P105397	-1.06	-0.69	9.07E-06	4.10E-03
NM_015678	NBEA	chr13:35144683-35144742	A_23_P65278	-1.06	-0.66	2.46E-04	2.89E-02
NM_001532	SLC29A2	chr11:65888359-65888300	A_23_P104705	-1.06	-0.60	6.70E-05	3.07E-02
NM_152429	C10orf13	chr10:93656943-93656884	A_24_P201381	-1.05	-1.50	3.57E-03	5.54E-05
AK096022	LOC286025	chr7:137974817-137974758	A_32_P103508	-1.05	-1.20	1.13E-05	8.34E-07
AK057604	CRYZL1	chr21:33889091-33889032	A_24_P408341	-1.05	-1.10	2.99E-07	1.23E-07
NM_152739	HOXA9	chr7:26975887-26975828	A_23_P500998	-1.05	-1.07	3.20E-05	2.74E-05
A_24_P213144	A_24_P213144	chr7:027275653-027275716	A_24_P213144	-1.05	-0.97	4.21E-04	1.37E-03
AB040937	EXOD1	chr16:20715896-20715837	A_24_P934800	-1.05	-0.85	3.05E-14	3.21E-11
A_24_P792748	A_24_P792748	chrX:057894805-057894864	A_24_P792748	-1.05	-0.77	1.14E-03	2.30E-02
NM_004059	CCBL1	chr9:128675355-128675296	A_23_P315206	-1.05	-0.75	1.75E-08	2.71E-05
NM_153000	APCDD1	chr18:10478470-10478529	A_23_P337262	-1.05	-0.75	1.63E-04	9.48E-03
NM_005030	PLK1	chr16:23608471-23608713	A_23_P118174	-1.05	-0.74	3.02E-07	2.07E-04
BC029785	FLJ42875	chr1:3007071-3003978	A_24_P366787	-1.05	-0.74	7.09E-06	1.65E-03
THC2266474	THC2266474	chr7:137973618-137973559	A_32_P226186	-1.05	-0.70	2.54E-06	1.44E-03
NM_033028	BBS4	chr15:70817256-70817315	A_23_P99967	-1.04	-1.49	2.85E-05	1.58E-08
BX419129	BX419129	chr11:125000372-125000313	A_32_P40999	-1.04	-1.41	1.07E-05	1.21E-08
CR738137	CR738137	chr9:044893195-044893253	A_32_P197720	-1.04	-1.34	7.73E-03	8.47E-04

Gene	Symbol	Chromosomal position	Probe	siCASP8AP2.3	CASP8AP2.6	siCASP8AP2.3	CASP8AP2.6
				M	M	Q	Q
NM_016422	RNF141	chr11:10490149-10490090	A_24_P372625	-1.04	-1.30	1.40E-03	9.95E-05
XM_930946	LOC653188	chr8_random:929370-929429	A_32_P146898	-1.04	-1.03	5.10E-03	7.94E-03
NM_022786	ARV1	chr1:227430912-227432641	A_24_P144527	-1.04	-1.00	1.45E-02	2.62E-02
NM_007244	PRR4	chr12:10891047-10890988	A_23_P76291	-1.04	-0.93	3.31E-04	1.53E-03
NM_017617	NOTCH1	chr9:136665660-136665601	A_23_P60387	-1.04	-0.93	6.55E-03	2.22E-02
BG741106	BG741106	chr17:78370793-78370852	A_24_P3704	-1.04	-0.90	1.73E-05	1.92E-04
CR592318	CR592318	chr10:81441767-81441708	A_32_P147297	-1.04	-0.83	6.55E-03	4.54E-02
NM_018691	C5orf3	chr5:153352027-153351968	A_23_P41908	-1.04	-0.80	1.19E-07	2.82E-05
NM_194247	HNRPA3	chr2:177911213-177911272	A_23_P86660	-1.04	-0.75	2.56E-07	1.35E-04
NM_178014	TUBB	chr6:30800501-30800560	A_23_P387057	-1.04	-0.74	1.55E-04	8.55E-03
DB040019	DB040019	chr1:9577732-9577791	A_32_P222277	-1.04	-0.70	8.17E-04	3.37E-02
NM_017661	SUHW4	chr15:54710369-54710310	A_23_P14708	-1.04	-0.62	2.83E-07	1.65E-03
NM_016079	VPS24	chr2:86642245-86642209	A_24_P945293	-1.04	-0.61	2.21E-05	1.53E-02
NM_015669	PCDHB5	chr5:140497639-140497698	A_23_P69863	-1.04	-0.61	1.01E-04	2.90E-02
BC006406	FAM104B	chrX:55055661-55055602	A_32_P194025	-1.03	-1.38	3.79E-08	8.97E-12
NM_002528	NTHL1	chr16:2034668-2033706	A_23_P88904	-1.03	-1.03	1.50E-04	1.91E-04
NM_001668	ARNT	chr1:147596176-147596117	A_24_P391568	-1.03	-1.01	1.67E-03	2.74E-03
AB007456	TP53AP1	chr7:86619112-86619053	A_24_P274842	-1.03	-0.92	4.07E-05	3.03E-04
NM_003486	SLC7A5	chr16:86421467-86421408	A_24_P335620	-1.03	-0.91	4.04E-04	2.17E-03
THC2303043	THC2303043	chr2:179130350-179130409	A_32_P66625	-1.03	-0.90	3.43E-06	4.52E-05
AY629351	ZCCHC4	chr4:25047066-25047125	A_23_P212983	-1.03	-0.88	9.99E-06	1.45E-04
NM_012227	GTPBP6	chrX:171124-171065	A_24_P358381	-1.03	-0.84	6.38E-04	6.75E-03
NM_003193	TBCE	chr1:231938185-231938244	A_23_P52147	-1.03	-0.82	2.05E-04	3.92E-03
BC018548	FBXL17	chr5:107223222-107223163	A_32_P137632	-1.03	-0.82	2.44E-04	4.65E-03
NM_003558	PIP5K1B	chr9:68853224-68853283	A_32_P465742	-1.03	-0.82	5.57E-03	3.88E-02
NM_002139	RBMX	chrX:135681371-135681312	A_32_P222857	-1.03	-0.81	2.69E-03	2.67E-02
ENST00000316634	ENST00000316634	chr15:64573580-64573521	A_23_P21776	-1.03	-0.77	3.33E-04	8.73E-03
A_23_P10605	A_23_P10605	chr10:125759178-125758773	A_23_P10605	-1.03	-0.75	4.04E-05	3.12E-03
NM_002969	MAPK12	chr22:48999177-48998069	A_23_P29347	-1.03	-0.73	2.01E-05	2.68E-03
NM_014615	KIAA0182	chr16:84266578-84266637	A_24_P943062	-1.03	-0.73	2.59E-03	4.53E-02
NM_018198	DNAJC11	chr1:6632626-6631777	A_24_P413735	-1.03	-0.72	5.76E-04	2.23E-02
NM_182616	C15orf38	chr15:88252626-88252567	A_23_P106425	-1.03	-0.65	6.20E-05	1.45E-02
NM_002873	RAD17	chr5:68731728-68742110	A_24_P97836	-1.03	-0.64	2.22E-04	2.86E-02
NM_139076	CCDC98	chr4:84739552-84739493	A_23_P253464	-1.03	-0.63	3.18E-07	1.27E-03
AI652920	AI652920	chr10:29011277-29011218	A_32_P122494	-1.03	-0.63	2.58E-04	3.53E-02
AK055981	AK055981	chr15:88418998-88418939	A_24_P765795	-1.02	-1.28	9.79E-03	1.78E-03
NM_000679	ADRA1B	chr5:159332521-159332580	A_23_P33326	-1.02	-1.23	1.84E-02	6.17E-03
A_32_P71113	A_32_P71113	chr9:124784761-124784702	A_32_P71113	-1.02	-1.22	5.59E-07	7.80E-09
NM_016588	NRN1	chr6:5943481-5943422	A_23_P82088	-1.02	-1.12	6.38E-03	3.60E-03
THC2306096	THC2306096	chr1:45829561-45829502	A_24_P842899	-1.02	-1.06	1.04E-07	4.75E-08
AB046850	AB046850	chr10:12234014-12234073	A_32_P875758	-1.02	-1.02	6.98E-04	8.67E-04
AB040888	ODZ3	chr4:184096532-184096591	A_24_P911420	-1.02	-0.94	4.88E-04	1.73E-03
NM_001009954	FLJ20105	chrX:71208190-71208131	A_23_P96325	-1.02	-0.89	1.04E-07	1.94E-06
AK126751	NHLRC2	chr10:115666454-115666513	A_23_P46769	-1.02	-0.86	2.96E-07	1.08E-05
CR610374	CR610374	chr9:124200246-124200305	A_32_P91042	-1.02	-0.84	9.63E-07	4.84E-05
NM_005600	NIT1	chr1:157903036-157903095	A_23_P376661	-1.02	-0.83	1.19E-07	9.87E-06
NM_014042	C11orf51	chr11:71498826-71498608	A_24_P244410	-1.02	-0.82	4.29E-07	3.65E-05
THC2317110	THC2317110	chr4:187382814-187382873	A_32_P171793	-1.02	-0.80	6.70E-08	1.20E-05
NM_000056	BCKDHB	chr6:80969578-80969637	A_23_P93464	-1.02	-0.73	5.30E-06	9.41E-04
NM_181519	SYT15	chr10:46379912-46379853	A_24_P100535	-1.02	-0.66	4.25E-05	9.86E-03
NM_053024	PFN2	chr3:151166092-151166033	A_23_P253301	-1.02	-0.64	3.27E-06	3.36E-03
BC036230	C9orf122	chr9:38612508-38612567	A_24_P886197	-1.02	-0.64	4.65E-06	3.86E-03
NM_001006617	MAPKAP1	chr9:125280177-125280118	A_23_P216894	-1.02	-0.61	3.80E-04	4.71E-02
NM_020853	KIAA1467	chr12:13127392-13127451	A_23_P394567	-1.02	-0.60	3.46E-05	1.85E-02
BC006406	FAM104B	chrX:55055438-55055379	A_23_P11341	-1.01	-1.16	3.01E-06	1.46E-07
NM_033160	ZNF658	chr9:40501292-40501233	A_23_P419202	-1.01	-1.14	3.39E-05	3.90E-06

Gene	Symbol	Chromosomal position	Probe	siCASP8AP2.3	CASP8AP2.6	siCASP8AP2.3	CASP8AP2.6
				M	M	Q	Q
AK123649	AK123649	chr4:129003690-129003749	A_32_P36582	-1.01	-1.06	1.16E-04	6.78E-05
NM_033114	ZCRB1	chr12:40993059-40993000	A_24_P4877	-1.01	-1.06	8.68E-03	8.69E-03
NM_005578	LPP	chr3:190074958-190075017	A_23_P251118	-1.01	-1.01	9.91E-05	1.19E-04
NM_181503	EXOSC8	chr13:36478282-36479155	A_23_P162822	-1.01	-0.95	1.54E-10	9.09E-10
NM_152440	FLJ32549	chr12:62873047-62872988	A_23_P370569	-1.01	-0.90	1.45E-07	1.79E-06
NM_033448	KRT6IRS	chr12:51224230-51224171	A_23_P99044	-1.01	-0.88	1.61E-04	1.13E-03
NM_005197	CHES1	chr14:88693351-88693292	A_23_P88435	-1.01	-0.88	1.62E-04	1.17E-03
NM_004627	WRB	chr21:39687048-39690692	A_23_P80122	-1.01	-0.82	2.04E-06	9.48E-05
NM_006203	PDE4D	chr5:58303115-58303056	A_24_P944519	-1.01	-0.82	4.92E-05	1.03E-03
NM_032172	USP42	chr7:5973672-5973731	A_23_P82394	-1.01	-0.79	8.24E-07	8.81E-05
NM_022039	FBXW4	chr10:103417665-103374531	A_23_P104295	-1.01	-0.77	2.61E-04	6.61E-03
AK022594	AK022594	chr1:202136215-202136156	A_32_P332317	-1.01	-0.75	6.98E-05	3.54E-03
NM_153811	SLC38A6	chr14:60588602-60588874	A_23_P88309	-1.01	-0.71	1.18E-04	7.89E-03
NM_022087	GALNT11	chr7:151255575-151255634	A_23_P8416	-1.01	-0.70	4.99E-06	1.50E-03
ENST00000337530	ENST00000337530	chr9:112511099-112511158	A_32_P35232	-1.01	-0.69	2.90E-07	3.71E-04
NM_006015	ARID1A	chr1:26791320-26791379	A_24_P92952	-1.01	-0.68	1.37E-04	1.24E-02
NM_033425	DIXDC1	chr11:111396977-111397036	A_23_P416289	-1.01	-0.64	4.40E-04	3.50E-02
NM_139076	CCDC98	chr4:84739552-84739493	A_23_P253464	-1.01	-0.63	1.06E-07	5.76E-04
NM_015901	NUDT13	chr10:74561281-74561340	A_32_P41471	-1.00	-1.13	2.72E-08	1.06E-09
NM_003810	TNFSF10	chr3:173706627-173706568	A_23_P121253	-1.00	-1.10	2.66E-02	2.03E-02
NM_031482	ATG10	chr5:81390072-81390131	A_24_P370471	-1.00	-1.02	3.29E-10	2.08E-10
NM_020421	ADCK1	chr14:77467803-77469370	A_23_P25945	-1.00	-1.00	4.67E-07	5.45E-07
AK123649	AK123649	chr4:129004375-129004434	A_32_P159320	-1.00	-0.97	3.33E-06	5.45E-06
A_24_P844100	A_24_P844100	chr5:097756485-097756426	A_24_P844100	-1.00	-0.97	6.60E-04	1.28E-03
NM_000532	PCCB	chr3:137463593-137485423	A_23_P121051	-1.00	-0.96	1.25E-04	2.86E-04
NM_024952	C14orf159	chr14:90761111-90761170	A_23_P48771	-1.00	-0.92	6.16E-10	5.82E-09
BI497361	BI497361	chr8:102266589-102266530	A_32_P220161	-1.00	-0.89	9.38E-04	4.16E-03
NM_001257	CDH13	chr16:82387528-82387587	A_32_P85999	-1.00	-0.89	3.40E-03	1.34E-02
NM_024072	DDX54	chr12:112061907-112061848	A_23_P162374	-1.00	-0.84	2.79E-07	1.08E-05
NM_003362	UNG	chr12:108003384-108003443	A_24_P398585	-1.00	-0.82	1.52E-04	2.33E-03
NM_153686	LCORL	chr4:17521766-17521707	A_32_P54242	-1.00	-0.77	1.57E-08	5.86E-06
NM_018934	PCDHB14	chr5:140585712-140585771	A_23_P133236	-1.00	-0.70	1.44E-06	7.10E-04
ENST00000316149	ENST00000316149	chr6:97444058-97443999	A_24_P46659	-1.00	-0.70	4.24E-05	5.15E-03
NM_181679	NFS1	chr20:33745976-33745917	A_24_P400140	-1.00	-0.70	7.03E-04	2.38E-02
CR592318	CR592318	chr10:81441545-81441486	A_24_P611903	-1.00	-0.68	4.16E-04	2.14E-02
NM_015409	EP400	chr12:131230626-131230685	A_23_P253158	-1.00	-0.64	8.12E-06	4.26E-03
THC2339772	THC2339772	chr2:227422182-227422123	A_24_P802145	-1.00	-0.63	2.45E-04	2.81E-02
NM_015332	NUDCD3	chr7:44196988-44196929	A_23_P31477	-1.00	-0.62	1.10E-04	2.26E-02
AF168717	DTWD1	chr15:47714239-47722833	A_23_P3215	-0.99	-1.19	2.39E-05	7.10E-07
NM_152391	PQLC3	chr2:11269272-11269331	A_23_P131375	-0.99	-1.15	1.04E-04	1.02E-05
NM_005896	IDH1	chr2:208926891-208926832	A_32_P45009	-0.99	-1.04	6.11E-06	2.84E-06
NM_007331	WHSC1	chr4:1910613-1910672	A_23_P307328	-0.99	-0.98	7.88E-07	8.95E-07
NM_178177	NMNAT3	chr3:140762264-140762205	A_24_P260134	-0.99	-0.98	1.90E-04	2.56E-04
THC2273687	THC2273687	chr13:110348571-110348512	A_32_P110433	-0.99	-0.96	6.15E-06	1.20E-05
NM_001668	ARNT	chr1:147597134-147597075	A_23_P550	-0.99	-0.94	1.21E-07	4.59E-07
NM_006963	ZNF22	chr10:44820494-44820553	A_23_P202458	-0.99	-0.92	3.22E-07	1.77E-06
NM_172178	MRPL42	chr12:92398300-92398359	A_24_P352445	-0.99	-0.92	2.45E-05	9.93E-05
NP107055	NP107055	chr7:26843674-26861062	A_24_P254915	-0.99	-0.85	1.81E-04	1.42E-03
NM_019845	RPRM	chr2:154159743-154159684	A_23_P5370	-0.99	-0.84	2.51E-03	1.47E-02
NM_152891	PRSS33	chr16:2774350-2774291	A_24_P327084	-0.99	-0.79	1.24E-03	1.43E-02
NM_003481	USP5	chr12:6844161-6844220	A_23_P64954	-0.99	-0.78	1.23E-03	1.42E-02
AF161353	AF161353	chr9:124809061-124809002	A_32_P193792	-0.99	-0.77	2.60E-05	1.21E-03
THC2304438	THC2304438	chr5:133966052-133965993	A_24_P916845	-0.99	-0.76	7.10E-09	3.38E-06
NM_002746	MAPK3	chr16:30035797-30035738	A_23_P37910	-0.99	-0.76	7.71E-06	6.08E-04
A_32_P94976	A_32_P94976	chr1:209282716-209282657	A_32_P94976	-0.99	-0.73	2.83E-06	5.56E-04
NM_182616	C15orf38	chr15:88245903-88245844	A_24_P404033	-0.99	-0.70	7.13E-04	2.37E-02

Gene	Symbol	Chromosomal position	Probe	siCASP8AP2.3	CASP8AP2.6	siCASP8AP2.3	CASP8AP2.6
				M	M	Q	Q
NM_018052	VAC14	chr16:69286957-69284326	A_23_P77593	-0.99	-0.69	2.66E-07	2.37E-04
BC110326	DKFZP779L1068	chr8:93965137-93965078	A_32_P117313	-0.99	-0.68	4.41E-04	2.18E-02
NM_007080	LSM6	chr4:147468576-147468635	A_23_P259451	-0.99	-0.67	3.18E-07	3.73E-04
A_24_P153003	A_24_P153003	chr3:171104328-171104269	A_24_P153003	-0.99	-0.63	4.10E-04	3.27E-02
BP872463	BP872463	chr14:34502974-34502915	A_32_P231265	-0.99	-0.62	9.54E-08	5.01E-04
NM_016319	COPS7A	chr12:6710591-6710650	A_23_P2474	-0.98	-1.24	1.79E-06	7.80E-09
NM_152444	ZADH1	chr14:73418886-73420607	A_23_P48713	-0.98	-1.02	5.49E-06	2.66E-06
AK054645	RP11-138L21.1	chr9:40136039-40130752	A_24_P652609	-0.98	-1.01	8.89E-05	6.92E-05
CR936771	CR936771	chr1:115025139-115025080	A_24_P937855	-0.98	-0.97	1.35E-07	1.82E-07
AF533250	ZNF397	chr18:31079956-31080015	A_23_P208143	-0.98	-0.97	5.25E-04	7.41E-04
NM_020747	ZNF608	chr5:124001444-124001385	A_23_P169978	-0.98	-0.91	7.58E-08	4.56E-07
THC2401493	THC2401493	chr5:34219955-34219896	A_32_P67577	-0.98	-0.85	6.08E-03	2.44E-02
NM_139076	CCDC98	chr4:84739552-84739493	A_23_P253464	-0.98	-0.74	4.02E-06	4.51E-04
AK130366	AK130366	chr13:22390221-22390280	A_32_P101334	-0.98	-0.74	4.60E-06	5.49E-04
NM_001813	CENPE	chr4:104385002-104384943	A_23_P253524	-0.98	-0.74	1.53E-05	1.04E-03
NM_032026	TATDN1	chr8:125589923-125585738	A_32_P224911	-0.98	-0.71	2.66E-07	1.26E-04
A_24_P471242	A_24_P471242	chr18:009668716-009668617	A_24_P471242	-0.98	-0.71	9.28E-05	5.08E-03
NM_018486	HDAC8	chrX:71464921-71354697	A_23_P84922	-0.98	-0.67	1.52E-05	3.20E-03
NM_032433	ZNF333	chr19:14692167-14692226	A_23_P5221	-0.98	-0.66	3.00E-07	3.52E-04
NM_015975	TAF9B	chrX_random:1392552-1392611	A_24_P391431	-0.98	-0.66	3.71E-04	2.28E-02
BE540788	BE540788	chr19:39653695-39653754	A_24_P926935	-0.98	-0.61	3.15E-06	3.74E-03
NM_018706	DHTKD1	chr10:12182228-12183066	A_23_P12601	-0.98	-0.61	3.70E-05	1.27E-02
A_32_P95973	A_32_P95973	chr1:042552887-042552946	A_32_P95973	-0.97	-1.68	1.27E-03	1.67E-07
ENST00000380239	ENST00000380239	chr8:28487515-28487574	A_32_P211708	-0.97	-1.10	7.22E-05	1.01E-05
NM_022037	TIA1	chr2:70369545-70368049	A_24_P417474	-0.97	-1.06	1.17E-04	3.37E-05
NM_080650	ATPBD4	chr15:33451468-33451409	A_23_P77135	-0.97	-0.94	4.85E-07	9.39E-07
NM_024047	NUDT9	chr4:88736472-88736531	A_23_P94860	-0.97	-0.87	2.60E-03	9.58E-03
BC007377	PCGF5	chr10:92977438-92977497	A_23_P202117	-0.97	-0.86	2.62E-10	6.61E-09
NM_018480	TMEM126B	chr11:85022891-85022950	A_23_P2129	-0.97	-0.83	5.56E-05	5.99E-04
A_24_P212834	A_24_P212834	chr11:059422955-059423016	A_24_P212834	-0.97	-0.82	8.29E-06	1.62E-04
NM_022748	TNS3	chr7:47088518-47088459	A_24_P346431	-0.97	-0.81	2.71E-06	8.33E-05
NM_016436	PHF20	chr20:33999513-33999572	A_23_P210969	-0.97	-0.79	1.68E-06	7.60E-05
NM_005170	ASCL2	chr11:2248027-2247968	A_23_P105088	-0.97	-0.79	1.51E-03	1.30E-02
A_24_P255314	A_24_P255314	chr12:006418105-006418164	A_24_P255314	-0.97	-0.77	2.19E-03	2.06E-02
NM_012279	ZNF346	chr5:176425838-176425897	A_23_P258124	-0.97	-0.76	3.30E-07	3.77E-05
NM_145012	C10orf9	chr10:35898735-35898794	A_23_P300905	-0.97	-0.76	2.73E-05	1.08E-03
NM_012415	RAD54B	chr8:95453591-95453532	A_23_P82738	-0.97	-0.74	8.77E-06	7.45E-04
NM_012415	RAD54B	chr8:95453591-95453532	A_23_P82738	-0.97	-0.73	6.68E-05	2.83E-03
A_24_P349606	A_24_P349606	chr9:085743537-085743598	A_24_P349606	-0.97	-0.73	5.66E-04	1.29E-02
BX537532	LOC550643	chrX:56727368-56727424	A_24_P637257	-0.97	-0.70	1.32E-03	2.94E-02
NM_012241	SIRT5	chr6:13720307-13720366	A_23_P214812	-0.97	-0.69	2.81E-05	3.23E-03
NM_001007794	CEPT1	chr1:111438126-111438185	A_24_P233944	-0.97	-0.69	2.73E-04	1.29E-02
AK026368	AK026368	chr9:045536673-045536614	A_32_P216888	-0.97	-0.66	5.23E-09	2.66E-05
THC2336404	THC2336404	chr3:121023359-121023300	A_24_P243044	-0.97	-0.65	6.18E-07	6.45E-04
NM_030790	ITFG1	chr16:45750391-45750332	A_23_P334123	-0.97	-0.64	4.40E-07	6.50E-04
NM_016653	ZAK	chr2:173958160-173958219	A_23_P366394	-0.97	-0.62	2.06E-05	6.96E-03
NM_016422	RNF141	chr11:10493053-10492994	A_23_P139066	-0.96	-1.35	2.54E-06	5.47E-10
NM_018951	HOXA10	chr7:26984924-26984865	A_23_P253368	-0.96	-1.23	4.14E-04	1.09E-05
NM_033160	ZNF658	chr9:40500832-40500773	A_32_P220739	-0.96	-1.21	1.51E-09	1.07E-12
NM_005647	TBL1X	chrX:9497208-9497267	A_24_P417162	-0.96	-1.16	1.09E-06	1.30E-08
NM_012160	FBXL4	chr6:99428963-99428904	A_23_P214739	-0.96	-1.13	4.10E-03	1.07E-03
NM_003810	TNFSF10	chr3:173706627-173706568	A_23_P121253	-0.96	-1.12	2.94E-02	1.55E-02
NR_002776	MCM3APAS	chr21:46495954-46496013	A_24_P117301	-0.96	-1.09	7.33E-03	3.22E-03
THC2437122	THC2437122	chr4:164403490-164403431	A_32_P13371	-0.96	-1.00	1.08E-10	3.15E-11
NM_014236	GNPAT	chr1:227717785-227717928	A_23_P85777	-0.96	-0.96	1.27E-03	1.50E-03
NM_178498	SLC5A12	chr11:26676596-26676537	A_23_P323943	-0.96	-0.95	3.48E-03	5.54E-03

Gene	Symbol	Chromosomal position	Probe	siCASP8AP2.3	CASP8AP2.6	siCASP8AP2.3	CASP8AP2.6
				M	M	Q	Q
NM_004375	COX11	chr17:50395761-50395702	A_24_P391531	-0.96	-0.90	4.36E-03	1.09E-02
A_24_P212355	A_24_P212355	chr5:078331819-078331758	A_24_P212355	-0.96	-0.87	1.05E-04	5.58E-04
NM_013438	UBQLN1	chr9:83505278-83505219	A_24_P329597	-0.96	-0.85	3.86E-04	1.96E-03
NM_003918	GYG2	chrX:2793476-2793535	A_23_P217704	-0.96	-0.85	4.79E-04	2.81E-03
ENST00000376356	ENST00000376356	chr10:25354583-25354642	A_23_P127002	-0.96	-0.83	1.23E-09	5.92E-08
NM_001813	CENPE	chr4:104385002-104384943	A_23_P253524	-0.96	-0.80	2.55E-05	4.66E-04
NM_004640	BAT1	chr6:31612339-31612280	A_24_P904903	-0.96	-0.76	4.36E-07	4.24E-05
NM_006347	PPIH	chr1:42793222-42793612	A_23_P200940	-0.96	-0.72	2.04E-04	6.58E-03
THC2302062	THC2302062	chr8:117723836-117723777	A_24_P501698	-0.96	-0.71	6.76E-09	6.91E-06
ENST00000366848	ENST00000366848	chr1:221461912-221461971	A_23_P333951	-0.96	-0.69	2.04E-03	3.87E-02
NM_003590	CUL3	chr2:225163942-225163883	A_24_P140030	-0.96	-0.67	9.28E-05	8.52E-03
NM_030914	C9orf74	chr9:128231097-128231512	A_23_P94689	-0.96	-0.61	1.35E-08	1.43E-04
NM_004849	ATG5	chr6:106739504-106739445	A_24_P175059	-0.95	-1.18	8.05E-05	1.86E-06
NM_016063	HDDC2	chr6:125640038-125639979	A_24_P55250	-0.95	-1.04	2.70E-03	1.41E-03
NM_000110	DPYD	chr1:97255980-97255921	A_23_P135548	-0.95	-1.03	5.66E-03	3.70E-03
NM_138493	C6orf129	chr6:37560586-37559007	A_23_P133770	-0.95	-1.01	5.71E-07	1.41E-07
BX648347	VPS41	chr7:38536254-38536195	A_32_P25823	-0.95	-0.98	1.24E-03	1.13E-03
NM_176869	PPA2	chr4:106665339-106648504	A_24_P214625	-0.95	-0.93	5.51E-03	9.09E-03
NM_014380	NGFRAP1	chrX:102439053-102439112	A_23_P45524	-0.95	-0.83	6.78E-08	1.40E-06
ENST00000367142	ENST00000367142	chr1:202413956-202413897	A_24_P145122	-0.95	-0.76	6.98E-09	1.59E-06
NM_001813	CENPE	chr4:104385002-104384943	A_23_P253524	-0.95	-0.76	2.38E-05	7.44E-04
NM_003905	APPBP1	chr16:65394410-65394351	A_23_P77459	-0.95	-0.75	4.14E-05	1.28E-03
THC2397757	THC2397757	chr8:56903419-56903478	A_32_P82610	-0.95	-0.71	6.15E-06	6.55E-04
NM_016055	MRPL48	chr11:73253230-73253289	A_23_P162106	-0.95	-0.71	1.70E-05	1.31E-03
NM_002926	RGS12	chr4:3470262-3470321	A_23_P352435	-0.95	-0.69	1.01E-04	5.94E-03
THC2405886	THC2405886	chr1:109192288-109192347	A_32_P9253	-0.95	-0.66	1.03E-03	3.32E-02
NM_139076	CCDC98	chr4:84739552-84739493	A_23_P253464	-0.95	-0.64	3.25E-06	1.62E-03
NM_139076	CCDC98	chr4:84739552-84739493	A_23_P253464	-0.95	-0.60	7.87E-06	4.97E-03
NM_016056	TMBIM4	chr12:64849916-64833451	A_23_P13701	-0.94	-1.45	2.41E-04	9.48E-08
NM_033028	BBS4	chr15:70815310-70816164	A_24_P176284	-0.94	-1.29	1.32E-03	1.77E-05
NM_015411	SUMF2	chr7:55921888-55921947	A_23_P123265	-0.94	-1.09	6.94E-06	4.12E-07
NM_032814	TMEM118	chr12:115753235-115753294	A_23_P204158	-0.94	-1.06	1.96E-05	2.21E-06
A_24_P401601	A_24_P401601	chr19:020937244-020937305	A_24_P401601	-0.94	-0.91	1.82E-04	3.51E-04
NM_014346	TBC1D22A	chr22:45827937-45827996	A_23_P109593	-0.94	-0.89	7.13E-08	2.92E-07
NM_022782	MPHOSPH9	chr12:122171602-122171543	A_24_P398500	-0.94	-0.88	1.75E-06	6.99E-06
NM_003909	CPNE3	chr8:87642058-87642117	A_24_P64126	-0.94	-0.82	3.05E-06	4.56E-05
NM_032143	ZRANB3	chr2:135798848-135798789	A_24_P367623	-0.94	-0.78	3.61E-08	2.28E-06
NM_152636	METT5D1	chr11:28310262-28310321	A_32_P19887	-0.94	-0.77	5.23E-05	1.08E-03
NM_013245	VPS4A	chr16:67916219-67916278	A_23_P329772	-0.94	-0.76	2.59E-07	1.94E-05
THC2314754	THC2314754	chr22:29376238-29376179	A_24_P669216	-0.94	-0.76	3.48E-06	1.72E-04
NM_015185	ARHGEF9	chrX:62658544-62658485	A_23_P251705	-0.94	-0.76	3.34E-05	8.03E-04
NM_016835	MAPT	chr17:41458383-41458442	A_23_P207699	-0.94	-0.75	7.16E-06	3.35E-04
NM_032311	POLDIP3	chr22:41304728-41304669	A_23_P502269	-0.94	-0.74	9.38E-10	3.95E-07
NM_014048	MKL2	chr16:14268047-14268106	A_23_P54556	-0.94	-0.73	1.14E-06	1.28E-04
NM_012415	RAD54B	chr8:95453591-95453532	A_23_P82738	-0.94	-0.71	3.38E-05	1.99E-03
NM_014763	MRPL19	chr2:75794314-75794373	A_23_P102262	-0.94	-0.66	1.11E-10	9.20E-07
NM_024900	PHF17	chr4:130142267-130142326	A_23_P410587	-0.94	-0.66	4.33E-05	4.57E-03
NM_016218	POLK	chr5:74928485-74928544	A_24_P303160	-0.94	-0.66	5.10E-04	2.15E-02
AK056550	ZBTB8OS	chr1:32820527-32820586	A_24_P468810	-0.94	-0.64	9.43E-07	7.37E-04
AV724325	AV724325	chr8:119271267-119271326	A_32_P47265	-0.94	-0.61	2.58E-06	2.08E-03
NM_002746	MAPK3	chr16:30035797-30035738	A_23_P37910	-0.94	-0.61	1.50E-05	5.95E-03
NM_145697	CDCA1	chr1:160056802-160056861	A_23_P74349	-0.94	-0.60	8.73E-08	4.22E-04
BQ184357	BQ184357	chr3:126167572-126167513	A_32_P166693	-0.93	-1.10	3.56E-02	1.74E-02
AK002152	STAU2	chr8:74495477-74495418	A_24_P374634	-0.93	-1.04	2.81E-05	4.05E-06
NM_001008397	LOC493869	chr5:54496011-54496070	A_23_P122052	-0.93	-0.96	3.02E-04	2.54E-04
BC062636	RHBDD1	chr2:227687954-227688013	A_23_P147514	-0.93	-0.92	4.82E-08	6.51E-08

Gene	Symbol	Chromosomal position	Probe	siCASP8AP2.3	CASP8AP2.6	siCASP8AP2.3	CASP8AP2.6
				M	M	Q	Q
X66610	ENO1B		A_23_P259892	-0.93	-0.92	1.80E-02	2.78E-02
NM_030962	SBF2	chr11:9757485-9757426	A_23_P419795	-0.93	-0.89	8.06E-06	1.84E-05
NM_145212	MRPL30	chr2:99272313-99272372	A_24_P302695	-0.93	-0.88	6.55E-04	1.61E-03
NM_001002258	ATP5G3	chr2:175867678-175867619	A_24_P174346	-0.93	-0.87	3.37E-03	8.46E-03
A_24_P15803	A_24_P15803	chr5:002841134-002841071	A_24_P15803	-0.93	-0.84	1.68E-04	7.89E-04
NM_007350	PHLDA1	chr12:74707272-74707213	A_24_P943597	-0.93	-0.84	2.73E-04	1.29E-03
THC2441234	THC2441234	chr4:144508594-144508653	A_32_P190613	-0.93	-0.82	6.32E-07	8.31E-06
AK027610	AK027610	chr2:3083539-3083480	A_24_P937582	-0.93	-0.77	4.10E-04	4.63E-03
NM_001813	CENPE	chr4:104385002-104384943	A_23_P253524	-0.93	-0.75	3.47E-05	8.61E-04
AK074557	TMED4	chr7:44393915-44393856	A_24_P297827	-0.93	-0.74	1.58E-08	3.42E-06
NM_033119	NKD1	chr16:49225386-49225445	A_23_P429449	-0.93	-0.74	3.36E-04	5.83E-03
A_32_P72477	A_32_P72477	chr5:022007685-022007744	A_32_P72477	-0.93	-0.73	4.36E-04	7.71E-03
NM_001966	EHHADH	chr3:186392185-186392126	A_24_P123119	-0.93	-0.70	1.73E-04	5.94E-03
NM_018073	TRIM68	chr11:4576915-4576856	A_23_P2097	-0.93	-0.69	1.63E-06	2.98E-04
NM_018098	ECT2	chr3:174021829-174021888	A_23_P9574	-0.93	-0.65	4.11E-05	4.95E-03
BC064616	ZRANB3	chr2:135791265-135791206	A_23_P412059	-0.93	-0.61	5.83E-07	7.89E-04
NM_020673	RAB22A	chr20:56374578-56374637	A_32_P179258	-0.93	-0.60	3.31E-04	2.88E-02
NM_018847	KLHL9	chr9:21321160-21321101	A_23_P83159	-0.92	-1.40	7.43E-06	2.59E-10
NM_020186	ACN9	chr7:96455438-96455497	A_23_P59528	-0.92	-1.19	5.58E-05	4.37E-07
NM_004536	BIRC1	chr5:70300406-70300347	A_23_P110473	-0.92	-1.03	1.52E-03	5.27E-04
NM_138711	PPARG	chr3:12433499-12433558	A_23_P252062	-0.92	-1.03	2.00E-03	7.98E-04
NM_006992	LRRC23	chr12:6886004-6886063	A_23_P36689	-0.92	-1.01	6.61E-04	2.37E-04
NM_138711	PPARG	chr3:12433499-12433558	A_23_P252062	-0.92	-1.00	1.99E-03	1.07E-03
NM_032042	C5orf21	chr5:92979751-92979692	A_24_P111912	-0.92	-0.95	7.66E-12	2.50E-12
NM_014990	GARNL1	chr14:35077827-35077768	A_23_P151570	-0.92	-0.95	5.17E-06	2.55E-06
NM_003409	ZFP161	chr18:5280698-5280639	A_23_P27334	-0.92	-0.93	1.57E-07	1.05E-07
NM_022104	C20orf67	chr20:44009424-44009483	A_23_P210496	-0.92	-0.92	9.45E-11	9.68E-11
NM_138362	FAM104B	chrX:55053293-55053234	A_23_P415061	-0.92	-0.88	4.78E-07	1.24E-06
NM_012415	RAD54B	chr8:95453591-95453532	A_23_P82738	-0.92	-0.86	3.93E-05	1.19E-04
NM_016948	PARD6A	chr16:66253663-66253722	A_23_P140821	-0.92	-0.83	6.24E-06	4.32E-05
NM_145290	GPR125	chr4:22066152-22066093	A_23_P158933	-0.92	-0.80	6.94E-04	4.06E-03
NM_015655	ZNF337	chr20:25603074-25603015	A_23_P154758	-0.92	-0.76	1.05E-04	1.63E-03
NM_014140	SMARCAL1	chr2:217168473-217172970	A_23_P131337	-0.92	-0.72	6.56E-07	7.16E-05
NM_006628	ARPP-19	chr15:50627354-50627295	A_24_P313262	-0.92	-0.69	2.87E-06	3.85E-04
NM_021069	SORBS2	chr4:186911146-186908237	A_24_P140475	-0.92	-0.68	2.49E-04	8.15E-03
NM_006706	TCERG1	chr5:145870399-145870458	A_23_P133365	-0.92	-0.68	2.86E-04	9.17E-03
NM_002607	PDGFA	chr7_random:671741-671682	A_23_P113701	-0.92	-0.65	4.29E-04	1.76E-02
NM_138376	TTC5	chr14:19833315-19830072	A_23_P88280	-0.92	-0.64	1.16E-03	3.15E-02
NM_006793	PRDX3	chr10:120921966-120921907	A_23_P63751	-0.91	-1.30	2.25E-02	1.54E-03
NM_052845	MMAB	chr12:108459643-108457625	A_24_P364381	-0.91	-1.13	2.26E-04	9.28E-06
NM_003810	TNFSF10	chr3:173706627-173706568	A_23_P121253	-0.91	-1.11	3.54E-02	1.32E-02
NM_004772	C5orf13	chr5:111093049-111092990	A_24_P149124	-0.91	-1.05	7.09E-03	2.47E-03
NM_145309	LRRC51	chr11:71485480-71485539	A_24_P184385	-0.91	-1.04	1.52E-08	3.38E-10
THC2338854	THC2338854	chr1:35735523-35735464	A_32_P212333	-0.91	-0.95	9.24E-09	2.82E-09
NM_018346	RSAD1	chr17:45918054-45918113	A_23_P152807	-0.91	-0.88	3.89E-05	7.02E-05
NM_019557	FAM54B	chr1:25842911-25842970	A_23_P97195	-0.91	-0.87	1.09E-05	2.88E-05
NM_032750	ABHD14B	chr3:51977999-51977940	A_23_P155417	-0.91	-0.71	3.84E-05	1.53E-03
THC2429214	THC2429214	chr19:37895630-37895689	A_32_P93391	-0.91	-0.71	1.10E-03	1.53E-02
NM_152587	C11orf65	chr11:107769270-107769211	A_23_P418485	-0.91	-0.66	3.77E-08	3.86E-05
AK000177	C6orf61	chr6:119242166-119242107	A_23_P30630	-0.91	-0.64	1.33E-06	4.98E-04
AF034176	AF034176	chr6:34362170-34362229	A_24_P826485	-0.91	-0.63	5.35E-05	6.56E-03
NM_020201	NT5M	chr17:17191313-17191372	A_23_P3946	-0.91	-0.60	6.00E-04	3.27E-02
AF168717	DTWD1	chr15:47714239-47722833	A_23_P3215	-0.90	-1.20	1.54E-04	1.13E-06
A_24_P418780	A_24_P418780	chr7:134587255-134587314	A_24_P418780	-0.90	-1.07	2.27E-02	9.26E-03
NM_005602	CLDN11	chr3:171633374-171633433	A_23_P29800	-0.90	-1.04	1.08E-07	2.95E-09
NM_024122	FAM121B	chrX:23611271-23611212	A_23_P61127	-0.90	-1.02	4.11E-05	4.60E-06

Gene	Symbol	Chromosomal position	Probe	siCASP8AP2.3	CASP8AP2.6	siCASP8AP2.3	CASP8AP2.6
				M	M	Q	Q
NM_152705	MGC9850	chr13:27138914-27138973	A_23_P409541	-0.90	-0.95	3.14E-03	2.46E-03
NM_033115	MGC16169	chr4:107395126-107395067	A_23_P133075	-0.90	-0.92	9.60E-07	5.74E-07
M64109	COL14A1	chr8:121298083-121298142	A_24_P222591	-0.90	-0.84	7.88E-07	3.50E-06
ENST00000373644	ENST00000373644	chr10:70123542-70123601	A_32_P202134	-0.90	-0.83	8.35E-04	2.88E-03
NM_013272	SLCO3A1	chr15:90495197-90506997	A_24_P336276	-0.90	-0.83	1.88E-02	4.44E-02
NM_002828	PTPN2	chr18:12784039-12783980	A_23_P309701	-0.90	-0.81	6.14E-04	2.50E-03
NM_001004356	FGFRL1	chr4:1008559-1008618	A_24_P46212	-0.90	-0.80	4.21E-04	2.20E-03
NM_024713	C15orf29	chr15:32226922-32226773	A_32_P58163	-0.90	-0.78	1.56E-07	3.35E-06
NM_018098	ECT2	chr3:174021032-174021091	A_23_P44684	-0.90	-0.77	6.70E-05	6.85E-04
ENST00000314571	ENST00000314571	chr3:12556040-12556099	A_32_P53538	-0.90	-0.75	9.25E-03	4.23E-02
BC040991	BC040991	chr3:143146595-143146536	A_24_P414169	-0.90	-0.74	7.21E-08	5.44E-06
NM_016331	ZNF639	chr3:180534591-180534650	A_24_P123155	-0.90	-0.73	1.74E-04	3.08E-03
NM_080476	CDC91L1	chr20:32696788-32695681	A_24_P457912	-0.90	-0.67	1.21E-05	1.20E-03
NM_001909	CTSD	chr11:1732780-1732721	A_23_P52556	-0.90	-0.64	2.00E-06	6.48E-04
XM_209655	LOC285544	chr4:9380982-9380923	A_32_P65395	-0.90	-0.62	3.71E-05	4.99E-03
NM_016436	PHF20	chr20:34001045-34001104	A_24_P297098	-0.90	-0.62	1.21E-04	9.47E-03
AF168717	DTWD1	chr15:47714239-47722833	A_23_P3215	-0.89	-1.34	1.50E-04	5.59E-08
NM_003810	TNFSF10	chr3:173706627-173706568	A_23_P121253	-0.89	-1.22	5.00E-02	8.97E-03
NM_002814	PSMD10	chrX:107134354-107134295	A_23_P258570	-0.89	-1.07	1.01E-05	2.69E-07
THC2436370	THC2436370	chr5:69321249-69321308	A_32_P187817	-0.89	-1.02	3.34E-03	9.92E-04
ENST00000380985	ENST00000380985	chr5:65160588-65160647	A_32_P99690	-0.89	-1.01	3.94E-06	2.42E-07
BG292169	BG292169	chr5:70367329-70367271	A_24_P586660	-0.89	-0.98	5.20E-05	1.24E-05
NM_002028	FNTB	chr14:64598767-64598826	A_23_P25835	-0.89	-0.98	1.62E-03	6.53E-04
AK098220	AK098220	chr5:69467081-69467022	A_32_P51518	-0.89	-0.95	3.82E-03	2.59E-03
NM_015684	ATP5S	chr14:49859145-49859204	A_23_P37296	-0.89	-0.93	1.71E-09	4.46E-10
NM_152522	ARL6IP6	chr2:153442296-153442355	A_23_P28169	-0.89	-0.93	1.41E-06	6.20E-07
THC2279735	THC2279735	chr2:120825342-120825401	A_23_P153958	-0.89	-0.91	5.67E-03	6.70E-03
ENST00000249760	ENST00000249760	chr15:38495656-38495822	A_23_P129313	-0.89	-0.90	2.44E-04	2.94E-04
AK123506	AK123506	chr3:190085261-190085320	A_32_P193218	-0.89	-0.89	9.04E-05	1.01E-04
NM_152417	TMEM68	chr8:56838034-56837975	A_32_P15464	-0.89	-0.88	1.28E-04	2.04E-04
NM_033300	LRP8	chr1:53424560-53424501	A_23_P34325	-0.89	-0.86	1.01E-03	1.96E-03
NM_006729	DIAPH2	chrX:96666022-96666081	A_32_P50834	-0.89	-0.84	1.17E-04	3.37E-04
NM_013380	ZNF228	chr19:49522823-49522764	A_23_P107724	-0.89	-0.83	8.34E-05	2.53E-04
NM_020234	DTWD1	chr15:47722909-47722968	A_23_P3212	-0.89	-0.81	6.34E-06	3.55E-05
NM_145201	NAPRT1	chr8:144730042-144729983	A_23_P43238	-0.89	-0.78	2.48E-04	1.62E-03
A_32_P182070	A_32_P182070	chrX:056114534-056114593	A_32_P182070	-0.89	-0.75	6.65E-06	1.28E-04
NM_002803	PSMC2	chr7:102601948-102602007	A_23_P304287	-0.89	-0.74	7.59E-05	1.09E-03
D13642	SF3B3	chr16:69166543-69166602	A_24_P945396	-0.89	-0.72	2.83E-03	2.36E-02
NM_015532	GRINL1A	chr15:55796958-55797017	A_32_P117098	-0.89	-0.69	8.24E-07	9.56E-05
A_32_P74901	A_32_P74901	chr15:059932948-059933007	A_32_P74901	-0.89	-0.67	4.70E-03	4.79E-02
NM_016647	C8orf55	chr8:143814812-143814871	A_23_P257057	-0.89	-0.66	1.32E-03	2.47E-02
ENST00000354937	ENST00000354937	chr3:197191020-197190961	A_24_P58944	-0.89	-0.66	2.51E-03	3.43E-02
NM_006390	IPO8	chr12:30678322-30676192	A_23_P98923	-0.89	-0.62	1.28E-05	2.44E-03
NM_005102	FEZ2	chr2:36691414-36691355	A_23_P39718	-0.89	-0.62	3.11E-05	4.24E-03
NM_002746	MAPK3	chr16:30035797-30035738	A_23_P37910	-0.89	-0.60	1.74E-04	1.43E-02
NM_018341	C6orf70	chr6:169993731-169993790	A_32_P129530	-0.88	-1.25	2.29E-06	4.68E-10
AF168717	DTWD1	chr15:47714239-47722833	A_23_P3215	-0.88	-1.17	1.70E-04	1.32E-06
NM_003810	TNFSF10	chr3:173706627-173706568	A_23_P121253	-0.88	-1.17	4.66E-02	1.11E-02
NM_138711	PPARG	chr3:12433499-12433558	A_23_P252062	-0.88	-1.04	2.73E-03	5.24E-04
BC003583	FTO	chr16:52705614-52705673	A_23_P113184	-0.88	-0.99	6.68E-08	3.35E-09
NM_152289	ZNF561	chr19:9580208-9580149	A_23_P433107	-0.88	-0.98	1.66E-02	1.06E-02
NM_138711	PPARG	chr3:12433499-12433558	A_23_P252062	-0.88	-0.97	2.37E-03	1.02E-03
NM_000424	KRT5	chr12:51194948-51194889	A_23_P218047	-0.88	-0.97	1.07E-02	7.12E-03
NM_000213	ITGB4	chr17:71265173-71265232	A_23_P66355	-0.88	-0.93	1.03E-02	1.01E-02
NM_012105	BACE2	chr21:41569566-41569625	A_23_P154875	-0.88	-0.92	8.37E-06	4.44E-06
NM_006393	NEBL	chr10:21110603-21110544	A_24_P398147	-0.88	-0.92	1.99E-02	2.15E-02

Gene	Symbol	Chromosomal position	Probe	siCASP8AP2.3	CASP8AP2.6	siCASP8AP2.3	CASP8AP2.6
				M	M	Q	Q
NM_016025	METTL9	chr16:21574132-21574191	A_24_P112984	-0.88	-0.89	1.45E-03	1.60E-03
NM_006403	NEDD9	chr6:11292688-11292629	A_23_P344555	-0.88	-0.89	9.38E-03	1.26E-02
NM_007043	KRR1	chr12:74178352-74178293	A_32_P326819	-0.88	-0.85	1.30E-03	2.59E-03
NM_201280	MUTED	chr6:7959277-7959218	A_24_P106591	-0.88	-0.77	5.40E-03	2.08E-02
NM_152834	TMEM18	chr2:659366-659307	A_23_P79599	-0.88	-0.75	6.79E-09	4.09E-07
NM_001813	CENPE	chr4:104385002-104384943	A_23_P253524	-0.88	-0.75	1.01E-04	1.07E-03
CR621132	CR621132	chr4:40655698-40655639	A_24_P234701	-0.88	-0.75	2.45E-04	2.33E-03
NM_001813	CENPE	chr4:104385002-104384943	A_23_P253524	-0.88	-0.72	6.37E-05	1.30E-03
NM_052875	VPS26B	chr11:133622498-133622557	A_24_P107336	-0.88	-0.72	1.13E-03	9.86E-03
NM_001813	CENPE	chr4:104385002-104384943	A_23_P253524	-0.88	-0.71	6.88E-05	1.65E-03
ENST00000324982	ENST00000324982	chr2:216909749-216909808	A_23_P369047	-0.88	-0.69	2.40E-03	2.29E-02
NM_012405	ICMT	chr1:6215641-6215582	A_24_P128524	-0.88	-0.69	2.53E-03	2.32E-02
NM_022909	CENPH	chr5:68541405-68541464	A_23_P110802	-0.88	-0.69	3.47E-03	3.11E-02
BE467780	BE467780	chr1:53421049-53420990	A_32_P98979	-0.88	-0.68	2.79E-07	4.56E-05
NM_005523	HOXA11	chr7:26995492-26995433	A_23_P42706	-0.88	-0.65	1.39E-05	1.39E-03
NM_004104	FASN	chr17:77631358-77630768	A_23_P44132	-0.88	-0.65	1.97E-03	3.26E-02
NM_139076	CCDC98	chr4:84739552-84739493	A_23_P253464	-0.88	-0.64	2.48E-06	4.85E-04
ENST00000361204	ENST00000361204	chr22:40627051-40627110	A_23_P166502	-0.88	-0.62	9.54E-05	7.74E-03
NM_031438	NUDT12	chr5:102913174-102913115	A_23_P259090	-0.87	-1.30	7.63E-05	2.28E-08
AF168717	DTWD1	chr15:47714239-47722833	A_23_P3215	-0.87	-1.14	2.80E-04	3.99E-06
NM_024611	NARG2	chr15:58501996-58501937	A_32_P115701	-0.87	-1.13	4.51E-03	3.53E-04
NM_015958	DPH5	chr1:101167914-101167855	A_23_P148821	-0.87	-1.07	9.51E-05	3.10E-06
NM_053053	TADA1L	chr1:163558054-163557995	A_23_P74344	-0.87	-0.96	7.49E-05	1.72E-05
NM_016625	RSRC1	chr3:159322711-159322770	A_23_P158794	-0.87	-0.96	1.26E-04	3.13E-05
NM_032151	PCBD2	chr5:134332778-134332837	A_24_P180423	-0.87	-0.92	1.16E-06	3.51E-07
NM_001813	CENPE	chr4:104385002-104384943	A_23_P253524	-0.87	-0.88	2.22E-05	2.42E-05
NM_018397	CHDH	chr3:53826779-53826720	A_23_P357185	-0.87	-0.88	6.16E-04	6.86E-04
NM_015892	GALNAC4S-6ST	chr10:125757350-125757291	A_23_P383986	-0.87	-0.81	6.35E-04	1.70E-03
NM_017410	HOXC13	chr12:52626301-52626360	A_23_P64808	-0.87	-0.79	3.22E-07	2.84E-06
NM_003940	USP13	chr3:180966259-180966318	A_23_P40989	-0.87	-0.77	1.69E-08	3.06E-07
NM_012415	RAD54B	chr8:95453591-95453532	A_23_P82738	-0.87	-0.76	1.08E-04	8.38E-04
AK130486	AK130486	chr12:119019388-119019447	A_32_P84580	-0.87	-0.72	1.10E-06	5.01E-05
NM_022336	EDAR	chr2:108970207-108970148	A_23_P120281	-0.87	-0.72	1.55E-03	1.14E-02
NM_001037332	CYFIP2	chr5:156754713-156754772	A_23_P167509	-0.87	-0.71	2.87E-04	3.67E-03
NM_004412	DNMT2	chr10:17229021-17228962	A_24_P252705	-0.87	-0.70	1.71E-04	2.71E-03
AB032969	KIAA1143	chr3:44765278-44765242	A_32_P26738	-0.87	-0.69	3.96E-04	6.58E-03
BC020640	C1orf121	chr1:241145650-241145709	A_24_P922808	-0.87	-0.69	4.17E-03	3.30E-02
AF161360	ZDHHC21	chr9:014545542-014545483	A_24_P102091	-0.87	-0.66	5.22E-05	2.48E-03
AY134745	SMAD2	chr18:43614245-43614186	A_32_P12580	-0.87	-0.65	1.06E-07	4.08E-05
NM_005487	HMG2L1	chr22:34015938-34015997	A_23_P431981	-0.87	-0.63	1.61E-12	2.41E-08
THC2339389	THC2339389	chr3:53825581-53825522	A_32_P73507	-0.87	-0.62	1.75E-06	6.35E-04
THC2283727	THC2283727	chr2:39618542-39618483	A_32_P117730	-0.87	-0.62	1.33E-04	7.78E-03
BF326020	BF326020	chr4:099721903-099721962	A_32_P85813	-0.87	-0.61	1.23E-03	3.02E-02
D14041	RBPSUH	chr4:026112459-026112519	A_23_P113245	-0.87	-0.60	2.01E-05	3.10E-03
CR599788	CR599788	chr17:76984311-76984252	A_32_P195255	-0.86	-1.36	5.48E-07	1.35E-12
AF168717	DTWD1	chr15:47714239-47722833	A_23_P3215	-0.86	-1.16	5.67E-05	2.23E-07
NM_138711	PPARG	chr3:12433499-12433558	A_23_P252062	-0.86	-1.16	6.42E-03	3.57E-04
NM_007047	BTN3A2	chr6:26481619-26482988	A_23_P391264	-0.86	-1.15	1.57E-02	1.70E-03
NM_015960	CUTC	chr10:101493020-101493079	A_23_P98015	-0.86	-1.14	3.39E-04	3.86E-06
NM_003810	TNFSF10	chr3:173706627-173706568	A_23_P121253	-0.86	-1.12	4.23E-02	1.12E-02
NM_003810	TNFSF10	chr3:173706627-173706568	A_23_P121253	-0.86	-1.07	4.19E-02	1.53E-02
NM_145306	C10orf35	chr10:71063197-71063256	A_23_P369328	-0.86	-1.00	4.01E-06	1.68E-07
NM_002764	PRPS1	chrX:106700002-106700061	A_23_P95764	-0.86	-0.98	4.89E-02	3.26E-02
NM_052965	C1orf19	chr1:180774547-180774606	A_23_P104025	-0.86	-0.97	2.25E-06	1.64E-07
NM_032836	FLJ14768	chr19:60794931-60794872	A_23_P107855	-0.86	-0.95	2.97E-06	4.18E-07
NM_001005340	GPNMB	chr7:23087736-23087795	A_23_P134426	-0.86	-0.89	1.91E-03	1.69E-03

Gene	Symbol	Chromosomal position	Probe	siCASP8AP2.3	CASP8AP2.6	siCASP8AP2.3	CASP8AP2.6
				M	M	Q	Q
A_32_P115043	A_32_P115043	chr7:104764845-104764786	A_32_P115043	-0.86	-0.88	4.08E-03	4.26E-03
THC2371272	THC2371272	chr17:25878850-25878909	A_32_P217330	-0.86	-0.86	1.48E-07	1.75E-07
THC2337923	THC2337923	chr8:144433847-144433788	A_24_P801825	-0.86	-0.86	1.88E-07	2.00E-07
NM_017906	PAK1IP1	chr6:10817501-10817560	A_23_P122674	-0.86	-0.85	1.23E-03	1.85E-03
NM_032211	LOXL4	chr10:100000817-99998685	A_23_P12755	-0.86	-0.81	1.16E-02	2.52E-02
NM_012415	RAD54B	chr8:95453591-95453532	A_23_P82738	-0.86	-0.79	8.74E-05	3.59E-04
NM_024782	NHEJ1	chr2:219766553-219766494	A_24_P251381	-0.86	-0.76	6.95E-03	2.38E-02
NM_032188	MYST1	chr16:31046907-31048843	A_23_P100478	-0.86	-0.75	8.31E-05	7.21E-04
A_32_P80523	A_32_P80523	chr10:101187783-101187842	A_32_P80523	-0.86	-0.73	2.28E-05	3.75E-04
BC029907	BC029907	chr1:93509953-93509894	A_24_P886336	-0.86	-0.70	5.16E-04	6.50E-03
NM_207352	CYP4V2	chr4:187509588-187509647	A_32_P23838	-0.86	-0.69	2.00E-08	3.26E-06
NM_006512	SAA4	chr11:18210619-18210560	A_23_P87238	-0.86	-0.68	5.51E-03	4.05E-02
NM_018464	ZCD1	chr10:59717445-59717504	A_23_P35467	-0.86	-0.67	2.03E-03	2.41E-02
NM_139076	CCDC98	chr4:84739552-84739493	A_23_P253464	-0.86	-0.66	6.34E-06	5.26E-04
NM_014639	KIAA0372	chr5:94886911-94886046	A_24_P93353	-0.86	-0.66	4.54E-04	9.33E-03
NM_024675	FLJ21816	chr16:23532896-23532837	A_23_P129569	-0.86	-0.63	4.03E-07	1.67E-04
AK091569	AK091569	chr14:62908163-62908104	A_24_P5026	-0.86	-0.63	4.99E-05	3.32E-03
NM_018048	FLJ10292	chr12:10648635-10648576	A_24_P330112	-0.86	-0.63	7.20E-05	4.00E-03
AK095151	AK095151	chr8:103334376-103334435	A_24_P450172	-0.86	-0.61	5.56E-09	1.31E-05
NM_138787	C11orf74	chr11:36637170-36637229	A_24_P252846	-0.85	-1.16	2.40E-03	4.82E-05
AF168717	DTWD1	chr15:47714239-47722833	A_23_P3215	-0.85	-1.12	6.38E-04	1.15E-05
NM_015185	ARHGEF9	chrX:62638418-62638359	A_24_P254551	-0.85	-1.10	1.11E-04	1.24E-06
NM_014396	VPS41	chr7:38537072-38537013	A_23_P215318	-0.85	-1.07	4.36E-06	2.89E-08
BC067871	LOC150383	chr22:44960491-44960432	A_24_P478726	-0.85	-1.03	2.05E-06	2.84E-08
NM_001008397	LOC493869	chr5:54496011-54496070	A_23_P122052	-0.85	-1.00	1.25E-03	2.17E-04
NM_005829	AP3S2	chr15:88178603-88178544	A_24_P287683	-0.85	-0.98	7.03E-06	4.24E-07
AB032983	PPM1H	chr12:61324847-61324788	A_23_P391443	-0.85	-0.89	5.65E-07	1.83E-07
NM_014234	HSD17B8	chr6:33281471-33281632	A_23_P81973	-0.85	-0.89	1.42E-04	8.16E-05
NM_001003408	ABLIM1	chr10:116180940-116180881	A_23_P202520	-0.85	-0.83	4.25E-03	7.71E-03
NM_016835	MAPT	chr17:41458383-41458442	A_23_P207699	-0.85	-0.81	7.68E-07	2.43E-06
NM_005481	THRAP5	chr19:819484-819425	A_23_P56170	-0.85	-0.79	1.16E-04	3.86E-04
NM_002346	LY6E	chr8:144174191-144174382	A_24_P317762	-0.85	-0.77	7.62E-08	6.67E-07
AK055252	AK055252	chr15:88241352-88241293	A_24_P196384	-0.85	-0.76	3.86E-06	3.36E-05
NM_016195	MPHOSPH1	chr10:91524035-91524094	A_23_P75071	-0.85	-0.72	1.08E-04	1.20E-03
BC009735	BC009735	chr18:32020811-32020794	A_32_P50187	-0.85	-0.70	1.71E-07	1.08E-05
AL832683	PTAR1	chr9:69554131-69554072	A_24_P75158	-0.85	-0.69	1.89E-04	3.33E-03
NM_002746	MAPK3	chr16:30035797-30035738	A_23_P37910	-0.85	-0.64	2.38E-04	7.48E-03
NM_004523	KIF11	chr10:94404607-94404666	A_24_P227091	-0.85	-0.62	2.06E-06	4.73E-04
NM_016835	MAPT	chr17:41458383-41458442	A_23_P207699	-0.85	-0.62	2.66E-06	4.97E-04
NM_006047	RBM12	chr20:33700462-33700403	A_23_P502832	-0.85	-0.61	1.20E-05	1.71E-03
THC2305027	THC2305027	chr5:36907875-36907816	A_32_P78131	-0.85	-0.61	1.13E-04	7.13E-03
NM_002709	PPP1CB	chr2:28937334-28937393	A_24_P396720	-0.85	-0.60	1.31E-04	8.60E-03
NM_021100	NFS1	chr20:33720520-33720461	A_23_P102842	-0.84	-1.36	7.47E-04	2.18E-07
NM_138711	PPARG	chr3:12433499-12433558	A_23_P252062	-0.84	-1.24	7.71E-03	1.29E-04
AF168717	DTWD1	chr15:47714239-47722833	A_23_P3215	-0.84	-1.17	1.81E-04	6.20E-07
NM_012105	BACE2	chr21:41569566-41569625	A_24_P14584	-0.84	-1.05	4.79E-05	7.83E-07
NM_138711	PPARG	chr3:12433499-12433558	A_23_P252062	-0.84	-0.98	4.93E-03	1.52E-03
NM_012415	RAD54B	chr8:95453591-95453532	A_23_P82738	-0.84	-0.97	1.43E-04	1.76E-05
NM_033407	DOCK7	chr1:62651737-62651678	A_32_P230547	-0.84	-0.96	2.98E-03	9.84E-04
BC057844	BC057844	chr1:52546637-52546696	A_24_P862207	-0.84	-0.89	3.58E-03	2.78E-03
NM_014673	KIAA0103	chr8:109567992-109568051	A_23_P60002	-0.84	-0.86	3.68E-05	2.76E-05
ENST00000372288	ENST00000372288	chr10:81774553-81774494	A_24_P281395	-0.84	-0.85	2.97E-03	3.53E-03
NM_003908	EIF2S2	chr20:32140368-32140309	A_24_P48791	-0.84	-0.84	6.61E-06	7.99E-06
ENST00000380021	ENST00000380021	chr5:94825599-94825540	A_23_P61854	-0.84	-0.83	2.96E-02	4.52E-02
NM_006390	IPO8	chr12:30678322-30676192	A_23_P98923	-0.84	-0.82	1.01E-04	1.86E-04
NM_022489	C14orf173	chr14:104256557-104256634	A_23_P394836	-0.84	-0.80	2.04E-05	5.56E-05

Gene	Symbol	Chromosomal position	Probe	siCASP8AP2.3	CASP8AP2.6	siCASP8AP2.3	CASP8AP2.6
				M	M	Q	Q
NM_032842	FLJ14803	chr7:129398986-129398927	A_23_P122805	-0.84	-0.78	6.74E-08	5.08E-07
NM_001813	CENPE	chr4:104385002-104384943	A_23_P253524	-0.84	-0.73	1.72E-04	1.28E-03
NM_024054	C7orf25	chr7:42723050-42722991	A_24_P300483	-0.84	-0.73	8.02E-04	4.80E-03
NM_018137	PRMT6	chr1:107313464-107313523	A_23_P12336	-0.84	-0.71	2.12E-03	1.21E-02
BC013250	BC013250	chr5:115657420-115657479	A_24_P20767	-0.84	-0.69	1.80E-06	7.11E-05
A_24_P853004	A_24_P853004	chr5:075253095-075253036	A_24_P853004	-0.84	-0.65	8.35E-04	1.24E-02
CA313037	CA313037	chr20:34282251-34282192	A_32_P57775	-0.84	-0.65	2.75E-03	2.84E-02
A_24_P534290	A_24_P534290	chr11:119197723-119197538	A_24_P534290	-0.84	-0.64	1.08E-05	7.89E-04
NM_080663	EXOD1	chr16:20699330-20699271	A_23_P129717	-0.84	-0.62	2.87E-06	4.52E-04
CR592968	RSBN1L	chr7:76971242-76971301	A_24_P925292	-0.84	-0.62	6.85E-04	1.62E-02
NM_005170	ASCL2	chr11:2246594-2246535	A_32_P171061	-0.83	-1.24	1.77E-02	5.34E-04
NM_144706	C2orf15	chr2:99225987-99226046	A_23_P415511	-0.83	-1.20	1.48E-07	3.82E-12
ENST00000379534	ENST00000379534	chr13:40284089-40284148	A_23_P128650	-0.83	-1.02	5.13E-05	1.39E-06
NM_145204	SENP8	chr15:70219718-70219777	A_23_P334263	-0.83	-0.89	3.47E-05	1.14E-05
NM_181503	EXOSC8	chr13:36481331-36481390	A_24_P241276	-0.83	-0.80	1.22E-07	2.83E-07
THC2268988	THC2268988	chr3:28337755-28337696	A_24_P105913	-0.83	-0.78	7.88E-07	3.50E-06
NM_006745	SC4MOL	chr4:166620795-166620854	A_23_P110184	-0.83	-0.76	1.65E-02	3.83E-02
NM_019049	FLJ20054	chr1:194211044-194210985	A_23_P320897	-0.83	-0.74	1.16E-04	7.50E-04
NM_020918	GPAM	chr10:113899876-113899817	A_24_P227069	-0.83	-0.71	4.34E-06	6.85E-05
NM_001012409	SGOL1	chr3:20187678-20187619	A_24_P225970	-0.83	-0.70	2.16E-04	2.12E-03
A_32_P108748	A_32_P108748	chr4:015359368-015359309	A_32_P108748	-0.83	-0.69	1.49E-04	1.77E-03
NM_170740	ALDH5A1	chr6:24643596-24643655	A_24_P923353	-0.83	-0.69	1.97E-03	1.43E-02
NM_022106	C20orf177	chr20:57955262-57955321	A_24_P6428	-0.83	-0.68	1.07E-05	2.83E-04
AK021820	GOLGA1	chr9:124786925-124786866	A_24_P481431	-0.83	-0.68	3.89E-05	8.09E-04
NM_005185	CALML3	chr10:5557079-5557138	A_23_P393080	-0.83	-0.68	4.75E-03	3.08E-02
AL133055	DKFZp434J1015	chr7:6436220-6436279	A_23_P302207	-0.83	-0.67	4.95E-03	3.33E-02
NM_016361	ACP6	chr1:144352172-144352113	A_23_P160240	-0.83	-0.66	6.17E-05	1.77E-03
NM_173084	TRIM59	chr3:161638575-161638516	A_23_P407718	-0.83	-0.62	3.01E-07	9.02E-05
NM_181806	AASDH	chr4:57056447-57056388	A_24_P383076	-0.83	-0.62	6.68E-07	1.59E-04
NM_016835	MAPT	chr17:41458383-41458442	A_23_P207699	-0.83	-0.62	1.89E-03	2.88E-02
NM_012415	RAD54B	chr8:95453591-95453532	A_23_P82738	-0.83	-0.61	1.46E-04	7.22E-03
NM_003662	PIR	chrX:15162774-15162715	A_23_P137035	-0.82	-1.58	3.56E-05	4.21E-12
NM_175921	LOC285636	chr5:41957186-41957245	A_32_P6832	-0.82	-1.31	2.48E-06	1.44E-11
NM_006306	SMC1A	chrX:53284233-53284174	A_24_P942604	-0.82	-1.19	1.57E-04	1.54E-07
ENST00000379156	ENST00000379156	chr2:37239294-37239235	A_32_P38003	-0.82	-0.99	1.33E-07	1.06E-09
NM_001033523	GUSBL1	chr6:26964934-26964875	A_24_P84822	-0.82	-0.99	1.62E-02	4.92E-03
NM_001008397	LOC493869	chr5:54496011-54496070	A_23_P122052	-0.82	-0.92	1.51E-03	5.18E-04
AK021933	AK021933	chr7_random:192516-192457	A_23_P31550	-0.82	-0.91	3.63E-04	9.94E-05
NM_017996	DET1	chr15:86856861-86856802	A_23_P26184	-0.82	-0.87	4.33E-06	1.45E-06
NM_022786	ARV1	chr1:227442779-227442838	A_23_P135995	-0.82	-0.85	2.07E-02	2.41E-02
NM_004990	MARS	chr12:56196640-56196699	A_24_P175909	-0.82	-0.84	3.73E-02	4.47E-02
NM_001813	CENPE	chr4:104385002-104384943	A_23_P253524	-0.82	-0.83	1.01E-04	1.14E-04
NM_015975	TAF9B	chrX_random:1385766-1389379	A_23_P254790	-0.82	-0.82	8.43E-03	1.23E-02
BC066984	BC066984	chr15:64569206-64569147	A_24_P287613	-0.82	-0.80	4.55E-05	7.21E-05
NM_004569	PIGH	chr14:67126411-67126352	A_32_P71736	-0.82	-0.78	6.95E-03	1.55E-02
BX095281	BX095281	chr3:158362607-158362666	A_32_P179351	-0.82	-0.77	7.13E-04	1.96E-03
NM_017906	PAK1IP1	chr6:10817501-10817560	A_23_P122674	-0.82	-0.77	2.04E-03	5.15E-03
NM_015341	BRRN1	chr2:96461200-96461259	A_23_P415443	-0.82	-0.76	5.70E-06	2.78E-05
NM_153034	ZNF488	chr10:47993511-47993570	A_23_P23966	-0.82	-0.67	9.89E-04	8.68E-03
ENST00000369572	ENST00000369572	chr6:88166121-88166180	A_23_P418431	-0.82	-0.64	3.02E-04	6.58E-03
ENST00000330640	ENST00000330640	chr6:138707306-138707365	A_32_P65473	-0.82	-0.63	3.43E-04	7.10E-03
NM_020451	SEPN1	chr1:25827767-25827826	A_24_P231250	-0.82	-0.62	1.08E-04	4.04E-03
AK096548	CEP192	chr18:12989503-12989562	A_32_P217870	-0.82	-0.60	1.96E-04	8.82E-03
AK000352	MKS1	chr17:53638205-53638147	A_23_P129778	-0.82	-0.60	2.96E-04	1.07E-02
NM_145261	DNAJC19	chr3:182186451-182185177	A_23_P431890	-0.81	-1.53	7.43E-04	4.23E-09
NM_012139	SERGEF	chr11:17766286-17766227	A_24_P364087	-0.81	-1.34	1.85E-02	1.51E-04

Gene	Symbol	Chromosomal position	Probe	siCASP8AP2.3	CASP8AP2.6	siCASP8AP2.3	CASP8AP2.6
				M	M	Q	Q
NM_017570	OPLAH	chr8:145178249-145178190	A_23_P170186	-0.81	-0.99	7.43E-04	6.01E-05
NM_032814	TMEM118	chr12:115753376-115753435	A_24_P13390	-0.81	-0.96	4.70E-04	4.96E-05
NM_003707	RUVBL1	chr3:129302213-129302154	A_24_P148811	-0.81	-0.92	2.64E-03	8.00E-04
AK092163	AK092163	chr3:129266570-129266511	A_32_P122891	-0.81	-0.86	5.51E-06	1.67E-06
NM_018178	GOLPH3L	chr1:147433636-147433577	A_23_P200890	-0.81	-0.85	4.90E-07	2.11E-07
AF085351	AF085351	chr2:36980152-36980093	A_32_P174398	-0.81	-0.84	7.66E-05	4.82E-05
NM_017906	PAK1IP1	chr6:10817501-10817560	A_23_P122674	-0.81	-0.80	2.05E-03	3.24E-03
NM_003622	PPFIBP1	chr12:27737226-27737285	A_23_P47867	-0.81	-0.79	3.46E-04	5.95E-04
THC2312785	THC2312785	chr2:36977709-36977650	A_32_P78904	-0.81	-0.79	2.22E-03	3.71E-03
NM_016835	MAPT	chr17:41458383-41458442	A_23_P207699	-0.81	-0.74	2.22E-05	1.24E-04
NM_002692	POLE2	chr14:49192220-49192161	A_23_P163099	-0.81	-0.72	1.23E-02	3.62E-02
NM_014450	SIT1	chr9:35639748-35639689	A_23_P43369	-0.81	-0.68	4.80E-03	2.46E-02
NM_206836	PECI	chr6:4061181-4061122	A_23_P156852	-0.81	-0.65	1.34E-05	4.89E-04
NM_015336	ZDHHC17	chr12:75749622-75749681	A_23_P314191	-0.81	-0.64	6.71E-07	7.15E-05
NM_020843	ZNF291	chr15:74427964-74427905	A_23_P163408	-0.81	-0.64	3.19E-06	2.11E-04
NM_016835	MAPT	chr17:41458383-41458442	A_23_P207699	-0.81	-0.64	8.55E-06	4.28E-04
NM_183009	KIAA1429	chr8:95593116-95593057	A_23_P215980	-0.81	-0.62	4.02E-04	9.83E-03
NM_133375	MGC4562	chr15:64412633-64412692	A_23_P65741	-0.81	-0.62	9.91E-04	1.66E-02
BC107586	METTL2B	chr7:127737621-127737680	A_24_P710171	-0.81	-0.60	1.70E-05	1.30E-03
AF168717	DTWD1	chr15:47714239-47722833	A_23_P3215	-0.80	-1.19	5.14E-04	9.22E-07
NM_001008397	LOC493869	chr5:54496011-54496070	A_23_P122052	-0.80	-1.19	5.40E-03	6.85E-05
NM_018245	OGDHL	chr10:50612863-50612804	A_23_P161297	-0.80	-1.18	1.75E-04	1.35E-07
NM_174893	C17orf49	chr17:6860955-6861014	A_23_P141520	-0.80	-1.13	6.72E-05	8.88E-08
NM_032813	TMTC4	chr13:100054472-100054413	A_23_P65230	-0.80	-1.11	5.34E-04	3.25E-06
AF168717	DTWD1	chr15:47714239-47722833	A_23_P3215	-0.80	-1.11	1.19E-03	1.22E-05
NM_018141	MRPS10	chr6:42282650-42282591	A_24_P209285	-0.80	-1.03	5.39E-11	7.69E-15
NM_138711	PPARG	chr3:12433499-12433558	A_23_P252062	-0.80	-1.01	7.63E-03	1.08E-03
NM_017906	PAK1IP1	chr6:10817501-10817560	A_23_P122674	-0.80	-0.98	1.32E-03	1.26E-04
THC2352042	THC2352042	chr2:223633108-223633163	A_23_P108394	-0.80	-0.90	6.72E-05	9.25E-06
NM_152658	THAP8	chr19:41218197-41218138	A_24_P296907	-0.80	-0.85	2.06E-04	1.22E-04
NM_017906	PAK1IP1	chr6:10817501-10817560	A_23_P122674	-0.80	-0.85	1.07E-03	6.91E-04
NM_016373	WWOX	chr16:77016423-77023914	A_23_P71972	-0.80	-0.81	4.22E-05	4.39E-05
NM_032316	NICN1	chr3:49435653-49435594	A_23_P110005	-0.80	-0.80	2.42E-04	2.85E-04
AK000053	AK000053	chr1:109188228-109188287	A_24_P658505	-0.80	-0.79	4.07E-05	5.46E-05
NM_004412	DNMT2	chr10:17236722-17235623	A_23_P115636	-0.80	-0.78	8.16E-04	1.32E-03
NM_017906	PAK1IP1	chr6:10817501-10817560	A_23_P122674	-0.80	-0.78	3.52E-03	6.58E-03
NM_025222	TMEM113	chr3:52265440-52265381	A_24_P388536	-0.80	-0.75	8.10E-04	2.10E-03
NM_020244	CHPT1	chr12:100612989-100613048	A_23_P105571	-0.80	-0.72	3.91E-03	1.28E-02
NM_016426	GTSE1	chr22:45045910-45045969	A_23_P57588	-0.80	-0.71	2.03E-03	8.55E-03
NM_002746	MAPK3	chr16:30035797-30035738	A_23_P37910	-0.80	-0.69	1.25E-04	1.09E-03
NM_024105	ALG12	chr22:48618335-48618276	A_23_P60718	-0.80	-0.66	7.99E-05	1.28E-03
NM_006677	USP19	chr3:49123216-49123157	A_23_P113789	-0.80	-0.65	4.08E-05	9.70E-04
AF088004	AF088004	chr3:53822711-53822652	A_24_P880000	-0.80	-0.64	4.71E-04	6.86E-03
NM_014480	ZNF544	chr19:63449583-63449925	A_24_P324640	-0.80	-0.63	1.38E-03	1.67E-02
BC044608	BC044608	chr7:63977912-63977853	A_24_P816664	-0.80	-0.60	4.00E-06	4.73E-04
BC039397	BC039397	chr15:69902172-69902113	A_32_P123168	-0.80	-0.60	5.26E-06	5.95E-04
BX647075	BX647075	chr6:100086347-100086406	A_32_P186138	-0.79	-1.13	2.21E-04	4.58E-07
NM_001008397	LOC493869	chr5:54496011-54496070	A_23_P122052	-0.79	-1.12	6.06E-03	1.76E-04
NM_014425	INVS	chr9:100142453-100142512	A_23_P157970	-0.79	-1.07	1.58E-06	1.06E-09
NM_017769	KIAA1333	chr14:30154437-30154496	A_23_P99604	-0.79	-1.05	1.58E-03	4.02E-05
AK097639	RP11-561O23.4	chr9:67970109-67970050	A_24_P145035	-0.79	-1.05	8.40E-03	6.42E-04
NM_001008397	LOC493869	chr5:54496011-54496070	A_23_P122052	-0.79	-0.98	1.34E-03	1.03E-04
NM_032565	EBPL	chr13:49135249-49133343	A_24_P84279	-0.79	-0.92	6.00E-06	2.78E-07
A_24_P264004	A_24_P264004	chr4:165211878-165211941	A_24_P264004	-0.79	-0.91	2.28E-02	1.21E-02
NM_152667	NANP	chr20:25543176-25543117	A_23_P427039	-0.79	-0.85	1.97E-03	1.10E-03
NM_022782	MPHOSPH9	chr12:122173452-122173393	A_23_P13657	-0.79	-0.80	1.34E-08	1.00E-08

Gene	Symbol	Chromosomal position	Probe	siCASP8AP2.3	CASP8AP2.6	siCASP8AP2.3	CASP8AP2.6
				M	M	Q	Q
NM_024808	FLJ22624	chr13:72227357-72227416	A_23_P25626	-0.79	-0.78	2.00E-06	2.84E-06
ENST00000371291	ENST00000371291	chr20:55177264-55177211	A_23_P154643	-0.79	-0.77	1.17E-03	2.10E-03
NM_004688	NMI	chr2:151952577-151952518	A_23_P154235	-0.79	-0.76	2.08E-02	3.68E-02
BQ354462	BQ354462	chr7:026920559-026920618	A_32_P198179	-0.79	-0.75	1.91E-05	4.84E-05
NM_019120	PCDHB8	chr5:140540034-140540093	A_23_P41599	-0.79	-0.74	4.26E-05	1.50E-04
NM_016581	SITPEC	chr19:11477951-11477892	A_23_P119295	-0.79	-0.74	2.84E-03	6.50E-03
THC2344914	THC2344914	chr7:27005806-27005748	A_32_P234853	-0.79	-0.73	3.25E-04	1.16E-03
NM_006512	SAA4	chr11:18210619-18210560	A_23_P87238	-0.79	-0.71	1.06E-02	3.20E-02
NM_006390	IPO8	chr12:30678322-30676192	A_23_P98923	-0.79	-0.69	4.11E-05	4.04E-04
NM_015266	SLC9A8	chr20:47941781-47941840	A_24_P58054	-0.79	-0.68	2.48E-06	4.65E-05
BM476468	BM476468	chr20:030909905-030909846	A_32_P128097	-0.79	-0.68	1.37E-04	1.28E-03
AK126014	KIAA1211	chr4:57031319-57034755	A_24_P222516	-0.79	-0.67	1.37E-04	1.61E-03
NM_001931	DLAT	chr11:111439649-111439708	A_23_P203030	-0.79	-0.67	9.30E-04	6.96E-03
THC2277620	THC2277620	chr3:101556930-101556989	A_24_P414282	-0.79	-0.66	9.54E-08	5.26E-06
NM_002746	MAPK3	chr16:30035797-30035738	A_23_P37910	-0.79	-0.65	4.85E-04	5.22E-03
NM_020367	PARP11	chr12:3789056-3788997	A_24_P59494	-0.79	-0.64	1.13E-04	2.19E-03
NM_014959	CARD8	chr19:53403392-53403333	A_24_P14260	-0.79	-0.62	4.01E-04	7.09E-03
NM_138578	BCL2L1	chr20:29717044-29716985	A_23_P210886	-0.79	-0.62	5.11E-03	3.81E-02
AK002019	SMURF2	chr17:59970481-59970422	A_24_P210888	-0.79	-0.60	4.50E-03	4.55E-02
A_32_P171232	A_32_P171232	chr17:053267210-053267151	A_32_P171232	-0.78	-1.07	3.78E-04	2.69E-06
NM_172250	MMAA	chr4:146932866-146933928	A_23_P144357	-0.78	-0.98	2.39E-04	6.93E-06
NM_023933	C16orf24	chr16:712389-712448	A_23_P54728	-0.78	-0.97	2.38E-03	2.30E-04
NM_018046	AGGF1	chr5:76395294-76395353	A_23_P250554	-0.78	-0.90	1.58E-06	7.74E-08
NM_012208	HARSL	chr5:140058675-140058734	A_23_P41588	-0.78	-0.87	3.11E-08	1.88E-09
A_24_P272735	A_24_P272735	chr19:020052132-020052073	A_24_P272735	-0.78	-0.87	1.86E-02	1.21E-02
BC009627	WIBG	chr12:54594250-54594191	A_24_P917612	-0.78	-0.83	1.21E-04	5.29E-05
NM_004393	DAG1	chr3:49547198-49547257	A_24_P225961	-0.78	-0.83	4.35E-04	2.23E-04
NM_014714	IFT140	chr16:1500670-1500611	A_23_P140725	-0.78	-0.81	1.28E-04	8.88E-05
ENST00000297423	ENST00000297423	chr8:48810646-48810705	A_23_P327013	-0.78	-0.80	2.26E-04	2.28E-04
AK126298	AK126298	chr2:212066080-212066021	A_32_P183765	-0.78	-0.78	1.70E-03	2.14E-03
NM_018292	QRSL1	chr6:107220394-107220453	A_23_P82181	-0.78	-0.77	5.38E-05	8.23E-05
CR602569	CR602569	chr6:169943289-169943348	A_32_P36143	-0.78	-0.77	4.91E-03	8.45E-03
NM_006739	MCM5	chr22:34143861-34144732	A_23_P132277	-0.78	-0.73	5.56E-06	1.93E-05
NM_145309	LRRC51	chr11:71482272-71482331	A_23_P98763	-0.78	-0.73	1.81E-04	5.36E-04
THC2337268	THC2337268	chr7:17604337-17604278	A_24_P200848	-0.78	-0.71	2.39E-03	7.39E-03
NM_016262	TUBE1	chr6:112502698-112502639	A_23_P145053	-0.78	-0.70	8.22E-03	2.69E-02
NM_021074	NDUFV2	chr18:9112594-9112653	A_23_P130418	-0.78	-0.65	7.75E-05	1.08E-03
AL110179	MCFP	chr7:87107648-87107589	A_24_P312164	-0.78	-0.65	3.17E-04	3.66E-03
THC2312955	THC2312955	chr10:89499404-89499345	A_32_P99116	-0.78	-0.60	8.95E-06	6.21E-04
AV739664	AV739664	chr20:48951204-48951263	A_32_P15756	-0.78	-0.60	1.51E-03	2.04E-02
NM_015957	APIP	chr11:34866451-34861604	A_23_P2066	-0.77	-1.22	4.19E-03	1.39E-05
NM_024611	NARG2	chr15:58502950-58502891	A_23_P54389	-0.77	-0.85	3.18E-07	3.31E-08
NM_014748	SNX17	chr2:27510487-27510680	A_23_P28233	-0.77	-0.85	1.89E-06	2.32E-07
NM_004294	MTRF1	chr13:40695445-40689359	A_23_P37005	-0.77	-0.84	3.63E-06	6.91E-07
NM_153345	TMEM139	chr7:142500959-142501018	A_23_P42909	-0.77	-0.84	1.57E-04	4.66E-05
NM_015696	GPX7	chr1:52786472-52786531	A_23_P73972	-0.77	-0.83	6.49E-05	2.23E-05
BC011238	PSD3	chr8:18429392-18429333	A_23_P20392	-0.77	-0.83	1.84E-03	1.03E-03
ENST00000380021	ENST00000380021	chr5:94825599-94825540	A_23_P61854	-0.77	-0.81	4.54E-02	4.84E-02
NM_001950	E2F4	chr16:65789011-65789290	A_23_P152218	-0.77	-0.80	3.41E-03	3.46E-03
NM_173830	C6orf182	chr6:109578097-109581749	A_23_P59358	-0.77	-0.75	4.41E-04	8.92E-04
NM_015696	GPX7	chr1:52786362-52786421	A_24_P418816	-0.77	-0.73	7.50E-04	1.94E-03
AK125393	AK125393	chr15:38773907-38773848	A_32_P30831	-0.77	-0.72	4.03E-03	9.29E-03
NM_018319	TDP1	chr14:89579275-89579334	A_23_P117623	-0.77	-0.71	1.52E-07	9.16E-07
NM_018197	ZFP64	chr20:50201610-50201551	A_23_P501877	-0.77	-0.71	1.26E-06	7.00E-06
CR597270	CR597270	chr4:120576076-120576017	A_23_P388433	-0.77	-0.71	1.06E-02	2.61E-02
A_24_P7510	A_24_P7510	chr8:123851144-123851206	A_24_P7510	-0.77	-0.69	1.72E-03	6.66E-03

Gene	Symbol	Chromosomal position	Probe	siCASP8AP2.3	CASP8AP2.6	siCASP8AP2.3	CASP8AP2.6
				M	M	Q	Q
NM_007048	BTN3A1	chr6:26522722-26522781	A_24_P329065	-0.77	-0.68	6.85E-06	6.54E-05
NM_007169	PEMT	chr17:17356572-17353550	A_23_P163955	-0.77	-0.68	1.10E-03	5.31E-03
AF161353	AF161353	chr9:124810433-124810370	A_24_P358116	-0.77	-0.67	1.94E-06	2.76E-05
NM_078470	COX15	chr10:101462361-101462302	A_24_P115700	-0.77	-0.67	5.60E-03	2.31E-02
BC021296	BC021296	chr17:18543439-18545397	A_24_P8304	-0.77	-0.65	1.22E-04	1.49E-03
NM_139076	CCDC98	chr4:84741125-84741066	A_24_P311577	-0.77	-0.65	3.72E-04	3.24E-03
NM_002793	PSMB1	chr6:170770393-170770334	A_23_P156531	-0.77	-0.62	1.09E-04	1.93E-03
NM_002077	GOLGA1	chr9:124720731-124720672	A_23_P258978	-0.76	-1.12	8.15E-05	3.38E-08
NM_001005912	IHPK2	chr3:48706115-48706056	A_23_P301133	-0.76	-1.12	5.20E-04	8.66E-07
NM_152658	THAP8	chr19:41217843-41217784	A_23_P433820	-0.76	-1.05	4.33E-11	4.76E-16
NM_004688	NMI	chr2:151952577-151952518	A_23_P154235	-0.76	-0.96	3.62E-02	1.09E-02
NM_138711	PPARG	chr3:12433499-12433558	A_23_P252062	-0.76	-0.93	7.54E-03	1.53E-03
NM_173082	SHPRH	chr6:146248233-146248174	A_23_P337790	-0.76	-0.91	4.56E-03	9.01E-04
CR609843	CR609843	chr1:101264563-101264622	A_24_P546003	-0.76	-0.86	2.39E-04	4.74E-05
NR_002776	MCM3APAS	chr21:46495740-46495799	A_23_P256694	-0.76	-0.86	1.21E-02	6.56E-03
NM_006048	UBE4B	chr1:10175341-10175400	A_23_P201279	-0.76	-0.84	4.45E-03	2.12E-03
NM_015061	JMJD2C	chr9:7165334-7165393	A_23_P112201	-0.76	-0.83	2.88E-02	2.27E-02
NM_001007258	SETD4	chr21:36329223-36329164	A_23_P211106	-0.76	-0.78	2.43E-03	2.61E-03
BC063022	BC063022	chr11:86341234-86341293	A_23_P87421	-0.76	-0.77	2.67E-02	3.45E-02
NM_002890	RASA1	chr5:86722521-86722580	A_23_P18939	-0.76	-0.74	1.88E-02	3.24E-02
NM_001005912	IHPK2	chr3:48706299-48706240	A_23_P301138	-0.76	-0.73	4.03E-07	8.29E-07
NM_001008572	TTLL1	chr22:41772389-41772330	A_23_P120970	-0.76	-0.72	7.86E-04	1.79E-03
NM_018205	LRRC20	chr10:71728838-71728779	A_23_P61487	-0.76	-0.72	8.29E-04	1.92E-03
NM_016835	MAPT	chr17:41458383-41458442	A_23_P207699	-0.76	-0.68	8.35E-04	3.58E-03
NM_003002	SDHD	chr11:111470917-111470976	A_23_P138967	-0.76	-0.68	8.92E-03	2.80E-02
NM_001017922	ERMAP	chr1:42979127-42979186	A_24_P105391	-0.76	-0.66	5.01E-05	4.95E-04
NM_152905	NEDD1	chr12:95847706-95848215	A_24_P198355	-0.76	-0.66	1.05E-03	5.84E-03
ENST00000222690	ENST00000222690	chr7:44640772-44640713	A_23_P316487	-0.76	-0.66	6.07E-03	2.49E-02
BC042034	MGC72075	chr7:23504420-23513668	A_32_P59678	-0.76	-0.65	3.52E-05	4.49E-04
NM_006388	HTATIP	chr11:65242938-65242997	A_23_P138849	-0.76	-0.63	1.68E-05	3.83E-04
NM_001003679	LEPR	chr1:65812670-65812729	A_24_P231104	-0.76	-0.61	5.03E-07	4.24E-05
NM_013444	UBQLN2	chrX:56476074-56476133	A_23_P114164	-0.76	-0.61	1.31E-04	2.68E-03
NM_024744	ALS2CR8	chr2:203673874-203673933	A_23_P165828	-0.76	-0.60	3.67E-07	4.52E-05
NM_152551	C6orf151	chr6:7556181-7556240	A_24_P46725	-0.76	-0.60	1.19E-04	2.91E-03
NM_004899	BRE	chr2:28432956-28461797	A_32_P132796	-0.75	-1.18	2.90E-06	2.66E-11
NM_004453	ETFDH	chr4:159987250-159987309	A_23_P61447	-0.75	-1.08	6.86E-03	1.50E-04
NM_015412	C3orf17	chr3:114204419-114204360	A_24_P921155	-0.75	-1.06	3.17E-05	2.41E-08
NM_138711	PPARG	chr3:12433499-12433558	A_23_P252062	-0.75	-1.03	1.60E-02	1.25E-03
NM_152387	KCTD18	chr2:201180035-201179976	A_23_P309515	-0.75	-0.95	3.71E-05	3.85E-07
NM_004688	NMI	chr2:151952577-151952518	A_23_P154235	-0.75	-0.95	3.90E-02	1.11E-02
NM_152511	DUSP18	chr22:29382655-29382596	A_24_P83118	-0.75	-0.94	1.85E-05	1.90E-07
XM_928889	LOC645919	chr16:76333422-76333481	A_23_P3602	-0.75	-0.90	8.01E-04	9.02E-05
NM_144598	LRRC28	chr15:97719229-97720882	A_24_P142151	-0.75	-0.90	1.60E-02	5.54E-03
NM_018135	MRPS18A	chr6:43747529-43747470	A_23_P122387	-0.75	-0.89	1.24E-08	9.83E-11
THC2368606	THC2368606	chr5:132009420-132009479	A_24_P930707	-0.75	-0.82	6.40E-04	2.89E-04
NM_014702	KIAA0408	chr6:127806692-127806633	A_23_P215048	-0.75	-0.82	1.95E-02	1.51E-02
A_24_P143785	A_24_P143785	chrX:140083711-140083650	A_24_P143785	-0.75	-0.81	1.83E-03	9.21E-04
THC2404072	THC2404072	chr4:159989035-159989094	A_32_P120484	-0.75	-0.77	3.97E-04	3.98E-04
NM_018999	KIAA1128	chr10:86267792-86267851	A_23_P404108	-0.75	-0.74	1.01E-04	1.51E-04
NM_004275	TRFP	chr6:41981344-41981285	A_24_P273865	-0.75	-0.70	3.26E-07	1.55E-06
THC2376027	THC2376027	chr2:219761051-219760992	A_24_P485742	-0.75	-0.70	4.67E-04	1.42E-03
ENST00000367003	ENST00000367003	chr1:207994135-207994194	A_23_P1014	-0.75	-0.69	4.45E-05	1.78E-04
A_24_P358474	A_24_P358474	chr2:203531719-203531660	A_24_P358474	-0.75	-0.68	2.27E-03	7.48E-03
NM_012415	RAD54B	chr8:95453591-95453532	A_23_P82738	-0.75	-0.65	7.97E-04	5.20E-03
BC014534	MBD5	chr2:149061985-149062044	A_23_P142537	-0.75	-0.64	1.13E-03	8.09E-03
NM_012176	FBXO4	chr5:41963019-41963078	A_23_P58815	-0.75	-0.64	1.45E-03	8.81E-03

Gene	Symbol	Chromosomal position	Probe	siCASP8AP2.3	CASP8AP2.6	siCASP8AP2.3	CASP8AP2.6
				M	M	Q	Q
NM_016835	MAPT	chr17:41458383-41458442	A_23_P207699	-0.75	-0.63	1.16E-04	1.40E-03
NM_016835	MAPT	chr17:41458383-41458442	A_23_P207699	-0.75	-0.62	1.92E-05	4.12E-04
NM_007350	PHLDA1	chr12:74705953-74705894	A_24_P915692	-0.75	-0.62	4.16E-04	4.78E-03
AL133090	AL133090	chr7:87476754-87476813	A_24_P355006	-0.75	-0.62	2.01E-03	1.48E-02
NM_017817	RAB20	chr13:109974079-109974020	A_24_P22050	-0.75	-0.62	6.98E-03	3.68E-02
NM_030800	C15orf44	chr15:63658429-63658370	A_23_P37475	-0.75	-0.61	2.66E-03	2.00E-02
NM_016485	C6orf55	chr6:142583095-142583154	A_24_P294982	-0.75	-0.60	2.63E-03	2.33E-02
A_24_P135771	A_24_P135771	chr12:118860195-118860254	A_24_P135771	-0.74	-1.52	1.97E-02	3.66E-06
NM_018383	WDR33	chr2:128180124-128180065	A_23_P414420	-0.74	-1.29	7.06E-03	5.72E-06
NM_001008397	LOC493869	chr5:54496011-54496070	A_23_P122052	-0.74	-1.05	9.58E-03	3.92E-04
NM_002495	NDUFS4	chr5:52977986-52990190	A_23_P257198	-0.74	-1.02	1.87E-05	2.22E-08
NM_001002031	ATP5G2	chr12:52345403-52345344	A_23_P87616	-0.74	-1.02	3.05E-05	3.83E-08
AK124192	AK124192	chr2:10028156-10028215	A_32_P154731	-0.74	-0.99	3.61E-05	1.14E-07
ENST00000374285	ENST00000374285	chr1:25971075-25971016	A_24_P71153	-0.74	-0.98	2.06E-07	1.25E-10
NM_002193	INHBB	chr2:120825010-120825069	A_23_P153964	-0.74	-0.97	1.24E-02	1.43E-03
NM_032970	SEC22C	chr3:42574118-42572477	A_23_P502641	-0.74	-0.95	1.49E-03	6.93E-05
NM_001698	AUH	chr9:91056083-91056024	A_23_P20852	-0.74	-0.94	8.73E-08	1.25E-10
NM_022344	C17orf75	chr17:27682959-27682900	A_24_P224526	-0.74	-0.94	2.02E-04	3.87E-06
NM_006452	PAICS	chr4:57168163-57168222	A_24_P200427	-0.74	-0.94	2.81E-02	7.48E-03
NM_003731	SSNA1	chr9:137360121-137360180	A_24_P303915	-0.74	-0.90	4.17E-04	2.97E-05
AB058761	ZNF469	chr16:87034588-87034647	A_32_P116556	-0.74	-0.89	2.16E-02	8.17E-03
NM_020679	MIF4GD	chr17:70775801-70775561	A_24_P382026	-0.74	-0.87	2.03E-03	3.96E-04
NM_080651	THRAP6	chr8:118621326-118621385	A_23_P31866	-0.74	-0.87	1.39E-02	5.10E-03
NM_016481	C9orf156	chr9:97755243-97752400	A_24_P212860	-0.74	-0.82	2.49E-03	1.07E-03
NM_017906	PAK1IP1	chr6:10817501-10817560	A_23_P122674	-0.74	-0.80	6.00E-03	3.71E-03
NM_022460	HS1BP3	chr2:20740067-20740008	A_23_P165548	-0.74	-0.78	1.24E-02	1.16E-02
NM_018216	PANK4	chr1:2474351-2474292	A_23_P149690	-0.74	-0.74	7.93E-04	1.06E-03
NM_015954	DERA	chr12:16081355-16081414	A_23_P25253	-0.74	-0.71	2.02E-05	4.14E-05
NM_004688	NMI	chr2:151952577-151952518	A_23_P154235	-0.74	-0.71	2.63E-02	4.75E-02
NM_015909	NAG	chr2:15277533-15277474	A_23_P90752	-0.74	-0.68	4.81E-06	2.39E-05
NM_003622	PPFIBP1	chr12:27739620-27739679	A_23_P373724	-0.74	-0.68	1.71E-02	4.17E-02
NM_002841	PTPRG	chr3:62254258-62254317	A_23_P41054	-0.74	-0.66	2.41E-05	1.70E-04
NM_001003684	UCRC	chr22:28490491-28490550	A_24_P66001	-0.74	-0.65	1.79E-03	7.85E-03
NM_003780	B4GALT2	chr1:44120356-44125091	A_24_P242440	-0.74	-0.65	1.50E-02	4.67E-02
NM_004457	ACSL3	chr2:223633000-223633059	A_24_P248606	-0.74	-0.64	8.47E-05	6.91E-04
NM_020424	LOC57149	chr16:20843068-20843127	A_23_P54929	-0.74	-0.62	1.66E-03	1.18E-02
NM_006837	COPS5	chr8:68126049-68120721	A_23_P71419	-0.74	-0.61	5.05E-04	5.15E-03
NM_015323	KIAA0776	chr6:97109681-97109740	A_23_P30956	-0.74	-0.60	2.05E-03	1.61E-02
NM_024728	C7orf10	chr7:40087476-40129638	A_23_P145711	-0.74	-0.60	5.38E-03	3.50E-02
NM_001005291	SREBF1	chr17:17656203-17656144	A_23_P129786	-0.73	-1.68	3.24E-02	2.05E-06
NM_001008397	LOC493869	chr5:54496011-54496070	A_23_P122052	-0.73	-1.10	6.79E-03	6.86E-05
NM_181708	LOC144233	chr12:48518039-48517980	A_23_P338233	-0.73	-0.95	5.77E-05	3.86E-07
NM_003634	NIPSNAP1	chr22:28275836-28275777	A_23_P120860	-0.73	-0.93	2.79E-03	2.08E-04
NM_001001890	RUNX1	chr21:35082320-35082261	A_24_P96403	-0.73	-0.91	9.14E-04	5.07E-05
NM_152463	EME1	chr17:45813626-45813685	A_23_P368225	-0.73	-0.86	2.70E-03	6.26E-04
NM_032547	SCOC	chr4:141660369-141660428	A_24_P46334	-0.73	-0.85	4.17E-03	1.20E-03
THC2275676	THC2275676	chr2:208299457-208299398	A_24_P854913	-0.73	-0.81	3.77E-03	1.95E-03
THC2405842	THC2405842	chr5:72829434-72829375	A_32_P115663	-0.73	-0.80	1.35E-03	6.37E-04
NM_005631	SMO	chr7:128447188-128447247	A_23_P70818	-0.73	-0.78	5.09E-03	3.78E-03
NM_031294	LRRC48	chr17:17860208-17860629	A_24_P270971	-0.73	-0.75	2.04E-06	1.19E-06
AL365520	AL365520	chr4:3542560-3542501	A_32_P460399	-0.73	-0.73	7.11E-06	8.61E-06
NM_000390	CHM	chrX:84923369-84923310	A_23_P22548	-0.73	-0.72	1.66E-05	2.25E-05
NM_183239	GSTO2	chr10:106047336-106047395	A_23_P202206	-0.73	-0.72	3.63E-03	5.74E-03
NM_016645	NGRN	chr15:88615477-88615536	A_23_P140602	-0.73	-0.72	1.06E-02	1.75E-02
NM_198433	AURKA	chr20:54378586-54378527	A_23_P131866	-0.73	-0.71	5.59E-04	1.00E-03
NM_015508	TIPARP	chr3:157907012-157907071	A_23_P143845	-0.73	-0.65	8.49E-05	5.68E-04

Gene	Symbol	Chromosomal position	Probe	siCASP8AP2.3	CASP8AP2.6	siCASP8AP2.3	CASP8AP2.6
				M	M	Q	Q
AK021668	AK021668	chr1:93515905-93515846	A_24_P481375	-0.73	-0.64	4.05E-04	2.20E-03
NM_000437	PAFAH2	chr1:25972479-25972420	A_23_P137765	-0.73	-0.61	5.68E-08	3.57E-06
NM_024662	NAT10	chr11:34124566-34124625	A_23_P87329	-0.73	-0.61	2.23E-03	1.49E-02
NM_015522	DYNC2LI1	chr2:43933330-43933389	A_23_P502170	-0.72	-1.28	1.47E-02	2.85E-05
NM_001008397	LOC493869	chr5:54496011-54496070	A_23_P122052	-0.72	-0.96	8.47E-03	6.47E-04
THC2337994	THC2337994	chr2:86163636-86163577	A_24_P936911	-0.72	-0.94	1.53E-04	1.70E-06
NM_018439	IMPACT	chr18:20285356-20285415	A_23_P208070	-0.72	-0.91	3.18E-04	1.00E-05
NM_002435	MPI	chr15:72977412-72977471	A_23_P60579	-0.72	-0.83	7.02E-03	2.45E-03
NM_172178	MRPL42	chr12:92397435-92397494	A_23_P25163	-0.72	-0.81	1.93E-02	1.16E-02
AI791206	AI791206	chr5:69241012-69241071	A_24_P856722	-0.72	-0.78	1.93E-02	1.60E-02
U09197	U09197	chr1:164409257-164409316	A_24_P937240	-0.72	-0.72	3.43E-04	4.92E-04
NM_022734	METT11D1	chr14:20534267-20534560	A_23_P205529	-0.72	-0.71	4.27E-05	5.46E-05
NM_023070	ZNF643	chr1:40598166-40598225	A_23_P160200	-0.72	-0.70	8.64E-04	1.68E-03
NM_173825	RABL3	chr3:121895716-121891992	A_24_P302785	-0.72	-0.70	1.88E-02	3.00E-02
NM_017684	VPS13C	chr15:59932183-59932124	A_23_P206228	-0.72	-0.68	1.71E-03	4.16E-03
NM_022039	FBXW4	chr10:103360897-103360838	A_23_P342825	-0.72	-0.66	7.48E-04	2.46E-03
NM_000291	PGK1	chrX:77187607-77187666	A_23_P125829	-0.72	-0.65	2.26E-05	1.20E-04
NM_152496	MANEAL	chr1:37935736-37935795	A_23_P391228	-0.72	-0.65	1.64E-02	4.54E-02
NM_004526	MCM2	chr3:128823503-128823562	A_32_P103633	-0.72	-0.61	6.33E-05	8.10E-04
NM_006390	IPO8	chr12:30678322-30676192	A_23_P98923	-0.72	-0.61	1.80E-04	1.89E-03
ENST00000282366	ENST00000282366	chr2:42895668-42895727	A_23_P411431	-0.71	-1.27	1.36E-03	7.14E-08
NM_001005368	ZNF32	chr10:43460158-43460099	A_23_P98057	-0.71	-1.13	2.76E-02	6.47E-04
NM_032632	PAPOLA	chr14:96038689-96056202	A_23_P419051	-0.71	-0.93	2.22E-04	3.32E-06
NM_003006	SELPLG	chr12:107518916-107518857	A_23_P64860	-0.71	-0.93	1.72E-03	6.46E-05
NM_005829	AP3S2	chr15:88179747-88179688	A_23_P100011	-0.71	-0.93	1.76E-02	2.40E-03
NM_015506	MMACHC	chr1:45645496-45645555	A_24_P83586	-0.71	-0.92	3.88E-02	9.41E-03
NM_017906	PAK1IP1	chr6:10817501-10817560	A_23_P122674	-0.71	-0.91	4.26E-03	4.12E-04
THC2357547	THC2357547	chr5:139469472-139469531	A_32_P66297	-0.71	-0.90	1.77E-04	4.12E-06
ENST00000335282	ENST00000335282	chr1:43287691-43291024	A_24_P847326	-0.71	-0.87	2.96E-03	4.01E-04
NM_014640	TTLL4	chr2:219443837-219443896	A_23_P142697	-0.71	-0.80	1.79E-04	3.23E-05
NM_017803	DUS2L	chr16:66666865-66668051	A_23_P88848	-0.71	-0.78	5.74E-04	1.96E-04
THC2376027	THC2376027	chr2:219761041-219760982	A_32_P203939	-0.71	-0.77	1.50E-03	7.80E-04
NM_020817	KIAA1407	chr3:115166014-115165955	A_23_P419213	-0.71	-0.74	4.04E-08	1.78E-08
NM_004849	ATG5	chr6:106740618-106740559	A_23_P111381	-0.71	-0.72	2.24E-03	2.50E-03
NM_018951	HOXA10	chr7:26984534-26984475	A_24_P77904	-0.71	-0.72	6.22E-03	7.72E-03
NM_030821	PLA2G12A	chr4:110993220-110993161	A_23_P30020	-0.71	-0.70	1.00E-02	1.56E-02
BC084557	BC084557	chr11:14869055-14868996	A_23_P335190	-0.71	-0.65	4.20E-03	1.18E-02
NM_145230	ATP6V0E2L	chr7:149014601-149014660	A_23_P61960	-0.71	-0.64	1.18E-04	5.98E-04
NM_015450	POT1	chr7:124057774-124057715	A_23_P84610	-0.71	-0.64	7.11E-04	2.61E-03
NM_003779	B4GALT3	chr1:157954479-157954420	A_23_P103919	-0.71	-0.64	2.81E-03	9.08E-03
NM_033655	CNTNAP3	chr9:39161403-39156052	A_24_P418203	-0.71	-0.64	3.80E-03	1.20E-02
ENST00000377047	ENST00000377047	chr13:93857335-93857394	A_32_P97169	-0.71	-0.64	4.93E-03	1.58E-02
NM_201453	CBWD3	chr9:68101409-68103019	A_24_P25080	-0.71	-0.64	1.49E-02	4.09E-02
NM_019887	DIABLO	chr12:121217497-121217438	A_23_P47800	-0.71	-0.62	4.28E-05	3.78E-04
D87470	KIAA0280	chr11:72789751-72789692	A_24_P188105	-0.71	-0.61	1.34E-02	4.79E-02
NM_005611	RBL2	chr16:52081616-52081675	A_23_P26413	-0.71	-0.60	6.62E-05	8.21E-04
ENST00000378953	ENST00000378953	chr5:131839514-131839573	A_24_P127641	-0.71	-0.60	2.12E-03	1.25E-02
NM_022090	LOC63920	chr5:159753568-159753509	A_24_P166311	-0.70	-1.14	1.05E-04	4.71E-09
NM_144680	ZNF18	chr17:11821691-11821632	A_23_P141302	-0.70	-0.98	1.61E-06	5.10E-10
A_24_P170203	A_24_P170203	chr9:106959246-106959311	A_24_P170203	-0.70	-0.96	2.96E-04	2.03E-06
NM_012089	ABCB10	chr1:225960831-225960772	A_24_P274073	-0.70	-0.93	1.34E-02	1.25E-03
A_32_P128399	A_32_P128399	chr2:074971578-074971519	A_32_P128399	-0.70	-0.92	1.95E-03	7.64E-05
NM_003409	ZFP161	chr18:5279958-5279899	A_24_P74064	-0.70	-0.89	1.38E-03	6.85E-05
NM_020242	KIF15	chr3:44869390-44869449	A_23_P80902	-0.70	-0.83	5.82E-06	1.72E-07
NM_015942	MTERFD1	chr8:97327311-97325473	A_23_P43071	-0.70	-0.83	1.22E-04	8.63E-06
CR612065	CR612065	chr10:102662285-102662226	A_32_P52251	-0.70	-0.80	2.16E-05	2.18E-06

Gene	Symbol	Chromosomal position	Probe	siCASP8AP2.3	CASP8AP2.6	siCASP8AP2.3	CASP8AP2.6
				M	M	Q	Q
NM_032970	SEC22C	chr3:42569756-42569697	A_23_P211997	-0.70	-0.79	2.62E-06	2.70E-07
NM_000017	ACADS	chr12:119639405-119639688	A_23_P65022	-0.70	-0.79	1.35E-04	2.50E-05
A_24_P341593	A_24_P341593	chr1:165151277-165151218	A_24_P341593	-0.70	-0.78	3.29E-03	1.42E-03
NM_025265	TSEN2	chr3:12549605-12549664	A_23_P92012	-0.70	-0.77	4.52E-04	1.46E-04
AK025818	AK025818	chr1:120743988-120744047	A_32_P15498	-0.70	-0.76	2.33E-02	1.84E-02
NM_004688	NMI	chr2:151952577-151952518	A_23_P154235	-0.70	-0.76	4.30E-02	3.57E-02
AK098811	LOC285398	chr3:53097629-53097570	A_24_P93741	-0.70	-0.72	1.11E-05	6.96E-06
NM_002775	HTRA1	chr10:124264328-124264387	A_23_P97990	-0.70	-0.71	6.99E-03	8.74E-03
NM_133510	RAD51L1	chr14:67947945-68004648	A_23_P48481	-0.70	-0.69	3.01E-09	4.09E-09
AK097550	FLJ39378	chr12:122481487-122481546	A_24_P922056	-0.70	-0.68	1.61E-04	2.67E-04
CR600369	CR600369	chr6:79702861-79702802	A_24_P630039	-0.70	-0.68	4.91E-03	8.42E-03
ENST00000329367	ENST00000329367	chr21:39316912-39316853	A_24_P324301	-0.70	-0.66	8.94E-03	1.96E-02
NM_006333	C1D	chr2:68181333-68181274	A_23_P67992	-0.70	-0.64	9.47E-03	2.47E-02
NM_014719	KIAA0738	chr7:142988170-142988111	A_32_P56525	-0.70	-0.62	5.27E-03	1.91E-02
NM_002807	PSMD1	chr2:231836560-231843826	A_24_P128205	-0.70	-0.61	3.05E-03	1.36E-02
NM_016030	TTC15	chr2:5019824-5020933	A_23_P256835	-0.70	-0.60	2.55E-05	3.81E-04
NM_013411	AK2	chr1:33144916-33144857	A_32_P207360	-0.70	-0.60	6.95E-03	3.15E-02
NM_001008397	LOC493869	chr5:54496011-54496070	A_23_P122052	-0.69	-1.10	1.18E-02	8.92E-05
NM_006947	SRP72	chr4:57185717-57190265	A_32_P174713	-0.69	-0.98	2.56E-02	1.84E-03
NM_022126	LHPP	chr10:126167095-126175539	A_24_P355493	-0.69	-0.97	4.54E-03	1.14E-04
NM_017906	PAK1IP1	chr6:10817501-10817560	A_23_P122674	-0.69	-0.86	5.22E-03	7.92E-04
NM_198401	ANKRD46	chr8:101602607-101602548	A_23_P94095	-0.69	-0.86	1.09E-02	2.09E-03
NM_018340	FLJ11151	chr16:12665108-12665049	A_23_P77401	-0.69	-0.84	4.03E-03	6.59E-04
NM_004332	BPHL	chr6:3097847-3097906	A_23_P42087	-0.69	-0.82	2.20E-04	1.66E-05
NM_053274	GLMN	chr1:92449120-92449061	A_23_P160742	-0.69	-0.81	1.60E-02	6.89E-03
BC111740	DNA2L	chr10:69848800-69846563	A_24_P366107	-0.69	-0.80	3.13E-03	8.36E-04
NM_004830	CRSP3	chr6:131949594-131949566	A_23_P145501	-0.69	-0.77	1.14E-04	2.69E-05
NM_002553	ORC5L	chr7:103360957-103360898	A_23_P31414	-0.69	-0.77	1.28E-03	4.95E-04
NM_032823	C9orf3	chr9:94928788-94928847	A_23_P73012	-0.69	-0.77	4.62E-03	2.26E-03
NM_144596	TTC8	chr14:88413494-88413553	A_32_P169735	-0.69	-0.76	2.64E-04	7.33E-05
NM_014748	SNX17	chr2:27508662-27508891	A_23_P28238	-0.69	-0.74	6.43E-03	4.82E-03
NM_001031703	TMEM103	chr3:47512311-47512252	A_23_P212310	-0.69	-0.73	1.54E-07	4.07E-08
A_24_P357836	A_24_P357836	chr2:183762461-183762402	A_24_P357836	-0.69	-0.73	1.76E-02	1.64E-02
THC2456973	THC2456973	chr3:37806696-37806637	A_32_P184509	-0.69	-0.72	4.32E-04	3.24E-04
NM_022362	MMS19L	chr10:99209194-99209135	A_24_P369263	-0.69	-0.71	9.45E-03	1.08E-02
NM_052950	WDFY2	chr13:51230394-51231892	A_24_P176131	-0.69	-0.69	1.58E-03	2.03E-03
BC107568	BC107568	chr17_random:2244135-2244194	A_24_P592544	-0.69	-0.67	7.97E-03	1.36E-02
NM_031954	KCTD10	chr12:108349739-108349680	A_24_P39101	-0.69	-0.66	1.22E-03	2.70E-03
A_24_P392742	A_24_P392742	chr1:078221458-078221397	A_24_P392742	-0.69	-0.66	7.95E-03	1.57E-02
NM_012145	DTYMK	chr2_random:328271-325998	A_23_P135364	-0.69	-0.65	1.50E-06	5.48E-06
NM_000535	PMS2	chr7:5786153-5786111	A_24_P410859	-0.69	-0.65	6.94E-05	1.81E-04
NM_134424	RAD52	chr12:892488-892429	A_23_P64990	-0.69	-0.65	2.49E-03	6.11E-03
AF217973	AF217973	chr1:54283648-54283707	A_23_P62684	-0.69	-0.64	1.23E-03	3.96E-03
NM_022474	MPP5	chr14:66849057-66849116	A_24_P347458	-0.69	-0.64	1.96E-02	4.36E-02
NM_024771	NAT11	chr11:63480378-63480437	A_23_P35782	-0.69	-0.61	7.71E-05	5.45E-04
THC2405262	THC2405262	chr20:23285335-23285276	A_32_P154223	-0.69	-0.61	4.73E-04	2.68E-03
NM_024086	METT10D	chr17:2268937-2268878	A_23_P365705	-0.69	-0.60	3.73E-04	2.53E-03
NM_018226	RNPEPL1	chr2:241237620-241237679	A_23_P17030	-0.69	-0.60	3.52E-03	1.47E-02
BC009539	C14orf46	chr14:73736466-73736525	A_23_P339309	-0.69	-0.60	6.43E-03	2.70E-02
NM_018218	USP40	chr2:234189217-234187443	A_24_P314688	-0.68	-0.98	8.36E-04	3.16E-06
NM_145080	NSMCE1	chr16:27153025-27151926	A_23_P95823	-0.68	-0.95	5.85E-05	9.96E-08
NM_015058	RP11-125A7.3	chr13:41039855-41039796	A_23_P432077	-0.68	-0.79	1.56E-05	7.38E-07
NM_015942	MTERFD1	chr8:97327777-97327718	A_24_P243834	-0.68	-0.77	2.74E-02	1.75E-02
NM_032547	SCOC	chr4:141659725-141659784	A_23_P167293	-0.68	-0.75	2.21E-02	1.65E-02
NM_004315	ASAH1	chr8:17961364-17961198	A_23_P216325	-0.68	-0.75	4.45E-02	3.81E-02
NM_015275	KIAA1033	chr12:104065263-104065322	A_32_P153725	-0.68	-0.74	6.53E-04	3.02E-04

Gene	Symbol	Chromosomal position	Probe	siCASP8AP2.3	CASP8AP2.6	siCASP8AP2.3	CASP8AP2.6
				M	M	Q	Q
NM_013396	USP25	chr21:16173427-16173486	A_24_P139208	-0.68	-0.74	1.81E-03	9.39E-04
NM_030794	TDRD3	chr13:60001255-60001314	A_32_P187617	-0.68	-0.74	4.26E-03	2.55E-03
A_24_P350136	A_24_P350136	chr2:065806365-065806426	A_24_P350136	-0.68	-0.74	1.07E-02	8.05E-03
NM_003820	TNFRSF14	chr1:2521576-2521517	A_23_P126908	-0.68	-0.72	6.88E-04	3.98E-04
NM_003142	SSB	chr2:170489044-170490513	A_32_P35512	-0.68	-0.70	1.37E-02	1.52E-02
NM_004289	NFE2L3	chr7:25998937-25998996	A_23_P42718	-0.68	-0.70	2.11E-02	2.65E-02
NM_025256	EHMT2	chr6:31972191-31972132	A_24_P303390	-0.68	-0.69	3.52E-03	4.07E-03
NM_032664	FUT10	chr8:33349240-33349181	A_23_P22409	-0.68	-0.67	1.47E-03	2.24E-03
NM_031450	C11orf68	chr11:65441140-65441081	A_23_P150238	-0.68	-0.64	4.35E-04	1.06E-03
A_32_P96124	A_32_P96124	chr14:104251821-104251762	A_32_P96124	-0.68	-0.64	7.72E-03	1.69E-02
NM_152327	AK7	chr14:96023030-96023089	A_23_P105963	-0.68	-0.61	4.80E-05	3.24E-04
A_24_P341731	A_24_P341731	chr22:029877116-029877057	A_24_P341731	-0.68	-0.61	1.24E-02	3.53E-02
NM_021931	DHX35	chr20:37101516-37101575	A_23_P5945	-0.67	-0.93	7.61E-06	4.40E-09
NM_015909	NAG	chr2:15269737-15258014	A_23_P332509	-0.67	-0.92	1.23E-04	4.58E-07
AB051436	ZNRF3	chr22:27777415-27777474	A_23_P427502	-0.67	-0.90	4.00E-03	1.62E-04
BE394309	BE394309	chr9:37766029-37765970	A_32_P149716	-0.67	-0.90	2.02E-02	2.31E-03
CR625571	CR625571	chr11:92908345-92908406	A_24_P289845	-0.67	-0.84	3.04E-03	2.81E-04
NM_000282	PCCA	chr13:99980385-99980444	A_23_P48358	-0.67	-0.79	3.60E-02	1.76E-02
NM_138573	NRG4	chr15:74088660-74088601	A_23_P334727	-0.67	-0.79	3.57E-02	1.86E-02
THC2432970	THC2432970	chr3:49696145-49696204	A_24_P200250	-0.67	-0.78	2.06E-05	1.09E-06
NM_152292	RG9MTD2	chr4:100825303-100825244	A_23_P398637	-0.67	-0.73	2.47E-05	4.97E-06
NM_138467	TYW3	chr1:74942001-74942060	A_23_P103172	-0.67	-0.71	7.15E-06	2.59E-06
A_32_P194182	A_32_P194182	chr21:016185923-016185982	A_32_P194182	-0.67	-0.67	6.21E-03	8.40E-03
NM_002857	PEX19	chr1:157059940-157059881	A_24_P19752	-0.67	-0.66	2.69E-03	4.23E-03
CR616033	CR616033	chr1:45832002-45831943	A_24_P855152	-0.67	-0.61	9.17E-04	3.02E-03
NM_001031711	ERGIC1	chr5:172311750-172311809	A_23_P404871	-0.67	-0.61	1.79E-03	6.05E-03
NM_001039613	LOC285148	chr2:9579037-9579096	A_23_P17287	-0.67	-0.60	3.30E-09	6.74E-08
NM_022905	TTC23	chr15:97494467-97494408	A_23_P140562	-0.67	-0.60	8.05E-07	7.22E-06
AK131430	AK131430	chr4:147039233-147039174	A_23_P72372	-0.67	-0.60	3.07E-04	1.64E-03
NM_001042552	TATDN3	chr1:209374035-209376778	A_23_P23522	-0.66	-1.11	1.26E-02	4.76E-05
NM_005834	TIMM17B	chrX:48507293-48507234	A_23_P33886	-0.66	-1.00	4.75E-03	3.31E-05
BU625522	BU625522	chr1:10453752-10453811	A_24_P914922	-0.66	-0.93	1.15E-03	7.74E-06
A_24_P195400	A_24_P195400	chr20:036677259-036677200	A_24_P195400	-0.66	-0.93	2.31E-02	1.84E-03
NM_000310	PPT1	chr1:40224242-40224183	A_24_P276628	-0.66	-0.93	4.41E-02	5.70E-03
NM_004569	PIGH	chr14:67126453-67126394	A_23_P2884	-0.66	-0.92	1.68E-03	2.37E-05
NM_001003676	C11orf49	chr11:47140024-47140083	A_23_P147605	-0.66	-0.89	3.05E-05	7.50E-08
NM_178454	TMEM77	chr1:111374606-111374547	A_24_P268160	-0.66	-0.88	1.26E-05	2.94E-08
A_24_P221724	A_24_P221724	chr22:017620444-017620503	A_24_P221724	-0.66	-0.84	1.16E-02	1.95E-03
A_23_P20793	A_23_P20793		A_23_P20793	-0.66	-0.81	1.51E-04	6.34E-06
NM_006371	CRTAP	chr3:33163810-33163869	A_24_P71661	-0.66	-0.79	5.49E-05	2.26E-06
NM_152564	VPS13B	chr8:100563203-100583146	A_24_P280628	-0.66	-0.76	3.69E-05	2.55E-06
NM_001916	CYC1	chr8:145223296-145223355	A_23_P20980	-0.66	-0.75	2.31E-03	7.19E-04
NM_144620	LRRC39	chr1:100326614-100326555	A_23_P330578	-0.66	-0.70	8.73E-04	5.58E-04
NM_001098	ACO2	chr22:40249318-40249377	A_23_P103149	-0.66	-0.67	8.80E-06	6.79E-06
NM_003677	DENR	chr12:121778517-121778576	A_23_P162256	-0.66	-0.66	6.95E-03	1.05E-02
NM_012102	RERE	chr1:8347311-8347252	A_24_P342591	-0.66	-0.63	3.32E-03	7.26E-03
NM_203487	PCDH9	chr13:65775446-65775387	A_23_P420236	-0.66	-0.62	8.90E-04	2.24E-03
NM_017623	CNNM3	chr2:96920692-96920751	A_23_P113405	-0.66	-0.61	4.97E-03	1.28E-02
NM_025137	KIAA1840	chr15:42642523-42642464	A_23_P65699	-0.65	-0.99	1.18E-03	2.29E-06
NM_018178	GOLPH3L	chr1:147431905-147431846	A_24_P345377	-0.65	-0.97	3.17E-03	2.03E-05
NM_022460	HS1BP3	chr2:20739563-20739504	A_24_P139943	-0.65	-0.94	4.30E-04	8.30E-07
THC2338229	THC2338229	chr16:49240539-49240598	A_32_P77742	-0.65	-0.94	3.74E-03	4.69E-05
NM_032823	C9orf3	chr9:94902646-94922588	A_24_P89887	-0.65	-0.90	3.10E-02	3.81E-03
NM_000281	PCBD1	chr10:72313517-72313458	A_23_P367405	-0.65	-0.89	8.84E-03	5.49E-04
NM_018710	TMEM55A	chr8:92075992-92075933	A_23_P422083	-0.65	-0.86	1.05E-04	8.22E-07
NM_032604	ABHD1	chr2:27265267-27265326	A_23_P17242	-0.65	-0.84	2.94E-02	6.86E-03

Gene	Symbol	Chromosomal position	Probe	siCASP8AP2.3	CASP8AP2.6	siCASP8AP2.3	CASP8AP2.6
				M	M	Q	Q
AK091942	AK091942	chr7:154935042-154934983	A_32_P13417	-0.65	-0.72	1.99E-03	7.28E-04
A_24_P409410	A_24_P409410	chr14:053528272-053528211	A_24_P409410	-0.65	-0.71	3.92E-02	3.33E-02
NM_004688	NMI	chr2:151952577-151952518	A_23_P154235	-0.65	-0.71	4.68E-02	4.05E-02
NM_004599	SREBF2	chr22:40626699-40626758	A_23_P419602	-0.65	-0.69	2.19E-03	1.57E-03
AK022223	DCAKD	chr17:40457998-40457939	A_24_P636441	-0.65	-0.68	3.31E-02	3.66E-02
NM_021177	LSM2	chr6:31874255-31873769	A_23_P59153	-0.65	-0.66	1.24E-03	1.48E-03
NM_014051	TMEM14A	chr6:52656858-52656917	A_23_P42080	-0.65	-0.66	6.72E-03	8.28E-03
NM_006473	TAF6L	chr11:62310929-62310988	A_23_P47541	-0.65	-0.66	6.79E-03	8.77E-03
CR626626	CR626626	chr7:96240058-96239999	A_32_P180524	-0.65	-0.65	5.40E-07	6.10E-07
AF086335	AF086335	chr15:99428238-99428179	A_24_P935667	-0.65	-0.65	6.50E-04	9.00E-04
NM_001008726	WDR89	chr14:63133690-63133631	A_23_P163047	-0.65	-0.64	3.42E-04	6.12E-04
NM_006567	FARS2	chr6:5376289-5376348	A_23_P33720	-0.65	-0.63	1.13E-06	2.51E-06
NM_015440	MTHFD1L	chr6:151447838-151450261	A_32_P135243	-0.65	-0.63	2.23E-03	3.90E-03
NM_024095	ASB8	chr12:46829683-46829624	A_23_P22200	-0.65	-0.63	3.17E-03	5.96E-03
NM_003458	BSN	chr3:49683791-49683850	A_23_P29735	-0.65	-0.60	5.79E-04	1.76E-03
NM_016143	NSFL1C	chr20:1371172-1371113	A_23_P210719	-0.64	-1.30	1.25E-02	1.30E-06
ENST00000333503	ENST00000333503	chr16:2202366-2202307	A_24_P942805	-0.64	-0.98	5.89E-03	4.67E-05
NM_001667	ARL2	chr11:64545845-64545904	A_23_P98252	-0.64	-0.87	9.37E-03	6.26E-04
NM_006907	PYCR1	chr17:77483681-77483622	A_23_P130194	-0.64	-0.79	8.56E-03	1.52E-03
NM_016085	C2orf28	chr2:27350210-27350269	A_23_P28652	-0.64	-0.78	2.82E-02	9.94E-03
THC2397883	THC2397883	chr3:190081160-190081219	A_32_P70519	-0.64	-0.76	4.68E-03	1.09E-03
NM_014168	METTL5	chr2:170497516-170494488	A_23_P142634	-0.64	-0.74	5.13E-04	9.53E-05
NM_003731	SSNA1	chr9:137359543-137360143	A_23_P159476	-0.64	-0.73	1.37E-03	3.74E-04
NM_000484	APP	chr21:26191816-26191757	A_24_P314159	-0.64	-0.73	3.11E-02	1.87E-02
NM_000110	DPYD	chr1:97276102-97260026	A_23_P43988	-0.64	-0.71	1.19E-02	7.18E-03
NM_005589	ALDH6A1	chr14:73596942-73596883	A_23_P128967	-0.64	-0.70	3.92E-04	1.47E-04
BC110499	USP22	chr17:20846946-20846887	A_23_P207068	-0.64	-0.70	4.52E-03	2.60E-03
NM_007208	MRPL3	chr3:132703257-132703198	A_23_P7030	-0.64	-0.68	1.39E-02	1.28E-02
A_24_P615462	A_24_P615462	chr10:097127775-097127716	A_24_P615462	-0.64	-0.67	3.37E-02	3.55E-02
AK022252	AK022252	chr17:72937963-72938022	A_23_P118370	-0.64	-0.64	1.27E-02	1.69E-02
ENST00000360201	ENST00000360201	chr3:127812588-127810126	A_32_P170925	-0.64	-0.60	1.75E-03	4.80E-03
NM_145267	C6orf57	chr6:71345934-71355041	A_23_P256279	-0.63	-1.22	2.18E-05	1.07E-12
A_24_P298928	A_24_P298928	chr7:126215119-126215178	A_24_P298928	-0.63	-1.16	1.31E-02	8.91E-06
NM_031446	C18orf21	chr18:31811458-31811517	A_23_P101237	-0.63	-0.93	9.53E-03	2.11E-04
NM_022070	ABC1	chr17:55475994-55475935	A_23_P77965	-0.63	-0.92	1.19E-05	2.29E-09
NM_004550	NDUFS2	chr1:157993210-157993470	A_23_P149470	-0.63	-0.92	9.31E-04	2.58E-06
NM_173825	RABL3	chr3:121932261-121911416	A_32_P109057	-0.63	-0.84	1.00E-03	2.11E-05
NM_018559	KIAA1704	chr13:44487665-44487724	A_24_P227585	-0.63	-0.80	4.11E-04	1.18E-05
NM_144998	STRA13	chr17:77569984-77569925	A_24_P65507	-0.63	-0.78	9.63E-04	5.97E-05
NM_000484	APP	chr21:26191816-26191757	A_24_P314159	-0.63	-0.78	3.79E-02	1.36E-02
NM_004060	CCNG1	chr5:162803587-162803646	A_23_P58606	-0.63	-0.77	1.66E-02	4.78E-03
NM_025132	WDR19	chr4:39094281-39097219	A_24_P394368	-0.63	-0.73	1.25E-04	1.41E-05
NM_006493	CLN5	chr13:76474420-76474479	A_23_P117286	-0.63	-0.70	5.55E-03	2.51E-03
BC041772	LOC124976	chr17:4382601-4382660	A_24_P230173	-0.63	-0.69	8.90E-04	4.01E-04
NM_004630	SF1	chr11:64288914-64288855	A_24_P266728	-0.63	-0.65	3.55E-02	4.19E-02
NM_032900	ARHGAP19	chr10:98975773-98975714	A_23_P334845	-0.63	-0.64	1.40E-04	1.30E-04
NM_138578	BCL2L1	chr20:29717044-29716985	A_23_P210886	-0.63	-0.64	2.65E-02	3.46E-02
NM_004344	CETN2	chrX:151666808-151666749	A_23_P73493	-0.63	-0.63	2.71E-04	3.15E-04
NM_014678	SAPS2	chr22:49168600-49169431	A_24_P186174	-0.63	-0.63	4.20E-03	5.70E-03
ENST00000341154	ENST00000341154	chr1:24036839-24036780	A_23_P311201	-0.63	-0.62	1.35E-04	1.92E-04
NM_022077	MANBAL	chr20:35378683-35378742	A_23_P131778	-0.63	-0.61	2.03E-03	3.97E-03
NM_005900	SMAD1	chr4:146836638-146836697	A_23_P212870	-0.63	-0.60	1.25E-03	2.77E-03
NM_001005920	LOC339123	chr16:672616-672557	A_23_P15073	-0.62	-1.08	2.88E-03	7.99E-07
BC024007	CTBS	chr1:84731483-84731424	A_24_P940135	-0.62	-0.93	1.27E-02	2.33E-04
AK021694	CXorf10	chr12:3586278-3586219	A_32_P462013	-0.62	-0.93	1.28E-02	3.17E-04
NM_021807	EXOC4	chr7:133207136-133207195	A_23_P135437	-0.62	-0.90	2.10E-02	1.13E-03

Gene	Symbol	Chromosomal position	Probe	siCASP8AP2.3	CASP8AP2.6	siCASP8AP2.3	CASP8AP2.6
				M	M	Q	Q
NM_020787	ZNF624	chr17:16465122-16465063	A_23_P153037	-0.62	-0.87	1.25E-05	8.86E-09
NM_018273	TMEM143	chr19:53527488-53527429	A_24_P412976	-0.62	-0.86	2.76E-03	5.90E-05
NM_005922	MAP3K4	chr6:161506642-161506701	A_23_P42096	-0.62	-0.84	5.11E-04	4.45E-06
NM_004747	DLG5	chr10:79222276-79222217	A_23_P161209	-0.62	-0.83	2.66E-02	3.99E-03
NM_032023	RASSF4	chr10:44806515-44807409	A_23_P75260	-0.62	-0.80	2.27E-02	4.04E-03
NM_024052	C17orf39	chr17:17905999-17909262	A_23_P164427	-0.62	-0.78	2.47E-03	2.11E-04
NM_080476	CDC91L1	chr20:32689387-32686168	A_23_P28980	-0.62	-0.77	1.27E-04	3.41E-06
NM_016495	TBC1D7	chr6:13415857-13414726	A_23_P111273	-0.62	-0.74	6.82E-04	7.83E-05
NM_194314	ZBTB41	chr1:193855052-193854993	A_32_P108826	-0.62	-0.71	4.65E-04	1.03E-04
NM_020909	EPB41L5	chr2:120652416-120652475	A_24_P944640	-0.62	-0.70	3.17E-03	1.30E-03
NM_000484	APP	chr21:26191816-26191757	A_24_P314159	-0.62	-0.70	4.94E-02	3.32E-02
A_24_P799580	A_24_P799580		A_24_P799580	-0.62	-0.66	2.58E-04	1.26E-04
THC2437154	THC2437154	chr16:31371265-31371206	A_32_P24295	-0.62	-0.65	2.20E-02	2.34E-02
NM_152395	NUDT16	chr3:132587563-132587622	A_23_P310560	-0.62	-0.64	3.11E-04	2.52E-04
NM_015305	ANGEL1	chr14:76323688-76323629	A_23_P363406	-0.62	-0.64	4.07E-03	4.52E-03
NM_004853	STX8	chr17:9349137-9335962	A_23_P146908	-0.62	-0.63	8.57E-05	7.10E-05
NM_020232	TNFSF5IP1	chr18:12710519-12710578	A_24_P399362	-0.62	-0.62	4.32E-04	5.20E-04
NM_018347	C20orf29	chr20:3753889-3753948	A_23_P57137	-0.62	-0.61	1.65E-03	2.35E-03
THC2315174	THC2315174	chr3:049329641-049329582	A_23_P58091	-0.62	-0.60	1.75E-03	3.32E-03
NM_025125	C10orf57	chr10:81842020-81842079	A_23_P97853	-0.61	-1.02	3.77E-03	3.23E-06
NM_017437	CPSF2	chr14:91698554-91698613	A_23_P99837	-0.61	-1.01	1.83E-03	1.07E-06
NM_007167	ZMYM6	chr1:35118590-35118531	A_24_P370670	-0.61	-1.00	1.97E-02	2.14E-04
NM_006788	RALBP1	chr18:9527396-9527455	A_23_P376599	-0.61	-0.97	2.56E-02	4.88E-04
ENST00000368025	ENST00000368025	chr1:157820896-157820837	A_23_P217901	-0.61	-0.91	9.71E-05	3.61E-08
NM_017763	RNF43	chr17:53786511-53786453	A_23_P3934	-0.61	-0.86	2.98E-03	5.04E-05
NM_194247	HNRPA3	chr2:177913816-177913875	A_24_P706312	-0.61	-0.85	6.71E-03	2.27E-04
ENST00000279968	ENST00000279968	chr11:124472477-124472418	A_23_P1676	-0.61	-0.84	3.58E-04	2.31E-06
NM_006324	CFDP1	chr16:73986580-73986521	A_23_P89123	-0.61	-0.83	4.43E-03	1.90E-04
NM_017868	TTC12	chr11:112739768-112739827	A_24_P73075	-0.61	-0.79	7.94E-03	8.72E-04
A_24_P633686	A_24_P633686	chr1:158145552-158145614	A_24_P633686	-0.61	-0.78	1.36E-02	2.32E-03
NM_004730	ETF1	chr5:137870358-137870299	A_23_P133582	-0.61	-0.77	2.41E-02	5.73E-03
NM_016565	CHCHD8	chr11:73261550-73261491	A_23_P13502	-0.61	-0.75	4.48E-07	2.31E-09
NM_001039845	MDH1B	chr2:207445721-207445662	A_24_P111547	-0.61	-0.74	1.78E-03	2.48E-04
AK075235	SVEP1	chr9:110217218-110211808	A_23_P216596	-0.61	-0.70	1.11E-03	2.60E-04
NM_000293	PHKB	chr16:46290901-46290960	A_23_P206532	-0.61	-0.70	1.88E-03	4.61E-04
NM_018684	KIAA1166	chrX:63919476-63919417	A_23_P62188	-0.61	-0.69	3.51E-03	1.32E-03
NM_018719	CDCA7L	chr7:21714131-21714072	A_24_P274795	-0.61	-0.69	3.34E-02	2.32E-02
NM_003079	SMARCE1	chr17:36037668-36037609	A_23_P164387	-0.61	-0.68	2.73E-04	6.31E-05
NM_015913	TXNDC12	chr1:52201215-52198678	A_23_P200199	-0.61	-0.67	6.39E-03	3.78E-03
NM_152678	FAM116A	chr3:57586343-57586284	A_32_P118586	-0.61	-0.66	1.33E-04	4.24E-05
NM_024648	FLJ22222	chr17:77957603-77956507	A_24_P364954	-0.61	-0.62	9.09E-05	7.51E-05
NM_001316	CSE1L	chr20:47146357-47146416	A_23_P17393	-0.61	-0.61	9.87E-04	1.27E-03
NM_015396	ARMC8	chr3:139492132-139497410	A_24_P211869	-0.61	-0.61	2.22E-03	3.04E-03
AF426262	MCM3APAS	chr21:46486975-46487034	A_23_P391238	-0.61	-0.61	6.81E-03	9.48E-03
NM_144765	EVA1	chr11:117633125-117633066	A_23_P150379	-0.61	-0.61	1.72E-02	2.49E-02
NM_025247	ACAD10	chr12:110656209-110656268	A_24_P227450	-0.61	-0.60	1.09E-03	1.66E-03
NM_015565	ZNF294	chr21:29223348-29223289	A_32_P115505	-0.60	-0.98	2.07E-04	1.46E-08
A_32_P16931	A_32_P16931	chr2:208946279-208946338	A_32_P16931	-0.60	-0.94	1.93E-02	3.15E-04
NM_139177	SLC39A11	chr17:68153865-68153806	A_24_P57528	-0.60	-0.90	1.36E-02	3.37E-04
AK023257	FLJ13195	chr7:66229627-66229686	A_32_P98927	-0.60	-0.74	5.22E-05	1.32E-06
THC2440782	THC2440782	chr7:149539318-149539259	A_32_P112078	-0.60	-0.72	1.16E-02	3.29E-03
BC107860	LOC401397	chr7:112351074-112351015	A_32_P54305	-0.60	-0.71	3.24E-02	1.50E-02
NM_018141	MRPS10	chr6:42286249-42284650	A_24_P283294	-0.60	-0.69	1.88E-02	8.86E-03
NM_022087	GALNT11	chr7:151256793-151256852	A_23_P8412	-0.60	-0.68	3.78E-05	3.79E-06
NM_001040431	CCDC56	chr17:38204013-38203654	A_24_P356453	-0.60	-0.68	6.54E-05	8.63E-06
NM_032813	TMTC4	chr13:100075877-100075818	A_24_P278853	-0.60	-0.68	2.55E-04	4.33E-05

Gene	Symbol	Chromosomal position	Probe	siCASP8AP2.3	CASP8AP2.6	siCASP8AP2.3	CASP8AP2.6
				M	M	Q	Q
AF323119	NFE2L2	chr2:177954984-177954925	A_24_P936444	-0.60	-0.67	3.19E-02	2.07E-02
NM_032280	ZCCHC9	chr5:80644318-80644377	A_23_P156156	-0.60	-0.66	9.40E-06	1.88E-06
NM_080414	VPS16	chr20:2794106-2794881	A_23_P143303	-0.60	-0.66	4.26E-03	2.40E-03
NM_001037165	FOXK1	chr7:4583640-4583699	A_24_P115199	-0.60	-0.66	6.86E-03	3.66E-03
NM_024328	THTPA	chr14:23098333-23098392	A_23_P54041	-0.60	-0.66	8.89E-03	5.54E-03
BX102314	BX102314	chr12:22732019-22731960	A_32_P149389	-0.60	-0.65	1.83E-04	5.88E-05
NM_018441	PECR	chr2:216729552-216729493	A_23_P91140	-0.60	-0.65	2.12E-04	6.73E-05
NM_018677	ACSS2	chr20:32979004-32979062	A_23_P210900	-0.60	-0.63	2.14E-02	2.35E-02
NM_199133	LOC134145	chr5:10289606-10288331	A_24_P926314	-0.60	-0.63	4.49E-02	4.63E-02
NM_004299	ABCB7	chrX:74056414-74056355	A_23_P171258	-0.60	-0.62	2.40E-03	2.19E-03
NM_014846	KIAA0196	chr8:126106049-126105990	A_23_P112113	-0.60	-0.61	1.03E-03	1.06E-03
NM_021914	CFL2	chr14:34251272-34251213	A_23_P65401	-0.60	-0.61	3.70E-02	4.69E-02
NM_014960	ARSG	chr17:63928124-63928183	A_24_P339416	0.60	0.62	1.47E-03	1.13E-03
NM_015528	RNF167	chr17:4789122-4789181	A_24_P76911	0.60	0.65	1.09E-03	5.78E-04
NM_001114	ADCY7	chr16:48907161-48907220	A_23_P106945	0.60	0.65	1.55E-03	8.48E-04
NM_002471	MYH6	chr14:22924078-22924019	A_23_P37167	0.60	0.69	1.47E-09	2.79E-11
NM_013447	EMR2	chr19:14704917-14704858	A_23_P502336	0.60	0.70	1.40E-02	5.54E-03
NM_031483	ITCH	chr20:32555830-32559180	A_23_P132019	0.60	0.74	2.16E-04	7.32E-06
NM_018841	GNG12	chr1:67879171-67879134	A_23_P311732	0.60	0.75	1.88E-02	4.72E-03
NM_013360	ZNF222	chr19:49228783-49228842	A_24_P165082	0.60	0.75	2.52E-02	6.69E-03
NM_006532	ELL	chr19:18414869-18414810	A_23_P16537	0.60	0.77	6.80E-04	1.77E-05
NM_001008493	ENAH	chr1:221990090-221990031	A_23_P51397	0.60	0.77	5.26E-03	5.71E-04
NM_005054	RGPD5	chr2:109970864-109970923	A_32_P205944	0.60	0.77	1.41E-02	2.28E-03
NM_173481	C19orf21	chr19:714774-714833	A_23_P390068	0.60	0.77	3.65E-02	9.92E-03
NM_173822	MGC39518	chr2:201670047-201669988	A_23_P334864	0.60	0.79	1.76E-02	2.53E-03
NM_002502	NFKB2	chr10:104151043-104151219	A_23_P202156	0.60	0.82	4.08E-03	1.64E-04
NM_052836	CDH23	chr10:73046995-73047054	A_23_P307968	0.60	0.82	9.15E-03	4.98E-04
NM_173357	SSX6	chrX:47735851-47735910	A_23_P33881	0.60	0.85	2.82E-05	1.78E-08
NM_003807	TNFSF14	chr19:6615998-6615939	A_24_P237036	0.60	0.88	7.34E-04	1.81E-06
NM_078481	CD97	chr19:14379022-14379759	A_23_P502314	0.60	0.90	1.87E-02	5.99E-04
NM_005366	MAGEA11	chrX:148474169-148474110	A_23_P113553	0.60	0.93	2.07E-06	2.68E-11
NM_003246	THBS1	chr15:37675202-37675261	A_23_P206212	0.60	0.93	2.26E-03	5.42E-06
NM_003246	THBS1	chr15:37675202-37675261	A_23_P206212	0.60	0.94	3.06E-03	8.76E-06
NM_001235	SERPINH1	chr11:74957490-74957658	A_23_P76006	0.60	0.94	4.91E-02	2.23E-03
NM_002084	GPX3	chr5:150388052-150388111	A_23_P133474	0.60	1.12	1.07E-02	5.37E-06
THC2391898	THC2391898	chr4:189457169-189457228	A_32_P203408	0.60	1.13	4.72E-03	4.75E-07
AK093229	AK093229	chr11:8959725-8959666	A_32_P486693	0.60	1.26	4.37E-05	2.31E-13
NM_002086	GRB2	chr17:70825989-70825930	A_23_P77847	0.61	0.60	5.71E-04	9.01E-04
NM_019013	FAM64A	chr17:6294966-6295025	A_23_P49878	0.61	0.61	5.22E-03	7.36E-03
NM_017705	PAQR5	chr15:67483280-67483339	A_23_P163251	0.61	0.63	6.35E-04	5.33E-04
ENST00000304372	ENST00000304372	chr16:65880938-65880879	A_23_P37785	0.61	0.64	3.51E-05	1.85E-05
NM_152468	TMC8	chr17:73646842-73646901	A_23_P411246	0.61	0.64	1.56E-02	1.70E-02
NM_181531	BTN2A2	chr6:26501788-26501847	A_24_P249072	0.61	0.68	2.71E-07	2.77E-08
NM_003844	TNFRSF10A	chr8:23105257-23105198	A_23_P255653	0.61	0.70	3.48E-03	1.01E-03
NM_024913	FLJ21986	chr7:120362418-120362477	A_24_P187799	0.61	0.75	1.64E-08	3.88E-11
THC2407823	THC2407823	chr6:71165725-71165784	A_32_P112263	0.61	0.75	2.41E-05	4.18E-07
NM_022168	IFIH1	chr2:162954372-162954313	A_23_P68155	0.61	0.78	1.89E-02	3.68E-03
NM_016190	CRNN	chr1:149194865-149194806	A_23_P115202	0.61	0.79	5.12E-05	3.72E-07
NM_130769	GPHA2	chr11:64459092-64458917	A_23_P47181	0.61	0.81	7.51E-05	4.24E-07
NM_000371	TTR	chr18:27432718-27432777	A_23_P130333	0.61	0.91	3.42E-07	5.34E-12
NM_003246	THBS1	chr15:37675202-37675261	A_23_P206212	0.61	0.93	2.21E-03	6.11E-06
AK026213	WDR42A	chr1:157045530-157045589	A_24_P886960	0.61	0.95	2.88E-04	8.72E-08
NM_018370	FLJ11259	chr12:100819796-100819855	A_24_P355816	0.61	0.95	1.17E-02	1.33E-04
NM_000697	ALOX12	chr17:6854634-6854693	A_23_P152906	0.61	0.96	1.15E-05	2.46E-10
ENST00000370306	ENST00000370306	chrX:151496228-151496287	A_32_P115840	0.61	1.00	5.08E-04	6.93E-08
XM_934719	LOC647322	chr2:87487448-87483993	A_24_P703642	0.61	1.11	2.24E-02	6.40E-05

Gene	Symbol	Chromosomal position	Probe	siCASP8AP2.3	CASP8AP2.6	siCASP8AP2.3	CASP8AP2.6
				M	M	Q	Q
NM_020179	C11orf75	chr11:92851439-92851380	A_23_P75430	0.62	0.62	2.03E-04	2.26E-04
BC007394	MGC16291	chr10:42293317-42293376	A_23_P63736	0.62	0.64	2.59E-05	1.46E-05
NM_001035	RYR2	chr1:234322464-234322523	A_23_P137797	0.62	0.64	8.74E-05	6.96E-05
NM_003463	PTP4A1	chr6:64346804-64346863	A_24_P252043	0.62	0.69	5.88E-03	2.93E-03
THC2311746	THC2311746	chr7:106977463-106977404	A_32_P100430	0.62	0.72	1.87E-03	4.18E-04
ENST00000360178	ENST00000360178	chr3:131344998-131344939	A_23_P159211	0.62	0.75	2.72E-05	7.58E-07
AK125850	AK125850	chr9:45621672-45621730	A_32_P154601	0.62	0.75	1.57E-03	2.20E-04
NM_144672	OTOA	chr16:21671852-21671911	A_32_P52519	0.62	0.76	2.49E-04	1.24E-05
NM_020168	PAK6	chr15:38356620-38356679	A_24_P159227	0.62	0.77	7.16E-04	4.31E-05
NM_001932	MPP3	chr17:39234461-39234402	A_23_P141345	0.62	0.77	1.50E-03	1.14E-04
NM_024821	FLJ22349	chr22:40546495-40546554	A_23_P155106	0.62	0.78	1.78E-03	1.28E-04
NM_000639	FASLG	chr1:169367342-169367401	A_23_P369815	0.62	0.78	4.45E-02	1.51E-02
NM_001004023	DYRK3	chr1:203210862-203210921	A_23_P12282	0.62	0.82	1.42E-04	1.10E-06
NM_182565	FAM100B	chr17:71778904-71778963	A_24_P194714	0.62	0.84	1.45E-02	1.27E-03
BF978194	BF978194	chr12:64327792-64327851	A_24_P461881	0.62	0.85	1.26E-13	3.72E-19
H81180	H81180	chr1:146670689-146670748	A_32_P143496	0.62	0.85	5.45E-09	3.01E-13
NM_181531	BTN2A2	chr6:26501895-26501954	A_24_P337592	0.62	0.93	9.04E-06	6.16E-10
NM_004210	NEURL	chr10:105342060-105342119	A_23_P322562	0.62	0.94	2.21E-04	1.06E-07
NM_001046	SLC12A2	chr5:127550303-127550362	A_23_P133606	0.62	0.94	3.50E-02	1.94E-03
NM_080731	HOM-TES-103	chr12:6519167-6519108	A_23_P87742	0.62	0.98	2.14E-03	3.06E-06
NM_001558	IL10RA	chr11:117376908-117376967	A_24_P107303	0.62	0.99	1.98E-02	2.43E-04
NM_000596	IGFBP1	chr7:45706206-45706265	A_23_P42868	0.62	1.03	2.21E-02	2.20E-04
NM_033069	C6orf114	chr6:13577837-13577778	A_23_P134058	0.62	1.14	4.83E-04	3.09E-09
NM_015368	PANX1	chr11:93552954-93553013	A_23_P47155	0.62	1.24	9.77E-03	8.38E-07
THC2407039	THC2407039	chr10:53749146-53749205	A_32_P71032	0.62	1.76	2.25E-02	2.98E-09
NM_017977	AIM1L	chr1:26332649-26332590	A_23_P360329	0.63	0.60	1.73E-02	3.40E-02
NM_005644	TAF12	chr1:28750405-28750346	A_23_P63178	0.63	0.61	4.30E-05	9.28E-05
NM_000089	COL1A2	chr7:93703294-93703353	A_24_P277934	0.63	0.61	2.58E-03	4.28E-03
NM_032855	HSH2D	chr19:16130004-16130063	A_23_P153372	0.63	0.62	1.49E-02	2.55E-02
NM_006368	CREB3	chr9:35723444-35725126	A_23_P423389	0.63	0.62	2.38E-02	3.46E-02
NM_019013	FAM64A	chr17:6294966-6295025	A_23_P49878	0.63	0.64	1.42E-03	1.57E-03
NM_032728	PPAPDC3	chr9:131214132-131214191	A_23_P157736	0.63	0.65	1.16E-04	8.88E-05
BC039117	OVOS2	chr12:31159067-31158636	A_23_P25069	0.63	0.65	2.37E-02	2.76E-02
NM_000089	COL1A2	chr7:93703294-93703353	A_24_P277934	0.63	0.66	1.18E-02	1.25E-02
NM_007225	NXPH3	chr17:45011995-45012054	A_24_P150931	0.63	0.67	2.77E-05	1.16E-05
NM_024902	FLJ13236	chr12:48029547-48029606	A_32_P407245	0.63	0.67	2.09E-02	2.07E-02
NM_145239	PRRT2	chr16:29734440-29734499	A_23_P66017	0.63	0.68	5.85E-03	3.69E-03
NM_031207	HYI	chr1:43586246-43586187	A_23_P200976	0.63	0.71	4.91E-03	2.22E-03
ENST00000342345	ENST00000342345	chr2:73955709-73955768	A_24_P170403	0.63	0.75	5.35E-03	1.30E-03
AL834308	C1orf167	chr1:11783742-11783801	A_24_P306594	0.63	0.78	6.08E-05	1.59E-06
NM_015683	ARRDC2	chr19:17985251-17985310	A_24_P22976	0.63	0.79	5.15E-04	2.38E-05
NM_014182	ORMDL2	chr12:54500404-54500463	A_23_P87500	0.63	0.79	9.93E-04	5.38E-05
NR_002211	MEIS3P1	chr17:20435191-20435250	A_24_P648880	0.63	0.79	3.81E-02	1.19E-02
NM_130766	SKIP	chr17:1344681-1344622	A_23_P55076	0.63	0.80	2.19E-02	5.33E-03
NM_018406	MUC4	chr3:196963807-196963748	A_24_P208825	0.63	0.81	2.32E-04	3.95E-06
NM_000955	PTGER1	chr19:14445264-14445205	A_23_P4808	0.63	0.82	4.31E-05	3.69E-07
NM_004428	EFNA1	chr1:151920281-151920340	A_23_P254512	0.63	0.83	3.68E-06	4.94E-09
NM_000639	FASLG	chr1:169367342-169367401	A_23_P369815	0.63	0.83	2.01E-02	3.20E-03
ENST00000357776	ENST00000357776	chr17:40338189-40338248	A_23_P26928	0.63	0.84	4.42E-02	9.26E-03
NM_006915	RP2	chrX:46497921-46497980	A_23_P22433	0.63	0.88	1.91E-03	3.04E-05
NM_004418	DUSP2	chr2:96231836-96231777	A_24_P37409	0.63	0.88	2.89E-02	2.94E-03
NM_003071	SMARCA3	chr3:150231148-150231089	A_24_P277155	0.63	0.89	6.94E-11	3.79E-16
NM_006087	TUBB4	chr19:6446948-6446889	A_23_P50338	0.63	0.89	1.08E-02	4.65E-04
NM_144583	ATP6V1C2	chr2:10873902-10873961	A_23_P250914	0.63	0.91	4.95E-03	7.89E-05
NM_001001664	LOC339745	chr2:139156040-139156099	A_23_P79681	0.63	0.94	4.40E-02	3.29E-03
NM_006418	OLFM4	chr13:52524096-52524155	A_24_P181254	0.63	1.02	3.92E-04	7.67E-08

Gene	Symbol	Chromosomal position	Probe	siCASP8AP2.3	CASP8AP2.6	siCASP8AP2.3	CASP8AP2.6
				M	M	Q	Q
NM_000185	SERPIND1	chr22:19466274-19466333	A_23_P6335	0.63	1.06	1.15E-07	1.42E-14
NM_001307	CLDN7	chr17:7104710-7104546	A_23_P164284	0.63	1.28	1.13E-02	8.40E-07
BC064621	VPS37D	chr7:72530777-72530836	A_23_P409417	0.64	0.60	3.03E-04	9.57E-04
NM_005414	SKIL	chr3:171592796-171592855	A_23_P351215	0.64	0.60	1.04E-03	2.63E-03
NM_199341	LOC374920	chr19:53392615-53392674	A_23_P4922	0.64	0.64	1.76E-02	2.40E-02
NM_016135	ETV7	chr6:36444774-36444715	A_23_P42353	0.64	0.65	1.66E-03	2.08E-03
NM_018357	LARP6	chr15:68911025-68910966	A_23_P117782	0.64	0.65	1.75E-02	2.31E-02
NM_032947	MST150	chr5:150156383-150156442	A_23_P414273	0.64	0.66	5.80E-03	6.06E-03
THC2383225	THC2383225	chr5:37286299-37286358	A_32_P202977	0.64	0.71	2.08E-03	8.79E-04
NM_012284	KCNH3	chr12:48237918-48237977	A_23_P87917	0.64	0.73	8.59E-06	6.22E-07
NM_005688	ABCC5	chr3:185120503-185120444	A_23_P258221	0.64	0.73	3.00E-04	5.18E-05
ENST00000321662	ENST00000321662	chr14:52172332-52172391	A_23_P140316	0.64	0.77	2.42E-06	3.99E-08
NM_000891	KCNJ2	chr17:65687611-65687670	A_23_P329261	0.64	0.77	4.25E-03	7.73E-04
NM_000274	OAT	chr10:126076306-126076247	A_23_P98092	0.64	0.82	5.49E-03	5.24E-04
NM_007253	CYP4F8	chr19:15600621-15601029	A_23_P131060	0.64	0.85	6.79E-04	1.22E-05
NM_052871	MGC4677	chr2:87660005-87660064	A_24_P273143	0.64	0.87	1.22E-02	8.86E-04
NM_014262	LEPREL2	chr12:6819014-6819073	A_23_P87752	0.64	0.89	3.53E-02	4.65E-03
NM_014571	HEYL	chr1:39758993-39758934	A_23_P430658	0.64	0.91	1.06E-02	4.52E-04
NM_001805	CEBPE	chr14:22656726-22656667	A_23_P2990	0.64	0.94	2.74E-02	1.55E-03
NM_005398	PPP1R3C	chr10:93378269-93378210	A_23_P35414	0.64	1.00	2.90E-04	7.76E-08
NM_006898	HOXD3	chr2:176863234-176863293	A_23_P323180	0.64	1.01	3.87E-06	4.83E-11
NM_080677	DYNLL2	chr17:53521746-53521805	A_23_P54991	0.64	1.01	3.70E-03	1.09E-05
NM_000185	SERPIND1	chr22:19466274-19466333	A_23_P6335	0.64	1.16	6.24E-05	7.37E-11
NM_002185	IL7R	chr5:35912538-35912597	A_23_P404494	0.64	1.18	4.27E-03	5.84E-07
BC032822	EPB41L5	chr2:120578165-120578224	A_23_P209298	0.64	1.27	3.43E-03	5.57E-08
NM_014504	RABGEF1	chr7:65718292-65718351	A_24_P232049	0.65	0.60	1.80E-03	5.54E-03
ENST00000265341	ENST00000265341	chr5:134091122-134091181	A_23_P394605	0.65	0.60	6.64E-03	1.74E-02
NM_021732	AVPI1	chr10:99427341-99427282	A_23_P1492	0.65	0.62	3.44E-04	7.84E-04
NM_001828	CLC	chr19:44913900-44913841	A_23_P101683	0.65	0.63	1.88E-10	3.53E-10
NM_005819	STX6	chr1:177674245-177674186	A_24_P639505	0.65	0.64	5.30E-03	8.17E-03
NM_001013002	HERC6	chr4:89721215-89721274	A_23_P250353	0.65	0.65	8.30E-06	1.00E-05
NM_153341	IBRDC3	chr1:33076979-33073197	A_23_P321388	0.65	0.68	4.83E-04	4.12E-04
NM_001730	KLF5	chr13:72548980-72549039	A_23_P53891	0.65	0.68	1.32E-03	1.07E-03
NM_002502	NFKB2	chr10:104151043-104151219	A_23_P202156	0.65	0.70	1.36E-02	1.20E-02
NM_003225	TFF1	chr21:42655541-42655482	A_23_P68759	0.65	0.72	1.08E-07	1.08E-08
NM_173854	SLC41A1	chr1:202490091-202490032	A_24_P122732	0.65	0.72	4.60E-06	5.56E-07
NM_004155	SERPINB9	chr6:2840633-2838737	A_32_P38323	0.65	0.72	2.66E-05	4.59E-06
NM_002502	NFKB2	chr10:104151043-104151219	A_23_P202156	0.65	0.73	6.27E-03	3.10E-03
A_24_P332263	A_24_P332263	chr6:159313103-159313042	A_24_P332263	0.65	0.76	3.36E-05	2.62E-06
NM_031886	KCNA7	chr19:54262638-54262579	A_23_P164897	0.65	0.77	3.63E-07	5.37E-09
BC036236	BC036236	chr3:183204371-183204430	A_24_P497524	0.65	0.77	5.83E-06	2.00E-07
NM_182901	C11orf17	chr11:8898321-8898380	A_23_P52161	0.65	0.79	5.10E-03	9.23E-04
NM_000121	EPOR	chr19:11349666-11349607	A_23_P367899	0.65	0.81	1.84E-03	1.72E-04
NM_002499	NEO1	chr15:71382673-71382732	A_23_P14798	0.65	0.84	2.81E-03	1.67E-04
NM_001145	ANG	chr14:20232101-20232160	A_23_P428738	0.65	0.90	1.48E-03	1.92E-05
NM_014969	WDR47	chr1:109225434-109225375	A_23_P23748	0.65	0.92	1.58E-04	3.57E-07
NM_017712	PGPEP1	chr19:18338072-18338131	A_23_P218531	0.65	0.94	3.41E-03	3.68E-05
NM_014400	LYPD3	chr19:48659159-48657822	A_24_P48495	0.65	0.97	3.35E-02	1.81E-03
NM_004089	TSC22D3	chrX:106763912-106763853	A_23_P217688	0.65	0.98	7.79E-03	1.07E-04
NM_198194	STOM	chr9:121182681-121182622	A_23_P255263	0.65	1.12	1.56E-02	5.56E-05
NM_017572	MKNK2	chr19:1988572-1988513	A_23_P142310	0.66	0.61	1.76E-02	4.17E-02
NM_006000	TUBA1	chr2:219940634-219940575	A_23_P154065	0.66	0.65	1.90E-06	2.75E-06
NM_130776	XAGE3	chrX:52776901-52776842	A_23_P114349	0.66	0.65	3.16E-04	4.89E-04
NM_152832	FAM89B	chr11:65098176-65098235	A_23_P124962	0.66	0.69	2.18E-05	9.91E-06
NM_173086	KRT6E	chr12:51150591-51149918	A_23_P366936	0.66	0.71	2.11E-03	1.33E-03
NM_022365	DNAJC1	chr10:22088233-22088174	A_23_P127128	0.66	0.73	2.43E-02	1.89E-02

Gene	Symbol	Chromosomal position	Probe	siCASP8AP2.3	CASP8AP2.6	siCASP8AP2.3	CASP8AP2.6
				M	M	Q	Q
A_24_P358606	A_24_P358606	chr5:144589484-144589543	A_24_P358606	0.66	0.73	4.87E-02	3.87E-02
NM_032490	C14orf142	chr14:92739756-92739697	A_23_P99579	0.66	0.75	3.18E-04	7.00E-05
NM_182556	LOC283130	chr11:64899637-64899578	A_23_P422511	0.66	0.75	2.26E-03	6.67E-04
NM_007312	HYAL1	chr3:50312736-50312677	A_23_P69329	0.66	0.79	1.18E-04	5.27E-06
U68019	SMAD3	chr15:65270577-65270637	A_23_P359091	0.66	0.80	2.84E-06	4.40E-08
NM_004183	VMD2	chr11:61486857-61486916	A_23_P104996	0.66	0.80	8.19E-04	8.54E-05
NM_031477	YPEL3	chr16:30011607-30011548	A_23_P15108	0.66	0.80	1.99E-02	6.28E-03
NM_001856	COL16A1	chr1:31787327-31787268	A_23_P160318	0.66	0.81	4.32E-03	6.18E-04
NM_002987	CCL17	chr16:56007389-56007448	A_23_P26325	0.66	0.82	8.91E-09	1.57E-11
NM_152793	Ells1	chr7:29975551-29975610	A_32_P100439	0.66	0.84	2.18E-02	5.05E-03
NM_000660	TGFB1	chr19:46539642-46529982	A_24_P79054	0.66	1.01	8.94E-03	8.50E-05
NM_032369	HVCN1	chr12:109549526-109549467	A_23_P151166	0.66	1.04	4.35E-05	2.71E-09
NM_025236	RNF39	chr6:30146764-30146705	A_24_P314931	0.66	1.12	2.27E-10	1.60E-18
THC2343897	THC2343897	chr4:90998260-90998319	A_32_P59355	0.66	1.17	5.18E-04	1.09E-08
NM_002119	HLA-DOA	chr6:33080007-33079948	A_32_P356316	0.67	0.60	2.98E-07	3.67E-06
NM_001540	HSPB1	chr7:75578028-75578087	A_24_P86537	0.67	0.62	6.90E-03	1.79E-02
NM_013361	ZNF223	chr19:49263097-49263156	A_24_P99838	0.67	0.65	1.01E-03	1.73E-03
NM_019013	FAM64A	chr17:6294966-6295025	A_23_P49878	0.67	0.65	1.74E-03	3.25E-03
NM_007219	RNF24	chr20:3862467-3862408	A_24_P333019	0.67	0.66	6.38E-06	1.14E-05
NM_020311	CMKOR1	chr2:237272783-237272842	A_23_P131676	0.67	0.67	5.82E-03	8.19E-03
NM_182488	USP12	chr13:26541088-26541029	A_24_P237613	0.67	0.70	4.37E-02	4.62E-02
NM_013259	TAGLN3	chr3:113202366-113202425	A_23_P80817	0.67	0.74	4.16E-06	5.45E-07
NM_000639	FASLG	chr1:169367342-169367401	A_23_P369815	0.67	0.80	1.59E-02	5.41E-03
AK090969	KCNH6	chr17:58977750-58977809	A_23_P390984	0.67	0.81	1.17E-03	1.45E-04
NM_144606	FLCN	chr17:17065452-17065393	A_23_P414308	0.67	0.83	2.53E-02	7.99E-03
NM_001009555	SH3D19	chr4:152399263-152399204	A_23_P33364	0.67	0.84	3.41E-03	3.50E-04
NM_006365	C1orf61	chr1:153189960-153187423	A_32_P138032	0.67	0.84	3.71E-02	1.15E-02
NM_002502	NFKB2	chr10:104151043-104151219	A_23_P202156	0.67	0.85	2.60E-03	2.27E-04
NM_001799	CDK7	chr5:68608176-68608235	A_23_P133585	0.67	0.86	4.22E-03	3.50E-04
NM_002456	MUC1	chr1:151972799-151971725	A_23_P137856	0.67	0.86	1.32E-02	2.05E-03
ENST00000380487	ENST00000380487	chr12:47445030-47445700	A_32_P161994	0.67	0.90	8.52E-09	8.91E-13
NM_003571	BFSP2	chr3:134676639-134676699	A_23_P159316	0.67	0.97	1.37E-04	1.76E-07
AK025431	LOC253981	chr4:37434907-37434848	A_32_P116206	0.67	0.99	4.06E-02	3.27E-03
NM_178450	3-Mar	chr5:126234341-126234282	A_23_P321511	0.67	1.07	7.11E-07	1.72E-12
THC2285742	THC2285742	chr13:50555126-50555067	A_32_P24068	0.67	1.08	5.88E-04	1.62E-07
AK098629	AK098629	chr2:96902934-96902875	A_32_P70891	0.68	0.61	3.55E-04	1.49E-03
NM_006915	RP2	chrX:46469672-46469731	A_24_P134568	0.68	0.65	1.95E-02	3.54E-02
NM_024843	CYBRD1	chr2:172236621-172236680	A_24_P345451	0.68	0.68	8.37E-03	1.28E-02
NM_207355	POTE15	chr15:19331121-19331062	A_32_P16007	0.68	0.70	3.97E-04	3.49E-04
NM_201612	IKIP	chr12:97510006-97509947	A_23_P53467	0.68	0.72	7.39E-05	3.96E-05
BX648591	BX648591	chr12:39751284-39751343	A_23_P204541	0.68	0.76	4.51E-02	3.44E-02
NM_004428	EFNA1	chr1:151919840-151919899	A_23_P113005	0.68	0.78	3.07E-05	2.35E-06
NM_017998	C9orf40	chr9:74791611-74791552	A_23_P43425	0.68	0.81	5.42E-05	2.80E-06
AK023445	LOC253264	chr10:76791165-76791224	A_24_P589001	0.68	0.82	1.08E-02	2.85E-03
AK123722	IL10RB	chr21:33559869-33559810	A_24_P783122	0.68	0.83	5.02E-07	3.85E-09
A_24_P655888	A_24_P655888	chr8:134360730-134360671	A_24_P655888	0.68	0.84	1.41E-03	1.08E-04
NM_006293	TYRO3	chr15:39658551-39658610	A_23_P54517	0.68	0.88	5.58E-05	5.38E-07
NM_006007	ZFAND5	chr9:72205158-72205099	A_24_P278172	0.68	0.88	8.46E-03	9.08E-04
NM_004907	IER2	chr19:13126586-13126645	A_32_P36235	0.68	0.89	7.89E-04	2.21E-05
NM_033177	BAT4	chr6:31737919-31737860	A_23_P133923	0.68	0.90	2.78E-05	1.07E-07
NM_020376	PNPLA2	chr11:814896-814955	A_23_P10077	0.68	0.90	2.95E-03	1.16E-04
A_24_P316059	A_24_P316059	chr16:045163013-045163072	A_24_P316059	0.68	0.90	1.42E-02	1.80E-03
NM_003246	THBS1	chr15:37675202-37675261	A_23_P206212	0.68	0.95	5.37E-03	1.69E-04
NM_003370	VASP	chr19:50721578-50721637	A_23_P119102	0.68	0.95	4.45E-02	6.23E-03
NM_203424	IQCF2	chr3:51872377-51872436	A_32_P165933	0.68	1.00	2.48E-09	8.18E-15
ENST00000283760	ENST00000283760	chr15:20813911-20813970	A_24_P229669	0.68	1.00	4.47E-03	4.80E-05

Gene	Symbol	Chromosomal position	Probe	siCASP8AP2.3	CASP8AP2.6	siCASP8AP2.3	CASP8AP2.6
				M	M	Q	Q
NM_052913	KIAA1913	chr6:130805686-130805745	A_32_P40288	0.68	1.03	6.14E-03	6.85E-05
NM_033064	ATCAY	chr19:3878951-3879010	A_23_P101671	0.68	1.04	1.38E-05	6.22E-10
NM_003395	WNT9A	chr1:224415856-224415797	A_23_P378329	0.68	1.05	1.30E-02	2.08E-04
NM_182487	OLFML2A	chr9:124656574-124656633	A_24_P220485	0.68	1.05	4.89E-02	3.06E-03
NM_183376	ARRDC4	chr15:96317899-96317958	A_23_P339818	0.68	1.08	1.30E-02	1.08E-04
NM_000743	CHRNA3	chr15:76675956-76675897	A_23_P253911	0.68	1.14	2.23E-02	1.91E-04
NM_014432	IL20RA	chr6:137362949-137362890	A_23_P145514	0.68	1.18	2.71E-03	6.75E-07
AF086126	AF086126	chr16:21999976-22000035	A_32_P97506	0.68	1.23	4.22E-02	2.83E-04
NM_018646	TRPV6	chr7:142086176-142086117	A_23_P71170	0.68	1.30	3.25E-02	6.93E-05
NM_006300	ZNF230	chr19:49206778-49206837	A_24_P370096	0.69	0.60	4.50E-03	1.92E-02
NM_000682	ADRA2B	chr2:96200835-96200776	A_23_P378926	0.69	0.61	1.42E-09	3.92E-08
NM_002618	PEX13	chr2:61187500-61187559	A_23_P257131	0.69	0.61	3.16E-05	2.66E-04
AK001072	FRMD4A	chr10:13775778-13775719	A_23_P22352	0.69	0.61	4.83E-03	1.83E-02
NM_016603	C5orf5	chr5:137303915-137303856	A_24_P20700	0.69	0.69	1.27E-03	1.57E-03
NM_006329	FBLN5	chr14:91405893-91405834	A_23_P151805	0.69	0.70	5.50E-04	5.77E-04
BC066644	KIAA1949	chr6:30752315-30752256	A_23_P331479	0.69	0.70	2.20E-02	2.72E-02
NM_012117	CBX5	chr12:52926243-52926184	A_23_P2355	0.69	0.72	9.27E-04	6.59E-04
NM_022648	TNS1	chr2:218493171-218493112	A_23_P351724	0.69	0.73	2.11E-05	8.80E-06
NM_001031628	LOC57228	chr12:49926002-49925943	A_23_P64837	0.69	0.73	2.31E-02	2.14E-02
A_32_P69987	A_32_P69987	chr10:033659750-033659809	A_32_P69987	0.69	0.73	4.16E-02	4.47E-02
BQ017638	BQ017638	chr2:208456222-208456281	A_32_P207789	0.69	0.75	2.64E-03	1.35E-03
A_24_P862251	A_24_P862251	chr10:003350355-003350296	A_24_P862251	0.69	0.77	1.74E-02	1.09E-02
NM_177925	H2AFJ	chr12:14819082-14819141	A_23_P204277	0.69	0.80	3.92E-06	1.51E-07
NM_004226	STK17B	chr2:196827717-196827658	A_23_P154367	0.69	0.83	3.34E-04	2.52E-05
XM_930891	FAM7A3	chr15:30486084-30486025	A_24_P724886	0.69	0.85	1.29E-03	1.11E-04
NM_014045	LRP10	chr14:22416873-22416932	A_23_P205499	0.69	0.86	1.73E-03	1.54E-04
AK131277	B4GALNT3	chr12:542218-542277	A_32_P49748	0.69	0.87	3.82E-02	1.22E-02
NM_021170	HES4	chr1:974767-974708	A_23_P149448	0.69	0.90	2.10E-02	3.22E-03
NM_001756	SERPINA6	chr14:93840442-93840383	A_23_P117363	0.69	0.93	1.52E-02	1.51E-03
NM_014476	PDLIM3	chr4:186798411-186798352	A_23_P110403	0.69	1.08	1.92E-02	3.96E-04
BC034487	HIST1H2AK	chr6:27911365-27911306	A_24_P940441	0.69	1.09	4.65E-04	1.54E-07
NM_152913	TMEM130	chr7:98088865-98088806	A_23_P349966	0.69	1.10	2.33E-04	3.76E-08
ENST00000382579	ENST00000382579	chr4:6795480-6795539	A_23_P428992	0.69	1.16	1.37E-03	3.51E-07
ENST00000334770	ENST00000334770	chr19:1486394-1486453	A_32_P50223	0.69	1.26	2.22E-03	1.35E-07
NM_006725	CD6	chr11:60544064-60544123	A_23_P311875	0.69	1.27	1.43E-02	1.22E-05
NM_004114	FGF13	chrX:137439467-137439408	A_23_P217319	0.69	1.40	3.67E-02	3.45E-05
NM_030769	NPL	chr1:179529679-179529738	A_23_P381431	0.70	0.63	1.51E-07	1.75E-06
THC2382022	THC2382022	chr17:12204806-12204865	A_32_P211885	0.70	0.64	1.20E-11	1.70E-10
NM_000089	COL1A2	chr7:93703294-93703353	A_24_P277934	0.70	0.64	3.51E-03	1.16E-02
AK024850	C2orf31	chr2:208453350-208453291	A_23_P108437	0.70	0.69	2.79E-05	3.91E-05
ENST00000331736	ENST00000331736	chr3:75488930-75488871	A_24_P341606	0.70	0.73	1.35E-02	1.36E-02
BX100535	BX100535	chr4:26537793-26537734	A_32_P107717	0.70	0.77	5.42E-06	9.54E-07
NM_003257	TJP1	chr15:27781176-27781117	A_23_P205828	0.70	0.77	2.22E-03	9.42E-04
NM_032831	TMEM142B	chr7:101673219-101673278	A_24_P337867	0.70	0.79	1.52E-04	2.88E-05
NM_001004320	FLJ16237	chr7:15178465-15178406	A_23_P136116	0.70	0.81	6.47E-09	9.32E-11
NM_002118	HLA-DMB	chr6:33010541-33010482	A_32_P351968	0.70	0.87	8.04E-04	4.61E-05
THC2455389	THC2455389	chr16:53443118-53443059	A_32_P113462	0.70	0.91	1.50E-04	1.74E-06
NM_016605	FAM53C	chr5:137712897-137712956	A_23_P167789	0.70	0.91	1.93E-04	2.93E-06
NM_014143	CD274	chr9:5458310-5458369	A_23_P338479	0.70	0.92	8.24E-04	1.80E-05
NR_002226	INGX	chrX:70494925-70494866	A_23_P125686	0.70	0.98	2.32E-07	2.71E-11
NM_006007	ZFAND5	chr9:72198958-72198928	A_24_P290354	0.70	0.99	5.87E-03	1.53E-04
NM_000185	SERPIND1	chr22:19466274-19466333	A_23_P6335	0.70	1.21	3.54E-08	1.10E-15
NM_001800	CDKN2D	chr19:10538285-10538226	A_23_P89941	0.70	1.32	1.09E-03	1.32E-08
NM_004288	PSCDBP	chr2:158097734-158097675	A_23_P90626	0.71	0.60	6.25E-11	7.62E-09
NM_017984	ZCWPW1	chr7:99643214-99643155	A_23_P70897	0.71	0.70	2.73E-04	4.18E-04
NM_000089	COL1A2	chr7:93703294-93703353	A_24_P277934	0.71	0.70	7.17E-04	1.14E-03

Gene	Symbol	Chromosomal position	Probe	siCASP8AP2.3	CASP8AP2.6	siCASP8AP2.3	CASP8AP2.6
				M	M	Q	Q
NM_203370	C3orf54	chr3:49817292-49817351	A_23_P158470	0.71	0.74	3.18E-04	2.43E-04
NM_024534	FLJ12684	chr4:53450900-53450841	A_23_P92583	0.71	0.76	5.95E-03	4.78E-03
NM_002286	LAG3	chr12:6757342-6757723	A_23_P116942	0.71	0.77	4.02E-05	1.06E-05
NM_025165	ELL3	chr15:41852501-41852442	A_23_P3237	0.71	0.79	6.14E-05	1.24E-05
NM_001976	ENO3	chr17:4800873-4801020	A_23_P130149	0.71	0.83	8.72E-03	2.99E-03
ENST00000371030	ENST00000371030	chr20:57267384-57267443	A_32_P206479	0.71	0.88	1.62E-02	3.97E-03
NM_003246	THBS1	chr15:37675202-37675261	A_23_P206212	0.71	0.90	2.21E-03	1.70E-04
NM_002983	CCL3	chr17:31440062-31440004	A_23_P373017	0.71	1.06	7.94E-06	5.71E-10
CR626252	CR626252	chr2:232090701-232090760	A_24_P20292	0.71	1.06	4.46E-03	3.41E-05
NM_139048	SMARCA3	chr3:150276408-150274817	A_24_P248251	0.71	1.07	1.45E-08	4.99E-14
NM_021077	NMB	chr15:82999465-82999406	A_23_P88522	0.71	1.07	1.96E-02	6.29E-04
BC035260	BC035260	chr12:52664049-52663990	A_32_P59308	0.71	1.09	1.68E-02	3.10E-04
NM_002279	KRT33B	chr17:36773645-36773586	A_23_P89665	0.71	1.15	1.09E-04	5.13E-09
NM_000185	SERPIND1	chr22:19466274-19466333	A_23_P6335	0.71	1.34	9.63E-07	9.13E-15
THC2408967	THC2408967	chr3:127111414-127111473	A_32_P141488	0.72	0.60	1.28E-06	3.86E-05
NM_003115	UAP1	chr1:159291820-159291879	A_23_P160460	0.72	0.62	3.47E-03	1.59E-02
NM_152405	JMY	chr5:78653565-78653624	A_32_P176550	0.72	0.70	5.70E-04	1.16E-03
NM_006832	PLEKHC1	chr14:52394992-52394933	A_23_P88347	0.72	0.72	1.54E-03	1.81E-03
BC016993	LOC147645	chr19:56526830-56526771	A_23_P101246	0.72	0.76	2.23E-03	1.73E-03
NM_001860	SLC31A2	chr9:113005794-113005853	A_23_P217109	0.72	0.76	2.84E-02	3.01E-02
NM_001514	GTF2B	chr1:89031022-89030963	A_23_P34628	0.72	0.77	1.06E-05	3.20E-06
NM_030775	WNT5B	chr12:1626062-1626121	A_23_P53588	0.72	0.81	1.58E-02	9.04E-03
NM_000543	SMPD1	chr11:6372012-6372071	A_23_P203488	0.72	0.82	8.17E-03	3.52E-03
NM_001014979	LOC90835	chr16:30678189-30678036	A_24_P842006	0.72	0.87	2.01E-05	6.22E-07
NM_003246	THBS1	chr15:37675202-37675261	A_23_P206212	0.72	0.88	2.11E-03	2.15E-04
NM_020856	TSHZ3	chr19:36457778-36457719	A_23_P361014	0.72	0.91	3.29E-03	3.09E-04
NM_003246	THBS1	chr15:37675202-37675261	A_23_P206212	0.72	0.93	9.27E-04	3.50E-05
NM_006670	TPBG	chr6:83132780-83132839	A_23_P59261	0.72	0.95	1.70E-02	2.24E-03
NM_198219	ING1	chr13:110170119-110170178	A_24_P39211	0.72	0.96	2.39E-04	2.01E-06
NM_032352	BRMS1L	chr14:35410130-35410189	A_24_P56484	0.72	0.97	7.64E-04	1.14E-05
NM_152426	APOBEC3D	chr22:37746121-37746180	A_23_P369966	0.72	1.04	2.84E-02	2.05E-03
NM_133498	SPACA4	chr19:53802410-53802469	A_23_P101573	0.72	1.06	3.21E-04	3.41E-07
NM_032413	C15orf48	chr15:43512587-43512646	A_23_P26024	0.72	1.17	4.14E-05	8.84E-10
NM_001012423	GOLGA8E	chr15:20999671-20999730	A_32_P103669	0.72	1.24	5.45E-04	2.56E-08
NM_012249	RHOQ	chr2:46719936-46719995	A_32_P83915	0.73	0.61	2.53E-03	1.48E-02
NM_000632	ITGAM	chr16:31251317-31251376	A_23_P124108	0.73	0.68	1.89E-03	4.91E-03
BC040902	PRAMEF2	chr1:12790793-12790852	A_24_P657487	0.73	0.69	1.97E-06	7.32E-06
NM_004415	DSP	chr6:7531581-7531640	A_32_P157945	0.73	0.70	4.99E-03	9.91E-03
AK124946	DPF3	chr14:72197630-72197571	A_24_P270424	0.73	0.72	1.09E-09	1.59E-09
AF318321	SLC16A3	chr17:77802811-77802870	A_24_P213134	0.73	0.87	4.47E-03	9.05E-04
A_24_P178167	A_24_P178167	chrX:048847063-048847004	A_24_P178167	0.73	0.88	2.69E-02	1.08E-02
NM_020836	KIAA1446	chr14:100073298-100073240	A_23_P72127	0.73	0.93	2.03E-03	1.18E-04
THC2280849	THC2280849	chr10:3097871-3097812	A_32_P211414	0.73	1.02	1.46E-03	1.56E-05
NM_152243	CDC42EP1	chr22:36289280-36289339	A_23_P166453	0.73	1.04	3.28E-02	2.96E-03
NM_133493	CD109	chr6:74590038-74590097	A_23_P214176	0.73	1.05	1.63E-03	1.25E-05
NM_006875	PIM2	chrX:48527078-48527019	A_24_P379104	0.73	1.08	2.97E-03	2.03E-05
NM_001343	DAB2	chr5:39408973-39408914	A_23_P257871	0.73	1.10	4.11E-05	8.10E-09
NM_032793	MFSD2	chr1:40103186-40103336	A_23_P43820	0.73	1.12	7.60E-06	3.13E-10
NM_000185	SERPIND1	chr22:19466274-19466333	A_23_P6335	0.73	1.13	2.10E-10	4.57E-17
AK124426	AK124426	chr17:59470405-59470346	A_24_P296689	0.73	1.18	7.00E-03	2.50E-05
NM_032682	FOXP1	chr3:71091157-71091098	A_23_P155257	0.73	1.18	2.02E-02	2.82E-04
NM_152280	SYT11	chr1:152665343-152665402	A_24_P139899	0.73	1.23	1.53E-04	4.22E-09
NM_003785	PAGE1	chrX:49162340-49161775	A_24_P314337	0.73	1.34	4.25E-05	2.29E-11
NM_014901	RNF44	chr5:175886856-175886797	A_23_P213592	0.74	0.60	8.61E-05	1.77E-03
NM_025080	ASRGL1	chr11:61881079-61881138	A_23_P1936	0.74	0.60	6.26E-03	3.79E-02
NM_019015	CSGlcA-T	chr7:150373426-150373485	A_32_P54442	0.74	0.62	6.29E-03	3.20E-02

Gene	Symbol	Chromosomal position	Probe	siCASP8AP2.3	CASP8AP2.6	siCASP8AP2.3	CASP8AP2.6
				M	M	Q	Q
A_32_P211765	A_32_P211765	chr11:067460847-067447769	A_32_P211765	0.74	0.65	5.21E-06	5.92E-05
NM_020812	DOCK6	chr19:11172405-11172100	A_23_P67299	0.74	0.66	1.05E-06	1.20E-05
NM_000719	CACNA1C	chr12:2671453-2671512	A_23_P373031	0.74	0.67	1.48E-04	6.22E-04
ENST00000331406	ENST00000331406	chr8:67687720-67687661	A_24_P911928	0.74	0.68	5.64E-03	1.52E-02
NM_001040	SHBG	chr17:7476860-7476919	A_23_P207089	0.74	0.69	6.35E-05	2.02E-04
AK055659	TSPAN5	chr4:99748832-99748773	A_23_P61886	0.74	0.88	7.11E-03	1.97E-03
NM_032199	ARID5B	chr10:63487052-63489035	A_24_P170667	0.74	0.90	3.57E-02	1.46E-02
NM_005928	MFGE8	chr15:87243767-87243708	A_24_P133584	0.74	0.92	3.66E-04	1.41E-05
NM_004594	SLC9A5	chr16:65863516-65863575	A_23_P129433	0.74	1.02	7.55E-05	1.64E-07
BC018691	GPR157	chr1:9121591-9121532	A_24_P71280	0.74	1.03	4.27E-03	9.86E-05
NM_005314	GRPR	chrX:15930708-15930767	A_24_P357266	0.74	1.05	3.00E-06	7.63E-10
NM_025107	MYCT1	chr6:153135466-153135525	A_23_P31046	0.74	1.06	9.27E-04	4.75E-06
NR_001443	LOC339240	chr17:20363327-20363268	A_32_P46238	0.74	1.13	6.68E-05	1.08E-08
NM_016217	HECA	chr6:139543338-139543397	A_23_P59349	0.74	1.20	7.66E-04	2.02E-07
NM_003485	GPR68	chr14:90768741-90768682	A_24_P931443	0.74	1.32	9.12E-07	4.94E-14
NM_001781	CD69	chr12:9796587-9796528	A_23_P87879	0.75	0.64	1.17E-08	6.91E-07
NM_030924	ACSBG2	chr19:6143982-6144041	A_23_P131050	0.75	0.66	2.00E-09	6.45E-08
NM_014387	LAT	chr16:28909489-28909548	A_23_P44105	0.75	0.66	4.98E-04	2.92E-03
NM_019013	FAM64A	chr17:6294966-6295025	A_23_P49878	0.75	0.70	3.38E-04	9.40E-04
NM_147780	CTSB	chr8:11739345-11739286	A_23_P215944	0.75	0.70	7.16E-03	1.59E-02
NM_019013	FAM64A	chr17:6294966-6295025	A_23_P49878	0.75	0.71	5.31E-04	1.45E-03
NM_001882	CRHBP	chr5:76300405-76300464	A_23_P30567	0.75	0.74	3.91E-10	5.05E-10
NM_006340	BAIAP2	chr17:76693079-76694500	A_24_P159648	0.75	0.77	3.70E-04	3.73E-04
NM_024490	ATP10A	chr15:23476185-23476126	A_23_P65659	0.75	0.82	7.51E-11	4.62E-12
NM_080875	MIB2	chr1_random:1263396-1263635	A_24_P259328	0.75	0.83	1.46E-03	5.46E-04
NM_001009812	LBX2	chr2:74636622-74636563	A_23_P79458	0.75	0.83	2.21E-03	9.61E-04
NM_198098	AQP1	chr7:30737999-30738058	A_23_P19894	0.75	0.85	2.85E-08	9.62E-10
NM_018318	CCDC91	chr12:28494416-28494571	A_24_P372959	0.75	0.85	3.79E-02	2.52E-02
NM_012104	BACE1	chr11:116661771-116661712	A_23_P52806	0.75	0.86	2.23E-04	3.82E-05
NM_171828	KCNMB3	chr3:180451268-180445160	A_24_P251661	0.75	0.87	1.43E-03	3.17E-04
NM_025160	WDR26	chr1:220880130-220880071	A_24_P36868	0.75	0.89	2.09E-03	3.92E-04
NM_005583	LYL1	chr19:13071418-13071359	A_23_P5281	0.75	0.89	9.34E-03	2.84E-03
NM_025106	SPSB1	chr1:9363761-9363820	A_24_P96961	0.75	0.90	3.01E-06	5.83E-08
NM_153487	MDGA1	chr6:37719611-37714411	A_24_P314640	0.75	0.90	1.18E-05	2.92E-07
THC2283468	THC2283468	chr7:22673881-22673940	A_24_P707102	0.75	0.93	3.60E-03	4.68E-04
NM_020184	CNNM4	chr2:96899355-96899414	A_23_P119923	0.75	1.01	1.20E-03	2.29E-05
NM_144573	NEXN	chr1:78110997-78111056	A_24_P409971	0.75	1.07	1.93E-02	1.10E-03
AA639753	AA639753	chr20:42494741-42494682	A_32_P201107	0.75	1.07	3.35E-02	3.11E-03
NM_152911	PAOX	chr10:135093958-135094017	A_23_P385771	0.75	1.08	2.93E-03	3.17E-05
NM_032321	MGC13057	chr2:190890001-190890060	A_23_P17130	0.75	1.11	5.85E-04	1.02E-06
A_23_P42595	A_23_P42595	chr7:150282864-150282805	A_23_P42595	0.75	1.13	7.67E-05	2.21E-08
NM_003483	HMGA2	chr12:64518599-64643300	A_24_P85099	0.75	1.29	6.66E-03	7.03E-06
NM_006634	VAMP5	chr2:85730611-85731755	A_23_P39840	0.76	0.62	6.25E-03	3.80E-02
THC2284306	THC2284306	chr12:88436484-88436543	A_32_P63324	0.76	0.71	2.49E-03	6.46E-03
NM_004414	DSCR1	chr21:34810938-34810879	A_23_P166248	0.76	0.73	2.34E-03	4.83E-03
NM_004362	CLGN	chr4:141668039-141667980	A_23_P18684	0.76	0.75	2.51E-05	4.11E-05
A_23_P111766	A_23_P111766	chr7:141947142-141947200	A_23_P111766	0.76	0.77	4.28E-03	5.33E-03
NM_000245	MET	chr7:116032241-116032300	A_23_P359245	0.76	0.77	3.72E-02	4.83E-02
NM_003842	TNFRSF10B	chr8:22933780-22933721	A_24_P218265	0.76	0.78	8.20E-05	6.83E-05
NM_019600	KIAA1370	chr15:50661709-50661650	A_24_P357576	0.76	0.79	2.33E-03	2.10E-03
NM_172140	IL29	chr19:44480931-44480990	A_23_P337800	0.76	0.80	3.38E-10	6.14E-11
ENST00000380456	ENST00000380456	chr19:11365932-11365873	A_23_P107903	0.76	0.82	2.74E-03	1.82E-03
NM_178518	TMEM102	chr17:7281350-7281409	A_32_P112380	0.76	0.83	8.27E-04	3.46E-04
NM_000639	FASLG	chr1:169367342-169367401	A_23_P369815	0.76	0.84	8.79E-03	5.08E-03
NM_001009813	MEIS3	chr19:52609798-52604570	A_23_P78795	0.76	0.85	4.79E-02	3.62E-02
NM_016323	HERC5	chr4:89784067-89784126	A_23_P110196	0.76	0.88	1.00E-03	2.25E-04

Gene	Symbol	Chromosomal position	Probe	siCASP8AP2.3	CASP8AP2.6	siCASP8AP2.3	CASP8AP2.6
				M	M	Q	Q
NM_198490	RAB43	chr3:130289195-130289136	A_32_P205859	0.76	0.90	2.56E-02	1.12E-02
AK090405	AK090405	chr19:5030088-5030147	A_24_P932648	0.76	0.96	2.48E-03	2.01E-04
NM_006106	YAP1	chr11:101605794-101605853	A_23_P104762	0.76	1.00	1.82E-05	6.40E-08
NM_032409	PINK1	chr1:20722631-20722690	A_23_P23194	0.76	1.03	3.03E-03	1.02E-04
ENST00000379019	ENST00000379019	chr20:5473220-5473161	A_23_P91350	0.77	0.66	6.06E-03	2.65E-02
NM_001613	ACTA2	chr10:90687938-90687879	A_23_P150053	0.77	0.66	1.09E-02	4.30E-02
NM_033106	GALP	chr19:61388676-61388735	A_23_P39171	0.77	0.67	2.84E-10	1.35E-08
NM_006763	BTG2	chr1:200010111-200010170	A_23_P62901	0.77	0.68	1.67E-03	7.15E-03
NM_005851	CDK2AP2	chr11:67031430-67031019	A_23_P104579	0.77	0.69	3.03E-05	2.03E-04
AF307332	MGEA5	chr10:103546881-103546822	A_24_P67898	0.77	0.69	1.82E-02	4.62E-02
NM_182903	KIF9	chr3:47291879-47291820	A_23_P212307	0.77	0.70	2.92E-03	9.66E-03
NM_174981	ANKRD21	chr21:13925192-13925251	A_23_P420348	0.77	0.71	1.22E-04	3.98E-04
NM_014504	RABGEF1	chr7:65718433-65718492	A_23_P250825	0.77	0.71	1.20E-04	5.00E-04
NM_004418	DUSP2	chr2:96231168-96231109	A_23_P108842	0.77	0.76	7.22E-04	1.07E-03
NM_152670	FLJ25369	chr2:88667833-88667892	A_23_P432005	0.77	0.83	1.24E-12	8.13E-14
BC033253	KIAA1602	chr12:48471273-48471214	A_24_P177964	0.77	0.83	2.15E-04	8.45E-05
NM_207459	FLJ35767	chr17:77914834-77914893	A_23_P375165	0.77	0.93	6.38E-04	5.26E-05
NM_005723	TSPAN5	chr4:99750645-99750586	A_23_P323930	0.77	1.00	1.95E-02	3.18E-03
BC038749	BC038749	chr7:115444117-115444058	A_32_P139367	0.77	1.02	2.02E-05	6.49E-08
BC000922	LOC400684	chr19:037572948-037572889	A_24_P59569	0.77	1.02	4.94E-05	2.45E-07
A_32_P101420	A_32_P101420	chr9:136971007-136970948	A_32_P101420	0.77	1.09	1.01E-04	1.71E-07
NM_004848	C1orf38	chr1:27896605-27896664	A_24_P85775	0.77	1.18	1.38E-03	2.64E-06
NM_020686	ABAT	chr16:8780872-8780931	A_24_P330684	0.77	1.24	5.95E-05	1.99E-09
AF010315	TP53I11		A_23_P368028	0.77	1.30	1.93E-02	1.18E-04
NM_004566	PFKFB3	chr10:6314902-6314961	A_24_P111096	0.77	1.32	5.79E-09	8.97E-17
NM_001003793	RBMS3	chr3:30021323-30021382	A_32_P228618	0.77	1.67	1.58E-06	9.32E-17
NM_002291	LAMB1	chr7:107158421-107158362	A_23_P94030	0.78	0.61	9.34E-05	2.70E-03
NM_002418	MLN	chr6:33870511-33870452	A_23_P19523	0.78	0.71	9.55E-12	1.65E-10
NM_018665	DDX43	chr6:74183658-74183717	A_23_P156445	0.78	0.72	1.67E-07	1.07E-06
NM_018207	TRIM62	chr1:33280187-33280128	A_23_P112602	0.78	0.78	2.03E-03	2.74E-03
NM_000639	FASLG	chr1:169367342-169367401	A_23_P369815	0.78	0.80	5.87E-03	6.48E-03
NM_004947	DOCK3	chr3:51396278-51396337	A_24_P153643	0.78	0.81	6.50E-03	6.46E-03
ENST00000354619	ENST00000354619	chr1:232705583-232705524	A_23_P97218	0.78	0.83	6.49E-04	4.33E-04
NM_021202	TP53INP2	chr20:32763818-32763877	A_24_P357465	0.78	0.83	4.50E-03	3.14E-03
NM_000245	MET	chr7:116032241-116032300	A_23_P359245	0.78	0.86	3.71E-02	2.93E-02
NM_001321	CSRP2	chr12:75755081-75755022	A_23_P44724	0.78	0.90	4.88E-03	1.74E-03
NM_001015880	PAPSS2	chr10:89496934-89496993	A_24_P940166	0.78	0.91	2.79E-06	8.29E-08
AK090611	FAM122C	chrX:133670543-133670602	A_24_P674479	0.78	0.92	4.56E-03	1.22E-03
ENST00000304963	ENST00000304963	chr1:18976174-18976115	A_23_P418031	0.78	0.95	3.54E-04	2.23E-05
NM_138340	ABHD3	chr18:17490895-17490836	A_23_P305759	0.78	0.96	1.83E-05	2.76E-07
NM_014417	BBC3	chr19:52416073-52416014	A_23_P382775	0.78	0.96	1.07E-02	2.21E-03
ENST00000358162	ENST00000358162	chr3:75730943-75730884	A_24_P332711	0.78	0.98	3.57E-07	1.17E-09
NM_002163	IRF8	chr16:84512492-84512551	A_23_P332190	0.78	0.99	3.04E-09	1.98E-12
NM_000660	TGFB1	chr19:46539642-46529982	A_24_P79054	0.78	1.01	1.75E-03	8.50E-05
NM_006665	HPSE	chr4:84584562-84580569	A_23_P256107	0.78	1.06	7.34E-06	6.34E-09
NM_015689	DENND2A	chr7:139675035-139674976	A_24_P334726	0.78	1.07	3.25E-05	6.55E-08
NM_021101	CLDN1	chr3:191510726-191510667	A_24_P165949	0.78	1.07	5.66E-05	1.28E-07
NM_001002914	KCTD11	chr17:7198658-7198717	A_23_P354027	0.78	1.07	2.69E-02	3.20E-03
NM_031957	KRTAP1-5	chr17:36435893-36435834	A_23_P118842	0.78	1.22	1.91E-03	2.80E-06
NM_000185	SERPIND1	chr22:19466274-19466333	A_23_P6335	0.78	1.26	3.93E-07	4.89E-13
NM_000185	SERPIND1	chr22:19466274-19466333	A_23_P6335	0.78	1.29	7.32E-10	1.55E-17
NM_001945	HBEGF	chr5:139695751-139695692	A_23_P213944	0.78	1.47	2.29E-03	7.40E-08
NM_000185	SERPIND1	chr22:19466089-19466148	A_24_P40306	0.78	1.51	7.76E-08	3.99E-17
NM_017736	THUMPD1	chr16:20655940-20655881	A_24_P99371	0.79	0.69	1.16E-03	5.72E-03
NM_014844	KIAA0329	chr14:102038472-102038531	A_32_P355396	0.79	0.70	4.10E-06	4.41E-05
ENST00000302001	ENST00000302001	chr11:64347635-64347576	A_23_P64051	0.79	0.73	3.05E-04	1.11E-03

Gene	Symbol	Chromosomal position	Probe	siCASP8AP2.3	CASP8AP2.6	siCASP8AP2.3	CASP8AP2.6
				M	M	Q	Q
NR_002804	SIGLECP3	chr19:56368470-56368529	A_32_P128258	0.79	0.74	1.06E-10	5.89E-10
NM_032782	HAVCR2	chr5:156445955-156445896	A_24_P411561	0.79	0.75	6.13E-05	1.52E-04
NM_024608	NEIL1	chr15:73434426-73434485	A_23_P129157	0.79	0.77	1.32E-04	2.25E-04
NM_014314	DDX58	chr9:32447078-32447019	A_23_P20814	0.79	0.78	1.32E-03	1.81E-03
THC2341837	THC2341837	chr14:102316231-102316290	A_32_P6972	0.79	0.78	4.50E-03	7.52E-03
NM_003344	UBE2H	chr7:129068218-129068159	A_23_P215096	0.79	0.78	2.24E-02	3.34E-02
ENST00000367001	ENST00000367001	chr1:208133899-208133840	A_24_P937095	0.79	0.81	1.38E-05	8.76E-06
NM_052970	HSPA12B	chr20:3681399-3681458	A_23_P91334	0.79	0.90	3.95E-08	1.06E-09
NM_013246	CLCF1	chr11:66888698-66888639	A_23_P138760	0.79	0.97	2.44E-03	2.71E-04
NM_003740	KCNK5	chr6:39264858-39264799	A_23_P319423	0.79	1.01	4.85E-07	1.10E-09
A_24_P724807	A_24_P724807	chr22:015436421-015436362	A_24_P724807	0.79	1.01	5.47E-04	1.82E-05
NM_003246	THBS1	chr15:37675202-37675261	A_23_P206212	0.79	1.01	1.03E-03	4.28E-05
NM_000940	PON3	chr7:94634015-94633956	A_23_P215549	0.79	1.03	6.74E-08	4.38E-11
NM_005202	COL8A2	chr1:36230015-36229956	A_24_P365975	0.79	1.21	1.79E-03	3.78E-06
ENST00000335571	ENST00000335571	chr6:88230777-88230836	A_23_P385980	0.80	0.61	3.29E-08	1.38E-05
NM_013974	DDAH2	chr6:31802956-31802897	A_23_P19482	0.80	0.63	3.36E-03	3.06E-02
ENST00000312785	ENST00000312785	chr19:5509291-5509232	A_23_P39294	0.80	0.70	9.34E-03	3.40E-02
AK056822	LOC400960	chr2:70168893-70168834	A_24_P650482	0.80	0.72	1.26E-03	4.72E-03
NM_000800	FGF1	chr5:141955160-141955101	A_24_P111106	0.80	0.72	3.25E-03	1.10E-02
NM_005384	NFIL3	chr9:91251014-91250955	A_23_P32253	0.80	0.91	2.05E-04	3.61E-05
NM_002305	LGALS1	chr22:36400152-36400211	A_23_P166459	0.80	0.92	7.64E-03	3.21E-03
NM_004823	KCNK6	chr19:43510657-43510716	A_23_P50591	0.80	0.97	2.64E-04	1.39E-05
NM_031866	FZD8	chr10:35967443-35967384	A_23_P396858	0.80	0.97	1.38E-03	1.54E-04
NM_006746	SCML1	chrX:17531935-17531994	A_24_P108863	0.80	1.09	4.35E-05	9.69E-08
NM_147164	CNTFR	chr9:34541529-34541470	A_23_P9402	0.80	1.23	1.95E-08	3.04E-14
NM_002999	SDC4	chr20:43392555-43392496	A_24_P373976	0.80	1.24	1.75E-02	3.64E-04
NM_005356	LCK	chr1:32420525-32420584	A_23_P103361	0.80	1.25	1.25E-03	1.40E-06
NM_138813	ATP8B3	chr19:1733142-1733083	A_23_P79108	0.80	1.31	3.15E-02	6.01E-04
NM_206956	PRAME	chr22:21215615-21215556	A_24_P216361	0.80	1.37	1.97E-06	1.08E-12
NM_000750	CHRNB4	chr15:76704271-76704212	A_23_P332789	0.81	0.64	4.00E-04	6.74E-03
NM_006195	PBX3	chr9:125808401-125808460	A_23_P71821	0.81	0.65	4.35E-03	3.05E-02
NM_144576	COQ10A	chr12:54950691-54950750	A_23_P354798	0.81	0.66	4.96E-06	1.59E-04
NM_015480	PVRL3	chr3:112335430-112335489	A_23_P401547	0.81	0.68	4.24E-06	9.91E-05
NM_000176	NR3C1	chr5:142639542-142639483	A_24_P214754	0.81	0.70	5.75E-03	2.45E-02
AK122643	GPR39	chr2:133237310-133237369	A_23_P79155	0.81	0.73	1.63E-06	1.40E-05
NM_006695	RPIP8	chr17:39749317-39749376	A_24_P91094	0.81	0.73	9.49E-03	2.68E-02
NM_002891	RASGRF1	chr15:77069627-77064584	A_23_P3379	0.81	0.74	7.87E-04	2.86E-03
NM_004252	SLC9A3R1	chr17:70276798-70276860	A_23_P308519	0.81	0.75	3.24E-04	1.12E-03
NM_001006641	SLC25A25	chr9:127950989-127951048	A_24_P355267	0.81	0.75	1.07E-02	2.76E-02
ENST00000276938	ENST00000276938	chr9:19451168-19451109	A_24_P58037	0.81	0.76	5.24E-03	1.26E-02
A_32_P162443	A_32_P162443	chr20:049833580-049833639	A_32_P162443	0.81	0.77	8.69E-05	2.09E-04
NM_139284	LGI4	chr19:40307551-40307492	A_24_P310256	0.81	0.80	1.93E-06	2.54E-06
NM_004762	PSCD1	chr17:74181792-74181733	A_23_P83781	0.81	0.80	5.20E-03	7.57E-03
BC043381	GJE1	chr7:99171030-99170971	A_23_P59869	0.81	0.81	1.78E-07	1.61E-07
NM_018404	CENTA2	chr17:26310276-26310335	A_24_P373562	0.81	0.81	1.40E-03	2.04E-03
NM_004603	STX1A	chr7:72558268-72558209	A_23_P82420	0.81	0.84	3.21E-03	3.20E-03
NM_000639	FASLG	chr1:169367342-169367401	A_23_P369815	0.81	0.84	4.61E-03	4.63E-03
NM_152523	FLJ40432	chr2:208444517-208444576	A_23_P90634	0.81	0.84	5.75E-03	5.70E-03
NM_005545	ISLR	chr15:72256174-72256233	A_23_P3312	0.81	0.84	6.06E-03	5.92E-03
NM_002928	RGS16	chr1:179302834-179302775	A_23_P217845	0.81	0.89	4.74E-02	4.18E-02
NM_006770	MARCO	chr2:119468207-119468266	A_23_P101992	0.81	0.90	4.63E-09	2.63E-10
NM_033285	TP53INP1	chr8:96007929-96007870	A_23_P168882	0.81	0.92	3.86E-02	2.50E-02
NM_006989	RASA4	chr7:101722752-101722693	A_23_P168449	0.81	0.99	4.30E-10	9.43E-13
NM_000660	TGFB1	chr19:46539642-46529982	A_24_P79054	0.81	1.02	8.85E-04	4.45E-05
NM_004744	LRAT	chr4:156031390-156031449	A_32_P113066	0.81	1.10	1.36E-04	5.84E-07
M31157	M31157	chr12:28005883-28005824	A_24_P17031	0.81	1.11	9.11E-04	9.41E-06

Gene	Symbol	Chromosomal position	Probe	siCASP8AP2.3	CASP8AP2.6	siCASP8AP2.3	CASP8AP2.6
				M	M	Q	Q
NM_001008530	LGMN	chr14:92247769-92245924	A_23_P25994	0.81	1.13	3.69E-04	1.72E-06
ENST00000367073	ENST00000367073	chr6:159301696-159301755	A_23_P257497	0.81	1.15	1.24E-03	1.01E-05
A_23_P15226	A_23_P15226	chr16:031050682-031050632	A_23_P15226	0.81	1.17	4.31E-08	8.70E-13
NM_015104	KIAA0404	chr11:64419361-64419222	A_23_P361820	0.81	1.19	4.80E-04	1.07E-06
NM_022343	C9orf19	chr9:36153763-36153822	A_23_P414913	0.81	1.21	1.23E-02	2.69E-04
NM_001965	EGR4	chr2:73429874-73429816	A_23_P380318	0.81	1.23	1.17E-03	1.92E-06
NM_018555	ZNF331	chr19:58772793-58772852	A_23_P433690	0.81	1.24	4.99E-04	3.82E-07
NM_015714	G0S2	chr1:206237992-206238051	A_23_P74609	0.81	1.25	2.00E-02	4.98E-04
ENST00000354653	ENST00000354653	chr1:224954271-224954330	A_32_P443407	0.81	1.47	1.23E-05	3.82E-12
NM_033049	MUC13	chr3:126108005-126107946	A_23_P155236	0.81	1.47	9.55E-05	1.53E-10
NM_207332	ERICH1	chr8:613979-613920	A_23_P368366	0.82	0.63	2.05E-03	2.57E-02
NM_001633	AMBP	chr9:113903311-113902927	A_23_P256504	0.82	0.68	9.19E-11	1.51E-08
NM_003116	SPAG4	chr20:33671067-33672063	A_23_P132027	0.82	0.68	5.03E-03	2.89E-02
NM_014553	TFCP2L1	chr2:121694854-121694795	A_23_P5301	0.82	0.71	2.16E-04	1.53E-03
NM_152495	CNIH3	chr1:221234899-221234958	A_23_P384044	0.82	0.72	2.09E-03	9.40E-03
NM_014964	EPN2	chr17:19180507-19180566	A_23_P89310	0.82	0.76	1.07E-06	4.63E-06
BF129169	BF129169	chr16:2144861-2144802	A_24_P902313	0.82	0.77	1.98E-02	3.98E-02
ENST00000292140	ENST00000292140	chr19:48671174-48671115	A_32_P184488	0.82	0.82	2.43E-04	3.11E-04
NM_012234	RYBP	chr3:72509011-72508952	A_23_P305711	0.82	0.84	1.86E-03	1.90E-03
NM_032298	SYT3	chr19:55817232-55817173	A_23_P55917	0.82	0.85	1.03E-05	5.98E-06
NM_001031716	OBFC2A	chr2:192369344-192372220	A_24_P229531	0.82	0.86	1.00E-02	9.27E-03
THC2271920	THC2271920	chrX:148769753-148769812	A_24_P874052	0.82	0.87	7.54E-11	1.17E-11
NM_000245	MET	chr7:116032241-116032300	A_23_P359245	0.82	0.89	4.28E-02	3.71E-02
NM_000660	TGFB1	chr19:46539642-46529982	A_24_P79054	0.82	0.90	1.24E-03	5.70E-04
NM_001002919	LOC285016	chr2:269994-269935	A_24_P561341	0.82	0.92	6.04E-07	4.36E-08
NM_032871	TNFRSF19L	chr11:72784612-72784671	A_23_P47735	0.82	0.95	5.69E-05	5.33E-06
NM_172109	KCNQ2	chr20:61535581-61535522	A_23_P351204	0.82	0.98	9.92E-03	2.90E-03
NM_030926	ITM2C	chr2:231569387-231569446	A_24_P402690	0.82	1.02	1.70E-04	5.83E-06
NM_173647	RNF149	chr2:101351633-101351574	A_23_P120153	0.82	1.04	1.11E-06	4.74E-09
NM_000121	EPOR	chr19:11349535-11349476	A_23_P381954	0.82	1.08	2.88E-06	5.17E-09
NM_016233	PADI3	chr1:17355235-17355294	A_23_P126869	0.82	1.13	8.24E-03	3.69E-04
A_23_P370707	A_23_P370707	chr6:029965546-029966027	A_23_P370707	0.82	1.15	8.78E-03	3.91E-04
NM_002593	PCOLCE	chr7:99848718-99848777	A_23_P251499	0.82	1.18	7.86E-04	3.25E-06
NM_004369	COL6A3	chr2:238014681-238014644	A_23_P131614	0.83	0.64	7.81E-04	1.29E-02
NM_145313	RASGEF1A	chr10:43011972-43011706	A_32_P223140	0.83	0.65	1.24E-06	1.16E-04
NM_032331	MGC2408	chr3:185459125-185459184	A_23_P92261	0.83	0.66	6.10E-07	5.56E-05
NM_017633	FAM46A	chr6:82516307-82516248	A_23_P70660	0.83	0.66	4.85E-06	2.60E-04
NM_001031628	LOC57228	chr12:49925823-49925764	A_23_P318115	0.83	0.70	1.90E-03	1.33E-02
NM_003959	HIP1R	chr12:121872327-121872386	A_23_P398294	0.83	0.71	7.61E-06	1.34E-04
NM_015318	ARHGEF18	chr19:7443261-7443320	A_23_P50357	0.83	0.74	2.67E-05	2.03E-04
NM_004093	EFNB2	chr13:105941689-105941630	A_23_P428139	0.83	0.74	3.11E-05	2.19E-04
NM_014387	LAT	chr16:28909233-28909292	A_23_P44112	0.83	0.84	1.45E-03	1.72E-03
NM_152333	SLC25A29	chr14:99827300-99827241	A_23_P77048	0.83	0.88	5.39E-06	2.16E-06
NM_004603	STX1A	chr7:72558253-72558194	A_23_P168556	0.83	0.88	1.55E-03	1.07E-03
NM_033181	CNR1	chr6:88906760-88906701	A_23_P214208	0.83	0.91	6.46E-05	1.44E-05
NM_001012302	TMEM16J	chr11:408916-408783	A_24_P8109	0.83	0.92	2.04E-04	5.90E-05
NM_144658	DOCK11	chrX:117601834-117601893	A_23_P148584	0.83	0.93	3.56E-03	1.50E-03
NM_000245	MET	chr7:116032241-116032300	A_23_P359245	0.83	0.93	3.51E-02	2.49E-02
NM_019112	ABCA7	chr19:1015196-1015932	A_23_P39481	0.83	0.95	3.20E-03	1.03E-03
NR_002947	TCAM1	chr17:59295400-59295459	A_24_P68088	0.83	1.05	1.02E-03	4.97E-05
NM_005931	MICB	chr6:31586812-31586871	A_23_P387471	0.83	1.10	2.97E-03	1.18E-04
NM_001560	IL13RA1	chrX:117707985-117708044	A_23_P137196	0.83	1.11	4.54E-03	2.16E-04
NM_006086	TUBB3	chr16:88529938-88529997	A_23_P77493	0.83	1.15	5.93E-03	2.19E-04
X66087	MYBL1	chr8:67637319-67637260	A_23_P43157	0.83	1.18	7.88E-03	2.28E-04
NM_001013642	LOC388610	chr1:27010643-27010702	A_24_P734953	0.83	1.50	1.66E-04	5.79E-10
NM_006072	CCL26	chr7:75043692-75043633	A_24_P12573	0.83	1.59	1.03E-02	2.29E-06

Gene	Symbol	Chromosomal position	Probe	siCASP8AP2.3	CASP8AP2.6	siCASP8AP2.3	CASP8AP2.6
				M	M	Q	Q
AK124788	GGTL3	chr20:32912521-32912462	A_23_P370027	0.84	0.60	4.92E-06	1.08E-03
NM_018964	SLC37A1	chr21:42874134-42874192	A_23_P17695	0.84	0.66	2.27E-04	4.28E-03
NM_033215	PPP1R3F	chrX:48900173-48900232	A_24_P177604	0.84	0.67	1.53E-06	9.94E-05
NM_000089	COL1A2	chr7:93703294-93703353	A_24_P277934	0.84	0.68	3.08E-04	4.64E-03
CN364769	CN364769	chr18:59186083-59186142	A_32_P55344	0.84	0.76	1.34E-06	1.14E-05
NM_003159	CDKL5	chrX:18397657-18397716	A_24_P282343	0.84	0.79	6.65E-04	1.66E-03
NM_005979	S100A13	chr1:150404417-150404358	A_23_P372874	0.84	0.81	8.78E-05	1.73E-04
NM_000660	TGFB1	chr19:46539642-46529982	A_24_P79054	0.84	0.86	8.77E-04	9.01E-04
ENST00000380739	ENST00000380739	chr6:2777714-2777655	A_24_P269315	0.84	0.87	1.13E-04	9.49E-05
NM_000660	TGFB1	chr19:46539642-46529982	A_24_P79054	0.84	0.94	6.31E-04	1.92E-04
NM_002344	LTK	chr15:39583276-39583217	A_23_P14853	0.84	0.95	8.39E-03	4.00E-03
NM_206894	MGC62100	chr19:42002642-42002583	A_24_P919279	0.84	1.02	9.70E-03	2.35E-03
NM_198833	SERPINB8	chr18:59805517-59805576	A_23_P4561	0.84	1.10	7.35E-03	6.69E-04
NM_003246	THBS1	chr15:37676123-37676182	A_24_P142118	0.84	1.24	1.03E-02	2.40E-04
NM_003799	RNMT	chr18:13732604-13736230	A_23_P55515	0.84	1.25	3.51E-05	7.39E-09
AK000872	AK000872	chr15:94613015-94613005	A_24_P795371	0.84	1.26	1.22E-03	2.70E-06
AK000085	AK000085	chr22:16935021-16934962	A_32_P91273	0.84	1.33	1.57E-07	2.87E-13
NM_000735	CGA	chr6:87852206-87852147	A_23_P42386	0.84	1.37	1.74E-07	8.31E-14
NM_080725	SRXN1	chr20:575622-575563	A_23_P320113	0.85	0.60	2.98E-09	1.13E-05
BC042539	LOC442421	chr9:64182223-64182282	A_23_P43549	0.85	0.67	1.04E-04	2.52E-03
NM_006423	RABAC1	chr19:47152741-47152682	A_23_P38864	0.85	0.74	2.47E-11	1.59E-09
NM_007161	LST1	chr6:31664372-31664431	A_24_P94916	0.85	0.77	1.51E-09	2.65E-08
NM_006000	TUBA1	chr2:219940711-219940652	A_23_P102109	0.85	0.79	4.58E-07	2.25E-06
NM_001793	CDH3	chr16:67290313-67290372	A_23_P49155	0.85	0.79	6.00E-04	2.02E-03
NM_000639	FASLG	chr1:169367342-169367401	A_23_P369815	0.85	0.82	3.66E-03	7.22E-03
U14391	MYO1E	chr15:57216200-57216139	A_23_P37497	0.85	0.85	6.23E-03	8.28E-03
A_24_P365349	A_24_P365349	chr19:059138711-059138770	A_24_P365349	0.85	0.89	8.13E-07	2.67E-07
NM_000602	SERPINE1	chr7:100374990-100375049	A_24_P158089	0.85	0.89	2.00E-02	1.98E-02
NM_000660	TGFB1	chr19:46539642-46529982	A_24_P79054	0.85	0.90	4.36E-04	2.61E-04
NM_000660	TGFB1	chr19:46539642-46529982	A_24_P79054	0.85	0.90	6.16E-04	3.70E-04
AK026328	FLJ22675	chr11:66500845-66500904	A_32_P524904	0.85	1.04	1.37E-08	4.64E-11
NM_178453	MGC52282	chr16:2829826-2829767	A_23_P350719	0.85	1.05	1.45E-10	1.32E-13
NM_002476	MYL4	chr17:42654813-42655879	A_24_P188218	0.85	1.06	1.43E-13	2.61E-17
NM_019848	SLC10A3	chrX:153279425-153279366	A_23_P366254	0.85	1.06	4.83E-04	2.11E-05
NM_004419	DUSP5	chr10:112260890-112260949	A_23_P150018	0.85	1.06	4.36E-02	1.58E-02
NM_030935	TSC22D4	chr7:99709845-99709403	A_23_P250866	0.85	1.10	9.93E-03	1.25E-03
NM_004405	DLX2	chr2:172789906-172789847	A_23_P28595	0.85	1.12	3.85E-07	3.46E-10
NM_194284	CLDN23	chr8:8598823-8598882	A_23_P134854	0.85	1.16	5.71E-03	2.27E-04
NM_005356	LCK	chr1:32420525-32420584	A_23_P103361	0.85	1.27	4.26E-04	4.27E-07
NM_006498	LGALS2	chr22:36290829-36290770	A_23_P120902	0.86	0.62	1.33E-11	1.49E-07
NM_000689	ALDH1A1	chr9:72745749-72745690	A_23_P83098	0.86	0.67	1.31E-03	1.63E-02
NM_001611	ACP5	chr19:11546584-11546525	A_23_P142075	0.86	0.69	8.23E-03	4.74E-02
NM_005187	CBFA2T3	chr16:87468908-87468849	A_23_P500741	0.86	0.72	3.28E-05	4.77E-04
NM_007314	ABL2	chr1:175808556-175808497	A_23_P138099	0.86	0.78	1.76E-02	4.53E-02
NM_016061	YPEL5	chr2:30294779-30294838	A_23_P108835	0.86	0.81	4.95E-03	1.18E-02
NM_000639	FASLG	chr1:169367342-169367401	A_23_P369815	0.86	0.85	3.13E-03	4.92E-03
AK095564	AK095564	chr1:19406181-19406240	A_24_P912228	0.86	0.88	1.94E-03	2.02E-03
NM_000602	SERPINE1	chr7:100374990-100375049	A_24_P158089	0.86	0.89	1.82E-02	2.17E-02
NM_033092	TRIM5	chr11:5643866-5643807	A_23_P356526	0.86	0.90	2.38E-05	1.29E-05
NM_022036	GPRC5C	chr17:69948481-69948540	A_32_P109029	0.86	0.92	3.78E-02	3.58E-02
NM_000433	NCF2	chr1:180256441-180256382	A_23_P138194	0.86	0.97	9.93E-03	5.23E-03
NM_000602	SERPINE1	chr7:100374990-100375049	A_24_P158089	0.86	0.99	1.89E-02	9.37E-03
NM_004838	HOMER3	chr19:18901086-18901027	A_23_P39364	0.86	1.01	4.05E-04	4.78E-05
NM_000639	FASLG	chr1:169367342-169367401	A_23_P369815	0.86	1.02	2.97E-03	6.50E-04
NM_005971	FXYD3	chr19:40303839-40305528	A_24_P293192	0.86	1.13	8.46E-03	7.21E-04
NM_006460	HEXIM1	chr17:40583698-40583757	A_23_P118552	0.86	1.14	4.33E-03	2.11E-04

Gene	Symbol	Chromosomal position	Probe	siCASP8AP2.3	CASP8AP2.6	siCASP8AP2.3	CASP8AP2.6
				M	M	Q	Q
NM_000185	SERPIND1	chr22:19466274-19466333	A_23_P6335	0.86	1.33	8.21E-15	1.45E-22
NM_033316	MFI2	chr3:198234240-198234181	A_23_P386320	0.86	1.40	1.84E-02	1.70E-04
ENST00000367245	ENST00000367245	chr6:153137107-153137166	A_24_P945059	0.86	1.41	1.14E-05	5.73E-11
THC2437757	THC2437757	chr9:114626600-114626541	A_32_P158181	0.86	1.58	2.64E-03	1.78E-07
BC043547	BC043547	chr8:6687279-6687338	A_32_P145769	0.87	0.65	2.63E-10	4.92E-07
NM_001338	CXADR	chr21:17860528-17860587	A_32_P29632	0.87	0.70	5.40E-03	3.53E-02
NM_001002915	IGFL2	chr19:51356199-51356258	A_23_P153571	0.87	0.85	3.73E-11	5.81E-11
NM_198511	LANCL3	chrX:37291309-37291368	A_32_P57292	0.87	0.88	1.25E-03	1.46E-03
ENST00000373268	ENST00000373268	chr2:242752815-242752874	A_32_P180971	0.87	0.92	1.54E-02	1.45E-02
NM_000894	LHB	chr19:54211276-54211217	A_23_P218505	0.87	0.93	7.15E-03	5.76E-03
NM_000660	TGFB1	chr19:46539642-46529982	A_24_P79054	0.87	0.96	5.31E-04	1.71E-04
NM_006007	ZFAND5	chr9:72203970-72203911	A_32_P109817	0.87	0.96	2.04E-03	9.26E-04
AF218008	C19orf28	chr19:3489383-3489324	A_23_P218523	0.87	0.97	1.25E-03	4.34E-04
NM_138461	TM4SF19	chr3:197539480-197539148	A_23_P372946	0.87	1.00	2.38E-02	1.27E-02
BM129308	BM129308	chr8:23755810-23755869	A_32_P192842	0.87	1.04	8.37E-10	3.07E-12
NM_033297	NALP12	chr19:58989179-58989120	A_23_P101434	0.87	1.05	1.23E-06	1.42E-08
AF086301	MGC24039	chr12:31426992-31426933	A_24_P541919	0.87	1.05	3.10E-05	9.39E-07
NR_002797	LOC255783	chr19:52470504-52470563	A_23_P386168	0.87	1.05	6.34E-05	2.32E-06
NM_004192	ASMTL	chrX:1581782-1581723	A_23_P159544	0.87	1.05	3.25E-03	5.23E-04
BC015442	LOC200383	chr2:84836535-84836594	A_23_P376014	0.87	1.17	3.51E-12	7.30E-17
NM_003970	MYOM2	chr8:2080477-2080536	A_23_P258912	0.87	1.20	5.56E-15	1.39E-20
NM_003468	FZD5	chr2:208456903-208456844	A_23_P397999	0.87	1.27	2.00E-04	2.10E-07
NM_005099	ADAMTS4	chr1:157972778-157972719	A_23_P360754	0.87	1.41	2.84E-07	2.36E-13
NM_024336	IRX3	chr16:52874790-52874731	A_23_P152235	0.87	1.42	5.59E-03	1.42E-05
NM_153488	MAGEA2B	chrX:151557232-151557291	A_23_P148255	0.87	1.45	9.62E-08	1.90E-14
NM_175744	RHOC	chr1:112955940-112955881	A_23_P12514	0.88	0.67	2.49E-03	2.87E-02
NM_178026	GGTL3	chr20:32896486-32896427	A_23_P325093	0.88	0.69	2.96E-03	2.66E-02
NM_005335	HCLS1	chr3:122833026-122832969	A_23_P73429	0.88	0.70	4.08E-05	1.21E-03
NM_004760	STK17A	chr7:43437613-43437672	A_24_P337796	0.88	0.75	1.71E-05	2.60E-04
NM_002770	PRSS2	chr7:141988987-141989046	A_32_P94444	0.88	0.78	2.32E-03	9.23E-03
NM_153006	NAGS	chr17:39441850-39441909	A_23_P406187	0.88	0.78	1.20E-02	3.90E-02
ENST00000367808	ENST00000367808	chr1:166088567-166088626	A_23_P23266	0.88	0.79	3.90E-05	2.48E-04
NM_080752	ZSWIM3	chr20:43940742-43940801	A_23_P165984	0.88	0.86	2.13E-08	3.92E-08
NM_018169	C12orf35	chr12:32037031-32037090	A_23_P98930	0.88	0.86	1.24E-02	1.98E-02
NM_006454	MXD4	chr4:2221384-2220164	A_23_P259490	0.88	0.88	2.16E-04	2.48E-04
NM_006528	TFPI2	chr7:93161285-93161226	A_23_P393620	0.88	0.90	4.58E-09	3.07E-09
NM_005421	TAL2	chr9:105504465-105504524	A_23_P409449	0.88	0.90	7.65E-08	5.59E-08
NM_004345	CAMP	chr3:48241860-48241919	A_23_P253791	0.88	0.90	2.99E-07	1.91E-07
NM_018948	ERRFI1	chr1:8007792-8007733	A_24_P11384	0.88	0.90	9.51E-05	8.15E-05
NM_000602	SERPINE1	chr7:100374990-100375049	A_24_P158089	0.88	0.90	1.74E-02	2.15E-02
NM_001031716	OBFC2A	chr2:192377061-192377120	A_23_P329198	0.88	0.92	6.05E-03	5.92E-03
NM_002334	LRP4	chr11:46835184-46835125	A_24_P403561	0.88	0.99	2.17E-04	4.91E-05
NM_015149	RGL1	chr1:180628899-180628958	A_23_P115417	0.88	0.99	1.51E-02	8.71E-03
THC2401542	THC2401542	chr19:13903224-13903165	A_23_P208567	0.88	1.01	1.39E-04	1.85E-05
THC2276324	THC2276324	chr12:54433239-54433298	A_24_P227350	0.88	1.02	7.36E-06	4.21E-07
NM_007352	ELA3B	chr1:22058471-22061108	A_23_P200579	0.88	1.03	3.81E-07	7.98E-09
NM_020904	PLEKHA4	chr19:54032227-54032168	A_24_P408047	0.88	1.05	3.41E-04	3.24E-05
NM_172006	WFDC10B	chr20:43746994-43746935	A_24_P7790	0.88	1.06	2.57E-03	4.35E-04
NM_138440	VASN	chr16:4373422-4373481	A_23_P129695	0.88	1.11	1.34E-03	7.04E-05
THC2415124	THC2415124	chr1:46693828-46693887	A_32_P220591	0.88	1.12	4.00E-05	3.72E-07
NM_015667	C9orf36	chr9:40880530-40880471	A_23_P435874	0.88	1.13	8.34E-10	2.81E-13
AK057922	CDH24	chr14:22590749-22590690	A_23_P405267	0.88	1.15	4.47E-05	2.53E-07
NM_019600	KIAA1370	chr15:50661450-50661391	A_23_P99853	0.88	1.18	1.53E-05	3.95E-08
NM_003933	BAIAP3	chr16:1338880-1338939	A_23_P163492	0.88	1.19	4.80E-05	1.59E-07
NM_001945	HBEGF	chr5:139693295-139693236	A_24_P140608	0.88	1.27	9.04E-03	2.45E-04
NM_005365	MAGEA9	chrX:148574783-148574842	A_32_P29965	0.88	1.32	1.07E-09	1.74E-15

Gene	Symbol	Chromosomal position	Probe	siCASP8AP2.3	CASP8AP2.6	siCASP8AP2.3	CASP8AP2.6
				M	M	Q	Q
NM_012068	ATF5	chr19:55128566-55128625	A_23_P119337	0.88	1.34	4.31E-03	2.89E-05
NM_021209	CARD12	chr2:32361413-32361354	A_23_P119835	0.88	1.37	4.17E-07	1.93E-12
NM_175884	FLJ36031	chr7:105893998-105893939	A_23_P406616	0.88	1.38	9.14E-04	8.19E-07
U78519	U78519	chr18:13653574-13653515	A_24_P898945	0.88	1.41	6.46E-05	2.61E-09
NM_004497	FOXA3	chr19:51068021-51068080	A_23_P142174	0.88	1.45	4.60E-03	7.84E-06
NM_001012974	C6orf154	chr6:43583008-43582949	A_24_P737939	0.89	0.71	1.56E-06	1.11E-04
NM_052884	SIGLEC11	chr19:55144427-55144368	A_23_P119322	0.89	0.73	6.27E-10	1.35E-07
NM_031421	TTC25	chr17:37371050-37371109	A_23_P73150	0.89	0.77	2.22E-04	1.66E-03
BC029107	FMN1	chr15:30845552-30845493	A_32_P180825	0.89	0.78	2.15E-04	1.49E-03
NM_182540	DDX26B	chrX:134441541-134441600	A_23_P363647	0.89	0.86	2.18E-03	3.86E-03
NM_004827	ABCG2	chr4:89370664-89370605	A_23_P18713	0.89	0.89	2.00E-08	1.69E-08
AK096606	AK096606	chr17:76699020-76698961	A_24_P8151	0.89	0.89	1.93E-06	2.33E-06
NM_004354	CCNG2	chr4:78444874-78444933	A_23_P110122	0.89	0.97	9.16E-04	3.79E-04
NM_018203	KLHDC8A	chr1:202037404-202037345	A_24_P62800	0.89	0.98	2.88E-11	1.39E-12
NM_006465	ARID3B	chr15:72677430-72677489	A_23_P88580	0.89	1.03	7.77E-08	1.66E-09
NM_005356	LCK	chr1:32420525-32420584	A_23_P103361	0.89	1.05	7.94E-05	5.26E-06
NM_005356	LCK	chr1:32420525-32420584	A_23_P103361	0.89	1.12	4.51E-05	6.76E-07
NM_005356	LCK	chr1:32420525-32420584	A_23_P103361	0.89	1.12	5.56E-05	7.99E-07
NM_005356	LCK	chr1:32420525-32420584	A_23_P103361	0.89	1.15	5.46E-05	5.63E-07
NM_030935	TSC22D4	chr7:99716657-99716598	A_24_P200942	0.89	1.17	2.99E-03	1.52E-04
NM_005356	LCK	chr1:32420525-32420584	A_23_P103361	0.89	1.18	8.53E-05	4.73E-07
NM_015205	ATP11A	chr13:112588919-112588978	A_24_P161973	0.89	1.33	3.63E-04	4.17E-07
BC036557	KIAA1394	chr11:66949109-66949168	A_23_P344884	0.89	1.57	9.75E-07	8.71E-14
NM_001002233	RAB11FIP1	chr8:37839732-37839673	A_23_P391198	0.90	0.61	5.77E-06	1.99E-03
NM_016580	PCDH12	chr5:141309247-141309188	A_23_P423309	0.90	0.62	8.80E-05	7.97E-03
NM_005672	PSCA	chr8:143760978-143761037	A_23_P71379	0.90	0.65	5.26E-07	2.09E-04
NM_033196	ZNF682	chr19:19978991-19978932	A_23_P28012	0.90	0.72	3.77E-03	2.94E-02
NM_032222	FLJ22374	chr7:30649580-30652074	A_24_P126425	0.90	0.75	2.66E-06	6.65E-05
NM_005555	KRT6B	chr12:51126882-51126823	A_23_P76249	0.90	0.76	7.07E-07	1.79E-05
NM_173833	SCARA5	chr8:27783853-27783794	A_23_P94103	0.90	0.77	5.27E-14	1.28E-11
NM_152910	DGKH	chr13:41691855-41691914	A_23_P502980	0.90	0.78	2.15E-06	3.60E-05
NM_017870	TMEM132A	chr11:60461128-60461187	A_23_P24716	0.90	0.82	7.80E-07	6.15E-06
NM_000148	FUT1	chr19:53943137-53943080	A_23_P107963	0.90	0.85	5.46E-06	1.52E-05
NM_013227	AGC1	chr15:87218775-87218834	A_23_P307310	0.90	0.89	6.02E-09	7.43E-09
NM_003246	THBS1	chr15:37675202-37675261	A_23_P206212	0.90	0.90	3.51E-04	4.51E-04
NM_003706	PLA2G4C	chr19:53243119-53243060	A_23_P50508	0.90	0.92	4.70E-04	4.10E-04
NM_022823	FNDC4	chr2:27626973-27626914	A_23_P16834	0.90	0.93	3.33E-03	3.30E-03
NM_006887	ZFP36L2	chr2:43362866-43362807	A_23_P101960	0.90	0.95	1.23E-03	8.89E-04
THC2412698	THC2412698	chr14:44437998-44437939	A_32_P14457	0.90	0.95	3.32E-03	2.84E-03
NM_178834	LAYN	chr11:110936670-110936729	A_23_P127565	0.90	1.00	2.78E-09	1.65E-10
NM_005409	CXCL11	chr4:77313601-77313446	A_24_P20607	0.90	1.07	3.90E-10	2.04E-12
NM_032356	LSMD1	chr17:7701771-7701712	A_23_P38206	0.90	1.07	5.79E-07	8.44E-09
NM_021255	PELI2	chr14:55837219-55837278	A_23_P65532	0.90	1.12	5.37E-06	4.22E-08
NM_003344	UBE2H	chr7:129068720-129068661	A_23_P145584	0.90	1.15	6.53E-03	7.84E-04
NM_005356	LCK	chr1:32420525-32420584	A_23_P103361	0.90	1.17	6.70E-05	6.07E-07
NM_001485	GBX2	chr2:236856711-236856652	A_23_P131183	0.90	1.28	2.34E-05	1.20E-08
NM_000735	CGA	chr6:87852140-87852081	A_24_P371782	0.90	1.32	8.76E-11	1.32E-16
NM_153266	TMEM151	chr11:65820419-65820478	A_23_P354217	0.90	1.32	6.17E-03	1.05E-04
NM_153685	C12orf53	chr12:6673334-6673275	A_24_P248185	0.90	1.41	8.87E-06	1.80E-10
NM_001063	TF	chr3:134980247-134980306	A_23_P212500	0.90	1.45	4.70E-06	2.14E-11
BC004565	MGC12935	chr6:26380825-26380884	A_23_P253762	0.91	0.65	6.64E-06	1.42E-03
NM_004292	RIN1	chr11:65856474-65856415	A_23_P64102	0.91	0.69	4.22E-06	3.98E-04
NM_006454	MXD4	chr4:2217049-2216990	A_24_P234732	0.91	0.72	4.41E-05	1.34E-03
NM_024927	PLEKHH3	chr17:38073637-38073578	A_32_P172188	0.91	0.78	2.07E-03	1.14E-02
NM_033128	SCIN	chr7:12465583-12465642	A_23_P157136	0.91	0.86	5.05E-11	2.21E-10
NM_001039650	ZMYM5	chr13:19310841-19309904	A_24_P321634	0.91	0.87	6.10E-03	1.25E-02

Gene	Symbol	Chromosomal position	Probe	siCASP8AP2.3	CASP8AP2.6	siCASP8AP2.3	CASP8AP2.6
				M	M	Q	Q
NM_198472	C10orf125	chr10:135057626-135057567	A_23_P97952	0.91	0.91	4.93E-06	5.03E-06
NM_016081	PALLD	chr4:170223556-170223615	A_23_P213102	0.91	0.94	4.94E-05	3.52E-05
NM_198472	C10orf125	chr10:135058194-135058135	A_24_P186379	0.91	1.05	3.79E-06	1.92E-07
NM_001040100	C3orf57	chr3:162546396-162546337	A_32_P199551	0.91	1.09	4.04E-04	3.61E-05
NM_182517	C1orf210	chr1:43417649-43417590	A_32_P42946	0.91	1.11	3.21E-07	2.69E-09
NM_015687	FILIP1	chr6:76080807-76080748	A_24_P84668	0.91	1.18	3.84E-05	3.03E-07
NM_001874	CPM	chr12:67535674-67535615	A_24_P938352	0.91	1.25	1.33E-06	4.98E-10
NM_001912	CTSL	chr9:87574880-87574939	A_23_P94533	0.91	1.30	4.31E-04	1.23E-06
NM_017589	BTG4	chr11:110873981-110873334	A_23_P47322	0.91	1.45	1.91E-11	4.81E-19
NM_002229	JUNB	chr19:12763998-12764057	A_24_P241815	0.91	1.45	7.49E-04	3.09E-07
ENST00000369615	ENST00000369615	chr1:113939174-113939233	A_24_P118171	0.92	0.63	4.59E-05	5.89E-03
NM_003804	RIPK1	chr6:3058940-3058999	A_23_P370005	0.92	0.70	2.13E-05	1.36E-03
NM_005655	KLF10	chr8:103730598-103730533	A_23_P168828	0.92	0.71	1.16E-04	3.39E-03
NM_030762	BHLHB3	chr12:26164958-26164899	A_23_P139500	0.92	0.80	1.52E-04	1.21E-03
NM_002105	H2AFX	chr11:118469860-118469801	A_24_P38895	0.92	0.82	8.28E-06	6.13E-05
BC035647	BC035647	chr6:30004277-30004779	A_23_P373126	0.92	0.85	1.17E-05	5.76E-05
NM_012249	RHOQ	chr2:46715030-46715398	A_32_P49844	0.92	0.88	6.14E-04	1.37E-03
NM_000952	PTAFR	chr1:28160355-28160296	A_23_P51926	0.92	0.94	4.30E-05	4.10E-05
NM_004557	NOTCH4	chr6:32270661-32270602	A_23_P365614	0.92	0.97	3.80E-06	1.37E-06
ENST00000361989	ENST00000361989	chr15:43247357-43247298	A_32_P183904	0.92	0.98	5.23E-03	3.98E-03
NM_004823	KCNK6	chr19:43510837-43510896	A_24_P262543	0.92	0.99	1.05E-05	2.68E-06
NM_018676	THSD1	chr13:51849494-51849435	A_23_P14184	0.92	1.04	7.06E-03	3.23E-03
NM_006472	TXNIP	chr1:142931491-142931550	A_23_P97700	0.92	1.06	9.81E-03	4.23E-03
ENST00000344556	ENST00000344556	chr12:73344083-73344024	A_24_P834110	0.92	1.11	3.13E-04	1.92E-05
THC2455550	THC2455550	chr20:49833002-49832943	A_32_P191746	0.92	1.12	9.97E-05	4.20E-06
NM_022818	MAP1LC3B	chr16:85994259-85994318	A_32_P220715	0.92	1.12	1.03E-03	9.30E-05
NM_032487	ARPM1	chr3:170968055-170967996	A_23_P121447	0.92	1.13	5.14E-05	1.39E-06
XM_495873	LOC440040	chr11:49788348-49788407	A_23_P150198	0.92	1.46	5.99E-05	3.62E-09
NM_080740	SUHW1	chr22:21193329-21193270	A_23_P154972	0.92	1.53	3.64E-09	1.30E-16
THC2340639	THC2340639	chr19:7889808-7889867	A_32_P100464	0.93	0.66	6.38E-05	5.59E-03
NM_021260	ZFYVE1	chr14:72507403-72507344	A_24_P287503	0.93	0.70	9.00E-05	3.51E-03
NM_005391	PDK3	chrX:24305865-24305924	A_23_P250478	0.93	0.84	7.01E-05	3.72E-04
NM_057749	CCNE2	chr8:95963035-95962976	A_23_P215976	0.93	0.86	2.01E-02	4.34E-02
NM_000602	SERPINE1	chr7:100374990-100375049	A_24_P158089	0.93	0.87	1.41E-02	3.10E-02
NM_005657	TP53BP1	chr15:41487474-41487043	A_23_P88703	0.93	0.90	2.42E-05	5.12E-05
BC039374	BC039374	chr2:97777921-97777980	A_24_P456723	0.93	1.00	1.08E-04	3.72E-05
NM_002962	S100A5	chr1:150325720-150325661	A_23_P115467	0.93	1.01	1.13E-06	1.84E-07
NM_003508	FZD9	chr7:72294994-72295053	A_23_P59613	0.93	1.09	3.84E-05	2.45E-06
NM_002194	INPP1	chr2:191057070-191059336	A_32_P44453	0.93	1.11	1.51E-04	9.90E-06
BC030123	LOC441461	chr9:99728300-99728241	A_32_P177955	0.93	1.15	7.41E-07	3.85E-09
NM_001753	CAV1	chr7:115794693-115794752	A_23_P134454	0.93	1.15	2.79E-03	3.42E-04
NM_032871	TNFRSF19L	chr11:72785271-72785330	A_24_P38951	0.93	1.23	7.06E-08	2.29E-11
A_32_P153361	A_32_P153361	chr5:179827227-179827277	A_32_P153361	0.93	1.30	6.52E-05	1.03E-07
NM_005367	MAGEA12	chrX:151570539-151570480	A_23_P252928	0.93	1.32	7.77E-14	8.82E-20
NM_002632	PGF	chr14:74485827-74484995	A_23_P76992	0.93	1.52	1.13E-04	5.21E-09
NM_005409	CXCL11	chr4:77312853-77312794	A_23_P125278	0.93	1.56	1.24E-06	1.01E-12
NM_182603	ANKRD42	chr11:82616499-82616558	A_24_P357572	0.94	0.66	2.54E-07	1.82E-04
ENST00000261569	ENST00000261569	chr5:66498564-66498623	A_23_P110571	0.94	0.74	7.29E-03	4.90E-02
NM_015723	PNPLA8	chr7:107713596-107707029	A_23_P312718	0.94	0.78	1.08E-03	9.47E-03
NM_006270	RRAS	chr19:54830604-54830545	A_23_P39076	0.94	0.82	4.92E-03	1.99E-02
NM_170717	RASSF1	chr3:50342383-50342324	A_23_P41066	0.94	0.83	1.00E-03	4.72E-03
NM_006795	EHD1	chr11:64376799-64376759	A_23_P52647	0.94	0.87	1.43E-04	4.99E-04
BC027875	MYO15B	chr17:71133843-71133902	A_32_P475513	0.94	0.88	8.66E-06	3.09E-05
NM_005853	IRX5	chr16:53525227-53525286	A_24_P48057	0.94	0.92	5.56E-08	1.02E-07
NM_014068	PSORS1C1	chr6:31215710-31215769	A_23_P133902	0.94	0.92	3.52E-03	6.15E-03
ENST00000299903	ENST00000299903	chrX:103165738-103165797	A_24_P6825	0.94	0.99	1.48E-07	4.78E-08

Gene	Symbol	Chromosomal position	Probe	siCASP8AP2.3	CASP8AP2.6	siCASP8AP2.3	CASP8AP2.6
				M	M	Q	Q
NM_138284	IL17D	chr13:20194978-20195037	A_23_P345692	0.94	1.06	1.06E-07	5.36E-09
NM_000930	PLAT	chr8:42152028-42151969	A_23_P82868	0.94	1.11	3.87E-02	1.87E-02
NM_000270	NP	chr14:20014944-20015003	A_23_P140256	0.94	1.12	5.73E-07	8.57E-09
NM_005356	LCK	chr1:32420525-32420584	A_23_P103361	0.94	1.17	3.56E-05	7.01E-07
NM_025079	ZC3H12A	chr1:37619004-37619063	A_23_P326160	0.94	1.18	1.13E-05	1.05E-07
NM_017594	DIRAS2	chr9:90452534-90452475	A_23_P20660	0.94	1.22	7.19E-04	1.95E-05
NM_007075	WDR45	chrX:48689074-48688792	A_23_P251717	0.94	1.49	3.06E-06	2.45E-11
NM_024684	C11orf67	chr11:77258446-77258505	A_24_P49383	0.94	1.52	1.41E-06	4.22E-12
A_24_P229911	A_24_P229911	chr4:154938278-154938337	A_24_P229911	0.94	1.60	5.70E-07	1.19E-13
NM_000014	A2M	chr12:9112685-9112626	A_23_P116898	0.94	1.72	4.58E-03	7.68E-07
NM_001132	AFG3L1	chr16:88589894-88589953	A_23_P355289	0.95	0.68	3.79E-05	3.56E-03
A_23_P99731	A_23_P99731	chr14:052314624-052314566	A_23_P99731	0.95	0.76	9.49E-06	3.96E-04
NM_031205	CABP1	chr12:119561603-119561662	A_23_P411761	0.95	0.80	1.70E-06	4.83E-05
BX100088	BX100088	chr1:39904487-39904295	A_32_P124833	0.95	0.85	1.82E-04	1.04E-03
NM_031886	KCNA7	chr19:54262578-54262519	A_24_P87686	0.95	0.89	5.61E-07	2.32E-06
THC2282516	THC2282516	chr20:48709980-48710039	A_24_P744957	0.95	0.93	5.45E-10	8.36E-10
NM_000387	SLC25A20	chr3:48870026-48869967	A_23_P72025	0.95	0.93	5.71E-08	1.20E-07
NM_001902	CTH	chr1:70617062-70617121	A_23_P126103	0.95	0.99	5.39E-04	3.96E-04
NM_024768	CCDC48	chr3:130242193-130242252	A_23_P166566	0.95	1.01	6.81E-13	6.00E-14
NM_000930	PLAT	chr8:42152028-42151969	A_23_P82868	0.95	1.04	2.85E-02	2.34E-02
NM_022169	ABCG4	chr11:118538507-118538566	A_24_P355626	0.95	1.07	2.42E-06	1.96E-07
BC071773	BC071773	chr17_random:90724-90665	A_32_P7516	0.95	1.15	2.11E-03	2.84E-04
NM_005356	LCK	chr1:32420525-32420584	A_23_P103361	0.95	1.18	3.95E-05	7.37E-07
NM_020299	AKR1B10	chr7:133683054-133683113	A_24_P129341	0.95	1.23	1.94E-06	4.36E-09
NM_001003682	TTMB	chr1:29267313-29267254	A_32_P77098	0.95	1.70	1.70E-08	8.97E-17
NM_024588	CTA-216E10.6	chr22:40417903-40417962	A_23_P132341	0.96	0.61	2.07E-04	2.27E-02
NM_001132	AFG3L1	chr16:88590005-88590064	A_23_P404893	0.96	0.67	2.22E-05	3.22E-03
NM_018071	FLJ10357	chr14:20627451-20627510	A_23_P99661	0.96	0.67	2.21E-03	4.77E-02
AB058754	GMPPB	chr3:49729607-49729548	A_23_P306655	0.96	0.73	1.45E-05	1.04E-03
NM_002970	SAT	chrX:23563712-23563771	A_23_P137016	0.96	0.75	4.57E-03	3.83E-02
BM928667	BM928667	chr3:32914115-32914174	A_32_P221429	0.96	0.82	7.15E-07	1.74E-05
NM_001005505	CACNA2D2	chr3:50375530-50375471	A_23_P346900	0.96	0.83	1.13E-07	2.97E-06
NM_178275	DKFZp434B1231	chr1:197929426-197929485	A_32_P425998	0.96	0.85	2.96E-07	3.88E-06
NM_000962	PTGS1	chr9:122237090-122237149	A_24_P64167	0.96	0.86	1.18E-10	2.62E-09
NM_000245	MET	chr7:116032241-116032300	A_23_P359245	0.96	0.92	1.53E-02	2.84E-02
NM_021153	CDH19	chr18:62322865-62322806	A_23_P38735	0.96	0.98	2.19E-05	1.67E-05
NM_145313	RASGEF1A	chr10:43010046-43009987	A_23_P391344	0.96	1.00	4.25E-06	2.16E-06
NM_198129	LAMA3	chr18:19788733-19788792	A_23_P89780	0.96	1.00	1.17E-05	5.34E-06
NM_003471	KCNAB1	chr3:157738549-157738608	A_24_P182929	0.96	1.02	2.56E-19	1.68E-20
A_24_P110601	A_24_P110601	chr22:023433018-023433077	A_24_P110601	0.96	1.02	4.05E-02	4.06E-02
NM_138444	KCTD12	chr13:76355597-76355538	A_23_P377957	0.96	1.08	9.82E-03	4.90E-03
NM_018423	STYK1	chr12:10663180-10663121	A_23_P13822	0.96	1.28	7.92E-07	6.34E-10
NM_006840	LILRB5	chr19:59450634-59450575	A_23_P164784	0.96	1.32	9.53E-07	2.40E-10
NM_130830	LRRC15	chr3:195557859-195557800	A_24_P827037	0.96	1.37	5.93E-15	3.19E-21
NM_004900	APOBEC3B	chr22:37712038-37712097	A_24_P66027	0.96	1.38	1.89E-02	9.23E-04
AA180985	AA180985	chr19:049622283-049622341	A_24_P822427	0.96	1.46	1.03E-13	7.03E-21
ENST00000379884	ENST00000379884	chr14:106038205-106038146	A_32_P190951	0.97	0.68	8.51E-06	1.80E-03
NM_003657	BCAS1	chr20:51994940-51994881	A_23_P17420	0.97	0.79	1.35E-10	3.48E-08
NM_152657	GGN	chr19:43566949-43566890	A_23_P333552	0.97	0.86	1.16E-06	1.16E-05
NM_016269	LEF1	chr4:109326409-109326350	A_24_P20630	0.97	0.93	2.70E-04	5.38E-04
NM_001505	GPR30	chr7:906577-906636	A_23_P8640	0.97	0.94	1.38E-02	2.56E-02
NM_020435	GJA12	chr1:224654089-224654148	A_23_P51729	0.97	0.96	4.43E-06	6.14E-06
NM_001015053	HDAC5	chr17:39509749-39509690	A_24_P125283	0.97	1.00	3.46E-03	3.43E-03
NM_032496	ARHGAP9	chr12:56152489-56152430	A_23_P64661	0.97	1.07	5.27E-09	3.26E-10
NM_021217	ZNF77	chr19:2885392-2885333	A_32_P182394	0.97	1.08	2.51E-02	1.73E-02
NM_144652	LETM2	chr8:38385127-38385186	A_23_P348264	0.97	1.09	2.94E-04	7.06E-05

Gene	Symbol	Chromosomal position	Probe	siCASP8AP2.3	CASP8AP2.6	siCASP8AP2.3	CASP8AP2.6
				M	M	Q	Q
NM_004029	IRF7	chr11:604820-604499	A_24_P118892	0.97	1.14	9.09E-06	3.72E-07
NM_018602	DNAJA4	chr15:76361405-76361464	A_23_P206140	0.97	1.19	8.46E-05	2.22E-06
NM_004143	CITED1	chrX:71305752-71305693	A_23_P73526	0.97	1.21	2.57E-05	3.44E-07
NM_172113	EYA2	chr20:45250503-45250562	A_23_P319859	0.97	1.29	4.90E-06	6.52E-09
NM_030812	ACTL8	chr1:17898696-17898755	A_32_P176018	0.97	1.41	1.27E-02	4.28E-04
ENST00000272035	ENST00000272035	chr10:82952-82893	A_32_P16315	0.97	1.60	3.96E-05	5.11E-10
NM_016427	TCEB3B	chr18:42813288-42813229	A_23_P141706	0.97	1.81	2.70E-05	6.38E-12
NM_004407	DMP1	chr4:88942463-88942522	A_23_P133153	0.98	0.62	1.21E-04	1.96E-02
ENST00000318291	ENST00000318291	chr12:624759-624816	A_23_P431569	0.98	0.69	3.62E-05	4.27E-03
NM_024778	LONRF3	chrX:117933562-117933621	A_23_P114414	0.98	0.78	4.84E-06	2.50E-04
NM_004970	IGFALS	chr16:1780515-1780456	A_23_P14892	0.98	0.79	3.96E-11	2.03E-08
NM_014505	KCNMB4	chr12:69110890-69110949	A_23_P64792	0.98	0.79	5.01E-06	1.99E-04
ENST00000264554	ENST00000264554	chr19:367892-367833	A_32_P205624	0.98	0.82	7.87E-03	3.86E-02
NM_207480	UNQ5830	chr2:10094402-10094343	A_24_P932646	0.98	0.83	8.31E-06	1.33E-04
NM_012413	QPCT	chr2:37511245-37511513	A_23_P16915	0.98	0.83	1.10E-02	4.43E-02
NM_000958	PTGER4	chr5:40729320-40729379	A_23_P148047	0.98	0.87	1.90E-07	2.93E-06
NM_145804	ABTB2	chr11:34129377-34129318	A_23_P356616	0.98	0.90	3.06E-04	1.15E-03
NM_000602	SERPINE1	chr7:100374990-100375049	A_24_P158089	0.98	0.91	1.21E-02	2.80E-02
NM_003971	SPAG9	chr17:46398154-46398095	A_24_P365025	0.98	0.92	1.22E-04	3.83E-04
NM_006741	PPP1R1A	chr12:53261040-53260430	A_23_P53417	0.98	0.95	3.37E-06	7.16E-06
NM_033339	CASP7	chr10:115480484-115480543	A_23_P12572	0.98	0.99	9.20E-06	9.14E-06
NM_001753	CAV1	chr7:115794693-115794752	A_23_P134454	0.98	1.07	2.02E-03	9.90E-04
NM_153344	C6orf141	chr6:49626843-49626902	A_32_P114483	0.98	1.09	6.47E-07	5.95E-08
NM_001753	CAV1	chr7:115794693-115794752	A_23_P134454	0.98	1.09	1.88E-03	7.09E-04
NM_207310	CCDC74B	chr2:130613192-130613133	A_23_P401718	0.98	1.16	2.59E-04	2.35E-05
THC2375957	THC2375957	chr16:087848904-087848845	A_32_P98776	0.98	1.21	7.01E-05	1.68E-06
NM_001103	ACTN2	chr1:233251984-233252043	A_23_P115021	0.98	1.46	8.40E-11	7.90E-17
A_32_P147603	A_32_P147603	chr9:129895363-129895304	A_32_P147603	0.99	0.63	1.38E-09	3.65E-05
NM_014646	LPIN2	chr18:2907332-2907273	A_24_P301557	0.99	0.80	5.10E-06	1.94E-04
ENST00000217537	ENST00000217537	chr18:72203284-72203225	A_23_P55606	0.99	0.81	6.51E-06	1.91E-04
NM_021114	SPINK2	chr4:57517140-57517081	A_23_P155688	0.99	0.90	8.38E-07	6.88E-06
NM_014007	ZBTB43	chr9:126675719-126675778	A_23_P135315	0.99	0.94	9.40E-03	1.84E-02
NM_006007	ZFAND5	chr9:72201410-72200522	A_24_P278167	0.99	0.98	3.11E-05	4.66E-05
NM_002425	MMP10	chr11:102146674-102146615	A_23_P13094	0.99	1.03	1.22E-08	4.31E-09
NM_012193	FZD4	chr11:86334507-86334448	A_23_P64617	0.99	1.06	5.43E-07	1.21E-07
NM_000930	PLAT	chr8:42152028-42151969	A_23_P82868	0.99	1.07	2.89E-02	2.47E-02
NM_013300	C12orf24	chr12:109388414-109390548	A_23_P65000	0.99	1.11	2.96E-04	6.91E-05
NM_001753	CAV1	chr7:115794693-115794752	A_23_P134454	0.99	1.20	8.98E-04	7.85E-05
NM_004881	TP53I3	chr2:24214007-24212266	A_23_P5392	0.99	1.21	2.12E-08	5.96E-11
NM_052972	LRG1	chr19:4488515-4488456	A_23_P50638	0.99	1.22	1.25E-07	4.83E-10
ENST00000244221	ENST00000244221	chr2:71328679-71327311	A_24_P860797	0.99	1.28	3.09E-03	1.98E-04
NM_002005	FES	chr15:89237927-89237986	A_23_P14769	0.99	1.33	5.42E-08	1.41E-11
NM_152506	C21orf129	chr21:42004835-42004776	A_23_P413693	0.99	1.37	3.58E-04	1.74E-06
NM_032521	PARD6B	chr20:48803184-48803243	A_32_P205637	0.99	1.41	3.45E-04	9.20E-07
CA945082	CA945082	chr11:67456438-67456379	A_32_P121704	1.00	0.64	2.19E-06	2.02E-03
NM_198540	B3GNT8	chr19:46623182-46623123	A_23_P101380	1.00	0.65	4.57E-05	1.01E-02
NM_005763	AASS	chr7:121311862-121310573	A_23_P8754	1.00	0.72	1.04E-03	2.41E-02
NM_138448	ACYP2	chr2:54277447-54277506	A_24_P336848	1.00	0.74	2.50E-04	8.19E-03
NM_213674	TPM2	chr9:35672147-35672088	A_23_P216501	1.00	0.76	3.39E-03	3.66E-02
NM_152304	RAB42	chr1:28741329-28741388	A_23_P434919	1.00	0.81	4.77E-03	3.04E-02
NM_138771	LOC90693	chr7:23456885-23456944	A_24_P354954	1.00	0.86	1.53E-07	4.70E-06
NM_000602	SERPINE1	chr7:100374990-100375049	A_24_P158089	1.00	0.89	1.06E-02	3.36E-02
NM_017723	FLJ20245	chr9:137452814-137452873	A_23_P147106	1.00	0.97	2.81E-02	4.76E-02
NM_002183	IL3RA	chrX:1515174-1518947	A_23_P253081	1.00	0.98	6.01E-09	1.04E-08
NM_020804	PACSIN1	chr6:34610721-34610780	A_24_P149266	1.00	1.03	1.38E-05	7.88E-06
NM_001753	CAV1	chr7:115794693-115794752	A_23_P134454	1.00	1.08	2.50E-03	1.44E-03

Gene	Symbol	Chromosomal position	Probe	siCASP8AP2.3	CASP8AP2.6	siCASP8AP2.3	CASP8AP2.6
				M	M	Q	Q
NM_006884	SHOX2	chr3:159297961-159297902	A_24_P300021	1.00	1.13	9.68E-07	6.50E-08
NM_004107	FCGRT	chr19:54721323-54721382	A_23_P55936	1.00	1.13	2.58E-04	5.37E-05
NM_024786	ZDHHC11	chr5:848845-848786	A_24_P153456	1.00	1.13	1.99E-03	6.56E-04
NM_024311	ET	chr17:72286080-72286139	A_23_P26704	1.00	1.17	6.62E-04	9.77E-05
NM_000434	NEU1	chr6:31935184-31935125	A_24_P394533	1.00	1.33	1.63E-06	1.83E-09
THC2373524	THC2373524	chr19:050103635-050103694	A_24_P788772	1.00	1.33	1.85E-02	2.38E-03
NM_001964	EGR1	chr5:137832790-137832849	A_23_P214080	1.00	1.36	1.38E-02	1.10E-03
NM_003613	CILP	chr15:63275726-63275667	A_23_P151895	1.00	1.42	1.56E-09	1.71E-14
NM_002994	CXCL5	chr4:75226992-75226933	A_24_P277367	1.00	1.72	6.72E-05	4.51E-10
NM_021229	NTN4	chr12:94554502-94554443	A_23_P204630	1.01	0.63	7.75E-05	1.82E-02
BC111002	ARSI	chr5:149656902-149656843	A_23_P19030	1.01	0.73	4.61E-05	3.93E-03
NM_004073	PLK3	chr1:44940687-44940747	A_23_P51646	1.01	0.78	5.25E-05	2.07E-03
A_24_P821146	A_24_P821146	chr6:147771038-147771097	A_24_P821146	1.01	0.85	6.48E-07	2.07E-05
BF972140	BF972140	chr3:130324989-130325048	A_32_P89432	1.01	0.85	3.56E-04	3.24E-03
NM_003660	PPFIA3	chr19:54345550-54345609	A_23_P320553	1.01	0.89	1.75E-06	2.10E-05
BX538015	DKFZP686E2158	chr5:60490808-60490749	A_24_P646185	1.01	0.91	6.65E-06	4.86E-05
NM_018393	TCP11L1	chr11:33050792-33050851	A_23_P47247	1.01	0.92	1.13E-08	1.39E-07
NM_031430	RILP	chr17:1496298-1496239	A_23_P26815	1.01	0.92	1.56E-04	6.49E-04
NM_002770	PRSS2	chr7:141988890-141988949	A_23_P310274	1.01	0.92	2.09E-04	9.21E-04
NM_145036	CCDC46	chr17:61115771-61115712	A_23_P83976	1.01	0.97	8.99E-07	2.23E-06
NM_000861	HRH1	chr3:11278801-11278860	A_24_P277211	1.01	1.12	2.27E-02	1.62E-02
NM_017622	C17orf59	chr17:8032906-8032847	A_23_P152963	1.01	1.16	1.17E-04	1.42E-05
NM_000930	PLAT	chr8:42152028-42151969	A_23_P82868	1.01	1.16	2.24E-02	1.24E-02
NM_002068	GNA15	chr19:3108749-3108808	A_24_P331128	1.01	1.22	6.73E-14	5.47E-17
NM_031476	CRISPLD2	chr16:83499639-83499698	A_23_P106602	1.01	1.24	2.04E-08	5.31E-11
NM_006674	HCP5	chr6:31540998-31541057	A_32_P85500	1.01	1.24	3.29E-07	2.04E-09
CA421238	CA421238	chr21:46376586-46376527	A_32_P22750	1.01	1.25	2.65E-02	8.32E-03
NM_002054	GCG	chr2:162825323-162825264	A_23_P254664	1.01	1.30	4.08E-05	3.13E-07
NM_033120	NKD2	chr5:1091719-1091778	A_23_P41804	1.01	1.30	2.84E-03	1.88E-04
BC007960	RAB3D	chr19:11293794-11293735	A_24_P236956	1.01	1.48	1.65E-03	8.32E-06
NM_145341	PDCD4	chr10:112648109-112648168	A_23_P258862	1.01	1.49	2.17E-03	1.19E-05
NM_002522	NPTX1	chr17:76055671-76055612	A_23_P124905	1.01	1.53	4.42E-07	4.72E-12
THC2448178	THC2448178	chr21:44722698-44722757	A_32_P50587	1.02	0.73	1.55E-04	8.68E-03
NM_002970	SAT	chrX:23563123-23563182	A_23_P378722	1.02	0.73	4.16E-04	1.50E-02
NM_001747	CAPG	chr2:85533626-85533567	A_23_P165636	1.02	0.78	2.32E-03	2.80E-02
CR623273	CR623273	chr11:62190891-62190950	A_24_P804667	1.02	0.79	2.20E-06	1.83E-04
NM_178568	RTN4RL1	chr17:1785277-1785218	A_23_P66481	1.02	0.89	5.74E-05	5.33E-04
NM_001007245	IFRD1	chr7:111706302-111706776	A_23_P251825	1.02	0.89	3.77E-04	2.21E-03
NM_001753	CAV1	chr7:115794693-115794752	A_23_P134454	1.02	1.03	1.25E-03	1.49E-03
NM_000725	CACNB3	chr12:47508588-47508647	A_23_P204016	1.02	1.07	1.56E-03	1.10E-03
ENST00000379884	ENST00000379884	chr14:106038157-106038099	A_24_P860662	1.02	1.10	4.55E-12	3.16E-13
NM_001753	CAV1	chr7:115794693-115794752	A_23_P134454	1.02	1.12	1.29E-03	5.24E-04
NM_001753	CAV1	chr7:115794693-115794752	A_23_P134454	1.02	1.15	1.25E-03	3.82E-04
NM_000930	PLAT	chr8:42152028-42151969	A_23_P82868	1.02	1.15	2.25E-02	1.34E-02
NM_152270	SLFN11	chr17:30701469-30701426	A_23_P129925	1.02	1.34	9.89E-05	7.99E-07
ENST00000300590	ENST00000300590	chr16:49259528-49259469	A_23_P352469	1.02	1.45	2.35E-02	1.65E-03
NM_014598	SOCS7	chr17:33809349-33809408	A_32_P43711	1.03	0.64	1.16E-04	2.09E-02
NM_198173	GRHL3	chr1:24426840-24426899	A_23_P52067	1.03	0.75	1.43E-08	1.60E-05
AK057267	RSNL2	chr2:29309062-29309121	A_23_P417363	1.03	0.78	2.75E-03	3.18E-02
NM_000142	FGFR3	chr4:1776285-1776344	A_23_P212830	1.03	0.88	2.10E-07	6.59E-06
BC031632	KIAA1257	chr3:130189195-130179706	A_32_P163739	1.03	0.88	9.70E-05	1.04E-03
NM_002975	CLEC11A	chr19:55920489-55920548	A_23_P153489	1.03	0.90	3.26E-07	5.98E-06
NM_000559	HBG1	chr11:5226169-5226110	A_23_P53137	1.03	0.99	3.62E-07	8.75E-07
NM_000660	TGFB1	chr19:46539642-46529982	A_24_P79054	1.03	1.02	6.88E-04	9.63E-04
NM_000785	CYP27B1	chr12:56442902-56442843	A_23_P36397	1.03	1.03	2.11E-09	2.17E-09
NM_052998	ADC	chr1:33255005-33255064	A_24_P11462	1.03	1.12	3.86E-10	3.04E-11

Gene	Symbol	Chromosomal position	Probe	siCASP8AP2.3	CASP8AP2.6	siCASP8AP2.3	CASP8AP2.6
				M	M	Q	Q
NM_016006	ABHD5	chr3:43735035-43735094	A_23_P250294	1.03	1.19	4.45E-09	6.14E-11
NM_181873	MTMR11	chr1:146713689-146713630	A_23_P51986	1.03	1.23	1.39E-02	4.45E-03
NM_144717	FNDC6	chr3:138212496-138212555	A_23_P91850	1.03	1.29	7.11E-06	5.30E-08
ENST00000280576	ENST00000280576	chr12:122944826-122944885	A_32_P228268	1.03	1.40	1.71E-07	4.17E-11
NM_021637	TMEM35	chrX:100157375-100157434	A_23_P45324	1.03	1.45	3.07E-08	1.25E-12
NM_024059	C20orf195	chr20:61658276-61658335	A_23_P131754	1.04	0.73	4.99E-04	2.04E-02
NM_001007245	IFRD1	chr7:111709468-111709527	A_24_P137897	1.04	0.74	5.16E-04	1.73E-02
NM_033035	TSLP	chr5:110439832-110439891	A_23_P121987	1.04	0.79	7.88E-04	1.49E-02
NM_033118	MYLK2	chr20:29886071-29886130	A_23_P80008	1.04	0.84	1.79E-05	5.77E-04
NM_003260	TLE2	chr19:2948943-2948884	A_23_P153676	1.04	0.84	5.62E-03	3.75E-02
BC028174	BC028174	chr17:6496728-6496787	A_32_P150391	1.04	0.85	4.50E-09	5.70E-07
BC018675	BC018675	chr2:196953881-196953940	A_24_P671115	1.04	0.96	3.34E-05	1.42E-04
NM_001033658	LOC283152	chr11:118566656-118566340	A_24_P450285	1.04	0.96	2.96E-04	1.02E-03
NM_003975	SH2D2A	chr1:153589316-153589257	A_23_P160618	1.04	0.98	6.53E-04	1.71E-03
XM_928397	LOC645356	chr20:25681270-25681329	A_32_P139351	1.04	1.06	3.99E-02	4.99E-02
NM_174899	FBXO36	chr2:230666468-230666527	A_24_P254702	1.04	1.07	3.16E-12	8.84E-13
NM_017707	DDEFL1	chr1:23500652-23500593	A_23_P114689	1.04	1.08	3.64E-05	2.36E-05
NR_002185	OR7E91P	chr2:71168002-71168061	A_24_P332718	1.04	1.10	5.02E-15	6.43E-16
NM_003353	UCN	chr2:27442316-27442257	A_24_P62883	1.04	1.13	1.11E-07	1.40E-08
NM_201636	TBXA2R	chr19:3545819-3545760	A_23_P90357	1.04	1.14	1.12E-05	2.15E-06
NM_005811	GDF11	chr12:54429716-54429775	A_23_P76102	1.04	1.15	6.15E-06	8.75E-07
NM_000765	CYP3A7	chr7:98947747-98947688	A_23_P358917	1.04	1.15	1.25E-05	2.03E-06
NM_002217	ITIH3	chr3:52817701-52817760	A_23_P6822	1.04	1.16	5.10E-05	8.91E-06
NM_013989	DIO2	chr14:79734152-79734093	A_23_P48740	1.04	1.21	5.87E-06	2.83E-07
THC2398598	THC2398598	chr16:030105976-030106034	A_24_P773012	1.04	1.23	8.44E-04	1.05E-04
NM_021101	CLDN1	chr3:191506762-191506703	A_23_P57784	1.04	1.24	3.67E-04	3.35E-05
NM_032581	DRCTNNB1A	chr7:22758437-22758378	A_23_P8582	1.04	1.30	4.60E-06	3.12E-08
NM_006096	NDRG1	chr8:134318960-134318901	A_23_P20494	1.04	1.39	2.42E-02	3.41E-03
NM_198194	STOM	chr9:121181489-121181430	A_24_P141214	1.04	1.48	2.16E-03	2.30E-05
NM_032269	C16orf50	chr16:56322372-56322431	A_23_P129367	1.05	0.64	8.95E-08	7.00E-04
NM_033297	NALP12	chr19:58999100-58999041	A_23_P90041	1.05	0.64	1.05E-06	2.65E-03
AY430414	ENTPD8	chr9:137606053-137605614	A_23_P44335	1.05	0.87	4.54E-03	2.65E-02
AI470277	AI470277	chr16:83500368-83500309	A_32_P68142	1.05	0.97	1.19E-08	8.83E-08
BF213738	BF213738	chr12:9898291-9897264	A_23_P76480	1.05	1.00	2.08E-17	1.23E-16
NM_022091	ASCC3	chr6:101413771-101413712	A_23_P382654	1.05	1.00	1.13E-02	2.23E-02
NM_001753	CAV1	chr7:115794693-115794752	A_23_P134454	1.05	1.03	1.29E-03	2.22E-03
NM_144618	MGC29891	chr1:147903920-147903979	A_23_P73208	1.05	1.06	8.67E-11	6.08E-11
NM_004865	TBPL1	chr6:134350108-134350167	A_23_P168276	1.05	1.22	6.42E-09	7.88E-11
NM_000185	SERPIND1	chr22:19466274-19466333	A_23_P6335	1.05	1.27	6.84E-12	7.69E-15
Z74615	COL1A1	chr17:45616625-45616564	A_23_P207520	1.05	1.38	1.88E-08	7.86E-12
NM_000737	CGB	chr19:54218112-54218053	A_24_P395814	1.05	1.47	1.45E-07	1.24E-11
NM_019618	IL1F9	chr2:113459238-113459297	A_23_P17053	1.06	0.68	2.53E-19	3.57E-12
NM_004556	NFKBIE	chr6:44334595-44334536	A_23_P30655	1.06	0.78	7.17E-07	1.98E-04
NM_033520	C19orf33	chr19:43486893-43486952	A_23_P39202	1.06	0.78	2.20E-03	3.47E-02
THC2419501	THC2419501	chr9:107882093-107882152	A_24_P255384	1.06	0.82	2.85E-08	9.02E-06
NM_022644	CSH2	chr17:59303401-59303342	A_23_P207154	1.06	0.85	1.51E-11	9.06E-09
NM_006613	GRAP	chr17:18983778-18983837	A_23_P49638	1.06	0.86	2.88E-03	2.31E-02
NM_004244	CD163	chr12:7515208-7515149	A_23_P33723	1.06	0.87	1.04E-12	3.88E-10
BC071797	BC071797	chr4:49347318-49347259	A_32_P13151	1.06	0.91	2.77E-04	2.36E-03
NM_006744	RBP4	chr10:95341718-95341659	A_23_P75283	1.06	0.97	4.00E-09	4.05E-08
NM_152701	ABCA13	chr7:48464115-48464174	A_23_P364324	1.06	1.16	3.15E-04	1.21E-04
NM_000290	PGAM2	chr7:43877974-43877812	A_23_P93940	1.06	1.17	2.23E-10	1.25E-11
NM_172229	KREMEN2	chr16:2958253-2958312	A_23_P77612	1.06	1.18	1.57E-04	3.55E-05
NM_006849	PDIA2	chr16:276604-276663	A_23_P325642	1.06	1.33	6.90E-10	4.08E-13
Z74615	COL1A1	chr17:45616625-45616564	A_23_P207520	1.06	1.34	2.44E-08	2.75E-11
NM_003088	FSCN1	chr7:5418183-5418242	A_23_P168531	1.06	1.67	5.28E-04	1.88E-07

Gene	Symbol	Chromosomal position	Probe	siCASP8AP2.3	CASP8AP2.6	siCASP8AP2.3	CASP8AP2.6
				M	M	Q	Q
AK090630	UTS2D	chr3:192467759-192467700	A_32_P225659	1.06	1.87	3.44E-09	1.03E-17
NM_024875	SYNPO2L	chr10:75075463-75075404	A_23_P355517	1.07	0.70	1.77E-09	2.87E-05
NM_002192	INHBA	chr7:41502545-41502486	A_23_P122924	1.07	0.74	4.00E-04	1.86E-02
NM_000862	HSD3B1	chr1:119769293-119769353	A_24_P350397	1.07	0.75	3.29E-16	7.80E-11
NM_006031	PCNT	chr21:46607847-46607906	A_24_P8350	1.07	0.76	1.69E-03	3.65E-02
NM_021614	KCNN2	chr5:113859734-113859793	A_23_P500353	1.07	0.78	2.66E-03	4.14E-02
NM_024119	LGP2	chr17:37507044-37506985	A_23_P38346	1.07	0.81	2.01E-04	5.91E-03
NM_152286	PNPLA7	chr9:137630560-137630501	A_23_P43455	1.07	0.83	8.84E-04	1.33E-02
NM_022818	MAP1LC3B	chr16:85995467-85995526	A_24_P108005	1.07	0.92	8.09E-03	3.19E-02
NM_005907	MAN1A1	chr6:119543249-119543190	A_23_P156431	1.07	0.93	4.80E-05	4.73E-04
ENST00000294485	ENST00000294485	chr1:11720098-11720157	A_24_P68008	1.07	0.98	6.63E-04	2.33E-03
NM_000602	SERPINE1	chr7:100374990-100375049	A_24_P158089	1.07	1.03	3.19E-03	6.28E-03
NM_000540	RYR1	chr19:43769805-43769864	A_23_P78867	1.07	1.09	4.51E-04	4.48E-04
THC2278725	THC2278725	chrX:145597260-145597319	A_23_P255457	1.07	1.10	4.92E-05	3.95E-05
NM_005574	LMO2	chr11:33836878-33836819	A_23_P53126	1.07	1.16	2.64E-03	1.59E-03
NM_006674	HCP5	chr6:31540624-31540683	A_24_P17870	1.07	1.19	2.64E-07	2.48E-08
AK097804	C20orf112	chr20:30572339-30572280	A_23_P303548	1.07	1.25	1.83E-08	2.38E-10
NM_024758	AGMAT	chr1:15645461-15645402	A_23_P103720	1.07	1.27	1.50E-03	2.33E-04
THC2249196	THC2249196	chr1:16128831-16128890	A_23_P149270	1.07	1.31	4.69E-10	6.61E-13
BX538015	DKFZP686E2158	chr5:60490491-60490432	A_32_P33821	1.07	1.42	1.51E-05	4.22E-08
NM_003842	TNFRSF10B	chr8:22936212-22936153	A_23_P169030	1.07	1.47	1.41E-08	9.44E-13
ENST00000375486	ENST00000375486	chr1:17139129-17139070	A_23_P201747	1.07	1.47	1.42E-07	1.97E-11
NM_014354	C6orf54	chr6:168212160-168212101	A_23_P93109	1.07	1.53	7.46E-09	1.21E-13
NM_002514	NOV	chr8:120505616-120505675	A_23_P82929	1.07	1.54	9.39E-06	2.52E-09
NM_000459	TEK	chr9:27220070-27220129	A_23_P374695	1.07	1.71	7.17E-09	1.41E-15
DB362335	DB362335	chrX:48526991-48527050	A_32_P114918	1.08	0.64	1.27E-04	3.15E-02
NM_021833	UCP1	chr4:141842097-141841932	A_23_P30091	1.08	0.80	6.61E-12	3.26E-08
NM_002288	LAIR2	chr19:59713621-59713680	A_23_P209129	1.08	0.84	2.57E-12	5.65E-09
NM_018167	BTBD7	chr14:92829361-92829302	A_23_P88184	1.08	0.85	1.29E-05	5.33E-04
NM_177925	H2AFJ	chr12:14818894-14818953	A_24_P236003	1.08	0.91	1.95E-12	4.53E-10
NM_058172	ANTXR2	chr4:81256461-81256402	A_24_P377144	1.08	0.94	2.43E-07	4.44E-06
NM_017567	NAGK	chr2:71211505-71212294	A_23_P154208	1.08	0.94	2.55E-04	1.93E-03
NM_147161	ACOT11	chr1:54787695-54787754	A_23_P417415	1.08	0.97	1.71E-07	2.07E-06
NM_153006	NAGS	chr17:39441828-39441887	A_32_P32739	1.08	0.98	6.20E-04	2.46E-03
NM_023940	RASL11B	chr4:53573770-53573830	A_23_P69738	1.08	0.99	7.79E-04	2.79E-03
NM_004171	SLC1A2	chr11:35243736-35243677	A_23_P162068	1.08	1.18	2.44E-08	2.31E-09
NM_005658	TRAF1	chr9:120745972-120745913	A_23_P216970	1.08	1.24	2.59E-05	2.13E-06
NM_078487	CDKN2B	chr9:21995004-21994945	A_24_P360674	1.08	1.26	1.85E-04	2.01E-05
NM_001365	DLG4	chr17:7034190-7034130	A_23_P411102	1.08	1.53	4.42E-04	1.58E-06
NM_000700	ANXA1	chr9:73014556-73014615	A_23_P94501	1.08	1.58	1.07E-04	6.67E-08
NM_174918	MCEMP1	chr19:7650452-7650511	A_23_P330561	1.08	1.75	7.72E-08	3.52E-14
NM_138771	LOC90693	chr7:23455959-23456018	A_23_P168592	1.09	0.72	7.96E-04	3.70E-02
NM_015205	ATP11A	chr13:112585879-112585938	A_24_P59403	1.09	0.85	5.18E-10	3.04E-07
NM_033397	KIAA1754	chr10:106062108-106062049	A_23_P340333	1.09	0.90	6.51E-05	1.11E-03
NM_002686	PNMT	chr17:35080100-35080159	A_23_P100642	1.09	0.95	1.20E-03	6.44E-03
NM_004360	CDH1	chr16:67426715-67426774	A_23_P206359	1.09	0.98	9.83E-07	9.48E-06
A_23_P64962	A_23_P64962	chr12:006307567-006307626	A_23_P64962	1.09	1.03	1.01E-05	2.96E-05
NM_152376	UBXD3	chr1:20263466-20263525	A_23_P85664	1.09	1.04	2.84E-03	6.28E-03
NM_001039650	ZMYM5	chr13:19309813-19309754	A_23_P65208	1.09	1.08	1.75E-03	2.44E-03
NM_144975	SLFN5	chr17:30610486-30610545	A_23_P402899	1.09	1.09	6.84E-03	9.62E-03
ENST00000295989	ENST00000295989	chr3:12851193-12851252	A_23_P250102	1.09	1.10	5.75E-14	3.81E-14
NM_005574	LMO2	chr11:33836878-33836819	A_23_P53126	1.09	1.12	2.53E-03	2.59E-03
NM_022040	LAT2	chr7:73079221-73079693	A_24_P351852	1.09	1.17	1.28E-06	2.77E-07
NM_005308	GRK5	chr10:121204957-121205013	A_23_P12884	1.09	1.17	1.78E-06	4.12E-07
THC2454860	THC2454860	chr2:217794553-217794612	A_32_P163891	1.09	1.22	8.05E-07	6.18E-08
NM_013262	MYLIP	chr6:16238808-16238867	A_24_P917123	1.09	1.22	1.35E-04	2.62E-05

Gene	Symbol	Chromosomal position	Probe	siCASP8AP2.3	CASP8AP2.6	siCASP8AP2.3	CASP8AP2.6
				M	M	Q	Q
NM_032034	SLC4A11	chr20:3156221-3156162	A_23_P154688	1.09	1.40	1.93E-09	7.86E-13
THC2432515	THC2432515	chr4:88159003-88158944	A_32_P184268	1.09	1.45	3.11E-05	1.03E-07
AK096449	LOC285986	chr7:94814004-94814063	A_32_P130999	1.09	1.62	1.07E-06	3.19E-11
BY798288	BY798288	chr17:1909383-1909535	A_23_P129856	1.09	1.83	4.86E-08	4.28E-15
NM_030615	KIF25	chr6:168264253-168264311	A_23_P59410	1.09	1.92	1.47E-11	7.46E-21
X84195	ACYP2	chr2:54274255-54277483	A_23_P159424	1.10	0.67	1.59E-04	2.91E-02
NM_176782	C1orf179	chr1:54786941-54786882	A_23_P407695	1.10	0.71	5.25E-05	1.13E-02
NM_175575	WFIKKN2	chr17:46274644-46274703	A_24_P399230	1.10	0.92	1.36E-17	9.57E-15
NM_182924	MICAL-L2	chr7:1247315-1247256	A_24_P303524	1.10	0.95	2.56E-08	8.30E-07
NM_001005339	RGS10	chr10:121249572-121249513	A_23_P138717	1.10	1.04	7.41E-05	1.96E-04
NM_000930	PLAT	chr8:42152028-42151969	A_23_P82868	1.10	1.13	1.33E-02	1.60E-02
NM_020686	ABAT	chr16:8785829-8785888	A_23_P152505	1.10	1.18	1.13E-04	5.01E-05
NR_003062	SPRR2C	chr1:149925773-149925714	A_23_P126089	1.10	1.22	4.74E-02	3.83E-02
NM_005213	CSTA	chr3:123543136-123543195	A_23_P170233	1.10	1.31	3.86E-07	4.79E-09
NM_004417	DUSP1	chr5:172127961-172127902	A_23_P110712	1.10	1.31	5.35E-04	5.60E-05
NM_000728	CALCB	chr11:15055491-15055550	A_24_P198178	1.10	1.36	3.63E-03	4.65E-04
NM_018440	PAG1	chr8:82049137-82049078	A_23_P347070	1.10	1.41	5.86E-04	1.91E-05
AV756170	AV756170	chr1:222494690-222494749	A_32_P2605	1.10	1.46	1.26E-03	2.82E-05
NM_000214	JAG1	chr20:10567120-10567061	A_23_P210763	1.10	1.50	1.81E-05	2.72E-08
NM_000594	TNF	chr6:31653816-31653875	A_23_P376488	1.10	1.54	1.68E-05	1.27E-08
NM_000494	COL17A1	chr10:105781132-105781073	A_23_P501010	1.10	1.56	1.53E-04	2.92E-07
NM_002429	MMP19	chr12:54516762-54516703	A_23_P203888	1.11	0.82	8.09E-05	4.08E-03
BC013171	BC013171	chrX:151566806-151566747	A_24_P101651	1.11	0.84	1.57E-11	3.93E-08
NM_030666	SERPINB1	chr6:2778922-2778863	A_23_P214330	1.11	0.87	2.56E-08	6.45E-06
A_23_P72014	A_23_P72014		A_23_P72014	1.11	0.87	1.37E-06	1.33E-04
NM_005532	IFI27	chr14:93652720-93652779	A_24_P270460	1.11	0.89	5.25E-04	7.29E-03
AF211119	C9orf53	chr9:21957537-21957596	A_23_P307502	1.11	0.90	3.24E-10	9.00E-08
NM_004360	CDH1	chr16:67426715-67426774	A_23_P206359	1.11	0.96	5.35E-07	1.04E-05
NM_000559	HBG1	chr11:5226169-5226110	A_23_P53137	1.11	1.01	1.53E-07	1.30E-06
NM_004443	EPHB3	chr3:185782527-185782586	A_23_P95060	1.11	1.10	1.35E-04	1.94E-04
ENST00000288548	ENST00000288548	chr2:23842960-23843019	A_23_P209360	1.11	1.13	4.22E-07	2.78E-07
NM_000930	PLAT	chr8:42152028-42151969	A_23_P82868	1.11	1.14	1.18E-02	1.33E-02
NM_003459	SLC30A3	chr2:27389258-27389199	A_23_P302568	1.11	1.20	7.45E-04	3.66E-04
NM_153750	C21orf81	chr21:14274111-14274052	A_32_P68942	1.11	1.22	2.63E-06	3.68E-07
NM_001953	ECGF1	chr22:49254629-49254488	A_23_P91802	1.11	1.24	1.55E-05	2.28E-06
BM461836	BM461836	chr15:28176109-28176168	A_32_P42236	1.11	1.34	4.02E-05	1.29E-06
NM_080489	SDCBP2	chr20:1241097-1241038	A_23_P131899	1.11	1.35	8.56E-10	1.83E-12
AF099011	EHD1	chr11:64378621-64378562	A_24_P184295	1.11	1.36	5.12E-05	1.35E-06
NM_006945	SPRR2D	chr1:149825485-149825426	A_23_P11644	1.11	1.37	4.57E-02	1.81E-02
NM_007261	CD300A	chr17:69992435-69992494	A_24_P159434	1.11	1.40	1.68E-04	3.59E-06
NM_001243	TNFRSF8	chr1:12138427-12138486	A_23_P500614	1.11	1.58	2.48E-07	1.57E-11
NM_138705	CALML6	chr1:1880649-1880780	A_23_P62588	1.12	0.92	2.54E-14	1.62E-11
NM_130853	PTPRS	chr19:5225264-5224590	A_24_P290856	1.12	0.93	5.19E-05	8.52E-04
NM_005194	CEBPB	chr20:48242396-48242455	A_23_P411296	1.12	1.00	8.21E-04	3.72E-03
NM_153206	AMICA1	chr11:117569958-117569899	A_24_P192914	1.12	1.03	8.74E-16	1.62E-14
NM_000559	HBG1	chr11:5226158-5226100	A_23_P64539	1.12	1.04	9.90E-08	5.69E-07
NM_000559	HBG1	chr11:5226169-5226110	A_23_P53137	1.12	1.06	1.40E-09	7.07E-09
NM_182924	MICAL-L2	chr7:1248013-1247526	A_24_P206305	1.12	1.09	2.36E-07	4.62E-07
THC2375612	THC2375612	chr6:44293831-44293890	A_32_P144999	1.12	1.13	2.00E-07	1.88E-07
NM_030792	GDPD5	chr11:74823576-74823517	A_23_P87401	1.12	1.13	7.33E-04	8.11E-04
NM_006673	ARID5A	chr2:96640058-96640117	A_23_P143016	1.12	1.14	1.91E-08	1.14E-08
NM_080744	SRCRB4D	chr7:75663359-75663300	A_23_P8571	1.12	1.19	3.01E-07	7.43E-08
NM_024786	ZDHHC11	chr5:849118-849059	A_23_P256008	1.12	1.21	1.00E-03	5.17E-04
AK021777	GALNT10	chr5:153753992-153754051	A_23_P19102	1.12	1.21	1.33E-02	1.08E-02
Z74615	COL1A1	chr17:45616625-45616564	A_23_P207520	1.12	1.32	1.22E-08	9.32E-11
NM_152397	IQCF1	chr3:51904008-51903949	A_32_P121834	1.12	1.35	6.24E-10	1.44E-12

Gene	Symbol	Chromosomal position	Probe	siCASP8AP2.3	CASP8AP2.6	siCASP8AP2.3	CASP8AP2.6
				M	M	Q	Q
NM_007365	PADI2	chr1:17155575-17154443	A_24_P187970	1.12	1.61	9.90E-08	2.91E-12
NM_014580	SLC2A8	chr9:127249373-127249432	A_23_P252783	1.13	0.67	1.37E-05	1.14E-02
NM_174937	TCERG1L	chr10:132780823-132780764	A_23_P368794	1.13	0.79	8.20E-05	7.71E-03
NM_016651	DACT1	chr14:58184466-58184525	A_23_P65518	1.13	0.81	2.87E-05	2.95E-03
NM_000610	CD44	chr11:35207510-35207569	A_23_P24870	1.13	0.94	1.04E-02	4.73E-02
NM_001114	ADCY7	chr16:48909348-48909407	A_24_P416177	1.13	0.96	1.12E-04	1.22E-03
NM_080878	ITLN2	chr1:157728100-157728041	A_24_P53778	1.13	1.00	3.77E-08	8.30E-07
NM_000559	HBG1	chr11:5226169-5226110	A_23_P53137	1.13	1.01	1.17E-08	1.59E-07
NM_018590	GALNACT-2	chr10:42999832-42999891	A_23_P149892	1.13	1.02	2.13E-09	2.88E-08
NM_181711	GRASP	chr12:50695827-50695886	A_23_P105442	1.13	1.10	1.36E-09	2.53E-09
NM_005574	LMO2	chr11:33836878-33836819	A_23_P53126	1.13	1.13	1.67E-03	2.30E-03
NM_000363	TNNI3	chr19:60357382-60357323	A_23_P67453	1.13	1.16	7.57E-06	5.81E-06
NM_025248	SNIP	chr17:33941460-33941401	A_24_P57977	1.13	1.23	3.15E-04	1.12E-04
NM_014861	KIAA0703	chr16:83054944-83055003	A_23_P117992	1.13	1.27	7.73E-05	1.30E-05
NM_000185	SERPIND1	chr22:19466274-19466333	A_23_P6335	1.13	1.30	4.77E-17	1.32E-19
Z74615	COL1A1	chr17:45616625-45616564	A_23_P207520	1.13	1.38	5.45E-09	1.73E-11
A_23_P123234	A_23_P123234	chr7:100374532-100374591	A_23_P123234	1.13	1.45	1.23E-03	5.92E-05
ENST00000278949	ENST00000278949	chr11:117602693-117602634	A_24_P270033	1.13	1.52	6.53E-05	2.72E-07
NM_006829	C10orf116	chr10:88720288-88720347	A_23_P161439	1.14	0.98	1.15E-04	1.14E-03
NM_005194	CEBPB	chr20:48242396-48242455	A_23_P411296	1.14	0.98	7.56E-04	5.25E-03
NM_020373	TMEM16B	chr12:5542200-5542141	A_32_P141238	1.14	1.00	1.49E-03	7.01E-03
NM_000930	PLAT	chr8:42152028-42151969	A_23_P82868	1.14	1.04	1.62E-02	4.05E-02
NM_002872	RAC2	chr22:35946011-35945952	A_23_P218770	1.14	1.07	2.72E-04	8.24E-04
NM_152310	ELOVL3	chr10:103978860-103978919	A_23_P149858	1.14	1.12	9.47E-13	1.96E-12
NM_080860	TSGA2	chr21:42765749-42765690	A_23_P102950	1.14	1.15	9.09E-07	8.32E-07
NM_015247	CYLD	chr16:49385742-49385801	A_24_P48078	1.14	1.27	2.58E-06	2.83E-07
NM_021603	FXYD2	chr11:117198605-117198348	A_24_P196562	1.14	1.29	2.69E-05	3.11E-06
Z74615	COL1A1	chr17:45616625-45616564	A_23_P207520	1.14	1.31	1.20E-09	1.88E-11
NM_019089	HES2	chr1:6410062-6410003	A_23_P304716	1.14	1.31	4.57E-06	2.71E-07
BC020884	HIST4H4	chr12:14813388-14813329	A_24_P261691	1.14	1.68	9.21E-09	5.34E-14
NM_033520	C19orf33	chr19:43487421-43487480	A_23_P208788	1.15	0.86	5.46E-04	1.23E-02
AK127078	AK127078	chr15:30855914-30855855	A_23_P206120	1.15	0.91	2.20E-03	2.25E-02
BC002778	MYLC2PL	chr7:100850211-100850152	A_23_P393015	1.15	0.94	2.30E-10	6.27E-08
NM_007105	SLC22A18AS	chr11:2866063-2866004	A_24_P372643	1.15	0.94	5.60E-07	3.20E-05
NM_145807	LOC126147	chr19:53856715-53856656	A_32_P394951	1.15	0.97	7.88E-16	4.46E-13
NM_004533	MYBPC2	chr19:55661292-55661351	A_23_P16262	1.15	0.97	1.28E-08	7.02E-07
NM_000559	HBG1	chr11:5226169-5226110	A_23_P53137	1.15	0.98	1.48E-08	6.99E-07
NM_022640	CSH1	chr17:59326294-59326235	A_23_P381347	1.15	0.99	3.53E-10	1.92E-08
THC2373975	THC2373975	chr11:17359356-17359415	A_32_P2050	1.15	1.03	1.07E-07	1.41E-06
NM_005574	LMO2	chr11:33836878-33836819	A_23_P53126	1.15	1.12	8.03E-04	1.45E-03
NM_005574	LMO2	chr11:33836878-33836819	A_23_P53126	1.15	1.18	1.07E-03	1.03E-03
NM_002462	MX1	chr21:41752884-41752943	A_23_P17663	1.15	1.24	3.75E-04	1.61E-04
NM_004864	GDF15	chr19:18360890-18360949	A_23_P16523	1.15	1.24	2.11E-02	1.78E-02
NM_003534	HIST1H3G	chr6:26379448-26379389	A_23_P42198	1.15	1.27	1.39E-04	3.34E-05
ENST00000328398	ENST00000328398	chr15:97329446-97329387	A_32_P3534	1.15	1.31	1.89E-13	1.64E-15
NM_002630	PGC	chr6:41812514-41812455	A_23_P7961	1.15	1.33	7.14E-03	2.55E-03
NM_015687	FILIP1	chr6:76075084-76075025	A_23_P436369	1.15	1.70	6.78E-10	1.43E-15
NM_001005356	POTE14	chr14:18623888-18628726	A_32_P43588	1.16	0.64	1.67E-05	2.14E-02
NM_033060	KRTAP4-10	chr17:36594106-36594047	A_23_P10936	1.16	0.72	2.62E-04	2.99E-02
NM_016605	FAM53C	chr5:137713196-137713255	A_24_P380234	1.16	0.73	7.80E-04	4.70E-02
NM_198075	LRRC56	chr11:544830-544889	A_23_P368996	1.16	0.93	2.25E-08	3.35E-06
ENST00000374314	ENST00000374314	chr10:46425532-46425591	A_23_P343382	1.16	0.95	6.25E-06	1.98E-04
NM_005194	CEBPB	chr20:48242396-48242455	A_23_P411296	1.16	0.97	6.17E-04	5.22E-03
NM_000610	CD44	chr11:35207510-35207569	A_23_P24870	1.16	0.97	8.81E-03	4.19E-02
NM_005574	LMO2	chr11:33836878-33836819	A_23_P53126	1.16	0.99	1.85E-03	1.09E-02
NM_012232	PTRF	chr17:37808062-37808003	A_23_P394064	1.16	1.11	6.35E-03	1.33E-02

Gene	Symbol	Chromosomal position	Probe	siCASP8AP2.3	CASP8AP2.6	siCASP8AP2.3	CASP8AP2.6
				M	M	Q	Q
ENST00000252744	ENST00000252744	chr5:60876965-60877024	A_23_P410859	1.16	1.12	1.11E-05	2.57E-05
NM_001753	CAV1	chr7:115794693-115794752	A_23_P134454	1.16	1.13	4.16E-04	7.77E-04
NM_145244	DDIT4L	chr4:101464473-101464414	A_23_P302672	1.16	1.14	4.04E-05	7.00E-05
Z74615	COL1A1	chr17:45616625-45616564	A_23_P207520	1.16	1.17	1.87E-07	1.67E-07
NM_015111	N4BP3	chr5:177481885-177481944	A_23_P58747	1.16	1.18	2.81E-05	2.46E-05
THC2438039	THC2438039	chr12:13048696-13048755	A_32_P174572	1.16	1.24	3.34E-08	6.94E-09
NM_016116	ASB4	chr7:94770003-94801817	A_23_P501319	1.16	1.24	7.01E-06	2.01E-06
A_23_P123234	A_23_P123234	chr7:100374532-100374591	A_23_P123234	1.16	1.32	1.71E-03	4.88E-04
NM_031468	CALN1	chr7:70720125-70720066	A_24_P380567	1.16	1.62	1.31E-06	3.56E-10
NM_003121	SPIB	chr19:55623849-55623908	A_23_P39067	1.16	1.64	6.99E-06	2.68E-09
NM_002089	CXCL2	chr4:75328079-75328020	A_23_P315364	1.16	1.69	1.76E-03	9.68E-06
NM_005954	MT3	chr16:55182434-55182492	A_23_P129629	1.17	0.77	3.19E-06	1.85E-03
NM_005194	CEBPB	chr20:48242396-48242455	A_23_P411296	1.17	0.86	1.06E-03	2.19E-02
NM_022574	PERQ1	chr7:99923749-99923690	A_24_P163574	1.17	0.90	3.80E-05	1.56E-03
THC2315024	THC2315024	chr5:15707469-15707528	A_32_P110485	1.17	0.96	3.16E-09	4.64E-07
BC107798	TNNT1	chr19:60340335-60337330	A_23_P56050	1.17	0.97	8.06E-05	1.15E-03
NM_005194	CEBPB	chr20:48242396-48242455	A_23_P411296	1.17	0.99	3.54E-04	3.24E-03
NM_005194	CEBPB	chr20:48242396-48242455	A_23_P411296	1.17	1.02	4.33E-04	2.77E-03
NM_005822	DSCR1L1	chr6:46296467-46296434	A_32_P156851	1.17	1.08	1.26E-08	1.04E-07
NM_001014279	LOC389289	chr5:43075280-43075221	A_23_P431591	1.17	1.19	2.16E-06	1.44E-06
NM_024034	GDAP1L1	chr20:42341139-42341198	A_23_P17354	1.17	1.21	8.37E-08	3.43E-08
NM_015559	SETBP1	chr18:40898691-40898750	A_23_P4551	1.17	1.23	9.86E-06	4.06E-06
NM_005242	F2RL1	chr5:76165394-76165453	A_23_P58835	1.17	1.25	2.87E-05	9.70E-06
NM_006884	SHOX2	chr3:159297786-159297727	A_23_P124384	1.17	1.32	4.70E-08	1.95E-09
A_23_P123234	A_23_P123234	chr7:100374532-100374591	A_23_P123234	1.17	1.45	8.46E-04	5.11E-05
NM_173198	NR4A3	chr9:99708540-99708599	A_23_P398566	1.17	1.50	2.71E-07	4.24E-10
AK027211	AK027211	chr16:21039136-21039077	A_23_P100522	1.17	1.57	5.36E-03	2.81E-04
NM_138328	RHBDL3	chr17:27672419-27672478	A_23_P308731	1.18	0.81	1.38E-04	1.06E-02
NM_000559	HBG1	chr11:5226169-5226110	A_23_P53137	1.18	0.90	6.97E-09	3.78E-06
ENST00000325900	ENST00000325900	chrX:134285006-134285065	A_24_P418250	1.18	0.91	1.38E-06	1.53E-04
THC2346121	THC2346121	chr5:2012212-2012271	A_32_P104023	1.18	1.00	5.43E-05	7.04E-04
NM_000814	GABRB3	chr15:24344171-24344112	A_23_P14821	1.18	1.07	1.22E-14	3.75E-13
NM_005194	CEBPB	chr20:48242396-48242455	A_23_P411296	1.18	1.08	2.70E-04	1.12E-03
BC018597	BC018597	chr5:10710025-10710084	A_32_P137604	1.18	1.14	1.85E-03	3.46E-03
NM_005574	LMO2	chr11:33836878-33836819	A_23_P53126	1.18	1.19	1.08E-03	1.26E-03
NM_014466	TEKT2	chr1:36222817-36222876	A_23_P45955	1.18	1.24	1.01E-12	1.76E-13
NM_001039212	FLJ37078	chr7:75560867-75560926	A_23_P331700	1.18	1.27	1.46E-04	6.46E-05
BC020630	CAMK2N1	chr1:20554766-20554707	A_23_P11800	1.18	1.33	1.18E-03	3.64E-04
Z74615	COL1A1	chr17:45616625-45616564	A_23_P207520	1.18	1.34	1.07E-07	4.23E-09
NM_152547	BTNL9	chr5:180419774-180419833	A_32_P187951	1.18	1.35	1.41E-12	1.10E-14
NM_144593	RHEBL1	chr12:47746724-47746579	A_23_P2307	1.18	1.39	1.91E-06	5.45E-08
NM_145341	PDCD4	chr10:112645766-112645825	A_23_P9932	1.18	1.49	2.93E-04	8.65E-06
NM_022873	IFI6	chr1:27677038-27676979	A_23_P201459	1.18	1.57	1.86E-06	2.19E-09
NM_001740	CALB2	chr16:69977010-69981169	A_23_P21092	1.19	0.89	4.05E-03	4.54E-02
NM_022579	CSHL1	chr17:59340985-59340926	A_23_P344408	1.19	0.91	1.21E-11	2.94E-08
NM_004360	CDH1	chr16:67426715-67426774	A_23_P206359	1.19	0.93	3.18E-07	4.61E-05
NM_019886	CHST7	chrX:46213662-46213721	A_23_P319617	1.19	0.99	3.33E-07	1.53E-05
NM_000906	NPR1	chr1:150475846-150475905	A_23_P147711	1.19	1.00	6.27E-18	5.84E-15
NM_007003	PAGE4	chrX:49300234-49301488	A_24_P254506	1.19	1.03	1.22E-11	8.86E-10
NM_005855	RAMP1	chr2:238602661-238602720	A_23_P50946	1.19	1.03	1.81E-08	5.14E-07
NM_002405	MFNG	chr22:36189674-36189615	A_24_P224926	1.19	1.04	1.56E-07	3.25E-06
NM_000930	PLAT	chr8:42152028-42151969	A_23_P82868	1.19	1.13	8.53E-03	1.72E-02
NM_004405	DLX2	chr2:172789928-172789869	A_23_P28598	1.19	1.23	4.48E-10	1.81E-10
NM_138325	PCSK6	chr15:99728091-99724030	A_23_P390006	1.19	1.41	3.83E-08	4.68E-10
NM_006079	CITED2	chr6:139735344-139735285	A_23_P214969	1.19	1.53	1.24E-04	1.78E-06
NM_133639	RHOV	chr15:38952298-38952239	A_23_P424561	1.19	1.57	4.47E-06	8.43E-09

Gene	Symbol	Chromosomal position	Probe	siCASP8AP2.3	CASP8AP2.6	siCASP8AP2.3	CASP8AP2.6
				M	M	Q	Q
NM_172113	EYA2	chr20:45250751-45250810	A_23_P500421	1.19	1.59	1.67E-05	3.93E-08
NM_032255	ZNF541	chr19:52715862-52715803	A_23_P50517	1.19	1.62	1.19E-10	2.87E-15
AB032985	NXPH3	chr17:45015492-45015551	A_24_P940086	1.20	0.78	8.77E-06	4.19E-03
NM_006270	RRAS	chr19:54830437-54830378	A_23_P39074	1.20	0.82	1.63E-03	4.33E-02
NM_018261	EXOC1	chr4:56609464-56609523	A_23_P113972	1.20	0.86	1.81E-07	1.16E-04
NM_005194	CEBPB	chr20:48242396-48242455	A_23_P411296	1.20	0.86	7.03E-04	1.99E-02
NM_030760	EDG8	chr19:10484943-10484884	A_23_P107744	1.20	0.97	2.58E-04	3.86E-03
NM_004360	CDH1	chr16:67426715-67426774	A_23_P206359	1.20	1.00	1.71E-07	7.86E-06
A_32_P25065	A_32_P25065	chr19:017787540-017787599	A_32_P25065	1.20	1.03	6.83E-04	4.58E-03
NM_003004	SECTM1	chr17:77872530-77872471	A_24_P48204	1.20	1.05	1.35E-03	6.67E-03
NM_001009609	SPANX-N3	chrX:142322411-142322352	A_24_P91830	1.20	1.18	2.62E-05	3.83E-05
A_23_P123234	A_23_P123234	chr7:100374532-100374591	A_23_P123234	1.20	1.21	1.43E-03	1.64E-03
NM_173812	DPY19L2	chr12:62260753-62250890	A_23_P374250	1.20	1.29	8.24E-21	3.25E-22
NM_003811	TNFSF9	chr19:6485725-6485784	A_23_P67224	1.20	1.35	4.47E-06	4.64E-07
A_23_P123234	A_23_P123234	chr7:100374532-100374591	A_23_P123234	1.20	1.39	8.80E-04	1.55E-04
NM_018194	HHAT	chr1:207237736-207237795	A_23_P136355	1.20	1.39	3.22E-03	9.29E-04
NM_177400	NKX6-2	chr10:134448505-134448446	A_23_P86587	1.21	0.60	4.59E-06	2.66E-02
NM_002298	LCP1	chr13:45598397-45598338	A_23_P204847	1.21	0.75	1.66E-04	2.55E-02
NM_001007279	RRP22	chr22:28033588-28033529	A_23_P166400	1.21	0.77	4.53E-11	4.22E-06
NM_207310	CCDC74B	chr2:130613263-130613204	A_23_P381102	1.21	0.83	2.03E-07	2.41E-04
NM_153376	CCDC96	chr4:7161091-7161032	A_32_P69930	1.21	0.85	2.13E-04	1.21E-02
NM_032790	TMEM142A	chr12:120542379-120542438	A_23_P258837	1.21	0.90	6.33E-08	3.09E-05
NM_004360	CDH1	chr16:67426715-67426774	A_23_P206359	1.21	0.91	2.56E-07	7.00E-05
NM_004360	CDH1	chr16:67426715-67426774	A_23_P206359	1.21	0.93	9.01E-08	2.03E-05
NM_001015880	PAPSS2	chr10:89494940-89495599	A_23_P104493	1.21	1.00	6.84E-12	2.43E-09
A_23_P123234	A_23_P123234	chr7:100374532-100374591	A_23_P123234	1.21	1.15	1.45E-03	3.17E-03
NM_001645	APOC1	chr19:50114276-50114335	A_24_P109214	1.21	1.17	1.08E-07	2.55E-07
NR_002815	LOC374491	chr13:24069688-24069747	A_32_P89827	1.21	1.19	1.04E-08	1.48E-08
NM_000399	EGR2	chr10:64241915-64241856	A_23_P46936	1.21	1.19	1.05E-04	1.54E-04
ENST00000367932	ENST00000367932	chr1:159088141-159088200	A_23_P103511	1.21	1.20	2.12E-04	3.02E-04
NM_005975	PTK6	chr20:61631242-61631183	A_23_P56978	1.21	1.22	7.45E-06	7.75E-06
NM_005574	LMO2	chr11:33836878-33836819	A_23_P53126	1.21	1.23	7.18E-04	7.44E-04
NM_000559	HBG1	chr11:5226169-5226110	A_23_P53137	1.21	1.24	5.45E-10	2.63E-10
NM_004004	GJB2	chr13:19659974-19659915	A_23_P204947	1.21	1.34	1.81E-06	2.03E-07
NM_005588	MEP1A	chr6:46915057-46915116	A_23_P93122	1.21	1.41	1.38E-12	5.19E-15
NM_004430	EGR3	chr8:22601968-22601909	A_23_P216225	1.21	1.44	1.34E-05	5.08E-07
NM_003088	FSCN1	chr7:5416881-5418181	A_23_P168532	1.21	1.51	1.55E-05	1.65E-07
AK124841	AK124841	chr15:71814993-71814934	A_24_P778844	1.22	0.61	4.29E-06	2.42E-02
NM_000515	GH1	chr17:59348591-59348532	A_23_P207194	1.22	0.91	1.31E-12	1.08E-08
NM_004360	CDH1	chr16:67426715-67426774	A_23_P206359	1.22	0.94	2.37E-07	4.76E-05
NM_002386	MC1R	chr16:88514777-88514836	A_23_P329271	1.22	0.95	5.94E-08	1.30E-05
NM_005574	LMO2	chr11:33836878-33836819	A_23_P53126	1.22	1.11	9.22E-04	3.40E-03
NM_019055	ROBO4	chr11:124261631-124261572	A_23_P104881	1.22	1.17	4.11E-05	9.85E-05
NM_001819	CHGB	chr20:5853937-5853996	A_23_P382584	1.22	1.17	2.03E-03	4.09E-03
NM_138931	BCL6	chr3:188922402-188922343	A_23_P57856	1.22	1.23	6.95E-04	7.78E-04
NM_006216	SERPINE2	chr2:224667800-224666102	A_23_P50919	1.22	1.23	5.64E-03	7.23E-03
A_23_P123234	A_23_P123234	chr7:100374532-100374591	A_23_P123234	1.22	1.35	6.49E-04	2.27E-04
NM_005508	CCR4	chr3:32970844-32970903	A_23_P72989	1.22	1.41	1.06E-05	6.65E-07
A_23_P123234	A_23_P123234	chr7:100374532-100374591	A_23_P123234	1.22	1.48	4.87E-04	3.55E-05
NM_024676	C1orf113	chr1:36459451-36459510	A_23_P307536	1.22	1.52	3.63E-05	5.81E-07
NM_005061	RPL3L	chr16:1935874-1935599	A_23_P118095	1.22	1.57	2.08E-04	3.33E-06
NM_000899	KITLG	chr12:87389180-87389121	A_24_P133253	1.22	1.60	3.06E-07	2.34E-10
THC2434152	THC2434152	chr17:20773344-20773403	A_24_P760368	1.22	1.72	1.66E-12	4.46E-18
NM_020407	RHBG	chr1:153167968-153168027	A_23_P51690	1.23	0.60	3.58E-14	1.27E-05
CB050071	LOC388160	chr15:82641811-82641870	A_24_P555170	1.23	0.69	4.32E-12	1.11E-05
NM_001276	CHI3L1	chr1:199884457-199883610	A_24_P274111	1.23	0.79	4.49E-07	9.56E-04

Gene	Symbol	Chromosomal position	Probe	siCASP8AP2.3	CASP8AP2.6	siCASP8AP2.3	CASP8AP2.6
				M	M	Q	Q
NM_144677	MGAT5B	chr17:72457940-72457999	A_24_P219474	1.23	0.80	1.55E-09	2.35E-05
NM_001009991	SYTL3	chr6:159156203-159156262	A_23_P168188	1.23	0.97	6.51E-08	1.02E-05
NM_058173	SBEM	chr12:53538293-53538352	A_23_P150979	1.23	1.05	1.53E-03	9.31E-03
NM_000862	HSD3B1	chr1:119769429-119769488	A_23_P97173	1.23	1.13	4.67E-09	4.63E-08
NM_000930	PLAT	chr8:42152028-42151969	A_23_P82868	1.23	1.17	6.14E-03	1.36E-02
NM_018943	TUBA8	chr22:16988374-16988433	A_24_P160104	1.23	1.21	6.74E-08	1.01E-07
NM_153690	FAM43A	chr3:195889912-195889971	A_23_P422144	1.23	1.23	6.26E-03	8.94E-03
NM_000441	SLC26A4	chr7:106951884-106951943	A_23_P331560	1.23	1.26	9.08E-05	7.07E-05
AK124080	AK124080	chr19:39090246-39090305	A_32_P80610	1.23	1.28	4.34E-07	1.85E-07
BC044246	KIAA1913	chr6:130806019-130806078	A_23_P391906	1.23	1.35	2.46E-05	5.08E-06
NM_001218	CA12	chr15:61404173-61404114	A_23_P372234	1.23	1.39	1.00E-10	2.27E-12
NM_004004	GJB2	chr13:19659974-19659915	A_23_P204947	1.23	1.41	6.59E-07	3.30E-08
NM_000482	APOA4	chr11:116196980-116196921	A_23_P87036	1.23	1.43	2.33E-13	8.80E-16
A_23_P123234	A_23_P123234	chr7:100374532-100374591	A_23_P123234	1.23	1.47	5.07E-04	5.11E-05
NM_152737	RNF182	chr6:14087976-14088035	A_23_P399255	1.23	1.53	1.01E-06	6.70E-09
NM_025201	PLEKHQ1	chr15:62947009-62947068	A_23_P129246	1.23	1.65	1.13E-07	3.47E-11
NM_024825	FLJ23447	chr19:13904548-13904489	A_23_P433798	1.23	1.74	2.52E-11	1.23E-16
NM_001178	ARNTL	chr11:13365159-13365218	A_23_P162037	1.24	0.69	1.86E-05	2.00E-02
NM_002905	RDH5	chr12:54404100-54404431	A_24_P218814	1.24	0.80	3.24E-07	7.30E-04
AK128103	AK128103	chr20:045956602-045956543	A_24_P887615	1.24	0.96	2.42E-17	2.50E-13
NM_198459	DENND2C	chr1:114837655-114837596	A_23_P46315	1.24	0.99	2.58E-07	2.30E-05
NM_000559	HBG1	chr11:5226169-5226110	A_23_P53137	1.24	1.00	9.88E-10	2.42E-07
NM_002231	CD82	chr11:44597472-44597531	A_23_P1782	1.24	1.00	6.09E-06	2.39E-04
NM_178514	LOC283487	chr13:110319860-110319801	A_23_P368909	1.24	1.00	7.70E-05	1.66E-03
NM_177528	SULT1A2	chr16:28510827-28510768	A_23_P106773	1.24	1.03	2.19E-08	1.61E-06
NM_005194	CEBPB	chr20:48242396-48242455	A_23_P411296	1.24	1.06	2.57E-04	2.19E-03
NM_007162	TFEB	chr6:41760036-41759977	A_23_P368729	1.24	1.15	2.01E-09	1.61E-08
NM_005574	LMO2	chr11:33836878-33836819	A_23_P53126	1.24	1.20	4.87E-04	9.20E-04
AK027091	AK027091	chr17_random:62817-62758	A_23_P399292	1.24	1.28	3.26E-04	2.87E-04
NM_052828	TRIM10	chr6:30227916-30227857	A_23_P420831	1.24	1.38	1.89E-07	1.33E-08
NM_004004	GJB2	chr13:19659974-19659915	A_23_P204947	1.24	1.41	6.49E-07	3.60E-08
NM_002776	KLK10	chr19:56209467-56209408	A_23_P107911	1.24	1.44	1.25E-03	2.48E-04
NM_000704	ATP4A	chr19:40732999-40732940	A_23_P430728	1.24	1.59	2.87E-06	9.22E-09
BC007394	MGC16291	chr10:42292827-42292886	A_24_P64401	1.25	0.84	3.09E-10	4.59E-06
NM_002015	FOXO1A	chr13:40031661-40031128	A_23_P151426	1.25	0.85	4.38E-05	6.46E-03
NM_022073	EGLN3	chr14:33464451-33464392	A_23_P360379	1.25	0.91	3.70E-04	1.27E-02
NM_020659	TTYH1	chr19:59639628-59639687	A_23_P50815	1.25	0.92	5.37E-04	1.49E-02
NM_000559	HBG1	chr11:5226169-5226110	A_23_P53137	1.25	1.00	2.65E-10	9.81E-08
NM_004360	CDH1	chr16:67426715-67426774	A_23_P206359	1.25	1.04	3.22E-08	2.22E-06
NM_005194	CEBPB	chr20:48242396-48242455	A_23_P411296	1.25	1.06	1.76E-04	1.86E-03
NM_016245	DHRS8	chr4:88615060-88615001	A_23_P21644	1.25	1.12	8.81E-10	1.60E-08
NM_012337	CCDC19	chr1:156659517-156659458	A_23_P62642	1.25	1.13	1.30E-05	8.15E-05
NM_013262	MYLIP	chr6:16251281-16251340	A_23_P31041	1.25	1.29	1.28E-06	7.09E-07
NM_000199	SGSH	chr17:75798048-75797989	A_23_P254254	1.25	1.30	9.82E-04	7.96E-04
NM_005258	GCHFR	chr15:38846802-38846861	A_23_P77328	1.25	1.31	3.79E-07	1.37E-07
NM_000483	APOC2	chr19:50144480-50144539	A_23_P208302	1.25	1.46	1.34E-02	5.20E-03
NM_004233	CD83	chr6:14244668-14244727	A_23_P70670	1.25	1.48	6.53E-08	8.25E-10
NM_031498	GNGT2	chr17:44639033-44638974	A_23_P26994	1.25	1.68	3.36E-10	1.64E-14
NM_145202	PRAP1	chr10:135054445-135054504	A_23_P202275	1.26	0.66	7.86E-09	1.52E-03
NM_003272	GPR137B	chr1:232697916-232697975	A_23_P46149	1.26	0.96	7.06E-06	5.53E-04
NM_003550	MAD1L1	chr7:1711229-1711170	A_23_P333735	1.26	1.02	4.32E-03	3.07E-02
NM_130777	XAGE2	chrX:51948736-51948795	A_23_P34031	1.26	1.08	8.09E-05	9.22E-04
THC2344809	THC2344809	chr19:57464453-57464394	A_32_P91507	1.26	1.21	1.39E-11	4.23E-11
NM_001124	ADM	chr11:10285356-10285415	A_23_P127948	1.26	1.22	1.26E-02	2.23E-02
NM_012258	HEY1	chr8:80838926-80838867	A_32_P83845	1.26	1.34	1.31E-08	2.25E-09
NM_144691	CAPN12	chr19:43916257-43916198	A_24_P79040	1.26	1.35	2.43E-06	5.95E-07

Gene	Symbol	Chromosomal position	Probe	siCASP8AP2.3	CASP8AP2.6	siCASP8AP2.3	CASP8AP2.6
				M	M	Q	Q
NM_005874	LILRB2	chr19:59470200-59470141	A_23_P208493	1.26	1.38	4.53E-13	1.67E-14
A_23_P123234	A_23_P123234	chr7:100374532-100374591	A_23_P123234	1.26	1.38	3.86E-04	1.39E-04
NM_152346	SLC43A2	chr17:1424511-1424452	A_24_P296508	1.26	1.42	6.51E-04	1.59E-04
ENST00000220507	ENST00000220507	chr15:38952216-38952157	A_23_P117912	1.26	1.45	3.12E-07	1.05E-08
NM_015432	PLEKHG4	chr16:65880735-65880794	A_23_P345460	1.26	1.46	1.18E-04	1.20E-05
NM_004933	CDH15	chr16:87789340-87789399	A_32_P25357	1.26	1.51	7.08E-05	3.19E-06
AF289566	AF289566	chr19:41495250-41495309	A_24_P375691	1.26	1.61	3.44E-14	2.88E-18
NM_153709	C1orf211	chr1:6233545-6233604	A_23_P343261	1.27	1.02	8.38E-14	1.12E-10
NM_006746	SCML1	chrX:17527209-17527268	A_24_P162319	1.27	1.06	6.61E-05	1.01E-03
NM_017622	C17orf59	chr17:8032452-8032393	A_23_P152955	1.27	1.09	6.46E-06	1.02E-04
NM_003387	WASPIP	chr2:175252637-175252578	A_23_P330611	1.27	1.10	8.44E-16	1.64E-13
NM_139172	MDAC1	chr19:60581178-60581237	A_23_P107775	1.27	1.23	1.56E-10	3.84E-10
NM_004474	FOXD2	chr1:47618277-47618336	A_23_P86171	1.27	1.23	2.18E-04	4.22E-04
NM_031908	C1QTNF2	chr5:159708882-159708823	A_23_P92903	1.27	1.24	1.33E-13	2.37E-13
Z74615	COL1A1	chr17:45616625-45616564	A_23_P207520	1.27	1.27	5.11E-10	5.44E-10
NM_078467	CDKN1A	chr6:36760156-36760215	A_24_P89457	1.27	1.28	7.14E-05	6.93E-05
NM_175735	LYG2	chr2:99319001-99317426	A_23_P5703	1.27	1.30	4.53E-11	2.30E-11
NM_000041	APOE	chr19:50104213-50104272	A_23_P164650	1.27	1.30	7.05E-03	8.17E-03
NM_020405	PLXDC1	chr17:34477001-34476942	A_23_P3911	1.27	1.35	1.48E-07	4.17E-08
NM_004004	GJB2	chr13:19659974-19659915	A_23_P204947	1.27	1.40	1.72E-07	1.55E-08
NM_198951	TGM2	chr20:36199919-36199860	A_24_P923251	1.27	1.42	3.25E-03	1.41E-03
NM_004004	GJB2	chr13:19659974-19659915	A_23_P204947	1.27	1.43	2.50E-07	1.29E-08
THC2333719	THC2333719	chrX:124184502-124184561	A_32_P86682	1.27	1.49	8.61E-09	9.69E-11
NM_004566	PFKFB3	chr10:6317068-6317127	A_24_P261259	1.27	1.57	1.42E-05	2.07E-07
BC007360	MGC16121	chrX:133404070-133404011	A_23_P159986	1.27	1.82	2.02E-04	3.45E-07
NM_001012421	ANKRD20A2	chr9:45558544-45558603	A_24_P64241	1.28	0.77	1.54E-05	1.06E-02
NM_004726	REPS2	chrX:16925285-16925344	A_23_P251387	1.28	0.95	1.21E-03	2.22E-02
NM_001001547	CD36	chr7:79947362-79947994	A_23_P111583	1.28	1.00	1.63E-07	2.83E-05
NM_182504	WBSCR28	chr7:72724387-72724601	A_23_P418274	1.28	1.08	5.53E-09	4.07E-07
BX108510	BX108510	chr3:197235374-197235315	A_24_P669329	1.28	1.19	6.61E-15	8.78E-14
NM_080818	OXGR1	chr13:96436202-96436143	A_24_P39195	1.28	1.28	7.06E-08	8.14E-08
NM_002429	MMP19	chr12:54516581-54516522	A_23_P203882	1.28	1.29	2.43E-04	2.81E-04
NM_004004	GJB2	chr13:19659974-19659915	A_23_P204947	1.28	1.45	1.15E-07	4.69E-09
NM_001275	CHGA	chr14:92471071-92471130	A_32_P27046	1.28	1.51	2.36E-06	6.38E-08
NM_025217	ULBP2	chr6:150358732-150358791	A_24_P149314	1.28	1.59	6.67E-07	3.54E-09
NM_004004	GJB2	chr13:19660317-19660258	A_23_P204941	1.28	1.59	5.46E-06	4.80E-08
NM_014224	PGA5	chr11:60772510-60772569	A_23_P150549	1.28	1.78	1.32E-08	7.06E-13
NM_000782	CYP24A1	chr20:52204308-52204249	A_24_P356930	1.28	1.97	5.58E-04	4.17E-07
NM_144964	RG9MTD3	chr9:37766355-37767609	A_23_P400459	1.29	0.72	9.33E-08	2.07E-03
NM_004192	ASMTL	chrX:1566007-1565964	A_23_P159539	1.29	1.08	3.34E-07	1.32E-05
NM_032048	EMILIN2	chr18:2903979-2904038	A_23_P27315	1.29	1.14	7.03E-07	9.28E-06
BC002831	MGC4294	chr20:56644047-56644106	A_23_P102681	1.29	1.26	6.41E-07	1.12E-06
NM_176072	P2RY2	chr11:72624564-72624623	A_23_P24903	1.29	1.27	2.72E-08	4.42E-08
Z74615	COL1A1	chr17:45616625-45616564	A_23_P207520	1.29	1.28	1.25E-08	1.54E-08
NM_000625	NOS2A	chr17:23108048-23107989	A_23_P502464	1.29	1.28	5.93E-08	8.03E-08
NM_000041	APOE	chr19:50104213-50104272	A_23_P164650	1.29	1.32	7.14E-03	8.32E-03
NM_006144	GZMA	chr5:54439927-54441612	A_23_P133445	1.29	1.35	7.99E-07	3.10E-07
NM_018602	DNAJA4	chr15:76354965-76355024	A_23_P129279	1.29	1.44	2.62E-10	8.01E-12
NM_000250	MPO	chr17:53702926-53702867	A_23_P141173	1.29	1.46	3.35E-18	1.72E-20
ENST00000339489	ENST00000339489	chr17:34462723-34462782	A_24_P848707	1.29	1.54	7.37E-15	7.56E-18
NM_012168	FBXO2	chr1:11642883-11642824	A_23_P45999	1.29	1.57	7.06E-05	2.36E-06
NM_000782	CYP24A1	chr20:52204412-52204353	A_23_P28815	1.29	1.84	4.58E-04	1.58E-06
NM_001823	CKB	chr14:103056278-103056219	A_23_P25674	1.29	2.20	4.15E-03	2.77E-06
ENST00000267857	ENST00000267857	chr15:57699411-57699470	A_23_P151915	1.30	0.66	2.04E-05	3.64E-02
NM_000603	NOS3	chr7:150148081-150148484	A_23_P70849	1.30	0.83	9.94E-08	3.87E-04
NM_203411	TMEM88	chr17:7700007-7700066	A_23_P77859	1.30	0.96	4.32E-06	5.95E-04

Gene	Symbol	Chromosomal position	Probe	siCASP8AP2.3	CASP8AP2.6	siCASP8AP2.3	CASP8AP2.6
				M	M	Q	Q
BX375060	BX375060	chr19:1252020-1252079	A_32_P9368	1.30	0.96	4.39E-06	6.42E-04
NM_014732	KIAA0513	chr16:83685140-83685199	A_23_P206310	1.30	1.00	2.88E-11	4.75E-08
NM_021064	HIST1H2AG	chr6:27209136-27209195	A_24_P303354	1.30	1.08	2.26E-10	3.92E-08
NM_001554	CYR61	chr1:85761192-85761251	A_23_P46426	1.30	1.09	1.03E-02	4.56E-02
NM_031453	FAM107B	chr10:14601842-14601783	A_23_P149975	1.30	1.11	2.72E-03	1.46E-02
A_24_P755069	A_24_P755069	chr9:087618060-087618417	A_24_P755069	1.30	1.23	7.69E-06	2.25E-05
NM_001005364	POTE2	chr2:131844334-131844393	A_24_P825874	1.30	1.34	4.22E-07	2.44E-07
NM_024579	C1orf54	chr1:147066324-147066383	A_23_P74778	1.30	1.37	7.43E-04	4.81E-04
NM_172037	RDH10	chr8:74399014-74399073	A_32_P25050	1.30	1.42	1.89E-07	2.09E-08
NM_207372	SH2D4B	chr10:82393858-82393917	A_24_P417904	1.30	1.80	1.99E-11	1.68E-16
NM_003317	TITF1	chr14:36055528-36055469	A_24_P61490	1.30	1.84	1.81E-05	9.49E-09
NM_033277	LACRT	chr12:53311834-53310991	A_23_P64825	1.31	0.71	3.08E-18	4.72E-09
NM_000603	NOS3	chr7:150148081-150148484	A_23_P70849	1.31	0.82	5.56E-09	9.88E-05
AL834308	C1orf167	chr1:11783838-11783897	A_32_P60145	1.31	0.84	1.24E-06	1.50E-03
NM_000176	NR3C1	chr5:142641189-142641130	A_23_P214059	1.31	0.86	1.74E-04	1.80E-02
NM_004672	MAP3K6	chr1:27365881-27365822	A_24_P145653	1.31	0.89	6.10E-09	2.83E-05
NM_009587	LGALS9	chr17:23000630-23000689	A_32_P452655	1.31	0.96	6.13E-05	3.67E-03
NM_016245	DHRS8	chr4:88615605-88615546	A_23_P408271	1.31	1.08	4.55E-08	3.67E-06
NM_203311	CSAG3A	chrX:151599075-151599134	A_24_P79529	1.31	1.14	2.61E-13	2.79E-11
NM_000041	APOE	chr19:50104213-50104272	A_23_P164650	1.31	1.19	6.43E-03	1.87E-02
NM_016445	PLEK2	chr14:66923623-66923564	A_23_P151506	1.31	1.21	2.39E-11	2.38E-10
NM_004004	GJB2	chr13:19659974-19659915	A_23_P204947	1.31	1.31	2.11E-07	2.39E-07
NM_002526	NT5E	chr6:86233711-86237724	A_24_P316430	1.31	1.43	1.21E-03	5.54E-04
AK124344	AK124344	chr17:22976296-22976355	A_24_P911112	1.31	1.97	5.47E-03	4.86E-05
NM_000565	IL6R	chr1:151251989-151252048	A_24_P379413	1.32	1.10	1.18E-03	9.58E-03
BG216262	BG216262	chr3:126999332-126999391	A_32_P133090	1.32	1.18	8.57E-06	6.60E-05
NM_000041	APOE	chr19:50104213-50104272	A_23_P164650	1.32	1.31	5.00E-03	7.30E-03
NM_003741	CHRD	chr3:185590195-185590254	A_23_P502047	1.32	1.35	2.55E-07	1.53E-07
NM_001741	CALCA	chr11:14946823-14946764	A_23_P301846	1.32	1.36	1.61E-13	5.38E-14
NM_004004	GJB2	chr13:19659974-19659915	A_23_P204947	1.32	1.40	3.13E-08	7.91E-09
NM_030568	C6orf148	chr6:73975495-73975436	A_24_P20814	1.32	1.49	7.18E-10	1.72E-11
NM_199511	CCDC80	chr3:113807225-113807166	A_23_P58082	1.32	1.53	3.00E-06	1.44E-07
BC110641	MGC102966	chr17:20346806-20346747	A_32_P62963	1.32	1.66	3.02E-07	9.48E-10
NM_004566	PFKFB3	chr10:6306172-6308209	A_24_P206604	1.33	1.01	4.00E-06	4.25E-04
AK094968	IFI16	chr1:155836516-155836575	A_23_P217866	1.33	1.05	1.74E-15	6.03E-12
NM_005442	EOMES	chr3:27733204-27733145	A_24_P97374	1.33	1.05	4.62E-11	3.79E-08
NM_001624	AIM1	chr6:107124456-107124515	A_23_P70785	1.33	1.17	2.01E-06	2.39E-05
NM_152621	MGC26963	chr4:109189305-109189364	A_23_P326204	1.33	1.19	5.42E-07	5.99E-06
BX648200	BHLHB8	chr7:97489268-97489327	A_23_P111525	1.33	1.25	8.05E-04	2.25E-03
NM_001079	ZAP70	chr2:97813041-97814367	A_24_P169234	1.33	1.41	1.68E-03	1.15E-03
NM_001195	BFSP1	chr20:17422629-17422570	A_23_P109171	1.33	1.49	1.95E-09	6.63E-11
NM_006291	TNFAIP2	chr14:102673370-102673429	A_23_P421423	1.33	1.51	1.43E-03	4.17E-04
NM_004004	GJB2	chr13:19659974-19659915	A_23_P204947	1.33	1.57	7.96E-08	1.02E-09
NM_144626	TMEM125	chr1:43408698-43408757	A_23_P115022	1.33	1.57	9.46E-07	2.08E-08
NM_003407	ZFP36	chr19:44591598-44591657	A_23_P39237	1.33	1.57	1.10E-05	4.64E-07
NM_000176	NR3C1	chr5:142641189-142641130	A_23_P214059	1.34	0.78	8.54E-05	2.79E-02
NM_198232	RNASE1	chr14:20339901-20339842	A_24_P403959	1.34	0.79	2.98E-11	1.57E-05
NM_020163	SEMA3G	chr3:52443241-52443182	A_23_P6818	1.34	0.95	6.35E-07	3.18E-04
NM_000603	NOS3	chr7:150148081-150148484	A_23_P70849	1.34	1.00	3.87E-08	1.93E-05
NM_181718	ASPHD1	chr16:29824745-29824804	A_23_P170667	1.34	1.06	7.75E-04	1.04E-02
NM_001838	CCR7	chr17:35963645-35963586	A_23_P343398	1.34	1.12	4.00E-03	2.19E-02
NM_000041	APOE	chr19:50104213-50104272	A_23_P164650	1.34	1.13	5.16E-03	2.67E-02
NM_020877	DNHD3	chr17:7677655-7677714	A_32_P206899	1.34	1.20	1.27E-05	9.63E-05
NM_002872	RAC2	chr22:35945923-35945864	A_23_P218774	1.34	1.20	1.20E-04	6.69E-04
NM_001124	ADM	chr11:10285356-10285415	A_23_P127948	1.34	1.21	8.49E-03	2.50E-02
NM_000041	APOE	chr19:50104213-50104272	A_23_P164650	1.34	1.24	4.73E-03	1.21E-02

Gene	Symbol	Chromosomal position	Probe	siCASP8AP2.3	CASP8AP2.6	siCASP8AP2.3	CASP8AP2.6
				M	M	Q	Q
A_32_P213948	A_32_P213948	chr17:001891061-001891002	A_32_P213948	1.34	1.26	5.26E-09	2.76E-08
NM_004024	ATF3	chr1:209181232-209181291	A_24_P33895	1.34	1.28	1.65E-04	3.96E-04
Z74615	COL1A1	chr17:45616625-45616564	A_23_P207520	1.34	1.41	1.05E-10	2.00E-11
NM_020127	TUFT1	chr1:148368937-148368996	A_23_P371824	1.34	1.42	9.57E-10	1.77E-10
A_24_P16353	A_24_P16353	chr11:089366996-089366937	A_24_P16353	1.34	1.70	3.72E-11	1.10E-14
NM_001216	CA9	chr9:35667817-35669204	A_23_P157793	1.34	1.72	6.66E-03	6.80E-04
NM_004360	CDH1	chr16:67426715-67426774	A_23_P206359	1.35	0.99	8.13E-09	8.99E-06
NM_004360	CDH1	chr16:67426715-67426774	A_23_P206359	1.35	1.02	1.75E-08	9.27E-06
NM_182603	ANKRD42	chr11:82636687-82636746	A_32_P69166	1.35	1.17	1.03E-08	3.49E-07
NM_003745	SOCS1	chr16:11255944-11255885	A_23_P420196	1.35	1.23	1.41E-05	8.42E-05
NM_000041	APOE	chr19:50104213-50104272	A_23_P164650	1.35	1.25	4.25E-03	1.16E-02
XM_379036	LOC400890	chr22:19643410-19643469	A_32_P73413	1.35	1.26	4.60E-07	2.03E-06
NM_020181	C14orf162	chr14:69106511-69106452	A_23_P14295	1.35	1.32	2.06E-10	3.25E-10
NM_139176	NALP7	chr19:60126909-60126850	A_23_P16384	1.35	1.34	2.11E-06	2.76E-06
NM_004004	GJB2	chr13:19659974-19659915	A_23_P204947	1.35	1.36	3.59E-07	2.98E-07
NM_000558	HBA1	chr16:167382-167441	A_23_P37856	1.35	1.42	1.58E-03	1.19E-03
NM_003106	SOX2	chr3:182913448-182913507	A_24_P379969	1.35	1.45	2.94E-04	1.31E-04
NM_178840	C1orf64	chr1:16078259-16078318	A_32_P232455	1.35	1.92	1.53E-08	2.76E-13
NM_000603	NOS3	chr7:150148081-150148484	A_23_P70849	1.36	0.76	1.99E-07	2.77E-03
AY358103	AY358103	chr7:75007475-75007416	A_24_P934455	1.36	0.79	1.59E-09	1.63E-04
NM_006454	MXD4	chr4:2218570-2218511	A_23_P423695	1.36	0.80	4.01E-06	6.37E-03
NM_002722	PPY	chr17:39374038-39373789	A_23_P207336	1.36	1.05	2.03E-09	1.12E-06
NM_033229	TRIM15	chr6:30248382-30248441	A_23_P500300	1.36	1.06	1.81E-04	4.54E-03
NM_033027	AXUD1	chr3:39159223-39159164	A_23_P121011	1.36	1.13	5.27E-04	5.16E-03
NM_016113	TRPV2	chr17:16280951-16281010	A_23_P207911	1.36	1.29	1.09E-03	2.47E-03
L06175	HCP5	chr6:31541152-31541212	A_23_P111126	1.36	1.40	1.22E-11	4.69E-12
L33930	CD24	chrY:19541002-19540943	A_23_P85250	1.36	1.90	3.71E-05	4.23E-08
NM_001038633	RSPO1	chr1:37746206-37746147	A_23_P22013	1.37	0.70	7.96E-06	2.60E-02
NM_012202	GNG3	chr11:62232383-62232442	A_23_P139192	1.37	0.79	3.44E-11	2.55E-05
NM_000603	NOS3	chr7:150148081-150148484	A_23_P70849	1.37	0.84	1.69E-07	9.89E-04
NM_001012334	MDK	chr11:46361807-46361866	A_23_P116235	1.37	1.05	1.73E-05	9.72E-04
NM_016140	CGI-38	chr16:65981890-65981715	A_23_P26386	1.37	1.07	5.99E-08	1.31E-05
NM_152612	CCDC116	chr22:20316085-20316144	A_23_P342108	1.37	1.25	4.36E-11	6.26E-10
NM_000625	NOS2A	chr17:23108048-23107989	A_23_P502464	1.37	1.29	2.79E-08	1.27E-07
NM_144947	KLK11	chr19:56217497-56217438	A_23_P101505	1.37	1.72	1.88E-10	8.71E-14
A_32_P117908	A_32_P117908	chr12:109356493-109356552	A_32_P117908	1.37	1.86	1.92E-15	9.68E-21
NM_001002233	RAB11FIP1	chr8:37837453-37837394	A_23_P31873	1.37	1.88	9.88E-10	3.82E-14
NM_052906	KIAA1904	chr22:36088746-36088687	A_32_P219279	1.38	0.91	1.49E-04	1.69E-02
NM_032148	SLC41A2	chr12:103701377-103701318	A_23_P204801	1.38	1.00	1.04E-08	1.31E-05
BX101288	BX101288	chr3:57713999-57713940	A_32_P34696	1.38	1.02	4.84E-06	6.80E-04
NM_080792	SIRPA	chr20:1868284-1868343	A_23_P210708	1.38	1.04	3.01E-09	2.62E-06
NM_001165	BIRC3	chr11:101713566-101713625	A_23_P98350	1.38	1.15	2.05E-03	1.39E-02
NM_001124	ADM	chr11:10285356-10285415	A_23_P127948	1.38	1.19	7.84E-03	3.03E-02
NM_024902	FLJ13236	chr12:48031617-48031676	A_23_P253661	1.38	1.20	5.41E-04	3.43E-03
NM_030753	WNT3	chr17:42196932-42196873	A_23_P130158	1.38	1.21	1.06E-05	1.15E-04
NM_152303	ZNF554	chr19:2785881-2785940	A_23_P343250	1.38	1.21	9.79E-05	7.53E-04
NM_000041	APOE	chr19:50104213-50104272	A_23_P164650	1.38	1.22	3.25E-03	1.31E-02
NM_000835	GRIN2C	chr17:70349858-70349799	A_23_P49546	1.38	1.25	1.67E-15	5.50E-14
NM_024307	GDPD3	chr16:30031167-30031024	A_23_P26511	1.38	1.31	2.43E-05	6.41E-05
NM_175616	FIS	chr5:95218421-95220302	A_23_P408323	1.38	1.90	7.14E-12	7.50E-17
NM_001432	EREG	chr4:75619044-75619103	A_23_P41344	1.38	2.02	2.62E-03	2.15E-05
NM_000176	NR3C1	chr5:142641189-142641130	A_23_P214059	1.39	0.79	8.17E-05	3.38E-02
BC030768	BC030768	chr1:26238538-26238597	A_24_P213827	1.39	0.92	3.36E-06	1.99E-03
NM_152351	SLC5A10	chr17:18864608-18864667	A_23_P328022	1.39	0.95	5.96E-10	6.67E-06
NM_001124	ADM	chr11:10285356-10285415	A_23_P127948	1.39	1.25	6.89E-03	2.16E-02
NM_001753	CAV1	chr7:115792952-115793011	A_24_P12626	1.39	1.27	6.95E-05	3.07E-04

Gene	Symbol	Chromosomal position	Probe	siCASP8AP2.3	CASP8AP2.6	siCASP8AP2.3	CASP8AP2.6
				M	M	Q	Q
A_24_P845631	A_24_P845631		A_24_P845631	1.39	1.29	1.03E-06	5.30E-06
NM_001124	ADM	chr11:10285356-10285415	A_23_P127948	1.39	1.29	5.01E-03	1.31E-02
NM_001124	ADM	chr11:10285356-10285415	A_23_P127948	1.39	1.30	5.63E-03	1.36E-02
NM_021194	SLC30A1	chr1:208137296-208137237	A_23_P23815	1.39	1.34	1.39E-06	3.00E-06
NM_000041	APOE	chr19:50104213-50104272	A_23_P164650	1.39	1.35	1.97E-03	3.49E-03
NM_001185	AZGP1	chr7:99209429-99209370	A_23_P71270	1.39	1.38	1.92E-05	2.62E-05
NM_001040066	PJCG6	chr8:7091961-7091902	A_32_P352358	1.39	1.49	3.47E-13	3.31E-14
NM_032588	TRIM63	chr1:26062043-26061984	A_23_P114983	1.39	1.51	6.18E-07	9.91E-08
NM_198098	AQP1	chr7:30738297-30738355	A_23_P372834	1.39	1.94	5.48E-09	1.52E-13
NM_000073	CD3G	chr11:117729141-117729200	A_23_P98410	1.40	0.64	1.22E-07	1.37E-02
NM_000176	NR3C1	chr5:142641189-142641130	A_23_P214059	1.40	0.80	5.64E-05	2.82E-02
BC047708	BC047708	chr7:29500142-29500201	A_32_P73139	1.40	1.03	1.10E-11	7.30E-08
NM_000418	IL4R	chr16:27283500-27283559	A_23_P129556	1.40	1.20	2.17E-12	2.30E-10
NM_213602	CD33L3	chr18:41676459-41676517	A_23_P50146	1.40	1.26	8.62E-07	8.34E-06
NM_000041	APOE	chr19:50104213-50104272	A_23_P164650	1.40	1.32	2.87E-03	6.79E-03
BC032041	BC032041	chr18:14080800-14080859	A_32_P85779	1.40	1.36	2.82E-18	9.12E-18
NM_015393	DKFZP564O0823	chr4:76329588-76329647	A_23_P212779	1.40	1.38	7.96E-06	1.17E-05
NM_006928	SILV	chr12:54634304-54634245	A_23_P2233	1.40	1.47	8.11E-09	2.27E-09
NM_017534	MYH2	chr17:10365357-10365298	A_23_P38271	1.40	1.56	1.84E-11	4.33E-13
NM_002135	NR4A1	chr12:50739445-50739504	A_23_P128230	1.40	1.65	7.13E-04	9.60E-05
NM_000176	NR3C1	chr5:142641189-142641130	A_23_P214059	1.41	0.83	1.16E-04	2.97E-02
NM_004936	CDKN2B	chr9:21999038-21998979	A_23_P216812	1.41	0.97	4.09E-05	5.59E-03
A_32_P174385	A_32_P174385	chr5:108092085-108092144	A_32_P174385	1.41	1.07	4.40E-09	3.27E-06
NM_000625	NOS2A	chr17:23108048-23107989	A_23_P502464	1.41	1.29	1.92E-07	1.44E-06
AK054935	TTC9B	chr19:45414032-45413973	A_23_P332392	1.41	1.31	2.14E-12	1.93E-11
AK131529	AATK	chr17:76705849-76705790	A_23_P10559	1.41	1.33	3.42E-04	9.22E-04
NM_007074	CORO1A	chr16:30106818-30106877	A_23_P106761	1.41	1.44	4.01E-05	3.41E-05
NM_002842	PTPRH	chr19:60384639-60384580	A_23_P101642	1.41	1.51	5.74E-06	1.45E-06
BC035960	PTPRO	chr12:15561113-15561172	A_24_P76809	1.41	1.71	1.91E-10	3.64E-13
NM_005860	FSTL3	chr19:633846-633905	A_23_P209167	1.41	1.75	4.29E-08	1.12E-10
NM_014365	HSPB8	chr12:118080184-118087588	A_23_P162579	1.42	0.88	5.01E-06	4.98E-03
NM_007197	FZD10	chr12:129175054-129175113	A_23_P203972	1.42	1.18	7.29E-08	4.07E-06
THC2274685	THC2274685	chr14:99680174-99680115	A_32_P224040	1.42	1.19	3.28E-13	9.83E-11
NM_000493	COL10A1	chr6:116547813-116547754	A_23_P214144	1.42	1.22	1.32E-12	1.43E-10
A_32_P169353	A_32_P169353	chr2:232090413-232090354	A_32_P169353	1.42	1.27	8.84E-10	1.70E-08
ENST00000343905	ENST00000343905	chrX:118675925-118675984	A_32_P123255	1.42	1.42	2.63E-09	2.62E-09
NM_018948	ERRFI1	chr1:8006533-8006474	A_23_P46470	1.42	1.66	4.07E-07	9.08E-09
CR603982	CR603982	chr11:94538581-94538522	A_32_P146635	1.42	1.86	9.27E-04	2.36E-05
NM_017527	LY6K	chr8:143781823-143781882	A_23_P397293	1.42	2.08	8.58E-04	2.80E-06
NM_001558	IL10RA	chr11:117377280-117377339	A_23_P203173	1.42	2.26	7.57E-07	2.19E-12
NM_031961	KRTAP9-2	chr17:36648514-36648573	A_23_P89646	1.43	0.81	4.81E-12	1.08E-05
NM_000559	HBG1	chr11:5226169-5226110	A_23_P53137	1.43	1.01	1.92E-14	1.21E-09
AB040974	AB040974	chr10:133623178-133623237	A_24_P940218	1.43	1.25	5.44E-05	4.83E-04
NM_001122	ADFP	chr9:19106429-19106370	A_23_P134953	1.43	1.37	7.91E-07	2.01E-06
NM_015689	DENND2A	chr7:139671702-139671643	A_23_P257583	1.43	1.44	6.80E-14	4.82E-14
BC028228	FLJ40125	chr19:50697298-50697357	A_23_P141893	1.43	1.45	8.28E-11	5.05E-11
AK123302	AK123302	chrX:133954636-133954578	A_32_P104432	1.43	1.46	3.22E-09	1.93E-09
CR601458	CR601458	chr2:28915-28856	A_32_P47643	1.43	1.54	4.66E-10	4.18E-11
NM_004364	CEBPA	chr19:38483252-38483193	A_24_P224727	1.43	1.94	1.92E-04	1.12E-06
NM_000603	NOS3	chr7:150148081-150148484	A_23_P70849	1.44	0.84	4.20E-07	2.77E-03
NM_144586	LYPD1	chr2:133236222-133236163	A_32_P101031	1.44	0.89	7.10E-05	1.79E-02
THC2369034	THC2369034	chr19:47958027-47958854	A_32_P11276	1.44	1.05	3.19E-07	1.20E-04
NM_032251	CCDC88	chr11:63879654-63881123	A_23_P24389	1.44	1.11	1.24E-12	3.55E-09
NM_003106	SOX2	chr3:182914579-182914638	A_23_P401055	1.44	1.13	1.64E-04	3.61E-03
ENST00000321715	ENST00000321715	chr14:105137591-105137532	A_24_P109921	1.44	1.24	1.84E-04	1.57E-03
NM_000625	NOS2A	chr17:23108048-23107989	A_23_P502464	1.44	1.27	3.36E-08	7.03E-07

Gene	Symbol	Chromosomal position	Probe	siCASP8AP2.3	CASP8AP2.6	siCASP8AP2.3	CASP8AP2.6
				M	M	Q	Q
NM_001718	BMP6	chr6:7826394-7826453	A_23_P19624	1.44	1.39	1.61E-07	4.41E-07
NM_001874	CPM	chr12:67536304-67536245	A_23_P162668	1.44	1.60	4.19E-10	1.73E-11
NM_001017920	LOC92196	chr2:159497908-159497967	A_23_P165598	1.44	1.72	5.42E-06	1.33E-07
AK094730	LOC283454	chr12:115756784-115756725	A_32_P170454	1.45	0.89	2.69E-04	3.53E-02
NM_020209	SHD	chr19:4241646-4241705	A_23_P142255	1.45	1.17	1.22E-05	4.16E-04
NR_002185	OR7E91P	chr2:71168119-71168178	A_23_P433218	1.45	1.19	2.60E-10	5.62E-08
NM_001124	ADM	chr11:10285356-10285415	A_23_P127948	1.45	1.22	4.77E-03	2.59E-02
NM_000043	FAS	chr10:90764525-90764584	A_23_P63896	1.45	1.27	1.71E-05	1.76E-04
AF067801	AF067801	chr5:103587358-103587299	A_23_P92809	1.45	1.29	5.66E-10	1.47E-08
NM_000625	NOS2A	chr17:23108048-23107989	A_23_P502464	1.45	1.29	2.72E-08	4.59E-07
NM_006671	SLC1A7	chr1:53264989-53264930	A_23_P325562	1.45	1.38	4.28E-10	1.83E-09
NM_002571	PAEP	chr9:135682036-135682095	A_23_P257129	1.45	1.43	4.76E-10	7.84E-10
NM_000517	HBA2	chr16:163560-163619	A_23_P26457	1.45	1.63	9.84E-04	3.01E-04
NM_181607	KRTAP19-1	chr21:30774343-30774284	A_24_P367242	1.45	1.72	4.22E-09	3.01E-11
NM_002407	SCGB2A1	chr11:61737920-61737980	A_23_P312300	1.45	1.79	3.31E-16	6.20E-20
NM_019859	HTR7	chr10:92490823-92490764	A_23_P500381	1.45	1.79	3.20E-12	1.55E-15
NM_017809	NXF2	chrX:101387714-101387773	A_23_P148568	1.45	2.29	3.55E-07	8.10E-13
NM_000176	NR3C1	chr5:142641189-142641130	A_23_P214059	1.46	0.80	5.21E-05	3.33E-02
NM_000625	NOS2A	chr17:23108048-23107989	A_23_P502464	1.46	1.21	1.19E-08	9.73E-07
NM_005588	MEP1A	chr6:46911141-46911200	A_24_P154868	1.46	1.24	2.49E-19	1.80E-16
NM_004909	CSAG2	chrX:151547453-151547397	A_24_P567298	1.46	1.30	7.03E-22	1.02E-19
NM_000625	NOS2A	chr17:23108048-23107989	A_23_P502464	1.46	1.33	2.55E-08	2.83E-07
THC2442021	THC2442021	chr6:168272470-168272411	A_32_P174285	1.46	1.36	5.02E-15	5.82E-14
NM_003279	TNNC2	chr20:43886046-43885376	A_23_P131825	1.46	1.39	7.76E-13	3.89E-12
NM_002965	S100A9	chr1:150143947-150146216	A_23_P23048	1.46	1.45	1.82E-16	2.17E-16
ENST00000327669	ENST00000327669	chr2:35533-35474	A_24_P50248	1.46	1.46	9.47E-13	8.57E-13
NM_005293	GPR20	chr8:142435886-142435827	A_23_P159237	1.46	1.50	4.81E-11	2.29E-11
NM_000176	NR3C1	chr5:142641189-142641130	A_23_P214059	1.47	0.87	6.32E-05	2.26E-02
NM_145755	TTC21A	chr3:39155313-39155372	A_23_P428248	1.47	0.97	6.54E-15	4.05E-09
BC037255	LOC389634	chr8:12561140-12561081	A_32_P148914	1.47	0.98	5.36E-12	3.94E-07
A_24_P400702	A_24_P400702	chr1:116994839-116994780	A_24_P400702	1.47	1.02	8.09E-06	2.07E-03
NM_004997	MYBPH	chr1:199868676-199868617	A_23_P148737	1.47	1.02	5.83E-05	6.38E-03
A_32_P52153	A_32_P52153	chr10:072647071-072647012	A_32_P52153	1.47	1.10	4.23E-05	2.40E-03
NM_000681	ADRA2A	chr10:112830342-112830401	A_23_P138706	1.47	1.16	1.22E-16	5.94E-13
NM_020820	PREX1	chr20:46675084-46675025	A_23_P413641	1.47	1.40	1.07E-10	4.46E-10
NM_000962	PTGS1	chr9:122234606-122234665	A_23_P216966	1.47	1.42	5.75E-13	1.54E-12
NM_004093	EFNB2	chr13:105940796-105940737	A_24_P355944	1.47	1.42	2.29E-11	6.27E-11
NM_030949	PPP1R14C	chr6:150663051-150663110	A_23_P45011	1.47	1.49	1.27E-05	1.11E-05
NM_138732	NRXN2	chr11:64131419-64131360	A_24_P261470	1.47	1.62	2.48E-09	1.61E-10
NM_199206	TCL1B	chr14:95228620-95228679	A_23_P48495	1.47	1.72	2.52E-06	7.93E-08
NM_020804	PACSIN1	chr6:34610842-34610901	A_23_P258088	1.47	1.72	1.51E-05	7.14E-07
NM_080588	PTPN7	chr1:198848161-198848102	A_23_P201778	1.47	1.76	3.86E-07	4.72E-09
NM_005269	GLI1	chr12:56152238-56152297	A_23_P105251	1.47	1.81	5.42E-13	2.81E-16
NM_000606	C8G	chr9:137116840-137117061	A_23_P20713	1.47	2.05	2.25E-11	1.80E-16
ENST00000357303	ENST00000357303	chr2:238859434-238859494	A_23_P364504	1.47	2.14	2.13E-09	1.12E-14
NM_032829	C12orf34	chr12:108670771-108670830	A_23_P128375	1.48	0.85	1.33E-08	6.29E-04
NM_173582	PGM2L1	chr11:73720314-73720255	A_32_P122703	1.48	1.09	1.90E-06	3.68E-04
NM_000625	NOS2A	chr17:23108048-23107989	A_23_P502464	1.48	1.25	1.42E-08	9.06E-07
NM_018058	CRTAC1	chr10:99630084-99630025	A_23_P431971	1.48	1.33	5.42E-13	1.98E-11
NM_017625	ITLN1	chr1:157659498-157659439	A_23_P95790	1.48	1.54	8.06E-21	1.74E-21
NM_002201	ISG20	chr15:86999788-86999847	A_23_P32404	1.48	1.58	6.77E-06	2.16E-06
NM_006928	SILV	chr12:54635339-54634356	A_23_P312851	1.48	1.66	1.39E-09	4.69E-11
NM_001772	CD33	chr19:56434663-56434722	A_24_P301655	1.48	1.83	5.04E-11	3.82E-14
NM_031272	TEX14	chr17:54062858-54055372	A_32_P126079	1.48	2.00	3.63E-15	2.21E-20
THC2314858	THC2314858	chr14:68094036-68093977	A_32_P81623	1.49	1.22	4.79E-04	5.67E-03
BC026998	BC026998	chr17:18233534-18233593	A_32_P141682	1.49	1.23	1.99E-13	1.03E-10

Gene	Symbol	Chromosomal position	Probe	siCASP8AP2.3	CASP8AP2.6	siCASP8AP2.3	CASP8AP2.6
				M	M	Q	Q
BC029038	BC029038	chr12:120301521-120301580	A_32_P70420	1.49	1.25	1.23E-09	1.14E-07
NM_022359	PDE4DIP	chr1:142444282-142441697	A_23_P149153	1.49	1.40	4.92E-12	4.12E-11
ENST00000341016	ENST00000341016	chrX:37606393-37606452	A_24_P281872	1.49	1.65	1.21E-13	2.91E-15
NM_170672	RASGRP3	chr2:33700945-33701004	A_24_P54390	1.49	1.75	2.55E-04	2.82E-05
NM_001561	TNFRSF9	chr1:7915048-7914989	A_23_P51936	1.49	1.80	7.40E-09	3.15E-11
NM_152599	FLJ35773	chr17:8641295-8641236	A_23_P340218	1.50	0.92	1.69E-09	7.66E-05
NM_021127	PMAIP1	chr18:55721258-55721317	A_23_P207999	1.50	1.03	1.51E-03	4.20E-02
BC034407	LOC643201	chr5:175545929-175545870	A_24_P914043	1.50	1.09	2.95E-08	2.54E-05
NM_006718	PLAGL1	chr6:144303811-144303752	A_23_P8175	1.50	1.15	2.74E-09	1.78E-06
NM_153498	CAMK1D	chr10:12911088-12911147	A_32_P208120	1.50	1.38	3.12E-10	3.57E-09
NM_013376	SERTAD1	chr19:45620334-45620275	A_23_P218463	1.50	1.48	7.72E-11	1.16E-10
NM_000603	NOS3	chr7:150148081-150148484	A_23_P70849	1.51	0.81	2.09E-09	6.36E-04
NM_002390	ADAM11	chr17:40214391-40214450	A_23_P207345	1.51	0.85	3.85E-10	1.30E-04
NM_133467	CITED4	chr1:40995887-40995828	A_32_P209230	1.51	0.88	2.54E-06	6.18E-03
NM_005532	IFI27	chr14:93652682-93652741	A_23_P48513	1.51	1.05	3.72E-07	3.16E-04
NM_000625	NOS2A	chr17:23108048-23107989	A_23_P502464	1.51	1.21	5.56E-09	1.20E-06
NM_017851	PARP16	chr15:63338025-63337966	A_23_P163278	1.51	1.38	2.83E-07	2.13E-06
AB075837	KIAA1957	chr19:356563-356504	A_32_P82895	1.51	1.51	4.07E-05	4.38E-05
NM_001185	AZGP1	chr7:99209214-99209155	A_23_P71268	1.51	1.54	2.88E-05	2.26E-05
NM_014290	TDRD7	chr9:97337791-97337850	A_23_P123672	1.51	1.57	2.27E-05	1.27E-05
NM_004364	CEBPA	chr19:38483047-38482988	A_23_P375494	1.51	1.93	1.32E-04	2.07E-06
NM_003722	TP73L	chr3:191097665-191097724	A_23_P327380	1.51	2.07	1.97E-06	8.82E-10
NM_005954	MT3	chr16:55182294-55182353	A_23_P315273	1.52	0.79	5.18E-08	3.44E-03
ENST00000371310	ENST00000371310	chrX:119790749-119790690	A_24_P221497	1.52	0.83	2.02E-05	2.40E-02
NM_021127	PMAIP1	chr18:55721258-55721317	A_23_P207999	1.52	1.02	1.57E-03	4.85E-02
THC2280799	THC2280799	chr1:58993662-58993721	A_32_P3932	1.52	1.14	5.13E-09	4.27E-06
CR597260	CR597260	chr17:35936273-35936332	A_32_P44349	1.52	1.38	8.56E-10	1.08E-08
NM_001005377	PLAUR	chr19:48861397-48861338	A_23_P16469	1.52	1.53	3.04E-04	3.67E-04
CB959193	CB959193	chr11:061737860-061737915	A_23_P139166	1.52	1.60	3.75E-12	6.45E-13
NM_181709	FAM101A	chr12:123325339-123325398	A_32_P200697	1.52	1.69	6.50E-08	4.05E-09
NM_021127	PMAIP1	chr18:55721258-55721317	A_23_P207999	1.53	1.13	8.61E-04	1.88E-02
NM_053044	HTRA3	chr4:8426803-8426862	A_23_P395438	1.53	1.39	8.68E-10	1.12E-08
BX101252	BX101252	chr14:70443203-70443145	A_32_P56463	1.53	1.56	2.80E-10	1.42E-10
NM_145653	TCEB3C	chr18:42808924-42808865	A_23_P315910	1.53	1.85	5.57E-11	8.98E-14
NM_000729	CCK	chr3:42274380-42274322	A_23_P425681	1.53	2.52	4.80E-13	1.61E-21
NM_021127	PMAIP1	chr18:55721258-55721317	A_23_P207999	1.54	1.10	7.62E-04	2.21E-02
NM_000389	CDKN1A	chr6:36762684-36762743	A_23_P59210	1.54	1.20	5.82E-05	1.94E-03
NM_000389	CDKN1A	chr6:36762684-36762743	A_23_P59210	1.54	1.34	4.03E-05	4.10E-04
A_24_P478940	A_24_P478940	chr9:129580243-129580184	A_24_P478940	1.54	1.45	2.88E-05	8.82E-05
NM_001645	APOC1	chr19:50111419-50114325	A_23_P4649	1.54	1.53	9.04E-09	1.08E-08
NM_144665	SESN3	chr11:94557295-94557236	A_23_P361448	1.54	1.55	1.01E-11	6.79E-12
NM_020379	MAN1C1	chr1:25794981-25795040	A_23_P103601	1.54	1.57	1.56E-08	9.49E-09
ENST00000381577	ENST00000381577	chr9:5460098-5460157	A_23_P256487	1.54	2.01	6.34E-12	4.60E-16
NM_016358	IRX4	chr5:1930677-1930616	A_23_P110837	1.54	2.14	1.84E-11	1.44E-16
NM_021127	PMAIP1	chr18:55721258-55721317	A_23_P207999	1.55	1.01	1.18E-03	4.78E-02
NM_014844	KIAA0329	chr14:102036124-102036183	A_23_P394216	1.55	1.20	3.17E-13	1.06E-09
NM_022370	ROBO3	chr11:124256440-124256499	A_23_P356581	1.55	1.20	9.13E-05	2.84E-03
NM_000625	NOS2A	chr17:23108048-23107989	A_23_P502464	1.55	1.27	9.21E-09	1.22E-06
NM_005557	KRT16	chr17:37020305-37020246	A_24_P392991	1.55	1.33	1.10E-08	4.28E-07
NM_003811	TNFSF9	chr19:6485700-6485759	A_24_P5856	1.55	1.33	6.09E-08	1.92E-06
NM_000389	CDKN1A	chr6:36762684-36762743	A_23_P59210	1.55	1.48	5.20E-06	1.37E-05
NM_014848	SV2B	chr15:89639333-89639392	A_23_P100022	1.55	1.52	1.89E-10	3.62E-10
A_32_P105940	A_32_P105940	chr17:061732700-061732759	A_32_P105940	1.55	1.52	4.74E-09	7.91E-09
NM_182832	PLAC4	chr21:41470744-41470685	A_24_P76558	1.55	1.55	1.31E-17	1.31E-17
BC045163	BC045163	chr22:44320970-44320911	A_32_P67266	1.55	1.55	4.78E-07	4.84E-07
NM_002612	PDK4	chr7:94858017-94857958	A_24_P243749	1.55	1.62	6.66E-08	2.10E-08

Gene	Symbol	Chromosomal position	Probe	siCASP8AP2.3	CASP8AP2.6	siCASP8AP2.3	CASP8AP2.6
				M	M	Q	Q
NM_000043	FAS	chr10:90764525-90764584	A_23_P63896	1.56	1.12	4.88E-05	4.10E-03
NM_021127	PMAIP1	chr18:55721258-55721317	A_23_P207999	1.56	1.16	6.85E-04	1.57E-02
A_23_P106814	A_23_P106814	chr16:002829368-002829427	A_23_P106814	1.56	1.30	4.45E-06	1.24E-04
NM_002847	PTPRN2	chr7:156831448-156831389	A_32_P79434	1.56	1.32	3.67E-06	8.59E-05
NM_006868	RAB31	chr18:9835592-9835651	A_23_P141688	1.56	1.36	9.17E-05	7.53E-04
BC047708	BC047708	chr7:29500206-29500266	A_24_P655833	1.56	1.38	2.14E-10	6.13E-09
NM_000389	CDKN1A	chr6:36762684-36762743	A_23_P59210	1.56	1.43	8.31E-06	4.38E-05
AL833749	LOC146439	chr16:3017826-3017767	A_24_P273647	1.56	1.47	1.70E-10	9.16E-10
NM_003510	HIST1H2AK	chr6:27913767-27913708	A_24_P217848	1.56	1.48	1.98E-13	1.27E-12
A_32_P35294	A_32_P35294		A_32_P35294	1.56	1.67	2.17E-15	1.49E-16
NM_005462	MAGEC1	chrX:140722417-140722476	A_23_P217178	1.56	1.86	5.03E-14	7.09E-17
NM_025217	ULBP2	chr6:150359828-150359887	A_23_P145485	1.56	2.18	1.78E-07	1.60E-11
NM_021127	PMAIP1	chr18:55721258-55721317	A_23_P207999	1.57	1.07	7.84E-04	3.08E-02
NM_003537	HIST1H3B	chr6:26139947-26139888	A_24_P174924	1.57	1.12	9.00E-06	1.50E-03
NM_152513	RP5-821D11.2	chr22:40519836-40519895	A_23_P211561	1.57	1.25	2.01E-03	1.87E-02
NM_001013642	LOC388610	chr1:27011061-27011120	A_24_P693986	1.57	1.58	4.17E-10	3.66E-10
NM_004425	ECM1	chr1:147298870-147298929	A_23_P160559	1.57	1.83	8.00E-07	2.43E-08
NM_003311	PHLDA2	chr11:2906331-2906272	A_23_P47614	1.57	1.88	1.77E-05	5.57E-07
NM_025217	ULBP2	chr6:150359786-150359845	A_23_P168259	1.57	2.03	3.02E-07	3.60E-10
NM_152352	C18orf19	chr18:13656194-13656135	A_23_P380978	1.57	2.04	6.77E-13	4.47E-17
NM_000603	NOS3	chr7:150148081-150148484	A_23_P70849	1.58	0.93	2.05E-10	4.56E-05
NM_001085	SERPINA3	chr14:94150936-94150995	A_23_P162918	1.58	0.96	1.44E-15	1.15E-08
NM_005532	IFI27	chr14:93652682-93652741	A_23_P48513	1.58	0.99	1.23E-07	5.84E-04
NM_144505	KLK8	chr19:56191187-56191128	A_23_P369343	1.58	1.12	5.42E-06	1.20E-03
NM_002575	SERPINB2	chr18:59721182-59721241	A_24_P245379	1.58	1.21	3.09E-26	3.89E-21
NM_000389	CDKN1A	chr6:36762684-36762743	A_23_P59210	1.58	1.34	4.52E-05	5.89E-04
NM_000389	CDKN1A	chr6:36762684-36762743	A_23_P59210	1.58	1.35	3.62E-05	4.51E-04
NM_013385	PSCD4	chr22:36035579-36035638	A_23_P155057	1.58	1.51	2.09E-10	8.24E-10
NM_000389	CDKN1A	chr6:36762684-36762743	A_23_P59210	1.58	1.54	8.88E-06	1.60E-05
NM_019894	TMPRSS4	chr11:117494258-117494317	A_23_P127608	1.58	1.63	1.03E-13	2.96E-14
NM_005532	IFI27	chr14:93652682-93652741	A_23_P48513	1.59	0.99	2.46E-07	1.01E-03
NM_005532	IFI27	chr14:93652682-93652741	A_23_P48513	1.59	1.07	6.09E-08	1.59E-04
NM_000043	FAS	chr10:90764525-90764584	A_23_P63896	1.59	1.10	2.70E-05	3.85E-03
NM_139247	ADCY4	chr14:23857567-23857508	A_23_P381261	1.59	1.13	6.58E-13	1.95E-08
NM_000043	FAS	chr10:90764525-90764584	A_23_P63896	1.59	1.20	2.94E-06	3.93E-04
NM_000043	FAS	chr10:90764525-90764584	A_23_P63896	1.59	1.24	6.70E-06	4.23E-04
NM_000043	FAS	chr10:90764525-90764584	A_23_P63896	1.59	1.26	1.00E-05	4.79E-04
NM_000389	CDKN1A	chr6:36762684-36762743	A_23_P59210	1.59	1.33	2.57E-05	4.41E-04
AK024488	FLJ21438	chr19:15423512-15423453	A_23_P79069	1.59	1.40	1.31E-09	4.06E-08
NM_031908	C1QTNF2	chr5:159708738-159708679	A_23_P92899	1.59	1.41	1.16E-11	4.34E-10
A_32_P232647	A_32_P232647	chr20:049832446-049832387	A_32_P232647	1.59	1.45	4.64E-06	2.57E-05
AK000959	BEXL1	chrX:102277427-102277486	A_24_P40551	1.59	1.50	3.84E-06	1.23E-05
AK026750	AK026750	chr8:142432768-142432709	A_24_P589028	1.59	1.91	1.80E-10	4.33E-13
NM_000043	FAS	chr10:90764525-90764584	A_23_P63896	1.60	1.05	3.16E-05	7.11E-03
NM_005532	IFI27	chr14:93652682-93652741	A_23_P48513	1.60	1.09	2.09E-08	6.66E-05
NM_031290	CCDC70	chr13:51337789-51337848	A_23_P2857	1.60	1.14	9.46E-19	4.31E-13
NM_000043	FAS	chr10:90764525-90764584	A_23_P63896	1.60	1.26	1.21E-05	5.77E-04
NM_003944	SELENBP1	chr1:148149979-148149920	A_23_P86021	1.60	1.37	1.28E-06	2.76E-05
NM_001014979	LOC90835	chr16:30676324-30676265	A_24_P842002	1.60	1.42	4.04E-14	2.09E-12
NM_001897	CSPG4	chr15:73754326-73754267	A_23_P21976	1.60	1.43	1.83E-17	1.41E-15
NM_001490	GCNT1	chr9:76348311-76348370	A_23_P9232	1.60	1.46	7.82E-09	8.10E-08
NM_001252	TNFSF7	chr19:6536916-6536857	A_23_P119202	1.60	1.72	5.67E-04	2.88E-04
NM_014470	RND1	chr12:47540867-47540808	A_23_P53370	1.60	1.75	4.48E-14	1.29E-15
NM_182755	ZNF438	chr10:31173835-31173776	A_23_P161156	1.60	1.76	7.20E-19	1.40E-20
NM_001251	CD68	chr17:7425603-7425662	A_23_P15394	1.60	1.99	1.37E-13	3.31E-17
NM_000201	ICAM1	chr19:10257298-10257358	A_23_P153320	1.60	2.24	5.98E-05	8.86E-08

Gene	Symbol	Chromosomal position	Probe	siCASP8AP2.3	CASP8AP2.6	siCASP8AP2.3	CASP8AP2.6
				M	M	Q	Q
NM_005532	IFI27	chr14:93652682-93652741	A_23_P48513	1.61	0.98	7.41E-08	6.76E-04
NM_005532	IFI27	chr14:93652682-93652741	A_23_P48513	1.61	1.06	4.74E-08	1.77E-04
NM_021127	PMAIP1	chr18:55721258-55721317	A_23_P207999	1.61	1.21	4.17E-04	1.10E-02
NM_018664	SNFT	chr1:209248482-209248423	A_23_P160720	1.61	1.39	8.73E-08	2.27E-06
NM_022910	NDRG4	chr16:57104610-57104669	A_23_P140748	1.61	1.55	3.24E-07	7.48E-07
NM_019891	ERO1LB	chr1:232707833-232707774	A_23_P347618	1.61	1.68	2.90E-09	8.94E-10
NM_001333	CTSL2	chr9:96874766-96874707	A_23_P146456	1.61	1.88	1.14E-11	4.92E-14
NM_032898	C3orf34	chr3:197922573-197922514	A_32_P212058	1.62	1.03	2.49E-12	6.35E-07
NM_002397	MEF2C	chr5:88052632-88052573	A_23_P320739	1.62	1.04	1.71E-05	6.27E-03
NM_005532	IFI27	chr14:93652682-93652741	A_23_P48513	1.62	1.06	1.87E-08	1.11E-04
NM_021127	PMAIP1	chr18:55721258-55721317	A_23_P207999	1.62	1.18	4.81E-04	1.45E-02
NM_003965	CCRL2	chr3:46425726-46425785	A_23_P69310	1.62	1.38	2.36E-09	1.61E-07
NM_144698	ANKRD35	chr1:143057473-143057532	A_23_P325690	1.62	1.44	6.19E-16	5.06E-14
NM_080912	ASGR2	chr17:6945463-6945404	A_23_P130113	1.62	1.59	1.34E-14	2.68E-14
NM_004497	FOXA3	chr19:51068670-51068729	A_23_P208737	1.62	1.65	1.29E-04	1.22E-04
NM_004566	PFKFB3	chr10:6315152-6315211	A_23_P95755	1.62	1.66	1.23E-10	5.68E-11
A_32_P103966	A_32_P103966	chr22:048127518-048127459	A_32_P103966	1.62	1.69	2.18E-09	7.09E-10
NM_198965	PTHLH	chr12:28007668-28007609	A_23_P2271	1.62	1.82	1.08E-06	8.58E-08
AF247042	AF247042	chr11:63828753-63828812	A_23_P202773	1.62	1.88	6.94E-11	4.83E-13
NR_003061	LILRP2	chr19:59913791-59913850	A_23_P27781	1.62	1.90	3.65E-12	1.25E-14
NM_005532	IFI27	chr14:93652682-93652741	A_23_P48513	1.63	1.08	1.08E-08	7.02E-05
NM_002020	FLT4	chr5:179969561-179968651	A_23_P356070	1.63	1.45	7.46E-18	7.75E-16
NM_144691	CAPN12	chr19:43912704-43912672	A_23_P16409	1.63	1.45	9.28E-09	1.75E-07
NM_004335	BST2	chr19:17375113-17375054	A_23_P39465	1.63	1.60	7.20E-07	1.03E-06
NM_203380	ACSL5	chr10:114175146-114175205	A_24_P201360	1.63	1.65	9.56E-07	7.99E-07
NM_007136	ZNF80	chr3:115437689-115437630	A_23_P256526	1.63	1.74	6.48E-13	5.24E-14
NM_022469	GREM2	chr1:236980077-236980018	A_24_P40626	1.63	2.07	2.51E-09	1.68E-12
NM_017709	FAM46C	chr1:117878946-117879005	A_23_P137751	1.63	2.12	7.83E-16	1.72E-20
ENST00000353250	ENST00000353250	chr8:22270466-22270525	A_32_P208654	1.64	1.22	7.90E-12	3.89E-08
NM_000043	FAS	chr10:90764525-90764584	A_23_P63896	1.64	1.26	8.30E-06	5.76E-04
NM_032262	DKFZp434N035	chr22:19383379-19383438	A_23_P166336	1.64	1.56	1.04E-16	6.30E-16
NM_174934	SCN4B	chr11:117509566-117509507	A_23_P303833	1.64	1.56	9.67E-08	3.49E-07
NM_003963	TM4SF5	chr17:4632995-4633054	A_23_P27107	1.64	1.64	1.69E-11	1.79E-11
A_32_P100830	A_32_P100830	chr17:069863473-069863532	A_32_P100830	1.64	1.74	3.99E-06	1.24E-06
AK098753	C22orf35	chr22:25391051-25391110	A_24_P595223	1.64	1.78	4.01E-10	3.03E-11
NM_019891	ERO1LB	chr1:232711312-232710295	A_23_P347623	1.64	1.82	7.24E-09	3.92E-10
NM_004155	SERPINB9	chr6:2832620-2832561	A_24_P295010	1.64	1.93	3.51E-05	1.97E-06
A_32_P208076	A_32_P208076	chr5:052424025-052424084	A_32_P208076	1.65	1.21	6.77E-06	9.24E-04
NM_032946	NXF5	chrX:100893304-100893245	A_23_P114353	1.65	2.16	3.15E-11	2.72E-15
NM_002277	KRT31	chr17:36803591-36803532	A_23_P107465	1.66	1.28	3.92E-11	5.63E-08
NM_015675	GADD45B	chr19:2428070-2428129	A_23_P142506	1.66	1.32	2.30E-03	2.18E-02
NM_004024	ATF3	chr1:209182251-209182310	A_23_P34915	1.66	1.74	3.24E-04	2.08E-04
NM_004210	NEURL	chr10:105340205-105340264	A_23_P138492	1.66	1.75	3.12E-11	5.56E-12
NM_000777	CYP3A5	chr7:98890609-98890550	A_23_P8801	1.67	1.01	1.68E-06	3.69E-03
NM_000043	FAS	chr10:90764525-90764584	A_23_P63896	1.67	1.20	6.18E-06	1.12E-03
NM_004669	CLIC3	chr9:137165090-137165031	A_23_P254654	1.67	1.38	6.20E-06	1.75E-04
NM_080596	HIST1H2AH	chr6:27223225-27223284	A_23_P81859	1.67	1.51	1.41E-12	3.81E-11
NM_020882	COL20A1	chr20:61431613-61431671	A_32_P185637	1.67	1.51	1.84E-09	3.02E-08
NM_014266	HCST	chr19:41086900-41086959	A_23_P39386	1.67	1.57	3.09E-11	1.73E-10
NM_024787	RNF122	chr8:33524905-33524846	A_23_P134744	1.67	1.59	5.71E-07	1.73E-06
BC030024	ASNS	chr7:97182846-97182787	A_32_P232747	1.67	1.68	3.93E-22	2.84E-22
NM_014271	IL1RAPL1	chrX:29733688-29733747	A_23_P84106	1.67	1.81	3.42E-04	1.37E-04
NM_000201	ICAM1	chr19:10257298-10257358	A_23_P153320	1.67	2.14	4.51E-05	4.16E-07
NM_003914	CCNA1	chr13:35912154-35912213	A_23_P48414	1.67	2.96	7.34E-14	6.38E-24
NM_005018	PDCD1	chr2_random:122302-122361	A_23_P136405	1.68	1.22	4.38E-07	1.67E-04
NM_000413	HSD17B1	chr17:37959140-37959374	A_24_P56833	1.68	1.23	8.17E-14	1.82E-09

Gene	Symbol	Chromosomal position	Probe	siCASP8AP2.3	CASP8AP2.6	siCASP8AP2.3	CASP8AP2.6
				M	M	Q	Q
NM_138451	IQCD	chr12:112096071-112096012	A_24_P390060	1.68	1.42	8.90E-12	1.64E-09
NM_002960	S100A3	chr1:150333130-150333071	A_23_P104073	1.68	1.45	7.32E-08	1.93E-06
NM_019858	GPR162	chr12:6806682-6806741	A_23_P99141	1.68	1.63	1.72E-07	3.72E-07
BC073935	BC073935	chr12:8400913-8400854	A_32_P221305	1.68	1.76	5.36E-08	1.66E-08
NM_003770	KRT37	chr17:36830417-36830358	A_23_P118854	1.68	1.91	3.44E-15	2.21E-17
NM_021990	GABRE	chrX:150792247-150792188	A_23_P159775	1.68	1.97	4.31E-09	3.88E-11
NM_000201	ICAM1	chr19:10257298-10257358	A_23_P153320	1.68	2.18	2.01E-05	9.99E-08
ENST00000211092	ENST00000211092	chr19:3425882-3425823	A_23_P397120	1.69	1.13	8.59E-07	7.90E-04
NM_198232	RNASE1	chr14:20339501-20339442	A_23_P48596	1.69	1.62	6.32E-09	2.06E-08
NM_017805	RASIP1	chr19:53915776-53915717	A_23_P119353	1.69	1.70	1.00E-12	8.48E-13
NM_020939	CPNE5	chr6:36816607-36816548	A_23_P360804	1.69	1.94	5.10E-10	6.85E-12
NM_000603	NOS3	chr7:150148081-150148484	A_23_P70849	1.70	0.82	1.35E-10	6.43E-04
NM_003802	MYH13	chr17:10147459-10147298	A_23_P89334	1.70	0.99	2.12E-11	1.42E-05
NM_005532	IFI27	chr14:93652682-93652741	A_23_P48513	1.70	1.10	8.83E-09	9.07E-05
NM_000603	NOS3	chr7:150148081-150148484	A_23_P70849	1.70	1.11	3.50E-09	4.40E-05
A_32_P101653	A_32_P101653	chr8:038360092-038360151	A_32_P101653	1.70	1.21	5.75E-14	2.62E-09
NM_173582	PGM2L1	chr11:73725315-73725256	A_23_P396765	1.70	1.40	2.27E-10	4.22E-08
NM_021217	ZNF77	chr19:2884493-2884434	A_32_P182388	1.70	1.47	2.34E-03	1.21E-02
BC016022	BC016022		A_24_P895836	1.70	1.52	5.03E-07	5.33E-06
NM_001657	AREG	chr4:75685553-75685612	A_23_P259071	1.70	1.88	7.46E-06	1.06E-06
NM_058172	ANTXR2	chr4:81184080-81184021	A_23_P170733	1.70	2.42	5.20E-09	8.12E-14
THC2313103	THC2313103	chr17:71662197-71662256	A_32_P90346	1.71	1.34	8.44E-07	8.73E-05
NM_006072	CCL26	chr7:75045870-75043712	A_23_P215484	1.71	1.80	5.59E-07	1.99E-07
THC2380864	THC2380864	chr22:40630102-40630043	A_32_P53603	1.71	1.86	8.17E-08	9.31E-09
NM_007329	DMBT1	chr10:124393142-124393201	A_23_P86599	1.71	1.90	7.84E-20	8.19E-22
NM_144569	SPOCD1	chr1:31925186-31925127	A_23_P431388	1.71	1.92	7.31E-10	2.06E-11
NM_000927	ABCB1	chr7:86783254-86779964	A_23_P82523	1.72	1.47	6.70E-07	1.61E-05
NM_003305	TRPC3	chr4:123158174-123158115	A_23_P41455	1.72	1.55	3.09E-06	2.32E-05
NM_000389	CDKN1A	chr6:36762684-36762743	A_23_P59210	1.72	1.57	1.30E-06	8.85E-06
NM_182628	CCDC37	chr3:127637282-127637341	A_23_P426153	1.72	1.59	9.92E-08	5.79E-07
NM_005428	VAV1	chr19:6804995-6805054	A_23_P38959	1.72	1.71	4.07E-10	4.66E-10
THC2403712	THC2403712	chr17:72061176-72061235	A_24_P746314	1.72	2.14	5.67E-10	5.33E-13
NM_000201	ICAM1	chr19:10257298-10257358	A_23_P153320	1.72	2.21	1.36E-05	8.20E-08
NM_058242	KRT6C	chr12:51168391-51167995	A_23_P87653	1.73	1.17	5.25E-10	7.03E-06
AK090478	TMC8	chr17:73650304-73650363	A_23_P346093	1.73	1.27	2.15E-06	4.49E-04
AK026312	FLJ22659	chr17:19064269-19064328	A_23_P4052	1.73	1.38	1.94E-13	2.71E-10
NM_003806	HRK	chr12:115761832-115761773	A_23_P25194	1.73	1.40	1.23E-04	2.16E-03
NM_021123	GAGE7	chrX:49030381-49030440	A_24_P228796	1.73	1.51	3.33E-11	2.06E-09
NM_005512	LRRC32	chr11:76046665-76046606	A_24_P389916	1.73	1.90	2.38E-18	4.71E-20
NM_003511	HIST1H2AL	chr6:27941481-27941540	A_23_P363174	1.73	1.97	2.13E-11	2.31E-13
NM_000201	ICAM1	chr19:10257298-10257358	A_23_P153320	1.73	2.11	6.97E-05	2.30E-06
NM_015675	GADD45B	chr19:2429178-2429237	A_24_P239606	1.74	1.32	1.22E-03	1.96E-02
NM_032262	DKFZp434N035	chr22:19383196-19383255	A_24_P787889	1.74	1.39	7.00E-12	5.88E-09
BX110856	BX110856	chr19:52623159-52623100	A_32_P45844	1.74	1.43	6.92E-11	1.59E-08
NM_004694	SLC16A6	chr17:63776558-63776499	A_23_P152791	1.74	1.62	8.67E-08	4.96E-07
NM_019018	FAM105A	chr5:14663510-14663569	A_23_P133438	1.74	1.83	2.52E-16	3.92E-17
NM_001958	EEF1A2	chr20:61590833-61590774	A_23_P256033	1.74	1.85	1.64E-07	3.96E-08
NM_001323	CST6	chr11:65537414-65537473	A_23_P146946	1.74	1.85	2.07E-05	7.24E-06
NM_152454	TMEM83	chr15:85923671-85923730	A_23_P382094	1.74	1.91	4.52E-17	1.10E-18
NM_000094	COL7A1	chr3:48577592-48577405	A_23_P144071	1.74	1.94	2.10E-10	7.35E-12
NM_000201	ICAM1	chr19:10257298-10257358	A_23_P153320	1.74	2.07	4.16E-05	1.92E-06
NM_001033719	ZNF404	chr19:49068716-49068657	A_23_P90333	1.74	2.13	4.48E-14	1.81E-17
NM_000201	ICAM1	chr19:10257298-10257358	A_23_P153320	1.74	2.13	3.67E-05	8.67E-07
NM_000201	ICAM1	chr19:10257298-10257358	A_23_P153320	1.74	2.14	3.45E-05	8.15E-07
NM_000389	CDKN1A	chr6:36762684-36762743	A_23_P59210	1.75	1.51	1.94E-06	3.51E-05
NM_000927	ABCB1	chr7:86783254-86779964	A_23_P82523	1.75	1.70	1.99E-06	3.65E-06

Gene	Symbol	Chromosomal position	Probe	siCASP8AP2.3	CASP8AP2.6	siCASP8AP2.3	CASP8AP2.6
				M	M	Q	Q
NM_178504	DNHD2	chr3:57304615-57303003	A_23_P372496	1.75	1.81	5.87E-16	1.43E-16
NM_000201	ICAM1	chr19:10257298-10257358	A_23_P153320	1.75	2.26	1.03E-05	4.63E-08
NM_003495	HIST1H4I	chr6:27215204-27215263	A_24_P20873	1.75	2.63	1.69E-06	4.38E-11
NM_000429	MAT1A	chr10:82021772-82021713	A_23_P23996	1.76	1.25	5.26E-10	2.34E-06
NM_002988	CCL18	chr17:31422793-31422852	A_23_P55270	1.76	1.42	1.37E-24	1.40E-20
BC046172	BC046172	chr6:17815428-17815487	A_32_P178696	1.76	1.44	9.34E-09	1.17E-06
NM_133458	ZFP90	chr16:67156831-67156890	A_24_P176409	1.76	1.51	8.24E-21	3.98E-18
NM_153487	MDGA1	chr6:37708332-37708273	A_23_P310460	1.76	1.54	5.24E-08	1.12E-06
NM_005634	SOX3	chrX:139310826-139310768	A_23_P85218	1.76	2.11	5.84E-11	1.42E-13
NM_001878	CRABP2	chr1:153482711-153482652	A_23_P115064	1.77	1.58	1.71E-07	2.03E-06
NM_016307	PRRX2	chr9:129564427-129564486	A_23_P83298	1.77	1.67	8.80E-05	2.50E-04
NM_006732	FOSB	chr19:50670045-50670104	A_23_P429998	1.77	2.37	6.58E-08	1.85E-11
NM_004079	CTSS	chr1:147535603-147533418	A_23_P46141	1.77	2.38	1.06E-07	3.01E-11
AK023401	AK023401	chr11:56715652-56715711	A_24_P819890	1.78	1.25	3.06E-21	4.15E-15
NM_181607	KRTAP19-1	chr21:30774393-30774334	A_24_P367247	1.78	1.54	8.61E-10	4.44E-08
BX106493	BX106493	chr1:146644712-146644771	A_24_P710681	1.78	1.64	2.53E-15	4.92E-14
NM_032782	HAVCR2	chr5:156445541-156445482	A_23_P18903	1.78	1.65	2.07E-07	1.20E-06
NM_004877	GMFG	chr19:44510986-44510927	A_23_P208866	1.78	1.71	1.58E-05	3.90E-05
NM_001628	AKR1B1	chr7:133589352-133587316	A_23_P258190	1.78	2.12	7.36E-11	1.95E-13
NM_000899	KITLG	chr12:87392757-87392698	A_23_P204654	1.79	1.29	2.29E-14	1.06E-09
NM_153607	LOC153222	chr5:172496411-172496470	A_23_P404606	1.79	1.29	7.22E-04	2.06E-02
AK123333	AK123333	chr19:46734909-46734968	A_32_P53234	1.79	1.37	1.07E-12	3.80E-09
NM_145006	SUSD3	chr9:92926859-92926918	A_23_P401076	1.79	1.49	1.04E-07	5.26E-06
NM_001004431	METRNL	chr17:78645910-78645969	A_23_P10591	1.79	1.70	1.02E-05	3.10E-05
NM_006426	DPYSL4	chr10:133869121-133869180	A_23_P331049	1.79	1.76	1.91E-11	3.01E-11
NM_181985	LILRA5	chr19:59514525-59510816	A_23_P107847	1.79	2.15	8.89E-12	1.41E-14
NM_000201	ICAM1	chr19:10257298-10257358	A_23_P153320	1.79	2.23	9.23E-06	1.00E-07
NM_031308	EPPK1	chr8:145012145-145012086	A_24_P357169	1.79	2.35	4.10E-07	3.66E-10
NM_207505	FLJ45248	chr8:103890964-103891023	A_32_P228348	1.80	1.45	1.27E-15	2.50E-12
NM_022742	NAG6	chr7:128048785-128048844	A_23_P416178	1.80	1.62	9.82E-09	1.32E-07
NM_001008707	EML1	chr14:99477245-99477304	A_23_P205746	1.80	1.72	8.65E-11	3.43E-10
A_32_P73991	A_32_P73991	chr12:004228258-004228199	A_32_P73991	1.80	1.94	8.06E-11	7.54E-12
BU678941	BU678941	chr2:87594315-87594256	A_32_P184937	1.80	1.96	2.92E-08	2.92E-09
NM_005994	TBX2	chr17:56841038-56841097	A_23_P164451	1.80	2.06	2.75E-05	2.49E-06
NM_021733	TSKS	chr19:54935171-54934986	A_23_P16275	1.81	1.16	9.47E-13	3.16E-07
NM_024869	GRRP1	chr1:26173136-26173195	A_23_P46131	1.81	1.19	3.05E-13	7.75E-08
AK131261	TMPRSS9	chr19:2377040-2377099	A_23_P209176	1.81	1.20	2.17E-11	1.25E-06
ENST00000344320	ENST00000344320	chr15:38851578-38851637	A_32_P183970	1.81	1.65	1.61E-09	2.08E-08
NM_000927	ABCB1	chr7:86783254-86779964	A_23_P82523	1.81	1.65	3.45E-07	2.59E-06
NM_014256	B3GNT3	chr19:17784824-17784883	A_23_P78980	1.81	1.97	2.71E-13	1.41E-14
NM_001147	ANGPT2	chr8:6347757-6347698	A_23_P60079	1.81	2.21	1.11E-07	5.77E-10
NM_000211	ITGB2	chr21:45130413-45130354	A_23_P329573	1.82	1.74	4.52E-13	1.90E-12
NM_000211	ITGB2	chr21:45130413-45130354	A_23_P329573	1.82	1.85	4.20E-12	2.64E-12
NM_022640	CSH1	chr17:59326067-59326008	A_23_P207170	1.82	2.00	4.81E-10	3.04E-11
NM_033191	KRTAP9-4	chr17:36660338-36660397	A_23_P207868	1.83	0.71	1.83E-17	2.23E-05
NM_032129	PLEKHN1	chr1:950367-950426	A_23_P23616	1.83	0.81	1.25E-06	3.59E-02
XM_496202	LOC440421	chr17:25920023-25919964	A_24_P887857	1.83	1.19	8.00E-07	1.09E-03
NM_003695	LY6D	chr8:143863453-143863394	A_23_P134764	1.83	1.37	1.20E-19	1.04E-14
NM_005101	ISG15	chr1:989634-989693	A_23_P819	1.83	1.59	1.28E-12	9.91E-11
NM_000927	ABCB1	chr7:86783254-86779964	A_23_P82523	1.83	1.59	2.42E-07	4.67E-06
BC052945	LOC643201	chr5:175507651-175507449	A_32_P30891	1.83	1.63	5.97E-11	1.66E-09
NM_006262	PRPH	chr12:47978579-47978638	A_23_P13713	1.83	1.75	1.67E-04	3.83E-04
NM_006208	ENPP1	chr6:132253564-132253623	A_23_P156880	1.83	1.84	1.85E-12	1.43E-12
AK023391	AK023391	chr8:124581645-124581586	A_24_P918147	1.83	1.97	6.95E-05	2.54E-05
NM_002229	JUNB	chr19:12765047-12765106	A_23_P4821	1.83	2.10	1.14E-09	1.73E-11
NM_000927	ABCB1	chr7:86783254-86779964	A_23_P82523	1.84	1.69	1.69E-07	1.15E-06

Gene	Symbol	Chromosomal position	Probe	siCASP8AP2.3	CASP8AP2.6	siCASP8AP2.3	CASP8AP2.6
				M	M	Q	Q
NM_000211	ITGB2	chr21:45130413-45130354	A_23_P329573	1.84	1.80	1.80E-11	3.13E-11
A_24_P152845	A_24_P152845	chr10:069180870-069180929	A_24_P152845	1.84	1.96	9.70E-22	7.59E-23
NM_139174	LOC161931	chr16:82788132-82788191	A_23_P49136	1.85	0.98	1.16E-08	1.55E-03
NM_000927	ABCB1	chr7:86783254-86779964	A_23_P82523	1.85	1.40	2.28E-07	5.92E-05
NM_003641	IFITM1	chr11:305209-305268	A_23_P72737	1.85	1.48	3.08E-05	9.43E-04
NM_000746	CHRNA7	chr15:30247843-30247902	A_23_P163390	1.85	1.57	4.77E-12	7.24E-10
NM_000927	ABCB1	chr7:86783254-86779964	A_23_P82523	1.85	1.66	3.15E-07	3.42E-06
NM_001039614	LOC388135	chr15:71819369-71819310	A_32_P210168	1.85	2.03	2.77E-08	2.52E-09
NM_000927	ABCB1	chr7:86783254-86779964	A_23_P82523	1.86	1.63	9.22E-08	1.99E-06
NM_012081	ELL2	chr5:95248974-95248915	A_23_P41645	1.86	1.90	1.90E-10	9.03E-11
NM_005248	FGR	chr1:27623039-27622980	A_23_P103932	1.87	1.34	1.33E-15	1.26E-10
NM_014957	DENND3	chr8:142274912-142274971	A_32_P175301	1.87	1.44	3.04E-09	1.77E-06
NM_000211	ITGB2	chr21:45130413-45130354	A_23_P329573	1.87	1.83	1.35E-11	2.72E-11
NM_177963	SYT12	chr11:66574795-66574854	A_23_P421306	1.88	1.42	3.41E-16	7.69E-12
NM_014400	LYPD3	chr19:48656934-48656875	A_23_P39265	1.88	1.56	2.85E-04	3.38E-03
AK124399	AK124399	chr4:142603617-142603558	A_32_P54160	1.88	1.80	4.06E-12	1.51E-11
ENST00000375672	ENST00000375672	chr20:30600080-30600021	A_24_P101800	1.88	1.85	4.50E-17	7.25E-17
BC031655	C1orf38	chr1:27897169-27897229	A_23_P873	1.88	1.98	6.89E-10	1.65E-10
THC2271582	THC2271582	chr17:19032212-19032270	A_32_P134634	1.88	2.21	2.26E-07	3.86E-09
NM_174921	LOC201895	chr4:39375887-39375828	A_23_P112634	1.89	1.44	1.08E-09	9.53E-07
THC2270724	THC2270724	chr17:56825660-56825601	A_24_P896205	1.89	1.62	2.73E-10	2.10E-08
NM_000063	C2	chr6:32021349-32021408	A_32_P162183	1.89	1.65	3.05E-11	1.56E-09
NM_021796	PLAC1	chrX:133425659-133425600	A_23_P148609	1.89	1.77	5.44E-21	7.86E-20
NM_002517	NPAS1	chr19:52240711-52240770	A_23_P27734	1.89	1.95	4.17E-12	1.30E-12
NM_080680	COL11A2	chr6:33238694-33238634	A_23_P42322	1.89	2.13	2.81E-17	1.94E-19
NM_020358	TRIM49	chr11:89171267-89171208	A_23_P1575	1.89	2.49	9.65E-10	1.37E-13
NM_203400	RPRML	chr17:42410605-42410546	A_32_P3476	1.90	1.50	3.79E-11	3.05E-08
NM_000927	ABCB1	chr7:86783254-86779964	A_23_P82523	1.90	1.71	9.99E-08	1.13E-06
NM_005557	KRT16	chr17:37020890-37020742	A_23_P38537	1.90	1.75	1.16E-12	1.67E-11
NM_001004434	SLC30A2	chr1:26048839-26048780	A_24_P156748	1.91	1.54	9.62E-05	1.90E-03
NM_000211	ITGB2	chr21:45130413-45130354	A_23_P329573	1.91	1.79	3.30E-12	2.87E-11
NM_003873	NRP1	chr10:33542356-33536628	A_24_P135322	1.91	1.88	2.73E-05	4.05E-05
NM_153750	C21orf81	chr21:14239284-14238049	A_23_P392529	1.92	0.87	3.68E-10	2.18E-03
NM_000327	ROM1	chr11:62139080-62139139	A_23_P105002	1.92	1.87	3.26E-15	7.84E-15
NM_152359	CPT1C	chr19:54908693-54908752	A_23_P16283	1.92	1.96	9.33E-10	4.66E-10
NM_214462	DACT2	chr6:168526250-168526191	A_24_P289260	1.92	2.00	1.80E-16	3.93E-17
NM_003978	PSTPIP1	chr15:75116510-75116569	A_23_P48997	1.92	2.01	2.19E-10	5.45E-11
NM_031308	EPPK1	chr8:145011953-145011894	A_23_P83388	1.92	2.35	2.95E-05	6.37E-07
NM_172374	IL4I1	chr19:55084784-55084725	A_23_P502520	1.93	1.85	3.32E-14	1.21E-13
NM_003513	HIST1H2AB	chr6:26141499-26141440	A_24_P223384	1.93	1.95	3.63E-10	2.41E-10
NM_001454	FOXJ1	chr17:71644250-71644191	A_23_P348636	1.93	2.22	1.23E-07	3.85E-09
NM_003890	FCGBP	chr19:45045966-45045907	A_23_P21495	1.94	1.73	3.33E-13	1.51E-11
NM_198584	CA13	chr8:86383217-86383276	A_23_P381714	1.94	1.96	7.46E-11	5.09E-11
CR596233	CR596233	chr12:50603301-50603360	A_24_P945113	1.94	2.03	6.44E-23	9.14E-24
NM_207372	SH2D4B	chr10:82395606-82395665	A_24_P273489	1.94	2.16	3.74E-19	3.54E-21
NM_002590	PCDH8	chr13:52316265-52316206	A_23_P36985	1.94	2.39	7.54E-17	1.40E-20
CR605947	CR605947	chr15:30249514-30249573	A_32_P35969	1.95	1.54	2.68E-09	9.46E-07
THC2400010	THC2400010	chr18:57424354-57424295	A_32_P196142	1.95	2.68	2.73E-17	4.03E-23
NM_001927	DES	chr2:220116728-220116787	A_23_P90710	1.96	1.32	1.80E-07	2.98E-04
AK125448	SETX	chr9:132166576-132166517	A_24_P940921	1.96	1.43	8.88E-08	5.17E-05
NM_144688	FLJ32658	chr10:75164094-75164153	A_23_P431284	1.96	1.61	3.44E-14	2.63E-11
NM_000927	ABCB1	chr7:86783254-86779964	A_23_P82523	1.96	1.64	1.17E-07	5.34E-06
NM_001079	ZAP70	chr2:97814773-97814832	A_23_P39682	1.96	1.64	3.95E-05	6.13E-04
ENST00000293201	ENST00000293201	chr17:71128275-71128334	A_24_P369898	1.96	1.82	1.89E-09	1.34E-08
NM_032251	CCDC88	chr11:63881511-63881570	A_23_P24384	1.96	1.98	2.32E-19	1.33E-19
NM_015166	MLC1	chr22:48800464-48800405	A_23_P211680	1.96	2.05	3.30E-13	7.28E-14

Gene	Symbol	Chromosomal position	Probe	siCASP8AP2.3	CASP8AP2.6	siCASP8AP2.3	CASP8AP2.6
				M	M	Q	Q
THC2343253	THC2343253	chr17:8034837-8034896	A_32_P184888	1.97	1.79	3.87E-09	5.01E-08
A_24_P174353	A_24_P174353	chr2:232612231-232612172	A_24_P174353	1.97	2.79	2.67E-13	3.89E-19
NM_178859	OSTbeta	chr15:63132462-63132521	A_23_P436284	1.98	1.22	1.93E-06	2.97E-03
A_24_P913576	A_24_P913576	chr14:100942156-100942097	A_24_P913576	1.98	2.08	2.39E-07	8.44E-08
NM_000507	FBP1	chr9:94445040-94444981	A_23_P257111	1.98	2.09	3.77E-08	8.99E-09
NM_000492	CFTR	chr7:116902504-116902563	A_23_P215720	1.98	2.67	5.56E-15	3.09E-20
NM_024409	NPPC	chr2:232615755-232615696	A_23_P301942	1.98	2.68	2.64E-16	1.08E-21
NM_000211	ITGB2	chr21:45130413-45130354	A_23_P329573	1.99	1.75	1.14E-08	2.79E-07
NR_002556	LOC388242	chr16:29383854-29383795	A_32_P135336	1.99	1.79	1.09E-15	4.25E-14
NR_001442	FER1L4	chr20:33609993-33609934	A_23_P80048	1.99	1.80	3.75E-09	5.78E-08
NM_138290	RPIB9	chr7:87104072-87104131	A_23_P111724	1.99	2.02	5.68E-09	4.18E-09
NM_003034	ST8SIA1	chr12:22245397-22245338	A_23_P354705	1.99	2.11	2.02E-15	2.10E-16
ENST00000324050	ENST00000324050	chr8:565974-565915	A_23_P303417	1.99	2.16	3.66E-17	1.12E-18
NM_002923	RGS2	chr1:189512708-189512767	A_23_P114947	1.99	2.30	3.10E-07	8.44E-09
NM_001079	ZAP70	chr2:97814773-97814832	A_23_P39682	2.00	1.59	4.02E-05	1.16E-03
CR608961	CR608961	chr19:50837712-50837771	A_32_P163392	2.00	1.61	5.12E-10	1.65E-07
NM_005209	CRYBA2	chr2:219680558-219680499	A_23_P165457	2.00	2.00	4.93E-10	4.83E-10
NM_006176	NRGN	chr11:124122002-124122061	A_23_P116264	2.00	2.12	2.37E-10	3.38E-11
NM_000492	CFTR	chr7:116902504-116902563	A_23_P215720	2.00	2.70	1.80E-16	8.12E-22
NM_000492	CFTR	chr7:116902504-116902563	A_23_P215720	2.00	2.73	1.81E-16	5.34E-22
NM_004694	SLC16A6	chr17:63776724-63776668	A_24_P731648	2.01	1.62	1.94E-09	4.14E-07
NM_000211	ITGB2	chr21:45130413-45130354	A_23_P329573	2.01	1.76	1.33E-12	8.65E-11
NM_000526	KRT14	chr17:36993133-36993074	A_24_P265346	2.02	1.28	2.45E-12	6.77E-07
THC2404004	THC2404004	chr11:61035009-61035068	A_24_P460419	2.02	1.47	3.36E-10	1.15E-06
NM_001079	ZAP70	chr2:97814773-97814832	A_23_P39682	2.02	1.53	6.87E-05	3.01E-03
ENST00000381655	ENST00000381655	chr13:25497056-25497115	A_24_P944714	2.02	1.96	1.72E-11	3.88E-11
NM_000960	PTGIR	chr19:51815802-51815743	A_23_P340848	2.02	2.06	1.07E-14	5.62E-15
NM_145173	DIRAS1	chr19:2665640-2665581	A_23_P386942	2.03	1.43	5.10E-10	2.80E-06
NM_001079	ZAP70	chr2:97814773-97814832	A_23_P39682	2.03	1.44	9.58E-05	6.75E-03
NM_001079	ZAP70	chr2:97814773-97814832	A_23_P39682	2.03	1.53	5.90E-05	2.93E-03
NM_001079	ZAP70	chr2:97814773-97814832	A_23_P39682	2.03	1.55	5.77E-05	2.52E-03
AB067468	KIAA1881	chr19:4453780-4453721	A_23_P27846	2.03	1.74	7.91E-12	9.40E-10
NM_000211	ITGB2	chr21:45130413-45130354	A_23_P329573	2.03	1.82	4.72E-11	1.06E-09
NM_138969	RDHE2	chr8:57381808-57380796	A_23_P257457	2.03	1.89	5.99E-17	8.41E-16
NM_058229	FBXO32	chr8:124587816-124586113	A_23_P82814	2.03	2.35	1.53E-08	2.83E-10
NM_001079	ZAP70	chr2:97814773-97814832	A_23_P39682	2.04	1.51	6.25E-05	3.62E-03
NM_001079	ZAP70	chr2:97814773-97814832	A_23_P39682	2.04	1.59	3.14E-05	1.27E-03
ENST00000305423	ENST00000305423	chr7:100204939-100204998	A_23_P349147	2.04	1.67	2.74E-18	7.37E-15
NM_021603	FXYD2	chr11:117196213-117196154	A_23_P161769	2.04	2.19	4.81E-19	2.50E-20
NM_031916	ROPN1L	chr5:10517994-10518053	A_32_P335921	2.05	1.56	5.41E-12	1.65E-08
ENST00000355077	ENST00000355077	chr14:18659722-18659781	A_32_P16323	2.05	2.44	2.27E-08	1.81E-10
AK021957	COL27A1	chr9:114153292-114153351	A_23_P158096	2.06	1.57	7.29E-10	7.28E-07
NM_018896	CACNA1G	chr17:46059243-46059302	A_23_P107247	2.06	1.61	2.36E-22	8.17E-18
NM_181501	ITGA1	chr5:52276569-52278937	A_23_P256334	2.06	2.20	9.56E-10	1.29E-10
NM_000412	HRG	chr3:187878616-187878675	A_23_P321892	2.07	1.05	1.98E-08	2.96E-03
NM_001079	ZAP70	chr2:97814773-97814832	A_23_P39682	2.07	1.55	4.34E-05	2.54E-03
NM_001079	ZAP70	chr2:97814773-97814832	A_23_P39682	2.07	1.63	3.20E-05	1.10E-03
NM_032536	NTNG2	chr9:132147660-132147719	A_23_P423331	2.08	0.90	4.19E-09	8.02E-03
NM_015046	SETX	chr9:132168975-132168916	A_24_P95273	2.08	1.60	1.07E-08	4.43E-06
NM_000211	ITGB2	chr21:45130413-45130354	A_23_P329573	2.08	1.79	9.67E-11	6.50E-09
W60781	W60781	chr8:082553651-082553593	A_23_P8812	2.08	2.03	5.00E-18	1.18E-17
A_32_P44932	A_32_P44932	chr8:030060416-030060475	A_32_P44932	2.08	2.06	1.48E-07	1.99E-07
ENST00000244321	ENST00000244321	chr19:48777979-48778038	A_23_P50646	2.08	2.09	2.24E-22	1.87E-22
AK124097	AK124097	chr4:59450-59509	A_32_P202798	2.08	2.75	3.41E-12	1.29E-16
NM_001007551	RP13-36C9.6	chrX:134673654-134673595	A_32_P170547	2.08	3.02	5.20E-10	1.59E-15
XM_932169	LOC643201	chr5:175535277-175535218	A_32_P153589	2.09	1.55	1.16E-08	1.03E-05

Gene	Symbol	Chromosomal position	Probe	siCASP8AP2.3	CASP8AP2.6	siCASP8AP2.3	CASP8AP2.6
				M	M	Q	Q
NM_020436	SALL4	chr20:49834322-49834263	A_23_P109072	2.09	1.83	2.39E-13	2.02E-11
NM_032463	LAT2	chr7:73088727-73088786	A_23_P259621	2.09	2.06	8.27E-16	1.22E-15
NM_147130	NCR3	chr6:31664737-31664678	A_23_P251881	2.10	1.43	1.53E-23	1.41E-16
CR616309	CR616309	chr16:965942-965883	A_24_P930963	2.10	1.68	1.91E-15	5.17E-12
NM_022640	CSH1	chr17:59326062-59326005	A_24_P211044	2.10	2.11	2.02E-12	1.49E-12
BC028204	LOC646241	chr5:17965313-17965372	A_32_P122128	2.10	2.25	4.90E-16	2.76E-17
BC042589	BC042589	chr6:26282349-26282408	A_32_P94685	2.10	2.57	8.44E-10	1.62E-12
THC2358447	THC2358447	chr15:19108269-19108210	A_32_P116957	2.11	2.08	2.20E-29	4.03E-29
NM_000211	ITGB2	chr21:45130413-45130354	A_23_P329573	2.12	1.71	2.26E-12	1.66E-09
NM_000413	HSD17B1	chr17:37960565-37960624	A_23_P15542	2.13	1.75	4.18E-17	7.20E-14
NM_175738	RAB37	chr17:70254817-70254876	A_23_P414654	2.13	1.98	2.29E-10	1.83E-09
NM_019105	TNXB	chr6:32117001-32116943	A_23_P156708	2.13	2.11	2.83E-18	4.26E-18
NM_031313	ALPPL2	chr2:233100852-233100911	A_24_P131580	2.13	2.24	1.33E-08	3.91E-09
NM_019010	KRT20	chr17:36286117-36286058	A_23_P66854	2.13	2.57	2.43E-04	1.53E-05
NM_004626	WNT11	chr11:75575293-75575234	A_24_P253003	2.14	2.31	6.17E-08	8.52E-09
NM_003275	TMOD1	chr9:97442376-97442435	A_23_P112289	2.14	2.34	2.00E-18	4.12E-20
THC2404058	THC2404058	chr19:4474409-4474350	A_23_P39251	2.15	2.31	2.77E-15	1.49E-16
NM_199180	KIRREL2	chr19:41049757-41049816	A_23_P315451	2.15	2.51	6.17E-16	1.06E-18
NM_012242	DKK1	chr10:53746693-53746752	A_23_P24129	2.16	1.34	6.14E-08	4.79E-04
NM_000518	HBB	chr11:5203353-5203294	A_23_P203558	2.16	1.83	9.61E-20	7.85E-17
NM_016529	ATP8A2	chr13:25492329-25492388	A_23_P258612	2.16	2.26	1.72E-16	2.76E-17
ENST00000270238	ENST00000270238	chr19:53680575-53680516	A_23_P164918	2.17	1.67	3.44E-11	5.36E-08
NM_006404	PROCR	chr20:33228649-33228708	A_23_P80040	2.17	1.78	9.43E-09	1.08E-06
NM_000492	CFTR	chr7:116902504-116902563	A_23_P215720	2.17	2.72	1.99E-14	3.13E-18
NM_032265	ZMYND15	chr17:4596048-4596107	A_23_P89570	2.18	1.55	1.80E-15	1.87E-10
NM_015364	LY96	chr8:75103765-75103824	A_23_P94230	2.18	2.05	6.28E-10	4.00E-09
NM_005985	SNAI1	chr20:48038577-48038636	A_23_P131846	2.18	2.45	1.01E-10	2.33E-12
NM_000422	KRT17	chr17:37030561-37030502	A_23_P96158	2.19	1.86	2.36E-08	1.14E-06
NM_016584	IL23A	chr12:55020350-55020409	A_23_P76078	2.19	2.29	2.01E-05	1.01E-05
ENST00000285206	ENST00000285206	chr17:45543812-45543753	A_23_P421843	2.20	2.04	5.87E-17	1.10E-15
THC2377845	THC2377845	chr21:36614047-36613988	A_32_P99804	2.20	2.04	1.31E-08	9.05E-08
NM_000355	TCN2	chr22:29336296-29337897	A_23_P40611	2.20	2.10	1.15E-15	5.88E-15
CR594735	CR594735	chr11:12958276-12958217	A_32_P136376	2.20	2.13	1.87E-12	5.18E-12
NM_198282	LOC340061	chr5:138835840-138835781	A_23_P61371	2.20	2.33	8.54E-11	1.29E-11
NM_025237	SOST	chr17:39187553-39187494	A_23_P118571	2.20	2.51	1.67E-15	9.12E-18
NM_016459	PACAP	chr5:138751109-138751074	A_23_P84596	2.20	2.73	6.40E-11	3.93E-14
NM_006866	LILRA2	chr19:59778606-59778665	A_23_P142205	2.20	2.89	1.90E-13	5.44E-18
NM_000271	NPC1	chr18:19367345-19366216	A_23_P107587	2.21	1.83	2.03E-18	2.57E-15
NM_018667	SMPD3	chr16:66949810-66949751	A_23_P163567	2.21	2.06	1.40E-15	1.70E-14
NM_206965	FTCD	chr21:46381618-46381394	A_23_P91552	2.21	2.63	3.04E-22	1.60E-25
NM_000518	HBB	chr11:5203353-5203294	A_23_P203558	2.22	1.84	5.88E-13	2.22E-10
NM_080430	SELM	chr22:29825476-29825417	A_23_P6413	2.23	1.84	1.72E-13	1.01E-10
NM_001012415	SOHLH1	chr9:135811270-135811211	A_24_P307964	2.23	1.90	4.14E-16	1.42E-13
NM_000518	HBB	chr11:5203353-5203294	A_23_P203558	2.24	1.81	8.98E-20	4.91E-16
NM_003528	HIST2H2BE	chr1:146670196-146670137	A_23_P149545	2.24	2.39	8.74E-14	7.62E-15
A_32_P49035	A_32_P49035	chr8:103892421-103892480	A_32_P49035	2.25	1.37	3.49E-17	7.79E-10
NM_006664	CCL27	chr9:34652020-34651961	A_23_P135248	2.25	1.78	2.06E-20	2.53E-16
NM_000024	ADRB2	chr5:148188273-148188332	A_23_P145024	2.25	2.34	1.60E-06	7.74E-07
NM_000492	CFTR	chr7:116902504-116902563	A_23_P215720	2.25	2.88	1.56E-18	4.71E-23
NM_000518	HBB	chr11:5203353-5203294	A_23_P203558	2.26	1.85	4.96E-22	2.15E-18
NM_001671	ASGR1	chr17:7017564-7017507	A_23_P118722	2.26	1.85	3.65E-10	8.14E-08
NM_016150	ASB2	chr14:93470413-93470354	A_23_P205370	2.26	1.87	4.10E-10	6.78E-08
NM_001362	DIO3	chr14:101099227-101099286	A_23_P105923	2.26	1.94	7.68E-05	7.74E-04
NM_080614	WFDC3	chr20:43837527-43837468	A_23_P120435	2.27	1.81	3.06E-07	2.81E-05
NM_000492	CFTR	chr7:116902504-116902563	A_23_P215720	2.27	2.90	7.84E-20	2.45E-24
ENST00000311208	ENST00000311208	chr17:16686122-16686063	A_24_P882732	2.28	1.47	1.77E-12	4.17E-07

Gene	Symbol	Chromosomal position	Probe	siCASP8AP2.3	CASP8AP2.6	siCASP8AP2.3	CASP8AP2.6
				M	M	Q	Q
NM_001546	ID4	chr6:19947489-19947548	A_23_P59375	2.28	2.19	6.72E-16	2.76E-15
NM_000024	ADRB2	chr5:148188273-148188332	A_23_P145024	2.28	2.31	9.41E-07	7.35E-07
THC2301362	THC2301362	chr1:146630988-146631047	A_24_P683829	2.28	2.55	3.56E-16	3.76E-18
NM_000024	ADRB2	chr5:148188273-148188332	A_23_P145024	2.29	2.41	6.96E-07	2.57E-07
NM_003462	DNALI1	chr1:37699759-37699818	A_23_P160377	2.29	2.44	1.07E-20	7.81E-22
NM_020857	VPS18	chr15:38983178-38983237	A_24_P18802	2.30	1.36	3.48E-04	4.87E-02
NM_000878	IL2RB	chr22:35846840-35846781	A_24_P203000	2.30	1.60	7.79E-15	1.26E-09
NM_021066	HIST1H2AJ	chr6:27890225-27890166	A_23_P168014	2.30	2.11	6.42E-10	6.71E-09
NM_004994	MMP9	chr20:44078528-44078587	A_23_P40174	2.30	2.17	1.11E-10	6.26E-10
NM_206956	PRAME	chr22:21214819-21214760	A_23_P166360	2.30	2.62	2.31E-12	2.14E-14
NM_000024	ADRB2	chr5:148188273-148188332	A_23_P145024	2.31	2.41	7.22E-07	3.05E-07
NM_138281	DLX4	chr17:45406865-45406924	A_23_P164196	2.32	1.74	1.49E-08	9.52E-06
NM_012189	CABYR	chr18:19993980-19995485	A_23_P314712	2.32	2.47	1.31E-15	1.20E-16
NM_001097	ACR	chr22:49473804-49473863	A_23_P40734	2.33	2.54	6.71E-15	2.53E-16
THC2443960	THC2443960	chr20:60876011-60875952	A_24_P732106	2.33	2.70	2.63E-10	2.39E-12
NM_004895	CIAS1	chr1:243938070-243938130	A_23_P9883	2.34	2.83	5.12E-16	2.15E-19
NM_000492	CFTR	chr7:116902504-116902563	A_23_P215720	2.34	2.84	3.51E-16	1.33E-19
NM_133458	ZFP90	chr16:67157138-67157197	A_24_P176404	2.35	2.06	1.92E-17	3.04E-15
NM_001888	CRYM	chr16:21177523-21177464	A_23_P77731	2.35	2.06	3.68E-10	1.54E-08
NM_000024	ADRB2	chr5:148188273-148188332	A_23_P145024	2.36	2.42	6.06E-07	3.50E-07
NM_000024	ADRB2	chr5:148188273-148188332	A_23_P145024	2.36	2.43	8.58E-07	4.90E-07
NM_004925	AQP3	chr9:33431824-33431765	A_23_P112482	2.36	3.44	4.87E-14	1.42E-20
NM_012385	NUPR1	chr16:28456381-28456322	A_24_P270728	2.37	1.89	1.32E-26	2.78E-22
NM_003881	WISP2	chr20:42789761-42789820	A_23_P102611	2.37	2.33	5.40E-17	9.59E-17
AW972815	AW972815	chr10:126123930-126123871	A_32_P104334	2.37	2.38	1.17E-13	8.59E-14
THC2287049	THC2287049	chr8:906253-906312	A_32_P184636	2.37	2.50	1.68E-26	1.80E-27
NM_173626	SLC26A11	chr17:75841751-75841810	A_23_P125078	2.37	2.57	5.56E-08	6.58E-09
NM_001017417	CT45-1	chrX:134580522-134580962	A_32_P780817	2.37	3.21	5.27E-14	3.78E-19
NM_001129	AEBP1	chr7:43927062-43927121	A_23_P157299	2.38	2.55	1.76E-24	1.02E-25
ENST00000339446	ENST00000339446	chr11:43921914-43921973	A_32_P74409	2.38	2.80	7.53E-09	6.27E-11
NM_019055	ROBO4	chr11:124259525-124259466	A_23_P344421	2.39	1.70	1.27E-08	2.18E-05
NM_000518	HBB	chr11:5203353-5203294	A_23_P203558	2.39	1.89	1.09E-20	1.43E-16
NM_031916	ROPN1L	chr5:10514437-10517984	A_23_P121885	2.40	1.84	1.51E-13	7.23E-10
NM_020645	NRIP3	chr11:8961434-8961375	A_23_P47682	2.40	2.07	1.17E-09	6.36E-08
NM_003514	HIST1H2AM	chr6:27968615-27968556	A_24_P86389	2.40	2.35	5.41E-15	1.10E-14
NM_174947	C19orf30	chr19:4720863-4720922	A_23_P353667	2.40	2.98	4.11E-19	4.10E-23
NM_012253	TKTL1	chrX:153079471-153079530	A_23_P259901	2.40	3.28	7.71E-07	2.87E-10
NM_001276	CHI3L1	chr1:199879912-199879853	A_23_P137665	2.41	1.82	1.64E-07	4.61E-05
NM_153361	MGC42105	chr5:43316460-43316519	A_23_P254863	2.41	2.09	5.07E-24	2.66E-21
THC2355280	THC2355280	chr14:55317690-55317631	A_32_P189781	2.41	2.16	1.30E-04	7.33E-04
NM_018661	DEFB103A	chr8:7777467-7777526	A_23_P169017	2.41	2.23	4.58E-04	1.54E-03
NM_138800	TRIM43	chr2:95687184-95687243	A_23_P17173	2.41	2.68	1.23E-23	1.29E-25
NM_005825	RASGRP2	chr11:64251049-64250990	A_23_P64058	2.42	2.57	3.57E-13	3.81E-14
NM_173798	ZCCHC12	chrX:117742147-117742206	A_23_P125809	2.42	2.64	1.30E-17	3.07E-19
NM_021013	KRT34	chr17:36787603-36787544	A_23_P101054	2.44	2.25	6.40E-14	9.31E-13
NM_000024	ADRB2	chr5:148188273-148188332	A_23_P145024	2.44	2.30	1.17E-06	3.99E-06
NM_004994	MMP9	chr20:44078528-44078587	A_23_P40174	2.44	2.31	3.25E-12	1.99E-11
NM_021066	HIST1H2AJ	chr6:27890432-27890373	A_24_P394510	2.44	2.34	5.52E-08	1.62E-07
NM_080625	C20orf160	chr20:30083257-30083316	A_23_P91414	2.44	2.66	9.47E-13	3.81E-14
NM_000492	CFTR	chr7:116902504-116902563	A_23_P215720	2.45	2.95	2.76E-21	9.19E-25
NM_002996	CX3CL1	chr16:55976366-55976425	A_23_P37727	2.46	1.82	6.16E-12	3.52E-08
NM_021785	RAI2	chrX:17578094-17578035	A_23_P254165	2.46	2.40	1.51E-16	3.40E-16
NM_000024	ADRB2	chr5:148188273-148188332	A_23_P145024	2.46	2.41	1.65E-07	2.83E-07
NM_000492	CFTR	chr7:116902504-116902563	A_23_P215720	2.46	2.92	3.55E-22	2.46E-25
NM_014370	STK23	chrX:152571713-152571772	A_23_P125772	2.46	3.31	6.40E-14	6.57E-19
NM_006705	GADD45G	chr9:89450443-89450502	A_24_P120934	2.47	2.17	1.07E-10	4.22E-09

Gene	Symbol	Chromosomal position	Probe	siCASP8AP2.3	CASP8AP2.6	siCASP8AP2.3	CASP8AP2.6
				M	M	Q	Q
NM_000024	ADRB2	chr5:148188273-148188332	A_23_P145024	2.47	2.47	1.25E-07	1.32E-07
NM_002674	PMCH	chr12:101092867-101092808	A_23_P321223	2.47	2.49	1.31E-22	1.10E-22
NM_001299	CNN1	chr19:11522005-11522064	A_23_P125233	2.47	2.53	2.89E-06	2.00E-06
NM_000492	CFTR	chr7:116902504-116902563	A_23_P215720	2.47	2.87	5.35E-23	8.86E-26
NM_030667	PTPRO	chr12:15640955-15641014	A_23_P204304	2.48	2.12	6.61E-15	1.64E-12
NM_144613	COX6B2	chr19:60552949-60552890	A_24_P267523	2.48	2.32	1.05E-08	6.31E-08
NM_000076	CDKN1C	chr11:2861538-2861479	A_23_P428129	2.48	2.69	9.95E-10	9.68E-11
NM_000518	HBB	chr11:5203353-5203294	A_23_P203558	2.49	1.88	5.25E-20	3.92E-15
NM_001772	CD33	chr19:56434810-56434869	A_23_P331748	2.49	2.41	4.59E-16	1.37E-15
NM_015444	TMEM158	chr3:45241210-45241151	A_23_P369899	2.50	1.97	5.38E-08	9.30E-06
NM_000024	ADRB2	chr5:148188273-148188332	A_23_P145024	2.51	2.45	2.56E-07	4.57E-07
NM_000518	HBB	chr11:5203353-5203294	A_23_P203558	2.52	1.94	4.60E-23	2.88E-18
NM_153344	C6orf141	chr6:49630593-49630652	A_23_P386398	2.52	2.44	2.42E-21	8.71E-21
NM_000518	HBB	chr11:5203353-5203294	A_23_P203558	2.54	1.88	6.27E-21	1.05E-15
ENST00000309878	ENST00000309878	chr1:158068094-158068153	A_32_P214665	2.54	2.32	6.61E-26	4.30E-24
NM_004994	MMP9	chr20:44078528-44078587	A_23_P40174	2.55	2.29	6.63E-13	2.41E-11
NM_004811	LPXN	chr11:58051466-58051407	A_23_P87150	2.55	2.35	1.51E-15	2.55E-14
AK055306	AK055306	chr22:35785941-35786000	A_24_P117368	2.55	2.40	4.22E-06	1.41E-05
NM_020989	CRYGC	chr2:208818473-208818414	A_23_P142606	2.55	2.82	1.55E-23	2.23E-25
NM_004994	MMP9	chr20:44078528-44078587	A_23_P40174	2.56	2.30	3.37E-12	8.71E-11
NM_007021	C10orf10	chr10:44791775-44791716	A_24_P329795	2.56	2.37	3.67E-09	3.05E-08
NM_000518	HBB	chr11:5203353-5203294	A_23_P203558	2.57	2.00	4.45E-22	1.73E-17
NM_003122	SPINK1	chr5:147187778-147184404	A_23_P214079	2.57	2.97	7.00E-11	6.01E-13
ENST00000343959	ENST00000343959	chr10:88774367-88774426	A_32_P34138	2.58	2.26	3.00E-13	2.29E-11
XM_942822	LOC646626	chr1:85455417-85455476	A_32_P703	2.58	2.63	9.89E-10	6.00E-10
NM_004994	MMP9	chr20:44078528-44078587	A_23_P40174	2.59	2.26	5.42E-13	4.16E-11
NM_014467	SRPX2	chrX:99732118-99732177	A_23_P136978	2.60	2.04	8.25E-20	1.24E-15
NM_000518	HBB	chr11:5203353-5203294	A_23_P203558	2.60	2.05	1.67E-23	4.47E-19
NM_000552	VWF	chr12:5928503-5928444	A_23_P105562	2.60	2.22	2.01E-25	2.36E-22
NM_004561	OVOL1	chr11:65321095-65321154	A_23_P202810	2.60	3.09	1.93E-23	1.26E-26
NM_004031	IRF7	chr11:602702-602643	A_24_P378019	2.61	2.01	5.50E-12	1.08E-08
NM_000552	VWF	chr12:5928503-5928444	A_23_P105562	2.61	2.26	1.14E-24	6.38E-22
NM_004994	MMP9	chr20:44078528-44078587	A_23_P40174	2.61	2.29	7.10E-12	3.87E-10
NM_000641	IL11	chr19:60568830-60568771	A_23_P67169	2.61	2.50	5.97E-11	2.26E-10
NM_004994	MMP9	chr20:44078528-44078587	A_23_P40174	2.62	2.24	4.04E-12	5.09E-10
NM_022052	NXF3	chrX:102136998-102136939	A_23_P171336	2.62	2.72	8.84E-16	1.88E-16
NM_024501	HOXD1	chr2:176880879-176880938	A_23_P120243	2.62	3.03	4.44E-23	8.86E-26
NM_000552	VWF	chr12:5928503-5928444	A_23_P105562	2.63	2.25	5.41E-24	4.07E-21
NM_004994	MMP9	chr20:44078528-44078587	A_23_P40174	2.63	2.25	5.41E-12	6.64E-10
ENST00000294663	ENST00000294663	chr1:89284498-89284439	A_24_P36898	2.63	2.34	8.63E-25	1.59E-22
NM_178504	DNHD2	chr3:57302896-57302837	A_32_P14721	2.63	2.48	5.64E-17	5.27E-16
NM_173571	RP6-166C19.1	chrX:119794660-119794601	A_24_P332857	2.63	3.26	1.26E-26	6.25E-31
NM_004994	MMP9	chr20:44078528-44078587	A_23_P40174	2.64	2.26	3.93E-12	4.31E-10
NM_001097	ACR	chr22:49473749-49473808	A_32_P191262	2.65	2.56	1.65E-15	5.19E-15
NM_014224	PGA5	chr11:60775243-60775302	A_23_P150547	2.67	2.85	6.03E-24	3.28E-25
NM_004994	MMP9	chr20:44078528-44078587	A_23_P40174	2.69	2.29	6.39E-13	1.11E-10
NM_000552	VWF	chr12:5928503-5928444	A_23_P105562	2.71	2.22	7.78E-26	5.71E-22
A_23_P170719	A_23_P170719	chr19:008344985-008345228	A_23_P170719	2.71	2.46	1.49E-12	3.36E-11
BC034407	LOC643201	chr5:175533068-175533009	A_24_P526190	2.74	2.50	1.51E-12	2.96E-11
NM_000552	VWF	chr12:5928503-5928444	A_23_P105562	2.75	2.21	9.59E-25	1.33E-20
NM_000064	C3	chr19:6630434-6630177	A_23_P101407	2.75	2.44	3.46E-20	4.35E-18
NM_024508	ZBED2	chr3:112794594-112794535	A_23_P113793	2.77	2.54	1.92E-05	9.56E-05
NM_003540	HIST1H4F	chr6:26348797-26348856	A_23_P359540	2.78	2.44	1.14E-16	1.42E-14
NM_015931	C3orf32	chr3:8636402-8636343	A_23_P144005	2.79	2.74	3.45E-16	6.08E-16
ENST00000341016	ENST00000341016	chrX:37606607-37606666	A_32_P90859	2.79	2.95	1.67E-23	1.90E-24
NM_000552	VWF	chr12:5928503-5928444	A_23_P105562	2.80	2.12	2.48E-26	4.23E-21

Gene	Symbol	Chromosomal position	Probe	siCASP8AP2.3	CASP8AP2.6	siCASP8AP2.3	CASP8AP2.6
				M	M	Q	Q
NM_024490	ATP10A	chr15:23475027-23474968	A_24_P215765	2.81	2.84	1.35E-14	9.13E-15
NM_014767	SPOCK2	chr10:73492248-73492189	A_23_P161280	2.81	3.07	3.77E-15	1.31E-16
NM_016571	GLULD1	chr6:64047639-64047580	A_23_P93169	2.81	3.32	8.06E-23	7.51E-26
NM_000552	VWF	chr12:5928503-5928444	A_23_P105562	2.82	2.13	1.85E-24	3.56E-19
NM_000552	VWF	chr12:5928503-5928444	A_23_P105562	2.82	2.23	7.62E-26	2.17E-21
NM_021972	SPHK1	chr17:71895336-71895395	A_23_P38106	2.82	2.69	1.52E-08	5.09E-08
NM_000552	VWF	chr12:5928503-5928444	A_23_P105562	2.83	2.22	1.95E-26	9.35E-22
NM_000257	MYH7	chr14:22952826-22951880	A_24_P282383	2.83	2.67	1.42E-28	2.06E-27
NM_145008	YPEL4	chr11:57169232-57169173	A_23_P300220	2.84	2.65	2.98E-13	2.78E-12
NM_014420	DKK4	chr8:42351498-42351439	A_23_P94275	2.84	2.98	1.53E-08	4.40E-09
NM_053017	ART5	chr11:3617501-3616836	A_23_P427122	2.85	3.21	5.09E-27	1.99E-29
NM_017820	FLJ20433	chr9:137494015-137479932	A_24_P49183	2.86	1.96	4.37E-22	2.19E-15
NM_016084	RASD1	chr17:17339388-17339329	A_24_P348006	2.86	3.22	6.94E-21	4.06E-23
NM_000552	VWF	chr12:5928503-5928444	A_23_P105562	2.87	2.27	1.62E-26	5.44E-22
BC004857	ZC3H7B	chr5:067654339-067654280	A_24_P797678	2.87	2.74	4.41E-27	3.88E-26
NM_001012631	IL32	chr16:3059309-3059368	A_23_P15146	2.87	2.80	6.68E-09	1.37E-08
NM_182573	LYPD5	chr19:48992375-48992316	A_24_P323148	2.87	2.84	2.83E-13	3.42E-13
NM_000426	LAMA2	chr6:129875315-129877253	A_23_P70719	2.87	3.52	1.08E-22	1.78E-26
THC2371963	THC2371963	chr15:60243129-60243070	A_24_P788878	2.88	2.84	7.80E-37	1.70E-36
NM_005618	DLL1	chr6:170509333-170509274	A_23_P167920	2.88	3.00	3.71E-19	6.25E-20
BC063625	KRTAP2-4	chr17:36469278-36469219	A_32_P24382	2.89	2.56	1.20E-15	1.02E-13
BC094802	FLJ36166	chr7:102429894-102419933	A_32_P78385	2.89	2.66	2.76E-39	1.15E-37
NM_003064	SLPI	chr20:43315693-43315634	A_24_P190472	2.90	3.00	1.24E-20	2.64E-21
NM_001085	SERPINA3	chr14:94158435-94158494	A_23_P2920	2.92	2.15	2.44E-21	6.24E-16
BG188151	BG188151	chr5:082251307-082251248	A_24_P532864	2.92	3.29	1.28E-04	2.30E-05
AK098637	AK098637	chr7:156819332-156819391	A_24_P358063	2.93	2.77	1.46E-14	1.01E-13
NM_030657	LIM2	chr19:56575199-56575140	A_23_P130435	2.94	2.88	2.93E-28	8.78E-28
NM_173571	RP6-166C19.1	chrX:119795555-119794609	A_24_P302070	2.94	3.30	4.60E-23	3.28E-25
NM_198517	TBC1D10C	chr11:66934045-66934104	A_24_P392201	2.96	2.14	2.69E-12	3.59E-08
NM_014587	SOX8	chr16:976894-976953	A_23_P66137	2.96	2.44	1.33E-07	8.25E-06
NM_080593	HIST1H2BK	chr6:27221956-27221897	A_23_P145238	2.96	3.34	4.33E-10	1.12E-11
A_24_P152345	A_24_P152345	chr3:115305098-115305037	A_24_P152345	2.97	3.06	9.83E-16	2.91E-16
NM_003530	HIST1H3D	chr6:26305296-26305237	A_24_P217834	2.99	3.09	3.58E-13	1.01E-13
NM_003548	HIST2H4A	chr1:146617469-146617528	A_23_P436281	3.01	2.61	1.21E-20	4.73E-18
NM_000142	FGFR3	chr4:1777594-1777653	A_23_P500501	3.01	3.43	1.24E-17	4.97E-20
THC2434166	THC2434166	chr17:22423693-22423634	A_24_P653054	3.02	2.78	7.99E-27	3.24E-25
NM_002471	MYH6	chr14:22921493-22921070	A_23_P37164	3.02	3.06	1.29E-30	6.04E-31
THC2406238	THC2406238	chr20:44962469-44962436	A_32_P175125	3.03	3.18	2.47E-23	3.31E-24
NM_000095	COMP	chr19:18756093-18756034	A_23_P90436	3.04	2.88	7.03E-24	8.86E-23
NM_133431	XAGE1	chrX:52074162-52074221	A_24_P271696	3.04	3.81	2.12E-19	1.37E-23
NM_001803	CD52	chr1:26331027-26331086	A_23_P85800	3.05	2.39	1.39E-13	3.59E-10
CR603668	CR603668	chr19:15807035-15807092	A_32_P200238	3.07	2.54	2.70E-09	3.28E-07
NM_005382	NEF3	chr8:24831089-24831148	A_24_P264832	3.07	2.80	1.31E-19	6.06E-18
NM_025047	ARL14	chr3:161878350-161878409	A_23_P92161	3.07	3.00	3.82E-10	7.74E-10
BC012185	HIST1H3G	chr6:26377826-26377767	A_32_P794894	3.07	3.54	7.43E-30	8.23E-33
NM_004165	RRAD	chr16:65513735-65513676	A_24_P262127	3.08	3.42	7.66E-10	3.52E-11
NM_003733	OASL	chr12:119921099-119921040	A_23_P139786	3.09	3.30	2.01E-21	1.24E-22
NM_005627	SGK	chr6:134532408-134532349	A_23_P19673	3.10	3.13	6.73E-14	4.21E-14
NM_033445	HIST3H2A	chr1:224952044-224951985	A_23_P149301	3.10	3.17	1.82E-16	7.19E-17
NM_013351	TBX21	chr17:43178388-43178447	A_23_P141555	3.10	3.70	5.86E-20	3.27E-23
NM_000240	MAOA	chrX:43359931-43359990	A_23_P83857	3.11	3.04	4.25E-12	8.97E-12
NM_021062	HIST1H2BB	chr6:26151544-26151485	A_23_P111054	3.14	2.93	2.36E-15	2.72E-14
NM_001678	ATP1B2	chr17:7501357-7501416	A_24_P31275	3.16	2.67	1.79E-19	1.68E-16
NM_003517	HIST2H2AC	chr1:146671911-146671970	A_24_P8721	3.17	2.97	3.73E-16	3.91E-15
NM_182634	FLJ36166	chr7:102448483-102448424	A_24_P186608	3.19	2.77	1.22E-26	6.38E-24
NM_003524	HIST1H2BH	chr6:26360201-26360260	A_23_P366216	3.19	3.04	4.08E-16	2.19E-15

Gene	Symbol	Chromosomal position	Probe	siCASP8AP2.3	CASP8AP2.6	siCASP8AP2.3	CASP8AP2.6
				M	M	Q	Q
NM_006398	UBD	chr6:29631515-29631456	A_23_P81898	3.20	2.98	3.28E-17	4.60E-16
NM_153187	SLC22A1	chr6:160531296-160535028	A_23_P145569	3.20	3.15	5.67E-28	1.17E-27
NM_002523	NPTX2	chr7:97903541-97903600	A_23_P82651	3.20	3.44	1.51E-15	9.19E-17
ENST00000299502	ENST00000299502	chr18:59721938-59721997	A_23_P153185	3.21	2.56	1.26E-23	2.15E-19
NM_002183	IL3RA	chrX:1545314-1545373	A_32_P217750	3.22	3.19	1.96E-15	2.70E-15
NM_015193	ARC	chr8:143689640-143689581	A_23_P365738	3.22	3.26	1.22E-24	8.81E-25
NM_139314	ANGPTL4	chr19:8345169-8345228	A_23_P159325	3.24	2.85	3.38E-10	1.19E-08
BC063625	KRTAP2-4	chr17:36469163-36469104	A_32_P24376	3.25	2.77	2.25E-17	9.32E-15
NM_003519	HIST1H2BL	chr6:27883324-27883265	A_23_P8013	3.25	3.10	1.72E-14	8.91E-14
NM_003527	HIST1H2BO	chr6:27969567-27969626	A_23_P59069	3.25	3.23	1.97E-17	2.20E-17
NM_003956	CH25H	chr10:90955840-90955781	A_23_P86470	3.25	3.77	3.98E-13	1.67E-15
NM_012276	LILRA4	chr19:59540180-59540121	A_23_P90497	3.26	3.61	1.90E-22	2.45E-24
NM_004750	CRLF1	chr19:18565169-18565110	A_23_P56197	3.28	2.74	4.85E-20	7.30E-17
AA837799	AA837799	chr22:31522983-31523042	A_23_P211468	3.28	2.94	1.54E-09	2.93E-08
NM_005461	MAFB	chr20:38748283-38748224	A_23_P17345	3.29	2.90	3.34E-21	6.45E-19
NM_003521	HIST1H2BM	chr6:27890982-27891041	A_24_P3783	3.29	3.39	2.61E-14	8.13E-15
NM_005319	HIST1H1C	chr6:26164088-26164029	A_23_P122443	3.33	3.40	1.27E-13	5.92E-14
NM_002421	MMP1	chr11:102165966-102165907	A_23_P1691	3.34	2.76	4.62E-12	1.56E-09
NM_003277	CLDN5	chr22:17885172-17885113	A_23_P6321	3.35	2.90	2.98E-14	4.21E-12
NM_005321	HIST1H1E	chr6:26264907-26264966	A_23_P7976	3.35	3.15	1.45E-13	1.18E-12
NM_002421	MMP1	chr11:102165966-102165907	A_23_P1691	3.36	2.83	2.26E-12	4.48E-10
NM_021018	HIST1H3F	chr6:26358626-26358567	A_23_P30799	3.36	3.15	2.65E-14	2.41E-13
NM_003526	HIST1H2BC	chr6:26232021-26231962	A_23_P93180	3.36	3.30	5.81E-17	1.03E-16
NM_002421	MMP1	chr11:102165966-102165907	A_23_P1691	3.37	2.79	1.75E-12	6.24E-10
NM_015162	ACSBG1	chr15:76252997-76250875	A_23_P54488	3.37	3.44	1.21E-27	5.10E-28
NM_000095	COMP	chr19:18754877-18754726	A_24_P264943	3.38	3.42	1.42E-26	1.01E-26
NM_012445	SPON2	chr4:1150627-1150568	A_23_P121533	3.39	3.66	3.32E-14	1.69E-15
NM_004925	AQP3	chr9:33431624-33431565	A_23_P112481	3.39	4.19	1.59E-17	2.17E-21
ENST00000356177	ENST00000356177	chr6:27884580-27884639	A_24_P97914	3.41	3.26	2.39E-23	1.81E-22
NM_003523	HIST1H2BE	chr6:26292319-26292378	A_23_P30776	3.42	3.44	2.47E-16	1.72E-16
BC068044	BC068044	chr1:146627841-146627900	A_24_P544661	3.44	3.32	2.42E-17	9.95E-17
NM_052942	GBP5	chr1:89438467-89438408	A_23_P74290	3.44	3.38	1.94E-27	4.34E-27
NM_003522	HIST1H2BF	chr6:26308082-26308141	A_23_P42178	3.44	3.41	3.50E-16	4.47E-16
NM_017539	DNAH3	chr16:20854186-20852287	A_23_P329340	3.46	2.35	2.79E-16	1.72E-10
NM_003520	HIST1H2BN	chr6:27914524-27914583	A_23_P402081	3.46	3.45	6.89E-19	7.27E-19
NM_033423	GZMH	chr14:24145608-24145549	A_23_P128993	3.47	1.99	1.96E-16	1.49E-08
NM_003537	HIST1H3B	chr6:26139960-26139901	A_23_P93258	3.48	3.31	1.00E-13	5.69E-13
NM_002421	MMP1	chr11:102165966-102165907	A_23_P1691	3.49	2.83	4.71E-12	2.52E-09
NM_032599	NYD-SP18	chr7:127965510-127965569	A_23_P134204	3.49	3.09	2.31E-20	3.51E-18
NM_175055	HIST3H2BB	chr1:224952760-224952819	A_23_P332992	3.50	3.42	2.92E-16	6.04E-16
NM_003525	HIST1H2BI	chr6:26381354-26381413	A_23_P111041	3.51	3.69	7.27E-15	1.12E-15
NM_001824	CKM	chr19:50501571-50501512	A_23_P50250	3.52	3.34	1.14E-18	9.12E-18
NM_003528	HIST2H2BE	chr1:146671162-146671103	A_24_P156911	3.52	3.49	6.07E-19	7.82E-19
NM_003719	PDE8B	chr5:76758549-76758608	A_24_P197537	3.53	3.25	1.54E-16	3.39E-15
NM_002421	MMP1	chr11:102165966-102165907	A_23_P1691	3.55	2.85	9.57E-12	6.49E-09
NM_002307	LGALS7	chr19:43973215-43973274	A_23_P108062	3.55	3.33	3.57E-19	5.01E-18
ENST00000314088	ENST00000314088	chr6:26246801-26246860	A_23_P167983	3.56	3.29	4.91E-11	5.10E-10
NM_002421	MMP1	chr11:102165966-102165907	A_23_P1691	3.58	2.94	9.49E-12	3.25E-09
NM_005320	HIST1H1D	chr6:26342737-26342678	A_24_P260639	3.59	3.22	1.39E-13	5.45E-12
NM_017445	H2BFS	chr21:43809613-43809672	A_23_P40470	3.59	3.56	3.69E-18	5.12E-18
NM_002421	MMP1	chr11:102165966-102165907	A_23_P1691	3.61	2.95	2.24E-11	7.39E-09
NM_003064	SLPI	chr20:43314423-43314364	A_23_P91230	3.61	3.51	3.00E-21	9.32E-21
NM_002421	MMP1	chr11:102165966-102165907	A_23_P1691	3.62	3.02	1.01E-11	2.27E-09
NM_003837	FBP2	chr9:94400704-94400645	A_23_P94472	3.64	2.97	3.92E-29	4.03E-25
NM_002421	MMP1	chr11:102165966-102165907	A_23_P1691	3.65	2.89	3.04E-11	2.33E-08
NM_004165	RRAD	chr16:65513189-65513130	A_23_P88849	3.68	3.79	3.53E-09	1.53E-09

Gene	Symbol	Chromosomal position	Probe	siCASP8AP2.3	CASP8AP2.6	siCASP8AP2.3	CASP8AP2.6
				M	M	Q	Q
NM_002421	MMP1	chr11:102165966-102165907	A_23_P1691	3.70	2.93	4.08E-12	4.55E-09
NM_005211	CSF1R	chr5:149413113-149413054	A_23_P110791	3.72	3.75	8.87E-23	7.05E-23
NM_001005217	FRG2	chr4:191321196-191321137	A_24_P623782	3.73	4.50	1.13E-27	1.61E-31
NM_005217	DEFA3	chr8:6860980-6860921	A_23_P31816	3.74	3.10	4.28E-25	1.61E-21
NM_003528	HIST2H2BE	chr1:146669546-146669487	A_24_P148321	3.78	3.63	5.33E-13	1.93E-12
NM_002307	LGALS7	chr19:43974108-43974167	A_24_P348118	3.80	3.60	9.68E-19	8.69E-18
NM_003516	HIST2H2AA3	chr1:146635701-146635760	A_23_P309381	3.80	4.24	9.00E-09	4.19E-10
NM_021058	HIST1H2BJ	chr6:27208356-27208297	A_24_P55148	3.81	3.81	3.69E-20	3.41E-20
NM_003530	HIST1H3D	chr6:26305256-26305197	A_23_P219045	3.82	3.83	1.30E-14	1.10E-14
NM_052957	ACRC	chrX:70616312-70616371	A_23_P171237	3.82	3.84	4.34E-15	3.31E-15
NM_005913	MC5R	chr18:13816278-13816337	A_23_P50039	3.83	3.12	1.92E-26	1.74E-22
NM_003516	HIST2H2AA3	chr1:146636178-146636234	A_23_P103981	3.83	3.72	3.55E-11	8.65E-11
NM_021063	HIST1H2BD	chr6:26266453-26266512	A_24_P146211	3.87	3.77	1.47E-19	4.38E-19
NM_175065	HIST2H2AB	chr1:146672317-146672258	A_24_P68631	3.88	3.77	1.18E-17	3.43E-17
THC2333977	THC2333977	chr6:26387020-26386961	A_32_P97780	3.90	3.61	4.14E-34	1.60E-32
NM_000805	GAST	chr17:37125599-37125658	A_23_P159191	3.93	3.65	1.35E-26	4.02E-25
NM_003518	HIST1H2BG	chr6:26324755-26324696	A_23_P167997	3.93	3.78	1.29E-20	6.51E-20
NM_198393	TEX14	chr17:53989168-53989109	A_23_P54996	3.96	3.95	1.60E-24	2.24E-24
NM_206818	OSCAR	chr19:59289969-59289910	A_23_P50368	3.96	3.98	1.42E-25	1.39E-25
NM_005218	DEFB1	chr8:6715650-6715591	A_23_P71480	3.97	3.16	2.39E-29	7.54E-25
NM_145056	MGC15476	chr19:51842805-51842746	A_23_P360964	4.00	3.76	4.30E-29	8.78E-28
NM_002307	LGALS7	chr19:43974125-43974184	A_24_P238250	4.01	3.73	3.03E-17	4.91E-16
NM_003543	HIST1H4H	chr6:26393535-26393476	A_23_P323685	4.03	3.97	1.13E-21	2.14E-21
NM_016378	VCX2	chrX:7948341-7948090	A_24_P230282	4.03	4.10	6.99E-20	3.02E-20
NM_145894	KLK12	chr19:56224231-56224172	A_23_P500010	4.07	3.90	3.66E-31	2.47E-30
NM_003514	HIST1H2AM	chr6:27968545-27968486	A_32_P221799	4.10	3.58	3.17E-22	8.93E-20
NM_004043	ASMT	chrX:1805699-1805758	A_23_P255535	4.13	4.15	3.11E-35	3.44E-35
A_23_P170713	A_23_P170713	chr6:026164200-026164151	A_23_P170713	4.17	3.65	3.61E-14	3.32E-12
NM_021064	HIST1H2AG	chr6:27210269-27210328	A_24_P414658	4.19	4.05	9.07E-25	4.31E-24
NM_021065	HIST1H2AD	chr6:26307121-26307062	A_23_P428184	4.23	4.21	6.78E-16	6.99E-16
NM_006840	LILRB5	chr19:59446311-59446252	A_23_P4773	4.29	4.49	6.30E-33	7.89E-34
NM_003803	MYOM1	chr18:3057225-3057166	A_23_P96271	4.31	4.58	1.24E-13	1.17E-14
NM_021052	HIST1H2AE	chr6:26325533-26325592	A_23_P59045	4.32	3.90	9.87E-24	7.90E-22
NM_001911	CTSG	chr14:24112687-24112628	A_23_P140384	4.33	4.05	4.81E-27	1.02E-25
NM_004679	VCY	chrY:14606849-14606908	A_23_P11390	4.35	4.63	8.95E-21	6.18E-22
NM_001734	C1S	chr12:7048354-7048413	A_23_P2492	4.38	4.15	1.56E-24	1.91E-23
NM_004131	GZMB	chr14:24170135-24170076	A_23_P117602	4.47	4.28	1.86E-16	9.32E-16
A_23_P156609	A_23_P156609	chr6:026296873-026296814	A_23_P156609	4.49	3.85	3.02E-27	2.76E-24
NM_018088	FAM90A1	chr12:8265371-8265312	A_24_P643028	4.50	5.22	2.37E-17	4.73E-20
NM_003536	HIST1H3H	chr6:27886003-27886062	A_23_P333484	4.52	4.05	3.48E-25	4.51E-23
NM_000547	TPO	chr2:1525339-1525398	A_24_P257224	4.54	4.39	3.98E-39	4.79E-38
NM_175616	FIS	chr5:95221458-95221517	A_32_P128209	4.57	4.44	2.41E-30	8.59E-30
NM_018088	FAM90A1	chr12:8265462-8265403	A_23_P151059	4.57	5.14	1.27E-21	8.20E-24
ENST00000382595	ENST00000382595	chr8:7659448-7659507	A_24_P515319	4.57	5.31	6.27E-21	9.84E-24
NM_016084	RASD1	chr17:17338571-17338512	A_23_P118392	4.58	5.16	2.56E-19	1.75E-21
NM_024318	LILRA6	chr19:59436220-59436161	A_23_P368711	4.60	4.72	2.92E-30	7.65E-31
NM_031958	KRTAP3-1	chr17:36418428-36418369	A_23_P107454	4.67	3.95	5.40E-25	7.81E-22
NM_182908	DHRS2	chr14:23184340-23184400	A_23_P321501	4.67	4.67	4.67E-24	5.32E-24
NM_016379	VCX3A	chrX:6311736-6311677	A_24_P23979	4.79	4.83	1.48E-20	1.06E-20
NM_013452	VCX	chrX:7621492-7621551	A_24_P245976	4.82	4.97	4.33E-20	1.18E-20
NM_153479	CSAG1	chrX:151579821-151579880	A_24_P11061	4.83	4.53	2.65E-35	5.42E-34
NM_006864	LILRB3	chr19:59412644-59412585	A_32_P70158	4.84	4.36	2.20E-29	2.68E-27
NM_182908	DHRS2	chr14:23184472-23184531	A_23_P48570	4.85	4.93	5.27E-26	3.18E-26
NM_016378	VCX2	chrX:7947897-7947838	A_32_P33083	4.85	5.02	8.97E-21	2.06E-21
NM_006865	LILRA3	chr19:59493967-59493908	A_23_P79094	4.90	5.27	2.29E-31	6.16E-33
ENST00000382591	ENST00000382591	chr8:7667387-7667446	A_24_P903680	5.03	5.36	6.33E-20	4.14E-21

Gene	Symbol	Chromosomal position	Probe	siCASP8AP2.3	CASP8AP2.6	siCASP8AP2.3	CASP8AP2.6
				M	M	Q	Q
NM_005767	P2RY5	chr13:47883731-47883672	A_23_P2705	5.33	4.69	7.28E-25	2.01E-22
ENST00000369158	ENST00000369158	chr1:146638814-146638873	A_23_P435029	5.37	5.07	7.26E-24	9.50E-23
NM_004120	GBP2	chr1:89290181-89287917	A_23_P85693	5.46	5.19	7.67E-36	8.56E-35
ENST00000356177	ENST00000356177	chr6:27886335-27886393	A_23_P8004	5.49	5.27	2.36E-34	1.79E-33
NM_005123	NR1H4	chr12:99459659-99459718	A_23_P25396	5.61	5.14	2.31E-38	1.70E-36
NM_021076	NEFH	chr22:28211748-28211807	A_23_P300600	5.72	6.29	1.81E-34	2.43E-36
A_23_P251002	A_23_P251002	chr6:026389784-026389729	A_23_P251002	6.40	6.33	1.46E-20	2.21E-20
X15675	X15675	chr11:67151358-67151417	A_32_P32406	6.65	6.18	7.15E-36	1.97E-34
