# Supplementary material for: Systems-wide RNAi analysis of CASP8AP2/FLASH shows transcriptional deregulation of the replication-dependent histone genes and extensive effects on the transcriptome of colorectal cancer cells
Source: Mol Cancer. 2012 Jan 4;11:1. doi: 10.1186/1476-4598-11-1 (PMC3281783; doi:10.1186/1476-4598-11-1)
Supplement: Additional file 8 — Table S6. Significant gene ontology categories within CASP8AP2/FLASH RNAi signatures. [file 1476-4598-11-1-S8.PDF]

Additional file 8, Table S6

Gene Ontology Category	Gene Ontology enrichment in CASP8AP2 RNAi signatures					
	72 hours: Experiment 1		48 hours: Experiment 2		72 hours: Experiment 2	
	Minus log 10 B-H p-value	B-H p-value	Minus log 10 B-H p-value	B-H p-value	Minus log 10 B-H p-value	B-H p-value
Cancer	19.149	7.09E-20	5.308	4.92E-06	6.410	3.89E-07
Cellular Growth and Proliferation	16.604	2.49E-17	4.527	2.97E-05	7.316	4.83E-08
Cellular Movement	12.377	4.20E-13	2.462	3.45E-03	6.410	3.89E-07
Inflammatory Response	11.870	1.35E-12	5.308	4.92E-06	7.316	4.83E-08
Cellular Development	11.827	1.49E-12	3.389	4.08E-04	5.420	3.80E-06
Hematological System Development and Function	9.277	5.28E-10	3.115	7.67E-04	5.987	1.03E-06
Cell Death	9.277	5.29E-10	2.842	1.44E-03	4.037	9.18E-05
Gastrointestinal Disease	9.142	7.21E-10	2.827	1.49E-03	2.355	4.42E-03
Cell-To-Cell Signaling and Interaction	9.038	9.17E-10	3.924	1.19E-04	5.914	1.22E-06
Reproductive System Disease	8.783	1.65E-09	2.701	1.99E-03	1.917	1.21E-02
Hematopoiesis	7.889	1.29E-08	2.582	2.62E-03	4.559	2.76E-05
Cellular Function and Maintenance	7.504	3.13E-08	2.582	2.62E-03	4.044	9.03E-05
Tissue Development	7.484	3.28E-08	2.514	3.06E-03	2.355	4.42E-03
Organismal Survival	7.481	3.30E-08	3.415	3.85E-04	5.316	4.83E-06
Dermatological Diseases and Conditions	7.245	5.69E-08	3.153	7.03E-04	3.648	2.25E-04
Connective Tissue Disorders	7.225	5.95E-08	1.580	2.63E-02	2.181	6.59E-03
Immunological Disease	7.225	5.95E-08	2.310	4.90E-03	2.903	1.25E-03
Inflammatory Disease	7.225	5.95E-08	1.481	3.30E-02	2.194	6.40E-03
Skeletal and Muscular Disorders	7.225	5.95E-08	2.144	7.18E-03	2.181	6.59E-03
Cell-mediated Immune Response	7.123	7.54E-08	2.487	3.26E-03	3.777	1.67E-04
Organismal Injury and Abnormalities	6.860	1.38E-07	3.153	7.03E-04	2.378	4.19E-03
Cardiovascular System Development and Function	6.070	8.52E-07	3.115	7.67E-04	3.939	1.15E-04
Organismal Development	6.070	8.52E-07	3.115	7.67E-04	2.393	4.05E-03
Respiratory Disease	5.836	1.46E-06	2.701	1.99E-03	2.030	9.34E-03
Immune Cell Trafficking	5.502	3.15E-06	2.462	3.45E-03	4.334	4.63E-05
Hepatic System Disease	5.322	4.76E-06	1.140	7.25E-02	1.848	1.42E-02
Tissue Morphology	4.483	3.29E-05	3.018	9.59E-04	3.959	1.10E-04
Tumor Morphology	4.275	5.31E-05	2.582	2.62E-03	2.115	7.67E-03
Nutritional Disease	4.081	8.29E-05	1.118	7.62E-02	1.833	1.47E-02
Lipid Metabolism	4.057	8.77E-05	1.395	4.03E-02	1.770	1.70E-02
Molecular Transport	4.057	8.77E-05	2.172	6.73E-03	3.036	9.20E-04
Small Molecule Biochemistry	4.057	8.77E-05	1.801	1.58E-02	3.321	4.77E-04
Cell Signaling	4.026	9.42E-05	1.710	1.95E-02	3.036	9.20E-04
Vitamin and Mineral Metabolism	4.026	9.42E-05	1.710	1.95E-02	3.036	9.20E-04
Cellular Compromise	3.680	2.09E-04	3.153	7.03E-04	2.903	1.25E-03
Cardiovascular Disease	3.644	2.27E-04	2.701	1.99E-03	1.456	3.50E-02
Hematological Disease	3.644	2.27E-04	2.002	9.96E-03	2.355	4.42E-03
Ophthalmic Disease	3.538	2.90E-04	2.310	4.90E-03	2.680	2.09E-03
Developmental Disorder	3.275	5.31E-04	2.310	4.90E-03	2.706	1.97E-03
Genetic Disorder	3.194	6.39E-04	2.184	6.54E-03	1.445	3.59E-02
Skeletal and Muscular System Development and Function	3.188	6.49E-04	1.449	3.56E-02	1.873	1.34E-02
Cell Morphology	3.152	7.04E-04	1.580	2.63E-02	1.764	1.72E-02
Embryonic Development	3.032	9.28E-04	1.762	1.73E-02	1.631	2.34E-02
Antimicrobial Response	2.955	1.11E-03	1.323	4.75E-02	1.721	1.90E-02
Infectious Disease	2.742	1.81E-03	1.893	1.28E-02	3.171	6.74E-04
Lymphoid Tissue Structure and Development	2.695	2.02E-03	1.893	1.28E-02	2.115	7.67E-03
Connective Tissue Development and Function	2.648	2.25E-03	1.449	3.56E-02	2.061	8.69E-03
Respiratory System Development and Function	2.569	2.70E-03	1.444	3.60E-02	1.102	7.90E-02
Nucleic Acid Metabolism	2.488	3.25E-03	1.801	1.58E-02	1.917	1.21E-02
Organismal Functions	2.488	3.25E-03	1.629	2.35E-02	n.s	n.s
Cell Cycle	2.451	3.54E-03	n.s	n.s	1.830	1.48E-02
Carbohydrate Metabolism	2.428	3.73E-03	n.s	n.s	3.321	4.77E-04
Neurological Disease	2.398	4.00E-03	1.355	4.42E-02	1.521	3.01E-02
Antigen Presentation	2.381	4.16E-03	1.449	3.56E-02	2.052	8.87E-03
Free Radical Scavenging	2.310	4.90E-03	1.506	3.12E-02	2.061	8.69E-03
Post-Translational Modification	2.309	4.91E-03	1.219	6.04E-02	1.565	2.72E-02
Behavior	2.180	6.60E-03	1.523	3.00E-02	n.s	n.s
Endocrine System Disorders	2.088	8.17E-03	1.355	4.42E-02	1.102	7.90E-02
Hair and Skin Development and Function	2.058	8.74E-03	1.604	2.49E-02	1.830	1.48E-02
Hypersensitivity Response	1.971	1.07E-02	n.s	n.s	n.s	n.s
Renal and Urological Disease	1.928	1.18E-02	1.413	3.86E-02	2.261	5.48E-03
Cellular Assembly and Organization	n.s	n.s	1.524	2.99E-02	1.102	7.90E-02
DNA Replication, Recombination, and Repair	n.s	n.s	1.524	2.99E-02	1.102	7.90E-02
Gene Expression	n.s	n.s	1.190	6.46E-02	3.171	6.74E-04
Infection Mechanism	n.s	n.s	1.762	1.73E-02	1.288	5.15E-02
Organ Morphology	n.s	n.s	1.219	6.04E-02	2.354	4.43E-03
Renal and Urological System Development and Function	n.s	n.s	1.323	4.75E-02	1.288	5.15E-02
Metabolic Disease	n.s	n.s	1.520	3.02E-02	n.s	n.s
Reproductive System Development and Function	n.s	n.s	1.394	4.04E-02	n.s	n.s
Drug Metabolism	n.s	n.s	1.355	4.42E-02	n.s	n.s
Organ Development	n.s	n.s	n.s	n.s	1.688	2.05E-02
Embryonic Development	n.s	n.s	n.s	n.s	1.445	3.59E-02
Amino Acid Metabolism	n.s	n.s	n.s	n.s	1.288	5.15E-02
