# Supplementary material for: Systems-wide RNAi analysis of CASP8AP2/FLASH shows transcriptional deregulation of the replication-dependent histone genes and extensive effects on the transcriptome of colorectal cancer cells
Source: Mol Cancer. 2012 Jan 4;11:1. doi: 10.1186/1476-4598-11-1 (PMC3281783; doi:10.1186/1476-4598-11-1)
Supplement: Additional file 11 — Table S7. Changes in gene expression over time following silencing of CASP8AP2/FLASH. Data is shown for the 2820 probes that measured a significant change in expression in SW480 cells transfected with each of the three CASP8AP2/FLASH siRNAs at 72 hours and the data for the same probes at 48, 24 and 10 hours post siRNA transfection. The median fold change for each probe is shown as well as the data for each of the three siRNAs; data is ranked on the median value at 72 hours. Fold changes ≥ Log2 -0.6 and q < 0.05 matched to any probe showing a decreased level of expression are marked in green and fold changes of ≥0.6 and q < 0.05 matched to any probe showing an increased level of expression are marked in red. The probe corresponding to CASP8AP2/FLASH is marked is highlighted in yellow. [file 1476-4598-11-1-S11.PDF]

Feature	Gene symbol	Probe	Transcript	72 hour								48 hours								24 hours								10 hours							
				siCASP8AP2								siCASP8AP2								siCASP8AP2								siCASP8AP2							
				Median	Median	1	1	3	3	6	6	Median	Median	1	1	3	3	6	6	Median	Median	1	1	3	3	6	6	Median	Median	1	1	3	3	6	6
				M	α	M	α	M	α	M	α	M	α	M	α	M	α	M	α	M	α	M	α	M	α	M	α	M	α	M	α	M	α	M	α
42448	HIST1H1B	A_23_P250385	NM_005322	-2.6	4.67E-05	-2.6	4.67E-05	-2.3	2.32E-03	-2.8	6.25E-06	-2.7	3.62E-03	-2.7	3.62E-03	-2.0	1.44E-01	-2.9	9.01E-05	-1.1	1.0E+00	-0.5	1.0E+00	-1.1	7.1E-01	-1.1	1.0E+00	-0.1	9.8E-01	-0.1	1.0E+00	0.0	9.8E-01	-0.3	8.5E-01
39178	TBC1D5	A_24_P289029	NM_014744	-2.2	6.92E-04	-2.2	6.92E-04	-2.2	1.34E-03	-1.8	3.64E-05	-1.5	2.15E-02	-1.6	4.29E-03	-1.4	2.36E-02	-1.5	2.15E-02	-0.3	1.0E+00	-0.3	1.0E+00	-0.3	7.4E-01	-0.1	1.0E+00	-0.2	9.2E-01	-0.3	1.0E+00	-0.2	7.6E-01	-0.1	9.2E-01
7978	NOTUM	A_23_P101007	NM_178493	-2.1	1.82E-04	-2.0	7.01E-04	-2.1	1.82E-04	-2.3	1.94E-07	-1.8	3.18E-03	-1.8	3.18E-03	-1.4	4.38E-03	-1.8	1.65E-03	-0.1	1.0E+00	-0.1	1.0E+00	-0.1	8.9E-01	-0.2	1.0E+00	0.5	6.1E-01	0.5	1.0E+00	0.4	5.8E-01	0.6	6.1E-01
26102	HIST1H4L	A_23_P70480	NM_003546	-2.1	2.52E-05	-1.9	2.52E-05	-2.2	3.30E-04	-2.1	3.01E-06	-1.9	7.02E-03	-2.4	7.02E-03	-1.7	1.42E-02	-1.9	7.51E-04	-0.3	1.0E+00	-0.3	1.0E+00	-0.3	7.2E-01	-0.7	1.0E+00	-0.1	1.0E+00	-0.2	1.0E+00	-0.1	9.4E-01	0.0	1.0E+00
5363	HIST1H4B	A_24_P166407	NM_003544	-2.0	3.65E-04	-1.8	3.65E-04	-2.0	3.45E-03	-2.2	4.53E-05	-1.7	6.11E-02	-2.1	6.11E-02	-1.7	8.55E-02	-1.7	1.94E-02	-0.3	1.0E+00	-0.3	1.0E+00	-0.3	7.1E-01	-0.7	1.0E+00	-0.2	1.0E+00	-0.2	1.0E+00	-0.2	8.9E-01	0.0	1.0E+00
2702	OSGEPL1	A_24_P302574	NM_022353	-2.0	8.72E-05	-2.0	8.72E-05	-2.2	1.27E-03	-1.9	1.66E-05	-1.3	3.27E-03	-1.1	3.41E-03	-1.3	3.27E-03	-1.6	1.15E-03	-0.3	1.0E+00	-0.2	1.0E+00	-0.3	7.1E-01	-0.3	1.0E+00	-0.2	7.0E-01	-0.2	1.0E+00	-0.1	7.0E-01	-0.3	4.8E-01
19569	RGMA	A_23_P372308	NM_020211	-2.0	1.26E-03	-1.4	1.26E-03	-2.4	1.84E-03	-2.0	1.33E-04	-0.9	7.60E-03	-0.9	2.09E-02	-0.9	7.60E-03	-1.4	1.15E-03	-0.2	1.0E+00	-0.3	1.0E+00	-0.2	7.5E-01	-0.2	1.0E+00	0.0	9.9E-01	-0.1	1.0E+00	0.1	7.9E-01	0.0	9.9E-01
15675	ATPBD4	A_24_P56557	NM_080650	-1.9	5.99E-04	-1.6	5.99E-04	-1.9	2.97E-03	-1.9	2.71E-05	-1.1	1.12E-02	-0.5	1.46E-01	-1.1	1.12E-02	-1.4	1.05E-03	-0.1	1.0E+00	-0.1	1.0E+00	-0.1	7.5E-01	-0.1	1.0E+00	-0.1	9.5E-01	0.0	1.0E+00	-0.1	8.8E-01	-0.1	9.5E-01
13219	HIST2H2AC	A_23_P301247	NM_003517	-1.9	5.29E-04	-1.9	5.29E-04	-1.3	5.17E-03	-2.2	6.60E-06	-1.8	1.22E-02	-1.8	1.22E-02	-0.9	6.49E-01	-2.3	5.25E-05	-0.9	1.0E+00	-0.6	1.0E+00	-0.9	7.1E-01	-0.9	1.0E+00	-0.2	9.1E-01	-0.3	1.0E+00	-0.2	8.7E-01	-0.2	9.1E-01
27115	BTBD3	A_24_P134356	NM_014962	-1.9	2.61E-04	-1.9	4.91E-04	-2.1	2.61E-04	-1.4	8.93E-06	-1.7	1.92E-03	-1.9	1.59E-03	-1.7	9.53E-02	-1.5	1.92E-03	-0.4	4.8E-01	-0.6	5.1E-02	-0.4	4.8E-01	-0.3	1.0E+00	0.0	9.9E-01	-0.3	1.0E+00	0.0	9.7E-01	0.0	9.9E-01
34877	HIST1H4C	A_23_P214487	NM_003542	-1.9	2.71E-05	-1.8	2.71E-05	-2.1	5.25E-04	-1.9	1.33E-05	-1.8	6.11E-03	-1.9	6.11E-03	-1.4	9.69E-03	-1.8	1.57E-03	-0.3	1.0E+00	-0.1	1.0E+00	-0.3	7.1E-01	-0.5	1.0E+00	-0.2	9.6E-01	-0.2	1.0E+00	-0.2	7.8E-01	-0.1	9.6E-01
19299	ALDH1A3	A_23_P205959	NM_000693	-1.8	4.00E-04	-1.6	4.00E-04	-2.1	2.07E-03	-1.8	1.77E-04	-1.3	1.76E-02	-1.0	1.76E-02	-1.3	4.60E-02	-1.4	5.66E-04	-0.3	1.0E+00	-0.1	1.0E+00	-0.3	7.1E-01	-0.5	1.0E+00	0.0	8.7E-01	0.0	1.0E+00	0.1	8.7E-01	-0.2	6.9E-01
33176	CASP8AP2	A_23_P58898	NM_012115	-1.8	4.20E-05	-1.8	4.20E-05	-2.4	4.61E-04	-1.8	2.05E-05	-1.6	3.98E-03	-1.5	3.98E-03	-1.9	1.38E-02	-1.6	1.52E-03	-1.2	9.4E-04	-1.1	9.4E-04	-1.3	7.0E-04	-1.2	1.3E-02	-0.7	1.9E-01	-0.5	1.0E+00	-0.7	5.6E-02	-0.7	1.9E-01
42961	AK057088	A_24_P554156	AK057088	-1.8	2.73E-03	-1.8	1.94E-03	-2.3	3.85E-03	-1.3	2.73E-03	-1.2	1.12E-02	-1.0	9.90E-03	-1.6	1.71E-02	-1.2	1.12E-02	0.0	1.0E+00	0.0	1.0E+00	-0.2	7.8E-01	0.1	1.0E+00	0.0	9.8E-01	-0.1	1.0E+00	0.0	9.8E-01	0.1	9.6E-01
13522	EY892390	A_32_P171043	EY892390	-1.8	2.34E-03	-1.5	2.34E-03	-2.7	2.74E-03	-1.8	2.98E-04	-1.3	7.48E-03	-0.7	7.10E-02	-1.3	7.48E-03	-1.3	4.67E-03	-0.2	1.0E+00	-0.3	1.0E+00	-0.2	7.5E-01	0.0	1.0E+00	-0.4	5.6E-01	-0.1	1.0E+00	-0.4	3.6E-01	-0.5	5.6E-01
4616	C5orf13	A_24_P149124	NM_004772	-1.8	3.84E-03	-1.0	2.59E-02	-2.6	3.84E-03	-1.8	6.34E-04	-0.7	1.84E-01	-0.2	7.24E-01	-0.7	1.84E-01	-1.0	3.81E-03	0.1	1.0E+00	0.1	1.0E+00	0.0	9.7E-01	0.2	1.0E+00	-0.1	8.8E-01	-0.1	1.0E+00	-0.1	8.8E-01	-0.4	7.1E-01
36427	TMEM170B	A_32_P178966	NM_001100829	-1.8	5.74E-04	-1.6	5.74E-04	-2.1	1.72E-03	-1.8	3.62E-05	-0.9	1.15E-01	-0.6	2.74E-01	-0.9	1.15E-01	-0.9	2.68E-02	0.1	1.0E+00	0.0	1.0E+00	0.1	9.3E-01	0.1	1.0E+00	-0.3	5.9E-01	-0.1	1.0E+00	-0.3	5.9E-01	-0.4	5.8E-01
23439	FAM36A	A_24_P79712	NM_198076	-1.7	7.20E-04	-1.7	7.20E-04	-2.6	1.10E-03	-1.6	1.06E-04	-1.5	5.95E-03	-1.3	5.95E-03	-1.9	2.49E-03	-1.5	1.76E-02	-0.3	1.0E+00	-0.3	1.0E+00	-0.8	1.3E-01	-0.2	1.0E+00	-0.2	9.5E-01	-0.2	1.0E+00	-0.4	3.3E-01	-0.1	9.5E-01
6759	C12orf48	A_23_P87769	NM_017915	-1.7	3.25E-04	-1.5	3.25E-04	-2.5	1.03E-03	-1.7	4.09E-05	-1.3	5.34E-03	-0.8	4.18E-02	-1.3	5.34E-03	-1.3	3.95E-03	0.0	1.0E+00	-0.1	1.0E+00	0.0	9.6E-01	0.0	1.0E+00	-0.3	5.4E-01	-0.2	1.0E+00	-0.3	5.4E-01	-0.5	4.3E-01
10775	OMA1	A_23_P138139	NM_145243	-1.7	8.99E-04	-1.3	8.99E-04	-1.7	3.39E-03	-1.8	4.38E-05	-1.0	3.45E-02	-0.7	7.82E-02	-1.0	3.45E-02	-1.2	1.56E-03	0.0	1.0E+00	0.0	1.0E+00	0.0	9.2E-01	0.0	1.0E+00	-0.2	6.2E-01	0.1	1.0E+00	-0.2	4.6E-01	-0.2	6.2E-01
33517	TMEM126A	A_23_P13524	NM_032273	-1.7	1.03E-04	-1.4	1.03E-04	-1.8	1.01E-03	-1.7	8.93E-06	-1.2	5.36E-03	-0.9	2.45E-02	-1.2	5.36E-03	-1.3	1.23E-03	-0.1	1.0E+00	-0.2	1.0E+00	-0.1	8.8E-01	-0.1	1.0E+00	0.0	9.5E-01	0.0	1.0E+00	-0.1	8.9E-01	0.1	9.5E-01
27165	FHIT	A_23_P125164	NM_002012	-1.7	3.17E-03	-1.3	1.28E-02	-2.0	3.17E-03	-1.7	1.70E-04	-0.4	5.23E-01	-0.2	8.45E-01	-0.4	5.23E-01	-0.6	4.40E-01	-0.3	1.0E+00	-0.3	1.0E+00	-0.3	7.1E-01	0.0	1.0E+00	-0.1	9.7E-01	-0.2	1.0E+00	0.0	9.7E-01	-0.1	9.3E-01
21609	CR620487	A_24_P415260	CR620487	-1.7	7.74E-03	-1.7	7.74E-03	-2.5	3.04E-03	-0.8	2.83E-02	-1.3	3.07E-01	-1.3	3.20E-02	-1.6	3.07E-01	-0.6	4.92E-01	0.1	1.0E+00	-0.7	1.0E+00	0.1	9.2E-01	0.4	1.0E+00	-0.2	9.6E-01	-0.4	1.0E+00	-0.2	8.5E-01	-0.1	9.6E-01
43572	CDCA7	A_23_P251421	NM_031942	-1.7	1.03E-03	-1.6	1.03E-03	-2.4	2.03E-03	-1.7	1.68E-04	-1.5	2.11E-02	-0.8	6.35E-02	-1.5	2.11E-02	-1.7	2.27E-03	0.1	1.0E+00	-0.1	1.0E+00	0.1	8.9E-01	0.1	1.0E+00	0.0	9.1E-01	0.0	1.0E+00	0.1	8.5E-01	-0.2	9.1E-01
10862	PLA2G12A	A_24_P706752	NM_030821	-1.7	5.02E-04	-1.7	5.02E-04	-2.1	1.24E-03	-1.4	1.39E-04	-1.2	1.97E-02	-1.0	3.11E-02	-1.5	1.35E-02	-1.2	1.97E-02	-0.1	1.0E+00	-0.2	1.0E+00	-0.1	7.9E-01	-0.1	1.0E+00	0.0	9.9E-01	-0.1	1.0E+00	0.1	6.3E-01	0.0	9.9E-01
28040	C10orf4	A_24_P189464	NM_145246	-1.7	2.92E-03	-1.4	2.99E-03	-1.7	2.92E-03	-1.8	1.30E-04	-0.7	3.85E-02	-0.7	5.29E-02	-0.7	3.85E-02	-1.4	1.09E-03	-0.2	1.0E+00	-0.2	1.0E+00	-0.1	7.6E-01	-0.2	1.0E+00	-0.2	9.5E-01	-0.2	1.0E+00	0.0	9.5E-01	-0.2	8.5E-01
2003	DNAJC19	A_23_P121396	NM_145261	-1.7	9.95E-04	-1.7	7.07E-03	-1.9	9.95E-04	-1.3	1.86E-04	-1.0	1.77E-01	-1.2	6.53E-03	-0.8	1.77E-01	-1.0	1.93E-01	0.0	1.0E+00	-0.3	1.0E+00	0.0	9.6E-01	0.1	1.0E+00	0.0	9.8E-01	0.1	1.0E+00	0.0	9.8E-01	-0.1	8.1E-01
27118	CGNL1	A_23_P163306	NM_032866	-1.7	7.69E-03	-1.5	7.69E-03	-2.0	2.21E-02	-1.7	2.51E-03	-0.8	1.47E-02	-0.7	3.87E-02	-0.8	1.47E-02	-0.8	9.77E-03	-0.2	1.0E+00	-0.2	1.0E+00	-0.2	7.5E-01	0.1	1.0E+00	-0.3	8.2E-01	-0.3	1.0E+00	-0.3	5.3E-01	-0.2	8.2E-01
10057	BCL11A	A_24_P402588	NM_018014	-1.7	7.60E-04	-1.2	1.19E-03	-2.2	7.60E-04	-1.7	1.67E-05	-0.7	7.76E-02	-0.7	1.27E-01	-0.7	7.76E-02	-1.2	4.55E-03	-0.1	1.0E+00	-0.2	1.0E+00	-0.1	7.9E-01	0.0	1.0E+00	-0.3	3.4E-01	-0.2	1.0E+00	-0.3	2.3E-01	-0.3	3.4E-01
2068	GTDC1	A_23_P153945	NM_001006636	-1.7	1.74E-04	-1.4	1.74E-04	-2.0	7.06E-04	-1.7	1.35E-05	-0.8	3.26E-02	-0.6	1.01E-01	-0.8	3.26E-02	-1.2	8.50E-03	0.1	1.0E+00	0.0	1.0E+00	0.1	8.2E-01	0.2	1.0E+00	0.0	9.9E-01	0.1	1.0E+00	0.0	9.9E-01	-0.3	6.4E-01
6280	MANEA	A_23_P255663	NM_024641	-1.7	2.01E-04	-1.5	2.01E-04	-2.4	8.27E-04	-1.7	6.77E-05	-1.1	3.18E-02	-0.6	3.18E-02	-1.4	3.34E-02	-1.1	3.24E-03	-0.1	1.0E+00	-0.1	1.0E+00	-0.2	7.1E-01	-0.1	1.0E+00	0.0	9.9E-01	0.1	1.0E+00	0.0	9.0E-01	0.0	9.9E-01
28091	RNF141	A_24_P372625	NM_016422	-1.6	1.94E-02	-1.6	7.33E-03	-1.9	2.63E-02	-1.3	1.94E-02	-1.1	6.54E-02	-1.1	6.54E-02	-0.7	1.20E-01	-1.4	7.82E-03	-0.1	1.0E+00	-0.1	1.0E+00	-0.1	9.0E-01	-0.2	1.0E+00	0.0	9.6E-01	0.0	1.0E+00	0.1	9.3E-01	-0.1	9.6E-01
26787	SSTR1	A_24_P244706	NM_001049	-1.6	1.75E-02	-1.3	1.75E-02	-1.9	3.68E-02	-1.6	3.90E-03	-0.9	1.14E-01	-0.6	2.42E-01	-0.9	1.14E-01	-1.3	4.68E-03	-0.2	1.0E+00	-0.2	1.0E+00	-0.3	7.2E-01	-0.2	1.0E+00	-0.4	6.5E-01	-0.2	1.0E+00	-0.4	4.0E-01	-0.5	6.5E-01
20244	TMEM48	A_24_P222184	AK091439	-1.6	2.13E-03	-1.0	2.13E-03	-1.8	2.22E-03	-1.6	5.15E-05	-1.0	4.11E-02	-0.6	5.34E-02	-1.1	4.11E-02	-1.0	4.47E-03	-0.2	1.0E+00	0.0	1.0E+00	-0.2	7.4E-01	-0.2	1.0E+00	0.2	9.4E-01	0.2	1.0E+00	0.2	6.6E-01	0.1	9.4E-01
31976	SRI	A_23_P358928	NM_003130	-1.6	5.74E-04	-1.2	2.40E-03	-1.8	5.74E-04	-1.6	5.68E-06	-0.9	4.81E-02	-0.5	1.86E-01	-0.9	4.81E-02	-1.0	1.75E-03	0.0	1.0E+00	0.0	1.0E+00	0.0	9.1E-01	0.0	1.0E+00	0.0	9.8E-01	0.0	1.0E+00	0.0	9.7E-01	0.0	9.8E-01
33114	ARHGAP19	A_23_P1387	NM_032900	-1.6	1.32E-02	-1.3	1.32E-02	-1.6	3.00E-02	-1.9	5.73E-04	-1.0	1.14E-01	-0.9	1.28E-01	-1.4	1.14E-01	-1.0	2.42E-02	-0.1	1.0E+00	-0.1	1.0E+00	-0.4	7.1E-01	-0.1	1.0E+00	-0.1	9.6E-01	-0.1	1.0E+00	-0.1	8.7E-01	0.1	9.6E-01
9879	COQ3	A_23_P111228	NM_017421	-1.6	1.74E-04	-1.4	1.74E-04	-1.9	1.10E-03	-1.6	1.56E-05	-1.1	3.79E-02	-0.4	9.40E-02	-1.1	3.79E-02	-1.1	8.04E-03	-0.1	1.0E+00	-0.1	1.0E+00	-0.1	7.4E-01	0.1	1.0E+00	0.1	9.0E-01	0.1	1.0E+00	0.1	9.0E-01	-0.2	9.0E-01
5652	GBAS	A_23_P82674	NM_001483	-1.6	5.29E-04	-1.3	5.29E-04	-2.5	7.04E-04	-1.6	3.90E-05	-1.2	4.21E-02	-0.6	1.02E-01	-2.0	4.21E-02	-1.2	5.99E-03	-0.1	1.0E+00	-0.1	1.0E+00	-0.2	7.5E-01	0.1	1.0E+00	-0.3	6.3E-01	-0.3	1.0E+00	-0.1	6.3E-01	-0.3	3.1E-01
25108	E2F5	A_23_P31721	NM_001951	-1.6	3.81E-03	-1.3	5.54E-03	-2.1	3.81E-03	-1.6	2.30E-04	-1.3	1.54E-01	-0.4	4.65E-01	-1.4	1.54E-01	-1.3	4.42E-03	-0.4	1.0E+00	-0.2	1.0E+00	-0.4	7.1E-01	-0.4	1.0E+00	-0.2	9.7E-01	-0.2	1.0E+00	0.0	9.7E-01	-0.3	5.9E-01
18799	HAS2	A_23_P10206	NM_005328	-1.6	2.11E-03	-1.0	3.28E-03	-1.9	2.11E-03	-1.6	9.43E-05	-1.0	2.92E-02	-0.4	2.34E-01	-1.2	2.92E-02	-1.0	1.57E-03	-0.2	1.0E+00	-0.2	1.0E+00	-0.2	7.1E-01	0.0	1.0E+00	-0.3	3.8E-01	-0.2	1.0E+00	-0.3	2.3E-01	-0.3	3.8E-01
13263	ZC3HAV1L	A_24_P923142	AK098156	-1.6	3.85E-03	-1.6	3.85E-03	-2.0	3.22E-03	-0.9	5.49E-03	-1.2	7.89E-02	-1.2	1.18E-02	-1.2	9.46E-02	-1.2	7.89E-02	0.1	1.0E+00	-0.1	1.0E+00	0.1	8.8E-01	0.3	1.0E+00	-0.1	6.8E-01	0.0	1.0E+00	-0.1	6.8E-01	-0.3	6.5E-01
43256	IGFBP3	A_23_P215634	NM_001013398	-1.6	2.99E-03	-1.6	2.99E-03	-1.7	9.95E-03	-1.2	2.15E-03	-1.0	5.79E-02	-1.0	1.36E-01	-0.9	5.79E-02	-1.4	2.38E-03	-0.2	1.0E+00	-0.1	1.0E+00	-0.3	7.1E-01	-0.2	1.0E+00	-0.2	9.7E-01	-0.2	1.0E+00	-0.2	8.8E-01	-0.1	9.7E-01
15224	TGM2	A_32_P86763	NM_004613	-1.6	1.29E-03	-1.6	2.06E-02	-2.8	1.29E-03	-1.3	7.84E-04	-1.0	1.31E-01	-1.0	1.31E-01	-1.6	1.56E-01	-1.0	5.09E-02	0.1	1.0E+00	0.5	1.0E+00	0.1	8.6E-01	0.1	1.0E+00	0.2	9.4E-01	0.0	1.0E+00	0.2	8.4E-01	0.2	9.4E-01
24355	FARSB	A_23_P165355	NM_005687	-1.6	1.49E-03	-1.3	1.49E-03	-2.1	2.55E-03	-1.6	2.05E-04	-1.2	5.09E-02	-0.6	3.23E-01	-1.4	5.09E-02	-1.2	1.29E-02	-0.2	1.0E+00	-0.2	1.0E+00	-0.3	7.1E-01	0.0	1.0E+00	0.1	9.7E-01	-0.1	1.0E+00	0.1	8.4E-01	0.1	9.7E-01
15543	FARSB	A_23_P165346	NM_005687	-1.6	4.65E-04	-1.2	4.65E-04	-1.7	9.96E-04	-1.6	6.68E-06	-0.9	4.54E-03	-0.7	1.75E-02	-0.9	4.54E-03	-1.1	1.27E-03	0.0	1.0E+00	0.0	1.0E+00	-0.1	7.8E-01	0.0	1.0E+00	0.0	8.7E-01	0.1	1.0E+00	0.0	8.7E-01	-0.1	8.1E-01
7781	AHRR	A_23_P358709	NM_020731	-1.6	9.41E-03	-1.3	9.41E-03	-1.7	1.21E-02	-1.6	5.29E-04	-1.0	2.17E-02	-0.9	2.17E-02	-1.0	3.12E-02	-1.4	1.02E-03	-0.2	1.0E+00	-0.3	1.0E+00	-0.2	7.3E-01	-0.2	1.0E+00	-0.2	7.9E-01	-0.1	1.0E+00	-0.2	6.9E-01	-0.2	7.9E-01
964	GJB1	A_24_P404840	NM_000166	-1.6	1.31E-03	-1.6	6.39E-03	-2.1	1.31E-03	-1.0	6.10E-04	-1.1	7.53E-02	-1.1	1.57E-02	-1.4	7.53E-02	-0.8	2.24E-01	-0.1	1.0E+00	-0.1	1.0E+00	-0.1	8.5E-01	0.1	1.0E+00	0.1	9.3E-01	-0.1	1.0E+00	0.1	8.2E-01	0.1	9.3E-01
21434	SRI	A_23_P59718	NM_003130	-1.6	3.86E-03	-1.6	3.61E-02	-2.4	3.86E-03	-1.5	1.23E-03	-0.8	1.13E-01	-0.7	2.28E-01	-0.8	1.13E-01	-0.9	5.86E-02	0.2	1.0E+00	0.0	1.0E+00	0.2	8.1E-01	0.2	1.0E+00	0.1	9.5E-01	0.1	1.0E+00	0.1	9.2E-01	-0.2	9.5E-01
26949	TMEM5	A_23_P2446	NM_014254	-1.6	2.65E-03	-1.6	2.65E-03	-0.8	2.65E-02	-1.6	2.40E-05	-1.3	2.32E-03	-1.3	2.32E-03	-0.2	5.24E-01	-1.4	1.56E-03	-0.3	9.0E-01	-0.5	1.7E-01	-0.1	9.0E-01	-0.3	1.0E+00	-0.1	9.2E-01	-0.1	1.0E+00	0.1	8.7E-01	-0.2	9.2E-01
18280	PIGF	A_23_P131653	NM_002643	-1.6	7.10E-05	-1.4	7.10E-05	-2.0	1.84E-04	-1.6	1.00E-06	-1.0	7.83E-03	-0.8	7.83E-03	-1.1	1.48E-01	-1.0	2.00E-03	0.1	1.0E+00	0.0	1.0E+00	0.1	8.1E-01	0.1	1.0E+00	0.1	9.3E-01	0.1	1.0E+00	0.1	7.5E-01	-0.1	9.3E-01
43841	C14orf126	A_23_P37144	NM_080664	-1.5	9.12E-04	-1.4	9.12E-04	-1.6	4.82E-03	-1.5	1.03E-04	-1.2	1.26E-02	-0.9	1.26E-02	-1.2	5.04E-02	-1.2	9.61E-04	0.0	1.0E+00	-0.2	1.0E+00	0.0	9.9E-01	0.0	1.0E+00	-0.1	8.7E-01	0.0	1.0E+00	-0.1	8.7E-01	-0.3	5.5E-01
38538	C1D	A_23_P67992	NM_006333	-1.5	6.30E-04	-1.5	5.70E-04	-1.5	3.18E-03	-1.1	6.30E-04	-0.9	3.78E-02	-0.9	3.26E-02	-1.1	3.78E-02	-0.9	8.76E-02	-0.2	1.0E+00	-0.2	1.0E+00	-0.1	9.1E-01	-0.2	1.0E+00	-0.1	7.7E-01	-0.1	1.0E+00	-0.1	7.7E-01	-0.2	7.4E-01
10060	DPP6	A_23_P170888	NM_001039350	-1.5	7.69E-04	-1.4	7.69E-04	-1.5	6.59E-03	-1.5	2.09E-04	-0.8	2.38E-01	-0.7	4.80E-01	-0.8	2.38E-01	-1.0	1.16E-01	-0.1	1.0E+00	-0.1	1.0E+00	-0.3	7.4E-01	0.0	1.0E+00	-0.3	6.4E-01	-0.2	1.0E+00	-0.4	3.0E-01	-0.3	6.4E-01
44845	ETFDH	A_23_P61447	NM_004453	-1.5	2.69E-04	-1.2	2.69E-04	-1.5	1.25E-03	-1.7	7.60E-06	-0.9	3.22E-02	-0.9	3.22E-02	-0.5	9.66E-02	-1.2	1.35E-03	-0.2	1.0E+00	-0.3	1.0E+00	-0.1	8.0E-01	-0.2	1.0E+00	-0.2	5.5E-01	-0.2	1.0E+00	-0.2	5.5E-01	-0.4	5.2E-01
31128	OMA1	A_23_P138137	NM_145243	-1.5	1.35E-03	-1.3	1.35E-03	-1.5	6.41E-03	-1.7	7.52E-05	-0.8	4.57E-02	-0.8	4.88E-02	-0.8	4.57E-02	-1.2	1.95E-03	0.0	1.0E+00	-0.1	1.0E+00	0.0	9.3E-01	0.0	1.0E+00	-0.2	5.1E-01	0.0	1.0E+00	-0.2	5.1E-01	-0.3	4.6E-01
37220	HIST3H2BB	A_24_P524452	DB010344	-1.5	8.37E-04	-1.3	8.37E-04	-2.1	1.56E-03	-1.5	3.74E-04	-1.4	1.63E-03	-1.3	5.24E-03	-1.5	1.63E-03	-1.4	1.05E-03	-0.4	1.0E+00	-0.3	1.0E+00	-0.4	7.1E-01	-0.5	1.0E+00	0.2	1.0E+00	0.4	1.0E+00	0.2	8.6E-01	0.0	1.0E+00
15024	COMMD8	A_23_P44257	NM_017845	-1.5	7.40E-04	-1.5	7.40E-04	-1.7	6.62E-03	-1.5	2.15E-04	-0.8	6.18E-02	-0.7	6.90E-02	-0.8	6.18E-02	-1.2	1.20E-02	0.0	1.0E+00	-0.1	1.0E+00	0.0	9.7E-01	0.0	1.0E+00	-0.1	9.7E-01	-0.1	1.0E+00	0.0	9.7E-01	-0.2	8.9E-01
35778	GCOM1	A_32_P117098	NM_001018090	-1.5	3.09E-03	-1.7	1.24E-03	-1.5	1.10E-02	-1.0	3.09E-03	-1.1	4.95E-02	-1.1	2.52E-02	-0.6	1.38E-01	-1.2	4.95E-02	0.1	1.0E+00	-0.6	1.0E+00	0.1	8.8E-01	0.2	1.0E+00	-0.3	9.1E-01	-0.4	1.0E+00	-0.1	9.1E-01	-0.3	8.4E-01
11739	TIMELESS	A_23_P53276	NM_003920	-1.5	2.15E-03	-1.7	2.15E-03	-1.5	2.90E-03	-1.4	8.77E-05	-1.3	2.38E-03	-1.3	1.92E-03	-0.8	5.49E-03	-1.7	2.38E-03	-0.2	1.0E+00	-0.1	1.0E+00	-0.2	7.3E-01	-0.2	1.0E+00	0.0	9.9E-01	-0.1	1.0E+00	0.1	8.5E-01	0.0	9.9E-01
39649	IGFBP3	A_23_P215634	NM_001013398	-1.5	4.26E-03	-1.6	4.26E-03	-1.5	5.92E-03	-1.1	4.97E-04	-1.0	7.77E-02	-1.0	1.61E-01	-0.8	7.77E-02	-1.3	1.69E-03	-0.3	1.0E+00	-0.1	1.0E+00	-0.3	7.1E-01	-0.3	1.0E+00	-0.1	9.9E-01	-0.2	1.0E+00	-0.1	9.4E-01	0.0	9.9E-01
8963	PHYHIPL	A_23_P97923	NM_032439	-1.5	2.39E-03	-1.2	1.40E-02	-2.6	2.39E-03	-1.5	5.47E-04	-0.9	7.56E-02	-0.5	4.12E-01	-1.7	6.75E-02	-0.9	7.56E-02	-0.1	1.0E+00	-0.1	1.0E+00	-0.3	7.1E-01	0.1	1.0E+00	-0.3	7.9E-01	-0.2	1.0E+00	-0.4	3.3E-01	-0.3	7.9E-01
5114	IGFBP3	A_23_P215634	NM_001013398	-1.5	4.14E-03	-1.5	4.14E-03	-1.7	1.09E-02	-1.1	2.84E-03	-1.0	1.04E-01	-1.0	1.34E-01	-0.8	1.04E-01	-1.4	2.72E-03	-0.2	1.0E+00	0.0	1.0E+00	-0.3	7.1E-01	-0.2	1.0E+00	-0.2	9.6E-01	-0.2	1.0E+00	-0.2	8.9E-01	-0.2	9.6E-01
25784	FGF3	A_23_P113204	NM_005247	-1.5	1.32E-03	-1.4	1.32E-03	-1.5	2.42E-03	-1.6	1.17E-05	-1.2	9.20E-03	-1.2	9.20E-03	-1.1	1.03E-02	-1.2	5.96E-03	-0.1	1.0E+00	-0.2	1.0E+00	-0.1	8.5E-01	-0.1	1.0E+00	0.1	8.8E-01	0.3	1.0E+00	0.1	6.9E-01	0.1	8.8E-01
12663	IGFBP3	A_23_P215634	NM_001013398	-1.5	4.02E-03	-1.5	5.55E-03	-1.5	4.02E-03	-1.1	3.25E-04	-0.8	7.69E-02	-0.8	2.18E-01	-0.8	7.69E-02	-1.3	5.55E-04	-0.3	1.0E+00	-0.1	1.0E+00	-0.3	7.1E-01	-0.3	1.0E+00	0.0	9.9E-01	-0.1	1.0E+00	0.0	9.9E-01	0.1	9.8E-01
40987	BCL11A	A_24_P411186	NM_022893	-1.5	1.25E-03	-1.3	1.25E-03	-1.5	1.54E-03	-1.5	6.53E-05	-0.8	5.62E-02	-0.8	5.62E-02	-0.5	5.52E-01	-1.2	2.30E-03	-0.2	7.1E-01	-0.5	6.5E-02	-0.2	7.1E-01	-0.2	1.0E+00	-0.1	8.6E-01	-0.1	1.0E+00	-0.1	8.1E-01	-0.1	8.6E-01
31436	IGFBP3	A_23_P215634	NM_001013398	-1.5	4.78E-03	-1.5	4.78E-03	-1.5	1.29E-02	-1.1	1.91E-03	-0.8	1.09E-01	-0.8	2.40E-01	-0.7	1.09E-01	-1.3	2.12E-03	-0.2	1.0E+00	-0.1	1.0E+00	-0.3	7.1E-01	-0.2	1.0E+00	-0.1	1.0E+00	-0.2	1.0E+00	-0.1	9.5E-01	0.0	1.0E+00
10662	ARL6IP6	A_23_P28169	NM_152522	-1.5	8.36E-04	-1.5	4.47E-04	-1.6	2.54E-03	-0.9	8.36E-04	-0.9	2.06E-01	-0.9	7.55E-02	-1.2	2.06E-01	-0.9	2.06E-01	0.1	1.0E+00	-0.1	1.0E+00	0.1	9.2E-01	0.1	1.0E+00	-0.2	8.8E-01	-0.2	1.0E+00	-0.1	8.8E-01	-0.2	7.9E-01
31584	MANEA	A_24_P69274	NM_024641	-1.5	8.17E-04	-1.2	8.17E-04	-2.2	1.05E-03	-1.5	1.41E-04	-0.8	1.61E-01	-0.4	3.99E-01	-1.1	1.61E-01	-0.8	2.92E-02	-0.1	1.0E+00	-0.1	1.0E+00	-0.2	7.4E-01	0.0	1.0E+00	-0.1	8.3E-01	0.0	1.0E+00	-0.1	8.3E-01	-0.1	7.3E-01
24461	IGFBP3	A_23_P215634	NM_001013398	-1.5	5.68E-03	-1.5	5.68E-03	-1.5	9.05E-03	-1.1	1.23E-03	-0.8	1.42E-01	-0.8	2.65E-01	-0.7	1.42E-01	-1.2	1.97E-03	-0.3	1.0E+00	0.0	1.0E+00	-0.3	7.1E-01	-0.3	1.0E+00	0.0	9.8E-01	-0.1	1.0E+00	0.0	9.8E-01	0.1	9.8E-01
40160	TMEM18	A_24_P385585	NM_152834	-1.5	7.58E-04	-1.5	7.58E-04	-1.5	1.24E-03	-1.2	3.12E-05	-0.9	1.65E-01	-0.7	2.09E-01	-1.0	1.65E-01	-0.9	9.21E-02	-0.2	1.0E+00	-0.2	1.0E+00	-0.3	7.1E-01	0.0	1.0E+00	-0.1	9.7E-01	-0.1	1.0E+00	-0.3	3.9E-01	0.0	9.7E-01
42200	IFT88	A_23_P48339	NM_175605	-1.5	4.81E-04	-1.5	4.81E-04	-1.3	1.10E-02	-1.7	1.35E-04	-0.9	2.50E-02	-0.9	2.50E-02	-0.5	4.67E-01	-1.4	9.37E-04	-0.2	1.0E+00	-0.1	1.0E+00	-0.3	7.1E-01	-0.2	1.0E+00	-0.3	5.3E-01	-0.3	1.0E+00	-0.3	3.7E-01	-0.4	5.3E-01
8390	INHBB	A_23_P153958	NM_002193	-1.5	6.63E-04	-1.4	6.63E-04	-1.5	7.87E-03	-1.5	2.22E-04	-0.8	1.02E-01	-0.8	1.03E-01	-0.7	1.02E-01	-1.1	1.35E-02	-0.3	1.0E+00	-0.3	1.0E+00	-0.3	7.2E-01	-0.2	1.0E+00	0.0	9.8E-01	0.0	1.0E+00	0.0	9.8E-01	0.1	9.4E-01
12464	COX18	A_23_P321485	NM_173827	-1.5	9.59E-04	-1.3	9.59E-04	-1.7	3.90E-03	-1.5	1.14E-04	-0.8	1.39E-01	-0.3	5.94E-01	-0.8	1.39E-01	-1.0	1.43E-02	-0.2	1.0E+00	-0.2	1.0E+00	-0.2	7.1E-01	0.0	1.0E+00	-0.1	8.1E-01	0.0	1.0E+00	-0.1	6.7E-01	-0.2	8.1E-01
1586	IGFBP3	A_23_P215634	NM_001013398	-1.5	4.48E-03	-1.5	4.48E-03	-1.5	7.83E-03	-1.1	9.56E-04	-0.9	8.97E-02	-0.9	2.17E-01	-0.7	8.97E-02	-1.3	9.33E-04	-0.2	1.0E+00	0.0	1.0E+00	-0.3	7.1E-01	-0.2	1.0E+00	-0.1	9.9E-01	-0.1	1.0E+00	-0.1	9.5E-01	0.1	9.9E-01
4201	HNRNPA3	A_23_P86660	NM_194247	-1.5	9.71E-04	-1.5	9.08E-03	-1.5	9.71E-04	-1.1	2.77E-04	-1.1	1.65E-02	-1.5	8.69E-04	-1.1	1.65E-02	-1.1	1.08E-01	0.0	9.8E-01	-0.5	6.2E-02	0.0	9.8E-01	0.2	1.0E+00	-0.1	7.8E-01	-0.1	1.0E+00	-0.1	7.8E-01	-0.3	3.5E-01
17870	KRT5	A_23_P218047	NM_000424	-1.5	9.73E-03	-1.0	1.66E-02	-1.5	9.73E-03	-1.5	2.22E-04	-0.7	2.95E-01	-0.3	3.43E-01	-0.7	2.95E-01	-0.8	5.65E-02	0.0	1.0E+00	0.0	1.0E+00	-0.1	8.4E-01	0.0	1.0E+00	0.1	9.8E-01	-0.1	1.0E+00	0.1	9.6E-01	0.1	9.8E-01
23936	DNAJC19	A_23_P431890	NM_145261	-1.5	9.30E-03	-2.1	9.30E-03	-1.5	1.82E-02	-1.1	3.48E-03	-1.6	3.70E-02	-1.9	2.84E-03	-0.7	3.70E-02	-1.6	4.38E-02	-0.1	7.9E-01	-0.4	4.7E-01	-0.1	7.9E-01	-0.1	1.0E+00	0.0	9.8E-01	-0.1	1.0E+00	0.1	8.0E-01	0.0	9.8E-01
37483	AK094603	A_24_P912136	AK094603	-1.5	6.84E-04	-1.5	6.84E-04	-2.2	1.29E-03	-1.4	1.72E-04	-1.0	6.41E-02	-0.6	1.08E-01	-1.2	6.41E-02	-1.0	1.65E-02	-0.1	1.0E+00	-0.2	1.0E+00	-0.1	9.3E-01	0.1	1.0E+00	-0.2	6.7E-01	-0.2	1.0E+00	-0.2	6.7E-01	-0.4	6.2E-01
39545	PPME1	A_24_P4701	NM_016147	-1.5	1.75E-03	-1.5	1.19E-02	-2.4	3.06E-04	-1.0	1.75E-03	-0.7	1.74E-01	-0.8	1.12E-01	-0.7	1.74E-01	-0.5	3.01E-01	-0.1	1.0E+00	0.1	1.0E+00	-0.1	8.9E-01	-0.1	1.0E+00	0.0	9.9E-01	-0.1	1.0E+00	0.0	9.7E-01	0.0	9.9E-01
30043	KLHL9	A_23_P83159	NM_018847	-1.5	1.56E-03	-1.3	1.56E-03	-1.5	1.09E-02	-1.5	3.15E-04	-0.6	2.14E-01	-0.4	2.37E-01	-0.6	2.14E-01	-1.2	1.24E-03	-0.1	1.0E+00	-0.1	1.0E+00	0.1	8.6E-01	-0.2	1.0E+00	-0.1	7.7E-01	-0.1	1.0E+00	0.1	7.7E-01	-0.3	6.3E-01
35637	MPP5	A_23_P99536	NM_022474	-1.4	2.10E-03	-1.4	1.44E-03	-1.5	4.02E-03	-0.9	2.10E-03	-0.9	8.93E-03	-1.3	5.76E-03	-0.6	2.06E-01	-0.9	8.93E-03	-0.1	1.0E+00	0.0	1.0E+00	-0.3	7.1E-01	-0.1	1.0E+00	0.0	1.0E+00	-0.1	1.0E+00	0.0	1.0E+00	0.2	8.6E-01
21779	KLRG1	A_23_P64898	NM_005810	-1.4	1.57E-03	-1.4	1.57E-03	-1.5	6.23E-03	-1.1	5.19E-04	-1.2	1.06E-02	-1.4	6.88E-03	-0.3	3.86E-01	-1.2	1.06E-02	-0.2	7.1E-01	-0.5	6.5E-02	-0.2	7.1E-01	-0.1	1.0E+00	-0.1	9.8E-01	-0.1	1.0E+00	-0.1	7.5E-01	0.0	9.8E-01
36843	C11orf74	A_24_P252846	NM_138787	-1.4	1.60E-02	-0.9	1.91E-02	-1.6	1.60E-02	-1.4	2.98E-03	-0.6	5.55E-02	-0.3	3.71E-01	-0.6	1.71E-02	-0.6	5.55E-02	-0.1	1.0E+00	-0.1	1.0E+00	-0.1	8.5E-01	-0.2	1.0E+00	-0.2	7.7E-01	0.0	1.0E+00	-0.2	7.7E-01	-0.3	7.7E-01
32343	APIP	A_23_P2066	NM_015957	-1.4	1.44E-02	-1.4	2.83E-02	-1.8	1.44E-02	-1.2	8.57E-03	-1.0	6.69E-02	-0.9	6.69E-02	-1.0	2.16E-01	-1.0	6.00E-03	-0.1	1.0E+00	-0.1	1.0E+00	0.0	9.8E-01	-0.1	1.0E+00	0.0	9.4E-01	0.0	1.0E+00	0.1	9.3E-01	-0.1	9.4E-01
39591	AK022936	A_24_P506726	AK022936	-1.4	1.74E-03	-1.4	1.31E-03	-1.8	6.70E-03	-1.1	1.74E-03	-1.0	1.41E-01	-0.9	1.41E-01	-1.1	2.70E-01	-1.0	1.09E-01	0.1	1.0E+00	-0.2	1.0E+00	0.1	9.4E-01	0.1	1.0E+00	-0.1	9.3E-01	-0.2	1.0E+00	0.1	9.3E-01	-0.1	9.3E-01
29944	IGFBP3	A_23_P215634	NM_001013398	-1.4	6.54E-03	-1.4	6.54E-03	-1.4	1.10E-02	-1.1	1.19E-03	-0.9	1.01E-01	-0.9	2.15E-01	-0.7	1.01E-01	-1.2	1.65E-03	-0.3	1.0E+00	-0.1	1.0E+00	-0.3	7.1E-01	-0.3	1.0E+00	0.0	9.8E-01	-0.1	1.0E+00	0.0	9.8E-01	0.1	9.8E-01
15003	IGFBP3	A_23_P215634	NM_001013398	-1.4	7.49E-03	-1.4	7.49E-03	-1.4	1.19E-02	-1.0	2.78E-03	-0.8	1.15E-01	-0.8	2.59E-01	-0.7	1.15E-01	-1.2	1.20E-03	-0.3	1.0E+00	-0.1	1.0E+00	-0.3	7.1E-01	-0.3	1.0E+00	0.0	1.0E+00	-0.1	1.0E+00	0.0	1.0E+00	0.1	9.9E-01
21831	ANKH	A_24_P303145	NM_054027	-1.4	5.97E-04	-1.4	5.45E-04	-1.6	4.76E-03	-1.2	5.97E-04	-0.8	6.43E-02	-0.5	1.22E-01	-0.8	2.93E-02	-0.8	6.43E-02	-0.1	1.0E+00	-0.1	1.0E+00	-0.3	7.1E-01	0.1	1.0E+00	0.0	9.4E-01	0.0	1.0E+00	0.0	9.4E-01	-0.1	8.6E-01
7221	THC2526647	A_32_P70491	THC2526647	-1.4	6.84E-04	-1.4	6.84E-04	-1.6	1.90E-03	-1.1	1.41E-04	-1.0	4.09E-02	-1.0	4.09E-02	-1.1	8.27E-03	-0.8	7.93E-02	0.1	1.0E+00	0.2	1.0E+00	-0.1	8.7E-01	0.1	1.0E+00	0.3	4.7E-01	0.3	1.0E+00	0.3	4.7E-01	0.7	2.1E-01
30850	VPS29	A_24_P175989	NM_016226	-1.4	4.33E-04	-1.4	8.17E-04	-2.3	4.33E-04	-1.3	4.23E-05	-1.2	3.03E-02	-0.9	3.03E-02	-1.4	3.92E-02	-1.2	2.48E-03	-0.1	1.0E+00	0.0	1.0E+00	-0.1	8.6E-01	-0.2	1.0E+00	0.1	9.7E-01	0.1	1.0E+00	0.2	7.9E-01	0.0	9.7E-01
41594	TBC1D5	A_23_P355455	NM_014744	-1.4	2.92E-03	-1.4	2.92E-03	-1.7	4.34E-03	-1.2	3.72E-04	-0.8	6.49E-02	-0.8	6.49E-02	-0.7	8.33E-02	-0.9	2.69E-02	-0.2	1.0E+00	-0.3	1.0E+00	-0.2	7.1E-01	-0.1	1.0E+00	-0.3	5.7E-01	-0.2	1.0E+00	-0.5	1.4E-01	-0.3	5.7E-01
24131	FPGT	A_23_P200030	NM_003838	-1.4	2.37E-03	-1.1	2.37E-03	-1.5	7.96E-03	-1.4	2.98E-04	-0.7	1.38E-01	-0.3	5.17E-01	-0.7	1.38E-01	-0.7	2.15E-02	-0.1	1.0E+00	-0.1	1.0E+00	-0.2	7.1E-01	-0.1	1.0E+00	-0.2	4.7E-01	0.0	1.0E+00	-0.2	4.6E-01	-0.4	4.7E-01
19323	SORBS2	A_23_P121795	NM_021069	-1.4	1.44E-03	-1.3	1.44E-03	-1.6	3.62E-03	-1.4	7.44E-05	-1.1	1.91E-02	-0.8	9.27E-02	-1.2	1.91E-02	-1.1	1.32E-02	-0.3	1.0E+00	-0.3	1.0E+00	-0.3	7.1E-01	-0.1	1.0E+00	-0.1	9.2E-01	-0.1	1.0E+00	-0.1	7.8E-01	0.1	9.2E-01
14163	CDCA7	A_24_P171549	NM_031942	-1.4	5.79E-03	-1.2	5.79E-03	-1.9	7.07E-03	-1.4	2.01E-03	-1.3	1.04E-01	-0.5	2.60E-01	-1.5	1.04E-01	-1.3	4.31E-03	-0.2	1.0E+00	-0.2	1.0E+00	-0.3	7.1E-01	-0.1	1.0E+00	0.0	9.8E-01	0.0	1.0E+00	0.1	9.1E-01	0.0	9.8E-01
6755	CETN3	A_23_P7732	NM_004365	-1.4	2.56E-03	-1.4	4.98E-03	-1.7	2.56E-03	-1.3	5.86E-04	-0.8	1.51E-01	-0.5	3.07E-01	-0.8	1.51E-01	-0.8	3.29E-02	0.1	1.0E+00	0.1	1.0E+00	0.1	8.6E-01	0.0	1.0E+00	-0.1	9.0E-01	0.0	1.0E+00	-0.1	9.0E-01	-0.3	7.3E-01
38684	IGFBP3	A_23_P215634	NM_001013398	-1.4	2.85E-03	-1.5	4.47E-03	-1.4	2.85E-03	-1.1	1.33E-04	-1.0	9.06E-02	-1.0	1.94E-01	-0.8	9.06E-02	-1.3	1.11E-03	-0.3	1.0E+00	-0.1	1.0E+00	-0.4	7.1E-01	-0.3	1.0E+00	-0.1	9.6E-01	-0.1	1.0E+00	-0.1	9.6E-01	0.2	9.6E-01
38751	DDX46	A_32_P105195	NM_014829	-1.4	9.36E-04	-1.4	5.67E-04	-2.4	9.36E-04	-1.0	2.99E-03	-0.8	2.20E-01	-0.7	2.20E-01	-1.5	8.29E-02	-0.8	2.39E-01	0.1	1.0E+00	0.0	1.0E+00	0.1	9.3E-01	0.2	1.0E+00	-0.1	9.9E-01	-0.1	1.0E+00	0.0	9.9E-01	-0.2	9.3E-01
29114	TMEM48	A_24_P222192	AK091439	-1.4	2.25E-03	-1.2	5.07E-03	-2.2	2.25E-03	-1.4	5.08E-04	-0.8	9.58E-02	-0.4	6.02E-01	-0.8	9.58E-02	-1.1	1.27E-02	-0.4	1.0E+00	-0.2	1.0E+00	-0.4	3.8E-01	-0.4	1.0E+00	0.0	9.9E-01	0.0	1.0E+00	0.3	6.8E-01	0.0	9.9E-01
34848	BC013295	A_24_P170874	BC013295	-1.4	1.37E-03	-1.5	1.37E-03	-1.1	2.14E-02	-1.4	2.17E-04	-0.8	3.56E-02	-0.8	3.56E-02	-0.6	8.18E-02	-1.1	8.41E-03	-0.1	1.0E+00	0.0	1.0E+00	-0.1	8.9E-01	-0.1	1.0E+00	-0.1	9.3E-01	0.0	1.0E+00	-0.1	8.8E-01	-0.1	9.3E-01
11386	C5orf22	A_23_P7761	NM_018356	-1.4	1.31E-03	-1.4	1.31E-03	-1.6	1.73E-03	-1.1	1.55E-04	-1.2	5.29E-02	-0.9	5.29E-02	-1.2	1.95E-01	-1.2	1.93E-03	-0.3	1.0E+00	-0.4	1.0E+00	-0.1	7.7E-01	-0.3	1.0E+00	-0.1	9.1E-01	-0.1	1.0E+00	-0.1	9.1E-01	-0.3	6.6E-01
15352	PPME1	A_23_P64567	NM_016147	-1.4	3.33E-04	-1.4	2.17E-02	-2.5	2.22E-04	-0.8	3.33E-04	-0.8	1.99E-01	-0.8	1.69E-01	-1.7	1.99E-01	-0.6	2.15E-01	0.0	1.0E+00	0.2	1.0E+00	0.0	1.0E+00	0.0	1.0E+00	0.1	9.5E-01	-0.1	1.0E+00	0.1	8.7E-01	0.1	9.5E-01
15948	DEPDC1B	A_23_P361419	NM_018369	-1.4	3.31E-03	-1.7	1.27E-03	-1.4	2.95E-02	-1.1	3.31E-03	-1.6	1.43E-02	-0.9	2.82E-02	-1.8	1.43E-02	-1.6	8.93E-04	-0.2	1.0E+00	-0.3	1.0E+00	-0.2	7.3E-01	-0.2	1.0E+00	-0.2	6.6E-01	-0.2	1.0E+00	-0.2	5.2E-01	-0.3	6.6E-01
44037	BBS4	A_24_P176284	NM_033028	-1.4	2.14E-02	-1.0	2.55E-02	-1.5	2.14E-02	-1.4	1.33E-03	-0.8	1.02E-01	-0.9	1.02E-01	-0.4	5.06E-01	-0.8	6.16E-02	-0.1	1.0E+00	-0.1	1.0E+00	0.0	9.9E-01	-0.2	1.0E+00	0.0	9.8E-01	0.1	1.0E+00	0.0	9.8E-01	-0.1	9.4E-01
1140	ARV1	A_24_P144527	NM_022786	-1.4	1.36E-03	-1.3	6.00E-03	-2.4	1.36E-03	-1.4	2.05E-04	-0.9	3.27E-02	-0.7	3.27E-02	-1.0	1.72E-01	-0.9	2.46E-02	0.1	1.0E+00	0.0	1.0E+00	0.1	9.1E-01	0.2	1.0E+00	0.0	9.5E-01	0.0	1.0E+00	0.1	9.0E-01	-0.1	9.5E-01
14205	HOXA9	A_23_P500998	NM_152739	-1.4	1.73E-03	-1.4	1.73E-03	-1.9	2.19E-03	-1.3	2.22E-04	-1.1	7.83E-03	-1.1	7.83E-03	-0.9	2.97E-02	-1.2	4.37E-03	-0.1	1.0E+00	-0.1	1.0E+00	0.0	9.9E-01	-0.1	1.0E+00	0.1	1.0E+00	0.1	1.0E+00	0.1	6.9E-01	0.0	1.0E+00
15721	CR624291	A_24_P726215	CR624291	-1.4	2.22E-04	-1.4	1.50E-03	-1.6	2.22E-04	-1.1	9.51E-06	-1.0	4.99E-03	-0.9	3.72E-03	-1.5	4.99E-03	-1.0	6.63E-03	-0.1	1.0E+00	-0.1	1.0E+00	-0.3	7.1E-01	-0.1	1.0E+00	0.0	9.4E-01	-0.1	1.0E+00	0.0	9.4E-01	0.2	7.5E-01
35236	KRR1	A_32_P326819	NM_007043	-1.4	5.88E-04	-1.1	3.45E-03	-2.7	5.88E-04	-1.4	1.50E-04	-0.6	1.73E-01	-0.6	1.73E-01	-1.2	8.87E-02	-0.5	2.01E-01	0.1	1.0E+00	0.0	1.0E+00	0.1	8.9E-01	0.2	1.0E+00	-0.1	7.2E-01	-0.1	1.0E+00	-0.1	7.2E-01	-0.3	5.2E-01

Feature	Gene symbol	Probe	Transcript	72 hour								48 hours								24 hours								10 hours							
				siCASP8AP2								siCASP8AP2								siCASP8AP2								siCASP8AP2							
				Median	Median	1	1	3	3	6	6	Median	Median	1	1	3	3	6	6	Median	Median	1	1	3	3	6	6	Median	Median	1	1	3	3	6	6
				M	q	M	q	M	q	M	q	M	q	M	q	M	q	M	q	M	q	M	q	M	q	M	q	M	q	M	q	M	q	M	q
41957	SCOC	A_24_P46334	NM_032547	-1.4	9.22E-04	-1.4	3.52E-03	-2.3	7.27E-04	-1.0	9.22E-04	-0.7	1.30E-01	-0.6	9.74E-02	-1.0	3.10E-01	-0.7	1.30E-01	0.2	1.0E+00	0.0	1.0E+00	0.2	7.5E-01	0.3	1.0E+00	-0.2	7.7E-01	-0.1	1.0E+00	-0.2	7.7E-01	-0.4	6.6E-01
9209	AI024778	A_32_P216507	AI024778	-1.4	6.39E-03	-1.4	6.39E-03	-1.9	9.64E-03	-1.2	5.97E-03	-0.7	4.60E-01	-0.3	8.33E-01	-1.0	4.60E-01	-0.7	4.49E-01	0.3	1.0E+00	0.3	1.0E+00	0.1	9.6E-01	0.4	1.0E+00	0.0	9.8E-01	0.0	1.0E+00	0.0	9.8E-01	-0.1	9.6E-01
26953	PRDM16	A_32_P225816	NM_022114	-1.4	3.91E-02	-1.0	3.91E-02	-1.6	4.67E-02	-1.4	7.30E-03	-0.7	2.96E-02	-0.7	2.96E-02	-0.5	1.08E-01	-1.0	5.84E-03	-0.2	1.0E+00	-0.2	1.0E+00	-0.3	7.1E-01	0.0	1.0E+00	-0.2	8.2E-01	-0.2	1.0E+00	-0.2	7.9E-01	-0.4	8.2E-01
39075	AK098220	A_32_P51518	AK098220	-1.4	6.77E-03	-1.1	6.77E-03	-1.6	1.25E-02	-1.4	1.80E-03	-1.3	8.84E-02	-0.9	1.60E-01	-1.4	5.38E-02	-1.3	8.84E-02	0.0	1.0E+00	0.1	1.0E+00	-0.2	8.4E-01	0.0	1.0E+00	-0.1	9.6E-01	-0.2	1.0E+00	0.1	8.3E-01	-0.1	9.6E-01
12475	BX107298	A_32_P115258	BX107298	-1.4	6.00E-04	-1.3	6.00E-04	-1.8	2.53E-03	-1.4	1.44E-04	-0.8	1.26E-01	-0.4	4.73E-01	-1.1	6.37E-02	-0.8	1.26E-01	0.0	1.0E+00	0.0	1.0E+00	-0.1	9.1E-01	0.1	1.0E+00	-0.1	9.2E-01	0.0	1.0E+00	-0.1	7.6E-01	-0.1	9.2E-01
10431	C17orf75	A_24_P224526	NM_022344	-1.4	2.85E-03	-1.4	2.85E-03	-1.1	2.29E-02	-1.4	3.30E-04	-0.8	3.63E-02	-0.8	3.63E-02	-0.7	5.11E-02	-1.3	4.74E-03	-0.2	9.2E-01	-0.3	9.2E-01	-0.2	7.1E-01	-0.2	1.0E+00	-0.1	9.5E-01	-0.1	1.0E+00	0.1	9.0E-01	-0.1	9.5E-01
44493	CRYZL1	A_23_P218731	NM_145858	-1.3	5.58E-04	-1.3	5.58E-04	-1.5	5.92E-03	-1.3	3.51E-04	-1.1	2.33E-02	-0.6	2.49E-02	-1.1	2.33E-02	-1.1	2.49E-03	-0.1	1.0E+00	-0.1	1.0E+00	0.0	9.4E-01	-0.1	1.0E+00	-0.1	1.0E+00	-0.1	1.0E+00	0.0	1.0E+00	-0.1	9.1E-01
44702	ZMYM3	A_23_P137073	NM_005096	-1.3	3.36E-03	-1.3	2.76E-02	-2.6	3.36E-03	-1.3	3.03E-03	-0.9	1.67E-01	-0.9	1.67E-01	-1.6	1.71E-01	-0.9	9.76E-02	0.1	1.0E+00	0.1	1.0E+00	-0.1	9.3E-01	0.1	1.0E+00	-0.2	9.6E-01	-0.5	1.0E+00	-0.2	8.5E-01	-0.1	9.6E-01
19619	FAM104B	A_23_P11341	BC006406	-1.3	2.10E-03	-0.9	2.19E-03	-1.6	2.10E-03	-1.3	4.10E-05	-0.7	1.25E-02	-0.6	5.75E-02	-0.7	1.25E-02	-1.1	1.11E-03	0.0	1.0E+00	0.0	1.0E+00	0.0	8.8E-01	-0.2	1.0E+00	0.0	9.6E-01	0.1	1.0E+00	0.0	9.6E-01	-0.1	9.0E-01
18807	A_24_P170203	A_24_P170203	A_24_P170203	-1.3	1.01E-02	-1.3	2.39E-02	-1.5	1.01E-02	-1.0	3.89E-03	-1.3	1.06E-02	-1.3	1.06E-02	-0.7	1.25E-01	-1.3	1.45E-03	-0.1	1.0E+00	-0.2	1.0E+00	0.0	9.6E-01	-0.1	1.0E+00	-0.1	9.7E-01	-0.1	1.0E+00	0.1	8.5E-01	-0.1	9.7E-01
35765	DTWD1	A_23_P3215	AF168717	-1.3	8.85E-04	-1.1	1.03E-03	-1.7	8.85E-04	-1.3	2.63E-05	-0.9	1.35E-02	-0.5	3.58E-02	-1.1	1.35E-02	-0.9	3.50E-03	0.1	1.0E+00	0.0	1.0E+00	0.1	8.5E-01	0.1	1.0E+00	0.0	9.7E-01	0.0	1.0E+00	0.0	9.7E-01	-0.3	5.8E-01
40104	DTWD1	A_23_P3215	AF168717	-1.3	1.02E-03	-1.1	1.02E-03	-1.6	1.60E-03	-1.3	4.47E-05	-0.9	2.28E-02	-0.6	2.28E-02	-1.1	3.07E-02	-0.9	3.38E-03	0.1	1.0E+00	0.1	1.0E+00	0.1	8.7E-01	0.1	1.0E+00	-0.1	7.0E-01	0.0	1.0E+00	-0.1	7.0E-01	-0.3	4.3E-01
24148	A_24_P482189	A_24_P482189	A_24_P482189	-1.3	1.12E-03	-1.0	1.12E-03	-1.6	2.52E-03	-1.3	8.34E-05	-0.5	7.53E-02	-0.1	8.64E-01	-0.5	7.53E-02	-0.8	4.00E-02	0.0	1.0E+00	0.1	1.0E+00	-0.1	8.6E-01	0.0	1.0E+00	-0.2	7.1E-01	0.0	1.0E+00	-0.2	5.3E-01	-0.2	7.1E-01
37805	ADAT2	A_23_P134014	NM_182503	-1.3	2.70E-04	-1.3	2.70E-04	-1.3	3.03E-03	-1.3	5.03E-05	-0.7	3.27E-02	-0.7	3.27E-02	-0.6	1.04E-01	-1.2	8.90E-04	-0.2	1.0E+00	-0.2	1.0E+00	-0.2	7.2E-01	0.0	1.0E+00	-0.1	6.7E-01	0.0	1.0E+00	-0.1	6.7E-01	-0.3	4.6E-01
15598	FAM104B	A_32_P194025	BC006406	-1.3	4.38E-03	-0.8	4.38E-03	-1.4	6.22E-03	-1.3	1.49E-04	-0.8	2.31E-02	-0.3	3.92E-01	-0.8	2.31E-02	-0.8	9.60E-03	-0.1	1.0E+00	0.0	1.0E+00	-0.1	8.6E-01	-0.1	1.0E+00	-0.1	8.2E-01	0.1	1.0E+00	-0.1	8.0E-01	-0.1	8.2E-01
13262	THC2617699	A_32_P13371	THC2617699	-1.3	4.72E-03	-1.3	4.72E-03	-1.3	2.19E-02	-1.3	1.53E-03	-0.8	1.85E-02	-0.8	2.09E-02	-0.7	1.85E-02	-0.9	1.74E-02	-0.1	1.0E+00	-0.1	1.0E+00	0.0	9.5E-01	-0.1	1.0E+00	0.0	8.4E-01	0.0	1.0E+00	0.1	7.3E-01	-0.2	8.4E-01
20742	TMEM68	A_23_P136172	NM_152417	-1.3	6.40E-04	-1.3	9.83E-04	-1.6	6.40E-04	-1.1	6.70E-05	-1.0	1.69E-02	-0.8	1.17E-02	-1.0	4.05E-02	-1.0	1.69E-02	-0.1	1.0E+00	-0.1	1.0E+00	-0.1	8.0E-01	0.1	1.0E+00	-0.2	4.3E-01	-0.1	1.0E+00	-0.2	4.3E-01	-0.3	4.1E-01
41926	GNPAT	A_23_P85777	NM_014236	-1.3	1.02E-02	-0.8	1.45E-02	-1.3	1.02E-02	-1.3	2.79E-04	-0.8	1.12E-01	-0.3	6.25E-01	-1.0	1.12E-01	-0.8	1.05E-02	-0.1	1.0E+00	-0.1	1.0E+00	-0.1	8.0E-01	0.0	1.0E+00	-0.1	9.3E-01	-0.1	1.0E+00	-0.1	8.1E-01	-0.1	9.3E-01
12935	VPS29	A_23_P128384	NM_016226	-1.3	1.27E-03	-1.3	1.27E-03	-1.9	2.15E-03	-1.3	1.60E-04	-1.3	1.36E-02	-1.1	2.76E-02	-1.3	1.36E-02	-1.6	5.47E-03	0.0	1.0E+00	0.0	1.0E+00	0.1	8.6E-01	-0.2	1.0E+00	0.3	5.7E-01	-0.1	1.0E+00	0.4	2.6E-01	0.3	5.7E-01
2915	THC2639718	A_24_P588897	THC2639718	-1.3	2.38E-03	-1.0	1.48E-02	-2.1	2.38E-03	-1.3	7.52E-04	-0.7	1.97E-02	-0.6	1.11E-01	-0.7	1.97E-02	-1.0	2.81E-03	-0.1	1.0E+00	-0.2	1.0E+00	-0.1	9.0E-01	0.1	1.0E+00	-0.2	7.9E-01	-0.2	1.0E+00	0.1	7.6E-01	-0.2	7.9E-01
9815	NOTCH1	A_23_P60387	NM_017617	-1.3	5.16E-03	-1.3	5.16E-03	-1.3	2.17E-02	-1.3	1.08E-03	-0.7	3.44E-01	-0.7	3.49E-01	-0.8	3.44E-01	-0.6	3.17E-01	0.1	1.0E+00	0.1	1.0E+00	-0.2	7.4E-01	0.1	1.0E+00	0.1	9.2E-01	-0.1	1.0E+00	0.1	9.2E-01	0.3	8.7E-01
40132	MRPL24	A_23_P137848	NM_145729	-1.3	1.52E-03	-1.3	1.52E-03	-1.1	2.14E-02	-1.6	1.24E-04	-1.0	8.51E-03	-1.0	1.10E-02	-0.9	8.51E-03	-1.1	1.45E-03	-0.1	1.0E+00	-0.1	1.0E+00	-0.1	8.3E-01	-0.2	1.0E+00	0.1	9.0E-01	0.1	1.0E+00	0.0	9.0E-01	0.1	7.2E-01
14358	AJ001827	A_32_P179572	AJ001827	-1.3	3.72E-03	-1.3	3.72E-03	-2.1	6.65E-04	-0.6	3.92E-03	-0.3	8.14E-01	0.0	9.75E-01	-1.0	2.31E-01	-0.3	8.14E-01	0.3	1.0E+00	0.3	1.0E+00	0.0	9.9E-01	0.5	1.0E+00	0.1	7.7E-01	0.3	1.0E+00	0.1	7.1E-01	0.1	7.7E-01
32748	PXMP3	A_23_P31702	NM_000318	-1.3	2.90E-03	-1.2	2.90E-03	-1.7	4.43E-03	-1.3	4.92E-04	-0.8	1.34E-01	-0.4	4.78E-01	-1.2	1.34E-01	-0.8	3.92E-02	-0.1	1.0E+00	-0.1	1.0E+00	-0.1	8.6E-01	0.0	1.0E+00	-0.1	8.8E-01	-0.1	1.0E+00	-0.1	8.1E-01	-0.2	8.8E-01
44332	C1D	A_23_P56590	NM_006333	-1.3	2.86E-03	-1.3	4.40E-03	-1.7	2.86E-03	-1.1	2.38E-03	-0.8	1.70E-02	-0.9	9.92E-03	-0.2	8.32E-01	-0.8	1.70E-02	-0.1	1.0E+00	-0.2	1.0E+00	-0.1	8.7E-01	-0.1	1.0E+00	-0.2	7.9E-01	-0.2	1.0E+00	-0.2	6.9E-01	-0.2	7.9E-01
38429	ARV1	A_23_P135995	NM_022786	-1.3	4.14E-03	-1.2	7.32E-03	-1.9	4.14E-03	-1.3	4.88E-04	-0.8	4.30E-02	-0.6	4.30E-02	-0.9	2.33E-01	-0.8	5.20E-03	-0.1	1.0E+00	-0.2	1.0E+00	-0.1	9.5E-01	-0.1	1.0E+00	0.0	9.3E-01	-0.1	1.0E+00	0.0	9.3E-01	0.1	9.2E-01
23610	ZSWIM7	A_23_P164421	NM_001042698	-1.3	9.72E-05	-1.3	9.72E-05	-1.1	4.08E-03	-1.3	1.35E-05	-0.7	1.82E-02	-0.7	2.68E-02	-0.7	1.82E-02	-1.2	1.19E-03	-0.1	1.0E+00	-0.1	1.0E+00	-0.2	7.1E-01	0.0	1.0E+00	-0.1	9.7E-01	-0.1	1.0E+00	-0.1	8.6E-01	0.0	9.7E-01
41681	CX783461	A_32_P146898	CX783461	-1.3	2.09E-03	-1.1	2.09E-03	-1.8	3.01E-03	-1.3	3.33E-04	-1.0	6.59E-02	-0.9	6.59E-02	-1.4	1.21E-01	-1.0	3.67E-02	0.0	1.0E+00	0.0	1.0E+00	-0.1	9.3E-01	0.0	1.0E+00	0.2	9.9E-01	0.2	1.0E+00	0.0	9.9E-01	0.2	8.1E-01
4538	ZNF639	A_24_P123155	NM_016331	-1.3	1.05E-03	-1.3	1.05E-03	-1.6	1.82E-03	-0.9	5.53E-04	-1.1	2.95E-02	-1.0	5.06E-03	-1.3	1.16E-01	-1.1	2.95E-02	0.0	1.0E+00	-0.2	1.0E+00	0.0	9.6E-01	0.1	1.0E+00	-0.1	9.5E-01	-0.1	1.0E+00	0.0	9.5E-01	-0.3	5.2E-01
30917	FAM36A	A_32_P106646	NM_198076	-1.3	1.27E-03	-1.1	4.37E-03	-3.1	7.41E-04	-1.3	1.27E-03	-1.0	2.59E-03	-0.8	3.43E-02	-1.9	1.81E-03	-1.0	2.59E-03	-0.3	1.0E+00	-0.1	1.0E+00	-1.0	9.6E-03	-0.3	1.0E+00	-0.2	8.6E-01	-0.1	1.0E+00	-0.4	2.1E-01	-0.2	8.6E-01
39017	DTWD1	A_23_P3212	NM_020234	-1.3	1.77E-04	-1.3	1.77E-04	-1.5	1.09E-03	-1.2	1.26E-05	-0.9	1.46E-02	-0.5	6.71E-02	-0.9	1.46E-02	-1.2	9.61E-04	0.1	1.0E+00	-0.1	1.0E+00	0.1	8.4E-01	0.1	1.0E+00	-0.1	9.6E-01	-0.1	1.0E+00	-0.1	9.2E-01	-0.1	9.6E-01
17435	ASCL2	A_32_P171061	NM_005170	-1.3	1.99E-03	-1.2	1.99E-03	-1.3	1.58E-02	-1.6	1.15E-04	-0.7	1.61E-02	-0.7	1.61E-02	-0.4	3.08E-01	-1.2	2.39E-04	-0.2	1.0E+00	-0.1	1.0E+00	-0.2	7.2E-01	-0.2	1.0E+00	0.1	8.7E-01	0.1	1.0E+00	0.1	8.5E-01	0.1	8.7E-01
25219	ARID2	A_32_P74955	NM_152641	-1.3	1.48E-03	-1.2	3.45E-03	-2.7	1.48E-03	-1.3	7.47E-04	-0.8	4.57E-02	-0.5	3.99E-01	-1.6	3.79E-02	-0.8	4.57E-02	-0.1	1.0E+00	-0.1	1.0E+00	-0.4	7.1E-01	0.1	1.0E+00	-0.2	7.8E-01	-0.1	1.0E+00	-0.2	6.5E-01	-0.2	7.8E-01
19410	AK123649	A_32_P36582	AK123649	-1.3	2.84E-03	-1.3	2.84E-03	-1.8	4.85E-03	-1.3	8.89E-04	-0.9	1.64E-01	-0.7	1.64E-01	-1.0	2.20E-01	-0.9	1.75E-02	0.0	1.0E+00	-0.1	1.0E+00	0.0	9.7E-01	0.1	1.0E+00	0.0	9.8E-01	0.0	1.0E+00	0.2	5.3E-01	0.0	9.8E-01
9393	MMAB	A_24_P56221	NM_052845	-1.3	1.35E-03	-1.3	1.35E-03	-1.3	1.05E-02	-1.3	2.47E-04	-0.6	4.26E-01	-0.8	1.85E-01	-0.6	4.26E-01	-0.6	4.64E-01	0.0	1.0E+00	0.0	1.0E+00	0.0	9.6E-01	0.2	1.0E+00	-0.1	9.7E-01	-0.1	1.0E+00	-0.2	6.7E-01	-0.1	9.7E-01
42194	BE703537	A_32_P104300	BE703537	-1.3	2.04E-04	-1.3	1.24E-04	-1.5	1.42E-03	-1.0	2.04E-04	-0.5	2.25E-01	-0.5	2.25E-01	-0.5	3.62E-01	-0.6	1.15E-01	0.0	1.0E+00	-0.2	1.0E+00	0.0	9.8E-01	0.2	1.0E+00	-0.3	3.8E-01	-0.2	1.0E+00	-0.3	3.8E-01	-0.5	2.6E-01
753	THC2734720	A_32_P211708	THC2734720	-1.3	8.65E-04	-1.3	8.65E-04	-0.8	4.00E-02	-1.3	1.91E-04	-0.5	2.89E-01	-0.4	3.67E-01	-0.5	2.89E-01	-0.8	9.21E-02	0.1	1.0E+00	0.1	1.0E+00	0.0	9.9E-01	0.1	1.0E+00	0.0	1.0E+00	0.0	1.0E+00	0.0	1.0E+00	-0.2	9.0E-01
40569	BTBD3	A_23_P102759	NM_014962	-1.3	2.40E-04	-1.3	2.40E-04	-1.4	2.40E-03	-1.0	1.60E-04	-0.7	9.99E-02	-0.7	9.99E-02	-0.4	2.64E-01	-0.7	3.29E-02	-0.3	1.0E+00	-0.3	1.0E+00	-0.5	7.1E-01	-0.3	1.0E+00	-0.2	7.2E-01	-0.2	1.0E+00	-0.3	3.0E-01	-0.2	7.2E-01
17120	PPA2	A_24_P214625	NM_176869	-1.3	1.08E-02	-1.3	2.90E-02	-2.3	1.08E-02	-1.2	1.05E-02	-0.8	1.10E-01	-0.4	6.16E-01	-0.8	1.10E-01	-0.9	1.04E-01	0.0	1.0E+00	-0.3	1.0E+00	0.1	9.5E-01	0.0	1.0E+00	0.2	9.0E-01	-0.1	1.0E+00	0.3	6.3E-01	0.2	9.0E-01
35214	TSNAX	A_23_P51572	NM_005999	-1.3	2.85E-02	-1.3	2.85E-02	-1.3	1.67E-02	-0.7	3.59E-02	-0.6	1.23E-01	-1.2	1.91E-02	-0.6	4.47E-01	-0.6	1.23E-01	-0.1	8.6E-01	-0.7	2.9E-01	-0.1	8.6E-01	-0.1	1.0E+00	-0.2	8.9E-01	-0.4	1.0E+00	-0.2	7.9E-01	-0.2	8.9E-01
13527	HDDC2	A_24_P55250	NM_016063	-1.3	6.48E-03	-1.3	8.02E-03	-1.4	6.48E-03	-1.3	2.31E-04	-1.0	1.26E-01	-0.8	1.26E-01	-1.0	1.44E-01	-1.0	5.57E-02	0.0	1.0E+00	-0.2	1.0E+00	0.0	9.9E-01	0.0	1.0E+00	0.2	8.2E-01	0.0	1.0E+00	0.2	6.7E-01	0.2	8.2E-01
42328	RRP15	A_32_P221822	NM_016052	-1.3	2.62E-03	-1.3	2.62E-03	-1.6	1.23E-02	-1.3	1.26E-03	-0.9	2.38E-02	-0.5	2.09E-01	-1.1	2.38E-02	-0.9	2.15E-02	0.0	1.0E+00	0.0	1.0E+00	-0.2	7.6E-01	0.1	1.0E+00	-0.2	7.3E-01	-0.2	1.0E+00	-0.2	7.3E-01	-0.4	4.4E-01
38239	AK055214	A_24_P778741	AK055214	-1.3	1.50E-03	-1.5	3.44E-04	-1.3	7.73E-03	-1.0	1.50E-03	-1.4	6.98E-03	-0.9	6.79E-02	-1.4	6.98E-03	-1.5	6.43E-03	-0.1	1.0E+00	0.0	1.0E+00	-0.5	7.1E-01	-0.1	1.0E+00	-0.2	7.5E-01	-0.1	1.0E+00	-0.2	7.5E-01	-0.4	7.0E-01
2021	ZMYM1	A_23_P161091	NM_024772	-1.3	5.73E-05	-1.3	5.73E-05	-1.6	6.15E-04	-1.1	5.15E-05	-1.3	6.85E-03	-0.9	6.85E-03	-1.3	1.70E-02	-1.3	1.04E-03	-0.1	1.0E+00	-0.1	1.0E+00	-0.1	7.6E-01	-0.1	1.0E+00	-0.1	9.2E-01	-0.1	1.0E+00	0.0	9.2E-01	-0.1	8.3E-01
34593	RNFT2	A_23_P204158	NM_032814	-1.3	5.01E-04	-1.0	5.01E-04	-1.3	2.11E-03	-1.3	1.82E-05	-0.4	3.21E-01	-0.3	7.62E-01	-0.4	3.21E-01	-0.6	9.71E-02	-0.2	1.0E+00	-0.2	1.0E+00	-0.2	7.2E-01	-0.1	1.0E+00	-0.2	8.8E-01	-0.2	1.0E+00	-0.3	6.1E-01	-0.2	8.8E-01
44046	NUDT9	A_23_P94860	NM_024047	-1.3	2.65E-03	-1.2	2.65E-03	-1.3	2.16E-02	-1.3	2.04E-03	-0.9	6.05E-02	-0.5	3.53E-01	-0.9	6.05E-02	-1.1	2.37E-02	-0.2	1.0E+00	-0.1	1.0E+00	-0.2	7.9E-01	-0.3	1.0E+00	0.1	9.5E-01	0.0	1.0E+00	0.2	7.3E-01	0.1	9.5E-01
40076	SORBS2	A_24_P140475	NM_021069	-1.3	4.54E-04	-1.3	4.54E-04	-1.6	1.73E-03	-1.2	9.53E-05	-0.8	9.40E-02	-0.6	2.55E-01	-1.0	9.40E-02	-0.8	4.68E-02	-0.2	1.0E+00	-0.2	1.0E+00	-0.3	7.1E-01	-0.1	1.0E+00	-0.2	7.7E-01	-0.2	1.0E+00	-0.3	2.2E-01	-0.2	7.7E-01
33846	BCORL1	A_32_P462013	AK021694	-1.3	3.76E-03	-1.1	3.76E-03	-1.3	5.79E-03	-1.3	1.15E-04	-0.5	3.55E-02	-0.5	4.13E-02	-0.5	3.55E-02	-0.8	1.89E-02	0.0	1.0E+00	0.0	1.0E+00	-0.1	8.0E-01	0.1	1.0E+00	0.1	9.8E-01	0.1	1.0E+00	0.1	9.0E-01	0.0	9.8E-01
25495	ANKRD46	A_23_P94095	NM_198401	-1.3	6.88E-03	-1.3	3.12E-03	-1.3	2.37E-02	-1.0	6.88E-03	-0.8	1.19E-01	-0.7	1.19E-01	-1.0	2.67E-01	-0.8	6.87E-02	-0.2	1.0E+00	-0.4	1.0E+00	-0.2	8.3E-01	-0.1	1.0E+00	-0.3	7.1E-01	-0.3	1.0E+00	-0.3	6.5E-01	-0.4	7.1E-01
7061	DTWD1	A_23_P3215	AF168717	-1.3	9.28E-04	-1.0	1.49E-03	-1.7	9.28E-04	-1.3	2.75E-05	-0.9	2.50E-02	-0.6	2.54E-02	-1.0	2.50E-02	-0.9	4.46E-03	0.2	1.0E+00	0.2	1.0E+00	0.2	7.9E-01	0.3	1.0E+00	-0.1	8.1E-01	0.0	1.0E+00	-0.1	8.1E-01	-0.3	4.8E-01
24136	TMTC2	A_23_P382705	NM_152588	-1.3	2.88E-03	-1.3	3.14E-03	-1.7	2.88E-03	-0.9	1.96E-03	-0.8	3.80E-02	-0.6	5.30E-02	-0.9	1.06E-02	-0.8	3.80E-02	-0.1	1.0E+00	-0.1	1.0E+00	-0.5	7.1E-01	0.0	1.0E+00	-0.1	9.7E-01	-0.3	1.0E+00	-0.1	8.0E-01	0.0	9.7E-01
11778	AK021744	A_32_P167459	AK021744	-1.3	2.25E-03	-1.1	2.25E-03	-1.3	1.39E-02	-1.3	6.57E-04	-0.8	1.63E-01	-0.5	3.33E-01	-0.8	1.03E-01	-0.8	1.63E-01	-0.1	1.0E+00	-0.1	1.0E+00	-0.1	8.9E-01	0.1	1.0E+00	0.0	9.4E-01	0.0	1.0E+00	-0.1	9.2E-01	0.1	9.4E-01
44667	DKFZP564C152	A_23_P336728	AL049980	-1.3	1.34E-03	-1.2	1.34E-03	-1.5	7.81E-03	-1.3	9.45E-04	-0.9	9.42E-02	-0.5	3.40E-01	-1.2	4.20E-02	-0.9	9.42E-02	0.0	1.0E+00	0.0	1.0E+00	-0.2	8.1E-01	0.1	1.0E+00	-0.1	9.8E-01	-0.2	1.0E+00	0.0	9.8E-01	-0.1	9.2E-01
393	ANKRD26L1	A_23_P97123	BC007072	-1.3	1.96E-03	-1.3	8.35E-04	-1.7	1.96E-03	-0.6	1.02E-02	-0.3	6.87E-01	-0.2	7.19E-01	-0.5	3.61E-01	-0.3	6.87E-01	0.3	1.0E+00	0.3	1.0E+00	0.0	9.9E-01	0.3	1.0E+00	0.2	9.2E-01	0.2	1.0E+00	0.1	9.2E-01	0.2	7.4E-01
10498	AK023647	A_24_P889070	AK023647	-1.2	6.26E-04	-1.1	6.26E-04	-1.5	2.25E-03	-1.2	1.51E-04	-0.9	9.15E-02	-0.4	4.15E-01	-0.9	9.15E-02	-1.0	2.45E-02	-0.2	1.0E+00	0.0	1.0E+00	-0.4	7.1E-01	-0.2	1.0E+00	-0.1	9.8E-01	-0.1	1.0E+00	-0.1	8.5E-01	0.0	9.8E-01
41468	JMJD2C	A_23_P112201	NM_015061	-1.2	1.20E-02	-0.9	1.78E-02	-1.6	1.20E-02	-1.2	3.02E-03	-0.8	2.86E-02	-0.7	4.72E-02	-1.0	2.86E-02	-0.8	3.87E-03	-0.2	1.0E+00	-0.2	1.0E+00	-0.3	7.1E-01	-0.1	1.0E+00	0.0	9.8E-01	0.0	1.0E+00	0.1	8.9E-01	0.0	9.8E-01
29754	A_32_P128399	A_32_P128399	A_32_P128399	-1.2	1.31E-03	-1.5	1.31E-03	-0.8	4.11E-03	-1.2	2.41E-06	-1.3	2.30E-03	-1.3	2.30E-03	-0.5	7.69E-02	-1.4	6.81E-04	-0.2	1.0E+00	-0.2	1.0E+00	-0.1	8.2E-01	-0.2	1.0E+00	0.0	1.0E+00	0.1	1.0E+00	0.0	9.9E-01	0.0	1.0E+00
35002	FBXL4	A_23_P214739	NM_012160	-1.2	5.19E-04	-1.3	5.19E-04	-1.1	1.50E-02	-1.2	2.82E-04	-0.7	1.34E-01	-0.7	1.34E-01	-0.7	1.77E-01	-1.1	1.03E-02	-0.1	1.0E+00	-0.1	1.0E+00	-0.2	7.1E-01	-0.1	1.0E+00	-0.2	9.1E-01	0.0	1.0E+00	-0.2	7.4E-01	-0.2	9.1E-01
31431	PLD6	A_23_P360626	NM_178836	-1.2	5.97E-03	-1.2	5.97E-03	-1.1	4.81E-02	-1.3	3.31E-03	-0.9	1.15E-02	-0.7	3.95E-02	-0.9	1.09E-02	-0.9	1.15E-02	-0.2	1.0E+00	-0.2	1.0E+00	-0.3	7.1E-01	-0.2	1.0E+00	0.1	9.6E-01	0.0	1.0E+00	0.1	7.5E-01	0.1	9.6E-01
41355	ADAT2	A_24_P186204	NM_182503	-1.2	2.00E-04	-1.2	2.00E-04	-1.1	2.98E-03	-1.4	1.62E-05	-0.9	8.71E-03	-0.8	8.71E-03	-0.9	1.15E-02	-1.3	1.30E-03	0.0	1.0E+00	-0.3	1.0E+00	0.0	9.0E-01	0.0	1.0E+00	-0.1	8.9E-01	-0.1	1.0E+00	-0.1	8.9E-01	-0.1	8.5E-01
6547	TMEM109	A_23_P203364	NM_024092	-1.2	1.30E-03	-1.2	2.26E-02	-2.1	1.30E-03	-1.2	2.81E-04	-0.6	4.62E-01	-0.6	4.36E-01	-0.7	4.62E-01	-0.5	4.79E-01	0.2	1.0E+00	0.2	1.0E+00	0.2	8.5E-01	0.3	1.0E+00	0.0	1.0E+00	-0.1	1.0E+00	0.0	9.9E-01	0.0	1.0E+00
24126	CLN6	A_23_P117797	NM_017882	-1.2	1.21E-02	-1.0	1.53E-02	-1.5	1.21E-02	-1.2	1.18E-03	-0.4	3.23E-01	-0.2	6.50E-01	-1.2	5.43E-02	-0.4	3.23E-01	-0.1	1.0E+00	0.0	1.0E+00	-0.1	7.6E-01	-0.1	1.0E+00	0.1	6.3E-01	0.1	1.0E+00	0.1	5.9E-01	0.2	6.3E-01
39358	TRDMT1	A_23_P115636	NM_004412	-1.2	7.27E-04	-1.2	5.03E-03	-1.7	7.27E-04	-1.0	2.04E-04	-0.6	1.72E-01	-0.6	1.72E-01	-0.6	3.32E-01	-0.5	1.33E-01	0.0	1.0E+00	-0.1	1.0E+00	0.0	1.0E+00	0.0	1.0E+00	-0.1	8.3E-01	0.1	1.0E+00	-0.1	7.4E-01	-0.2	8.3E-01
41013	STRA6	A_23_P65779	NM_022369	-1.2	8.90E-03	-1.3	8.90E-03	-1.1	2.25E-02	-1.2	4.49E-04	-0.9	1.47E-02	-1.1	6.33E-03	-0.9	1.47E-02	-0.9	1.15E-01	-0.1	1.0E+00	-0.1	1.0E+00	0.0	9.3E-01	-0.1	1.0E+00	0.1	9.8E-01	0.1	1.0E+00	0.0	9.8E-01	0.1	8.4E-01
44559	FDXACB1	A_23_P362261	NM_138378	-1.2	3.28E-05	-1.4	3.28E-05	-1.2	9.71E-04	-1.2	7.12E-06	-0.8	5.43E-02	-0.6	1.89E-01	-0.9	5.43E-02	-0.8	3.50E-02	-0.3	1.0E+00	-0.3	1.0E+00	-0.4	4.5E-01	-0.2	1.0E+00	-0.1	8.8E-01	-0.1	1.0E+00	-0.1	8.4E-01	-0.2	8.8E-01
24045	BC004287	A_32_P229493	BC004287	-1.2	6.15E-04	-1.2	6.15E-04	-1.4	5.29E-03	-1.2	2.79E-04	-1.1	4.82E-02	-0.4	5.25E-01	-1.5	4.82E-02	-1.1	1.25E-02	-0.2	1.0E+00	-0.2	1.0E+00	-0.5	5.6E-01	-0.2	1.0E+00	-0.2	8.7E-01	-0.2	1.0E+00	-0.1	8.7E-01	-0.3	6.1E-01
19364	LOC153346	A_24_P560519	AK024346	-1.2	6.98E-03	-1.2	6.98E-03	-1.3	2.19E-02	-0.9	5.14E-03	-0.8	1.09E-02	-0.6	9.05E-02	-0.8	1.09E-02	-0.9	9.29E-03	-0.2	1.0E+00	-0.2	1.0E+00	-0.4	6.7E-01	0.1	1.0E+00	0.1	9.7E-01	-0.1	1.0E+00	0.1	8.8E-01	0.1	9.7E-01
34922	NIPSNAP1	A_24_P237278	NM_003634	-1.2	4.60E-03	-0.7	2.51E-02	-1.5	4.60E-03	-1.2	2.91E-04	-0.3	8.04E-01	-0.1	9.28E-01	-0.3	8.04E-01	-0.3	7.28E-01	0.0	1.0E+00	0.2	1.0E+00	0.0	9.6E-01	0.0	1.0E+00	0.0	9.9E-01	-0.2	1.0E+00	0.0	9.9E-01	0.1	9.6E-01
21265	NUDT12	A_23_P259090	NM_031438	-1.2	2.68E-03	-1.1	2.68E-03	-1.5	5.83E-03	-1.2	4.82E-04	-0.4	4.57E-01	-0.4	4.84E-01	-0.4	4.57E-01	-0.7	7.50E-02	-0.1	1.0E+00	0.0	1.0E+00	-0.1	9.0E-01	-0.1	1.0E+00	-0.1	7.9E-01	0.0	1.0E+00	-0.1	7.9E-01	-0.2	6.0E-01
26280	RHBDD1	A_23_P147514	ENST00000392062	-1.2	6.66E-04	-1.2	6.66E-04	-1.5	1.81E-03	-1.1	2.57E-04	-0.8	3.38E-02	-0.8	3.38E-02	-0.3	4.79E-01	-1.0	6.58E-03	-0.2	9.8E-01	-0.2	9.8E-01	-0.2	7.1E-01	-0.1	1.0E+00	-0.1	7.7E-01	0.0	1.0E+00	-0.1	7.1E-01	-0.2	7.7E-01
37046	DTWD1	A_24_P70117	NM_020234	-1.2	1.31E-03	-1.1	1.31E-03	-1.8	1.62E-03	-1.2	1.74E-04	-0.9	5.20E-02	-0.6	5.64E-02	-0.9	5.20E-02	-1.0	1.87E-03	0.1	1.0E+00	0.1	1.0E+00	0.1	8.2E-01	0.2	1.0E+00	0.0	9.3E-01	0.0	1.0E+00	0.0	9.3E-01	-0.2	8.1E-01
5004	UBXN2B	A_32_P148796	NM_001077619	-1.2	4.32E-03	-1.1	4.32E-03	-1.5	7.27E-03	-1.2	1.31E-03	-0.8	8.26E-02	-0.7	8.46E-02	-0.8	8.26E-02	-0.8	5.74E-02	-0.1	1.0E+00	-0.3	1.0E+00	-0.1	8.0E-01	-0.1	1.0E+00	0.0	1.0E+00	-0.1	1.0E+00	0.1	7.3E-01	0.0	1.0E+00
5946	THC2695604	A_32_P99690	THC2695604	-1.2	1.37E-03	-1.2	1.37E-03	-1.5	7.12E-03	-1.2	1.03E-03	-0.8	2.12E-01	-0.3	6.68E-01	-0.8	2.12E-01	-0.8	9.78E-02	-0.2	1.0E+00	-0.2	1.0E+00	-0.3	7.1E-01	-0.1	1.0E+00	0.0	9.7E-01	0.0	1.0E+00	-0.1	9.0E-01	0.0	9.7E-01
15173	NFE2L3	A_23_P42718	NM_004289	-1.2	8.76E-03	-1.3	7.76E-03	-1.2	1.09E-02	-0.6	8.76E-03	-1.1	2.39E-02	-0.7	3.38E-02	-1.1	2.39E-02	-1.1	2.34E-03	-0.2	1.0E+00	-0.2	1.0E+00	-0.2	7.3E-01	-0.3	1.0E+00	-0.1	8.0E-01	-0.1	1.0E+00	0.2	4.7E-01	-0.1	8.0E-01
8554	UBXN2A	A_23_P131240	NM_181713	-1.2	3.54E-03	-1.2	1.11E-02	-2.1	3.54E-03	-1.1	2.24E-03	-0.8	7.28E-02	-0.7	2.44E-01	-0.8	7.28E-02	-0.8	3.75E-02	-0.3	1.0E+00	-0.1	1.0E+00	-0.4	7.1E-01	-0.3	1.0E+00	0.0	9.7E-01	0.0	1.0E+00	-0.1	8.8E-01	0.1	9.7E-01
35111	CHDH	A_23_P69293	NM_018397	-1.2	7.98E-03	-1.3	7.98E-03	-1.2	1.25E-02	-1.1	6.98E-04	-0.9	3.30E-02	-0.9	9.90E-03	-0.9	3.30E-02	-0.6	1.20E-01	-0.1	1.0E+00	0.0	1.0E+00	-0.1	8.5E-01	-0.1	1.0E+00	0.2	8.8E-01	0.2	1.0E+00	0.1	8.8E-01	0.4	6.3E-01
40065	MTMR9	A_24_P261052	NM_015458	-1.2	4.66E-04	-1.4	4.66E-04	-1.1	1.10E-03	-1.2	6.35E-06	-0.7	1.08E-01	-1.0	8.56E-03	-0.3	6.85E-01	-0.7	1.08E-01	-0.3	8.0E-01	-0.6	4.0E-01	0.1	8.0E-01	-0.3	1.0E+00	-0.3	9.7E-01	-0.3	1.0E+00	0.0	9.7E-01	-0.4	1.8E-01
26166	FNTB	A_23_P25835	NM_002028	-1.2	6.05E-03	-0.8	2.52E-02	-1.5	6.05E-03	-1.2	6.01E-04	-0.5	6.81E-02	-0.4	9.56E-02	-0.9	6.81E-02	-0.5	6.44E-02	0.0	1.0E+00	0.0	1.0E+00	0.0	9.9E-01	-0.1	1.0E+00	0.2	4.3E-01	0.0	1.0E+00	0.2	2.0E-01	0.3	4.3E-01
4684	NOTCH1	A_23_P60387	NM_017617	-1.2	8.10E-03	-1.1	8.10E-03	-1.4	1.61E-02	-1.2	1.50E-03	-0.8	2.48E-01	-0.8	2.48E-01	-1.0	3.05E-01	-0.7	2.26E-01	0.0	1.0E+00	0.0	1.0E+00	-0.2	8.1E-01	0.1	1.0E+00	-0.1	1.0E+00	-0.2	1.0E+00	-0.1	9.5E-01	0.0	1.0E+00
25592	BX393727	A_32_P47543	BX393727	-1.2	8.17E-03	-1.1	8.17E-03	-1.2	4.44E-02	-1.3	3.63E-03	-1.0	4.18E-02	-1.1	4.18E-02	-0.8	1.21E-01	-1.0	3.91E-02	-0.2	1.0E+00	0.2	1.0E+00	-0.4	7.1E-01	-0.2	1.0E+00	-0.1	9.8E-01	0.2	1.0E+00	-0.1	8.9E-01	-0.1	9.8E-01
36681	HIST2H2AB	A_23_P343927	NM_175065	-1.2	1.37E-03	-1.2	1.37E-03	-1.1	9.34E-03	-1.3	6.98E-05	-1.2	4.81E-02	-1.2	4.81E-02	-0.8	2.02E-01	-1.2	1.81E-03	-0.4	1.0E+00	-0.3	1.0E+00	-0.6	7.1E-01	-0.4	1.0E+00	-0.3	9.2E-01	0.1	1.0E+00	-0.3	8.0E-01	-0.3	9.2E-01
42077	CLYBL	A_23_P376591	NM_206808	-1.2	1.95E-03	-1.1	1.95E-03	-1.3	3.36E-03	-1.2	5.47E-05	-0.4	2.45E-01	-0.4	3.44E-01	-0.4	2.45E-01	-0.6	1.97E-02	0.0	1.0E+00	-0.1	1.0E+00	0.0	9.3E-01	0.0	1.0E+00	0.2	9.3E-01	0.2	1.0E+00	0.2	8.3E-01	0.2	9.3E-01
17351	TBCE	A_23_P52147	NM_001079515	-1.2	1.26E-03	-1.1	1.26E-03	-1.6	2.31E-03	-1.2	9.39E-05	-0.9	7.65E-03	-0.9	7.65E-03	-0.9	1.02E-02	-1.1	8.74E-04	-0.1	1.0E+00	-0.1	1.0E+00	0.0	9.9E-01	-0.1	1.0E+00	0.0	9.9E-01	0.0	1.0E+00	0.1	5.8E-01	0.0	9.9E-01
38479	GAL3ST1	A_23_P120863	NM_004861	-1.2	1.10E-02	-0.8	3.39E-02	-1.3	1.10E-02	-1.2	1.42E-03	-0.5	1.93E-01	-0.4	5.05E-01	-0.5	1.93E-01	-0.9	1.30E-02	0.0	1.0E+00	-0.2	1.0E+00	0.0	1.0E+00	0.1	1.0E+00	-0.4	5.1E-01	-0.3	1.0E+00	-0.4	3.9E-01	-0.5	5.1E-01
17077	FBXL4	A_32_P67610	CR605444	-1.2	7.06E-04	-1.2	7.06E-04	-1.4	5.82E-03	-1.1	6.64E-04	-0.5	2.10E-01	-0.5	3.94E-01	-0.5	2.10E-01	-0.8	8.27E-02	-0.1	1.0E+00	-0.1	1.0E+00	-0.2	7.9E-01	0.0	1.0E+00	0.0	9.3E-01	0.0	1.0E+00	0.0	9.3E-01	-0.2	8.0E-01
4713	FLJ27365	A_32_P330000	AK023424	-1.2	1.45E-03	-0.9	7.05E-03	-1.5	1.45E-03	-1.2	7.19E-05	-0.9	7.13E-02	-0.9	7.13E-02	-0.9	5.75E-02	-1.0	1.28E-01	0.0	1.0E+00	-0.1	1.0E+00	0.0	9.7E-01	0.0	1.0E+00	0.7	5.2E-01	0.8	1.0E+00	0.3	5.2E-01	0.7	3.2E-01
36497	TARDBP	A_23_P403955	NM_007375	-1.2	7.41E-03	-1.3	1.36E-02	-1.2	7.41E-03	-0.8	2.25E-03	-1.3	2.84E-02	-1.3	2.84E-02	-1.4	9.26E-03	-1.3	5.87E-02	-0.3	7.1E-01	-0.5	6.6E-01	-0.3	7.1E-01	-0.2	1.0E+00	0.0	1.0E+00	-0.1	1.0E+00	0.0	9.7E-01	0.0	1.0E+00
16048	C1orf53	A_32_P210572	NM_001024594	-1.2	1.15E-02	-1.0	1.15E-02	-1.2	4.13E-02	-1.2	2.11E-03	-0.6	3.15E-02	-0.2	5.10E-01	-0.6	3.15E-02	-0.8	4.74E-03	-0.2	1.0E+00	-0.1	1.0E+00	-0.2	7.1E-01	-0.2	1.0E+00	-0.1	9.0E-01	0.0	1.0E+00	-0.1	7.3E-01	-0.1	9.0E-01
39026	GYLTL1B	A_23_P104617	NM_152312	-1.2	3.37E-03	-0.8	3.37E-03	-1.3	4.62E-03	-1.2	1.11E-04	-0.5	1.38E-01	-0.5	1.88E-01	-0.5	1.38E-01	-0.6	4.28E-02	-0.1	1.0E+00	-0.1	1.0E+00	0.1	9.0E-01	-0.1	1.0E+00	0.1	8.9E-01	0.1	1.0E+00	0.1	8.9E-01	0.2	7.9E-01
19219	CEP290	A_23_P25463	NM_025114	-1.2	1.65E-02	-0.9	2.59E-02	-1.5	1.65E-02	-1.2	7.69E-03	-0.8	1.22E-01	-1.2	7.78E-02	-0.2	7.57E-01	-0.8	1.22E-01	-0.1	1.0E+00	0.1	1.0E+00	-0.1	8.6E-01	-0.2	1.0E+00	-0.1	8.9E-01	0.0	1.0E+00	-0.1	8.7E-01	-0.2	8.9E-01
34350	ZNF658B	A_23_P419202	NM_001032297	-1.2	1.74E-04	-1.2	1.74E-04	-0.9	9.53E-03	-1.3	5.22E-05	-0.9	8.56E-02	-0.9	8.56E-02	-0.5	2.82E-01	-1.1	1.37E-02	-0.1	1.0E+00	-0.1	1.0E+00	-0.1	8.2E-01	-0.2	1.0E+00	0.0	9.7E-01	0.2	1.0E+00	-0.2	7.0E-01	0.0	9.7E-01
9909	DHFRL1	A_24_P186065	NM_176815	-1.2	9.33E-04	-1.2	9.33E-04	-1.2	1.07E-02	-1.1	4.28E-04	-0.7	1.70E-01	-0.7	1.70E-01	-0.5	2.44E-01	-0.8	3.96E-02	-0.3	1.0E+00	-0.4	1.0E+00	-0.3	7.1E-01	-0.2	1.0E+00	-0.3	3.7E-01	-0.3	1.0E+00	-0.3	1.4E-01	-0.3	3.7E-01
6074	BACE2	A_23_P154875	NM_012105	-1.2	4.72E-04	-1.2	4.72E-04	-1.3	1.78E-03	-1.1	1.52E-04	-1.1	4.14E-02	-1.1	4.14E-02	-0.6	2.39E-01	-1.2	1.92E-02	0.0	1.0E+00	0.0	1.0E+00	0.1	8.2E-01	-0.1	1.0E+00	-0.2	6.5E-01	-0.1	1.0E+00	-0.2	5.9E-01	-0.3	6.5E-01
36916	NKD1	A_23_P429449	NM_033119	-1.2	8.50E-04	-1.2	8.50E-04	-1.9	9.11E-04	-1.2	1.11E-04	-0.8	2.41E-02	-0.8	2.69E-02	-1.0	1.35E-02	-0.7	2.41E-02	-0.2	1.0E+00	-0.2	1.0E+00	-0.2	7.7E-01	-0.2	1.0E+00	-0.2	9.8E-01	-0.2	1.0E+00	-0.2	7.7E-01	0.0	9.8E-01
12220	C20orf200	A_23_P432506	NM_152757	-1.2	8.45E-03	-1.2	8.45E-03	-1.5	1.84E-02	-1.2	2.33E-03	-0.8	1.60E-01	-0.8	1.75E-01	-1.1	7.24E-02	-0.7	1.60E-01	0.0	1.0E+00	0.0	1.0E+00	-0.2	7.7E-01	0.1	1.0E+00	-0.2	8.3E-01	-0.1	1.0E+00	-0.3	5.7E-01	-0.2	8.3E-01
1841	MRPL42	A_24_P352445	NM_172178	-1.2	3.43E-04	-1.1	3.43E-04	-1.5	3.69E-04	-1.2	2.08E-05	-1.0	3.08E-02	-0.7	3.08E-02	-1.1	1.52E-01	-1.0	9.30E-04	-0.1	1.0E+00	-0.1	1.0E+00	0.0	9.3E-01	-0.1	1.0E+00	-0.1	7.8E-01	0.0	1.0E+00	-0.1	7.8E-01	-0.2	6.7E-01
6352	BC030084	A_24_P385012	BC030084	-1.2	1.48E-03	-1.2	9.83E-04	-1.4	7.16E-03	-1.1	1.48E-03	-0.8	6.51E-02	-0.7	6.51E-02	-0.9	1.57E-02	-0.8	1.01E-01	0.0	1.0E+00	-0.2	1.0E+00	0.0	9.6E-01	0.1	1.0E+00	0.0	1.0E+00	-0.1	1.0E+00	0.0	1.0E+00	0.2	9.1E-01
22481	GIN1	A_23_P110611	NM_017676	-1.2	9.63E-03	-1.1	9.63E-03	-1.7	1.68E-02	-1.2	3.82E-03	-1.0	1.54E-02	-0.7	1.03E-01	-1.1	1.54E-02	-1.0	6.42E-03	-0.1	1.0E+00	-0.1	1.0E+00	-0.1	7.6E-01	-0.1	1.0E+00	0.0	9.3E-01	0.1	1.0E+00	0.0	9.3E-01	-0.1	9.2E-01
30831	LIG3	A_23_P501134	NM_002311	-1.2	8.04E-03	-0.9	4.09E-02	-1.8	8.04E-03	-1.2	2.13E-03	-0.6	2.32E-01	-0.4	6.65E-01	-0.9	1.76E-01	-0.6	2.32E-01	0.0	1.0E+00	0.0	1.0E+00	-0.1	8.6E-01	0.1	1.0E+00	0.1	9.1E-01	0.1	1.0E+00	0.1	9.0E-01	0.2	9.1E-01
10651	LOC644563	A_24_P127121	XR_016572	-1.2	5.01E-04	-1.1	2.46E-03	-2.1	5.01E-04	-1.2	9.71E-05	-1.1	2.18E-02	-0.6	8.23E-02	-1.2	2.18E-02	-1.1	7.43E-03	-0.1	1.0E+00	-0.1	1.0E+00	-0.1	7.4E-01	-0.1	1.0E+00	0.2	8.1E-01	-0.1	1.0E+00	0.2	6.0E-01	0.2	8.1E-01
42342	AI267321	A_32_P209104	AI267321	-1.2	1.09E-02	-1.2	3.86E-03	-1.5	1.09E-02	-0.7	2.04E-02	-0.1	9.21E-01	0.0	9.89E-01	-0.4	4.92E-01	-0.1	9.21E-01	0.1	1.0E+00	0.1	1.0E+00	-0.1	9.4E-01	0.3	1.0E+00	-0.2	8.6E-01	0.1	1.0E+00	-0.3	3.4E-01	-0.2	8.6E-01
30510	THC2746310	A_32_P185029	THC2746310	-1.2	9.43E-04	-1.2	9.43E-04	-1.5	3.58E-03	-1.2	3.28E-04	-0.8	1.72E-02	-0.4	3.12E-01	-0.8	1.72E-02	-0.9	9.85E-03	-0.2	1.0E+00	-0.2	1.0E+00	-0.3	7.1E-01	-0.1	1.0E+00	-0.1	8.6E-01	-0.1	1.0E+00	0.1	8.6E-01	-0.2	8.5E-01
28337	EDAR	A_23_P120281	NM_022336	-1.2	1.52E-02	-1.2	2.52E-02	-1.6	6.45E-03	-0.7	1.52E-02	-0.8	3.56E-01	-0.6	3.62E-01	-1.2	3.56E-01	-0.8	1.22E-01	-0.1	1.0E+00	-0.2	1.0E+00	-0.1	7.1E-01	0.1	1.0E+00	-0.1	9.4E-01	-0.2	1.0E+00	-0.1	8.5E-01	-0.1	9.4E-01
13680	TATDN1	A_23_P254978	NM_032026	-1.2	3.32E-04	-1.0	3.32E-04	-1.9	6.65E-04	-1.2	3.71E-05	-0.9	2.38E-02	-0.4	3.41E-01	-1.3	2.38E-02	-0.9	5.46E-03	-0.1	1.0E+00	-0.1	1.0E+00	-0.1	8.9E-01	0.0	1.0E+00	0.0	9.2E-01	0.0	1.0E+00	0.1	8.8E-01	-0.1	9.2E-01
3776	ATF2	A_23_P209879	AK128731	-1.2	1.33E-03	-1.2	9.33E-04	-1.8	1.33E-03	-0.7	3.43E-03	-1.3	8.77E-02	-1.3	3.98E-02	-1.3	8.77E-02	-0.9	1.19E-01	-0.1	1.0E+00	-0.5	1.0E+00	-0.1	8.7E-01	0.0	1.0E+00	-0.4	8.6E-01	-0.4	1.0E+00	-0.1	8.6E-01	-0.4	7.0E-01
19966	NOTCH1	A_23_P60387	NM_017617	-1.2	5.47E-03	-1.1	8.35E-03	-1.3	5.47E-03	-1.2	2.93E-04	-0.7	2.35E-01	-0.7	2.35E-01	-1.0	2.52E-01	-0.7	1.65E-01	-0.1	1.0E+00	-0.1	1.0E+00	-0.2	7.1E-01	0.0	1.0E+00	0.1	9.5E-01	-0.1	1.0E+00	0.1	9.5E-01	0.2	9.0E-01
27434	ADCK1	A_23_P25945	NM_020421	-1.2	3.99E-03	-1.1	3.99E-03	-1.3	5.59E-03	-1.2	2.15E-04	-0.5	3.30E-01	-0.3	6.63E-01	-0.7	3.30E-01	-0.5	2.55E-01	0.0	1.0E+00	0.0	1.0E+00	-0.1	8.2E-01	0.0	1.0E+00	0.2	8.7E-01	0.1	1.0E+00	0.2	6.6E-01	0.2	8.7E-01
23754	LOC647946	A_32_P72181	NR_024391	-1.2	1.53E-02	-1.1	1.53E-02	-1.3	3.41E-02	-1.2	2.76E-03	-0.9	1.01E-01	-0.7	2.20E-01	-0.9	1.01E-01	-0.9	2.67E-02	-0.2	1.0E+00	-0.2	1.0E+00	-0.2	7.1E-01	0.0	1.0E+00	-0.1	8.8E-01	0.0	1.0E+00	-0.1	8.8E-01	-0.4	6.6E-01

Feature	Gene symbol	Probe	Transcript	72 hour								48 hours								24 hours								10 hours							
				siCASP8AP2								siCASP8AP2								siCASP8AP2								siCASP8AP2							
				Median	Median	1	1	3	3	6	6	Median	Median	1	1	3	3	6	6	Median	Median	1	1	3	3	6	6	Median	Median	1	1	3	3	6	6
				M	q	M	q	M	q	M	q	M	q	M	q	M	q	M	q	M	q	M	q	M	q	M	q	M	q	M	q	M	q	M	q
25701	LOC283859	A_24_P304881	AK000276	-1.2	2.02E-03	-1.1	4.15E-03	-1.6	2.02E-03	-1.2	1.44E-04	-0.8	5.13E-02	0.0	9.56E-01	-1.0	5.13E-02	-0.8	4.66E-02	-0.3	1.0E+00	-0.3	1.0E+00	-0.3	7.3E-01	0.1	1.0E+00	-0.2	8.8E-01	-0.2	1.0E+00	-0.2	6.0E-01	-0.1	8.8E-01
12308	CALD1	A_24_P921366	NM_033138	-1.2	3.51E-03	-1.2	3.51E-03	-2.8	6.65E-04	-0.8	6.50E-03	-0.9	8.84E-02	-0.5	2.96E-01	-2.3	1.23E-03	-0.9	8.84E-02	-0.3	1.0E+00	-0.3	1.0E+00	-0.9	1.7E-02	0.0	1.0E+00	-0.1	9.7E-01	-0.1	1.0E+00	-0.4	3.3E-01	0.0	9.7E-01
29502	TMEM109	A_24_P47467	NM_024092	-1.2	1.67E-03	-1.2	1.67E-03	-1.4	3.41E-03	-0.9	1.07E-03	-0.6	7.74E-02	-0.5	8.43E-02	-0.6	7.74E-02	-0.9	1.68E-02	0.1	1.0E+00	-0.2	1.0E+00	0.1	8.6E-01	0.2	1.0E+00	0.0	9.8E-01	-0.1	1.0E+00	0.0	9.6E-01	0.0	9.8E-01
20125	ZNF658B	A_32_P220739	NM_001032297	-1.2	1.55E-03	-1.2	1.55E-03	-1.3	1.23E-02	-1.1	8.74E-04	-0.5	1.90E-01	-0.4	4.58E-01	-0.5	1.90E-01	-0.8	4.49E-02	-0.2	1.0E+00	-0.2	1.0E+00	-0.3	7.1E-01	0.0	1.0E+00	-0.1	6.3E-01	-0.1	1.0E+00	-0.1	6.3E-01	-0.2	5.9E-01
34300	TAF1A	A_23_P74663	NM_005681	-1.2	1.38E-02	-0.7	1.49E-02	-1.2	1.38E-02	-1.2	6.77E-04	-0.6	1.28E-01	-0.6	1.28E-01	-0.4	3.78E-01	-0.7	2.49E-02	-0.1	1.0E+00	-0.1	1.0E+00	-0.1	9.0E-01	0.0	1.0E+00	0.1	9.6E-01	0.1	1.0E+00	0.1	7.4E-01	0.0	9.6E-01
34679	SLC7A5	A_23_P3792	NM_003486	-1.2	2.08E-02	-1.2	8.57E-03	-1.4	2.08E-02	-0.8	2.53E-02	-1.0	1.37E-02	-1.4	6.52E-03	-0.5	1.76E-01	-1.0	1.37E-02	0.1	1.0E+00	0.0	1.0E+00	0.4	7.1E-01	0.1	1.0E+00	0.3	7.6E-01	0.3	1.0E+00	0.2	7.6E-01	0.5	5.4E-01
35306	MRPL30	A_24_P257201	NM_145213	-1.2	1.65E-03	-0.9	1.65E-03	-1.3	3.76E-03	-1.2	9.43E-05	-0.6	3.58E-02	-0.7	3.58E-02	-0.3	7.63E-01	-0.6	1.63E-02	-0.3	1.0E+00	-0.1	1.0E+00	-0.3	7.1E-01	-0.3	1.0E+00	0.0	9.5E-01	0.0	1.0E+00	-0.1	9.5E-01	0.2	9.0E-01
39759	MTERFD1	A_24_P243834	NM_015942	-1.2	6.51E-03	-0.9	6.51E-03	-1.5	8.45E-03	-1.2	1.53E-03	-0.9	1.55E-01	-0.4	1.72E-01	-0.9	1.55E-01	-0.9	4.54E-03	-0.1	1.0E+00	-0.2	1.0E+00	-0.1	9.1E-01	-0.1	1.0E+00	0.0	8.3E-01	0.0	1.0E+00	0.2	7.5E-01	-0.3	8.3E-01
22378	STAU2	A_24_P374634	AK002152	-1.2	2.18E-03	-1.2	2.18E-03	-1.0	1.37E-02	-1.2	8.81E-05	-0.6	1.55E-01	-0.6	1.18E-01	-0.3	6.78E-01	-0.8	1.55E-01	0.0	1.0E+00	-0.1	1.0E+00	0.0	9.3E-01	0.1	1.0E+00	-0.1	9.8E-01	-0.2	1.0E+00	0.0	9.7E-01	-0.1	9.8E-01
26438	METTL9	A_23_P206347	NM_016025	-1.2	3.90E-03	-1.1	4.79E-03	-1.7	3.90E-03	-1.2	1.62E-03	-0.8	2.02E-02	-0.7	1.72E-01	-1.1	7.16E-03	-0.8	2.02E-02	-0.4	1.0E+00	-0.1	1.0E+00	-0.6	7.0E-01	-0.4	1.0E+00	-0.2	8.9E-01	-0.2	1.0E+00	-0.2	7.6E-01	-0.2	8.9E-01
38015	NUDT19	A_32_P93391	NM_001105570	-1.2	2.64E-04	-1.1	3.21E-02	-2.0	2.64E-04	-1.2	2.97E-05	-0.5	2.85E-01	-0.5	5.80E-01	-1.2	2.85E-01	-0.5	6.43E-02	0.1	1.0E+00	0.0	1.0E+00	0.2	8.4E-01	0.1	1.0E+00	0.0	9.9E-01	0.2	1.0E+00	0.0	9.8E-01	0.0	9.9E-01
37338	C14orf139	A_23_P14302	BC008299	-1.2	2.45E-02	-1.1	2.45E-02	-1.4	2.55E-02	-1.2	2.28E-03	-1.0	1.53E-02	-1.0	1.53E-02	-0.9	2.83E-02	-1.2	9.22E-04	0.0	1.0E+00	0.0	1.0E+00	-0.1	7.3E-01	0.0	1.0E+00	-0.2	8.1E-01	-0.1	1.0E+00	-0.2	5.6E-01	-0.2	8.1E-01
35586	ZMYM1	A_24_P53985	NM_024772	-1.2	1.40E-03	-1.2	1.40E-03	-1.5	3.24E-03	-1.0	7.10E-04	-1.0	1.10E-01	-0.7	2.32E-01	-1.1	1.10E-01	-1.0	2.31E-02	0.0	1.0E+00	0.1	1.0E+00	-0.2	7.5E-01	0.0	1.0E+00	-0.2	6.3E-01	-0.2	1.0E+00	-0.2	6.3E-01	-0.5	5.0E-01
16985	NOTCH1	A_23_P60387	NM_017617	-1.2	6.49E-03	-1.1	7.09E-03	-1.3	6.49E-03	-1.2	2.47E-04	-0.7	2.80E-01	-0.6	2.92E-01	-0.8	2.80E-01	-0.7	1.38E-01	0.0	1.0E+00	0.0	1.0E+00	-0.1	7.6E-01	0.0	1.0E+00	0.1	9.2E-01	-0.1	1.0E+00	0.1	9.2E-01	0.3	8.8E-01
25928	PTBP2	A_23_P46396	NM_021190	-1.2	1.11E-02	-1.2	1.72E-03	-1.2	2.07E-02	-0.8	1.11E-02	-0.5	3.14E-01	-0.5	3.14E-01	-0.5	4.93E-01	-0.7	1.24E-02	0.1	1.0E+00	-0.2	1.0E+00	0.1	8.8E-01	0.1	1.0E+00	-0.1	8.8E-01	-0.1	1.0E+00	-0.1	8.8E-01	-0.2	8.1E-01
34670	PPFIBP1	A_23_P337917	NM_177444	-1.2	6.48E-03	-1.1	7.39E-03	-1.8	6.48E-03	-1.2	2.97E-03	-0.8	1.53E-01	-1.1	1.34E-01	-0.5	5.52E-01	-0.8	1.53E-01	-0.2	1.0E+00	0.4	1.0E+00	-0.2	7.9E-01	-0.3	1.0E+00	-0.1	9.4E-01	0.1	1.0E+00	-0.1	9.0E-01	-0.2	9.4E-01
36081	GTF2H2D	A_24_P274640	NM_001042490	-1.2	1.12E-02	-1.0	1.23E-02	-1.7	1.12E-02	-1.2	3.10E-03	-0.8	1.69E-01	-0.5	1.69E-01	-1.1	2.10E-01	-0.8	1.89E-02	0.1	1.0E+00	-0.1	1.0E+00	0.1	8.7E-01	0.1	1.0E+00	0.0	9.9E-01	0.0	1.0E+00	0.2	7.9E-01	0.0	9.9E-01
35951	NUDT4	A_23_P2366	NM_199040	-1.2	6.47E-03	-1.2	6.47E-03	-2.3	1.26E-03	-0.8	9.01E-03	-0.9	2.17E-02	-0.9	1.84E-02	-1.4	1.00E-01	-0.8	2.17E-02	0.0	1.0E+00	-0.2	1.0E+00	0.0	9.9E-01	0.1	1.0E+00	-0.2	8.5E-01	-0.2	1.0E+00	-0.1	8.5E-01	-0.2	8.3E-01
33761	GTPBP6	A_24_P358381	NM_012227	-1.2	1.03E-03	-0.9	3.54E-02	-1.7	1.03E-03	-1.2	2.06E-04	-0.3	4.44E-01	-0.3	4.09E-01	-0.2	6.33E-01	-0.3	4.44E-01	0.1	1.0E+00	0.1	1.0E+00	0.2	8.1E-01	0.1	1.0E+00	0.1	9.3E-01	0.0	1.0E+00	0.2	7.4E-01	0.1	9.3E-01
2090	SLC7A5	A_24_P335620	NM_003486	-1.2	4.26E-03	-1.4	4.26E-03	-1.0	1.13E-02	-1.2	8.44E-05	-1.0	3.16E-02	-1.3	4.63E-03	-0.7	6.53E-02	-1.0	3.16E-02	-0.1	1.0E+00	-0.2	1.0E+00	0.0	1.0E+00	-0.1	1.0E+00	0.1	9.3E-01	-0.3	1.0E+00	0.1	9.0E-01	0.1	9.3E-01
12202	LRP8	A_23_P200222	NM_033300	-1.2	5.22E-03	-1.2	2.13E-02	-1.5	5.22E-03	-0.8	4.30E-03	-0.8	3.52E-02	-0.7	2.71E-02	-0.8	2.48E-01	-0.9	3.52E-02	-0.3	1.0E+00	-0.3	1.0E+00	-0.4	7.1E-01	0.0	1.0E+00	0.1	8.9E-01	-0.2	1.0E+00	0.1	8.9E-01	0.2	8.4E-01
35195	ABCB10	A_23_P201918	NM_012089	-1.2	4.15E-03	-1.0	4.15E-03	-1.6	4.84E-03	-1.2	1.09E-03	-0.7	1.72E-01	-0.4	2.42E-01	-1.0	1.72E-01	-0.7	2.29E-02	-0.1	1.0E+00	-0.2	1.0E+00	0.0	9.9E-01	-0.1	1.0E+00	0.0	9.8E-01	-0.1	1.0E+00	0.1	8.6E-01	0.0	9.8E-01
10910	THC2730125	A_32_P196233	THC2730125	-1.2	9.53E-03	-1.2	7.30E-03	-1.5	2.67E-02	-1.0	9.53E-03	-0.6	3.02E-01	-0.5	5.19E-01	-0.8	2.04E-01	-0.6	3.02E-01	0.0	1.0E+00	0.0	1.0E+00	-0.2	8.3E-01	0.3	1.0E+00	0.0	1.0E+00	0.1	1.0E+00	0.0	1.0E+00	0.0	9.9E-01
34606	OSGEPL1	A_23_P210081	NM_022353	-1.2	5.13E-03	-0.9	5.13E-03	-1.3	8.97E-03	-1.2	6.43E-04	-0.6	7.65E-02	-0.5	1.77E-01	-0.7	7.65E-02	-0.6	2.56E-02	-0.2	1.0E+00	-0.2	1.0E+00	-0.3	7.1E-01	-0.1	1.0E+00	0.0	9.7E-01	0.1	1.0E+00	0.0	9.7E-01	-0.3	6.2E-01
2308	THC2689427	A_24_P485742	THC2689427	-1.2	1.63E-03	-1.2	1.63E-03	-0.9	2.37E-02	-1.2	4.90E-04	-0.8	1.08E-01	-0.8	1.08E-01	-0.5	4.27E-01	-1.2	1.07E-02	-0.1	1.0E+00	0.3	1.0E+00	-0.1	9.5E-01	-0.1	1.0E+00	0.0	9.9E-01	0.2	1.0E+00	-0.1	8.8E-01	0.0	9.9E-01
24402	ATG10	A_23_P92824	NM_031482	-1.2	2.98E-04	-1.0	2.98E-04	-1.3	9.48E-04	-1.2	6.79E-06	-0.5	3.58E-01	-0.4	3.89E-01	-0.5	3.58E-01	-0.6	1.05E-01	0.0	1.0E+00	0.0	1.0E+00	-0.1	8.9E-01	0.1	1.0E+00	0.0	9.3E-01	0.2	1.0E+00	0.0	9.3E-01	-0.1	9.0E-01
38155	AL831999	A_32_P161455	AL831999	-1.2	1.89E-02	-1.2	1.20E-02	-1.3	2.59E-02	-0.8	1.89E-02	-0.6	1.09E-01	-0.6	5.53E-02	-1.0	1.09E-01	-0.6	3.23E-01	-0.2	1.0E+00	-0.2	1.0E+00	-0.6	7.1E-01	-0.1	1.0E+00	-0.2	4.4E-01	-0.2	1.0E+00	-0.2	2.9E-01	-0.5	4.4E-01
1242	NAE1	A_23_P77459	NM_001018159	-1.2	2.86E-04	-1.2	2.86E-04	-1.7	1.10E-03	-1.1	1.62E-04	-0.9	1.36E-02	-0.6	6.26E-02	-0.9	1.36E-02	-1.0	4.46E-03	-0.1	1.0E+00	0.0	1.0E+00	-0.1	8.9E-01	-0.1	1.0E+00	0.1	9.2E-01	0.0	1.0E+00	0.1	8.0E-01	0.1	9.2E-01
40495	DHPS	A_23_P501887	NM_013407	-1.2	4.16E-03	-1.1	5.36E-03	-1.7	4.16E-03	-1.2	4.60E-04	-0.9	7.37E-02	-0.5	1.84E-01	-1.3	7.37E-02	-0.9	2.70E-02	-0.2	9.7E-01	-0.1	1.0E+00	-0.2	7.1E-01	-0.4	9.7E-01	0.0	9.0E-01	-0.1	1.0E+00	0.0	9.0E-01	0.2	6.8E-01
10260	METAP2	A_24_P141736	NM_006838	-1.2	1.07E-02	-1.3	7.46E-03	-1.2	2.17E-02	-0.7	1.07E-02	-0.9	3.27E-01	-0.9	5.22E-02	-1.0	3.27E-01	-0.7	4.76E-01	0.0	1.0E+00	-0.4	1.0E+00	0.0	9.7E-01	0.1	1.0E+00	0.0	9.9E-01	-0.1	1.0E+00	0.0	9.9E-01	0.0	9.7E-01
8050	ZCCHC4	A_24_P248863	NM_024936	-1.2	7.97E-03	-1.1	8.92E-03	-1.5	7.97E-03	-1.2	7.09E-03	-0.7	1.31E-01	-0.7	1.31E-01	-0.7	1.46E-01	-0.9	6.39E-02	-0.3	1.0E+00	-0.3	1.0E+00	-0.3	7.1E-01	-0.1	1.0E+00	-0.1	9.8E-01	-0.1	1.0E+00	0.0	9.8E-01	-0.1	9.5E-01
32602	PYCR1	A_23_P130194	NM_006907	-1.2	2.69E-03	-0.6	1.94E-02	-1.2	2.69E-03	-1.2	3.88E-05	-0.1	8.77E-01	0.0	9.41E-01	-0.1	8.77E-01	-0.4	2.24E-01	0.1	1.0E+00	0.0	1.0E+00	0.1	8.1E-01	0.1	1.0E+00	0.1	7.5E-01	0.0	1.0E+00	0.1	5.2E-01	0.1	7.5E-01
43907	ZZZ3	A_23_P11507	NM_015534	-1.2	6.13E-03	-1.0	6.13E-03	-1.5	9.21E-03	-1.2	4.54E-03	-0.7	1.51E-01	-0.5	3.23E-01	-1.2	1.51E-01	-0.7	4.70E-02	-0.2	1.0E+00	-0.2	1.0E+00	-0.4	7.1E-01	-0.2	1.0E+00	0.0	9.8E-01	0.0	1.0E+00	0.0	9.8E-01	-0.2	8.2E-01
19301	DPH5	A_24_P385376	NM_001077395	-1.1	1.10E-03	-0.6	6.97E-03	-1.2	1.10E-03	-1.1	2.59E-04	-0.5	5.64E-01	-0.1	9.10E-01	-0.5	5.64E-01	-0.6	1.10E-01	0.1	1.0E+00	0.0	1.0E+00	0.1	8.4E-01	0.1	1.0E+00	0.0	9.1E-01	0.0	1.0E+00	0.1	9.1E-01	-0.2	8.6E-01
25439	BX113895	A_32_P69076	BX113895	-1.1	1.34E-03	-1.1	1.34E-03	-1.4	7.38E-03	-1.1	6.67E-04	-0.7	2.11E-01	-0.4	6.49E-01	-0.9	5.59E-02	-0.7	2.11E-01	-0.1	1.0E+00	0.0	1.0E+00	-0.5	7.1E-01	-0.1	1.0E+00	-0.1	8.8E-01	-0.1	1.0E+00	-0.1	8.7E-01	-0.1	8.8E-01
34896	NHLRC2	A_23_P46769	AK126751	-1.1	1.25E-02	-1.1	9.60E-03	-1.8	1.25E-02	-1.0	1.58E-02	-0.7	2.25E-01	-0.5	5.16E-01	-1.0	2.25E-01	-0.7	1.76E-01	0.0	1.0E+00	0.0	1.0E+00	-0.3	7.5E-01	0.0	1.0E+00	-0.1	8.9E-01	-0.1	1.0E+00	-0.1	8.9E-01	-0.3	7.0E-01
37143	SFRS12IP1	A_32_P198330	NM_173829	-1.1	7.24E-04	-1.1	5.19E-04	-1.9	7.24E-04	-0.7	2.74E-03	-0.5	4.98E-01	-0.5	4.98E-01	-0.7	3.86E-01	-0.3	7.63E-01	-0.1	1.0E+00	-0.1	1.0E+00	-0.1	8.7E-01	0.2	1.0E+00	-0.3	7.8E-01	-0.3	1.0E+00	-0.2	7.4E-01	-0.3	7.8E-01
38936	BC032027	A_24_P93931	BC032027	-1.1	5.67E-04	-1.1	5.67E-04	-1.1	5.18E-03	-1.1	6.63E-05	-0.7	1.54E-01	-0.3	6.43E-01	-0.7	1.54E-01	-0.8	6.22E-02	-0.1	1.0E+00	-0.1	1.0E+00	-0.3	7.1E-01	-0.1	1.0E+00	-0.2	3.8E-01	-0.1	1.0E+00	-0.2	3.3E-01	-0.3	3.8E-01
12961	CDH11	A_23_P152305	NM_001797	-1.1	1.51E-02	-0.8	2.13E-02	-1.1	1.51E-02	-1.3	3.11E-04	-0.7	1.73E-01	-0.4	2.93E-01	-0.8	1.73E-01	-0.7	2.67E-02	-0.2	1.0E+00	-0.2	1.0E+00	-0.2	7.1E-01	-0.2	1.0E+00	-0.2	6.5E-01	-0.2	1.0E+00	-0.2	6.0E-01	-0.3	6.5E-01
43500	CHDH	A_32_P73507	NM_018397	-1.1	6.65E-03	-1.1	6.65E-03	-1.2	2.63E-02	-1.0	2.21E-03	-0.9	1.79E-02	-0.9	1.79E-02	-0.5	2.97E-01	-1.0	8.50E-03	-0.2	1.0E+00	-0.2	1.0E+00	-0.2	7.1E-01	-0.1	1.0E+00	-0.1	9.1E-01	-0.1	1.0E+00	-0.1	9.1E-01	-0.1	9.1E-01
10001	CA313037	A_32_P57775	CA313037	-1.1	2.10E-03	-1.1	5.68E-03	-1.8	1.92E-03	-0.9	2.10E-03	-0.7	2.10E-01	-0.7	1.79E-01	-0.7	2.10E-01	-0.7	2.13E-01	-0.1	1.0E+00	0.2	1.0E+00	-0.2	7.9E-01	-0.1	1.0E+00	0.1	1.0E+00	0.1	1.0E+00	0.0	1.0E+00	0.1	9.2E-01
33231	NOTCH1	A_23_P60387	NM_017617	-1.1	9.60E-03	-1.1	9.60E-03	-1.4	1.15E-02	-1.1	1.32E-03	-0.8	1.88E-01	-0.7	3.25E-01	-1.1	1.88E-01	-0.8	1.58E-01	0.0	1.0E+00	0.0	1.0E+00	-0.1	8.6E-01	0.1	1.0E+00	0.0	9.9E-01	-0.2	1.0E+00	0.0	9.9E-01	0.1	9.6E-01
21185	PTAR1	A_24_P75158	NM_001099666	-1.1	3.73E-03	-1.7	2.72E-03	-1.1	1.85E-02	-0.8	3.73E-03	-1.2	1.27E-02	-1.3	5.24E-03	-0.8	1.27E-02	-1.2	2.84E-02	0.0	1.0E+00	-0.2	1.0E+00	0.0	9.7E-01	0.2	1.0E+00	-0.1	9.3E-01	-0.1	1.0E+00	0.1	6.0E-01	-0.1	9.3E-01
8193	RP11-138L21.1	A_24_P652609	AK054645	-1.1	3.12E-03	-0.9	3.12E-03	-1.1	6.80E-03	-1.2	1.01E-04	-0.4	2.68E-01	-0.3	6.53E-01	-0.4	2.68E-01	-0.6	1.14E-01	0.1	1.0E+00	0.1	1.0E+00	-0.1	8.8E-01	0.1	1.0E+00	-0.2	6.4E-01	-0.2	1.0E+00	-0.1	6.2E-01	-0.2	6.4E-01
38844	C6orf162	A_23_P59255	NM_001042493	-1.1	1.90E-03	-1.2	1.37E-03	-1.1	1.57E-02	-0.9	1.90E-03	-1.0	1.93E-02	-0.7	8.20E-02	-1.0	1.93E-02	-1.0	1.95E-03	0.0	1.0E+00	0.1	1.0E+00	-0.3	7.1E-01	0.0	1.0E+00	-0.3	6.6E-01	0.1	1.0E+00	-0.3	4.3E-01	-0.3	6.6E-01
43191	PPID	A_23_P386411	NM_005038	-1.1	3.52E-04	-0.8	6.80E-03	-2.2	3.52E-04	-1.1	1.34E-04	-0.6	2.93E-02	-0.3	2.62E-01	-1.1	2.93E-02	-0.6	1.91E-02	-0.1	1.0E+00	-0.1	1.0E+00	-0.2	7.3E-01	0.0	1.0E+00	-0.1	8.4E-01	-0.1	1.0E+00	-0.1	7.2E-01	-0.1	8.4E-01
27277	TSNAX	A_24_P148151	NM_005999	-1.1	7.56E-03	-1.6	5.19E-04	-1.1	8.25E-03	-0.6	7.56E-03	-1.2	2.71E-02	-1.4	3.64E-03	-1.0	5.82E-02	-1.2	2.71E-02	0.0	1.0E+00	-0.6	8.4E-01	0.0	1.0E+00	0.0	1.0E+00	-0.1	9.5E-01	-0.4	1.0E+00	-0.1	9.4E-01	-0.1	9.5E-01
36229	NANP	A_23_P427039	NM_152667	-1.1	1.40E-03	-0.7	7.33E-03	-1.2	1.40E-03	-1.1	3.70E-05	-0.5	1.19E-01	-0.5	1.19E-01	-0.4	1.34E-01	-0.7	3.87E-03	-0.2	1.0E+00	-0.2	1.0E+00	-0.1	8.4E-01	-0.2	1.0E+00	0.1	8.9E-01	0.1	1.0E+00	0.2	7.9E-01	-0.2	8.9E-01
28993	AK092810	A_24_P696507	AK092810	-1.1	2.37E-02	-1.1	1.11E-02	-1.3	2.37E-02	-1.1	3.15E-02	-0.5	5.42E-01	-0.1	8.93E-01	-0.5	5.42E-01	-0.5	3.91E-01	0.0	1.0E+00	0.0	1.0E+00	-0.1	9.1E-01	0.0	1.0E+00	-0.1	8.7E-01	-0.1	1.0E+00	-0.2	5.7E-01	-0.1	8.7E-01
40439	DLAT	A_23_P203030	NM_001931	-1.1	8.07E-03	-1.0	1.10E-02	-1.8	8.07E-03	-1.1	2.90E-03	-0.8	1.66E-01	-0.4	3.77E-01	-0.8	1.66E-01	-0.9	3.02E-02	0.0	1.0E+00	-0.1	1.0E+00	0.0	9.6E-01	0.0	1.0E+00	-0.2	6.3E-01	-0.1	1.0E+00	-0.2	6.3E-01	-0.4	4.2E-01
40313	SLC26A9	A_23_P201248	NM_052934	-1.1	1.89E-02	-1.1	1.89E-02	-1.3	3.84E-02	-1.0	6.87E-03	-1.2	3.61E-03	-1.2	3.85E-03	-1.2	1.53E-03	-1.2	3.61E-03	-0.1	1.0E+00	-0.1	1.0E+00	-0.5	6.0E-01	-0.1	1.0E+00	-0.4	6.8E-01	-0.1	1.0E+00	-0.4	5.2E-01	-0.4	6.8E-01
36961	METTL2B	A_23_P59836	NM_018396	-1.1	3.99E-03	-0.8	3.16E-03	-1.1	6.73E-03	-1.5	3.99E-03	-1.0	1.10E-01	-1.0	1.10E-01	-0.4	5.26E-01	-1.1	3.68E-02	0.0	1.0E+00	0.1	1.0E+00	0.0	9.6E-01	-0.4	1.0E+00	0.1	9.4E-01	0.2	1.0E+00	-0.2	7.8E-01	0.1	9.4E-01
21796	PIGK	A_24_P379512	NM_005482	-1.1	4.63E-03	-0.9	1.69E-02	-1.7	4.63E-03	-1.1	7.17E-04	-1.0	9.66E-02	-0.3	5.39E-01	-1.1	9.66E-02	-1.0	1.46E-02	-0.1	1.0E+00	-0.1	1.0E+00	0.0	9.1E-01	-0.1	1.0E+00	0.0	9.3E-01	0.0	1.0E+00	0.1	9.0E-01	-0.1	9.3E-01
4558	CR591289	A_32_P39431	CR591289	-1.1	2.30E-03	-1.1	1.11E-03	-1.2	1.33E-02	-0.9	2.30E-03	-0.7	6.91E-02	-0.6	6.91E-02	-0.7	9.25E-02	-0.8	1.18E-02	-0.2	1.0E+00	-0.2	1.0E+00	-0.3	7.1E-01	0.0	1.0E+00	-0.2	5.3E-01	-0.2	1.0E+00	-0.2	5.3E-01	-0.4	5.2E-01
33679	FZD3	A_23_P347468	NM_017412	-1.1	1.09E-02	-1.0	1.09E-02	-1.5	2.30E-02	-1.1	7.12E-03	-0.5	5.84E-01	-0.2	8.50E-01	-0.5	5.84E-01	-0.5	3.57E-01	0.0	1.0E+00	0.1	1.0E+00	-0.2	8.3E-01	0.0	1.0E+00	0.0	9.2E-01	0.0	1.0E+00	0.1	9.2E-01	-0.1	9.0E-01
30077	THC2509143	A_24_P928947	THC2509143	-1.1	6.69E-04	-1.1	6.69E-04	-0.9	1.21E-02	-1.2	3.35E-05	-0.8	1.44E-01	-0.8	1.44E-01	0.0	9.48E-01	-0.8	7.67E-02	0.0	1.0E+00	-0.3	1.0E+00	0.1	9.6E-01	0.0	1.0E+00	0.1	9.5E-01	0.2	1.0E+00	0.1	9.5E-01	0.1	9.5E-01
3192	BC013792	A_23_P333802	BC013792	-1.1	4.25E-03	-0.9	4.25E-03	-1.2	7.86E-03	-1.1	1.87E-04	-0.9	7.53E-02	-0.5	3.13E-01	-1.5	7.53E-02	-0.9	2.77E-02	-0.3	1.0E+00	0.0	1.0E+00	-0.3	3.4E-01	-0.3	1.0E+00	-0.3	6.5E-01	-0.1	1.0E+00	-0.3	4.2E-01	-0.3	6.5E-01
43977	NUBPL	A_23_P170908	NM_025152	-1.1	3.46E-04	-1.1	1.87E-04	-1.5	9.71E-04	-1.1	3.46E-04	-0.7	1.03E-01	-0.2	7.82E-01	-0.9	7.69E-02	-0.7	1.03E-01	0.0	1.0E+00	0.0	1.0E+00	-0.2	7.1E-01	0.0	1.0E+00	-0.2	6.1E-01	-0.1	1.0E+00	-0.2	2.7E-01	-0.2	6.1E-01
37277	ZZZ3	A_23_P11507	NM_015534	-1.1	1.13E-02	-1.1	1.13E-02	-1.6	1.87E-02	-1.1	6.36E-03	-0.7	1.02E-01	-0.6	2.43E-01	-1.2	1.02E-01	-0.7	4.71E-02	-0.2	1.0E+00	-0.2	1.0E+00	-0.3	7.4E-01	-0.1	1.0E+00	-0.2	8.5E-01	-0.1	1.0E+00	-0.2	7.5E-01	-0.2	8.5E-01
2129	THEM4	A_23_P149375	NM_053055	-1.1	8.05E-03	-1.1	2.33E-02	-1.3	8.05E-03	-1.0	7.18E-04	-0.7	1.88E-01	-0.7	2.26E-01	-0.7	1.88E-01	-0.7	7.20E-02	-0.1	1.0E+00	-0.1	1.0E+00	0.0	9.6E-01	-0.1	1.0E+00	0.0	9.3E-01	0.0	1.0E+00	-0.3	7.1E-01	0.1	9.3E-01
31796	DYNC2LI1	A_23_P502174	NM_015522	-1.1	1.53E-02	-1.0	1.76E-02	-1.5	1.53E-02	-1.1	2.37E-03	-0.6	3.78E-01	-0.4	6.22E-01	-0.6	3.78E-01	-0.6	1.28E-02	0.1	1.0E+00	0.1	1.0E+00	0.2	7.5E-01	0.1	1.0E+00	-0.1	9.3E-01	0.0	1.0E+00	-0.1	8.9E-01	-0.1	9.3E-01
35726	ZZZ3	A_23_P11507	NM_015534	-1.1	1.17E-02	-1.0	1.17E-02	-1.4	2.13E-02	-1.1	5.26E-03	-0.6	8.93E-02	-0.5	2.28E-01	-1.0	8.93E-02	-0.6	6.42E-02	-0.2	1.0E+00	-0.2	1.0E+00	-0.3	7.1E-01	-0.2	1.0E+00	0.0	9.9E-01	0.0	1.0E+00	0.0	9.9E-01	-0.1	9.6E-01
37329	MMACHC	A_24_P83586	NM_015506	-1.1	2.80E-03	-1.2	3.69E-03	-1.1	2.80E-03	-1.1	3.39E-05	-0.7	4.58E-02	-0.4	2.60E-01	-0.8	4.58E-02	-0.7	3.16E-02	-0.3	1.0E+00	-0.3	1.0E+00	-0.4	7.1E-01	-0.2	1.0E+00	0.1	9.3E-01	0.1	1.0E+00	0.1	9.2E-01	-0.1	9.3E-01
27099	TMEM48	A_23_P73982	NM_018087	-1.1	5.31E-03	-1.1	5.31E-03	-1.1	1.73E-02	-1.3	1.38E-04	-0.5	1.91E-01	-0.5	1.91E-01	-0.3	4.51E-01	-1.2	2.04E-03	-0.1	1.0E+00	-0.1	1.0E+00	-0.1	7.9E-01	-0.3	1.0E+00	-0.1	8.7E-01	-0.1	1.0E+00	-0.1	8.7E-01	-0.2	7.5E-01
1683	MRPL42	A_23_P25163	NM_172178	-1.1	3.65E-03	-0.9	3.65E-03	-1.1	8.81E-03	-1.1	2.44E-04	-0.6	1.79E-01	-0.6	1.79E-01	-0.5	1.83E-01	-0.6	3.42E-02	0.0	1.0E+00	0.0	1.0E+00	0.0	9.6E-01	-0.1	1.0E+00	0.0	9.7E-01	0.2	1.0E+00	-0.1	8.8E-01	0.0	9.7E-01
17416	CDAN1	A_23_P349127	NM_138477	-1.1	1.10E-03	-1.0	1.10E-03	-1.1	8.41E-03	-1.1	2.18E-04	-0.6	2.62E-01	-0.5	5.00E-01	-0.6	2.62E-01	-0.6	2.36E-01	0.0	1.0E+00	0.1	1.0E+00	-0.1	9.1E-01	0.0	1.0E+00	0.0	1.0E+00	0.1	1.0E+00	-0.1	7.4E-01	0.0	1.0E+00
12484	MMAB	A_23_P2537	NM_052845	-1.1	8.73E-04	-1.2	8.73E-04	-0.9	9.98E-03	-1.1	9.17E-05	-0.6	2.03E-01	-0.8	5.73E-02	-0.6	3.29E-01	-0.6	2.03E-01	0.0	1.0E+00	-0.1	1.0E+00	0.0	9.6E-01	0.1	1.0E+00	-0.1	9.6E-01	-0.1	1.0E+00	-0.1	6.8E-01	0.1	9.6E-01
26499	TMBIM4	A_23_P13701	NM_016056	-1.1	2.69E-03	-1.1	1.63E-02	-1.6	2.69E-03	-1.0	4.60E-04	-0.5	3.45E-01	-0.5	3.45E-01	-0.8	4.44E-01	-0.5	3.31E-02	0.0	1.0E+00	0.1	1.0E+00	0.0	9.9E-01	-0.1	1.0E+00	0.0	9.9E-01	0.2	1.0E+00	0.0	9.7E-01	0.0	9.9E-01
184	ATG5	A_23_P111381	NM_004849	-1.1	6.07E-03	-0.8	2.31E-02	-1.1	6.07E-03	-1.3	3.86E-05	-0.7	1.40E-01	-0.3	5.14E-01	-0.7	1.40E-01	-1.3	3.14E-03	-0.2	1.0E+00	-0.2	1.0E+00	0.0	9.3E-01	-0.2	1.0E+00	-0.1	9.3E-01	-0.1	1.0E+00	0.1	9.3E-01	-0.5	5.1E-01
43477	NOTCH1	A_23_P60387	NM_017617	-1.1	4.31E-03	-1.2	4.31E-03	-1.1	1.44E-02	-1.1	5.47E-04	-0.7	1.71E-01	-0.7	1.57E-01	-0.9	2.16E-01	-0.7	1.71E-01	0.0	1.0E+00	0.0	1.0E+00	-0.1	8.1E-01	0.0	1.0E+00	0.0	9.9E-01	-0.2	1.0E+00	0.0	9.9E-01	0.2	9.4E-01
30971	ZNF337	A_24_P318939	NM_015655	-1.1	1.63E-03	-1.0	1.63E-03	-1.5	2.82E-03	-1.1	1.63E-04	-0.8	9.48E-02	-0.5	4.22E-01	-1.1	7.53E-02	-0.8	9.48E-02	-0.1	1.0E+00	-0.1	1.0E+00	-0.4	7.1E-01	0.0	1.0E+00	0.2	7.1E-01	-0.1	1.0E+00	0.2	7.1E-01	0.4	6.0E-01
22447	FNTB	A_24_P124831	NM_002028	-1.1	3.21E-03	-0.7	2.22E-02	-1.5	3.21E-03	-1.1	1.79E-04	-0.3	6.56E-01	-0.3	6.56E-01	-0.2	7.07E-01	-0.4	3.15E-01	0.1	1.0E+00	0.1	1.0E+00	0.0	1.0E+00	0.2	1.0E+00	0.0	9.5E-01	0.0	1.0E+00	0.0	9.4E-01	0.1	9.5E-01
35588	PPFIBP1	A_24_P811704	NM_003622	-1.1	3.09E-02	-0.9	3.09E-02	-1.3	4.15E-02	-1.1	7.99E-03	-0.9	2.94E-02	-1.4	2.94E-02	-0.3	7.40E-01	-0.9	2.20E-02	-0.2	1.0E+00	0.3	1.0E+00	-0.2	8.8E-01	-0.4	1.0E+00	0.0	9.9E-01	0.0	1.0E+00	0.0	9.9E-01	-0.2	9.4E-01
18779	GSTA4	A_23_P110941	NM_001512	-1.1	1.63E-02	-0.7	4.40E-02	-1.5	1.63E-02	-1.1	2.20E-03	-0.9	1.20E-01	-0.4	4.50E-01	-1.2	1.20E-01	-0.9	1.81E-02	-0.1	1.0E+00	-0.2	1.0E+00	-0.1	8.3E-01	0.0	1.0E+00	-0.1	9.9E-01	-0.2	1.0E+00	-0.1	9.3E-01	0.0	9.9E-01
32135	PRKAA2	A_32_P94160	NM_006252	-1.1	1.25E-03	-1.1	2.59E-04	-1.2	3.99E-03	-0.8	1.25E-03	-0.5	3.43E-01	-0.4	4.03E-01	-0.5	3.43E-01	-0.6	2.14E-01	0.0	1.0E+00	-0.1	1.0E+00	0.1	9.4E-01	0.0	1.0E+00	-0.1	9.7E-01	-0.1	1.0E+00	0.0	9.7E-01	-0.2	6.0E-01
35252	CR591264	A_32_P204624	CR591264	-1.1	2.46E-03	-0.9	2.46E-03	-1.2	1.13E-02	-1.1	2.98E-04	-0.8	8.04E-02	-0.3	4.83E-01	-0.9	8.04E-02	-0.8	1.86E-02	-0.1	1.0E+00	-0.1	1.0E+00	-0.1	7.5E-01	0.1	1.0E+00	-0.1	6.0E-01	-0.1	1.0E+00	-0.1	6.0E-01	-0.3	3.9E-01
44478	NOTCH1	A_23_P60387	NM_017617	-1.1	8.00E-03	-1.1	8.00E-03	-1.1	1.23E-02	-1.1	6.59E-04	-0.7	1.67E-01	-0.7	1.67E-01	-0.8	2.55E-01	-0.7	1.06E-01	0.0	1.0E+00	0.0	1.0E+00	-0.2	7.5E-01	0.0	1.0E+00	0.0	1.0E+00	-0.1	1.0E+00	0.0	1.0E+00	0.2	8.9E-01
44573	CBWD5	A_24_P25080	NM_001024916	-1.1	3.25E-03	-1.1	2.28E-02	-2.1	1.33E-03	-0.9	3.25E-03	-0.6	2.73E-01	-0.5	2.73E-01	-0.8	3.98E-01	-0.6	3.26E-02	0.0	1.0E+00	0.1	1.0E+00	0.0	9.5E-01	0.0	1.0E+00	0.1	9.8E-01	0.1	1.0E+00	0.3	7.3E-01	0.1	9.8E-01
17342	RBBP9	A_23_P257538	NM_006606	-1.1	9.29E-03	-1.0	9.29E-03	-1.3	1.31E-02	-1.1	1.43E-03	-0.4	5.71E-01	-0.2	8.33E-01	-0.5	4.59E-01	-0.4	5.71E-01	-0.1	1.0E+00	-0.1	1.0E+00	-0.1	8.5E-01	0.1	1.0E+00	-0.2	8.2E-01	-0.2	1.0E+00	-0.2	6.9E-01	-0.3	8.2E-01
29065	SFRS1	A_24_P921933	NM_001078166	-1.1	2.63E-03	-1.6	2.63E-03	-1.1	2.38E-02	-1.1	1.44E-03	-0.9	1.59E-02	-1.0	9.51E-03	-0.8	1.59E-02	-0.9	5.72E-02	-0.2	7.8E-01	-0.4	7.8E-01	-0.2	7.1E-01	0.0	1.0E+00	-0.2	9.3E-01	-0.3	1.0E+00	0.0	9.3E-01	-0.2	8.9E-01
40136	NCAPH	A_23_P415443	NM_015341	-1.1	1.44E-02	-1.0	1.44E-02	-1.2	2.92E-02	-1.1	5.27E-03	-1.0	5.24E-03	-0.7	3.61E-02	-1.0	5.24E-03	-1.0	8.49E-04	-0.3	1.0E+00	-0.2	1.0E+00	-0.4	7.1E-01	-0.3	1.0E+00	-0.1	9.5E-01	0.0	1.0E+00	-0.1	8.1E-01	-0.1	9.5E-01
38890	LHFP	A_23_P88069	NM_005780	-1.1	2.17E-03	-1.1	1.53E-03	-1.1	1.13E-02	-0.8	2.17E-03	-0.6	1.02E-01	-0.6	1.02E-01	-0.4	3.07E-01	-0.8	3.66E-02	0.0	1.0E+00	-0.3	1.0E+00	0.0	9.8E-01	0.1	1.0E+00	-0.2	8.2E-01	-0.2	1.0E+00	-0.2	7.1E-01	-0.2	8.2E-01
29085	NOTCH1	A_23_P60387	NM_017617	-1.1	1.12E-02	-1.1	1.12E-02	-1.1	1.20E-02	-1.2	3.83E-04	-0.7	1.83E-01	-0.7	2.79E-01	-0.9	1.83E-01	-0.7	1.29E-01	0.0	1.0E+00	0.1	1.0E+00	-0.1	8.0E-01	0.0	1.0E+00	0.1	9.5E-01	-0.2	1.0E+00	0.1	9.5E-01	0.2	9.1E-01
34915	A_24_P254915	A_24_P254915	A_24_P254915	-1.1	1.25E-02	-0.8	2.10E-02	-1.3	1.25E-02	-1.1	6.97E-04	-0.3	7.83E-01	-0.2	7.99E-01	-0.3	7.83E-01	-0.5	2.85E-01	0.0	1.0E+00	0.1	1.0E+00	-0.3	7.3E-01	0.0	1.0E+00	0.0	9.6E-01	0.0	1.0E+00	-0.1	8.7E-01	0.0	9.6E-01
38486	THC2739249	A_32_P3527	THC2739249	-1.1	1.53E-03	-1.1	1.53E-03	-1.2	1.14E-02	-1.0	5.99E-04	-1.0	2.11E-02	-0.8	1.45E-01	-1.2	9.02E-03	-1.0	2.11E-02	-0.1	1.0E+00	-0.1	1.0E+00	-0.3	7.3E-01	-0.1	1.0E+00	-0.2	9.7E-01	-0.2	1.0E+00	0.0	9.7E-01	-0.2	7.3E-01
39562	CENPK	A_23_P155989	NM_022145	-1.1	1.22E-02	-1.1	1.22E-02	-1.9	3.57E-03	-0.8	1.59E-02	-1.0	1.34E-01	-0.9	1.34E-01	-1.3	2.20E-01	-1.0	4.20E-02	0.0	1.0E+00	-0.2	1.0E+00	0.1	9.5E-01	0.0	1.0E+00	-0.1	9.3E-01	-0.4	1.0E+00	0.2	8.0E-01	-0.1	9.3E-01
24237	PTGR2	A_23_P48713	NM_152444	-1.1	5.49E-03	-1.1	5.49E-03	-1.3	1.58E-02	-1.1	1.67E-03	-0.7	9.91E-02	-0.5	9.91E-02	-0.8	1.59E-01	-0.7	9.21E-03	0.0	1.0E+00	-0.1	1.0E+00	0.0	9.9E-01	0.1	1.0E+00	-0.2	7.0E-01	0.0	1.0E+00	-0.2	6.9E-01	-0.3	7.0E-01
9371	PFAS	A_23_P402610	NM_012393	-1.1	1.15E-03	-1.1	1.15E-03	-1.0	5.46E-03	-1.3	1.34E-05	-0.9	8.82E-02	-0.5	3.03E-01	-1.2	8.82E-02	-0.9	1.03E-02	-0.2	1.0E+00	0.0	1.0E+00	-0.3	7.1E-01	-0.2	1.0E+00	0.0	9.2E-01	0.0	1.0E+00	-0.1	8.8E-01	0.1	9.2E-01
8146	NOTCH1	A_23_P60387	NM_017617	-1.1	9.40E-03	-1.1	9.40E-03	-1.1	1.25E-02	-1.1	5.01E-04	-0.7	2.61E-01	-0.6	3.01E-01	-0.9	2.61E-01	-0.7	1.45E-01	-0.1	1.0E+00	0.0	1.0E+00	-0.2	7.2E-01	-0.1	1.0E+00	0.0	9.6E-01	-0.1	1.0E+00	0.0	9.6E-01	0.3	8.5E-01
11292	SVIP	A_24_P111242	NM_148893	-1.1	1.48E-03	-1.1	1.48E-03	-2.0	1.54E-03	-1.1	7.09E-04	-0.5	4.38E-01	0.0	9.76E-01	-0.5	4.38E-01	-0.5	2.90E-01	-0.1	1.0E+00	-0.1	1.0E+00	-0.1	8.9E-01	0.1	1.0E+00	-0.2	5.1E-01	-0.1	1.0E+00	-0.2	5.1E-01	-0.4	5.1E-01
7478	C6orf70	A_32_P129527	NM_018341	-1.1	1.67E-03	-1.1	1.67E-03	-0.9	2.78E-02	-1.3	9.29E-04	-0.5	2.28E-01	-0.5	2.28E-01	-0.5	2.59E-01	-1.1	2.15E-02	-0.2	1.0E+00	-0.3	1.0E+00	-0.2	7.3E-01	-0.2	1.0E+00	0.2	8.8E-01	0.0	1.0E+00	0.2	5.6E-01	0.2	8.8E-01
43310	TATDN1	A_32_P224911	NM_032026	-1.1	1.05E-03	-0.9	2.80E-03	-2.0	1.05E-03	-1.1	2.54E-04	-0.8	4.49E-02	-0.7	4.49E-02	-1.3	1.36E-01	-0.8	5.87E-03	-0.1	1.0E+00	-0.1	1.0E+00	-0.2	7.8E-01	0.0	1.0E+00	-0.1	9.7E-01	-0.1	1.0E+00	-0.1	9.0E-01	0.0	9.7E-01
24231	BU540282	A_32_P57013	BU540282	-1.1	4.83E-03	-0.9	7.44E-03	-1.8	2.07E-03	-1.1	4.83E-03	-0.9	1.32E-01	-0.7	1.38E-01	-0.9	1.32E-01	-0.9	2.09E-02	0.0	1.0E+00	0.0	1.0E+00	-0.2	8.2E-01	0.0	1.0E+00	0.1	9.5E-01	0.1	1.0E+00	-0.1	9.5E-01	0.1	9.4E-01
40189	SETD4	A_23_P211106	NM_017438	-1.1	3.22E-03	-1.0	3.22E-03	-1.1	1.62E-02	-1.3	1.58E-04	-0.7	1.81E-02	-0.7	2.26E-02	-0.7	1.81E-02	-1.0	7.06E-03	-0.1	1.0E+00	-0.1	1.0E+00	-0.1	7.4E-01	0.0	1.0E+00	0.1	8.7E-01	0.0	1.0E+00	0.1	6.6E-01	0.1	8.7E-01
1821	ANP32A	A_23_P3242	ENST00000409628	-1.1	2.04E-02	-0.9	2.26E-02	-1.5	2.04E-02	-1.1	6.66E-03	-0.6	9.32E-02	-0.4	2.81E-01	-0.6	9.32E-02	-0.7	1.56E-02	-0.2	1.0E+00	-0.1	1.0E+00	-0.2	8.0E-01	-0.2	1.0E+00	0.0	9.7E-01	0.0	1.0E+00	0.0	9.7E-01	-0.3	5.8E-01
9793	FAM172A	A_24_P111912	NM_032042	-1.1	1.56E-03	-1.1	1.56E-03	-0.9	2.64E-02	-1.1	4.08E-04	-0.6	1.92E-01	-0.6	1.92E-01	0.0	9.45E-01	-0.8	1.28E-01	-0.1	1.0E+00	-0.2	1.0E+00	-0.1	8.5E-01	0.1	1.0E+00	-0.3	4.0E-01	-0.1	1.0E+00	-0.3	4.0E-01	-0.4	3.6E-01
24179	DTWD1	A_23_P3215	AF168717	-1.1	9.83E-04	-1.0	9.83E-04	-1.5	3.16E-03	-1.1	2.09E-04	-0.9	7.34E-02	-0.6	2.49E-01	-1.0	7.34E-02	-0.9	1.84E-02	0.1	1.0E+00	0.1	1.0E+00	0.1	7.9E-01	0.1	1.0E+00	0.1	9.2E-01	0.2	1.0E+00	0.1	7.7E-01	-0.1	9.2E-01
27902	EXOSC8	A_23_P162822	NM_181503	-1.1	3.23E-03	-1.1	1.93E-02	-1.5	3.23E-03	-0.8	1.44E-03	-0.8	8.26E-02	-0.8	8.26E-02	-1.0	2.74E-01	-0.8	3.11E-02	0.2	1.0E+00	0.1	1.0E+00	0.2	7.3E-01	0.2	1.0E+00	0.0	9.9E-01	0.1	1.0E+00	0.0	9.9E-01	-0.1	9.1E-01
34204	KCTD20	A_23_P374351	NM_173562	-1.1	2.63E-03	-0.9	2.65E-03	-1.5	2.63E-03	-1.1	4.60E-04	-0.6	6.79E-02	-0.6	8.91E-02	-1.2	8.31E-03	-0.5	6.79E-02	-0.2	1.0E+00	-0.2	1.0E+00	-0.6	3.7E-01	-0.1	1.0E+00	-0.1	9.5E-01	-0.1	1.0E+00	-0.3	6.1E-01	0.1	9.5E-01
42835	ALG14	A_23_P257423	NM_144988	-1.1	9.06E-04	-1.1	9.06E-04	-1.1	4.85E-03	-1.1	9.93E-05	-0.6	7.80E-02	-0.4	1.12E-01	-0.6	7.80E-02	-0.9	1.56E-02	0.0	1.0E+00	0.0	1.0E+00	0.0	9.6E-01	0.1	1.0E+00	-0.1	8.9E-01	0.0	1.0E+00	-0.1	8.9E-01	-0.2	7.1E-01
6744	TTC1	A_23_P19164	NM_003314	-1.1	1.10E-03	-0.9	1.10E-03	-1.1	5.15E-03	-1.1	2.64E-04	-0.7	1.65E-01	-0.7	1.65E-01	-0.3	5.18E-01	-0.9	1.98E-02	-0.1	1.0E+00	-0.1	1.0E+00	0.0	9.9E-01	-0.3	1.0E+00	-0.2	6.1E-01	-0.2	1.0E+00	-0.2	6.1E-01	-0.5	2.1E-01
42069	THC2672257	A_32_P218671	THC2672257	-1.1	6.40E-03	-1.1	1.09E-02	-1.4	4.10E-03	-0.7	6.40E-03	-0.4	6.31E-01	-0.4	6.01E-01	-0.5	6.31E-01	-0.4	6.81E-01	0.2	1.0E+00	0.2	1.0E+00	0.0	1.0E+00	0.3	1.0E+00	-0.1	8.9E-01	0.1	1.0E+00	-0.1	8.9E-01	-0.3	7.1E-01
18230	ELOVL6	A_32_P80597	ENST00000394607	-1.1	3.91E-03	-1.2	3.91E-03	-1.1	1.64E-02	-0.9	1.74E-03	-0.7	1.36E-01	-0.9	6.50E-02	-0.4	4.60E-01	-0.7	1.36E-01	-0.1	8.6E-01	-0.7	4.0E-02	-0.1	8.6E-01	0.2	1.0E+00	-0.3	8.1E-01	-0.4	1.0E+00	-0.3	8.1E-01	-0.3	8.1E-01
3732	FASTKD1	A_23_P28590	NM_024622	-1.1	1.22E-04	-0.8	4.68E-04	-2.0	1.22E-04	-1.1	9.14E-06	-0.8	3.94E-02	-0.8	1.03E-01	-1.2	3.94E-02	-0.8	1.57E-02	-0.3	1.0E+00	-0.2	1.0E+00	-0.5	7.1E-01	-0.3	1.0E+00	0.1	9.3E-01	0.2	1.0E+00	-0.1	8.8E-01	0.1	9.3E-01
3057	ENOX1	A_23_P128574	NM_017993	-1.1	1.11E-03	-0.6	1.77E-02	-1.5	1.11E-03	-1.1	4.07E-05	-0.1	7.80E-01	0.6	3.81E-02	-0.1	7.80E-01	-0.1	8.75E-01	-0.1	1.0E+00	-0.2	1.0E+00	-0.1	7.4E-01	0.2	1.0E+00	0.0	9.8E-01	0.0	1.0E+00	-0.1	8.6E-01	0.0	9.8E-01
39611	IGFBP3	A_24_P320699	NM_001013398	-1.1	5.10E-03	-1.1	3.85E-02	-1.7	8.81E-04	-0.7	5.10E-03	-0.9	1.08E-01	-1.0	1.08E-01	-0.4	2.97E-01	-0.9	3.67E-03	-0.1	1.0E+00	0.0	1.0E+00	-0.1	9.2E-01	-0.3	1.0E+00	0.1	9.7E-01	-0.1	1.0E+00	0.1	9.6E-01	0.2	9.7E-01
27391	CHDH	A_23_P357185	NM_018397	-1.1	6.52E-03	-1.1	2.33E-02	-1.8	6.52E-03	-1.0	3.42E-03	-0.6	1.95E-01	-0.6	1.72E-01	-0.7	1.95E-01	-0.3	5.79E-01	0.1	1.0E+00	0.0	1.0E+00	0.1	8.9E-01	0.1	1.0E+00	0.2	7.5E-01	0.2	1.0E+00	0.2	7.5E-01	0.3	7.3E-01
35401	AK055438	A_24_P35935	AK055438	-1.1	2.22E-03	-1.2	1.56E-03	-1.1	2.68E-02	-1.0	2.22E-03	-0.5	6.43E-01	-0.7	3.84E-01	0.0	9.78E-01	-0.5	6.43E-01	-0.1	1.0E+00	-0.1	1.0E+00	-0.2	8.6E-01	0.1	1.0E+00	-0.2	8.0E-01	0.0	1.0E+00	-0.2	7.8E-01	-0.4	8.0E-01

Feature	Gene symbol	Probe	Transcript	72 hour siCASP8AP2								48 hours siCASP8AP2								24 hours siCASP8AP2								10 hours siCASP8AP2							
				Median M	Median q	1 M	1 q	3 M	3 q	6 M	6 q	Median M	Median q	1 M	1 q	3 M	3 q	6 M	6 q	Median M	Median q	1 M	1 q	3 M	3 q	6 M	6 q	Median M	Median q	1 M	1 q	3 M	3 q	6 M	6 q
24132	THC2507792	A_32_P155026	THC2507792	-1.1	3.14E-02	-1.1	1.22E-02	-1.3	3.14E-02	-0.7	3.59E-02	-0.8	9.36E-03	-0.8	1.03E-02	-0.9	9.36E-03	-0.8	6.65E-03	-0.6	4.7E-01	-0.6	4.7E-01	-0.6	2.9E-01	-0.1	1.0E+00	-0.2	9.3E-01	-0.3	1.0E+00	-0.2	6.9E-01	-0.1	9.3E-01
21187	PERLD1	A_24_P275828	NM_033419	-1.1	8.91E-04	-1.1	8.91E-04	-0.7	2.64E-02	-1.7	9.14E-06	-0.6	1.17E-01	-0.6	1.17E-01	-0.6	3.05E-01	-1.2	2.13E-03	-0.2	1.0E+00	-0.1	1.0E+00	-0.2	7.1E-01	-0.2	1.0E+00	0.1	7.9E-01	0.1	1.0E+00	0.1	6.9E-01	0.2	7.9E-01
38553	SNX17	A_23_P28238	NM_014748	-1.1	1.02E-03	-1.1	1.85E-02	-2.1	7.23E-04	-1.0	1.02E-03	-0.5	4.34E-01	-0.3	5.79E-01	-1.3	4.34E-01	-0.5	3.68E-01	0.2	1.0E+00	0.2	1.0E+00	0.1	8.4E-01	0.2	1.0E+00	0.0	1.0E+00	-0.2	1.0E+00	0.1	9.1E-01	0.0	1.0E+00
31350	INHBB	A_23_P153964	NM_002193	-1.1	2.08E-03	-1.1	2.08E-03	-1.0	2.35E-02	-1.4	1.19E-04	-1.0	6.18E-03	-1.1	6.18E-03	-0.9	1.91E-02	-1.0	2.65E-03	-0.2	1.0E+00	-0.2	1.0E+00	0.0	9.9E-01	-0.2	1.0E+00	0.0	9.8E-01	0.0	1.0E+00	0.1	9.2E-01	0.0	9.8E-01
38598	GEMIN8	A_24_P156576	NM_017856	-1.1	3.00E-02	-0.8	3.00E-02	-1.1	3.53E-02	-1.2	1.01E-03	-0.6	1.34E-01	-0.5	1.34E-01	-0.9	2.11E-01	-0.6	5.04E-02	-0.1	1.0E+00	-0.1	1.0E+00	-0.1	8.9E-01	-0.1	1.0E+00	0.2	4.7E-01	0.2	1.0E+00	0.2	4.7E-01	0.3	3.4E-01
33680	THC2641587	A_32_P174258	THC2641587	-1.1	3.86E-02	-0.9	4.54E-02	-1.4	3.86E-02	-1.1	8.15E-03	-1.0	8.09E-02	-1.0	1.49E-01	-0.9	8.09E-02	-1.3	2.10E-02	-0.2	1.0E+00	0.2	1.0E+00	-0.6	7.1E-01	-0.2	1.0E+00	0.5	8.3E-01	0.1	1.0E+00	0.5	4.8E-01	0.5	8.3E-01
1879	GK5	A_24_P362805	BX648681	-1.1	3.71E-03	-0.8	5.68E-03	-1.3	3.71E-03	-1.1	1.49E-03	-0.6	1.54E-01	-0.3	6.98E-01	-0.7	1.54E-01	-0.6	1.01E-01	0.1	1.0E+00	0.1	1.0E+00	-0.1	9.1E-01	0.1	1.0E+00	-0.3	1.8E-01	-0.1	1.0E+00	-0.3	1.4E-01	-0.5	1.8E-01
35153	PCBD1	A_23_P367405	NM_000281	-1.1	1.32E-03	-0.9	3.35E-03	-1.3	1.32E-03	-1.1	2.97E-05	-0.5	2.07E-01	-0.5	2.07E-01	-0.6	2.20E-01	-0.5	1.60E-01	0.0	1.0E+00	0.0	1.0E+00	-0.1	7.1E-01	0.0	1.0E+00	-0.2	8.8E-01	0.0	1.0E+00	-0.2	7.3E-01	-0.2	8.8E-01
3412	AK055302	A_32_P109242	AK055302	-1.1	1.45E-02	-1.1	1.45E-02	-2.0	1.14E-02	-0.9	1.83E-02	-0.5	3.41E-01	-0.3	4.46E-01	-0.5	3.41E-01	-0.7	1.50E-01	0.3	1.0E+00	-0.1	1.0E+00	0.3	7.2E-01	0.5	1.0E+00	-0.4	3.8E-01	-0.3	1.0E+00	-0.4	3.8E-01	-0.6	3.8E-01
44299	CBWD2	A_32_P161432	NM_172003	-1.1	1.00E-03	-1.1	1.83E-02	-1.8	1.00E-03	-0.9	9.12E-04	-0.5	2.87E-01	-0.4	3.99E-01	-0.8	2.87E-01	-0.5	1.09E-01	0.0	1.0E+00	0.0	1.0E+00	0.0	9.6E-01	0.0	1.0E+00	0.2	9.3E-01	0.2	1.0E+00	0.3	6.8E-01	0.1	9.3E-01
15296	C20orf177	A_23_P68505	ENST00000358293	-1.1	1.64E-03	-1.1	1.64E-03	-1.3	5.13E-03	-0.9	9.99E-04	-0.8	4.20E-02	-0.7	4.20E-02	-0.9	6.73E-02	-0.8	1.44E-02	-0.1	1.0E+00	0.0	1.0E+00	-0.1	8.8E-01	-0.1	1.0E+00	0.0	9.9E-01	0.0	1.0E+00	0.0	9.9E-01	0.0	9.6E-01
10136	TMEM68	A_32_P15464	NM_152417	-1.1	2.29E-03	-1.1	2.29E-03	-1.7	2.70E-03	-0.9	1.61E-03	-0.6	2.08E-01	-0.3	5.70E-01	-1.1	2.08E-01	-0.6	1.26E-01	0.1	1.0E+00	0.1	1.0E+00	-0.1	9.1E-01	0.2	1.0E+00	-0.2	7.2E-01	-0.1	1.0E+00	-0.2	4.3E-01	-0.2	7.2E-01
43911	SERBP1	A_23_P337424	NM_001018067	-1.1	2.01E-02	-1.1	5.49E-03	-1.3	2.03E-02	-0.9	2.01E-02	-0.4	5.72E-01	-0.2	7.50E-01	-0.7	1.96E-01	-0.4	5.72E-01	0.0	1.0E+00	0.0	1.0E+00	-0.3	7.1E-01	0.0	1.0E+00	-0.2	8.3E-01	-0.1	1.0E+00	-0.2	5.2E-01	-0.2	8.3E-01
32039	LOC100132763	A_24_P934245	AK092801	-1.1	9.25E-04	-1.1	9.25E-04	-1.0	5.10E-03	-1.1	5.39E-05	-1.1	1.58E-02	-1.2	1.58E-02	-1.0	3.71E-02	-1.1	1.38E-02	-0.1	1.0E+00	-0.1	1.0E+00	0.0	1.0E+00	-0.2	1.0E+00	0.5	2.8E-01	0.5	1.0E+00	0.4	2.3E-01	0.6	2.8E-01
7181	FASTK	A_23_P502930	NM_006712	-1.1	8.23E-04	-1.1	8.23E-04	-0.7	2.76E-02	-1.2	5.71E-05	-0.7	1.87E-02	-0.7	1.87E-02	-0.3	3.27E-01	-1.2	8.36E-04	-0.1	1.0E+00	-0.1	1.0E+00	-0.1	8.2E-01	-0.2	1.0E+00	-0.2	8.2E-01	-0.2	1.0E+00	-0.1	7.6E-01	-0.2	8.2E-01
12979	RNF160	A_32_P115505	NM_015565	-1.1	1.77E-02	-1.1	1.77E-02	-1.8	1.13E-02	-1.0	3.11E-02	-0.7	2.17E-01	-0.5	2.33E-01	-0.8	2.17E-01	-0.7	2.75E-02	-0.2	1.0E+00	-0.1	1.0E+00	-0.2	7.1E-01	-0.2	1.0E+00	-0.1	8.1E-01	-0.1	1.0E+00	-0.1	8.0E-01	-0.3	8.1E-01
14394	HDAC4	A_24_P8892	NM_006037	-1.1	2.40E-02	-0.7	2.40E-02	-1.1	3.67E-02	-1.1	1.75E-03	-0.2	4.98E-01	-0.2	4.98E-01	-0.2	5.60E-01	-0.7	4.67E-03	-0.1	1.0E+00	-0.1	1.0E+00	0.0	9.6E-01	-0.2	1.0E+00	0.0	4.5E-01	0.0	1.0E+00	0.2	3.4E-01	-0.2	4.5E-01
16430	MRPL30	A_24_P302695	NM_145212	-1.1	2.11E-03	-1.0	2.11E-03	-1.5	4.74E-03	-1.1	5.04E-04	-0.7	1.61E-02	-0.6	6.59E-02	-0.7	1.61E-02	-1.0	2.34E-03	-0.1	1.0E+00	-0.2	1.0E+00	-0.1	7.2E-01	-0.1	1.0E+00	0.0	9.6E-01	-0.1	1.0E+00	0.0	9.6E-01	0.0	9.6E-01
10624	NEBL	A_24_P398147	NM_006393	-1.1	7.35E-04	-1.1	7.35E-04	-1.0	3.61E-03	-1.1	3.55E-05	-0.7	5.46E-02	-0.7	5.46E-02	-0.7	1.22E-01	-1.1	7.40E-03	-0.2	1.0E+00	-0.1	1.0E+00	-0.3	7.1E-01	-0.2	1.0E+00	0.0	9.4E-01	0.0	1.0E+00	0.2	5.8E-01	-0.1	9.4E-01
8500	RNFT2	A_24_P13390	NM_032814	-1.1	1.03E-02	-0.9	1.03E-02	-1.1	3.07E-02	-1.2	1.38E-03	-0.3	1.59E-01	-0.3	1.74E-01	-0.3	1.59E-01	-0.8	1.76E-02	-0.2	1.0E+00	-0.1	1.0E+00	-0.2	7.1E-01	-0.2	1.0E+00	0.0	1.0E+00	0.0	1.0E+00	-0.1	9.0E-01	0.0	1.0E+00
28979	CEP290	A_23_P36865	NM_025114	-1.1	3.74E-03	-1.1	3.74E-03	-1.2	6.72E-03	-1.1	2.57E-04	-0.5	6.80E-02	-0.5	7.36E-02	-0.5	6.80E-02	-0.8	3.58E-02	0.2	1.0E+00	-0.2	1.0E+00	0.2	7.7E-01	0.2	1.0E+00	-0.1	6.5E-01	-0.1	1.0E+00	-0.1	6.5E-01	-0.2	5.9E-01
6182	USP5	A_23_P64954	NM_003481	-1.1	1.50E-03	-1.1	1.68E-02	-1.9	1.50E-03	-1.1	1.45E-03	-0.4	5.24E-01	-0.2	6.18E-01	-0.8	5.24E-01	-0.4	4.60E-01	-0.1	1.0E+00	0.1	1.0E+00	-0.1	8.8E-01	-0.1	1.0E+00	0.1	8.7E-01	-0.1	1.0E+00	0.1	8.7E-01	0.3	8.7E-01
7946	COMMD9	A_23_P87323	NM_014186	-1.1	1.04E-02	-0.8	1.04E-02	-1.1	2.95E-02	-1.3	1.24E-03	-0.8	1.62E-02	-0.4	2.01E-01	-0.8	1.62E-02	-0.9	8.93E-03	0.1	1.0E+00	0.0	1.0E+00	0.1	8.3E-01	0.1	1.0E+00	0.0	9.9E-01	-0.1	1.0E+00	0.0	9.7E-01	0.0	9.9E-01
41135	ZZZ3	A_23_P11507	NM_015534	-1.1	7.98E-03	-1.0	7.98E-03	-1.5	1.07E-02	-1.1	3.27E-03	-0.8	1.28E-01	-0.5	2.55E-01	-1.2	1.28E-01	-0.8	4.54E-02	-0.2	1.0E+00	-0.2	1.0E+00	-0.3	7.2E-01	-0.1	1.0E+00	-0.1	8.8E-01	-0.1	1.0E+00	-0.1	8.8E-01	-0.3	7.9E-01
20277	AQR	A_24_P316305	ENST00000156471	-1.1	3.14E-02	-1.1	3.14E-02	-2.2	1.48E-02	-1.0	3.24E-02	-0.8	3.60E-02	-0.3	5.56E-01	-1.1	1.04E-02	-0.8	3.60E-02	0.1	1.0E+00	0.1	1.0E+00	0.0	9.5E-01	0.2	1.0E+00	0.1	9.3E-01	0.1	1.0E+00	0.2	6.1E-01	0.1	9.3E-01
17069	TM2D3	A_24_P193498	NM_078474	-1.1	6.87E-04	-1.1	7.64E-03	-1.7	6.87E-04	-0.9	1.65E-04	-0.7	9.50E-02	-0.7	9.50E-02	-0.9	1.30E-01	-0.7	1.50E-02	0.0	1.0E+00	-0.1	1.0E+00	0.1	9.2E-01	0.0	1.0E+00	0.1	9.8E-01	0.1	1.0E+00	0.2	7.8E-01	0.1	9.8E-01
40753	FAM44B	A_24_P117964	NM_138369	-1.1	5.82E-03	-0.8	6.19E-03	-1.1	5.82E-03	-1.1	5.71E-05	-0.2	6.75E-01	-0.2	6.75E-01	-0.1	7.37E-01	-0.6	3.25E-02	0.0	1.0E+00	-0.1	1.0E+00	0.0	9.9E-01	0.0	1.0E+00	0.0	9.9E-01	0.0	1.0E+00	0.0	9.9E-01	-0.1	9.8E-01
44570	CISD1	A_23_P35467	NM_018464	-1.1	1.49E-02	-1.0	1.49E-02	-1.2	1.97E-02	-1.1	2.79E-03	-0.7	4.92E-02	-0.5	2.96E-01	-0.9	4.92E-02	-0.7	1.74E-02	-0.2	1.0E+00	-0.1	1.0E+00	-0.5	5.7E-01	-0.2	1.0E+00	0.0	9.9E-01	0.0	1.0E+00	-0.2	4.9E-01	0.0	9.9E-01
39272	IPP	A_23_P9662	NM_005897	-1.1	9.13E-03	-1.1	9.13E-03	-0.9	4.02E-02	-1.2	4.01E-04	-0.7	1.86E-01	-0.7	2.21E-01	-0.7	1.86E-01	-1.0	2.57E-02	-0.1	1.0E+00	0.1	1.0E+00	-0.1	8.9E-01	-0.2	1.0E+00	-0.1	9.2E-01	0.2	1.0E+00	-0.3	5.4E-01	-0.1	9.2E-01
40690	ZNF434	A_23_P218282	NM_017810	-1.1	8.82E-03	-1.1	8.82E-03	-1.5	1.08E-02	-1.0	2.07E-03	-0.9	1.08E-01	-0.7	1.08E-01	-1.2	1.71E-01	-0.9	1.42E-02	-0.1	1.0E+00	-0.1	1.0E+00	-0.2	7.1E-01	-0.1	1.0E+00	-0.2	9.1E-01	-0.2	1.0E+00	-0.3	5.3E-01	-0.1	9.1E-01
35437	THC2661861	A_32_P232682	THC2661861	-1.1	1.95E-02	-0.8	1.95E-02	-1.1	2.74E-02	-1.1	1.33E-03	-0.5	1.07E-01	-0.4	1.50E-01	-0.5	1.07E-01	-0.8	1.98E-03	-0.2	1.0E+00	-0.3	1.0E+00	-0.2	7.9E-01	0.0	1.0E+00	-0.3	5.9E-01	-0.2	1.0E+00	-0.3	3.6E-01	-0.3	5.9E-01
7882	DNAJC11	A_24_P413735	NM_018198	-1.1	6.45E-03	-0.7	1.77E-02	-1.1	6.45E-03	-1.1	1.05E-04	-0.3	7.78E-01	-0.3	7.78E-01	-0.2	8.27E-01	-0.4	5.08E-01	-0.1	1.0E+00	0.0	1.0E+00	-0.3	7.1E-01	-0.1	1.0E+00	-0.1	9.2E-01	-0.1	1.0E+00	-0.1	8.8E-01	0.1	9.2E-01
7128	SOLH	A_24_P928639	BC032648	-1.1	3.04E-02	-1.0	3.71E-02	-1.6	3.04E-02	-1.1	1.69E-02	-0.6	1.27E-01	-0.1	8.84E-01	-0.9	1.27E-01	-0.6	8.94E-02	-0.2	1.0E+00	-0.2	1.0E+00	-0.3	7.1E-01	-0.2	1.0E+00	0.1	9.1E-01	0.1	1.0E+00	0.1	8.6E-01	0.2	9.1E-01
34651	AK054572	A_32_P3317	AK054572	-1.1	4.50E-03	-1.0	4.50E-03	-1.3	1.21E-02	-1.1	8.38E-04	-0.7	1.35E-01	-0.5	2.09E-01	-0.9	3.71E-02	-0.7	1.35E-01	0.0	1.0E+00	0.0	1.0E+00	-0.1	8.9E-01	0.1	1.0E+00	-0.3	8.1E-01	0.1	1.0E+00	-0.4	5.1E-01	-0.3	8.1E-01
34599	TTC37	A_23_P61854	ENST00000358746	-1.1	1.29E-03	-0.9	1.29E-03	-1.2	6.14E-03	-1.1	1.61E-04	-0.8	7.96E-02	-0.4	5.25E-01	-0.9	7.96E-02	-0.8	7.34E-02	0.0	1.0E+00	-0.1	1.0E+00	0.1	9.2E-01	0.0	1.0E+00	0.0	9.9E-01	-0.1	1.0E+00	0.1	7.0E-01	0.0	9.9E-01
9256	A_24_P932706	A_24_P932706	A_24_P932706	-1.1	2.25E-03	-1.1	9.50E-04	-0.7	3.14E-02	-1.1	2.25E-03	-0.5	8.16E-02	-0.5	8.16E-02	-0.1	7.27E-01	-0.8	2.05E-02	-0.2	1.0E+00	-0.2	1.0E+00	-0.1	8.1E-01	-0.2	1.0E+00	0.0	1.0E+00	0.0	1.0E+00	0.2	7.3E-01	0.0	1.0E+00
40600	BCKDHB	A_24_P239664	NM_183050	-1.1	3.74E-03	-1.1	3.74E-03	-1.2	1.60E-02	-1.0	2.53E-03	-0.5	3.60E-01	0.0	9.90E-01	-0.5	3.60E-01	-0.5	2.11E-01	0.0	1.0E+00	0.0	1.0E+00	-0.3	7.6E-01	0.2	1.0E+00	-0.3	3.0E-01	-0.2	1.0E+00	-0.3	2.0E-01	-0.5	3.0E-01
43391	PLA2G12A	A_23_P30020	NM_030821	-1.1	1.17E-03	-0.9	1.17E-03	-1.1	3.61E-03	-1.1	1.62E-04	-0.7	2.72E-02	-0.7	2.72E-02	-0.4	1.87E-01	-0.7	5.19E-03	-0.2	1.0E+00	-0.2	1.0E+00	-0.3	7.1E-01	-0.2	1.0E+00	0.0	9.7E-01	0.0	1.0E+00	-0.1	7.3E-01	0.0	9.7E-01
9790	KRR1	A_23_P2216	NM_007043	-1.1	5.82E-04	-1.0	5.82E-04	-1.6	1.41E-03	-1.1	1.25E-04	-1.0	1.68E-02	-1.0	1.68E-02	-0.6	2.79E-01	-1.0	3.85E-03	-0.1	1.0E+00	0.1	1.0E+00	-0.1	8.8E-01	-0.1	1.0E+00	-0.1	9.7E-01	0.0	1.0E+00	-0.1	8.7E-01	-0.1	9.7E-01
8687	AK021664	A_32_P31744	AK021664	-1.1	3.26E-03	-1.1	3.26E-03	-1.1	1.77E-02	-0.9	1.73E-03	-0.6	1.98E-01	-0.5	3.42E-01	-0.6	1.98E-01	-0.8	1.02E-01	0.0	1.0E+00	0.0	1.0E+00	-0.5	7.1E-01	0.0	1.0E+00	-0.3	4.4E-01	-0.2	1.0E+00	-0.3	3.9E-01	-0.4	4.4E-01
25376	ACN9	A_23_P59528	NM_020186	-1.1	9.27E-03	-0.9	3.63E-02	-2.4	5.31E-03	-1.1	9.27E-03	-0.7	8.51E-02	-0.1	8.20E-01	-1.5	8.51E-02	-0.7	2.42E-02	-0.2	1.0E+00	0.1	1.0E+00	-0.2	7.5E-01	-0.2	1.0E+00	-0.1	8.8E-01	0.0	1.0E+00	-0.1	8.8E-01	-0.1	8.4E-01
39015	HSPA12A	A_23_P408376	NM_025015	-1.1	3.45E-03	-0.9	3.45E-03	-1.6	4.32E-03	-1.1	7.16E-04	-0.7	1.14E-01	-0.5	1.75E-01	-0.7	1.14E-01	-0.7	4.95E-02	0.0	1.0E+00	-0.2	1.0E+00	0.0	9.8E-01	0.1	1.0E+00	-0.1	8.8E-01	-0.1	1.0E+00	0.1	8.8E-01	-0.1	8.4E-01
41128	TSEN15	A_23_P104025	NM_052965	-1.1	8.08E-03	-0.7	8.08E-03	-1.1	9.30E-03	-1.2	6.77E-05	-0.9	3.73E-02	-0.3	5.92E-01	-1.0	3.73E-02	-0.9	3.78E-03	0.0	1.0E+00	-0.2	1.0E+00	0.0	9.4E-01	0.0	1.0E+00	-0.2	7.5E-01	-0.2	1.0E+00	-0.1	7.5E-01	-0.3	3.8E-01
407	ZZZ3	A_23_P11507	NM_015534	-1.1	3.22E-03	-1.0	3.22E-03	-1.7	4.89E-03	-1.1	2.61E-03	-0.7	7.82E-02	-0.5	2.45E-01	-1.1	7.82E-02	-0.7	4.34E-02	-0.2	1.0E+00	-0.2	1.0E+00	-0.4	7.1E-01	-0.2	1.0E+00	0.0	1.0E+00	0.1	1.0E+00	0.0	9.5E-01	0.0	1.0E+00
18652	C6orf162	A_24_P388940	NM_001042493	-1.1	3.08E-03	-1.0	3.08E-03	-1.5	8.02E-03	-1.1	2.27E-03	-1.1	5.20E-02	-0.3	7.21E-01	-1.2	5.20E-02	-1.1	3.89E-02	-0.2	1.0E+00	-0.2	1.0E+00	-0.5	7.1E-01	0.0	1.0E+00	-0.2	6.8E-01	0.0	1.0E+00	-0.3	4.9E-01	-0.2	6.8E-01
29913	THC2668907	A_23_P124313	THC2668907	-1.1	1.10E-03	-1.1	1.10E-03	-1.2	4.58E-03	-0.8	9.51E-04	-0.5	1.35E-01	-0.1	8.89E-01	-0.9	8.93E-02	-0.5	1.35E-01	0.0	1.0E+00	0.0	1.0E+00	-0.3	7.1E-01	0.0	1.0E+00	-0.2	8.3E-01	-0.2	1.0E+00	-0.3	5.3E-01	-0.2	8.3E-01
14453	TNS3	A_24_P346431	NM_022748	-1.1	9.73E-03	-0.7	1.93E-02	-1.1	9.73E-03	-1.1	1.68E-04	-0.6	3.02E-02	-0.1	8.10E-01	-1.0	2.56E-02	-0.6	3.02E-02	-0.3	1.0E+00	0.0	1.0E+00	-0.4	7.1E-01	-0.3	1.0E+00	-0.3	6.4E-01	-0.2	1.0E+00	-0.3	3.6E-01	-0.3	6.4E-01
3470	C8orf55	A_23_P257057	NM_016647	-1.1	5.56E-03	-0.9	8.08E-03	-1.2	5.56E-03	-1.1	2.25E-04	-0.4	3.37E-01	-0.3	4.14E-01	-1.0	3.37E-01	-0.4	8.09E-02	0.1	1.0E+00	0.1	1.0E+00	0.1	8.9E-01	0.1	1.0E+00	0.0	9.8E-01	0.0	1.0E+00	-0.1	9.3E-01	0.0	9.8E-01
32889	KBTBD7	A_23_P25605	NM_032138	-1.0	2.19E-03	-1.0	2.19E-03	-1.2	5.87E-03	-1.0	5.17E-04	-0.8	1.20E-01	-0.4	3.96E-01	-0.9	1.20E-01	-0.8	2.10E-02	-0.2	1.0E+00	-0.2	1.0E+00	-0.3	7.1E-01	-0.1	1.0E+00	-0.3	5.3E-01	-0.1	1.0E+00	-0.3	4.5E-01	-0.4	5.3E-01
42427	TMEM18	A_23_P79599	NM_152834	-1.0	1.27E-03	-1.1	1.27E-03	-0.8	5.59E-03	-1.0	2.54E-05	-0.7	8.50E-03	-0.5	7.58E-02	-0.7	8.50E-03	-0.9	1.52E-03	-0.2	1.0E+00	-0.1	1.0E+00	-0.3	7.1E-01	-0.2	1.0E+00	0.0	9.7E-01	0.0	1.0E+00	-0.1	8.3E-01	0.0	9.7E-01
31762	PRR4	A_23_P76291	NM_007244	-1.0	2.70E-03	-1.0	2.70E-03	-1.3	4.08E-03	-0.9	2.43E-03	-0.6	1.02E-01	-0.8	2.71E-02	-0.5	1.27E-01	-0.6	1.02E-01	0.0	1.0E+00	-0.1	1.0E+00	0.0	1.0E+00	0.0	1.0E+00	0.0	9.9E-01	0.0	1.0E+00	0.0	9.9E-01	0.2	8.5E-01
32046	ESD	A_23_P87964	NM_001984	-1.0	1.04E-03	-1.1	1.04E-03	-1.0	3.18E-03	-1.0	2.34E-04	-0.9	2.08E-02	-0.9	1.96E-02	-0.7	1.08E-01	-0.9	2.08E-02	-0.1	1.0E+00	-0.1	1.0E+00	0.0	9.7E-01	-0.1	1.0E+00	0.1	7.4E-01	0.0	1.0E+00	0.3	2.0E-01	0.1	7.4E-01
30998	FAM179A	A_24_P595717	NM_199280	-1.0	3.77E-03	-0.8	6.92E-03	-1.5	3.77E-03	-1.0	1.23E-03	-0.4	5.80E-01	-0.4	6.96E-01	-0.4	5.80E-01	-0.6	2.16E-01	0.0	1.0E+00	-0.2	1.0E+00	0.0	9.8E-01	0.1	1.0E+00	-0.2	9.4E-01	-0.2	1.0E+00	-0.1	9.4E-01	-0.2	8.4E-01
35760	BC062473	A_32_P146815	BC062473	-1.0	9.38E-03	-0.9	9.38E-03	-1.4	9.83E-03	-1.0	1.55E-03	-0.9	2.60E-01	-0.5	5.33E-01	-0.9	1.55E-01	-0.9	2.60E-01	0.1	1.0E+00	0.1	1.0E+00	-0.2	7.9E-01	0.2	1.0E+00	0.0	9.7E-01	0.0	1.0E+00	0.2	5.5E-01	0.0	9.7E-01
39434	BCDIN3D	A_23_P338233	NM_181708	-1.0	5.50E-03	-1.0	5.50E-03	-1.0	4.69E-02	-1.3	5.68E-04	-0.8	5.14E-02	-0.6	2.10E-01	-0.8	5.14E-02	-1.2	4.50E-03	-0.1	1.0E+00	-0.2	1.0E+00	-0.1	8.5E-01	0.0	1.0E+00	-0.1	7.9E-01	-0.1	1.0E+00	-0.1	7.9E-01	-0.4	5.0E-01
41852	ZZZ3	A_23_P11507	NM_015534	-1.0	3.45E-03	-1.0	2.66E-03	-1.4	7.66E-03	-1.0	3.45E-03	-0.7	9.50E-02	-0.5	2.97E-01	-1.2	9.50E-02	-0.7	4.78E-02	-0.2	1.0E+00	-0.2	1.0E+00	-0.3	7.1E-01	-0.2	1.0E+00	0.0	9.9E-01	0.0	1.0E+00	0.0	9.9E-01	0.0	9.8E-01
32536	HOXA13	A_23_P389281	NM_000522	-1.0	3.22E-03	-1.0	3.22E-03	-1.2	1.22E-02	-1.0	1.32E-03	-0.3	5.45E-01	-0.3	5.45E-01	-0.3	5.97E-01	-0.7	2.48E-02	-0.1	1.0E+00	0.1	1.0E+00	-0.3	7.1E-01	-0.1	1.0E+00	-0.2	8.4E-01	0.0	1.0E+00	-0.3	4.6E-01	-0.2	8.4E-01
31511	PEX19	A_23_P160188	NM_002857	-1.0	1.19E-03	-1.0	1.19E-03	-1.8	1.68E-03	-1.0	3.35E-04	-0.4	3.55E-01	0.0	9.64E-01	-0.4	3.55E-01	-0.5	1.81E-01	-0.1	1.0E+00	-0.1	1.0E+00	-0.3	7.4E-01	0.0	1.0E+00	-0.3	7.7E-01	-0.4	1.0E+00	-0.3	4.0E-01	-0.2	7.7E-01
2929	WWOX	A_23_P83368	NM_016373	-1.0	8.50E-03	-0.8	2.33E-02	-1.1	8.50E-03	-1.0	3.95E-04	-0.2	5.51E-01	-0.2	7.31E-01	0.2	5.51E-01	-0.5	3.03E-01	0.2	1.0E+00	0.0	1.0E+00	0.2	7.1E-01	0.2	1.0E+00	-0.3	6.1E-01	0.0	1.0E+00	-0.3	3.5E-01	-0.3	6.1E-01
19061	MYH7B	A_24_P130621	NM_020884	-1.0	7.50E-03	-1.0	7.50E-03	-1.0	2.17E-02	-1.3	5.82E-04	-0.8	1.77E-01	-0.8	1.77E-01	-0.6	3.94E-01	-0.9	1.36E-01	0.1	1.0E+00	0.0	1.0E+00	0.1	8.3E-01	0.2	1.0E+00	0.3	5.2E-01	0.1	1.0E+00	0.3	3.8E-01	0.3	5.2E-01
34421	TAF9B	A_24_P391431	NM_015975	-1.0	3.66E-03	-1.0	3.66E-03	-1.3	4.84E-03	-1.0	3.66E-04	-0.6	1.87E-01	-0.1	7.63E-01	-1.0	1.51E-01	-0.6	1.87E-01	0.1	1.0E+00	0.1	1.0E+00	-0.1	9.3E-01	0.1	1.0E+00	-0.2	9.4E-01	-0.2	1.0E+00	0.1	9.4E-01	-0.3	8.3E-01
42209	C6orf167	A_32_P95914	NM_198468	-1.0	1.10E-02	-1.0	1.10E-02	-1.9	1.68E-03	-0.6	1.22E-02	-0.6	3.22E-01	-0.4	3.22E-01	-0.7	2.03E-01	-0.6	4.14E-01	0.1	1.0E+00	0.1	1.0E+00	0.1	7.3E-01	0.2	1.0E+00	0.0	8.0E-01	0.0	1.0E+00	0.1	6.5E-01	-0.1	8.0E-01
40540	MRPL48	A_23_P162106	NM_016055	-1.0	2.06E-04	-1.0	2.06E-04	-1.3	1.06E-03	-1.0	2.58E-05	-0.6	2.76E-02	-0.4	1.59E-01	-0.6	2.76E-02	-0.8	1.08E-02	0.0	1.0E+00	-0.2	1.0E+00	0.0	9.9E-01	0.1	1.0E+00	-0.2	7.4E-01	-0.3	1.0E+00	-0.2	4.5E-01	-0.2	7.4E-01
42033	GPX7	A_23_P73972	NM_015696	-1.0	5.83E-03	-0.8	5.83E-03	-1.1	1.30E-02	-1.0	9.38E-04	-0.3	3.11E-01	-0.2	6.97E-01	-0.3	3.11E-01	-0.4	7.44E-02	-0.1	1.0E+00	-0.1	1.0E+00	-0.2	7.3E-01	0.0	1.0E+00	-0.1	8.3E-01	0.0	1.0E+00	-0.2	4.9E-01	-0.1	8.3E-01
16301	AI910722	A_32_P178537	AI910722	-1.0	2.71E-03	-1.0	2.71E-03	-1.3	8.45E-03	-0.9	1.44E-03	-1.1	2.00E-03	-0.7	1.68E-02	-1.1	1.73E-03	-1.1	2.00E-03	-0.3	1.0E+00	0.2	1.0E+00	-0.4	7.1E-01	-0.3	1.0E+00	0.2	8.6E-01	0.2	1.0E+00	0.2	8.6E-01	0.4	7.5E-01
25861	AP3S2	A_23_P100011	NM_005829	-1.0	9.00E-03	-1.0	1.63E-02	-1.3	9.00E-03	-1.0	7.64E-04	-0.6	4.59E-01	-0.5	5.10E-01	-0.6	4.59E-01	-0.6	2.55E-01	0.1	1.0E+00	0.1	1.0E+00	0.0	9.6E-01	0.1	1.0E+00	0.0	9.8E-01	-0.1	1.0E+00	0.1	8.7E-01	0.0	9.8E-01
12478	BTN3A2	A_24_P252078	NM_007047	-1.0	9.73E-03	-0.9	9.73E-03	-1.0	3.17E-02	-1.0	1.38E-03	-0.6	7.84E-02	-0.3	5.66E-01	-0.6	7.84E-02	-0.7	1.36E-02	-0.2	1.0E+00	-0.2	1.0E+00	-0.3	7.1E-01	-0.1	1.0E+00	-0.1	1.0E+00	-0.1	1.0E+00	-0.1	7.7E-01	0.0	1.0E+00
18403	CBWD5	A_24_P145035	AK097639	-1.0	1.56E-03	-1.0	1.56E-03	-1.3	7.12E-03	-0.9	1.46E-03	-0.7	1.24E-01	-0.5	2.82E-01	-0.7	1.24E-01	-0.7	4.49E-02	0.0	1.0E+00	-0.1	1.0E+00	0.0	9.7E-01	0.0	1.0E+00	0.0	9.7E-01	0.4	1.0E+00	0.0	9.4E-01	0.0	9.7E-01
42921	LYRM2	A_23_P42042	NM_020466	-1.0	4.61E-04	-1.0	5.70E-04	-1.9	4.61E-04	-0.9	1.68E-04	-0.8	1.05E-01	-0.4	3.74E-01	-0.9	1.05E-01	-0.8	2.90E-02	0.1	1.0E+00	0.1	1.0E+00	-0.2	8.2E-01	0.1	1.0E+00	-0.1	9.3E-01	0.0	1.0E+00	-0.2	6.3E-01	-0.1	9.3E-01
4415	ARL4A	A_23_P145761	NM_005738	-1.0	6.67E-04	-1.0	6.05E-04	-1.6	1.25E-03	-1.0	6.67E-04	-0.6	1.46E-01	-0.6	1.46E-01	-0.6	3.95E-01	-0.7	3.20E-02	-0.2	1.0E+00	-0.1	1.0E+00	-0.2	7.4E-01	-0.3	1.0E+00	0.1	8.1E-01	0.0	1.0E+00	0.1	8.1E-01	0.3	7.4E-01
42753	FAM165B	A_24_P6030	BC045820	-1.0	3.77E-03	-1.0	1.02E-03	-1.5	3.77E-03	-0.7	4.07E-03	-0.6	2.83E-02	-0.4	1.08E-01	-1.1	1.50E-03	-0.6	2.83E-02	-0.3	1.0E+00	-0.3	1.0E+00	-0.7	4.2E-01	0.2	1.0E+00	-0.3	5.4E-01	-0.2	1.0E+00	-0.5	1.2E-01	-0.3	5.4E-01
26556	BACE2	A_24_P14584	NM_012105	-1.0	3.20E-03	-1.1	3.20E-03	-1.0	6.43E-03	-0.8	8.01E-04	-0.9	1.31E-02	-0.9	1.31E-02	-0.5	1.36E-01	-1.1	5.77E-03	0.0	1.0E+00	0.0	1.0E+00	0.1	8.4E-01	-0.1	1.0E+00	0.0	1.0E+00	0.0	1.0E+00	0.0	1.0E+00	-0.1	9.1E-01
7805	C3orf17	A_24_P921155	NM_015412	-1.0	1.26E-03	-1.1	1.26E-03	-0.7	1.02E-02	-1.0	6.63E-05	-0.6	1.10E-01	-0.6	1.10E-01	-0.8	2.64E-01	-0.6	7.86E-02	-0.1	1.0E+00	-0.1	1.0E+00	-0.3	7.1E-01	-0.1	1.0E+00	0.0	9.9E-01	0.0	1.0E+00	0.0	9.9E-01	0.0	9.9E-01
16561	AHRR	A_23_P344400	NM_020731	-1.0	2.26E-02	-0.8	2.26E-02	-1.1	3.41E-02	-1.0	3.00E-03	-0.4	1.44E-01	-0.4	1.44E-01	-0.3	2.95E-01	-0.8	1.25E-02	0.1	1.0E+00	-0.3	1.0E+00	0.2	7.3E-01	0.1	1.0E+00	0.1	9.3E-01	-0.2	1.0E+00	0.2	5.2E-01	0.1	9.3E-01
30224	ZNF397	A_23_P208143	NM_001135178	-1.0	6.13E-03	-0.9	6.13E-03	-1.0	3.12E-02	-1.1	1.62E-03	-0.5	4.01E-01	-0.1	9.13E-01	-0.6	4.01E-01	-0.5	3.62E-01	-0.1	1.0E+00	-0.1	1.0E+00	-0.3	7.1E-01	-0.1	1.0E+00	-0.1	9.7E-01	-0.2	1.0E+00	-0.1	8.8E-01	0.0	9.7E-01
33982	BC057844	A_24_P862207	BC057844	-1.0	1.83E-03	-0.9	1.66E-02	-1.4	1.83E-03	-1.0	7.06E-05	-0.7	8.32E-02	-0.7	1.11E-01	-0.7	8.32E-02	-0.6	3.20E-02	-0.1	1.0E+00	-0.1	1.0E+00	-0.1	8.2E-01	-0.2	1.0E+00	0.0	9.9E-01	-0.1	1.0E+00	0.0	9.7E-01	0.0	9.9E-01
7756	THC2502568	A_32_P40999	THC2502568	-1.0	1.42E-02	-1.0	1.42E-02	-0.8	1.93E-02	-1.4	2.12E-05	-0.6	2.37E-01	-0.6	2.37E-01	-0.2	8.02E-01	-1.2	7.55E-03	-0.1	1.0E+00	-0.1	1.0E+00	-0.1	8.2E-01	-0.1	1.0E+00	-0.4	6.0E-01	0.0	1.0E+00	-0.4	3.8E-01	-0.4	6.0E-01
25091	THC2762599	A_32_P174832	THC2762599	-1.0	7.18E-03	-1.0	5.08E-03	-1.5	7.18E-03	-0.8	1.64E-02	-0.3	6.54E-01	-0.3	6.54E-01	-0.1	8.54E-01	-0.4	4.41E-01	0.1	1.0E+00	0.1	1.0E+00	0.1	9.1E-01	0.3	1.0E+00	0.0	8.5E-01	0.0	1.0E+00	0.1	8.2E-01	-0.1	8.5E-01
29900	WDR3	A_23_P23318	NM_006784	-1.0	1.57E-02	-1.7	1.57E-02	-1.0	3.25E-02	-0.7	1.08E-02	-1.3	3.13E-02	-2.1	1.94E-03	-0.7	1.29E-01	-1.3	3.13E-02	-0.2	7.8E-01	-1.3	9.4E-04	0.1	7.8E-01	-0.2	1.0E+00	0.2	9.2E-01	-0.6	1.0E+00	0.4	5.3E-01	0.2	9.2E-01
17861	TMEM120B	A_24_P119131	NM_001080825	-1.0	7.49E-03	-1.0	7.49E-03	-0.9	4.14E-02	-1.1	9.40E-04	-0.4	5.18E-01	0.0	9.38E-01	-0.4	5.18E-01	-0.7	6.74E-02	-0.2	1.0E+00	-0.2	1.0E+00	-0.2	8.7E-01	0.2	1.0E+00	-0.2	8.8E-01	-0.1	1.0E+00	-0.2	7.2E-01	-0.2	8.8E-01
36198	TSEN2	A_32_P53538	AK027821	-1.0	2.74E-03	-0.9	2.74E-03	-1.1	2.87E-03	-1.0	7.49E-05	-0.4	2.13E-01	-0.2	7.48E-01	-0.4	2.13E-01	-0.6	3.26E-02	0.0	1.0E+00	-0.2	1.0E+00	0.0	9.7E-01	0.0	1.0E+00	0.0	9.1E-01	0.0	1.0E+00	0.0	9.1E-01	-0.2	8.2E-01
8223	C11orf58	A_24_P675386	NM_001142705	-1.0	1.27E-03	-1.0	7.67E-04	-1.3	3.90E-03	-0.8	1.27E-03	-0.5	3.47E-01	-0.6	1.58E-01	-0.5	3.47E-01	-0.5	4.86E-01	0.4	1.0E+00	0.0	1.0E+00	0.4	7.1E-01	0.4	1.0E+00	0.0	9.8E-01	-0.1	1.0E+00	0.0	9.5E-01	0.0	9.8E-01
34085	PHKB	A_23_P206532	NM_001031835	-1.0	1.81E-02	-0.7	3.32E-02	-1.3	1.81E-02	-1.0	2.13E-03	-0.5	3.14E-01	-0.3	4.14E-01	-0.5	3.14E-01	-0.6	6.09E-02	0.0	1.0E+00	0.0	1.0E+00	-0.1	7.9E-01	0.0	1.0E+00	-0.3	6.2E-01	-0.2	1.0E+00	-0.3	5.1E-01	-0.4	6.2E-01
35420	C21orf2	A_23_P211167	NM_004928	-1.0	3.99E-03	-0.8	3.99E-03	-1.0	1.61E-02	-1.1	3.66E-04	-0.4	5.08E-01	-0.1	8.72E-01	-0.4	5.08E-01	-0.5	3.49E-01	0.1	1.0E+00	0.0	1.0E+00	0.1	8.8E-01	0.2	1.0E+00	-0.1	9.1E-01	-0.1	1.0E+00	-0.1	7.7E-01	-0.1	9.1E-01
44436	RNF187	A_24_P23995	NM_001010858	-1.0	6.32E-03	-0.9	9.71E-03	-1.4	6.32E-03	-1.0	7.31E-04	-0.6	2.82E-01	-0.3	4.61E-01	-0.6	6.39E-02	-0.7	2.82E-01	0.1	1.0E+00	0.1	1.0E+00	0.1	9.0E-01	0.1	1.0E+00	0.0	9.8E-01	-0.1	1.0E+00	0.1	9.1E-01	0.0	9.8E-01
12996	BU616488	A_32_P212654	BU616488	-1.0	6.30E-03	-1.0	2.82E-03	-1.4	6.30E-03	-0.6	3.50E-02	-0.5	1.33E-01	-0.2	6.21E-01	-0.6	4.65E-02	-0.5	1.33E-01	-0.2	1.0E+00	-0.2	1.0E+00	-0.3	7.1E-01	0.0	1.0E+00	0.0	9.8E-01	0.1	1.0E+00	-0.2	7.2E-01	0.0	9.8E-01
12242	SNRNP35	A_23_P159101	NM_022717	-1.0	2.52E-03	-1.0	2.52E-03	-0.9	1.40E-02	-1.5	2.37E-05	-0.7	4.17E-02	-0.6	1.35E-01	-0.7	4.17E-02	-1.0	3.92E-03	-0.1	1.0E+00	-0.1	1.0E+00	-0.1	8.3E-01	0.1	1.0E+00	-0.3	6.7E-01	0.0	1.0E+00	-0.3	3.7E-01	-0.3	6.7E-01
16287	DQ786246	A_24_P3704	DQ786246	-1.0	6.07E-03	-1.0	6.07E-03	-1.5	1.36E-02	-1.0	3.06E-03	-0.8	1.77E-01	-0.6	1.77E-01	-1.2	1.90E-01	-0.8	8.92E-02	0.0	1.0E+00	-0.2	1.0E+00	0.0	9.9E-01	0.0	1.0E+00	0.0	6.9E-01	0.0	1.0E+00	0.1	6.8E-01	-0.2	6.9E-01
44179	SMARCE1	A_23_P164387	NM_003079	-1.0	8.35E-04	-1.0	8.35E-04	-1.4	2.92E-03	-0.9	2.84E-04	-0.7	1.84E-02	-0.6	6.19E-02	-0.7	1.84E-02	-0.8	1.79E-02	0.0	1.0E+00	0.0	1.0E+00	0.0	1.0E+00	0.1	1.0E+00	-0.2	6.5E-01	-0.1	1.0E+00	-0.2	6.5E-01	-0.3	5.2E-01
44340	SLC25A10	A_23_P146830	NM_012140	-1.0	2.18E-02	-0.6	2.71E-02	-1.0	2.18E-02	-1.1	1.14E-03	-0.4	4.76E-01	-0.2	7.39E-01	-0.6	9.63E-02	-0.4	4.76E-01	0.0	1.0E+00	0.0	1.0E+00	0.0	9.9E-01	0.0	1.0E+00	0.0	1.0E+00	0.1	1.0E+00	0.0	9.6E-01	0.0	1.0E+00
3102	TTC37	A_23_P61854	ENST00000358746	-1.0	3.13E-03	-0.9	3.13E-03	-1.3	6.16E-03	-1.0	5.88E-04	-0.9	8.48E-02	-0.4	4.49E-01	-0.9	8.48E-02	-0.9	5.40E-02	0.1	1.0E+00	0.0	1.0E+00	0.1	9.0E-01	0.1	1.0E+00	-0.1	8.0E-01	-0.1	1.0E+00	0.1	8.0E-01	-0.2	6.7E-01
14963	A_23_P331087	A_23_P331087	A_23_P331087	-1.0	8.11E-04	-1.0	1.35E-03	-1.4	8.11E-04	-0.6	4.32E-04	-1.1	6.84E-03	-1.1	3.39E-03	-1.1	9.85E-02	-0.7	6.84E-03	-0.1	1.0E+00	-0.1	1.0E+00	-0.1	8.6E-01	-0.1	1.0E+00	0.1	9.5E-01	0.2	1.0E+00	0.1	6.6E-01	0.0	9.5E-01
2670	C15orf29	A_32_P58163	NM_024713	-1.0	2.21E-03	-1.0	4.83E-03	-1.7	2.21E-03	-0.9	1.84E-03	-1.0	4.72E-02	-1.0	1.47E-01	-1.1	4.72E-02	-0.9	4.61E-02	0.0	1.0E+00	0.1	1.0E+00	0.0	9.9E-01	0.0	1.0E+00	-0.1	9.7E-01	0.1	1.0E+00	-0.1	8.9E-01	-0.1	9.7E-01
1952	JAG2	A_23_P106024	NM_002226	-1.0	2.05E-02	-0.9	4.44E-02	-1.3	2.05E-02	-1.0	3.18E-03	-0.4	1.50E-01	-0.4	1.50E-01	-0.8	2.00E-01	-0.4	1.49E-01	0.1	1.0E+00	0.2	1.0E+00	0.1	8.1E-01	0.1	1.0E+00	0.3	6.9E-01	0.0	1.0E+00	0.3	6.8E-01	0.5	6.9E-01
27054	BRE	A_32_P132796	NM_004899	-1.0	1.27E-03	-0.8	1.27E-03	-1.0	2.42E-03	-1.1	2.12E-05	-0.4	4.78E-01	-0.1	9.37E-01	-0.6	4.78E-01	-0.4	4.31E-01	-0.1	1.0E+00	-0.1	1.0E+00	-0.2	7.3E-01	-0.1	1.0E+00	-0.1	9.6E-01	-0.1	1.0E+00	-0.1	8.3E-01	0.0	9.6E-01
39807	MACC1	A_32_P171923	NM_182762	-1.0	3.19E-04	-1.0	3.57E-03	-1.9	3.19E-04	-1.0	2.58E-04	-0.6	1.83E-01	-0.6	1.23E-01	-1.1	1.83E-01	-0.4	4.41E-01	-0.1	1.0E+00	-0.1	1.0E+00	-0.1	8.5E-01	0.1	1.0E+00	0.0	9.7E-01	0.0	1.0E+00	0.0	8.8E-01	0.0	9.7E-01
3633	EFCAB7	A_32_P137266	NM_032437	-1.0	1.85E-03	-1.0	1.85E-03	-1.1	1.29E-02	-1.0	4.84E-04	-0.5	2.01E-01	-0.4	2.22E-01	-0.5	2.01E-01	-0.9	7.55E-03	-0.2	1.0E+00	-0.2	1.0E+00	-0.2	7.4E-01	0.0	1.0E+00	-0.2	7.8E-01	-0.2	1.0E+00	-0.2	6.5E-01	-0.3	7.8E-01
20229	NDRG3	A_24_P185604	NM_032013	-1.0	2.51E-03	-0.9	2.51E-03	-1.0	1.21E-02	-1.0	2.43E-04	-0.9	9.39E-02	-0.2	7.33E-01	-1.0	9.39E-02	-0.9	2.19E-02	-0.1	1.0E+00	-0.2	1.0E+00	0.0	9.6E-01	-0.1	1.0E+00	0.1	8.1E-01	-0.2	1.0E+00	0.1	8.1E-01	0.2	7.6E-01
25407	ATG5	A_24_P175059	NM_004849	-1.0	1.94E-03	-1.0	1.94E-03	-0.9	1.96E-02	-1.3	1.05E-04	-1.0	1.38E-02	-0.5	1.52E-01	-1.2	1.38E-02	-1.0	2.38E-03	-0.3	1.0E+00	-0.2	1.0E+00	-0.4	7.1E-01	-0.3	1.0E+00	-0.3	8.1E-01	-0.3	1.0E+00	-0.1	8.1E-01	-0.5	4.3E-01
15309	TMEM209	A_23_P122805	NM_032842	-1.0	1.01E-02	-0.9	1.35E-02	-1.3	1.01E-02	-1.0	1.15E-03	-1.0	8.77E-02	-0.6	1.66E-01	-1.5	8.77E-02	-1.0	1.06E-02	-0.1	1.0E+00	0.0	1.0E+00	-0.2	7.1E-01	-0.1	1.0E+00	-0.1	7.9E-01	0.0	1.0E+00	-0.1	7.9E-01	-0.2	7.5E-01
13062	MRPS10	A_24_P209285	NM_018141	-1.0	3.32E-04	-1.0	3.32E-04	-0.8	9.02E-03	-1.1	4.05E-05	-0.7	2.19E-02	-0.5	5.89E-02	-0.7	2.19E-02	-0.9	1.30E-03	-0.1	1.0E+00	-0.1	1.0E+00	0.0	9.6E-01	-0.1	1.0E+00	0.0	9.1E-01	-0.1	1.0E+00	0.0	8.9E-01	0.1	9.1E-01
17877	PIGK	A_23_P34307	NM_005482	-1.0	7.53E-04	-0.9	1.56E-03	-1.8	7.53E-04	-1.0	1.55E-04	-0.6	7.06E-02	-0.2	3.23E-01	-0.6	7.06E-02	-0.7	9.24E-03	0.2	1.0E+00	0.1	1.0E+00	0.2	7.3E-01	0.2	1.0E+00	0.0	9.5E-01	0.1	1.0E+00	0.0	9.5E-01	-0.1	8.8E-01
35429	AGL	A_23_P200298	NM_000028	-1.0	1.37E-02	-0.7	3.84E-02	-1.7	1.37E-02	-1.0	6.43E-03	-0.5	2.75E-01	-0.2	8.13E-01	-0.9	2.75E-01	-0.5	2.21E-01	0.0	1.0E+00	0.0	1.0E+00	0.0	1.0E+00	0.1	1.0E+00	0.0	9.3E-01	0.0	1.0E+00	0.1	9.3E-01	-0.1	9.3E-01
26167	LOC653884	A_23_P311201	ENST00000344989	-1.0	1.20E-02	-0.8	1.44E-02	-1.3	1.20E-02	-1.0	1.28E-03	-0.2	6.77E-01	-0.1	9.08E-01	-0.2	6.77E-01	-0.5	2.35E-01	0.2	1.0E+00	0.0	1.0E+00	0.2	7.8E-01	0.2	1.0E+00	0.0	1.0E+00	0.0	1.0E+00	0.0	1.0E+00	0.0	1.0E+00
1763	MIRHG1	A_32_P139196	AB176708	-1.0	6.11E-04	-1.0	9.90E-04	-2.2	4.71E-04	-1.0	6.11E-04	-0.7	1.21E-01	-0.7	1.21E-01	-1.3	6.87E-02	-0.7	1.30E-01	0.0	1.0E+00	0.0	1.0E+00	-0.1	8.8E-01	0.2	1.0E+00	0.3	9.1E-01	0.4	1.0E+00	0.1	9.1E-01	0.3	7.8E-01
9105	C11orf54	A_24_P201404	NM_014039	-1.0	6.39E-03	-1.0	6.39E-03	-1.3	1.96E-02	-1.0	2.76E-03	-0.6	8.09E-02	-0.6	8.12E-02	-0.6	8.09E-02	-0.9	1.14E-02	-0.1	1.0E+00	-0.1	1.0E+00	-0.1	8.7E-01	0.0	1.0E+00	-0.2	6.9E-01	-0.1	1.0E+00	-0.2	6.9E-01	-0.4	6.5E-01
30579	TTRAP	A_23_P8311	NM_016614	-1.0	9.82E-03	-1.0	7.82E-03	-1.3	2.34E-02	-0.8	9.82E-03	-0.6	2.34E-01	-0.6	2.34E-01	-0.5	4.83E-01	-0.8	3.55E-02	0.0	1.0E+00	-0.3	1.0E+00	0.0	9.9E-01	0.0	1.0E+00	-0.3	9.9E-01	-0.3	1.0E+00	0.0	9.9E-01	-0.3	7.0E-01
41293	SLC25A26	A_23_P169879	NM_173471	-1.0	7.34E-03	-1.2	7.34E-03	-1.0	2.04E-02	-0.9	2.48E-03	-0.7	2.12E-01	-0.7	8.16E-02	-0.9	2.30E-01	-0.7	2.12E-01	-0.1	1.0E+00	-0.2	1.0E+00	-0.1	8.8E-01	0.2	1.0E+00	-0.1	9.2E-01	0.0	1.0E+00	-0.1	9.2E-01	-0.2	8.9E-01

Feature	Gene symbol	Probe	Transcript	72 hour								48 hours								24 hours								10 hours							
				siCASP8AP2								siCASP8AP2								siCASP8AP2								siCASP8AP2							
				Median M	Median q	1 M	1 q	3 M	3 q	6 M	6 q	Median M	Median q	1 M	1 q	3 M	3 q	6 M	6 q	Median M	Median q	1 M	1 q	3 M	3 q	6 M	6 q	Median M	Median q	1 M	1 q	3 M	3 q	6 M	6 q
34542	PPP1CB	A_24_P396720	NM_002709	-1.0	8.79E-03	-1.0	1.41E-02	-1.3	8.79E-03	-0.8	1.91E-03	-0.5	1.25E-01	-0.4	1.96E-01	-0.5	4.89E-02	-0.5	1.25E-01	0.1	1.0E+00	-0.1	1.0E+00	0.1	8.1E-01	0.2	1.0E+00	-0.2	6.7E-01	0.0	1.0E+00	-0.2	5.6E-01	-0.2	6.7E-01
35070	RP5-1000E10.4	A_24_P937855	NM_001102396	-1.0	2.38E-03	-1.0	2.19E-03	-1.8	2.38E-03	-0.9	2.58E-03	-0.5	1.27E-01	-0.2	7.53E-01	-0.9	1.13E-01	-0.5	1.27E-01	-0.1	1.0E+00	-0.1	1.0E+00	-0.3	7.4E-01	0.1	1.0E+00	-0.4	7.1E-02	-0.1	1.0E+00	-0.4	1.7E-02	-0.5	7.1E-02
41799	A_24_P108281	A_24_P108281	A_24_P108281	-1.0	6.45E-03	-1.0	4.67E-03	-1.3	1.77E-02	-0.9	6.45E-03	-0.1	8.74E-01	0.1	8.74E-01	-0.1	9.51E-01	-0.5	2.80E-01	-0.2	1.0E+00	-0.2	1.0E+00	-0.2	8.6E-01	0.2	1.0E+00	-0.2	9.2E-01	-0.2	1.0E+00	-0.2	7.2E-01	-0.2	9.2E-01
10745	THC2643320	A_32_P135601	THC2643320	-1.0	1.56E-03	-1.1	1.56E-03	-1.0	2.64E-02	-1.0	1.55E-03	-0.6	1.88E-01	-0.6	2.77E-01	-0.6	1.88E-01	-0.8	7.97E-02	-0.1	1.0E+00	-0.1	1.0E+00	-0.2	7.4E-01	-0.1	1.0E+00	0.1	1.0E+00	0.2	1.0E+00	0.0	1.0E+00	0.1	9.2E-01
15896	ESD	A_24_P841662	AK093643	-1.0	4.10E-03	-0.8	4.10E-03	-1.1	5.78E-03	-1.0	1.71E-04	-0.7	1.32E-01	-0.3	5.01E-01	-0.7	1.32E-01	-0.8	9.03E-03	0.0	1.0E+00	0.0	1.0E+00	-0.1	8.8E-01	0.1	1.0E+00	-0.1	7.9E-01	0.1	1.0E+00	-0.1	7.9E-01	-0.3	4.6E-01
23787	NFE2L2	A_24_P936444	AF323119	-1.0	1.63E-02	-1.0	1.63E-02	-1.1	2.31E-02	-0.9	6.76E-03	-0.5	3.05E-01	-0.5	4.70E-01	-0.8	2.44E-01	-0.5	3.05E-01	-0.2	1.0E+00	-0.1	1.0E+00	-0.5	7.1E-01	-0.2	1.0E+00	-0.1	9.2E-01	-0.2	1.0E+00	-0.1	7.4E-01	-0.1	9.2E-01
10147	ZNF337	A_23_P154758	NM_015655	-1.0	2.42E-03	-0.8	2.42E-03	-1.0	4.10E-03	-1.1	4.12E-05	-0.9	3.89E-02	-0.6	1.88E-01	-1.0	1.48E-02	-0.9	3.89E-02	-0.1	1.0E+00	-0.1	1.0E+00	-0.3	7.1E-01	0.0	1.0E+00	0.1	9.0E-01	-0.1	1.0E+00	0.1	9.0E-01	0.1	9.0E-01
35240	GABRG1	A_32_P89899	NM_173536	-1.0	2.10E-03	-0.9	2.10E-03	-1.2	5.74E-03	-1.0	2.74E-04	-0.5	1.59E-01	-0.4	6.19E-01	-0.5	1.54E-01	-0.5	1.59E-01	-0.1	1.0E+00	0.0	1.0E+00	-0.3	7.1E-01	-0.1	1.0E+00	-0.3	5.3E-01	-0.1	1.0E+00	-0.3	4.0E-01	-0.4	5.3E-01
18974	BC110864	A_32_P144326	BC110864	-1.0	2.69E-03	-0.8	2.69E-03	-1.4	3.53E-03	-1.0	9.19E-04	-0.6	6.95E-02	-0.3	3.61E-01	-0.6	6.95E-02	-0.7	8.75E-03	0.0	1.0E+00	0.1	1.0E+00	-0.2	7.2E-01	0.0	1.0E+00	-0.1	7.0E-01	-0.1	1.0E+00	-0.1	7.0E-01	-0.3	1.8E-01
44919	CRTAP	A_24_P140171	NM_006371	-1.0	1.18E-02	-1.0	1.34E-02	-1.3	1.18E-02	-1.0	2.67E-03	-0.8	4.49E-01	-0.4	5.00E-01	-0.8	4.49E-01	-0.8	5.10E-02	-0.2	1.0E+00	0.0	1.0E+00	-0.3	7.1E-01	-0.2	1.0E+00	-0.1	9.6E-01	-0.1	1.0E+00	-0.1	8.4E-01	0.0	9.6E-01
19883	CTNNB1	A_24_P608330	AK095242	-1.0	2.52E-03	-0.9	2.52E-03	-1.0	3.80E-03	-1.0	6.53E-05	-1.0	5.27E-02	-0.7	1.03E-01	-1.4	3.34E-02	-1.0	5.27E-02	-0.2	1.0E+00	-0.2	1.0E+00	-0.2	8.6E-01	0.1	1.0E+00	0.2	8.2E-01	0.2	1.0E+00	0.4	3.6E-01	0.2	8.2E-01
39635	ANKH	A_24_P371628	NM_054027	-1.0	1.77E-04	-1.0	1.77E-04	-1.0	2.62E-03	-0.8	1.66E-04	-0.5	7.00E-02	-0.5	5.68E-02	-0.4	1.46E-01	-0.6	7.00E-02	-0.1	7.1E-01	-0.3	1.0E-01	-0.1	7.1E-01	-0.1	1.0E+00	-0.1	8.5E-01	-0.1	1.0E+00	-0.1	8.5E-01	-0.2	6.8E-01
6475	KIAA0556	A_23_P381203	NM_015202	-1.0	1.01E-02	-0.9	1.01E-02	-1.3	1.26E-02	-1.0	1.75E-03	-0.6	3.94E-01	-0.4	5.75E-01	-0.8	3.94E-01	-0.6	2.38E-01	0.1	1.0E+00	0.1	1.0E+00	-0.2	7.6E-01	0.2	1.0E+00	-0.3	8.3E-01	-0.3	1.0E+00	-0.4	3.8E-01	-0.2	8.3E-01
7231	TTC37	A_23_P61854	ENST00000358746	-1.0	6.77E-03	-0.9	6.77E-03	-1.3	7.58E-03	-1.0	1.08E-03	-0.8	1.35E-01	-0.4	5.40E-01	-0.9	1.35E-01	-0.8	6.57E-02	0.1	1.0E+00	-0.1	1.0E+00	0.1	9.4E-01	0.1	1.0E+00	-0.1	8.8E-01	-0.1	1.0E+00	0.1	8.8E-01	-0.3	7.1E-01
25626	TBC1D8	A_24_P576445	AK098124	-1.0	3.79E-03	-0.8	4.73E-03	-1.4	3.79E-03	-1.0	5.81E-04	-0.5	2.96E-01	-0.1	9.39E-01	-0.6	2.96E-01	-0.5	2.53E-01	0.0	1.0E+00	0.0	1.0E+00	-0.4	7.1E-01	0.1	1.0E+00	-0.2	8.7E-01	-0.1	1.0E+00	-0.3	5.0E-01	-0.2	8.7E-01
27551	A_32_P69653	A_32_P69653	A_32_P69653	-1.0	1.21E-02	-1.0	1.21E-02	-1.6	1.08E-02	-0.9	1.70E-02	-0.8	6.61E-02	-0.8	5.55E-02	-0.6	1.67E-01	-0.8	6.61E-02	-0.1	1.0E+00	-0.4	1.0E+00	-0.1	8.2E-01	0.0	1.0E+00	-0.1	9.7E-01	-0.1	1.0E+00	0.1	8.7E-01	-0.1	9.7E-01
8829	RABL3	A_23_P158349	NM_173825	-1.0	6.08E-03	-1.0	3.22E-03	-1.5	6.54E-03	-1.0	6.08E-03	-0.6	4.02E-01	-0.4	6.13E-01	-0.6	4.02E-01	-0.6	1.78E-01	-0.1	1.0E+00	-0.1	1.0E+00	-0.2	7.4E-01	-0.1	1.0E+00	-0.2	4.8E-01	-0.1	1.0E+00	-0.2	4.8E-01	-0.4	4.6E-01
22935	SEC62	A_23_P357856	NM_003262	-1.0	2.07E-03	-1.0	1.07E-03	-1.6	2.07E-03	-0.7	2.71E-03	-0.6	5.78E-02	-0.2	5.14E-01	-1.0	3.19E-02	-0.6	5.78E-02	0.1	1.0E+00	0.1	1.0E+00	-0.1	7.9E-01	0.2	1.0E+00	0.0	9.6E-01	0.0	1.0E+00	0.0	9.6E-01	-0.2	7.8E-01
16029	THC2508355	A_32_P71113	THC2508355	-1.0	2.18E-02	-1.0	2.18E-02	-1.5	2.91E-02	-1.0	1.19E-02	-0.5	3.63E-01	-0.5	5.03E-01	-0.8	2.82E-01	-0.5	3.63E-01	-0.1	1.0E+00	-0.1	1.0E+00	-0.1	8.8E-01	0.1	1.0E+00	0.0	9.6E-01	0.0	1.0E+00	0.2	7.0E-01	-0.1	9.6E-01
32620	PSD3	A_23_P20392	NM_015310	-1.0	2.10E-02	-1.0	2.10E-02	-1.3	4.71E-02	-0.9	1.89E-02	-0.6	1.80E-01	-0.2	7.24E-01	-0.8	1.80E-01	-0.6	6.81E-02	0.0	1.0E+00	0.0	1.0E+00	-0.2	7.8E-01	0.1	1.0E+00	-0.2	7.7E-01	-0.4	1.0E+00	-0.2	6.9E-01	-0.2	7.7E-01
44982	MGC27345	A_23_P336218	BC024231	-1.0	7.87E-04	-1.0	7.87E-04	-1.0	9.88E-03	-0.9	5.96E-04	-0.7	7.50E-02	-0.6	1.66E-01	-0.7	7.50E-02	-0.9	1.26E-02	-0.1	1.0E+00	-0.1	1.0E+00	-0.1	7.7E-01	-0.1	1.0E+00	0.1	9.5E-01	0.0	1.0E+00	0.1	8.7E-01	0.1	9.5E-01
7964	BCL2	A_23_P352266	NM_000633	-1.0	1.70E-03	-0.8	4.80E-03	-1.6	1.70E-03	-1.0	2.13E-04	-0.2	5.72E-01	0.3	5.72E-01	-0.6	1.59E-01	-0.2	7.42E-01	0.0	1.0E+00	0.0	1.0E+00	-0.3	7.3E-01	0.2	1.0E+00	-0.1	9.5E-01	0.0	1.0E+00	-0.3	3.8E-01	-0.1	9.5E-01
6390	MGST1	A_23_P36658	NM_145791	-1.0	1.02E-03	-0.9	1.31E-02	-2.1	1.02E-03	-1.0	4.38E-04	-0.3	5.86E-01	-0.1	8.54E-01	-0.7	5.40E-01	-0.3	5.86E-01	0.0	1.0E+00	0.0	1.0E+00	-0.1	9.0E-01	0.2	1.0E+00	-0.1	7.7E-01	0.0	1.0E+00	-0.1	6.6E-01	-0.2	7.7E-01
12420	TTC37	A_23_P61854	ENST00000358746	-1.0	8.29E-03	-0.9	8.29E-03	-1.3	1.12E-02	-1.0	1.79E-03	-0.9	9.61E-02	-0.4	5.17E-01	-0.9	9.61E-02	-0.9	4.76E-02	0.1	1.0E+00	-0.1	1.0E+00	0.1	9.0E-01	0.1	1.0E+00	-0.1	8.4E-01	-0.1	1.0E+00	0.2	6.2E-01	-0.2	8.4E-01
42152	SF3B3	A_24_P945396	D13642	-1.0	7.66E-03	-0.8	8.67E-03	-1.3	7.66E-03	-1.0	8.71E-04	-0.6	1.01E-01	-0.3	5.08E-01	-0.6	1.01E-01	-0.7	4.41E-02	-0.2	1.0E+00	-0.2	1.0E+00	-0.2	7.6E-01	0.0	1.0E+00	-0.1	9.4E-01	-0.1	1.0E+00	-0.1	8.9E-01	-0.1	9.4E-01
1824	URM1	A_23_P94689	NM_030914	-1.0	4.27E-03	-1.0	9.20E-03	-1.2	4.27E-03	-0.8	1.27E-03	-0.9	9.60E-02	-0.9	9.94E-02	-0.9	9.15E-02	-0.7	9.60E-02	-0.1	1.0E+00	0.0	1.0E+00	-0.2	7.6E-01	-0.1	1.0E+00	0.0	9.6E-01	0.0	1.0E+00	0.1	9.2E-01	-0.1	9.6E-01
567	SORBS1	A_24_P326511	NM_001034954	-1.0	3.31E-03	-1.3	1.69E-03	-1.0	2.86E-02	-0.9	3.31E-03	-1.1	1.40E-02	-0.9	7.92E-02	-1.1	1.40E-02	-1.3	1.09E-03	-0.3	1.0E+00	0.0	1.0E+00	-0.5	6.6E-01	-0.3	1.0E+00	-0.2	8.8E-01	-0.1	1.0E+00	-0.2	6.6E-01	-0.2	8.8E-01
4630	SMA4	A_23_P121869	BC050737	-1.0	1.71E-03	-0.9	1.71E-03	-1.3	3.41E-03	-1.0	1.90E-04	-0.7	1.82E-01	-0.7	1.82E-01	-0.7	2.17E-01	-0.7	7.06E-02	0.0	1.0E+00	0.0	1.0E+00	-0.1	8.7E-01	0.1	1.0E+00	-0.1	8.7E-01	0.0	1.0E+00	-0.1	8.7E-01	-0.2	7.8E-01
9995	METTL3	A_23_P54064	NM_019852	-1.0	1.56E-03	-0.8	4.26E-02	-2.1	1.56E-03	-1.0	1.01E-03	-0.3	3.76E-01	-0.2	6.79E-01	-1.3	3.76E-01	-0.3	2.86E-01	0.0	1.0E+00	-0.1	1.0E+00	0.0	9.9E-01	0.2	1.0E+00	0.2	9.1E-01	0.1	1.0E+00	0.2	7.4E-01	0.2	9.1E-01
9031	LSM6	A_23_P259451	NM_007080	-1.0	3.36E-02	-0.9	3.80E-02	-1.3	3.36E-02	-1.0	9.80E-03	-0.7	1.50E-01	-0.4	2.46E-01	-0.7	1.50E-01	-0.9	2.92E-03	-0.1	1.0E+00	-0.1	1.0E+00	-0.2	7.6E-01	0.0	1.0E+00	-0.1	7.1E-01	0.0	1.0E+00	-0.1	7.1E-01	-0.3	6.8E-01
5595	FGF7	A_23_P14612	NM_002009	-1.0	7.58E-04	-1.0	7.58E-04	-1.4	2.52E-03	-0.9	7.57E-04	-0.4	9.74E-02	-0.4	2.48E-01	-0.4	9.74E-02	-0.7	1.94E-02	0.0	1.0E+00	-0.2	1.0E+00	0.0	9.5E-01	0.1	1.0E+00	-0.1	9.9E-01	-0.1	1.0E+00	0.0	9.9E-01	-0.2	8.6E-01
10596	HSP90AB1	A_24_P823684	NM_007355	-1.0	3.43E-03	-0.7	3.24E-02	-1.3	3.43E-03	-1.0	3.97E-04	0.0	9.77E-01	0.0	9.77E-01	0.0	9.93E-01	-0.2	5.49E-01	-0.1	1.0E+00	-0.1	1.0E+00	-0.1	8.3E-01	-0.1	1.0E+00	0.1	9.4E-01	-0.3	1.0E+00	0.1	9.4E-01	0.3	8.8E-01
15219	C6orf129	A_23_P133770	NM_138493	-1.0	4.54E-04	-1.0	4.54E-04	-0.6	2.98E-02	-1.0	8.11E-05	-0.6	1.03E-01	-0.6	1.39E-01	-0.5	1.03E-01	-0.9	6.21E-03	-0.1	1.0E+00	-0.2	1.0E+00	-0.1	7.4E-01	-0.1	1.0E+00	0.0	9.7E-01	-0.1	1.0E+00	0.0	9.6E-01	0.0	9.7E-01
18361	MTERFD1	A_23_P43071	NM_015942	-1.0	1.26E-02	-0.7	1.67E-02	-1.2	1.26E-02	-1.0	1.11E-03	-0.6	1.81E-01	-0.3	3.46E-01	-0.6	1.81E-01	-0.8	2.34E-02	-0.1	1.0E+00	-0.2	1.0E+00	0.0	9.8E-01	-0.1	1.0E+00	0.0	9.1E-01	0.0	1.0E+00	0.1	9.1E-01	-0.3	7.7E-01
44082	FGF9	A_23_P105803	NM_002010	-1.0	1.50E-02	-0.8	1.93E-02	-1.0	1.50E-02	-1.1	3.58E-04	-0.7	3.89E-02	-0.7	3.89E-02	-0.4	3.32E-01	-0.9	4.18E-03	-0.1	1.0E+00	-0.1	1.0E+00	-0.1	8.6E-01	-0.2	1.0E+00	0.1	9.5E-01	0.2	1.0E+00	-0.3	4.4E-01	0.1	9.5E-01
38667	THC2652817	A_32_P77742	THC2652817	-1.0	8.80E-03	-0.8	3.07E-02	-1.7	8.80E-03	-1.0	4.75E-03	-0.7	2.56E-01	-0.6	2.56E-01	-0.7	2.69E-01	-0.8	6.74E-02	0.0	1.0E+00	0.1	1.0E+00	0.0	9.9E-01	0.0	1.0E+00	-0.2	7.7E-01	-0.2	1.0E+00	-0.1	7.7E-01	-0.2	7.2E-01
42396	C9orf125	A_24_P886096	ENST00000374851	-1.0	1.35E-02	-0.7	1.35E-02	-1.1	1.94E-02	-1.0	3.59E-03	0.0	7.29E-01	0.0	9.27E-01	0.1	7.29E-01	-0.5	1.41E-01	0.0	1.0E+00	-0.1	1.0E+00	0.0	9.9E-01	0.1	1.0E+00	0.1	8.8E-01	0.1	1.0E+00	0.1	8.4E-01	-0.1	8.8E-01
4266	CR627122	A_32_P192480	CR627122	-1.0	7.17E-03	-0.8	1.66E-02	-1.5	7.17E-03	-1.0	1.97E-03	-0.5	4.44E-01	-0.1	9.26E-01	-0.5	4.44E-01	-0.8	1.27E-01	0.1	1.0E+00	0.1	1.0E+00	0.1	9.1E-01	0.3	1.0E+00	-0.2	5.7E-01	-0.1	1.0E+00	-0.2	5.7E-01	-0.5	2.9E-01
25013	ZZZ3	A_23_P11507	NM_015534	-1.0	1.37E-02	-0.9	1.37E-02	-1.5	1.74E-02	-1.0	8.15E-03	-0.7	1.10E-01	-0.4	4.15E-01	-1.1	1.10E-01	-0.7	5.90E-02	-0.3	1.0E+00	-0.1	1.0E+00	-0.3	7.1E-01	-0.3	1.0E+00	0.0	9.9E-01	0.0	1.0E+00	0.1	7.9E-01	0.0	9.9E-01
31040	N6AMT1	A_23_P80086	NM_013240	-1.0	1.52E-03	-0.7	2.14E-02	-2.5	1.02E-03	-1.0	1.52E-03	-0.8	6.98E-02	-0.4	1.13E-01	-1.9	1.23E-03	-0.8	6.98E-02	-0.1	1.0E+00	-0.1	1.0E+00	-0.9	9.4E-03	-0.1	1.0E+00	0.0	9.8E-01	0.1	1.0E+00	-0.4	2.9E-01	0.0	9.8E-01
1316	NBEA	A_23_P65278	NM_015678	-1.0	1.40E-03	-0.6	6.99E-03	-1.7	1.40E-03	-1.0	4.99E-04	-0.4	3.07E-01	0.0	9.86E-01	-0.6	2.00E-01	-0.4	3.07E-01	0.1	1.0E+00	0.1	1.0E+00	-0.2	8.0E-01	0.2	1.0E+00	-0.2	2.9E-01	-0.1	1.0E+00	-0.2	2.9E-01	-0.4	2.3E-01
38729	LOC641746	A_32_P57702	XR_036993	-1.0	5.21E-03	-1.0	4.40E-03	-1.0	1.58E-02	-0.8	5.21E-03	-0.6	6.96E-02	-0.6	6.96E-02	-0.2	4.67E-01	-0.7	1.85E-02	-0.3	1.0E+00	0.0	1.0E+00	-0.3	7.3E-01	-0.3	1.0E+00	0.0	1.0E+00	0.0	1.0E+00	0.0	9.4E-01	0.0	1.0E+00
36281	BCL2	A_23_P352266	NM_000633	-1.0	3.16E-03	-0.8	1.56E-02	-1.7	3.16E-03	-1.0	1.64E-03	-0.2	6.15E-01	0.3	6.15E-01	-0.7	1.56E-01	-0.2	7.31E-01	0.0	1.0E+00	0.0	1.0E+00	-0.2	8.0E-01	0.3	1.0E+00	-0.2	8.6E-01	-0.1	1.0E+00	-0.3	5.0E-01	-0.2	8.6E-01
27658	GTF3C6	A_23_P400465	NM_138408	-1.0	3.24E-03	-0.9	7.49E-03	-1.8	3.24E-03	-1.0	2.21E-03	-0.8	8.25E-03	-0.4	1.55E-01	-1.1	3.49E-03	-0.8	8.25E-03	-0.1	1.0E+00	0.0	1.0E+00	-0.3	7.1E-01	-0.1	1.0E+00	0.0	9.8E-01	0.0	1.0E+00	0.0	8.8E-01	0.0	9.8E-01
4440	ZMYM6	A_24_P25326	NM_007167	-1.0	4.50E-03	-0.9	5.17E-03	-1.3	4.50E-03	-1.0	7.38E-04	-0.9	2.15E-02	-0.6	1.84E-01	-1.0	2.15E-02	-0.9	2.01E-02	0.0	1.0E+00	0.0	1.0E+00	-0.4	7.1E-01	0.0	1.0E+00	-0.3	6.2E-01	-0.1	1.0E+00	-0.4	3.2E-01	-0.3	6.2E-01
39665	PEMT	A_23_P163955	NM_007169	-1.0	1.88E-03	-0.8	3.28E-03	-1.8	1.88E-03	-1.0	7.05E-04	-0.4	4.21E-01	-0.4	5.28E-01	-0.7	4.21E-01	-0.4	3.47E-01	0.0	1.0E+00	0.0	1.0E+00	0.0	9.8E-01	0.1	1.0E+00	0.0	1.0E+00	0.0	1.0E+00	-0.1	8.2E-01	0.0	1.0E+00
23408	C6orf70	A_32_P129530	NM_018341	-1.0	1.59E-03	-1.0	1.59E-03	-0.8	1.89E-02	-1.1	1.01E-04	-1.0	1.01E-01	-0.6	1.43E-01	-1.0	1.01E-01	-1.1	7.11E-03	-0.1	1.0E+00	-0.2	1.0E+00	-0.1	9.1E-01	0.0	1.0E+00	0.2	9.2E-01	0.2	1.0E+00	0.3	3.6E-01	0.1	9.2E-01
2083	RPRD1A	A_24_P382253	NM_018170	-1.0	3.18E-03	-0.9	1.15E-02	-2.0	2.41E-03	-1.0	3.18E-03	-0.4	1.98E-01	-0.3	4.90E-01	-0.9	2.06E-02	-0.4	1.98E-01	-0.1	1.0E+00	-0.1	1.0E+00	-0.1	9.2E-01	0.0	1.0E+00	0.0	9.6E-01	0.1	1.0E+00	0.0	9.6E-01	-0.1	8.8E-01
16165	MRPL1	A_23_P84821	NM_020236	-1.0	3.68E-03	-0.7	1.69E-02	-1.6	3.68E-03	-1.0	1.52E-03	-0.8	2.16E-02	-0.4	3.38E-01	-1.1	2.16E-02	-0.8	1.16E-02	0.0	1.0E+00	0.0	1.0E+00	0.0	1.0E+00	0.1	1.0E+00	0.2	9.8E-01	0.2	1.0E+00	0.2	7.6E-01	0.1	9.8E-01
28389	ATRX	A_24_P348660	NM_000489	-1.0	1.32E-03	-1.0	1.32E-03	-2.0	1.21E-03	-0.9	3.31E-03	-0.7	2.07E-01	-0.7	2.07E-01	-1.2	2.52E-01	-0.7	2.60E-02	0.2	1.0E+00	0.1	1.0E+00	0.2	8.3E-01	0.3	1.0E+00	-0.1	9.9E-01	-0.1	1.0E+00	0.0	9.9E-01	-0.1	9.6E-01
2435	AK091357	A_32_P1144	AK091357	-1.0	8.35E-04	-1.0	8.35E-04	-1.2	2.66E-03	-0.7	7.35E-04	-0.4	1.95E-01	-0.4	1.95E-01	-0.7	2.16E-02	-0.4	2.56E-01	-0.1	1.0E+00	-0.1	1.0E+00	-0.2	7.1E-01	0.3	1.0E+00	-0.2	5.9E-01	0.0	1.0E+00	-0.2	2.9E-01	-0.2	5.9E-01
43855	A_32_P185530	A_32_P185530	A_32_P185530	-1.0	6.15E-03	-1.0	6.15E-03	-1.2	7.73E-03	-0.6	5.09E-03	-0.6	3.17E-01	-0.2	6.92E-01	-0.6	3.17E-01	-0.6	1.39E-01	0.1	1.0E+00	0.1	1.0E+00	0.0	9.9E-01	0.3	1.0E+00	0.0	9.5E-01	0.1	1.0E+00	0.0	9.5E-01	-0.3	8.0E-01
37386	AF088004	A_24_P880000	AF088004	-1.0	2.63E-03	-1.0	2.63E-03	-1.3	5.58E-03	-1.0	8.59E-04	-0.9	7.97E-02	-0.6	1.28E-01	-1.1	7.89E-02	-0.9	7.97E-02	-0.1	1.0E+00	-0.1	1.0E+00	-0.4	7.1E-01	-0.1	1.0E+00	-0.1	9.3E-01	0.0	1.0E+00	-0.1	7.5E-01	-0.1	9.3E-01
41021	FAM116A	A_32_P118586	NM_152678	-1.0	1.38E-03	-1.0	1.38E-03	-0.6	1.96E-02	-1.0	5.83E-05	-0.7	1.82E-01	-0.7	1.82E-01	0.0	9.78E-01	-1.0	8.09E-03	0.0	1.0E+00	-0.1	1.0E+00	0.1	8.4E-01	0.0	1.0E+00	-0.1	8.5E-01	-0.1	1.0E+00	-0.1	8.1E-01	-0.2	8.5E-01
15563	TTC15	A_23_P347131	AK094181	-1.0	7.41E-03	-0.8	7.41E-03	-1.0	1.86E-02	-1.2	2.76E-04	-0.5	2.83E-01	-0.3	6.12E-01	-0.5	2.83E-01	-0.6	9.16E-02	-0.1	1.0E+00	-0.2	1.0E+00	-0.1	8.7E-01	-0.1	1.0E+00	0.2	3.2E-01	-0.1	1.0E+00	0.2	1.5E-01	0.3	3.2E-01
44860	SGOL1	A_23_P29723	NM_001012410	-1.0	5.02E-04	-1.0	9.10E-04	-1.5	5.02E-04	-0.8	8.87E-05	-1.1	3.69E-02	-1.0	8.74E-02	-1.1	3.69E-02	-1.2	1.18E-02	-0.1	1.0E+00	-0.1	1.0E+00	-0.1	7.3E-01	-0.1	1.0E+00	-0.2	5.6E-01	-0.1	1.0E+00	-0.2	5.4E-01	-0.3	5.6E-01
7202	HOXA11	A_23_P42706	NM_005523	-1.0	2.10E-03	-1.0	2.10E-03	-1.0	3.39E-03	-1.0	7.97E-05	-0.3	4.84E-01	-0.2	6.97E-01	-0.4	4.84E-01	-0.3	3.64E-01	0.0	1.0E+00	0.0	1.0E+00	-0.2	8.0E-01	0.1	1.0E+00	-0.2	9.1E-01	0.0	1.0E+00	-0.2	8.0E-01	-0.2	9.1E-01
4098	LOC552889	A_24_P263956	NM_001136262	-1.0	4.28E-03	-1.1	2.23E-03	-1.0	3.30E-02	-0.8	4.28E-03	-0.9	4.41E-02	-1.1	1.33E-02	-0.8	4.41E-02	-0.9	6.47E-02	-0.3	7.1E-01	-0.4	4.2E-01	-0.3	7.1E-01	-0.1	1.0E+00	-0.3	7.0E-01	-0.3	1.0E+00	-0.3	2.1E-01	-0.2	7.0E-01
1395	PIGH	A_32_P71736	NM_004569	-1.0	1.71E-03	-1.0	1.71E-03	-1.2	4.81E-03	-0.9	9.29E-04	-0.8	1.05E-01	-0.7	1.05E-01	-0.8	1.52E-01	-0.9	6.65E-03	-0.1	1.0E+00	-0.1	1.0E+00	0.1	9.2E-01	-0.1	1.0E+00	0.1	9.6E-01	0.0	1.0E+00	0.2	7.4E-01	0.1	9.6E-01
33820	PHLDA1	A_24_P943597	NM_007350	-1.0	1.38E-02	-0.9	2.77E-02	-1.5	1.38E-02	-1.0	3.58E-03	-0.6	4.53E-02	-0.3	3.19E-01	-0.6	4.53E-02	-0.9	4.12E-03	0.0	1.0E+00	-0.2	1.0E+00	0.0	9.7E-01	0.0	1.0E+00	-0.1	9.9E-01	-0.2	1.0E+00	0.0	9.9E-01	-0.1	9.2E-01
11124	C11orf71	A_23_P13150	BC009041	-1.0	1.52E-02	-0.8	1.52E-02	-1.0	2.27E-02	-1.0	1.60E-03	-0.4	3.22E-01	0.1	8.27E-01	-0.4	3.22E-01	-0.6	4.27E-02	0.0	1.0E+00	0.0	1.0E+00	-0.2	7.1E-01	0.0	1.0E+00	-0.3	5.3E-01	-0.2	1.0E+00	-0.3	3.0E-01	-0.4	5.3E-01
37555	ILF3	A_23_P434944	NM_004516	-1.0	1.37E-02	-0.6	2.05E-02	-1.2	1.37E-02	-1.0	1.27E-03	-0.7	2.68E-01	-0.3	5.72E-01	-0.9	2.68E-01	-0.7	7.88E-02	-0.1	1.0E+00	-0.1	1.0E+00	-0.2	7.7E-01	-0.1	1.0E+00	-0.2	8.3E-01	-0.2	1.0E+00	-0.2	7.6E-01	-0.2	8.3E-01
21209	LOC257396	A_32_P92563	AK097893	-1.0	3.05E-02	-0.7	3.44E-02	-1.3	3.05E-02	-1.0	8.64E-03	-0.7	1.57E-02	-0.5	1.55E-01	-1.0	1.17E-02	-0.7	1.57E-02	-0.2	1.0E+00	-0.2	1.0E+00	-0.4	7.1E-01	-0.1	1.0E+00	-0.2	7.6E-01	0.1	1.0E+00	-0.2	7.6E-01	-0.3	7.6E-01
29234	FAM183A	A_24_P847326	NM_001101376	-1.0	9.40E-03	-0.6	9.40E-03	-1.0	1.04E-02	-1.0	2.99E-04	-0.7	1.94E-01	-0.2	8.28E-01	-0.7	1.94E-01	-0.9	1.79E-01	-0.1	1.0E+00	0.0	1.0E+00	-0.5	7.1E-01	-0.1	1.0E+00	0.0	9.5E-01	0.1	1.0E+00	0.0	9.5E-01	-0.1	9.3E-01
33284	PIGH	A_23_P2884	NM_004569	-1.0	2.68E-03	-0.9	2.68E-03	-1.1	4.23E-03	-1.0	2.03E-04	-0.4	3.19E-01	-0.4	3.25E-01	-0.7	3.19E-01	-0.4	1.20E-01	0.0	1.0E+00	-0.1	1.0E+00	0.0	1.0E+00	0.1	1.0E+00	0.1	8.9E-01	0.1	1.0E+00	0.1	8.9E-01	0.2	8.6E-01
4084	RAB3IP	A_32_P180920	NM_175623	-1.0	1.65E-03	-0.8	2.37E-03	-1.3	1.65E-03	-1.0	1.00E-04	-0.8	1.79E-01	-0.4	4.49E-01	-1.0	1.79E-01	-0.8	1.16E-01	0.1	1.0E+00	0.1	1.0E+00	-0.1	8.0E-01	0.1	1.0E+00	-0.2	4.8E-01	-0.2	1.0E+00	-0.2	4.5E-01	-0.2	4.8E-01
39804	MUTED	A_24_P77870	NM_201280	-1.0	1.00E-02	-1.0	7.43E-03	-1.0	3.36E-02	-0.9	1.00E-02	-0.5	4.40E-01	-0.5	5.19E-01	-0.5	4.40E-01	-0.5	3.01E-01	-0.1	1.0E+00	0.1	1.0E+00	-0.3	7.1E-01	-0.1	1.0E+00	-0.2	8.8E-01	-0.1	1.0E+00	-0.2	6.7E-01	-0.2	8.8E-01
23643	THC2606573	A_24_P105913	THC2606573	-1.0	2.61E-03	-0.7	6.86E-03	-1.4	2.61E-03	-1.0	4.96E-04	-0.3	6.04E-01	-0.1	9.20E-01	-0.4	4.06E-01	-0.3	6.04E-01	0.1	1.0E+00	0.1	1.0E+00	0.0	9.8E-01	0.2	1.0E+00	-0.1	7.0E-01	0.0	1.0E+00	-0.1	6.7E-01	-0.2	7.0E-01
37520	MYST1	A_23_P100478	NM_032188	-1.0	9.83E-04	-0.7	3.64E-02	-1.5	9.83E-04	-1.0	7.12E-05	-0.2	6.00E-01	-0.2	8.01E-01	-1.1	2.47E-01	-0.2	6.00E-01	0.0	1.0E+00	0.0	1.0E+00	0.0	9.7E-01	0.0	1.0E+00	-0.1	9.8E-01	-0.1	1.0E+00	-0.1	6.4E-01	0.0	9.8E-01
4496	MKL2	A_23_P54556	NM_014048	-1.0	1.24E-03	-1.0	1.24E-03	-1.6	2.00E-03	-0.9	4.89E-04	-0.7	2.67E-02	-0.6	2.67E-02	-0.7	2.88E-02	-0.8	1.95E-03	0.0	1.0E+00	0.0	1.0E+00	-0.1	9.1E-01	0.0	1.0E+00	0.0	1.0E+00	0.0	1.0E+00	0.1	7.3E-01	0.0	1.0E+00
43917	SFRS7	A_24_P222911	NM_001031684	-1.0	5.95E-03	-1.0	4.08E-03	-1.5	7.07E-03	-0.9	5.95E-03	-0.6	2.48E-01	-0.2	6.95E-01	-1.0	2.48E-01	-0.6	1.51E-01	-0.1	1.0E+00	-0.2	1.0E+00	-0.1	8.4E-01	0.0	1.0E+00	-0.1	9.4E-01	-0.1	1.0E+00	0.1	9.1E-01	-0.1	9.4E-01
7941	BC048201	A_32_P134427	BC048201	-1.0	1.89E-02	-0.9	1.89E-02	-1.2	2.96E-02	-1.0	5.11E-03	-0.4	5.30E-01	-0.4	5.30E-01	-0.3	5.71E-01	-0.5	2.55E-01	-0.2	1.0E+00	-0.2	1.0E+00	-0.2	7.9E-01	0.1	1.0E+00	-0.6	3.8E-01	-0.4	1.0E+00	-0.6	1.7E-01	-0.6	3.8E-01
13105	MRPL43	A_23_P382154	NM_176794	-1.0	4.71E-03	-0.8	1.17E-02	-1.3	4.71E-03	-1.0	4.21E-04	-0.6	5.12E-02	-0.2	5.14E-01	-0.6	5.12E-02	-0.9	6.83E-03	0.1	1.0E+00	-0.2	1.0E+00	0.1	8.9E-01	0.2	1.0E+00	-0.1	8.9E-01	-0.1	1.0E+00	-0.1	8.7E-01	-0.1	8.9E-01
37855	A_23_P4462	A_23_P4462	A_23_P4462	-1.0	7.57E-03	-1.0	3.65E-03	-1.3	1.02E-02	-0.9	7.57E-03	-0.5	3.09E-01	-0.3	5.14E-01	-0.5	3.09E-01	-0.7	5.25E-02	-0.2	1.0E+00	-0.3	1.0E+00	-0.2	7.3E-01	0.0	1.0E+00	-0.2	7.6E-01	-0.2	1.0E+00	-0.1	7.6E-01	-0.3	6.2E-01
42657	DYNC1I1	A_23_P93737	NM_004411	-1.0	2.10E-02	-0.7	4.93E-02	-1.2	2.10E-02	-1.0	4.33E-03	-0.2	8.56E-01	0.2	8.56E-01	-0.2	8.56E-01	-0.4	4.08E-01	0.1	1.0E+00	0.0	1.0E+00	0.1	8.7E-01	0.2	1.0E+00	-0.4	7.6E-01	-0.3	1.0E+00	-0.4	5.8E-01	-0.4	7.6E-01
30739	CUTC	A_23_P98015	NM_015960	-1.0	4.48E-03	-1.0	1.18E-02	-1.5	4.48E-03	-0.9	1.16E-03	-0.8	2.62E-01	-0.6	3.96E-01	-1.2	2.62E-01	-0.8	1.70E-02	0.1	1.0E+00	-0.1	1.0E+00	0.1	9.3E-01	0.1	1.0E+00	0.1	9.6E-01	0.1	1.0E+00	0.1	9.4E-01	-0.1	9.6E-01
3490	AK127132	A_32_P222362	AK127132	-1.0	4.57E-03	-1.0	4.57E-03	-1.0	1.62E-02	-0.8	2.01E-03	-0.7	9.48E-02	-0.9	8.56E-02	-0.7	9.48E-02	-0.6	1.96E-01	-0.3	1.0E+00	-0.3	1.0E+00	-0.3	7.1E-01	0.1	1.0E+00	-0.2	5.4E-01	-0.2	1.0E+00	-0.1	5.4E-01	-0.2	4.9E-01
22534	BCL2	A_23_P352266	NM_000633	-1.0	1.13E-03	-0.7	6.03E-03	-1.6	1.13E-03	-1.0	2.62E-04	-0.3	5.51E-01	0.3	5.85E-01	-0.7	5.44E-02	-0.3	5.51E-01	0.0	1.0E+00	0.0	1.0E+00	-0.2	7.7E-01	0.4	1.0E+00	-0.1	9.5E-01	0.0	1.0E+00	-0.3	5.6E-01	-0.1	9.5E-01
17513	ENST00000373061	A_32_P50834	ENST00000373061	-1.0	3.36E-03	-0.7	3.36E-03	-1.1	6.19E-03	-1.0	3.55E-04	-0.2	4.93E-01	-0.1	7.78E-01	-0.2	4.93E-01	-0.6	7.55E-02	0.1	1.0E+00	0.1	1.0E+00	0.1	8.5E-01	0.2	1.0E+00	-0.1	6.3E-01	-0.1	1.0E+00	-0.1	6.3E-01	-0.2	5.8E-01
16549	ARL4A	A_32_P806841	NM_005738	-1.0	1.89E-02	-1.0	3.34E-02	-1.6	1.74E-02	-0.9	1.89E-02	-0.8	1.84E-02	-0.2	7.72E-01	-1.1	9.26E-03	-0.8	1.84E-02	-0.1	1.0E+00	-0.2	1.0E+00	-0.1	8.5E-01	0.1	1.0E+00	0.1	9.5E-01	-0.5	1.0E+00	0.1	9.3E-01	0.1	9.5E-01
40472	GMFB	A_24_P172768	NM_004124	-1.0	2.70E-03	-1.0	4.17E-03	-1.6	2.70E-03	-0.8	2.69E-03	-0.7	3.16E-01	-0.7	1.35E-01	-1.0	3.69E-01	-0.6	3.16E-01	0.0	1.0E+00	-0.2	1.0E+00	0.1	8.9E-01	0.0	1.0E+00	-0.1	9.8E-01	-0.1	1.0E+00	-0.1	8.9E-01	0.0	9.8E-01
1779	POLK	A_23_P386450	NM_016218	-1.0	3.55E-02	-1.3	1.37E-03	-1.0	4.04E-02	-0.7	3.55E-02	-0.7	2.43E-01	-0.9	7.78E-02	-0.6	2.95E-01	-0.7	2.43E-01	-0.1	1.0E+00	-0.3	1.0E+00	-0.1	8.5E-01	0.0	1.0E+00	-0.1	9.9E-01	-0.1	1.0E+00	-0.1	9.3E-01	0.0	9.9E-01
34645	THEM4	A_24_P928510	NM_053055	-1.0	1.75E-02	-0.7	1.75E-02	-1.0	2.28E-02	-1.0	4.98E-04	-0.4	3.78E-01	-0.3	6.56E-01	-0.6	3.06E-01	-0.4	3.78E-01	-0.1	1.0E+00	-0.1	1.0E+00	-0.1	8.8E-01	0.2	1.0E+00	-0.2	6.6E-01	0.0	1.0E+00	-0.3	3.8E-01	-0.2	6.6E-01
37536	SCAMP1	A_24_P149023	NM_004866	-1.0	1.28E-02	-1.0	4.25E-03	-1.0	3.80E-02	-0.7	1.28E-02	-0.2	6.67E-01	-0.2	7.17E-01	-0.2	6.67E-01	-0.3	5.77E-01	-0.1	1.0E+00	-0.1	1.0E+00	-0.1	9.6E-01	0.3	1.0E+00	-0.4	3.0E-01	-0.2	1.0E+00	-0.4	2.9E-01	-0.5	3.0E-01
37823	A_32_P83547	A_32_P83547	A_32_P83547	-1.0	1.30E-03	-1.0	1.62E-03	-1.6	1.30E-03	-0.8	8.92E-04	-0.4	4.16E-01	-0.3	6.23E-01	-0.9	3.94E-01	-0.4	4.16E-01	0.0	1.0E+00	0.0	1.0E+00	-0.2	7.7E-01	0.1	1.0E+00	0.0	9.8E-01	0.0	1.0E+00	0.0	9.6E-01	0.0	9.8E-01
1055	DYNC2LI1	A_23_P502170	NM_015522	-1.0	5.16E-03	-0.9	5.16E-03	-1.3	7.74E-03	-1.0	1.45E-03	-0.6	3.32E-01	-0.4	3.32E-01	-0.6	3.90E-01	-0.6	2.70E-02	0.1	1.0E+00	0.1	1.0E+00	0.0	9.7E-01	0.2	1.0E+00	-0.2	7.7E-01	-0.2	1.0E+00	-0.2	7.7E-01	-0.5	5.0E-01
1749	SIT1	A_23_P43369	NM_014450	-1.0	1.57E-03	-0.8	1.57E-03	-1.2	4.51E-03	-1.0	2.69E-04	-0.3	5.52E-01	0.2	8.16E-01	-0.3	5.52E-01	-0.3	4.43E-01	0.1	1.0E+00	0.0	1.0E+00	0.1	8.2E-01	0.1	1.0E+00	-0.3	7.4E-01	-0.1	1.0E+00	-0.3	4.2E-01	-0.3	7.4E-01
20740	PTGR2	A_23_P48705	NM_152444	-1.0	1.64E-03	-1.0	1.64E-03	-1.0	1.47E-02	-0.9	1.41E-03	-0.4	5.44E-01	-0.1	9.04E-01	-0.4	5.44E-01	-0.5	1.91E-01	0.0	1.0E+00	0.0	1.0E+00	-0.3	7.1E-01	0.0	1.0E+00	-0.2	7.0E-01	0.0	1.0E+00	-0.3	4.1E-01	-0.2	7.0E-01
28526	ZZZ3	A_23_P11507	NM_015534	-1.0	1.88E-02	-1.0	1.58E-02	-1.4	2.97E-02	-0.9	1.88E-02	-0.7	1.28E-01	-0.4	4.56E-01	-1.0	1.28E-01	-0.7	5.50E-02	-0.2	1.0E+00	-0.2	1.0E+00	-0.3	7.1E-01	-0.2	1.0E+00	0.0	9.9E-01	0.0	1.0E+00	0.1	9.0E-01	0.0	9.9E-01
6718	BC030122	A_32_P215556	BC030122	-1.0	1.94E-03	-0.9	1.94E-03	-1.0	5.65E-03	-1.0	5.97E-05	-0.4	4.95E-01	-0.4	4.95E-01	-0.7	9.94E-02	-0.2	6.93E-01	-0.1	1.0E+00	-0.1	1.0E+00	-0.1	8.4E-01	0.2	1.0E+00	-0.2	7.9E-01	0.0	1.0E+00	-0.3	3.3E-01	-0.2	7.9E-01
19252	SSB	A_32_P35512	NM_003142	-1.0	4.88E-03	-1.0	3.35E-03	-1.0	1.57E-02	-0.8	4.88E-03	-1.2	6.28E-03	-1.5	6.28E-03	-0.5	2.31E-01	-1.2	1.26E-03	-0.2	1.0E+00	-0.1	1.0E+00	-0.2	8.0E-01	-0.3	1.0E+00	0.1	9.2E-01	0.0	1.0E+00	0.2	5.0E-01	0.1	9.2E-01
28854	MED23	A_23_P330999	NM_015979	-1.0	1.94E-03	-1.0	5.34E-04	-1.0	6.51E-03	-0.7	1.94E-03	-0.3	5.73E-01	-0.2	8.50E-01	-0.3	5.73E-01	-0.5	3.28E-01	-0.2	1.0E+00	-0.2	1.0E+00	-0.3	7.1E-01	0.0	1.0E+00	-0.1	7.6E-01	-0.1	1.0E+00	-0.1	7.6E-01	-0.3	5.1E-01
8877	PBX1	A_24_P173823	NM_002585	-1.0	7.18E-03	-0.6	4.47E-02	-1.5	7.18E-03	-1.0	1.75E-03	-0.3	4.98E-01	0.2	7.35E-01	-0.3	4.98E-01	-0.3	4.41E-01	0.1	1.0E+00	0.1	1.0E+00	-0.1	9.0E-01	0.2	1.0E+00	-0.4	1.1E-01	-0.1	1.0E+00	-0.4	8.6E-02	-0.5	1.1E-01
43823	BC015835	A_32_P123990	BC015835	-1.0	1.65E-03	-0.9	1.65E-03	-1.2	4.33E-03	-1.0	3.80E-04	-0.6	6.63E-02	0.1	8.83E-01	-0.7	4.64E-02	-0.6	6.63E-02	-0.1	1.0E+00	-0.1	1.0E+00	-0.3	7.1E-01	0.0	1.0E+00	-0.1	7.9E-01	0.0	1.0E+00	-0.1	7.9E-01	-0.2	7.6E-01
34133	LOC170082	A_23_P320159	ENST00000380600	-1.0	9.55E-03	-0.9	9.55E-03	-1.0	2.05E-02	-1.1	2.43E-04	-0.4	4.29E-01	-0.4	5.38E-01	-0.4	4.29E-01	-0.7	6.57E-02	-0.2	1.0E+00	-0.2	1.0E+00	-0.3	7.3E-01	0.0	1.0E+00	-0.3	5.5E-01	0.0	1.0E+00	-0.3	3.1E-01	-0.3	5.5E-01
15445	BCL2	A_23_P352266	NM_000633	-1.0	2.75E-03	-0.7	8.78E-03	-1.6	2.75E-03	-1.0	7.85E-04	-0.1	5.28E-01	0.4	5.28E-01	-0.7	1.85E-01	-0.1	8.56E-01	0.1	1.0E+00	0.1	1.0E+00	-0.2	7.4E-01	0.3	1.0E+00	-0.1	9.5E-01	0.0	1.0E+00	-0.3	5.6E-01	-0.1	9.5E-01
44089	HNRNPA3	A_24_P706314	NM_194247	-1.0	1.08E-02	-1.0	3.78E-03	-1.3	1.22E-02	-0.7	1.08E-02	-1.2	3.54E-03	-0.9	2.84E-02	-1.4	3.54E-03	-1.2	2.93E-03	0.0	1.0E+00	0.0	1.0E+00	-0.1	8.4E-01	0.0	1.0E+00	0.0	9.9E-01	0.0	1.0E+00	0.0	9.9E-01	-0.2	8.1E-01
9524	BC066984	A_24_P287613	BC066984	-0.9	2.75E-03	-0.8	1.02E-02	-1.3	2.75E-03	-0.9	4.99E-04	-0.2	7.30E-01	-0.2	7.30E-01	-0.1	9.19E-01	-0.4	3.37E-01	-0.1	1.0E+00	-0.1	1.0E+00	-0.1	8.2E-01	0.1	1.0E+00	-0.3	6.7E-01	-0.1	1.0E+00	-0.3	4.9E-01	-0.3	6.7E-01
31049	PSMA1	A_23_P150286	NM_148976	-0.9	5.01E-03	-0.9	5.01E-03	-1.3	7.17E-03	-0.9	2.80E-03	-0.5	1.07E-01	-0.5	1.71E-01	-0.5	1.07E-01	-0.6	1.81E-02	-0.1	1.0E+00	-0.1	1.0E+00	-0.1	8.6E-01	-0.1	1.0E+00	-0.1	9.3E-01	-0.1	1.0E+00	-0.1	9.2E-01	-0.1	9.3E-01
43835	WDR72	A_24_P263330	NM_182758	-0.9	1.68E-03	-0.9	1.53E-03	-1.5	3.23E-03	-0.9	1.68E-03	-0.4	2.80E-01	-0.2	7.19E-01	-0.7	2.62E-01	-0.4	2.80E-01	0.0	1.0E+00	0.0	1.0E+00	-0.1	8.8E-01	0.2	1.0E+00	-0.4	3.0E-01	-0.3	1.0E+00	-0.4	2.0E-01	-0.5	3.0E-01
27063	SRP72	A_32_P174713	NM_006947	-0.9	2.16E-02	-0.9	2.16E-02	-1.2	4.68E-02	-0.9	1.17E-02	-1.1	1.55E-02	-1.2	1.55E-02	-0.5	3.11E-01	-1.1	9.27E-03	0.1	1.0E+00	0.2	1.0E+00	0.1	8.9E-01	-0.4	1.0E+00	0.0	1.0E+00	0.0	1.0E+00	0.0	1.0E+00	0.1	9.4E-01
36929	THC2586873	A_24_P842899	THC2586873	-0.9	2.01E-03	-1.0	2.01E-03	-0.8	2.11E-02	-0.9	4.89E-04	-0.4	4.79E-01	-0.4	4.79E-01	-0.4	4.80E-01	-0.7	3.71E-02	-0.3	1.0E+00	-0.1	1.0E+00	-0.3	7.4E-01	-0.3	1.0E+00	-0.3	6.2E-01	0.0	1.0E+00	-0.3	2.9E-01	-0.3	6.2E-01
14415	CYP4V2	A_32_P23838	NM_207352	-0.9	4.85E-03	-0.7	4.95E-03	-1.3	4.85E-03	-0.9	4.50E-04	-0.3	3.72E-01	0.0	9.28E-01	-0.3	3.72E-01	-0.5	1.63E-01	0.0	1.0E+00	-0.1	1.0E+00	0.0	1.0E+00	0.0	1.0E+00	0.0	9.7E-01	0.0	1.0E+00	0.0	9.7E-01	-0.1	8.4E-01
11859	C1orf67	A_32_P87531	BC042869	-0.9	4.50E-03	-0.8	8.69E-03	-1.3	4.50E-03	-0.9	1.09E-03	-0.3	5.23E-01	-0.2	7.61E-01	-0.5	1.72E-01	-0.3	5.23E-01	0.0	1.0E+00	0.0	1.0E+00	-0.1	8.9E-01	0.0	1.0E+00	0.2	8.0E-01	-0.1	1.0E+00	0.2	6.3E-01	0.2	8.0E-01
7711	CSRP2BP	A_23_P501435	NM_020536	-0.9	5.45E-04	-0.9	5.45E-04	-0.8	1.11E-02	-1.1	3.53E-05	-0.6	3.31E-02	-0.6	3.31E-02	-0.4	1.68E-01	-0.8	2.72E-02	-0.1	1.0E+00	-0.1	1.0E+00	-0.1	8.5E-01	-0.2	1.0E+00	0.0	9.9E-01	0.0	1.0E+00	-0.1	8.3E-01	0.0	9.9E-01
25536	TIA1	A_24_P405430	NM_022173	-0.9	2.36E-02	-0.8	2.36E-02	-1.3	2.41E-02	-0.9	5.59E-03	-0.8	1.96E-02	-0.1	9.25E-01	-1.3	1.78E-02	-0.8	1.96E-02	0.0	1.0E+00	0.0	1.0E+00	-0.2	7.1E-01	0.0	1.0E+00	0.0	9.8E-01	0.0	1.0E+00	0.0	9.8E-01	-0.1	8.7E-01
37884	CBWD5	A_32_P35220	NM_001024916	-0.9	1.95E-03	-0.9	1.95E-03	-1.0	1.50E-02	-0.9	1.20E-03	-0.7	1.18E-01	-0.7	1.18E-01	-0.5	3.19E-01	-0.9	1.30E-02	0.0	1.0E+00	0.1	1.0E+00	0.0	9.7E-01	0.0	1.0E+00	-0.1	9.6E-01	-0.1	1.0E+00	0.0	9.6E-01	-0.1	9.6E-01
28311	USP42	A_23_P82394	NM_032172	-0.9	3.79E-03	-0.9	3.79E-03	-1.1	1.06E-02	-0.9	9.75E-04	-0.9	1.69E-02	-0.9	1.69E-02	-0.9	9.60E-02	-0.7	7.08E-03	0.0	1.0E+00	0.0	1.0E+00	0.0	9.6E-01	-0.2	1.0E+00	0.0	9.9E-01	-0.1	1.0E+00	0.0	9.4E-01	0.0	9.9E-01

Feature	Gene symbol	Probe	Transcript	72 hour siCASP8AP2								48 hours siCASP8AP2								24 hours siCASP8AP2								10 hours siCASP8AP2							
				Median	Median	1	1	3	3	6	6	Median	Median	1	1	3	3	6	6	Median	Median	1	1	3	3	6	6	Median	Median	1	1	3	3	6	6
				M	q	M	q	M	q	M	q	M	q	M	q	M	q	M	q	M	q	M	q	M	q	M	q	M	q	M	q	M	q	M	q
41045	SNAP23	A_23_P206177	NM_003825	-0.9	4.35E-02	-0.9	4.68E-02	-1.6	2.43E-02	-0.8	4.35E-02	-0.8	1.08E-01	-0.5	3.72E-01	-1.1	1.08E-01	-0.8	2.44E-02	0.0	1.0E+00	-0.1	1.0E+00	0.0	9.6E-01	0.0	1.0E+00	-0.2	9.1E-01	-0.2	1.0E+00	-0.1	9.1E-01	-0.6	6.4E-01
36080	PAK1IP1	A_23_P122674	NM_017906	-0.9	1.12E-02	-0.9	1.12E-02	-0.9	2.37E-02	-1.5	1.25E-04	-1.2	1.25E-02	-1.2	1.25E-02	-1.1	1.74E-01	-1.3	3.66E-03	-0.3	1.0E+00	-0.2	1.0E+00	-0.3	7.6E-01	-0.5	1.0E+00	0.1	1.0E+00	0.1	1.0E+00	0.2	8.3E-01	0.0	1.0E+00
38747	PPP2R5C	A_24_P369694	NM_178588	-0.9	9.00E-03	-0.9	9.00E-03	-1.7	6.80E-03	-0.9	1.06E-02	-0.5	2.98E-01	-0.3	5.12E-01	-0.8	2.98E-01	-0.5	1.95E-01	-0.2	1.0E+00	-0.2	1.0E+00	-0.2	7.9E-01	0.0	1.0E+00	-0.2	8.2E-01	-0.2	1.0E+00	-0.1	8.2E-01	-0.3	7.2E-01
14292	ZNF343	A_23_P218706	NM_024325	-0.9	4.94E-03	-0.8	8.70E-03	-1.0	4.94E-03	-0.9	2.19E-04	-0.6	2.03E-01	-0.2	8.41E-01	-0.9	1.87E-01	-0.6	2.03E-01	-0.1	1.0E+00	-0.1	1.0E+00	-0.5	7.1E-01	0.0	1.0E+00	-0.1	9.6E-01	-0.1	1.0E+00	-0.3	6.5E-01	-0.1	9.6E-01
18896	RAD23A	A_24_P22887	NM_005053	-0.9	6.28E-03	-0.9	6.28E-03	-0.7	2.83E-02	-1.2	6.34E-05	-0.8	4.25E-02	-0.8	5.94E-02	-1.1	4.25E-02	-0.8	1.65E-02	-0.1	1.0E+00	0.2	1.0E+00	-0.1	7.9E-01	-0.3	1.0E+00	0.2	9.7E-01	0.2	1.0E+00	0.0	9.7E-01	0.2	8.8E-01
12130	IFT74	A_23_P255714	NM_025103	-0.9	2.62E-02	-0.9	1.83E-02	-1.0	4.88E-02	-0.9	2.62E-02	-0.7	1.12E-01	-0.7	7.55E-02	-0.4	3.39E-01	-0.7	1.12E-01	-0.1	1.0E+00	-0.3	1.0E+00	-0.1	8.1E-01	-0.1	1.0E+00	-0.1	9.1E-01	-0.1	1.0E+00	0.1	8.7E-01	-0.1	9.1E-01
23895	BCL2	A_23_P352266	NM_000633	-0.9	9.55E-04	-0.7	8.57E-03	-1.7	9.55E-04	-0.9	3.55E-04	-0.2	4.81E-01	0.3	4.81E-01	-0.6	9.51E-02	-0.2	6.31E-01	0.1	1.0E+00	0.1	1.0E+00	-0.2	7.3E-01	0.4	1.0E+00	-0.2	8.7E-01	0.0	1.0E+00	-0.3	4.9E-01	-0.2	8.7E-01
11644	MED20	A_24_P273865	NM_004275	-0.9	3.14E-04	-1.2	3.14E-04	-0.7	1.38E-02	-0.9	8.03E-05	-0.9	1.35E-02	-0.9	8.01E-03	-0.7	7.89E-02	-1.0	1.35E-02	-0.1	7.2E-01	-0.5	1.8E-02	-0.1	7.2E-01	-0.1	1.0E+00	-0.1	8.8E-01	-0.3	1.0E+00	-0.1	7.7E-01	-0.1	8.8E-01
39450	RHBDD1	A_24_P134834	NM_032276	-0.9	8.88E-05	-1.0	8.88E-05	-0.9	2.58E-03	-0.9	3.28E-05	-0.6	3.87E-02	-0.3	3.91E-01	-0.6	3.87E-02	-0.7	3.63E-02	-0.2	1.0E+00	-0.2	1.0E+00	-0.3	7.1E-01	-0.2	1.0E+00	-0.1	8.7E-01	-0.1	1.0E+00	-0.1	5.8E-01	-0.1	8.7E-01
24157	LOC554203	A_32_P223935	NR_024582	-0.9	2.51E-03	-0.8	4.28E-03	-1.2	2.51E-03	-0.9	1.88E-04	-0.5	1.94E-02	-0.5	1.43E-01	-0.7	1.70E-02	-0.5	1.94E-02	0.0	1.0E+00	0.0	1.0E+00	0.0	9.1E-01	-0.1	1.0E+00	-0.1	9.2E-01	0.0	1.0E+00	-0.1	7.8E-01	-0.1	9.2E-01
8522	RABL3	A_24_P302785	NM_173825	-0.9	2.02E-03	-0.8	2.02E-03	-1.2	2.53E-03	-0.9	9.43E-05	-0.8	1.75E-01	-0.4	2.77E-01	-0.8	1.75E-01	-0.8	1.95E-02	0.1	1.0E+00	0.1	1.0E+00	0.0	9.1E-01	0.1	1.0E+00	-0.1	7.7E-01	0.0	1.0E+00	-0.1	7.7E-01	-0.3	5.6E-01
44275	LRRC23	A_23_P36689	NM_006992	-0.9	8.18E-03	-0.6	8.25E-03	-0.9	8.18E-03	-1.1	7.98E-05	-0.5	5.41E-01	0.0	9.24E-01	-0.6	5.41E-01	-0.5	2.29E-02	0.1	1.0E+00	0.1	1.0E+00	0.1	7.3E-01	0.1	1.0E+00	-0.2	9.7E-01	-0.2	1.0E+00	0.0	9.7E-01	-0.2	7.6E-01
18030	NHEJ1	A_24_P251381	NM_024782	-0.9	3.97E-04	-0.8	3.97E-04	-1.2	9.38E-04	-0.9	1.62E-05	-0.5	2.09E-01	-0.2	7.14E-01	-0.5	1.28E-01	-0.5	2.09E-01	0.0	1.0E+00	-0.1	1.0E+00	0.0	9.4E-01	0.0	1.0E+00	0.0	9.8E-01	-0.1	1.0E+00	0.0	9.8E-01	0.0	9.5E-01
13676	FAM123B	A_23_P308150	NM_152424	-0.9	6.57E-03	-0.9	6.57E-03	-1.3	1.01E-02	-0.9	4.49E-03	-0.4	3.63E-01	0.0	9.22E-01	-0.6	3.63E-01	-0.4	2.38E-01	-0.2	1.0E+00	-0.2	1.0E+00	-0.3	7.1E-01	-0.1	1.0E+00	-0.2	9.9E-01	-0.2	1.0E+00	-0.2	8.6E-01	0.0	9.9E-01
14012	C9orf156	A_24_P212860	NM_016481	-0.9	1.54E-03	-0.9	7.25E-03	-1.4	1.54E-03	-0.9	1.22E-04	-0.3	5.71E-01	-0.1	9.15E-01	-0.5	4.59E-01	-0.3	5.71E-01	-0.1	1.0E+00	-0.1	1.0E+00	-0.1	8.1E-01	0.1	1.0E+00	0.0	9.8E-01	0.2	1.0E+00	0.0	9.8E-01	-0.1	9.4E-01
42599	QRSL1	A_24_P163477	NM_018292	-0.9	8.27E-04	-0.8	1.56E-03	-1.1	8.27E-04	-0.9	1.50E-04	-0.5	2.11E-01	-0.3	2.59E-01	-1.0	2.11E-01	-0.5	3.76E-02	-0.2	1.0E+00	-0.2	1.0E+00	-0.2	7.3E-01	0.0	1.0E+00	0.1	7.4E-01	0.0	1.0E+00	0.1	7.2E-01	0.1	7.4E-01
40167	PHLDA1	A_24_P915692	NM_007350	-0.9	1.01E-03	-0.9	1.01E-02	-1.6	1.01E-03	-0.9	1.97E-04	-1.0	1.24E-01	-0.7	1.24E-01	-1.3	1.49E-01	-1.0	2.03E-02	0.0	1.0E+00	-0.2	1.0E+00	0.1	9.1E-01	0.0	1.0E+00	0.1	9.2E-01	-0.2	1.0E+00	0.3	3.9E-01	0.1	9.2E-01
25203	COPS7A	A_23_P2474	NM_016319	-0.9	5.58E-03	-0.9	3.95E-02	-1.3	5.58E-03	-0.9	9.48E-04	-0.6	3.22E-01	-0.6	3.93E-01	-1.0	3.22E-01	-0.6	1.29E-01	0.0	1.0E+00	0.0	1.0E+00	-0.1	8.8E-01	0.0	1.0E+00	0.0	9.8E-01	-0.1	1.0E+00	0.0	9.8E-01	0.1	9.7E-01
4190	GOLPH3L	A_23_P200890	NM_018178	-0.9	7.15E-03	-0.8	7.15E-03	-1.0	9.64E-03	-0.9	2.77E-04	-0.6	1.92E-01	-0.4	3.85E-01	-0.6	1.92E-01	-0.8	2.23E-02	0.1	1.0E+00	0.0	1.0E+00	0.1	8.6E-01	0.1	1.0E+00	-0.3	4.6E-01	-0.2	1.0E+00	-0.3	4.3E-01	-0.5	4.6E-01
27547	AUH	A_23_P20852	NM_001698	-0.9	5.31E-03	-0.9	5.31E-03	-0.9	1.30E-02	-1.0	2.83E-04	-0.7	1.51E-01	-0.3	2.81E-01	-0.7	1.51E-01	-0.7	6.70E-03	-0.2	1.0E+00	-0.2	1.0E+00	-0.2	7.3E-01	-0.1	1.0E+00	-0.1	8.8E-01	-0.1	1.0E+00	-0.1	8.8E-01	-0.3	7.3E-01
44010	ORC3L	A_23_P42045	NM_181837	-0.9	7.84E-03	-0.9	6.28E-03	-1.5	8.38E-03	-0.8	7.84E-03	-0.8	1.28E-01	-0.6	1.28E-01	-0.8	1.84E-01	-0.8	9.14E-03	-0.1	1.0E+00	-0.1	1.0E+00	-0.1	9.2E-01	0.0	1.0E+00	-0.3	8.5E-01	-0.4	1.0E+00	-0.1	8.5E-01	-0.3	8.3E-01
41882	DHX35	A_23_P5945	NM_021931	-0.9	2.69E-03	-0.9	2.69E-03	-0.8	2.26E-02	-1.3	6.98E-05	-0.7	4.74E-02	-0.7	5.96E-02	-0.7	4.74E-02	-1.1	9.29E-03	-0.2	1.0E+00	-0.2	1.0E+00	-0.2	7.3E-01	-0.1	1.0E+00	-0.2	8.3E-01	-0.2	1.0E+00	-0.2	4.7E-01	-0.2	8.3E-01
27898	ANP32A	A_32_P186027	ENST00000409628	-0.9	1.65E-02	-0.8	1.72E-02	-1.4	1.65E-02	-0.9	9.29E-03	-0.6	2.27E-01	-0.2	5.83E-01	-0.7	2.27E-01	-0.6	2.02E-02	-0.2	1.0E+00	-0.2	1.0E+00	-0.2	7.6E-01	-0.2	1.0E+00	0.0	9.4E-01	0.1	1.0E+00	0.0	9.4E-01	-0.1	8.8E-01
5045	A_24_P212834	A_24_P212834	A_24_P212834	-0.9	1.32E-02	-0.6	3.07E-02	-1.0	1.32E-02	-0.9	6.63E-04	-0.5	5.06E-01	-0.2	5.24E-01	-0.6	5.06E-01	-0.5	7.78E-02	0.1	1.0E+00	-0.1	1.0E+00	0.2	7.2E-01	0.1	1.0E+00	0.0	8.8E-01	0.0	1.0E+00	0.1	8.8E-01	-0.1	8.6E-01
15893	EIF2B4	A_23_P154058	NM_001034116	-0.9	3.31E-03	-0.7	2.36E-02	-1.8	3.31E-03	-0.9	2.97E-03	-0.2	6.90E-01	0.0	9.38E-01	-1.1	2.89E-01	-0.2	6.90E-01	0.0	1.0E+00	0.0	1.0E+00	0.0	9.6E-01	0.1	1.0E+00	0.1	8.0E-01	0.0	1.0E+00	0.1	7.8E-01	0.2	8.0E-01
41942	MRPS10	A_24_P283294	NM_018141	-0.9	5.13E-04	-1.0	5.13E-04	-0.8	5.19E-03	-0.9	6.87E-05	-0.9	5.20E-02	-0.9	5.20E-02	-0.5	2.79E-01	-1.0	1.61E-02	-0.3	1.0E+00	0.0	1.0E+00	-0.3	7.1E-01	-0.4	1.0E+00	0.0	9.5E-01	0.1	1.0E+00	0.0	9.5E-01	-0.1	9.5E-01
41696	AK023633	A_32_P69475	AK023633	-0.9	1.01E-02	-1.1	5.48E-04	-0.9	1.08E-02	-0.6	1.01E-02	-0.9	8.91E-02	-0.7	8.91E-02	-0.9	2.97E-01	-0.9	2.78E-02	0.0	1.0E+00	0.2	1.0E+00	0.0	9.8E-01	0.0	1.0E+00	-0.1	1.0E+00	-0.1	1.0E+00	-0.2	8.3E-01	0.0	1.0E+00
38558	TMED4	A_24_P297827	AK074557	-0.9	5.51E-03	-0.9	4.16E-02	-1.9	5.50E-03	-0.9	5.51E-03	-0.6	2.52E-01	-0.5	4.37E-01	-0.8	2.52E-01	-0.6	1.48E-01	0.1	1.0E+00	0.1	1.0E+00	-0.1	9.0E-01	0.2	1.0E+00	-0.2	7.0E-01	-0.1	1.0E+00	-0.2	5.7E-01	-0.3	7.0E-01
4459	THC2740111	A_32_P154121	THC2740111	-0.9	1.37E-03	-1.5	1.37E-03	-0.9	2.85E-03	-0.8	7.61E-05	-0.7	2.78E-01	-1.0	1.79E-02	-0.2	6.20E-01	-0.7	2.78E-01	0.0	9.6E-01	-0.5	7.1E-01	0.0	9.6E-01	0.2	1.0E+00	-0.1	9.2E-01	-0.3	1.0E+00	-0.1	7.8E-01	-0.1	9.2E-01
1490	NFX1	A_23_P94683	NM_002504	-0.9	3.66E-03	-0.7	2.47E-02	-1.4	3.66E-03	-0.9	4.91E-04	-0.6	1.27E-01	-0.6	1.27E-01	-1.2	1.64E-01	-0.6	8.66E-02	-0.1	1.0E+00	-0.1	1.0E+00	-0.1	9.0E-01	-0.1	1.0E+00	0.2	7.4E-01	0.1	1.0E+00	0.2	6.7E-01	0.2	7.4E-01
16141	AV645774	A_32_P112034	AV645774	-0.9	1.10E-02	-0.7	1.10E-02	-1.0	1.57E-02	-0.9	1.45E-03	-0.7	6.75E-02	-0.3	4.92E-01	-1.0	6.75E-02	-0.7	1.71E-02	-0.1	1.0E+00	-0.2	1.0E+00	-0.1	9.1E-01	0.1	1.0E+00	-0.2	6.9E-01	-0.2	1.0E+00	-0.2	6.9E-01	-0.4	5.9E-01
391	FBXL17	A_32_P137632	BC018548	-0.9	5.26E-03	-0.9	4.11E-03	-1.2	1.41E-02	-0.7	5.26E-03	-0.2	7.16E-01	0.0	9.82E-01	-0.6	1.84E-01	-0.2	7.16E-01	0.0	1.0E+00	0.0	1.0E+00	-0.2	7.4E-01	0.2	1.0E+00	0.1	9.5E-01	0.1	1.0E+00	-0.2	5.1E-01	0.1	9.5E-01
902	SRP9	A_32_P151594	NM_003133	-0.9	1.02E-02	-0.9	1.02E-02	-2.4	8.61E-04	-0.8	1.23E-02	-0.5	3.23E-01	-0.3	3.23E-01	-1.6	3.73E-01	-0.5	1.09E-01	0.2	1.0E+00	0.1	1.0E+00	0.2	8.8E-01	0.3	1.0E+00	-0.1	9.7E-01	-0.1	1.0E+00	-0.1	8.6E-01	0.1	9.7E-01
44273	FAM120C	A_32_P67623	NM_017848	-0.9	1.94E-02	-0.9	3.89E-02	-1.9	7.43E-03	-0.8	1.94E-02	-0.4	2.64E-01	-0.4	2.64E-01	-0.2	4.08E-01	-0.6	9.07E-02	0.1	1.0E+00	0.1	1.0E+00	0.1	9.1E-01	0.2	1.0E+00	0.1	9.3E-01	0.1	1.0E+00	0.1	8.5E-01	-0.1	9.3E-01
40874	THC2537951	A_32_P217330	THC2537951	-0.9	1.92E-03	-0.9	1.92E-03	-1.0	3.01E-03	-0.7	1.28E-03	-0.6	2.77E-01	-0.6	3.71E-01	-0.7	2.77E-01	-0.6	1.53E-01	-0.1	1.0E+00	-0.1	1.0E+00	-0.2	7.4E-01	0.1	1.0E+00	-0.2	5.0E-01	-0.1	1.0E+00	-0.2	5.0E-01	-0.3	4.6E-01
37090	IVD	A_23_P129322	NM_002225	-0.9	2.87E-03	-0.9	2.87E-03	-1.0	6.41E-03	-0.8	7.16E-04	-0.3	7.35E-01	-0.3	7.44E-01	-0.3	6.56E-01	-0.2	7.35E-01	0.0	1.0E+00	0.1	1.0E+00	-0.1	8.5E-01	0.0	1.0E+00	0.0	9.5E-01	0.0	1.0E+00	-0.1	9.3E-01	0.1	9.5E-01
7600	KLRC1	A_23_P151046	NM_002259	-0.9	5.59E-03	-0.9	1.75E-02	-2.5	1.63E-03	-0.9	5.59E-03	-0.6	1.50E-01	-0.3	6.02E-01	-0.9	8.44E-02	-0.6	1.50E-01	0.0	1.0E+00	-0.2	1.0E+00	0.0	1.0E+00	0.3	1.0E+00	-0.1	8.4E-01	-0.1	1.0E+00	-0.1	8.4E-01	-0.3	7.7E-01
6153	PAK1IP1	A_23_P122674	NM_017906	-0.9	1.02E-02	-0.8	1.02E-02	-0.9	1.84E-02	-1.4	8.97E-05	-1.1	1.86E-02	-1.1	1.86E-02	-1.0	1.76E-01	-1.5	1.64E-03	-0.3	1.0E+00	-0.2	1.0E+00	-0.3	7.6E-01	-0.4	1.0E+00	0.0	9.3E-01	0.0	1.0E+00	0.1	8.8E-01	-0.2	9.3E-01
31655	NSL1	A_24_P370970	NM_015471	-0.9	8.38E-03	-0.8	8.38E-03	-1.1	1.05E-02	-0.9	1.92E-03	-0.3	6.10E-01	-0.3	6.10E-01	-0.1	7.89E-01	-0.5	1.58E-01	-0.1	1.0E+00	-0.1	1.0E+00	-0.2	7.2E-01	0.0	1.0E+00	-0.1	9.7E-01	-0.2	1.0E+00	0.0	9.7E-01	-0.1	8.5E-01
15517	ZZZ3	A_23_P11507	NM_015534	-0.9	2.88E-02	-0.9	2.37E-02	-1.4	3.30E-02	-0.9	2.88E-02	-0.6	1.37E-01	-0.4	5.03E-01	-1.1	1.37E-01	-0.6	5.18E-02	-0.2	1.0E+00	-0.2	1.0E+00	-0.2	7.1E-01	-0.2	1.0E+00	0.1	9.7E-01	0.1	1.0E+00	0.1	8.4E-01	0.0	9.7E-01
40285	BM479077	A_32_P212333	BM479077	-0.9	3.06E-03	-1.0	5.38E-03	-0.9	3.06E-03	-0.8	1.69E-04	-0.9	1.49E-01	-1.0	1.69E-01	-0.9	1.46E-01	-0.7	1.49E-01	0.0	1.0E+00	0.0	1.0E+00	0.2	7.5E-01	-0.1	1.0E+00	-0.1	8.6E-01	-0.1	1.0E+00	-0.1	8.0E-01	-0.2	8.6E-01
3572	IMMP2L	A_32_P87872	NM_032549	-0.9	1.56E-02	-0.7	1.56E-02	-1.0	2.28E-02	-0.9	1.50E-03	-0.2	7.19E-01	-0.2	7.19E-01	0.0	9.87E-01	-0.7	1.02E-01	0.2	1.0E+00	0.0	1.0E+00	0.2	7.1E-01	0.2	1.0E+00	-0.2	5.3E-01	-0.1	1.0E+00	-0.2	5.3E-01	-0.3	5.1E-01
39254	AQP5	A_23_P117104	NM_001651	-0.9	1.38E-03	-1.2	1.13E-03	-0.8	4.15E-02	-0.9	1.38E-03	-0.7	6.51E-02	-0.7	6.51E-02	-0.5	2.28E-01	-1.0	6.46E-03	-0.1	1.0E+00	0.1	1.0E+00	-0.2	7.8E-01	-0.1	1.0E+00	-0.4	4.9E-01	-0.3	1.0E+00	-0.4	3.7E-01	-0.5	4.9E-01
7245	RPL5	A_23_P12140	NM_000969	-0.9	2.50E-03	-0.9	1.19E-02	-1.7	1.23E-03	-0.7	2.50E-03	-0.5	1.19E-01	-0.4	1.19E-01	-0.9	3.11E-01	-0.5	1.11E-01	0.1	1.0E+00	0.0	1.0E+00	0.1	9.1E-01	0.3	1.0E+00	-0.1	9.6E-01	-0.1	1.0E+00	0.1	9.3E-01	-0.1	9.6E-01
5791	BU679017	A_32_P163386	BU679017	-0.9	8.03E-04	-0.9	8.03E-04	-1.2	1.98E-03	-0.7	6.89E-04	-0.6	8.10E-02	-0.4	1.60E-01	-0.8	7.15E-02	-0.6	8.10E-02	0.1	1.0E+00	0.1	1.0E+00	-0.2	7.4E-01	0.2	1.0E+00	0.0	9.5E-01	0.0	1.0E+00	0.0	9.5E-01	-0.1	9.2E-01
33711	ECHDC1	A_23_P215051	NM_018479	-0.9	9.24E-04	-0.7	3.18E-03	-1.5	9.24E-04	-0.9	1.37E-04	-0.4	1.33E-01	-0.3	3.74E-01	-0.6	5.58E-02	-0.4	1.33E-01	0.2	1.0E+00	0.0	1.0E+00	0.2	7.1E-01	0.2	1.0E+00	0.0	9.5E-01	0.0	1.0E+00	0.0	9.5E-01	-0.1	6.8E-01
19458	AK123649	A_32_P159320	AK123649	-0.9	2.94E-03	-0.9	3.54E-03	-1.1	2.94E-03	-0.9	1.67E-04	-0.3	4.29E-01	-0.3	4.90E-01	-0.4	4.29E-01	-0.3	3.54E-01	0.1	1.0E+00	0.0	1.0E+00	0.1	9.1E-01	0.3	1.0E+00	-0.1	8.5E-01	-0.1	1.0E+00	-0.1	8.1E-01	-0.2	8.5E-01
8345	CISD1	A_24_P38754	NM_018464	-0.9	3.97E-03	-0.9	1.23E-02	-1.6	3.97E-03	-0.9	2.62E-03	-0.6	1.16E-01	-0.2	7.08E-01	-1.1	4.97E-02	-0.6	1.16E-01	0.0	1.0E+00	0.0	1.0E+00	-0.2	7.4E-01	0.2	1.0E+00	-0.4	6.6E-01	-0.3	1.0E+00	-0.4	4.5E-01	-0.4	6.6E-01
7630	C17orf49	A_23_P141520	NM_174893	-0.9	1.93E-03	-0.9	1.93E-03	-0.7	2.67E-02	-1.2	8.41E-05	-0.8	1.03E-02	-0.5	5.26E-02	-0.9	1.03E-02	-0.8	2.38E-03	-0.2	1.0E+00	0.0	1.0E+00	-0.2	7.1E-01	-0.2	1.0E+00	0.1	1.0E+00	0.1	1.0E+00	0.0	1.0E+00	0.2	7.8E-01
43512	GPX8	A_23_P122052	NM_001008397	-0.9	5.68E-03	-0.9	5.68E-03	-1.0	3.86E-02	-0.8	5.41E-03	-0.2	6.87E-01	-0.2	6.87E-01	-0.2	7.04E-01	-0.6	1.63E-01	0.0	1.0E+00	-0.1	1.0E+00	0.0	9.9E-01	0.2	1.0E+00	-0.4	5.2E-01	-0.2	1.0E+00	-0.4	5.2E-01	-0.6	5.0E-01
23478	THC2710559	A_32_P190613	THC2710559	-0.9	4.86E-03	-0.7	4.86E-03	-0.9	1.36E-02	-0.9	3.91E-04	-0.3	3.39E-01	0.2	7.83E-01	-0.5	1.98E-01	-0.3	3.39E-01	0.0	1.0E+00	0.0	1.0E+00	-0.3	7.1E-01	0.0	1.0E+00	-0.1	7.5E-01	0.1	1.0E+00	-0.1	7.5E-01	-0.3	7.0E-01
13818	ZNF25	A_24_P69691	NM_145011	-0.9	1.32E-02	-0.9	5.94E-03	-1.3	1.32E-02	-0.7	1.89E-02	-0.4	5.09E-01	-0.4	5.09E-01	-0.9	5.56E-02	-0.4	5.46E-01	-0.3	1.0E+00	-0.3	1.0E+00	-1.0	2.1E-02	0.0	1.0E+00	-0.2	7.0E-01	-0.2	1.0E+00	-0.6	1.7E-02	-0.2	7.0E-01
7732	PPAPDC1B	A_23_P329971	NM_032483	-0.9	2.04E-03	-1.2	5.99E-04	-0.8	3.88E-02	-0.9	2.04E-03	-0.6	8.66E-02	-0.3	3.80E-01	-0.6	8.66E-02	-0.7	4.10E-02	0.0	1.0E+00	0.0	1.0E+00	-0.2	7.7E-01	0.0	1.0E+00	-0.3	3.3E-01	0.0	1.0E+00	-0.3	2.7E-01	-0.3	3.3E-01
19775	ZZZ3	A_23_P11507	NM_015534	-0.9	2.56E-02	-0.9	2.40E-02	-1.4	3.96E-02	-0.9	2.56E-02	-0.7	1.37E-01	-0.4	4.06E-01	-1.2	1.37E-01	-0.7	3.44E-02	-0.2	1.0E+00	-0.2	1.0E+00	-0.3	7.1E-01	-0.2	1.0E+00	0.1	9.7E-01	0.1	1.0E+00	0.1	7.9E-01	0.0	9.7E-01
2928	MSH3	A_23_P122001	NM_002439	-0.9	4.67E-03	-0.7	1.44E-02	-1.2	4.67E-03	-0.9	9.23E-04	-0.7	1.89E-01	-0.7	1.89E-01	0.0	9.73E-01	-0.9	3.49E-02	-0.1	1.0E+00	0.1	1.0E+00	-0.1	9.2E-01	-0.2	1.0E+00	-0.1	9.6E-01	-0.1	1.0E+00	0.0	9.6E-01	-0.2	7.9E-01
8284	MOBKL3	A_24_P225719	NM_015387	-0.9	2.97E-02	-0.9	3.35E-02	-1.3	2.41E-02	-0.7	2.97E-02	-0.7	1.96E-01	-0.7	1.96E-01	-0.7	1.96E-01	-0.6	2.65E-01	-0.1	1.0E+00	-0.1	1.0E+00	-0.1	9.6E-01	0.0	1.0E+00	-0.2	6.8E-01	-0.2	1.0E+00	-0.2	6.8E-01	-0.4	6.4E-01
5511	EEPD1	A_23_P333498	NM_030636	-0.9	3.39E-03	-0.9	3.39E-03	-0.9	9.02E-03	-0.7	1.04E-03	-0.4	1.42E-01	-0.4	1.01E-01	-0.4	1.42E-01	-0.3	2.48E-01	-0.2	1.0E+00	-0.3	1.0E+00	-0.2	7.1E-01	-0.1	1.0E+00	-0.1	9.8E-01	-0.1	1.0E+00	-0.1	7.4E-01	0.0	9.8E-01
41088	LOC202451	A_32_P65473	BC022980	-0.9	1.61E-02	-1.0	5.49E-03	-0.9	4.73E-02	-0.6	1.61E-02	-0.6	8.21E-02	-0.3	2.96E-01	-0.6	8.21E-02	-0.6	3.74E-02	0.1	1.0E+00	0.1	1.0E+00	-0.1	9.0E-01	0.1	1.0E+00	0.0	9.8E-01	0.0	1.0E+00	0.0	9.8E-01	-0.2	8.1E-01
5382	THC2495785	A_24_P80571	THC2495785	-0.9	1.02E-02	-0.9	1.89E-02	-1.3	9.02E-03	-0.7	1.02E-02	-0.5	1.73E-01	-0.8	4.95E-02	-0.5	1.73E-01	-0.5	3.73E-01	-0.1	1.0E+00	-0.2	1.0E+00	-0.1	9.1E-01	0.3	1.0E+00	-0.3	5.5E-01	-0.3	1.0E+00	-0.2	5.5E-01	-0.4	4.5E-01
27291	BCL2	A_23_P352266	NM_000633	-0.9	2.08E-03	-0.7	4.08E-03	-1.4	2.08E-03	-0.9	4.50E-04	-0.5	2.18E-01	0.4	2.49E-01	-0.8	6.88E-02	-0.5	2.18E-01	0.0	1.0E+00	0.0	1.0E+00	-0.2	7.4E-01	0.3	1.0E+00	-0.1	9.2E-01	0.0	1.0E+00	-0.3	5.4E-01	-0.1	9.2E-01
43428	PDCD6	A_23_P218997	NM_013232	-0.9	7.77E-03	-0.8	7.77E-03	-1.3	1.10E-02	-0.9	3.46E-03	-0.7	1.88E-01	-0.4	1.88E-01	-0.7	2.17E-01	-0.8	1.05E-02	0.0	1.0E+00	-0.1	1.0E+00	0.0	1.0E+00	0.1	1.0E+00	-0.2	6.4E-01	-0.1	1.0E+00	-0.2	6.4E-01	-0.3	6.2E-01
1151	PQLC3	A_23_P131375	NM_152391	-0.9	1.83E-03	-0.9	1.83E-03	-1.1	5.47E-03	-0.9	3.55E-04	-0.4	5.03E-01	-0.4	5.03E-01	-0.4	5.05E-01	-0.7	6.21E-02	0.0	1.0E+00	0.0	1.0E+00	-0.1	8.0E-01	0.0	1.0E+00	-0.1	9.1E-01	-0.1	1.0E+00	0.0	9.1E-01	-0.2	7.0E-01
15607	TTC37	A_23_P61854	ENST00000358746	-0.9	7.86E-03	-0.9	7.86E-03	-1.2	2.54E-02	-0.9	3.81E-03	-0.8	1.23E-01	-0.3	6.84E-01	-0.8	1.23E-01	-0.8	6.70E-02	0.0	1.0E+00	-0.1	1.0E+00	0.1	9.2E-01	0.0	1.0E+00	0.0	8.6E-01	0.0	1.0E+00	0.3	3.8E-01	-0.1	8.6E-01
6469	BTN3A2	A_23_P391264	NM_007047	-0.9	5.95E-03	-0.9	5.95E-03	-0.8	4.32E-02	-1.3	2.58E-04	-0.6	3.26E-01	-0.6	3.26E-01	-0.1	8.01E-01	-0.8	2.74E-02	-0.2	1.0E+00	-0.2	1.0E+00	-0.2	7.1E-01	-0.1	1.0E+00	-0.3	7.4E-01	-0.1	1.0E+00	-0.3	5.3E-01	-0.4	7.4E-01
30964	NUDT13	A_23_P161324	NM_015901	-0.9	3.83E-03	-0.9	3.83E-03	-0.9	1.68E-02	-1.0	1.21E-03	-0.8	1.38E-01	-0.8	1.44E-01	-0.7	1.38E-01	-0.8	4.68E-02	-0.2	1.0E+00	-0.2	1.0E+00	-0.4	7.1E-01	-0.2	1.0E+00	-0.1	9.3E-01	0.0	1.0E+00	-0.1	8.7E-01	-0.1	9.3E-01
36457	C9orf3	A_23_P73012	NM_032823	-0.9	4.35E-03	-0.9	4.35E-03	-1.1	6.16E-03	-0.9	4.70E-04	-1.0	1.61E-02	-0.6	1.01E-01	-1.0	1.61E-02	-1.0	3.21E-03	0.0	1.0E+00	0.0	1.0E+00	-0.1	8.3E-01	0.0	1.0E+00	-0.2	9.2E-01	-0.2	1.0E+00	0.0	9.2E-01	-0.2	7.7E-01
43249	FUSIP1	A_23_P352291	NM_054016	-0.9	4.01E-03	-0.9	9.63E-03	-2.1	1.10E-03	-0.8	4.01E-03	-0.6	1.16E-01	-0.6	1.16E-01	-1.7	3.13E-01	-0.6	7.86E-02	0.2	1.0E+00	0.1	1.0E+00	0.2	7.4E-01	0.3	1.0E+00	0.0	8.9E-01	0.0	1.0E+00	0.1	8.9E-01	-0.2	7.7E-01
40979	ZDHHC17	A_23_P314191	NM_015336	-0.9	3.43E-04	-0.8	3.43E-04	-1.0	2.80E-03	-0.9	3.99E-05	-0.8	8.33E-02	-0.4	3.06E-01	-1.1	8.33E-02	-0.8	2.00E-02	-0.1	1.0E+00	-0.2	1.0E+00	-0.1	7.4E-01	-0.1	1.0E+00	-0.2	8.2E-01	-0.2	1.0E+00	-0.1	8.2E-01	-0.3	5.9E-01
30254	AK001846	A_24_P724984	AK001846	-0.9	2.30E-03	-0.9	1.97E-03	-1.2	7.55E-03	-0.8	2.30E-03	-0.4	1.85E-01	-0.2	7.37E-01	-0.6	1.85E-01	-0.4	1.44E-01	0.0	1.0E+00	0.0	1.0E+00	-0.2	7.1E-01	0.1	1.0E+00	-0.2	4.6E-01	-0.2	1.0E+00	-0.2	4.6E-01	-0.4	3.2E-01
32751	CR624880	A_32_P150418	CR624880	-0.9	1.65E-03	-0.9	1.65E-03	-1.4	4.18E-03	-0.9	8.42E-04	-0.3	5.85E-01	-0.2	7.40E-01	-0.9	4.31E-02	-0.3	5.85E-01	0.0	1.0E+00	0.0	1.0E+00	-0.4	7.1E-01	0.1	1.0E+00	-0.1	8.7E-01	0.0	1.0E+00	-0.1	5.3E-01	-0.1	8.7E-01
1518	FTO	A_23_P113184	NM_001080432	-0.9	4.70E-03	-0.9	4.70E-03	-0.6	4.32E-02	-1.0	1.21E-04	-0.5	5.45E-02	-0.4	1.98E-01	-0.5	5.45E-02	-0.7	7.18E-03	-0.2	1.0E+00	-0.2	1.0E+00	-0.2	7.5E-01	-0.3	1.0E+00	0.0	9.9E-01	0.0	1.0E+00	-0.1	8.0E-01	0.0	9.9E-01
18178	ATG10	A_24_P370471	NM_031482	-0.9	3.93E-04	-0.8	3.93E-04	-1.0	1.79E-03	-0.9	1.80E-05	-0.3	4.18E-01	-0.3	4.18E-01	-0.3	4.22E-01	-0.5	1.66E-01	0.0	1.0E+00	-0.1	1.0E+00	0.0	9.5E-01	0.1	1.0E+00	-0.1	8.5E-01	-0.1	1.0E+00	-0.2	5.2E-01	-0.1	8.5E-01
33073	CDC42	A_23_P300056	NM_044472	-0.9	2.15E-03	-0.9	2.15E-03	-1.0	1.86E-02	-0.8	1.43E-03	-0.2	7.07E-01	-0.2	7.07E-01	0.0	9.65E-01	-0.5	1.68E-01	0.0	1.0E+00	-0.2	1.0E+00	0.0	9.3E-01	0.0	1.0E+00	0.1	9.5E-01	0.0	1.0E+00	0.1	8.1E-01	0.1	9.5E-01
20234	BCLAF1	A_24_P89512	NM_014739	-0.9	6.28E-03	-0.9	6.28E-03	-1.5	5.53E-03	-0.6	1.09E-02	-0.6	1.13E-01	-0.5	3.44E-01	-0.7	7.18E-02	-0.6	1.13E-01	0.1	1.0E+00	0.1	1.0E+00	0.0	9.1E-01	0.1	1.0E+00	0.1	9.1E-01	0.1	1.0E+00	0.1	7.6E-01	-0.1	9.1E-01
42495	MTRF1	A_23_P37005	NM_004294	-0.9	1.32E-02	-0.7	1.92E-02	-0.9	1.32E-02	-1.0	2.74E-04	-0.6	2.59E-01	-0.1	9.29E-01	-0.7	2.59E-01	-0.6	6.05E-02	0.0	1.0E+00	-0.1	1.0E+00	0.0	9.6E-01	0.1	1.0E+00	-0.4	3.6E-01	-0.2	1.0E+00	-0.4	3.0E-01	-0.5	3.6E-01
12666	ZNF271	A_32_P42780	NR_024565	-0.9	8.70E-04	-0.7	1.94E-03	-1.2	8.70E-04	-0.9	2.21E-04	-0.3	6.58E-01	-0.1	8.85E-01	-0.3	6.58E-01	-0.5	2.19E-01	-0.1	1.0E+00	-0.1	1.0E+00	-0.1	9.0E-01	0.1	1.0E+00	-0.3	4.8E-01	-0.1	1.0E+00	-0.3	4.3E-01	-0.5	4.8E-01
34807	COX11	A_24_P391531	NM_004375	-0.9	2.10E-02	-0.9	2.10E-02	-1.1	1.28E-02	-0.8	2.23E-02	-0.4	5.34E-01	-0.4	5.34E-01	-0.6	2.24E-01	-0.3	6.57E-01	0.0	1.0E+00	0.0	1.0E+00	0.0	9.8E-01	0.3	1.0E+00	-0.1	9.1E-01	-0.1	1.0E+00	-0.1	9.1E-01	-0.4	7.9E-01
28434	TIA1	A_24_P417474	NM_022037	-0.9	2.50E-02	-0.9	2.50E-02	-1.6	2.52E-02	-0.9	2.24E-02	-0.8	1.60E-01	-0.5	3.62E-01	-0.8	1.60E-01	-0.8	7.06E-02	-0.2	1.0E+00	-0.2	1.0E+00	-0.3	7.1E-01	0.0	1.0E+00	-0.2	8.2E-01	-0.1	1.0E+00	-0.2	7.7E-01	-0.3	8.2E-01
14628	MANEA	A_24_P187921	NM_024641	-0.9	2.43E-03	-0.9	2.30E-03	-1.2	8.62E-03	-0.8	2.43E-03	-0.2	5.53E-01	-0.1	9.15E-01	-0.2	5.53E-01	-0.3	4.59E-01	0.0	1.0E+00	0.0	1.0E+00	-0.1	9.2E-01	0.1	1.0E+00	-0.1	9.4E-01	0.0	1.0E+00	-0.1	9.4E-01	-0.2	9.0E-01
6771	ITGB8	A_24_P759477	NM_002214	-0.9	2.76E-03	-0.9	2.76E-03	-1.0	1.72E-02	-0.9	9.66E-04	-0.1	8.36E-01	0.1	9.32E-01	-0.1	8.36E-01	-0.3	3.01E-01	-0.1	1.0E+00	-0.1	1.0E+00	-0.1	8.8E-01	-0.1	1.0E+00	-0.2	4.9E-01	-0.1	1.0E+00	-0.2	4.9E-01	-0.4	4.3E-01
28387	B4GALT2	A_24_P242440	NM_003780	-0.9	1.01E-02	-0.8	3.37E-02	-1.2	1.01E-02	-0.9	9.93E-04	-0.3	5.50E-01	-0.1	8.13E-01	-0.7	5.50E-01	-0.3	4.10E-01	0.1	1.0E+00	0.1	1.0E+00	0.1	8.6E-01	0.1	1.0E+00	0.1	9.3E-01	-0.1	1.0E+00	0.1	8.6E-01	0.2	9.3E-01
6015	SC4MOL	A_23_P110184	NM_006745	-0.9	2.14E-02	-0.9	3.25E-02	-1.6	2.04E-02	-0.8	2.14E-02	-0.6	3.39E-01	-0.3	3.39E-01	-0.7	3.69E-01	-0.6	2.73E-02	0.2	1.0E+00	-0.1	1.0E+00	0.2	7.7E-01	0.2	1.0E+00	-0.2	1.0E+00	-0.3	1.0E+00	0.0	1.0E+00	-0.2	9.0E-01
43216	A_24_P58607	A_24_P58607	A_24_P58607	-0.9	2.55E-02	-0.9	2.55E-02	-2.1	2.21E-03	-0.8	3.13E-02	-0.5	3.50E-01	-0.5	3.50E-01	-1.2	1.85E-01	-0.3	5.73E-01	0.0	1.0E+00	0.1	1.0E+00	0.0	9.9E-01	-0.1	1.0E+00	0.1	9.9E-01	0.1	1.0E+00	0.1	9.4E-01	0.0	9.9E-01
12651	AP3S2	A_24_P287683	NM_005829	-0.9	2.19E-03	-0.9	2.19E-03	-0.7	4.13E-02	-1.1	5.70E-04	-0.5	3.16E-02	-0.5	3.16E-02	-0.4	4.23E-01	-1.1	1.17E-03	-0.1	1.0E+00	-0.2	1.0E+00	-0.1	8.5E-01	-0.1	1.0E+00	0.0	9.7E-01	-0.1	1.0E+00	0.0	9.7E-01	0.1	9.2E-01
33783	C6orf206	A_24_P52189	AK055407	-0.9	2.14E-04	-0.8	2.14E-04	-0.9	1.97E-03	-1.1	6.25E-06	-0.4	4.29E-01	-0.4	6.32E-01	-0.4	4.29E-01	-0.7	1.33E-01	0.0	1.0E+00	0.0	1.0E+00	-0.2	7.4E-01	0.0	1.0E+00	0.0	9.9E-01	0.2	1.0E+00	-0.1	8.4E-01	0.0	9.9E-01
28359	USP40	A_23_P343594	NM_018218	-0.9	2.75E-03	-0.9	2.75E-03	-0.6	4.15E-02	-0.9	2.62E-04	-0.4	3.01E-01	-0.3	6.77E-01	-0.4	3.01E-01	-0.6	7.16E-02	0.0	1.0E+00	0.0	1.0E+00	-0.1	7.9E-01	0.1	1.0E+00	-0.1	9.3E-01	-0.1	1.0E+00	-0.1	7.7E-01	-0.1	9.3E-01
5307	YEATS4	A_23_P87591	NM_006530	-0.9	1.31E-02	-0.9	6.76E-03	-0.9	1.67E-02	-0.8	1.31E-02	-0.8	1.53E-02	-0.8	1.41E-02	-0.8	1.58E-01	-0.8	1.53E-02	-0.1	1.0E+00	-0.1	1.0E+00	0.0	9.7E-01	-0.1	1.0E+00	-0.3	5.1E-01	-0.3	1.0E+00	-0.2	5.1E-01	-0.4	4.9E-01
40610	RBMX	A_32_P193646	NM_002139	-0.9	6.65E-03	-0.9	6.65E-03	-1.7	3.78E-03	-0.8	7.56E-03	-0.7	4.13E-02	-0.7	4.13E-02	-0.9	1.05E-01	-0.7	1.47E-02	-0.1	1.0E+00	-0.1	1.0E+00	-0.2	7.1E-01	-0.1	1.0E+00	-0.1	9.7E-01	-0.2	1.0E+00	0.0	9.7E-01	-0.1	9.5E-01
3339	MSH3	A_24_P203479	NM_002439	-0.9	8.64E-03	-0.6	8.64E-03	-0.9	1.03E-02	-0.9	7.05E-04	-0.7	1.95E-01	-0.7	1.95E-01	-0.2	6.90E-01	-0.7	8.09E-02	-0.1	1.0E+00	0.0	1.0E+00	-0.1	8.3E-01	-0.1	1.0E+00	-0.3	6.3E-01	-0.1	1.0E+00	-0.3	2.7E-01	-0.3	6.3E-01
42872	THC2610658	A_23_P200843	THC2610658	-0.9	1.10E-03	-0.9	7.59E-04	-1.4	1.10E-03	-0.6	2.32E-03	-0.4	1.27E-01	-0.4	2.09E-01	-0.6	1.99E-02	-0.4	1.27E-01	-0.1	1.0E+00	-0.1	1.0E+00	-0.4	7.1E-01	0.0	1.0E+00	0.1	9.4E-01	0.1	1.0E+00	-0.4	5.7E-01	0.2	9.4E-01
19739	TTC15	A_23_P256835	NM_016030	-0.9	2.94E-03	-0.6	2.94E-03	-0.9	7.18E-03	-1.2	2.13E-05	-0.5	1.88E-01	-0.2	7.10E-01	-0.5	1.88E-01	-0.5	7.17E-02	-0.1	1.0E+00	-0.1	1.0E+00	-0.2	7.1E-01	0.0	1.0E+00	0.0	9.9E-01	-0.1	1.0E+00	0.0	9.9E-01	0.1	8.8E-01
21012	GALNAC4S-6ST	A_23_P383986	NM_015892	-0.9	1.15E-02	-0.7	3.48E-02	-1.6	1.15E-02	-0.9	8.62E-03	-0.4	1.62E-01	-0.2	6.22E-01	-0.4	1.62E-01	-0.4	7.69E-02	0.0	1.0E+00	0.0	1.0E+00	0.0	9.9E-01	0.1	1.0E+00	-0.1	9.7E-01	-0.1	1.0E+00	0.0	9.7E-01	-0.2	7.8E-01
16352	LOC641844	A_24_P418780	XR_018036	-0.9	1.13E-02	-0.9	1.68E-02	-1.7	7.11E-03	-0.9	1.13E-02	-0.1	7.29E-01	0.3	7.29E-01	-1.1	1.38E-01	-0.1	9.28E-01	0.0	1.0E+00	0.0	1.0E+00	-0.1	9.4E-01	0.2	1.0E+00	-0.2	8.6E-01	-0.1	1.0E+00	-0.3	6.5E-01	-0.2	8.6E-01
40662	BCL2	A_23_P352266	NM_000633	-0.9	2.00E-03	-0.8	2.38E-03	-1.3	2.00E-03	-0.9	2.56E-04	-0.1	5.60E-01	0.2	5.60E-01	-0.7	6.33E-02	-0.1	7.73E-01	0.0	1.0E+00	0.0	1.0E+00	-0.3	7.1E-01	0.3	1.0E+00	-0.1	9.1E-01	-0.1	1.0E+00	-0.3	3.7E-01	-0.1	9.1E-01
27930	PDE4D	A_24_P944519	NM_006203	-0.9	6.60E-03	-0.9	2.21E-02	-1.7	6.60E-03	-0.9	5.08E-03	-0.4	2.47E-01	-0.3	4.56E-01	-0.4	2.47E-01	-0.7	1.16E-02	0.0	1.0E+00	-0.2	1.0E+00	0.1	8.3E-01	0.0	1.0E+00	-0.1	9.3E-01	-0.1	1.0E+00	0.0	9.3E-01	-0.2	8.7E-01
11182	HMBOX1	A_23_P134684	NM_024567	-0.9	2.65E-02	-0.9	2.65E-02	-1.3	2.74E-02	-0.7	2.15E-02	-0.6	1.66E-01	-0.6	1.46E-01	-0.7	1.66E-01	-0.5	2.78E-01	0.1	1.0E+00	0.1	1.0E+00	0.1	9.0E-01	0.3	1.0E+00	0.0	1.0E+00	0.0	1.0E+00	0.0	1.0E+00	0.1	9.0E-01
13529	THC2693398	A_32_P149716	THC2693398	-0.9	4.71E-04	-0.9	4.71E-04	-1.0	4.13E-03	-0.9	2.08E-04	-0.8	3.01E-01	-0.8	3.01E-01	-0.4	5.54E-01	-0.8	1.75E-01	0.1	1.0E+00	0.2	1.0E+00	0.1	8.2E-01	-0.2	1.0E+00	0.0	9.9E-01	0.2	1.0E+00	-0.2	7.7E-01	0.0	9.9E-01
38743	CCNF	A_24_P193592	NM_001761	-0.9	1.98E-03	-0.9	1.02E-02	-1.7	1.58E-03	-0.8	1.98E-03	-0.2	7.57E-01	-0.1	9.34E-01	-0.8	3.63E-01	-0.2	7.57E-01	-0.1	1.0E+00	-0.1	1.0E+00	-0.5	7.1E-01	0.0	1.0E+00	-0.4	8.9E-01	-0.5	1.0E+00	-0.4	5.2E-01	-0.3	8.9E-01
28819	GCSH	A_23_P117933	NM_004483	-0.9	5.15E-03	-0.9	5.15E-03	-1.1	6.98E-03	-0.9	1.02E-03	-0.4	2.13E-01	-0.4	2.13E-01	-0.3	3.17E-01	-0.6	5.09E-02	0.1	1.0E+00	0.1	1.0E+00	0.2	7.1E-01	0.0	1.0E+00	0.0	1.0E+00	0.0	1.0E+00	0.0	1.0E+00	-0.1	8.4E-01
14876	BCL2	A_23_P352266	NM_000633	-0.9	3.13E-03	-0.7	8.08E-03	-1.5	3.13E-03	-0.9	1.43E-03	-0.2	5.89E-01	0.3	5.89E-01	-0.7	1.00E-01	-0.2	6.41E-01	0.0	1.0E+00	0.0	1.0E+00	-0.3	7.1E-01	0.3	1.0E+00	-0.2	8.8E-01	-0.1	1.0E+00	-0.3	4.1E-01	-0.2	8.8E-01
9613	BAT1	A_24_P904903	NM_004640	-0.9	4.01E-04	-0.7	8.66E-03	-1.8	3.73E-04	-0.9	4.01E-04	-0.3	6.87E-01	0.0	9.83E-01	-0.9	3.75E-01	-0.3	6.87E-01	0.0	1.0E+00	0.0	1.0E+00	-0.1	9.3E-01	0.2	1.0E+00	-0.1	9.8E-01	-0.2	1.0E+00	-0.1	8.9E-01	-0.1	9.8E-01
33419	BCL2	A_23_P352266	NM_000633	-0.9	1.18E-03	-0.6	1.40E-02	-1.5	1.18E-03	-0.9	4.13E-04	-0.2	6.73E-01	0.2	6.73E-01	-0.7	6.48E-02	-0.2	7.01E-01	0.0	1.0E+00	0.0	1.0E+00	-0.3	7.1E-01	0.3	1.0E+00	-0.1	8.9E-01	-0.1	1.0E+00	-0.3	4.8E-01	-0.1	8.9E-01
18836	SR140	A_24_P943106	NM_001080415	-0.9	8.81E-03	-0.9	8.81E-03	-1.5	6.15E-03	-0.7	1.59E-02	-0.9	1.03E-01	-0.6	2.12E-01	-1.7	1.03E-01	-0.9	5.89E-02	-0.1	1.0E+00	-0.1	1.0E+00	-0.1	9.1E-01	0.1	1.0E+00	0.0	9.6E-01	0.0	1.0E+00	0.2	6.4E-01	0.0	9.6E-01
5447	PPT1	A_24_P276628	NM_000310	-0.9	3.36E-03	-0.9	3.06E-02	-1.4	3.36E-03	-0.8	9.91E-04	-0.4	4.03E-01	-0.4	5.48E-01	-0.4	4.03E-01	-0.6	7.89E-02	0.0	1.0E+00	0.0	1.0E+00	0.0	9.8E-01	0.2	1.0E+00	-0.1	9.5E-01	-0.1	1.0E+00	-0.1	9.5E-01	-0.2	9.0E-01
24288	RSRC1	A_32_P34589	NM_016625	-0.9	3.97E-03	-0.8	1.87E-02	-1.6	3.97E-03	-0.9	2.35E-03	-0.8	8.75E-03	-0.6	3.10E-02	-1.6	8.75E-03	-0.8	1.28E-03	-0.2	1.0E+00	0.0	1.0E+00	-0.2	7.1E-01	-0.2	1.0E+00	0.0	9.3E-01	0.0	1.0E+00	0.1	8.2E-01	-0.1	9.3E-01
3679	WDR7	A_23_P164528	NM_015285	-0.9	1.03E-02	-0.7	1.41E-02	-1.3	1.03E-02	-0.9	2.15E-03	0.0	7.73E-01	0.2	7.73E-01	0.0	9.36E-01	-0.2	7.49E-01	0.1	1.0E+00	0.1	1.0E+00	0.1	8.8E-01	0.2	1.0E+00	-0.1	6.5E-01	-0.1	1.0E+00	-0.1	6.5E-01	-0.4	4.0E-01
12990	ST8SIA5	A_23_P379789	NM_013305	-0.9	1.47E-03	-0.6	1.47E-03	-0.9	3.57E-03	-0.9	6.32E-05	-0.4	1.40E-01	-0.1	7.98E-01	-0.4	1.40E-01	-0.4	1.08E-01	0.0	1.0E+00	-0.1	1.0E+00	0.0	9.6E-01	0.1	1.0E+00	-0.2	8.3E-01	-0.2	1.0E+00	-0.2	4.6E-01	-0.1	8.3E-01
16955	ZNF22	A_23_P202458	NM_006963	-0.9	2.76E-03	-0.9	9.99E-04	-0.9	1.24E-02	-0.8	2.76E-03	-1.2	3.68E-02	-0.7	1.09E-01	-1.2	3.68E-02	-1.2	4.19E-03	0.0	1.0E+00	-0.1	1.0E+00	0.2	8.2E-01	0.0	1.0E+00	0.0	9.4E-01	-0.1	1.0E+00	0.2	7.3E-01	0.0	9.4E-01

Feature	Gene symbol	Probe	Transcript	72 hour								48 hours								24 hours								10 hours							
				siCASP8AP2								siCASP8AP2								siCASP8AP2								siCASP8AP2							
				Median	Median	1	1	3	3	6	6	Median	Median	1	1	3	3	6	6	Median	Median	1	1	3	3	6	6	Median	Median	1	1	3	3	6	6
				M	q	M	q	M	q	M	q	M	q	M	q	M	q	M	q	M	q	M	q	M	q	M	q	M	q	M	q	M	q	M	q
7952	MYO1B	A_23_P361049	NM_012223	-0.9	5.43E-03	-0.7	4.41E-02	-1.6	5.43E-03	-0.9	4.56E-03	-0.6	2.18E-01	-0.3	5.42E-01	-0.6	2.18E-01	-0.6	3.23E-02	-0.1	1.0E+00	-0.1	1.0E+00	-0.1	9.1E-01	0.0	1.0E+00	-0.1	9.1E-01	-0.1	1.0E+00	-0.1	9.1E-01	-0.2	8.4E-01
35258	BC029473	A_32_P12065	BC029473	-0.9	1.26E-03	-0.9	1.26E-03	-0.9	1.26E-02	-0.9	1.01E-03	-0.2	7.28E-01	-0.2	7.28E-01	-0.1	9.05E-01	-0.6	1.15E-01	0.0	1.0E+00	0.0	1.0E+00	-0.2	7.1E-01	0.0	1.0E+00	-0.3	7.0E-01	0.0	1.0E+00	-0.3	4.7E-01	-0.3	7.0E-01
4601	DCP1A	A_24_P576174	NM_018403	-0.9	3.35E-03	-0.7	3.35E-03	-1.0	4.11E-03	-0.9	1.30E-04	-0.9	1.25E-02	-0.9	1.93E-02	-1.0	1.25E-02	-0.7	1.07E-02	0.0	1.0E+00	0.0	1.0E+00	0.0	9.3E-01	0.0	1.0E+00	-0.2	5.7E-01	-0.2	1.0E+00	-0.2	5.7E-01	-0.5	4.4E-01
5571	C17orf58	A_24_P49190	NM_181655	-0.9	1.64E-03	-0.9	1.64E-03	-1.0	1.11E-02	-0.8	1.53E-03	-0.9	1.44E-01	-0.6	2.27E-01	-1.1	1.44E-01	-0.9	2.98E-02	-0.2	1.0E+00	-0.2	1.0E+00	-0.2	7.7E-01	-0.2	1.0E+00	0.0	9.6E-01	-0.1	1.0E+00	0.2	7.0E-01	0.0	9.6E-01
36242	SLC36A4	A_23_P86838	NM_152313	-0.9	5.28E-03	-0.7	2.34E-02	-1.4	5.28E-03	-0.9	2.10E-03	-0.2	6.93E-01	-0.2	4.78E-01	0.0	9.70E-01	-0.2	6.93E-01	0.0	1.0E+00	-0.1	1.0E+00	0.0	1.0E+00	0.0	1.0E+00	-0.1	9.0E-01	0.1	1.0E+00	-0.1	8.4E-01	-0.1	9.0E-01
36609	CR614040	A_23_P166502	CR614040	-0.9	3.37E-03	-0.9	1.02E-02	-1.3	3.37E-03	-0.8	8.14E-04	-0.1	9.46E-01	-0.1	9.49E-01	-0.4	6.93E-01	-0.1	9.46E-01	0.0	1.0E+00	0.0	1.0E+00	-0.2	8.3E-01	0.1	1.0E+00	-0.2	9.1E-01	-0.3	1.0E+00	-0.2	6.2E-01	-0.1	9.1E-01
26448	EPRS	A_23_P97632	NM_004446	-0.9	4.25E-03	-0.6	2.12E-02	-1.4	4.25E-03	-0.9	7.11E-04	-0.6	1.15E-01	-0.5	1.15E-01	-1.0	1.83E-01	-0.6	1.01E-02	0.0	1.0E+00	-0.1	1.0E+00	0.0	9.6E-01	0.0	1.0E+00	0.1	9.2E-01	-0.1	1.0E+00	0.1	8.2E-01	0.1	9.2E-01
42978	KRT74	A_23_P379147	NM_175053	-0.9	6.77E-03	-0.9	1.26E-02	-1.2	6.77E-03	-0.8	1.49E-03	-0.7	6.77E-02	-0.7	6.77E-02	-0.7	2.57E-01	-0.6	5.52E-02	0.1	1.0E+00	0.1	1.0E+00	0.0	9.5E-01	0.1	1.0E+00	-0.1	9.6E-01	-0.1	1.0E+00	-0.1	7.9E-01	-0.1	9.6E-01
42232	NUDCD1	A_24_P106794	NM_032869	-0.9	1.42E-02	-0.9	1.09E-02	-1.5	1.42E-02	-0.7	3.76E-02	-0.5	3.71E-01	-0.2	7.59E-01	-0.7	3.71E-01	-0.5	2.69E-01	-0.1	1.0E+00	-0.1	1.0E+00	-0.3	7.4E-01	0.0	1.0E+00	-0.2	6.4E-01	-0.1	1.0E+00	-0.2	6.4E-01	-0.4	5.3E-01
22270	BE540788	A_24_P926935	BE540788	-0.9	8.44E-03	-0.7	8.44E-03	-1.1	1.37E-02	-0.9	1.75E-03	-0.7	2.52E-02	-0.6	3.23E-01	-0.9	1.67E-02	-0.7	2.52E-02	-0.2	1.0E+00	-0.1	1.0E+00	-0.2	7.1E-01	-0.2	1.0E+00	-0.1	8.8E-01	0.0	1.0E+00	-0.1	8.7E-01	-0.2	8.8E-01
12074	BCL2	A_23_P352266	NM_000633	-0.9	3.21E-03	-0.7	1.75E-02	-1.5	3.21E-03	-0.9	1.60E-03	-0.2	4.94E-01	0.3	4.94E-01	-0.6	1.50E-01	-0.2	7.29E-01	0.0	1.0E+00	0.0	1.0E+00	-0.3	7.4E-01	0.3	1.0E+00	-0.2	8.9E-01	-0.1	1.0E+00	-0.3	4.6E-01	-0.2	8.9E-01
6723	EXOSC2	A_23_P216396	NM_014285	-0.9	6.78E-03	-0.9	6.78E-03	-1.1	2.02E-02	-0.8	4.25E-03	-0.3	3.72E-01	-0.3	3.72E-01	-0.3	4.72E-01	-0.8	1.51E-02	-0.1	1.0E+00	-0.1	1.0E+00	-0.2	7.3E-01	0.0	1.0E+00	0.0	9.5E-01	0.1	1.0E+00	0.0	9.5E-01	-0.2	8.3E-01
44377	CNTNAP3	A_24_P418203	NM_033655	-0.9	5.92E-03	-0.9	5.92E-03	-0.9	1.33E-02	-0.9	9.65E-04	-0.4	4.61E-01	-0.2	7.83E-01	-0.5	4.61E-01	-0.4	4.09E-01	-0.1	1.0E+00	0.0	1.0E+00	-0.2	7.3E-01	-0.1	1.0E+00	-0.1	9.3E-01	0.0	1.0E+00	-0.3	2.2E-01	-0.1	9.3E-01
22260	KIAA1211	A_24_P331882	NM_020722	-0.9	8.15E-04	-0.6	1.60E-02	-2.1	8.15E-04	-0.9	6.75E-04	-0.5	1.58E-02	-0.1	7.61E-01	-1.2	1.58E-02	-0.5	1.37E-02	0.0	1.0E+00	0.0	1.0E+00	0.0	1.0E+00	0.2	1.0E+00	-0.2	6.8E-01	0.0	1.0E+00	-0.2	5.9E-01	-0.2	6.8E-01
19235	GJB1	A_23_P250444	NM_000166	-0.9	3.02E-03	-1.3	3.02E-03	-0.9	5.38E-03	-0.7	2.93E-04	-0.9	1.17E-01	-0.9	2.89E-02	-0.6	1.63E-01	-0.9	1.17E-01	-0.2	1.0E+00	-0.3	1.0E+00	-0.2	7.1E-01	-0.1	1.0E+00	0.1	8.5E-01	0.1	1.0E+00	0.1	8.5E-01	0.1	8.3E-01
38421	PAK1IP1	A_23_P122674	NM_017906	-0.9	3.13E-03	-0.9	3.13E-03	-0.6	2.22E-02	-1.3	2.77E-05	-1.1	5.26E-02	-1.1	5.26E-02	-1.0	2.68E-01	-1.4	1.17E-02	-0.4	1.0E+00	-0.2	1.0E+00	-0.4	7.4E-01	-0.6	1.0E+00	0.1	9.6E-01	0.1	1.0E+00	0.2	7.7E-01	0.1	9.6E-01
20129	EIF2S2	A_24_P48791	NM_003908	-0.9	3.73E-03	-0.9	3.73E-03	-1.0	5.31E-03	-0.8	3.19E-04	-0.5	2.27E-01	-0.5	2.27E-01	-0.6	1.98E-01	-0.4	2.72E-01	-0.1	1.0E+00	-0.1	1.0E+00	-0.2	7.1E-01	0.0	1.0E+00	-0.1	9.6E-01	-0.1	1.0E+00	-0.1	8.1E-01	0.0	9.6E-01
23366	DPH5	A_23_P148821	NM_001077394	-0.9	1.08E-03	-0.8	1.08E-03	-0.9	5.05E-03	-1.2	8.56E-06	-0.5	1.34E-01	-0.2	5.63E-01	-0.5	1.34E-01	-0.8	7.53E-03	0.1	1.0E+00	0.0	1.0E+00	0.1	8.1E-01	0.1	1.0E+00	0.0	9.7E-01	-0.1	1.0E+00	0.0	9.7E-01	0.0	9.5E-01
43385	PCGF5	A_23_P202117	BC007377	-0.9	9.71E-04	-0.8	1.23E-02	-1.8	9.71E-04	-0.9	8.63E-04	-0.5	8.71E-02	-0.5	8.71E-02	-0.9	2.66E-01	-0.4	6.93E-02	0.0	1.0E+00	0.0	1.0E+00	0.1	8.8E-01	-0.1	1.0E+00	0.0	9.9E-01	0.0	1.0E+00	0.0	9.9E-01	-0.1	9.5E-01
23914	IVD	A_23_P129313	ENST00000249760	-0.9	1.73E-02	-0.9	1.73E-02	-0.9	1.79E-02	-0.9	7.05E-04	-0.5	3.00E-01	-0.6	3.00E-01	-0.4	4.41E-01	-0.5	2.74E-01	0.0	1.0E+00	0.2	1.0E+00	0.0	9.3E-01	-0.1	1.0E+00	0.2	7.2E-01	0.1	1.0E+00	0.2	7.0E-01	0.3	7.2E-01
43114	SPG11	A_23_P65699	NM_025137	-0.9	4.11E-03	-0.9	4.11E-03	-0.8	4.17E-02	-1.2	2.97E-04	-0.8	2.34E-01	-0.4	2.38E-01	-0.8	2.34E-01	-0.8	1.26E-02	-0.1	1.0E+00	-0.1	1.0E+00	-0.2	7.1E-01	-0.1	1.0E+00	-0.1	8.5E-01	-0.1	1.0E+00	-0.2	4.2E-01	-0.1	8.5E-01
3474	APBB2	A_24_P234701	NM_173075	-0.9	2.11E-03	-0.9	2.47E-03	-1.2	2.11E-03	-0.8	4.70E-04	-0.4	3.00E-01	-0.3	6.12E-01	-0.6	2.56E-01	-0.4	3.00E-01	-0.1	1.0E+00	-0.1	1.0E+00	-0.3	7.1E-01	-0.1	1.0E+00	-0.1	8.9E-01	-0.1	1.0E+00	-0.1	8.7E-01	-0.1	8.9E-01
3404	CSRP1	A_24_P225448	NM_004078	-0.9	1.85E-03	-0.9	3.22E-02	-1.7	8.46E-04	-0.6	1.85E-03	-0.2	8.51E-01	-0.4	5.98E-01	-0.2	8.51E-01	-0.2	8.67E-01	0.2	1.0E+00	0.2	1.0E+00	0.1	9.1E-01	0.2	1.0E+00	0.0	9.9E-01	-0.1	1.0E+00	0.0	9.8E-01	0.0	9.9E-01
35169	SELT	A_23_P250042	NM_016275	-0.9	5.19E-03	-0.9	6.53E-03	-1.1	5.19E-03	-0.7	1.37E-03	-0.6	2.73E-01	-0.4	3.56E-01	-1.2	2.73E-01	-0.6	2.15E-02	0.1	1.0E+00	0.1	1.0E+00	0.1	8.1E-01	0.0	1.0E+00	0.0	9.4E-01	0.0	1.0E+00	0.0	9.4E-01	-0.2	8.6E-01
27876	SCOC	A_23_P167293	NM_032547	-0.9	1.25E-02	-0.9	1.25E-02	-1.1	2.14E-02	-0.7	1.25E-02	-0.3	3.85E-01	-0.3	3.85E-01	0.1	8.18E-01	-0.4	2.67E-01	-0.1	1.0E+00	-0.1	1.0E+00	-0.2	8.0E-01	-0.1	1.0E+00	-0.2	8.8E-01	-0.2	1.0E+00	-0.1	8.8E-01	-0.4	8.3E-01
26511	THC2497780	A_32_P66625	THC2497780	-0.9	6.22E-03	-0.8	7.77E-03	-1.5	6.22E-03	-0.9	2.43E-03	-0.6	1.23E-01	-0.2	7.89E-01	-0.6	1.23E-01	-0.8	1.95E-02	0.0	1.0E+00	0.1	1.0E+00	0.0	9.9E-01	-0.1	1.0E+00	-0.1	9.9E-01	-0.2	1.0E+00	0.0	9.9E-01	-0.1	8.2E-01
13761	SCCPDH	A_23_P62807	NM_016002	-0.9	3.54E-03	-0.7	3.69E-02	-2.4	1.26E-03	-0.9	3.54E-03	-0.5	5.21E-02	-0.1	8.50E-01	-1.2	9.55E-03	-0.5	5.21E-02	-0.1	1.0E+00	-0.1	1.0E+00	-0.2	7.1E-01	0.0	1.0E+00	0.0	9.3E-01	0.0	1.0E+00	0.0	9.3E-01	-0.1	9.3E-01
45182	OXR1	A_23_P256244	AK096148	-0.9	3.82E-04	-0.9	9.25E-04	-1.9	3.78E-04	-0.8	3.82E-04	-0.7	2.27E-02	-0.7	2.27E-02	-1.5	9.90E-02	-0.7	3.71E-03	-0.1	1.0E+00	-0.3	1.0E+00	-0.1	8.2E-01	-0.1	1.0E+00	-0.2	5.0E-01	-0.2	1.0E+00	-0.2	3.2E-01	-0.2	5.0E-01
25564	NMNAT2	A_23_P354908	NM_015039	-0.9	1.03E-03	-1.0	7.33E-04	-0.8	1.87E-02	-0.9	1.03E-03	-0.7	6.62E-02	-0.2	7.21E-01	-0.8	6.62E-02	-0.7	2.77E-02	-0.1	1.0E+00	-0.1	1.0E+00	-0.3	7.1E-01	0.0	1.0E+00	-0.1	6.7E-01	-0.1	1.0E+00	-0.1	6.7E-01	-0.2	5.6E-01
17073	EXOC2	A_23_P214354	NM_018303	-0.9	1.55E-02	-1.1	1.55E-02	-0.9	3.20E-02	-0.9	1.66E-03	-1.0	7.11E-02	-1.0	5.17E-02	-1.1	2.19E-01	-0.8	7.11E-02	-0.2	7.1E-01	-0.5	3.4E-01	-0.2	7.1E-01	0.0	1.0E+00	-0.2	7.1E-01	-0.2	1.0E+00	-0.2	4.9E-01	-0.2	7.1E-01
11276	CTDSPL	A_23_P212436	NM_001008392	-0.9	8.93E-03	-1.0	8.93E-03	-0.9	1.62E-02	-0.7	2.76E-03	-0.7	1.16E-01	-0.6	1.16E-01	-1.3	2.09E-01	-0.7	2.86E-02	0.0	1.0E+00	0.1	1.0E+00	0.0	9.4E-01	0.0	1.0E+00	0.2	5.0E-01	0.1	1.0E+00	0.2	5.0E-01	0.3	4.9E-01
27344	SHPRH	A_23_P337790	NM_001042683	-0.9	6.71E-03	-0.9	9.49E-03	-1.4	6.71E-03	-0.7	5.17E-03	-0.8	3.13E-01	-0.4	3.67E-01	-0.9	3.13E-01	-0.8	2.26E-02	0.0	1.0E+00	0.0	1.0E+00	0.0	9.7E-01	0.0	1.0E+00	-0.1	9.5E-01	-0.2	1.0E+00	0.1	8.0E-01	-0.1	9.5E-01
40343	CLASP2	A_23_P44295	NM_015097	-0.9	6.53E-03	-0.6	2.50E-02	-1.4	6.53E-03	-0.9	3.74E-03	-0.5	1.48E-01	-0.5	3.09E-01	-0.6	1.48E-01	-0.4	6.70E-02	0.0	1.0E+00	0.0	1.0E+00	0.0	1.0E+00	-0.1	1.0E+00	-0.1	9.1E-01	-0.1	1.0E+00	-0.1	9.1E-01	-0.3	8.4E-01
3851	THC2548755	A_32_P132276	THC2548755	-0.9	1.26E-03	-0.9	1.63E-03	-1.8	1.10E-03	-0.8	1.26E-03	-0.5	2.12E-01	-0.4	3.61E-01	-1.0	3.47E-02	-0.5	2.12E-01	0.1	1.0E+00	0.1	1.0E+00	0.0	9.8E-01	0.3	1.0E+00	-0.2	3.8E-01	0.0	1.0E+00	-0.2	3.8E-01	-0.4	3.3E-01
2244	TDG	A_32_P218249	NM_003211	-0.9	6.76E-04	-0.9	6.76E-04	-1.6	1.01E-03	-0.9	2.34E-04	-0.8	3.18E-02	-0.8	3.18E-02	-1.3	3.27E-02	-0.8	1.49E-02	-0.1	1.0E+00	-0.1	1.0E+00	-0.2	7.7E-01	0.1	1.0E+00	-0.3	4.2E-01	-0.1	1.0E+00	-0.3	3.5E-01	-0.4	4.2E-01
4796	AF116624	A_32_P327810	AF116624	-0.9	1.25E-02	-0.9	1.25E-02	-1.1	2.34E-02	-0.8	7.98E-03	-0.8	1.51E-01	-0.7	3.14E-01	-1.0	9.83E-02	-0.8	1.51E-01	0.2	1.0E+00	0.2	1.0E+00	0.2	7.1E-01	0.0	1.0E+00	0.0	1.0E+00	0.0	1.0E+00	0.0	1.0E+00	0.0	9.9E-01
40907	GCOM1	A_24_P116378	NM_001018090	-0.9	4.60E-04	-1.5	1.64E-04	-0.9	8.73E-03	-0.9	4.60E-04	-1.0	7.48E-02	-1.0	1.93E-02	-1.0	1.24E-01	-1.1	7.48E-02	-0.2	7.3E-01	-0.5	6.7E-01	-0.2	7.3E-01	-0.1	1.0E+00	-0.2	8.7E-01	-0.3	1.0E+00	-0.1	8.6E-01	-0.2	8.7E-01
3605	TM2D3	A_23_P140515	NM_078474	-0.9	2.96E-03	-0.9	2.96E-03	-1.1	8.76E-03	-0.7	1.43E-03	-0.7	8.48E-02	-0.7	8.48E-02	-0.6	1.00E-01	-0.9	6.06E-03	0.0	1.0E+00	-0.2	1.0E+00	0.0	9.9E-01	0.1	1.0E+00	-0.1	5.7E-01	-0.1	1.0E+00	-0.1	5.7E-01	-0.3	3.9E-01
37749	TARBP1	A_23_P52058	NM_005646	-0.9	2.43E-02	-0.9	2.43E-02	-0.9	2.62E-02	-0.8	2.97E-03	-0.9	4.65E-02	-1.0	5.02E-02	-0.9	4.65E-02	-0.8	1.49E-02	-0.3	1.0E+00	-0.2	1.0E+00	-0.3	7.1E-01	-0.3	1.0E+00	0.3	7.0E-01	0.1	1.0E+00	0.3	4.6E-01	0.3	7.0E-01
36334	SEC22C	A_23_P502641	NM_032970	-0.9	1.25E-02	-0.7	1.25E-02	-1.0	2.11E-02	-0.9	1.68E-03	-0.4	3.64E-01	-0.4	4.09E-01	-1.2	2.57E-01	-0.3	3.64E-01	-0.1	1.0E+00	-0.1	1.0E+00	-0.2	7.7E-01	-0.1	1.0E+00	-0.1	8.8E-01	0.0	1.0E+00	-0.2	6.6E-01	-0.1	8.8E-01
9865	DNA2	A_24_P366107	NM_001080449	-0.9	2.02E-03	-0.8	4.79E-03	-1.7	2.02E-03	-0.9	1.89E-03	-0.4	3.63E-01	-0.3	5.20E-01	-0.4	3.63E-01	-0.6	6.67E-02	-0.1	1.0E+00	0.0	1.0E+00	-0.2	7.1E-01	-0.1	1.0E+00	-0.1	7.2E-01	0.0	1.0E+00	-0.1	7.2E-01	-0.2	6.9E-01
32091	NFS1	A_23_P102842	NM_021100	-0.9	3.79E-03	-0.9	3.79E-03	-0.9	1.49E-02	-1.3	7.20E-05	-0.4	2.84E-01	-0.2	7.12E-01	-0.4	2.84E-01	-0.6	8.65E-03	0.0	1.0E+00	0.0	1.0E+00	0.0	9.9E-01	0.0	1.0E+00	0.0	9.0E-01	0.0	1.0E+00	-0.1	8.8E-01	0.1	9.0E-01
7361	DSG2	A_23_P141730	NM_001943	-0.9	4.49E-04	-0.8	1.54E-02	-1.6	4.49E-04	-0.9	9.68E-05	-0.4	3.97E-01	-0.3	6.47E-01	-0.8	3.41E-01	-0.4	3.97E-01	-0.1	1.0E+00	0.0	1.0E+00	-0.1	9.2E-01	-0.1	1.0E+00	0.1	9.5E-01	-0.1	1.0E+00	0.1	9.5E-01	0.2	9.0E-01
26093	C15orf52	A_23_P163475	NM_207380	-0.9	4.17E-02	-0.9	4.17E-02	-1.0	4.97E-02	-0.7	1.73E-02	-0.9	2.77E-02	-0.8	4.76E-02	-1.4	2.77E-02	-0.9	2.70E-02	-0.1	1.0E+00	0.1	1.0E+00	-0.2	7.1E-01	-0.1	1.0E+00	0.1	8.1E-01	0.0	1.0E+00	0.1	8.1E-01	0.2	7.1E-01
27562	ALDH5A1	A_24_P923353	NM_170740	-0.9	4.56E-03	-0.8	4.56E-03	-1.0	7.60E-03	-0.9	6.98E-04	0.0	9.75E-01	0.0	9.75E-01	0.3	5.99E-01	0.0	9.93E-01	0.1	1.0E+00	0.1	1.0E+00	-0.4	7.1E-01	0.1	1.0E+00	-0.1	9.8E-01	-0.2	1.0E+00	0.1	8.5E-01	-0.1	9.8E-01
35378	PXMP2	A_23_P124122	NM_018663	-0.9	9.83E-04	-0.9	9.83E-04	-0.8	1.13E-02	-1.3	3.53E-05	-0.4	2.86E-01	-0.3	4.98E-01	-0.4	2.86E-01	-0.9	1.11E-02	0.0	1.0E+00	0.0	1.0E+00	0.0	9.5E-01	-0.3	1.0E+00	0.1	9.3E-01	0.0	1.0E+00	0.1	8.2E-01	0.1	9.3E-01
9887	PDE4D	A_24_P419351	NM_006203	-0.9	5.36E-03	-0.9	1.49E-03	-0.9	1.07E-02	-0.7	5.36E-03	-0.1	9.11E-01	-0.1	9.54E-01	-0.1	9.11E-01	-0.4	4.58E-01	0.1	1.0E+00	0.2	1.0E+00	-0.3	7.4E-01	0.1	1.0E+00	-0.2	1.0E+00	-0.3	1.0E+00	-0.2	7.9E-01	0.0	1.0E+00
19198	ABCB10	A_32_P23624	NM_012089	-0.9	2.46E-03	-0.8	2.46E-03	-1.3	3.76E-03	-0.9	1.29E-03	-0.7	2.17E-01	-0.4	3.38E-01	-0.8	2.17E-01	-0.7	3.65E-02	-0.2	1.0E+00	-0.2	1.0E+00	-0.2	7.4E-01	-0.1	1.0E+00	0.0	9.9E-01	-0.2	1.0E+00	0.0	9.3E-01	0.0	9.9E-01
16494	VAPA	A_23_P207957	NM_003574	-0.9	8.31E-03	-0.9	8.14E-03	-1.3	8.85E-03	-0.6	8.31E-03	-0.6	2.32E-01	-0.6	2.32E-01	-0.6	2.33E-01	-0.7	1.28E-01	0.1	1.0E+00	0.0	1.0E+00	0.1	8.0E-01	0.1	1.0E+00	0.0	9.1E-01	0.0	1.0E+00	0.1	7.6E-01	-0.1	9.1E-01
32048	ZAK	A_23_P366394	NM_016653	-0.9	8.34E-04	-0.7	2.53E-03	-1.1	8.34E-04	-0.9	2.33E-05	-0.3	4.40E-01	-0.3	4.40E-01	-0.3	5.89E-01	-0.9	9.91E-03	-0.1	1.0E+00	-0.1	1.0E+00	-0.1	7.4E-01	-0.1	1.0E+00	-0.1	8.4E-01	0.0	1.0E+00	-0.1	8.4E-01	-0.2	7.7E-01
31095	SCMH1	A_23_P12477	NM_012236	-0.9	6.75E-03	-0.8	1.86E-02	-1.5	6.75E-03	-0.9	2.49E-03	-0.5	4.06E-01	-0.5	4.06E-01	-1.2	1.21E-01	-0.4	5.03E-01	0.0	1.0E+00	0.0	1.0E+00	-0.1	7.9E-01	0.1	1.0E+00	0.0	9.7E-01	-0.1	1.0E+00	0.0	9.7E-01	0.1	9.0E-01
3697	CR591764	A_32_P102252	CR591764	-0.9	1.48E-02	-0.9	1.65E-02	-1.3	1.44E-02	-0.7	1.48E-02	-0.7	1.37E-01	-0.7	1.37E-01	-1.1	1.51E-01	-0.7	2.63E-02	-0.1	1.0E+00	-0.1	1.0E+00	-0.2	7.1E-01	0.0	1.0E+00	-0.1	9.2E-01	-0.1	1.0E+00	0.0	9.1E-01	-0.1	9.2E-01
34804	SAA4	A_23_P87238	NM_006512	-0.9	1.34E-03	-1.3	6.18E-04	-0.8	4.41E-02	-0.9	1.34E-03	-0.7	8.52E-02	-0.5	3.47E-01	-0.8	5.43E-02	-0.7	8.52E-02	0.0	1.0E+00	0.0	1.0E+00	-0.3	7.1E-01	0.1	1.0E+00	0.0	1.0E+00	0.0	1.0E+00	0.0	1.0E+00	0.0	9.6E-01
36157	ZNF767	A_23_P323143	NM_024910	-0.9	1.62E-02	-0.6	1.65E-02	-1.0	1.62E-02	-0.9	9.71E-04	-0.5	3.02E-01	-0.5	4.23E-01	-0.9	1.28E-01	-0.5	3.02E-01	0.0	1.0E+00	0.0	1.0E+00	-0.2	7.3E-01	0.0	1.0E+00	0.0	9.7E-01	0.0	1.0E+00	0.0	9.7E-01	0.3	4.4E-01
44437	ENST00000361534	A_24_P408341	ENST00000361534	-0.9	4.41E-03	-0.9	4.41E-03	-0.9	3.39E-02	-0.8	2.54E-03	-0.6	9.90E-02	-0.4	3.74E-01	-0.6	9.90E-02	-0.6	3.63E-02	0.0	1.0E+00	0.0	1.0E+00	-0.1	8.0E-01	0.0	1.0E+00	-0.3	5.3E-01	0.0	1.0E+00	-0.3	3.6E-01	-0.3	5.3E-01
42560	MYO5C	A_23_P140434	NM_018728	-0.9	2.40E-02	-0.9	2.40E-02	-1.6	8.58E-03	-0.8	2.51E-02	-0.7	2.42E-01	-0.3	2.42E-01	-0.7	2.60E-01	-0.9	1.84E-02	0.0	1.0E+00	-0.1	1.0E+00	0.0	9.6E-01	0.0	1.0E+00	-0.2	9.9E-01	-0.2	1.0E+00	0.0	9.9E-01	-0.3	7.3E-01
15014	EWSR1	A_32_P207054	NM_013986	-0.9	7.41E-03	-0.6	3.81E-02	-1.0	7.41E-03	-0.9	2.34E-03	-0.3	4.23E-01	0.3	7.10E-01	-0.4	4.23E-01	-0.3	3.60E-01	0.1	1.0E+00	0.1	1.0E+00	0.0	9.6E-01	0.1	1.0E+00	0.2	9.2E-01	-0.1	1.0E+00	0.2	7.7E-01	0.2	9.2E-01
39315	FASN	A_23_P44132	NM_004104	-0.9	2.97E-03	-0.9	4.56E-02	-1.8	1.41E-03	-0.7	2.97E-03	-0.5	4.72E-01	-0.5	3.53E-01	-0.7	4.72E-01	-0.2	7.86E-01	0.1	1.0E+00	0.2	1.0E+00	0.0	9.6E-01	0.1	1.0E+00	0.2	7.9E-01	0.2	1.0E+00	0.2	7.0E-01	0.3	7.9E-01
12232	METTL5	A_23_P142634	NM_014168	-0.9	2.31E-03	-0.7	4.50E-03	-1.4	2.31E-03	-0.9	8.01E-04	-0.5	2.55E-02	-0.4	1.55E-01	-0.9	2.10E-02	-0.5	2.55E-02	0.0	1.0E+00	0.0	1.0E+00	0.0	1.0E+00	-0.1	1.0E+00	0.1	1.0E+00	0.1	1.0E+00	0.1	8.6E-01	0.0	1.0E+00
42126	DDX10	A_23_P32938	NM_004398	-0.9	5.75E-03	-0.7	8.81E-03	-1.1	5.75E-03	-0.9	6.39E-04	-0.6	1.10E-02	-0.6	2.42E-02	-1.3	1.14E-03	-0.6	1.10E-02	-0.2	1.0E+00	-0.2	1.0E+00	-0.4	3.7E-01	-0.2	1.0E+00	-0.2	7.1E-01	-0.1	1.0E+00	-0.2	4.0E-01	-0.2	7.1E-01
13139	PRMT6	A_23_P12336	NM_018137	-0.9	1.34E-02	-0.8	1.34E-02	-1.1	2.52E-02	-0.9	5.26E-03	-0.6	3.03E-01	-0.4	5.47E-01	-0.7	3.03E-01	-0.6	7.64E-02	-0.1	1.0E+00	-0.2	1.0E+00	-0.1	7.5E-01	-0.1	1.0E+00	-0.1	9.3E-01	-0.1	1.0E+00	0.0	9.3E-01	-0.1	9.3E-01
34042	A_24_P641673	A_24_P641673	A_24_P641673	-0.9	2.75E-03	-0.7	6.51E-03	-1.3	2.75E-03	-0.9	1.60E-03	-0.3	4.81E-01	-0.3	4.81E-01	-0.4	4.81E-01	-0.3	3.98E-01	-0.2	1.0E+00	0.1	1.0E+00	-0.2	7.3E-01	-0.2	1.0E+00	0.1	9.1E-01	-0.3	1.0E+00	0.1	9.1E-01	0.3	8.3E-01
43933	PAK1IP1	A_23_P122674	NM_017906	-0.9	4.17E-03	-0.9	4.17E-03	-0.9	1.03E-02	-1.3	4.38E-05	-1.1	2.79E-02	-1.1	2.79E-02	-1.1	1.89E-01	-1.5	3.14E-03	-0.3	1.0E+00	-0.2	1.0E+00	-0.3	7.9E-01	-0.4	1.0E+00	0.0	9.5E-01	0.0	1.0E+00	0.2	8.3E-01	-0.1	9.5E-01
5988	IMP4	A_23_P131518	CR621399	-0.9	9.42E-03	-0.9	9.42E-03	-0.8	3.71E-02	-0.9	1.66E-03	-0.6	2.13E-01	-0.4	2.13E-01	-0.6	4.48E-01	-0.6	3.71E-02	-0.2	1.0E+00	-0.2	1.0E+00	-0.2	7.1E-01	0.0	1.0E+00	-0.1	9.7E-01	-0.1	1.0E+00	0.1	9.0E-01	-0.1	9.7E-01
31173	A_24_P341593	A_24_P341593	A_24_P341593	-0.9	9.58E-03	-0.6	4.15E-02	-1.4	9.58E-03	-0.9	5.36E-03	-0.1	9.02E-01	-0.1	9.55E-01	-0.4	6.18E-01	-0.1	9.02E-01	-0.1	1.0E+00	-0.1	1.0E+00	-0.2	7.1E-01	0.0	1.0E+00	0.0	9.7E-01	0.0	1.0E+00	0.0	9.7E-01	-0.1	9.1E-01
2397	PAK1IP1	A_23_P122674	NM_017906	-0.9	1.16E-02	-0.9	1.16E-02	-0.9	1.58E-02	-1.3	7.60E-05	-1.1	2.58E-02	-1.1	2.58E-02	-1.0	2.02E-01	-1.6	2.61E-03	-0.3	1.0E+00	-0.1	1.0E+00	-0.3	7.9E-01	-0.4	1.0E+00	0.1	9.7E-01	0.1	1.0E+00	0.2	7.8E-01	-0.1	9.7E-01
4687	WHSC1	A_23_P7301	NM_133334	-0.9	1.06E-02	-0.7	1.06E-02	-1.0	1.32E-02	-0.9	2.23E-03	-0.6	3.04E-01	-0.5	4.96E-01	-0.9	1.66E-01	-0.6	3.04E-01	0.0	1.0E+00	0.0	1.0E+00	-0.4	7.1E-01	0.0	1.0E+00	-0.1	7.9E-01	0.0	1.0E+00	-0.1	6.6E-01	-0.2	7.9E-01
42718	C16orf63	A_24_P193509	NM_144600	-0.9	5.72E-04	-0.9	4.28E-04	-1.3	7.10E-04	-0.7	5.72E-04	-0.4	4.50E-01	-0.3	7.44E-01	-0.5	3.85E-01	-0.4	4.50E-01	-0.1	1.0E+00	0.0	1.0E+00	-0.3	7.1E-01	-0.1	1.0E+00	-0.3	6.2E-01	0.0	1.0E+00	-0.3	2.7E-01	-0.3	6.2E-01
42884	LOC401397	A_32_P54305	NR_024412	-0.9	3.49E-03	-0.8	8.02E-03	-1.7	2.98E-03	-0.9	3.49E-03	-0.4	1.74E-01	-0.3	1.74E-01	-0.5	1.97E-01	-0.4	1.72E-01	-0.1	1.0E+00	-0.1	1.0E+00	-0.1	8.2E-01	-0.1	1.0E+00	-0.2	5.2E-01	-0.1	1.0E+00	-0.2	5.2E-01	-0.3	5.2E-01
28555	DPY19L4	A_24_P481824	NM_181787	-0.9	2.48E-02	-0.9	7.59E-03	-1.0	2.84E-02	-0.6	2.48E-02	0.0	8.15E-01	0.2	8.15E-01	-0.3	5.26E-01	0.0	9.53E-01	-0.1	1.0E+00	-0.1	1.0E+00	-0.2	8.2E-01	0.3	1.0E+00	-0.4	4.4E-01	-0.2	1.0E+00	-0.4	2.7E-01	-0.4	4.4E-01
10535	DTYMK	A_23_P135364	NM_012145	-0.9	9.37E-03	-0.9	9.37E-03	-0.8	3.97E-02	-0.9	1.18E-03	-0.7	5.56E-02	-0.6	8.62E-02	-0.7	5.56E-02	-0.8	1.62E-02	-0.1	1.0E+00	-0.1	1.0E+00	-0.1	7.9E-01	-0.2	1.0E+00	0.1	8.3E-01	0.0	1.0E+00	0.1	6.5E-01	0.1	8.3E-01
1165	BCL11A	A_23_P218584	NM_022893	-0.9	1.27E-03	-0.9	1.27E-03	-1.1	5.59E-03	-0.8	8.43E-04	-0.5	5.89E-02	-0.5	5.89E-02	-0.4	1.73E-01	-0.6	1.17E-02	-0.3	1.0E+00	-0.3	1.0E+00	-0.4	6.7E-01	-0.3	1.0E+00	-0.3	1.8E-01	-0.1	1.0E+00	-0.3	1.6E-01	-0.4	1.8E-01
40262	CRBN	A_24_P351435	NM_016302	-0.9	1.58E-03	-0.9	1.58E-03	-1.4	3.69E-04	-0.7	3.67E-03	-0.8	8.50E-02	-0.8	1.24E-02	-1.1	1.07E-01	-0.5	8.50E-02	-0.1	1.0E+00	-0.1	1.0E+00	-0.1	7.5E-01	-0.1	1.0E+00	-0.1	8.0E-01	0.0	1.0E+00	-0.1	8.0E-01	-0.3	5.3E-01
28202	THC2737176	A_32_P111019	THC2737176	-0.9	1.37E-02	-0.9	1.40E-02	-1.5	1.37E-02	-0.9	1.32E-02	-0.5	5.35E-01	-0.4	7.01E-01	-0.7	4.27E-01	-0.5	5.35E-01	0.0	1.0E+00	0.0	1.0E+00	-0.3	7.5E-01	0.0	1.0E+00	-0.1	9.8E-01	-0.3	1.0E+00	0.0	9.8E-01	-0.1	9.2E-01
44245	A_24_P229658	A_24_P229658	A_24_P229658	-0.9	3.80E-02	-0.7	2.85E-02	-1.0	4.48E-02	-0.9	3.80E-02	-0.8	1.25E-01	-0.5	5.03E-01	-1.0	1.25E-01	-0.8	1.59E-02	-0.2	1.0E+00	-0.1	1.0E+00	-0.2	8.2E-01	-0.2	1.0E+00	0.1	9.6E-01	0.1	1.0E+00	0.1	9.6E-01	-0.3	9.0E-01
33632	SNX24	A_23_P19095	NM_014035	-0.9	6.51E-03	-0.9	6.51E-03	-1.4	6.98E-03	-0.8	3.12E-03	-0.7	4.03E-02	-0.6	1.49E-01	-1.5	2.04E-02	-0.7	4.03E-02	0.0	1.0E+00	0.1	1.0E+00	-0.4	4.7E-01	0.0	1.0E+00	-0.2	7.5E-01	0.0	1.0E+00	-0.4	2.3E-01	-0.2	7.5E-01
10808	SNHG7	A_24_P352116	NR_003672	-0.9	5.07E-03	-0.9	5.07E-03	-0.9	8.51E-03	-0.7	1.74E-03	-0.8	1.79E-02	-0.8	2.40E-02	-0.8	1.79E-02	-0.9	2.91E-03	-0.1	1.0E+00	-0.1	1.0E+00	-0.1	7.6E-01	-0.1	1.0E+00	0.3	8.8E-01	0.3	1.0E+00	-0.1	8.8E-01	0.3	7.4E-01
27828	PTPN2	A_23_P309701	NM_002828	-0.9	2.94E-03	-0.9	2.65E-03	-0.9	2.63E-02	-0.7	2.94E-03	-0.8	5.65E-02	-0.3	4.55E-01	-0.8	5.65E-02	-0.8	2.83E-02	0.0	1.0E+00	-0.1	1.0E+00	0.0	9.1E-01	0.0	1.0E+00	0.1	1.0E+00	0.1	1.0E+00	0.2	6.7E-01	0.0	1.0E+00
4352	TGFB1I1	A_23_P141055	NM_001042454	-0.9	1.98E-02	-0.7	1.98E-02	-1.0	3.86E-02	-0.9	4.59E-03	-0.4	4.80E-01	-0.2	8.61E-01	-0.7	3.68E-01	-0.4	4.80E-01	0.0	1.0E+00	0.3	1.0E+00	0.0	9.7E-01	0.0	1.0E+00	-0.1	8.2E-01	-0.1	1.0E+00	-0.1	8.2E-01	-0.3	7.9E-01
40442	RNF138	A_23_P89755	NM_016271	-0.9	2.50E-03	-0.9	4.00E-03	-1.7	1.84E-03	-0.7	2.50E-03	-0.7	1.37E-01	-0.8	6.49E-02	-0.7	3.49E-01	-0.7	1.37E-01	0.1	1.0E+00	-0.2	1.0E+00	0.1	8.7E-01	0.1	1.0E+00	-0.2	7.8E-01	-0.2	1.0E+00	0.1	7.8E-01	-0.2	7.7E-01
12482	ERI2	A_24_P934800	NM_001142725	-0.9	1.54E-03	-0.6	6.29E-03	-1.3	1.54E-03	-0.9	1.61E-04	-0.7	1.50E-01	-0.2	6.02E-01	-1.3	1.50E-01	-0.7	5.77E-03	0.0	1.0E+00	0.0	1.0E+00	-0.1	8.1E-01	0.0	1.0E+00	0.0	8.9E-01	0.0	1.0E+00	0.0	8.9E-01	-0.1	6.9E-01
36278	C12orf66	A_24_P68294	BC036246	-0.9	2.34E-03	-0.8	2.34E-03	-1.0	1.04E-02	-0.9	6.81E-04	-0.5	3.41E-01	-0.2	7.19E-01	-0.5	3.41E-01	-0.5	1.01E-01	0.0	1.0E+00	0.0	1.0E+00	-0.3	7.1E-01	0.0	1.0E+00	-0.3	1.7E-01	-0.1	1.0E+00	-0.3	1.7E-01	-0.5	1.5E-01
36335	DEPDC1	A_24_P25872	NM_017779	-0.9	1.09E-03	-0.9	1.07E-03	-1.7	1.09E-03	-0.8	7.27E-03	-0.5	9.84E-02	-0.4	2.51E-01	-0.9	9.84E-02	-0.5	3.91E-02	-0.3	1.0E+00	-0.3	1.0E+00	-0.3	7.1E-01	-0.2	1.0E+00	-0.3	3.2E-01	-0.1	1.0E+00	-0.3	2.8E-01	-0.4	3.2E-01
38346	C20orf177	A_24_P6428	NM_022106	-0.9	1.37E-02	-0.9	1.37E-02	-1.0	1.92E-02	-0.7	1.02E-02	-0.3	6.50E-01	-0.2	8.28E-01	-0.5	3.43E-01	-0.3	6.50E-01	0.0	1.0E+00	0.0	1.0E+00	-0.3	7.1E-01	0.0	1.0E+00	-0.1	9.3E-01	0.1	1.0E+00	-0.2	4.9E-01	-0.1	9.3E-01
37037	KIAA0776	A_23_P30956	NM_015323	-0.9	5.69E-03	-0.7	1.01E-02	-1.4	5.69E-03	-0.9	1.74E-03	-0.6	1.30E-01	-0.1	8.20E-01	-0.6	1.30E-01	-0.7	1.08E-01	0.1	1.0E+00	0.1	1.0E+00	0.1	8.1E-01	0.1	1.0E+00	-0.2	4.7E-01	-0.1	1.0E+00	-0.2	4.7E-01	-0.5	1.9E-01
19342	AK123506	A_32_P193218	AK123506	-0.9	1.88E-02	-0.9	1.83E-02	-1.1	4.17E-02	-0.7	1.88E-02	-0.5	2.93E-01	-0.3	6.82E-01	-0.6	2.75E-01	-0.5	2.93E-01	0.2	1.0E+00	0.2	1.0E+00	0.2	8.4E-01	0.1	1.0E+00	0.0	9.8E-01	0.2	1.0E+00	-0.1	8.6E-01	0.0	9.8E-01
2960	KLRC2	A_23_P22232	NM_002260	-0.9	2.23E-03	-0.9	3.46E-03	-2.2	7.27E-04	-0.7	2.23E-03	-0.5	1.38E-01	-0.3	5.76E-01	-0.7	1.25E-01	-0.5	1.38E-01	0.0	1.0E+00	-0.2	1.0E+00	0.0	9.3E-01	0.1	1.0E+00	0.4	9.1E-01	0.4	1.0E+00	0.5	7.7E-01	0.4	9.1E-01
40788	TAF1B	A_23_P165891	NM_005680	-0.9	1.97E-02	-0.8	1.97E-02	-1.0	4.06E-02	-0.9	6.13E-03	-0.7	4.18E-02	-0.7	4.18E-02	-0.4	2.09E-01	-0.8	2.44E-02	-0.3	1.0E+00	0.0	1.0E+00	-0.4	7.1E-01	-0.3	1.0E+00	-0.1	9.7E-01	0.1	1.0E+00	-0.1	9.4E-01	-0.1	9.7E-01
43250	KIAA0182	A_24_P943062	NM_014615	-0.8	3.17E-02	-0.6	3.43E-02	-1.2	2.06E-02	-0.8	3.17E-02	-0.3	6.04E-01	-0.3	6.69E-01	-0.7	3.90E-01	-0.3	6.04E-01	-0.1	1.0E+00	-0.1	1.0E+00	-0.3	7.1E-01	0.1	1.0E+00	-0.4	1.9E-01	-0.2	1.0E+00	-0.4	1.2E-01	-0.4	1.9E-01
45198	PDCD2	A_23_P145153	NM_002598	-0.8	2.85E-03	-0.8	2.85E-03	-1.0	4.61E-03	-0.8	1.06E-03	-0.6	3.88E-02	-0.6	3.88E-02	-0.9	7.18E-02	-0.6	6.19E-03	-0.1	1.0E+00	0.0	1.0E+00	-0.2	7.1E-01	-0.1	1.0E+00	0.0	1.0E+00	0.1	1.0E+00	0.0	9.4E-01	0.0	1.0E+00
6692	TMEM163	A_23_P39550	NM_030923	-0.8	1.03E-02	-0.7	3.02E-02	-1.4	1.03E-02	-0.8	3.68E-03	-0.4	3.03E-01	-0.3	3.89E-01	-1.0	3.03E-01	-0.4	3.23E-02	-0.1	1.0E+00	-0.1	1.0E+00	-0.1	8.3E-01	0.0	1.0E+00	-0.1	9.4E-01	-0.1	1.0E+00	-0.1	9.0E-01	0.1	9.4E-01
42707	A_32_P234853	A_32_P234853	A_32_P234853	-0.8	2.12E-03	-0.8	2.12E-03	-0.6	3.12E-02	-0.9	2.43E-04	-0.1	7.41E-01	-0.1	8.22E-01	0.2	7.41E-01	-0.6	6.94E-02	0.0	1.0E+00	0.1	1.0E+00	-0.3	7.1E-01	0.0	1.0E+00	-0.1	9.2E-01	0.1	1.0E+00	-0.2	5.1E-01	-0.1	9.2E-01
29752	C7orf25	A_24_P300483	NM_024054	-0.8	1.22E-02	-0.8	2.92E-02	-1.4	1.22E-02	-0.8	1.16E-02	-0.3	3.98E-01	-0.1	8.33E-01	-0.3	3.98E-01	-0.5	4.56E-02	-0.1	1.0E+00	-0.1	1.0E+00	-0.1	8.8E-01	0.1	1.0E+00	-0.1	9.8E-01	-0.1	1.0E+00	0.0	9.8E-01	-0.1	9.3E-01
12971	BBS4	A_23_P99967	NM_033028	-0.8	1.71E-02	-0.8	1.71E-02	-0.8	4.70E-02	-1.4	1.36E-04	-0.7	1.74E-01	-0.7	1.74E-01	-0.3	6.85E-01	-1.0	1.37E-02	-0.2	1.0E+00	-0.2	1.0E+00	-0.1	9.2E-01	-0.3	1.0E+00	0.0	9.8E-01	0.1	1.0E+00	-0.1	8.4E-01	0.0	9.8E-01
10485	THC2595726	A_32_P98098	THC2595726	-0.8	1.46E-02	-0.7	2.08E-02	-1.6	6.08E-03	-0.8	1.46E-02	-0.5	3.35E-01	-0.5	3.35E-01	-0.3	5.49E-01	-0.6	1.05E-01	0.0	1.0E+00	0.2	1.0E+00	-0.1	8.7E-01	0.0	1.0E+00	0.1	9.7E-01	0.3	1.0E+00	0.1	9.5E-01	0.1	9.7E-01
6317	LYRM7	A_32_P211141	NM_181705	-0.8	9.40E-03	-0.8	6.31E-03	-1.3	9.40E-03	-0.7	9.91E-03	-0.5	3.51E-01	-0.4	4.68E-01	-0.6	1.65E-01	-0.5	3.51E-01	0.0	1.0E+00	0.0	1.0E+00	0.0	9.9E-01	0.2	1.0E+00	-0.1	9.5E-01	0.0	1.0E+00	-0.1	9.5E-01	-0.3	8.3E-01
40344	AK091178	A_24_P178834	AK091178	-0.8	3.00E-02	-0.8	3.00E-02	-0.9	3.83E-02	-0.8	9.59E-03	-0.5	2.47E-01	-0.4	2.81E-01	-0.5	2.47E-01	-0.7	3.46E-02	0.1	1.0E+00	0.1	1.0E+00	0.1	7.7E-01	-0.1	1.0E+00	0.2	8.9E-01	-0.2	1.0E+00	0.3	5.8E-01	0.2	8.9E-01
36816	BX116014	A_32_P223327	BX116014	-0.8	1.03E-03	-0.8	1.03E-03	-1.1	3.01E-03	-0.7	4.21E-04	-0.6	1.33E-01	-0.3	3.94E-01	-1.0	8.08E-02	-0.6	1.33E-01	-0.1	1.0E+00	-0.1	1.0E+00	-0.2	7.1E-01	-0.1	1.0E+00	-0.1	8.5E-01	0.0	1.0E+00	-0.1	7.5E-01	-0.1	8.5E-01
15146	A_24_P238266	A_24_P238266	A_24_P238266	-0.8	6.52E-03	-0.8	1.86E-02	-1.2	4.80E-03	-0.6	6.52E-03	-0.7	2.99E-01	-0.7	1.01E-01	-1.0	2.99E-01	-0.3	4.15E-01	0.0	1.0E+00	0.0	1.0E+00	0.0	9.9E-01	0.1	1.0E+00	0.1	8.7E-01	0.1	1.0E+00	0.1	8.5E-01	0.2	8.7E-01
7338	SFRS16	A_24_P920748	AK074590	-0.8	1.34E-03	-0.8	1.34E-03	-0.8	9.53E-03	-0.9	3.91E-04	-1.0	5.98E-02	-1.0	4.69E-02	-0.9	5.98E-02	-1.0	8.89E-02	0.0	1.0E+00	0.0	1.0E+00	0.2	8.4E-01	0.0	1.0E+00	0.2	9.1E-01	0.4	1.0E+00	0.2	7.3E-01	0.1	9.1E-01
43404	DDX54	A_23_P162374	NM_024072	-0.8	1.04E-03	-0.8	1.04E-03	-0.8	1.33E-02	-1.3	5.39E-05	-0.7	4.52E-02	-0.4	1.41E-01	-0.8	4.25E-02	-0.7	4.52E-02	0.0	1.0E+00	-0.1	1.0E+00	0.1	8.7E-01	0.0	1.0E+00	0.2	8.0E-01	0.1	1.0E+00	0.3	2.7E-01	0.2	8.0E-01
32702	APOBEC3C	A_23_P120931	NM_014508	-0.8	4.87E-03	-0.8	6.77E-03	-1.2	4.86E-03	-0.6	4.87E-03	-0.5	3.64E-01	-0.5	3.64E-01	-0.5	5.28E-01	-0.4	2.92E-01	0.2	1.0E+00	0.2	1.0E+00	0.2	7.3E-01	-0.2	1.0E+00	0.1	9.7E-01	0.0	1.0E+00	0.1	9.2E-01	0.1	9.7E-01
44017	LYRM7	A_24_P256603	NM_181705	-0.8	7.71E-03	-0.8	1.73E-02	-1.5	7.71E-03	-0.8	5.45E-03	-0.7	1.01E-01	-0.4	5.30E-01	-0.7	1.01E-01	-0.9	4.37E-02	0.1	1.0E+00	-0.2	1.0E+00	0.1	9.3E-01	0.2	1.0E+00	-0.2	9.2E-01	-0.2	1.0E+00	0.1	9.2E-01	-0.2	9.1E-01
45020	GTF3C2	A_24_P316363	NM_001521	-0.8	5.81E-03	-0.8	2.16E-02	-1.6	2.84E-03	-0.6	5.81E-03	-0.4	4.60E-01	-0.4	4.60E-01	-0.6	3.78E-01	-0.4	4.93E-01	0.1	1.0E+00	0.1	1.0E+00	0.0	9.6E-01	0.2	1.0E+00	-0.1	8.8E-01	-0.1	1.0E+00	-0.1	7.8E-01	-0.2	8.8E-01
24405	TTC35	A_23_P60002	NM_014673	-0.8	4.80E-03	-0.8	4.80E-03	-1.1	1.85E-02	-0.8	2.62E-03	-0.8	1.56E-01	-0.5	1.56E-01	-0.8	2.40E-01	-0.8	1.13E-02	0.0	1.0E+00	-0.1	1.0E+00	0.0	9.8E-01	0.1	1.0E+00	-0.1	9.9E-01	-0.1	1.0E+00	0.0	9.9E-01	-0.2	7.2E-01
33937	DGKH	A_24_P220575	BC044822	-0.8	1.09E-03	-0.8	5.88E-03	-1.8	1.09E-03	-0.8	1.09E-03	-0.5	1.52E-01	-0.2	6.27E-01	-0.8	5.75E-02	-0.5	1.52E-01	-0.1	1.0E+00	-0.1	1.0E+00	-0.2	7.2E-01	0.0	1.0E+00	0.0	9.7E-01	0.0	1.0E+00	0.0	9.7E-01	0.1	9.4E-01
42019	CINP	A_23_P88134	NM_032630	-0.8	5.23E-03	-0.6	8.97E-03	-1.3	5.23E-03	-0.8	2.97E-03	-0.3	5.91E-01	-0.2	8.45E-01	-0.5	3.54E-01	-0.3	5.91E-01	-0.1	1.0E+00	0.0	1.0E+00	-0.3	7.1E-01	-0.1	1.0E+00	0.0	8.5E-01	0.0	1.0E+00	0.1	8.5E-01	-0.2	7.8E-01
39597	POLK	A_24_P303160	NM_016218	-0.8	4.10E-03	-0.9	2.58E-03	-0.8	2.03E-02	-0.6	4.10E-03	-0.4	2.76E-01	-0.4	2.76E-01	-0.4	3.94E-01	-0.4	1.46E-01	-0.2	1.0E+00	-0.2	1.0E+00	-0.2	7.2E-01	0.1	1.0E+00	-0.2	4.6E-01	-0.2	1.0E+00	-0.2	4.6E-01	-0.4	3.2E-01

Feature	Gene symbol	Probe	Transcript	72 hour								48 hours								24 hours								10 hours							
				siCASP8AP2								siCASP8AP2								siCASP8AP2								siCASP8AP2							
				Median	Median	1	1	3	3	6	6	Median	Median	1	1	3	3	6	6	Median	Median	1	1	3	3	6	6	Median	Median	1	1	3	3	6	6
				M	q	M	q	M	q	M	q	M	q	M	q	M	q	M	q	M	q	M	q	M	q	M	q	M	q	M	q	M	q	M	q
4136	TTC37	A_24_P93353	NM_014639	-0.8	2.84E-03	-0.8	2.84E-03	-0.8	1.42E-02	-0.9	2.20E-04	-0.2	7.07E-01	-0.2	7.07E-01	0.1	8.98E-01	-0.5	1.86E-01	-0.1	1.0E+00	0.1	1.0E+00	-0.2	7.9E-01	-0.1	1.0E+00	-0.2	7.3E-01	-0.2	1.0E+00	-0.2	5.2E-01	-0.3	7.3E-01
6069	A_24_P41483	A_24_P41483	A_24_P41483	-0.8	3.08E-03	-0.6	7.91E-03	-1.6	1.78E-03	-0.8	3.08E-03	-0.1	8.90E-01	0.1	9.32E-01	-0.5	4.77E-01	-0.1	8.90E-01	0.0	1.0E+00	0.0	1.0E+00	-0.4	7.1E-01	0.2	1.0E+00	-0.3	8.2E-01	-0.2	1.0E+00	-0.3	6.2E-01	-0.3	8.2E-01
36959	ROBO2	A_24_P180151	NM_002942	-0.8	3.65E-03	-0.8	3.65E-03	-1.1	1.11E-02	-0.7	3.63E-03	-0.3	5.66E-01	-0.1	8.24E-01	-0.3	5.66E-01	-0.3	3.98E-01	0.0	1.0E+00	0.0	1.0E+00	-0.2	7.6E-01	0.1	1.0E+00	-0.3	1.4E-01	-0.1	1.0E+00	-0.3	1.1E-01	-0.4	1.4E-01
40288	ATP5S	A_23_P37296	NM_015684	-0.8	1.40E-03	-0.8	1.40E-03	-0.8	1.21E-02	-0.8	4.83E-04	-0.5	1.61E-01	-0.4	1.61E-01	-0.5	1.64E-01	-0.9	2.92E-03	-0.1	1.0E+00	-0.1	1.0E+00	-0.1	7.6E-01	0.0	1.0E+00	0.0	1.0E+00	0.2	1.0E+00	0.0	1.0E+00	-0.1	9.7E-01
41112	EBPL	A_23_P128554	NM_032565	-0.8	4.90E-03	-0.8	6.48E-03	-1.5	4.90E-03	-0.8	3.50E-03	-0.9	8.50E-02	-0.6	1.94E-01	-0.9	8.50E-02	-0.9	1.74E-02	0.0	1.0E+00	0.0	1.0E+00	0.0	9.4E-01	-0.1	1.0E+00	0.0	9.4E-01	0.1	1.0E+00	0.0	9.4E-01	-0.1	8.7E-01
12723	XPO7	A_23_P146237	NM_015024	-0.8	1.07E-02	-0.7	1.69E-02	-0.9	1.07E-02	-0.8	3.44E-04	-0.2	8.10E-01	-0.2	8.10E-01	0.1	9.59E-01	-0.2	7.22E-01	-0.1	1.0E+00	0.1	1.0E+00	-0.4	7.1E-01	-0.1	1.0E+00	0.2	9.1E-01	0.2	1.0E+00	-0.1	9.1E-01	0.3	8.2E-01
21576	MRPL19	A_23_P102262	NM_014763	-0.8	3.12E-03	-0.8	1.24E-02	-1.9	1.64E-03	-0.7	3.12E-03	-0.7	1.52E-02	-0.5	5.93E-02	-1.1	1.52E-02	-0.7	8.31E-03	-0.1	1.0E+00	0.0	1.0E+00	-0.2	7.1E-01	-0.1	1.0E+00	-0.1	7.4E-01	0.0	1.0E+00	-0.1	6.3E-01	-0.2	7.4E-01
37185	INPP5E	A_24_P317835	NM_019892	-0.8	4.47E-03	-0.8	5.36E-03	-1.0	4.47E-03	-0.7	3.79E-04	-0.6	7.54E-02	-0.7	7.54E-02	-0.6	5.66E-02	-0.6	1.43E-01	0.0	1.0E+00	0.0	1.0E+00	0.1	9.2E-01	0.0	1.0E+00	0.0	1.0E+00	0.1	1.0E+00	-0.1	8.6E-01	0.0	1.0E+00
6399	THAP8	A_24_P296907	NM_152658	-0.8	1.80E-02	-0.8	1.80E-02	-0.7	3.52E-02	-1.0	1.52E-04	-0.6	2.52E-01	-0.2	8.38E-01	-0.6	2.52E-01	-0.7	7.31E-02	0.0	1.0E+00	0.0	1.0E+00	-0.1	8.6E-01	0.1	1.0E+00	-0.3	6.4E-01	0.0	1.0E+00	-0.3	3.6E-01	-0.3	6.4E-01
43885	AK095738	A_32_P20221	AK095738	-0.8	5.81E-03	-0.8	5.81E-03	-0.9	1.52E-02	-0.8	5.58E-04	-0.4	4.46E-02	-0.3	3.00E-01	-0.6	2.53E-02	-0.4	4.46E-02	0.0	1.0E+00	0.0	1.0E+00	-0.1	8.4E-01	0.0	1.0E+00	-0.1	8.8E-01	0.1	1.0E+00	-0.1	8.8E-01	-0.1	8.8E-01
18876	UBXN2B	A_24_P222591	NM_001077619	-0.8	4.00E-04	-1.0	4.00E-04	-0.7	9.91E-03	-0.8	1.00E-04	-0.4	2.07E-01	-0.4	2.07E-01	-0.2	5.01E-01	-0.6	1.65E-01	-0.2	1.0E+00	-0.2	1.0E+00	-0.2	7.7E-01	0.1	1.0E+00	-0.1	9.2E-01	-0.1	1.0E+00	-0.1	8.6E-01	-0.1	9.2E-01
22503	TDG	A_23_P204579	NM_003211	-0.8	4.75E-03	-0.8	9.14E-03	-1.8	2.00E-03	-0.7	4.75E-03	-0.7	2.20E-02	-0.7	4.49E-02	-0.9	7.95E-03	-0.6	2.20E-02	-0.2	1.0E+00	-0.2	1.0E+00	-0.2	7.1E-01	-0.1	1.0E+00	-0.1	9.6E-01	-0.1	1.0E+00	0.0	9.2E-01	-0.1	9.6E-01
42813	TARDBP	A_24_P276703	NM_007375	-0.8	3.06E-02	-0.8	4.25E-02	-1.1	1.55E-02	-0.6	3.06E-02	-0.8	2.88E-01	-0.8	1.26E-02	-1.2	4.00E-01	-0.5	2.88E-01	0.1	1.0E+00	-0.4	1.0E+00	0.1	8.5E-01	0.1	1.0E+00	-0.2	9.4E-01	-0.3	1.0E+00	0.1	9.4E-01	-0.2	8.2E-01
12726	A_24_P922120	A_24_P922120	A_24_P922120	-0.8	1.75E-02	-0.8	6.54E-03	-0.9	3.12E-02	-0.6	1.75E-02	-0.5	2.06E-01	-0.4	4.79E-01	-0.7	1.78E-01	-0.5	2.06E-01	0.0	1.0E+00	0.0	1.0E+00	-0.3	7.1E-01	0.0	1.0E+00	0.0	9.9E-01	0.1	1.0E+00	0.0	9.8E-01	0.0	9.9E-01
18888	CCDC3	A_24_P369232	NM_031455	-0.8	2.72E-03	-0.8	3.86E-03	-1.6	2.72E-03	-0.8	1.51E-03	-0.9	1.86E-03	-0.7	1.68E-02	-1.4	1.86E-03	-0.9	9.30E-04	-0.2	1.0E+00	-0.2	1.0E+00	-0.5	8.2E-02	-0.1	1.0E+00	-0.1	9.3E-01	-0.1	1.0E+00	-0.2	3.8E-01	-0.1	9.3E-01
33020	PSMD14	A_23_P165691	NM_005805	-0.8	3.80E-03	-0.9	3.80E-03	-0.8	2.49E-02	-0.7	2.82E-03	-0.5	2.68E-01	-0.4	2.68E-01	-0.5	3.20E-01	-0.6	5.20E-02	0.0	1.0E+00	0.0	1.0E+00	0.0	9.3E-01	0.1	1.0E+00	0.1	8.6E-01	0.0	1.0E+00	0.1	8.6E-01	0.1	8.5E-01
38778	DTWD2	A_23_P346982	NM_173666	-0.8	1.46E-03	-0.8	1.46E-03	-0.9	7.44E-03	-0.8	2.22E-04	-0.5	2.98E-01	-0.4	4.58E-01	-0.5	2.98E-01	-0.6	9.90E-02	0.0	1.0E+00	0.0	1.0E+00	0.0	9.1E-01	-0.1	1.0E+00	-0.3	4.4E-01	0.0	1.0E+00	-0.3	1.8E-01	-0.3	4.4E-01
20064	DGUOK	A_24_P285623	NM_080916	-0.8	3.70E-03	-0.8	3.47E-03	-0.9	1.63E-02	-0.8	3.70E-03	-0.7	3.14E-02	-0.7	7.49E-02	-0.6	3.14E-02	-0.8	1.90E-02	-0.2	1.0E+00	-0.2	1.0E+00	-0.2	7.6E-01	-0.2	1.0E+00	0.0	9.9E-01	0.0	1.0E+00	0.1	9.2E-01	0.0	9.9E-01
7609	C6orf66	A_24_P171983	NM_014165	-0.8	1.54E-02	-0.8	1.91E-02	-1.5	1.54E-02	-0.8	1.09E-02	-0.5	4.90E-02	-0.5	1.51E-01	-0.8	2.38E-02	-0.5	4.90E-02	-0.3	1.0E+00	-0.3	1.0E+00	-0.3	7.1E-01	-0.2	1.0E+00	-0.1	9.4E-01	0.0	1.0E+00	-0.1	9.0E-01	-0.1	9.4E-01
37308	A_32_P186678	A_32_P186678	A_32_P186678	-0.8	8.89E-03	-0.8	8.89E-03	-1.3	4.41E-03	-0.6	9.36E-03	-0.2	6.30E-01	-0.2	5.62E-01	-0.4	6.30E-01	-0.1	8.32E-01	0.3	1.0E+00	0.3	1.0E+00	0.2	8.1E-01	0.4	1.0E+00	0.1	9.8E-01	0.2	1.0E+00	0.1	9.1E-01	0.0	9.8E-01
44346	CB049993	A_32_P92212	CB049993	-0.8	5.71E-03	-0.8	1.56E-03	-1.1	6.80E-03	-0.6	5.71E-03	-0.5	1.76E-01	-0.3	2.77E-01	-0.7	1.06E-01	-0.5	1.76E-01	0.0	1.0E+00	0.0	1.0E+00	-0.1	8.9E-01	0.2	1.0E+00	-0.4	2.6E-01	-0.2	1.0E+00	-0.4	2.0E-01	-0.5	2.6E-01
35222	POLR3E	A_23_P152181	NM_018119	-0.8	4.28E-03	-0.8	4.28E-03	-0.8	1.33E-02	-1.0	5.53E-04	-0.5	9.48E-02	-0.4	3.88E-01	-0.7	9.48E-02	-0.5	3.90E-02	-0.2	1.0E+00	-0.2	1.0E+00	-0.2	7.1E-01	-0.3	1.0E+00	0.1	9.8E-01	0.1	1.0E+00	0.0	9.8E-01	0.2	8.9E-01
4653	SLC25A15	A_23_P128650	AF112968	-0.8	1.53E-03	-0.8	1.53E-03	-0.7	3.17E-02	-1.3	2.34E-05	-0.9	1.09E-02	-0.5	1.04E-01	-1.1	1.09E-02	-0.9	1.93E-03	-0.3	1.0E+00	-0.1	1.0E+00	-0.3	7.1E-01	-0.3	1.0E+00	0.0	9.6E-01	0.1	1.0E+00	-0.1	7.5E-01	0.0	9.6E-01
275	POLE2	A_23_P163099	NM_002692	-0.8	5.73E-03	-0.8	1.17E-02	-1.8	2.03E-03	-0.7	5.73E-03	-0.5	4.12E-01	-0.2	6.92E-01	-0.8	4.12E-01	-0.5	2.13E-01	0.2	1.0E+00	0.2	1.0E+00	0.1	8.1E-01	0.2	1.0E+00	0.0	9.3E-01	0.2	1.0E+00	0.0	9.3E-01	-0.2	7.2E-01
15664	CDH13	A_32_P85999	NM_001257	-0.8	1.84E-02	-0.7	3.88E-02	-1.4	1.84E-02	-0.8	1.77E-02	-0.6	5.62E-02	0.2	3.66E-01	-0.7	5.62E-02	-0.6	3.67E-02	-0.1	1.0E+00	-0.1	1.0E+00	-0.2	8.1E-01	0.0	1.0E+00	-0.2	5.6E-01	-0.1	1.0E+00	-0.2	5.6E-01	-0.4	4.2E-01
16433	PAK1IP1	A_23_P122674	NM_017906	-0.8	6.99E-03	-0.8	6.99E-03	-0.8	3.62E-02	-1.3	3.05E-04	-1.0	8.69E-02	-1.0	8.69E-02	-0.9	2.92E-01	-1.4	1.54E-02	-0.3	1.0E+00	-0.2	1.0E+00	-0.3	7.9E-01	-0.4	1.0E+00	0.1	9.8E-01	0.1	1.0E+00	0.3	6.4E-01	0.1	9.8E-01
43373	KIAA0090	A_23_P97553	D42044	-0.8	7.11E-03	-0.8	6.23E-03	-1.1	1.16E-02	-0.6	7.11E-03	-0.4	4.38E-01	0.0	9.55E-01	-0.4	4.38E-01	-0.4	2.06E-01	0.0	1.0E+00	0.0	1.0E+00	-0.3	7.1E-01	0.1	1.0E+00	-0.2	8.7E-01	0.2	1.0E+00	-0.3	6.3E-01	-0.2	8.7E-01
34294	RASSF8	A_24_P407224	AY665468	-0.8	2.68E-02	-0.8	4.05E-02	-1.4	8.90E-03	-0.6	2.68E-02	-0.7	1.47E-01	-0.7	5.04E-02	-1.3	1.47E-01	-0.4	2.33E-01	-0.1	1.0E+00	-0.2	1.0E+00	-0.1	7.8E-01	0.1	1.0E+00	-0.1	9.8E-01	-0.2	1.0E+00	0.0	9.8E-01	-0.1	9.1E-01
17910	HNRPDL	A_23_P213153	NM_031372	-0.8	3.50E-02	-0.8	3.50E-02	-1.4	1.86E-02	-0.7	4.53E-02	-0.7	1.31E-01	-0.6	1.31E-01	-1.1	1.96E-01	-0.7	3.28E-02	0.1	1.0E+00	-0.1	1.0E+00	0.1	8.8E-01	0.1	1.0E+00	0.0	9.9E-01	-0.2	1.0E+00	0.1	8.4E-01	0.0	9.9E-01
15738	SLC38A6	A_23_P88309	NM_153811	-0.8	9.41E-03	-0.8	1.23E-02	-1.3	8.97E-03	-0.7	9.41E-03	-0.4	1.89E-01	-0.4	1.89E-01	-0.2	4.76E-01	-0.7	4.50E-02	0.1	1.0E+00	0.1	1.0E+00	0.2	7.1E-01	0.1	1.0E+00	0.0	9.2E-01	0.1	1.0E+00	0.0	9.2E-01	-0.2	7.9E-01
5616	USP14	A_24_P245330	AK098497	-0.8	7.09E-04	-0.7	2.98E-02	-2.2	6.52E-05	-0.8	7.09E-04	-0.6	1.41E-01	-0.4	3.51E-01	-1.5	1.41E-01	-0.6	8.29E-02	0.1	1.0E+00	0.1	1.0E+00	0.0	9.6E-01	0.2	1.0E+00	0.0	9.2E-01	0.0	1.0E+00	0.1	8.6E-01	-0.2	9.2E-01
32826	C6orf182	A_24_P189112	NM_173830	-0.8	2.27E-02	-0.6	2.27E-02	-0.9	3.89E-02	-0.8	2.62E-03	-0.4	8.43E-02	-0.3	2.68E-01	-0.4	8.43E-02	-0.6	5.87E-03	0.0	1.0E+00	0.0	1.0E+00	0.0	9.3E-01	0.1	1.0E+00	-0.2	7.1E-01	-0.2	1.0E+00	-0.1	7.1E-01	-0.3	4.2E-01
16270	TTC37	A_23_P61854	ENST00000358746	-0.8	1.27E-02	-0.8	1.27E-02	-1.0	4.28E-02	-0.8	8.28E-03	-0.8	1.04E-01	-0.3	6.41E-01	-0.9	1.04E-01	-0.8	5.45E-02	0.0	1.0E+00	-0.1	1.0E+00	0.0	9.7E-01	0.0	1.0E+00	0.0	9.3E-01	0.0	1.0E+00	0.3	3.5E-01	-0.1	9.3E-01
43566	FAM104B	A_23_P415061	NM_138362	-0.8	3.64E-03	-0.8	3.64E-03	-0.9	9.47E-03	-0.7	5.63E-04	-0.6	1.69E-01	-0.5	3.31E-01	-0.6	1.69E-01	-0.7	6.19E-02	0.0	1.0E+00	0.0	1.0E+00	-0.1	8.3E-01	0.1	1.0E+00	-0.1	9.2E-01	0.0	1.0E+00	-0.2	7.3E-01	-0.1	9.2E-01
30689	BAG2	A_23_P356554	NM_004282	-0.8	8.08E-04	-0.8	8.08E-04	-1.0	1.62E-03	-0.7	1.19E-04	-0.5	2.36E-01	-0.2	5.81E-01	-0.6	2.36E-01	-0.5	1.15E-01	0.0	1.0E+00	0.0	1.0E+00	0.1	8.5E-01	0.0	1.0E+00	0.0	9.0E-01	0.0	1.0E+00	0.0	9.0E-01	-0.1	8.8E-01
25477	GPAM	A_24_P227069	NM_020918	-0.8	1.32E-02	-0.6	1.32E-02	-1.0	1.63E-02	-0.8	1.61E-03	-0.6	1.28E-01	-0.3	4.57E-01	-0.8	1.28E-01	-0.6	3.75E-02	0.0	1.0E+00	-0.1	1.0E+00	0.0	9.3E-01	0.0	1.0E+00	0.1	1.0E+00	0.1	1.0E+00	0.1	7.3E-01	0.0	1.0E+00
36897	TIMELESS	A_24_P231004	NM_003920	-0.8	3.65E-04	-0.9	3.65E-04	-0.8	5.70E-03	-0.8	7.73E-05	-0.7	1.66E-01	-0.8	1.66E-01	-0.4	4.34E-01	-0.7	9.49E-02	-0.2	1.0E+00	0.1	1.0E+00	-0.3	7.1E-01	-0.2	1.0E+00	0.0	9.7E-01	0.0	1.0E+00	-0.2	5.2E-01	0.0	9.7E-01
8014	CD46	A_23_P35230	NM_172350	-0.8	7.92E-03	-0.7	3.40E-02	-1.9	3.01E-03	-0.8	7.92E-03	-0.3	3.48E-01	-0.3	4.61E-01	-0.3	3.48E-01	-0.4	2.01E-01	-0.1	1.0E+00	-0.1	1.0E+00	-0.1	7.6E-01	-0.1	1.0E+00	0.1	8.1E-01	0.1	1.0E+00	0.2	7.8E-01	-0.3	8.1E-01
23341	KIAA1549	A_32_P103508	NM_020910	-0.8	4.51E-02	-0.8	4.51E-02	-1.3	4.54E-02	-0.8	3.84E-02	-0.5	3.47E-01	-0.1	8.88E-01	-0.6	3.09E-01	-0.5	3.47E-01	-0.1	1.0E+00	-0.1	1.0E+00	-0.5	7.1E-01	-0.1	1.0E+00	0.0	9.8E-01	0.1	1.0E+00	0.0	9.8E-01	0.0	9.8E-01
14612	C14orf94	A_23_P65455	NM_017815	-0.8	2.10E-03	-0.8	2.10E-03	-0.8	2.14E-02	-0.7	1.92E-03	-0.3	3.99E-01	-0.2	6.23E-01	-0.3	3.99E-01	-0.6	1.05E-01	-0.1	1.0E+00	-0.2	1.0E+00	-0.1	7.1E-01	0.0	1.0E+00	-0.1	8.9E-01	-0.2	1.0E+00	-0.1	8.9E-01	-0.1	8.8E-01
29855	EXOSC8	A_24_P241276	NM_181503	-0.8	1.12E-02	-0.9	1.12E-02	-0.8	3.64E-02	-0.8	1.23E-03	-0.6	5.95E-02	-0.6	5.95E-02	-0.4	6.95E-02	-0.8	6.57E-03	0.1	1.0E+00	0.0	1.0E+00	0.1	7.9E-01	0.1	1.0E+00	-0.1	7.0E-01	0.0	1.0E+00	-0.1	6.8E-01	-0.2	7.0E-01
6603	HNRNPH3	A_23_P115824	NM_012207	-0.8	1.07E-03	-0.8	2.39E-02	-1.3	7.85E-04	-0.7	1.07E-03	-0.7	5.23E-02	-0.7	1.44E-02	-0.8	5.23E-02	-0.5	1.99E-01	0.2	1.0E+00	0.1	1.0E+00	0.2	8.1E-01	0.2	1.0E+00	0.0	9.0E-01	0.1	1.0E+00	0.0	8.7E-01	-0.1	9.0E-01
19288	PAK1IP1	A_23_P122674	NM_017906	-0.8	6.60E-03	-0.8	6.60E-03	-0.8	1.61E-02	-1.3	3.83E-05	-1.1	1.92E-02	-1.1	1.92E-02	-1.1	7.81E-02	-1.5	4.95E-03	-0.3	1.0E+00	-0.2	1.0E+00	-0.3	7.6E-01	-0.5	1.0E+00	0.1	9.7E-01	0.1	1.0E+00	0.3	6.4E-01	0.1	9.7E-01
12610	SNAPC5	A_23_P383977	NM_006049	-0.8	2.71E-02	-0.7	2.71E-02	-1.0	4.68E-02	-0.8	7.37E-03	-0.2	6.32E-01	-0.2	6.69E-01	-0.2	6.32E-01	-0.5	3.53E-02	0.0	1.0E+00	-0.1	1.0E+00	0.0	9.7E-01	0.1	1.0E+00	-0.1	9.8E-01	-0.1	1.0E+00	-0.1	8.2E-01	0.0	9.8E-01
6227	C1orf67	A_23_P333951	NM_144989	-0.8	4.84E-03	-0.7	8.51E-03	-1.5	4.84E-03	-0.8	3.45E-03	-0.8	7.13E-02	-0.6	7.13E-02	-0.9	8.15E-02	-0.8	1.15E-02	0.1	1.0E+00	0.0	1.0E+00	0.1	7.7E-01	0.1	1.0E+00	-0.1	8.5E-01	-0.1	1.0E+00	-0.1	8.5E-01	-0.2	5.7E-01
29769	FAM173B	A_23_P133279	NM_199133	-0.8	4.35E-03	-0.8	4.78E-03	-1.5	4.35E-03	-0.7	3.45E-03	-0.7	1.09E-01	-0.3	4.21E-01	-0.7	1.09E-01	-0.7	2.02E-02	0.0	1.0E+00	-0.1	1.0E+00	0.0	9.6E-01	0.0	1.0E+00	0.0	9.2E-01	0.0	1.0E+00	0.0	9.2E-01	-0.2	8.3E-01
22272	ATP5SL	A_24_P219769	NM_018035	-0.8	2.19E-03	-0.7	2.34E-02	-1.4	2.19E-03	-0.8	1.06E-03	-0.2	7.62E-01	-0.1	9.07E-01	-0.6	4.69E-01	-0.2	7.62E-01	0.1	1.0E+00	0.1	1.0E+00	-0.1	9.1E-01	0.1	1.0E+00	-0.1	9.9E-01	-0.1	1.0E+00	-0.1	9.1E-01	0.0	9.9E-01
38806	A_24_P16230	A_24_P16230	A_24_P16230	-0.8	3.81E-03	-0.7	1.77E-02	-1.3	3.81E-03	-0.8	7.45E-04	-0.4	3.25E-01	-0.2	6.10E-01	-1.3	3.25E-01	-0.4	1.25E-01	0.2	1.0E+00	0.1	1.0E+00	0.2	7.3E-01	0.2	1.0E+00	0.1	9.8E-01	0.1	1.0E+00	0.1	8.2E-01	0.0	9.8E-01
42712	SAA4	A_23_P87238	NM_006512	-0.8	1.26E-03	-1.5	1.77E-04	-0.7	4.26E-02	-0.8	1.26E-03	-0.8	2.92E-02	-0.5	1.58E-01	-0.9	2.92E-02	-0.8	2.15E-02	0.0	1.0E+00	0.1	1.0E+00	-0.2	7.1E-01	0.0	1.0E+00	-0.1	9.3E-01	0.0	1.0E+00	-0.1	9.3E-01	-0.1	9.1E-01
39595	BCKDHB	A_24_P914513	NM_183050	-0.8	2.11E-03	-0.8	2.11E-03	-1.0	6.37E-03	-0.8	7.07E-04	-0.4	1.07E-01	-0.2	5.76E-01	-0.6	1.07E-01	-0.4	9.21E-02	0.0	1.0E+00	0.0	1.0E+00	-0.2	7.1E-01	0.1	1.0E+00	-0.2	2.2E-01	-0.2	1.0E+00	-0.2	2.2E-01	-0.4	1.8E-01
20777	ENST00000237536	A_32_P32179	ENST00000237536	-0.8	1.28E-02	-0.7	1.28E-02	-0.9	2.47E-02	-0.8	2.03E-03	-0.4	3.52E-01	-0.4	3.96E-01	-0.4	3.52E-01	-0.7	5.14E-02	-0.1	1.0E+00	-0.1	1.0E+00	-0.2	7.1E-01	-0.1	1.0E+00	0.2	7.9E-01	-0.2	1.0E+00	0.2	5.9E-01	0.2	7.9E-01
6520	NHEDC2	A_23_P341567	NM_178833	-0.8	6.06E-03	-0.8	6.06E-03	-0.8	1.87E-02	-1.0	4.09E-04	-0.3	4.71E-01	-0.3	4.71E-01	-0.2	6.06E-01	-0.6	6.39E-02	-0.1	1.0E+00	-0.2	1.0E+00	-0.1	7.5E-01	0.0	1.0E+00	0.1	8.5E-01	0.1	1.0E+00	0.1	8.5E-01	0.2	8.1E-01
4474	CR600369	A_24_P630039	CR600369	-0.8	8.42E-04	-0.8	8.42E-04	-1.4	1.46E-03	-0.8	4.34E-04	-0.6	1.17E-01	-0.3	3.94E-01	-1.0	9.95E-02	-0.6	1.17E-01	0.0	1.0E+00	0.0	1.0E+00	0.0	9.9E-01	0.0	1.0E+00	-0.1	8.9E-01	-0.2	1.0E+00	0.1	8.0E-01	-0.1	8.9E-01
25332	KIAA1549	A_32_P226186	NM_020910	-0.8	1.11E-03	-0.7	4.91E-04	-0.8	1.78E-03	-0.8	1.11E-03	-0.5	1.72E-01	-0.2	4.37E-01	-1.0	3.37E-02	-0.5	1.72E-01	0.0	1.0E+00	0.0	1.0E+00	-0.1	9.2E-01	0.2	1.0E+00	-0.1	9.1E-01	0.0	1.0E+00	-0.1	9.1E-01	-0.2	8.0E-01
3379	CCT5	A_24_P328872	NM_012073	-0.8	5.67E-03	-0.8	5.67E-03	-1.5	4.91E-03	-0.7	6.15E-03	-0.5	2.16E-01	0.0	9.63E-01	-0.8	2.16E-01	-0.5	1.98E-01	-0.2	1.0E+00	-0.2	1.0E+00	-0.3	7.1E-01	-0.1	1.0E+00	-0.1	9.8E-01	-0.2	1.0E+00	-0.1	9.1E-01	0.0	9.8E-01
27009	METT5D1	A_32_P19887	NM_152636	-0.8	2.85E-03	-0.8	2.26E-02	-1.4	2.85E-03	-0.8	9.17E-04	-0.5	2.91E-01	-0.5	2.42E-01	0.0	9.84E-01	-0.5	2.91E-01	0.0	1.0E+00	0.0	1.0E+00	0.0	9.5E-01	-0.1	1.0E+00	0.0	9.5E-01	0.1	1.0E+00	0.0	9.5E-01	-0.1	9.0E-01
16869	CYP20A1	A_32_P110433	NM_177538	-0.8	1.03E-02	-0.7	3.49E-02	-0.8	1.03E-02	-0.8	3.02E-04	-0.8	1.72E-01	-0.6	3.12E-01	-1.0	1.72E-01	-0.8	7.17E-02	0.3	1.0E+00	0.3	1.0E+00	0.3	7.1E-01	-0.1	1.0E+00	0.2	9.8E-01	0.4	1.0E+00	0.0	9.8E-01	0.2	8.2E-01
43537	ZNF449	A_23_P309865	NM_152695	-0.8	3.08E-03	-0.8	1.94E-03	-1.0	5.43E-03	-0.8	3.08E-03	-0.5	3.67E-01	-0.2	7.53E-01	-0.5	3.67E-01	-0.6	7.86E-02	-0.1	1.0E+00	-0.1	1.0E+00	-0.3	7.1E-01	0.0	1.0E+00	-0.2	6.3E-01	-0.1	1.0E+00	-0.2	6.3E-01	-0.3	6.0E-01
18266	ATP5C1	A_23_P63655	NM_005174	-0.8	2.03E-02	-0.8	4.08E-02	-2.4	1.53E-03	-0.8	2.03E-02	-0.2	6.40E-01	-0.2	7.52E-01	-0.7	2.97E-01	-0.2	6.40E-01	0.0	1.0E+00	0.0	1.0E+00	-0.1	9.1E-01	0.1	1.0E+00	0.1	9.7E-01	-0.1	1.0E+00	0.1	9.3E-01	0.1	9.7E-01
16313	TBL1X	A_24_P417162	NM_005647	-0.8	4.84E-03	-0.7	4.84E-03	-0.8	1.89E-02	-1.0	7.87E-04	-0.6	6.37E-02	-0.5	1.95E-01	-0.7	6.37E-02	-0.6	2.81E-02	-0.1	1.0E+00	-0.1	1.0E+00	-0.1	8.8E-01	-0.2	1.0E+00	0.0	1.0E+00	-0.1	1.0E+00	0.0	9.9E-01	0.0	1.0E+00
23883	CDK10	A_24_P65199	NM_052987	-0.8	1.07E-02	-0.8	1.35E-02	-1.1	1.07E-02	-0.7	6.60E-03	-0.3	7.14E-01	-0.3	7.14E-01	-0.6	5.64E-01	-0.2	7.50E-01	0.2	1.0E+00	0.2	1.0E+00	0.1	9.3E-01	0.2	1.0E+00	0.1	9.8E-01	0.1	1.0E+00	0.1	9.4E-01	0.0	9.8E-01
33472	CHPT1	A_23_P105571	NM_020244	-0.8	4.59E-03	-0.7	2.26E-02	-1.6	4.59E-03	-0.8	2.68E-03	-0.3	4.12E-01	0.1	9.12E-01	-0.8	3.76E-01	-0.3	4.12E-01	-0.1	1.0E+00	-0.1	1.0E+00	-0.1	8.8E-01	0.0	1.0E+00	0.0	9.6E-01	0.0	1.0E+00	0.0	9.6E-01	-0.2	8.3E-01
10050	MGC16384	A_24_P301954	AK001057	-0.8	7.05E-03	-0.8	7.05E-03	-0.9	2.88E-02	-0.8	3.81E-03	-0.7	3.65E-01	-0.5	5.27E-01	-0.7	2.41E-01	-0.7	3.65E-01	0.0	1.0E+00	0.1	1.0E+00	-0.4	7.1E-01	0.0	1.0E+00	0.3	8.0E-01	0.3	1.0E+00	0.3	6.4E-01	0.3	8.0E-01
1063	CAB39L	A_24_P56363	NM_030925	-0.8	4.86E-03	-0.8	4.86E-03	-0.8	1.15E-02	-1.1	3.30E-05	-0.5	1.03E-01	-0.3	7.50E-01	-1.2	9.75E-02	-0.5	1.03E-01	-0.1	1.0E+00	-0.2	1.0E+00	-0.1	8.5E-01	-0.1	1.0E+00	-0.5	6.8E-01	-0.5	1.0E+00	-0.4	6.1E-01	-0.5	6.8E-01
3650	SMAD1	A_24_P71938	NM_005900	-0.8	4.32E-03	-0.8	4.32E-03	-0.9	1.77E-02	-0.7	1.70E-03	-0.5	2.22E-01	-0.5	2.22E-01	-0.4	3.96E-01	-0.7	3.13E-02	-0.1	1.0E+00	-0.1	1.0E+00	0.0	9.5E-01	-0.1	1.0E+00	0.1	9.5E-01	0.2	1.0E+00	0.0	9.5E-01	0.1	8.8E-01
35710	AHI1	A_24_P38143	NM_017651	-0.8	1.13E-03	-0.8	1.13E-03	-0.6	2.65E-02	-0.8	3.51E-04	0.0	9.09E-01	0.1	9.09E-01	0.0	9.48E-01	-0.4	3.54E-01	-0.1	1.0E+00	-0.1	1.0E+00	-0.2	7.3E-01	0.1	1.0E+00	-0.2	6.3E-01	-0.2	1.0E+00	-0.2	3.4E-01	-0.2	6.3E-01
29893	C14orf100	A_23_P205584	NM_016475	-0.8	8.71E-03	-0.7	8.71E-03	-1.0	1.99E-02	-0.8	4.72E-03	-0.2	6.93E-01	0.2	7.83E-01	-0.6	5.05E-01	-0.2	6.93E-01	0.0	1.0E+00	0.0	1.0E+00	-0.1	9.4E-01	0.0	1.0E+00	-0.2	5.4E-01	0.0	1.0E+00	-0.2	5.4E-01	-0.4	3.1E-01
25198	BX538256	A_32_P10123	BX538256	-0.8	5.27E-03	-0.7	1.85E-02	-1.9	3.11E-03	-0.8	5.27E-03	-0.5	2.18E-01	0.0	9.75E-01	-1.4	6.81E-03	-0.5	2.18E-01	0.0	1.0E+00	0.0	1.0E+00	-0.4	7.1E-01	0.1	1.0E+00	0.0	9.9E-01	0.2	1.0E+00	0.0	9.9E-01	0.0	9.8E-01
26054	PCCA	A_23_P48358	NM_000282	-0.8	4.92E-03	-0.8	1.40E-02	-1.6	4.92E-03	-0.8	3.78E-03	-0.6	1.40E-01	-0.6	1.40E-01	-0.6	1.84E-01	-0.6	1.01E-01	-0.2	1.0E+00	-0.2	1.0E+00	-0.2	7.1E-01	-0.1	1.0E+00	0.0	9.5E-01	0.1	1.0E+00	0.0	8.9E-01	0.0	9.5E-01
19026	A_24_P195400	A_24_P195400	A_24_P195400	-0.8	3.54E-03	-0.7	3.95E-02	-1.3	3.54E-03	-0.8	9.75E-04	-0.3	6.71E-01	-0.1	8.80E-01	-1.0	3.03E-01	-0.3	6.71E-01	-0.1	1.0E+00	0.0	1.0E+00	-0.1	8.8E-01	-1.0	1.0E+00	0.1	9.5E-01	-0.1	1.0E+00	0.2	8.5E-01	0.1	9.5E-01
42617	NIPSNAP3A	A_23_P20606	NM_015469	-0.8	9.07E-03	-0.7	9.07E-03	-1.1	1.23E-02	-0.8	3.19E-03	-0.7	8.70E-02	-0.1	8.57E-01	-0.7	8.70E-02	-0.7	2.53E-02	0.0	1.0E+00	-0.1	1.0E+00	0.0	9.7E-01	0.0	1.0E+00	-0.1	8.2E-01	0.0	1.0E+00	-0.1	8.2E-01	-0.2	7.3E-01
43248	TOM1L1	A_24_P196658	NM_005486	-0.8	1.17E-02	-0.8	2.71E-02	-1.2	1.15E-02	-0.7	1.17E-02	-0.4	6.16E-01	-0.1	8.94E-01	-0.5	4.52E-01	-0.4	6.16E-01	-0.1	1.0E+00	-0.1	1.0E+00	-0.1	8.1E-01	0.2	1.0E+00	-0.2	8.7E-01	-0.2	1.0E+00	-0.1	8.7E-01	-0.3	6.9E-01
28966	ZNF343	A_23_P357248	NM_024325	-0.8	6.89E-04	-0.8	6.89E-04	-0.6	2.38E-02	-0.8	2.04E-04	-0.2	7.30E-01	-0.2	7.30E-01	-0.1	8.91E-01	-0.3	3.68E-01	0.0	1.0E+00	0.0	1.0E+00	-0.2	7.1E-01	0.0	1.0E+00	-0.2	5.0E-01	-0.1	1.0E+00	-0.3	1.3E-01	-0.2	5.0E-01
4114	PRPF38B	A_23_P201567	NM_018061	-0.8	2.38E-03	-0.7	7.56E-03	-1.4	2.38E-03	-0.8	1.23E-03	-0.5	1.80E-01	-0.3	4.28E-01	-0.9	1.80E-01	-0.5	9.21E-02	0.0	1.0E+00	0.0	1.0E+00	-0.1	8.0E-01	0.1	1.0E+00	-0.1	7.7E-01	0.0	1.0E+00	-0.1	7.7E-01	-0.3	5.6E-01
28843	EBPL	A_24_P84279	NM_032565	-0.8	7.17E-03	-0.7	2.85E-02	-1.4	7.17E-03	-0.8	3.32E-03	-0.4	1.09E-01	-0.2	7.09E-01	-0.8	1.09E-01	-0.4	5.10E-02	0.0	1.0E+00	0.0	1.0E+00	-0.1	8.2E-01	0.0	1.0E+00	0.0	9.7E-01	0.1	1.0E+00	0.0	9.7E-01	0.0	9.7E-01
25421	TRDMT1	A_24_P252705	NM_004412	-0.8	6.29E-03	-0.8	5.08E-03	-1.0	2.46E-02	-0.8	6.29E-03	-0.4	4.91E-01	-0.2	7.62E-01	-0.4	4.91E-01	-0.5	2.07E-01	-0.1	1.0E+00	-0.1	1.0E+00	-0.2	7.4E-01	0.1	1.0E+00	-0.1	6.4E-01	0.0	1.0E+00	-0.1	6.4E-01	-0.2	4.2E-01
38485	ENTPD4	A_23_P216017	NM_004901	-0.8	1.38E-02	-0.8	1.38E-02	-0.8	3.38E-02	-0.8	1.50E-03	-0.4	3.91E-01	-0.4	4.18E-01	-0.4	3.91E-01	-0.6	1.51E-01	0.0	1.0E+00	0.0	1.0E+00	-0.2	7.5E-01	0.0	1.0E+00	-0.2	8.0E-01	-0.2	1.0E+00	-0.2	6.2E-01	-0.2	8.0E-01
12696	SAA4	A_23_P87238	NM_006512	-0.8	1.98E-03	-1.4	2.63E-04	-0.8	2.85E-02	-0.7	1.98E-03	-0.7	6.50E-02	-0.5	2.81E-01	-0.7	6.50E-02	-0.7	5.31E-02	0.0	1.0E+00	0.0	1.0E+00	-0.2	7.1E-01	0.1	1.0E+00	0.0	9.8E-01	0.0	1.0E+00	0.1	8.8E-01	0.0	9.8E-01
43899	C1orf156	A_23_P85726	NM_033418	-0.8	3.84E-03	-0.6	8.17E-03	-1.4	3.43E-03	-0.8	3.84E-03	-0.5	2.98E-01	-0.3	3.74E-01	-1.0	2.98E-01	-0.5	3.33E-02	0.0	1.0E+00	-0.1	1.0E+00	0.0	9.4E-01	0.1	1.0E+00	-0.1	7.8E-01	-0.1	1.0E+00	-0.1	7.7E-01	-0.2	7.8E-01
8976	ZNF649	A_24_P233256	NM_023074	-0.8	5.17E-03	-0.8	5.17E-03	-0.8	2.54E-02	-0.8	1.75E-03	-0.3	6.02E-01	0.0	9.79E-01	-0.3	6.02E-01	-0.4	3.81E-01	0.0	1.0E+00	0.0	1.0E+00	-0.2	8.4E-01	0.1	1.0E+00	-0.4	4.0E-01	-0.2	1.0E+00	-0.4	1.8E-01	-0.4	4.0E-01
8674	MKLN1	A_24_P170327	NM_013255	-0.8	2.14E-03	-0.8	3.38E-03	-1.0	2.14E-03	-0.6	8.38E-04	-0.6	1.51E-01	-0.3	3.41E-01	-1.2	1.51E-01	-0.6	9.55E-03	0.1	1.0E+00	0.1	1.0E+00	-0.1	9.0E-01	0.1	1.0E+00	-0.1	9.0E-01	-0.1	1.0E+00	-0.1	8.7E-01	-0.2	9.0E-01
26029	ZNF573	A_23_P339079	NM_152360	-0.8	1.59E-02	-0.8	1.44E-02	-1.1	2.25E-02	-0.6	1.59E-02	-0.9	2.36E-02	-0.5	1.84E-01	-1.0	2.36E-02	-0.9	3.59E-03	-0.1	1.0E+00	-0.1	1.0E+00	-0.3	7.1E-01	0.0	1.0E+00	0.1	8.7E-01	-0.1	1.0E+00	0.2	3.7E-01	0.1	8.7E-01
32025	AK095167	A_24_P931579	AK095167	-0.8	1.02E-02	-0.8	1.78E-02	-1.1	1.02E-02	-0.7	2.82E-03	-0.4	2.47E-01	-0.2	6.81E-01	-0.7	1.07E-01	-0.4	2.47E-01	-0.2	1.0E+00	-0.2	1.0E+00	-0.6	5.9E-01	0.1	1.0E+00	-0.3	3.0E-01	-0.3	1.0E+00	-0.3	1.7E-01	-0.4	3.0E-01
37346	DCP1A	A_23_P166826	NM_018403	-0.8	3.13E-03	-0.9	3.13E-03	-0.8	1.86E-02	-0.8	7.56E-04	-0.8	1.16E-02	-0.9	1.63E-02	-0.8	1.16E-02	-0.7	9.85E-03	0.0	1.0E+00	-0.1	1.0E+00	0.0	1.0E+00	0.0	1.0E+00	-0.1	9.7E-01	-0.1	1.0E+00	-0.2	5.9E-01	0.0	9.7E-01
45071	BCKDHB	A_23_P93464	NM_000056	-0.8	8.44E-03	-0.8	4.39E-02	-1.5	8.44E-03	-0.8	6.54E-03	-0.5	2.04E-01	-0.3	6.79E-01	-0.7	2.04E-01	-0.5	6.00E-02	-0.1	1.0E+00	0.0	1.0E+00	-0.1	8.9E-01	-0.1	1.0E+00	0.0	1.0E+00	0.1	1.0E+00	0.0	9.7E-01	0.0	1.0E+00
5979	TMEM126B	A_23_P2129	NM_018480	-0.8	2.32E-03	-0.8	7.98E-03	-1.6	2.25E-03	-0.7	2.32E-03	-0.6	2.83E-01	-0.4	3.04E-01	-1.2	2.83E-01	-0.6	3.38E-02	0.1	1.0E+00	0.0	1.0E+00	0.1	8.5E-01	0.2	1.0E+00	-0.2	6.2E-01	-0.1	1.0E+00	-0.2	5.3E-01	-0.3	6.2E-01
31587	THC2645336	A_32_P190316	THC2645336	-0.8	9.32E-03	-0.8	9.32E-03	-0.8	3.22E-02	-0.8	1.59E-03	-0.3	5.76E-01	-0.2	8.15E-01	-0.4	5.36E-01	-0.3	5.76E-01	-0.1	1.0E+00	-0.1	1.0E+00	-0.3	7.3E-01	0.2	1.0E+00	-0.1	9.3E-01	-0.1	1.0E+00	-0.2	5.9E-01	-0.1	9.3E-01
36002	NUDT4	A_32_P117723	NM_199040	-0.8	3.68E-03	-0.8	1.58E-02	-1.9	1.70E-03	-0.8	3.68E-03	-0.4	3.56E-01	-0.4	3.56E-01	-0.5	2.04E-01	-0.3	3.86E-01	0.0	1.0E+00	-0.2	1.0E+00	0.0	9.6E-01	0.0	1.0E+00	-0.1	9.3E-01	0.0	1.0E+00	-0.1	9.3E-01	-0.2	8.4E-01
8980	NIPSNAP1	A_23_P120860	NM_003634	-0.8	2.17E-03	-0.8	2.17E-03	-0.7	1.51E-02	-1.2	5.19E-05	-0.1	8.98E-01	0.0	9.88E-01	-0.1	8.98E-01	-0.7	1.52E-02	-0.1	1.0E+00	0.0	1.0E+00	-0.1	7.1E-01	-0.2	1.0E+00	0.0	1.0E+00	-0.2	1.0E+00	0.0	1.0E+00	0.2	6.5E-01
15626	ACAD10	A_23_P25348	NM_025247	-0.8	5.35E-03	-0.7	1.75E-02	-1.0	5.35E-03	-0.8	3.56E-04	-0.4	2.28E-01	-0.3	5.01E-01	-0.4	2.28E-01	-0.5	1.22E-01	0.0	1.0E+00	0.1	1.0E+00	0.0	9.5E-01	0.0	1.0E+00	0.0	1.0E+00	-0.1	1.0E+00	0.1	8.2E-01	0.0	1.0E+00
38507	BI913527	A_32_P35595	BI913527	-0.8	4.99E-03	-0.8	4.99E-03	-0.8	2.99E-02	-0.8	1.04E-03	-0.5	1.02E-01	-0.5	1.02E-01	-0.5	6.78E-02	-0.3	3.15E-01	0.0	1.0E+00	0.0	1.0E+00	-0.2	7.9E-01	0.2	1.0E+00	-0.1	8.7E-01	0.0	1.0E+00	-0.1	5.5E-01	-0.1	8.7E-01
4568	WDR36	A_24_P106357	NM_139281	-0.8	2.05E-03	-0.7	1.12E-02	-1.5	2.03E-03	-0.8	2.05E-03	-0.5	2.73E-01	-0.2	6.81E-01	-1.1	2.73E-01	-0.5	1.91E-01	-0.1	1.0E+00	-0.1	1.0E+00	-0.1	9.0E-01	0.0	1.0E+00	0.1	9.8E-01	0.1	1.0E+00	0.3	5.8E-01	0.0	9.8E-01
9935	ZRANB3	A_23_P412059	BC064616	-0.8	8.35E-04	-0.7	8.35E-04	-0.8	4.13E-03	-1.0	2.95E-05	-0.5	1.64E-01	-0.1	6.80E-01	-0.5	1.64E-01	-0.6	9.03E-03	-0.1	1.0E+00	-0.1	1.0E+00	-0.1	7.1E-01	-0.1	1.0E+00	-0.1	7.9E-01	-0.1	1.0E+00	-0.1	6.3E-01	-0.1	7.9E-01
32933	HNRNPA3	A_24_P706312	NM_194247	-0.8	2.14E-02	-0.8	2.14E-02	-0.8	2.62E-02	-0.8	1.82E-03	-0.8	1.71E-02	-0.7	5.34E-02	-1.1	1.71E-02	-0.8	1.57E-02	0.0	1.0E+00	0.1	1.0E+00	-0.1	8.5E-01	0.0	1.0E+00	0.1	9.6E-01	0.1	1.0E+00	0.2	5.9E-01	0.1	9.6E-01
2763	CCBE1	A_23_P55544	NM_133459	-0.8	6.94E-03	-0.7	6.94E-03	-1.0	1.30E-02	-0.8	9.82E-04	-0.4	2.30E-01	-0.3	5.40E-01	-0.5	2.30E-01	-0.4	1.70E-01	-0.1	1.0E+00	-0.1	1.0E+00	-0.2	7.1E-01	-0.1	1.0E+00	-0.2	7.5E-01	-0.1	1.0E+00	-0.2	5.1E-01	-0.2	7.5E-01
37889	AK024921	A_24_P620456	AK024921	-0.8	2.07E-02	-0.8	7.56E-03	-0.9	3.31E-02	-0.6	2.07E-02	-0.4	4.48E-01	-0.3	7.22E-01	-0.7	2.99E-01	-0.4	4.48E-01	0.1	1.0E+00	0.1	1.0E+00	-0.3	7.3E-01	0.1	1.0E+00	-0.2	7.1E-01	-0.1	1.0E+00	-0.2	6.5E-01	-0.3	7.1E-01
10278	SYT15	A_24_P100535	NM_181519	-0.8	1.49E-03	-0.8	1.49E-03	-0.6	2.04E-02	-0.8	1.96E-04	-0.6	1.73E-01	-0.5	3.95E-01	-0.6	1.73E-01	-0.7	7.06E-02	-0.1	1.0E+00	-0.1	1.0E+00	0.1	8.2E-01	-0.1	1.0E+00	0.0	9.8E-01	0.0	1.0E+00	0.0	9.2E-01	0.0	9.8E-01
41566	DUS4L	A_23_P215778	NM_181581	-0.8	2.40E-02	-0.6	4.39E-02	-1.0	2.40E-02	-0.8	7.81E-03	-0.7	1.21E-01	-0.5	1.21E-01	-0.8	2.21E-01	-0.7	4.12E-02	-0.3	1.0E+00	-0.3	1.0E+00	-0.2	7.3E-01	-0.3	1.0E+00	0.0	9.1E-01	0.0	1.0E+00	0.1	9.1E-01	-0.1	9.0E-01
20636	VPS13B	A_24_P280628	NM_152564	-0.8	9.70E-03	-0.8	1.26E-02	-1.2	9.70E-03	-0.7	2.51E-03	-0.6	1.74E-01	-0.4	5.16E-01	-1.0	1.74E-01	-0.6	9.16E-02	0.0	1.0E+00	0.1	1.0E+00	-0.3	7.1E-01	0.0	1.0E+00	0.2	6.6E-01	0.0	1.0E+00	0.2	6.6E-01	0.3	4.2E-01
10448	DEPDC1	A_23_P200310	NM_017779	-0.8	2.10E-02	-0.8	7.83E-03	-0.9	3.84E-02	-0.7	2.10E-02	-0.9	1.89E-01	-0.9	1.89E-01	-0.9	2.80E-01	-1.0	9.49E-02	-0.5	1.0E+00	0.0	1.0E+00	-0.5	7.1E-01	-0.6	1.0E+00	0.0	9.9E-01	0.1	1.0E+00	-0.2	7.9E-01	0.0	9.9E-01
2484	FAM119A	A_23_P209337	NM_145280	-0.8	7.61E-03	-0.8	7.61E-03	-0.6	3.35E-02	-1.1	8.11E-05	-0.3	4.27E-01	-0.3	4.27E-01	-0.3	5.77E-01	-0.9	1.11E-02	0.0	1.0E+00	0.0	1.0E+00	0.0	9.6E-01	-0.4	1.0E+00	-0.2	5.0E-01	-0.1	1.0E+00	-0.2	5.0E-01	-0.5	2.0E-01
25452	THC2510958	A_24_P936911	THC2510958	-0.8	3.28E-03	-0.8	3.28E-03	-0.9	4.30E-03	-0.7	1.68E-04	-0.6	2.19E-01	-0.5	3.06E-01	-0.7	2.19E-01	-0.6	6.83E-02	-0.1	1.0E+00	-0.1	1.0E+00	-0.3	7.1E-01	-0.1	1.0E+00	0.1	8.7E-01	0.1	1.0E+00	0.1	8.7E-01	0.2	8.3E-01
36750	C15orf29	A_23_P77286	NM_024713	-0.8	5.89E-03	-0.8	5.89E-03	-1.0	1.61E-02	-0.7	5.15E-03	-0.1	8.49E-01	0.0	9.81E-01	-0.4	4.28E-01	-0.1	8.49E-01	0.0	1.0E+00	0.0	1.0E+00	-0.1	8.5E-01	0.2	1.0E+00	-0.3	5.3E-01	-0.1	1.0E+00	-0.4	1.7E-01	-0.3	5.3E-01
19432	PCBD2	A_24_P180423	AK098495	-0.8	2.31E-02	-0.8	2.03E-02	-1.3	2.31E-02	-0.7	2.44E-02	-0.4	4.80E-01	-0.1	9.51E-01	-0.8	3.54E-01	-0.4	4.80E-01	0.0	1.0E+00	0.0	1.0E+00	0.0	9.6E-01	0.2	1.0E+00	-0.1	9.2E-01	-0.1	1.0E+00	-0.1	9.2E-01	-0.3	6.4E-01
34614	SLC35E3	A_23_P147729	NM_018656	-0.8	1.23E-03	-0.7	9.80E-03	-1.3	1.23E-03	-0.8	2.94E-04	-0.3	5.88E-01	-0.4	5.82E-01	-0.1	9.29E-01	-0.3	5.88E-01	0.0	1.0E+00	0.1	1.0E+00	0.0	1.0E+00	0.0	1.0E+00	-0.1	8.6E-01	0.0	1.0E+00	-0.3	4.2E-01	-0.1	8.6E-01
40481	DDX54	A_32_P430359	NM_024072	-0.8	1.28E-03	-0.8	1.28E-03	-0.7	1.03E-02	-0.9	5.56E-04	-0.4	7.89E-02	-0.4	8.86E-02	-0.4	7.89E-02	-0.7	1.63E-02	0.0	1.0E+00	-0.1	1.0E+00	0.0	9.3E-01	0.0	1.0E+00	0.0	9.7E-01	0.0	1.0E+00	0.0	9.5E-01	0.1	9.7E-01
9902	ATP5G2	A_23_P87616	NM_001002031	-0.8	1.67E-03	-0.8	1.67E-03	-0.7	2.69E-02	-1.0	1.49E-04	-0.4	1.19E-01	-0.4	1.19E-01	-0.2	3.19E-01	-0.5	1.17E-01	0.0	1.0E+00	0.1	1.0E+00	-0.1	8.4E-01	0.0	1.0E+00	0.0	9.8E-01	0.1	1.0E+00	0.0	9.5E-01	0.0	9.8E-01
43374	HDDC2	A_23_P122796	NM_016063	-0.8	1.30E-02	-0.8	1.30E-02	-0.7	2.39E-02	-0.8	6.11E-04	-0.3	3.28E-01	-0.2	6.42E-01	-0.3	3.28E-01	-0.3	2.00E-01	0.0	1.0E+00	0.0	1.0E+00	-0.1	8.0E-01	0.0	1.0E+00	-0.1	9.0E-01	0.0	1.0E+00	-0.2	5.8E-01	-0.1	9.0E-01

Feature	Gene symbol	Probe	Transcript	72 hour								48 hours								24 hours								10 hours							
				siCASP8AP2								siCASP8AP2								siCASP8AP2								siCASP8AP2							
				Median M	Median q	1 M	1 q	3 M	3 q	6 M	6 q	Median M	Median q	1 M	1 q	3 M	3 q	6 M	6 q	Median M	Median q	1 M	1 q	3 M	3 q	6 M	6 q	Median M	Median q	1 M	1 q	3 M	3 q	6 M	6 q
40920	CETN2	A_23_P73493	NM_004344	-0.8	4.72E-03	-0.8	4.72E-03	-0.7	4.74E-02	-0.8	2.35E-03	-0.7	3.01E-01	-0.3	5.12E-01	-0.7	3.01E-01	-0.8	1.64E-02	0.1	1.0E+00	-0.1	1.0E+00	0.1	8.1E-01	0.1	1.0E+00	0.0	9.7E-01	-0.2	1.0E+00	0.1	9.1E-01	0.0	9.7E-01
44078	PDE4D	A_23_P124456	NM_006203	-0.8	8.97E-03	-0.6	8.97E-03	-0.8	2.84E-02	-0.8	1.70E-03	-0.5	6.08E-02	-0.4	1.44E-01	-0.5	6.08E-02	-0.8	2.36E-03	-0.2	1.0E+00	-0.2	1.0E+00	-0.5	7.1E-01	-0.1	1.0E+00	-0.2	4.0E-01	-0.1	1.0E+00	-0.3	1.6E-01	-0.2	4.0E-01
11307	CEPT1	A_24_P233944	NM_001007794	-0.8	1.93E-03	-0.6	3.57E-03	-1.1	1.93E-03	-0.8	2.20E-04	0.0	7.13E-01	0.0	9.89E-01	-0.5	3.69E-01	0.4	7.13E-01	0.0	1.0E+00	0.1	1.0E+00	-0.3	7.1E-01	0.0	1.0E+00	-0.1	9.5E-01	-0.1	1.0E+00	0.0	9.5E-01	-0.2	8.4E-01
39436	PPIH	A_23_P200940	NM_006347	-0.8	1.58E-02	-0.7	3.29E-02	-1.2	1.58E-02	-0.8	6.17E-03	-0.5	4.09E-01	0.1	8.82E-01	-0.5	4.09E-01	-0.5	3.00E-02	0.0	1.0E+00	0.0	1.0E+00	-0.1	8.1E-01	0.2	1.0E+00	-0.1	8.7E-01	-0.1	1.0E+00	-0.1	8.7E-01	-0.4	6.5E-01
28898	XRCC6	A_23_P120947	NM_001469	-0.8	2.80E-02	-0.8	2.80E-02	-0.9	3.24E-02	-0.7	1.14E-02	-0.4	2.92E-01	-0.4	2.30E-01	-0.7	3.65E-01	-0.4	2.92E-01	0.0	1.0E+00	0.0	1.0E+00	0.0	9.4E-01	0.1	1.0E+00	0.0	9.7E-01	-0.2	1.0E+00	0.0	9.7E-01	0.2	9.0E-01
25502	THC2657938	A_24_P141804	THC2657938	-0.8	8.08E-04	-0.8	5.52E-03	-1.6	8.08E-04	-0.7	6.65E-04	-0.3	6.04E-01	0.0	9.92E-01	-0.5	3.10E-01	-0.3	6.04E-01	0.1	1.0E+00	0.0	1.0E+00	0.1	8.2E-01	0.4	1.0E+00	-0.1	9.0E-01	-0.1	1.0E+00	0.0	9.0E-01	-0.1	8.4E-01
10064	SMAD1	A_23_P212870	NM_005900	-0.8	1.35E-03	-0.8	1.35E-03	-0.8	6.73E-03	-0.8	7.45E-04	-0.4	1.22E-01	-0.4	2.67E-01	-0.4	1.22E-01	-0.6	2.53E-02	0.0	1.0E+00	-0.1	1.0E+00	0.0	9.4E-01	0.0	1.0E+00	0.0	9.3E-01	-0.1	1.0E+00	0.0	9.3E-01	0.1	9.3E-01
8865	POLI	A_23_P306890	NM_007195	-0.8	5.14E-03	-0.8	5.14E-03	-1.3	7.05E-03	-0.8	4.55E-03	-0.4	2.39E-01	-0.4	2.43E-01	-0.4	2.39E-01	-0.6	3.52E-02	-0.1	1.0E+00	-0.2	1.0E+00	-0.1	8.4E-01	0.2	1.0E+00	-0.2	9.8E-01	-0.2	1.0E+00	0.0	9.8E-01	-0.3	6.7E-01
38283	MAP2K5	A_24_P356130	NM_002757	-0.8	1.89E-02	-0.6	3.94E-02	-0.8	1.89E-02	-0.9	6.56E-04	-0.1	8.16E-01	0.0	9.81E-01	-0.3	5.93E-01	-0.1	8.16E-01	-0.1	1.0E+00	-0.1	1.0E+00	-0.2	7.1E-01	0.0	1.0E+00	-0.2	9.6E-01	-0.2	1.0E+00	-0.2	5.2E-01	-0.1	9.6E-01
9498	SFRS2B	A_32_P17635	NM_032102	-0.8	2.43E-03	-1.2	1.25E-03	-0.8	4.10E-02	-0.8	2.43E-03	-0.6	1.21E-01	-0.6	1.21E-01	-0.3	5.97E-01	-0.8	8.55E-02	0.2	1.0E+00	-0.2	1.0E+00	0.2	8.4E-01	0.2	1.0E+00	-0.2	9.2E-01	-0.2	1.0E+00	-0.2	7.7E-01	-0.1	9.2E-01
13425	ECT2	A_23_P44684	NM_018098	-0.8	1.10E-02	-0.8	9.13E-03	-1.1	1.10E-02	-0.6	1.35E-02	-0.7	9.15E-02	-0.7	9.76E-02	-1.4	9.15E-02	-0.7	1.03E-02	-0.3	1.0E+00	0.0	1.0E+00	-0.3	7.1E-01	-0.3	1.0E+00	0.0	9.2E-01	0.1	1.0E+00	0.0	9.2E-01	-0.1	9.0E-01
32546	LETM1P2	A_24_P911191	XR_038110	-0.8	1.98E-03	-0.8	3.30E-02	-1.8	9.81E-04	-0.6	1.98E-03	0.0	9.63E-01	0.0	9.71E-01	-0.5	6.06E-01	0.0	9.63E-01	0.1	1.0E+00	0.1	1.0E+00	0.1	8.9E-01	0.4	1.0E+00	0.1	9.6E-01	-0.1	1.0E+00	0.2	8.3E-01	0.1	9.6E-01
16151	ZNF22	A_23_P384512	NM_006963	-0.8	2.01E-02	-0.6	2.62E-02	-1.1	2.01E-02	-0.8	5.24E-03	-0.5	3.77E-01	-0.6	3.77E-01	-0.5	4.11E-01	-0.5	3.40E-01	-0.2	1.0E+00	0.0	1.0E+00	-0.2	8.0E-01	-0.2	1.0E+00	-0.2	7.9E-01	0.1	1.0E+00	-0.2	7.9E-01	-0.4	7.3E-01
15035	TMEM168	A_24_P414952	NM_022484	-0.8	4.48E-03	-0.7	8.89E-03	-1.4	4.48E-03	-0.8	2.64E-03	-0.5	2.03E-01	-0.2	7.58E-01	-1.0	2.03E-01	-0.5	2.94E-02	-0.1	1.0E+00	-0.1	1.0E+00	-0.1	7.8E-01	-0.1	1.0E+00	-0.2	9.9E-01	-0.2	1.0E+00	0.0	9.9E-01	-0.2	3.9E-01
1721	SMYD3	A_23_P51410	NM_022743	-0.8	2.22E-03	-0.6	9.72E-03	-1.4	2.22E-03	-0.8	6.72E-04	-0.3	5.20E-01	-0.2	7.97E-01	-0.5	3.53E-01	-0.3	5.20E-01	0.0	1.0E+00	0.0	1.0E+00	-0.1	8.0E-01	0.0	1.0E+00	0.0	9.8E-01	-0.1	1.0E+00	0.1	8.1E-01	0.0	9.8E-01
28780	SDCCAG1	A_24_P6808	NM_004713	-0.8	4.73E-03	-0.8	3.78E-03	-1.0	1.21E-02	-0.6	4.73E-03	-0.6	2.54E-01	-0.5	3.68E-01	-0.6	2.54E-01	-0.6	4.44E-02	0.1	1.0E+00	0.0	1.0E+00	0.1	8.8E-01	0.1	1.0E+00	0.1	8.8E-01	0.0	1.0E+00	0.1	8.0E-01	0.1	8.8E-01
10860	HGSNAT	A_23_P112061	NM_152419	-0.8	1.47E-03	-0.6	1.03E-02	-1.2	1.47E-03	-0.8	1.96E-04	-0.1	7.61E-01	0.0	9.75E-01	-0.1	7.61E-01	-0.3	2.24E-01	0.2	1.0E+00	0.1	1.0E+00	0.2	7.8E-01	0.3	1.0E+00	0.0	9.6E-01	0.0	1.0E+00	-0.1	8.8E-01	0.1	9.6E-01
7410	VSNL1	A_23_P209978	NM_003385	-0.8	8.01E-03	-0.8	1.36E-02	-1.6	7.19E-03	-0.8	8.01E-03	-0.4	5.95E-01	-0.2	6.74E-01	-0.4	5.95E-01	-0.6	5.62E-02	0.0	1.0E+00	0.0	1.0E+00	0.0	9.7E-01	-0.1	1.0E+00	0.0	9.9E-01	0.0	1.0E+00	0.0	9.9E-01	-0.3	7.9E-01
31186	PSMA3	A_23_P140301	NM_002788	-0.8	7.05E-03	-0.8	7.05E-03	-1.3	7.55E-03	-0.7	3.92E-03	-0.5	2.25E-01	-0.3	5.92E-01	-0.5	2.25E-01	-0.6	2.64E-02	-0.1	1.0E+00	-0.1	1.0E+00	-0.1	8.6E-01	-0.1	1.0E+00	-0.1	9.9E-01	-0.1	1.0E+00	0.0	9.9E-01	-0.1	9.5E-01
18215	SLC35B4	A_24_P367561	NM_032826	-0.8	2.25E-03	-0.7	9.32E-03	-1.4	2.25E-03	-0.8	8.08E-04	-0.4	3.42E-01	-0.2	6.10E-01	-1.0	2.51E-01	-0.4	3.42E-01	0.0	1.0E+00	0.0	1.0E+00	-0.1	8.2E-01	0.1	1.0E+00	0.1	9.5E-01	0.0	1.0E+00	0.1	8.4E-01	0.1	9.5E-01
5452	THC2682280	A_32_P232237	THC2682280	-0.8	9.42E-03	-0.8	4.38E-02	-1.7	3.88E-03	-0.7	9.42E-03	-0.4	2.14E-01	0.2	5.85E-01	-0.9	3.73E-02	-0.4	2.14E-01	0.1	1.0E+00	0.1	1.0E+00	-0.2	7.5E-01	0.3	1.0E+00	-0.3	4.6E-01	-0.1	1.0E+00	-0.5	4.6E-01	-0.3	2.7E-01
28069	RABL3	A_32_P221452	NM_173825	-0.8	4.62E-03	-0.6	7.59E-03	-0.8	4.62E-03	-0.8	8.42E-05	-0.5	4.44E-01	-0.5	4.44E-01	-0.3	5.41E-01	-0.6	1.57E-01	-0.1	1.0E+00	-0.1	1.0E+00	-0.1	7.5E-01	-0.1	1.0E+00	0.0	9.9E-01	-0.1	1.0E+00	0.0	9.4E-01	0.0	9.9E-01
26688	EIF3E	A_32_P229760	NM_001568	-0.8	6.49E-04	-0.8	8.58E-03	-2.0	5.22E-04	-0.8	6.49E-04	-0.4	3.80E-01	-0.2	5.78E-01	-0.8	3.80E-01	-0.4	1.67E-01	0.1	1.0E+00	0.0	1.0E+00	0.2	8.1E-01	0.1	1.0E+00	-0.1	9.6E-01	-0.2	1.0E+00	0.1	9.6E-01	-0.1	9.5E-01
32142	LOC100130794	A_24_P76078	XM_001724177	-0.8	1.75E-02	-0.8	1.75E-02	-0.7	4.75E-02	-0.9	1.53E-03	-0.4	4.47E-01	-0.2	6.12E-01	-0.4	4.47E-01	-0.7	8.03E-02	0.0	1.0E+00	0.0	1.0E+00	-0.2	7.7E-01	0.1	1.0E+00	0.1	9.9E-01	0.2	1.0E+00	0.0	9.9E-01	0.1	9.5E-01
720	SNAPC3	A_23_P71908	NM_001039697	-0.8	1.27E-03	-0.8	9.43E-04	-1.3	1.27E-03	-0.7	3.81E-03	-0.3	4.14E-01	-0.2	7.63E-01	-0.6	2.12E-01	-0.3	4.14E-01	-0.1	1.0E+00	0.0	1.0E+00	-0.3	7.1E-01	-0.1	1.0E+00	-0.2	3.4E-01	0.0	1.0E+00	-0.2	3.4E-01	-0.4	2.7E-01
38590	RAD54B	A_23_P82738	NM_012415	-0.8	1.72E-02	-0.6	2.30E-02	-1.0	1.72E-02	-0.8	6.45E-03	-0.7	1.60E-01	-0.4	5.10E-01	-0.7	1.60E-01	-0.7	7.09E-02	0.0	1.0E+00	-0.1	1.0E+00	0.0	9.4E-01	0.1	1.0E+00	-0.1	8.1E-01	-0.1	1.0E+00	-0.1	8.1E-01	-0.3	6.4E-01
42131	C15orf61	A_23_P26143	AK294012	-0.8	2.58E-02	-0.8	3.46E-02	-1.2	2.14E-02	-0.8	2.58E-02	-0.6	3.37E-02	-0.5	1.85E-01	-0.6	3.37E-02	-0.9	4.22E-03	-0.1	1.0E+00	-0.2	1.0E+00	-0.1	9.1E-01	0.0	1.0E+00	-0.2	6.7E-01	-0.2	1.0E+00	-0.2	6.7E-01	-0.3	6.0E-01
10481	ALDH5A1	A_24_P115007	NM_170740	-0.8	2.76E-02	-0.6	2.76E-02	-1.0	3.91E-02	-0.8	8.72E-03	-0.3	7.03E-01	0.1	8.49E-01	-0.3	7.03E-01	-0.5	1.61E-01	0.0	1.0E+00	0.0	1.0E+00	0.0	9.9E-01	0.1	1.0E+00	0.0	9.8E-01	-0.1	1.0E+00	0.0	9.8E-01	0.0	9.5E-01
32775	NCK1	A_23_P255785	NM_006153	-0.8	1.79E-02	-0.8	1.21E-02	-0.9	3.71E-02	-0.7	1.79E-02	-0.6	5.50E-02	-0.6	2.82E-02	-1.0	1.66E-01	-0.4	5.50E-02	-0.1	1.0E+00	0.0	1.0E+00	-0.1	8.4E-01	-0.1	1.0E+00	0.0	9.7E-01	-0.1	1.0E+00	-0.1	9.4E-01	0.0	9.7E-01
2146	PCGF5	A_24_P880043	NM_032373	-0.8	2.13E-02	-0.8	2.13E-02	-0.9	3.84E-02	-0.6	1.99E-02	-0.7	1.23E-01	-0.4	3.43E-01	-0.7	1.23E-01	-0.8	5.17E-02	0.1	1.0E+00	0.1	1.0E+00	0.1	9.0E-01	0.2	1.0E+00	-0.1	8.4E-01	0.0	1.0E+00	-0.1	8.4E-01	-0.4	4.3E-01
18	EHMT2	A_24_P801451	NM_006709	-0.8	3.43E-03	-0.6	3.83E-02	-1.4	3.43E-03	-0.8	1.84E-03	-0.3	6.32E-01	-0.2	7.81E-01	-0.9	4.45E-01	-0.3	6.32E-01	0.2	1.0E+00	0.2	1.0E+00	0.1	9.5E-01	0.2	1.0E+00	0.0	1.0E+00	0.0	1.0E+00	0.1	9.3E-01	0.0	1.0E+00
4109	PSMD1	A_24_P128205	NM_002807	-0.8	6.05E-03	-0.7	6.05E-03	-0.8	1.50E-02	-0.8	8.79E-04	-0.7	5.79E-02	-0.5	8.88E-02	-0.7	5.79E-02	-0.8	5.84E-03	-0.1	1.0E+00	0.1	1.0E+00	-0.1	8.0E-01	-0.1	1.0E+00	-0.1	8.8E-01	-0.3	1.0E+00	-0.1	7.1E-01	-0.1	8.8E-01
18110	PAK1IP1	A_23_P122674	NM_017906	-0.8	8.41E-03	-0.8	8.41E-03	-0.8	1.87E-02	-1.2	6.34E-05	-1.1	3.46E-02	-1.1	3.46E-02	-1.0	2.12E-01	-1.5	5.73E-03	-0.3	1.0E+00	-0.2	1.0E+00	-0.3	7.7E-01	-0.5	1.0E+00	0.1	1.0E+00	0.1	1.0E+00	0.2	7.4E-01	0.0	1.0E+00
15153	THC2504565	A_24_P801825	THC2504565	-0.8	2.79E-03	-0.7	1.19E-02	-1.0	2.79E-03	-0.8	4.25E-04	-0.5	2.58E-01	-0.1	8.74E-01	-0.6	1.91E-01	-0.5	2.58E-01	-0.1	1.0E+00	-0.1	1.0E+00	-0.4	7.1E-01	0.0	1.0E+00	-0.2	7.0E-01	-0.1	1.0E+00	-0.3	2.7E-01	-0.2	7.0E-01
7215	CCDC76	A_24_P396557	NM_019083	-0.8	2.65E-03	-0.6	2.65E-03	-1.2	2.70E-03	-0.8	7.17E-04	-0.6	1.61E-01	-0.7	1.61E-01	-0.3	6.04E-01	-0.6	1.31E-01	-0.1	1.0E+00	0.1	1.0E+00	-0.2	7.1E-01	-0.1	1.0E+00	0.1	9.8E-01	0.1	1.0E+00	0.1	8.0E-01	0.0	9.8E-01
29750	FBXO17	A_23_P101871	NM_024907	-0.8	1.56E-03	-0.8	1.56E-03	-0.9	2.75E-03	-0.6	6.40E-04	-0.4	5.13E-01	-0.3	6.17E-01	-0.6	5.13E-01	-0.4	3.68E-01	0.0	1.0E+00	-0.1	1.0E+00	0.0	9.5E-01	0.0	1.0E+00	-0.1	9.5E-01	0.0	1.0E+00	-0.1	8.4E-01	-0.1	9.5E-01
21627	TTC37	A_23_P61854	ENST00000358746	-0.8	3.68E-02	-0.7	3.68E-02	-1.2	4.07E-02	-0.8	2.13E-02	-0.8	7.37E-02	-0.3	5.45E-01	-0.9	7.37E-02	-0.8	2.50E-02	0.0	1.0E+00	-0.1	1.0E+00	0.0	9.3E-01	0.0	1.0E+00	0.0	9.1E-01	0.0	1.0E+00	0.3	3.8E-01	-0.1	9.1E-01
45110	LOC345645	A_24_P84873	XR_018613	-0.8	5.36E-03	-0.7	1.70E-02	-1.7	2.53E-03	-0.8	5.36E-03	-0.7	1.52E-01	-0.5	1.52E-01	-1.2	3.16E-01	-0.7	4.36E-02	0.1	1.0E+00	-0.1	1.0E+00	0.1	8.5E-01	0.0	1.0E+00	-0.1	9.6E-01	-0.2	1.0E+00	0.2	8.0E-01	-0.1	9.6E-01
11870	BCL2L1	A_23_P210886	NM_138578	-0.8	7.46E-03	-0.8	2.20E-02	-1.4	1.59E-03	-0.6	7.46E-03	-0.3	4.73E-01	-0.2	6.42E-01	-0.9	2.32E-01	-0.3	4.73E-01	-0.1	1.0E+00	0.1	1.0E+00	-0.1	8.8E-01	-0.1	1.0E+00	0.1	9.2E-01	-0.1	1.0E+00	0.1	9.2E-01	0.3	8.6E-01
4839	ANAPC7	A_23_P44643	NM_016238	-0.8	7.27E-04	-0.7	2.02E-02	-1.6	7.27E-04	-0.8	3.72E-04	-0.5	1.50E-01	-0.3	4.11E-01	-0.8	1.50E-01	-0.5	9.59E-02	-0.1	1.0E+00	-0.1	1.0E+00	-0.1	7.9E-01	-0.1	1.0E+00	0.1	9.0E-01	-0.1	1.0E+00	0.3	6.5E-01	0.1	9.0E-01
35709	AL133090	A_24_P355006	AL133090	-0.8	1.27E-02	-0.7	2.32E-02	-1.1	1.27E-02	-0.8	3.58E-03	-0.4	4.47E-01	-0.2	6.95E-01	-0.4	4.47E-01	-0.6	1.10E-01	0.1	1.0E+00	0.1	1.0E+00	0.1	8.5E-01	0.2	1.0E+00	0.1	9.7E-01	0.1	1.0E+00	0.1	7.3E-01	0.0	9.7E-01
38925	RWDD1	A_23_P167828	NM_016104	-0.8	4.15E-03	-0.8	4.15E-03	-0.8	3.89E-02	-0.7	2.58E-03	-0.6	1.50E-01	-0.6	1.50E-01	-0.5	2.50E-01	-0.7	9.05E-02	0.0	1.0E+00	0.0	1.0E+00	0.0	9.8E-01	0.1	1.0E+00	-0.2	9.4E-01	-0.2	1.0E+00	0.0	9.4E-01	-0.2	6.2E-01
9584	THC2507829	A_32_P72477	THC2507829	-0.8	9.31E-03	-0.8	9.31E-03	-0.9	3.73E-02	-0.7	6.15E-03	-0.5	2.76E-01	-0.4	4.43E-01	-0.5	2.76E-01	-0.5	1.44E-01	0.0	1.0E+00	0.0	1.0E+00	-0.1	7.8E-01	0.1	1.0E+00	-0.1	8.3E-01	-0.1	1.0E+00	-0.1	8.3E-01	-0.2	7.2E-01
34972	PRR4	A_23_P62042	NM_001098538	-0.8	1.32E-03	-0.8	1.32E-03	-0.8	1.02E-02	-0.7	4.81E-04	-0.2	6.04E-01	-0.1	8.65E-01	-0.5	4.22E-01	-0.2	6.04E-01	-0.1	1.0E+00	-0.1	1.0E+00	-0.1	8.5E-01	0.1	1.0E+00	0.0	1.0E+00	0.0	1.0E+00	-0.1	7.8E-01	0.0	1.0E+00
42410	PCNP	A_23_P155332	NM_020357	-0.8	1.56E-03	-0.8	4.47E-03	-1.8	6.44E-04	-0.8	1.56E-03	-0.6	1.71E-01	-0.4	1.71E-01	-1.3	3.24E-01	-0.6	8.93E-02	0.2	1.0E+00	0.1	1.0E+00	0.2	7.3E-01	0.2	1.0E+00	-0.1	8.8E-01	-0.1	1.0E+00	-0.1	8.8E-01	-0.4	5.1E-01
22714	DENND1B	A_23_P320897	AK024832	-0.8	2.19E-02	-0.8	2.19E-02	-1.5	1.18E-02	-0.7	2.40E-02	-0.3	5.41E-01	0.1	8.65E-01	-0.7	3.79E-01	-0.3	5.41E-01	0.0	1.0E+00	0.0	1.0E+00	-0.3	7.4E-01	0.2	1.0E+00	-0.2	6.7E-01	-0.1	1.0E+00	-0.2	3.2E-01	-0.2	6.7E-01
37876	MEST	A_23_P156970	NM_002402	-0.8	2.49E-02	-0.6	2.49E-02	-1.0	3.09E-02	-0.8	6.24E-03	-0.5	3.29E-01	-0.3	4.96E-01	-0.9	3.29E-01	-0.5	2.62E-01	-0.2	1.0E+00	-0.2	1.0E+00	-0.2	8.1E-01	0.1	1.0E+00	-0.4	4.9E-01	-0.4	1.0E+00	-0.2	4.9E-01	-0.4	2.8E-01
28251	PCDHGA8	A_23_P354734	NM_032088	-0.8	8.46E-04	-0.8	1.28E-02	-1.3	8.46E-04	-0.6	4.37E-04	-0.3	6.96E-01	-0.3	6.96E-01	-0.3	5.75E-01	-0.2	7.18E-01	0.0	1.0E+00	0.1	1.0E+00	0.0	1.0E+00	0.0	1.0E+00	0.0	9.9E-01	0.0	1.0E+00	0.0	9.9E-01	-0.1	9.7E-01
38477	CNIH	A_23_P14473	NM_005776	-0.8	3.54E-02	-0.8	2.79E-02	-1.2	3.54E-02	-0.7	3.98E-02	-0.3	5.46E-01	-0.2	6.91E-01	-0.3	5.46E-01	-0.5	9.54E-02	0.0	1.0E+00	0.1	1.0E+00	0.0	9.9E-01	0.0	1.0E+00	-0.1	9.5E-01	-0.1	1.0E+00	0.0	9.5E-01	-0.2	7.5E-01
42740	LOC100132288	A_32_P449517	NM_001033515	-0.8	4.78E-03	-0.7	2.86E-02	-1.2	4.78E-03	-0.8	3.18E-03	-0.7	1.68E-01	-1.2	6.16E-02	-0.7	2.18E-01	-0.6	1.68E-01	0.1	1.0E+00	-0.1	1.0E+00	0.2	7.1E-01	0.1	1.0E+00	0.0	9.8E-01	-0.1	1.0E+00	0.2	7.1E-01	0.0	9.8E-01
30661	SIRT3	A_24_P923684	NM_012239	-0.8	2.00E-02	-1.1	2.85E-03	-0.8	3.76E-02	-0.6	2.00E-02	-0.7	1.97E-01	-0.7	1.97E-01	0.1	8.33E-01	-0.8	1.47E-01	-0.1	1.0E+00	-0.3	1.0E+00	-0.1	9.3E-01	0.0	1.0E+00	0.0	9.8E-01	-0.3	1.0E+00	0.0	9.1E-01	0.0	9.8E-01
4137	MKRN2	A_23_P57807	NM_014160	-0.8	2.25E-03	-0.7	2.25E-03	-1.1	3.79E-03	-0.8	1.87E-03	-0.6	4.55E-02	-0.2	4.87E-01	-0.7	4.55E-02	-0.6	1.51E-02	-0.1	1.0E+00	-0.2	1.0E+00	-0.1	7.5E-01	0.0	1.0E+00	0.1	1.0E+00	0.1	1.0E+00	0.1	9.0E-01	0.0	1.0E+00
3484	A_32_P41594	A_32_P41594	A_32_P41594	-0.8	2.82E-02	-0.8	2.82E-02	-0.8	3.66E-02	-0.9	3.34E-03	-0.7	1.94E-01	-0.4	6.47E-01	-0.8	1.94E-01	-0.7	1.58E-01	-0.1	1.0E+00	-0.2	1.0E+00	0.2	8.2E-01	-0.1	1.0E+00	0.2	8.1E-01	0.1	1.0E+00	0.2	6.9E-01	0.2	8.1E-01
10523	NHP2	A_24_P63608	NM_017838	-0.8	3.16E-03	-0.7	3.16E-03	-0.8	8.27E-03	-0.9	6.44E-05	-0.3	2.52E-01	-0.1	8.23E-01	-0.3	2.52E-01	-0.4	1.44E-01	-0.2	1.0E+00	0.1	1.0E+00	-0.3	7.1E-01	-0.2	1.0E+00	0.0	9.7E-01	0.0	1.0E+00	-0.1	8.6E-01	0.0	9.7E-01
39294	B3GNT1	A_23_P86900	NM_006876	-0.8	2.47E-02	-0.8	2.47E-02	-0.9	2.87E-02	-0.7	2.31E-02	-0.4	5.45E-01	-0.3	6.05E-01	-0.5	1.72E-01	-0.4	5.45E-01	-0.2	1.0E+00	-0.2	1.0E+00	-0.6	3.4E-01	0.0	1.0E+00	-0.2	9.9E-01	-0.2	1.0E+00	-0.2	4.7E-01	0.0	9.9E-01
38696	GPR125	A_23_P158925	NM_145290	-0.8	8.09E-03	-0.7	1.18E-02	-1.1	8.09E-03	-0.8	1.79E-03	-0.4	1.31E-01	-0.4	1.70E-01	-0.4	1.31E-01	-0.6	2.78E-02	-0.1	1.0E+00	-0.1	1.0E+00	0.0	9.0E-01	-0.1	1.0E+00	0.0	9.9E-01	0.0	1.0E+00	0.1	8.7E-01	0.0	9.9E-01
42782	THC2706736	A_32_P119604	THC2706736	-0.8	5.68E-03	-0.6	5.68E-03	-0.9	1.46E-02	-0.8	1.12E-03	-0.5	2.91E-01	0.0	9.56E-01	-0.5	2.91E-01	-0.5	2.33E-01	0.0	1.0E+00	0.0	1.0E+00	-0.4	7.1E-01	0.0	1.0E+00	-0.2	7.1E-01	0.1	1.0E+00	-0.2	6.7E-01	-0.2	7.1E-01
34890	RAD54B	A_23_P82738	NM_012415	-0.8	2.53E-02	-0.7	3.52E-02	-0.8	2.53E-02	-1.0	4.18E-04	-0.5	2.23E-01	-0.3	6.04E-01	-0.6	2.23E-01	-0.5	1.14E-01	0.0	1.0E+00	-0.1	1.0E+00	0.0	1.0E+00	0.1	1.0E+00	-0.1	9.2E-01	0.0	1.0E+00	-0.1	8.4E-01	-0.1	9.2E-01
5868	ABCD3	A_24_P221154	NM_002858	-0.8	1.30E-02	-0.8	3.94E-02	-1.2	1.30E-02	-0.6	1.22E-02	-0.4	5.62E-01	0.1	9.16E-01	-0.4	3.66E-01	-0.4	5.62E-01	0.0	1.0E+00	0.0	1.0E+00	-0.3	7.3E-01	0.1	1.0E+00	-0.2	7.3E-01	-0.1	1.0E+00	-0.2	7.1E-01	-0.3	7.3E-01
16574	NHEDC2	A_23_P81094	NM_178833	-0.8	3.26E-03	-0.7	4.35E-03	-1.1	3.26E-03	-0.8	2.68E-04	-0.5	3.94E-01	-0.3	5.28E-01	-0.5	3.94E-01	-0.6	5.49E-02	-0.1	1.0E+00	-0.1	1.0E+00	-0.1	8.1E-01	-0.1	1.0E+00	-0.1	5.8E-01	0.0	1.0E+00	-0.1	5.5E-01	-0.2	5.8E-01
14128	DKFZp434H1419	A_23_P369047	AK125369	-0.8	3.05E-02	-0.8	3.43E-02	-1.2	3.05E-02	-0.8	2.81E-02	-0.4	2.10E-01	0.0	9.68E-01	-0.5	2.10E-01	-0.4	1.74E-01	0.1	1.0E+00	0.2	1.0E+00	-0.1	8.3E-01	0.1	1.0E+00	0.1	9.8E-01	0.1	1.0E+00	0.2	7.0E-01	0.0	9.8E-01
39058	SUMF1	A_23_P69242	NM_182760	-0.8	6.28E-04	-0.7	6.28E-04	-1.2	1.05E-03	-0.8	9.32E-05	-0.7	8.92E-03	-0.5	4.71E-02	-1.0	3.89E-03	-0.7	8.92E-03	-0.1	1.0E+00	-0.1	1.0E+00	-0.4	3.8E-01	-0.1	1.0E+00	-0.1	9.0E-01	-0.1	1.0E+00	-0.3	2.9E-01	-0.1	9.0E-01
41107	CYP4V2	A_24_P945228	NM_207352	-0.8	5.72E-03	-0.7	5.72E-03	-1.0	1.25E-02	-0.8	2.39E-03	-0.2	6.82E-01	0.2	8.36E-01	-0.3	6.21E-01	-0.2	6.82E-01	-0.1	1.0E+00	-0.1	1.0E+00	-0.2	7.3E-01	0.1	1.0E+00	-0.2	5.3E-01	-0.2	1.0E+00	-0.2	4.9E-01	-0.4	5.3E-01
38554	SIP1	A_23_P88194	NM_003616	-0.8	7.09E-03	-0.7	1.66E-02	-1.2	7.09E-03	-0.8	2.43E-03	-0.4	2.09E-01	-0.3	5.18E-01	-1.2	9.60E-02	-0.4	2.09E-01	0.0	1.0E+00	-0.1	1.0E+00	0.0	9.4E-01	0.1	1.0E+00	-0.1	7.1E-01	-0.1	1.0E+00	-0.1	7.1E-01	-0.2	6.6E-01
33744	FAM114A2	A_23_P41908	NM_018691	-0.8	9.21E-03	-0.7	9.21E-03	-0.8	1.77E-02	-0.8	1.33E-03	-0.5	3.38E-01	-0.1	9.09E-01	-0.6	3.38E-01	-0.5	6.97E-02	-0.1	1.0E+00	-0.1	1.0E+00	-0.2	7.4E-01	0.0	1.0E+00	-0.4	1.4E-01	-0.2	1.0E+00	-0.4	1.4E-01	-0.5	7.0E-02
36977	FGGY	A_23_P22169	NM_018291	-0.8	1.89E-02	-0.8	2.20E-02	-0.8	1.89E-02	-0.8	1.56E-03	0.0	9.44E-01	0.0	9.75E-01	0.0	9.44E-01	-0.4	1.92E-01	-0.3	1.0E+00	-0.3	1.0E+00	-0.4	7.1E-01	0.1	1.0E+00	0.0	9.4E-01	-0.2	1.0E+00	0.0	9.4E-01	0.1	9.1E-01
10261	FAM44C	A_23_P345212	BC021740	-0.8	3.77E-02	-0.7	3.77E-02	-0.8	4.95E-02	-1.1	3.77E-04	-0.5	4.21E-01	-0.3	5.55E-01	-0.5	4.21E-01	-0.5	4.96E-02	0.0	1.0E+00	-0.2	1.0E+00	0.0	9.6E-01	0.0	1.0E+00	0.1	9.3E-01	0.1	1.0E+00	0.1	8.8E-01	0.1	9.3E-01
6228	CACYBP	A_24_P45379	NM_014412	-0.8	7.52E-03	-0.8	1.43E-02	-1.3	7.52E-03	-0.7	5.24E-03	-0.3	6.31E-01	-0.2	8.60E-01	-0.3	6.31E-01	-0.4	5.52E-01	-0.1	1.0E+00	-0.1	1.0E+00	-0.1	8.7E-01	0.2	1.0E+00	-0.3	4.2E-01	-0.1	1.0E+00	-0.3	3.8E-01	-0.4	4.2E-01
1067	PTPLAD1	A_23_P99920	AF161470	-0.8	2.53E-03	-0.8	4.52E-02	-2.0	1.21E-03	-0.7	2.53E-03	-0.5	4.43E-01	-0.5	4.12E-01	-0.4	6.61E-01	-0.5	4.43E-01	0.1	1.0E+00	0.1	1.0E+00	0.2	8.4E-01	0.0	1.0E+00	0.1	9.8E-01	0.0	1.0E+00	0.2	7.9E-01	0.1	9.8E-01
30838	C22orf30	A_24_P404628	NM_173566	-0.8	1.04E-02	-0.8	1.04E-02	-1.0	1.68E-02	-0.8	4.87E-03	-0.2	8.63E-01	-0.5	6.72E-01	-0.1	9.12E-01	-0.2	8.63E-01	-0.1	1.0E+00	0.3	1.0E+00	-0.2	8.6E-01	-0.1	1.0E+00	0.0	9.9E-01	0.0	1.0E+00	0.0	9.9E-01	0.1	9.8E-01
34502	LTV1	A_23_P122624	NM_032860	-0.8	7.67E-03	-0.6	1.48E-02	-1.0	7.67E-03	-0.8	2.24E-03	-0.8	4.16E-02	-0.6	4.69E-02	-0.9	4.16E-02	-0.8	8.81E-03	-0.1	1.0E+00	-0.2	1.0E+00	-0.1	8.5E-01	0.0	1.0E+00	-0.2	9.9E-01	-0.2	1.0E+00	0.0	9.9E-01	-0.2	7.8E-01
898	NDUFV2	A_23_P130418	NM_021074	-0.8	2.10E-03	-0.8	7.23E-03	-1.2	2.09E-03	-0.7	2.10E-03	-0.5	2.12E-01	-0.4	2.12E-01	-0.9	2.31E-01	-0.5	5.72E-02	0.1	1.0E+00	0.2	1.0E+00	0.0	9.3E-01	0.1	1.0E+00	-0.1	8.8E-01	-0.2	1.0E+00	-0.2	5.3E-01	-0.1	8.8E-01
42659	CR590573	A_32_P154445	CR590573	-0.8	1.59E-02	-0.6	4.04E-02	-0.8	1.59E-02	-0.8	1.18E-03	-0.4	3.25E-01	-0.1	8.43E-01	-0.4	3.25E-01	-0.6	2.49E-01	0.2	1.0E+00	0.2	1.0E+00	0.0	9.9E-01	0.4	1.0E+00	0.0	9.9E-01	0.0	1.0E+00	0.0	9.9E-01	-0.1	9.1E-01
272	THC2642694	A_32_P105397	THC2642694	-0.8	1.35E-02	-0.7	1.35E-02	-1.1	1.52E-02	-0.8	4.05E-03	-0.3	5.77E-01	-0.1	9.12E-01	-0.5	3.17E-01	-0.3	5.77E-01	0.0	1.0E+00	0.0	1.0E+00	-0.2	7.9E-01	0.2	1.0E+00	-0.3	4.0E-01	-0.1	1.0E+00	-0.3	4.0E-01	-0.4	3.9E-01
26508	TET1	A_32_P202134	NM_030625	-0.8	3.08E-02	-0.8	3.08E-02	-1.0	4.20E-02	-0.6	2.23E-02	-0.2	6.57E-01	-0.2	6.57E-01	-0.3	5.10E-01	-0.1	7.24E-01	-0.1	1.0E+00	-0.2	1.0E+00	-0.1	7.6E-01	0.0	1.0E+00	-0.2	8.1E-01	-0.2	1.0E+00	-0.2	5.3E-01	-0.2	8.1E-01
30562	BX648207	A_32_P15156	BX648207	-0.8	6.37E-03	-0.6	3.42E-02	-1.3	6.37E-03	-0.8	6.36E-03	-0.5	3.03E-01	-0.1	9.18E-01	-1.0	2.29E-01	-0.5	3.03E-01	0.1	1.0E+00	0.1	1.0E+00	-0.1	9.3E-01	0.1	1.0E+00	-0.1	9.4E-01	-0.2	1.0E+00	0.0	9.4E-01	-0.2	6.7E-01
13375	ARMC9	A_24_P945408	AB058771	-0.7	1.75E-03	-0.8	1.48E-03	-0.7	1.81E-02	-0.6	1.75E-03	-0.2	7.89E-01	-0.2	7.89E-01	-0.1	8.43E-01	-0.3	5.56E-01	-0.1	1.0E+00	-0.1	1.0E+00	-0.2	7.1E-01	0.0	1.0E+00	-0.1	9.2E-01	-0.1	1.0E+00	-0.2	4.6E-01	0.1	9.2E-01
36075	ZMYM6	A_23_P201845	NM_007167	-0.7	5.31E-03	-0.7	5.31E-03	-0.8	2.26E-02	-0.7	4.13E-03	-0.3	5.61E-01	-0.3	7.36E-01	-0.4	4.51E-01	-0.3	5.61E-01	-0.1	1.0E+00	-0.1	1.0E+00	-0.5	6.0E-01	-0.1	1.0E+00	-0.2	9.1E-01	-0.2	1.0E+00	-0.3	4.6E-01	-0.1	9.1E-01
22853	PDSS2	A_23_P134167	NM_020381	-0.7	5.04E-04	-0.7	5.04E-04	-1.3	8.35E-04	-0.7	1.04E-04	-0.6	9.68E-02	-0.4	2.32E-01	-1.3	1.27E-02	-0.6	9.68E-02	-0.2	1.0E+00	-0.2	1.0E+00	-0.4	6.7E-01	-0.2	1.0E+00	0.0	9.7E-01	0.0	1.0E+00	0.0	9.6E-01	0.0	9.7E-01
3250	AW946823	A_32_P96752	AW946823	-0.7	8.78E-03	-0.6	2.01E-02	-2.8	5.35E-04	-0.7	8.78E-03	-0.2	6.81E-01	0.2	7.95E-01	-0.7	5.32E-01	-0.2	6.81E-01	0.0	1.0E+00	0.0	1.0E+00	0.0	9.8E-01	0.3	1.0E+00	0.0	9.9E-01	0.1	1.0E+00	0.0	9.9E-01	-0.1	9.7E-01
28142	A_32_P148407	A_32_P148407	A_32_P148407	-0.7	3.28E-02	-0.7	3.28E-02	-0.9	4.28E-02	-0.6	1.41E-02	-0.6	2.49E-02	-0.4	3.39E-01	-0.8	1.26E-02	-0.6	2.49E-02	0.0	1.0E+00	0.1	1.0E+00	-0.1	7.7E-01	0.0	1.0E+00	0.0	9.7E-01	0.0	1.0E+00	0.1	7.9E-01	0.0	9.7E-01
22327	APOO	A_23_P61127	NM_024122	-0.7	2.82E-02	-0.7	3.11E-02	-1.5	1.69E-02	-0.7	2.82E-02	-0.7	1.69E-02	-0.3	1.98E-01	-0.9	6.62E-03	-0.7	1.69E-02	0.0	1.0E+00	0.0	1.0E+00	-0.1	7.7E-01	0.0	1.0E+00	-0.1	9.1E-01	-0.1	1.0E+00	-0.1	6.9E-01	-0.1	9.1E-01
37085	C5orf35	A_23_P433111	NM_153706	-0.7	5.45E-04	-0.9	5.45E-04	-0.7	8.27E-03	-0.7	1.15E-04	-0.7	6.14E-02	-0.5	6.14E-02	-0.8	2.14E-01	-0.7	5.80E-03	-0.1	1.0E+00	-0.1	1.0E+00	0.0	9.5E-01	-0.1	1.0E+00	0.0	9.7E-01	0.0	1.0E+00	-0.2	4.7E-01	0.0	9.7E-01
18627	PRKAR1B	A_23_P83599	NM_002735	-0.7	1.27E-03	-0.6	1.38E-02	-1.9	4.49E-04	-0.7	1.27E-03	-0.4	2.80E-01	-0.4	2.80E-01	-1.4	1.10E-02	-0.2	6.24E-01	-0.1	1.0E+00	-0.1	1.0E+00	-0.7	2.9E-01	-0.1	1.0E+00	-0.1	8.3E-01	-0.1	1.0E+00	-0.1	8.3E-01	0.2	7.8E-01
38256	FBXO22	A_23_P65870	NM_012170	-0.7	8.35E-04	-0.6	1.21E-02	-1.4	8.35E-04	-0.7	2.08E-04	-0.5	2.28E-01	-0.1	8.32E-01	-1.0	2.28E-01	-0.5	5.35E-02	0.0	1.0E+00	0.0	1.0E+00	-0.1	7.9E-01	0.0	1.0E+00	0.1	9.6E-01	0.0	1.0E+00	0.1	8.5E-01	0.1	9.6E-01
5299	MGAT5	A_23_P16743	NM_002410	-0.7	7.87E-04	-0.7	7.87E-04	-0.8	4.63E-03	-0.6	2.76E-04	-0.3	6.30E-01	-0.3	6.30E-01	-0.1	9.57E-01	-0.4	3.89E-01	0.0	1.0E+00	0.2	1.0E+00	-0.3	7.1E-01	0.0	1.0E+00	-0.2	7.0E-01	0.0	1.0E+00	-0.2	6.7E-01	-0.3	7.0E-01
7722	TM9SF3	A_24_P218509	NM_020123	-0.7	1.53E-03	-0.7	7.33E-03	-1.1	1.53E-03	-0.6	5.57E-04	-0.4	1.60E-01	-0.3	1.60E-01	-0.8	2.68E-01	-0.4	1.02E-01	0.0	1.0E+00	0.0	1.0E+00	0.0	9.9E-01	0.0	1.0E+00	0.1	8.6E-01	0.1	1.0E+00	0.2	5.4E-01	0.1	8.6E-01
32834	DMTF1	A_23_P8664	NM_021145	-0.7	3.78E-03	-0.7	3.78E-03	-1.0	8.08E-03	-0.7	9.75E-04	-0.7	2.17E-02	-0.7	7.17E-02	-1.1	2.17E-02	-0.7	1.56E-02	-0.2	1.0E+00	-0.2	1.0E+00	-0.2	7.3E-01	-0.2	1.0E+00	0.0	1.0E+00	0.0	1.0E+00	0.1	9.2E-01	0.0	1.0E+00
33842	GNL3L	A_23_P22499	NM_019067	-0.7	1.29E-02	-0.7	1.29E-02	-0.9	3.92E-02	-0.7	5.31E-03	-0.3	4.78E-01	-0.6	1.69E-01	-0.3	5.96E-01	-0.3	4.78E-01	0.0	1.0E+00	0.0	1.0E+00	0.1	8.3E-01	-0.1	1.0E+00	0.2	9.7E-01	0.2	1.0E+00	0.0	9.7E-01	0.4	8.0E-01
8025	TMEM14A	A_23_P42080	NM_014051	-0.7	8.32E-03	-0.6	3.32E-02	-1.9	3.43E-03	-0.7	8.32E-03	-0.5	4.02E-01	-0.3	5.81E-01	-0.7	4.02E-01	-0.5	8.99E-02	0.1	1.0E+00	0.1	1.0E+00	0.0	9.6E-01	0.1	1.0E+00	-0.1	7.6E-01	0.0	1.0E+00	-0.1	7.6E-01	-0.2	6.5E-01
7684	ABCB10	A_24_P324449	NM_012089	-0.7	4.41E-03	-0.7	4.41E-03	-0.8	3.20E-02	-0.6	3.72E-03	-0.2	6.96E-01	-0.2	6.96E-01	0.0	9.21E-01	-0.3	1.42E-01	-0.1	1.0E+00	-0.1	1.0E+00	-0.3	7.1E-01	-0.1	1.0E+00	-0.1	9.3E-01	-0.1	1.0E+00	-0.1	9.3E-01	-0.4	7.3E-01
8012	CCNF	A_23_P37954	NM_001761	-0.7	5.64E-03	-0.6	5.64E-03	-0.7	2.11E-02	-0.9	5.30E-04	-0.6	2.24E-01	-0.6	3.51E-01	-0.9	6.12E-02	-0.5	2.24E-01	-0.3	1.0E+00	-0.1	1.0E+00	-0.4	6.7E-01	-0.3	1.0E+00	-0.2	8.3E-01	-0.2	1.0E+00	-0.2	3.9E-01	-0.1	8.3E-01
6572	AK023737	A_24_P333306	AK023737	-0.7	1.58E-02	-0.7	9.55E-03	-0.9	3.85E-02	-0.6	1.58E-02	-0.4	4.78E-01	-0.3	6.89E-01	-0.6	2.57E-01	-0.4	4.78E-01	0.0	1.0E+00	0.0	1.0E+00	-0.3	7.2E-01	0.1	1.0E+00	-0.1	8.2E-01	0.0	1.0E+00	-0.1	8.2E-01	-0.2	8.2E-01
10112	UBE2N	A_23_P116829	NM_003348	-0.7	2.30E-03	-0.7	5.07E-03	-1.4	2.30E-03	-0.7	2.01E-03	-0.4	3.47E-01	-0.2	7.31E-01	-1.1	2.42E-01	-0.4	3.47E-01	0.0	1.0E+00	0.0	1.0E+00	0.0	9.4E-01	0.1	1.0E+00	0.0	1.0E+00	0.0	1.0E+00	0.1	7.8E-01	0.0	1.0E+00
42552	ZNRF3	A_24_P162373	NM_032173	-0.7	1.06E-02	-0.6	1.83E-02	-1.0	1.06E-02	-0.7	3.79E-03	-0.6	1.01E-01	-1.0	1.01E-01	-0.1	8.14E-01	-0.6	8.42E-02	0.0	1.0E+00	0.2	1.0E+00	0.0	9.9E-01	-0.2	1.0E+00	0.1	9.5E-01	0.1	1.0E+00	0.1	9.5E-01	0.3	8.2E-01
40158	PA2G4	A_24_P221123	NM_006191	-0.7	9.57E-03	-0.7	2.06E-02	-0.8	9.57E-03	-0.6	8.42E-04	-0.5	1.24E-01	-0.8	1.01E-02	-0.5	1.24E-01	-0.4	2.81E-01	0.0	1.0E+00	0.1	1.0E+00	0.0	9.6E-01	-0.2	1.0E+00	0.0	9.4E-01	-0.1	1.0E+00	0.0	9.4E-01	0.1	9.2E-01
24550	BG534208	A_23_P8123	BG534208	-0.7	1.33E-02	-0.7	8.20E-03	-0.9	2.97E-02	-0.6	1.33E-02	-0.5	4.28E-01	-0.6	4.28E-01	-0.5	4.34E-01	-0.5	4.24E-01	-0.1	1.0E+00	0.2	1.0E+00	-0.1	8.0E-01	-0.1	1.0E+00	0.1	9.3E-01	0.2	1.0E+00	0.0	9.3E-01	0.1	8.8E-01
11273	BCL2L1	A_23_P210886	NM_138578	-0.7	7.66E-03	-0.7	2.14E-02	-1.3	1.71E-03	-0.6	7.66E-03	-0.3	4.41E-01	-0.2	5.81E-01	-0.9	2.14E-01	-0.3	4.41E-01	-0.1	1.0E+00	0.1	1.0E+00	-0.1	9.1E-01	-0.1	1.0E+00	0.1	9.1E-01	-0.1	1.0E+00	0.1	9.1E-01	0.3	8.4E-01
6968	TERF1	A_32_P220696	NM_017489	-0.7	2.48E-03	-0.7	4.83E-03	-1.3	2.48E-03	-0.7	2.19E-03	-0.6	4.59E-02	-0.1	7.78E-01	-0.6	4.59E-02	-0.6	4.16E-02	-0.1	1.0E+00	-0.1	1.0E+00	-0.1	8.1E-01	0.0	1.0E+00	-0.1	9.3E-01	0.0	1.0E+00	-0.1	9.3E-01	-0.1	8.9E-01
3649	TAF9B	A_23_P254790	NM_015975	-0.7	9.76E-03	-0.7	3.91E-02	-1.0	9.76E-03	-0.6	3.93E-03	0.0	9.38E-01	0.0	9.41E-01	0.0	9.38E-01	-0.2	7.23E-01	0.1	1.0E+00	0.1	1.0E+00	0.1	9.4E-01	0.3	1.0E+00	-0.1	9.2E-01	-0.1	1.0E+00	-0.1	9.1E-01	-0.2	9.2E-01
38727	SECISBP2	A_24_P119482	NM_024077	-0.7	1.15E-03	-1.0	1.74E-04	-0.7	7.90E-03	-0.7	1.15E-03	-0.6	1.51E-01	-0.5	2.39E-01	-0.6	1.51E-01	-0.9	8.50E-03	0.0	1.0E+00	0.2	1.0E+00	0.0	9.6E-01	0.0	1.0E+00	-0.1	9.8E-01	-0.1	1.0E+00	-0.2	7.4E-01	0.0	9.8E-01
18600	SETD4	A_24_P228637	NM_001007259	-0.7	2.09E-02	-0.7	2.22E-02	-1.1	2.09E-02	-0.7	5.91E-03	-0.5	1.76E-01	-0.2	5.14E-01	-0.5	1.76E-01	-0.5	7.58E-02	-0.2	1.0E+00	-0.1	1.0E+00	-0.2	7.1E-01	-0.2	1.0E+00	0.0	9.8E-01	0.0	1.0E+00	0.1	7.8E-01	0.0	9.8E-01
37047	SEPT9	A_24_P147910	NM_006640	-0.7	2.48E-03	-1.0	1.15E-03	-0.7	3.23E-02	-0.7	2.48E-03	-0.4	5.43E-01	-0.4	5.43E-01	0.0	9.67E-01	-0.6	2.92E-01	0.1	1.0E+00	0.1	1.0E+00	-0.1	9.1E-01	0.1	1.0E+00	-0.3	7.8E-01	-0.3	1.0E+00	-0.3	6.9E-01	-0.4	7.8E-01
9413	THC2641611	A_23_P165541	THC2641611	-0.7	4.56E-03	-0.7	4.56E-03	-0.9	7.71E-03	-0.7	1.80E-03	-0.1	7.21E-01	0.0	9.53E-01	-0.5	5.11E-01	-0.1	7.21E-01	0.0	1.0E+00	0.0	1.0E+00	-0.1	9.0E-01	0.1	1.0E+00	-0.1	8.1E-01	0.1	1.0E+00	-0.2	4.7E-01	-0.1	8.1E-01
1230	ZNF766	A_24_P935276	NM_001010851	-0.7	1.90E-03	-0.7	7.65E-03	-1.3	1.90E-03	-0.7	8.21E-04	-0.5	5.98E-01	-0.5	5.98E-01	-0.3	7.60E-01	-0.5	4.41E-01	0.1	1.0E+00	0.1	1.0E+00	0.2	7.5E-01	0.0	1.0E+00	-0.2	8.6E-01	0.1	1.0E+00	-0.2	6.7E-01	-0.2	8.6E-01
4577	A_24_P894132	A_24_P894132	A_24_P894132	-0.7	1.74E-03	-0.7	1.74E-03	-1.0	4.80E-03	-0.7	4.20E-04	-0.5	5.12E-02	-0.4	2.10E-01	-0.5	5.12E-02	-0.5	3.01E-02	0.0	1.0E+00	-0.1	1.0E+00	0.0	1.0E+00	0.1	1.0E+00	0.0	9.8E-01	0.2	1.0E+00	-0.1	6.9E-01	0.0	9.8E-01
757	TMEM128	A_23_P29924	NM_032927	-0.7	3.33E-04	-0.7	1.04E-03	-1.6	3.33E-04	-0.7	9.93E-05	-0.7	2.93E-02	-0.3	5.09E-01	-2.0	4.76E-03	-0.7	2.93E-02	-0.1	1.0E+00	-0.1	1.0E+00	-0.5	6.8E-01	0.0	1.0E+00	0.0	9.9E-01	0.2	1.0E+00	0.0	9.9E-01	0.0	9.9E-01
9368	SLC36A4	A_23_P345093	NM_152313	-0.7	4.49E-03	-0.7	1.83E-02	-2.0	3.96E-04	-0.7	4.49E-03	-0.4	3.20E-01	-0.3	3.20E-01	-0.9	3.70E-01	-0.4	1.36E-01	0.1	1.0E+00	0.1	1.0E+00	0.1	8.6E-01	0.1	1.0E+00	0.0	9.7E-01	0.1	1.0E+00	0.0	9.7E-01	-0.2	7.3E-01

Feature	Gene symbol	Probe	Transcript	72 hour								48 hours								24 hours								10 hours							
				siCASP8AP2								siCASP8AP2								siCASP8AP2								siCASP8AP2							
				Median	Median	1	1	3	3	6	6	Median	Median	1	1	3	3	6	6	Median	Median	1	1	3	3	6	6	Median	Median	1	1	3	3	6	6
				M	q	M	q	M	q	M	q	M	q	M	q	M	q	M	q	M	q	M	q	M	q	M	q	M	q	M	q	M	q	M	q
41605	THC2543594	A_32_P231265	THC2543594	-0.7	6.69E-03	-0.7	1.55E-02	-1.1	6.69E-03	-0.7	6.18E-03	-0.4	3.86E-01	0.0	9.59E-01	-0.4	3.86E-01	-0.4	2.57E-01	-0.1	1.0E+00	-0.1	1.0E+00	-0.3	7.1E-01	0.0	1.0E+00	-0.4	6.1E-01	-0.1	1.0E+00	-0.4	3.6E-01	-0.4	6.1E-01
4058	MRPS10	A_23_P70417	NM_018141	-0.7	2.71E-02	-0.7	2.71E-02	-0.7	3.64E-02	-0.9	2.89E-03	-0.6	1.27E-01	-0.6	3.59E-01	-0.6	1.27E-01	-0.6	1.25E-01	-0.1	1.0E+00	-0.1	1.0E+00	-0.2	7.8E-01	-0.1	1.0E+00	-0.1	9.3E-01	0.0	1.0E+00	-0.1	8.9E-01	-0.1	9.3E-01
6016	THC2679484	A_32_P48526	THC2679484	-0.7	2.21E-02	-0.7	2.21E-02	-0.9	3.55E-02	-0.7	1.99E-02	-0.4	2.75E-01	-0.2	7.38E-01	-0.5	3.83E-02	-0.4	2.75E-01	-0.1	1.0E+00	-0.1	1.0E+00	-0.2	7.3E-01	0.0	1.0E+00	0.0	9.3E-01	0.0	1.0E+00	0.0	9.0E-01	-0.1	9.3E-01
8538	METTL9	A_24_P202139	NM_016025	-0.7	1.65E-03	-0.7	1.65E-03	-1.2	2.31E-03	-0.7	3.10E-04	-0.3	4.68E-01	-0.2	7.89E-01	-0.6	1.63E-01	-0.3	4.68E-01	0.0	1.0E+00	0.0	1.0E+00	-0.5	7.1E-01	0.0	1.0E+00	-0.3	7.1E-02	-0.1	1.0E+00	-0.3	6.2E-02	-0.5	7.1E-02
1951	PPP3CC	A_23_P157495	NM_005605	-0.7	1.75E-02	-0.7	1.75E-02	-0.7	3.68E-02	-0.9	1.16E-03	-0.6	3.27E-01	-0.4	3.62E-01	-0.6	3.27E-01	-0.7	8.05E-02	-0.1	1.0E+00	-0.1	1.0E+00	-0.1	8.6E-01	-0.2	1.0E+00	0.1	9.7E-01	0.1	1.0E+00	0.2	7.9E-01	0.1	9.7E-01
2888	NECAP1	A_32_P144920	NM_015509	-0.7	5.86E-03	-0.7	1.09E-02	-0.9	5.86E-03	-0.7	1.98E-03	-0.6	3.16E-01	-0.1	8.45E-01	-0.8	3.16E-01	-0.6	6.44E-02	-0.1	1.0E+00	-0.1	1.0E+00	-0.3	7.1E-01	-0.1	1.0E+00	0.2	9.8E-01	0.2	1.0E+00	0.3	5.8E-01	0.0	9.8E-01
23983	NDUFB4	A_23_P69468	NM_004547	-0.7	1.22E-02	-0.7	1.41E-02	-1.1	1.22E-02	-0.7	4.17E-03	-0.2	2.50E-01	-0.2	3.70E-01	-0.2	2.50E-01	-0.7	1.12E-02	-0.1	1.0E+00	0.0	1.0E+00	-0.1	8.1E-01	-0.2	1.0E+00	-0.1	9.0E-01	0.0	1.0E+00	-0.1	7.3E-01	-0.1	9.0E-01
27119	PUS7	A_23_P82478	NM_019042	-0.7	1.38E-02	-0.7	1.38E-02	-0.7	3.04E-02	-0.8	1.54E-03	-0.8	3.33E-02	-0.4	4.33E-01	-0.8	3.33E-02	-0.8	5.85E-03	-0.1	1.0E+00	-0.1	1.0E+00	-0.1	8.5E-01	-0.1	1.0E+00	-0.1	9.4E-01	0.0	1.0E+00	-0.1	9.4E-01	-0.1	9.3E-01
20136	RG9MTD2	A_23_P398637	NM_152292	-0.7	7.35E-04	-0.7	1.20E-02	-1.7	7.35E-04	-0.7	4.02E-04	-0.4	4.33E-01	-0.1	8.67E-01	-0.5	4.33E-01	-0.4	2.82E-01	-0.1	1.0E+00	-0.1	1.0E+00	-0.1	7.9E-01	0.0	1.0E+00	0.0	9.9E-01	0.0	1.0E+00	0.0	9.9E-01	-0.2	8.0E-01
22696	CDCA3	A_24_P218979	NM_031299	-0.7	1.76E-02	-0.7	1.76E-02	-0.8	1.76E-02	-0.7	5.11E-03	-0.5	3.87E-02	-0.5	3.87E-02	-0.7	2.20E-02	-0.5	6.25E-02	-0.2	1.0E+00	-0.2	1.0E+00	-0.3	4.6E-01	-0.2	1.0E+00	0.1	9.1E-01	0.1	1.0E+00	0.0	8.9E-01	0.1	9.1E-01
4420	TMTC4	A_24_P278853	NM_032813	-0.7	1.77E-03	-0.8	1.77E-03	-0.7	4.04E-02	-0.7	1.26E-03	-0.2	6.59E-01	-0.2	6.59E-01	-0.1	9.15E-01	-0.5	7.72E-02	-0.1	1.0E+00	-0.1	1.0E+00	-0.4	7.1E-01	-0.1	1.0E+00	0.0	1.0E+00	0.0	1.0E+00	-0.1	7.6E-01	0.0	1.0E+00
14314	MRPS18A	A_23_P122387	NM_018135	-0.7	5.54E-03	-0.6	5.54E-03	-0.7	1.72E-02	-0.9	1.88E-04	-0.6	6.77E-02	-0.2	6.34E-01	-0.8	6.77E-02	-0.6	1.67E-02	0.0	1.0E+00	-0.1	1.0E+00	0.0	9.8E-01	0.0	1.0E+00	0.0	9.6E-01	0.0	1.0E+00	0.0	9.4E-01	0.0	9.6E-01
7493	AK095715	A_24_P817236	AK095715	-0.7	6.30E-03	-0.7	6.70E-03	-1.7	2.44E-03	-0.7	6.30E-03	-0.3	7.42E-01	-0.3	7.97E-01	-0.9	2.58E-01	-0.3	7.42E-01	-0.1	1.0E+00	-0.1	1.0E+00	-0.4	7.1E-01	0.2	1.0E+00	-0.2	7.5E-01	-0.2	1.0E+00	-0.3	4.6E-01	-0.2	7.5E-01
37214	SMA4	A_24_P568190	BC035411	-0.7	2.16E-02	-0.7	2.16E-02	-0.9	4.54E-02	-0.7	9.50E-03	-0.8	7.48E-02	-0.8	1.36E-01	-0.9	7.18E-02	-0.8	7.48E-02	0.0	1.0E+00	0.1	1.0E+00	-0.2	8.3E-01	0.0	1.0E+00	0.0	9.8E-01	-0.2	1.0E+00	0.0	9.6E-01	0.0	9.8E-01
41516	DBT	A_32_P50005	NM_001918	-0.7	4.11E-03	-0.8	4.11E-03	-0.7	1.69E-02	-0.7	1.45E-03	-0.5	3.25E-01	-0.3	3.25E-01	-0.5	3.29E-01	-0.5	1.16E-01	-0.1	1.0E+00	-0.1	1.0E+00	-0.1	7.6E-01	0.0	1.0E+00	0.0	1.0E+00	0.0	1.0E+00	0.0	1.0E+00	0.1	9.2E-01
2561	C11orf1	A_23_P150350	NM_022761	-0.7	1.29E-02	-0.6	3.64E-02	-1.4	1.29E-02	-0.7	1.02E-02	-0.4	3.64E-01	-0.4	1.72E-01	-0.6	3.94E-01	-0.4	3.64E-01	0.1	1.0E+00	0.1	1.0E+00	0.1	8.5E-01	0.1	1.0E+00	-0.1	8.4E-01	0.1	1.0E+00	-0.1	8.2E-01	-0.1	8.4E-01
16472	GARNL1	A_23_P151565	NM_014990	-0.7	1.71E-02	-0.7	2.31E-02	-1.3	1.71E-02	-0.7	1.08E-02	-0.5	5.52E-02	-0.2	4.61E-01	-0.5	5.52E-02	-0.5	1.80E-02	-0.2	1.0E+00	0.0	1.0E+00	-0.3	7.1E-01	-0.2	1.0E+00	0.0	9.9E-01	-0.1	1.0E+00	0.1	8.6E-01	0.0	9.9E-01
37779	FAM165B	A_23_P82051	NM_058182	-0.7	4.16E-03	-0.6	4.16E-03	-0.7	1.14E-02	-0.8	1.40E-03	-0.4	2.02E-01	-0.4	2.02E-01	-0.1	7.89E-01	-0.6	1.63E-02	-0.1	1.0E+00	-0.1	1.0E+00	0.0	9.5E-01	-0.1	1.0E+00	0.0	9.4E-01	0.0	1.0E+00	0.1	8.6E-01	-0.1	9.4E-01
10486	RPL32P3	A_24_P841622	AK096589	-0.7	7.41E-03	-0.7	7.41E-03	-0.9	1.79E-02	-0.7	2.76E-03	-0.9	4.01E-02	-0.6	1.17E-01	-1.2	1.62E-02	-0.9	4.01E-02	0.0	1.0E+00	0.0	1.0E+00	-0.3	7.1E-01	0.0	1.0E+00	0.1	9.1E-01	0.1	1.0E+00	0.1	7.6E-01	0.1	9.1E-01
34863	AF161353	A_24_P358116	AF161353	-0.7	5.69E-03	-0.9	5.69E-03	-0.7	1.24E-02	-0.6	9.41E-04	-0.6	6.59E-02	-0.4	1.85E-01	-0.6	6.59E-02	-0.7	9.08E-03	-0.2	1.0E+00	0.0	1.0E+00	-0.2	7.3E-01	-0.2	1.0E+00	0.1	8.8E-01	0.1	1.0E+00	0.1	8.1E-01	0.1	8.8E-01
2669	PAFAH2	A_23_P137765	NM_000437	-0.7	8.79E-03	-0.7	1.25E-02	-1.1	8.79E-03	-0.7	2.25E-03	-0.5	4.01E-01	-0.3	6.53E-01	-0.5	4.01E-01	-0.5	1.96E-01	-0.1	1.0E+00	-0.1	1.0E+00	-0.2	7.1E-01	0.0	1.0E+00	-0.2	6.5E-01	-0.1	1.0E+00	-0.3	3.5E-01	-0.2	6.5E-01
23817	C8orf33	A_23_P33119	NM_023080	-0.7	1.98E-03	-0.7	3.78E-02	-1.7	1.27E-03	-0.7	1.98E-03	-0.2	7.98E-01	-0.2	8.37E-01	-0.6	5.28E-01	-0.2	7.98E-01	0.0	1.0E+00	0.0	1.0E+00	0.0	1.0E+00	0.0	1.0E+00	0.1	8.6E-01	0.0	1.0E+00	0.1	8.2E-01	0.2	8.6E-01
33042	THC2626557	A_24_P345781	THC2626557	-0.7	1.30E-02	-0.7	1.98E-02	-1.2	1.30E-02	-0.7	8.11E-03	-0.2	8.00E-01	-0.1	9.28E-01	-0.2	8.00E-01	-0.6	9.79E-02	0.0	1.0E+00	0.0	1.0E+00	-0.2	7.6E-01	0.2	1.0E+00	-0.3	5.1E-01	-0.2	1.0E+00	-0.3	4.7E-01	-0.4	5.1E-01
39778	VPS24	A_23_P79510	NM_016079	-0.7	2.35E-03	-0.7	2.85E-03	-1.2	2.35E-03	-0.6	1.26E-03	-0.4	2.82E-01	-0.4	3.59E-01	-0.4	2.82E-01	-0.6	6.74E-02	0.1	1.0E+00	0.0	1.0E+00	0.1	7.6E-01	0.2	1.0E+00	0.7	9.5E-01	0.7	1.0E+00	0.7	8.4E-01	0.5	9.5E-01
40951	THC2551927	A_32_P70519	THC2551927	-0.7	7.35E-04	-0.7	7.35E-04	-0.7	9.12E-03	-0.6	6.32E-04	-0.2	6.37E-01	-0.2	6.92E-01	-0.2	4.65E-01	-0.1	6.37E-01	0.0	1.0E+00	0.0	1.0E+00	-0.1	8.2E-01	0.1	1.0E+00	-0.3	7.6E-01	0.2	1.0E+00	-0.3	5.6E-01	-0.3	7.6E-01
959	LRRC40	A_23_P148969	NM_017768	-0.7	7.59E-03	-0.6	1.28E-02	-2.9	3.12E-04	-0.7	7.59E-03	-0.3	5.20E-01	-0.2	7.83E-01	-1.6	8.79E-03	-0.3	5.20E-01	0.1	1.0E+00	0.1	1.0E+00	-0.4	7.1E-01	0.2	1.0E+00	-0.3	6.5E-01	0.0	1.0E+00	-0.3	4.0E-01	-0.4	6.5E-01
37625	CIAPIN1P	A_24_P332364	XR_015541	-0.7	9.63E-04	-0.7	1.17E-03	-1.3	6.09E-04	-0.7	9.63E-04	-0.3	5.69E-01	0.0	9.74E-01	-0.6	2.63E-01	-0.3	5.69E-01	0.0	1.0E+00	0.0	1.0E+00	-0.3	7.3E-01	0.3	1.0E+00	-0.3	8.6E-01	-0.3	1.0E+00	-0.2	7.3E-01	-0.3	8.6E-01
3767	HDAC6	A_24_P205137	BC011498	-0.7	9.07E-03	-0.8	9.07E-03	-0.7	2.43E-02	-0.6	1.89E-03	-0.3	6.31E-01	-0.2	8.11E-01	-0.3	6.31E-01	-0.5	1.76E-01	0.0	1.0E+00	0.0	1.0E+00	-0.2	7.4E-01	0.1	1.0E+00	-0.4	4.9E-01	-0.2	1.0E+00	-0.4	3.4E-01	-0.5	4.9E-01
4153	BM475547	A_32_P40456	BM475547	-0.7	9.36E-03	-0.7	9.36E-03	-1.1	1.79E-02	-0.7	5.44E-03	-0.9	7.45E-02	-0.8	1.55E-01	-1.0	7.45E-02	-0.9	5.92E-02	-0.1	1.0E+00	0.2	1.0E+00	-0.5	7.1E-01	-0.1	1.0E+00	0.0	9.7E-01	0.0	1.0E+00	0.0	9.7E-01	0.1	9.5E-01
40734	TRUB1	A_23_P63908	NM_139169	-0.7	7.23E-03	-0.7	3.89E-02	-1.4	5.82E-03	-0.7	7.23E-03	-0.6	1.34E-01	-0.5	2.62E-01	-0.6	5.38E-02	-1.2	1.34E-01	-0.1	1.0E+00	-0.3	1.0E+00	0.0	9.3E-01	-0.1	1.0E+00	0.0	9.8E-01	-0.1	1.0E+00	0.1	7.4E-01	0.0	9.8E-01
39676	LCORL	A_32_P54242	NM_153686	-0.7	4.31E-03	-0.7	4.31E-03	-1.0	6.16E-03	-0.7	7.25E-04	-0.4	2.87E-01	-0.2	5.26E-01	-0.6	2.87E-01	-0.4	1.21E-01	0.0	1.0E+00	0.0	1.0E+00	0.0	9.9E-01	0.0	1.0E+00	-0.2	3.7E-01	-0.2	1.0E+00	-0.2	3.7E-01	-0.5	2.7E-01
15497	FBXW4P1	A_23_P252603	AF174606	-0.7	2.92E-03	-0.7	1.87E-02	-1.0	2.92E-03	-0.7	4.62E-04	-0.5	3.93E-01	-0.5	3.35E-01	-0.7	4.15E-01	-0.4	3.93E-01	0.2	1.0E+00	0.1	1.0E+00	0.2	7.7E-01	0.2	1.0E+00	0.1	9.6E-01	-0.1	1.0E+00	0.2	7.4E-01	0.1	9.6E-01
2704	PLK1	A_24_P313504	NM_005030	-0.7	1.10E-03	-0.7	1.10E-03	-1.5	8.81E-04	-0.7	1.95E-03	-0.7	9.92E-03	-0.4	1.04E-01	-0.8	8.31E-03	-0.7	9.92E-03	-0.3	1.0E+00	-0.1	1.0E+00	-0.3	7.1E-01	-0.4	1.0E+00	-0.1	9.7E-01	-0.1	1.0E+00	-0.1	8.0E-01	0.0	9.7E-01
8415	MTBP	A_23_P357794	NM_022045	-0.7	3.57E-02	-0.7	2.56E-02	-1.1	3.57E-02	-0.7	4.46E-02	-0.2	6.20E-01	-0.1	8.85E-01	-0.2	6.20E-01	-0.2	5.59E-01	0.0	1.0E+00	0.0	1.0E+00	0.0	9.4E-01	-0.1	1.0E+00	-0.1	7.8E-01	0.0	1.0E+00	-0.1	7.8E-01	-0.3	7.8E-01
8721	TSPAN6	A_23_P171143	NM_003270	-0.7	1.10E-02	-0.7	1.14E-02	-1.1	1.10E-02	-0.7	2.29E-03	-0.3	5.79E-01	-0.2	7.53E-01	-0.6	5.06E-01	-0.3	5.79E-01	-0.1	1.0E+00	-0.2	1.0E+00	-0.1	8.6E-01	0.0	1.0E+00	-0.2	8.8E-01	-0.2	1.0E+00	-0.2	3.5E-01	-0.1	8.8E-01
42549	CCDC34	A_24_P195831	NM_030771	-0.7	5.37E-03	-0.7	2.86E-02	-1.3	5.37E-03	-0.7	2.94E-03	-0.5	3.37E-01	-0.4	3.32E-01	-0.5	5.65E-01	-0.5	3.37E-01	0.1	1.0E+00	0.0	1.0E+00	0.3	7.1E-01	0.1	1.0E+00	0.0	9.6E-01	-0.1	1.0E+00	0.1	8.2E-01	0.0	9.6E-01
38267	GLIPR1	A_24_P390096	NM_006851	-0.7	9.97E-04	-0.7	9.97E-04	-0.8	4.74E-03	-0.7	6.88E-04	-0.2	6.43E-01	-0.1	8.62E-01	-0.2	6.06E-01	-0.2	6.43E-01	-0.1	1.0E+00	-0.1	1.0E+00	-0.2	8.2E-01	0.3	1.0E+00	-0.1	7.6E-01	0.1	1.0E+00	-0.3	1.7E-01	-0.1	7.6E-01
3417	ZNF414	A_23_P340922	NM_032370	-0.7	3.31E-03	-0.6	3.78E-03	-1.0	3.31E-03	-0.7	4.60E-04	-0.3	5.32E-01	-0.2	7.09E-01	-0.5	4.77E-01	-0.3	5.32E-01	0.1	1.0E+00	0.1	1.0E+00	0.0	9.7E-01	0.1	1.0E+00	0.0	1.0E+00	-0.1	1.0E+00	0.0	1.0E+00	0.0	9.9E-01
44531	BC067077	A_24_P920922	BC067077	-0.7	8.77E-03	-0.7	1.05E-02	-1.0	8.77E-03	-0.7	2.70E-03	-0.5	4.82E-01	-0.5	4.82E-01	0.4	7.74E-01	-0.5	2.26E-01	-0.3	1.0E+00	0.0	1.0E+00	-0.5	7.1E-01	-0.3	1.0E+00	-0.3	7.2E-01	0.0	1.0E+00	-0.4	3.8E-01	-0.3	7.2E-01
32946	LOC100131149	A_32_P184509	XR_039101	-0.7	1.11E-03	-0.7	1.05E-03	-0.9	4.91E-03	-0.6	1.11E-03	-0.1	8.74E-01	-0.1	8.74E-01	-0.2	6.90E-01	0.0	9.66E-01	-0.1	1.0E+00	0.0	1.0E+00	-0.4	7.1E-01	-0.1	1.0E+00	-0.3	2.9E-01	-0.1	1.0E+00	-0.4	2.6E-02	-0.3	2.9E-01
38149	ARAF	A_23_P73511	NM_001654	-0.7	2.78E-03	-0.7	3.94E-02	-1.4	2.77E-03	-0.7	2.78E-03	-0.2	8.22E-01	-0.2	8.22E-01	-0.8	5.76E-01	-0.1	9.35E-01	0.1	1.0E+00	0.1	1.0E+00	0.0	9.9E-01	0.1	1.0E+00	-0.1	9.8E-01	-0.2	1.0E+00	0.0	9.8E-01	-0.1	9.7E-01
11478	VPS29	A_24_P928415	BC032462	-0.7	4.50E-03	-0.7	7.62E-03	-1.1	4.50E-03	-0.6	2.29E-03	-0.2	8.09E-01	-0.2	8.09E-01	-0.4	4.81E-01	-0.2	8.39E-01	0.1	1.0E+00	0.1	1.0E+00	-0.1	9.1E-01	0.1	1.0E+00	-0.1	9.3E-01	0.1	1.0E+00	-0.2	4.6E-01	-0.1	9.3E-01
3936	MCM9	A_23_P397347	NM_153255	-0.7	5.29E-03	-0.7	5.29E-03	-0.9	8.13E-03	-0.7	1.61E-03	-0.8	5.51E-02	-0.6	2.18E-01	-0.8	5.51E-02	-0.8	3.86E-02	0.0	1.0E+00	0.0	1.0E+00	-0.3	7.1E-01	0.0	1.0E+00	-0.1	7.4E-01	-0.1	1.0E+00	-0.1	7.4E-01	-0.2	7.4E-01
44782	HNRNPR	A_23_P45726	NM_005826	-0.7	7.41E-03	-0.7	1.00E-02	-1.8	1.45E-03	-0.7	7.41E-03	-0.9	3.58E-02	-0.7	3.58E-02	-1.4	5.20E-02	-0.9	6.14E-03	-0.2	1.0E+00	-0.1	1.0E+00	-0.2	7.1E-01	-0.2	1.0E+00	-0.2	7.4E-01	-0.3	1.0E+00	0.1	7.4E-01	-0.2	7.4E-01
28355	VPS13B	A_24_P98251	NM_017890	-0.7	1.80E-02	-0.7	2.65E-02	-1.4	1.22E-02	-0.7	1.80E-02	-0.6	1.38E-02	-0.1	8.03E-01	-0.9	1.38E-02	-0.6	6.26E-03	0.1	1.0E+00	0.1	1.0E+00	0.0	9.9E-01	0.2	1.0E+00	0.0	9.6E-01	0.0	1.0E+00	0.0	9.6E-01	-0.1	9.1E-01
37137	BF373107	A_32_P75115	BF373107	-0.7	9.59E-03	-0.7	7.04E-03	-1.0	1.58E-02	-0.6	9.59E-03	-0.3	3.92E-01	-0.3	3.92E-01	-0.5	2.58E-01	-0.2	6.57E-01	0.1	1.0E+00	0.2	1.0E+00	-0.2	7.6E-01	0.1	1.0E+00	-0.2	8.9E-01	0.2	1.0E+00	-0.3	5.1E-01	-0.2	8.9E-01
18441	QRSL1	A_23_P82181	NM_018292	-0.7	6.95E-03	-0.6	6.95E-03	-0.7	7.87E-03	-0.8	1.01E-04	-0.6	2.57E-02	-0.4	1.31E-01	-1.0	2.57E-02	-0.6	1.48E-02	-0.1	1.0E+00	-0.1	1.0E+00	-0.1	7.1E-01	-0.1	1.0E+00	0.1	8.6E-01	0.0	1.0E+00	0.2	4.2E-01	0.1	8.6E-01
3229	A_32_P2925	A_32_P2925	A_32_P2925	-0.7	3.52E-03	-0.7	3.52E-03	-0.7	1.29E-02	-0.6	1.38E-03	-0.3	6.61E-01	-0.3	6.61E-01	0.0	9.83E-01	-0.3	4.73E-01	0.0	1.0E+00	0.1	1.0E+00	-0.2	7.6E-01	0.0	1.0E+00	-0.2	6.8E-01	-0.2	1.0E+00	-0.2	6.8E-01	0.3	6.7E-01
31647	A_24_P290348	A_24_P290348	A_24_P290348	-0.7	3.42E-03	-0.6	3.42E-03	-0.7	9.57E-03	-1.1	8.03E-05	-0.4	5.56E-01	-0.2	8.58E-01	-0.4	5.56E-01	-0.4	3.51E-01	0.0	1.0E+00	0.0	1.0E+00	-0.3	7.1E-01	0.1	1.0E+00	-0.1	9.3E-01	0.2	1.0E+00	-0.2	5.4E-01	-0.1	9.3E-01
38711	ZNF273	A_23_P251962	BC019234	-0.7	2.36E-02	-0.7	1.80E-02	-1.0	2.36E-02	-0.6	4.25E-02	-0.1	8.48E-01	-0.1	8.71E-01	-0.1	8.48E-01	-0.5	1.43E-01	0.2	1.0E+00	-0.1	1.0E+00	0.2	7.9E-01	0.2	1.0E+00	-0.2	6.5E-01	0.0	1.0E+00	-0.2	6.5E-01	-0.3	6.4E-01
33394	CYC1	A_23_P20980	NM_001916	-0.7	7.71E-04	-0.6	2.18E-02	-1.6	7.71E-04	-0.7	6.76E-04	-0.2	5.92E-01	-0.2	6.64E-01	-1.0	3.98E-01	-0.2	5.92E-01	0.0	1.0E+00	0.0	1.0E+00	0.0	9.8E-01	0.0	1.0E+00	0.0	9.8E-01	0.0	1.0E+00	0.1	8.2E-01	0.0	9.8E-01
12614	SDCCAG1	A_23_P140328	NM_004713	-0.7	3.61E-02	-0.7	3.61E-02	-1.5	1.43E-02	-0.7	3.97E-02	-0.4	2.47E-01	-0.3	4.40E-01	-0.6	2.47E-01	-0.4	1.88E-01	0.0	1.0E+00	0.0	1.0E+00	0.0	9.9E-01	0.0	1.0E+00	0.0	9.7E-01	0.0	1.0E+00	0.1	9.0E-01	0.0	9.7E-01
793	THAP5	A_24_P23522	NM_182529	-0.7	7.29E-03	-0.7	7.53E-03	-1.0	7.29E-03	-0.7	6.95E-03	-0.3	5.53E-01	-0.3	7.07E-01	-0.3	5.53E-01	-0.4	3.43E-01	0.0	1.0E+00	0.0	1.0E+00	-0.4	7.1E-01	0.1	1.0E+00	-0.3	3.3E-01	-0.1	1.0E+00	-0.3	1.3E-01	-0.4	3.3E-01
36286	TMEM101	A_23_P15516	NM_032376	-0.7	2.33E-02	-0.7	2.85E-02	-0.7	2.33E-02	-1.1	6.94E-05	-0.9	5.75E-02	-0.5	1.09E-01	-1.0	5.75E-02	-0.9	2.20E-03	-0.3	1.0E+00	-0.3	1.0E+00	-0.6	5.3E-01	-0.2	1.0E+00	-0.1	9.1E-01	-0.1	1.0E+00	-0.2	5.4E-01	-0.1	9.1E-01
26348	ZNF248	A_23_P35343	NM_021045	-0.7	6.51E-03	-0.7	6.51E-03	-0.8	1.70E-02	-0.7	1.38E-03	-0.5	1.64E-01	-0.1	9.07E-01	-0.7	1.64E-01	-0.5	1.54E-01	-0.1	1.0E+00	-0.1	1.0E+00	-0.3	7.1E-01	0.1	1.0E+00	-0.1	9.7E-01	-0.1	1.0E+00	-0.2	6.2E-01	0.0	9.7E-01
15251	GNPDA2	A_32_P19608	AY173948	-0.7	2.77E-03	-0.6	1.27E-02	-2.1	9.06E-04	-0.7	2.77E-03	-0.4	3.08E-01	0.0	9.56E-01	-1.0	5.97E-02	-0.4	3.08E-01	0.0	1.0E+00	0.0	1.0E+00	-0.3	7.1E-01	0.0	1.0E+00	-0.2	6.5E-01	0.0	1.0E+00	-0.2	6.5E-01	-0.3	6.1E-01
7848	TBX19	A_23_P137705	NM_005149	-0.7	3.22E-03	-0.7	3.22E-03	-0.8	1.69E-02	-0.7	1.03E-03	-0.7	2.93E-01	-0.4	7.18E-01	-0.8	2.14E-01	-0.7	2.93E-01	0.2	1.0E+00	0.0	1.0E+00	0.3	7.1E-01	0.2	1.0E+00	0.1	9.5E-01	-0.1	1.0E+00	0.1	7.2E-01	0.1	9.5E-01
8004	ADH5	A_23_P18692	NM_000671	-0.7	1.28E-02	-0.7	1.72E-02	-1.9	2.78E-03	-0.6	1.28E-02	-0.7	1.49E-01	0.0	9.63E-01	-1.0	1.49E-01	-0.7	3.13E-02	0.0	1.0E+00	0.0	1.0E+00	-0.2	7.1E-01	0.0	1.0E+00	-0.1	9.8E-01	-0.1	1.0E+00	0.0	9.8E-01	-0.2	7.8E-01
25858	MRPL3	A_23_P18422	NM_007208	-0.7	1.46E-03	-0.6	4.34E-02	-1.9	8.85E-04	-0.7	1.46E-03	-0.5	1.40E-01	-0.3	3.17E-01	-0.9	1.40E-01	-0.5	3.90E-02	0.0	1.0E+00	0.0	1.0E+00	0.1	8.0E-01	0.0	1.0E+00	0.1	9.5E-01	0.1	1.0E+00	0.2	3.0E-01	0.0	9.5E-01
30265	CTNNB1	A_23_P29499	NM_001904	-0.7	3.54E-02	-0.7	3.54E-02	-0.7	4.68E-02	-0.7	3.02E-03	-0.7	8.33E-02	-0.2	8.46E-01	-0.7	8.33E-02	-0.7	6.11E-02	-0.1	1.0E+00	0.1	1.0E+00	-0.2	8.4E-01	-0.1	1.0E+00	-0.1	9.7E-01	-0.1	1.0E+00	-0.2	8.2E-01	0.1	9.7E-01
30078	NOC2L	A_24_P917951	BC009786	-0.7	3.56E-03	-0.6	9.63E-03	-1.2	3.56E-03	-0.7	1.40E-03	-0.7	1.10E-01	-0.7	1.10E-01	-0.7	2.95E-01	-0.6	1.01E-01	0.0	1.0E+00	0.0	1.0E+00	0.1	8.2E-01	-0.1	1.0E+00	0.7	6.3E-01	0.9	1.0E+00	0.4	6.3E-01	0.7	5.5E-01
27251	WDR42A	A_24_P76725	BC111063	-0.7	1.01E-03	-0.8	6.01E-04	-0.7	9.26E-03	-0.6	1.01E-03	-0.5	3.01E-01	-0.4	4.29E-01	-0.6	3.01E-01	-0.5	1.52E-01	-0.1	1.0E+00	-0.1	1.0E+00	-0.1	8.3E-01	0.0	1.0E+00	-0.2	7.7E-01	0.0	1.0E+00	-0.2	6.4E-01	-0.2	7.7E-01
27211	A_24_P324506	A_24_P324506	A_24_P324506	-0.7	1.02E-02	-0.7	6.14E-03	-1.0	1.02E-02	-0.6	1.43E-02	-0.2	6.43E-01	-0.1	9.12E-01	-0.9	2.15E-01	-0.2	6.43E-01	0.1	1.0E+00	0.1	1.0E+00	-0.1	8.9E-01	0.1	1.0E+00	-0.1	9.6E-01	0.1	1.0E+00	-0.1	9.3E-01	-0.1	9.6E-01
1968	KIRREL	A_24_P393312	AK090554	-0.7	8.35E-03	-0.7	8.35E-03	-1.0	1.12E-02	-0.7	2.46E-03	-0.6	5.30E-02	-0.4	2.23E-01	-0.6	5.30E-02	-0.7	1.25E-02	-0.3	1.0E+00	0.0	1.0E+00	-0.6	7.1E-01	-0.3	1.0E+00	-0.3	3.9E-01	-0.3	1.0E+00	-0.3	2.2E-01	-0.3	3.9E-01
15399	UCRC	A_24_P66001	NM_001003684	-0.7	1.47E-02	-1.4	1.47E-02	-0.7	2.41E-02	-0.6	7.09E-03	-0.7	1.53E-01	-1.5	1.37E-03	-0.6	3.81E-01	-0.7	1.53E-01	0.0	9.9E-01	-0.7	6.7E-02	0.0	9.9E-01	0.1	1.0E+00	-0.1	9.7E-01	-0.2	1.0E+00	-0.1	9.4E-01	0.1	9.7E-01
13950	CR610374	A_32_P91042	CR610374	-0.7	1.91E-02	-0.6	2.13E-02	-0.9	1.91E-02	-0.7	3.27E-03	-0.3	3.41E-01	-0.1	8.58E-01	-0.6	2.29E-01	-0.3	3.41E-01	0.0	1.0E+00	0.0	1.0E+00	-0.4	7.1E-01	0.0	1.0E+00	-0.2	8.1E-01	-0.1	1.0E+00	-0.3	2.5E-01	-0.2	8.1E-01
40895	FUSIP1	A_32_P45493	NM_006625	-0.7	1.52E-03	-0.7	1.91E-02	-1.4	1.50E-03	-0.7	1.52E-03	-0.5	1.45E-01	-0.5	1.45E-01	-0.4	2.03E-01	-0.5	4.42E-02	-0.1	1.0E+00	0.0	1.0E+00	-0.1	9.0E-01	-0.1	1.0E+00	0.1	8.9E-01	0.1	1.0E+00	0.2	5.8E-01	0.1	8.9E-01
22839	CCDC76	A_23_P51317	NM_019083	-0.7	7.57E-03	-0.7	1.25E-02	-1.2	7.52E-03	-0.6	7.57E-03	-0.6	1.05E-01	-0.2	7.60E-01	-0.6	1.05E-01	-0.6	8.96E-02	0.0	1.0E+00	-0.1	1.0E+00	0.0	9.4E-01	0.1	1.0E+00	0.0	1.0E+00	-0.2	1.0E+00	0.2	5.8E-01	0.0	1.0E+00
43785	A_23_P120606	A_23_P120606	A_23_P120606	-0.7	2.08E-03	-0.8	2.08E-03	-0.6	4.99E-02	-0.7	1.54E-03	-0.9	1.09E-02	-0.8	2.84E-02	-0.9	1.09E-02	-1.0	4.82E-03	-0.1	1.0E+00	0.0	1.0E+00	-0.1	9.2E-01	-0.2	1.0E+00	0.4	5.6E-01	0.5	1.0E+00	0.3	5.0E-01	0.4	5.6E-01
156	C5orf24	A_23_P433829	NM_152409	-0.7	5.21E-03	-0.7	5.21E-03	-1.6	1.21E-03	-0.6	6.90E-03	-0.5	1.43E-01	-0.4	2.49E-01	-1.0	1.01E-01	-0.5	1.43E-01	-0.2	1.0E+00	-0.2	1.0E+00	-0.4	2.8E-01	-0.2	1.0E+00	-0.1	8.8E-01	0.0	1.0E+00	-0.1	8.8E-01	-0.1	8.8E-01
24910	AKT2	A_23_P373475	NM_001626	-0.7	8.45E-04	-0.7	4.73E-02	-1.5	4.20E-04	-0.6	8.45E-04	-0.1	9.16E-01	-0.1	9.16E-01	-0.6	5.67E-01	0.1	9.44E-01	0.2	1.0E+00	0.2	1.0E+00	0.1	9.4E-01	0.3	1.0E+00	0.1	9.8E-01	0.1	1.0E+00	0.0	9.8E-01	0.2	8.4E-01
10442	MCTS1	A_24_P808100	NM_014060	-0.7	1.18E-03	-0.7	1.18E-03	-0.8	6.84E-03	-0.7	2.07E-04	-0.4	3.79E-01	-0.2	7.86E-01	-0.4	3.69E-01	-0.4	3.79E-01	0.0	1.0E+00	0.0	1.0E+00	-0.2	7.7E-01	0.1	1.0E+00	-0.1	9.0E-01	0.0	1.0E+00	-0.1	8.0E-01	-0.1	9.0E-01
21503	THC2730719	A_32_P42518	THC2730719	-0.7	4.26E-03	-0.7	4.26E-03	-0.8	6.74E-03	-0.6	7.38E-04	-0.4	1.33E-01	0.0	9.61E-01	-0.8	1.33E-01	-0.4	1.11E-01	0.0	1.0E+00	0.0	1.0E+00	-0.3	7.1E-01	0.1	1.0E+00	-0.1	7.7E-01	-0.1	1.0E+00	-0.1	7.7E-01	-0.3	6.8E-01
2765	THC2631150	A_32_P181826	THC2631150	-0.7	3.90E-03	-0.7	3.90E-03	-0.8	1.40E-02	-0.7	1.89E-03	-0.3	6.35E-01	-0.3	6.35E-01	-0.2	6.45E-01	-0.4	2.92E-01	0.0	1.0E+00	0.1	1.0E+00	-0.4	7.1E-01	0.0	1.0E+00	-0.2	8.0E-01	-0.1	1.0E+00	-0.2	4.5E-01	-0.2	8.0E-01
44019	L48692	A_23_P102183	L48692	-0.7	1.44E-02	-0.7	1.51E-02	-1.7	2.83E-03	-0.6	1.44E-02	-0.6	4.90E-02	-0.4	5.29E-01	-1.1	9.78E-03	-0.6	4.90E-02	0.0	1.0E+00	0.0	1.0E+00	-0.1	8.6E-01	0.2	1.0E+00	-0.1	9.1E-01	-0.1	1.0E+00	-0.1	9.1E-01	-0.3	8.8E-01
15811	PPP6C	A_24_P81298	NM_001123355	-0.7	2.89E-02	-0.6	4.63E-02	-0.9	2.89E-02	-0.7	9.36E-03	-0.3	5.60E-01	-0.1	8.37E-01	-0.3	5.60E-01	-0.5	2.46E-01	0.0	1.0E+00	0.0	1.0E+00	-0.3	7.3E-01	0.1	1.0E+00	-0.2	6.6E-01	0.0	1.0E+00	-0.2	4.3E-01	-0.3	6.6E-01
31629	RABL3	A_32_P109057	NM_173825	-0.7	2.42E-03	-0.6	2.31E-02	-1.2	2.42E-03	-0.7	8.70E-04	-0.5	3.48E-01	-0.5	3.48E-01	0.0	9.66E-01	-0.5	1.97E-01	0.0	1.0E+00	0.0	1.0E+00	-0.1	8.2E-01	0.0	1.0E+00	0.0	9.9E-01	0.1	1.0E+00	-0.1	8.8E-01	0.0	9.9E-01
33043	OSTCL	A_24_P317450	NM_145303	-0.7	4.45E-03	-0.7	5.31E-03	-1.2	4.45E-03	-0.6	2.93E-03	-0.3	5.85E-01	-0.3	6.94E-01	-0.5	2.87E-01	-0.3	5.85E-01	0.1	1.0E+00	0.1	1.0E+00	-0.2	7.8E-01	0.1	1.0E+00	-0.3	2.3E-01	0.1	1.0E+00	-0.3	1.2E-01	-0.4	2.3E-01
31824	THC2534106	A_32_P96043	THC2534106	-0.7	1.04E-02	-0.7	1.21E-02	-1.4	5.97E-03	-0.6	1.04E-02	-0.4	2.24E-01	0.1	8.51E-01	-0.8	7.75E-02	-0.4	2.24E-01	-0.1	1.0E+00	-0.1	1.0E+00	-0.2	7.1E-01	0.2	1.0E+00	0.0	9.7E-01	0.1	1.0E+00	-0.2	6.0E-01	0.0	9.7E-01
7086	HOXB3	A_23_P316511	NM_002146	-0.7	9.31E-03	-0.6	3.07E-02	-1.1	9.31E-03	-0.7	5.30E-03	-0.5	5.26E-01	-0.5	5.26E-01	-0.3	7.30E-01	-0.5	3.73E-01	0.0	1.0E+00	0.1	1.0E+00	-0.3	7.3E-01	0.0	1.0E+00	-0.2	8.6E-01	0.0	1.0E+00	-0.2	7.2E-01	-0.2	8.6E-01
9126	MBOAT2	A_32_P119096	AF052107	-0.7	3.07E-02	-0.7	3.08E-02	-1.1	3.07E-02	-0.7	1.87E-02	-0.7	2.91E-01	-0.4	6.58E-01	-0.7	2.91E-01	-0.7	1.60E-01	0.0	1.0E+00	0.0	1.0E+00	0.0	9.7E-01	0.1	1.0E+00	0.0	9.7E-01	0.0	1.0E+00	0.0	9.7E-01	-0.1	9.4E-01
44022	NEDD9	A_23_P344555	NM_006403	-0.7	5.31E-03	-0.7	5.31E-03	-1.0	1.13E-02	-0.7	4.41E-03	-0.4	3.59E-01	-0.3	5.50E-01	-0.4	3.59E-01	-0.5	9.84E-02	-0.1	1.0E+00	-0.1	1.0E+00	-0.4	7.1E-01	-0.1	1.0E+00	-0.2	5.7E-01	-0.1	1.0E+00	-0.2	5.0E-01	-0.3	5.7E-01
21403	MRRF	A_24_P175427	NM_199177	-0.7	1.71E-03	-0.7	6.84E-03	-1.3	1.71E-03	-0.7	1.30E-03	-0.2	6.58E-01	0.0	9.54E-01	-0.5	4.58E-01	-0.2	6.58E-01	-0.1	1.0E+00	-0.1	1.0E+00	-0.2	7.1E-01	0.1	1.0E+00	-0.1	9.6E-01	-0.1	1.0E+00	0.1	8.8E-01	-0.1	9.6E-01
41637	DERA	A_23_P25253	NM_015954	-0.7	1.51E-02	-0.7	2.77E-02	-1.5	1.08E-02	-0.7	1.51E-02	-0.5	4.83E-01	-0.1	7.77E-01	-0.5	4.83E-01	-0.6	3.26E-02	0.2	1.0E+00	0.1	1.0E+00	0.2	7.1E-01	0.2	1.0E+00	-0.2	5.7E-01	-0.1	1.0E+00	-0.2	5.7E-01	-0.4	4.1E-01
9832	ESPN	A_23_P12405	NM_031475	-0.7	9.21E-03	-0.7	9.21E-03	-1.0	4.34E-03	-0.6	1.17E-02	-0.1	9.04E-01	-0.2	4.88E-01	-0.1	9.04E-01	0.0	9.49E-01	-0.1	1.0E+00	-0.1	1.0E+00	0.1	8.6E-01	-0.1	1.0E+00	0.0	9.7E-01	0.0	1.0E+00	0.0	9.7E-01	0.2	8.6E-01
30467	ZNF649	A_23_P208208	NM_023074	-0.7	1.35E-03	-0.7	1.35E-03	-0.8	7.70E-03	-0.7	2.76E-04	-0.2	5.08E-01	-0.2	7.30E-01	-0.2	5.08E-01	-0.5	1.91E-01	0.0	1.0E+00	0.0	1.0E+00	-0.1	8.0E-01	0.0	1.0E+00	-0.1	8.7E-01	0.0	1.0E+00	-0.1	8.7E-01	-0.2	7.7E-01
13325	RBM15B	A_24_P945181	NM_013286	-0.7	1.04E-02	-0.7	1.04E-02	-0.7	1.31E-02	-0.6	1.68E-03	-0.3	5.08E-01	-0.3	5.94E-01	-0.4	3.33E-01	-0.3	5.08E-01	0.0	1.0E+00	0.0	1.0E+00	-0.1	9.2E-01	0.1	1.0E+00	-0.1	9.2E-01	-0.1	1.0E+00	-0.2	7.2E-01	-0.1	9.2E-01
8330	TMEM135	A_24_P181101	NM_022918	-0.7	2.49E-02	-0.7	1.64E-02	-0.9	4.92E-02	-0.6	2.49E-02	-0.3	5.14E-01	-0.1	7.86E-01	-0.5	5.14E-01	-0.3	3.19E-01	-0.1	1.0E+00	-0.1	1.0E+00	-0.1	8.8E-01	0.1	1.0E+00	-0.1	7.6E-01	-0.1	1.0E+00	-0.1	7.6E-01	-0.2	6.6E-01
11741	LEAP2	A_24_P268993	NM_052971	-0.7	5.66E-03	-0.7	5.66E-03	-1.0	8.85E-03	-0.7	3.18E-03	-0.5	1.66E-01	-0.4	4.54E-01	-0.5	1.66E-01	-0.8	5.53E-02	-0.1	1.0E+00	-0.1	1.0E+00	-0.1	8.0E-01	0.1	1.0E+00	0.1	9.9E-01	0.1	1.0E+00	0.1	8.3E-01	0.0	9.9E-01
39574	FBXO4	A_23_P58815	NM_012176	-0.7	3.17E-02	-0.7	3.17E-02	-1.0	3.65E-02	-0.7	2.09E-02	-0.1	8.16E-01	0.2	7.49E-01	-0.2	8.16E-01	-0.1	8.78E-01	-0.1	1.0E+00	-0.1	1.0E+00	-0.2	8.8E-01	0.3	1.0E+00	-0.3	7.3E-01	0.0	1.0E+00	-0.3	6.5E-01	-0.4	7.3E-01
21512	CHRM3	A_23_P401472	NM_000740	-0.7	2.05E-04	-0.7	2.61E-02	-1.9	2.05E-04	-0.7	2.05E-04	-0.3	5.81E-01	-0.3	5.81E-01	-0.8	1.02E-01	-0.2	7.14E-01	-0.1	1.0E+00	-0.1	1.0E+00	-0.4	7.1E-01	0.2	1.0E+00	-0.1	7.3E-01	-0.1	1.0E+00	-0.2	4.2E-01	0.1	7.3E-01
39261	ALS2CR4	A_24_P941831	NM_001044385	-0.7	8.18E-03	-0.6	4.02E-02	-1.5	5.43E-03	-0.7	8.18E-03	0.0	7.68E-01	0.2	7.68E-01	-0.6	3.48E-01	0.0	9.77E-01	-0.1	1.0E+00	-0.1	1.0E+00	-0.2	7.6E-01	0.2	1.0E+00	-0.1	8.5E-01	0.0	1.0E+00	-0.1	8.5E-01	-0.3	5.8E-01
28213	ENST00000326140	A_24_P341222	ENST00000326140	-0.7	2.08E-03	-0.7	4.36E-03	-1.7	9.66E-04	-0.7	2.08E-03	-0.6	6.40E-02	-0.3	2.99E-01	-1.4	1.14E-03	-0.6	6.40E-02	-0.1	1.0E+00	-0.1	1.0E+00	-0.5	5.2E-01	-0.1	1.0E+00	-0.1	9.5E-01	0.0	1.0E+00	-0.1	8.6E-01	-0.1	9.5E-01
17927	ATAD1	A_23_P75288	NM_032810	-0.7	1.57E-02	-0.7	3.40E-02	-2.0	2.52E-03	-0.7	1.57E-02	-0.5	1.46E-01	-0.5	2.20E-01	-0.6	6.73E-02	-0.5	1.46E-01	-0.1	1.0E+00	0.0	1.0E+00	-0.2	7.1E-01	-0.1	1.0E+00	0.1	9.9E-01	0.1	1.0E+00	0.1	7.0E-01	0.0	9.9E-01
11054	EIF2B3	A_23_P115046	NM_020365	-0.7	4.20E-04	-0.6	6.19E-03	-1.6	4.20E-04	-0.7	3.52E-04	-0.3	4.07E-01	-0.3	4.07E-01	-0.7	1.01E-01	-0.2	6.92E-01	0.0	1.0E+00	0.0	1.0E+00	-0.2	7.1E-01	0.0	1.0E+00	0.1	9.9E-01	0.2	1.0E+00	0.1	8.9E-01	0.0	9.9E-01
44813	PAIP1	A_23_P207811	NM_006451	-0.7	2.63E-02	-0.7	2.63E-02	-1.1	1.90E-02	-0.6	2.81E-02	-0.7	1.24E-01	-0.4	5.12E-01	-0.7	1.24E-01	-0.7	6.82E-02	0.0	1.0E+00	0.0	1.0E+00	0.1	8.5E-01	0.0	1.0E+00	0.0	9.6E-01	0.0	1.0E+00	0.0	9.5E-01	-0.1	9.6E-01
33084	CHRM2	A_23_P145606	NM_001006630	-0.7	1.97E-03	-0.7	1.24E-02	-1.5	1.73E-03	-0.6	1.97E-03	-0.4	5.12E-01	-0.4	5.12E-01	-0.7	1.26E-01	-0.1	8.83E-01	-0.1	1.0E+00	-0.1	1.0E+00	-0.3	7.1E-01	0.1	1.0E+00	-0.1	7.1E-01	-0.1	1.0E+00	-0.1	6.3E-01	0.2	7.1E-01
3138	ABCA17P	A_32_P29703	BC042853	-0.7	5.19E-03	-0.6	1.56E-02	-1.2	5.19E-03	-0.7	3.22E-03	-0.4	2.19E-01	-0.2	4.94E-01	-0.4	2.19E-01	-0.4	9.92E-02	-0.1	1.0E+00	-0.1	1.0E+00	-0.2	7.1E-01	-0.1	1.0E+00	0.0	9.9E-01	0.2	1.0E+00	-0.1	7.0E-01	0.0	9.9E-01
22698	THC2606487	A_32_P24656	THC2606487	-0.7	1.48E-02	-0.7	1.48E-02	-1.1	7.11E-03	-0.6	1.59E-02	-0.4	5.67E-01	-0.2	8.49E-01	-0.4	5.67E-01	-0.4	3.55E-01	0.0	1.0E+00	0.0	1.0E+00	-0.4	7.1E-01	0.0	1.0E+00	0.0	9.9E-01	0.0	1.0E+00	0.0	9.9E-01	0.0	9.9E-01
38357	GNPDA2	A_23_P144578	NM_138335	-0.7	8.48E-03	-0.7	8.48E-03	-2.2	9.66E-04	-0.6	1.08E-02	-0.2	5.25E-01	0.0	9.75E-01	-1.3	1.50E-01	-0.2	5.25E-01	0.0	1.0E+00	0.0	1.0E+00	-0.4	7.1E-01	0.0	1.0E+00	-0.4	6.7E-01	-0.1	1.0E+00	-0.4	3.3E-01	-0.4	6.7E-01
35100	SMCR7L	A_23_P109547	NM_019008	-0.7	6.50E-03	-0.6	6.50E-03	-0.7	2.39E-02	-0.7	1.63E-03	-0.4	3.59E-01	-0.4	2.77E-01	-0.5	4.69E-01	-0.3	3.59E-01	-0.1	1.0E+00	-0.1	1.0E+00	-0.2	7.1E-01	-0.1	1.0E+00	-0.1	9.9E-01	-0.1	1.0E+00	-0.1	8.5E-01	0.0	9.9E-01
3501	RPS6KC1	A_23_P104109	NM_012424	-0.7	1.79E-02	-0.6	1.79E-02	-0.7	2.03E-02	-0.7	1.16E-03	-0.6	1.06E-01	-0.6	1.06E-01	0.0	9.92E-01	-0.7	2.84E-02	-0.2	1.0E+00	-0.2	1.0E+00	-0.2	7.4E-01	-0.2	1.0E+00	-0.2	7.2E-01	-0.2	1.0E+00	-0.2	3.8E-01	-0.2	7.2E-01
38123	LRCH3	A_23_P435904	AL137527	-0.7	2.83E-03	-1.1	6.39E-04	-0.7	3.24E-02	-0.7	2.83E-03	-0.4	5.02E-01	-0.4	5.02E-01	-0.5	5.35E-01	-0.4	4.03E-01	-0.1	1.0E+00	-0.2	1.0E+00	-0.1	9.1E-01	0.1	1.0E+00	-0.4	7.0E-01	-0.2	1.0E+00	-0.4	4.9E-01	-0.4	7.0E-01
786	KSR2	A_32_P216635	NM_173598	-0.7	9.19E-03	-0.6	9.19E-03	-0.8	2.20E-02	-0.7	1.67E-03	-0.1	7.80E-01	0.0	9.93E-01	-0.2	6.33E-01	-0.1	7.80E-01	0.0	1.0E+00	0.0	1.0E+00	-0.3	7.1E-01	0.1	1.0E+00	-0.3	2.1E-01	-0.3	1.0E+00	-0.3	1.0E-01	-0.4	2.1E-01
8295	BC032585	A_24_P222432	BC032585	-0.7	3.50E-03	-0.8	1.62E-03	-0.7	2.05E-02	-0.7	3.50E-03	-0.3	5.75E-01	-0.2	5.75E-01	-0.3	6.63E-01	-0.3	4.47E-01	-0.1	1.0E+00	-0.1	1.0E+00	-0.2	7.3E-01	0.0	1.0E+00	-0.2	4.0E-01	-0.1	1.0E+00	-0.3	1.7E-01	-0.2	4.0E-01
44250	CNDP2	A_23_P89812	NM_018235	-0.7	1.28E-02	-0.6	1.28E-02	-0.7	4.05E-02	-1.1	1.82E-04	-0.6	3.71E-01	-0.6	3.71E-01	-0.5	3.85E-01	-0.7	1.50E-01	0.0	1.0E+00	0.0	1.0E+00	0.0	9.8E-01	-0.1	1.0E+00	0.1	8.9E-01	0.0	1.0E+00	0.1	7.3E-01	0.1	8.9E-01
38257	CCDC58	A_32_P215113	NM_001017928	-0.7	1.49E-02	-1.0	1.49E-02	-0.7	2.66E-02	-0.6	2.00E-03	-1.0	1.91E-02	-1.2	1.91E-02	-1.0	1.51E-02	-0.6	1.11E-01	-0.2	1.0E+00	-0.2	1.0E+00	-0.1	9.4E-01	-0.2	1.0E+00	0.1	9.0E-01	-0.1	1.0E+00	0.1	9.0E-01	0.1	9.0E-01
23561	TERF1	A_24_P192434	NM_017489	-0.7	1.49E-02	-0.7	1.49E-02	-1.1	1.42E-02	-0.7	2.04E-02	-0.5	1.67E-01	0.0	9.84E-01	-0.5	1.60E-01	-0.5	1.67E-01	-0.1	1.0E+00	-0.1	1.0E+00	-0.1	8.2E-01	-0.1	1.0E+00	0.0	9.9E-01	0.1	1.0E+00	0.0	9.9E-01	-0.1	9.0E-01

Feature	Gene symbol	Probe	Transcript	72 hour								48 hours								24 hours								10 hours							
				siCASP8AP2								siCASP8AP2								siCASP8AP2								siCASP8AP2							
				Median	Median	1	1	3	3	6	6	Median	Median	1	1	3	3	6	6	Median	Median	1	1	3	3	6	6	Median	Median	1	1	3	3	6	6
				M	q	M	q	M	q	M	q	M	q	M	q	M	q	M	q	M	q	M	q	M	q	M	q	M	q	M	q	M	q	M	q
16206	AGGF1	A_23_P250554	NM_018046	-0.7	1.36E-02	-0.6	3.71E-02	-1.4	1.25E-02	-0.7	1.36E-02	-0.5	3.88E-01	0.1	9.15E-01	-0.8	3.88E-01	-0.5	7.44E-02	0.0	1.0E+00	0.1	1.0E+00	0.0	9.1E-01	0.0	1.0E+00	0.0	9.4E-01	0.0	1.0E+00	0.0	9.4E-01	-0.1	8.9E-01
8406	KRT40	A_24_P280333	NM_182497	-0.7	1.57E-02	-0.6	1.72E-02	-1.0	1.57E-02	-0.7	9.66E-03	-0.3	3.67E-01	-0.1	8.88E-01	-0.7	1.07E-01	-0.3	3.67E-01	-0.2	1.0E+00	-0.2	1.0E+00	-0.4	7.1E-01	0.1	1.0E+00	-0.2	3.5E-01	-0.1	1.0E+00	-0.2	2.3E-01	-0.2	3.5E-01
13099	HNRNPU	A_32_P51894	NM_031844	-0.7	1.11E-02	-0.6	3.60E-02	-2.0	2.30E-03	-0.7	1.11E-02	-0.4	2.37E-01	-0.2	6.83E-01	-1.0	4.03E-02	-0.4	2.37E-01	0.0	1.0E+00	0.0	1.0E+00	-0.1	9.0E-01	0.1	1.0E+00	0.0	9.7E-01	0.0	1.0E+00	0.0	9.7E-01	-0.2	8.2E-01
41951	PWP1	A_23_P105753	CR621244	-0.7	7.78E-03	-0.6	1.05E-02	-1.1	6.45E-03	-0.7	7.78E-03	-0.4	2.83E-01	-0.1	7.95E-01	-0.4	8.71E-02	-0.4	2.83E-01	-0.1	1.0E+00	-0.1	1.0E+00	-0.1	9.0E-01	0.0	1.0E+00	0.0	9.9E-01	0.0	1.0E+00	0.0	9.9E-01	0.0	9.5E-01
25755	KGFLP1	A_32_P11372	NR_003674	-0.7	9.21E-03	-0.7	9.21E-03	-0.9	9.69E-03	-0.6	2.70E-03	-0.4	2.10E-01	-0.4	2.10E-01	-0.2	5.29E-01	-0.4	4.17E-02	0.0	1.0E+00	-0.2	1.0E+00	0.0	9.1E-01	0.0	1.0E+00	-0.2	7.5E-01	0.1	1.0E+00	-0.2	6.7E-01	-0.3	7.5E-01
35980	GINS2	A_23_P118246	NM_016095	-0.7	3.58E-03	-0.6	1.37E-02	-0.9	3.58E-03	-0.7	7.01E-04	-0.2	5.26E-01	-0.2	5.26E-01	-0.1	5.72E-01	-0.4	8.88E-02	-0.1	1.0E+00	-0.1	1.0E+00	-0.1	7.6E-01	-0.2	1.0E+00	0.1	8.9E-01	-0.1	1.0E+00	0.1	8.5E-01	0.1	8.9E-01
13497	THC2491396	A_24_P270452	THC2491396	-0.7	7.45E-03	-0.7	7.45E-03	-0.9	1.67E-02	-0.6	6.99E-03	-0.2	6.99E-01	-0.1	9.04E-01	-0.4	4.31E-01	-0.2	6.99E-01	-0.1	1.0E+00	-0.1	1.0E+00	-0.1	8.6E-01	0.1	1.0E+00	-0.2	6.5E-01	-0.2	1.0E+00	-0.1	6.5E-01	-0.2	5.4E-01
38541	DBF4	A_23_P254612	NM_006716	-0.7	1.40E-02	-0.6	1.40E-02	-1.4	3.36E-03	-0.7	1.65E-02	-0.6	2.37E-02	-0.6	7.10E-02	-1.3	7.33E-03	-0.6	2.37E-02	-0.4	1.0E+00	-0.2	1.0E+00	-0.5	4.0E-01	-0.4	1.0E+00	-0.2	8.2E-01	-0.2	1.0E+00	-0.1	7.0E-01	-0.2	8.2E-01
37638	A_24_P534290	A_24_P534290	A_24_P534290	-0.7	5.79E-03	-0.6	5.79E-03	-0.7	1.72E-02	-0.9	3.23E-03	-0.4	4.18E-01	-0.1	8.53E-01	-0.6	2.31E-01	-0.4	4.18E-01	0.0	1.0E+00	0.0	1.0E+00	-0.3	7.1E-01	0.0	1.0E+00	-0.1	7.4E-01	0.0	1.0E+00	-0.2	3.8E-01	-0.1	7.4E-01
8686	JMJD2C	A_24_P93754	AB018323	-0.7	6.26E-03	-0.7	6.26E-03	-1.0	8.96E-03	-0.7	1.86E-03	-0.6	8.93E-02	-0.3	5.79E-01	-0.7	8.42E-02	-0.6	8.93E-02	-0.1	1.0E+00	-0.1	1.0E+00	-0.3	7.1E-01	0.1	1.0E+00	0.0	9.6E-01	0.1	1.0E+00	0.0	9.6E-01	-0.2	8.1E-01
30133	AI571129	A_32_P118522	AI571129	-0.7	1.32E-02	-0.7	2.62E-02	-1.2	1.32E-02	-0.7	1.23E-02	-0.4	5.22E-01	0.1	8.70E-01	-0.7	2.54E-01	-0.4	5.22E-01	0.1	1.0E+00	0.1	1.0E+00	-0.2	8.0E-01	0.2	1.0E+00	0.1	9.9E-01	0.3	1.0E+00	0.1	8.3E-01	0.0	9.9E-01
6156	CA5B	A_23_P307346	NM_007220	-0.7	5.57E-03	-0.7	5.53E-03	-0.9	1.39E-02	-0.6	5.57E-03	-0.3	5.12E-01	-0.3	5.80E-01	-0.3	5.12E-01	-0.6	1.20E-01	-0.1	1.0E+00	0.0	1.0E+00	-0.3	7.1E-01	-0.1	1.0E+00	-0.1	8.5E-01	-0.1	1.0E+00	-0.1	8.5E-01	-0.3	5.8E-01
39227	TUSC1	A_32_P133884	NM_001004125	-0.7	2.30E-02	-0.7	2.30E-02	-1.2	1.85E-02	-0.6	4.96E-02	0.2	8.53E-01	0.2	8.53E-01	0.3	6.92E-01	-0.1	9.43E-01	-0.1	1.0E+00	-0.1	1.0E+00	-0.3	7.1E-01	0.0	1.0E+00	-0.3	6.1E-01	-0.1	1.0E+00	-0.3	2.9E-01	-0.3	6.1E-01
10612	NNT	A_23_P70148	NM_182977	-0.7	1.45E-02	-0.7	1.67E-02	-0.9	1.45E-02	-0.7	3.76E-03	-0.1	6.87E-01	-0.1	7.41E-01	0.1	6.87E-01	-0.5	3.24E-02	-0.2	1.0E+00	0.0	1.0E+00	-0.2	7.1E-01	-0.2	1.0E+00	0.0	9.9E-01	0.0	1.0E+00	0.0	9.9E-01	-0.1	9.3E-01
4293	THC2691928	A_32_P167103	THC2691928	-0.7	5.70E-03	-0.6	5.70E-03	-0.8	1.88E-02	-0.7	1.86E-03	-0.1	7.52E-01	-0.1	7.52E-01	-0.1	8.76E-01	-0.3	2.42E-01	-0.1	1.0E+00	-0.1	1.0E+00	-0.1	7.9E-01	0.1	1.0E+00	-0.1	8.8E-01	0.0	1.0E+00	-0.2	5.7E-01	-0.1	8.8E-01
41236	LOC202051	A_24_P220897	NM_194296	-0.7	3.43E-02	-0.6	3.43E-02	-0.8	3.92E-02	-0.7	1.12E-02	-0.1	8.22E-01	0.2	8.22E-01	-0.1	9.03E-01	-0.2	7.18E-01	0.0	1.0E+00	0.0	1.0E+00	-0.3	7.6E-01	0.0	1.0E+00	-0.3	5.1E-01	-0.1	1.0E+00	-0.4	1.1E-01	-0.3	5.1E-01
6303	ZNF564	A_24_P14328	NM_144976	-0.7	2.24E-02	-0.6	2.24E-02	-0.8	3.22E-02	-0.7	3.35E-03	-0.3	7.24E-01	-0.1	9.36E-01	-0.3	7.24E-01	-0.3	5.97E-01	0.0	1.0E+00	0.0	1.0E+00	-0.2	7.7E-01	0.1	1.0E+00	-0.3	6.8E-01	-0.2	1.0E+00	-0.3	4.6E-01	-0.3	6.8E-01
6997	MTDH	A_23_P250196	NM_178812	-0.7	5.08E-03	-0.6	2.07E-02	-2.1	8.42E-04	-0.7	5.08E-03	-0.4	1.98E-01	-0.4	1.35E-01	-1.1	1.98E-01	-0.3	2.19E-01	0.1	1.0E+00	0.1	1.0E+00	0.1	8.6E-01	0.1	1.0E+00	0.0	9.7E-01	0.0	1.0E+00	0.0	9.7E-01	-0.3	6.4E-01
9091	SENP7	A_24_P265856	NM_020654	-0.7	3.44E-02	-0.7	4.42E-02	-1.3	1.73E-02	-0.6	3.44E-02	-0.5	1.47E-01	-0.4	1.47E-01	-0.8	2.19E-01	-0.5	3.92E-02	0.0	1.0E+00	0.0	1.0E+00	0.0	1.0E+00	0.0	1.0E+00	-0.1	9.2E-01	0.0	1.0E+00	-0.1	9.2E-01	-0.2	8.8E-01
13469	TET1	A_23_P322756	NM_030625	-0.7	3.50E-02	-0.7	4.35E-02	-1.2	3.08E-02	-0.7	3.50E-02	-0.1	9.19E-01	-0.1	9.19E-01	-0.4	5.71E-01	-0.1	9.44E-01	-0.3	1.0E+00	-0.3	1.0E+00	-0.4	7.1E-01	0.0	1.0E+00	-0.4	3.0E-01	-0.3	1.0E+00	-0.4	9.8E-02	-0.4	3.0E-01
5924	A_24_P392842	A_24_P392842	A_24_P392842	-0.7	1.02E-02	-0.6	7.64E-03	-0.7	2.05E-02	-0.7	1.02E-02	-0.7	1.27E-01	-0.3	6.69E-01	-0.8	1.27E-01	-0.7	8.58E-02	0.1	1.0E+00	-0.2	1.0E+00	0.2	8.3E-01	0.1	1.0E+00	0.0	9.8E-01	0.0	1.0E+00	0.0	9.4E-01	0.0	9.8E-01
18243	LOC550643	A_24_P867868	BX537532	-0.7	1.41E-02	-0.7	1.68E-02	-0.9	1.41E-02	-0.7	2.76E-03	-0.1	8.64E-01	-0.1	8.64E-01	0.0	9.39E-01	-0.5	1.93E-02	0.1	1.0E+00	0.0	1.0E+00	0.2	7.1E-01	0.1	1.0E+00	0.0	9.5E-01	0.0	1.0E+00	0.0	9.5E-01	-0.1	9.3E-01
36486	FAM165B	A_24_P74487	NM_058182	-0.7	1.24E-02	-0.6	1.24E-02	-0.7	4.57E-02	-0.8	1.38E-03	-0.1	9.29E-01	-0.1	9.32E-01	0.0	9.29E-01	-0.5	6.43E-02	0.0	1.0E+00	-0.1	1.0E+00	0.0	9.7E-01	0.1	1.0E+00	-0.1	9.6E-01	0.0	1.0E+00	-0.1	8.2E-01	-0.1	9.6E-01
43255	SFRS2	A_23_P77776	NM_003016	-0.7	1.02E-02	-0.7	1.09E-02	-1.6	3.01E-03	-0.6	1.02E-02	-0.5	2.47E-01	-0.3	4.59E-01	-1.2	2.47E-01	-0.5	2.32E-01	-0.1	1.0E+00	-0.1	1.0E+00	-0.2	7.4E-01	0.0	1.0E+00	-0.2	7.7E-01	-0.2	1.0E+00	-0.2	7.5E-01	-0.3	7.7E-01
8626	C14orf102	A_24_P398790	AK024113	-0.7	6.46E-03	-0.6	9.13E-03	-0.8	6.46E-03	-0.7	6.19E-04	-0.3	4.52E-01	-0.2	7.66E-01	-0.4	4.52E-01	-0.3	3.91E-01	-0.1	1.0E+00	-0.1	1.0E+00	-0.3	7.1E-01	0.1	1.0E+00	0.0	9.8E-01	0.0	1.0E+00	-0.2	7.3E-01	0.0	9.8E-01
3905	AF289610	A_24_P118211	AF289610	-0.7	3.26E-02	-0.6	3.26E-02	-0.7	4.79E-02	-0.7	1.38E-02	-0.7	8.47E-02	-0.5	2.66E-01	-0.7	8.47E-02	-0.9	3.64E-02	-0.1	1.0E+00	0.2	1.0E+00	-0.4	7.1E-01	-0.1	1.0E+00	0.3	8.3E-01	0.4	1.0E+00	0.3	5.8E-01	0.3	8.3E-01
11677	ZNF527	A_23_P402000	NM_032453	-0.7	1.18E-02	-0.7	1.52E-02	-1.0	1.18E-02	-0.7	4.37E-03	-0.1	7.81E-01	0.2	7.81E-01	-0.1	8.47E-01	-0.3	6.01E-01	0.1	1.0E+00	0.0	1.0E+00	0.1	8.9E-01	0.3	1.0E+00	-0.3	6.1E-01	-0.1	1.0E+00	-0.3	5.6E-01	-0.4	6.1E-01
26936	LARS2	A_23_P212397	NM_015340	-0.7	4.92E-04	-0.6	1.77E-03	-1.9	3.30E-04	-0.7	4.92E-04	-0.5	1.25E-02	-0.2	6.20E-01	-1.8	3.11E-04	-0.5	1.25E-02	-0.1	1.0E+00	-0.1	1.0E+00	-0.7	8.8E-03	-0.1	1.0E+00	-0.1	9.2E-01	-0.1	1.0E+00	-0.3	1.9E-01	-0.1	9.2E-01
45153	RAD17	A_24_P97836	NM_002873	-0.7	1.94E-02	-0.7	1.94E-02	-0.8	2.86E-02	-0.6	1.08E-02	-0.6	1.82E-01	-0.4	2.58E-01	-0.8	1.82E-01	-0.6	1.26E-02	-0.1	1.0E+00	-0.2	1.0E+00	0.0	1.0E+00	-0.1	1.0E+00	-0.1	9.7E-01	-0.1	1.0E+00	0.0	9.7E-01	-0.2	7.6E-01
32128	RBM41	A_32_P135091	NM_018301	-0.7	1.89E-02	-0.7	4.64E-02	-0.8	1.89E-02	-0.6	3.58E-03	-0.4	3.15E-01	-0.4	3.15E-01	-0.2	7.19E-01	-0.8	5.15E-02	0.0	1.0E+00	-0.1	1.0E+00	0.2	7.4E-01	0.0	1.0E+00	-0.1	8.2E-01	-0.1	1.0E+00	-0.1	8.2E-01	-0.4	3.5E-01
16588	VTA1	A_24_P294982	NM_016485	-0.7	1.48E-02	-0.7	1.37E-02	-1.1	1.48E-02	-0.6	1.64E-02	-0.3	4.78E-01	-0.1	8.99E-01	-0.6	4.78E-01	-0.3	4.22E-01	-0.2	1.0E+00	-0.2	1.0E+00	-0.2	7.5E-01	0.0	1.0E+00	-0.2	8.7E-01	-0.1	1.0E+00	-0.2	6.5E-01	-0.2	8.7E-01
9402	NUDCD1	A_23_P123343	NM_032869	-0.7	2.42E-02	-0.6	3.42E-02	-1.1	2.42E-02	-0.7	1.32E-02	-0.5	2.18E-01	-0.5	2.18E-01	-0.6	1.95E-01	-0.4	2.31E-01	-0.1	1.0E+00	-0.1	1.0E+00	-0.1	8.8E-01	-0.1	1.0E+00	0.0	1.0E+00	0.1	1.0E+00	-0.1	8.3E-01	0.0	1.0E+00
18691	PHF17	A_23_P167263	NM_199320	-0.6	2.83E-03	-0.6	1.60E-02	-1.2	1.09E-03	-0.6	2.83E-03	-0.4	5.77E-01	-0.2	8.64E-01	-1.1	3.71E-01	-0.4	5.77E-01	-0.2	1.0E+00	-0.2	1.0E+00	-0.3	7.1E-01	0.1	1.0E+00	-0.2	9.3E-01	-0.2	1.0E+00	-0.2	4.7E-01	-0.1	9.3E-01
1524	ZNRF3	A_23_P427502	NM_032173	-0.6	2.96E-02	-0.6	4.70E-02	-0.9	2.96E-02	-0.6	8.43E-03	-0.5	2.54E-01	-0.5	4.02E-01	-1.0	2.54E-01	-0.3	1.23E-01	-0.2	1.0E+00	-0.1	1.0E+00	-0.2	7.3E-01	-0.3	1.0E+00	0.1	9.3E-01	0.2	1.0E+00	0.1	8.6E-01	0.1	9.3E-01
29122	EPB41L5	A_24_P944640	NM_020909	-0.6	7.49E-03	-0.6	7.49E-03	-0.8	1.72E-02	-0.6	4.16E-03	-0.2	7.35E-01	-0.1	8.90E-01	-0.5	5.49E-01	-0.2	7.35E-01	0.0	1.0E+00	0.0	1.0E+00	-0.3	7.1E-01	0.0	1.0E+00	-0.2	7.5E-01	-0.1	1.0E+00	-0.2	3.3E-01	-0.2	7.5E-01
7459	SPCS2	A_23_P2143	NM_014752	-0.6	1.32E-03	-0.6	4.15E-03	-1.0	1.32E-03	-0.6	3.54E-04	-0.4	1.56E-01	-0.3	4.81E-01	-0.9	1.56E-01	-0.4	1.05E-01	0.0	1.0E+00	0.0	1.0E+00	0.0	9.6E-01	0.0	1.0E+00	0.0	9.1E-01	0.0	1.0E+00	0.1	9.1E-01	-0.1	9.1E-01
40716	ZNF485	A_23_P115861	NM_145312	-0.6	2.77E-03	-0.6	1.27E-02	-1.4	2.77E-03	-0.6	2.46E-03	-0.1	8.34E-01	0.0	9.67E-01	-0.3	7.02E-01	-0.1	8.34E-01	-0.1	1.0E+00	-0.1	1.0E+00	-0.1	7.7E-01	0.0	1.0E+00	0.0	9.6E-01	0.0	1.0E+00	0.0	9.5E-01	0.1	9.6E-01
38664	MDH1B	A_24_P111547	NM_001039845	-0.6	2.57E-02	-0.6	2.57E-02	-0.9	2.70E-02	-0.6	6.31E-03	-0.2	8.19E-01	0.2	8.64E-01	-0.2	8.19E-01	-0.2	7.30E-01	0.0	1.0E+00	0.0	1.0E+00	-0.1	8.1E-01	0.3	1.0E+00	-0.4	4.5E-01	-0.1	1.0E+00	-0.4	2.7E-01	-0.5	4.5E-01
26202	CPSF6	A_23_P204033	NM_007007	-0.6	4.26E-02	-0.6	4.47E-02	-1.1	4.26E-02	-0.6	4.00E-02	-0.2	7.99E-01	0.2	8.27E-01	-0.5	3.94E-01	-0.2	7.99E-01	0.0	1.0E+00	0.0	1.0E+00	-0.4	7.1E-01	0.0	1.0E+00	-0.2	5.9E-01	0.1	1.0E+00	-0.2	4.1E-01	-0.3	5.9E-01
43589	TMLHE	A_23_P62335	NM_018196	-0.6	3.93E-03	-0.6	5.43E-03	-1.0	3.90E-03	-0.6	3.93E-03	-0.3	7.05E-01	-0.1	8.15E-01	-0.3	7.05E-01	-0.4	4.82E-01	0.1	1.0E+00	0.0	1.0E+00	0.1	8.8E-01	0.1	1.0E+00	0.0	9.7E-01	0.0	1.0E+00	0.0	9.7E-01	-0.2	7.1E-01
10512	ENST00000370548	A_32_P112592	ENST00000370548	-0.6	3.65E-03	-0.6	3.65E-03	-0.6	2.76E-02	-0.7	1.09E-03	-0.3	3.69E-01	-0.1	8.77E-01	-0.3	3.69E-01	-0.3	2.60E-01	-0.1	1.0E+00	-0.1	1.0E+00	-0.1	8.6E-01	0.0	1.0E+00	-0.2	5.9E-01	-0.1	1.0E+00	-0.2	3.4E-01	-0.2	5.9E-01
210	LOC100128361	A_32_P93996	AK094300	-0.6	2.11E-02	-0.6	2.11E-02	-0.6	4.30E-02	-0.8	8.28E-04	-0.3	5.09E-01	-0.2	7.89E-01	-0.7	3.94E-01	-0.3	5.09E-01	0.1	1.0E+00	0.2	1.0E+00	-0.1	8.8E-01	0.1	1.0E+00	0.0	9.5E-01	0.3	1.0E+00	-0.1	8.4E-01	0.0	9.5E-01
8420	CCNY	A_23_P434421	NM_181698	-0.6	2.43E-02	-0.6	4.81E-02	-0.8	2.43E-02	-0.6	4.17E-03	-0.3	5.03E-01	-0.3	6.45E-01	-0.7	5.03E-01	-0.2	5.01E-01	0.0	1.0E+00	0.0	1.0E+00	0.0	9.1E-01	-0.1	1.0E+00	0.0	9.7E-01	-0.1	1.0E+00	0.0	9.7E-01	0.1	9.6E-01
34834	THC2626223	A_32_P170759	THC2626223	-0.6	3.08E-03	-0.6	4.96E-03	-1.3	2.46E-03	-0.6	3.08E-03	-0.2	6.80E-01	0.0	9.60E-01	-0.5	5.66E-01	-0.2	6.80E-01	-0.1	1.0E+00	-0.1	1.0E+00	-0.1	8.3E-01	0.2	1.0E+00	-0.2	2.7E-01	-0.1	1.0E+00	-0.2	2.7E-01	-0.4	1.3E-01
12718	TSGA14	A_23_P215070	NM_018718	-0.6	1.38E-02	-0.6	1.38E-02	-0.7	3.73E-02	-0.6	2.46E-03	-0.3	6.56E-01	0.1	9.49E-01	-0.3	6.56E-01	-0.5	3.08E-01	0.2	1.0E+00	-0.1	1.0E+00	0.2	7.8E-01	0.2	1.0E+00	-0.1	9.4E-01	-0.1	1.0E+00	-0.1	8.3E-01	-0.1	9.4E-01
8590	ECHDC1	A_23_P82206	NM_018479	-0.6	1.86E-02	-0.6	2.85E-02	-1.1	1.86E-02	-0.6	1.78E-02	-0.6	2.54E-01	-0.5	2.54E-01	-0.6	3.47E-01	-0.6	1.97E-02	0.1	1.0E+00	0.0	1.0E+00	0.1	7.4E-01	0.2	1.0E+00	0.0	9.6E-01	0.0	1.0E+00	0.0	9.6E-01	-0.2	8.3E-01
34700	AK098210	A_32_P214817	AK098210	-0.6	6.90E-03	-0.6	8.10E-03	-1.0	6.90E-03	-0.6	1.96E-03	-0.3	4.87E-01	-0.2	5.60E-01	-0.3	4.87E-01	-0.4	1.86E-01	0.0	1.0E+00	0.0	1.0E+00	-0.2	7.1E-01	0.0	1.0E+00	-0.2	6.4E-01	0.0	1.0E+00	-0.2	3.4E-01	-0.2	6.4E-01
20924	COPS7B	A_23_P79725	NM_022730	-0.6	3.69E-02	-0.6	3.69E-02	-0.7	4.76E-02	-0.6	5.05E-03	-0.3	7.18E-01	-0.3	7.74E-01	-0.8	3.62E-01	-0.2	7.18E-01	0.0	1.0E+00	0.0	1.0E+00	-0.2	8.0E-01	0.0	1.0E+00	-0.1	9.8E-01	-0.1	1.0E+00	-0.1	9.0E-01	0.0	9.8E-01
12931	DUT	A_23_P88484	NM_001025248	-0.6	8.67E-03	-0.6	3.50E-02	-2.7	5.22E-04	-0.6	8.67E-03	-0.5	1.64E-02	-0.4	1.61E-01	-2.1	4.26E-03	-0.5	1.64E-02	-0.1	1.0E+00	0.1	1.0E+00	-1.0	5.0E-02	-0.1	1.0E+00	0.0	9.8E-01	0.0	1.0E+00	-0.2	5.4E-01	0.0	9.8E-01
38792	MRPS22	A_23_P72138	NM_020191	-0.6	4.40E-03	-0.6	4.40E-03	-0.6	1.86E-02	-0.6	5.83E-04	-0.5	1.31E-01	-0.5	1.31E-01	-0.4	2.13E-01	-0.7	1.50E-02	-0.1	1.0E+00	-0.1	1.0E+00	0.0	9.9E-01	-0.2	1.0E+00	0.1	8.2E-01	-0.1	1.0E+00	0.1	7.4E-01	0.1	8.2E-01
32706	CB243784	A_32_P104407	CB243784	-0.6	1.11E-02	-0.6	1.11E-02	-1.0	1.25E-02	-0.6	9.39E-03	-0.2	6.67E-01	0.0	9.64E-01	-0.3	5.88E-01	-0.2	6.67E-01	0.0	1.0E+00	0.0	1.0E+00	0.0	9.9E-01	0.2	1.0E+00	-0.1	8.3E-01	0.0	1.0E+00	-0.2	4.0E-01	-0.1	8.3E-01
9819	ZNF717	A_23_P6980	NM_001128223	-0.6	3.63E-03	-0.6	8.00E-03	-0.9	3.63E-03	-0.6	6.52E-04	-0.2	6.50E-01	-0.1	8.91E-01	-0.3	5.73E-01	-0.2	6.50E-01	0.0	1.0E+00	0.1	1.0E+00	-0.3	7.1E-01	0.0	1.0E+00	-0.1	7.9E-01	0.1	1.0E+00	-0.2	3.4E-01	-0.1	7.9E-01
11111	STK38L	A_24_P99046	NM_015000	-0.6	2.23E-02	-0.6	2.23E-02	-0.9	2.73E-02	-0.6	1.58E-02	-0.3	5.30E-01	0.3	5.30E-01	-0.5	3.44E-01	-0.3	5.44E-01	0.0	1.0E+00	-0.1	1.0E+00	0.0	9.8E-01	0.1	1.0E+00	-0.2	7.2E-01	-0.2	1.0E+00	-0.1	6.3E-01	-0.2	7.2E-01
2133	MSH6	A_23_P102202	NM_000179	-0.6	2.93E-02	-0.6	2.93E-02	-1.0	3.46E-02	-0.6	2.63E-02	-0.4	3.21E-01	-0.4	3.21E-01	-0.3	5.39E-01	-0.5	1.05E-01	0.1	1.0E+00	0.0	1.0E+00	0.1	9.1E-01	0.2	1.0E+00	-0.1	8.3E-01	-0.1	1.0E+00	-0.1	8.3E-01	-0.3	4.6E-01
42512	PPP6C	A_23_P94623	NM_001123355	-0.6	5.49E-03	-0.6	5.49E-03	-0.8	6.09E-03	-0.6	5.29E-04	-0.4	3.20E-01	-0.1	8.64E-01	-0.5	1.57E-01	-0.4	3.20E-01	0.0	1.0E+00	0.0	1.0E+00	-0.1	7.9E-01	0.0	1.0E+00	-0.2	7.0E-01	-0.2	1.0E+00	-0.3	3.7E-01	-0.2	7.0E-01
10922	BC040307	A_32_P53884	BC040307	-0.6	1.04E-02	-0.6	4.44E-03	-0.8	1.22E-02	-0.6	1.04E-02	-0.4	6.00E-01	-0.4	6.65E-01	-0.4	6.00E-01	-0.5	3.13E-01	0.0	1.0E+00	0.1	1.0E+00	0.0	9.6E-01	-0.2	1.0E+00	0.3	9.9E-01	0.3	1.0E+00	0.0	9.9E-01	0.4	7.8E-01
2230	TUSC1	A_23_P373819	NM_001004125	-0.6	2.99E-02	-0.6	4.87E-02	-1.3	2.05E-02	-0.6	2.99E-02	0.0	8.07E-01	0.0	9.73E-01	0.2	8.07E-01	-0.3	5.84E-01	-0.1	1.0E+00	-0.1	1.0E+00	-0.1	8.2E-01	0.1	1.0E+00	-0.2	8.3E-01	0.0	1.0E+00	-0.2	7.3E-01	-0.2	8.3E-01
1204	RGS3	A_24_P377775	NM_017790	-0.6	1.80E-02	-0.6	1.80E-02	-0.8	2.50E-02	-0.6	8.41E-03	-0.5	3.48E-01	-0.3	7.42E-01	-0.7	3.48E-01	-0.5	2.99E-01	0.0	1.0E+00	0.1	1.0E+00	-0.3	7.1E-01	0.0	1.0E+00	-0.3	3.5E-01	-0.2	1.0E+00	-0.3	2.6E-01	-0.4	3.5E-01
806	FEZ2	A_23_P39718	NM_001042548	-0.6	8.74E-03	-0.6	9.69E-03	-1.0	8.74E-03	-0.6	7.97E-03	-0.6	9.56E-02	-0.6	9.56E-02	-1.0	1.35E-01	-0.6	3.58E-02	0.0	1.0E+00	0.0	1.0E+00	0.0	1.0E+00	0.0	1.0E+00	-0.2	6.9E-01	-0.1	1.0E+00	-0.2	6.9E-01	-0.5	5.1E-01
37236	THC2647388	A_32_P120484	THC2647388	-0.6	2.85E-03	-0.6	2.85E-03	-0.6	1.85E-02	-0.6	4.26E-04	-0.2	6.54E-01	-0.2	7.77E-01	-0.2	6.43E-01	-0.2	6.54E-01	0.0	1.0E+00	0.0	1.0E+00	-0.2	7.7E-01	0.1	1.0E+00	-0.2	6.5E-01	-0.1	1.0E+00	-0.2	2.1E-01	-0.2	6.5E-01
15136	ANKRD23	A_23_P305120	NM_144994	-0.6	1.15E-02	-0.6	1.15E-02	-0.9	1.74E-02	-0.6	7.39E-03	-0.3	5.35E-01	-0.2	7.56E-01	-0.3	5.35E-01	-0.7	5.55E-02	0.2	1.0E+00	0.0	1.0E+00	0.2	7.9E-01	0.2	1.0E+00	0.1	8.8E-01	0.0	1.0E+00	0.1	8.2E-01	0.1	8.8E-01
3564	RP9	A_23_P255215	NM_203288	-0.6	5.31E-03	-0.6	5.31E-03	-0.7	1.28E-02	-0.6	1.54E-03	-0.3	2.05E-01	-0.1	8.09E-01	-0.7	9.26E-03	-0.3	2.05E-01	-0.1	1.0E+00	-0.1	1.0E+00	-0.3	7.1E-01	0.2	1.0E+00	0.0	9.3E-01	-0.1	1.0E+00	0.0	9.1E-01	0.1	9.3E-01
36712	FAM49B	A_23_P43255	NM_016623	-0.6	1.15E-02	-0.6	1.87E-02	-1.1	1.15E-02	-0.6	1.01E-02	-0.4	3.76E-01	-0.1	8.40E-01	-0.4	3.76E-01	-0.8	2.48E-02	0.1	1.0E+00	0.1	1.0E+00	0.3	7.1E-01	0.0	1.0E+00	0.0	7.3E-01	0.0	1.0E+00	0.2	7.1E-01	-0.3	7.3E-01
37599	RAD17	A_23_P159053	NM_002873	-0.6	1.03E-02	-0.6	1.03E-02	-0.7	2.56E-02	-0.6	3.43E-03	-0.6	8.33E-02	-0.6	8.33E-02	-0.8	1.36E-01	-0.6	2.67E-02	-0.2	1.0E+00	-0.3	1.0E+00	-0.1	9.2E-01	-0.2	1.0E+00	-0.1	9.2E-01	-0.1	1.0E+00	-0.1	6.4E-01	-0.1	9.2E-01
7679	CTDSPL2	A_32_P76060	NM_016396	-0.6	1.40E-02	-0.6	3.46E-02	-0.9	1.40E-02	-0.6	8.24E-03	-0.4	4.19E-01	-0.4	5.01E-01	-0.4	4.19E-01	-0.6	7.59E-02	0.3	1.0E+00	0.2	1.0E+00	0.3	7.1E-01	0.4	1.0E+00	-0.1	8.8E-01	0.0	1.0E+00	-0.1	8.8E-01	-0.3	4.2E-01
16056	AA126789	A_32_P42426	AA126789	-0.6	3.83E-03	-0.6	3.82E-03	-0.9	4.72E-03	-0.6	3.83E-03	-0.4	2.09E-01	-0.4	2.09E-01	-0.4	2.19E-01	-0.4	6.79E-02	0.2	1.0E+00	0.3	1.0E+00	0.2	7.5E-01	0.1	1.0E+00	0.2	9.9E-01	0.3	1.0E+00	0.0	9.9E-01	0.2	8.7E-01
16080	NUFIP1	A_23_P36939	NM_012345	-0.6	1.19E-02	-0.6	7.87E-03	-0.9	1.28E-02	-0.6	1.19E-02	-0.4	1.27E-01	-0.3	4.40E-01	-0.4	1.27E-01	-0.5	1.08E-01	0.0	1.0E+00	-0.1	1.0E+00	0.2	8.1E-01	0.0	1.0E+00	0.1	9.1E-01	0.1	1.0E+00	0.1	7.4E-01	-0.1	9.1E-01
30953	EDEM2	A_24_P627306	NM_018217	-0.6	1.23E-02	-0.6	1.23E-02	0.7	1.38E-02	-0.8	1.42E-03	-0.4	1.83E-01	-0.4	1.83E-01	0.3	6.55E-01	-0.7	1.17E-02	-0.2	1.0E+00	-0.2	1.0E+00	-0.2	7.2E-01	-0.2	1.0E+00	-0.2	7.4E-01	-0.1	1.0E+00	-0.3	3.8E-01	-0.2	7.4E-01
29476	LST1	A_24_P103469	NM_007161	0.6	1.21E-03	0.6	1.40E-02	0.9	1.21E-03	0.6	3.30E-04	0.2	8.61E-01	0.2	9.05E-01	0.2	8.61E-01	0.2	8.19E-01	0.1	1.0E+00	0.1	1.0E+00	-0.1	7.8E-01	0.4	1.0E+00	-0.2	5.8E-01	0.2	1.0E+00	-0.2	3.2E-01	-0.2	5.8E-01
4469	MYLC2PL	A_23_P393015	ENST00000223167	0.6	1.09E-02	0.6	2.00E-02	0.8	1.09E-02	0.6	3.72E-03	0.4	1.80E-01	0.2	7.04E-01	0.8	5.34E-02	0.4	1.80E-01	0.0	1.0E+00	0.0	1.0E+00	-0.1	7.5E-01	0.1	1.0E+00	-0.1	7.9E-01	0.0	1.0E+00	-0.1	6.2E-01	-0.1	7.9E-01
24878	THC2540294	A_24_P741450	THC2540294	0.6	1.32E-02	0.6	1.32E-02	0.6	2.39E-02	0.8	1.44E-04	0.4	5.62E-01	0.2	7.80E-01	0.4	5.62E-01	0.6	1.67E-01	-0.1	1.0E+00	-0.1	1.0E+00	0.0	1.0E+00	-0.1	1.0E+00	0.1	8.3E-01	-0.1	1.0E+00	0.1	7.0E-01	0.1	8.3E-01
37324	DDB2	A_23_P52610	NM_000107	0.6	2.96E-02	0.6	2.96E-02	1.0	3.65E-02	0.6	2.01E-02	0.5	9.51E-02	0.5	1.17E-01	0.6	9.51E-02	0.5	3.11E-02	0.1	1.0E+00	0.2	1.0E+00	0.1	7.5E-01	0.1	1.0E+00	0.0	1.0E+00	0.0	1.0E+00	-0.1	8.0E-01	0.0	1.0E+00
43261	CARHSP1	A_23_P38085	NM_001042476	0.6	1.64E-02	0.6	1.64E-02	0.8	2.67E-02	0.6	7.43E-03	0.0	8.65E-01	0.0	9.88E-01	-0.2	8.65E-01	0.5	1.02E-01	0.2	1.0E+00	0.2	1.0E+00	0.2	7.1E-01	0.2	1.0E+00	0.0	9.7E-01	0.2	1.0E+00	-0.1	8.4E-01	0.0	9.7E-01
28336	WWC3	A_23_P34176	NM_015691	0.6	6.51E-04	0.6	2.58E-03	1.4	6.51E-04	0.6	2.79E-04	0.5	2.23E-01	0.7	2.00E-01	0.5	3.02E-01	0.5	2.23E-01	0.1	1.0E+00	0.1	1.0E+00	-0.1	9.2E-01	0.1	1.0E+00	-0.1	9.2E-01	0.1	1.0E+00	-0.1	8.9E-01	-0.1	9.2E-01
21243	TRADD	A_23_P54649	NM_003789	0.6	2.73E-03	0.6	8.72E-03	1.3	2.73E-03	0.6	1.70E-03	0.4	7.67E-02	0.4	7.86E-02	0.5	7.67E-02	0.4	5.02E-02	0.1	1.0E+00	0.2	1.0E+00	0.1	7.1E-01	0.1	1.0E+00	0.1	9.8E-01	0.1	1.0E+00	0.0	9.8E-01	0.1	9.5E-01
11897	TFPT	A_23_P27573	NM_013342	0.6	9.96E-03	0.6	1.31E-02	1.3	5.82E-03	0.6	9.96E-03	0.5	1.87E-01	0.3	4.70E-01	0.7	1.87E-01	0.5	3.38E-02	0.0	1.0E+00	0.0	1.0E+00	0.0	9.2E-01	-0.2	1.0E+00	0.0	9.9E-01	0.1	1.0E+00	0.0	9.9E-01	0.0	9.9E-01
4805	LOC200383	A_23_P376014	BC015442	0.6	9.28E-03	0.6	3.42E-02	0.6	9.28E-03	0.8	1.06E-04	0.3	3.63E-01	0.2	6.32E-01	0.3	3.63E-01	0.4	8.85E-02	0.0	1.0E+00	0.0	1.0E+00	0.0	8.8E-01	0.0	1.0E+00	-0.1	9.6E-01	-0.1	1.0E+00	0.0	9.6E-01	-0.1	8.1E-01
37598	PDE4A	A_24_P322474	NM_006202	0.6	1.82E-02	0.6	4.88E-02	1.3	1.13E-02	0.6	1.82E-02	0.7	2.28E-02	0.7	2.28E-02	1.1	5.08E-02	0.7	2.04E-02	0.1	1.0E+00	0.1	1.0E+00	0.1	7.8E-01	0.0	1.0E+00	0.0	8.9E-01	0.0	1.0E+00	-0.1	8.7E-01	0.1	8.9E-01
14825	FAM70B	A_24_P242138	NM_182614	0.6	2.39E-03	0.6	2.63E-02	1.5	1.09E-03	0.6	2.39E-03	0.2	7.78E-01	0.1	9.07E-01	0.2	7.78E-01	0.4	4.89E-01	-0.1	1.0E+00	-0.1	1.0E+00	0.6	7.1E-01	-0.2	1.0E+00	0.1	9.5E-01	0.0	1.0E+00	0.4	7.4E-02	0.1	9.5E-01
44818	C12orf24	A_23_P65000	NM_013300	0.6	1.67E-02	0.6	1.67E-02	0.6	2.54E-02	0.6	2.14E-03	0.3	3.40E-01	0.3	3.40E-01	0.9	3.17E-01	0.3	6.32E-01	-0.2	1.0E+00	-0.1	1.0E+00	-0.2	8.0E-01	-0.2	1.0E+00	0.1	9.8E-01	0.1	1.0E+00	0.1	9.1E-01	0.0	9.8E-01
34903	FLJ11235	A_24_P188975	AK002097	0.6	3.73E-03	0.6	3.73E-03	1.0	4.84E-03	0.6	2.52E-03	0.5	3.55E-01	-0.2	8.19E-01	0.6	3.55E-01	0.5	2.99E-01	0.1	1.0E+00	0.1	1.0E+00	-0.1	9.3E-01	0.1	1.0E+00	0.0	1.0E+00	0.0	1.0E+00	0.0	1.0E+00	-0.1	9.4E-01
23179	FLJ32255	A_24_P829181	AK056817	0.6	2.78E-02	0.6	2.78E-02	1.5	6.39E-03	0.6	3.46E-02	0.4	4.26E-01	0.1	9.00E-01	1.4	3.31E-01	0.4	4.26E-01	-0.1	1.0E+00	-0.1	1.0E+00	-0.1	9.4E-01	-0.4	1.0E+00	0.0	9.6E-01	0.0	1.0E+00	0.2	6.0E-01	-0.1	9.6E-01
27816	PIP5KL1	A_24_P129483	NM_173492	0.6	5.47E-03	0.6	4.14E-03	0.6	1.53E-02	0.8	5.47E-03	0.4	4.36E-01	0.4	4.98E-01	0.4	4.36E-01	0.4	2.66E-01	0.0	1.0E+00	0.1	1.0E+00	0.0	1.0E+00	0.0	1.0E+00	0.1	7.1E-01	0.0	1.0E+00	0.3	2.3E-01	0.1	7.1E-01
35684	HLA-G	A_24_P311926	NM_002127	0.6	1.03E-02	0.6	1.03E-02	0.6	3.25E-02	0.6	1.50E-03	0.4	1.57E-01	0.4	1.57E-01	0.2	6.45E-01	0.5	3.65E-02	0.1	1.0E+00	0.1	1.0E+00	0.1	8.0E-01	0.0	1.0E+00	-0.1	7.6E-01	-0.1	1.0E+00	0.1	7.6E-01	-0.2	6.5E-01
6418	ACY1	A_23_P57868	NM_000666	0.6	6.17E-03	0.6	3.83E-02	1.3	5.16E-03	0.6	6.17E-03	0.6	3.75E-02	0.4	2.78E-01	0.7	3.75E-02	0.6	2.60E-02	0.0	1.0E+00	0.1	1.0E+00	0.0	9.2E-01	-0.1	1.0E+00	0.0	9.9E-01	0.1	1.0E+00	0.0	9.9E-01	0.0	9.8E-01
14573	HLA-C	A_23_P113716	BC002463	0.6	2.83E-03	0.6	2.83E-03	0.6	1.61E-02	0.7	2.06E-04	0.5	7.99E-02	0.5	7.41E-02	0.3	2.46E-01	0.5	7.99E-02	0.0	1.0E+00	0.0	1.0E+00	0.0	1.0E+00	0.0	1.0E+00	0.0	1.0E+00	0.0	1.0E+00	0.1	8.0E-01	0.0	1.0E+00
7874	C1orf187	A_24_P109417	NM_198545	0.6	1.33E-02	0.6	1.33E-02	0.6	4.71E-02	0.7	1.55E-03	0.8	1.67E-01	0.4	5.09E-01	0.8	1.67E-01	0.9	3.34E-02	0.3	1.0E+00	0.2	1.0E+00	0.3	7.2E-01	0.3	1.0E+00	0.0	9.9E-01	0.0	1.0E+00	0.0	9.8E-01	0.0	9.9E-01
19091	HLA-C	A_23_P113716	BC002463	0.6	2.97E-03	0.6	2.97E-03	0.6	1.96E-02	0.8	1.65E-04	0.4	1.54E-01	0.4	1.39E-01	0.3	4.17E-01	0.4	1.54E-01	0.0	1.0E+00	0.0	1.0E+00	0.0	9.9E-01	0.0	1.0E+00	0.0	1.0E+00	0.0	1.0E+00	0.1	7.4E-01	0.0	1.0E+00
37286	FAM108A1	A_23_P5075	NM_031213	0.6	2.12E-02	0.6	4.53E-02	1.1	2.12E-02	0.6	1.61E-02	0.4	1.76E-01	0.1	8.31E-01	0.4	1.76E-01	0.6	1.57E-01	0.1	1.0E+00	0.1	1.0E+00	0.2	7.1E-01	0.1	1.0E+00	-0.2	8.1E-01	0.2	1.0E+00	-0.2	7.4E-01	-0.3	8.1E-01
4776	PHLDB3	A_32_P184488	NM_198850	0.6	1.02E-02	0.6	4.78E-02	0.7	1.02E-02	0.6	1.04E-03	0.5	3.50E-02	0.5	7.42E-02	0.9	1.92E-02	0.5	3.50E-02	0.2	1.0E+00	0.2	1.0E+00	0.1	8.4E-01	0.2	1.0E+00	-0.1	8.3E-01	0.0	1.0E+00	-0.1	8.3E-01	-0.2	7.4E-01
29395	LRP11	A_24_P135841	BC016788	0.6	2.61E-03	0.6	2.61E-03	0.8	6.95E-03	0.6	1.75E-03	1.0	1.03E-02	1.0	1.03E-02	1.0	4.99E-03	0.6	2.83E-02	0.2	1.0E+00	0.2	1.0E+00	0.0	9.8E-01	0.2	1.0E+00	-0.2	8.4E-01	0.0	1.0E+00	-0.2	4.1E-01	-0.2	8.4E-01
1221	KIF12	A_23_P386356	NM_138424	0.6	1.02E-02	0.7	1.02E-02	0.6	1.83E-02	0.6	1.97E-03	0.5	2.62E-01	0.5	2.62E-01	0.4	4.22E-01	0.6	7.26E-02	0.0	1.0E+00	0.1	1.0E+00	-0.1	8.2E-01	0.0	1.0E+00	-0.2	3.4E-01	-0.1	1.0E+00	-0.2	9.3E-02	-0.2	3.4E-01
36741	BFSP2	A_23_P159316	NM_003571	0.6	7.79E-03	0.6	7.79E-03	0.8	1.15E-02	0.6	8.01E-04	0.7	3.02E-02	0.7	1.58E-01	1.1	2.93E-02	0.6	3.02E-02	0.0	1.0E+00	0.0	1.0E+00	-0.1	7.1E-01	0.2	1.0E+00	-0.1	8.3E-01	-0.1	1.0E+00	-0.2	5.2E-01	-0.1	8.3E-01
39793	ETHE1	A_23_P142294	NM_014297	0.6	2.51E-02	0.6	2.88E-02	1.0	2.51E-02	0.6	2.28E-02	0.4	4.40E-01	0.3	6.56E-01	0.4	4.40E-01	0.5	2.04E-01	-0.1	1.0E+00	0.1	1.0E+00	-0.1	8.4E-01	-0.3	1.0E+00	-0.1	9.9E-01	-0.1	1.0E+00	-0.1	8.9E-01	0.0	9.9E-01
12670	M6PR	A_23_P204252	NM_002355	0.6	1.73E-03	0.9	1.01E-03	0.6	4.95E-02	0.6	1.73E-03	0.6	2.23E-01	0.7	2.23E-01	0.4	2.87E-01	0.6	6.31E-02	0.1	1.0E+00	0.1	1.0E+00	-0.2	7.1E-01	0.1	1.0E+00	-0.1	9.6E-01	0.0	1.0E+00	-0.1	9.5E-01	-0.1	9.6E-01
33736	LAG3	A_23_P116942	NM_002286	0.6	1.41E-02	0.8	2.73E-02	0.6	1.41E-02	0.6	5.13E-04	0.3	4.95E-01	0.1	8.84E-01	0.3	4.95E-01	0.4	2.44E-01	-0.1	1.0E+00	-0.1	1.0E+00	-0.3	7.1E-01	-0.1	1.0E+00	0.0	9.8E-01	0.1	1.0E+00	0.0	9.6E-01	0.0	9.8E-01
20270	BEX2	A_23_P22735	NM_032621	0.6	1.02E-02	0.6	1.53E-02	1.1	1.02E-02	0.6	4.09E-03	0.3	3.57E-01	0.3	3.57E-01	0.4	2.08E-01	0.3	4.14E-01	0.1	1.0E+00	0.0	1.0E+00	0.1	7.1E-01	0.2	1.0E+00	0.0	9.8E-01	0.0	1.0E+00	0.0	9.4E-01	0.0	9.8E-01
43996	DACT3	A_24_P33014	NM_145056	0.6	1.03E-03	0.6	1.03E-03	0.9	3.27E-03	0.6	2.12E-04	-0.1	9.62E-01	-0.3	8.23E-01	-0.1	9.62E-01	0.0	9.69E-01	0.0	1.0E+00	0.0	1.0E+00	0.2	7.5E-01	-0.1	1.0E+00	0.2	5.0E-01	0.1	1.0E+00	0.2	3.6E-01	0.2	5.0E-01
34276	FILIP1	A_24_P84668	NM_015687	0.6	6.84E-03	0.6	1.28E-02	0.7	6.84E-03	0.6	3.84E-03	0.6	7.97E-02	0.2	6.53E-01	0.7	7.97E-02	0.6	5.25E-02	-0.1	1.0E+00	-0.1	1.0E+00	-0.1	7.1E-01	0.0	1.0E+00	-0.1	7.4E-01	0.0	1.0E+00	-0.2	2.9E-01	-0.1	7.4E-01
25846	PTGS1	A_24_P64167	NM_000962	0.7	1.14E-02	0.6	1.53E-02	0.9	1.14E-02	0.7	3.24E-03	0.7	6.25E-03	0.7	1.25E-02	0.8	6.25E-03	0.7	3.83E-03	0.1	1.0E+00	0.1	1.0E+00	0.0	9.3E-01	0.1	1.0E+00	0.0	9.7E-01	0.1	1.0E+00	-0.1	7.7E-01	0.0	9.7E-01
34174	LST1	A_24_P94916	NM_007161	0.7	8.80E-04	0.6	6.26E-03	1.2	8.80E-04	0.7	1.58E-04	0.4	1.69E-01	0.4	2.16E-01	0.5	1.69E-01	0.4	9.47E-02	0.0	1.0E+00	0.0	1.0E+00	0.0	9.3E-01	0.0	1.0E+00	-0.1	8.8E-01	0.1	1.0E+00	-0.2	3.7E-01	-0.1	8.8E-01
21221	LOC641518	A_24_P213788	BC020624	0.7	1.84E-02	0.7	2.10E-02	0.6	1.84E-02	1.0	1.06E-04	0.7	7.44E-02	0.7	7.44E-02	0.4	2.70E-01	0.7	2.94E-02	0.0	1.0E+00	0.0	1.0E+00	-0.2	7.1E-01	0.1	1.0E+00	-0.2	7.3E-01	-0.1	1.0E+00	-0.2	4.6E-01	-0.2	7.3E-01
27733	MICB	A_32_P74983	NM_005931	0.7	2.82E-03	0.7	2.82E-03	0.7	1.59E-02	0.6	6.23E-04	0.6	1.10E-02	0.6	1.58E-02	0.6	1.10E-02	0.7	2.06E-03	0.1	1.0E+00	0.1	1.0E+00	0.1	7.9E-01	0.1	1.0E+00	-0.1	8.3E-01	0.1	1.0E+00	-0.1	6.0E-01	-0.1	8.3E-01
36339	TMEM2	A_23_P94552	NM_013390	0.7	7.08E-03	0.7	7.08E-03	0.7	2.37E-02	0.7	8.40E-04	0.5	1.41E-01	0.5	1.41E-01	0.2	6.62E-01	0.6	3.23E-02	0.1	1.0E+00	0.1	1.0E+00	0.1	8.0E-01	0.3	1.0E+00	-0.3	5.9E-01	-0.1	1.0E+00	-0.3	4.7E-01	-0.3	5.9E-01
31967	RP4-692D3.1	A_24_P349196	NM_001080850	0.7	2.04E-02	0.7	4.55E-02	0.7	2.04E-02	1.0	6.19E-05	0.6	7.21E-02	0.6	7.21E-02	0.4	1.25E-01	0.7	1.85E-02	0.1	1.0E+00	0.1	1.0E+00	0.0	9.7E-01	0.2	1.0E+00	-0.2	7.3E-01	-0.1	1.0E+00	-0.2	3.7E-01	-0.2	7.3E-01
29792	PWWP2B	A_23_P374389	NM_138499	0.7	3.50E-03	0.6	1.09E-02	1.2	3.50E-03	0.7	2.38E-03	0.7	3.89E-02	0.3	2.39E-01	0.7	3.89E-02	0.7	2.44E-02	0.3	1.0E+00	0.0	1.0E+00	0.3	7.1E-01	0.3	1.0E+00	0.2	8.6E-01	0.0	1.0E+00	0.2	7.0E-01	0.2	8.6E-01
36099	PRKCD	A_23_P144054	NM_006254	0.7	4.05E-02	0.7	3.44E-02	0.9	4.66E-02	0.6	4.05E-02	0.4	2.97E-01	0.4	2.97E-01	0.2	7.77E-01	0.7	7.37E-02	0.3	1.0E+00	0.3	1.0E+00	0.2	7.1E-01	0.3	1.0E+00	-0.1	9.7E-01	-0.1	1.0E+00	-0.1	8.4E-01	0.0	9.7E-01
21583	PRKCD	A_23_P144054	NM_006254	0.7	1.85E-02	0.7	3.55E-02	1.1	1.85E-02	0.6	1.74E-02	0.5	1.20E-01	0.5	1.20E-01	0.2	7.32E-01	0.6	1.95E-02	0.2	1.0E+00	0.2	1.0E+00	0.2	7.1E-01	0.1	1.0E+00	0.1	8.8E-01	0.0	1.0E+00	0.1	8.8E-01	0.2	8.3E-01

Feature	Gene symbol	Probe	Transcript	72 hour								48 hours							
				siCASP8AP2								siCASP8AP2							
				Median	Median	1	1	3	3	6	6	Median	Median	1	1	3	3	6	6
				M	q	M	q	M	q	M	q	M	q	M	q	M	q	M	q
34693	SLC32A1	A_23_P307382	NM_080552	0.7	1.36E-03	0.7	1.36E-03	0.7	6.94E-03	0.7	1.22E-04	0.4	3.68E-01	0.2	7.67E-01	0.4	3.68E-01	0.5	2.94E-01
34374	CALN1	A_24_P380567	NM_031468	0.7	7.03E-03	0.8	7.03E-03	0.6	2.08E-02	0.7	3.94E-04	0.3	3.66E-01	0.3	3.66E-01	0.2	4.28E-01	0.5	9.85E-02
31001	FOXA1	A_24_P347431	NM_004496	0.7	5.64E-03	0.7	5.64E-03	0.7	2.15E-02	0.6	2.21E-03	0.8	3.94E-02	0.9	4.58E-02	0.8	3.94E-02	0.7	2.61E-02
34308	B3GNT8	A_23_P101380	NM_198540	0.7	5.46E-03	1.0	1.78E-03	0.7	2.48E-02	0.6	5.46E-03	0.3	7.47E-01	0.2	9.00E-01	0.9	4.44E-01	0.3	7.47E-01
44330	NTRK1	A_23_P34804	NM_002529	0.7	5.35E-03	0.8	2.07E-02	0.7	5.35E-03	0.7	1.58E-03	0.3	4.64E-01	0.3	4.64E-01	0.1	7.70E-01	0.5	8.80E-02
26335	P2RY6	A_23_P64611	NM_176798	0.7	8.27E-04	0.7	4.15E-03	1.1	8.27E-04	0.6	1.73E-04	0.6	1.53E-02	0.4	1.83E-01	0.6	1.53E-02	0.6	9.75E-03
16209	CASP10	A_24_P139993	NM_032977	0.7	7.41E-04	0.7	3.23E-03	1.5	7.41E-04	0.7	4.26E-04	0.7	1.65E-02	0.8	1.65E-02	0.5	6.36E-02	0.7	7.24E-03
35224	ZFAND5	A_32_P109817	NM_006007	0.7	4.53E-03	0.6	1.51E-02	1.2	4.53E-03	0.7	1.85E-03	0.5	3.14E-01	0.2	6.99E-01	0.9	3.14E-01	0.5	1.62E-01
27466	IL28A	A_23_P409438	NM_172138	0.7	2.82E-02	0.7	2.82E-02	1.0	7.67E-03	0.7	2.92E-02	0.4	7.25E-01	-0.3	8.59E-01	0.6	6.12E-01	0.4	7.25E-01
10976	SYK	A_23_P9255	NM_003177	0.7	5.49E-03	0.7	4.15E-03	0.7	2.30E-02	0.7	5.49E-03	0.4	1.71E-01	0.2	6.96E-01	0.4	1.71E-01	0.4	8.59E-02
13629	ATP7B	A_23_P205228	NM_000053	0.7	3.65E-03	0.7	3.65E-03	0.7	1.63E-02	0.6	2.83E-03	0.6	1.28E-01	0.7	1.28E-01	0.1	8.05E-01	0.6	1.01E-01
18085	MAP2K3	A_23_P118427	NM_145109	0.7	3.51E-02	0.7	4.61E-02	0.9	3.51E-02	0.7	9.14E-03	0.5	3.30E-01	0.5	3.30E-01	0.4	4.88E-01	0.8	3.75E-02
24151	PRKCD	A_23_P144054	NM_006254	0.7	1.20E-02	0.7	2.30E-02	1.1	1.20E-02	0.6	9.59E-03	0.6	1.01E-01	0.6	1.01E-01	0.4	5.02E-01	0.7	2.45E-02
19460	BC010544	A_32_P34522	BC010544	0.7	1.23E-02	0.6	3.39E-02	2.0	2.59E-03	0.7	1.23E-02	0.6	8.38E-02	0.3	3.84E-01	1.1	8.38E-02	0.6	1.75E-02
38176	FGD3	A_24_P153840	NM_033086	0.7	2.55E-03	0.6	7.92E-03	1.2	2.55E-03	0.7	9.45E-04	1.1	1.58E-02	1.0	5.36E-02	1.1	1.58E-02	1.1	6.08E-03
19691	PLA2G1B	A_24_P224116	NM_000928	0.7	3.58E-04	0.8	2.63E-04	0.7	4.91E-03	0.7	3.58E-04	0.1	9.36E-01	0.1	9.70E-01	0.1	9.36E-01	0.2	8.71E-01
37067	CCIN	A_23_P60227	NM_005893	0.7	1.46E-02	0.7	4.36E-02	0.7	1.46E-02	0.7	9.30E-03	0.4	7.69E-01	-0.3	8.92E-01	0.6	6.86E-01	0.4	7.69E-01
20677	THC2532155	A_23_P114582	THC2532155	0.7	3.58E-02	0.7	4.79E-02	1.4	1.28E-02	0.6	3.58E-02	0.8	2.55E-01	0.7	3.92E-01	1.4	2.55E-01	0.8	2.54E-01
1216	SYK	A_23_P9255	NM_003177	0.7	9.69E-03	0.7	5.20E-03	0.8	2.89E-02	0.6	9.69E-03	0.3	3.09E-01	0.1	8.88E-01	0.4	3.09E-01	0.3	2.59E-01
37231	PGM5	A_24_P254949	NM_021965	0.7	5.68E-03	0.7	5.68E-03	0.9	1.32E-02	0.7	1.73E-03	0.4	4.95E-01	0.0	9.72E-01	0.5	4.95E-01	0.4	4.63E-01
40184	BEGAIN	A_23_P72127	NM_020836	0.7	3.16E-02	0.6	4.14E-02	0.9	3.16E-02	0.7	5.11E-03	0.6	5.95E-02	0.6	9.42E-02	0.8	3.83E-02	0.5	5.95E-02
20456	CREB3L3	A_23_P108082	NM_032607	0.7	4.41E-03	0.7	4.41E-03	0.7	9.78E-03	0.8	2.21E-04	0.5	1.26E-01	0.4	4.35E-01	0.5	1.26E-01	0.6	8.09E-02
15685	BQ072652	A_32_P69399	BQ072652	0.7	1.74E-03	0.6	3.32E-02	1.4	8.23E-04	0.7	1.74E-03	0.4	5.62E-01	0.3	7.31E-01	0.4	5.62E-01	0.4	4.35E-01
23849	MAGEB2	A_23_P254831	NM_002364	0.7	4.38E-03	0.7	4.38E-03	0.7	1.35E-02	0.9	1.82E-04	0.3	6.09E-01	0.2	7.98E-01	0.3	6.09E-01	0.4	3.41E-01
27911	A_24_P152315	A_24_P152315	A_24_P152315	0.7	3.82E-02	0.6	4.76E-02	0.8	3.82E-02	0.7	5.75E-03	0.5	3.03E-01	0.5	4.13E-01	0.5	3.03E-01	0.7	1.07E-01
39952	LOC100131576	A_32_P214340	XM_001723507	0.7	2.82E-02	0.7	3.94E-02	1.1	2.82E-02	0.7	1.47E-02	0.4	5.15E-01	0.2	7.92E-01	0.4	5.14E-01	0.4	5.15E-01
34774	FAM108A1	A_24_P187583	NM_031213	0.7	1.20E-02	0.7	1.20E-02	1.1	1.62E-02	0.7	7.26E-03	0.5	1.52E-01	0.2	7.53E-01	0.5	1.52E-01	0.7	1.57E-02
34359	SYK	A_23_P9255	NM_003177	0.7	8.62E-03	0.7	7.39E-03	0.9	2.28E-02	0.6	8.62E-03	0.5	1.93E-01	0.2	6.89E-01	0.5	1.93E-01	0.5	9.54E-02
13110	IL28RA	A_23_P74112	NM_170743	0.7	3.94E-02	0.6	4.77E-02	0.8	3.94E-02	0.7	4.94E-03	0.6	3.92E-02	1.0	1.89E-02	0.6	1.50E-01	0.6	3.92E-02
9254	RAB11FIP1	A_23_P391198	NM_001002233	0.7	2.98E-03	0.6	1.27E-02	1.4	2.43E-03	0.7	2.98E-03	0.6	2.57E-01	0.2	7.68E-01	1.1	2.57E-01	0.6	2.13E-01
21302	HMOX2	A_23_P100501	NM_002134	0.7	2.09E-02	0.7	2.09E-02	0.9	4.08E-02	0.7	1.10E-02	0.5	2.96E-01	0.5	2.96E-01	0.4	3.78E-01	0.6	1.29E-01
21297	GOLM1	A_24_P394865	NM_016548	0.7	2.63E-02	0.6	2.63E-02	0.8	3.69E-02	0.7	6.18E-03	0.4	3.32E-01	0.2	7.25E-01	0.9	2.66E-01	0.4	3.32E-01
33507	TIGIT	A_23_P80528	NM_173799	0.7	1.48E-03	0.8	1.48E-03	0.7	7.55E-03	0.6	2.86E-04	0.5	9.03E-02	0.1	7.93E-01	0.6	9.03E-02	0.5	7.64E-02
11144	P2RY8	A_24_P340128	NM_178129	0.7	9.47E-04	0.7	9.47E-04	0.8	3.69E-03	0.7	8.94E-04	1.0	3.72E-03	1.0	3.72E-03	1.3	3.68E-03	0.8	7.22E-03
31978	A_32_P101420	A_32_P101420	A_32_P101420	0.7	9.83E-04	1.1	3.60E-04	0.6	4.83E-02	0.7	9.83E-04	1.0	4.27E-03	1.1	4.27E-03	1.0	3.50E-02	1.0	1.19E-03
25883	LONRF3	A_24_P185909	NM_001031855	0.7	1.02E-02	0.7	4.24E-02	1.4	5.33E-03	0.7	1.02E-02	0.8	1.44E-01	0.8	2.42E-01	1.3	1.44E-01	0.7	1.22E-01
35036	FASLG	A_23_P369815	NM_000639	0.7	3.02E-03	0.7	3.02E-03	0.7	1.62E-02	0.9	1.14E-04	0.8	1.68E-02	0.7	4.25E-02	0.8	1.68E-02	1.0	1.69E-03
33501	RHOQ	A_32_P49844	NM_012249	0.7	2.74E-03	0.6	3.29E-03	1.0	2.74E-03	0.7	5.47E-04	0.4	4.22E-01	0.0	9.93E-01	1.2	2.34E-01	0.4	4.22E-01
26755	SAMD8	A_23_P63870	NM_144660	0.7	1.05E-02	0.7	1.05E-02	0.7	2.89E-02	0.9	3.02E-04	0.9	4.05E-02	0.9	4.05E-02	0.5	3.88E-01	1.0	5.64E-03
13088	LARP6	A_23_P117782	NM_018357	0.7	1.29E-02	0.7	1.29E-02	0.9	4.00E-02	0.6	9.16E-03	0.6	5.49E-02	0.7	5.49E-02	0.6	5.04E-02	0.5	1.43E-01
15951	RNF181	A_23_P108708	NM_016494	0.7	3.93E-03	0.7	3.93E-03	1.0	1.00E-02	0.7	1.98E-03	0.6	1.24E-01	0.6	1.35E-01	0.5	1.24E-01	0.6	8.48E-02
3367	GRB2	A_23_P77847	NM_002086	0.7	1.35E-02	0.6	1.35E-02	0.8	3.87E-02	0.7	2.64E-03	0.7	2.11E-02	0.7	2.11E-02	0.5	2.43E-02	0.7	2.50E-03
24206	BC010544	A_24_P42495	BC010544	0.7	1.42E-02	0.6	3.04E-02	2.2	1.96E-03	0.7	1.42E-02	0.5	1.56E-01	0.3	6.25E-01	1.1	1.56E-01	0.5	1.02E-01
27206	GNG3	A_23_P139192	NM_012202	0.7	4.76E-03	0.7	2.34E-02	0.6	4.76E-03	0.9	9.51E-06	0.6	7.76E-02	0.9	7.76E-02	0.5	1.08E-01	0.6	3.25E-02
1545	FASLG	A_23_P369815	NM_000639	0.7	4.37E-03	0.6	4.37E-03	0.7	1.50E-02	0.8	2.42E-04	0.6	1.17E-01	0.6	1.17E-01	0.6	1.66E-01	0.8	2.37E-02
40108	APC2	A_23_P101905	NM_005883	0.7	3.23E-02	0.7	3.23E-02	0.8	3.82E-02	0.7	3.81E-03	0.6	5.23E-02	0.5	1.23E-01	0.6	5.23E-02	0.7	5.39E-03
36901	GRB2	A_23_P77847	NM_002086	0.7	7.47E-03	0.6	7.47E-03	0.8	1.71E-02	0.7	9.09E-04	0.7	1.71E-02	0.7	1.71E-02	0.5	2.34E-02	0.7	1.52E-03
31947	DOK4	A_23_P77381	NM_018110	0.7	6.14E-03	0.7	6.14E-03	1.0	7.47E-03	0.7	1.67E-03	0.7	3.53E-02	0.6	1.41E-01	0.7	3.53E-02	0.9	1.26E-02
39657	CLDN23	A_23_P134854	NM_194284	0.7	2.77E-03	0.7	2.77E-03	0.7	5.65E-03	0.9	1.16E-04	0.7	1.88E-01	0.7	1.88E-01	0.5	3.25E-01	0.7	1.06E-01
41043	IER2	A_23_P153905	NM_004907	0.7	1.37E-02	0.6	1.37E-02	0.7	2.65E-02	0.7	1.29E-03	0.5	7.20E-02	0.5	2.28E-01	0.7	5.86E-02	0.5	7.20E-02
33240	HSD17B14	A_23_P78762	NM_016246	0.7	2.73E-02	0.7	4.20E-02	1.2	4.87E-03	0.6	2.73E-02	0.4	7.73E-01	-0.2	9.23E-01	1.5	4.78E-01	0.4	7.73E-01
23045	SRC	A_23_P308603	NM_005417	0.7	2.23E-02	0.7	4.01E-02	1.1	2.23E-02	0.7	2.23E-02	0.6	4.81E-01	0.6	5.80E-01	0.5	4.81E-01	1.0	7.36E-02
3983	LRRC8A	A_23_P250274	NM_019594	0.7	7.03E-03	0.7	7.03E-03	0.8	2.76E-02	0.7	1.98E-03	0.5	2.57E-01	0.5	3.64E-01	0.4	2.57E-01	0.5	1.72E-01
26252	RNF183	A_23_P83339	NM_145051	0.7	3.28E-03	0.6	3.28E-03	0.8	6.15E-03	0.7	1.56E-04	0.5	2.46E-01	0.4	5.69E-01	0.9	1.15E-01	0.5	2.46E-01
12155	THC2655298	A_32_P211414	THC2655298	0.7	6.78E-03	0.6	6.78E-03	1.0	7.41E-03	0.7	8.26E-04	0.7	3.25E-02	0.7	5.96E-02	0.7	3.25E-02	0.7	1.26E-02
42288	GRB2	A_23_P77847	NM_002086	0.7	8.92E-03	0.6	8.92E-03	0.7	3.33E-02	0.7	1.50E-03	0.7	2.15E-02	0.7	2.15E-02	0.4	6.81E-02	0.7	4.19E-03
6780	NUDT9P1	A_32_P40463	NR_002779	0.7	5.70E-03	0.7	9.47E-03	0.7	5.70E-03	0.6	4.37E-04	0.6	5.35E-02	1.0	2.55E-02	0.6	1.01E-01	0.6	5.35E-02
33303	CORO6	A_23_P107322	NM_032854	0.7	1.03E-02	0.7	1.93E-02	1.0	1.03E-02	0.7	2.66E-03	0.4	2.31E-01	0.4	3.16E-01	0.5	2.31E-01	0.4	1.66E-01
17161	SYK	A_23_P9255	NM_003177	0.7	6.63E-03	0.7	6.63E-03	0.9	1.56E-02	0.7	4.01E-03	0.4	2.35E-01	0.2	7.53E-01	0.4	2.35E-01	0.4	1.65E-01
15689	PCDH12	A_23_P423309	NM_016580	0.7	1.93E-03	0.7	7.03E-03	0.8	1.93E-03	0.7	6.24E-05	0.4	2.50E-01	0.4	2.50E-01	0.3	4.81E-01	0.4	7.42E-02
1167	FLJ20674	A_24_P914000	CR621447	0.7	2.10E-03	0.7	3.47E-03	1.2	2.10E-03	0.7	1.14E-03	1.0	5.54E-02	1.0	1.33E-02	1.0	5.54E-02	0.6	5.62E-02
20388	LOC728855	A_24_P823708	NR_024510	0.7	3.74E-02	0.6	3.74E-02	0.7	4.49E-02	0.8	1.23E-03	0.4	1.33E-01	0.4	1.33E-01	0.4	1.30E-01	0.3	2.13E-01
28104	C17orf62	A_23_P118406	NM_001033046	0.7	9.16E-03	0.7	1.63E-02	1.6	5.38E-03	0.7	9.16E-03	0.5	1.07E-01	0.5	2.09E-01	0.6	8.21E-02	0.4	1.07E-01
17713	DUSP26	A_23_P146134	NM_024025	0.7	3.38E-02	0.7	4.17E-02	0.7	3.38E-02	0.8	9.45E-04	0.3	7.32E-01	0.4	7.32E-01	0.3	6.85E-01	0.1	9.03E-01
4888	RSPH3	A_23_P59397	NM_031924	0.7	2.75E-03	0.7	4.06E-02	0.9	2.75E-03	0.7	2.12E-04	0.6	8.28E-02	0.5	2.37E-01	0.6	8.28E-02	0.6	6.55E-02
21843	RARA	A_32_P5251	NM_001024809	0.7	1.12E-02	0.7	1.75E-02	1.3	1.08E-02	0.6	1.12E-02	0.6	2.61E-01	0.7	2.61E-01	0.6	4.59E-01	0.6	2.38E-01
28803	ENST00000397841	A_23_P309198	ENST00000397841	0.7	3.37E-02	0.7	3.39E-02	0.7	3.37E-02	0.7	2.34E-03	0.7	7.34E-02	0.7	7.34E-02	0.7	1.05E-01	0.7	1.50E-02
5107	KRTAP17-1	A_23_P66858	NM_031964	0.7	1.16E-02	0.9	1.16E-02	0.7	3.24E-02	0.7	3.45E-03	0.6	2.65E-01	0.7	2.65E-01	0.6	2.65E-01	0.5	2.22E-01
15810	TESK2	A_23_P9875	NM_007170	0.7	2.13E-02	0.7	2.13E-02	1.0	2.38E-02	0.7	5.26E-03	0.3	7.92E-01	-0.2	8.91E-01	0.3	7.92E-01	0.3	7.17E-01
32854	SRC	A_23_P308603	NM_005417	0.7	1.88E-02	0.7	3.39E-02	1.1	1.03E-02	0.7	1.88E-02	0.7	3.50E-01	0.7	4.92E-01	0.7	3.50E-01	1.1	5.79E-02
2366	NEO1	A_23_P14798	NM_002499	0.7	4.73E-03	0.9	4.73E-03	0.6	3.72E-02	0.7	9.19E-04	0.6	1.07E-01	0.7	5.75E-02	0.5	1.07E-01	0.6	2.77E-01
26327	FLJ20021	A_32_P131929	AK000028	0.7	7.71E-03	0.7	7.78E-03	1.1	7.71E-03	0.7	2.14E-03	0.7	2.10E-01	0.4	4.98E-01	0.7	2.10E-01	0.7	6.75E-02
5232	LHX2	A_23_P32165	NM_004789	0.7	1.91E-02	0.7	1.91E-02	0.7	2.08E-02	0.8	8.38E-04	0.8	9.58E-02	0.4	3.28E-01	0.8	9.58E-02	0.8	2.60E-02
27890	FASLG	A_23_P369815	NM_000639	0.7	6.51E-03	0.6	6.56E-03	0.7	6.51E-03	0.9	3.46E-05	0.7	1.45E-01	0.7	1.45E-01	0.6	1.48E-01	0.9	1.75E-02
38473	ACY3	A_23_P47102	NM_080658	0.7	2.50E-04	0.9	2.50E-04	0.7	8.27E-03	0.7	1.63E-04	0.5	4.08E-01	0.4	6.52E-01	0.5	4.08E-01	0.7	1.03E-01
13609	SYK	A_23_P9255	NM_003177	0.7	4.43E-03	0.7	3.46E-03	0.8	2.09E-02	0.7	4.43E-03	0.4	1.70E-01	0.2	6.50E-01	0.4	1.70E-01	0.4	8.15E-02
6575	C20orf112	A_23_P372096	AK097804	0.7	1.39E-02	0.8	3.39E-02	0.7	1.39E-02	0.7	2.86E-04	0.3	3.30E-01	0.6	9.07E-02	0.3	3.30E-01	0.3	3.80E-01
18711	ADRBK2	A_24_P378928	NM_005160	0.7	6.21E-03	0.6	9.02E-03	1.2	6.21E-03	0.7	1.71E-03	0.5	2.59E-01	0.4	5.94E-01	0.7	1.98E-01	0.5	2.59E-01
6297	LOC441768	A_24_P316059	XM_001713699	0.7	1.60E-02	0.7	1.60E-02	0.7	4.63E-02	1.2	2.21E-04	0.6	1.50E-01	0.6	1.50E-01	0.1	8.90E-01	1.0	2.61E-02
30834	TIMP2	A_23_P107401	NM_003255	0.7	8.25E-04	0.6	2.27E-02	1.2	8.25E-04	0.7	6.02E-05	0.5	1.42E-01	0.3	2.02E-01	0.5	1.42E-01	0.5	9.55E-02
39534	FDXR	A_23_P38154	NM_004110	0.7	7.98E-03	0.7	7.98E-03	1.4	3.36E-03	0.6	1.52E-02	0.5	5.38E-02	0.3	3.67E-01	0.9	5.38E-02	0.5	4.08E-02
23867	ZNF394	A_23_P157416	NM_032164	0.7	1.29E-02	0.7	1.29E-02	0.7	1.36E-02	0.8	6.21E-04	0.7	6.14E-02	0.5	1.65E-01	0.7	6.14E-02	0.8	1.34E-02
2453	SLC25A20	A_23_P72025	NM_000387	0.7	4.80E-03	0.7	4.80E-03	0.9	1.50E-02	0.7	1.49E-03	0.6	1.17E-01	0.8	6.58E-02	0.4	3.85E-01	0.6	1.17E-01
15150	MYOM2	A_23_P258912	NM_003970	0.7	3.18E-03	0.7	2.09E-02	0.8	3.18E-03	0.7	3.35E-05	0.5	9.65E-02	0.5	1.19E-01	0.5	9.65E-02	0.7	9.29E-03
11060	SYK	A_23_P9255	NM_003177	0.7	4.51E-03	0.8	3.13E-03	0.7	2.98E-02	0.7	4.51E-03	0.4	1.37E-01	0.1	7.92E-01	0.5	1.37E-01	0.4	1.01E-01
23812	TMC8	A_23_P411246	NM_152468	0.7	9.47E-04	0.6	2.71E-03	1.1	9.47E-04	0.7	2.51E-04	0.8	1.06E-01	0.5	3.87E-01	0.9	8.55E-02	0.8	1.06E-01
43027	MFGE8	A_23_P48951	NM_005928	0.7	9.21E-03	0.6	9.21E-03	0.7	3.23E-02	1.1	2.90E-04	0.8	2.43E-02	0.6	6.24E-02	0.8	2.43E-02	0.9	1.04E-02
17269	GRB2	A_23_P77847	NM_002086	0.7	1.06E-02	0.7	1.06E-02	0.8	3.82E-02	0.7	2.47E-03	0.7	7.97E-03	0.7	7.97E-03	0.6	2.38E-02	0.7	1.31E-03
26442	MAL2	A_23_P60130	NM_052886	0.7	2.90E-02	0.7	2.90E-02	1.1	3.93E-02	0.7	1.50E-02	0.3	3.63E-01	0.3	3.63E-01	0.4	4.87E-01	0.2	2.91E-01
19601	ANKHD1-EIF4EBP3	A_23_P58443	NM_020690	0.7	2.96E-02	0.7	2.96E-02	1.0	3.36E-02	0.7	1.64E-02	0.5	2.82E-01	0.6	2.82E-01	0.5	3.03E-01	0.5	2.05E-01
17970	C9orf61	A_23_P422831	NM_004816	0.8	6.50E-03	0.7	1.21E-02	1.5	6.50E-03	0.8	6.31E-03	1.0	6.14E-02	1.1	6.14E-02	1.0	4.49E-02	0.7	6.63E-02
38624	IL3RA	A_23_P253081	NM_002183	0.8	7.99E-03	0.6	7.99E-03	0.8	8.79E-03	0.9	1.57E-04	0.3	6.45E-01	0.2	8.17E-01	0.3	6.45E-01	0.5	3.84E-01
12737	GRB2	A_23_P77847	NM_002086	0.8	1.50E-02	0.6	1.50E-02	0.8	3.23E-02	0.8	1.99E-03	0.7	8.24E-03	0.7	8.24E-03	0.5	2.61E-02	0.7	1.31E-03
10829	CIB2	A_23_P65890	NM_006383	0.8	1.69E-02	0.8	1.69E-02	1.4	4.61E-03	0.6	2.03E-02	0.6	2.10E-01	0.2	7.66E-01	1.2	1.61E-01	0.6	2.10E-01
42845	NRAP	A_23_P402765	NM_198060	0.8	1.42E-03	0.8	7.33E-03	1.0	1.42E-03	0.7	2.93E-04	0.4	1.48E-01	0.3	2.04E-01	0.4	1.10E-01	0.4	1.48E-01
23534	TPM2	A_23_P216501	NM_213674	0.8	9.26E-03	0.7	2.37E-02	1.1	9.26E-03	0.8	2.22E-03	0.3	4.95E-01	0.3	7.24E-01	0.4	4.95E-01	0.3	4.30E-01
2875	INPP5K	A_24_P279328	NM_130766	0.8	3.21E-02	0.8	3.78E-02	1.2	3.21E-02	0.7	3.04E-02	0.8	1.39E-02	0.8	2.72E-02	0.8	1.39E-02	0.8	6.02E-03
7050	HSPA12B	A_23_P91334	NM_052970	0.8	3.65E-03	0.8	3.65E-03	0.8	7.57E-03	0.7	1.25E-03	0.3	4.60E-01	0.3	6.75E-01	0.4	4.27E-01	0.3	4.60E-01
36517	C2orf51	A_23_P432005	NM_152670	0.8	2.51E-03	0.8	2.51E-03	0.7	7.37E-03	1.1	3.14E-05	0.7	3.84E-02	0.3	4.04E-01	0.7	3.84E-02	0.8	7.43E-03
38949	HOXD3	A_23_P323180	NM_006898	0.8	7.65E-03	0.8	7.65E-03	0.7	8.79E-03	0.8	5.97E-05	0.5	1.30E-01	0.5	1.30E-01	0.4	2.76E-01	0.5	6.85E-02
41054	RBP4	A_23_P75283	NM_006744	0.8	1.71E-02	0.8	3.40E-02	0.9	1.71E-02	0.7	2.55E-03	0.5	1.22E-01	0.5	1.22E-01	0.4	1.76E-01	0.7	2.44E-02
3455	PCNT	A_23_P57347	NM_006031	0.8	2.13E-03	0.8	2.13E-03	0.8	1.73E-02	0.7	6.28E-04	0.6	4.04E-02	0.6	5.66E-02	0.5	4.04E-02	0.8	6.06E-03
26737	GMEB1	A_23_P137586	NM_006582	0.8	3.30E-03	0.6	6.89E-03	1.4	3.30E-03	0.8	1.20E-03	0.3	4.34E-01	0.1	9.01E-01	0.7	1.34E-01	0.3	4.34E-01
15821	KCNG1	A_23_P210581	NM_002237	0.8	8.89E-03	0.8	1.26E-02	1.2	6.97E-03	0.7	8.89E-03	0.6	1.61E-01	0.5	3.43E-01	0.9	8.33E-02	0.6	1.61E-01
22684	LOC100131004	A_23_P309619	AK123704	0.8	1.75E-03	0.7	1.75E-03	0.8	4.21E-03	0.8	1.89E-04	0.8	1.74E-01	0.8	1.74E-01	-0.2	8.86E-01	0.8	2.01E-02
14857	SPSB3	A_23_P3496	NM_080861	0.8	1.15E-02	0.6	1.37E-02	1.1	1.15E-02	0.8	2.21E-03	0.6	5.23E-02	0.3	2.04E-01	0.6	5.23E-02	0.6	1.65E-02
38563	DNASE1L1	A_23_P85164	NM_006730	0.8	1.13E-02	0.7	3.18E-02	1.1	1.13E-02	0.8	3.71E-03	0.7	1.13E-01	0.7	1.13E-01	0.4	3.97E-01	0.7	4.03E-02
33344	SDSL	A_23_P53439	NM_138432	0.8	1.34E-03	0.7	4.99E-03	1.7	1.34E-03	0.8	1.14E-03	0.4	6.14E-02	0.4	6.14E-02	0.6	1.10E-01	0.3	5.02E-02

Feature	24 hours								10 hours							
	siCASP8AP2								siCASP8AP2							
	Median	Median	1	1	3	3	6	6	Median	Median	1	1	3	3	6	6
	M	q	M	q	M	q	M	q	M	q	M	q	M	q	M	q
34693	0.0	1.0E+00	0.0	1.0E+00	0.0	1.0E+00	-0.1	1.0E+00	0.2	6.1E-01	0.0	1.0E+00	0.2	3.8E-01	0.2	6.1E-01
34374	0.0	1.0E+00	0.1	1.0E+00	-0.1	8.0E-01	0.0	1.0E+00	0.0	9.5E-01	0.1	1.0E+00	-0.1	7.6E-01	0.0	9.5E-01
31001	0.2	1.0E+00	0.2	1.0E+00	0.2	7.2E-01	0.3	1.0E+00	-0.3	7.4E-01	-0.1	1.0E+00	-0.3	6.1E-01	-0.3	7.4E-01
34308	0.2	1.0E+00	0.2	1.0E+00	0.3	7.1E-01	0.2	1.0E+00	0.0	9.9E-01	0.2	1.0E+00	0.0	9.9E-01	0.0	9.8E-01
44330	0.0	1.0E+00	0.1	1.0E+00	-0.1	7.6E-01	0.0	1.0E+00	0.0	9.7E-01	0.1	1.0E+00	0.0	8.9E-01	0.0	9.7E-01
26335	0.0	1.0E+00	0.0	1.0E+00	0.0	9.9E-01	0.0	1.0E+00	0.0	9.5E-01	0.0	1.0E+00	0.0	9.1E-01	0.0	9.5E-01
16209	0.1	1.0E+00	0.0	1.0E+00	0.1	8.1E-01	0.1	1.0E+00	-0.2	7.5E-01	-0.1	1.0E+00	-0.2	6.5E-01	-0.2	7.5E-01
35224	-0.1	1.0E+00	-0.1	1.0E+00	0.0	9.7E-01	-0.2	1.0E+00	0.2	6.8E-01	0.0	1.0E+00	0.2	5.9E-01	0.2	6.8E-01
27466	-0.1	1.0E+00	-0.1	1.0E+00	0.2	8.0E-01	-0.3	1.0E+00	0.8	1.9E-01	0.0	1.0E+00	0.8	5.8E-02	0.8	1.9E-01
10976	-0.1	1.0E+00	-0.2	1.0E+00	-0.1	9.2E-01	-0.1	1.0E+00	-0.1	9.8E-01	-0.1	1.0E+00	-0.1	8.5E-01	0.0	9.8E-01
13629	0.0	1.0E+00	0.1	1.0E+00	0.0	9.9E-01	0.0	1.0E+00	-0.1	8.7E-01	-0.1	1.0E+00	-0.1	6.6E-01	-0.1	8.7E-01
18085	0.1	1.0E+00	0.2	1.0E+00	0.0	9.6E-01	0.1	1.0E+00	0.0	9.9E-01	-0.1	1.0E+00	0.0	9.9E-01	0.2	9.1E-01
24151	0.2	1.0E+00	0.3	1.0E+00	0.2	7.1E-01	0.2	1.0E+00	0.0	9.8E-01	-0.1	1.0E+00	0.0	9.8E-01	0.1	9.1E-01
19460	0.0	1.0E+00	0.0	1.0E+00	0.1	8.0E-01	0.0	1.0E+00	-0.1	9.6E-01	-0.1	1.0E+00	-0.1	8.2E-01	-0.1	9.6E-01
38176	0.1	1.0E+00	0.1	1.0E+00	-0.1	7.8E-01	0.1	1.0E+00	-0.2	7.5E-01	0.0	1.0E+00	-0.2	5.0E-01	-0.2	7.5E-01
19691	0.0	1.0E+00	-0.1	1.0E+00	0.5	7.1E-01	0.0	1.0E+00	0.2	7.6E-01	0.0	1.0E+00	0.3	8.4E-02	0.2	7.6E-01
37067	-0.1	1.0E+00	-0.1	1.0E+00	0.4	7.2E-01	-0.2	1.0E+00	0.2	9.2E-01	0.0	1.0E+00	0.4	5.1E-01	0.2	9.2E-01
20677	-0.2	1.0E+00	-0.2	1.0E+00	-0.1	9.1E-01	-0.6	1.0E+00	0.5	5.9E-01	0.0	1.0E+00	0.6	2.8E-01	0.5	5.9E-01
1216	-0.1	1.0E+00	-0.2	1.0E+00	-0.1	9.5E-01	-0.1	1.0E+00	0.0	1.0E+00	-0.1	1.0E+00	0.0	9.9E-01	0.0	1.0E+00
37231	0.0	1.0E+00	0.0	1.0E+00	0.1	8.7E-01	-0.2	1.0E+00	0.1	9.1E-01	0.1	1.0E+00	0.1	7.6E-01	0.1	9.1E-01
40184	0.2	1.0E+00	0.0	1.0E+00	0.2	7.3E-01	0.2	1.0E+00	-0.1	8.9E-01	-0.1	1.0E+00	-0.1	7.9E-01	-0.1	8.9E-01
20456	0.0	1.0E+00	0.0	1.0E+00	0.0	1.0E+00	0.1	1.0E+00	0.0	9.3E-01	0.2	1.0E+00	0.0	9.3E-01	-0.1	8.8E-01
15685	-0.1	1.0E+00	-0.1	1.0E+00	-0.4	4.7E-01	-0.1	1.0E+00	0.0	9.7E-01	0.0	1.0E+00	-0.1	8.5E-01	0.0	9.7E-01
23849	-0.2	1.0E+00	-0.2	1.0E+00	-0.2	7.5E-01	-0.2	1.0E+00	0.1	9.5E-01	0.1	1.0E+00	0.2	3.9E-01	0.0	9.5E-01
27911	-0.2	1.0E+00	0.1	1.0E+00	-0.5	7.1E-01	-0.2	1.0E+00	0.1	9.6E-01	0.2	1.0E+00	-0.2	6.0E-01	0.1	9.6E-01
39952	0.0	1.0E+00	0.0	1.0E+00	0.5	7.1E-01	0.0	1.0E+00	0.0	9.9E-01	0.0	1.0E+00	0.0	9.9E-01	-0.2	8.8E-01
34774	0.1	1.0E+00	0.1	1.0E+00	0.2	7.3E-01	-0.1	1.0E+00	0.1	9.7E-01	0.2	1.0E+00	0.0	9.7E-01	0.1	9.4E-01
34359	-0.2	1.0E+00	-0.2	1.0E+00	-0.1	9.1E-01	-0.2	1.0E+00	0.1	9.5E-01	-0.1	1.0E+00	0.1	9.5E-01	0.2	9.0E-01
13110	0.2	1.0E+00	0.2	1.0E+00	-0.2	7.4E-01	0.2	1.0E+00	-0.4	4.1E-01	-0.1	1.0E+00	-0.4	1.4E-01	-0.4	4.1E-01
9254	0.0	1.0E+00	0.3	1.0E+00	-0.2	7.3E-01	0.0	1.0E+00	0.1	9.1E-01	0.1	1.0E+00	0.1	9.1E-01	0.4	4.6E-01
21302	0.0	1.0E+00	0.0	1.0E+00	0.0	9.4E-01	0.0	1.0E+00	0.0	9.9E-01	0.0	1.0E+00	0.1	8.7E-01	0.0	9.9E-01
21297	0.0	1.0E+00	0.1	1.0E+00	0.0	9.5E-01	0.0	1.0E+00	0.0	9.8E-01	0.1	1.0E+00	0.0	8.7E-01	0.0	9.8E-01
33507	0.1	1.0E+00	0.1	1.0E+00	0.1	8.6E-01	-0.1	1.0E+00	0.1	8.2E-01	0.1	1.0E+00	0.1	7.1E-01	0.1	8.2E-01
11144	0.0	1.0E+00	0.0	1.0E+00	0.0	9.7E-01	0.1	1.0E+00	0.0	1.0E+00	-0.1	1.0E+00	0.0	8.6E-01	0.0	1.0E+00
31978	0.1	1.0E+00	0.1	1.0E+00	0.0	9.2E-01	0.1	1.0E+00	0.0	9.7E-01	0.2	1.0E+00	-0.1	8.8E-01	0.0	9.7E-01
25883	0.0	1.0E+00	0.0	1.0E+00	-0.2	7.1E-01	0.1	1.0E+00	-0.1	8.9E-01	0.2	1.0E+00	-0.1	7.9E-01	-0.2	8.9E-01
35036	0.0	1.0E+00	0.0	1.0E+00	-0.3	7.1E-01	0.2	1.0E+00	-0.2	7.8E-01	0.0	1.0E+00	-0.2	4.7E-01	-0.2	7.8E-01
33501	-0.2	1.0E+00	0.2	1.0E+00	-0.2	8.5E-01	-0.4	1.0E+00	0.1	9.5E-01	0.1	1.0E+00	-0.3	6.5E-01	0.1	9.5E-01
26755	0.1	1.0E+00	0.0	1.0E+00	0.1	8.1E-01	0.2	1.0E+00	-0.2	4.8E-01	-0.1	1.0E+00	-0.2	4.8E-01	-0.4	4.7E-01
13088	0.2	1.0E+00	0.2	1.0E+00	0.2	7.1E-01	0.3	1.0E+00	-0.1	9.7E-01	-0.2	1.0E+00	-0.1	8.1E-01	0.0	9.7E-01
15951	0.1	1.0E+00	0.1	1.0E+00	0.1	8.7E-01	0.1	1.0E+00	-0.2	9.2E-01	0.0	1.0E+00	-0.2	7.9E-01	-0.2	9.2E-01
3367	0.2	7.1E-01	0.2	5.1E-01	0.2	7.1E-01	0.2	1.0E+00	0.1	8.7E-01	0.2	1.0E+00	0.1	8.4E-01	0.1	8.7E-01
24206	0.0	1.0E+00	0.0	1.0E+00	0.2	8.0E-01	-0.1	1.0E+00	0.0	9.7E-01	-0.1	1.0E+00	0.0	9.7E-01	0.1	9.6E-01
27206	0.1	9.8E-01	0.1	1.0E+00	0.0	9.6E-01	0.5	9.8E-01	-0.2	5.6E-01	0.0	1.0E+00	-0.2	2.3E-01	-0.2	5.6E-01
1545	0.1	1.0E+00	0.1	1.0E+00	-0.2	7.1E-01	0.2	1.0E+00	-0.3	3.9E-01	0.0	1.0E+00	-0.3	1.9E-01	-0.3	3.9E-01
40108	0.0	1.0E+00	0.0	1.0E+00	0.0	1.0E+00	0.1	1.0E+00	-0.2	3.8E-01	-0.1	1.0E+00	-0.2	3.7E-01	-0.3	3.8E-01
36901	0.2	1.0E+00	0.2	1.0E+00	0.2	7.1E-01	0.1	1.0E+00	0.2	7.7E-01	0.2	1.0E+00	0.1	7.7E-01	0.3	6.7E-01
31947	0.1	1.0E+00	0.1	1.0E+00	0.1	8.4E-01	0.1	1.0E+00	0.0	1.0E+00	-0.1	1.0E+00	0.0	9.1E-01	0.0	1.0E+00
39657	0.1	1.0E+00	0.2	1.0E+00	-0.1	9.2E-01	0.1	1.0E+00	-0.3	3.9E-01	-0.1	1.0E+00	-0.4	1.0E-01	-0.3	3.9E-01
41043	0.2	1.0E+00	0.1	1.0E+00	0.2	7.1E-01	0.3	1.0E+00	-0.2	4.0E-01	-0.1	1.0E+00	-0.2	4.0E-01	-0.3	2.2E-01
33240	-0.2	1.0E+00	-0.2	1.0E+00	0.2	9.0E-01	-0.4	1.0E+00	0.6	2.3E-01	0.0	1.0E+00	0.6	2.3E-01	0.7	1.4E-01
23045	0.1	1.0E+00	0.1	1.0E+00	0.1	8.7E-01	0.0	1.0E+00	0.0	1.0E+00	-0.2	1.0E+00	0.2	6.7E-01	0.0	1.0E+00
3983	0.1	1.0E+00	0.1	1.0E+00	0.1	8.3E-01	0.0	1.0E+00	0.0	1.0E+00	0.0	1.0E+00	0.0	9.9E-01	0.0	1.0E+00
26252	-0.1	1.0E+00	-0.1	1.0E+00	0.1	9.1E-01	-0.1	1.0E+00	0.1	8.3E-01	-0.1	1.0E+00	0.1	8.0E-01	0.1	8.3E-01
12155	0.0	1.0E+00	0.0	1.0E+00	-0.2	8.1E-01	0.1	1.0E+00	-0.3	5.5E-01	-0.1	1.0E+00	-0.3	3.0E-01	-0.3	5.5E-01
42288	0.2	8.0E-01	0.2	8.0E-01	0.2	7.1E-01	0.1	1.0E+00	0.1	9.1E-01	0.1	1.0E+00	0.1	8.8E-01	0.1	9.1E-01
6780	0.0	1.0E+00	0.1	1.0E+00	0.0	9.5E-01	0.0	1.0E+00	-0.2	5.5E-01	0.0	1.0E+00	-0.2	1.9E-01	-0.2	5.5E-01
33303	0.0	1.0E+00	0.0	1.0E+00	0.1	8.6E-01	0.0	1.0E+00	0.1	9.7E-01	0.1	1.0E+00	0.0	9.7E-01	0.2	9.0E-01
17161	-0.2	1.0E+00	-0.2	1.0E+00	-0.2	8.5E-01	-0.2	1.0E+00	0.2	8.6E-01	0.0	1.0E+00	0.2	8.1E-01	0.3	8.6E-01
15689	0.0	1.0E+00	0.0	1.0E+00	0.0	9.9E-01	0.3	1.0E+00	-0.1	1.0E+00	-0.1	1.0E+00	-0.1	8.2E-01	0.0	1.0E+00
1167	0.1	1.0E+00	0.1	1.0E+00	-0.2	7.5E-01	0.1	1.0E+00	-0.3	2.2E-01	0.0	1.0E+00	-0.3	2.2E-01	-0.5	2.0E-01
20388	0.0	1.0E+00	-0.1	1.0E+00	0.0	9.9E-01	0.0	1.0E+00	0.0	1.0E+00	0.0	1.0E+00	-0.1	9.1E-01	0.0	1.0E+00
28104	0.1	1.0E+00	0.1	1.0E+00	0.1	8.7E-01	-0.1	1.0E+00	0.1	9.4E-01	0.0	1.0E+00	0.1	9.4E-01	0.1	9.2E-01
17713	-0.3	1.0E+00	-0.4	1.0E+00	-0.3	7.4E-01	-0.3	1.0E+00	-0.1	8.3E-01	0.0	1.0E+00	-0.1	7.0E-01	-0.2	8.3E-01
4888	0.0	1.0E+00	-0.1	1.0E+00	0.1	8.5E-01	0.0	1.0E+00	-0.2	7.0E-01	0.0	1.0E+00	-0.2	2.5E-01	-0.2	7.0E-01
21843	0.0	1.0E+00	0.0	1.0E+00	0.0	9.9E-01	0.0	1.0E+00	-0.1	9.3E-01	0.0	1.0E+00	-0.2	7.8E-01	-0.1	9.3E-01
28803	0.0	1.0E+00	0.1	1.0E+00	0.0	9.8E-01	0.0	1.0E+00	-0.1	8.4E-01	0.1	1.0E+00	-0.2	2.4E-01	-0.1	8.4E-01
5107	0.3	4.3E-01	0.3	2.5E-01	0.3	4.3E-01	0.3	5.0E-01	0.0	9.5E-01	0.1	1.0E+00	-0.1	8.3E-01	0.0	9.5E-01
15810	0.0	1.0E+00	-0.1	1.0E+00	0.1	8.7E-01	0.0	1.0E+00	-0.1	9.4E-01	-0.2	1.0E+00	0.1	9.3E-01	-0.1	9.4E-01
32854	0.1	1.0E+00	0.1	1.0E+00	0.1	8.6E-01	0.1	1.0E+00	0.0	9.9E-01	-0.2	1.0E+00	0.0	9.3E-01	0.0	9.9E-01
2366	0.0	1.0E+00	0.0	1.0E+00	-0.1	8.5E-01	0.1	1.0E+00	-0.2	8.0E-01	-0.2	1.0E+00	-0.2	5.8E-01	-0.2	8.0E-01
26327	-0.1	1.0E+00	-0.1	1.0E+00	0.1	8.5E-01	-0.1	1.0E+00	0.0	1.0E+00	0.0	1.0E+00	0.1	9.2E-01	0.0	1.0E+00
5232	0.0	1.0E+00	0.2	1.0E+00	-0.1	7.7E-01	0.0	1.0E+00	-0.3	5.3E-01	-0.1	1.0E+00	-0.3	3.9E-01	-0.3	5.3E-01
27890	0.0	1.0E+00	0.0	1.0E+00	-0.3	7.1E-01	0.1	1.0E+00	-0.2	8.2E-01	0.1	1.0E+00	-0.2	5.3E-01	-0.2	8.2E-01
38473	0.2	1.0E+00	0.2	1.0E+00	0.3	7.1E-01	0.0	1.0E+00	0.0	1.0E+00	-0.2	1.0E+00	0.0	1.0E+00	0.1	9.3E-01
13609	-0.1	1.0E+00	-0.2	1.0E+00	-0.1	8.9E-01	-0.1	1.0E+00	0.0	9.9E-01	-0.1	1.0E+00	0.0	9.9E-01	0.1	9.5E-01
6575	0.2	1.0E+00	0.2	1.0E+00	0.0	9.5E-01	0.2	1.0E+00	-0.3	7.3E-01	-0.1	1.0E+00	-0.3	6.6E-01	-0.4	7.3E-01
18711	0.0	1.0E+00	0.0	1.0E+00	-0.1	8.2E-01	0.1	1.0E+00	-0.1	8.6E-01	0.0	1.0E+00	-0.1	8.6E-01	-0.2	6.9E-01
6297	0.2	1.0E+00	0.2	1.0E+00	0.0	9.0E-01	0.2	1.0E+00	-0.1	9.1E-01	-0.1	1.0E+00	-0.2	5.9E-01	-0.1	9.1E-01
30834	-0.1	1.0E+00	-0.1	1.0E+00	-0.1	7.5E-01	-0.2	1.0E+00	-0.1	8.8E-01	0.0	1.0E+00	-0.1	8.0E-01	-0.2	8.8E-01
39534	-0.1	1.0E+00	0.1	1.0E+00	-0.1	8.9E-01	-0.1	1.0E+00	0.1	9.6E-01	0.1	1.0E+00	0.0	9.6E-01	0.1	9.1E-01
23867	0.2	1.0E+00	0.2	1.0E+00	0.1	8.0E-01	0.2	1.0E+00	0.0	9.7E-01	-0.1	1.0E+00	0.0	9.7E-01	0.1	9.3E-01
2453	-0.1	1.0E+00	0.0	1.0E+00	-0.2	7.1E-01	-0.1	1.0E+00	-0.1	8.8E-01	0.1	1.0E+00	-0.1	8.6E-01	-0.2	8.8E-01
15150	0.0	1.0E+00	0.0	1.0E+00	-0.1	8.1E-01	0.2	1.0E+00	-0.1	6.8E-01	-0.1	1.0E+00	-0.1	5.4E-01	-0.1	6.8E-01
11060	-0.1	1.0E+00	-0.2	1.0E+00	-0.1	9.2E-01	-0.1	1.0E+00	0.0	9.9E-01	-0.1	1.0E+00	0.0	9.9E-01	0.0	9.8E-01
23812	0.1	1.0E+00	0.0	1.0E+00	0.1	9.5E-01	0.1	1.0E+00	0.3	4.3E-01	0.2	1.0E+00	0.3	4.2E-01	0.4	4.3E-01
43027	0.0	1.0E+00	0.1	1.0E+00	-0.1	8.6E-01	0.0	1.0E+00	0.0	9.6E-01	0.0	1.0E+00	0.0	9.4E-01	0.0	9.6E-01
17269	0.2	7.1E-01	0.3	3.8E-01	0.2	7.1E-01	0.2	1.0E+00	0.1	8.8E-01	0.1	1.0E+00	0.1	6.9E-01	0.1	8.8E-01
26442	0.1	1.0E+00	0.1	1.0E+00	0.2	7.6E-01	0.0	1.0E+00	0.1	9.3E-01	0.1	1.0E+00	0.2	6.9E-01	0.1	9.3E-01
19601	-0.1	1.0E+00	0.0	1.0E+00	-0.1	9.0E-01	-0.1	1.0E+00	0.2	7.1E-01	0.1	1.0E+00	0.3	2.9E-01	0.2	7.1E-01
17970	0.2	1.0E+00	0.2	1.0E+00	0.0	9.5E-01	0.2	1.0E+00	0.0	9.8E-01	0.1	1.0E+00	0.0	9.8E-01	0.0	9.8E-01
38624	0.0	1.0E+00	0.0	1.0E+00	0.0	1.0E+00	-0.1	1.0E+00	0.0	9.8E-01	-0.1	1.0E+00	0.0	9.8E-01	0.1	8.7E-01
12737	0.1	7.3E-01	0.3	7.3E-01	0.1	7.1E-01	0.1	1.0E+00	0.2	7.7E-01	0.2	1.0E+00	0.1	6.6E-01	0.2	7.7E-01
10829	0.2	1.0E+00	0.2	1.0E+00	0.4	7.1E-01	-0.1	1.0E+00	0.2	9.6E-01	0.3	1.0E+00	0.1	9.6E-01	0.2	9.1E-01
42845	0.0	1.0E+00	0.0	1.0E+00	0.2	7.1E-01	0.0	1.0E+00	0.1	9.6E-01	0.1	1.0E+00	0.0	9.6E-01	0.1	8.8E-01
23534	-0.1	1.0E+00	0.1	1.0E+00	-0.1	8.3E-01	-0.1	1.0E+00	0.3	6.5E-01	0.3	1.0E+00	0.4	2.9E-01	0.3	6.5E-01
2875	0.1	1.0E+00	0.2	1.0E+00	0.1	7.4E-01	0.1	1.0E+00	-0.1	9.7E-01	-0.2	1.0E+00	-0.1	4.7E-01	0.0	9.7E-01
7050	0.1	1.0E+00	0.0	1.0E+00	0.1	8.0E-01	0.1	1.0E+00	-0.1	9.2E-01	-0.1	1.0E+00	0.0	8.3E-01	-0.1	9.2E-01
36517	0.0	1.0E+00	0.1	1.0E+00	0.0	9.5E-01	0.0	1.0E+00	0.1	9.9E-01	0.1	1.0E+00	0.0	9.9E-01	0.1	9.2E-01
38949	0.0	1.0E+00	0.1	1.0E+00	0.0	8.9E-01	0.0	1.0E+00	0.0	9.8E-01	0.0	1.0E+00	0.0	9.7E-01	0.0	9.8E-01
41054	0.1	1.0E+00	0.1	1.0E+00	-0.1	8.8E-01	0.1	1.0E+00	-0.1	5.9E-01	0.0	1.0E+00	-0.1	4.5E-01	-0.2	5.9E-01
3455	0.1	1.0E+00	0.2	1.0E+00	-0.1	7.6E-01	0.1	1.0E+00	0.0	1.0E+00	0.0	1.0E+00	0.0	9.9E-01	0.0	1.0E+00
26737	-0.1	1.0E+00	0.0	1.0E+00	-0.1	8.3E-01	-0.2	1.0E+00	0.0	9.7E-01	0.1	1.0E+00	-0.2	6.3E-01	0.0	9.7E-01
15821	0.0	1.0E+00	0.1	1.0E+00	0.0	9.7E-01	-0.1	1.0E+00	-0.1	9.3E-01	-0.2	1.0E+00	-0.1	9.2E-01	-0.1	9.3E-01
22684	0.0	1.0E+00	0.1	1.0E+00	-0.2	7.7E-01	0.0	1.0E+00	0.0	9.8E-01	0.0	1.0E+00	0.0	9.8E-01	0.0	9.8E-01
14857	0.2	1.0E+00	0.1	1.0E+00	0.2	7.1E-01	0.2	1.0E+00	0.0	1.0E+00	0.1	1.0E+00	0.0	1.0E+00	0.0	1.0E+00
38563	0.1	1.0E+00	0.1	1.0E+00	-0.1	8.2E-01	0.1	1.0E+00	-0.2	7.7E-01	-0.2	1.0E+00	-0.3	5.3E-01	-0.2	7.7E-01
33344	0.0	1.0E+00	0.2	1.0E+00	0.0	9.3E-01	0.0	1.0E+00	0.0	9.7E-01	0.0	1.0E+00	0.0	9.7E-01	0.1	9.7E-01

Feature	Gene symbol	Probe	Transcript	72 hour siCASP8AP2 Median M	72 hour Median q	72 hour 1 M	72 hour 1 q	72 hour 3 M	72 hour 3 q	72 hour 6 M	72 hour 6 q	48 hours siCASP8AP2 Median M	48 hours Median q	48 hours 1 M	48 hours 1 q	48 hours 3 M	48 hours 3 q	48 hours 6 M	48 hours 6 q	24 hours siCASP8AP2 Median M	24 hours Median q	24 hours 1 M	24 hours 1 q	24 hours 3 M	24 hours 3 q	24 hours 6 M	24 hours 6 q	10 hours siCASP8AP2 Median M	10 hours Median q	10 hours 1 M	10 hours 1 q	10 hours 3 M	10 hours 3 q	10 hours 6 M	10 hours 6 q
21454	PPP1R16A	A_23_P157715	NM_032902	0.8	2.20E-02	0.6	2.20E-02	0.9	3.91E-02	0.8	4.77E-03	0.5	7.78E-02	0.5	7.78E-02	0.7	3.09E-02	0.3	1.83E-01	0.1	1.0E+00	0.1	1.0E+00	0.2	7.1E-01	0.1	1.0E+00	0.2	6.7E-01	0.1	1.0E+00	0.2	3.3E-01	0.2	6.7E-01
12040	OR7E5P	A_23_P408930	AK055955	0.8	8.50E-04	0.6	7.24E-03	1.5	8.50E-04	0.8	2.58E-04	0.5	3.78E-01	0.4	4.46E-01	0.5	3.78E-01	0.8	5.99E-02	0.0	1.0E+00	0.0	1.0E+00	0.3	7.1E-01	0.0	1.0E+00	0.1	9.2E-01	0.0	1.0E+00	0.1	6.5E-01	0.1	9.2E-01
8679	EOMES	A_24_P97374	NM_005442	0.8	1.39E-02	1.1	1.39E-02	0.7	3.69E-02	0.8	3.47E-03	1.1	1.51E-02	1.2	2.51E-02	1.0	1.51E-02	1.1	4.29E-03	0.1	1.0E+00	0.1	1.0E+00	-0.1	8.9E-01	0.2	1.0E+00	-0.3	3.5E-01	-0.1	1.0E+00	-0.3	2.3E-01	-0.3	3.5E-01
30170	CD97	A_23_P502312	NM_078481	0.8	3.86E-03	0.8	3.86E-03	1.3	4.00E-03	0.8	1.04E-03	0.7	1.75E-02	0.7	1.75E-02	0.8	1.99E-02	0.7	5.62E-03	0.1	1.0E+00	0.1	1.0E+00	0.0	9.3E-01	0.1	1.0E+00	0.1	9.5E-01	0.1	1.0E+00	0.1	9.2E-01	0.1	9.5E-01
13712	USH1C	A_23_P116430	NM_005709	0.8	8.70E-03	0.8	8.70E-03	0.8	1.39E-02	0.6	2.05E-03	0.9	4.90E-02	0.8	2.05E-01	1.1	4.82E-02	0.9	4.90E-02	0.1	1.0E+00	0.0	1.0E+00	0.1	8.2E-01	0.2	1.0E+00	0.0	9.9E-01	0.0	1.0E+00	0.0	9.9E-01	0.1	9.1E-01
12291	SOCS3	A_23_P207058	NM_003955	0.8	9.01E-03	0.8	1.75E-02	1.4	6.43E-03	0.7	9.01E-03	0.3	7.44E-01	0.3	7.44E-01	0.3	7.57E-01	0.5	3.64E-01	-0.1	1.0E+00	0.1	1.0E+00	-0.3	7.1E-01	-0.1	1.0E+00	-0.1	9.7E-01	-0.1	1.0E+00	-0.4	4.9E-01	-0.1	9.7E-01
37786	RNF149	A_23_P120153	NM_173647	0.8	1.22E-02	0.7	1.22E-02	0.8	3.77E-02	0.8	1.23E-03	0.6	9.61E-02	0.5	2.81E-01	0.6	9.61E-02	0.7	6.15E-02	0.3	1.0E+00	0.2	1.0E+00	0.3	2.4E-01	0.3	1.0E+00	0.0	9.8E-01	0.1	1.0E+00	0.0	9.8E-01	-0.2	8.2E-01
17467	PLCD4	A_23_P385105	NM_032726	0.8	4.54E-03	0.8	4.54E-03	0.7	4.56E-02	0.8	2.04E-03	0.7	5.80E-02	0.6	7.48E-02	0.7	5.80E-02	0.7	3.63E-02	0.0	1.0E+00	0.0	1.0E+00	0.0	9.0E-01	0.0	1.0E+00	0.0	9.8E-01	0.2	1.0E+00	-0.1	8.4E-01	0.0	9.8E-01
25374	FHL2	A_23_P108751	NM_001039492	0.8	1.06E-02	0.7	1.06E-02	0.8	2.95E-02	0.9	6.53E-04	0.5	1.84E-01	0.5	1.84E-01	0.3	5.64E-01	0.5	1.40E-01	0.2	1.0E+00	0.2	1.0E+00	0.1	7.5E-01	0.2	1.0E+00	0.0	9.8E-01	0.1	1.0E+00	0.0	9.8E-01	-0.1	9.6E-01
21390	JAM3	A_23_P217998	NM_032801	0.8	1.46E-03	0.7	3.45E-02	1.4	7.64E-04	0.8	1.46E-03	0.4	2.62E-01	0.1	6.59E-01	0.4	2.62E-01	0.5	2.03E-01	-0.1	1.0E+00	-0.1	1.0E+00	-0.2	7.1E-01	-0.1	1.0E+00	-0.1	8.9E-01	-0.1	1.0E+00	-0.1	8.9E-01	-0.2	8.0E-01
29991	TMCC2	A_32_P133840	NM_014858	0.8	8.77E-04	0.8	8.77E-04	0.8	9.90E-03	0.7	4.26E-04	1.1	1.71E-02	1.1	1.71E-02	0.8	6.10E-02	1.1	4.41E-03	0.2	1.0E+00	0.2	1.0E+00	0.2	7.1E-01	0.2	1.0E+00	-0.1	9.9E-01	-0.2	1.0E+00	-0.1	8.5E-01	0.0	9.9E-01
19185	CAMK1D	A_23_P124252	NM_020397	0.8	2.55E-03	0.8	1.75E-02	1.1	2.55E-03	0.6	7.10E-04	0.7	1.06E-01	0.7	1.06E-01	0.4	2.30E-01	0.7	3.55E-02	0.0	1.0E+00	0.0	1.0E+00	0.1	8.0E-01	0.0	1.0E+00	0.0	8.9E-01	0.0	1.0E+00	0.1	7.7E-01	-0.1	8.9E-01
38385	CXorf27	A_24_P281872	NM_012274	0.8	3.18E-03	0.8	3.18E-03	0.6	8.18E-03	1.3	1.76E-06	0.5	2.64E-01	0.4	3.13E-01	0.5	2.64E-01	0.7	1.87E-02	0.0	1.0E+00	0.0	1.0E+00	0.0	9.0E-01	0.0	1.0E+00	0.0	9.7E-01	0.0	1.0E+00	0.0	9.6E-01	0.0	9.7E-01
20659	ANKRD37	A_24_P237586	NM_181726	0.8	1.16E-02	0.8	1.16E-02	0.7	2.46E-02	1.0	9.06E-05	0.5	1.27E-01	0.5	1.27E-01	0.4	2.59E-01	0.5	8.37E-02	0.0	1.0E+00	-0.2	1.0E+00	0.0	9.3E-01	0.0	1.0E+00	-0.2	7.9E-01	-0.2	1.0E+00	-0.3	3.6E-01	-0.2	7.9E-01
26689	EMR1	A_23_P27556	NM_001974	0.8	4.16E-03	0.7	4.68E-03	0.8	4.16E-03	0.8	9.00E-05	0.1	9.52E-01	0.1	9.52E-01	0.0	9.93E-01	0.3	7.98E-01	0.0	1.0E+00	0.0	1.0E+00	0.3	7.1E-01	-0.1	1.0E+00	0.1	8.9E-01	-0.1	1.0E+00	0.1	7.6E-01	0.1	8.9E-01
16110	TEP1	A_23_P65481	NM_007110	0.8	7.56E-03	0.7	1.40E-02	1.2	7.56E-03	0.8	2.48E-03	0.7	1.47E-01	0.7	1.47E-01	0.9	8.22E-02	0.5	2.92E-01	0.0	1.0E+00	0.0	1.0E+00	0.0	9.4E-01	0.0	1.0E+00	0.0	9.9E-01	0.0	1.0E+00	0.0	9.9E-01	-0.2	8.6E-01
23735	SIGLEC11	A_23_P119322	NM_052884	0.8	3.21E-03	1.0	7.86E-03	0.8	3.21E-03	0.7	7.40E-05	0.7	6.11E-02	0.7	6.11E-02	0.7	7.29E-02	0.6	5.73E-02	0.2	1.0E+00	0.2	1.0E+00	0.1	8.9E-01	0.2	1.0E+00	0.0	9.7E-01	-0.1	1.0E+00	0.0	9.7E-01	0.0	9.6E-01
14328	CD518596	A_32_P110485	CD518596	0.8	1.25E-02	0.6	2.57E-02	0.8	1.25E-02	1.2	1.05E-04	0.4	2.30E-01	0.4	2.30E-01	0.1	6.66E-01	0.4	1.29E-01	0.1	1.0E+00	0.1	1.0E+00	0.1	7.8E-01	0.0	1.0E+00	0.1	7.5E-01	0.1	1.0E+00	0.1	5.7E-01	0.2	7.5E-01
1936	EFNA1	A_23_P113005	NM_004428	0.8	1.86E-02	0.7	2.97E-02	1.2	1.86E-02	0.8	7.19E-03	0.5	2.68E-01	0.3	5.32E-01	0.5	2.68E-01	0.6	1.40E-01	0.1	1.0E+00	-0.1	1.0E+00	0.2	7.2E-01	0.1	1.0E+00	-0.1	9.6E-01	-0.1	1.0E+00	-0.1	9.0E-01	0.0	9.6E-01
23899	GGT1	A_24_P76267	NM_005265	0.8	6.23E-03	0.8	2.70E-03	1.1	6.23E-03	0.6	2.90E-02	0.6	2.94E-01	0.5	4.53E-01	0.7	2.94E-01	0.6	2.58E-01	-0.1	1.0E+00	-0.1	1.0E+00	0.1	8.6E-01	-0.2	1.0E+00	0.5	6.3E-02	0.1	1.0E+00	0.5	4.9E-02	0.6	6.3E-02
34526	A_32_P113462	A_32_P113462	A_32_P113462	0.8	1.74E-02	0.8	2.03E-02	0.8	1.74E-02	0.7	1.16E-03	0.6	5.58E-02	0.6	8.40E-02	0.6	5.58E-02	0.7	2.05E-02	0.0	1.0E+00	0.0	1.0E+00	0.0	9.1E-01	0.2	1.0E+00	-0.3	4.8E-01	-0.1	1.0E+00	-0.3	3.4E-01	-0.3	4.8E-01
13964	BAD	A_23_P150207	NM_004322	0.8	1.96E-03	0.8	2.61E-03	1.5	1.96E-03	0.8	6.86E-04	0.8	6.51E-03	0.8	1.08E-02	1.0	6.51E-03	0.8	4.07E-03	0.1	1.0E+00	0.1	1.0E+00	0.1	8.3E-01	0.2	1.0E+00	-0.1	9.1E-01	-0.1	1.0E+00	-0.1	9.0E-01	-0.2	9.1E-01
42167	GNG8	A_24_P79070	NM_033258	0.8	9.11E-04	0.8	6.45E-03	1.4	9.11E-04	0.7	6.82E-04	0.5	4.40E-01	0.1	9.46E-01	0.5	4.40E-01	0.5	3.97E-01	0.1	1.0E+00	0.1	1.0E+00	0.3	7.1E-01	0.0	1.0E+00	0.1	9.5E-01	0.1	1.0E+00	0.2	5.6E-01	0.1	9.5E-01
18578	GCNT3	A_23_P151915	ENST00000267857	0.8	8.79E-03	0.6	1.41E-02	1.0	8.79E-03	0.8	1.09E-03	0.7	4.59E-02	0.6	7.15E-02	1.0	5.10E-03	0.7	4.59E-02	0.2	1.0E+00	0.2	1.0E+00	0.3	7.1E-01	0.0	1.0E+00	0.0	9.9E-01	0.0	1.0E+00	0.1	6.2E-01	0.0	9.9E-01
6791	SAT1	A_23_P137016	NM_002970	0.8	1.04E-02	0.8	1.15E-02	1.2	1.04E-02	0.7	8.24E-03	0.8	1.55E-01	0.9	1.55E-01	0.6	3.33E-01	0.8	1.07E-01	0.0	1.0E+00	0.0	1.0E+00	0.0	1.0E+00	-0.1	1.0E+00	-0.1	9.7E-01	-0.1	1.0E+00	0.1	9.1E-01	-0.1	9.7E-01
10940	SLC41A2	A_23_P204801	NM_032148	0.8	7.61E-03	0.7	7.61E-03	0.9	1.71E-02	0.8	1.72E-03	0.8	9.62E-03	0.7	3.34E-02	1.0	9.09E-03	0.8	9.62E-03	0.0	1.0E+00	0.0	1.0E+00	-0.3	7.1E-01	0.1	1.0E+00	-0.2	5.9E-01	0.1	1.0E+00	-0.2	3.6E-01	-0.2	5.9E-01
26928	ROBO3	A_23_P87082	NM_022370	0.8	3.23E-03	0.8	4.57E-03	1.5	2.32E-03	0.8	3.23E-03	0.6	4.63E-01	0.3	7.98E-01	0.6	4.63E-01	0.6	2.74E-01	-0.1	1.0E+00	-0.1	1.0E+00	0.1	9.1E-01	-0.2	1.0E+00	0.1	8.4E-01	0.1	1.0E+00	0.1	7.7E-01	0.2	8.4E-01
32461	TUBA4A	A_23_P154065	NM_006000	0.8	1.38E-03	0.9	1.38E-03	0.6	2.64E-02	0.8	4.82E-04	0.7	1.72E-02	0.8	2.91E-02	0.7	1.72E-02	0.7	1.58E-02	0.1	1.0E+00	0.1	1.0E+00	0.0	9.3E-01	0.1	1.0E+00	-0.1	8.5E-01	-0.1	1.0E+00	-0.1	7.9E-01	-0.1	8.5E-01
718	C10orf10	A_23_P35597	NM_007021	0.8	7.90E-03	0.8	7.90E-03	0.7	1.15E-02	1.1	6.98E-05	0.4	2.10E-01	0.3	2.77E-01	0.4	2.10E-01	0.7	7.06E-03	0.1	1.0E+00	0.1	1.0E+00	-0.1	8.2E-01	0.1	1.0E+00	0.0	9.7E-01	-0.1	1.0E+00	0.0	9.6E-01	0.0	9.7E-01
34335	PLAC4	A_24_P76558	NM_182832	0.8	4.74E-03	0.8	4.74E-03	0.8	7.78E-03	1.2	7.83E-06	0.6	4.94E-02	0.3	3.74E-01	0.6	4.94E-02	0.7	3.42E-02	0.0	1.0E+00	0.0	1.0E+00	0.0	8.9E-01	0.0	1.0E+00	0.0	1.0E+00	0.0	1.0E+00	0.0	9.9E-01	0.0	1.0E+00
30720	AK024898	A_32_P170664	AK024898	0.8	8.23E-04	0.8	8.23E-04	0.6	1.70E-02	1.0	2.27E-05	0.9	1.23E-01	0.9	1.23E-01	0.6	4.12E-01	1.2	9.46E-03	-0.1	1.0E+00	-0.1	1.0E+00	-0.2	7.5E-01	0.3	1.0E+00	-0.1	9.3E-01	0.0	1.0E+00	-0.2	5.5E-01	-0.1	9.3E-01
26961	GMIP	A_23_P56228	NM_016573	0.8	2.26E-03	0.8	5.31E-03	1.4	2.26E-03	0.7	1.75E-03	0.7	2.57E-02	0.7	2.57E-02	1.1	2.95E-02	0.7	9.14E-03	0.1	1.0E+00	0.1	1.0E+00	0.1	7.9E-01	0.1	1.0E+00	0.0	1.0E+00	0.0	1.0E+00	-0.1	9.4E-01	0.0	1.0E+00
13153	THC2656077	A_32_P206618	THC2656077	0.8	5.25E-03	0.7	3.93E-02	1.5	4.98E-03	0.8	5.25E-03	0.5	1.88E-01	0.5	1.88E-01	0.9	6.77E-02	0.3	2.36E-01	0.0	1.0E+00	0.0	1.0E+00	0.1	7.8E-01	-0.1	1.0E+00	-0.2	9.0E-01	-0.1	1.0E+00	-0.2	8.6E-01	-0.2	9.0E-01
14471	GPR162	A_24_P287392	NM_019858	0.8	3.21E-03	0.7	7.19E-03	0.8	3.21E-03	0.8	2.61E-04	0.3	8.05E-01	0.3	8.05E-01	0.2	8.84E-01	0.4	6.15E-01	0.1	1.0E+00	0.2	1.0E+00	0.0	9.9E-01	0.1	1.0E+00	0.0	9.6E-01	0.3	1.0E+00	-0.1	8.2E-01	0.0	9.6E-01
42306	FBLN5	A_23_P151805	NM_006329	0.8	3.81E-03	0.6	3.85E-02	1.4	3.81E-03	0.8	2.08E-03	0.6	3.16E-02	0.5	1.50E-01	0.7	2.43E-02	0.6	3.16E-02	0.0	1.0E+00	0.1	1.0E+00	-0.1	8.1E-01	0.0	1.0E+00	-0.3	4.3E-01	-0.1	1.0E+00	-0.3	1.3E-01	-0.3	4.3E-01
26673	MYLK2	A_23_P80008	NM_033118	0.8	7.82E-03	0.8	2.92E-02	1.2	5.49E-03	0.6	7.82E-03	0.5	2.61E-01	0.5	2.65E-01	0.8	2.61E-01	0.5	7.67E-02	-0.1	1.0E+00	-0.1	1.0E+00	-0.1	7.1E-01	-0.1	1.0E+00	0.1	9.9E-01	0.1	1.0E+00	0.0	9.9E-01	0.1	8.8E-01
10832	PDCL	A_23_P216949	NM_005388	0.8	9.96E-03	0.6	1.93E-02	0.9	9.96E-03	0.8	7.73E-04	0.7	8.93E-02	0.8	8.60E-02	0.7	8.94E-02	0.5	8.93E-02	0.3	1.0E+00	0.2	1.0E+00	0.3	7.1E-01	0.3	1.0E+00	-0.3	6.7E-01	-0.1	1.0E+00	-0.3	3.6E-01	-0.3	6.7E-01
14933	FUCA1	A_23_P11543	NM_000147	0.8	3.74E-02	0.7	3.74E-02	1.0	4.28E-02	0.8	7.56E-03	0.8	8.48E-02	0.8	8.48E-02	0.4	5.10E-01	0.9	3.10E-03	0.1	1.0E+00	0.1	1.0E+00	0.0	9.5E-01	0.1	1.0E+00	-0.1	9.3E-01	-0.1	1.0E+00	-0.1	7.7E-01	-0.1	9.3E-01
19115	LOC442421	A_23_P43549	NR_024496	0.8	3.16E-03	0.9	3.16E-03	0.8	4.41E-02	0.8	2.59E-03	0.6	1.29E-01	0.6	1.29E-01	0.4	5.98E-01	0.8	8.60E-03	0.2	1.0E+00	0.1	1.0E+00	0.3	7.1E-01	0.2	1.0E+00	-0.1	9.6E-01	-0.1	1.0E+00	-0.1	9.4E-01	-0.1	9.6E-01
27081	SERPINF1	A_23_P100660	NM_002615	0.8	5.46E-03	0.7	5.46E-03	0.8	1.86E-02	1.1	9.63E-05	0.6	1.92E-01	0.6	2.02E-01	0.6	1.92E-01	0.8	2.18E-02	0.0	1.0E+00	0.1	1.0E+00	0.0	9.6E-01	0.0	1.0E+00	0.0	9.9E-01	0.0	1.0E+00	-0.1	9.0E-01	0.0	9.9E-01
11767	ORAI1	A_24_P146892	NM_032790	0.8	6.09E-03	0.8	8.71E-03	1.3	6.09E-03	0.8	4.21E-03	0.9	2.70E-02	0.7	5.73E-02	1.0	2.70E-02	0.9	5.98E-03	0.0	1.0E+00	-0.1	1.0E+00	0.2	7.5E-01	0.0	1.0E+00	0.2	4.0E-01	-0.1	1.0E+00	0.2	2.9E-01	0.3	4.0E-01
13921	ASRGL1	A_23_P1936	NM_025080	0.8	1.60E-02	0.7	2.45E-02	1.0	1.60E-02	0.8	1.33E-03	0.7	2.03E-01	0.5	2.80E-01	0.7	4.44E-02	0.7	2.03E-01	0.0	1.0E+00	0.0	1.0E+00	-0.1	7.4E-01	0.0	1.0E+00	-0.1	9.5E-01	0.0	1.0E+00	-0.2	7.5E-01	-0.1	9.5E-01
29031	C17orf90	A_24_P45160	NM_001039842	0.8	3.22E-03	0.8	3.22E-03	1.1	6.02E-03	0.7	2.43E-03	0.6	1.59E-01	0.5	1.82E-01	0.6	1.59E-01	0.6	9.54E-02	-0.1	1.0E+00	-0.1	1.0E+00	-0.1	8.7E-01	-0.2	1.0E+00	0.1	9.7E-01	0.2	1.0E+00	0.0	9.7E-01	0.1	9.6E-01
11281	FAM150B	A_24_P561341	NM_001002919	0.8	3.99E-03	0.8	7.70E-03	0.8	3.99E-03	0.7	6.67E-04	0.9	7.01E-03	0.9	7.01E-03	0.7	5.38E-02	1.0	4.76E-04	0.1	1.0E+00	0.1	1.0E+00	0.0	9.5E-01	0.1	1.0E+00	-0.2	4.6E-01	0.0	1.0E+00	-0.2	2.9E-01	-0.2	4.6E-01
6562	DAB2	A_23_P257871	NM_001343	0.8	1.55E-02	0.9	1.55E-02	0.6	3.02E-02	0.8	4.82E-04	0.6	4.64E-01	0.5	7.18E-01	0.6	4.64E-01	0.6	4.08E-01	0.1	1.0E+00	0.0	1.0E+00	0.3	7.0E-01	0.1	1.0E+00	-0.1	9.5E-01	-0.1	1.0E+00	0.0	9.5E-01	-0.1	9.2E-01
24576	FLJ11235	A_23_P69941	AK002097	0.8	1.51E-03	0.7	1.51E-03	1.0	2.42E-03	0.8	1.00E-04	0.9	4.62E-02	0.9	4.62E-02	0.9	5.06E-02	0.8	2.25E-02	0.1	1.0E+00	0.1	1.0E+00	-0.2	7.9E-01	0.2	1.0E+00	-0.2	7.4E-01	-0.1	1.0E+00	-0.4	3.0E-01	-0.2	7.4E-01
26983	PTGER4	A_23_P148047	NM_000958	0.8	3.52E-02	0.8	4.63E-02	0.8	3.52E-02	1.0	1.07E-03	0.7	2.10E-01	0.8	2.10E-01	0.5	3.73E-01	0.7	1.10E-01	0.0	1.0E+00	0.0	1.0E+00	-0.1	8.1E-01	0.1	1.0E+00	-0.4	7.5E-01	-0.2	1.0E+00	-0.4	5.8E-01	-0.4	7.5E-01
33886	PEX13	A_23_P257131	NM_002618	0.8	2.29E-02	0.7	2.29E-02	0.9	4.80E-02	0.8	5.61E-03	0.7	6.96E-02	0.7	1.08E-01	0.7	5.07E-02	0.6	6.96E-02	0.1	1.0E+00	0.0	1.0E+00	0.1	8.9E-01	0.1	1.0E+00	-0.3	3.1E-01	-0.1	1.0E+00	-0.3	1.8E-01	-0.4	3.1E-01
40418	TMEM200B	A_32_P77098	NM_001003682	0.8	6.72E-03	0.7	1.51E-02	0.8	6.72E-03	1.2	1.33E-05	0.9	2.07E-02	0.8	2.07E-02	0.9	8.56E-02	1.0	4.81E-03	0.2	1.0E+00	0.2	1.0E+00	0.0	9.8E-01	0.2	1.0E+00	-0.2	5.6E-01	-0.2	1.0E+00	-0.3	2.0E-01	-0.2	5.6E-01
20186	HLA-A	A_23_P408353	NM_002116	0.8	2.20E-02	0.8	1.12E-02	0.9	4.13E-02	0.6	2.20E-02	0.3	4.40E-01	0.4	4.40E-01	0.2	7.98E-01	0.3	3.65E-01	-0.1	1.0E+00	-0.1	1.0E+00	0.0	9.9E-01	-0.1	1.0E+00	0.1	9.7E-01	0.1	1.0E+00	0.4	4.6E-01	0.1	9.7E-01
2994	ADM	A_23_P127948	NM_001124	0.8	9.79E-03	0.8	9.79E-03	0.8	3.85E-02	1.1	2.53E-04	0.4	3.19E-01	0.4	3.19E-01	0.3	4.56E-01	0.6	6.82E-02	0.1	1.0E+00	0.1	1.0E+00	0.1	8.9E-01	0.1	1.0E+00	-0.2	3.9E-01	-0.2	1.0E+00	-0.2	3.9E-01	-0.3	3.8E-01
33051	THC2578493	A_32_P158524	THC2578493	0.8	1.00E-03	0.7	3.65E-02	1.7	5.96E-04	0.8	1.00E-03	0.7	1.99E-01	0.6	1.99E-01	1.5	2.84E-01	0.7	7.55E-02	-0.2	1.0E+00	-0.2	1.0E+00	-0.2	7.4E-01	-0.1	1.0E+00	-0.3	8.5E-01	0.0	1.0E+00	-0.4	4.4E-01	-0.3	8.5E-01
35244	LOC90835	A_24_P842006	NM_001014979	0.8	5.17E-03	1.1	5.17E-03	0.8	2.04E-02	0.6	4.27E-03	1.0	7.89E-02	1.1	8.52E-02	0.8	7.89E-02	1.0	2.29E-02	0.2	1.0E+00	0.2	1.0E+00	0.0	9.8E-01	0.2	1.0E+00	-0.2	8.3E-01	0.1	1.0E+00	-0.2	6.0E-01	-0.2	8.3E-01
20104	DEFA4	A_23_P326080	NM_001925	0.8	3.65E-04	0.9	3.65E-04	0.8	2.87E-03	0.6	1.10E-04	0.4	1.04E-01	0.4	1.61E-01	0.4	1.04E-01	0.4	6.01E-02	-0.1	1.0E+00	-0.1	1.0E+00	0.0	9.0E-01	-0.2	1.0E+00	0.0	9.5E-01	0.0	1.0E+00	0.1	9.2E-01	-0.1	9.5E-01
44481	LOC100128737	A_32_P100430	XM_001715932	0.8	2.16E-03	0.8	2.16E-03	0.9	6.29E-03	0.8	2.17E-04	0.5	1.70E-01	0.4	3.60E-01	0.5	1.70E-01	0.7	1.53E-02	0.0	1.0E+00	0.0	1.0E+00	0.0	9.4E-01	-0.1	1.0E+00	-0.1	9.4E-01	0.1	1.0E+00	-0.2	5.7E-01	-0.1	9.4E-01
24046	ENST00000390610	A_24_P860662	ENST00000390610	0.8	1.23E-03	0.6	2.02E-02	1.4	1.23E-03	0.8	4.26E-04	0.6	8.19E-02	0.3	1.93E-01	1.0	8.19E-02	0.6	4.11E-03	0.2	8.0E-01	0.2	1.0E+00	-0.1	8.0E-01	0.4	6.6E-01	-0.5	7.2E-01	-0.4	1.0E+00	-0.5	4.0E-01	-0.5	7.2E-01
30895	GRB2	A_23_P77847	NM_002086	0.8	1.61E-02	0.7	1.61E-02	0.8	3.80E-02	0.8	2.51E-03	0.8	9.53E-03	0.9	9.53E-03	0.6	2.93E-02	0.8	1.90E-03	0.2	8.2E-01	0.3	8.2E-01	0.2	7.1E-01	0.2	1.0E+00	0.2	5.9E-01	0.1	1.0E+00	0.2	5.0E-01	0.2	5.9E-01
39063	RGL3	A_23_P107903	ENST00000380456	0.8	4.94E-04	0.8	4.94E-04	1.0	1.84E-03	0.8	1.32E-04	0.7	3.27E-02	0.9	3.27E-02	0.7	1.70E-02	0.7	5.71E-02	0.3	1.0E+00	0.3	1.0E+00	0.1	9.0E-01	0.3	1.0E+00	0.0	9.9E-01	-0.1	1.0E+00	0.1	8.8E-01	0.0	9.9E-01
2339	CU450678	A_32_P176911	CU450678	0.8	5.89E-03	0.6	5.89E-03	0.8	8.10E-03	1.1	3.11E-05	0.6	5.34E-02	0.5	8.94E-02	0.6	5.34E-02	0.8	1.57E-02	0.0	1.0E+00	0.0	1.0E+00	-0.2	7.5E-01	0.1	1.0E+00	-0.2	8.9E-01	-0.1	1.0E+00	-0.2	6.4E-01	-0.2	8.9E-01
26004	S1PR4	A_23_P119502	NM_003775	0.8	7.56E-03	0.8	1.01E-02	1.5	4.06E-03	0.8	7.56E-03	0.0	8.91E-01	-0.1	8.91E-01	0.5	5.26E-01	0.0	9.48E-01	-0.1	1.0E+00	-0.1	1.0E+00	0.1	9.5E-01	-0.2	1.0E+00	0.3	5.3E-01	0.1	1.0E+00	0.3	5.3E-01	0.4	5.2E-01
10868	GGT7	A_23_P325093	NM_178026	0.8	1.95E-02	0.8	1.95E-02	1.2	2.20E-02	0.7	9.76E-03	0.8	1.58E-02	0.8	3.86E-02	0.8	1.58E-02	1.0	1.67E-03	0.0	1.0E+00	0.0	1.0E+00	-0.1	8.1E-01	0.0	1.0E+00	0.2	8.3E-01	0.3	1.0E+00	0.2	6.3E-01	0.2	8.3E-01
5570	MUC13	A_23_P155236	NM_033049	0.8	5.13E-03	0.8	8.12E-03	0.8	5.13E-03	1.4	4.16E-06	0.5	1.32E-01	0.5	1.32E-01	0.4	4.91E-01	0.7	1.05E-02	0.1	1.0E+00	0.1	1.0E+00	0.1	8.3E-01	0.1	1.0E+00	-0.1	6.4E-01	-0.1	1.0E+00	-0.1	3.5E-01	-0.1	6.4E-01
28690	PKNOX1	A_23_P211299	NM_004571	0.8	8.80E-03	0.8	8.10E-03	1.1	2.30E-02	0.7	8.80E-03	1.0	2.83E-02	1.0	2.83E-02	1.4	3.08E-02	0.7	2.11E-02	0.1	1.0E+00	0.0	1.0E+00	0.1	8.5E-01	0.1	1.0E+00	0.0	9.7E-01	0.1	1.0E+00	0.0	9.7E-01	-0.1	9.2E-01
44836	CYP2F1	A_23_P89981	NM_000774	0.8	1.18E-03	0.7	1.18E-03	0.9	4.86E-03	0.8	1.27E-04	0.1	8.80E-01	-0.2	8.80E-01	0.2	8.01E-01	0.1	9.04E-01	0.0	1.0E+00	0.0	1.0E+00	0.1	8.7E-01	-0.2	1.0E+00	0.2	6.7E-01	0.0	1.0E+00	0.2	4.5E-01	0.2	6.7E-01
10447	BF213738	A_23_P76480	BF213738	0.8	4.11E-03	1.1	1.81E-02	0.8	4.11E-03	0.8	8.03E-05	0.7	1.07E-02	0.7	4.96E-02	0.7	1.07E-02	0.8	9.28E-03	0.0	1.0E+00	0.0	1.0E+00	0.0	9.6E-01	0.0	1.0E+00	-0.1	8.6E-01	0.0	1.0E+00	-0.1	8.0E-01	-0.1	8.6E-01
9196	BU602485	A_32_P4433	BU602485	0.8	8.77E-03	0.8	4.59E-02	0.9	8.77E-03	0.6	1.96E-03	1.0	2.10E-02	1.0	5.17E-02	1.0	2.10E-02	0.9	1.94E-02	0.0	1.0E+00	0.0	1.0E+00	-0.1	7.9E-01	0.2	1.0E+00	-0.4	5.0E-01	-0.1	1.0E+00	-0.4	2.7E-01	-0.4	5.0E-01
31679	A_24_P513764	A_24_P513764	A_24_P513764	0.8	2.30E-02	0.8	2.30E-02	1.0	7.47E-03	0.6	4.97E-02	0.5	6.38E-01	-0.2	9.03E-01	1.2	4.64E-01	0.5	6.38E-01	-0.1	1.0E+00	-0.1	1.0E+00	0.2	8.8E-01	-0.2	1.0E+00	0.3	5.9E-01	0.0	1.0E+00	0.3	5.9E-01	0.7	9.8E-02
6599	MPP2	A_23_P394259	NM_005374	0.8	1.34E-03	0.8	1.34E-03	0.8	7.36E-03	0.8	6.19E-04	0.8	2.28E-01	0.8	2.28E-01	0.6	5.24E-01	0.9	6.57E-02	0.1	1.0E+00	0.1	1.0E+00	0.1	9.1E-01	0.2	1.0E+00	-0.1	8.4E-01	-0.1	1.0E+00	-0.2	5.6E-01	-0.1	8.4E-01
15443	CLCNKA	A_24_P2361	NM_004070	0.8	6.83E-03	0.7	1.44E-02	0.9	6.83E-03	0.8	1.80E-03	0.8	4.49E-02	0.8	4.49E-02	0.2	5.49E-01	0.8	2.78E-02	0.0	1.0E+00	0.0	1.0E+00	0.4	7.1E-01	-0.2	1.0E+00	0.2	6.8E-01	0.0	1.0E+00	0.3	8.1E-02	0.2	6.8E-01
18302	ZC3HAV1	A_23_P20122	NM_024625	0.8	8.18E-04	0.8	2.35E-02	1.8	8.18E-04	0.8	5.16E-04	0.6	1.03E-01	0.9	4.26E-02	0.6	1.03E-01	0.4	1.51E-01	0.0	1.0E+00	0.0	1.0E+00	-0.1	8.3E-01	0.1	1.0E+00	-0.1	9.1E-01	0.0	1.0E+00	-0.2	5.6E-01	-0.1	9.1E-01
22876	GPR4	A_23_P357284	NM_005282	0.8	3.13E-03	0.8	6.22E-03	0.9	3.13E-03	0.7	9.15E-04	0.4	5.65E-01	0.4	5.65E-01	0.3	5.75E-01	0.8	1.28E-01	0.1	1.0E+00	0.1	1.0E+00	0.0	1.0E+00	0.1	1.0E+00	0.0	9.8E-01	0.0	1.0E+00	0.0	9.8E-01	0.0	9.7E-01
24221	USP11	A_23_P171366	NM_004651	0.8	5.64E-03	0.7	5.64E-03	0.9	6.41E-03	0.8	2.43E-04	0.7	2.17E-02	0.4	1.81E-01	0.7	2.17E-02	0.8	1.25E-02	0.2	1.0E+00	0.2	1.0E+00	0.2	7.1E-01	0.1	1.0E+00	0.0	9.7E-01	0.0	1.0E+00	0.0	9.7E-01	0.2	8.6E-01
10451	CN364769	A_32_P55344	CN364769	0.8	2.46E-03	0.8	2.46E-03	0.9	8.45E-03	0.8	3.13E-04	0.6	1.13E-01	0.6	1.13E-01	0.5	1.47E-01	0.6	5.81E-02	-0.1	1.0E+00	-0.1	1.0E+00	-0.1	8.6E-01	0.0	1.0E+00	-0.1	9.1E-01	0.0	1.0E+00	-0.2	6.5E-01	-0.1	9.1E-01
15886	JARID1B	A_32_P70724	NM_006618	0.8	6.93E-03	0.8	1.43E-02	1.0	6.93E-03	0.8	5.79E-04	0.7	2.59E-02	0.7	2.59E-02	0.6	4.17E-02	0.7	1.31E-02	-0.2	7.1E-01	-0.2	5.4E-01	-0.2	7.1E-01	-0.1	1.0E+00	-0.2	9.1E-01	-0.3	1.0E+00	-0.2	7.0E-01	-0.2	9.1E-01
33860	ITIH3	A_23_P6822	NM_002217	0.8	2.19E-03	0.8	2.96E-02	1.5	2.19E-03	0.7	2.15E-03	0.6	1.76E-01	0.4	4.13E-01	0.7	1.69E-01	0.6	1.76E-01	0.0	1.0E+00	0.0	1.0E+00	-0.2	7.1E-01	0.0	1.0E+00	-0.1	8.8E-01	-0.1	1.0E+00	-0.2	3.4E-01	-0.1	8.8E-01
22439	GRN	A_23_P49708	NM_002087	0.8	3.30E-03	0.7	3.30E-03	0.8	1.04E-02	0.9	1.81E-04	0.6	3.36E-02	0.6	3.36E-02	0.5	1.80E-01	0.9	3.10E-03	0.1	1.0E+00	0.2	1.0E+00	0.1	7.4E-01	0.1	1.0E+00	-0.1	9.0E-01	0.0	1.0E+00	-0.1	9.0E-01	-0.1	8.9E-01
33826	AIM1L	A_23_P360329	NM_001039775	0.8	2.70E-03	0.7	1.05E-02	1.6	2.70E-03	0.8	1.24E-03	0.6	7.09E-02	0.3	4.65E-01	0.8	7.09E-02	0.6	2.01E-02	0.2	1.0E+00	0.2	1.0E+00	0.1	8.3E-01	0.2	1.0E+00	-0.2	9.0E-01	0.0	1.0E+00	-0.2	8.0E-01	-0.2	9.0E-01
13498	SAT1	A_23_P378722	NM_002970	0.8	1.08E-02	0.8	1.08E-02	0.9	3.85E-02	0.8	3.68E-03	0.9	1.68E-02	1.0	1.06E-02	0.3	6.39E-01	0.9	1.68E-02	0.0	1.0E+00	0.0	1.0E+00	0.0	9.6E-01	0.1	1.0E+00	-0.1	9.5E-01	-0.3	1.0E+00	0.0	9.5E-01	-0.1	9.1E-01
4376	THC2773657	A_23_P42595	THC2773657	0.8	4.14E-03	0.7	3.30E-02	1.1	4.14E-03	0.8	1.93E-03	0.6	5.33E-02	0.6	2.03E-01	0.7	5.33E-02	0.6	4.98E-02	0.1	1.0E+00	0.2	1.0E+00	0.0	1.0E+00	0.1	1.0E+00	-0.2	3.5E-01	0.0	1.0E+00	-0.2	2.1E-01	-0.3	3.5E-01
8815	RABAC1	A_23_P38864	NM_006423	0.8	1.87E-03	0.8	3.74E-03	1.4	1.87E-03	0.7	1.31E-03	0.9	2.28E-02	0.9	2.28E-02	1.1	2.56E-02	0.9	7.72E-03	0.0	1.0E+00	0.0	1.0E+00	0.0	9.1E-01	0.0	1.0E+00	0.0	9.8E-01	0.1	1.0E+00	-0.1	7.2E-01	0.0	9.8E-01
26835	ENST00000312015	A_24_P220495	ENST00000312015	0.8	2.43E-03	0.6	1.03E-02	1.2	2.43E-03	0.8	2.14E-04	0.2	7.69E-01	-0.1	9.16E-01	0.3	6.91E-01	0.2	7.69E-01	-0.1	1.0E+00	-0.2	1.0E+00	-0.1	9.2E-01	-0.1	1.0E+00	0.2	5.4E-01	0.2	1.0E+00	0.2	5.1E-01	0.3	5.4E-01
17605	FNDC4	A_23_P16834	NM_022823	0.8	8.43E-03	0.7	2.50E-02	1.4	8.43E-03	0.8	4.22E-03	0.9	1.46E-01	0.9	2.12E-01	1.0	1.46E-01	0.9	1.01E-01	0.2	1.0E+00	0.2	1.0E+00	0.2	7.1E-01	0.4	1.0E+00	-0.1	9.8E-01	-0.1	1.0E+00	-0.1	8.0E-01	0.0	9.8E-01
44487	A_24_P709844	A_24_P709844	A_24_P709844	0.8	1.67E-03	0.8	3.97E-04	1.0	1.67E-03	0.7	1.79E-03	0.1	9.21E-01	-0.1	9.57E-01	0.3	7.68E-01	0.1	9.21E-01	0.0	1.0E+00	0.0	1.0E+00	0.3	7.2E-01	-0.1	1.0E+00	0.1	9.2E-01	0.1	1.0E+00	0.1	8.7E-01	0.1	9.2E-01
15998	CIB3	A_23_P130585	NM_054113	0.8	9.98E-04	0.8	1.69E-02	1.6	9.98E-04	0.8	5.91E-04	0.4	2.73E-01	0.3	4.67E-01	0.8	2.73E-01	0.4	4.55E-02	0.2	1.0E+00	0.2	1.0E+00	0.4	7.1E-01	-0.2	1.0E+00	0.3	3.4E-01	0.2	1.0E+00	0.3	3.4E-01	0.4	2.4E-01
37838	AK093229	A_32_P486693	AK093229	0.8	2.92E-03	0.8	6.74E-03	0.8	2.92E-03	1.1	8.54E-06	0.7	8.08E-02	0.7	8.08E-02	0.7	1.89E-01	1.1	2.53E-03	0.0	1.0E+00	0.1	1.0E+00	0.0	9.4E-01	0.0	1.0E+00	-0.1	9.4E-01	-0.1	1.0E+00	-0.1	8.0E-01	-0.1	9.4E-01
26557	LGALS2	A_23_P120902	NM_006498	0.8	1.82E-03	0.8	8.94E-03	0.9	1.82E-03	0.8	3.51E-05	0.4	2.11E-01	0.2	4.25E-01	0.4	1.69E-01	0.4	2.11E-01	0.0	1.0E+00	0.0	1.0E+00	-0.1	7.4E-01	0.0	1.0E+00	0.0	8.4E-01	0.0	1.0E+00	0.1	6.4E-01	-0.1	8.4E-01
27689	THC2692456	A_32_P3932	THC2692456	0.8	7.41E-04	0.7	1.45E-02	1.6	7.41E-04	0.8	6.53E-04	1.2	3.79E-02	1.2	2.30E-02	1.2	3.79E-02	0.9	3.95E-02	0.1	1.0E+00	0.1	1.0E+00	0.0	9.6E-01	0.3	1.0E+00	-0.2	6.0E-01	-0.1	1.0E+00	-0.2	3.3E-01	-0.2	6.0E-01
37268	TGFB3	A_23_P88404	NM_003239	0.8	2.33E-03	0.6	6.35E-03	1.2	2.33E-03	0.8	4.50E-04	0.7	1.08E-01	0.8	1.08E-01	0.5	2.54E-01	0.7	3.42E-02	0.0	1.0E+00	0.1	1.0E+00	-0.2	7.1E-01	0.0	1.0E+00	-0.3	5.5E-01	0.0	1.0E+00	-0.3	1.2E-01	-0.3	5.5E-01
33219	DSE	A_23_P19226	NM_013352	0.8	8.43E-03	0.9	1.91E-02	0.8	8.43E-03	0.7	3.41E-03	0.7	4.58E-02	1.3	6.18E-03	0.2	7.60E-01	0.7	4.58E-02	0.2	1.0E+00	0.2	1.0E+00	-0.4	7.1E-01	0.2	1.0E+00	-0.2	9.8E-01	-0.2	1.0E+00	-0.4	3.6E-01	0.0	9.8E-01
14952	ZNHIT1	A_23_P111843	NM_006349	0.8	3.69E-03	0.8	8.43E-03	1.1	3.69E-03	0.7	5.63E-04	0.7	8.56E-02	0.8	2.34E-02	0.7	1.60E-01	0.5	8.56E-02	-0.1	1.0E+00	0.1	1.0E+00	-0.1	8.2E-01	-0.1	1.0E+00	0.0	9.9E-01	0.0	1.0E+00	-0.1	8.8E-01	0.0	9.9E-01
27916	STARD5	A_23_P158880	NM_181900	0.8	2.39E-03	1.1	2.39E-03	0.8	2.15E-02	0.8	4.77E-04	0.8	3.38E-01	0.8	3.92E-01	0.8	3.38E-01	0.9	2.05E-01	0.1	1.0E+00	0.1	1.0E+00	0.0	9.2E-01	0.2	1.0E+00	-0.1	9.2E-01	0.0	1.0E+00	-0.1	9.2E-01	-0.2	8.8E-01
37448	THC2691500	A_32_P100464	THC2691500	0.8	7.77E-03	0.9	7.77E-03	0.8	4.85E-02	0.8	5.34E-03	0.9	6.94E-02	0.5	2.35E-01	1.1	6.94E-02	0.9	1.26E-02	0.0	1.0E+00	0.1	1.0E+00	-0.1	9.2E-01	0.0	1.0E+00	-0.1	9.6E-01	0.1	1.0E+00	-0.1	9.1E-01	-0.1	9.6E-01
4428	UTS2D	A_32_P225659	NM_198152	0.8	8.43E-03	0.8	2.17E-02	0.8	8.43E-03	1.6	6.39E-06	0.7	3.98E-02	0.5	1.23E-01	0.7	3.98E-02	0.7	2.04E-03	0.0	1.0E+00	0.0	1.0E+00	0.0	9.6E-01	0.0	1.0E+00	0.0	9.6E-01	-0.1	1.0E+00	0.0	9.6E-01	0.0	9.6E-01
38533	HIST3H2BB	A_32_P443407	AK091220	0.8	1.71E-03	1.1	1.71E-03	0.8	1.98E-02	0.8	8.49E-04	1.4	3.72E-03	1.8	3.72E-03	1.4	4.54E-03	1.2	1.55E-03	0.4	4.5E-01	0.4	2.5E-01	0.2	7.1E-01	0.6	4.5E-01	-0.2	8.8E-01	0.0	1.0E+00	-0.3	4.1E-01	-0.2	8.8E-01
17982	C1orf106	A_23_P1043	NM_018265	0.8	4.45E-03	0.7	4.45E-03	1.0	9.96E-03	0.8	4.67E-04	0.6	1.63E-01	0.6	1.63E-01	0.5	2.13E-01	0.6	7.77E-02	0.0	1.0E+00	0.0	1.0E+00	-0.2	7.1E-01	0.0	1.0E+00	-0.1	7.4E-01	-0.1	1.0E+00	-0.1	5.4E-01	-0.1	7.4E-01
10711	ARPM1	A_23_P121447	NM_032487	0.8	1.50E-03	1.0	1.29E-03	0.6	4.89E-02	0.8	1.50E-03	0.9	7.92E-02	0.9	7.92E-02	0.7	1.63E-01	1.1	2.02E-02	0.1	1.0E+00	0.1	1.0E+00	0.0	9.8E-01	0.1	1.0E+00	-0.2	5.4E-01	0.1	1.0E+00	-0.2	4.5E-01	-0.3	5.4E-01
11762	RDH5	A_24_P218814	NM_002905	0.8	1.54E-03	0.6	1.55E-02	1.8	1.54E-03	0.8	9.19E-04	0.8	1.73E-02	0.6	1.80E-01	1.0	1.61E-02	0.8	1.73E-02	0.0	1.0E+00	0.1	1.0E+00	0.0	9.5E-01	0.0	1.0E+00	0.1	9.7E-01	0.1	1.0E+00	0.0	9.7E-01	0.1	9.0E-01
26643	GPR35	A_23_P154245	NM_005301	0.8	3.96E-02	0.8	3.96E-02	1.1	4.48E-02	0.7	2.55E-02	1.0	1.38E-01	1.2	1.38E-01	0.8	2.07E-01	1.0	5.14E-02	0.1	1.0E+00	0.2	1.0E+00	-0.2	8.2E-01	0.1	1.0E+00	0.0	9.9E-01	0.1	1.0E+00	0.0	9.9E-01	-0.1	9.1E-01
43539	TCEA3	A_24_P327886	NM_003196	0.8	1.65E-02	0.8	4.80E-03	1.0	1.78E-02	0.6	1.65E-02	1.1	2.71E-03	1.1	2.71E-03	1.3	2.15E-02	1.0	9.54E-04	0.2	1.0E+00	0.2	1.0E+00	0.2	7.1E-01	0.2	1.0E+00	0.1	9.8E-01	0.2	1.0E+00	0.0	9.8E-01	0.1	9.3E-01
22895	ARID5B	A_23_P97871	NM_032199	0.8	4.76E-03	0.8	4.76E-03	1.0	1.21E-02	0.7	3.80E-03	0.7	2.56E-01	0.5	3.67E-01	1.1	2.56E-01	0.7	1.04E-01	-0.2	1.0E+00	-0.1	1.0E+00	-0.2	7.4E-01	-0.2	1.0E+00	0.0	9.3E-01	0.0	1.0E+00	0.1	7.3E-01	-0.1	9.3E-01
23929	A_24_P307395	A_24_P307395	A_24_P307395	0.8	6.97E-03	0.9	6.97E-03	0.8	3.17E-02	0.8	2.64E-03	0.7	1.11E-01	0.7	1.11E-01	0.3	7.35E-01	0.8	2.87E-02	0.0	1.0E+00	0.0	1.0E+00	-0.3	7.1E-01	0.0	1.0E+00	-0.1	8.8E-01	-0.1	1.0E+00	-0.1	7.0E-01	-0.1	8.8E-01
16275	PADI2	A_24_P205604	NM_007365	0.8	1.07E-02	0.8	2.83E-02	0.8	1.07E-02	0.9	1.18E-04	0.6	4.10E-02	0.6	4.10E-02	0.5	7.28E-02	0.7	1.48E-02	0.0	1.0E+00	0.0	1.0E+00	0.2	7.7E-01	-0.1	1.0E+00	0.0	9.5E-01	0.0	1.0E+00	0.1	8.1E-01	0.0	9.5E-01
4867	THC2528427	A_32_P91507	THC2528427	0.8	7.66E-03	1.4	7.66E-03	0.8	1.30E-02	0.8	1.25E-03	1.4	2.92E-02	1.5	3.56E-02	1.4	2.77E-02	1.1	2.92E-02	-0.1	1.0E+00	-0.1	1.0E+00	-0.2	7.6E-01	0.2	1.0E+00	-0.5	3.1E-01	0.0	1.0E+00	-0.5	7.4E-02	-0.5	3.1E-01
24107	FAM113A	A_23_P17471	NM_022760	0.8	6.91E-03	0.8	6.91E-03	1.0	1.79E-02	0.8	3.18E-03	0.3	7.76E-01	0.2	9.29E-01	0.3	7.76E-01	0.6	5.52E-01	0.0	1.0E+00	0.0	1.0E+00	0.0	9.4E-01	-0.1	1.0E+00	0.3	3.7E-01	0.2	1.0E+00	0.4	6.2E-02	0.3	3.7E-01
42709	C11orf16	A_23_P13482	NM_020643	0.8	8.31E-03	0.7	1.16E-02	0.8	8.31E-03	1.0	1.19E-03	0.3	8.30E-01	-0.3	8.80E-01	0.5	7.26E-01	0.3	8.30E-01	0.0	1.0E+00	0.0	1.0E+00	0.8	7.1E-01	0.0	1.0E+00	0.2	8.4E-01	0.1	1.0E+00	0.2	8.4E-01	0.4	8.2E-01
14433	IFI16	A_23_P217866	NM_005531	0.8	6.87E-04	0.8	7.74E-03	1.3	6.45E-04	0.8	6.87E-04	0.5	2.90E-01	0.4	5.00E-01	0.5	2.60E-01	0.5	2.90E-01	0.1	1.0E+00	0.1	1.0E+00	-0.1	8.7E-01	0.2	1.0E+00	-0.2	5.7E-01	-0.1	1.0E+00	-0.2	4.1E-01	-0.2	5.7E-01
42646	ANKRD42	A_32_P69166	NM_182603	0.8	1.35E-02	0.8	2.37E-02	1.3	5.31E-03	0.8	1.35E-02	0.9	9.78E-03	0.9	2.43E-02	1.0	9.78E-03	0.8	8.07E-03	-0.1	1.0E+00	-0.1	1.0E+00	-0.3	7.4E-01	-0.1	1.0E+00	-0.2	2.1E-01	-0.1	1.0E+00	-0.2	2.1E-01	-0.5	1.1E-01
20183	MYH13	A_23_P89334	NM_003802	0.8	4.54E-03	0.6	1.06E-02	1.0	4.54E-03	0.8	9.08E-04	0.3	5.59E-01	0.1	8.72E-01	0.3	5.59E-01	0.3	3.74E-01	0.0	1.0E+00	0.0	1.0E+00	0.0	9.6E-01	0.0	1.0E+00	-0.1	8.5E-01	0.1	1.0E+00	-0.1	7.4E-01	-0.1	8.5E-01

Feature	Gene symbol	Probe	Transcript	72 hour								48 hours								24 hours								10 hours							
				siCASP8AP2								siCASP8AP2								siCASP8AP2								siCASP8AP2							
				Median M	Median q	1 M	1 q	3 M	3 q	6 M	6 q	Median M	Median q	1 M	1 q	3 M	3 q	6 M	6 q	Median M	Median q	1 M	1 q	3 M	3 q	6 M	6 q	Median M	Median q	1 M	1 q	3 M	3 q	6 M	6 q
12824	PHF15	A_24_P226278	NM_015288	0.8	2.17E-02	0.8	2.17E-02	1.1	2.66E-02	0.8	7.02E-03	0.6	6.58E-02	0.6	6.58E-02	0.4	2.69E-01	0.7	1.21E-02	-0.1	1.0E+00	0.1	1.0E+00	-0.1	8.0E-01	-0.2	1.0E+00	0.1	8.8E-01	0.1	1.0E+00	0.1	8.1E-01	-0.2	8.8E-01
43238	C11orf86	A_32_P524904	NM_001136485	0.8	2.37E-03	0.8	7.68E-03	1.0	2.37E-03	0.8	9.06E-05	0.2	6.51E-01	0.2	8.15E-01	0.2	6.51E-01	0.3	3.81E-01	0.0	1.0E+00	0.0	1.0E+00	0.1	7.3E-01	-0.1	1.0E+00	0.1	6.9E-01	0.0	1.0E+00	0.1	5.2E-01	0.2	6.9E-01
28257	PRKCD	A_23_P144054	NM_006254	0.8	5.06E-03	0.7	1.30E-02	1.2	5.06E-03	0.8	1.13E-03	0.5	2.73E-01	0.5	2.73E-01	0.3	6.36E-01	0.7	5.58E-02	0.2	1.0E+00	0.3	1.0E+00	0.1	7.3E-01	0.2	1.0E+00	0.1	8.8E-01	0.0	1.0E+00	0.1	8.3E-01	0.2	8.8E-01
1253	C19orf28	A_23_P218523	NM_021731	0.8	4.61E-03	0.7	4.61E-03	1.0	1.23E-02	0.8	6.58E-04	0.5	1.69E-02	0.5	1.28E-01	0.7	1.69E-02	0.5	1.60E-02	0.1	1.0E+00	0.2	1.0E+00	0.0	8.9E-01	0.1	1.0E+00	0.0	9.8E-01	0.2	1.0E+00	0.0	9.2E-01	0.0	9.8E-01
27181	hCG_2008140	A_32_P100258	NR_024279	0.8	1.75E-02	0.8	1.75E-02	1.0	2.47E-02	0.7	6.59E-03	0.6	2.85E-02	1.0	1.18E-02	0.6	2.85E-02	0.6	3.91E-02	0.1	1.0E+00	0.1	1.0E+00	-0.1	7.6E-01	0.1	1.0E+00	-0.2	8.5E-01	-0.1	1.0E+00	-0.2	6.8E-01	-0.2	8.5E-01
32597	LOC253264	A_24_P589001	AK023445	0.8	1.01E-02	0.8	1.03E-02	1.1	1.01E-02	0.8	1.15E-03	0.7	8.50E-02	0.9	8.50E-02	0.4	4.00E-01	0.7	4.44E-02	0.0	1.0E+00	0.0	1.0E+00	-0.2	7.2E-01	0.1	1.0E+00	-0.1	9.1E-01	-0.1	1.0E+00	-0.3	2.5E-01	-0.1	9.1E-01
15914	GPRC5C	A_23_P38167	NM_022036	0.8	3.17E-02	0.8	3.17E-02	1.0	4.43E-02	0.8	8.97E-03	0.7	2.06E-02	0.7	4.37E-02	0.7	2.06E-02	1.0	1.56E-02	0.1	1.0E+00	0.3	1.0E+00	0.1	7.6E-01	0.1	1.0E+00	-0.2	9.1E-01	0.0	1.0E+00	-0.2	8.4E-01	-0.2	9.1E-01
24552	SCARA5	A_23_P94103	NM_173833	0.8	2.83E-03	0.8	9.21E-03	1.0	2.83E-03	0.8	8.90E-05	0.3	3.16E-01	0.3	3.32E-01	0.3	3.16E-01	0.6	4.11E-02	0.0	1.0E+00	0.0	1.0E+00	0.1	7.6E-01	-0.1	1.0E+00	-0.1	9.3E-01	-0.2	1.0E+00	-0.1	9.0E-01	-0.1	9.3E-01
41070	SNCG	A_23_P251293	NM_003087	0.8	4.19E-04	0.8	4.74E-03	2.4	2.32E-04	0.8	4.19E-04	0.5	2.17E-01	0.3	4.87E-01	1.5	2.17E-01	0.5	1.01E-01	0.0	1.0E+00	0.1	1.0E+00	0.0	9.9E-01	-0.1	1.0E+00	-0.1	9.3E-01	-0.1	1.0E+00	-0.1	7.7E-01	-0.1	9.3E-01
24905	TMIE	A_24_P234462	NM_147196	0.8	7.41E-03	0.7	3.24E-02	1.9	4.61E-03	0.8	7.41E-03	0.7	1.47E-01	0.3	7.06E-01	1.7	1.47E-01	0.7	5.19E-02	0.1	1.0E+00	0.1	1.0E+00	0.0	9.9E-01	0.2	1.0E+00	-0.3	2.4E-01	0.0	1.0E+00	-0.3	1.9E-01	-0.3	2.4E-01
24569	CLDN5	A_24_P79300	NM_003277	0.8	5.98E-03	0.8	1.29E-03	0.9	6.15E-03	0.6	5.98E-03	0.4	6.57E-01	0.1	9.31E-01	0.4	6.45E-01	0.4	6.57E-01	0.0	1.0E+00	0.1	1.0E+00	0.0	9.7E-01	-0.3	1.0E+00	0.5	1.2E-01	-0.1	1.0E+00	0.5	8.4E-02	0.5	1.2E-01
21689	THC2732175	A_32_P50587	THC2732175	0.8	1.32E-03	0.8	4.15E-03	1.4	1.32E-03	0.8	2.43E-04	0.8	2.17E-02	0.8	4.19E-02	1.2	3.52E-03	0.7	2.17E-02	0.0	1.0E+00	0.0	1.0E+00	0.0	9.5E-01	0.2	1.0E+00	-0.1	8.7E-01	0.0	1.0E+00	-0.1	5.4E-01	-0.1	8.7E-01
38020	KRT33B	A_23_P89665	NM_002279	0.8	5.01E-04	0.8	1.03E-03	1.6	5.01E-04	0.8	1.37E-04	0.9	2.44E-02	0.7	7.40E-02	1.0	2.44E-02	0.9	1.28E-02	0.2	1.0E+00	0.2	1.0E+00	0.1	7.6E-01	0.2	1.0E+00	0.1	6.4E-01	0.0	1.0E+00	0.1	4.5E-01	0.1	6.4E-01
23396	C4orf12	A_32_P116271	NR_015359	0.8	2.07E-02	0.7	2.07E-02	0.9	3.96E-02	0.8	3.36E-03	0.6	1.45E-01	0.6	2.43E-01	1.0	3.99E-02	0.6	1.45E-01	0.1	1.0E+00	0.1	1.0E+00	-0.2	8.0E-01	0.1	1.0E+00	-0.2	8.5E-01	0.1	1.0E+00	-0.2	6.8E-01	-0.2	8.5E-01
42420	DDX58	A_23_P20814	NM_014314	0.8	1.92E-02	0.8	1.85E-02	1.3	1.92E-02	0.7	2.05E-02	0.7	8.40E-02	0.6	4.95E-02	0.9	8.40E-02	0.7	9.27E-02	0.0	1.0E+00	0.0	1.0E+00	-0.1	7.5E-01	0.0	1.0E+00	-0.3	7.9E-01	-0.2	1.0E+00	-0.4	4.3E-01	-0.3	7.9E-01
32884	HLA-F	A_23_P145264	NM_018950	0.8	3.07E-02	0.8	2.03E-02	1.1	3.35E-02	0.6	3.07E-02	0.5	2.35E-01	0.5	2.35E-01	0.1	8.77E-01	0.6	4.34E-02	0.1	1.0E+00	0.1	1.0E+00	0.1	7.7E-01	0.1	1.0E+00	0.1	9.5E-01	0.0	1.0E+00	0.1	6.6E-01	0.1	9.5E-01
21114	SLC25A29	A_23_P77049	NM_001039355	0.8	1.70E-03	1.0	1.05E-03	0.8	2.38E-02	0.8	1.70E-03	0.6	7.20E-02	0.6	7.82E-02	0.5	7.20E-02	0.7	1.39E-02	0.3	1.0E+00	0.2	1.0E+00	0.3	7.1E-01	0.3	1.0E+00	0.0	9.9E-01	0.0	1.0E+00	0.0	9.5E-01	0.0	9.9E-01
22818	TAP1	A_23_P59005	NM_000593	0.8	3.46E-03	0.8	3.46E-03	1.2	3.77E-03	0.8	4.95E-04	0.5	2.68E-01	0.7	1.63E-01	0.3	5.18E-01	0.5	2.68E-01	0.0	1.0E+00	0.2	1.0E+00	0.0	9.8E-01	-0.1	1.0E+00	0.1	8.9E-01	0.1	1.0E+00	0.1	8.9E-01	0.1	8.9E-01
12377	BEST2	A_23_P16225	NM_017682	0.8	1.54E-02	0.8	3.40E-02	1.8	3.11E-03	0.6	1.54E-02	0.5	5.06E-01	0.5	6.49E-01	1.1	5.06E-01	0.5	4.24E-01	-0.5	1.0E+00	-0.5	1.0E+00	0.4	7.6E-01	-0.6	1.0E+00	0.5	5.3E-01	0.2	1.0E+00	0.9	3.9E-02	0.5	5.3E-01
2750	AK123066	A_32_P153781	AK123066	0.8	6.47E-03	0.8	1.73E-02	1.1	6.47E-03	0.8	7.26E-04	0.9	2.27E-02	1.0	3.23E-02	0.9	2.27E-02	0.9	1.06E-02	0.2	1.0E+00	0.3	1.0E+00	0.0	9.7E-01	0.2	1.0E+00	-0.3	6.7E-01	0.0	1.0E+00	-0.3	4.0E-01	-0.3	6.7E-01
3864	LOC400684	A_24_P59569	BC000922	0.8	4.61E-03	0.6	3.46E-02	1.2	4.61E-03	0.8	5.19E-04	0.5	3.69E-01	0.5	4.68E-01	0.7	1.10E-01	0.4	3.69E-01	0.0	1.0E+00	0.0	1.0E+00	-0.3	7.1E-01	0.1	1.0E+00	-0.2	7.0E-01	-0.2	1.0E+00	-0.2	7.0E-01	-0.4	6.6E-01
31681	NPR1	A_23_P147711	NM_000906	0.8	1.71E-03	1.1	1.71E-03	0.8	3.78E-03	0.8	4.20E-05	0.8	1.98E-01	0.9	1.98E-01	0.4	2.80E-01	0.8	2.37E-02	0.1	1.0E+00	0.1	1.0E+00	0.1	8.0E-01	0.1	1.0E+00	0.1	9.1E-01	0.1	1.0E+00	0.0	9.1E-01	0.1	6.7E-01
7408	SPSB1	A_24_P96961	NM_025106	0.8	7.57E-03	0.8	7.57E-03	0.6	4.61E-02	1.0	1.77E-04	0.8	2.31E-02	0.6	1.05E-01	1.0	2.23E-02	0.8	2.31E-02	0.1	1.0E+00	0.1	1.0E+00	0.1	8.4E-01	0.3	1.0E+00	-0.2	7.8E-01	-0.2	1.0E+00	-0.2	6.5E-01	-0.2	7.8E-01
14782	ARHGEF18	A_23_P50357	NM_015318	0.9	4.35E-03	0.8	4.35E-03	0.9	1.23E-02	0.9	1.27E-03	0.6	2.07E-01	0.6	2.07E-01	0.4	2.99E-01	0.7	3.67E-02	0.2	1.0E+00	0.2	1.0E+00	0.1	8.4E-01	0.2	1.0E+00	0.0	9.9E-01	0.1	1.0E+00	0.0	9.9E-01	0.0	9.9E-01
22471	AF086154	A_24_P548297	AF086154	0.9	3.18E-03	0.9	3.30E-02	1.4	2.06E-03	0.6	3.18E-03	0.8	1.17E-01	0.8	1.11E-01	1.0	1.17E-01	0.7	1.40E-01	0.1	1.0E+00	0.2	1.0E+00	-0.1	7.4E-01	0.1	1.0E+00	-0.1	8.6E-01	0.1	1.0E+00	-0.2	5.6E-01	-0.1	8.6E-01
23649	ABTB1	A_24_P200297	NM_172027	0.9	1.22E-02	0.8	1.22E-02	1.0	2.47E-02	0.9	2.67E-03	1.0	1.18E-01	1.0	1.18E-01	0.6	5.09E-01	1.1	2.53E-02	0.3	9.5E-01	0.3	6.3E-01	0.0	9.5E-01	0.3	1.0E+00	0.0	1.0E+00	0.2	1.0E+00	-0.1	9.0E-01	0.0	1.0E+00
23149	TCIRG1	A_23_P75369	NM_006019	0.9	2.41E-03	0.9	1.85E-03	1.4	3.28E-03	0.7	2.41E-03	0.6	7.09E-02	0.6	2.09E-01	1.0	7.09E-02	0.6	6.63E-02	0.2	1.0E+00	0.3	1.0E+00	0.2	7.1E-01	0.1	1.0E+00	0.2	9.3E-01	0.2	1.0E+00	0.2	7.6E-01	0.1	9.3E-01
29683	FAM105A	A_32_P162066	NM_019018	0.9	1.57E-03	0.9	1.57E-03	0.8	1.25E-02	0.9	1.06E-04	1.2	1.87E-02	1.2	1.87E-02	1.3	2.99E-02	1.0	7.21E-03	0.3	1.0E+00	0.3	1.0E+00	0.0	9.7E-01	0.4	1.0E+00	-0.3	3.2E-01	0.0	1.0E+00	-0.3	1.7E-01	-0.4	3.2E-01
4472	ADM	A_23_P127948	NM_001124	0.9	5.16E-03	0.8	5.16E-03	0.9	3.53E-02	1.2	1.74E-04	0.4	1.84E-01	0.4	1.84E-01	0.4	1.84E-01	0.6	7.72E-02	0.0	1.0E+00	0.1	1.0E+00	0.0	1.0E+00	-0.1	1.0E+00	-0.1	8.0E-01	-0.1	1.0E+00	-0.1	7.2E-01	-0.1	8.0E-01
21266	BCL2L12	A_23_P50477	NM_138639	0.9	3.74E-04	0.9	3.74E-04	0.7	8.91E-03	0.9	4.33E-05	0.7	1.72E-02	0.7	1.72E-02	0.6	3.35E-02	0.8	1.72E-03	0.1	1.0E+00	0.1	1.0E+00	0.0	9.3E-01	0.1	1.0E+00	-0.1	9.6E-01	0.1	1.0E+00	-0.1	9.0E-01	-0.1	9.6E-01
38146	ENST00000399134	A_32_P141682	ENST00000399134	0.9	2.42E-03	0.9	4.97E-03	0.9	2.42E-03	0.7	1.32E-04	0.5	4.94E-02	0.6	5.44E-02	0.5	4.94E-02	0.5	3.00E-02	0.1	1.0E+00	0.0	1.0E+00	0.1	8.2E-01	0.1	1.0E+00	0.0	9.9E-01	0.1	1.0E+00	0.0	9.1E-01	0.0	9.9E-01
24941	SSX4B	A_23_P33881	NM_001034832	0.9	7.01E-03	0.6	1.48E-02	0.9	7.01E-03	1.1	6.67E-05	0.7	6.58E-02	0.6	6.58E-02	0.7	1.10E-01	0.8	1.72E-02	0.0	1.0E+00	0.1	1.0E+00	0.0	9.0E-01	0.0	1.0E+00	-0.1	7.1E-01	-0.1	1.0E+00	-0.2	2.7E-01	-0.1	7.1E-01
26954	ING1	A_24_P39211	NM_198219	0.9	5.58E-03	0.8	5.58E-03	1.1	1.07E-02	0.9	8.02E-04	0.4	3.28E-01	0.4	3.84E-01	0.4	3.28E-01	0.9	1.24E-02	0.0	1.0E+00	0.1	1.0E+00	0.0	8.9E-01	0.0	1.0E+00	-0.1	9.1E-01	0.1	1.0E+00	-0.1	8.3E-01	-0.1	9.1E-01
22158	FASLG	A_23_P369815	NM_000639	0.9	6.85E-03	0.6	8.33E-03	0.9	6.85E-03	1.1	2.12E-05	0.8	5.36E-02	0.8	5.36E-02	0.8	6.41E-02	1.0	5.39E-03	0.0	1.0E+00	0.0	1.0E+00	-0.1	7.1E-01	0.1	1.0E+00	-0.1	9.0E-01	0.0	1.0E+00	-0.1	7.7E-01	-0.1	9.0E-01
29888	FLJ10357	A_23_P99661	NM_018071	0.9	1.58E-03	0.9	1.58E-03	0.8	2.52E-02	1.0	1.76E-04	0.9	3.28E-03	1.1	3.22E-03	0.9	3.32E-02	0.9	3.28E-03	0.2	7.1E-01	0.3	5.2E-01	0.2	7.1E-01	0.2	1.0E+00	0.0	1.0E+00	-0.1	1.0E+00	0.0	9.4E-01	0.0	1.0E+00
17010	TNFSF14	A_24_P237036	NM_003807	0.9	9.66E-04	0.6	1.08E-02	1.6	9.66E-04	0.9	4.36E-04	0.5	4.48E-02	0.5	7.19E-02	0.9	4.48E-02	0.5	1.41E-02	0.1	1.0E+00	0.0	1.0E+00	0.1	8.1E-01	0.1	1.0E+00	0.1	8.0E-01	-0.1	1.0E+00	0.2	5.1E-01	0.1	8.0E-01
31706	PAK6	A_24_P159227	NM_020168	0.9	2.19E-03	0.9	9.63E-03	1.4	2.19E-03	0.8	4.38E-04	1.1	1.88E-02	1.0	8.73E-02	1.1	1.88E-02	1.1	9.91E-03	0.2	1.0E+00	0.2	1.0E+00	0.1	8.3E-01	0.2	1.0E+00	0.0	9.4E-01	0.0	1.0E+00	0.0	9.4E-01	0.1	9.3E-01
14068	tcag7.1177	A_32_P141374	NM_178831	0.9	7.49E-03	0.8	7.49E-03	0.9	8.80E-03	0.9	2.43E-04	0.9	2.99E-02	0.7	7.55E-02	0.9	2.99E-02	0.9	3.60E-03	0.2	1.0E+00	0.2	1.0E+00	0.2	7.1E-01	0.1	1.0E+00	0.1	1.0E+00	0.2	1.0E+00	0.0	1.0E+00	0.1	9.4E-01
30990	C9orf89	A_23_P146367	NM_032310	0.9	1.39E-03	0.7	1.39E-03	0.9	4.25E-03	1.0	4.24E-05	0.8	1.62E-02	0.8	1.62E-02	0.7	1.16E-01	0.9	1.74E-03	0.2	1.0E+00	0.1	1.0E+00	0.3	7.1E-01	0.2	1.0E+00	0.0	9.6E-01	0.0	1.0E+00	-0.1	8.6E-01	0.0	9.6E-01
42521	ADA	A_23_P210482	NM_000022	0.9	1.26E-02	0.6	3.64E-02	1.1	1.26E-02	0.9	1.19E-03	0.6	5.07E-02	0.5	9.13E-02	0.8	1.21E-02	0.6	5.07E-02	0.1	1.0E+00	0.1	1.0E+00	0.1	7.6E-01	0.0	1.0E+00	0.0	9.3E-01	0.0	1.0E+00	0.0	9.3E-01	0.1	8.6E-01
7728	BU732811	A_24_P584992	BU732811	0.9	6.50E-03	0.9	1.17E-03	0.9	1.04E-02	0.6	6.50E-03	0.6	2.77E-01	0.7	2.77E-01	0.2	6.34E-01	0.6	4.61E-02	0.0	1.0E+00	0.0	1.0E+00	-0.3	7.1E-01	0.0	1.0E+00	0.0	9.9E-01	0.1	1.0E+00	-0.1	9.2E-01	0.0	9.9E-01
35763	CCDC116	A_23_P342108	NM_152612	0.9	2.17E-02	0.9	2.17E-02	0.6	3.95E-02	0.9	2.28E-04	0.7	1.65E-01	0.7	1.65E-01	0.7	1.88E-01	0.8	2.14E-02	0.0	1.0E+00	0.0	1.0E+00	-0.1	7.7E-01	0.2	1.0E+00	-0.3	5.9E-01	-0.2	1.0E+00	-0.3	2.8E-01	-0.3	5.9E-01
7927	MAGI3	A_24_P118171	NM_152900	0.9	4.29E-04	0.9	2.37E-02	1.6	2.81E-04	0.7	4.29E-04	0.5	3.64E-01	0.4	3.61E-01	0.9	3.64E-01	0.5	4.52E-01	-0.1	1.0E+00	0.2	1.0E+00	-0.3	7.5E-01	-0.1	1.0E+00	-0.2	9.2E-01	0.0	1.0E+00	-0.2	8.1E-01	-0.2	9.2E-01
34523	TFPI2	A_23_P393620	NM_006528	0.9	5.59E-04	0.9	2.72E-03	1.3	5.59E-04	0.8	7.19E-05	0.7	8.77E-02	0.6	8.90E-02	1.0	8.77E-02	0.7	4.11E-02	0.0	1.0E+00	0.1	1.0E+00	0.0	9.3E-01	0.0	1.0E+00	0.1	9.7E-01	0.1	1.0E+00	0.0	9.7E-01	0.1	9.0E-01
1415	PHF15	A_23_P156198	NM_015288	0.9	2.19E-02	0.8	2.67E-02	1.1	2.19E-02	0.9	3.11E-03	0.5	6.20E-02	0.5	6.20E-02	0.5	2.19E-01	0.6	1.66E-02	-0.2	1.0E+00	0.1	1.0E+00	-0.2	7.3E-01	-0.2	1.0E+00	0.0	1.0E+00	0.1	1.0E+00	0.0	9.9E-01	0.0	1.0E+00
12174	IRX5	A_24_P48057	NM_005853	0.9	3.51E-03	0.6	4.98E-03	1.1	3.51E-03	0.9	1.86E-04	0.9	4.24E-02	0.5	3.52E-01	1.1	4.24E-02	0.9	2.03E-02	0.0	1.0E+00	0.0	1.0E+00	-0.1	8.3E-01	0.1	1.0E+00	-0.2	6.9E-01	-0.1	1.0E+00	-0.3	3.3E-01	-0.2	6.9E-01
9550	NPL	A_23_P381431	NM_030769	0.9	2.97E-02	0.9	4.79E-02	1.0	2.97E-02	0.7	1.17E-02	0.7	1.95E-01	0.7	1.95E-01	0.5	2.76E-01	0.7	1.21E-01	0.0	1.0E+00	0.0	1.0E+00	-0.1	9.0E-01	0.2	1.0E+00	-0.2	4.8E-01	-0.1	1.0E+00	-0.2	4.8E-01	-0.3	4.5E-01
1553	FHOD1	A_23_P37778	NM_013241	0.9	7.12E-03	0.9	7.12E-03	1.2	1.55E-02	0.8	6.35E-03	0.9	2.13E-02	0.9	2.13E-02	0.7	4.96E-02	0.9	7.32E-03	0.3	1.0E+00	0.3	1.0E+00	0.2	7.3E-01	0.3	1.0E+00	0.0	1.0E+00	0.1	1.0E+00	0.0	1.0E+00	0.0	9.6E-01
15404	SPN	A_23_P433760	NM_001030288	0.9	1.94E-03	0.7	1.94E-03	0.9	7.63E-03	0.9	1.62E-04	0.7	7.17E-02	0.5	1.39E-01	0.8	4.84E-02	0.7	7.17E-02	-0.1	1.0E+00	-0.1	1.0E+00	0.5	7.1E-01	-0.1	1.0E+00	0.2	8.1E-01	0.1	1.0E+00	0.2	4.7E-01	0.2	8.1E-01
26253	ZFHX2	A_32_P226205	AB051549	0.9	9.81E-03	0.9	5.80E-03	1.0	1.96E-02	0.6	9.81E-03	1.2	5.34E-02	1.2	1.34E-02	1.4	7.18E-02	0.9	5.34E-02	0.1	1.0E+00	0.1	1.0E+00	-0.2	7.7E-01	0.2	1.0E+00	-0.1	8.5E-01	0.0	1.0E+00	-0.2	6.6E-01	-0.1	8.5E-01
29262	TCEAL3	A_23_P434442	NM_001006933	0.9	1.08E-02	0.7	3.67E-02	1.5	1.08E-02	0.9	4.78E-03	0.5	8.01E-02	0.5	3.39E-01	0.6	7.54E-02	0.5	8.01E-02	0.2	1.0E+00	0.3	1.0E+00	0.1	7.5E-01	0.2	1.0E+00	-0.3	7.3E-01	-0.1	1.0E+00	-0.3	4.4E-01	-0.3	7.3E-01
24770	CA945082	A_32_P121704	CA945082	0.9	1.11E-02	0.7	3.28E-02	1.5	1.11E-02	0.9	6.88E-03	0.7	3.13E-01	0.7	3.13E-01	0.9	9.63E-02	0.5	3.49E-01	0.3	1.0E+00	0.3	1.0E+00	-0.2	7.1E-01	0.3	1.0E+00	0.2	8.4E-01	0.1	1.0E+00	0.2	6.3E-01	0.2	8.4E-01
13474	SOD2	A_23_P134176	NM_001024465	0.9	1.55E-03	0.7	4.40E-02	1.4	1.40E-03	0.9	1.55E-03	0.6	1.30E-01	0.6	1.96E-01	0.7	6.76E-02	0.6	1.30E-01	0.0	1.0E+00	0.0	1.0E+00	0.0	9.4E-01	0.1	1.0E+00	-0.1	9.8E-01	-0.1	1.0E+00	-0.3	4.1E-01	0.0	9.8E-01
22800	TSGA10	A_23_P17103	NM_025244	0.9	8.51E-03	1.0	6.75E-03	0.9	3.06E-02	0.7	8.51E-03	0.9	2.07E-02	1.3	1.00E-02	0.9	2.86E-02	0.8	2.07E-02	0.1	1.0E+00	0.1	1.0E+00	-0.1	9.0E-01	0.3	1.0E+00	-0.1	8.7E-01	0.0	1.0E+00	-0.1	8.3E-01	-0.2	8.7E-01
40219	C19orf33	A_23_P208788	NM_033520	0.9	9.81E-03	0.8	3.24E-02	1.6	9.41E-03	0.9	9.81E-03	0.5	1.77E-01	0.4	1.77E-01	0.8	2.47E-01	0.5	7.70E-02	-0.1	1.0E+00	0.0	1.0E+00	-0.1	8.0E-01	-0.3	1.0E+00	-0.1	9.4E-01	0.1	1.0E+00	-0.1	8.1E-01	-0.1	9.4E-01
31035	THC2668196	A_32_P203408	THC2668196	0.9	1.19E-02	0.9	1.19E-02	0.7	2.32E-02	0.9	1.98E-04	0.3	8.15E-01	-0.1	9.71E-01	0.3	8.15E-01	0.4	7.60E-01	0.0	1.0E+00	0.0	1.0E+00	0.2	8.0E-01	-0.1	1.0E+00	0.2	5.7E-01	0.1	1.0E+00	0.2	5.7E-01	0.6	9.8E-02
8022	KLHDC8B	A_23_P125117	NM_173546	0.9	2.53E-02	0.9	3.69E-02	1.1	2.53E-02	0.7	1.36E-02	0.8	4.48E-02	0.8	4.48E-02	0.6	5.33E-02	0.8	8.92E-03	0.2	1.0E+00	0.2	1.0E+00	0.2	7.2E-01	0.3	1.0E+00	-0.1	9.5E-01	-0.1	1.0E+00	-0.2	7.7E-01	-0.1	9.5E-01
42862	SIGLECP3	A_32_P128258	NR_002804	0.9	2.69E-04	0.6	1.77E-02	1.6	2.69E-04	0.9	4.96E-05	0.4	2.53E-01	0.2	3.52E-01	0.4	2.53E-01	0.4	4.56E-02	0.1	1.0E+00	0.1	1.0E+00	0.1	7.6E-01	0.0	1.0E+00	0.0	9.6E-01	0.0	1.0E+00	0.0	9.4E-01	0.0	9.6E-01
30267	ENST00000333156	A_24_P316019	ENST00000333156	0.9	6.14E-03	0.8	6.14E-03	0.9	1.55E-02	0.9	6.45E-04	0.5	3.73E-01	0.3	7.30E-01	0.5	3.73E-01	0.6	2.50E-01	0.1	1.0E+00	0.1	1.0E+00	-0.1	8.5E-01	0.2	1.0E+00	0.1	9.1E-01	0.1	1.0E+00	-0.1	7.9E-01	0.1	9.1E-01
4433	GMPR	A_24_P277657	NM_006877	0.9	3.69E-02	0.9	4.30E-02	1.3	2.90E-02	0.7	3.69E-02	0.8	2.14E-01	0.9	2.14E-01	0.8	2.47E-01	0.8	9.29E-02	0.2	1.0E+00	0.2	1.0E+00	0.0	9.9E-01	0.3	1.0E+00	-0.3	7.5E-01	-0.1	1.0E+00	-0.3	5.1E-01	-0.3	7.5E-01
43186	ORMDL2	A_23_P87500	NM_014182	0.9	4.78E-03	0.9	4.78E-03	0.7	3.92E-02	0.9	5.50E-04	0.6	1.07E-01	0.6	4.48E-02	0.4	2.17E-01	0.7	1.07E-01	-0.1	1.0E+00	0.1	1.0E+00	-0.1	8.8E-01	-0.2	1.0E+00	0.1	9.6E-01	0.2	1.0E+00	0.0	9.6E-01	0.1	9.4E-01
34907	PCK2	A_23_P140207	NM_004563	0.9	5.36E-03	1.0	5.36E-03	0.9	9.52E-03	0.7	8.70E-04	0.8	1.57E-02	0.7	9.68E-02	0.9	1.50E-02	0.8	1.57E-02	0.1	1.0E+00	0.1	1.0E+00	0.2	7.1E-01	0.1	1.0E+00	-0.2	7.6E-01	0.0	1.0E+00	-0.2	6.3E-01	-0.2	7.6E-01
21808	PPP1R12C	A_23_P50575	NM_017607	0.9	3.45E-03	1.1	3.45E-03	0.9	3.72E-02	0.9	1.50E-03	0.6	1.50E-01	0.6	1.50E-01	0.1	9.59E-01	0.8	4.25E-02	0.1	1.0E+00	0.1	1.0E+00	0.2	7.3E-01	0.1	1.0E+00	0.1	9.1E-01	0.1	1.0E+00	0.1	8.2E-01	0.1	9.1E-01
12772	SYT11	A_24_P139899	NM_152280	0.9	3.22E-03	0.9	3.22E-03	0.9	6.43E-03	0.9	9.32E-05	0.9	9.51E-03	1.0	9.51E-03	0.9	4.98E-02	0.9	2.13E-03	0.0	1.0E+00	0.0	1.0E+00	-0.1	8.2E-01	0.1	1.0E+00	-0.2	4.7E-01	-0.1	1.0E+00	-0.2	3.6E-01	-0.2	4.7E-01
7031	MAGEA9	A_32_P29965	NM_005365	0.9	3.36E-03	0.7	9.52E-03	0.9	3.36E-03	0.9	2.27E-04	0.3	2.79E-01	0.3	2.79E-01	0.3	3.12E-01	0.5	6.24E-02	-0.1	1.0E+00	-0.1	1.0E+00	-0.1	8.8E-01	0.0	1.0E+00	0.0	9.5E-01	-0.1	1.0E+00	0.1	7.7E-01	0.0	9.5E-01
12117	PAPSS2	A_23_P104493	NM_001015880	0.9	1.71E-02	0.9	1.71E-02	0.7	2.72E-02	0.9	2.83E-04	0.9	2.98E-02	0.9	2.98E-02	0.6	1.70E-01	0.9	1.51E-02	0.1	1.0E+00	0.1	1.0E+00	0.0	8.9E-01	0.1	1.0E+00	-0.1	9.0E-01	0.0	1.0E+00	-0.2	4.8E-01	-0.1	9.0E-01
25130	A_23_P129405	A_23_P129405	A_23_P129405	0.9	4.89E-03	0.7	4.89E-03	0.9	7.78E-03	1.0	2.83E-04	0.6	9.06E-02	0.6	9.06E-02	0.5	1.51E-01	0.8	1.74E-02	0.2	1.0E+00	0.2	1.0E+00	0.2	7.2E-01	0.1	1.0E+00	0.1	9.2E-01	0.1	1.0E+00	0.1	8.4E-01	0.1	9.2E-01
11452	ZMYM5	A_24_P321634	NM_001039650	0.9	2.75E-03	0.7	2.75E-03	1.0	6.81E-03	0.9	2.50E-04	0.8	1.03E-02	0.8	9.30E-03	0.3	3.03E-01	0.8	1.03E-02	0.0	1.0E+00	0.1	1.0E+00	0.0	9.5E-01	0.0	1.0E+00	-0.2	7.6E-01	-0.1	1.0E+00	-0.2	5.8E-01	-0.2	7.6E-01
43633	OSTF1	A_23_P60296	NM_012383	0.9	2.63E-02	0.6	4.40E-02	1.1	2.63E-02	0.9	4.76E-03	0.7	6.88E-02	0.7	5.91E-02	0.7	6.88E-02	0.4	1.74E-01	0.1	1.0E+00	0.1	1.0E+00	0.1	8.8E-01	0.0	1.0E+00	-0.2	7.1E-01	0.0	1.0E+00	-0.2	4.7E-01	-0.2	7.1E-01
9908	MAP3K12	A_23_P105409	NM_006301	0.9	3.36E-03	0.9	3.36E-03	1.0	3.44E-03	0.9	7.36E-05	1.0	1.42E-01	1.0	1.42E-01	0.5	5.33E-01	1.0	4.97E-02	0.1	1.0E+00	0.2	1.0E+00	0.0	9.5E-01	0.1	1.0E+00	-0.2	8.3E-01	-0.1	1.0E+00	-0.3	4.5E-01	-0.2	8.3E-01
13508	TAL2	A_23_P409449	NM_005421	0.9	3.12E-04	0.9	3.12E-04	1.0	2.70E-03	0.8	1.85E-04	0.5	2.87E-01	0.5	4.46E-01	0.5	2.87E-01	0.6	1.26E-01	0.1	1.0E+00	0.1	1.0E+00	0.0	9.2E-01	0.1	1.0E+00	0.0	9.5E-01	0.0	1.0E+00	0.0	9.4E-01	0.0	9.5E-01
42901	KIAA0329	A_32_P355396	NM_014844	0.9	1.49E-02	0.9	1.60E-02	1.7	3.81E-03	0.6	1.49E-02	1.1	2.76E-02	1.1	1.84E-02	1.3	2.76E-02	0.7	4.68E-02	0.6	4.2E-01	0.5	4.2E-01	0.6	3.8E-01	0.6	8.2E-01	0.0	9.9E-01	0.1	1.0E+00	0.0	9.9E-01	-0.1	9.1E-01
18841	ABCB9	A_24_P312895	NM_019625	0.9	1.99E-03	0.8	1.99E-03	1.1	3.32E-03	0.9	1.10E-04	0.7	2.15E-01	0.7	2.84E-01	0.7	2.15E-01	0.8	8.49E-02	0.2	1.0E+00	0.2	1.0E+00	0.0	9.5E-01	0.2	1.0E+00	-0.2	7.8E-01	-0.1	1.0E+00	-0.2	5.2E-01	-0.2	7.8E-01
30182	GPSM3	A_23_P219060	NM_022107	0.9	7.75E-03	0.7	1.75E-02	1.3	5.66E-03	0.9	7.75E-03	0.7	4.06E-01	0.1	9.69E-01	1.2	4.06E-01	0.7	2.04E-01	-0.1	1.0E+00	-0.1	1.0E+00	0.3	7.8E-01	-0.3	1.0E+00	0.3	7.0E-01	0.1	1.0E+00	0.3	7.0E-01	0.6	5.6E-01
14412	IL29	A_23_P337800	NM_172140	0.9	3.20E-03	0.7	3.57E-03	0.9	3.20E-03	0.9	1.18E-04	0.6	3.96E-02	0.6	3.96E-02	0.4	1.99E-01	0.6	1.75E-02	0.0	1.0E+00	0.0	1.0E+00	0.3	7.3E-01	-0.1	1.0E+00	0.2	7.4E-01	0.1	1.0E+00	0.3	1.2E-01	0.2	7.4E-01
8015	TRIM15	A_23_P214554	NM_033229	0.9	1.43E-02	0.8	1.43E-02	0.9	3.83E-02	0.9	2.25E-03	1.0	5.17E-02	1.0	7.32E-02	1.0	5.17E-02	1.2	1.38E-02	0.1	1.0E+00	0.1	1.0E+00	0.1	8.9E-01	0.4	1.0E+00	0.0	9.9E-01	0.0	1.0E+00	0.0	9.9E-01	0.0	9.9E-01
29529	TFCP2L1	A_23_P5301	NM_014553	0.9	6.47E-03	0.9	6.47E-03	1.2	1.09E-02	0.9	1.23E-03	0.6	2.08E-02	0.6	1.91E-02	0.8	2.08E-02	0.2	6.01E-01	0.0	1.0E+00	0.0	1.0E+00	0.0	9.9E-01	0.0	1.0E+00	0.0	9.9E-01	0.0	1.0E+00	0.0	9.9E-01	0.0	9.6E-01
35685	DKFZP564O0823	A_23_P212779	NM_015393	0.9	2.44E-03	1.6	1.35E-03	0.9	3.40E-02	0.8	2.44E-03	1.6	4.59E-03	1.5	4.59E-03	1.9	3.00E-02	1.6	1.67E-03	0.1	1.0E+00	0.3	1.0E+00	-0.1	9.0E-01	0.1	1.0E+00	-0.2	4.4E-01	-0.2	1.0E+00	-0.2	4.4E-01	-0.4	4.1E-01
14879	BTNL9	A_32_P187951	NM_152547	0.9	1.46E-03	1.0	1.46E-03	0.8	1.32E-02	0.9	2.15E-04	0.9	5.26E-02	0.9	1.21E-01	1.0	5.13E-02	0.8	5.26E-02	0.1	1.0E+00	0.2	1.0E+00	-0.2	7.1E-01	0.1	1.0E+00	-0.1	9.2E-01	0.1	1.0E+00	-0.1	7.1E-01	-0.1	9.2E-01
37893	MAGEA6	A_23_P136870	NM_175868	0.9	3.32E-02	0.8	3.54E-02	1.7	1.81E-03	0.9	3.32E-02	0.0	6.84E-01	-0.7	6.84E-01	1.9	5.74E-01	0.0	9.81E-01	-0.5	1.0E+00	-0.5	1.0E+00	-0.3	9.0E-01	-0.8	1.0E+00	0.4	8.5E-01	0.0	1.0E+00	0.4	5.6E-01	0.4	8.5E-01
40056	MAP1LC3A	A_23_P154786	NM_032514	0.9	3.64E-03	0.8	3.64E-03	1.0	6.93E-03	0.9	1.09E-03	0.2	8.35E-01	-0.4	8.27E-01	0.3	8.35E-01	0.2	9.02E-01	0.0	1.0E+00	0.0	1.0E+00	0.1	8.4E-01	-0.1	1.0E+00	0.2	6.7E-01	0.0	1.0E+00	0.2	5.3E-01	0.3	6.7E-01
28740	SERPIND1	A_24_P40306	NM_000185	0.9	2.38E-03	0.9	8.59E-03	0.9	2.38E-03	1.3	3.05E-06	0.4	6.24E-02	0.4	6.24E-02	0.4	1.22E-01	0.8	1.95E-03	0.0	1.0E+00	0.0	1.0E+00	0.0	8.8E-01	0.0	1.0E+00	-0.1	1.0E+00	-0.2	1.0E+00	-0.1	7.7E-01	0.0	1.0E+00
28689	DENND5B	A_24_P541919	NM_144973	0.9	4.58E-03	0.9	4.58E-03	0.8	1.72E-02	1.1	1.53E-04	1.0	2.15E-02	1.0	2.15E-02	1.1	3.04E-02	0.9	1.52E-02	0.4	8.2E-01	0.4	8.2E-01	0.1	8.1E-01	0.5	1.0E+00	-0.1	8.5E-01	0.0	1.0E+00	-0.2	6.7E-01	-0.1	8.5E-01
7127	HPSE	A_23_P256107	NM_006665	0.9	1.56E-03	0.8	6.85E-03	1.4	1.56E-03	0.9	2.74E-04	0.6	2.50E-01	0.6	2.50E-01	0.4	4.92E-01	0.7	9.25E-02	0.1	1.0E+00	0.1	1.0E+00	0.1	7.3E-01	0.2	1.0E+00	-0.1	7.9E-01	-0.1	1.0E+00	-0.2	3.1E-01	-0.1	7.9E-01
17829	ST3GAL5	A_23_P136573	NM_003896	0.9	3.37E-03	0.9	3.37E-03	1.1	9.28E-03	0.8	1.37E-03	1.1	2.51E-02	1.1	1.72E-02	1.5	1.70E-01	0.9	2.51E-02	0.0	1.0E+00	0.1	1.0E+00	0.0	9.0E-01	0.0	1.0E+00	-0.2	8.0E-01	0.0	1.0E+00	-0.2	5.6E-01	-0.2	8.0E-01
21726	APOB48R	A_23_P54770	NM_018690	0.9	5.47E-04	0.8	1.02E-02	1.7	5.47E-04	0.9	1.37E-04	0.7	4.50E-03	0.7	2.08E-02	0.9	3.50E-03	0.7	4.50E-03	0.1	1.0E+00	0.1	1.0E+00	0.1	7.5E-01	0.2	1.0E+00	-0.2	5.5E-01	-0.1	1.0E+00	-0.3	2.0E-01	-0.2	5.5E-01
11890	C17orf100	A_32_P150391	NM_001105520	0.9	2.72E-03	0.8	2.72E-03	0.9	1.10E-02	0.9	3.95E-04	0.8	4.93E-02	0.7	1.69E-01	0.8	3.63E-02	0.9	4.93E-02	0.1	1.0E+00	0.1	1.0E+00	-0.1	8.6E-01	0.1	1.0E+00	-0.2	7.1E-01	-0.1	1.0E+00	-0.2	4.8E-01	-0.2	7.1E-01
24534	RIBC1	A_23_P73667	NM_001031745	0.9	4.98E-03	0.9	4.98E-03	0.9	1.50E-02	0.8	1.13E-03	1.0	6.30E-02	1.0	1.06E-01	1.0	6.30E-02	0.9	5.86E-02	0.0	1.0E+00	0.0	1.0E+00	0.1	8.7E-01	0.0	1.0E+00	-0.2	8.3E-01	-0.1	1.0E+00	-0.2	7.5E-01	-0.2	8.3E-01
8800	CDKN1A	A_24_P89457	NM_078467	0.9	1.25E-02	0.9	1.25E-02	0.8	4.94E-02	1.4	8.83E-05	1.1	7.70E-02	0.6	2.26E-01	1.1	7.70E-02	1.4	7.21E-03	0.3	1.0E+00	0.2	1.0E+00	0.3	7.1E-01	0.3	1.0E+00	-0.2	8.8E-01	-0.2	1.0E+00	-0.2	6.5E-01	-0.2	8.8E-01
1346	RHOC	A_23_P12514	NM_175744	0.9	1.49E-02	0.7	2.53E-02	1.4	1.49E-02	0.9	4.45E-03	0.6	8.22E-02	0.4	2.02E-01	0.7	8.22E-02	0.6	6.42E-02	0.0	1.0E+00	0.1	1.0E+00	0.0	9.4E-01	-0.1	1.0E+00	0.0	9.8E-01	0.0	1.0E+00	0.0	9.7E-01	0.0	9.8E-01
32673	AK123722	A_24_P783122	AK123722	0.9	5.02E-04	0.8	3.10E-02	1.2	5.02E-04	0.9	2.12E-05	0.8	3.09E-02	0.8	3.09E-02	1.0	9.63E-02	0.7	1.75E-02	0.0	1.0E+00	0.1	1.0E+00	0.0	9.1E-01	0.0	1.0E+00	-0.2	6.2E-01	0.0	1.0E+00	-0.2	2.0E-01	-0.2	6.2E-01
27346	ELL	A_23_P27936	NM_006532	0.9	1.24E-03	0.6	3.22E-03	1.4	1.24E-03	0.9	9.89E-05	0.8	9.89E-02	0.5	4.05E-01	0.9	9.89E-02	0.8	6.25E-02	0.1	1.0E+00	0.0	1.0E+00	0.1	8.8E-01	0.2	1.0E+00	-0.2	7.4E-01	-0.1	1.0E+00	-0.2	7.4E-01	-1.0	5.6E-01
3103	LPIN2	A_24_P301557	NM_014646	0.9	2.93E-02	0.7	3.57E-02	0.9	2.93E-02	0.9	7.93E-03	0.6	7.38E-02	0.5	1.49E-01	0.6	7.38E-02	0.8	4.13E-03	0.0	1.0E+00	0.0	1.0E+00	-0.2	8.2E-01	0.1	1.0E+00	0.0	9.7E-01	0.1	1.0E+00	0.0	9.7E-01	-0.1	9.5E-01
26969	NARF	A_23_P218317	NM_001038618	0.9	6.64E-04	1.0	6.64E-04	0.8	9.80E-03	0.9	2.15E-04	0.8	4.13E-02	0.8	4.13E-02	0.6	4.82E-02	1.0	3.61E-03	0.0	1.0E+00	0.0	1.0E+00	0.0	8.8E-01	0.2	1.0E+00	0.0	1.0E+00	0.0	1.0E+00	-0.1	7.8E-01	0.0	1.0E+00
37235	PCSK1N	A_23_P22487	NM_013271	0.9	1.19E-02	0.7	1.41E-02	1.3	1.19E-02	0.9	4.15E-03	0.7	1.71E-01	0.6	2.60E-01	0.7	1.71E-01	1.0	1.25E-02	0.0	1.0E+00	0.1	1.0E+00	0.0	9.7E-01	-0.2	1.0E+00	0.1	9.6E-01	0.3	1.0E+00	0.0	9.6E-01	0.1	9.6E-01
12169	ARSI	A_23_P19030	NM_001012301	0.9	1.44E-03	0.7	3.24E-03	1.4	1.44E-03	0.9	2.38E-04	0.5	3.14E-01	0.4	5.64E-01	0.6	1.92E-01	0.5	3.14E-01	0.0	1.0E+00	0.1	1.0E+00	0.0	1.0E+00	-0.1	1.0E+00	0.0	9.5E-01	-0.2	1.0E+00	0.0	8.7E-01	0.0	9.5E-01
42893	NOTCH4	A_23_P365614	NM_004557	0.9	3.67E-03	0.8	3.67E-03	1.0	7.77E-03	0.9	5.61E-04	1.2	9.90E-03	1.2	9.90E-03	1.2	5.70E-03	0.9	1.01E-02	0.2	1.0E+00	0.2	1.0E+00	0.2	7.2E-01	0.3	1.0E+00	-0.3	6.8E-01	-0.2	1.0E+00	-0.4	2.5E-01	-0.3	6.8E-01
41611	SIRPA	A_24_P267619	NM_001040022	0.9	6.57E-04	0.8	6.57E-04	1.4	7.18E-04	0.9	2.99E-05	0.5	2.03E-01	0.1	8.78E-01	0.8	2.03E-01	0.5	8.78E-02	0.1	1.0E+00	0.1	1.0E+00	0.2	7.9E-01	0.0	1.0E+00	0.0	9.6E-01	0.0	1.0E+00	-0.1	7.1E-01	0.0	9.6E-01
18431	TUBA4A	A_23_P102109	NM_006000	0.9	1.74E-03	0.9	1.74E-03	0.9	8.39E-03	0.8	3.35E-04	0.7	4.07E-02	0.7	6.71E-02	0.8	2.89E-02	0.7	4.07E-02	0.1	1.0E+00	0.1	1.0E+00	0.0	9.7E-01	0.1	1.0E+00	0.0	9.6E-01	0.0	1.0E+00	0.1	7.7E-01	0.0	9.6E-01
6772	TYRO3	A_23_P54517	NM_006293	0.9	3.78E-03	0.9	3.78E-03	1.3	9.34E-03	0.8	2.74E-03	0.8	7.14E-02	0.7	7.45E-02	0.8	7.14E-02	0.8	1.28E-02	0.2	1.0E+00	0.2	1.0E+00	0.3	7.2E-01	0.1	1.0E+00	0.0	9.4E-01	0.0	1.0E+00	0.0	9.4E-01	0.2	8.4E-01
3931	PLAC2	A_23_P39294	BC036545	0.9	3.36E-03	0.9	1.11E-02	1.0	3.36E-03	0.6	6.53E-04	0.8	3.95E-02	0.8	4.71E-02	0.8	3.95E-02	0.7	3.38E-02	0.2	1.0E+00	0.1	1.0E+00	0.2	7.4E-01	0.4	1.0E+00	-0.3	3.4E-01	0.0	1.0E+00	-0.3	2.3E-01	-0.3	3.4E-01
40382	FAM53C	A_23_P167789	NM_016605	0.9	4.61E-03	0.9	4.61E-03	1.1	1.68E-02	0.9	1.40E-03	0.6	1.73E-01	0.6	1.73E-01	0.5	1.84E-01	1.0	2.09E-03	0.1	1.0E+00	0.1	1.0E+00	0.1	8.5E-01	0.1	1.0E+00	0.1	8.1E-01	0.0	1.0E+00	0.1	8.1E-01	0.3	6.9E-01
30692	EPN2	A_23_P89310	NM_014964	0.9	1.95E-03	0.9	1.95E-03	1.4	2.99E-03	0.9	4.66E-04	0.9	4.13E-02	0.8	4.49E-02	0.9	4.13E-02	1.0	7.56E-03	0.0	1.0E+00	0.1	1.0E+00	-0.1	8.4E-01	0.0	1.0E+00	0.1	9.4E-01	0.2	1.0E+00	0.0	9.4E-01	0.1	8.9E-01
21295	GSC	A_23_P76774	NM_173849	0.9	9.59E-03	0.9	1.23E-02	1.3	9.59E-03	0.9	1.44E-03	0.9	6.06E-02	0.9	6.66E-02	1.0	6.06E-02	0.8	4.50E-02	0.1	1.0E+00	0.1	1.0E+00	-0.1	8.4E-01	0.1	1.0E+00	-0.1	8.9E-01	0.2	1.0E+00	-0.1	8.8E-01	-0.1	8.9E-01
28161	CBLC	A_23_P397910	NM_012116	0.9	5.74E-04	0.8	5.23E-03	1.7	5.74E-04	0.9	7.72E-05	0.7	2.33E-02	0.7	7.01E-02	0.7	2.33E-02	0.7	1.57E-02	0.2	1.0E+00	0.2	1.0E+00	0.0	9.5E-01	0.2	1.0E+00	-0.1	9.1E-01	0.0	1.0E+00	-0.1	6.3E-01	-0.1	9.1E-01
37179	NTF4	A_23_P4899	NM_006179	0.9	9.96E-04	0.9	1.01E-02	1.6	9.96E-04	0.9	1.72E-04	1.0	3.18E-03	1.1	3.18E-03	0.9	3.54E-03	1.0	4.81E-04	0.1	1.0E+00	0.1	1.0E+00	0.0	9.5E-01	0.1	1.0E+00	-0.2	8.4E-01	-0.1	1.0E+00	-0.2	6.9E-01	-0.2	8.4E-01
9293	DAPK3	A_23_P165028	NM_001348	0.9	1.27E-02	0.9	1.27E-02	1.4	9.55E-03	0.7	1.29E-02	0.7	7.25E-02	0.5	3.09E-01	0.7	7.25E-02	0.9	1.91E-02	0.2	1.0E+00	0.3	1.0E+00	0.2	7.2E-01	0.2	1.0E+00	0.1	9.6E-01	0.1	1.0E+00	-0.2	7.8E-01	0.1	9.6E-01
5659	ATF5	A_23_P119337	NM_012068	0.9	2.34E-02	0.9	2.20E-02	1.3	2.34E-02	0.6	3.81E-02	0.8	1.62E-02	0.7	8.20E-02	1.2	1.10E-02	0.8	1.62E-02	0.1	1.0E+00	0.1	1.0E+00	0.0	9.8E-01	0.1	1.0E+00	0.1	9.0E-01	0.0	1.0E+00	0.1	9.0E-01	0.2	8.6E-01

Feature	Gene symbol	Probe	Transcript	72 hour siCASP8AP2								48 hours siCASP8AP2								24 hours siCASP8AP2								10 hours siCASP8AP2							
				Median	Median	1	1	3	3	6	6	Median	Median	1	1	3	3	6	6	Median	Median	1	1	3	3	6	6	Median	Median	1	1	3	3	6	6
				M	q	M	q	M	q	M	q	M	q	M	q	M	q	M	q	M	q	M	q	M	q	M	q	M	q	M	q	M	q	M	q
26148	KIAA0323	A_24_P205268	NM_015299	0.9	9.43E-03	0.7	9.43E-03	0.9	1.94E-02	0.9	7.64E-04	0.6	7.07E-02	0.6	7.07E-02	0.5	2.30E-01	0.6	1.90E-02	0.1	1.0E+00	0.2	1.0E+00	0.1	9.1E-01	-0.1	1.0E+00	0.2	4.3E-01	0.1	1.0E+00	0.2	2.9E-01	0.2	4.3E-01
15758	KIAA0247	A_23_P371787	NM_014734	0.9	1.01E-03	0.8	1.49E-03	1.6	1.01E-03	0.9	2.33E-04	0.7	3.30E-02	0.6	1.30E-01	0.8	1.94E-02	0.7	3.30E-02	0.1	1.0E+00	0.0	1.0E+00	0.1	8.5E-01	0.2	1.0E+00	-0.1	9.9E-01	-0.1	1.0E+00	-0.1	8.8E-01	0.0	9.9E-01
33182	TTC25	A_23_P73150	NM_031421	0.9	2.59E-02	0.9	2.59E-02	1.6	9.02E-03	0.6	3.96E-02	0.8	4.14E-02	0.8	4.14E-02	1.5	4.04E-02	0.7	8.55E-02	0.0	1.0E+00	0.0	1.0E+00	0.0	1.0E+00	0.1	1.0E+00	-0.1	9.5E-01	-0.1	1.0E+00	-0.2	7.0E-01	-0.1	9.5E-01
8242	MAP3K6	A_24_P145653	NM_004672	0.9	1.14E-03	0.9	8.39E-04	1.2	2.62E-03	0.7	1.14E-03	0.9	1.70E-03	0.9	3.97E-03	1.1	1.40E-03	0.8	1.70E-03	0.2	1.0E+00	0.1	1.0E+00	0.2	7.1E-01	0.2	1.0E+00	0.1	9.1E-01	0.1	1.0E+00	0.1	8.2E-01	0.1	9.1E-01
26040	GJC3	A_24_P43723	NM_181538	0.9	1.96E-03	0.9	1.96E-03	0.7	1.19E-02	0.9	6.60E-05	0.8	3.41E-01	0.8	3.41E-01	0.5	5.25E-01	0.9	1.27E-01	0.0	1.0E+00	0.1	1.0E+00	-0.1	7.1E-01	0.0	1.0E+00	0.0	9.7E-01	0.0	1.0E+00	0.0	9.4E-01	0.0	9.7E-01
3028	LAT2	A_24_P351852	NM_032464	0.9	1.62E-02	0.9	4.18E-02	0.9	1.62E-02	1.3	6.76E-05	0.9	6.41E-03	0.6	4.68E-02	0.9	6.41E-03	1.2	9.66E-04	0.2	1.0E+00	0.2	1.0E+00	0.1	7.5E-01	0.3	1.0E+00	0.0	9.8E-01	0.0	1.0E+00	-0.1	8.4E-01	0.0	9.8E-01
14439	VAMP5	A_23_P39840	NM_006634	0.9	1.76E-03	0.8	1.76E-03	1.2	3.51E-03	0.9	2.58E-04	0.9	2.86E-02	0.9	2.86E-02	0.7	6.36E-02	1.1	3.06E-03	0.2	1.0E+00	0.2	1.0E+00	0.1	7.4E-01	0.2	1.0E+00	0.0	9.5E-01	0.1	1.0E+00	0.0	9.5E-01	-0.2	8.7E-01
19437	MAGEA12	A_23_P252928	NM_005367	0.9	5.63E-03	0.8	5.63E-03	0.9	1.10E-02	1.2	4.97E-05	0.3	7.46E-01	0.3	7.46E-01	0.1	8.42E-01	0.6	3.71E-01	0.3	1.0E+00	0.1	1.0E+00	0.3	5.5E-01	0.4	1.0E+00	0.1	9.6E-01	0.1	1.0E+00	0.1	9.2E-01	-0.1	9.6E-01
12330	LILRB2	A_23_P208493	NM_005874	0.9	3.18E-03	0.9	1.33E-02	0.8	3.18E-03	1.0	9.00E-06	0.5	2.01E-02	0.5	5.02E-02	0.6	1.32E-02	0.5	2.01E-02	0.0	1.0E+00	0.0	1.0E+00	0.0	9.2E-01	0.0	1.0E+00	0.0	9.3E-01	0.0	1.0E+00	0.0	9.2E-01	0.0	9.3E-01
36708	DYRK1B	A_23_P90296	NM_004714	0.9	1.22E-02	0.9	4.00E-03	0.9	2.89E-02	0.7	1.22E-02	0.4	3.20E-01	0.2	8.19E-01	0.6	2.52E-01	0.4	3.20E-01	0.0	1.0E+00	0.0	1.0E+00	0.4	7.1E-01	0.0	1.0E+00	0.1	9.7E-01	0.0	1.0E+00	0.1	8.7E-01	0.1	9.7E-01
19032	SPINK2	A_23_P155688	NM_021114	0.9	4.84E-03	0.9	3.27E-02	1.1	4.84E-03	0.8	1.10E-03	1.0	2.01E-02	1.0	2.21E-02	1.0	1.38E-02	1.0	2.01E-02	0.0	1.0E+00	0.0	1.0E+00	-0.1	7.9E-01	0.1	1.0E+00	-0.3	2.6E-01	0.0	1.0E+00	-0.3	1.1E-01	-0.3	2.6E-01
23920	C15orf27	A_23_P88678	NM_152335	0.9	1.44E-02	1.1	2.14E-02	0.9	1.44E-02	0.8	1.65E-03	0.9	5.34E-02	1.2	2.23E-02	0.7	9.35E-02	0.9	5.34E-02	0.1	1.0E+00	0.1	1.0E+00	0.0	9.0E-01	0.1	1.0E+00	-0.1	7.8E-01	0.1	1.0E+00	-0.1	6.5E-01	-0.1	7.8E-01
12517	PLXDC1	A_23_P3911	NM_020405	0.9	7.61E-03	1.1	7.61E-03	0.8	1.09E-02	0.9	2.36E-04	1.0	1.20E-02	1.0	1.20E-02	0.8	1.40E-02	1.0	9.66E-04	0.1	1.0E+00	0.0	1.0E+00	0.1	7.4E-01	0.1	1.0E+00	-0.1	9.0E-01	-0.1	1.0E+00	-0.1	7.3E-01	-0.1	9.0E-01
21084	BG821581	A_32_P117503	BG821581	0.9	1.53E-03	0.9	1.53E-03	0.9	2.65E-03	1.1	1.62E-05	0.9	1.95E-02	1.0	1.95E-02	0.7	3.34E-02	0.9	8.20E-03	0.2	1.0E+00	0.2	1.0E+00	0.0	9.7E-01	0.3	1.0E+00	-0.2	6.1E-01	-0.1	1.0E+00	-0.2	3.3E-01	-0.2	6.1E-01
42177	CCDC61	A_23_P400147	NM_001080402	0.9	1.78E-02	0.9	1.78E-02	1.0	2.47E-02	0.6	1.11E-02	0.6	1.75E-01	0.6	1.52E-01	0.6	3.24E-01	0.6	1.75E-01	0.0	1.0E+00	0.0	1.0E+00	0.1	7.7E-01	-0.1	1.0E+00	0.0	9.8E-01	0.1	1.0E+00	0.0	9.8E-01	0.0	9.7E-01
41580	HBG1	A_23_P53137	NM_000559	0.9	1.81E-03	0.9	3.74E-02	1.5	1.81E-03	0.9	4.28E-04	0.5	8.37E-02	0.5	8.37E-02	0.4	9.10E-02	0.8	3.37E-02	0.0	1.0E+00	0.1	1.0E+00	0.0	8.8E-01	-0.1	1.0E+00	-0.1	7.1E-01	0.2	1.0E+00	-0.2	2.7E-01	-0.1	7.1E-01
21884	KCNA7	A_23_P164897	NM_031886	0.9	1.55E-03	0.8	1.55E-03	0.9	2.34E-03	1.1	2.50E-05	0.6	1.97E-02	0.5	7.72E-02	0.6	1.97E-02	0.7	6.90E-03	0.1	1.0E+00	0.1	1.0E+00	-0.1	8.3E-01	0.1	1.0E+00	-0.1	8.8E-01	0.1	1.0E+00	-0.1	6.6E-01	-0.1	8.8E-01
1648	KCNK6	A_24_P262543	NM_004823	0.9	3.39E-02	0.9	4.13E-02	1.7	2.76E-02	0.9	3.39E-02	0.6	3.67E-02	0.5	1.46E-01	0.6	3.67E-02	0.6	1.50E-02	0.2	1.0E+00	0.1	1.0E+00	0.2	7.1E-01	0.2	1.0E+00	0.2	8.9E-01	0.2	1.0E+00	0.1	8.7E-01	0.2	8.9E-01
7448	KIAA1602	A_24_P177964	NM_001037806	0.9	1.20E-02	0.9	1.20E-02	1.1	4.91E-02	0.9	8.76E-03	0.5	2.69E-01	0.5	3.93E-01	0.5	2.69E-01	0.8	4.10E-02	0.1	1.0E+00	0.1	1.0E+00	0.3	7.1E-01	0.0	1.0E+00	0.1	9.6E-01	0.0	1.0E+00	0.1	9.0E-01	0.1	9.6E-01
28310	ACTN2	A_23_P115021	NM_001103	0.9	2.42E-03	0.9	4.61E-02	1.1	2.42E-03	0.9	1.08E-04	0.6	1.33E-01	0.6	1.33E-01	0.6	1.51E-01	0.9	2.62E-02	0.2	1.0E+00	0.2	1.0E+00	-0.1	8.4E-01	0.2	1.0E+00	-0.1	9.3E-01	-0.1	1.0E+00	-0.2	5.0E-01	-0.1	9.3E-01
21144	ZNF720	A_23_P77661	NM_001130913	0.9	3.57E-03	0.9	3.18E-03	1.2	1.19E-02	0.9	3.57E-03	0.7	6.77E-02	0.5	1.73E-01	1.2	3.79E-02	0.7	6.77E-02	0.2	1.0E+00	0.2	1.0E+00	0.2	7.6E-01	0.0	1.0E+00	-0.2	8.0E-01	0.1	1.0E+00	-0.2	6.7E-01	-0.3	8.0E-01
11391	ZFAND5	A_24_P290354	NM_006007	0.9	5.96E-03	0.9	5.96E-03	0.9	8.80E-03	1.0	7.62E-05	0.7	1.76E-01	0.2	5.22E-01	0.8	1.76E-01	0.7	1.91E-02	0.0	1.0E+00	0.1	1.0E+00	0.0	9.7E-01	-0.1	1.0E+00	0.0	9.9E-01	0.0	1.0E+00	0.0	9.9E-01	-0.1	8.2E-01
12376	MXD4	A_23_P259490	NM_006454	0.9	1.73E-03	1.5	1.73E-03	0.9	2.49E-02	0.9	1.02E-03	1.3	3.72E-02	1.3	3.72E-02	0.9	5.82E-02	1.5	6.48E-03	0.2	1.0E+00	0.2	1.0E+00	0.2	7.1E-01	0.3	1.0E+00	0.0	9.6E-01	0.1	1.0E+00	0.0	9.6E-01	-0.1	9.5E-01
17008	ABTB2	A_23_P356616	NM_145804	0.9	3.12E-03	0.9	9.62E-03	0.9	3.12E-03	0.9	1.71E-04	0.6	2.73E-02	0.6	2.73E-02	0.4	1.35E-01	0.7	1.56E-02	0.0	1.0E+00	0.1	1.0E+00	0.0	9.5E-01	0.0	1.0E+00	0.0	9.9E-01	0.1	1.0E+00	-0.1	8.6E-01	0.0	9.9E-01
35579	C19orf33	A_23_P39202	NM_033520	0.9	2.34E-02	0.9	3.25E-02	1.5	1.61E-02	0.7	2.34E-02	0.6	1.57E-01	0.4	1.57E-01	0.6	1.67E-01	0.6	3.64E-02	0.0	1.0E+00	0.2	1.0E+00	0.0	9.7E-01	0.0	1.0E+00	-0.1	8.0E-01	0.2	1.0E+00	-0.1	8.0E-01	-0.3	7.5E-01
13320	PTRF	A_23_P394064	NM_012232	0.9	3.54E-02	0.8	4.20E-02	1.4	3.54E-02	0.9	1.83E-02	0.8	5.75E-02	0.7	9.27E-02	0.9	5.75E-02	0.8	1.15E-02	0.0	1.0E+00	0.1	1.0E+00	0.0	9.6E-01	0.0	1.0E+00	0.0	9.9E-01	0.1	1.0E+00	0.0	9.5E-01	0.0	9.9E-01
39901	ADM	A_23_P127948	NM_001124	0.9	8.58E-03	0.7	8.58E-03	0.9	2.97E-02	1.2	2.22E-04	0.3	3.20E-01	0.3	4.07E-01	0.3	3.20E-01	0.6	8.46E-02	0.0	1.0E+00	0.0	1.0E+00	0.0	9.9E-01	-0.1	1.0E+00	-0.2	7.7E-01	-0.2	1.0E+00	-0.2	4.9E-01	-0.1	7.7E-01
21060	SEPT6	A_23_P22614	NM_145802	0.9	3.24E-03	0.9	6.19E-03	1.6	3.24E-03	0.8	2.59E-03	0.6	3.09E-01	0.6	3.56E-01	1.7	3.09E-01	0.5	2.25E-01	-0.3	1.0E+00	0.1	1.0E+00	-0.5	7.1E-01	-0.3	1.0E+00	-0.1	1.0E+00	-0.1	1.0E+00	-0.2	7.7E-01	0.0	1.0E+00
9969	FLJ20433	A_23_P170088	BC065002	0.9	2.14E-03	0.9	3.89E-02	1.6	9.71E-04	0.7	2.14E-03	0.6	6.42E-02	0.6	5.67E-02	0.0	9.67E-01	0.8	6.42E-02	0.2	1.0E+00	0.2	1.0E+00	0.6	7.1E-01	0.1	1.0E+00	0.1	8.6E-01	0.0	1.0E+00	0.1	8.6E-01	0.2	8.6E-01
31818	ZMYM5	A_23_P65208	NM_001039650	0.9	5.53E-03	0.9	5.53E-03	0.9	2.42E-02	1.1	8.53E-04	0.8	2.67E-02	0.8	2.67E-02	0.6	2.96E-02	0.9	4.81E-03	0.0	1.0E+00	0.1	1.0E+00	0.0	9.9E-01	0.0	1.0E+00	-0.1	9.0E-01	-0.1	1.0E+00	-0.1	9.0E-01	-0.2	8.3E-01
7237	THC2726213	A_24_P666340	THC2726213	0.9	3.59E-02	0.9	4.52E-02	1.7	6.16E-03	0.6	3.59E-02	0.4	8.10E-01	0.4	8.20E-01	0.8	4.83E-01	0.3	8.10E-01	0.0	1.0E+00	-0.1	1.0E+00	0.6	7.1E-01	0.0	1.0E+00	0.3	3.5E-01	0.0	1.0E+00	0.3	3.5E-01	0.5	1.7E-01
1830	ENST00000307533	A_23_P428992	ENST00000307533	0.9	3.82E-03	0.9	8.10E-03	1.1	3.82E-03	0.9	4.19E-04	0.7	2.61E-02	0.5	7.54E-02	0.9	2.61E-02	0.7	2.41E-02	0.0	1.0E+00	0.1	1.0E+00	0.0	9.5E-01	0.1	1.0E+00	0.0	9.9E-01	0.1	1.0E+00	0.0	9.5E-01	0.0	9.9E-01
8908	HSPB1	A_32_P76247	NM_001540	0.9	1.57E-03	0.8	1.57E-03	1.2	4.88E-03	0.9	2.86E-04	0.7	6.52E-02	0.5	8.52E-02	0.7	6.52E-02	0.7	5.34E-03	-0.1	1.0E+00	0.2	1.0E+00	-0.1	9.4E-01	-0.1	1.0E+00	0.1	9.7E-01	0.1	1.0E+00	0.0	9.5E-01	0.1	9.7E-01
33573	MARCO	A_23_P101992	NM_006770	0.9	7.80E-03	0.7	3.16E-02	1.6	7.80E-03	0.9	3.31E-03	-0.2	8.66E-01	-0.5	7.14E-01	-0.1	9.33E-01	-0.2	8.66E-01	-0.1	1.0E+00	-0.1	1.0E+00	0.1	8.8E-01	-0.2	1.0E+00	0.3	3.5E-01	0.0	1.0E+00	0.3	3.5E-01	0.4	1.8E-01
27241	MAP2K3	A_24_P296698	NM_145109	0.9	2.96E-02	0.8	2.96E-02	1.1	3.94E-02	0.9	4.90E-03	0.8	9.60E-02	0.7	2.06E-01	0.9	9.60E-02	0.8	2.51E-02	0.1	1.0E+00	0.2	1.0E+00	0.1	8.6E-01	0.1	1.0E+00	0.1	1.0E+00	0.2	1.0E+00	0.1	8.8E-01	0.0	1.0E+00
44823	DMBX1	A_23_P500542	NM_147192	0.9	1.84E-03	0.9	1.12E-03	1.2	1.84E-03	0.7	2.14E-03	0.5	4.03E-01	0.5	4.03E-01	0.3	6.39E-01	0.6	1.40E-01	0.0	1.0E+00	0.1	1.0E+00	0.0	9.6E-01	-0.1	1.0E+00	-0.1	9.1E-01	-0.1	1.0E+00	-0.1	7.3E-01	-0.1	9.1E-01
16365	LOC572558	A_32_P157208	NR_015423	0.9	1.57E-03	0.9	7.49E-03	1.3	1.57E-03	0.9	1.67E-04	0.9	3.06E-02	0.9	3.06E-02	0.5	1.42E-01	1.0	1.03E-02	0.1	1.0E+00	-0.1	1.0E+00	0.1	8.3E-01	0.5	1.0E+00	0.0	9.8E-01	0.0	1.0E+00	0.0	9.8E-01	-0.1	9.0E-01
37328	RGL1	A_23_P115417	NM_015149	0.9	6.29E-03	0.8	6.29E-03	1.1	1.66E-02	0.9	2.38E-03	0.9	8.17E-02	0.6	8.58E-02	1.2	8.17E-02	0.9	2.37E-03	0.1	1.0E+00	0.1	1.0E+00	0.1	8.7E-01	0.2	1.0E+00	-0.2	9.0E-01	0.0	1.0E+00	-0.3	5.3E-01	-0.2	9.0E-01
44729	TRPV6	A_23_P71170	NM_018646	0.9	1.83E-03	0.9	6.94E-04	1.4	1.83E-03	0.6	1.25E-02	0.6	1.87E-01	0.5	3.93E-01	1.0	1.87E-01	0.6	1.30E-01	0.2	1.0E+00	0.0	1.0E+00	0.2	7.1E-01	0.2	1.0E+00	0.0	9.9E-01	0.0	1.0E+00	0.0	9.9E-01	-0.1	9.3E-01
26902	SLC1A4	A_23_P91151	NM_003038	0.9	1.97E-02	0.6	2.85E-02	1.1	1.97E-02	0.9	1.69E-03	0.8	4.11E-02	0.5	3.41E-01	1.2	2.95E-02	0.8	4.11E-02	0.1	1.0E+00	0.0	1.0E+00	0.1	8.9E-01	0.1	1.0E+00	0.1	9.9E-01	0.2	1.0E+00	0.1	7.9E-01	0.0	9.9E-01
5228	FUT1	A_23_P107963	NM_000148	0.9	1.54E-03	0.9	3.89E-03	1.6	1.54E-03	0.9	2.94E-04	0.6	4.58E-02	0.5	1.17E-01	1.1	4.58E-02	0.6	3.01E-02	0.1	1.0E+00	0.1	1.0E+00	0.2	7.1E-01	0.1	1.0E+00	-0.2	7.1E-01	-0.2	1.0E+00	-0.3	2.5E-01	-0.2	7.1E-01
2302	ASL	A_23_P26223	NM_001024943	0.9	1.48E-02	0.9	1.48E-02	1.2	1.54E-02	0.8	7.03E-03	0.7	4.20E-02	0.7	4.45E-02	0.5	4.20E-02	0.8	7.11E-03	0.2	1.0E+00	0.3	1.0E+00	0.1	9.1E-01	0.2	1.0E+00	-0.1	8.7E-01	0.0	1.0E+00	-0.1	8.6E-01	-0.2	8.7E-01
27704	C7orf61	A_23_P93704	NM_001004323	0.9	8.81E-03	0.7	8.81E-03	1.1	7.15E-03	0.9	9.34E-03	0.0	8.04E-01	-0.4	8.04E-01	0.8	6.21E-01	0.0	9.70E-01	-0.1	1.0E+00	-0.1	1.0E+00	0.1	9.2E-01	-0.4	1.0E+00	0.3	6.9E-01	-0.2	1.0E+00	0.3	6.9E-01	0.6	3.3E-01
15152	A4GALT	A_23_P57570	NM_017436	0.9	9.55E-03	0.9	9.55E-03	2.1	2.15E-03	0.7	1.19E-02	0.5	4.32E-01	0.4	7.30E-01	0.7	2.98E-01	0.5	4.32E-01	0.1	1.0E+00	0.1	1.0E+00	-0.1	8.6E-01	0.2	1.0E+00	-0.3	7.2E-01	0.1	1.0E+00	-0.3	5.0E-01	-0.3	7.2E-01
11166	THC2570737	A_23_P208567	THC2570737	0.9	1.94E-03	1.4	3.80E-03	0.9	1.94E-03	0.9	1.02E-05	0.9	8.76E-02	1.0	9.56E-02	0.8	8.76E-02	0.9	3.48E-02	0.1	1.0E+00	0.1	1.0E+00	0.2	7.1E-01	0.1	1.0E+00	0.0	9.8E-01	0.0	1.0E+00	0.0	9.8E-01	0.0	9.6E-01
42117	GRK5	A_23_P12884	NM_005308	0.9	1.20E-02	1.0	1.21E-03	0.9	1.29E-02	0.6	1.20E-02	1.1	2.59E-03	1.2	1.92E-03	1.1	4.54E-03	1.1	2.59E-03	-0.1	1.0E+00	0.1	1.0E+00	-0.2	7.1E-01	-0.1	1.0E+00	-0.1	7.1E-01	-0.1	1.0E+00	-0.1	6.5E-01	-0.2	7.1E-01
9270	LYL1	A_23_P5281	NM_005583	0.9	9.02E-03	0.9	1.65E-02	1.4	9.02E-03	0.9	1.98E-03	0.7	1.51E-01	0.7	2.05E-01	0.7	1.51E-01	0.8	6.50E-02	0.3	9.7E-01	0.3	9.7E-01	0.4	5.3E-01	0.3	1.0E+00	0.1	8.7E-01	0.1	1.0E+00	0.1	8.7E-01	0.2	8.4E-01
7689	ABHD5	A_23_P250294	NM_016006	0.9	8.23E-04	0.9	8.23E-04	0.6	1.38E-02	1.1	1.36E-05	0.8	1.34E-02	0.8	1.34E-02	0.6	2.28E-01	1.0	2.81E-03	0.2	8.2E-01	0.3	8.2E-01	0.1	7.2E-01	0.2	1.0E+00	-0.1	6.5E-01	-0.1	1.0E+00	-0.1	6.5E-01	-0.3	3.2E-01
11275	FCHO1	A_32_P459533	NM_015122	0.9	1.91E-03	0.9	1.29E-03	1.5	1.91E-03	0.6	9.05E-03	0.8	2.62E-02	0.8	7.92E-02	0.9	2.17E-02	0.8	2.62E-02	0.0	1.0E+00	0.1	1.0E+00	0.0	1.0E+00	-0.1	1.0E+00	-0.1	8.9E-01	-0.1	1.0E+00	-0.1	8.9E-01	-0.1	8.9E-01
32371	MAST4	A_23_P110571	NM_015183	0.9	2.64E-03	0.8	6.95E-03	1.6	2.64E-03	0.9	5.07E-04	0.6	5.06E-02	0.5	2.63E-01	0.7	5.06E-02	0.6	4.74E-02	0.2	1.0E+00	0.1	1.0E+00	0.2	8.2E-01	0.3	1.0E+00	-0.2	9.3E-01	-0.1	1.0E+00	-0.2	7.7E-01	-0.2	9.3E-01
24844	MGC13057	A_23_P17130	NM_001042519	0.9	3.02E-03	0.8	3.02E-03	0.9	1.89E-02	1.0	6.58E-04	1.0	1.86E-02	1.0	1.86E-02	0.8	4.04E-02	1.0	4.80E-03	0.1	1.0E+00	0.1	1.0E+00	-0.1	8.1E-01	0.3	1.0E+00	-0.1	8.8E-01	0.0	1.0E+00	-0.2	4.2E-01	-0.1	8.8E-01
29072	TMEM86A	A_24_P175707	NM_153347	0.9	1.74E-02	0.7	3.09E-02	1.0	1.74E-02	0.9	5.99E-03	0.4	7.18E-01	-0.3	8.76E-01	0.7	5.35E-01	0.4	7.18E-01	0.1	1.0E+00	0.1	1.0E+00	0.2	7.7E-01	-0.2	1.0E+00	0.7	1.5E-01	0.0	1.0E+00	0.7	3.1E-02	0.7	1.5E-01
6880	CX3CL1	A_24_P390495	NM_002996	0.9	5.66E-03	0.9	5.66E-03	1.5	5.66E-03	0.9	1.40E-03	0.5	2.99E-01	0.5	2.99E-01	0.3	7.43E-01	0.9	2.39E-02	0.0	1.0E+00	0.1	1.0E+00	0.1	7.4E-01	0.0	1.0E+00	0.1	9.0E-01	-0.1	1.0E+00	0.1	8.2E-01	0.1	9.0E-01
33899	MYO15B	A_32_P475513	NR_003587	0.9	6.81E-03	0.9	5.16E-03	1.6	7.30E-03	0.8	6.81E-03	1.0	1.69E-02	0.8	7.27E-02	1.2	4.83E-03	1.0	1.69E-02	0.0	1.0E+00	0.0	1.0E+00	0.0	9.4E-01	0.1	1.0E+00	0.1	7.7E-01	0.1	1.0E+00	0.1	7.7E-01	0.1	7.5E-01
26931	NLRP12	A_23_P101434	NM_033297	0.9	1.56E-03	0.8	1.56E-03	1.1	2.54E-03	0.9	9.43E-05	0.8	8.24E-03	0.8	8.24E-03	0.8	1.98E-02	0.8	7.06E-03	0.1	1.0E+00	0.0	1.0E+00	0.1	7.9E-01	0.2	1.0E+00	0.0	9.7E-01	0.1	1.0E+00	0.0	9.7E-01	0.0	9.5E-01
27846	MAP3K6	A_32_P223017	NM_004672	0.9	9.29E-04	0.9	9.29E-04	1.3	2.75E-03	0.8	7.57E-04	0.9	1.96E-02	1.0	1.96E-02	0.9	2.20E-02	0.9	8.25E-03	0.2	1.0E+00	0.3	1.0E+00	0.2	7.1E-01	0.2	1.0E+00	0.1	7.7E-01	0.2	1.0E+00	0.1	5.9E-01	0.1	7.7E-01
27855	FZD5	A_23_P108437	NM_003468	0.9	1.65E-03	0.9	1.65E-03	0.9	1.73E-02	1.0	3.95E-04	0.9	2.68E-02	1.0	2.68E-02	0.4	7.54E-01	0.9	1.02E-02	0.2	1.0E+00	0.2	1.0E+00	0.2	7.1E-01	0.3	1.0E+00	0.0	9.8E-01	0.2	1.0E+00	0.0	9.8E-01	0.0	9.5E-01
13480	OR2B6	A_23_P70468	NM_012367	0.9	1.15E-03	1.0	1.15E-03	0.8	2.73E-03	0.9	7.31E-06	1.3	2.16E-02	1.4	3.75E-03	0.9	1.06E-01	1.3	2.16E-02	0.5	9.4E-01	0.3	1.0E+00	0.6	4.7E-01	0.5	9.4E-01	0.1	8.6E-01	0.1	1.0E+00	0.3	1.3E-01	0.1	8.6E-01
45183	C1orf226	A_23_P103511	NM_001085375	0.9	1.89E-02	0.7	2.64E-02	1.2	1.89E-02	0.9	3.91E-03	0.3	4.79E-01	0.3	4.79E-01	0.7	5.08E-02	0.3	4.88E-01	0.0	1.0E+00	0.1	1.0E+00	0.0	9.7E-01	0.0	1.0E+00	-0.3	7.1E-01	-0.1	1.0E+00	-0.3	5.1E-01	-0.4	7.1E-01
42172	RSPH1	A_23_P102950	NM_080860	0.9	6.99E-04	0.9	1.91E-02	1.8	6.99E-04	0.9	1.45E-04	1.2	2.95E-03	1.2	2.28E-03	1.6	2.95E-03	0.9	3.57E-02	0.1	1.0E+00	0.0	1.0E+00	0.1	8.8E-01	0.1	1.0E+00	0.0	9.9E-01	0.1	1.0E+00	-0.1	8.2E-01	0.0	9.9E-01
28658	HSH2D	A_23_P153372	NM_032855	0.9	2.62E-03	0.7	9.89E-03	1.3	2.62E-03	0.9	2.36E-04	0.6	2.37E-01	0.6	4.02E-01	0.9	1.06E-01	0.5	2.37E-01	0.2	1.0E+00	0.2	1.0E+00	0.2	7.3E-01	0.0	1.0E+00	0.1	9.0E-01	0.0	1.0E+00	0.1	9.0E-01	0.2	7.4E-01
6868	HBG1	A_23_P53137	NM_000559	0.9	1.24E-03	0.9	3.75E-02	1.5	1.24E-03	0.9	2.89E-04	0.5	6.75E-02	0.5	6.30E-02	0.5	7.01E-02	0.8	6.75E-02	-0.1	1.0E+00	0.0	1.0E+00	-0.1	8.6E-01	-0.1	1.0E+00	-0.1	9.0E-01	0.1	1.0E+00	-0.1	6.0E-01	-0.1	9.0E-01
5231	SLC29A3	A_23_P46871	NM_018344	0.9	3.64E-02	0.9	2.99E-02	1.2	4.57E-02	0.7	3.64E-02	1.0	4.43E-02	1.1	4.43E-02	0.8	1.08E-01	1.0	2.04E-02	0.2	1.0E+00	0.2	1.0E+00	0.3	6.6E-01	0.2	1.0E+00	0.0	9.8E-01	0.0	1.0E+00	0.0	9.8E-01	0.1	9.3E-01
10953	SEPT5	A_23_P29124	NM_002688	0.9	5.10E-03	0.9	3.28E-02	2.0	3.37E-03	0.9	5.10E-03	0.7	7.39E-02	0.7	7.39E-02	1.2	1.08E-01	0.7	2.70E-02	0.0	1.0E+00	0.1	1.0E+00	-0.1	9.0E-01	0.0	1.0E+00	0.0	9.7E-01	0.2	1.0E+00	0.0	9.7E-01	0.0	9.7E-01
19448	HLA-F	A_23_P314024	ENST00000376844	0.9	3.38E-03	0.9	3.38E-03	1.2	9.18E-03	0.8	2.49E-03	0.6	5.33E-02	0.6	4.98E-02	0.4	2.59E-01	0.6	5.33E-02	0.0	1.0E+00	0.0	1.0E+00	0.0	9.9E-01	0.0	1.0E+00	0.0	9.8E-01	0.0	1.0E+00	0.2	4.9E-01	0.0	9.8E-01
17490	PAQR6	A_23_P97283	NM_024897	0.9	1.94E-03	0.9	1.94E-03	1.1	3.78E-03	0.7	9.29E-04	0.4	2.82E-01	0.5	2.82E-01	0.4	4.17E-01	0.4	2.23E-01	0.0	1.0E+00	0.0	1.0E+00	0.1	8.9E-01	-0.1	1.0E+00	0.2	4.8E-01	0.2	1.0E+00	0.2	4.7E-01	0.3	4.8E-01
35874	PRRT3	A_23_P80626	NM_207351	0.9	4.79E-03	0.9	7.95E-03	1.6	2.45E-03	0.7	4.79E-03	1.2	5.07E-02	1.2	1.32E-02	1.4	5.07E-02	0.8	6.98E-02	0.0	1.0E+00	0.0	1.0E+00	-0.1	8.6E-01	0.0	1.0E+00	0.0	9.3E-01	0.0	1.0E+00	-0.1	8.8E-01	0.1	9.3E-01
14938	HERC6	A_23_P250353	NM_017912	1.0	3.22E-03	1.2	3.22E-03	1.0	5.35E-03	0.8	2.73E-04	1.2	1.61E-02	1.6	1.61E-02	1.0	3.19E-02	1.2	8.01E-03	0.0	1.0E+00	0.0	1.0E+00	-0.1	8.6E-01	0.2	1.0E+00	-0.2	7.5E-01	-0.1	1.0E+00	-0.2	5.8E-01	-0.2	7.5E-01
31003	OVOL2	A_23_P143348	NM_021220	1.0	1.38E-03	0.9	1.38E-03	1.5	1.71E-03	1.0	5.71E-04	0.9	1.62E-02	0.9	6.16E-02	0.9	1.62E-02	0.9	1.45E-02	0.1	1.0E+00	0.1	1.0E+00	0.1	7.2E-01	0.2	1.0E+00	-0.1	9.0E-01	0.0	1.0E+00	-0.2	4.5E-01	-0.1	9.0E-01
9462	PLEKHG2	A_23_P323563	NM_022835	1.0	2.90E-03	1.0	2.90E-03	0.8	3.82E-03	1.0	1.98E-05	0.7	4.97E-02	0.7	4.97E-02	0.7	1.49E-01	1.0	1.77E-03	0.2	1.0E+00	0.2	1.0E+00	-0.1	9.0E-01	0.3	1.0E+00	-0.1	9.0E-01	-0.1	1.0E+00	-0.2	6.3E-01	-0.1	9.0E-01
43059	FZD5	A_23_P397999	NM_003468	1.0	3.70E-04	0.8	3.70E-04	1.0	2.18E-03	1.1	7.16E-06	0.8	2.63E-02	0.6	2.18E-01	0.9	2.63E-02	0.8	1.94E-02	0.2	1.0E+00	0.2	1.0E+00	0.2	7.1E-01	0.2	1.0E+00	-0.1	8.9E-01	0.0	1.0E+00	-0.1	7.3E-01	-0.1	8.9E-01
35732	SMPD1	A_23_P203488	NM_000543	1.0	3.44E-02	0.7	4.80E-02	1.0	3.44E-02	1.0	2.74E-03	0.8	1.21E-01	0.8	1.21E-01	0.4	6.56E-01	1.3	3.82E-03	0.2	1.0E+00	0.3	1.0E+00	0.1	7.9E-01	0.2	1.0E+00	0.0	9.3E-01	0.0	1.0E+00	-0.1	9.1E-01	0.2	9.3E-01
13623	CRISPLD2	A_23_P106602	NM_031476	1.0	6.95E-04	1.0	6.95E-04	0.8	6.70E-03	1.2	2.75E-05	0.7	1.12E-01	0.7	1.12E-01	0.5	3.51E-01	0.9	7.18E-03	-0.1	1.0E+00	-0.1	1.0E+00	-0.1	8.4E-01	0.1	1.0E+00	-0.1	8.5E-01	0.0	1.0E+00	-0.2	4.0E-01	-0.1	8.5E-01
28020	MYCT1	A_23_P31046	NM_025107	1.0	8.37E-04	1.0	8.37E-04	0.8	2.19E-03	1.4	7.36E-07	0.5	3.46E-01	0.0	9.72E-01	0.5	3.25E-01	0.7	3.46E-01	-0.1	1.0E+00	0.3	1.0E+00	-0.1	8.3E-01	-0.1	1.0E+00	0.0	9.9E-01	-0.3	1.0E+00	0.1	7.7E-01	0.0	9.9E-01
20280	LOC129293	A_23_P56703	NM_001080824	1.0	3.14E-02	1.0	3.14E-02	1.8	1.65E-02	0.8	4.16E-02	0.6	1.98E-01	0.6	1.98E-01	0.6	1.43E-01	0.5	3.10E-01	0.0	1.0E+00	0.0	1.0E+00	0.0	9.2E-01	-0.1	1.0E+00	0.1	9.8E-01	0.3	1.0E+00	0.1	7.2E-01	0.0	9.8E-01
21263	CD163	A_23_P33723	NM_004244	1.0	6.94E-04	1.0	4.80E-03	1.7	6.24E-04	0.8	6.94E-04	0.5	3.53E-02	0.4	1.97E-01	0.5	3.53E-02	1.0	7.32E-03	0.0	1.0E+00	0.0	1.0E+00	0.0	9.5E-01	0.0	1.0E+00	0.2	4.5E-01	0.1	1.0E+00	0.2	1.6E-01	0.2	4.5E-01
41015	CRB3	A_24_P108517	NM_174881	1.0	3.35E-03	1.0	3.35E-03	1.3	6.95E-03	1.0	9.98E-04	0.6	2.78E-01	0.6	2.78E-01	0.4	4.81E-01	0.8	4.00E-02	0.1	1.0E+00	0.1	1.0E+00	0.3	7.1E-01	0.1	1.0E+00	0.0	1.0E+00	0.0	1.0E+00	0.0	9.3E-01	0.0	1.0E+00
3189	ISM1	A_32_P186731	NM_080826	1.0	4.68E-03	0.7	9.29E-03	1.4	4.68E-03	1.0	1.00E-03	0.7	1.00E-01	0.7	1.66E-01	1.0	1.00E-01	0.6	7.53E-02	-0.1	1.0E+00	0.1	1.0E+00	-0.2	7.1E-01	-0.1	1.0E+00	-0.2	7.6E-01	0.0	1.0E+00	-0.2	7.3E-01	-0.3	7.6E-01
2056	LOC339240	A_32_P46238	NR_001443	1.0	1.10E-02	1.0	2.95E-02	1.4	1.08E-02	0.7	1.10E-02	0.6	6.67E-01	0.6	6.67E-01	0.6	4.80E-01	0.4	6.67E-01	0.1	1.0E+00	0.1	1.0E+00	0.5	7.1E-01	0.1	1.0E+00	0.3	1.6E-01	0.0	1.0E+00	0.3	1.1E-01	0.4	1.6E-01
32348	AK027091	A_23_P399292	AK027091	1.0	1.08E-03	1.1	1.08E-03	0.7	2.83E-02	1.0	5.49E-04	0.6	3.47E-01	0.6	3.47E-01	0.2	8.81E-01	1.0	8.60E-02	0.2	1.0E+00	0.3	1.0E+00	0.0	9.6E-01	0.2	1.0E+00	0.4	7.2E-01	0.3	1.0E+00	0.4	4.3E-01	0.4	7.2E-01
37926	RHBDL3	A_23_P308731	NM_138328	1.0	9.71E-04	1.0	9.88E-03	1.9	9.71E-04	0.9	4.14E-04	0.8	1.38E-01	0.5	1.38E-01	1.0	1.81E-01	0.8	5.36E-02	0.0	1.0E+00	0.1	1.0E+00	-0.2	7.3E-01	0.0	1.0E+00	-0.1	8.7E-01	0.2	1.0E+00	-0.1	8.6E-01	-0.1	8.7E-01
35901	GPX3	A_23_P133474	NM_002084	1.0	2.27E-02	0.6	4.11E-02	1.0	2.27E-02	1.0	1.74E-03	1.0	4.63E-02	0.8	1.55E-01	1.0	4.63E-02	1.2	6.53E-03	0.1	1.0E+00	0.2	1.0E+00	-0.2	8.0E-01	0.1	1.0E+00	-0.2	9.1E-01	-0.1	1.0E+00	-0.3	5.5E-01	-0.2	9.1E-01
40190	CNTFR	A_23_P9402	NM_147164	1.0	1.80E-03	1.0	1.67E-02	1.0	1.80E-03	0.9	4.05E-05	0.9	3.45E-03	1.0	3.45E-03	0.6	7.45E-02	0.9	2.21E-03	0.1	1.0E+00	0.1	1.0E+00	0.1	7.2E-01	0.1	1.0E+00	-0.2	4.0E-01	-0.1	1.0E+00	-0.2	1.4E-01	-0.2	4.0E-01
23985	FAM104A	A_24_P91232	NM_032837	1.0	1.87E-03	1.0	1.87E-03	1.3	6.41E-03	1.0	8.02E-04	0.7	1.89E-01	0.7	1.89E-01	0.6	2.46E-01	0.9	2.58E-02	0.0	1.0E+00	0.1	1.0E+00	0.0	9.9E-01	0.0	1.0E+00	0.0	9.5E-01	0.2	1.0E+00	0.0	9.5E-01	-0.1	9.0E-01
37675	STMN4	A_23_P31739	NM_030795	1.0	8.52E-04	0.8	8.52E-04	1.1	1.86E-03	1.0	8.16E-04	0.2	8.05E-01	-0.4	8.05E-01	0.3	8.14E-01	0.2	8.02E-01	0.0	1.0E+00	0.0	1.0E+00	0.0	9.7E-01	-0.1	1.0E+00	0.2	7.1E-01	0.0	1.0E+00	0.2	5.1E-01	0.2	7.1E-01
37687	TEX14	A_32_P126079	NM_031272	1.0	7.19E-04	1.0	7.19E-04	0.8	3.95E-03	1.4	9.07E-06	0.7	2.72E-02	0.7	2.72E-02	0.7	9.56E-02	1.1	4.16E-04	0.0	1.0E+00	0.0	1.0E+00	0.0	9.9E-01	0.0	1.0E+00	0.0	9.4E-01	0.0	1.0E+00	-0.1	3.9E-01	0.0	9.4E-01
39033	SPACA4	A_32_P101573	NM_133498	1.0	1.24E-04	1.0	1.24E-04	1.0	7.92E-04	0.9	8.84E-06	0.7	2.63E-02	0.7	2.63E-02	0.6	6.89E-02	0.8	5.47E-03	0.0	1.0E+00	0.0	1.0E+00	0.1	7.8E-01	-0.1	1.0E+00	0.0	8.9E-01	-0.1	1.0E+00	0.0	8.6E-01	0.1	8.9E-01
42454	TMEM35	A_23_P45324	NM_021637	1.0	1.80E-03	1.0	6.87E-03	0.9	1.80E-03	1.1	3.97E-05	0.5	5.26E-02	0.5	5.26E-02	0.5	3.09E-02	0.7	8.94E-02	0.1	1.0E+00	0.1	1.0E+00	0.1	7.7E-01	0.0	1.0E+00	-0.1	7.8E-01	0.1	1.0E+00	-0.2	3.8E-01	-0.1	7.8E-01
32721	THC2701341	A_24_P454931	THC2701341	1.0	4.27E-03	1.0	4.27E-03	0.8	5.49E-03	1.0	2.54E-05	1.1	3.23E-02	1.1	3.23E-02	1.1	6.30E-02	1.0	9.12E-03	0.1	1.0E+00	0.1	1.0E+00	0.1	8.3E-01	0.2	1.0E+00	-0.2	7.0E-01	0.0	1.0E+00	-0.2	3.5E-01	-0.2	7.0E-01
11489	C1orf190	A_32_P129010	NM_001013615	1.0	4.03E-03	1.0	3.35E-02	1.9	2.45E-03	0.8	4.03E-03	0.8	1.36E-01	0.8	2.06E-01	1.1	1.22E-01	0.7	1.36E-01	0.1	1.0E+00	0.2	1.0E+00	-0.1	8.6E-01	0.1	1.0E+00	-0.3	4.6E-01	0.1	1.0E+00	-0.3	3.3E-01	-0.4	4.6E-01
6391	TMC6	A_23_P101013	NM_007267	1.0	1.96E-02	1.0	1.17E-02	1.3	1.96E-02	0.7	3.55E-02	0.7	4.10E-02	0.7	7.32E-02	0.8	1.15E-02	0.5	4.10E-02	0.3	1.0E+00	0.3	1.0E+00	0.3	7.1E-01	0.3	1.0E+00	0.3	6.5E-01	0.1	1.0E+00	0.3	5.4E-01	0.3	6.5E-01
10267	THC2631248	A_32_P2050	THC2631248	1.0	2.20E-04	1.0	1.87E-04	1.1	2.03E-03	0.9	2.20E-04	0.8	4.72E-02	0.8	4.72E-02	0.6	1.08E-01	1.2	1.02E-03	0.0	1.0E+00	0.0	1.0E+00	-0.1	8.2E-01	0.1	1.0E+00	0.1	9.1E-01	0.0	1.0E+00	0.1	9.1E-01	0.1	8.7E-01
19687	THC2771832	A_32_P114918	THC2771832	1.0	3.18E-02	0.8	4.57E-02	1.4	3.18E-02	1.0	1.01E-02	0.9	9.08E-02	0.8	2.30E-01	1.1	9.08E-02	0.9	7.74E-02	-0.1	1.0E+00	-0.1	1.0E+00	-0.1	8.9E-01	0.0	1.0E+00	-0.2	9.0E-01	-0.2	1.0E+00	-0.2	6.6E-01	-0.2	9.0E-01
15274	AK1	A_23_P217088	NM_000476	1.0	8.85E-04	1.0	5.20E-03	1.4	8.85E-04	0.8	1.19E-04	0.7	5.37E-02	0.8	4.39E-02	0.7	7.26E-02	0.7	5.37E-02	0.0	1.0E+00	0.1	1.0E+00	0.0	9.5E-01	0.0	1.0E+00	0.0	9.7E-01	0.0	1.0E+00	0.0	9.4E-01	0.1	9.7E-01
10971	SQRDL	A_23_P3221	NM_021199	1.0	8.78E-03	0.8	1.83E-02	1.3	8.78E-03	1.0	2.62E-03	0.7	4.43E-02	0.7	5.62E-02	0.8	1.25E-02	0.6	4.43E-02	0.3	6.5E-01	0.4	3.4E-01	0.2	6.5E-01	0.3	1.0E+00	-0.2	7.0E-01	0.0	1.0E+00	-0.2	6.3E-01	-0.3	7.0E-01
29202	RAB36	A_23_P103011	NM_004914	1.0	2.46E-02	0.7	4.74E-02	1.5	2.46E-02	1.0	8.56E-03	0.8	1.52E-02	0.7	5.64E-02	1.1	1.52E-02	0.8	7.63E-03	0.1	1.0E+00	0.1	1.0E+00	0.2	7.2E-01	0.0	1.0E+00	0.0	9.7E-01	0.1	1.0E+00	0.0	9.5E-01	0.0	9.7E-01
39829	MGC52282	A_23_P350719	BC042597	1.0	5.36E-04	0.9	5.43E-04	1.5	5.36E-04	1.0	1.84E-05	0.8	1.29E-03	0.7	1.06E-02	1.0	1.29E-03	0.8	1.22E-03	0.0	1.0E+00	0.0	1.0E+00	0.1	7.3E-01	0.0	1.0E+00	0.1	9.9E-01	0.1	1.0E+00	0.0	9.9E-01	0.1	8.9E-01
7165	MAP1LC3B	A_32_P220715	NM_022818	1.0	3.04E-03	1.0	3.04E-03	1.0	2.28E-02	1.2	2.45E-04	0.6	3.28E-02	0.5	1.13E-01	0.6	3.28E-02	0.9	4.96E-03	0.2	1.0E+00	0.1	1.0E+00	0.2	7.1E-01	0.2	1.0E+00	-0.2	8.1E-01	-0.2	1.0E+00	-0.2	7.3E-01	-0.3	8.1E-01
36772	PAIP2B	A_23_P301051	NM_020459	1.0	8.89E-03	1.0	8.89E-03	1.8	4.96E-03	0.7	1.14E-02	1.0	4.88E-02	1.0	5.62E-02	1.3	4.88E-02	1.0	4.40E-02	0.0	1.0E+00	-0.1	1.0E+00	0.0	9.5E-01	0.2	1.0E+00	-0.6	4.5E-01	-0.3	1.0E+00	-0.6	2.5E-01	-0.6	4.5E-01
28374	DLG4	A_23_P411102	NM_001365	1.0	2.03E-03	1.0	6.78E-03	2.2	6.65E-04	0.9	2.03E-03	0.9	9.35E-02	1.0	4.33E-02	0.9	2.94E-01	0.8	9.35E-02	0.0	1.0E+00	-0.1	1.0E+00	0.0	1.0E+00	0.0	1.0E+00	0.0	9.8E-01	0.1	1.0E+00	0.0	9.8E-01	0.0	9.8E-01
26399	AMIGO3	A_23_P306655	ENST00000320431	1.0	3.51E-03	1.0	3.51E-03	1.4	3.70E-03	0.9	5.13E-04	0.8	1.61E-01	0.5	3.44E-01	0.8	1.61E-01	0.8	5.79E-02	0.0	1.0E+00	0.0	1.0E+00	-0.1	8.5E-01	0.2	1.0E+00	0.0	9.6E-01	0.0	1.0E+00	0.0	9.2E-01	0.0	9.6E-01
35460	ZFAND5	A_24_P278167	NM_006007	1.0	8.40E-03	0.8	8.40E-03	1.0	1.57E-02	1.0	6.53E-04	0.8	4.62E-02	0.2	4.41E-01	0.9	4.62E-02	0.8	1.50E-02	-0.1	1.0E+00	0.0	1.0E+00	-0.1	8.1E-01	-0.2	1.0E+00	-0.1	1.0E+00	-0.1	1.0E+00	-0.1	8.6E-01	0.0	1.0E+00
42958	MXD4	A_24_P234732	NM_006454	1.0	2.53E-03	1.0	2.53E-03	1.0	6.46E-03	0.7	9.15E-04	0.9	1.21E-01	0.8	1.21E-01	0.9	1.23E-01	1.1	9.33E-03	0.2	1.0E+00	0.2	1.0E+00	0.2	7.1E-01	0.3	1.0E+00	-0.1	8.9E-01	0.1	1.0E+00	-0.3	4.9E-01	-0.1	8.9E-01
30228	VTI1A	A_23_P10785	NM_145206	1.0	1.61E-03	1.0	1.61E-03	0.9	3.63E-03	1.1	2.78E-05	0.7	1.19E-01	0.6	1.19E-01	0.9	1.95E-01	0.7	5.96E-02	-0.1	1.0E+00	-0.1	1.0E+00	-0.2	7.2E-01	-0.1	1.0E+00	0.0	9.7E-01	-0.1	1.0E+00	0.0	9.7E-01	0.0	9.7E-01
31353	WIPF1	A_23_P330611	NM_001077269	1.0	9.71E-04	0.7	7.51E-03	1.2	9.71E-04	1.0	1.62E-05	0.6	3.27E-03	0.5	5.97E-02	0.8	3.27E-03	0.6	3.05E-03	0.0	1.0E+00	0.0	1.0E+00	0.0	9.1E-01	0.0	1.0E+00	0.0	8.9E-01	0.2	1.0E+00	0.0	8.6E-01	-0.1	8.9E-01
17921	CTA-216E10.6	A_23_P132341	NM_001142964	1.0	5.42E-03	0.9	7.21E-03	1.1	5.42E-03	1.0	2.20E-04	0.6	2.62E-01	0.6	2.62E-01	0.5	4.23E-01	0.8	4.54E-02	0.1	1.0E+00	0.0	1.0E+00	0.1	8.4E-01	0.1	1.0E+00	0.0	9.7E-01	0.0	1.0E+00	0.1	8.9E-01	0.0	9.7E-01
44748	A_32_P45727	A_32_P45727	A_32_P45727	1.0	5.94E-04	1.0	4.07E-04	1.4	8.35E-04	0.6	5.94E-04	0.2	7.79E-01	-0.1	9.62E-01	0.3	6.89E-01	0.2	7.79E-01	0.1	1.0E+00	0.1	1.0E+00	0.1	8.8E-01	-0.1	1.0E+00	0.0	9.7E-01	0.1	1.0E+00	0.0	9.6E-01	0.0	9.7E-01
37351	TEKT2	A_23_P45955	NM_014466	1.0	6.12E-04	1.0	2.87E-03	1.3	6.12E-04	1.0	1.38E-05	0.8	3.12E-02	0.7	1.01E-01	0.9	3.12E-02	0.8	6.56E-03	0.0	1.0E+00	0.0	1.0E+00	0.0	9.9E-01	0.0	1.0E+00	0.0	9.8E-01	-0.1	1.0E+00	0.0	8.4E-01	0.0	9.8E-01

Feature	Gene symbol	Probe	Transcript	72 hour								48 hours								24 hours								10 hours							
				siCASP8AP2								siCASP8AP2								siCASP8AP2								siCASP8AP2							
				Median	Median	1	1	3	3	6	6	Median	Median	1	1	3	3	6	6	Median	Median	1	1	3	3	6	6	Median	Median	1	1	3	3	6	6
				M	q	M	q	M	q	M	q	M	q	M	q	M	q	M	q	M	q	M	q	M	q	M	q	M	q	M	q	M	q	M	q
9973	ZER1	A_23_P146631	NM_006336	1.0	8.32E-03	1.1	8.32E-03	0.8	3.91E-02	1.0	6.96E-04	1.1	1.40E-01	1.1	1.40E-01	0.4	6.67E-01	1.2	3.40E-02	0.3	9.9E-01	0.3	5.8E-01	0.0	9.9E-01	0.3	1.0E+00	-0.4	5.6E-01	-0.2	1.0E+00	-0.4	2.9E-01	-0.4	5.6E-01
12845	BC047708	A_24_P655833	BC047708	1.0	1.13E-02	0.7	1.13E-02	1.0	1.13E-02	1.5	2.76E-05	0.9	1.17E-01	0.8	1.40E-01	0.9	1.17E-01	0.9	3.92E-02	-0.1	1.0E+00	-0.1	1.0E+00	-0.3	7.1E-01	0.1	1.0E+00	-0.2	4.8E-01	-0.1	1.0E+00	-0.2	3.6E-01	-0.3	4.8E-01
37407	THC2495068	A_24_P227350	THC2495068	1.0	7.58E-04	1.0	7.58E-04	0.8	1.15E-02	1.0	7.89E-05	1.1	8.71E-03	1.2	8.71E-03	0.6	2.42E-01	1.1	1.41E-03	0.1	1.0E+00	0.2	1.0E+00	-0.1	8.7E-01	0.1	1.0E+00	0.1	7.5E-01	0.1	1.0E+00	-0.1	6.7E-01	0.2	7.5E-01
44666	SLC37A1	A_23_P17695	NM_018964	1.0	1.32E-02	1.0	1.32E-02	1.1	1.66E-02	0.9	2.31E-03	0.7	3.39E-01	0.7	3.39E-01	0.2	7.63E-01	0.7	1.73E-01	0.0	1.0E+00	0.3	1.0E+00	-0.1	8.0E-01	0.0	1.0E+00	-0.1	9.3E-01	0.0	1.0E+00	-0.2	6.6E-01	-0.1	9.3E-01
19444	LGI4	A_24_P310256	NM_139284	1.0	8.89E-04	1.2	8.89E-04	1.0	2.54E-03	0.8	7.89E-05	0.7	2.20E-01	0.7	2.20E-01	0.5	2.79E-01	0.7	7.28E-02	0.0	1.0E+00	-0.1	1.0E+00	0.3	7.1E-01	0.0	1.0E+00	0.1	8.6E-01	0.1	1.0E+00	0.2	2.5E-01	0.1	8.6E-01
45082	CLDN9	A_23_P54809	NM_020982	1.0	2.49E-03	1.1	2.49E-03	0.9	2.75E-02	1.0	2.24E-03	0.3	8.31E-01	-0.3	9.06E-01	0.6	7.33E-01	0.3	8.31E-01	0.0	1.0E+00	0.0	1.0E+00	0.5	7.4E-01	-0.2	1.0E+00	0.3	6.4E-01	0.1	1.0E+00	0.3	6.4E-01	0.5	4.5E-01
20021	SIRPB1	A_24_P143574	NM_006065	1.0	4.77E-03	0.7	9.40E-03	1.0	4.77E-03	1.1	6.19E-05	0.5	2.27E-01	0.4	4.97E-01	0.5	2.27E-01	0.8	3.11E-02	0.1	1.0E+00	0.1	1.0E+00	0.1	7.1E-01	0.0	1.0E+00	0.1	9.9E-01	0.1	1.0E+00	0.1	8.2E-01	0.0	9.9E-01
15390	PAGE4	A_24_P254506	NM_007003	1.0	1.37E-02	1.0	4.34E-02	1.0	1.37E-02	1.0	3.26E-04	0.6	1.01E-01	0.6	8.19E-02	0.4	1.85E-01	0.6	1.01E-01	0.0	1.0E+00	0.0	1.0E+00	-0.1	8.7E-01	0.0	1.0E+00	-0.1	8.2E-01	0.0	1.0E+00	-0.2	3.3E-01	-0.1	8.2E-01
37943	C9orf53	A_23_P307502	NR_024274	1.0	2.35E-03	0.7	3.35E-03	1.6	6.58E-04	1.0	2.35E-03	0.6	3.58E-01	0.0	9.91E-01	1.4	6.81E-02	0.6	3.58E-01	0.0	1.0E+00	0.0	1.0E+00	0.1	9.0E-01	-0.1	1.0E+00	0.3	5.8E-01	0.1	1.0E+00	0.3	5.8E-01	0.6	3.9E-01
20584	IL17D	A_23_P345692	NM_138284	1.0	9.18E-03	1.0	9.18E-03	1.2	3.11E-02	0.9	6.34E-03	1.2	5.47E-03	1.2	5.47E-03	1.3	5.45E-03	0.9	7.10E-03	-0.1	1.0E+00	-0.1	1.0E+00	-0.2	7.2E-01	0.1	1.0E+00	0.1	9.5E-01	0.1	1.0E+00	0.1	8.8E-01	-0.1	9.5E-01
7779	C11orf75	A_23_P75430	NM_020179	1.0	7.48E-04	1.0	7.40E-04	1.1	6.09E-03	0.8	7.48E-04	0.9	1.39E-02	0.9	3.63E-02	0.8	1.39E-02	0.9	3.66E-03	0.3	1.0E+00	0.2	1.0E+00	0.3	6.9E-01	0.4	1.0E+00	-0.3	4.6E-01	0.0	1.0E+00	-0.3	2.6E-01	-0.3	4.6E-01
35715	A_24_P755069	A_24_P755069	A_24_P755069	1.0	8.64E-04	1.3	8.64E-04	0.9	2.78E-02	1.0	7.45E-04	1.1	4.06E-02	1.1	3.91E-02	0.2	8.88E-01	1.1	4.06E-02	0.0	1.0E+00	0.0	1.0E+00	-0.1	8.6E-01	0.1	1.0E+00	0.1	1.0E+00	0.1	1.0E+00	0.1	9.6E-01	0.0	1.0E+00
25999	RASL10A	A_23_P166400	NM_001007279	1.0	8.03E-03	0.8	1.20E-02	1.4	8.03E-03	1.0	1.57E-03	1.1	3.47E-03	0.9	1.11E-02	1.3	3.47E-03	1.1	1.28E-03	0.1	1.0E+00	0.1	1.0E+00	0.0	1.0E+00	0.1	1.0E+00	0.2	5.9E-01	0.3	1.0E+00	0.2	5.2E-01	0.2	5.9E-01
2423	CYP3A7	A_23_P358917	NM_000765	1.0	4.30E-03	0.7	2.03E-02	1.3	4.30E-03	1.0	5.66E-04	0.7	7.67E-02	0.5	2.20E-01	0.9	7.67E-02	0.7	2.84E-02	0.1	1.0E+00	0.1	1.0E+00	0.1	8.7E-01	0.2	1.0E+00	-0.2	8.2E-01	-0.1	1.0E+00	-0.3	3.5E-01	-0.2	8.2E-01
16040	ADM	A_23_P127948	NM_001124	1.0	5.14E-03	0.9	5.14E-03	1.0	2.76E-02	1.3	2.12E-04	0.6	8.75E-02	0.6	8.75E-02	0.5	9.42E-02	0.7	3.17E-02	0.0	1.0E+00	0.0	1.0E+00	0.0	9.6E-01	-0.1	1.0E+00	0.0	9.8E-01	-0.1	1.0E+00	0.0	9.5E-01	0.0	9.8E-01
17735	A_32_P35294	A_32_P35294	A_32_P35294	1.0	4.09E-03	1.0	4.09E-03	0.8	2.15E-02	1.7	4.24E-05	0.4	5.62E-01	0.4	5.62E-01	0.3	6.24E-01	0.8	1.47E-01	0.2	1.0E+00	0.2	1.0E+00	-0.1	9.1E-01	0.3	1.0E+00	-0.1	9.2E-01	0.2	1.0E+00	-0.2	6.3E-01	-0.1	9.2E-01
35807	PSCA	A_23_P71379	NM_005672	1.0	3.06E-03	1.0	1.96E-02	1.7	1.13E-03	0.7	3.06E-03	0.8	8.41E-02	0.7	8.41E-02	1.3	1.40E-01	0.8	1.84E-02	0.0	1.0E+00	0.1	1.0E+00	-0.2	7.1E-01	0.0	1.0E+00	-0.1	8.5E-01	0.0	1.0E+00	-0.2	6.0E-01	-0.1	8.5E-01
40129	ABTB1	A_23_P109643	NM_172027	1.0	1.32E-02	1.0	1.61E-02	1.8	1.32E-02	1.0	1.02E-02	0.8	2.48E-01	0.4	5.86E-01	0.8	2.48E-01	0.8	8.26E-02	0.1	1.0E+00	0.1	1.0E+00	0.5	7.1E-01	0.0	1.0E+00	0.1	9.7E-01	-0.1	1.0E+00	0.1	8.6E-01	0.1	9.7E-01
41465	SLC44A2	A_23_P208340	NM_020428	1.0	6.57E-03	1.0	6.57E-03	1.2	2.41E-02	1.0	2.87E-03	0.4	5.99E-02	0.6	3.20E-02	0.4	2.32E-01	0.4	5.99E-02	0.3	1.0E+00	0.4	1.0E+00	0.1	9.0E-01	0.3	1.0E+00	-0.3	8.8E-01	-0.3	1.0E+00	-0.2	8.2E-01	-0.3	8.8E-01
40040	HSPB1	A_24_P86537	NM_001540	1.0	5.74E-03	0.9	5.74E-03	1.3	1.05E-02	1.0	1.15E-03	0.6	1.54E-01	0.5	2.12E-01	0.6	1.54E-01	0.8	9.11E-03	-0.1	1.0E+00	0.2	1.0E+00	-0.1	9.4E-01	-0.2	1.0E+00	0.1	9.2E-01	0.1	1.0E+00	-0.1	9.1E-01	0.1	9.2E-01
24510	SLC9A3R1	A_23_P308519	AF015926	1.0	3.21E-03	1.0	3.21E-03	0.9	2.82E-02	1.2	3.91E-04	1.0	2.20E-02	1.0	2.20E-02	0.8	5.75E-02	1.1	4.04E-03	0.1	1.0E+00	0.2	1.0E+00	0.1	8.2E-01	0.1	1.0E+00	0.1	8.7E-01	0.1	1.0E+00	0.2	6.1E-01	0.1	8.7E-01
31352	S1PR2	A_32_P827528	NM_004230	1.0	1.92E-02	1.0	1.13E-02	1.0	3.47E-02	0.6	1.92E-02	0.9	2.67E-02	1.0	2.67E-02	0.9	1.33E-01	0.9	6.70E-03	0.1	1.0E+00	0.1	1.0E+00	-0.1	8.4E-01	0.1	1.0E+00	0.0	9.7E-01	0.0	1.0E+00	-0.1	6.5E-01	0.0	9.7E-01
21093	THC2651395	A_32_P115451	THC2651395	1.0	4.68E-03	1.0	5.58E-03	1.2	4.68E-03	1.0	2.77E-04	0.7	1.12E-01	0.7	1.45E-01	0.6	1.12E-01	0.7	4.86E-02	0.0	1.0E+00	0.0	1.0E+00	-0.3	7.3E-01	0.0	1.0E+00	-0.3	8.1E-01	-0.1	1.0E+00	-0.3	5.9E-01	-0.3	8.1E-01
5031	LOC100131170	A_24_P8151	AK096606	1.0	2.82E-03	1.0	5.38E-03	1.4	2.82E-03	1.0	4.53E-04	1.0	8.63E-02	0.8	2.65E-01	1.1	8.63E-02	1.0	7.13E-02	0.1	1.0E+00	0.1	1.0E+00	0.0	9.4E-01	0.3	1.0E+00	-0.3	3.7E-01	0.0	1.0E+00	-0.3	2.0E-01	-0.3	3.7E-01
42065	MEP1A	A_23_P93122	NM_005588	1.0	4.32E-03	0.7	1.03E-02	1.0	4.32E-03	1.1	7.30E-05	0.5	2.47E-01	0.3	4.96E-01	0.5	2.47E-01	0.6	2.11E-02	0.0	1.0E+00	0.0	1.0E+00	0.1	7.7E-01	0.0	1.0E+00	0.0	9.6E-01	0.0	1.0E+00	0.1	5.2E-01	0.0	9.6E-01
14596	CR596670	A_23_P97218	CR596670	1.0	2.47E-03	1.0	2.47E-03	0.9	1.69E-02	1.0	3.28E-04	1.0	1.38E-02	1.0	1.38E-02	0.8	2.70E-02	1.0	2.09E-03	0.3	1.0E+00	0.2	1.0E+00	0.3	7.1E-01	0.4	1.0E+00	-0.1	8.6E-01	-0.1	1.0E+00	-0.1	8.6E-01	-0.2	7.2E-01
22621	ADM	A_23_P127948	NM_001124	1.0	5.78E-03	0.8	5.78E-03	1.0	2.67E-02	1.2	2.76E-04	0.4	8.61E-02	0.4	1.69E-01	0.4	8.61E-02	0.6	3.58E-02	0.1	1.0E+00	0.2	1.0E+00	0.0	9.8E-01	0.1	1.0E+00	-0.1	8.2E-01	-0.1	1.0E+00	-0.1	8.0E-01	-0.1	8.2E-01
38279	NAGS	A_32_P32739	NM_153006	1.0	7.50E-03	0.8	3.03E-02	1.7	7.50E-03	1.0	2.43E-03	0.8	1.03E-01	0.7	1.03E-01	1.0	1.11E-01	0.8	2.22E-02	0.2	1.0E+00	0.2	1.0E+00	0.2	7.7E-01	0.0	1.0E+00	0.0	9.8E-01	0.1	1.0E+00	0.0	9.7E-01	0.0	9.8E-01
18168	RNF24	A_24_P333019	NM_007219	1.0	7.20E-03	0.9	7.20E-03	1.0	3.41E-02	1.1	1.66E-03	0.8	2.82E-02	0.8	2.88E-02	0.8	2.82E-02	1.0	1.81E-03	0.2	1.0E+00	0.2	1.0E+00	0.3	7.1E-01	0.2	1.0E+00	0.2	2.7E-01	0.2	1.0E+00	0.2	2.7E-01	0.4	1.8E-01
28776	PPP1R13B	A_23_P205273	NM_015316	1.0	3.39E-03	1.0	3.39E-03	1.1	6.56E-03	1.0	1.38E-04	1.0	1.90E-02	0.9	4.76E-02	1.0	1.90E-02	1.0	9.09E-03	0.1	1.0E+00	0.2	1.0E+00	0.0	9.8E-01	0.1	1.0E+00	0.0	9.8E-01	0.0	1.0E+00	0.0	9.8E-01	0.1	9.2E-01
18353	CYP27B1	A_23_P36397	NM_000785	1.0	2.97E-03	1.0	2.97E-03	1.1	1.54E-02	1.0	7.73E-04	0.7	2.84E-01	0.7	2.84E-01	0.4	6.52E-01	0.8	8.15E-02	0.1	1.0E+00	0.1	1.0E+00	0.1	7.1E-01	0.1	1.0E+00	0.1	7.9E-01	0.0	1.0E+00	0.1	6.6E-01	0.2	7.9E-01
5155	IFI27L1	A_24_P261929	NM_206949	1.0	1.48E-03	0.8	1.38E-02	1.7	1.48E-03	1.0	2.77E-04	0.7	4.69E-02	0.5	4.69E-02	0.8	7.70E-02	0.7	3.84E-02	0.0	1.0E+00	0.1	1.0E+00	0.0	9.6E-01	-0.1	1.0E+00	0.1	9.7E-01	0.1	1.0E+00	-0.1	8.6E-01	0.1	9.7E-01
36236	ASPHD1	A_23_P170667	NM_181718	1.0	2.11E-03	1.0	1.24E-02	2.3	1.84E-03	1.0	2.11E-03	0.8	1.59E-01	0.4	3.06E-01	1.0	1.59E-01	0.8	1.75E-02	-0.1	1.0E+00	0.1	1.0E+00	-0.1	9.0E-01	-0.1	1.0E+00	0.0	1.0E+00	0.1	1.0E+00	-0.1	8.7E-01	0.0	1.0E+00
43135	HBEGF	A_23_P213944	NM_001945	1.0	4.09E-02	0.7	4.09E-02	1.0	4.45E-02	1.1	1.31E-03	0.6	2.98E-01	0.2	7.95E-01	0.8	2.98E-01	0.6	5.93E-02	0.2	1.0E+00	0.2	1.0E+00	0.2	7.4E-01	0.1	1.0E+00	0.0	9.0E-01	0.0	1.0E+00	0.0	9.0E-01	0.2	8.3E-01
29368	SLC2A8	A_23_P252783	NM_014580	1.0	5.58E-03	1.0	5.60E-03	1.7	4.01E-03	0.7	5.58E-03	0.9	2.21E-02	0.9	2.24E-02	1.3	1.55E-02	0.7	2.21E-02	0.2	1.0E+00	0.2	1.0E+00	0.2	7.1E-01	0.1	1.0E+00	0.2	9.7E-01	0.2	1.0E+00	0.2	6.0E-01	0.0	9.7E-01
16545	HAR1A	A_32_P226498	NR_003244	1.0	1.43E-03	0.9	1.99E-02	1.8	1.43E-03	1.0	4.18E-04	0.3	7.88E-01	0.2	8.46E-01	0.3	7.88E-01	0.6	4.12E-01	-0.2	1.0E+00	-0.2	1.0E+00	0.6	7.1E-01	-0.3	1.0E+00	0.4	4.0E-01	0.2	1.0E+00	0.7	1.0E-02	0.4	4.0E-01
28425	HLA-E	A_24_P326082	NM_005516	1.0	5.72E-03	1.0	5.72E-03	1.2	1.40E-02	1.0	1.38E-03	0.6	4.24E-02	0.6	4.24E-02	0.4	4.08E-01	0.7	1.46E-02	0.1	1.0E+00	0.1	1.0E+00	0.1	8.7E-01	0.1	1.0E+00	0.2	8.8E-01	0.1	1.0E+00	0.3	4.4E-01	0.2	8.8E-01
193	PANX1	A_23_P47155	NM_015368	1.0	1.80E-02	0.9	1.80E-02	1.0	4.46E-02	1.1	3.14E-03	0.6	1.81E-01	0.6	1.81E-01	0.5	2.01E-01	0.7	4.27E-02	0.0	1.0E+00	0.0	1.0E+00	-0.1	9.1E-01	0.3	1.0E+00	0.0	9.6E-01	0.0	1.0E+00	0.0	9.6E-01	-0.2	9.0E-01
30936	HBG1	A_23_P53137	NM_000559	1.0	2.50E-03	1.0	4.28E-02	1.5	2.50E-03	0.9	1.02E-03	0.6	5.07E-02	0.5	6.66E-02	0.6	4.48E-02	0.7	5.07E-02	0.1	1.0E+00	0.1	1.0E+00	0.0	8.8E-01	0.1	1.0E+00	-0.1	8.8E-01	0.2	1.0E+00	-0.1	3.6E-01	-0.1	8.8E-01
16122	EPOR	A_23_P367899	NM_000121	1.0	1.94E-03	1.4	1.94E-03	0.9	2.36E-03	1.0	7.08E-06	1.4	4.15E-02	1.4	4.15E-02	0.9	1.59E-01	1.4	3.51E-02	0.1	1.0E+00	0.2	1.0E+00	-0.1	8.9E-01	0.1	1.0E+00	-0.2	8.2E-01	0.0	1.0E+00	-0.3	3.4E-01	-0.2	8.2E-01
30304	BATF	A_23_P128974	NM_006399	1.0	7.27E-04	0.9	1.06E-02	2.2	7.27E-04	1.0	2.98E-04	0.8	3.85E-02	0.8	1.93E-02	1.2	4.82E-02	0.6	3.85E-02	0.0	1.0E+00	0.0	1.0E+00	0.1	8.6E-01	0.0	1.0E+00	-0.1	9.1E-01	0.0	1.0E+00	-0.1	9.1E-01	-0.2	8.9E-01
33118	JUP	A_23_P501822	NM_002230	1.0	4.99E-03	1.0	4.99E-03	1.1	9.38E-03	0.7	2.43E-03	1.0	2.20E-02	0.8	3.23E-02	1.0	2.20E-02	1.0	6.39E-03	0.0	1.0E+00	0.0	1.0E+00	0.0	9.4E-01	0.0	1.0E+00	0.0	9.7E-01	0.0	1.0E+00	-0.3	7.0E-01	0.0	9.7E-01
16200	CDC42EP1	A_23_P166453	NM_152243	1.0	4.05E-03	1.0	4.05E-03	1.0	3.99E-02	1.5	2.93E-04	0.4	3.06E-01	0.4	3.06E-01	0.3	5.94E-01	0.8	2.82E-02	0.1	1.0E+00	0.1	1.0E+00	0.1	9.2E-01	-0.1	1.0E+00	0.2	8.2E-01	0.0	1.0E+00	0.3	3.8E-01	0.2	8.2E-01
9508	SLFN5	A_23_P402899	NM_144975	1.0	3.68E-03	1.0	4.97E-03	2.5	5.59E-04	0.9	3.68E-03	0.7	3.17E-01	0.7	2.46E-01	1.8	3.69E-01	0.7	3.17E-01	-0.2	1.0E+00	0.1	1.0E+00	-0.4	7.3E-01	-0.2	1.0E+00	-0.5	4.8E-01	-0.1	1.0E+00	-0.5	2.2E-01	-0.5	4.8E-01
34643	KIAA1257	A_32_P163739	NM_020741	1.0	1.48E-02	1.1	1.48E-02	1.0	3.49E-02	0.8	1.22E-02	1.1	9.66E-02	0.9	2.60E-01	1.5	9.66E-02	1.1	2.75E-02	0.2	1.0E+00	0.2	1.0E+00	-0.1	9.1E-01	0.2	1.0E+00	-0.3	6.4E-01	-0.1	1.0E+00	-0.3	4.2E-01	-0.3	6.4E-01
30561	ADM	A_23_P127948	NM_001124	1.0	5.33E-03	0.9	5.33E-03	1.0	3.26E-02	1.4	2.42E-04	0.6	1.28E-01	0.6	1.28E-01	0.5	1.34E-01	0.8	4.97E-02	0.0	1.0E+00	0.0	1.0E+00	0.0	9.9E-01	-0.1	1.0E+00	-0.1	9.7E-01	-0.2	1.0E+00	-0.1	9.1E-01	0.0	9.7E-01
40597	ELL3	A_23_P3237	NM_025165	1.0	2.16E-03	1.0	2.16E-03	1.0	2.71E-03	1.0	6.60E-05	0.9	1.87E-02	0.9	1.87E-02	0.8	3.01E-02	0.9	4.85E-03	0.1	1.0E+00	0.2	1.0E+00	0.1	8.4E-01	0.1	1.0E+00	-0.4	5.5E-01	-0.1	1.0E+00	-0.4	3.1E-01	-0.4	5.5E-01
22523	GOLGA8E	A_32_P103669	NM_001012423	1.0	1.02E-02	1.0	1.48E-02	1.0	1.02E-02	1.2	7.26E-05	0.5	6.98E-01	0.5	7.32E-01	0.4	6.98E-01	0.7	3.08E-01	-0.1	1.0E+00	-0.1	1.0E+00	-0.1	8.5E-01	0.2	1.0E+00	-0.2	8.7E-01	0.0	1.0E+00	-0.2	6.7E-01	-0.2	8.7E-01
31753	ATCAY	A_23_P101671	NM_033064	1.0	3.10E-03	1.0	7.15E-03	1.0	3.10E-03	0.9	2.01E-04	0.8	1.39E-02	0.8	2.72E-02	0.6	1.39E-02	0.9	3.04E-03	-0.1	1.0E+00	-0.1	1.0E+00	-0.1	8.9E-01	-0.1	1.0E+00	-0.2	8.1E-01	-0.2	1.0E+00	-0.2	4.1E-01	-0.1	8.1E-01
34209	CNTNAP1	A_23_P411113	NM_003632	1.0	3.69E-02	1.1	8.70E-03	1.0	3.69E-02	0.7	3.95E-02	1.1	3.63E-01	0.5	7.70E-01	1.1	3.63E-01	1.1	1.81E-01	0.0	1.0E+00	0.0	1.0E+00	0.1	9.5E-01	0.0	1.0E+00	0.0	1.0E+00	-0.1	1.0E+00	0.0	1.0E+00	0.3	7.5E-01
33747	PNPLA2	A_23_P10077	NM_020376	1.0	8.35E-04	1.0	8.35E-04	1.0	8.83E-03	1.1	2.26E-04	0.8	4.26E-02	0.7	4.26E-02	0.8	8.80E-02	1.2	1.77E-03	0.1	1.0E+00	0.1	1.0E+00	0.0	9.6E-01	0.1	1.0E+00	0.0	1.0E+00	0.0	1.0E+00	0.0	9.8E-01	0.0	1.0E+00
18208	THC2664465	A_32_P208654	THC2664465	1.0	4.59E-03	0.7	2.51E-02	1.3	4.59E-03	1.0	3.90E-04	0.7	2.32E-01	0.4	2.67E-01	0.9	2.32E-01	0.7	1.03E-01	0.0	1.0E+00	0.0	1.0E+00	-0.2	8.2E-01	0.2	1.0E+00	-0.2	7.5E-01	0.2	1.0E+00	-0.3	4.2E-01	-0.2	7.5E-01
24932	MEP1A	A_24_P154868	NM_005588	1.0	1.26E-03	0.9	2.52E-03	1.0	1.26E-03	1.0	1.72E-05	0.7	1.97E-02	0.2	6.23E-01	0.9	7.46E-03	0.7	1.97E-02	0.0	1.0E+00	0.1	1.0E+00	0.0	9.9E-01	-0.1	1.0E+00	0.1	7.1E-01	0.1	1.0E+00	0.2	1.8E-01	0.1	7.1E-01
27849	SLC24A6	A_23_P328600	NM_024959	1.0	3.04E-03	0.8	3.04E-03	1.3	4.05E-03	1.0	2.09E-04	0.9	2.60E-02	0.9	2.60E-02	0.8	3.50E-02	0.9	4.72E-03	0.1	1.0E+00	0.2	1.0E+00	0.1	8.4E-01	0.1	1.0E+00	0.1	5.5E-01	0.1	1.0E+00	0.1	5.2E-01	0.2	5.5E-01
26308	NFKB2	A_23_P202156	NM_001077493	1.0	2.85E-03	1.0	2.85E-03	0.9	3.65E-02	1.3	1.68E-04	0.9	1.67E-01	0.9	1.67E-01	0.6	4.55E-01	1.1	2.36E-02	0.2	1.0E+00	0.2	1.0E+00	0.1	7.9E-01	0.2	1.0E+00	0.0	9.7E-01	0.0	1.0E+00	0.0	9.7E-01	0.1	9.3E-01
11393	PFKFB3	A_24_P206604	NM_004566	1.0	3.50E-03	1.0	1.34E-02	1.0	3.50E-03	1.0	3.57E-05	0.9	9.07E-02	0.7	2.47E-01	0.9	9.07E-02	0.9	5.97E-02	0.2	1.0E+00	0.2	1.0E+00	-0.1	8.8E-01	0.2	1.0E+00	-0.2	6.7E-01	0.1	1.0E+00	-0.2	4.6E-01	-0.2	6.7E-01
31529	THC2719076	A_32_P13823	THC2719076	1.0	1.33E-03	1.0	3.05E-02	2.3	8.92E-04	0.9	1.33E-03	0.8	1.15E-01	0.8	4.97E-02	1.2	1.15E-01	0.6	2.30E-01	0.0	1.0E+00	0.1	1.0E+00	0.0	9.7E-01	-0.1	1.0E+00	-0.2	8.2E-01	0.1	1.0E+00	-0.2	6.4E-01	-0.2	8.2E-01
13017	CD511705	A_32_P126832	CD511705	1.0	2.28E-02	1.1	2.28E-02	1.0	4.80E-02	0.9	6.48E-03	1.0	3.29E-02	1.0	3.29E-02	0.9	2.16E-02	1.0	9.08E-02	0.0	1.0E+00	0.0	1.0E+00	-0.1	7.4E-01	0.0	1.0E+00	-0.3	8.6E-01	-0.2	1.0E+00	-0.4	6.0E-01	-0.3	8.6E-01
30114	ADM	A_23_P127948	NM_001124	1.0	4.10E-03	0.9	4.10E-03	1.0	1.80E-02	1.3	1.42E-04	0.5	9.87E-02	0.5	1.40E-01	0.5	9.87E-02	0.7	2.95E-02	0.0	1.0E+00	0.0	1.0E+00	0.0	1.0E+00	-0.1	1.0E+00	-0.1	8.1E-01	-0.1	1.0E+00	-0.1	8.1E-01	-0.1	7.9E-01
14428	PLCD1	A_23_P80739	NM_006225	1.0	3.41E-03	1.0	3.41E-03	1.3	5.79E-03	0.8	2.18E-03	0.7	6.46E-02	0.7	1.04E-01	0.6	6.46E-02	0.8	1.97E-02	0.1	1.0E+00	0.0	1.0E+00	0.3	7.1E-01	0.1	1.0E+00	0.0	9.9E-01	0.0	1.0E+00	0.0	9.9E-01	0.0	9.9E-01
32438	GPR137B	A_23_P46149	NM_003272	1.0	9.07E-03	1.0	9.07E-03	1.1	2.07E-02	1.0	1.24E-03	1.1	4.22E-03	0.7	8.05E-02	1.1	4.22E-03	1.2	4.78E-04	0.1	1.0E+00	0.0	1.0E+00	0.1	7.8E-01	0.2	1.0E+00	-0.2	9.0E-01	-0.2	1.0E+00	-0.2	7.2E-01	-0.2	9.0E-01
6900	NPTX1	A_23_P124905	NM_002522	1.0	1.58E-02	1.1	2.90E-02	0.8	1.58E-02	1.0	7.99E-05	0.6	6.37E-02	0.6	6.37E-02	0.4	1.49E-01	0.7	1.09E-02	0.0	1.0E+00	0.0	1.0E+00	0.0	9.7E-01	0.1	1.0E+00	-0.1	7.3E-01	-0.1	1.0E+00	-0.1	6.6E-01	-0.2	7.3E-01
30722	ITGA7	A_23_P128084	NM_002206	1.0	6.46E-04	0.9	6.46E-04	1.4	2.15E-03	1.0	1.52E-04	0.9	6.75E-02	0.8	8.00E-02	1.0	6.75E-02	0.9	2.31E-02	0.1	1.0E+00	0.2	1.0E+00	0.1	8.8E-01	0.1	1.0E+00	0.2	8.1E-01	0.2	1.0E+00	0.2	4.6E-01	0.2	8.1E-01
41825	CAPN12	A_24_P79040	NM_144691	1.0	1.65E-03	1.0	1.65E-03	1.5	1.65E-03	1.0	1.68E-04	1.1	2.15E-02	0.9	5.27E-02	1.1	2.15E-02	1.1	5.55E-03	0.1	1.0E+00	0.2	1.0E+00	0.1	8.0E-01	0.1	1.0E+00	0.1	9.8E-01	0.1	1.0E+00	0.0	9.8E-01	0.1	9.2E-01
24945	PLSCR3	A_23_P49597	NM_020360	1.0	7.77E-03	0.9	7.77E-03	1.1	1.97E-02	1.0	7.66E-04	0.8	6.65E-02	0.9	7.15E-02	0.8	6.65E-02	0.8	2.15E-02	0.2	1.0E+00	0.2	1.0E+00	0.2	7.3E-01	0.1	1.0E+00	0.1	9.7E-01	0.2	1.0E+00	0.1	9.1E-01	0.0	9.7E-01
12063	HTR7P	A_32_P174572	NR_002774	1.0	1.62E-03	1.0	1.62E-03	1.1	5.53E-03	1.0	2.55E-04	0.8	3.58E-02	0.9	1.05E-02	0.6	3.58E-02	0.8	1.35E-01	-0.1	1.0E+00	0.0	1.0E+00	-0.3	7.1E-01	-0.1	1.0E+00	-0.1	9.2E-01	0.0	1.0E+00	-0.1	6.7E-01	-0.1	9.2E-01
31737	FAM151A	A_23_P407695	NM_176782	1.0	2.93E-04	0.8	3.79E-04	1.5	2.93E-04	1.0	1.20E-05	0.4	5.06E-01	0.0	9.83E-01	0.5	5.06E-01	0.4	4.59E-01	-0.1	1.0E+00	0.0	1.0E+00	-0.2	7.7E-01	-0.1	1.0E+00	0.0	8.7E-01	0.0	1.0E+00	-0.2	3.8E-01	0.1	8.7E-01
38781	IFI27L1	A_23_P53976	NM_206949	1.0	2.25E-03	0.9	2.25E-03	1.4	2.83E-03	1.0	2.01E-04	0.7	3.04E-02	0.5	3.04E-02	0.7	5.54E-02	0.8	9.91E-03	-0.1	1.0E+00	0.1	1.0E+00	-0.1	8.0E-01	-0.1	1.0E+00	0.0	1.0E+00	0.1	1.0E+00	-0.1	8.2E-01	0.0	1.0E+00
28860	ADM	A_23_P127948	NM_001124	1.0	7.17E-03	0.9	7.17E-03	1.0	3.78E-02	1.4	2.26E-04	0.5	1.40E-01	0.5	1.44E-01	0.5	1.40E-01	0.7	5.04E-02	0.0	1.0E+00	0.0	1.0E+00	0.0	1.0E+00	-0.1	1.0E+00	-0.1	9.5E-01	-0.1	1.0E+00	0.0	9.5E-01	-0.1	9.4E-01
16777	LMBR1L	A_23_P150931	NM_018113	1.0	3.83E-02	1.0	3.83E-02	2.2	4.20E-03	0.6	3.95E-02	0.6	5.89E-02	0.6	5.89E-02	0.8	5.34E-02	0.6	1.30E-01	-0.1	1.0E+00	-0.1	1.0E+00	0.1	9.0E-01	-0.1	1.0E+00	-0.1	9.7E-01	0.0	1.0E+00	-0.1	9.4E-01	-0.1	9.7E-01
42990	SPAG4	A_23_P132027	NM_003116	1.0	7.56E-03	1.0	1.08E-02	1.9	3.01E-03	0.7	7.56E-03	0.8	7.31E-02	0.7	1.43E-01	1.0	7.31E-02	0.8	4.46E-02	0.2	1.0E+00	0.1	1.0E+00	0.2	7.1E-01	0.2	1.0E+00	-0.1	7.8E-01	0.0	1.0E+00	-0.1	6.4E-01	-0.2	7.8E-01
860	A_24_P332711	A_24_P332711	A_24_P332711	1.0	7.18E-03	0.8	1.16E-02	1.1	7.18E-03	1.0	4.65E-04	0.5	4.78E-01	0.4	6.68E-01	0.5	4.78E-01	0.7	2.13E-01	0.1	1.0E+00	0.1	1.0E+00	0.4	7.1E-01	0.1	1.0E+00	0.1	4.9E-01	-0.1	1.0E+00	0.1	4.9E-01	0.2	4.4E-01
19008	NFKB2	A_23_P202156	NM_001077493	1.0	3.63E-03	1.0	3.63E-03	0.9	4.02E-02	1.3	2.79E-04	0.9	8.40E-02	0.9	8.40E-02	0.5	5.33E-01	1.2	9.97E-03	0.2	1.0E+00	0.2	1.0E+00	0.2	7.1E-01	0.1	1.0E+00	0.0	9.7E-01	0.0	1.0E+00	0.0	9.7E-01	0.1	9.6E-01
39617	TMEM121	A_23_P61987	NM_025268	1.0	5.87E-03	1.0	5.87E-03	1.3	1.30E-02	0.9	3.50E-03	1.0	1.71E-02	1.0	1.71E-02	1.1	5.09E-02	1.0	8.52E-03	0.1	1.0E+00	0.3	1.0E+00	0.1	8.0E-01	0.1	1.0E+00	0.1	9.9E-01	0.2	1.0E+00	0.0	9.9E-01	0.1	9.5E-01
12109	SMAD3	A_23_P359091	U68019	1.0	1.34E-03	0.8	1.34E-03	1.0	1.55E-03	1.1	8.72E-05	0.7	3.74E-02	0.7	3.74E-02	0.4	1.05E-01	0.9	2.88E-03	-0.1	1.0E+00	0.0	1.0E+00	-0.2	7.4E-01	-0.1	1.0E+00	0.0	8.2E-01	0.0	1.0E+00	-0.2	5.7E-01	0.2	8.2E-01
12173	C1orf210	A_32_P42946	NM_182517	1.0	9.95E-04	1.0	1.26E-03	1.3	9.95E-04	0.9	5.19E-05	0.8	7.16E-03	0.7	2.35E-02	0.8	7.16E-03	0.8	1.72E-03	0.1	1.0E+00	0.2	1.0E+00	0.1	7.9E-01	0.1	1.0E+00	-0.1	9.2E-01	0.0	1.0E+00	-0.1	6.1E-01	-0.1	9.2E-01
33659	LRP4	A_24_P403561	NM_002334	1.0	1.62E-02	1.0	1.62E-02	1.1	1.81E-02	0.9	2.83E-03	0.8	5.81E-03	1.1	3.33E-03	0.8	5.81E-03	0.8	5.86E-02	0.0	1.0E+00	0.0	1.0E+00	-0.1	8.9E-01	0.1	1.0E+00	-0.2	3.4E-01	-0.2	1.0E+00	-0.2	3.4E-01	-0.3	2.6E-01
12934	MAGEB6	A_23_P378574	NM_173523	1.0	8.51E-03	1.0	8.51E-03	0.8	2.57E-02	1.1	2.88E-04	0.8	2.20E-01	1.0	2.20E-01	0.5	4.91E-01	0.8	1.50E-01	0.1	1.0E+00	0.1	1.0E+00	-0.1	7.9E-01	0.2	1.0E+00	-0.4	1.6E-01	-0.1	1.0E+00	-0.4	5.2E-02	-0.4	1.6E-01
38913	FZD9	A_23_P59613	NM_003508	1.0	7.65E-03	1.0	7.65E-03	1.3	1.65E-02	0.8	7.57E-03	1.0	5.75E-03	1.0	5.75E-03	1.3	1.36E-02	1.0	1.93E-03	0.2	1.0E+00	0.1	1.0E+00	0.2	7.4E-01	0.3	1.0E+00	-0.1	6.7E-01	0.1	1.0E+00	-0.1	6.3E-01	-0.2	6.7E-01
11861	CGN	A_24_P45728	NM_020770	1.0	6.56E-03	1.0	6.56E-03	1.6	2.88E-03	0.6	1.10E-02	1.3	8.94E-02	0.8	3.23E-01	1.6	8.94E-02	1.3	1.75E-02	0.0	1.0E+00	0.1	1.0E+00	-0.2	7.1E-01	0.0	1.0E+00	0.0	9.9E-01	0.1	1.0E+00	0.0	9.6E-01	0.0	9.9E-01
41447	GSTM3	A_23_P12343	NM_000849	1.0	3.41E-03	0.7	3.41E-03	1.0	5.41E-03	1.2	3.46E-04	0.0	9.23E-01	-0.5	7.77E-01	0.1	9.23E-01	0.0	9.93E-01	0.0	1.0E+00	0.0	1.0E+00	0.5	7.4E-01	-0.2	1.0E+00	0.3	6.3E-01	0.0	1.0E+00	0.3	5.0E-01	0.4	6.3E-01
24431	OPN4	A_23_P24234	NM_001030015	1.0	1.35E-02	1.0	1.35E-02	1.7	2.69E-03	0.8	3.54E-02	0.6	6.09E-01	-0.3	8.45E-01	0.6	6.09E-01	0.6	5.82E-01	-0.3	1.0E+00	-0.2	1.0E+00	-0.3	7.4E-01	-0.6	1.0E+00	0.4	7.8E-01	0.1	1.0E+00	0.6	2.1E-01	0.4	7.8E-01
24224	HES4	A_23_P149448	NM_021170	1.0	2.19E-02	1.0	2.19E-02	1.4	4.59E-02	1.0	1.30E-02	0.9	2.84E-02	0.6	1.19E-01	0.9	2.84E-02	1.1	9.89E-03	0.3	1.0E+00	0.2	1.0E+00	0.4	3.8E-01	0.3	1.0E+00	0.2	7.7E-01	0.3	1.0E+00	0.1	7.7E-01	0.2	7.0E-01
34928	RNMT	A_23_P55515	NM_003799	1.0	2.02E-02	0.7	2.02E-02	1.0	3.24E-02	1.6	2.02E-04	1.0	9.69E-02	0.2	7.33E-01	1.0	9.69E-02	1.0	7.79E-03	0.1	1.0E+00	0.1	1.0E+00	0.0	1.0E+00	0.1	1.0E+00	-0.3	6.6E-01	-0.3	1.0E+00	-0.3	4.9E-01	-0.4	6.6E-01
27438	A_24_P703642	A_24_P703642	A_24_P703642	1.0	2.51E-03	1.4	1.06E-03	0.9	3.23E-02	1.0	2.51E-03	1.0	4.67E-01	0.3	8.81E-01	1.0	4.67E-01	1.0	4.06E-01	-0.2	1.0E+00	-0.2	1.0E+00	0.3	8.6E-01	-0.4	1.0E+00	0.5	3.8E-01	0.0	1.0E+00	0.5	3.8E-01	0.6	3.0E-01
29614	CCR4	A_23_P72989	NM_005508	1.0	2.92E-03	0.8	1.51E-02	1.0	2.92E-03	1.6	2.57E-06	0.9	7.22E-03	0.4	1.28E-01	0.9	7.22E-03	1.0	4.92E-03	-0.1	1.0E+00	-0.1	1.0E+00	0.2	7.9E-01	-0.2	1.0E+00	0.2	9.7E-01	0.3	1.0E+00	0.2	7.4E-01	0.0	9.7E-01
26716	KCNH6	A_23_P390984	AK090969	1.0	4.15E-04	1.0	5.68E-03	1.4	4.15E-04	0.9	2.28E-05	0.7	5.90E-02	0.7	6.19E-02	0.8	5.90E-02	0.7	5.25E-02	0.0	1.0E+00	0.1	1.0E+00	-0.1	8.2E-01	0.0	1.0E+00	-0.1	9.6E-01	-0.2	1.0E+00	-0.1	6.6E-01	0.0	9.6E-01
40935	THC2664068	A_24_P460419	THC2664068	1.0	6.57E-04	1.0	3.64E-02	1.5	6.57E-04	1.0	1.65E-04	1.0	1.38E-02	1.3	3.18E-03	1.0	1.55E-02	0.9	1.38E-02	0.1	1.0E+00	0.1	1.0E+00	0.0	9.7E-01	0.1	1.0E+00	-0.3	3.4E-01	0.0	1.0E+00	-0.3	1.2E-01	-0.3	3.4E-01
37498	BAT4	A_23_P133923	NM_033177	1.0	2.23E-03	1.0	2.90E-03	1.1	2.23E-03	1.0	5.08E-05	0.7	1.08E-01	0.6	1.08E-01	0.7	1.26E-01	0.8	4.17E-02	0.0	1.0E+00	0.0	1.0E+00	0.0	9.9E-01	-0.1	1.0E+00	0.0	9.8E-01	0.1	1.0E+00	-0.1	6.6E-01	0.0	9.8E-01
24204	SHBG	A_23_P207089	NM_001040	1.0	1.98E-03	1.0	6.31E-03	1.6	1.98E-03	0.9	5.18E-04	0.8	1.58E-01	0.7	2.30E-01	0.8	1.58E-01	0.8	6.61E-02	0.1	1.0E+00	0.1	1.0E+00	0.0	9.9E-01	0.1	1.0E+00	0.0	9.8E-01	0.1	1.0E+00	-0.1	8.6E-01	0.0	9.8E-01
20542	DDX26B	A_23_P363647	NM_182540	1.0	6.27E-03	0.9	6.27E-03	1.2	8.14E-03	1.0	5.95E-04	0.7	4.27E-02	1.1	2.12E-02	0.4	5.44E-01	0.7	4.27E-02	0.2	1.0E+00	0.2	1.0E+00	-0.1	8.8E-01	0.2	1.0E+00	-0.1	7.5E-01	0.0	1.0E+00	-0.1	7.5E-01	-0.2	5.6E-01
12064	ADM	A_23_P127948	NM_001124	1.0	7.03E-03	0.8	7.03E-03	1.0	2.30E-02	1.3	2.81E-04	0.4	1.32E-01	0.4	1.68E-01	0.4	1.32E-01	0.6	9.40E-02	0.0	1.0E+00	0.0	1.0E+00	0.0	9.4E-01	-0.1	1.0E+00	-0.1	9.0E-01	-0.1	1.0E+00	-0.1	7.9E-01	-0.1	9.0E-01
32317	C6orf141	A_32_P114483	BC036917	1.0	1.26E-03	1.3	1.26E-03	0.7	1.25E-02	1.0	3.90E-05	0.7	1.27E-01	0.4	1.81E-01	0.7	1.27E-01	0.7	6.19E-02	0.0	1.0E+00	0.1	1.0E+00	0.0	9.4E-01	0.0	1.0E+00	-0.2	8.4E-01	-0.1	1.0E+00	-0.3	4.8E-01	-0.2	8.4E-01
31870	C14orf132	A_24_P920447	NR_023938	1.0	3.35E-03	1.5	4.15E-03	1.0	3.35E-03	1.0	6.63E-05	1.0	1.11E-02	1.2	6.75E-03	0.8	1.11E-02	1.0	1.28E-02	0.2	7.1E-01	0.3	6.7E-01	0.2	7.1E-01	0.2	1.0E+00	-0.1	9.7E-01	0.0	1.0E+00	-0.2	8.1E-01	-0.1	9.7E-01
14097	NP	A_23_P140256	NM_000270	1.0	2.62E-03	1.1	2.62E-03	0.9	4.54E-02	1.0	1.64E-03	0.9	3.71E-03	1.1	3.71E-03	0.9	4.19E-03	0.9	1.79E-03	0.1	1.0E+00	0.0	1.0E+00	0.1	8.7E-01	0.1	1.0E+00	0.1	9.7E-01	0.1	1.0E+00	0.3	5.8E-01	0.1	9.7E-01
38308	PAOX	A_23_P385771	NM_152911	1.1	4.93E-03	0.8	4.93E-03	1.1	8.65E-03	1.1	1.27E-04	0.9	7.53E-03	0.6	3.63E-02	0.9	3.27E-03	0.9	7.53E-03	0.2	1.0E+00	0.2	1.0E+00	0.2	7.1E-01	0.3	1.0E+00	-0.2	8.3E-01	0.0	1.0E+00	-0.2	6.3E-01	-0.2	8.3E-01
4535	COL1A1	A_23_P207520	Z74615	1.1	8.83E-04	1.1	1.69E-02	2.2	7.55E-04	0.9	8.83E-04	1.1	8.21E-03	0.9	2.71E-02	1.3	8.21E-03	1.1	6.52E-03	0.2	1.0E+00	0.1	1.0E+00	0.2	7.1E-01	0.2	1.0E+00	-0.2	5.6E-01	-0.2	1.0E+00	-0.2	4.5E-01	-0.3	5.6E-01
25470	DRAM	A_23_P99163	NM_018370	1.1	1.26E-02	1.1	1.08E-02	1.6	1.26E-02	0.8	1.30E-02	1.1	1.84E-02	1.4	1.84E-02	1.1	2.33E-02	1.1	1.80E-02	0.3	1.0E+00	0.3	1.0E+00	0.2	7.3E-01	0.4	1.0E+00	0.0	8.9E-01	0.0	1.0E+00	-0.1	8.7E-01	0.1	8.9E-01
31719	TUBB3	A_23_P77493	NM_006086	1.1	2.76E-03	1.1	2.76E-03	1.1	9.72E-03	1.0	7.71E-04	1.0	4.62E-03	1.3	2.51E-03	1.0	4.62E-03	0.8	1.53E-02	0.2	1.0E+00	0.1	1.0E+00	0.3	7.1E-01	0.2	1.0E+00	-0.1	9.5E-01	-0.2	1.0E+00	0.0	9.5E-01	-0.1	9.5E-01
18657	PLCH2	A_24_P62469	NM_014638	1.1	3.98E-03	1.1	3.98E-03	1.3	7.36E-03	0.8	2.16E-03	1.2	9.79E-02	0.8	1.43E-01	1.2	9.79E-02	1.4	2.06E-02	-0.1	1.0E+00	-0.1	1.0E+00	0.3	7.1E-01	-0.1	1.0E+00	0.2	7.7E-01	0.3	1.0E+00	0.2	4.6E-01	0.2	7.7E-01
23681	SMCR7	A_23_P501372	NM_139162	1.1	2.14E-03	1.1	2.14E-03	1.3	7.22E-03	1.0	7.26E-04	0.8	1.43E-01	0.7	1.43E-01	0.8	1.97E-01	0.8	4.55E-02	0.1	1.0E+00	0.1	1.0E+00	0.1	8.4E-01	0.1	1.0E+00	0.0	9.9E-01	0.0	1.0E+00	0.0	9.9E-01	0.1	9.6E-01
32782	ZCWPW1	A_23_P70897	NM_017984	1.1	2.88E-03	1.1	3.04E-02	1.1	2.88E-03	0.6	2.81E-03	1.0	9.48E-02	1.0	5.34E-02	1.0	9.48E-02	0.5	2.27E-01	0.0	1.0E+00	-0.1	1.0E+00	0.0	9.8E-01	0.1	1.0E+00	-0.2	9.4E-01	-0.2	1.0E+00	-0.2	7.4E-01	-0.1	9.4E-01
1520	LTK	A_23_P14853	NM_002344	1.1	3.00E-03	1.1	2.65E-03	1.5	4.35E-03	0.8	3.00E-03	1.2	7.16E-03	1.2	7.16E-03	0.9	3.60E-02	1.4	4.81E-04	0.1	1.0E+00	0.2	1.0E+00	0.1	8.7E-01	0.1	1.0E+00	0.0	9.8E-01	0.0	1.0E+00	-0.1	8.6E-01	0.0	9.8E-01
2486	CDC42EP4	A_24_P19054	NM_012121	1.1	7.07E-03	1.1	1.29E-02	1.7	7.07E-03	1.0	5.46E-03	0.6	1.94E-01	0.4	6.09E-01	0.8	1.87E-01	0.6	1.94E-01	0.1	1.0E+00	0.1	1.0E+00	0.2	7.1E-01	0.0	1.0E+00	-0.1	9.6E-01	-0.2	1.0E+00	-0.1	8.2E-01	-0.1	9.6E-01
27235	OLFM1	A_23_P219161	NM_006334	1.1	3.39E-02	0.8	4.06E-02	1.3	3.39E-02	1.1	5.00E-03	0.7	8.16E-02	0.8	8.16E-02	0.7	6.80E-02	0.5	1.20E-01	-0.1	1.0E+00	0.1	1.0E+00	-0.2	7.5E-01	-0.1	1.0E+00	0.2	9.0E-01	0.2	1.0E+00	0.1	7.9E-01	0.2	9.0E-01
24919	HEY1	A_32_P83845	NM_001040708	1.1	5.48E-03	1.1	5.48E-03	0.9	4.37E-02	1.1	7.92E-04	1.4	7.01E-03	1.5	7.01E-03	1.4	5.02E-03	1.1	9.63E-03	0.1	1.0E+00	0.1	1.0E+00	0.1	8.8E-01	0.4	1.0E+00	-0.3	5.4E-01	-0.1	1.0E+00	-0.3	4.7E-01	-0.3	5.4E-01
6629	NLGN2	A_23_P207125	NM_020795	1.1	3.90E-03	1.1	3.90E-03	1.2	2.02E-02	0.9	3.38E-03	0.4	7.04E-01	0.4	7.04E-01	0.0	9.90E-01	0.5	4.29E-01	0.1	1.0E+00	0.1	1.0E+00	0.4	7.1E-01	-0.1	1.0E+00	0.3	5.6E-01	0.0	1.0E+00	0.3	4.3E-01	0.4	5.6E-01
11375	UCN	A_24_P62883	NM_003353	1.1	1.71E-03	1.1	2.50E-02	1.9	1.70E-03	0.8	1.71E-03	0.6	5.37E-01	0.2	8.09E-01	0.7	5.37E-01	0.6	2.30E-01	-0.1	1.0E+00	-0.1	1.0E+00	-0.2	7.1E-01	0.0	1.0E+00	0.0	1.0E+00	0.1	1.0E+00	0.0	1.0E+00	0.0	9.7E-01

Feature	Gene symbol	Probe	Transcript	72 hour siCASP8AP2 Median M	72 hour siCASP8AP2 Median q	72 hour 1 M	72 hour 1 q	72 hour 3 M	72 hour 3 q	72 hour 6 M	72 hour 6 q	48 hours siCASP8AP2 Median M	48 hours Median q	48 hours 1 M	48 hours 1 q	48 hours 3 M	48 hours 3 q	48 hours 6 M	48 hours 6 q	24 hours siCASP8AP2 Median M	24 hours Median q	24 hours 1 M	24 hours 1 q	24 hours 3 M	24 hours 3 q	24 hours 6 M	24 hours 6 q	10 hours siCASP8AP2 Median M	10 hours Median q	10 hours 1 M	10 hours 1 q	10 hours 3 M	10 hours 3 q	10 hours 6 M	10 hours 6 q
17047	GH1	A_23_P207194	NM_000515	1.1	2.27E-04	1.1	2.69E-02	1.7	2.27E-04	1.0	1.01E-05	-0.1	9.62E-01	-0.3	9.14E-01	-0.1	9.63E-01	0.1	9.62E-01	-0.1	1.0E+00	-0.1	1.0E+00	0.2	7.1E-01	-0.2	1.0E+00	0.1	9.3E-01	0.1	1.0E+00	0.1	5.0E-01	-0.1	9.3E-01
38305	STAP2	A_23_P382188	NM_001013841	1.1	2.45E-03	0.8	8.70E-03	1.8	2.45E-03	1.1	6.80E-04	0.8	3.12E-02	0.6	8.56E-02	0.9	3.12E-02	0.8	6.70E-03	0.1	1.0E+00	0.1	1.0E+00	0.0	1.0E+00	0.1	1.0E+00	-0.3	8.4E-01	-0.1	1.0E+00	-0.3	6.7E-01	-0.3	8.4E-01
1938	COL1A1	A_23_P207520	Z74615	1.1	4.17E-04	1.1	3.57E-03	2.4	4.17E-04	0.9	3.95E-04	1.0	1.74E-02	0.9	2.15E-02	1.2	1.74E-02	1.0	9.63E-03	0.1	1.0E+00	0.1	1.0E+00	0.1	7.5E-01	0.2	1.0E+00	-0.1	9.8E-01	-0.1	1.0E+00	-0.1	6.9E-01	0.0	9.8E-01
7537	SERPINH1	A_23_P76006	NM_001235	1.1	6.52E-03	1.1	2.23E-03	1.5	6.52E-03	0.6	1.03E-02	0.8	8.33E-02	0.8	8.33E-02	1.5	2.61E-01	0.8	6.64E-02	-0.1	1.0E+00	0.2	1.0E+00	-0.1	8.2E-01	-0.1	1.0E+00	0.0	1.0E+00	0.0	1.0E+00	-0.2	6.4E-01	0.0	1.0E+00
22827	C4orf23	A_23_P29985	AK022953	1.1	1.59E-03	0.9	1.59E-03	1.1	8.63E-03	1.6	1.36E-05	0.8	2.27E-01	0.2	8.61E-01	0.8	2.27E-01	1.0	6.43E-02	-0.1	1.0E+00	-0.1	1.0E+00	-0.1	7.3E-01	-0.1	1.0E+00	0.1	9.4E-01	0.0	1.0E+00	0.2	6.8E-01	0.1	9.4E-01
2158	PLAGL1	A_23_P8175	NM_006718	1.1	8.42E-04	1.0	8.42E-04	1.1	1.80E-03	1.1	3.93E-05	0.6	8.18E-02	0.5	2.65E-01	0.6	8.18E-02	0.7	3.64E-02	0.2	1.0E+00	0.3	1.0E+00	-0.1	8.5E-01	0.2	1.0E+00	-0.2	7.7E-01	-0.1	1.0E+00	-0.2	5.1E-01	-0.2	7.7E-01
42720	ZFP90	A_24_P176409	NM_133458	1.1	1.72E-03	0.9	3.13E-03	1.1	1.72E-03	1.5	8.19E-07	0.9	2.12E-02	0.9	2.12E-02	0.9	2.58E-01	1.1	9.62E-03	0.0	1.0E+00	0.0	1.0E+00	-0.1	7.7E-01	0.1	1.0E+00	-0.3	4.4E-01	-0.2	1.0E+00	-0.3	2.0E-01	-0.3	4.4E-01
31045	LAYN	A_23_P127565	NM_178834	1.1	1.56E-03	1.0	1.56E-03	1.1	6.33E-03	1.2	8.45E-05	0.9	1.83E-02	0.8	6.79E-02	0.9	1.83E-02	1.1	9.06E-03	0.3	7.6E-01	0.3	6.8E-01	0.1	7.6E-01	0.5	8.0E-01	0.0	8.9E-01	0.0	1.0E+00	-0.1	6.2E-01	0.1	8.9E-01
33721	THC2710780	A_32_P24068	THC2710780	1.1	3.13E-03	1.1	3.13E-03	0.8	3.00E-02	1.3	1.43E-04	0.6	5.14E-01	0.1	9.59E-01	0.6	5.14E-01	0.8	3.00E-01	-0.1	1.0E+00	-0.1	1.0E+00	0.4	7.9E-01	-0.1	1.0E+00	0.2	7.6E-01	-0.1	1.0E+00	0.3	5.4E-01	0.2	7.6E-01
13723	YPEL4	A_24_P21770	NM_145008	1.1	7.48E-04	1.1	5.43E-03	2.0	7.48E-04	1.0	1.62E-04	0.4	5.18E-01	0.2	8.79E-01	1.2	5.18E-01	0.4	5.06E-01	-0.3	1.0E+00	-0.3	1.0E+00	0.4	7.2E-01	-0.5	1.0E+00	0.3	8.4E-01	-0.2	1.0E+00	0.7	2.3E-01	0.3	8.4E-01
18900	PRSS3	A_23_P135257	NM_002771	1.1	3.21E-03	0.7	3.21E-03	1.1	6.41E-03	1.2	7.77E-05	0.6	1.95E-01	0.4	4.16E-01	0.6	1.95E-01	0.8	2.85E-02	0.0	1.0E+00	-0.1	1.0E+00	0.0	9.5E-01	0.0	1.0E+00	0.3	6.0E-01	0.1	1.0E+00	0.3	4.6E-01	0.3	6.0E-01
15930	LOC401577	A_24_P68222	AK023533	1.1	2.41E-03	0.8	2.01E-02	1.5	2.41E-03	1.1	1.91E-04	0.8	1.45E-02	0.8	1.03E-01	0.8	1.45E-02	0.9	6.50E-03	0.0	1.0E+00	0.0	1.0E+00	-0.1	7.6E-01	0.0	1.0E+00	0.0	9.3E-01	0.0	1.0E+00	0.0	9.3E-01	0.1	9.2E-01
1519	GGN	A_23_P333552	NM_152657	1.1	8.46E-04	1.1	6.47E-04	1.1	6.04E-03	0.9	8.46E-04	1.0	3.16E-02	1.0	5.12E-02	0.9	3.16E-02	1.2	4.80E-03	0.1	1.0E+00	0.0	1.0E+00	0.2	7.1E-01	0.1	1.0E+00	0.0	9.3E-01	0.2	1.0E+00	0.0	9.3E-01	-0.1	9.2E-01
40032	COL1A1	A_23_P207520	Z74615	1.1	3.37E-04	1.1	6.82E-03	2.3	3.37E-04	1.0	2.80E-04	1.0	7.88E-03	0.9	1.65E-02	1.2	7.88E-03	1.0	2.93E-03	0.1	1.0E+00	0.1	1.0E+00	0.1	7.7E-01	0.1	1.0E+00	-0.1	8.7E-01	-0.1	1.0E+00	-0.2	5.2E-01	-0.1	8.7E-01
21085	FAM43A	A_23_P422144	NM_153690	1.1	4.58E-03	1.0	6.83E-03	1.8	4.58E-03	1.1	3.45E-03	0.8	1.04E-01	0.1	9.03E-01	0.8	1.04E-01	1.0	2.01E-02	-0.2	1.0E+00	0.1	1.0E+00	-0.2	7.7E-01	-0.2	1.0E+00	-0.2	9.1E-01	0.0	1.0E+00	-0.2	7.9E-01	-0.3	9.1E-01
21549	CD19	A_23_P113572	NM_001770	1.1	4.50E-04	0.9	4.50E-04	1.1	2.58E-03	1.1	4.04E-05	0.8	5.95E-02	0.6	1.03E-01	0.8	5.95E-02	0.8	1.48E-02	-0.2	1.0E+00	-0.2	1.0E+00	0.1	9.0E-01	-0.2	1.0E+00	0.1	8.9E-01	0.0	1.0E+00	0.3	8.9E-02	0.1	8.9E-01
29277	LAT	A_23_P60977	NM_014387	1.1	7.33E-03	0.9	7.33E-03	1.2	1.77E-02	1.1	1.00E-03	0.9	5.87E-02	0.9	5.87E-02	0.6	2.48E-01	0.9	8.20E-03	0.3	9.8E-01	0.3	9.8E-01	0.3	7.1E-01	0.2	1.0E+00	0.3	6.0E-01	0.1	1.0E+00	0.3	3.3E-01	0.3	6.0E-01
3631	PADI3	A_23_P126869	NM_016233	1.1	1.18E-02	1.1	2.18E-02	1.2	1.18E-02	1.0	8.95E-04	0.7	2.66E-01	0.7	2.66E-01	0.4	7.61E-01	1.0	4.50E-02	0.1	1.0E+00	0.1	1.0E+00	0.4	7.1E-01	0.1	1.0E+00	0.1	8.4E-01	0.1	1.0E+00	-0.1	4.8E-01	0.1	8.4E-01
22181	MYO1A	A_23_P162288	NM_005379	1.1	6.45E-03	1.1	2.09E-03	1.3	6.45E-03	0.6	7.98E-03	0.2	8.35E-01	0.0	9.84E-01	0.3	8.25E-01	0.2	8.35E-01	-0.1	1.0E+00	-0.1	1.0E+00	0.4	7.1E-01	-0.2	1.0E+00	0.3	4.1E-01	-0.2	1.0E+00	0.3	3.6E-01	0.3	4.1E-01
27128	FGR	A_23_P103932	NM_001042747	1.1	1.35E-04	0.8	1.83E-04	1.9	1.35E-04	1.1	6.48E-06	0.8	5.88E-02	0.6	3.79E-01	1.7	2.46E-02	0.8	5.88E-02	0.0	1.0E+00	0.0	1.0E+00	0.2	7.1E-01	0.0	1.0E+00	0.2	5.0E-01	0.0	1.0E+00	0.2	4.7E-01	0.2	5.0E-01
2194	AF067801	A_23_P92809	AF067801	1.1	3.57E-03	1.1	3.35E-02	1.7	3.57E-03	1.1	8.13E-04	1.0	1.38E-02	0.6	1.07E-01	1.1	1.38E-02	1.0	1.87E-03	0.0	1.0E+00	-0.1	1.0E+00	0.0	9.0E-01	0.2	1.0E+00	-0.2	6.8E-01	-0.1	1.0E+00	-0.2	3.5E-01	-0.2	6.8E-01
3407	C21orf67	A_23_P211196	AY040088	1.1	2.25E-02	1.1	2.54E-02	1.1	2.25E-02	0.7	1.33E-02	0.7	1.36E-01	0.7	1.83E-01	0.6	1.36E-01	0.7	5.95E-02	0.1	1.0E+00	0.2	1.0E+00	0.1	7.8E-01	0.1	1.0E+00	-0.1	9.1E-01	0.0	1.0E+00	-0.1	8.8E-01	-0.1	9.1E-01
17812	FBLN1	A_23_P211631	NM_006486	1.1	1.96E-02	1.1	2.02E-02	1.7	1.96E-02	1.0	1.14E-02	1.2	7.64E-02	1.2	2.93E-02	1.3	9.63E-02	1.0	7.64E-02	0.0	1.0E+00	0.1	1.0E+00	-0.1	9.2E-01	0.0	1.0E+00	0.0	9.9E-01	0.2	1.0E+00	0.0	9.9E-01	-0.1	9.6E-01
24082	HBZ	A_23_P3651	NM_005332	1.1	1.80E-03	0.9	1.80E-03	1.3	1.82E-03	1.1	5.76E-05	0.6	3.16E-01	0.3	4.95E-01	1.1	3.16E-01	0.6	1.12E-01	0.0	1.0E+00	0.0	1.0E+00	0.0	9.8E-01	-0.3	1.0E+00	0.1	8.8E-01	0.0	1.0E+00	0.4	1.1E-01	0.1	8.8E-01
15966	SLFN11	A_23_P129925	NM_152270	1.1	1.59E-03	0.9	1.59E-03	1.1	1.81E-03	1.5	1.12E-06	0.6	7.02E-02	0.6	7.02E-02	0.4	2.53E-01	0.9	1.64E-02	0.0	1.0E+00	0.0	1.0E+00	0.0	9.4E-01	0.2	1.0E+00	-0.1	8.8E-01	-0.1	1.0E+00	-0.1	8.8E-01	-0.2	8.0E-01
21383	HLA-J	A_24_P418044	NR_024240	1.1	1.59E-02	0.9	1.59E-02	1.1	2.33E-02	1.1	1.61E-03	0.5	3.32E-01	0.5	3.32E-01	0.0	9.84E-01	0.7	6.69E-02	0.1	1.0E+00	0.1	1.0E+00	0.2	7.9E-01	0.0	1.0E+00	0.2	8.4E-01	0.0	1.0E+00	0.2	6.2E-01	0.2	8.4E-01
21693	IL13RA1	A_23_P137196	NM_001560	1.1	2.37E-03	0.9	2.37E-03	1.2	2.69E-03	1.1	4.13E-05	0.6	3.31E-02	0.5	7.29E-02	0.6	3.31E-02	0.6	1.46E-02	0.0	1.0E+00	0.2	1.0E+00	-0.1	7.8E-01	0.0	1.0E+00	-0.1	1.0E+00	-0.1	1.0E+00	0.0	1.0E+00	-0.2	4.9E-01
25695	CLDN3	A_23_P71017	NM_001306	1.1	4.73E-03	1.1	1.19E-02	1.8	4.73E-03	1.0	2.29E-03	0.9	8.15E-02	0.8	8.15E-02	0.9	2.18E-01	1.1	2.27E-02	-0.1	1.0E+00	0.0	1.0E+00	-0.1	9.3E-01	-0.2	1.0E+00	-0.3	9.1E-01	0.1	1.0E+00	-0.3	7.9E-01	-0.3	9.1E-01
13192	PRSS2	A_32_P94444	NM_002770	1.1	4.25E-03	0.7	8.00E-03	1.2	4.25E-03	1.1	9.95E-05	0.8	8.22E-03	0.3	3.38E-01	1.1	8.22E-03	0.8	4.12E-03	0.0	1.0E+00	-0.1	1.0E+00	0.0	9.4E-01	0.0	1.0E+00	0.1	9.6E-01	0.1	1.0E+00	0.0	9.6E-01	0.1	9.6E-01
37686	CR597260	A_32_P44349	CR597260	1.1	6.21E-04	0.9	5.30E-03	1.8	6.21E-04	1.1	8.33E-05	1.3	2.28E-02	1.3	2.28E-02	1.4	5.45E-03	1.1	3.95E-02	0.0	1.0E+00	0.0	1.0E+00	0.0	9.8E-01	0.5	1.0E+00	-0.2	7.1E-01	-0.2	1.0E+00	-0.2	5.9E-01	-0.2	7.1E-01
26112	GJC3	A_23_P59869	ENST00000312891	1.1	5.51E-03	1.0	9.60E-03	1.2	5.51E-03	1.1	1.19E-04	1.3	1.61E-02	1.4	1.61E-02	1.1	2.10E-02	1.3	8.51E-03	0.0	1.0E+00	0.0	1.0E+00	-0.1	8.1E-01	0.1	1.0E+00	-0.3	5.2E-01	-0.1	1.0E+00	-0.4	1.9E-01	-0.3	5.2E-01
11411	THC2665061	A_23_P155425	THC2665061	1.1	9.83E-03	1.1	1.87E-02	1.3	9.83E-03	0.7	7.90E-03	0.8	1.02E-01	0.8	1.02E-01	1.2	1.34E-01	0.7	7.36E-02	0.1	1.0E+00	0.0	1.0E+00	0.1	8.8E-01	0.1	1.0E+00	-0.1	9.7E-01	0.0	1.0E+00	-0.2	7.5E-01	-0.1	9.7E-01
40029	SV2B	A_23_P100022	NM_014848	1.1	3.86E-03	1.8	3.86E-03	0.9	1.19E-02	1.1	1.07E-04	0.9	2.03E-01	0.9	1.65E-01	0.7	2.61E-01	0.9	2.03E-01	0.2	1.0E+00	0.0	1.0E+00	0.2	7.1E-01	0.2	1.0E+00	-0.1	5.4E-01	0.0	1.0E+00	-0.1	5.4E-01	-0.3	4.7E-01
15193	S1PR5	A_23_P107744	NM_030760	1.1	5.90E-04	1.0	2.83E-03	2.0	5.90E-04	1.1	1.55E-04	1.1	1.68E-02	1.1	4.32E-02	1.2	1.58E-02	1.1	1.68E-02	0.1	1.0E+00	0.2	1.0E+00	-0.1	9.1E-01	0.1	1.0E+00	-0.2	6.8E-01	0.0	1.0E+00	-0.2	6.8E-01	-0.3	6.8E-01
21010	RTN4RL1	A_23_P66481	NM_178568	1.1	4.15E-02	1.4	1.92E-02	1.1	4.76E-02	0.6	4.15E-02	1.1	7.23E-02	1.1	7.23E-02	0.9	1.01E-01	1.1	4.88E-02	0.0	1.0E+00	0.1	1.0E+00	-0.2	7.3E-01	0.0	1.0E+00	0.0	9.2E-01	0.2	1.0E+00	0.0	8.9E-01	-0.1	9.2E-01
35345	GABPB2	A_23_P73208	NM_144618	1.1	7.29E-04	1.2	7.29E-04	1.0	3.10E-03	1.1	2.26E-05	0.9	1.24E-02	0.8	3.23E-02	0.9	1.24E-02	0.9	2.68E-03	-0.1	1.0E+00	-0.1	1.0E+00	-0.2	7.6E-01	0.0	1.0E+00	-0.2	6.4E-01	0.0	1.0E+00	-0.2	6.4E-01	-0.4	5.5E-01
37987	RHOF	A_23_P203983	NM_019034	1.1	1.73E-02	1.1	1.73E-02	1.6	1.80E-02	0.9	1.66E-02	0.8	8.07E-02	0.7	9.08E-02	0.8	8.07E-02	0.9	6.29E-03	0.1	1.0E+00	0.1	1.0E+00	0.1	8.3E-01	0.1	1.0E+00	-0.1	8.8E-01	0.2	1.0E+00	-0.1	8.8E-01	-0.2	8.0E-01
42425	BX106493	A_24_P710681	BX106493	1.1	2.16E-03	1.1	2.16E-03	0.9	3.74E-03	1.4	2.04E-06	1.3	4.29E-03	1.3	4.29E-03	1.2	6.04E-02	1.4	4.12E-03	0.7	7.8E-02	0.6	7.8E-02	0.7	1.6E-01	1.0	3.5E-02	-0.1	8.8E-01	0.1	1.0E+00	-0.2	5.2E-01	-0.1	8.8E-01
22157	PRKCH	A_23_P205567	NM_006255	1.1	2.04E-03	0.9	2.04E-03	1.4	2.90E-03	1.1	2.32E-04	0.9	9.80E-02	0.9	9.80E-02	0.6	2.47E-01	0.9	2.77E-02	0.1	1.0E+00	0.1	1.0E+00	0.2	7.1E-01	0.0	1.0E+00	-0.1	8.6E-01	-0.1	1.0E+00	0.0	8.6E-01	-0.2	4.5E-01
45085	SHOX2	A_24_P300021	NM_006884	1.1	9.82E-04	0.7	1.32E-02	1.5	9.82E-04	1.1	9.17E-04	0.8	7.20E-02	0.6	1.01E-01	1.6	7.20E-02	0.8	1.26E-02	-0.1	1.0E+00	0.1	1.0E+00	-0.3	7.1E-01	-0.1	1.0E+00	-0.4	3.2E-01	0.0	1.0E+00	-0.4	5.7E-02	-0.4	3.2E-01
39326	CCL17	A_23_P26325	NM_002987	1.1	1.08E-03	1.1	1.08E-03	1.0	2.19E-03	1.1	1.06E-05	0.5	2.80E-01	-0.1	9.28E-01	0.6	2.80E-01	0.5	1.96E-01	0.0	1.0E+00	0.0	1.0E+00	0.1	8.4E-01	-0.1	1.0E+00	0.0	9.2E-01	0.0	1.0E+00	0.0	9.2E-01	0.1	8.8E-01
37662	DNAH10	A_32_P228268	NM_207437	1.1	1.38E-03	1.0	1.38E-03	1.1	2.19E-03	1.1	2.18E-05	0.6	6.04E-02	0.6	1.06E-01	0.6	6.04E-02	0.9	3.55E-02	0.0	1.0E+00	0.0	1.0E+00	0.0	9.9E-01	0.0	1.0E+00	-0.2	4.7E-01	-0.1	1.0E+00	-0.2	3.5E-01	-0.2	4.7E-01
38007	ADM	A_23_P127948	NM_001124	1.1	1.27E-02	0.8	1.27E-02	1.1	2.93E-02	1.2	6.87E-04	0.4	1.11E-01	0.4	3.04E-01	0.4	1.11E-01	0.7	4.86E-02	0.0	1.0E+00	0.0	1.0E+00	0.0	9.3E-01	-0.2	1.0E+00	-0.1	9.7E-01	-0.1	1.0E+00	-0.1	7.1E-01	0.0	9.7E-01
7903	WDR42A	A_24_P886960	AK026213	1.1	1.41E-03	1.1	1.17E-02	1.4	8.56E-04	0.8	1.41E-03	1.0	1.16E-01	1.1	5.56E-02	1.0	1.34E-01	0.8	1.16E-01	0.0	1.0E+00	0.0	1.0E+00	0.0	9.4E-01	0.1	1.0E+00	-0.2	8.7E-01	0.0	1.0E+00	-0.2	6.0E-01	-0.2	8.7E-01
266	IL2RA	A_23_P127288	NM_000417	1.1	4.46E-03	1.1	1.22E-02	0.8	4.46E-03	1.1	6.60E-05	0.9	7.83E-03	1.1	7.83E-03	0.8	1.70E-02	0.9	9.61E-04	0.0	1.0E+00	0.1	1.0E+00	0.0	9.0E-01	0.0	1.0E+00	-0.1	7.4E-01	-0.1	1.0E+00	-0.1	5.2E-01	-0.1	7.4E-01
37391	ABLIM3	A_23_P256205	NM_014945	1.1	8.53E-04	1.1	4.03E-02	1.8	7.64E-04	0.7	8.53E-04	0.9	3.23E-02	0.9	3.23E-02	0.8	8.18E-02	1.0	2.70E-02	0.1	1.0E+00	0.1	1.0E+00	-0.1	8.0E-01	0.1	1.0E+00	-0.2	6.5E-01	0.0	1.0E+00	-0.2	3.9E-01	-0.2	6.5E-01
8668	MEIS3	A_24_P207503	NM_001009813	1.1	1.53E-03	1.1	1.53E-03	0.8	2.76E-02	1.2	1.42E-03	1.2	7.83E-03	1.2	7.83E-03	0.8	1.91E-01	1.4	4.54E-03	0.1	1.0E+00	0.2	1.0E+00	0.1	8.5E-01	0.1	1.0E+00	0.1	8.8E-01	0.0	1.0E+00	0.1	8.4E-01	0.2	8.8E-01
5605	LEPREL2	A_23_P87752	NM_014262	1.1	1.65E-02	1.1	1.03E-02	1.5	1.65E-02	0.7	2.27E-02	0.9	3.40E-02	0.9	3.40E-02	0.9	3.85E-02	0.9	4.37E-03	0.0	1.0E+00	0.1	1.0E+00	0.0	9.6E-01	0.0	1.0E+00	0.1	1.0E+00	0.2	1.0E+00	0.0	1.0E+00	0.1	8.9E-01
10004	ROPN1L	A_32_P335921	NM_031916	1.1	6.69E-03	1.1	1.20E-02	1.2	5.40E-03	0.8	6.69E-03	1.2	1.61E-02	1.3	1.61E-02	1.2	1.47E-02	1.2	1.91E-02	0.0	1.0E+00	0.0	1.0E+00	-0.2	7.6E-01	0.0	1.0E+00	-0.2	5.0E-01	0.0	1.0E+00	-0.3	2.2E-01	-0.2	5.0E-01
15188	LOC284344	A_24_P882959	AK097672	1.1	4.80E-03	0.7	3.24E-02	1.1	4.80E-03	1.2	1.38E-04	0.9	1.94E-02	0.7	5.62E-02	0.9	1.94E-02	1.2	5.85E-03	0.1	1.0E+00	0.1	1.0E+00	0.0	9.9E-01	0.1	1.0E+00	0.0	1.0E+00	-0.1	1.0E+00	0.1	8.2E-01	0.0	1.0E+00
29128	MATN4	A_23_P102622	NM_003833	1.1	1.93E-03	1.1	1.75E-02	1.8	1.93E-03	1.0	3.46E-04	0.8	2.63E-01	0.6	2.74E-01	0.9	2.63E-01	0.8	6.79E-02	0.0	1.0E+00	0.0	1.0E+00	-0.2	7.2E-01	0.1	1.0E+00	0.0	1.0E+00	0.1	1.0E+00	-0.1	9.4E-01	0.0	1.0E+00
31258	SPSB1	A_23_P200096	NM_025106	1.1	2.12E-03	1.2	2.12E-03	0.9	2.31E-02	1.1	2.79E-04	0.7	1.03E-01	0.7	3.72E-01	0.7	1.03E-01	1.1	1.68E-02	0.1	1.0E+00	0.0	1.0E+00	0.1	8.6E-01	0.1	1.0E+00	-0.2	9.8E-01	-0.2	1.0E+00	-0.2	6.9E-01	0.0	9.8E-01
5453	GNG7	A_32_P136295	NM_052847	1.1	1.16E-03	1.1	3.13E-02	1.7	1.10E-03	0.7	1.16E-03	1.0	8.95E-02	1.0	1.61E-01	1.1	8.95E-02	1.0	6.74E-02	0.3	1.0E+00	0.1	1.0E+00	0.3	6.7E-01	0.3	1.0E+00	-0.2	3.0E-01	-0.2	1.0E+00	-0.2	3.0E-01	-0.3	2.7E-01
8148	FLJ35767	A_23_P375165	NM_207459	1.1	1.71E-02	0.7	4.16E-02	1.4	1.71E-02	1.1	2.00E-03	0.8	4.37E-03	0.8	1.88E-02	1.3	3.28E-03	0.7	4.37E-03	0.3	1.0E+00	0.3	1.0E+00	0.3	7.1E-01	0.4	1.0E+00	0.0	9.8E-01	0.1	1.0E+00	-0.1	9.3E-01	0.0	9.8E-01
14423	RAB6B	A_23_P109974	NM_016577	1.1	8.40E-03	1.1	5.47E-03	1.4	9.08E-03	0.9	8.40E-03	0.9	2.12E-01	0.9	2.12E-01	0.6	3.91E-01	0.9	8.33E-02	0.0	1.0E+00	0.2	1.0E+00	-0.1	8.5E-01	0.0	1.0E+00	-0.4	5.9E-01	0.1	1.0E+00	-0.5	2.3E-01	-0.4	5.9E-01
1271	NCRNA00086	A_24_P418250	NR_024359	1.1	3.45E-03	0.9	3.45E-03	1.2	3.59E-03	1.1	7.26E-05	1.2	1.27E-02	1.2	1.27E-02	0.8	1.50E-01	1.2	3.28E-03	0.2	1.0E+00	0.3	1.0E+00	0.0	1.0E+00	0.2	1.0E+00	0.3	9.0E-01	0.3	1.0E+00	0.1	9.0E-01	0.3	8.7E-01
7582	FOXD2	A_23_P86171	NM_004474	1.1	5.43E-04	0.9	2.98E-02	2.1	5.43E-04	1.1	8.59E-05	0.9	4.69E-02	0.9	4.51E-02	1.4	8.15E-02	0.7	4.69E-02	0.1	1.0E+00	0.1	1.0E+00	0.1	9.3E-01	0.3	1.0E+00	-0.2	5.3E-01	-0.2	1.0E+00	-0.2	5.3E-01	-0.5	2.4E-01
29949	RILP	A_24_P185117	NM_031430	1.1	6.07E-03	1.1	5.53E-03	1.4	1.90E-02	1.0	6.07E-03	1.0	2.00E-02	1.0	2.00E-02	0.8	1.49E-01	1.2	1.17E-03	0.2	1.0E+00	0.3	1.0E+00	0.2	7.2E-01	0.2	1.0E+00	0.2	6.8E-01	0.1	1.0E+00	0.2	5.1E-01	0.2	6.8E-01
16479	IL20RB	A_23_P91850	NM_144717	1.1	1.99E-03	1.1	1.35E-02	1.6	1.99E-03	1.0	2.39E-04	0.7	3.57E-02	0.7	3.57E-02	0.6	4.17E-01	0.7	2.98E-02	-0.1	1.0E+00	-0.1	1.0E+00	-0.2	7.1E-01	0.2	1.0E+00	-0.1	9.5E-01	-0.1	1.0E+00	-0.2	5.4E-01	-0.1	9.5E-01
14538	RGS10	A_23_P138717	NM_001005339	1.1	4.03E-03	0.8	4.03E-03	1.3	4.39E-03	1.1	4.78E-04	0.7	1.97E-02	0.5	1.52E-01	0.9	1.97E-02	0.7	8.94E-03	0.1	1.0E+00	0.1	1.0E+00	0.1	9.2E-01	0.1	1.0E+00	-0.1	8.0E-01	0.0	1.0E+00	-0.1	8.0E-01	-0.2	7.1E-01
21793	ADAMTS4	A_23_P360754	NM_005099	1.1	8.37E-04	1.1	8.37E-04	1.1	1.52E-03	1.3	1.06E-04	0.8	1.21E-02	0.8	1.62E-02	0.7	1.21E-02	0.9	9.91E-03	0.0	1.0E+00	0.0	1.0E+00	0.0	9.2E-01	0.0	1.0E+00	0.0	9.9E-01	0.1	1.0E+00	0.0	9.9E-01	0.0	9.6E-01
41306	MDGA1	A_24_P314640	NM_153487	1.1	3.72E-03	1.2	3.72E-03	1.1	2.68E-02	1.1	2.08E-03	0.4	6.83E-01	0.4	8.15E-01	0.4	6.83E-01	0.6	5.09E-01	0.1	1.0E+00	0.1	1.0E+00	0.3	7.3E-01	0.0	1.0E+00	0.1	9.4E-01	0.0	1.0E+00	0.1	6.8E-01	0.1	9.4E-01
12349	LOC145814	A_32_P3534	NM_001102612	1.1	1.74E-04	0.9	1.74E-04	1.5	3.03E-04	1.1	6.32E-06	0.8	1.11E-02	0.6	3.23E-02	0.8	1.02E-02	1.0	1.11E-02	0.1	1.0E+00	0.1	1.0E+00	0.3	7.1E-01	0.1	1.0E+00	0.2	3.7E-01	0.1	1.0E+00	0.2	3.7E-01	0.3	3.5E-01
4799	TLE1	A_23_P135239	NM_005077	1.1	3.96E-03	0.7	3.81E-02	1.2	3.96E-03	1.1	5.55E-04	0.4	6.73E-01	0.1	9.17E-01	0.4	6.73E-01	0.5	5.35E-01	-0.1	1.0E+00	0.2	1.0E+00	-0.3	7.1E-01	-0.1	1.0E+00	-0.5	3.4E-01	0.0	1.0E+00	-0.5	1.9E-01	-0.5	3.4E-01
1268	LOC283454	A_32_P170454	AK094730	1.1	1.57E-02	1.1	3.68E-02	2.6	6.52E-03	1.1	1.57E-02	0.8	8.87E-02	0.8	8.87E-02	1.2	7.01E-02	0.7	9.20E-02	0.1	1.0E+00	0.0	1.0E+00	0.1	8.9E-01	0.3	1.0E+00	-0.3	8.9E-01	-0.2	1.0E+00	-0.3	7.1E-01	-0.3	8.9E-01
12869	LOC100130967	A_23_P257497	ENST00000367073	1.1	3.65E-04	1.1	3.65E-04	1.0	4.20E-03	1.2	1.85E-05	0.8	1.47E-02	1.0	1.38E-02	0.8	1.47E-02	0.8	1.74E-02	0.1	1.0E+00	0.1	1.0E+00	0.0	1.0E+00	0.1	1.0E+00	-0.2	6.6E-01	0.1	1.0E+00	-0.2	5.4E-01	-0.3	6.6E-01
23573	HSPB1	A_23_P257704	NM_001540	1.1	2.91E-03	0.9	2.91E-03	1.2	4.60E-03	1.1	8.84E-05	0.6	1.05E-01	0.6	1.49E-01	0.6	1.05E-01	0.7	2.31E-02	0.0	1.0E+00	0.3	1.0E+00	0.0	9.9E-01	0.0	1.0E+00	0.0	9.9E-01	0.1	1.0E+00	0.0	9.9E-01	0.0	9.8E-01
28892	MUC1	A_23_P137856	NM_002456	1.1	3.00E-03	1.1	1.25E-02	2.0	2.21E-03	0.9	3.00E-03	1.2	1.91E-02	1.2	1.91E-02	1.3	6.01E-02	1.1	9.46E-03	0.0	1.0E+00	0.0	1.0E+00	-0.3	7.1E-01	0.0	1.0E+00	0.0	9.7E-01	0.1	1.0E+00	0.0	9.2E-01	0.0	9.7E-01
9047	LMO2	A_23_P53126	NM_005574	1.1	2.61E-03	1.3	2.61E-03	1.1	3.58E-02	1.1	2.05E-03	1.0	2.89E-02	0.9	5.29E-02	1.0	2.89E-02	1.0	1.46E-02	0.0	1.0E+00	0.0	1.0E+00	-0.1	9.1E-01	0.0	1.0E+00	-0.4	6.2E-01	-0.3	1.0E+00	-0.4	4.4E-01	-0.5	6.2E-01
35609	COL1A1	A_23_P207520	Z74615	1.1	3.26E-04	1.1	4.76E-03	2.3	3.26E-04	1.0	2.24E-04	1.0	7.47E-03	0.8	1.90E-02	1.3	7.47E-03	1.0	5.02E-03	0.1	1.0E+00	0.1	1.0E+00	0.1	7.4E-01	0.2	1.0E+00	-0.2	6.7E-01	-0.1	1.0E+00	-0.2	2.5E-01	-0.2	6.7E-01
14290	THC2662584	A_24_P269315	THC2662584	1.1	1.10E-03	1.1	4.57E-03	2.1	1.10E-03	1.1	2.28E-04	1.1	1.42E-02	1.1	1.42E-02	1.4	2.26E-02	0.8	5.10E-03	0.1	1.0E+00	0.0	1.0E+00	0.1	9.4E-01	0.1	1.0E+00	-0.1	8.6E-01	0.0	1.0E+00	-0.1	8.3E-01	-0.2	8.6E-01
32575	LGALS1	A_23_P166459	NM_002305	1.1	1.85E-03	1.0	1.85E-03	1.1	1.20E-02	1.2	4.69E-04	0.9	4.74E-02	0.9	8.57E-02	0.8	4.74E-02	1.1	6.58E-03	0.1	1.0E+00	0.3	1.0E+00	0.0	9.4E-01	0.1	1.0E+00	-0.2	8.8E-01	-0.1	1.0E+00	-0.4	5.1E-01	-0.2	8.8E-01
22706	CSHL1	A_23_P344408	NM_022579	1.1	9.82E-04	1.1	1.82E-03	1.2	9.82E-04	0.8	1.01E-04	0.6	9.21E-02	0.3	4.14E-01	0.6	5.61E-02	0.6	9.21E-02	-0.1	1.0E+00	-0.1	1.0E+00	-0.1	7.6E-01	-0.3	1.0E+00	0.3	4.2E-01	0.0	1.0E+00	0.3	2.2E-01	0.3	4.2E-01
9930	PDE4DIP	A_23_P149153	NM_022359	1.1	5.74E-04	0.9	6.47E-04	1.4	5.74E-04	1.1	5.63E-06	0.9	1.38E-02	0.9	1.42E-02	0.8	1.38E-02	1.1	2.43E-03	0.0	1.0E+00	0.0	1.0E+00	-0.1	7.2E-01	0.1	1.0E+00	-0.1	7.4E-01	0.0	1.0E+00	-0.2	2.3E-01	-0.1	7.4E-01
41081	SOCS1	A_23_P420196	NM_003745	1.1	1.57E-02	1.1	1.57E-02	1.2	2.13E-02	1.1	1.00E-03	0.8	4.41E-02	0.5	1.77E-01	0.8	3.73E-02	0.8	4.41E-02	0.2	1.0E+00	0.2	1.0E+00	0.2	7.1E-01	-0.1	1.0E+00	-0.2	8.7E-01	0.0	1.0E+00	-0.3	4.7E-01	-0.2	8.7E-01
37588	BX375060	A_32_P9368	BX375060	1.1	1.54E-03	1.1	5.28E-03	2.0	1.32E-03	1.0	1.54E-03	0.7	2.54E-01	0.4	6.34E-01	1.4	2.18E-01	0.7	2.54E-01	0.0	1.0E+00	0.1	1.0E+00	0.0	9.7E-01	0.0	1.0E+00	0.2	8.7E-01	-0.1	1.0E+00	0.2	7.0E-01	0.2	8.7E-01
11967	MICALL2	A_24_P206305	NM_182924	1.1	1.45E-03	1.1	3.56E-03	1.6	1.45E-03	0.8	4.48E-04	0.7	3.19E-02	0.7	3.67E-02	0.9	3.19E-02	0.7	6.52E-03	0.2	1.0E+00	0.2	1.0E+00	0.2	7.4E-01	0.2	1.0E+00	0.3	7.9E-01	0.3	1.0E+00	0.2	7.1E-01	0.3	7.9E-01
2813	PLEKHA4	A_23_P67360	NM_020904	1.1	4.59E-03	1.1	2.07E-02	1.6	4.59E-03	0.9	4.38E-03	1.1	1.81E-02	1.1	4.94E-02	1.1	1.81E-02	1.1	1.62E-02	0.3	1.0E+00	0.3	1.0E+00	0.2	7.7E-01	0.3	1.0E+00	-0.2	7.5E-01	0.0	1.0E+00	-0.2	7.4E-01	-0.2	7.5E-01
40620	GJC2	A_23_P51729	NM_020435	1.1	1.56E-02	1.1	7.26E-03	1.5	1.71E-02	0.8	1.56E-02	0.5	5.10E-01	0.5	6.61E-01	0.7	3.91E-01	0.5	5.10E-01	0.1	1.0E+00	0.1	1.0E+00	0.2	7.1E-01	0.0	1.0E+00	0.1	9.5E-01	0.2	1.0E+00	0.1	4.9E-01	0.0	9.5E-01
42726	IRF7	A_24_P118892	NM_004029	1.1	2.11E-03	1.1	2.11E-03	1.1	1.33E-02	1.2	3.41E-04	1.0	1.46E-01	1.0	1.46E-01	0.6	5.53E-01	1.4	1.27E-02	0.3	1.0E+00	0.3	1.0E+00	0.1	8.6E-01	0.3	1.0E+00	-0.1	9.0E-01	0.0	1.0E+00	-0.1	8.1E-01	-0.1	9.0E-01
29077	THC2537219	A_24_P707530	THC2537219	1.1	1.65E-02	1.1	1.65E-02	1.6	1.74E-02	1.1	4.55E-03	0.6	1.03E-01	0.6	1.03E-01	0.6	3.91E-02	0.6	1.78E-01	0.1	1.0E+00	0.1	1.0E+00	0.1	9.3E-01	0.1	1.0E+00	-0.4	7.1E-01	-0.2	1.0E+00	-0.4	5.9E-01	-0.5	7.1E-01
43700	ADRA2A	A_23_P138706	NM_000681	1.1	1.01E-03	1.2	6.60E-03	1.1	1.01E-03	0.8	7.26E-05	0.6	4.26E-01	0.5	6.48E-01	0.6	3.86E-01	0.6	4.26E-01	0.1	1.0E+00	0.1	1.0E+00	0.1	7.1E-01	0.0	1.0E+00	-0.1	8.2E-01	0.1	1.0E+00	-0.1	6.7E-01	-0.1	8.2E-01
1517	TSNARE1	A_23_P319520	NM_145003	1.1	1.75E-02	1.1	1.75E-02	2.1	1.26E-02	1.1	1.83E-02	0.6	6.36E-01	0.3	8.71E-01	0.8	5.79E-01	0.6	6.36E-01	-0.1	1.0E+00	-0.1	1.0E+00	0.5	7.1E-01	-0.1	1.0E+00	0.4	5.1E-01	0.2	1.0E+00	0.4	2.3E-01	0.4	5.1E-01
29216	RDH12	A_23_P37088	NM_152443	1.1	4.35E-03	1.1	7.62E-03	0.9	4.35E-03	1.1	1.69E-05	1.0	1.47E-02	1.0	1.47E-02	0.8	4.24E-02	1.2	3.99E-03	0.1	1.0E+00	0.0	1.0E+00	0.1	7.8E-01	0.2	1.0E+00	-0.2	7.0E-01	-0.1	1.0E+00	-0.3	3.0E-01	-0.2	7.0E-01
44144	SLC6A20	A_23_P212379	NM_020208	1.1	4.07E-04	1.1	1.01E-02	1.5	4.07E-04	0.8	1.05E-04	0.5	5.80E-01	0.5	5.80E-01	0.2	8.40E-01	0.5	4.53E-01	0.1	1.0E+00	0.0	1.0E+00	0.1	8.6E-01	0.1	1.0E+00	-0.1	9.5E-01	0.0	1.0E+00	-0.1	8.5E-01	-0.1	9.5E-01
16893	MGAT5B	A_24_P219474	NM_144677	1.1	8.86E-03	1.1	1.02E-02	1.4	6.75E-03	0.7	8.86E-03	1.5	2.37E-02	1.5	7.92E-03	1.6	2.37E-02	1.3	4.07E-02	0.1	1.0E+00	0.1	1.0E+00	-0.2	8.3E-01	0.2	1.0E+00	0.0	9.8E-01	0.0	1.0E+00	-0.2	6.6E-01	0.0	9.8E-01
34395	SYTL3	A_23_P168188	NM_001009991	1.1	1.74E-03	1.1	1.74E-03	1.1	1.55E-02	1.1	4.14E-04	1.3	2.04E-02	1.0	9.27E-02	1.3	2.04E-02	1.3	6.88E-03	0.0	1.0E+00	0.0	1.0E+00	0.0	9.6E-01	0.1	1.0E+00	-0.2	9.0E-01	-0.1	1.0E+00	-0.2	7.0E-01	-0.2	9.0E-01
23225	OSTM1	A_23_P31097	NM_014028	1.1	1.09E-02	0.9	1.09E-02	1.2	3.04E-02	1.1	4.30E-03	0.3	4.75E-01	0.3	4.75E-01	0.2	6.01E-01	0.5	1.93E-01	0.0	1.0E+00	0.1	1.0E+00	-0.1	8.9E-01	0.0	1.0E+00	-0.1	9.5E-01	0.1	1.0E+00	-0.1	9.5E-01	-0.1	9.5E-01
35934	LRRC56	A_23_P368996	NM_198075	1.1	7.41E-04	1.1	5.98E-03	1.9	7.41E-04	1.1	7.19E-05	1.1	2.03E-02	1.0	8.68E-02	1.5	2.03E-02	1.1	1.79E-02	0.0	1.0E+00	0.1	1.0E+00	0.0	9.9E-01	0.0	1.0E+00	-0.1	9.3E-01	0.0	1.0E+00	-0.1	8.9E-01	-0.1	9.3E-01
6917	LMO2	A_23_P53126	NM_005574	1.1	4.04E-03	1.3	4.04E-03	1.1	4.21E-02	1.1	2.87E-03	0.9	2.90E-02	0.9	3.20E-02	0.9	2.90E-02	0.9	2.17E-02	0.0	1.0E+00	0.0	1.0E+00	-0.1	9.3E-01	0.0	1.0E+00	-0.5	5.9E-01	-0.3	1.0E+00	-0.5	4.0E-01	-0.5	5.9E-01
39653	WFDC10B	A_32_P951	NM_172006	1.1	1.82E-02	1.1	1.82E-02	1.6	1.82E-02	1.1	1.06E-02	0.7	5.52E-01	0.0	9.90E-01	0.7	5.52E-01	0.8	3.30E-01	0.1	1.0E+00	0.1	1.0E+00	0.6	7.1E-01	-0.1	1.0E+00	0.3	7.4E-01	0.2	1.0E+00	0.3	6.1E-01	0.4	7.4E-01
5359	AK027211	A_23_P100522	AK027211	1.1	4.52E-03	0.7	2.07E-02	1.7	4.52E-03	1.1	1.38E-03	0.3	7.07E-01	-0.1	9.35E-01	0.7	5.07E-01	0.3	7.07E-01	0.2	1.0E+00	0.2	1.0E+00	0.4	7.1E-01	0.0	1.0E+00	0.3	7.5E-01	0.3	1.0E+00	-0.3	7.5E-01	0.6	5.1E-01
20828	H2AFJ	A_23_P204277	NM_177925	1.1	1.09E-03	1.1	1.18E-03	1.4	1.09E-03	1.1	9.28E-05	0.9	1.99E-02	0.8	1.99E-02	0.9	1.31E-02	1.0	2.67E-02	0.1	1.0E+00	0.1	1.0E+00	0.0	9.0E-01	0.1	1.0E+00	-0.1	8.7E-01	0.0	1.0E+00	-0.1	8.3E-01	-0.2	8.7E-01
35861	ANG	A_23_P428738	NM_001145	1.1	7.33E-04	1.1	7.33E-04	1.1	5.95E-03	1.2	1.14E-04	1.0	1.84E-03	1.0	1.72E-02	1.3	1.84E-03	1.0	1.44E-03	0.2	7.1E-01	0.2	1.0E+00	0.2	7.1E-01	0.4	2.3E-01	-0.3	8.1E-01	-0.2	1.0E+00	-0.3	6.0E-01	-0.3	8.1E-01
38420	SERHL2	A_23_P120953	NM_014509	1.1	2.06E-02	1.1	2.24E-02	1.6	1.24E-02	0.8	2.06E-02	0.3	7.96E-01	0.2	9.09E-01	0.6	5.73E-01	0.3	7.96E-01	0.0	1.0E+00	-0.1	1.0E+00	0.2	8.1E-01	0.0	1.0E+00	0.0	9.9E-01	0.0	1.0E+00	0.1	8.8E-01	0.0	9.9E-01
25660	LETM2	A_23_P348264	NM_144652	1.1	2.01E-02	0.7	2.01E-02	1.1	2.47E-02	1.3	4.91E-04	1.0	5.54E-02	0.6	2.56E-01	1.0	5.54E-02	1.0	2.16E-02	0.1	1.0E+00	0.1	1.0E+00	0.2	7.4E-01	0.1	1.0E+00	0.1	8.5E-01	0.0	1.0E+00	0.1	7.7E-01	0.1	8.5E-01
27806	LOC153546	A_32_P197698	AK055939	1.1	2.30E-03	1.2	2.30E-03	1.1	4.73E-03	0.7	1.18E-03	1.0	6.81E-02	1.0	6.81E-02	1.1	3.82E-02	1.0	7.09E-02	0.0	1.0E+00	0.1	1.0E+00	0.0	9.9E-01	-0.1	1.0E+00	0.0	9.8E-01	0.0	1.0E+00	0.0	9.8E-01	0.0	9.8E-01
11852	SLC25A45	A_23_P422511	NM_182556	1.1	7.05E-04	1.1	4.00E-04	1.5	1.58E-03	0.8	7.05E-04	1.0	2.02E-02	0.9	3.18E-02	1.0	2.02E-02	1.1	8.24E-03	0.1	1.0E+00	0.1	1.0E+00	0.9	7.0E-01	0.1	1.0E+00	0.3	7.9E-01	0.0	1.0E+00	0.5	2.2E-01	0.3	7.9E-01
18463	BCL3	A_23_P4662	NM_005178	1.1	2.16E-02	1.1	9.50E-03	1.2	3.79E-02	0.9	2.16E-02	1.0	4.69E-02	1.0	4.69E-02	0.8	9.89E-02	1.0	3.88E-02	0.2	1.0E+00	0.3	1.0E+00	0.1	9.5E-01	0.2	1.0E+00	-0.3	9.0E-01	0.0	1.0E+00	-0.3	7.3E-01	-0.3	9.0E-01
6505	C20orf195	A_23_P131754	NM_024059	1.1	6.60E-03	1.1	6.84E-03	1.8	6.60E-03	1.1	2.43E-03	0.8	1.72E-01	0.7	3.80E-01	0.9	1.72E-01	0.8	1.50E-01	0.1	1.0E+00	0.1	1.0E+00	0.8	7.1E-01	0.1	1.0E+00	0.1	9.5E-01	0.0	1.0E+00	0.1	8.2E-01	0.1	9.5E-01
3569	A_23_P370707	A_23_P370707	A_23_P370707	1.1	3.69E-03	0.9	7.83E-03	1.9	3.69E-03	1.1	1.18E-03	0.7	1.40E-01	0.4	4.87E-01	0.7	1.40E-01	0.7	1.28E-01	0.1	1.0E+00	0.1	1.0E+00	0.1	9.0E-01	0.0	1.0E+00	0.3	7.5E-01	0.2	1.0E+00	0.3	4.8E-01	0.3	7.5E-01
20608	PMM1	A_23_P211598	NM_002676	1.1	1.13E-03	1.1	1.13E-03	1.7	3.58E-03	1.1	9.06E-04	0.8	1.34E-01	0.8	1.34E-01	0.7	1.96E-01	0.9	4.55E-02	0.1	1.0E+00	0.3	1.0E+00	0.1	8.3E-01	0.1	1.0E+00	0.0	1.0E+00	0.0	1.0E+00	0.0	9.9E-01	0.0	1.0E+00
42343	BFSP1	A_23_P109171	NM_001195	1.1	9.77E-04	1.5	9.77E-04	1.1	7.14E-03	1.0	3.96E-04	1.6	1.13E-03	1.8	5.54E-04	1.6	1.13E-03	1.6	5.22E-03	0.1	1.0E+00	0.0	1.0E+00	0.1	9.0E-01	0.1	1.0E+00	0.0	1.0E+00	0.1	1.0E+00	0.0	9.4E-01	0.0	1.0E+00
28702	TMEM88	A_23_P77859	NM_203411	1.1	4.11E-04	1.1	4.11E-04	2.1	5.47E-04	1.1	2.14E-04	1.0	9.60E-02	0.4	5.84E-01	1.1	9.60E-02	1.0	3.38E-02	0.1	1.0E+00	0.1	1.0E+00	0.1	7.6E-01	0.1	1.0E+00	0.1	9.0E-01	0.1	1.0E+00	0.2	7.3E-01	0.1	9.0E-01
15945	RYR1	A_23_P78867	NM_000540	1.1	4.59E-02	1.0	4.59E-02	1.5	4.67E-02	1.1	1.53E-02	1.2	3.99E-02	1.2	1.11E-01	1.3	3.43E-02	1.1	3.99E-02	0.0	1.0E+00	0.1	1.0E+00	-0.1	8.3E-01	0.0	1.0E+00	0.1	9.3E-01	0.1	1.0E+00	0.0	9.1E-01	0.1	9.3E-01
40927	CCL18	A_23_P55270	NM_002988	1.1	3.65E-04	0.7	1.35E-02	1.3	3.65E-04	1.1	4.78E-05	0.3	1.76E-01	0.3	1.76E-01	0.2	4.10E-01	0.5	7.00E-02	0.0	1.0E+00	0.0	1.0E+00	0.1	7.8E-01	0.0	1.0E+00	0.0	9.5E-01	0.0	1.0E+00	0.1	7.4E-01	0.0	9.5E-01
26752	TMEM107	A_24_P284353	NM_032354	1.1	7.12E-03	1.1	7.12E-03	1.1	1.97E-02	1.2	5.15E-04	0.9	1.33E-02	0.9	1.33E-02	1.0	3.80E-03	0.8	2.20E-02	0.1	1.0E+00	0.2	1.0E+00	0.1	9.2E-01	0.0	1.0E+00	-0.1	9.5E-01	0.0	1.0E+00	-0.1	9.5E-01	-0.1	9.4E-01
25114	ENST00000401649	A_24_P50057	ENST00000401649	1.1	5.75E-03	1.0	5.75E-03	1.1	7.12E-03	1.3	1.79E-04	0.5	2.98E-01	0.5	1.71E-01	0.2	5.16E-01	0.5	2.98E-01	0.0	1.0E+00	0.0	1.0E+00	-0.1	8.7E-01	0.0	1.0E+00	0.1	9.6E-01	0.1	1.0E+00	0.1	6.4E-01	0.0	9.6E-01

Feature	Gene symbol	Probe	Transcript	72 hour								48 hours								24 hours								10 hours							
				siCASP8AP2								siCASP8AP2								siCASP8AP2								siCASP8AP2							
				Median	Median	1	1	3	3	6	6	Median	Median	1	1	3	3	6	6	Median	Median	1	1	3	3	6	6	Median	Median	1	1	3	3	6	6
				M	q	M	q	M	q	M	q	M	q	M	q	M	q	M	q	M	q	M	q	M	q	M	q	M	q	M	q	M	q	M	q
24240	CD109	A_23_P214176	NM_133493	1.1	3.32E-03	0.8	1.21E-02	1.4	3.32E-03	1.1	4.78E-04	0.8	1.58E-02	0.8	1.24E-02	0.8	1.70E-02	0.6	1.58E-02	0.0	1.0E+00	0.0	1.0E+00	0.0	9.3E-01	0.0	1.0E+00	-0.1	9.3E-01	0.0	1.0E+00	-0.1	9.2E-01	-0.1	9.3E-01
43460	TSC22D4	A_24_P200942	NM_030935	1.1	1.22E-02	0.9	1.22E-02	1.1	2.51E-02	1.3	3.59E-04	0.6	5.79E-02	0.6	5.79E-02	0.4	5.26E-01	1.0	2.16E-03	0.3	7.1E-01	0.4	7.1E-01	0.3	7.1E-01	0.2	1.0E+00	0.0	1.0E+00	0.0	1.0E+00	0.0	9.5E-01	0.0	1.0E+00
10425	NOS2	A_23_P502464	NM_000625	1.1	9.72E-03	1.2	9.72E-03	1.0	2.30E-02	1.1	3.43E-04	1.1	1.93E-02	1.1	1.93E-02	0.7	1.08E-01	1.2	8.94E-03	0.0	1.0E+00	0.0	1.0E+00	0.1	8.8E-01	-0.1	1.0E+00	-0.1	7.7E-01	0.0	1.0E+00	-0.1	7.1E-01	-0.1	7.7E-01
24112	BC032041	A_32_P85779	BC032041	1.1	4.97E-04	0.7	6.21E-03	1.4	4.97E-04	1.1	1.01E-05	0.5	3.74E-02	0.5	1.44E-01	0.4	3.74E-02	0.7	2.40E-03	0.1	1.0E+00	0.1	1.0E+00	0.2	7.1E-01	0.0	1.0E+00	0.0	9.7E-01	0.0	1.0E+00	0.0	9.1E-01	0.0	9.7E-01
23913	GFI1B	A_23_P216845	NM_004188	1.1	1.32E-02	1.1	1.49E-02	1.2	1.32E-02	0.8	1.20E-02	0.8	1.97E-01	0.1	8.66E-01	0.9	1.97E-01	0.8	1.88E-01	0.0	1.0E+00	0.0	1.0E+00	0.3	7.9E-01	-0.4	1.0E+00	0.4	7.5E-01	-0.1	1.0E+00	1.0	3.5E-02	0.4	7.5E-01
18565	BLVRB	A_23_P153351	NM_000713	1.1	4.38E-03	1.1	1.02E-02	1.8	4.38E-03	1.0	1.72E-03	1.0	1.10E-01	0.8	1.70E-01	1.0	1.10E-01	1.0	7.82E-02	0.2	1.0E+00	0.2	1.0E+00	0.2	8.2E-01	0.1	1.0E+00	-0.1	9.6E-01	0.1	1.0E+00	-0.1	9.6E-01	-0.1	9.6E-01
31166	KRTAP4-1	A_23_P10936	NM_033060	1.1	1.55E-03	0.9	1.74E-02	1.2	1.55E-03	1.1	1.55E-05	1.0	2.10E-02	1.0	2.10E-02	0.7	2.60E-01	1.1	5.73E-03	0.1	1.0E+00	0.2	1.0E+00	-0.1	8.1E-01	0.1	1.0E+00	-0.2	6.4E-01	-0.1	1.0E+00	-0.2	3.4E-01	-0.2	6.4E-01
24214	MICB	A_23_P387471	NM_005931	1.1	5.36E-03	1.1	5.36E-03	1.1	4.46E-02	1.7	4.20E-04	1.2	1.07E-02	1.3	1.25E-02	1.2	1.07E-02	1.1	2.44E-03	0.4	1.0E+00	0.3	1.0E+00	0.4	7.1E-01	0.4	1.0E+00	0.0	1.0E+00	-0.1	1.0E+00	0.1	8.1E-01	0.0	1.0E+00
15170	ESAM	A_23_P47410	NM_138961	1.1	1.76E-03	1.1	8.71E-03	1.2	1.76E-03	1.1	7.95E-05	0.8	7.43E-02	0.8	7.43E-02	0.4	2.75E-01	0.8	2.34E-02	-0.1	1.0E+00	-0.1	1.0E+00	0.5	7.1E-01	-0.1	1.0E+00	0.2	8.4E-01	0.0	1.0E+00	0.3	2.0E-01	0.2	8.4E-01
2570	CTSL1	A_23_P94533	NM_001912	1.2	3.02E-03	1.2	3.02E-03	1.0	4.51E-02	1.2	1.49E-03	0.9	3.57E-02	1.0	1.82E-02	0.4	6.73E-01	0.9	3.57E-02	0.2	1.0E+00	0.2	1.0E+00	0.0	1.0E+00	0.2	1.0E+00	0.0	9.7E-01	0.0	1.0E+00	0.0	9.7E-01	-0.2	9.1E-01
13276	CGA	A_24_P371782	NM_000735	1.2	9.48E-04	0.7	4.90E-03	1.2	9.48E-04	1.2	6.36E-06	0.5	5.96E-02	0.4	1.69E-01	0.7	1.93E-02	0.5	5.96E-02	0.0	1.0E+00	0.0	1.0E+00	0.2	7.1E-01	-0.2	1.0E+00	0.2	5.7E-01	0.0	1.0E+00	0.2	3.0E-01	0.2	5.7E-01
9608	TMPRSS4	A_23_P127608	NM_019894	1.2	2.11E-02	1.2	2.11E-02	1.0	2.42E-02	1.2	3.75E-04	0.8	2.68E-01	0.9	3.15E-01	0.7	2.68E-01	0.8	1.76E-01	0.2	1.0E+00	0.2	1.0E+00	0.4	4.4E-01	0.2	1.0E+00	0.0	9.8E-01	0.0	1.0E+00	-0.1	8.1E-01	0.0	9.8E-01
40515	COL1A1	A_23_P207520	Z74615	1.2	1.40E-04	1.2	3.22E-03	2.3	1.40E-04	1.0	4.35E-05	1.1	4.83E-03	0.9	1.71E-02	1.3	4.83E-03	1.1	3.44E-03	0.2	9.7E-01	0.2	1.0E+00	0.2	7.1E-01	0.3	9.7E-01	-0.2	4.5E-01	-0.1	1.0E+00	-0.2	2.4E-01	-0.3	4.5E-01
5108	PCSK6	A_23_P390006	NM_138325	1.2	1.30E-02	0.9	1.55E-02	1.4	1.30E-02	1.2	1.17E-03	0.8	5.89E-02	0.7	1.57E-01	0.8	5.89E-02	0.8	3.11E-02	0.1	1.0E+00	0.1	1.0E+00	-0.2	7.5E-01	0.1	1.0E+00	-0.1	8.1E-01	-0.1	1.0E+00	-0.2	5.9E-01	-0.1	8.1E-01
20513	FKBP1B	A_23_P142631	NM_054033	1.2	8.42E-04	0.9	8.42E-04	1.2	1.03E-03	1.2	7.68E-06	1.0	1.56E-02	1.1	1.56E-02	0.6	1.51E-01	1.0	4.52E-03	0.2	1.0E+00	0.2	1.0E+00	0.0	9.4E-01	0.3	1.0E+00	0.0	9.8E-01	0.1	1.0E+00	0.0	9.6E-01	0.0	9.8E-01
14850	BC036435	A_24_P631848	BC036435	1.2	1.88E-03	0.7	1.86E-02	1.9	1.88E-03	1.2	1.75E-03	0.7	3.12E-01	0.4	7.11E-01	0.7	3.12E-01	0.7	2.15E-01	-0.2	1.0E+00	-0.4	1.0E+00	0.5	7.1E-01	-0.2	1.0E+00	0.4	3.1E-01	0.1	1.0E+00	0.8	1.6E-02	0.4	3.1E-01
11059	BC033513	A_24_P7807	BC033513	1.2	6.15E-04	1.2	7.95E-03	1.2	6.15E-04	0.9	7.69E-06	0.9	4.61E-03	0.9	1.26E-02	1.0	4.61E-03	0.9	2.36E-03	0.1	1.0E+00	0.0	1.0E+00	0.1	7.6E-01	0.1	1.0E+00	0.0	9.9E-01	-0.1	1.0E+00	0.0	9.9E-01	0.0	9.7E-01
27569	KRTAP9-4	A_23_P207868	NM_033191	1.2	7.44E-05	1.2	1.50E-03	2.7	3.99E-05	1.0	7.44E-05	1.2	9.03E-04	1.2	7.24E-03	2.1	1.07E-04	1.1	9.03E-04	0.2	1.0E+00	0.2	1.0E+00	0.1	7.1E-01	0.2	1.0E+00	0.0	9.9E-01	0.0	1.0E+00	0.0	8.1E-01	0.0	9.9E-01
35228	MAGEA6	A_23_P136870	NM_175868	1.2	2.50E-02	1.2	2.50E-02	2.4	1.09E-03	1.0	3.77E-02	0.4	7.87E-01	-0.4	8.14E-01	2.2	5.10E-01	0.4	7.87E-01	-0.6	1.0E+00	-0.4	1.0E+00	-0.6	7.9E-01	-0.7	1.0E+00	0.2	8.8E-01	-0.1	1.0E+00	0.2	8.8E-01	0.4	8.5E-01
29150	RAB8B	A_23_P37535	NM_016530	1.2	9.96E-03	0.9	1.29E-02	1.4	9.96E-03	1.2	1.91E-03	0.8	3.92E-02	0.7	1.09E-01	0.8	3.92E-02	0.8	3.44E-02	0.1	1.0E+00	0.0	1.0E+00	0.2	7.3E-01	0.1	1.0E+00	-0.2	6.0E-01	-0.1	1.0E+00	-0.2	5.9E-01	-0.3	6.0E-01
27402	MYL5	A_23_P258018	NM_002477	1.2	3.36E-03	1.2	3.36E-03	1.6	5.27E-03	0.9	2.35E-03	0.9	7.15E-02	0.9	8.30E-02	1.3	7.15E-02	0.9	2.49E-02	0.0	1.0E+00	0.0	1.0E+00	-0.1	9.0E-01	0.1	1.0E+00	0.2	9.2E-01	0.2	1.0E+00	0.2	6.8E-01	0.1	9.2E-01
30773	RILP	A_23_P26815	NM_031430	1.2	2.37E-03	1.1	2.37E-03	1.6	7.21E-03	1.2	1.23E-03	1.3	7.62E-03	1.1	1.77E-02	1.3	7.62E-03	1.3	1.40E-03	0.1	1.0E+00	0.2	1.0E+00	0.1	7.5E-01	0.1	1.0E+00	0.1	8.9E-01	0.2	1.0E+00	0.1	8.9E-01	0.1	8.8E-01
25306	ZFP36L2	A_23_P101960	NM_006887	1.2	2.70E-02	0.8	3.22E-02	1.2	2.70E-02	1.2	1.02E-03	0.5	2.34E-01	0.5	2.34E-01	0.7	3.49E-01	0.5	1.75E-01	0.0	1.0E+00	0.0	1.0E+00	-0.1	9.3E-01	0.1	1.0E+00	-0.1	9.4E-01	-0.2	1.0E+00	-0.1	9.0E-01	0.1	9.4E-01
25867	SGMS2	A_23_P326204	NM_152621	1.2	4.16E-03	0.9	4.16E-03	1.2	6.72E-03	1.2	1.04E-04	0.9	9.56E-02	0.9	1.11E-01	0.8	9.56E-02	0.9	3.47E-02	0.2	1.0E+00	0.2	1.0E+00	0.0	1.0E+00	0.2	1.0E+00	-0.1	8.9E-01	0.2	1.0E+00	-0.1	7.4E-01	-0.1	8.9E-01
29809	NAGS	A_23_P406187	NM_153006	1.2	3.71E-03	0.8	5.87E-03	1.6	3.71E-03	1.2	6.16E-04	1.0	1.77E-02	1.0	1.77E-02	1.2	2.27E-02	0.8	8.55E-03	0.2	1.0E+00	0.3	1.0E+00	0.2	7.1E-01	0.2	1.0E+00	0.0	9.8E-01	0.0	1.0E+00	0.0	8.8E-01	0.0	9.8E-01
34673	VPS37D	A_23_P409417	NM_001077621	1.2	4.33E-04	1.2	2.00E-04	1.4	1.68E-03	1.0	4.33E-04	0.6	3.53E-01	0.6	4.64E-01	0.7	3.53E-01	0.6	2.55E-01	0.1	1.0E+00	0.1	1.0E+00	0.2	7.7E-01	0.0	1.0E+00	0.0	9.6E-01	0.0	1.0E+00	0.0	9.6E-01	0.1	9.2E-01
13317	HSD3B1	A_23_P97173	NM_000862	1.2	6.08E-04	1.0	2.40E-03	1.5	6.08E-04	1.2	6.82E-06	1.0	7.83E-03	1.0	7.83E-03	1.0	5.64E-02	1.1	7.47E-04	0.1	1.0E+00	0.1	1.0E+00	0.1	8.2E-01	0.2	1.0E+00	-0.2	6.0E-01	0.0	1.0E+00	-0.2	3.9E-01	-0.2	6.0E-01
13438	BC038749	A_32_P139367	BC038749	1.2	3.02E-03	1.2	3.02E-03	1.2	3.15E-03	1.1	5.20E-05	0.9	5.93E-03	0.9	6.53E-03	0.9	5.93E-03	0.8	3.56E-03	0.0	1.0E+00	-0.1	1.0E+00	0.1	8.3E-01	0.0	1.0E+00	-0.1	9.7E-01	-0.2	1.0E+00	-0.1	8.5E-01	0.0	9.7E-01
16447	MICALL2	A_24_P303524	NM_182924	1.2	1.31E-03	1.2	1.31E-03	1.4	4.93E-03	1.1	3.25E-04	0.8	7.82E-02	0.7	1.45E-01	0.8	7.82E-02	0.8	3.42E-02	0.2	8.9E-01	0.3	8.9E-01	0.1	7.1E-01	0.2	1.0E+00	0.3	6.8E-01	0.4	1.0E+00	0.3	3.6E-01	0.2	6.8E-01
10586	TCP11L1	A_23_P47247	NM_018393	1.2	1.26E-03	0.9	1.26E-03	1.5	1.61E-03	1.2	5.15E-05	1.1	1.77E-02	0.8	5.83E-02	1.1	1.77E-02	1.1	3.45E-03	0.0	1.0E+00	0.0	1.0E+00	-0.2	7.1E-01	0.0	1.0E+00	-0.1	8.7E-01	0.1	1.0E+00	-0.1	7.2E-01	-0.1	8.7E-01
39486	NOS2	A_23_P502464	NM_000625	1.2	4.52E-03	1.2	4.52E-03	0.8	2.28E-02	1.2	7.36E-05	1.0	1.71E-02	1.0	1.71E-02	0.8	4.00E-02	1.2	3.24E-03	0.1	1.0E+00	0.1	1.0E+00	0.1	8.4E-01	0.1	1.0E+00	-0.2	4.2E-01	-0.1	1.0E+00	-0.2	2.1E-01	-0.2	4.2E-01
8270	NEURL	A_23_P322562	NM_004210	1.2	2.48E-03	1.2	2.16E-02	2.3	8.55E-04	0.9	2.48E-03	0.6	5.03E-01	0.5	7.16E-01	1.0	4.69E-01	0.6	5.03E-01	0.0	1.0E+00	-0.1	1.0E+00	0.3	7.1E-01	0.0	1.0E+00	0.2	6.2E-01	0.0	1.0E+00	0.3	2.8E-01	0.2	6.2E-01
23960	FLT4	A_23_P356070	NM_002020	1.2	4.27E-05	1.1	4.27E-05	1.2	8.81E-04	1.2	1.03E-05	0.8	1.38E-02	0.7	1.25E-01	1.0	1.38E-02	0.8	1.27E-02	0.0	1.0E+00	-0.1	1.0E+00	0.0	9.6E-01	0.0	1.0E+00	0.1	8.1E-01	0.0	1.0E+00	0.1	8.1E-01	0.2	7.9E-01
3947	HDAC5	A_23_P26916	NM_001015053	1.2	7.65E-03	1.3	7.65E-03	1.2	2.18E-02	1.0	1.93E-03	0.8	8.09E-02	0.8	8.51E-02	0.6	8.09E-02	1.2	4.37E-03	0.3	1.0E+00	0.2	1.0E+00	0.3	4.1E-01	0.3	1.0E+00	-0.1	9.2E-01	0.0	1.0E+00	-0.2	5.9E-01	-0.1	9.2E-01
21337	PLEKHA4	A_24_P408047	NM_020904	1.2	1.01E-03	1.1	1.18E-02	1.8	1.01E-03	1.2	6.02E-05	1.2	1.36E-02	1.2	7.83E-03	1.5	1.36E-02	0.9	1.79E-02	0.2	1.0E+00	0.0	1.0E+00	0.3	7.1E-01	0.2	1.0E+00	-0.1	1.0E+00	-0.1	1.0E+00	0.0	1.0E+00	-0.1	9.1E-01
11366	THC2642537	A_32_P144999	THC2642537	1.2	2.00E-03	1.2	8.84E-03	1.2	2.00E-03	1.1	1.44E-04	1.0	2.15E-02	1.0	2.15E-02	0.8	2.64E-02	1.0	3.54E-03	0.0	1.0E+00	-0.1	1.0E+00	0.1	7.2E-01	0.0	1.0E+00	-0.1	8.7E-01	0.0	1.0E+00	-0.1	7.3E-01	-0.1	8.7E-01
13851	ADCY4	A_23_P381261	NM_139247	1.2	1.34E-03	1.2	1.34E-03	1.6	1.90E-03	1.1	1.66E-04	0.8	1.27E-01	0.6	1.92E-01	0.9	1.27E-01	0.8	3.79E-02	0.0	1.0E+00	0.0	1.0E+00	0.0	9.9E-01	0.1	1.0E+00	0.1	8.4E-01	0.2	1.0E+00	0.1	8.4E-01	0.1	7.8E-01
26774	MFSD6L	A_23_P340218	NM_152599	1.2	1.08E-02	1.2	1.17E-02	2.0	1.08E-02	1.0	9.30E-03	1.3	4.52E-03	1.3	1.20E-02	1.8	4.52E-03	1.1	3.01E-03	0.1	1.0E+00	0.1	1.0E+00	0.1	8.3E-01	0.1	1.0E+00	-0.2	2.8E-01	0.1	1.0E+00	-0.2	2.8E-01	-0.4	2.3E-01
26738	C1orf64	A_32_P232455	NM_178840	1.2	3.77E-04	1.1	3.77E-04	1.2	2.94E-03	1.9	1.25E-06	0.9	1.36E-01	0.9	1.36E-01	0.8	1.48E-01	1.2	1.32E-02	0.1	1.0E+00	0.1	1.0E+00	-0.1	8.4E-01	0.4	1.0E+00	-0.2	6.8E-01	0.1	1.0E+00	-0.3	2.7E-01	-0.2	6.8E-01
39175	LMO2	A_23_P53126	NM_005574	1.2	3.53E-03	1.2	3.53E-03	1.2	3.71E-02	1.1	2.43E-03	0.8	5.04E-02	0.8	5.04E-02	0.7	1.36E-01	0.9	2.22E-02	0.0	1.0E+00	-0.1	1.0E+00	0.0	9.6E-01	0.0	1.0E+00	-0.4	5.9E-01	-0.3	1.0E+00	-0.4	4.8E-01	-0.5	5.9E-01
24710	AK127078	A_23_P206120	AK127078	1.2	1.65E-03	0.8	5.38E-03	2.0	1.65E-03	1.2	6.13E-04	0.5	4.06E-01	0.2	8.12E-01	1.1	3.79E-01	0.5	4.06E-01	-0.3	1.0E+00	0.2	1.0E+00	-0.4	7.2E-01	-0.3	1.0E+00	-0.3	8.7E-01	0.1	1.0E+00	-0.4	5.6E-01	-0.3	8.7E-01
43282	AIM1	A_23_P70785	NM_001624	1.2	5.29E-04	1.4	3.09E-04	0.9	1.89E-02	1.2	5.29E-04	1.2	1.72E-02	1.2	1.72E-02	0.9	2.04E-01	1.6	1.03E-03	0.2	1.0E+00	0.2	1.0E+00	0.0	9.6E-01	0.2	1.0E+00	-0.1	9.3E-01	0.1	1.0E+00	-0.1	7.7E-01	-0.1	9.3E-01
37672	MMP19	A_23_P203888	NM_002429	1.2	9.57E-03	0.7	2.60E-02	1.6	9.57E-03	1.2	3.19E-03	0.6	3.68E-01	-0.2	8.65E-01	0.9	3.17E-01	0.6	3.68E-01	-0.1	1.0E+00	-0.1	1.0E+00	0.1	9.0E-01	-0.1	1.0E+00	0.1	9.3E-01	-0.1	1.0E+00	0.1	8.7E-01	0.2	9.3E-01
10281	ATP2C2	A_23_P117992	NM_014861	1.2	3.69E-03	1.2	3.18E-03	1.2	3.44E-02	1.1	3.69E-03	1.0	9.48E-03	1.0	9.48E-03	0.8	4.25E-02	1.2	5.40E-04	0.1	1.0E+00	0.0	1.0E+00	0.1	8.9E-01	0.1	1.0E+00	0.2	8.3E-01	0.2	1.0E+00	0.1	7.6E-01	0.2	8.3E-01
19536	COL1A1	A_23_P207520	Z74615	1.2	1.55E-04	1.2	1.56E-03	2.6	1.55E-04	1.1	3.90E-05	1.1	1.25E-02	1.1	2.10E-02	1.3	1.25E-02	1.1	7.18E-03	0.2	9.7E-01	0.1	1.0E+00	0.2	7.1E-01	0.3	9.7E-01	-0.1	6.9E-01	0.0	1.0E+00	-0.1	6.9E-01	-0.2	6.1E-01
31334	CHST7	A_23_P319617	NM_019886	1.2	6.89E-04	1.2	6.89E-04	1.2	9.55E-03	1.1	3.44E-04	1.3	7.83E-03	1.3	7.83E-03	1.3	5.15E-03	1.1	1.22E-02	0.1	1.0E+00	0.2	1.0E+00	0.1	7.8E-01	0.1	1.0E+00	0.1	9.4E-01	0.1	1.0E+00	0.1	7.9E-01	0.0	9.4E-01
41095	THC2499508	A_32_P147603	THC2499508	1.2	2.14E-03	1.2	1.41E-02	1.8	2.07E-03	0.8	2.14E-03	0.9	6.94E-02	0.9	8.54E-03	1.0	6.94E-02	0.7	1.73E-01	0.0	1.0E+00	0.0	1.0E+00	-0.1	7.8E-01	0.0	1.0E+00	-0.2	9.0E-01	-0.1	1.0E+00	-0.3	7.4E-01	-0.2	9.0E-01
28883	DYNLL2	A_23_P54991	NM_080677	1.2	2.38E-03	0.7	7.97E-03	1.2	2.38E-03	1.6	7.12E-06	0.5	6.80E-02	0.4	1.08E-01	0.5	6.80E-02	0.7	1.99E-03	0.1	1.0E+00	0.1	1.0E+00	0.1	9.0E-01	0.0	1.0E+00	0.0	9.8E-01	0.0	1.0E+00	-0.1	8.4E-01	0.0	9.8E-01
9904	C7orf41	A_32_P100439	NM_152793	1.2	5.52E-03	1.2	1.25E-02	1.5	5.23E-03	0.7	5.52E-03	1.1	5.08E-03	1.2	5.08E-03	1.1	3.67E-02	1.0	1.52E-03	0.3	1.0E+00	0.3	1.0E+00	0.0	9.6E-01	0.3	1.0E+00	-0.1	9.0E-01	-0.1	1.0E+00	-0.2	5.8E-01	-0.1	9.0E-01
25431	LOC441461	A_32_P177955	BC030123	1.2	3.44E-03	1.0	3.44E-03	1.2	1.57E-02	1.3	2.63E-04	1.3	3.92E-03	1.3	3.92E-03	1.4	4.73E-03	1.3	8.95E-04	0.1	1.0E+00	0.1	1.0E+00	0.0	9.6E-01	0.3	1.0E+00	-0.2	8.1E-01	0.0	1.0E+00	-0.3	3.6E-01	-0.2	8.1E-01
329	LOC374491	A_32_P89827	NR_002815	1.2	1.89E-02	0.6	4.06E-02	1.3	1.89E-02	1.2	1.11E-03	0.4	7.00E-01	0.2	8.84E-01	0.4	7.00E-01	0.5	5.73E-01	0.0	1.0E+00	-0.1	1.0E+00	0.4	7.1E-01	0.0	1.0E+00	0.3	6.0E-01	0.0	1.0E+00	0.4	2.6E-01	0.3	6.0E-01
37455	SERPINB1	A_23_P214330	NM_030666	1.2	2.94E-03	1.2	6.19E-03	1.6	2.94E-03	1.1	5.81E-04	1.1	2.46E-03	1.1	5.29E-03	1.1	2.46E-03	1.2	1.40E-03	0.2	1.0E+00	0.1	1.0E+00	0.2	7.6E-01	0.2	1.0E+00	-0.3	6.4E-01	-0.1	1.0E+00	-0.4	3.5E-01	-0.3	6.4E-01
27961	PIM2	A_24_P379104	NM_006875	1.2	2.27E-04	1.1	6.62E-04	2.2	2.27E-04	1.2	1.86E-05	1.0	6.24E-02	1.0	7.58E-02	1.0	6.24E-02	1.1	7.06E-03	0.0	1.0E+00	-0.2	1.0E+00	0.0	9.6E-01	0.1	1.0E+00	-0.1	9.0E-01	-0.1	1.0E+00	-0.2	6.3E-01	-0.1	9.0E-01
30896	CHRFAM7A	A_32_P35969	CR605947	1.2	3.43E-03	1.0	3.43E-03	1.2	1.09E-02	1.4	1.07E-04	1.3	1.63E-02	1.1	9.35E-02	1.6	1.11E-02	1.3	1.63E-02	-0.1	1.0E+00	-0.2	1.0E+00	-0.1	8.5E-01	0.2	1.0E+00	-0.1	8.7E-01	-0.2	1.0E+00	-0.1	6.2E-01	-0.1	8.7E-01
40643	NOS2	A_23_P502464	NM_000625	1.2	3.30E-03	1.2	3.30E-03	1.1	8.11E-03	1.2	9.28E-05	1.0	2.67E-02	1.0	2.67E-02	0.9	2.94E-02	1.2	1.18E-02	0.0	1.0E+00	0.1	1.0E+00	0.0	9.9E-01	0.0	1.0E+00	0.0	8.7E-01	0.0	1.0E+00	0.0	8.7E-01	-0.1	8.7E-01
35320	MAGEA6	A_23_P136870	NM_175868	1.2	2.10E-02	1.2	2.10E-02	1.9	1.29E-03	0.9	3.72E-02	0.3	8.49E-01	-0.4	8.49E-01	2.1	5.18E-01	0.3	8.49E-01	-0.5	1.0E+00	-0.3	1.0E+00	-0.6	8.1E-01	-0.5	1.0E+00	0.1	9.1E-01	0.0	1.0E+00	0.1	9.1E-01	0.5	8.0E-01
20906	CSH1	A_23_P381347	NM_022640	1.2	1.33E-02	1.2	1.33E-02	1.4	2.76E-02	1.1	3.98E-03	0.2	8.69E-01	0.0	9.92E-01	0.2	8.69E-01	0.3	8.50E-01	-0.1	1.0E+00	-0.1	1.0E+00	0.2	8.0E-01	-0.2	1.0E+00	0.4	4.2E-01	0.0	1.0E+00	0.5	6.9E-02	0.4	4.2E-01
42004	ZNF80	A_23_P256526	NM_007136	1.2	6.15E-05	0.8	1.86E-03	2.2	6.15E-05	1.2	7.56E-06	0.7	1.26E-01	0.2	8.29E-01	1.6	1.09E-01	0.7	1.26E-01	0.0	1.0E+00	0.0	1.0E+00	0.3	7.3E-01	-0.1	1.0E+00	0.1	8.6E-01	0.0	1.0E+00	0.2	3.6E-01	0.1	8.6E-01
10736	HECA	A_23_P59349	NM_016217	1.2	1.62E-03	1.2	1.62E-03	1.2	1.30E-02	1.5	1.50E-04	0.8	3.27E-02	0.8	3.27E-02	0.7	4.94E-02	1.1	3.93E-03	0.2	1.0E+00	0.2	1.0E+00	0.2	7.1E-01	0.5	1.0E+00	-0.4	7.2E-01	-0.2	1.0E+00	-0.4	5.2E-01	-0.4	7.2E-01
11886	ZNF541	A_23_P50517	NM_001101419	1.2	5.12E-04	1.3	1.85E-03	1.2	5.12E-04	1.1	1.02E-05	0.8	1.45E-01	0.8	1.45E-01	0.8	1.53E-01	0.9	3.72E-02	0.0	1.0E+00	-0.1	1.0E+00	0.1	7.3E-01	0.0	1.0E+00	0.1	9.1E-01	-0.1	1.0E+00	0.1	4.7E-01	0.1	9.1E-01
42043	NOS2	A_23_P502464	NM_000625	1.2	7.51E-03	1.2	7.51E-03	0.9	3.01E-02	1.2	3.37E-04	0.8	2.16E-02	0.8	2.16E-02	0.7	5.39E-02	1.1	6.00E-03	0.0	1.0E+00	0.0	1.0E+00	0.0	9.7E-01	0.1	1.0E+00	-0.2	4.1E-01	-0.1	1.0E+00	-0.2	1.5E-01	-0.2	4.1E-01
6025	KCNQ2	A_23_P351204	NM_172109	1.2	9.62E-03	1.2	9.62E-03	1.2	1.97E-02	0.9	3.23E-03	1.1	1.85E-03	1.5	1.85E-03	1.1	1.46E-03	1.0	2.95E-02	0.1	1.0E+00	0.1	1.0E+00	0.0	9.6E-01	0.2	1.0E+00	-0.1	9.5E-01	-0.1	1.0E+00	-0.1	8.8E-01	0.1	9.5E-01
25259	MGC16121	A_23_P159986	BC007360	1.2	1.57E-03	1.6	1.57E-03	0.8	4.50E-02	1.2	2.90E-04	0.8	9.31E-02	1.4	2.57E-02	0.7	1.83E-01	0.8	9.31E-02	0.4	7.2E-01	0.5	4.1E-01	0.2	7.2E-01	0.4	1.0E+00	0.5	5.1E-01	0.5	1.0E+00	0.4	4.8E-01	0.5	5.1E-01
4576	LOC440104	A_24_P93703	AK057179	1.2	5.14E-03	1.1	5.14E-03	1.6	1.12E-02	1.2	1.35E-03	0.8	3.75E-02	0.6	3.75E-02	0.8	1.18E-01	0.8	1.98E-03	0.0	1.0E+00	0.1	1.0E+00	-0.1	7.2E-01	0.0	1.0E+00	0.0	9.8E-01	0.0	1.0E+00	-0.1	8.9E-01	0.1	9.8E-01
24912	ID4	A_23_P59375	NM_001546	1.2	9.12E-04	1.2	9.12E-04	0.9	6.48E-03	1.4	9.93E-05	1.1	4.59E-02	0.6	1.11E-01	1.1	3.28E-02	1.1	4.59E-02	0.0	1.0E+00	0.0	1.0E+00	-0.1	7.7E-01	0.1	1.0E+00	-0.3	5.5E-01	-0.1	1.0E+00	-0.3	2.8E-01	-0.3	5.5E-01
15542	PRSS2	A_23_P310274	NM_002770	1.2	8.03E-03	0.8	9.22E-03	1.4	8.03E-03	1.2	3.63E-04	0.7	2.14E-02	0.2	6.52E-01	0.9	2.14E-02	0.7	6.34E-03	-0.1	1.0E+00	-0.1	1.0E+00	0.0	9.7E-01	-0.2	1.0E+00	0.1	9.2E-01	0.1	1.0E+00	0.2	7.1E-01	0.1	9.2E-01
30489	CACNA1H	A_23_P3483	NM_021098	1.2	2.20E-02	1.2	7.34E-03	1.5	2.20E-02	1.2	4.66E-02	0.6	7.15E-01	-0.2	9.21E-01	1.2	4.86E-01	0.6	7.15E-01	-0.1	1.0E+00	-0.1	1.0E+00	-0.1	9.3E-01	0.0	1.0E+00	0.7	3.1E-01	0.5	1.0E+00	0.7	3.1E-01	1.0	2.8E-01
23402	WNT10A	A_23_P102113	NM_025216	1.2	1.17E-02	1.0	1.17E-02	1.4	2.31E-02	1.2	1.85E-03	0.8	6.88E-02	0.8	6.88E-02	0.6	1.45E-01	0.9	1.79E-02	0.1	1.0E+00	0.1	1.0E+00	0.1	7.5E-01	0.1	1.0E+00	0.2	8.3E-01	0.2	1.0E+00	0.1	8.0E-01	0.2	8.3E-01
15716	THC2672319	A_32_P163891	THC2672319	1.2	2.33E-03	0.8	2.96E-03	1.2	2.33E-03	1.2	3.89E-05	0.2	7.97E-01	0.2	8.07E-01	0.2	7.97E-01	0.6	2.64E-01	-0.1	1.0E+00	-0.1	1.0E+00	0.1	8.2E-01	-0.1	1.0E+00	0.2	6.8E-01	-0.1	1.0E+00	0.2	2.9E-01	0.2	6.8E-01
23155	CXCR3	A_23_P114299	NM_001504	1.2	1.43E-02	1.0	4.66E-02	2.0	1.43E-02	1.2	1.03E-02	1.4	2.30E-02	1.1	5.53E-02	2.0	2.30E-02	1.4	4.37E-03	0.0	1.0E+00	-0.1	1.0E+00	0.2	7.9E-01	0.0	1.0E+00	-0.2	8.4E-01	-0.1	1.0E+00	-0.2	8.1E-01	-0.3	8.4E-01
6951	NTN5	A_32_P394951	NM_145807	1.2	3.52E-04	1.2	9.43E-04	1.7	3.52E-04	1.1	1.70E-05	0.9	6.56E-02	0.9	6.56E-02	0.7	7.23E-02	1.0	1.02E-02	0.0	1.0E+00	0.0	1.0E+00	0.1	7.4E-01	0.0	1.0E+00	0.0	9.6E-01	-0.1	1.0E+00	0.1	8.1E-01	0.0	9.6E-01
22708	A_23_P111766	A_23_P111766	A_23_P111766	1.2	2.05E-03	0.7	9.91E-03	1.5	2.05E-03	1.2	9.77E-05	0.8	2.95E-02	0.3	4.76E-01	1.2	2.95E-02	0.8	5.50E-03	0.0	1.0E+00	-0.1	1.0E+00	0.1	8.4E-01	0.0	1.0E+00	0.1	9.2E-01	0.1	1.0E+00	-0.1	8.8E-01	0.1	9.2E-01
34404	HVCN1	A_23_P151166	NM_001040107	1.2	5.11E-04	1.2	5.11E-04	0.7	7.63E-03	1.2	5.35E-06	1.0	1.31E-02	1.0	1.31E-02	0.9	2.95E-02	1.2	9.61E-04	0.1	1.0E+00	0.1	1.0E+00	0.1	7.1E-01	0.1	1.0E+00	0.0	9.8E-01	-0.1	1.0E+00	0.0	8.8E-01	0.0	9.8E-01
39961	DLX2	A_23_P28598	NM_004405	1.2	7.35E-04	0.8	2.17E-02	2.2	7.35E-04	1.2	2.07E-04	1.1	2.22E-02	1.1	1.76E-02	1.4	9.08E-02	1.1	2.22E-02	-0.2	1.0E+00	-0.2	1.0E+00	-0.5	7.1E-01	-0.1	1.0E+00	-0.3	4.0E-01	-0.2	1.0E+00	-0.5	6.6E-02	-0.3	4.0E-01
13804	BDKRB2	A_23_P304897	NM_000623	1.2	5.56E-04	1.1	5.56E-04	1.2	3.03E-03	1.7	7.36E-06	1.5	3.78E-02	0.9	8.74E-02	1.5	3.78E-02	1.6	4.42E-03	-0.1	1.0E+00	-0.1	1.0E+00	-0.1	8.3E-01	0.5	1.0E+00	-0.3	7.9E-01	-0.2	1.0E+00	-0.6	1.8E-01	-0.3	7.9E-01
21804	USP20	A_23_P217028	NM_001008563	1.2	7.63E-03	1.2	7.63E-03	1.8	1.03E-02	1.1	4.44E-03	0.9	1.02E-01	0.9	1.02E-01	0.8	1.26E-01	1.0	1.62E-02	0.3	1.0E+00	0.3	1.0E+00	0.3	7.5E-01	0.3	1.0E+00	0.0	9.9E-01	0.2	1.0E+00	0.0	9.6E-01	0.0	9.9E-01
16708	GDPD3	A_23_P26511	NM_024307	1.2	1.34E-03	1.3	1.34E-03	0.9	1.55E-02	1.2	1.61E-04	1.4	2.64E-02	1.5	2.64E-02	1.4	5.50E-02	1.4	1.07E-02	0.1	1.0E+00	0.1	1.0E+00	0.1	8.5E-01	0.3	1.0E+00	0.0	9.8E-01	0.1	1.0E+00	0.0	9.6E-01	0.0	9.8E-01
10477	LMO2	A_23_P53126	NM_005574	1.2	3.57E-03	1.3	3.57E-03	1.2	3.97E-02	1.2	1.93E-03	1.0	1.06E-02	0.9	2.08E-02	1.1	8.83E-03	1.0	1.06E-02	-0.1	1.0E+00	-0.1	1.0E+00	-0.1	9.3E-01	0.0	1.0E+00	-0.4	7.1E-01	-0.2	1.0E+00	-0.4	4.4E-01	-0.4	7.1E-01
25274	CCDC88B	A_23_P24389	NM_032251	1.2	1.34E-04	1.2	4.08E-03	1.9	1.34E-04	1.2	2.59E-06	1.0	8.66E-03	1.3	8.66E-03	1.0	1.62E-02	1.0	5.02E-03	0.1	1.0E+00	0.1	1.0E+00	0.0	9.0E-01	0.2	1.0E+00	-0.3	7.5E-01	-0.1	1.0E+00	-0.3	5.7E-01	-0.3	7.5E-01
45004	PLEKHH3	A_32_P172188	NM_024927	1.2	1.58E-02	1.2	1.58E-02	1.8	2.21E-02	1.2	1.00E-02	1.0	2.99E-02	0.8	4.46E-02	1.0	2.99E-02	1.2	4.96E-03	0.3	8.1E-01	0.5	8.1E-01	0.3	7.1E-01	0.3	1.0E+00	-0.2	9.1E-01	0.1	1.0E+00	-0.2	7.4E-01	-0.2	9.1E-01
8126	MDK	A_23_P116235	NM_001012334	1.2	3.82E-03	1.1	3.82E-03	1.4	1.16E-02	1.2	1.14E-03	1.2	9.61E-03	1.0	1.03E-02	1.2	9.61E-03	1.3	6.22E-04	0.0	1.0E+00	0.1	1.0E+00	0.0	9.8E-01	0.0	1.0E+00	-0.1	9.6E-01	0.1	1.0E+00	-0.1	9.0E-01	-0.1	9.6E-01
36052	IFFO1	A_23_P87742	NM_001039670	1.2	3.04E-03	0.9	1.83E-02	1.2	3.04E-03	1.2	2.66E-04	0.6	5.43E-01	0.0	9.97E-01	0.7	5.43E-01	0.6	5.30E-01	0.0	1.0E+00	0.1	1.0E+00	0.0	9.8E-01	-0.1	1.0E+00	0.3	6.7E-01	0.2	1.0E+00	0.5	2.3E-01	0.3	6.7E-01
44485	HSPA6	A_23_P114903	NM_002155	1.2	1.12E-03	1.5	7.73E-04	1.2	2.25E-02	1.1	1.12E-03	1.0	4.34E-02	1.1	4.34E-02	0.8	7.22E-02	1.0	1.24E-02	0.2	1.0E+00	0.2	1.0E+00	0.1	8.7E-01	0.3	1.0E+00	0.0	9.1E-01	0.0	1.0E+00	-0.1	8.0E-01	0.1	9.1E-01
26235	MMP25	A_23_P376557	NM_022468	1.2	1.71E-02	1.2	3.56E-03	1.3	1.71E-02	0.8	1.83E-02	1.0	2.93E-01	0.8	5.15E-01	1.1	2.93E-01	1.0	2.57E-01	-0.4	1.0E+00	-0.3	1.0E+00	-0.5	7.1E-01	-0.4	1.0E+00	0.2	8.8E-01	0.2	1.0E+00	0.4	5.6E-01	0.2	8.8E-01
792	TF	A_23_P212500	NM_001063	1.2	5.43E-03	1.5	5.43E-03	1.0	1.90E-02	1.2	4.08E-04	1.0	5.15E-01	1.0	5.15E-01	0.7	5.78E-01	1.1	3.02E-01	0.0	1.0E+00	0.0	1.0E+00	0.6	7.1E-01	-0.1	1.0E+00	0.2	5.1E-01	0.0	1.0E+00	0.2	3.8E-01	0.2	5.1E-01
6369	SOHLH1	A_24_P307964	NM_001012415	1.2	1.80E-03	1.0	2.29E-03	1.6	1.80E-03	1.2	1.89E-04	0.7	1.67E-01	0.6	4.63E-01	1.0	1.09E-01	0.7	1.67E-01	0.1	1.0E+00	0.1	1.0E+00	0.0	1.0E+00	0.1	1.0E+00	-0.1	7.8E-01	0.0	1.0E+00	-0.2	3.6E-01	-0.1	7.8E-01
5841	CD22	A_23_P209055	NM_001771	1.2	1.01E-03	0.7	1.67E-02	1.5	1.01E-03	1.2	3.33E-05	0.5	1.55E-01	0.5	1.55E-01	0.4	4.35E-01	1.0	1.05E-03	0.0	1.0E+00	0.0	1.0E+00	-0.1	9.0E-01	0.0	1.0E+00	0.0	9.5E-01	-0.1	1.0E+00	0.0	9.1E-01	0.0	9.5E-01
4174	CPM	A_23_P162668	NM_001874	1.2	2.80E-02	1.3	2.80E-02	0.7	3.56E-02	1.2	8.58E-05	1.3	3.54E-02	1.2	3.54E-02	1.6	4.25E-02	1.3	3.54E-03	0.1	1.0E+00	0.1	1.0E+00	-0.1	7.3E-01	0.1	1.0E+00	-0.2	7.7E-01	0.0	1.0E+00	-0.3	3.6E-01	-0.2	7.7E-01
31653	HOMER3	A_23_P39364	NM_004838	1.2	1.82E-03	1.2	2.58E-03	1.5	1.82E-03	1.0	7.47E-04	1.2	4.97E-03	1.2	3.64E-03	1.4	4.97E-03	1.1	5.50E-03	0.0	1.0E+00	0.0	1.0E+00	0.0	9.6E-01	0.1	1.0E+00	0.1	7.2E-01	0.1	1.0E+00	0.1	7.0E-01	0.2	7.2E-01
22676	DENND2C	A_23_P46315	CR749576	1.2	4.66E-04	1.2	4.76E-03	1.7	4.66E-04	1.1	2.06E-05	1.2	1.59E-02	1.2	1.93E-02	1.3	1.59E-02	1.0	1.96E-03	0.3	1.0E+00	0.2	1.0E+00	0.3	7.1E-01	0.3	1.0E+00	-0.1	9.5E-01	-0.1	1.0E+00	-0.2	7.1E-01	-0.1	9.5E-01
29702	ELFN2	A_32_P219279	NM_052906	1.2	1.32E-03	1.2	1.32E-03	2.3	1.47E-03	1.2	8.85E-04	1.3	7.03E-02	1.3	9.83E-02	1.3	7.03E-02	1.3	3.42E-02	0.2	1.0E+00	0.5	1.0E+00	0.1	8.7E-01	0.2	1.0E+00	0.2	9.1E-01	0.2	1.0E+00	0.1	9.1E-01	0.2	8.8E-01
28657	PI3	A_23_P210465	NM_002638	1.2	1.49E-03	0.7	6.67E-03	1.7	1.49E-03	1.2	7.26E-04	0.3	7.78E-01	-0.6	6.56E-01	0.3	7.78E-01	0.3	8.08E-01	0.1	1.0E+00	0.1	1.0E+00	0.3	7.2E-01	-0.2	1.0E+00	0.6	2.7E-01	0.1	1.0E+00	0.6	1.4E-01	0.7	2.7E-01
2109	MGC4294	A_23_P102681	BC002831	1.2	3.24E-03	1.2	3.24E-03	1.4	7.17E-03	1.2	4.40E-04	1.0	2.50E-02	1.0	2.50E-02	1.0	6.06E-02	1.3	8.36E-03	-0.1	1.0E+00	0.1	1.0E+00	-0.3	7.1E-01	-0.1	1.0E+00	0.1	9.3E-01	0.1	1.0E+00	0.0	9.3E-01	0.1	9.0E-01
43723	AK000085	A_32_P91273	AK000085	1.2	1.84E-04	1.2	1.84E-04	1.0	1.16E-03	1.2	1.13E-05	1.4	1.45E-02	1.4	1.45E-02	1.2	3.05E-02	1.4	2.50E-03	0.2	1.0E+00	0.2	1.0E+00	0.1	8.2E-01	0.4	1.0E+00	0.0	9.4E-01	0.0	1.0E+00	-0.2	2.2E-01	0.0	9.4E-01
5279	LMO2	A_23_P53126	NM_005574	1.2	3.54E-03	1.3	3.54E-03	1.2	3.47E-02	1.2	2.24E-03	0.9	2.15E-02	0.8	2.99E-02	0.9	1.69E-02	0.9	2.15E-02	0.0	1.0E+00	0.0	1.0E+00	0.0	9.8E-01	0.0	1.0E+00	-0.4	5.8E-01	-0.3	1.0E+00	-0.4	4.4E-01	-0.5	5.8E-01
18577	A_23_P247	A_23_P247	A_23_P247	1.2	3.17E-03	1.2	3.17E-03	1.5	6.41E-03	1.1	1.45E-03	1.0	1.47E-01	0.9	1.47E-01	1.0	1.96E-01	1.2	1.21E-02	0.1	1.0E+00	0.0	1.0E+00	0.2	7.3E-01	0.1	1.0E+00	0.0	1.0E+00	0.1	1.0E+00	0.0	9.4E-01	0.0	1.0E+00
41821	TCEB3B	A_23_P141706	NM_016427	1.2	8.50E-04	0.9	3.72E-03	1.2	8.50E-04	2.1	1.30E-06	0.3	9.02E-02	0.3	2.92E-01	0.3	9.02E-02	0.8	2.78E-02	0.0	1.0E+00	0.0	1.0E+00	0.1	7.1E-01	0.0	1.0E+00	0.0	9.5E-01	0.0	1.0E+00	0.0	9.5E-01	0.1	8.8E-01
36221	C5orf27	A_23_P408323	AK314737	1.2	8.50E-04	1.2	3.56E-03	1.2	8.50E-04	1.7	1.07E-06	1.3	1.42E-02	0.7	5.36E-02	1.4	1.42E-02	1.3	1.74E-03	0.0	1.0E+00	0.0	1.0E+00	-0.1	8.1E-01	0.0	1.0E+00	0.0	9.4E-01	-0.1	1.0E+00	0.1	6.3E-01	0.0	9.4E-01
13368	NOS2	A_23_P502464	NM_000625	1.2	1.46E-02	1.2	1.46E-02	1.1	2.34E-02	1.3	2.93E-04	1.2	1.25E-02	1.2	1.25E-02	0.9	4.20E-02	1.4	4.18E-03	0.1	1.0E+00	0.0	1.0E+00	0.1	8.6E-01	0.1	1.0E+00	-0.1	7.6E-01	0.0	1.0E+00	-0.1	6.7E-01	-0.1	7.6E-01
30418	YPEL1	A_23_P40548	NM_013313	1.2	5.78E-03	1.2	7.00E-03	1.5	5.78E-03	1.0	1.66E-03	0.9	6.84E-02	1.0	4.33E-02	0.9	6.84E-02	0.8	1.01E-01	0.1	1.0E+00	0.1	1.0E+00	0.1	8.5E-01	0.1	1.0E+00	-0.1	1.0E+00	-0.1	1.0E+00	-0.1	9.4E-01	0.0	1.0E+00
39888	DNAJC22	A_23_P253661	NM_024902	1.2	1.70E-03	1.0	1.03E-02	1.7	1.70E-03	1.2	1.09E-04	0.8	1.33E-01	0.4	6.22E-01	0.8	1.33E-01	0.8	3.60E-02	0.0	1.0E+00	0.2	1.0E+00	0.0	9.4E-01	0.1	1.0E+00	-0.3	6.4E-01	0.0	1.0E+00	-0.3	3.5E-01	-0.3	6.4E-01
42618	LMO2	A_23_P53126	NM_005574	1.2	5.99E-03	1.2	5.99E-03	1.4	3.74E-02	1.2	4.07E-03	1.0	9.09E-03	0.8	9.09E-03	1.0	2.96E-03	1.0	1.62E-02	-0.1	1.0E+00	-0.1	1.0E+00	-0.1	8.5E-01	-0.2	1.0E+00	-0.3	8.5E-01	-0.2	1.0E+00	-0.4	5.4E-01	-0.3	8.5E-01
18817	ITGA5	A_23_P36562	NM_002205	1.2	4.37E-03	1.0	4.37E-03	1.2	9.74E-03	1.4	1.12E-04	0.9	1.01E-01	0.8	1.70E-01	0.9	1.01E-01	1.3	1.29E-02	0.1	1.0E+00	0.1	1.0E+00	0.2	7.1E-01	-0.1	1.0E+00	0.2	9.4E-01	0.0	1.0E+00	0.3	7.3E-01	0.2	9.4E-01
18811	FAM122C	A_24_P674479	AK090611	1.2	9.32E-03	1.2	9.32E-03	1.4	1.23E-02	1.1	1.13E-03	0.9	4.22E-03	1.3	2.63E-03	0.8	2.84E-02	0.9	4.22E-03	0.1	1.0E+00	0.1	1.0E+00	0.0	9.8E-01	0.2	1.0E+00	-0.4	5.8E-01	-0.1	1.0E+00	-0.4	4.4E-01	-0.4	5.8E-01
19394	ZFYVE1	A_24_P287503	NM_021260	1.2	1.02E-02	1.2	1.86E-02	1.8	1.02E-02	1.0	5.27E-03	0.9	4.00E-02	0.7	4.15E-02	1.0	4.00E-02	0.9	1.62E-02	0.1	1.0E+00	0.1	1.0E+00	0.1	7.1E-01	0.1	1.0E+00	-0.3	8.8E-01	0.0	1.0E+00	-0.3	7.3E-01	-0.3	8.8E-01
13094	MT3	A_23_P129629	NM_005954	1.2	2.24E-02	1.2	3.77E-02	2.0	1.57E-02	0.9	2.24E-02	0.9	4.63E-02	0.7	8.48E-02	1.3	4.63E-02	0.9	2.36E-02	-0.1	1.0E+00	-0.1	1.0E+00	0.3	7.1E-01	-0.1	1.0E+00	0.2	8.1E-01	0.3	1.0E+00	0.2	6.6E-01	0.2	8.1E-01
14271	COLEC11	A_24_P388322	NM_199235	1.2	1.79E-02	1.2	1.79E-02	2.2	9.10E-03	0.8	3.86E-02	1.2	2.36E-02	1.2	2.36E-02	0.9	1.07E-01	1.2	1.52E-02	0.2	1.0E+00	0.2	1.0E+00	0.0	9.4E-01	0.2	1.0E+00	-0.3	8.1E-01	0.0	1.0E+00	-0.3	6.7E-01	-0.4	8.1E-01
42705	LMO2	A_23_P53126	NM_005574	1.2	3.93E-03	1.2	3.86E-03	1.3	3.38E-02	1.2	3.93E-03	0.9	1.03E-02	0.8	1.03E-02	0.9	6.81E-03	0.9	2.04E-02	-0.1	1.0E+00	-0.1	1.0E+00	-0.1	8.7E-01	-0.1	1.0E+00	-0.4	7.6E-01	-0.2	1.0E+00	-0.4	5.1E-01	-0.4	7.6E-01

Feature	Gene symbol	Probe	Transcript	72 hour								48 hours								24 hours								10 hours							
				siCASP8AP2								siCASP8AP2								siCASP8AP2								siCASP8AP2							
				Median	Median	1	1	3	3	6	6	Median	Median	1	1	3	3	6	6	Median	Median	1	1	3	3	6	6	Median	Median	1	1	3	3	6	6
				M	q	M	q	M	q	M	q	M	q	M	q	M	q	M	q	M	q	M	q	M	q	M	q	M	q	M	q	M	q	M	q
40407	MYL4	A_24_P188218	NM_002476	1.2	2.75E-03	1.2	2.95E-03	1.6	2.75E-03	0.8	1.94E-03	1.2	3.45E-03	1.3	3.45E-03	1.2	6.12E-03	1.0	1.18E-03	0.0	1.0E+00	0.1	1.0E+00	0.0	9.7E-01	0.0	1.0E+00	-0.1	7.1E-01	0.0	1.0E+00	-0.1	6.1E-01	-0.2	7.1E-01
36888	CCL27	A_23_P135248	NM_006664	1.2	4.31E-04	1.1	1.64E-03	1.4	4.31E-04	1.2	2.24E-06	0.7	1.90E-02	0.3	3.41E-01	1.0	8.17E-03	0.7	1.90E-02	0.1	1.0E+00	0.1	1.0E+00	0.0	9.5E-01	0.1	1.0E+00	0.0	9.7E-01	0.0	1.0E+00	-0.1	7.6E-01	0.0	9.7E-01
31816	LOC340888	A_24_P152845	XR_018726	1.2	1.58E-04	1.2	1.58E-04	1.1	1.73E-03	1.5	3.06E-06	1.0	1.19E-02	0.6	4.13E-02	1.0	1.19E-02	1.3	2.96E-04	0.1	1.0E+00	0.1	1.0E+00	0.0	9.2E-01	0.1	1.0E+00	0.0	9.8E-01	0.0	1.0E+00	0.0	9.4E-01	0.0	9.8E-01
30341	ABHD8	A_23_P50857	NM_024527	1.2	4.63E-03	1.2	1.04E-02	2.0	4.63E-03	1.1	1.75E-03	0.8	7.77E-02	0.8	7.91E-02	0.6	7.77E-02	1.1	3.67E-03	0.1	1.0E+00	0.1	1.0E+00	0.0	9.4E-01	0.1	1.0E+00	-0.2	9.1E-01	-0.1	1.0E+00	-0.3	7.2E-01	-0.2	9.1E-01
24880	SLC5A10	A_23_P328022	NM_152351	1.2	2.47E-04	1.2	2.90E-03	1.5	2.47E-04	1.2	1.12E-06	0.9	1.68E-01	0.6	3.59E-01	0.9	1.68E-01	0.9	1.00E-01	0.1	1.0E+00	0.1	1.0E+00	0.2	7.2E-01	0.1	1.0E+00	-0.1	9.2E-01	0.1	1.0E+00	-0.1	8.2E-01	-0.1	9.2E-01
12423	LOC255783	A_23_P386168	NM_178511	1.2	1.29E-03	1.2	1.96E-03	1.6	1.29E-03	1.2	3.76E-05	1.0	6.99E-03	0.8	2.84E-02	1.1	6.99E-03	1.0	3.02E-03	0.2	1.0E+00	0.1	1.0E+00	0.2	7.1E-01	0.2	1.0E+00	0.0	9.7E-01	0.0	1.0E+00	-0.1	9.0E-01	0.0	9.7E-01
10882	ATG2A	A_23_P361820	NM_015104	1.2	4.20E-03	1.4	4.20E-03	1.1	2.69E-02	1.2	5.45E-04	0.9	4.25E-02	0.9	4.25E-02	0.6	3.67E-01	1.2	1.55E-03	0.3	1.0E+00	0.3	1.0E+00	0.2	7.2E-01	0.3	1.0E+00	-0.2	9.1E-01	-0.1	1.0E+00	-0.3	7.4E-01	-0.2	9.1E-01
23432	EHD2	A_24_P156113	NM_014601	1.2	1.79E-03	1.2	2.43E-02	2.5	1.43E-03	1.2	1.79E-03	1.0	1.44E-01	0.9	2.20E-01	1.6	1.44E-01	1.0	1.13E-01	0.1	1.0E+00	0.1	1.0E+00	0.1	8.1E-01	0.0	1.0E+00	0.0	9.7E-01	0.1	1.0E+00	0.0	9.7E-01	-0.1	9.1E-01
26654	MAGIX	A_23_P84941	NM_024859	1.2	5.10E-03	1.2	5.10E-03	1.4	3.43E-03	0.7	5.52E-03	1.1	7.53E-02	1.2	7.53E-02	1.1	1.34E-01	1.1	3.54E-02	0.0	1.0E+00	0.0	1.0E+00	-0.2	7.1E-01	0.0	1.0E+00	-0.2	7.9E-01	0.0	1.0E+00	-0.2	7.1E-01	-0.3	7.9E-01
40661	PADI2	A_23_P201747	NM_007365	1.2	4.71E-04	1.4	4.71E-04	1.1	3.40E-03	1.2	9.90E-05	0.5	5.58E-01	0.5	6.53E-01	0.4	5.58E-01	0.8	3.28E-01	0.0	1.0E+00	0.0	1.0E+00	0.3	7.5E-01	-0.3	1.0E+00	0.2	7.4E-01	0.0	1.0E+00	0.2	7.4E-01	0.5	7.1E-01
24336	C1QTNF2	A_23_P92903	NM_031908	1.2	1.80E-04	1.2	1.80E-04	1.2	1.61E-03	1.6	3.44E-06	1.6	3.27E-03	1.6	1.01E-02	1.6	3.27E-03	1.5	1.82E-03	0.4	2.9E-01	0.4	2.2E-01	0.1	8.3E-01	0.5	2.9E-01	0.0	9.8E-01	0.0	1.0E+00	-0.1	8.5E-01	0.0	9.8E-01
1766	TP53I3	A_23_P5392	NM_004881	1.2	5.13E-04	1.4	5.13E-04	1.2	5.06E-03	1.1	2.04E-04	1.2	1.21E-03	1.6	5.71E-04	0.9	6.19E-03	1.2	1.21E-03	0.1	1.0E+00	0.2	1.0E+00	0.0	9.3E-01	0.1	1.0E+00	0.0	9.9E-01	0.1	1.0E+00	0.0	9.9E-01	0.0	9.7E-01
7639	PDCD4	A_23_P9932	NM_145341	1.2	2.63E-04	1.2	2.63E-04	1.2	1.00E-03	1.4	1.09E-05	1.0	9.41E-03	1.0	1.55E-02	1.1	9.41E-03	1.0	5.16E-03	0.1	1.0E+00	-0.1	1.0E+00	0.1	7.9E-01	0.2	1.0E+00	-0.1	1.0E+00	-0.1	1.0E+00	0.0	1.0E+00	-0.1	9.3E-01
15703	SHF	A_32_P183904	NM_138356	1.2	1.52E-03	1.2	1.52E-03	1.6	2.52E-03	1.2	1.16E-04	1.4	3.42E-03	1.3	1.64E-02	1.5	3.42E-03	1.4	1.92E-03	0.2	1.0E+00	0.1	1.0E+00	0.2	7.1E-01	0.2	1.0E+00	0.1	8.3E-01	0.0	1.0E+00	0.1	7.9E-01	0.1	8.3E-01
3638	TSKS	A_23_P16275	NM_021733	1.2	1.44E-03	1.1	5.32E-03	2.0	1.44E-03	1.2	2.09E-04	0.7	2.82E-01	0.5	5.40E-01	0.7	2.82E-01	0.7	1.72E-01	0.0	1.0E+00	0.0	1.0E+00	0.0	9.6E-01	0.0	1.0E+00	0.2	5.7E-01	0.2	1.0E+00	0.1	4.2E-01	0.2	5.7E-01
28920	TP63	A_23_P327380	NM_003722	1.2	1.40E-02	1.2	1.40E-02	1.2	4.80E-02	1.4	2.07E-03	1.3	2.13E-03	0.8	1.87E-02	1.3	2.13E-03	1.3	1.77E-03	0.0	1.0E+00	0.1	1.0E+00	0.0	9.0E-01	0.0	1.0E+00	-0.2	4.8E-01	0.0	1.0E+00	-0.3	1.7E-01	-0.2	4.8E-01
26801	JAK3	A_24_P59667	NM_000215	1.2	7.10E-04	0.9	9.70E-03	2.5	7.10E-04	1.2	1.74E-04	0.9	6.79E-02	0.9	7.29E-02	1.7	4.32E-02	0.8	6.79E-02	0.0	1.0E+00	0.0	1.0E+00	-0.1	8.5E-01	0.0	1.0E+00	-0.1	9.7E-01	-0.1	1.0E+00	-0.2	8.0E-01	-0.1	9.7E-01
6906	PFKFB3	A_24_P111096	NM_004566	1.2	1.98E-03	1.0	3.40E-02	1.5	1.98E-03	1.2	8.21E-05	0.5	4.92E-01	0.5	5.43E-01	0.5	4.92E-01	0.8	1.26E-01	0.2	1.0E+00	0.0	1.0E+00	0.3	7.1E-01	0.2	1.0E+00	-0.1	9.0E-01	0.0	1.0E+00	-0.1	8.6E-01	-0.1	9.0E-01
36827	SLC22A18AS	A_24_P372643	NM_007105	1.3	1.43E-03	1.3	1.14E-03	2.2	1.43E-03	1.2	1.47E-03	1.0	2.77E-01	0.4	7.26E-01	1.4	2.77E-01	1.0	1.16E-01	0.0	1.0E+00	0.0	1.0E+00	0.2	7.7E-01	0.0	1.0E+00	0.3	8.4E-01	0.1	1.0E+00	0.3	6.2E-01	0.4	8.4E-01
27444	COL1A1	A_23_P207520	Z74615	1.3	1.58E-04	1.3	2.96E-03	2.5	1.58E-04	1.0	7.22E-05	1.2	7.83E-03	1.2	7.83E-03	1.4	8.51E-03	1.2	3.60E-03	0.0	1.0E+00	0.0	1.0E+00	-0.1	8.5E-01	0.1	1.0E+00	-0.1	8.8E-01	0.0	1.0E+00	-0.1	5.9E-01	-0.1	8.8E-01
38985	NINJ2	A_23_P48109	NM_016533	1.3	6.70E-04	1.3	6.70E-04	1.1	1.29E-03	1.3	5.49E-06	1.0	1.09E-01	1.0	1.09E-01	0.4	5.65E-01	1.2	1.60E-02	0.2	1.0E+00	0.1	1.0E+00	0.3	7.1E-01	0.2	1.0E+00	-0.4	5.9E-01	-0.2	1.0E+00	-0.4	2.7E-01	-0.4	5.9E-01
9614	CSAG2	A_24_P567298	NM_004909	1.3	2.55E-03	0.9	3.07E-02	1.3	2.55E-03	1.4	1.11E-05	0.4	2.94E-01	0.4	4.27E-01	0.4	2.94E-01	0.6	6.79E-02	0.0	1.0E+00	0.0	1.0E+00	0.2	7.1E-01	0.0	1.0E+00	0.0	9.2E-01	0.0	1.0E+00	0.0	9.2E-01	0.1	6.8E-01
41471	IL7R	A_23_P404494	NM_002185	1.3	2.83E-03	0.7	4.40E-02	1.5	2.83E-03	1.3	3.98E-04	0.3	5.52E-01	0.1	9.15E-01	0.3	5.52E-01	0.4	4.08E-01	0.0	1.0E+00	0.1	1.0E+00	0.0	9.8E-01	-0.1	1.0E+00	-0.1	9.5E-01	0.1	1.0E+00	-0.2	6.3E-01	-0.1	9.5E-01
38482	MDGA1	A_23_P310460	NM_153487	1.3	6.60E-03	0.9	1.48E-02	1.8	6.60E-03	1.3	1.23E-03	1.4	7.36E-03	1.3	2.66E-02	1.4	7.36E-03	1.4	7.05E-03	0.2	1.0E+00	0.2	1.0E+00	-0.1	8.8E-01	0.2	1.0E+00	-0.4	1.7E-01	-0.1	1.0E+00	-0.4	6.0E-02	-0.4	1.7E-01
26473	CCDC96	A_32_P69930	NM_153376	1.3	4.26E-04	1.1	4.26E-04	1.7	1.23E-03	1.3	3.47E-05	0.9	3.56E-02	0.9	1.08E-01	1.2	3.56E-02	0.9	3.08E-02	0.1	1.0E+00	0.2	1.0E+00	0.1	7.1E-01	0.1	1.0E+00	0.0	9.7E-01	0.1	1.0E+00	0.0	9.7E-01	-0.1	8.6E-01
36598	PPFIA3	A_23_P320553	NM_003660	1.3	6.41E-03	1.3	1.28E-03	1.3	6.49E-03	0.8	6.41E-03	1.3	1.17E-02	1.2	1.75E-02	1.3	1.17E-02	1.5	1.52E-03	0.2	1.0E+00	0.3	1.0E+00	0.1	7.8E-01	0.2	1.0E+00	-0.1	7.8E-01	0.0	1.0E+00	-0.1	7.8E-01	-0.2	6.7E-01
40133	LMO2	A_23_P53126	NM_005574	1.3	6.59E-03	1.3	6.59E-03	1.3	4.68E-02	1.2	5.70E-03	1.0	1.11E-02	0.9	1.11E-02	1.1	1.72E-03	1.0	1.59E-02	-0.1	1.0E+00	-0.1	1.0E+00	-0.1	8.7E-01	-0.2	1.0E+00	-0.3	8.3E-01	-0.2	1.0E+00	-0.3	5.7E-01	-0.3	8.3E-01
1371	CLDN7	A_23_P164284	NM_001307	1.3	5.93E-03	1.3	5.93E-03	1.4	2.36E-02	1.2	2.44E-03	1.0	9.83E-03	1.0	9.83E-03	0.9	2.13E-02	1.0	1.40E-03	0.2	1.0E+00	0.2	1.0E+00	0.1	7.8E-01	0.3	1.0E+00	0.0	9.8E-01	0.0	1.0E+00	0.0	9.8E-01	0.1	9.4E-01
24213	ATP10A	A_23_P65659	NM_024490	1.3	1.66E-04	1.3	1.66E-04	1.7	2.59E-04	1.1	2.33E-05	1.0	3.08E-02	1.0	3.08E-02	0.8	5.52E-02	1.1	6.56E-03	0.2	1.0E+00	0.1	1.0E+00	0.2	7.3E-01	0.2	1.0E+00	0.1	5.7E-01	-0.1	1.0E+00	0.1	5.4E-01	0.2	5.7E-01
21850	GOLGA9P	A_24_P229669	NR_024074	1.3	1.94E-03	1.3	1.94E-03	1.6	4.50E-03	1.1	7.07E-04	0.5	2.80E-01	0.2	7.75E-01	0.5	2.80E-01	0.6	1.91E-01	0.1	1.0E+00	0.1	1.0E+00	0.3	7.2E-01	-0.3	1.0E+00	0.7	9.8E-02	0.1	1.0E+00	0.7	5.6E-02	0.9	9.8E-02
31720	C17orf59	A_23_P152963	NM_017622	1.3	1.86E-03	1.3	1.91E-03	2.0	1.86E-03	1.2	5.16E-04	1.0	1.44E-02	1.0	1.44E-02	1.1	1.89E-02	1.0	5.28E-03	0.3	1.0E+00	0.3	1.0E+00	0.4	6.3E-01	0.3	1.0E+00	-0.1	9.5E-01	-0.1	1.0E+00	-0.1	8.5E-01	0.1	9.5E-01
2863	DLX2	A_23_P28595	NM_004405	1.3	7.23E-04	1.0	1.40E-02	2.4	7.23E-04	1.3	2.13E-04	0.7	3.36E-01	0.6	3.36E-01	0.9	3.54E-01	0.7	1.83E-01	-0.1	1.0E+00	-0.2	1.0E+00	0.1	7.5E-01	-0.1	1.0E+00	0.0	9.2E-01	0.0	1.0E+00	0.0	9.2E-01	0.1	9.2E-01
24586	AK124080	A_32_P80610	AK124080	1.3	4.65E-04	1.1	4.65E-04	1.6	1.10E-03	1.3	7.64E-05	1.2	2.02E-02	1.2	3.85E-02	1.2	2.02E-02	1.5	5.32E-03	0.2	1.0E+00	0.2	1.0E+00	-0.1	7.7E-01	0.2	1.0E+00	-0.2	6.6E-01	0.0	1.0E+00	-0.2	3.2E-01	-0.2	6.6E-01
23189	LILRB5	A_23_P164784	NM_006840	1.3	1.28E-03	1.3	1.45E-03	1.2	1.28E-03	1.3	9.37E-06	0.8	4.56E-03	0.6	2.04E-02	0.8	4.56E-03	1.2	2.44E-04	0.1	1.0E+00	0.1	1.0E+00	0.1	8.3E-01	0.1	1.0E+00	0.0	9.9E-01	0.1	1.0E+00	0.0	9.2E-01	0.0	9.9E-01
26742	HIST1H3G	A_23_P42198	NM_003534	1.3	1.28E-03	1.4	1.28E-03	1.0	3.46E-02	1.3	1.01E-03	1.3	5.37E-02	1.3	5.37E-02	1.1	5.92E-02	1.8	5.01E-03	0.3	1.0E+00	0.4	1.0E+00	0.1	8.9E-01	0.3	1.0E+00	-0.1	9.2E-01	0.0	1.0E+00	-0.1	9.0E-01	-0.2	9.2E-01
36407	PELI2	A_23_P65532	NM_021255	1.3	1.74E-04	1.3	1.74E-04	1.8	8.73E-04	1.2	4.99E-05	0.8	1.67E-01	0.8	3.28E-01	0.8	1.67E-01	1.0	1.20E-01	0.0	1.0E+00	0.0	1.0E+00	0.0	9.8E-01	0.2	1.0E+00	-0.3	6.1E-01	-0.2	1.0E+00	-0.4	4.0E-01	-0.3	6.1E-01
24009	GIGYF1	A_24_P163574	NM_022574	1.3	1.09E-02	1.3	3.39E-02	2.8	3.45E-03	1.1	1.09E-02	0.4	7.30E-01	0.4	7.30E-01	1.1	5.82E-01	0.3	7.82E-01	-0.2	1.0E+00	0.2	1.0E+00	-0.2	9.0E-01	-0.2	1.0E+00	0.2	9.9E-01	0.2	1.0E+00	0.0	9.9E-01	0.2	9.4E-01
25907	AF086126	A_32_P97506	AF086126	1.3	1.78E-02	1.0	2.04E-02	1.3	1.78E-02	1.5	2.22E-04	0.5	1.37E-01	0.5	1.37E-01	0.3	5.49E-01	0.6	1.80E-02	0.2	1.0E+00	0.2	1.0E+00	-0.1	8.1E-01	0.3	1.0E+00	0.1	9.6E-01	0.1	1.0E+00	0.0	9.6E-01	0.1	9.1E-01
30306	POLD4	A_23_P127367	NM_021173	1.3	2.05E-04	1.1	1.56E-03	2.0	2.05E-04	1.3	1.17E-05	1.0	9.71E-03	0.8	1.70E-02	1.2	7.50E-03	1.0	9.71E-03	0.1	1.0E+00	0.1	1.0E+00	0.1	7.8E-01	0.0	1.0E+00	0.1	9.2E-01	0.1	1.0E+00	0.1	9.2E-01	0.1	9.1E-01
5458	GCNT1	A_23_P9232	NM_001490	1.3	1.48E-03	1.3	1.48E-03	1.3	3.05E-03	1.6	1.24E-05	1.2	3.06E-02	1.0	8.55E-02	1.2	3.06E-02	1.3	8.90E-03	0.1	1.0E+00	0.1	1.0E+00	-0.1	8.8E-01	0.3	1.0E+00	-0.2	3.0E-01	0.0	1.0E+00	-0.2	3.0E-01	-0.3	2.4E-01
41486	SERTAD1	A_23_P218463	NM_013376	1.3	1.68E-03	1.3	1.68E-03	1.4	1.07E-02	1.3	3.58E-04	1.0	6.11E-03	1.0	6.11E-03	1.0	8.70E-03	1.2	2.65E-03	0.1	1.0E+00	0.1	1.0E+00	0.1	8.0E-01	0.0	1.0E+00	0.1	9.2E-01	0.1	1.0E+00	0.0	9.2E-01	0.2	7.9E-01
42064	DUSP1	A_23_P110712	NM_004417	1.3	9.71E-03	1.0	1.06E-02	1.3	9.71E-03	1.3	2.68E-04	0.6	7.05E-02	0.6	1.11E-01	0.5	7.05E-02	0.8	2.42E-02	0.0	1.0E+00	0.0	1.0E+00	0.1	8.8E-01	0.0	1.0E+00	-0.3	4.9E-01	-0.2	1.0E+00	-0.3	3.2E-01	-0.4	4.9E-01
40292	ABAT	A_24_P330684	NM_000663	1.3	5.35E-03	0.9	9.32E-03	1.8	5.35E-03	1.3	7.34E-04	0.9	3.12E-02	0.5	1.62E-01	0.9	3.12E-02	1.0	5.43E-03	0.1	1.0E+00	0.1	1.0E+00	0.1	8.1E-01	0.0	1.0E+00	0.0	9.8E-01	0.1	1.0E+00	-0.1	7.5E-01	0.0	9.8E-01
28254	CDH24	A_23_P405267	AK057922	1.3	2.42E-03	1.3	2.42E-03	1.2	4.30E-03	1.4	1.64E-05	1.2	1.47E-02	1.7	8.74E-03	1.2	1.72E-02	1.2	1.47E-02	0.2	1.0E+00	0.2	1.0E+00	-0.1	9.0E-01	0.2	1.0E+00	-0.1	7.7E-01	0.0	1.0E+00	-0.1	7.7E-01	-0.4	3.8E-01
16514	TSC22D4	A_23_P250866	NM_030935	1.3	4.47E-04	1.1	4.47E-04	1.3	4.12E-03	1.3	4.13E-05	0.9	2.10E-02	0.9	2.10E-02	0.7	3.20E-01	1.2	9.56E-04	0.4	4.1E-01	0.5	1.5E-01	0.4	4.1E-01	0.3	1.0E+00	0.0	9.6E-01	0.0	1.0E+00	0.0	9.6E-01	-0.1	9.5E-01
36515	TPPP3	A_23_P26386	NM_016140	1.3	1.05E-02	1.3	1.05E-02	1.4	1.74E-02	1.0	2.51E-03	1.2	9.77E-02	1.2	1.97E-01	1.1	9.77E-02	1.2	2.83E-02	0.0	1.0E+00	0.0	1.0E+00	0.0	9.8E-01	0.2	1.0E+00	-0.1	9.3E-01	0.1	1.0E+00	-0.1	7.1E-01	-0.1	9.3E-01
20283	MAGEA6	A_23_P136870	NM_175868	1.3	1.64E-02	1.3	1.64E-02	3.7	2.27E-04	1.1	2.17E-02	0.6	5.33E-01	0.0	9.75E-01	2.1	5.33E-01	0.6	5.17E-01	-0.7	1.0E+00	-0.7	1.0E+00	0.0	9.9E-01	-0.8	1.0E+00	0.2	9.3E-01	0.0	1.0E+00	0.7	2.7E-01	0.2	9.3E-01
43873	FAM126A	A_23_P8582	NM_032581	1.3	3.11E-03	0.9	5.98E-03	1.3	3.11E-03	1.3	3.43E-05	1.0	2.86E-02	1.0	2.86E-02	0.7	8.94E-02	1.1	1.35E-02	0.0	1.0E+00	0.0	1.0E+00	-0.2	7.1E-01	0.0	1.0E+00	-0.3	6.2E-01	-0.1	1.0E+00	-0.3	2.5E-01	-0.3	6.2E-01
18873	HSD17B1	A_24_P56833	NM_000413	1.3	5.53E-03	1.3	7.64E-03	1.8	5.53E-03	1.2	8.93E-04	0.8	5.20E-02	0.6	1.77E-01	0.8	5.20E-02	0.9	1.53E-02	0.0	1.0E+00	0.0	1.0E+00	0.3	7.1E-01	0.0	1.0E+00	0.2	6.5E-01	0.3	1.0E+00	0.1	5.4E-01	0.2	6.5E-01
24914	SH2D2A	A_23_P160618	NM_003975	1.3	4.11E-03	1.3	4.11E-03	1.8	8.93E-03	1.2	3.88E-03	1.1	1.77E-01	0.7	5.12E-01	1.4	1.77E-01	1.1	1.07E-01	-0.2	1.0E+00	-0.2	1.0E+00	0.0	9.5E-01	-0.2	1.0E+00	0.4	4.1E-01	0.3	1.0E+00	0.4	2.6E-01	0.4	4.1E-01
20508	COL1A1	A_23_P207520	Z74615	1.3	5.24E-05	1.3	1.83E-03	2.9	5.24E-05	1.1	3.46E-05	1.3	1.32E-02	1.2	3.20E-02	1.5	1.32E-02	1.3	1.12E-02	0.1	1.0E+00	0.1	1.0E+00	0.0	9.6E-01	0.1	1.0E+00	-0.1	7.4E-01	0.0	1.0E+00	-0.1	6.8E-01	-0.2	7.4E-01
40391	PLA2G4C	A_23_P50508	NM_003706	1.3	3.44E-03	1.3	4.00E-04	1.3	3.72E-03	0.7	3.44E-03	0.5	6.40E-01	0.5	7.10E-01	0.4	6.40E-01	0.8	3.93E-01	0.1	1.0E+00	0.1	1.0E+00	0.1	9.3E-01	0.0	1.0E+00	0.0	9.8E-01	0.0	1.0E+00	0.3	4.9E-01	0.0	9.8E-01
29149	A_23_P72014	A_23_P72014	A_23_P72014	1.3	3.39E-03	1.3	3.79E-03	2.2	3.39E-03	1.2	2.39E-03	1.3	2.84E-02	1.1	3.28E-02	1.3	2.84E-02	1.3	4.53E-03	0.1	1.0E+00	0.1	1.0E+00	0.1	8.7E-01	0.1	1.0E+00	0.0	9.7E-01	0.1	1.0E+00	0.0	9.7E-01	0.0	9.7E-01
30493	LOC100131606	A_24_P375691	AF289566	1.3	1.82E-03	0.6	1.75E-02	1.4	1.82E-03	1.3	3.18E-05	0.5	2.36E-01	0.1	9.34E-01	0.5	2.36E-01	0.9	1.62E-02	0.0	1.0E+00	-0.1	1.0E+00	0.0	9.1E-01	0.1	1.0E+00	0.0	9.8E-01	0.1	1.0E+00	-0.1	8.3E-01	0.0	9.8E-01
15423	A_23_P15226	A_23_P15226	A_23_P15226	1.3	5.50E-04	1.0	5.03E-03	1.9	5.50E-04	1.3	2.35E-05	0.7	1.70E-02	0.7	3.49E-02	0.7	1.70E-02	0.8	3.61E-03	-0.1	1.0E+00	-0.1	1.0E+00	0.3	7.1E-01	-0.1	1.0E+00	0.0	9.6E-01	-0.1	1.0E+00	0.2	6.2E-01	0.0	9.6E-01
10637	ZSWIM3	A_23_P165984	NM_080752	1.3	2.06E-03	1.3	3.78E-03	1.4	2.06E-03	1.0	1.05E-04	1.0	1.80E-02	1.0	1.77E-02	0.9	1.80E-02	1.0	2.10E-02	0.1	1.0E+00	0.2	1.0E+00	0.1	7.7E-01	0.1	1.0E+00	0.0	9.8E-01	0.0	1.0E+00	0.0	9.7E-01	0.0	9.8E-01
3235	NR4A3	A_23_P398566	NM_173198	1.3	1.43E-03	1.2	1.25E-02	1.4	1.43E-03	1.3	4.73E-05	1.1	9.89E-02	1.3	9.89E-02	1.1	1.00E-01	1.1	3.03E-02	0.2	1.0E+00	0.2	1.0E+00	0.1	7.3E-01	0.4	1.0E+00	-0.2	8.3E-01	-0.1	1.0E+00	-0.2	4.3E-01	-0.2	8.3E-01
25935	MAD1L1	A_23_P333735	NM_003550	1.3	1.78E-03	1.0	1.10E-02	2.4	1.78E-03	1.3	5.92E-04	0.8	4.12E-02	0.6	1.88E-01	0.8	4.12E-02	1.0	4.29E-03	0.1	1.0E+00	0.1	1.0E+00	0.1	8.9E-01	0.1	1.0E+00	0.1	9.4E-01	0.0	1.0E+00	0.1	9.4E-01	0.2	9.1E-01
25965	SLIT1	A_23_P161386	NM_003061	1.3	1.03E-03	1.3	1.03E-03	2.0	1.88E-03	1.3	2.13E-04	0.9	6.00E-02	0.9	1.13E-01	1.3	6.00E-02	0.9	3.63E-02	0.2	1.0E+00	0.2	1.0E+00	0.3	7.1E-01	0.1	1.0E+00	0.2	8.2E-01	0.4	1.0E+00	0.2	7.1E-01	0.2	8.2E-01
17892	EGR4	A_23_P380318	NM_001965	1.3	3.27E-03	1.2	3.27E-03	1.4	1.20E-02	1.3	5.89E-04	1.3	1.71E-02	0.9	1.71E-02	1.3	2.95E-02	1.3	3.21E-03	0.3	1.0E+00	-0.1	1.0E+00	0.3	7.1E-01	0.4	1.0E+00	0.2	9.7E-01	0.2	1.0E+00	0.0	9.7E-01	0.2	8.8E-01
20966	CALHM2	A_23_P202219	NM_015916	1.3	2.67E-04	1.3	2.67E-04	1.6	1.88E-03	1.2	8.45E-05	1.3	2.71E-03	1.3	8.32E-03	1.3	2.71E-03	1.3	1.84E-03	0.3	7.1E-01	0.4	5.1E-01	0.3	7.1E-01	0.3	1.0E+00	0.1	9.0E-01	0.1	1.0E+00	0.1	9.0E-01	-0.2	8.8E-01
14701	RND1	A_23_P53370	NM_014470	1.3	1.17E-02	1.1	1.35E-02	1.3	1.17E-02	1.8	1.37E-04	0.6	6.16E-01	0.6	6.16E-01	0.1	9.14E-01	1.2	1.10E-01	0.4	1.0E+00	0.1	1.0E+00	0.4	7.1E-01	0.5	1.0E+00	-0.1	9.7E-01	-0.2	1.0E+00	0.0	9.7E-01	-0.1	9.2E-01
33196	CITED4	A_32_P209230	NM_133467	1.3	6.46E-04	1.3	6.46E-04	1.8	1.09E-03	1.1	1.33E-04	1.4	1.28E-03	1.0	8.15E-03	1.6	1.28E-03	1.4	1.64E-04	0.3	1.0E+00	0.2	1.0E+00	0.3	7.1E-01	0.3	1.0E+00	0.0	9.9E-01	0.0	1.0E+00	-0.1	8.7E-01	0.0	9.9E-01
22937	TRIM63	A_23_P114983	NM_032588	1.3	7.14E-03	1.3	2.73E-02	2.0	7.14E-03	1.3	1.74E-03	1.1	2.62E-02	0.9	4.12E-02	1.2	1.64E-02	1.1	2.62E-02	0.1	1.0E+00	0.1	1.0E+00	0.0	9.5E-01	0.6	1.0E+00	0.0	9.5E-01	0.0	1.0E+00	0.0	9.5E-01	-0.2	7.5E-01
16344	TP53I11	A_23_P368028	AF010315	1.3	2.54E-03	0.8	1.40E-02	1.6	2.54E-03	1.3	1.37E-04	0.9	4.21E-02	0.7	4.26E-02	0.9	4.21E-02	1.0	5.76E-03	0.2	1.0E+00	0.2	1.0E+00	0.2	7.1E-01	0.2	1.0E+00	0.1	9.2E-01	-0.1	1.0E+00	0.2	6.8E-01	0.1	9.2E-01
36406	APOC1	A_23_P4649	NM_001645	1.3	1.20E-02	1.0	4.05E-02	1.8	1.20E-02	1.3	2.34E-03	0.9	2.21E-02	0.9	3.01E-02	1.4	2.00E-02	0.9	2.21E-02	-0.1	1.0E+00	-0.1	1.0E+00	0.0	9.8E-01	-0.1	1.0E+00	0.0	9.3E-01	-0.1	1.0E+00	0.0	9.3E-01	0.1	9.0E-01
7683	WNT3	A_23_P130158	NM_030753	1.3	3.40E-03	0.7	2.14E-02	1.7	3.40E-03	1.3	2.17E-04	0.5	2.83E-01	0.0	9.80E-01	1.3	1.29E-01	0.5	2.83E-01	0.0	1.0E+00	0.2	1.0E+00	0.0	1.0E+00	0.0	1.0E+00	-0.3	8.1E-01	0.0	1.0E+00	-0.3	5.3E-01	-0.3	8.1E-01
34891	THC2698927	A_32_P86682	THC2698927	1.3	4.58E-04	1.1	4.58E-04	1.3	1.67E-03	1.4	1.46E-05	0.8	9.07E-02	0.3	7.59E-01	1.1	8.53E-02	0.8	9.07E-02	0.0	1.0E+00	0.0	1.0E+00	0.0	9.5E-01	0.2	1.0E+00	0.0	9.6E-01	0.0	1.0E+00	-0.1	8.8E-01	0.1	9.6E-01
25816	LAT	A_23_P44112	NM_014387	1.3	2.43E-03	1.0	2.43E-03	1.6	3.33E-03	1.3	1.30E-04	1.0	1.04E-02	1.1	1.04E-02	1.0	3.60E-02	0.9	1.02E-02	0.2	1.0E+00	0.3	1.0E+00	0.2	7.4E-01	0.1	1.0E+00	0.1	9.4E-01	0.1	1.0E+00	0.1	5.7E-01	0.1	9.4E-01
414	SULT1A2	A_23_P106773	NM_177528	1.3	1.09E-03	1.2	1.40E-03	2.2	1.09E-03	1.3	1.88E-04	1.1	4.52E-03	1.1	6.11E-03	1.9	4.52E-03	1.1	1.70E-03	0.1	1.0E+00	0.1	1.0E+00	0.1	8.5E-01	0.1	1.0E+00	0.3	3.9E-01	0.2	1.0E+00	0.3	2.8E-01	0.3	3.9E-01
17718	THC2611974	A_23_P139104	THC2611974	1.3	2.34E-03	1.3	2.34E-03	1.3	1.81E-02	1.2	1.44E-03	1.3	2.12E-02	1.3	2.12E-02	1.2	3.48E-02	1.3	5.82E-03	0.1	1.0E+00	0.1	1.0E+00	0.2	7.4E-01	0.1	1.0E+00	0.1	1.0E+00	0.2	1.0E+00	0.1	9.0E-01	0.0	1.0E+00
13654	KCNC1	A_23_P203439	NM_004976	1.3	2.31E-03	1.7	2.76E-02	1.3	2.31E-03	0.7	1.49E-03	1.2	6.57E-02	2.3	1.06E-03	1.0	6.57E-02	1.2	1.84E-01	0.2	7.6E-01	0.5	7.9E-02	-0.1	7.6E-01	0.2	1.0E+00	-0.2	7.6E-01	0.1	1.0E+00	-0.3	2.2E-01	-0.2	7.6E-01
38651	TNFRSF10B	A_23_P169030	NM_003842	1.3	3.34E-03	1.3	8.10E-03	1.7	3.34E-03	1.2	4.67E-04	1.0	4.96E-02	0.8	4.96E-02	1.0	5.54E-02	1.0	1.81E-02	0.0	1.0E+00	0.1	1.0E+00	-0.1	8.6E-01	0.0	1.0E+00	-0.2	3.4E-01	-0.2	1.0E+00	-0.2	3.4E-01	-0.3	2.7E-01
10095	GCHFR	A_23_P77328	NM_005258	1.3	9.43E-04	1.1	9.43E-04	1.7	1.83E-03	1.3	6.16E-05	1.1	2.49E-03	1.1	4.83E-03	1.1	2.49E-03	1.1	1.52E-03	0.2	1.0E+00	0.2	1.0E+00	0.2	7.1E-01	0.2	1.0E+00	0.0	1.0E+00	0.1	1.0E+00	0.0	1.0E+00	0.0	9.9E-01
7914	BAIAP3	A_23_P163492	NM_003933	1.3	1.48E-03	1.3	1.48E-02	1.5	1.48E-03	0.9	2.19E-04	1.2	4.33E-03	1.0	4.90E-02	1.2	4.33E-03	1.2	2.03E-03	0.2	1.0E+00	0.2	1.0E+00	0.1	8.8E-01	0.2	1.0E+00	-0.1	8.5E-01	0.1	1.0E+00	-0.1	6.5E-01	-0.1	8.5E-01
7323	GRB7	A_23_P163992	NM_005310	1.3	1.14E-02	1.4	1.14E-02	1.3	3.94E-02	1.0	7.17E-03	0.8	2.49E-01	0.8	2.49E-01	0.6	3.77E-01	1.0	7.53E-02	0.4	1.0E+00	0.3	1.0E+00	0.6	4.3E-01	0.4	1.0E+00	0.1	9.7E-01	-0.1	1.0E+00	0.1	9.7E-01	0.1	9.6E-01
36794	C1orf187	A_24_P68008	AK075558	1.3	1.73E-03	1.3	4.26E-03	2.1	1.73E-03	1.3	2.60E-04	1.0	7.15E-03	1.0	8.72E-03	1.5	7.15E-03	1.0	2.09E-03	0.2	1.0E+00	0.1	1.0E+00	0.2	7.1E-01	0.4	1.0E+00	-0.3	7.9E-01	-0.2	1.0E+00	-0.3	5.6E-01	-0.3	7.9E-01
3170	G0S2	A_23_P74609	NM_015714	1.3	1.22E-02	1.3	2.32E-02	1.5	1.22E-02	1.3	9.11E-04	0.9	7.50E-02	1.1	2.40E-02	0.6	1.17E-01	0.9	7.50E-02	0.3	1.0E+00	0.2	1.0E+00	0.3	7.1E-01	0.4	1.0E+00	0.0	9.8E-01	0.1	1.0E+00	0.0	9.8E-01	-0.1	9.3E-01
33457	BX100535	A_32_P107717	BX100535	1.3	9.38E-04	1.3	5.78E-03	1.5	9.38E-04	1.2	5.20E-05	1.0	6.52E-02	1.0	1.02E-01	1.3	2.17E-02	1.0	6.52E-02	0.0	1.0E+00	0.1	1.0E+00	0.0	9.2E-01	0.0	1.0E+00	-0.1	9.8E-01	-0.1	1.0E+00	-0.2	7.1E-01	0.0	9.8E-01
14611	AKR1B10	A_24_P129341	NM_020299	1.3	6.67E-04	0.9	6.67E-04	1.4	8.47E-04	1.3	7.66E-06	0.9	3.07E-02	0.5	1.45E-01	0.9	3.07E-02	1.0	3.61E-03	0.0	1.0E+00	0.0	1.0E+00	-0.1	7.1E-01	0.1	1.0E+00	-0.1	9.3E-01	-0.1	1.0E+00	0.0	9.2E-01	-0.1	9.3E-01
7407	MEI1	A_23_P211561	NM_152513	1.3	3.52E-04	1.0	3.18E-03	2.0	3.52E-04	1.3	1.62E-05	0.9	5.01E-02	0.8	1.23E-01	1.7	2.06E-02	0.9	5.01E-02	0.2	1.0E+00	0.2	1.0E+00	0.0	9.8E-01	0.2	1.0E+00	-0.4	7.1E-01	-0.1	1.0E+00	-0.4	4.8E-01	-0.4	7.1E-01
20865	NAPSA	A_23_P90130	NM_004851	1.3	2.41E-03	1.3	6.31E-04	1.6	2.41E-03	1.1	5.84E-03	0.8	1.02E-01	0.8	1.02E-01	1.2	2.31E-01	0.8	8.62E-02	-0.2	1.0E+00	-0.2	1.0E+00	-0.1	9.4E-01	-0.2	1.0E+00	0.1	9.3E-01	0.0	1.0E+00	0.5	1.9E-01	0.1	9.3E-01
2982	ASAP3	A_23_P114689	NM_017707	1.3	8.91E-04	1.3	1.37E-03	1.8	8.91E-04	1.0	1.24E-04	1.3	2.51E-03	1.3	2.51E-03	1.6	3.90E-03	1.2	5.27E-04	0.2	1.0E+00	0.2	1.0E+00	0.1	9.2E-01	0.2	1.0E+00	-0.2	8.6E-01	-0.1	1.0E+00	-0.2	6.0E-01	-0.2	8.6E-01
38991	MTMR11	A_23_P51986	NM_181873	1.3	5.29E-03	1.0	1.22E-02	1.7	5.29E-03	1.3	4.66E-04	0.9	9.86E-03	0.9	3.02E-02	1.2	3.50E-03	0.9	9.86E-03	0.0	1.0E+00	0.0	1.0E+00	0.0	1.0E+00	0.2	1.0E+00	-0.5	4.8E-01	-0.3	1.0E+00	-0.5	2.5E-01	-0.5	4.8E-01
10104	A_32_P153361	A_32_P153361	A_32_P153361	1.3	1.50E-03	1.1	1.50E-03	1.5	4.00E-03	1.3	1.14E-04	0.6	5.10E-01	0.5	7.11E-01	0.6	5.10E-01	0.8	2.68E-01	-0.1	1.0E+00	-0.1	1.0E+00	0.3	7.1E-01	-0.1	1.0E+00	0.2	6.8E-01	0.0	1.0E+00	0.3	2.4E-01	0.2	6.8E-01
25548	DNAJA4	A_23_P129279	NM_018602	1.3	6.95E-04	1.3	6.95E-04	1.2	6.31E-03	1.5	2.33E-05	1.2	2.67E-02	1.2	5.75E-02	1.2	2.67E-02	1.2	1.62E-02	0.0	1.0E+00	0.0	1.0E+00	0.0	9.4E-01	0.2	1.0E+00	0.1	1.0E+00	0.1	1.0E+00	0.2	8.6E-01	0.0	1.0E+00
17282	FZD10	A_23_P203972	NM_007197	1.3	3.63E-03	1.3	4.95E-03	1.5	3.63E-03	1.2	1.38E-04	1.0	5.32E-02	0.8	7.32E-02	1.2	2.77E-02	1.0	5.32E-02	0.0	1.0E+00	-0.2	1.0E+00	0.0	9.8E-01	0.1	1.0E+00	-0.1	7.2E-01	-0.1	1.0E+00	-0.1	7.2E-01	-0.3	5.8E-01
25052	BC030768	A_24_P213827	BC030768	1.3	2.37E-04	1.0	2.37E-04	1.4	1.55E-03	1.3	1.90E-04	1.2	6.82E-02	1.2	1.35E-01	1.2	6.82E-02	1.2	5.53E-02	0.1	1.0E+00	0.2	1.0E+00	-0.3	7.1E-01	0.1	1.0E+00	-0.2	7.2E-01	0.1	1.0E+00	-0.2	4.5E-01	-0.2	7.2E-01
16009	STAT6	A_23_P47879	NM_003153	1.3	3.97E-03	0.9	6.20E-03	1.8	3.97E-03	1.3	2.77E-04	0.9	5.93E-03	0.9	5.93E-03	1.0	5.94E-03	0.7	4.46E-03	0.1	1.0E+00	0.1	1.0E+00	0.0	9.6E-01	0.1	1.0E+00	-0.2	9.3E-01	-0.2	1.0E+00	-0.1	9.3E-01	-0.2	9.1E-01
35013	A_24_P178167	A_24_P178167	A_24_P178167	1.3	5.03E-03	1.3	4.52E-03	1.5	2.89E-02	1.1	5.03E-03	0.5	1.59E-01	0.5	1.59E-01	0.5	2.60E-01	1.1	4.48E-03	0.1	1.0E+00	0.3	1.0E+00	0.1	9.1E-01	0.0	1.0E+00	-0.1	9.4E-01	0.1	1.0E+00	-0.1	9.4E-01	-0.1	9.3E-01
41358	SLC1A7	A_23_P325562	NM_006671	1.3	1.73E-03	1.2	1.73E-03	1.3	5.50E-03	1.4	6.68E-05	0.4	7.15E-01	-0.4	8.11E-01	0.5	6.36E-01	0.4	7.15E-01	0.0	1.0E+00	0.0	1.0E+00	0.4	8.0E-01	0.0	1.0E+00	0.2	7.7E-01	0.1	1.0E+00	0.2	7.7E-01	0.4	6.3E-01
17388	KCNC3	A_23_P101516	NM_004977	1.3	2.01E-02	1.3	2.22E-02	1.4	6.77E-03	0.6	2.01E-02	0.8	2.27E-01	1.2	4.88E-02	0.8	4.24E-01	0.7	2.27E-01	0.0	1.0E+00	0.0	1.0E+00	0.5	7.1E-01	-0.2	1.0E+00	0.3	8.5E-01	0.3	1.0E+00	0.5	2.5E-02	0.2	8.5E-01
21614	NOS2	A_23_P502464	NM_000625	1.3	9.57E-03	1.3	9.57E-03	1.2	1.94E-02	1.4	2.39E-04	1.3	1.67E-02	1.3	2.39E-02	1.2	1.67E-02	1.5	9.43E-03	0.1	1.0E+00	0.1	1.0E+00	-0.1	8.7E-01	0.1	1.0E+00	0.0	9.7E-01	0.0	1.0E+00	0.0	9.7E-01	0.0	9.3E-01
24047	TNFRSF6B	A_23_P218646	NM_032945	1.3	1.87E-02	1.3	1.87E-02	1.6	3.72E-02	1.1	1.49E-02	1.0	3.57E-02	1.1	7.96E-03	0.6	6.94E-02	1.0	3.57E-02	0.3	1.0E+00	0.3	1.0E+00	0.3	7.1E-01	0.0	1.0E+00	0.1	9.8E-01	0.2	1.0E+00	0.1	9.3E-01	0.0	9.8E-01
10093	PTPRN2	A_32_P79434	NM_002847	1.3	4.46E-02	0.9	4.46E-02	1.3	4.95E-02	1.5	2.07E-03	1.2	3.81E-03	1.4	2.72E-03	1.1	8.48E-03	1.2	3.81E-03	0.0	1.0E+00	-0.1	1.0E+00	0.0	9.8E-01	0.0	1.0E+00	-0.1	9.8E-01	-0.3	1.0E+00	-0.1	9.3E-01	0.0	9.8E-01
35151	SDR16C5	A_23_P257457	NM_138969	1.3	6.52E-04	1.3	6.52E-04	0.9	3.22E-02	1.7	3.96E-05	1.3	7.87E-03	1.0	2.08E-02	1.3	7.87E-03	1.6	1.16E-03	0.1	1.0E+00	0.1	1.0E+00	0.1	8.1E-01	0.2	1.0E+00	-0.2	3.4E-01	0.1	1.0E+00	-0.2	1.5E-01	-0.2	3.4E-01
32694	OTUD1	A_32_P60459	AB188491	1.3	1.25E-03	1.3	5.76E-03	1.6	1.25E-03	1.2	1.28E-04	0.8	7.64E-02	1.0	1.51E-02	0.7	8.84E-02	0.8	7.64E-02	0.1	1.0E+00	0.1	1.0E+00	-0.2	8.1E-01	0.3	1.0E+00	-0.3	8.5E-01	-0.1	1.0E+00	-0.4	6.2E-01	-0.3	8.5E-01
12588	PTGER1	A_23_P4808	NM_000955	1.3	7.11E-05	1.3	2.52E-03	2.3	7.11E-05	1.2	9.96E-06	1.0	1.10E-02	1.0	1.83E-02	1.0	1.10E-02	1.0	1.94E-03	0.1	1.0E+00	0.1	1.0E+00	0.2	7.1E-01	0.0	1.0E+00	0.1	8.6E-01	-0.1	1.0E+00	0.1	8.6E-01	0.2	5.1E-01
21998	MR1	A_23_P431939	NM_001531	1.3	1.24E-03	0.9	1.30E-02	1.8	1.24E-03	1.3	5.10E-05	1.0	3.33E-02	0.9	8.16E-02	1.0	3.33E-02	1.0	1.75E-02	0.1	1.0E+00	0.1	1.0E+00	-0.1	7.9E-01	0.1	1.0E+00	-0.3	8.3E-01	-0.2	1.0E+00	-0.4	5.1E-01	-0.3	8.3E-01
42062	GDF11	A_23_P76102	NM_005811	1.3	5.36E-03	1.3	5.36E-03	1.5	9.31E-03	1.3	4.95E-04	1.0	1.68E-02	0.9	1.68E-02	1.0	2.37E-02	1.2	5.86E-04	0.1	1.0E+00	0.2	1.0E+00	0.0	9.8E-01	0.1	1.0E+00	-0.2	7.0E-01	0.0	1.0E+00	-0.2	4.8E-01	-0.2	7.0E-01
38874	THC2683231	A_32_P101653	THC2683231	1.3	3.58E-04	1.3	9.86E-03	2.2	2.59E-04	0.9	3.58E-04	1.5	4.63E-03	1.5	2.49E-03	1.8	4.63E-03	1.3	1.38E-02	0.0	1.0E+00	0.0	1.0E+00	0.0	9.8E-01	0.1	1.0E+00	-0.2	6.3E-01	-0.1	1.0E+00	-0.4	1.3E-01	-0.2	6.3E-01
32987	LMO2	A_23_P53126	NM_005574	1.3	3.76E-03	1.3	3.76E-03	1.4	3.29E-02	1.3	2.85E-03	1.0	9.27E-03	0.9	9.27E-03	1.0	2.15E-03	1.0	1.15E-02	-0.1	1.0E+00	-0.1	1.0E+00	-0.1	8.6E-01	-0.2	1.0E+00	-0.3	8.5E-01	-0.2	1.0E+00	-0.3	5.6E-01	-0.3	8.5E-01
4858	CD33	A_24_P301655	NM_001772	1.3	6.95E-03	1.3	6.95E-03	1.2	8.42E-03	1.6	2.92E-05	0.8	3.71E-02	0.8	3.71E-02	0.7	2.58E-01	1.2	8.00E-04	0.1	1.0E+00	0.1	1.0E+00	-0.1	7.7E-01	0.1	1.0E+00	-0.1	8.2E-01	-0.1	1.0E+00	-0.1	5.9E-01	-0.1	8.2E-01
33024	TMEM102	A_23_P334892	NM_178518	1.3	1.34E-03	1.3	1.34E-03	1.3	6.80E-03	0.9	1.20E-03	1.2	5.71E-03	1.0	3.20E-02	1.2	5.71E-03	1.4	1.14E-03	0.1	1.0E+00	0.1	1.0E+00	0.2	7.3E-01	0.1	1.0E+00	0.2	9.4E-01	0.2	1.0E+00	-0.1	9.4E-01	0.2	9.3E-01
39755	C13orf29	A_23_P368909	BC093904	1.3	2.78E-03	1.3	5.36E-03	2.5	2.30E-03	1.3	2.78E-03	0.7	5.62E-01	0.1	9.57E-01	1.0	4.49E-01	0.7	5.62E-01	0.2	1.0E+00	0.2	1.0E+00	0.7	7.2E-01	0.0	1.0E+00	0.2	8.4E-01	-0.2	1.0E+00	0.2	8.4E-01	0.4	6.9E-01
20273	CCDC155	A_23_P431284	NM_144688	1.3	1.95E-03	1.2	3.65E-03	1.3	1.95E-03	1.6	9.25E-06	1.0	7.79E-03	1.0	1.69E-02	1.0	5.28E-03	1.1	7.79E-03	0.0	1.0E+00	0.1	1.0E+00	0.0	9.3E-01	0.0	1.0E+00	-0.1	8.6E-01	-0.1	1.0E+00	-0.1	8.0E-01	-0.1	8.6E-01
747	AGMAT	A_23_P103720	NM_024758	1.3	2.83E-03	1.3	2.83E-03	1.5	1.56E-02	1.3	9.80E-04	1.0	5.59E-03	1.0	5.76E-03	1.1	5.59E-03	0.9	4.74E-03	0.1	1.0E+00	0.1	1.0E+00	0.0	9.9E-01	0.1	1.0E+00	0.1	9.7E-01	0.2	1.0E+00	0.1	9.2E-01	0.1	9.7E-01
7677	A_23_P64962	A_23_P64962	A_23_P64962	1.3	3.52E-04	1.3	1.74E-03	1.9	3.52E-04	1.2	4.57E-05	1.1	9.53E-03	1.1	3.59E-02	1.2	9.53E-03	1.1	6.53E-03	0.3	1.0E+00	0.3	1.0E+00	0.1	7.6E-01	0.4	1.0E+00	-0.2	8.9E-01	-0.1	1.0E+00	-0.2	6.3E-01	-0.2	8.9E-01

Feature	Gene symbol	Probe	Transcript	72 hour								48 hours								24 hours								10 hours							
				siCASP8AP2								siCASP8AP2								siCASP8AP2								siCASP8AP2							
				Median	Median	1	1	3	3	6	6	Median	Median	1	1	3	3	6	6	Median	Median	1	1	3	3	6	6	Median	Median	1	1	3	3	6	6
				M	q	M	q	M	q	M	q	M	q	M	q	M	q	M	q	M	q	M	q	M	q	M	q	M	q	M	q	M	q	M	q
21612	GPR68	A_24_P931443	NM_003485	1.3	3.30E-04	1.3	1.52E-03	1.4	3.30E-04	1.3	1.22E-06	1.3	1.48E-03	1.4	1.48E-03	1.3	2.10E-03	1.2	2.38E-04	0.1	1.0E+00	0.1	1.0E+00	0.1	8.7E-01	0.3	1.0E+00	-0.1	9.1E-01	-0.1	1.0E+00	0.0	8.3E-01	-0.1	9.1E-01
22224	COL1A1	A_23_P207520	Z74615	1.3	8.10E-05	1.3	1.39E-03	3.0	8.10E-05	1.3	1.81E-05	1.3	1.59E-02	1.2	3.95E-02	1.6	1.59E-02	1.3	9.60E-03	0.1	1.0E+00	0.1	1.0E+00	0.1	8.8E-01	0.2	1.0E+00	-0.2	7.3E-01	-0.1	1.0E+00	-0.2	4.7E-01	-0.2	7.3E-01
33343	FILIP1	A_23_P436369	NM_015687	1.3	1.82E-03	0.9	1.82E-03	1.5	2.04E-03	1.3	4.20E-05	0.6	1.80E-01	0.3	7.08E-01	0.8	1.70E-01	0.6	1.80E-01	0.0	1.0E+00	0.0	1.0E+00	0.0	8.6E-01	0.0	1.0E+00	-0.1	9.0E-01	-0.1	1.0E+00	-0.1	7.3E-01	-0.1	9.0E-01
31512	COL1A1	A_23_P207520	Z74615	1.3	1.39E-04	1.3	3.77E-03	2.5	1.39E-04	1.1	3.78E-05	1.4	1.41E-02	1.2	3.10E-02	1.5	1.41E-02	1.4	6.36E-03	0.1	1.0E+00	0.1	1.0E+00	0.1	8.2E-01	0.2	1.0E+00	-0.2	6.7E-01	-0.1	1.0E+00	-0.2	3.0E-01	-0.2	6.7E-01
13940	HYAL1	A_23_P69329	NM_007312	1.3	1.28E-03	1.3	1.46E-03	1.5	1.28E-03	1.3	3.18E-05	1.1	7.76E-03	1.1	7.76E-03	0.8	3.63E-02	1.4	1.55E-03	0.3	7.1E-01	0.3	7.7E-01	0.2	7.1E-01	0.4	5.2E-01	-0.1	8.6E-01	0.0	1.0E+00	-0.3	4.1E-01	-0.1	8.6E-01
27887	SLC26A4	A_23_P331560	NM_000441	1.3	2.61E-03	1.5	2.61E-03	1.3	2.85E-03	1.2	6.44E-05	1.3	6.17E-03	1.6	7.83E-03	1.1	6.17E-03	1.3	1.19E-03	0.1	1.0E+00	0.1	1.0E+00	0.0	9.6E-01	0.1	1.0E+00	-0.3	4.3E-01	0.0	1.0E+00	-0.3	4.1E-01	-0.3	4.3E-01
24806	NOS2	A_23_P502464	NM_000625	1.3	5.64E-03	1.3	5.64E-03	1.1	2.26E-02	1.3	2.42E-04	1.2	1.27E-02	1.2	1.27E-02	1.0	1.37E-02	1.3	6.19E-03	0.0	1.0E+00	0.0	1.0E+00	0.0	9.7E-01	0.1	1.0E+00	-0.1	7.3E-01	0.0	1.0E+00	-0.1	5.6E-01	-0.1	7.3E-01
34666	RRAS	A_23_P39076	NM_006270	1.3	4.44E-04	1.3	4.44E-04	1.8	1.10E-03	1.3	4.45E-05	1.0	1.12E-02	0.9	1.12E-02	1.0	1.73E-02	1.3	9.61E-04	0.3	1.0E+00	0.3	1.0E+00	0.3	7.1E-01	0.1	1.0E+00	0.0	9.6E-01	0.0	1.0E+00	0.0	9.6E-01	-0.1	9.6E-01
21869	NOS2	A_23_P502464	NM_000625	1.3	4.48E-03	1.3	4.48E-03	1.1	1.65E-02	1.4	9.68E-05	1.3	3.55E-02	1.3	3.55E-02	1.1	3.73E-02	1.4	1.44E-02	0.1	1.0E+00	0.1	1.0E+00	0.0	9.6E-01	0.1	1.0E+00	-0.1	8.3E-01	-0.1	1.0E+00	0.0	8.3E-01	-0.1	6.8E-01
20382	FES	A_23_P14769	NM_002005	1.3	1.97E-03	1.0	1.97E-03	1.3	3.78E-03	1.3	4.07E-05	1.3	3.81E-02	1.3	3.81E-02	1.1	4.63E-02	1.3	1.74E-02	0.2	1.0E+00	0.2	1.0E+00	0.1	8.8E-01	0.5	1.0E+00	-0.1	9.4E-01	0.0	1.0E+00	-0.2	6.3E-01	-0.1	9.4E-01
23724	CACNG7	A_24_P365349	ENST00000391767	1.3	1.04E-03	1.3	2.88E-03	1.4	1.04E-03	1.3	1.67E-05	0.9	1.09E-01	1.0	1.23E-01	0.9	1.02E-01	0.8	1.09E-01	0.0	1.0E+00	0.1	1.0E+00	0.0	9.3E-01	-0.1	1.0E+00	0.0	9.9E-01	-0.1	1.0E+00	0.0	9.0E-01	0.0	9.9E-01
10165	DACT2	A_24_P289260	NM_214462	1.3	8.03E-04	1.4	8.03E-04	1.2	1.01E-02	1.3	4.55E-04	1.5	2.69E-03	1.3	7.83E-03	1.5	2.69E-03	1.6	1.16E-03	0.1	1.0E+00	0.1	1.0E+00	-0.1	8.7E-01	0.1	1.0E+00	-0.3	6.6E-01	-0.2	1.0E+00	-0.3	2.8E-01	-0.3	6.6E-01
32301	KRTAP11-1	A_32_P481377	NM_175858	1.3	8.92E-03	1.3	3.65E-03	1.4	8.92E-03	1.1	1.04E-02	0.4	8.34E-01	-0.1	9.77E-01	0.6	7.33E-01	0.4	8.34E-01	-0.1	1.0E+00	-0.1	1.0E+00	0.5	7.5E-01	-0.3	1.0E+00	0.9	2.6E-01	0.1	1.0E+00	0.9	2.6E-01	1.2	7.1E-02
28826	RASAL3	A_23_P79069	NM_022904	1.3	1.03E-03	1.3	5.97E-03	1.8	1.03E-03	1.3	3.31E-05	0.9	2.24E-02	0.7	4.91E-02	1.0	1.72E-02	0.9	2.24E-02	0.0	1.0E+00	0.0	1.0E+00	0.1	7.3E-01	0.0	1.0E+00	0.0	9.7E-01	0.0	1.0E+00	0.0	9.7E-01	0.0	9.7E-01
19639	SCGB2A1	A_23_P312300	NM_002407	1.3	1.21E-03	1.0	1.06E-02	1.4	1.21E-03	1.3	1.33E-05	0.8	9.99E-03	0.6	5.26E-02	0.8	9.99E-03	1.0	2.94E-03	0.1	1.0E+00	0.1	1.0E+00	0.0	1.0E+00	0.1	1.0E+00	-0.1	8.9E-01	0.0	1.0E+00	-0.2	4.1E-01	-0.1	8.9E-01
28700	CCDC70	A_23_P2857	NM_031290	1.3	2.52E-05	1.2	2.52E-05	2.3	4.12E-05	1.3	1.73E-05	1.2	1.53E-03	0.9	3.20E-02	1.6	4.44E-04	1.2	1.53E-03	0.1	1.0E+00	0.1	1.0E+00	0.0	9.3E-01	0.1	1.0E+00	0.0	9.4E-01	0.0	1.0E+00	0.0	8.3E-01	0.0	9.4E-01
30042	NOS2	A_23_P502464	NM_000625	1.3	3.92E-03	1.3	3.92E-03	1.0	2.23E-02	1.4	9.82E-05	1.2	4.82E-02	1.2	4.82E-02	1.0	5.80E-02	1.4	1.52E-02	0.0	1.0E+00	0.0	1.0E+00	0.0	9.5E-01	0.0	1.0E+00	0.0	9.8E-01	0.0	1.0E+00	0.0	9.6E-01	0.0	9.8E-01
23859	ELOVL3	A_23_P149858	NM_152310	1.3	1.05E-03	1.3	1.05E-03	1.3	3.79E-03	1.4	3.28E-05	0.6	6.83E-02	0.6	6.83E-02	0.5	1.20E-01	1.1	2.27E-02	0.1	1.0E+00	0.0	1.0E+00	0.1	8.6E-01	0.1	1.0E+00	0.0	9.6E-01	0.1	1.0E+00	0.0	9.6E-01	0.0	9.4E-01
37262	IFI27	A_23_P48513	NM_005532	1.3	1.34E-03	1.1	1.09E-02	2.7	1.34E-03	1.3	5.47E-04	1.4	1.76E-02	1.0	2.51E-02	1.9	1.76E-02	1.4	1.18E-03	0.0	1.0E+00	-0.1	1.0E+00	0.0	9.9E-01	0.0	1.0E+00	0.0	9.8E-01	0.1	1.0E+00	-0.1	7.6E-01	0.0	9.8E-01
39259	DENND2A	A_23_P257583	NM_015689	1.3	1.48E-03	1.3	1.48E-03	1.4	1.49E-03	1.1	4.26E-05	0.3	8.15E-01	0.3	8.15E-01	0.2	8.55E-01	0.5	6.12E-01	0.0	1.0E+00	-0.1	1.0E+00	0.1	8.5E-01	0.0	1.0E+00	0.1	9.0E-01	0.1	1.0E+00	0.2	5.6E-01	0.1	9.0E-01
13547	APOC1	A_24_P109214	NM_001645	1.3	1.88E-02	1.0	4.34E-02	1.8	1.88E-02	1.3	3.39E-03	1.1	2.27E-02	1.1	2.27E-02	1.5	2.62E-02	1.0	9.75E-03	-0.1	1.0E+00	-0.1	1.0E+00	-0.1	8.8E-01	0.0	1.0E+00	-0.1	9.5E-01	-0.1	1.0E+00	-0.1	9.0E-01	-0.1	9.5E-01
18008	ICAM2	A_23_P152655	NM_000873	1.3	2.69E-03	0.8	3.27E-02	2.1	2.69E-03	1.3	3.72E-04	0.5	2.80E-01	0.5	2.80E-01	0.9	2.49E-01	0.5	2.90E-01	-0.1	1.0E+00	0.1	1.0E+00	-0.1	8.6E-01	-0.1	1.0E+00	0.0	9.8E-01	0.2	1.0E+00	0.0	9.8E-01	-0.1	9.5E-01
245	SHOX2	A_23_P124384	NM_006884	1.3	3.18E-04	1.1	3.09E-03	2.1	3.18E-04	1.3	9.17E-05	1.0	9.07E-02	0.6	9.07E-02	1.6	1.23E-01	1.0	5.25E-03	0.1	1.0E+00	0.1	1.0E+00	0.1	8.6E-01	0.0	1.0E+00	0.0	9.7E-01	0.2	1.0E+00	-0.1	8.9E-01	0.0	9.7E-01
35944	CSAG2	A_24_P79529	NM_001080848	1.3	2.03E-04	1.1	2.03E-04	1.3	4.52E-04	1.4	1.00E-06	0.3	6.04E-01	0.0	9.62E-01	0.3	6.04E-01	0.7	1.82E-01	0.0	1.0E+00	0.0	1.0E+00	0.1	8.8E-01	0.0	1.0E+00	0.0	9.6E-01	0.0	1.0E+00	0.1	7.9E-01	0.0	9.6E-01
4880	DNASE1L2	A_23_P3643	NM_001374	1.4	2.59E-03	1.4	9.19E-03	1.4	2.23E-03	0.8	2.59E-03	0.9	4.69E-02	0.9	4.69E-02	0.7	6.63E-02	1.0	2.50E-02	0.0	1.0E+00	0.0	1.0E+00	0.0	9.3E-01	0.0	1.0E+00	0.0	1.0E+00	0.1	1.0E+00	0.0	1.0E+00	0.0	1.0E+00
19122	TNFSF9	A_23_P67224	NM_003811	1.4	6.62E-03	1.3	6.62E-03	1.4	4.22E-02	1.5	1.19E-03	0.9	7.07E-02	0.9	7.07E-02	0.7	1.94E-01	1.4	4.08E-03	0.1	1.0E+00	0.1	1.0E+00	0.1	8.5E-01	0.1	1.0E+00	-0.1	9.6E-01	-0.2	1.0E+00	-0.1	9.1E-01	-0.1	9.6E-01
20399	A_32_P224040	A_32_P224040	A_32_P224040	1.4	1.67E-04	1.4	3.93E-03	2.0	1.67E-04	1.1	8.22E-06	1.2	2.01E-02	1.1	2.01E-02	1.7	4.77E-02	1.2	7.25E-03	0.0	1.0E+00	0.0	1.0E+00	-0.1	8.4E-01	0.2	1.0E+00	-0.3	6.5E-01	0.1	1.0E+00	-0.4	2.0E-01	-0.3	6.5E-01
2488	KREMEN2	A_23_P77612	NM_172229	1.4	8.21E-03	1.4	3.01E-03	1.4	2.30E-02	0.9	8.21E-03	1.2	1.83E-03	1.2	1.83E-03	1.3	1.09E-03	1.2	2.31E-03	0.4	8.4E-01	0.3	8.4E-01	0.4	7.1E-01	0.5	1.0E+00	0.0	9.8E-01	0.2	1.0E+00	0.0	9.6E-01	0.0	9.8E-01
37742	TNFRSF8	A_23_P500614	NM_001243	1.4	6.63E-04	1.4	6.63E-04	1.2	7.35E-03	1.4	9.79E-05	1.2	6.29E-02	1.0	2.25E-01	1.4	6.07E-02	1.2	6.29E-02	0.1	1.0E+00	0.1	1.0E+00	0.2	7.3E-01	0.1	1.0E+00	0.2	9.1E-01	0.0	1.0E+00	0.2	6.6E-01	0.2	9.1E-01
40243	NLRC4	A_23_P119835	NM_021209	1.4	3.52E-04	1.2	3.93E-04	1.7	3.52E-04	1.4	5.45E-06	0.9	3.54E-03	0.6	1.07E-01	1.3	3.01E-03	0.9	3.54E-03	0.1	1.0E+00	0.1	1.0E+00	0.1	7.1E-01	-0.1	1.0E+00	0.0	9.7E-01	-0.2	1.0E+00	0.0	9.3E-01	0.0	9.7E-01
33730	APOA4	A_23_P87036	NM_000482	1.4	4.67E-04	1.4	6.43E-03	1.4	4.67E-04	1.3	6.48E-06	0.4	4.65E-01	0.2	8.16E-01	0.4	4.65E-01	0.5	2.71E-01	0.0	1.0E+00	0.0	1.0E+00	0.0	9.1E-01	0.0	1.0E+00	0.1	5.2E-01	0.0	1.0E+00	0.1	5.2E-01	0.2	4.6E-01
34973	NPC1	A_23_P107587	NM_000271	1.4	2.96E-03	1.4	2.96E-03	0.9	3.37E-02	1.6	4.52E-05	1.2	7.07E-02	1.1	7.07E-02	1.2	2.64E-01	1.7	1.43E-03	0.2	1.0E+00	0.2	1.0E+00	0.0	9.5E-01	0.3	1.0E+00	0.0	9.7E-01	0.0	1.0E+00	-0.1	8.3E-01	0.1	9.7E-01
26970	ROBO3	A_23_P356581	NM_022370	1.4	2.45E-03	1.0	1.02E-02	2.1	2.45E-03	1.4	2.47E-04	0.9	4.81E-02	0.8	4.81E-02	1.1	6.44E-02	0.9	9.77E-03	0.0	1.0E+00	0.1	1.0E+00	-0.2	7.2E-01	0.0	1.0E+00	-0.2	9.0E-01	-0.1	1.0E+00	-0.2	8.0E-01	-0.2	9.0E-01
40276	SETX	A_24_P940921	NM_015046	1.4	1.63E-03	1.2	1.63E-03	1.9	2.69E-03	1.4	2.77E-04	1.2	3.01E-03	1.2	6.77E-03	1.2	3.01E-03	1.3	7.09E-04	0.3	1.0E+00	0.3	1.0E+00	0.3	7.1E-01	0.4	1.0E+00	-0.1	9.0E-01	-0.1	1.0E+00	0.0	9.0E-01	-0.4	4.5E-01
13210	CCDC80	A_23_P58082	NM_199511	1.4	3.12E-04	1.1	7.51E-04	1.9	3.12E-04	1.4	2.66E-05	1.3	2.68E-02	1.4	4.26E-02	1.0	2.68E-02	1.3	5.50E-03	0.1	1.0E+00	0.1	1.0E+00	-0.3	7.1E-01	0.1	1.0E+00	-0.4	6.2E-01	-0.1	1.0E+00	-0.4	3.7E-01	-0.4	6.2E-01
7594	AK124399	A_32_P54160	AK124399	1.4	3.66E-04	1.4	3.66E-04	1.3	3.82E-03	1.5	1.81E-05	1.3	2.03E-02	1.0	1.10E-01	1.7	2.03E-02	1.3	1.00E-02	0.4	1.0E+00	0.4	1.0E+00	0.3	7.1E-01	0.5	1.0E+00	-0.3	5.3E-01	-0.2	1.0E+00	-0.4	1.7E-01	-0.3	5.3E-01
29989	RUNDC3A	A_24_P91094	NM_006695	1.4	1.95E-03	1.9	1.95E-03	1.4	4.86E-03	1.1	2.38E-04	1.5	5.79E-02	1.5	5.79E-02	0.5	4.98E-01	1.6	3.17E-02	0.2	1.0E+00	0.2	1.0E+00	0.2	7.2E-01	0.1	1.0E+00	0.1	7.3E-01	0.1	1.0E+00	0.1	7.3E-01	0.2	6.2E-01
9543	CCL3	A_23_P373017	D00044	1.4	5.01E-04	0.9	5.01E-04	1.5	8.37E-04	1.4	7.80E-06	0.8	2.78E-01	0.8	2.78E-01	0.7	3.11E-01	1.0	5.18E-02	0.4	1.0E+00	0.2	1.0E+00	0.4	4.7E-01	0.4	1.0E+00	-0.2	7.0E-01	0.0	1.0E+00	-0.2	4.6E-01	-0.2	7.0E-01
19033	C1orf211	A_23_P343261	BC030279	1.4	2.02E-04	1.3	2.02E-04	1.6	6.96E-04	1.4	6.68E-06	1.2	1.83E-02	1.2	2.85E-02	1.2	1.83E-02	1.1	6.63E-03	0.1	1.0E+00	0.1	1.0E+00	-0.2	7.5E-01	0.2	1.0E+00	-0.1	9.2E-01	0.1	1.0E+00	-0.3	5.9E-01	-0.1	9.2E-01
4345	NOS3	A_23_P70849	NM_000603	1.4	2.30E-03	1.3	2.11E-02	2.6	2.30E-03	1.4	9.11E-04	0.6	5.70E-01	0.2	8.72E-01	0.6	5.70E-01	0.7	3.82E-01	0.0	1.0E+00	-0.1	1.0E+00	0.6	7.1E-01	0.0	1.0E+00	0.5	3.5E-01	0.0	1.0E+00	0.6	8.9E-02	0.5	3.5E-01
31050	TFEB	A_23_P368729	NM_007162	1.4	9.95E-04	1.4	1.40E-03	2.4	9.95E-04	1.2	4.37E-04	1.2	2.77E-02	0.8	1.51E-01	1.7	2.77E-02	1.2	1.14E-02	0.3	1.0E+00	0.2	1.0E+00	0.4	7.1E-01	0.3	1.0E+00	0.1	9.5E-01	0.1	1.0E+00	0.0	9.5E-01	0.2	7.5E-01
24415	BMF	A_23_P379649	NM_001003940	1.4	2.09E-02	1.4	2.09E-02	1.8	2.38E-02	1.0	1.69E-02	1.4	2.55E-02	1.3	5.92E-02	1.6	1.55E-02	1.4	2.55E-02	0.2	1.0E+00	0.2	1.0E+00	0.2	7.5E-01	0.3	1.0E+00	-0.3	9.1E-01	-0.2	1.0E+00	-0.4	6.4E-01	-0.3	9.1E-01
11388	OR7E91P	A_24_P332718	NR_002185	1.4	5.45E-04	1.1	5.45E-04	1.8	5.47E-04	1.4	6.82E-06	0.8	1.53E-01	0.6	3.31E-01	0.8	1.53E-01	1.2	1.70E-02	0.1	1.0E+00	0.0	1.0E+00	0.2	7.1E-01	0.1	1.0E+00	0.1	9.1E-01	-0.2	1.0E+00	0.1	6.8E-01	0.1	9.1E-01
19483	ABCA7	A_23_P39481	NM_019112	1.4	5.87E-04	1.4	1.35E-03	2.0	5.87E-04	1.0	1.39E-04	1.5	1.38E-02	1.5	2.74E-02	1.5	1.38E-02	1.5	5.84E-03	0.2	9.9E-01	0.3	9.8E-01	0.0	9.9E-01	0.2	1.0E+00	0.1	8.8E-01	0.1	1.0E+00	-0.1	8.7E-01	0.2	8.8E-01
35655	GNA15	A_24_P331128	NM_002068	1.4	3.49E-05	1.4	3.49E-05	1.4	1.24E-03	1.3	2.97E-05	1.2	1.12E-02	1.1	2.38E-02	1.2	1.12E-02	1.5	1.31E-03	0.2	1.0E+00	0.2	1.0E+00	0.0	9.6E-01	0.2	1.0E+00	0.0	9.7E-01	0.0	1.0E+00	0.0	9.7E-01	0.1	9.3E-01
2164	NOS3	A_23_P70849	NM_000603	1.4	2.69E-03	1.3	1.91E-02	2.7	2.69E-03	1.4	1.57E-03	0.5	6.32E-01	0.4	8.30E-01	0.5	6.32E-01	0.9	2.90E-01	0.0	1.0E+00	-0.2	1.0E+00	0.6	7.1E-01	0.0	1.0E+00	0.5	3.7E-01	-0.1	1.0E+00	0.6	6.2E-02	0.5	3.7E-01
22544	GDAP1L1	A_23_P17354	NM_024034	1.4	2.66E-04	1.4	1.50E-04	1.8	6.33E-04	1.1	2.66E-04	1.2	8.87E-03	1.1	8.87E-03	2.0	4.76E-02	1.2	1.17E-03	0.2	1.0E+00	0.2	1.0E+00	0.2	7.1E-01	0.3	1.0E+00	0.1	6.5E-01	-0.1	1.0E+00	0.1	5.5E-01	0.2	6.5E-01
16294	GAGE7	A_24_P228796	NM_021123	1.4	5.33E-04	1.2	1.53E-03	1.8	5.33E-04	1.4	1.70E-05	0.7	1.42E-01	0.7	1.57E-01	0.6	1.42E-01	1.0	1.96E-02	0.0	1.0E+00	0.0	1.0E+00	0.0	1.0E+00	0.2	1.0E+00	-0.1	6.2E-01	0.2	1.0E+00	-0.1	6.0E-01	-0.2	6.2E-01
22729	NOS2	A_23_P502464	NM_000625	1.4	4.49E-03	1.4	4.49E-03	1.1	2.74E-02	1.5	1.64E-04	1.3	2.67E-02	1.3	2.67E-02	1.0	4.44E-02	1.5	1.07E-02	0.0	1.0E+00	0.0	1.0E+00	0.0	9.5E-01	0.0	1.0E+00	0.0	8.6E-01	0.0	1.0E+00	0.0	8.6E-01	-0.1	8.6E-01
13909	ADAM11	A_23_P207345	NM_002390	1.4	2.54E-03	1.4	2.77E-03	2.0	1.23E-03	0.8	2.54E-03	1.5	7.15E-03	1.6	9.20E-03	1.2	7.15E-03	1.5	1.40E-03	0.0	1.0E+00	0.0	1.0E+00	0.0	9.6E-01	0.0	1.0E+00	-0.1	9.1E-01	-0.1	1.0E+00	-0.2	6.1E-01	-0.1	9.1E-01
28541	BCL6	A_23_P57856	NM_001130845	1.4	8.03E-03	1.3	1.82E-02	1.7	8.03E-03	1.4	6.40E-04	1.3	2.43E-02	1.3	1.50E-02	1.1	3.70E-02	1.3	2.43E-02	0.0	1.0E+00	0.0	1.0E+00	-0.2	7.9E-01	0.1	1.0E+00	-0.7	5.3E-01	-0.2	1.0E+00	-0.7	2.7E-01	-0.7	5.3E-01
41315	KIAA1881	A_23_P27846	NM_001080400	1.4	3.30E-04	1.5	4.18E-04	1.4	3.30E-04	1.3	6.95E-06	0.7	3.29E-01	0.7	4.52E-01	0.7	3.29E-01	1.3	5.86E-02	0.1	1.0E+00	0.1	1.0E+00	0.1	7.1E-01	0.1	1.0E+00	-0.1	8.2E-01	0.2	1.0E+00	-0.1	8.1E-01	-0.1	8.2E-01
15835	RRAS	A_23_P39074	NM_006270	1.4	9.35E-04	1.2	9.35E-04	1.6	1.51E-03	1.4	2.12E-05	1.2	1.72E-03	1.0	6.72E-03	1.3	1.72E-03	1.2	5.95E-04	0.2	1.0E+00	0.2	1.0E+00	0.3	7.1E-01	0.1	1.0E+00	0.0	1.0E+00	0.0	1.0E+00	0.0	1.0E+00	-0.1	9.5E-01
23523	C17orf59	A_23_P152955	NM_017622	1.4	2.58E-03	1.2	3.92E-03	1.9	2.58E-03	1.4	1.72E-04	1.2	2.56E-03	1.2	1.48E-03	1.6	2.56E-03	0.9	1.61E-02	0.2	1.0E+00	0.2	1.0E+00	0.4	7.1E-01	0.2	1.0E+00	0.0	1.0E+00	-0.1	1.0E+00	0.0	9.3E-01	0.0	1.0E+00
18750	C14orf162	A_23_P14295	NR_024630	1.4	2.87E-04	1.5	2.87E-04	1.2	6.70E-03	1.4	5.63E-05	1.7	1.11E-03	1.8	1.59E-03	1.6	8.00E-04	1.7	1.11E-03	0.1	1.0E+00	0.1	1.0E+00	0.6	7.1E-01	-0.1	1.0E+00	0.1	8.9E-01	0.0	1.0E+00	0.4	5.8E-02	0.1	8.9E-01
25268	BM458245	A_32_P188	BM458245	1.4	5.06E-04	1.4	1.67E-02	2.2	5.06E-04	1.0	2.10E-04	0.9	1.11E-01	0.9	4.18E-02	1.1	1.11E-01	0.6	3.29E-01	-0.1	1.0E+00	0.0	1.0E+00	-0.3	7.1E-01	-0.1	1.0E+00	-0.1	9.6E-01	0.0	1.0E+00	-0.1	8.4E-01	-0.1	9.6E-01
25240	HIST1H2AG	A_24_P303354	NM_021064	1.4	1.03E-03	1.4	1.03E-03	1.6	1.27E-03	1.2	7.77E-05	1.0	1.34E-02	1.0	1.34E-02	1.0	1.38E-02	1.2	5.45E-03	-0.1	1.0E+00	0.0	1.0E+00	-0.1	8.9E-01	-0.1	1.0E+00	-0.1	9.3E-01	0.1	1.0E+00	-0.2	7.4E-01	-0.1	9.3E-01
10059	PTK6	A_23_P56978	NM_005975	1.4	1.57E-03	1.4	1.57E-03	1.6	7.50E-03	1.4	2.86E-04	1.2	4.54E-03	1.0	5.78E-02	1.4	4.54E-03	1.2	2.96E-03	0.2	1.0E+00	0.2	1.0E+00	0.2	7.6E-01	0.2	1.0E+00	0.0	9.3E-01	0.0	1.0E+00	0.0	9.1E-01	0.0	9.3E-01
23871	EML1	A_23_P205746	NM_001008707	1.4	1.37E-03	1.7	1.37E-03	1.0	1.56E-02	1.4	7.96E-05	1.7	1.36E-02	1.8	1.29E-02	1.6	1.36E-02	1.7	1.52E-02	0.0	1.0E+00	0.0	1.0E+00	0.0	9.7E-01	0.2	1.0E+00	-0.1	8.4E-01	0.0	1.0E+00	-0.1	5.4E-01	-0.1	8.4E-01
4025	KHDC1	A_24_P20814	NM_030568	1.4	2.05E-04	1.0	2.17E-02	2.2	2.05E-04	1.4	4.82E-06	0.9	1.95E-02	0.9	4.59E-02	1.1	1.95E-02	0.9	6.37E-03	0.0	1.0E+00	0.0	1.0E+00	-0.1	8.0E-01	0.1	1.0E+00	-0.2	4.1E-01	0.1	1.0E+00	-0.2	2.2E-01	-0.2	4.1E-01
30670	GLIPR2	A_23_P414913	NM_022343	1.4	1.24E-03	1.7	1.24E-03	1.4	1.26E-02	1.4	2.73E-04	1.5	3.20E-03	1.5	1.37E-03	0.9	1.35E-01	1.5	3.20E-03	0.0	1.0E+00	0.1	1.0E+00	-0.4	7.1E-01	0.0	1.0E+00	-0.1	8.2E-01	0.2	1.0E+00	-0.2	5.7E-01	-0.1	8.2E-01
11784	AA812298	A_32_P99533	AA812298	1.4	2.00E-04	1.4	1.49E-02	2.6	8.17E-05	1.1	2.00E-04	0.8	8.15E-02	0.8	8.15E-02	1.7	1.46E-01	0.8	3.53E-02	0.0	1.0E+00	0.0	1.0E+00	0.2	7.6E-01	0.0	1.0E+00	0.0	1.0E+00	0.2	1.0E+00	-0.1	9.4E-01	0.0	1.0E+00
44251	C19orf38	A_23_P142055	NM_001136482	1.4	1.49E-03	1.4	4.00E-04	1.4	1.70E-03	1.0	1.49E-03	0.1	8.72E-01	-0.4	8.59E-01	0.3	8.72E-01	0.1	9.55E-01	0.0	1.0E+00	0.0	1.0E+00	0.4	8.1E-01	-0.1	1.0E+00	0.1	8.7E-01	0.1	1.0E+00	0.1	8.7E-01	0.3	8.5E-01
19995	ARID5A	A_23_P143016	NM_212481	1.4	1.79E-04	1.3	1.79E-04	1.8	1.24E-03	1.4	2.92E-05	1.3	8.17E-03	1.1	1.25E-02	1.7	8.17E-03	1.3	9.03E-04	0.2	1.0E+00	0.2	1.0E+00	0.1	8.7E-01	0.2	1.0E+00	0.2	9.2E-01	0.2	1.0E+00	0.2	6.8E-01	0.1	9.2E-01
9069	PFKFB3	A_24_P261259	NM_004566	1.4	1.35E-03	1.2	1.35E-03	1.6	3.35E-03	1.4	1.55E-04	1.0	6.95E-02	0.9	1.48E-01	1.0	6.95E-02	1.2	6.21E-03	0.0	1.0E+00	0.0	1.0E+00	0.0	9.5E-01	0.0	1.0E+00	0.1	9.8E-01	0.1	1.0E+00	0.0	9.8E-01	0.2	8.5E-01
21256	HIST1H2AL	A_23_P363174	NM_003511	1.4	3.52E-04	1.4	3.52E-04	1.2	3.63E-03	1.4	3.43E-05	1.2	6.32E-03	1.1	1.71E-02	1.2	6.32E-03	1.4	4.74E-03	0.3	1.0E+00	0.4	1.0E+00	0.2	7.3E-01	0.3	1.0E+00	-0.1	9.6E-01	0.0	1.0E+00	-0.1	7.8E-01	-0.1	9.6E-01
22947	PCDH8	A_23_P36985	NM_002590	1.4	3.45E-03	1.4	3.45E-03	0.7	5.62E-03	1.8	2.81E-06	1.1	1.16E-02	1.1	1.23E-02	1.0	1.16E-02	1.5	5.27E-04	0.1	1.0E+00	0.1	1.0E+00	0.0	9.1E-01	0.2	1.0E+00	-0.2	5.1E-01	-0.1	1.0E+00	-0.2	3.1E-01	-0.2	5.1E-01
9931	CYLD	A_24_P48078	NM_015247	1.4	4.61E-03	1.2	4.61E-03	2.0	7.10E-03	1.4	8.84E-04	1.0	3.35E-02	1.0	3.35E-02	0.7	1.01E-01	1.0	5.09E-03	0.1	1.0E+00	0.0	1.0E+00	0.1	8.7E-01	0.2	1.0E+00	-0.2	7.6E-01	-0.2	1.0E+00	-0.2	4.6E-01	-0.2	7.6E-01
24287	LOC389634	A_32_P148914	BC037255	1.4	2.32E-03	1.3	5.54E-03	2.2	2.32E-03	1.4	3.90E-04	1.4	8.03E-03	1.2	9.91E-03	1.4	8.03E-03	1.4	4.22E-03	0.1	1.0E+00	0.1	1.0E+00	0.1	8.2E-01	0.1	1.0E+00	0.1	8.6E-01	0.3	1.0E+00	0.1	7.0E-01	0.1	8.6E-01
4251	TRAF1	A_23_P216970	NM_005658	1.4	1.75E-02	1.0	1.75E-02	1.5	3.25E-02	1.4	2.12E-03	0.8	4.02E-01	0.5	7.36E-01	0.8	4.02E-01	0.9	2.51E-01	0.1	1.0E+00	0.1	1.0E+00	0.5	7.1E-01	-0.2	1.0E+00	0.4	2.1E-01	0.1	1.0E+00	0.4	2.1E-01	0.4	1.6E-01
32983	CB959193	A_23_P139166	CB959193	1.4	9.84E-04	1.2	9.84E-04	1.4	1.54E-03	1.4	9.38E-06	0.8	1.02E-01	0.6	3.94E-01	0.8	1.02E-01	1.0	6.04E-02	-0.1	1.0E+00	0.0	1.0E+00	-0.1	7.9E-01	-0.1	1.0E+00	-0.1	9.1E-01	-0.1	1.0E+00	-0.1	8.3E-01	0.1	9.1E-01
35056	PPP1R1A	A_23_P53417	NM_006741	1.4	1.40E-02	1.4	2.16E-02	1.7	6.53E-03	0.7	1.40E-02	1.4	1.63E-02	1.5	1.63E-02	1.4	2.56E-02	1.4	1.22E-02	0.1	1.0E+00	0.1	1.0E+00	-0.1	7.5E-01	0.1	1.0E+00	-0.2	7.5E-01	0.1	1.0E+00	-0.2	4.0E-01	-0.2	7.5E-01
42359	A_32_P174385	A_32_P174385	A_32_P174385	1.4	9.82E-04	1.4	4.08E-03	2.0	9.82E-04	1.1	1.66E-04	1.1	7.83E-03	1.1	7.83E-03	1.6	3.25E-02	1.1	5.55E-03	0.0	1.0E+00	0.0	1.0E+00	0.0	8.7E-01	0.0	1.0E+00	-0.2	8.8E-01	0.1	1.0E+00	-0.3	4.9E-01	-0.2	8.8E-01
11961	CHRNA7	A_23_P163390	NM_000746	1.4	9.43E-04	1.3	9.43E-04	1.4	3.31E-03	1.4	3.87E-05	0.3	8.36E-01	0.3	8.51E-01	0.3	8.36E-01	1.0	2.13E-01	-0.2	1.0E+00	-0.2	1.0E+00	0.5	7.1E-01	-0.2	1.0E+00	0.4	3.7E-01	0.1	1.0E+00	0.5	2.9E-02	0.4	3.7E-01
16345	CAMK1D	A_32_P208120	NM_153498	1.4	3.69E-04	1.3	1.49E-03	1.9	3.69E-04	1.4	9.37E-06	1.6	1.18E-02	1.6	1.18E-02	1.6	6.83E-03	1.4	1.31E-02	0.1	1.0E+00	0.1	1.0E+00	0.0	9.0E-01	0.1	1.0E+00	-0.1	8.8E-01	0.1	1.0E+00	-0.2	4.9E-01	-0.1	8.8E-01
41425	PADI2	A_24_P187970	NM_007365	1.4	1.31E-03	1.4	1.32E-03	1.2	1.31E-03	1.4	5.48E-06	1.3	2.28E-03	1.3	2.28E-03	1.1	7.30E-02	1.6	1.64E-04	0.1	1.0E+00	0.1	1.0E+00	-0.1	7.7E-01	0.1	1.0E+00	0.0	9.6E-01	0.1	1.0E+00	-0.1	6.5E-01	0.0	9.6E-01
32418	ERRFI1	A_23_P46470	NM_018948	1.4	5.02E-04	1.2	5.02E-04	1.4	4.45E-03	1.6	4.76E-05	0.9	5.29E-02	0.8	5.29E-02	0.9	2.12E-01	1.1	6.96E-03	0.4	1.0E+00	0.2	1.0E+00	0.4	7.1E-01	0.4	1.0E+00	0.3	7.2E-01	0.2	1.0E+00	0.3	5.4E-01	0.4	7.2E-01
3771	IFI27	A_23_P48513	NM_005532	1.4	2.19E-03	1.2	1.36E-02	2.6	2.19E-03	1.4	7.52E-04	1.2	9.99E-03	1.1	4.55E-02	1.9	9.99E-03	1.2	2.85E-03	0.0	1.0E+00	0.0	1.0E+00	0.0	9.7E-01	0.2	1.0E+00	-0.1	6.4E-01	0.1	1.0E+00	-0.1	6.4E-01	-0.3	6.2E-01
9859	A_23_P149270	A_23_P149270	A_23_P149270	1.4	2.60E-03	1.3	3.10E-03	1.6	2.60E-03	1.4	6.44E-05	1.0	1.08E-03	1.0	5.95E-03	1.0	1.08E-03	1.3	4.52E-04	0.0	1.0E+00	0.0	1.0E+00	0.0	9.0E-01	0.0	1.0E+00	0.0	9.5E-01	0.0	1.0E+00	-0.1	6.9E-01	0.0	9.5E-01
7663	THC2582296	A_24_P788772	THC2582296	1.4	1.54E-02	1.2	2.83E-02	1.8	1.54E-02	1.4	1.82E-03	1.0	1.19E-02	0.9	6.32E-02	1.0	1.19E-02	1.1	5.32E-03	0.2	1.0E+00	0.2	1.0E+00	0.1	8.5E-01	0.2	1.0E+00	0.0	8.7E-01	0.0	1.0E+00	0.1	8.7E-01	-0.1	8.7E-01
9678	GPRIN2	A_23_P343382	AB011086	1.4	5.47E-03	1.4	2.82E-03	2.1	5.47E-03	1.0	5.67E-03	0.9	1.70E-01	0.9	2.25E-01	1.1	1.61E-01	0.8	1.70E-01	0.0	1.0E+00	0.0	1.0E+00	0.4	7.1E-01	0.0	1.0E+00	0.4	4.3E-01	0.1	1.0E+00	0.4	1.7E-01	0.4	4.3E-01
2388	FLJ36031	A_23_P406616	NM_175884	1.4	7.04E-03	1.4	7.04E-03	1.7	1.96E-02	1.3	2.31E-03	1.3	3.91E-02	1.4	4.19E-02	1.3	2.22E-02	1.2	3.91E-02	0.3	1.0E+00	0.3	1.0E+00	0.3	7.1E-01	0.4	1.0E+00	0.1	9.6E-01	0.1	1.0E+00	0.1	9.6E-01	0.1	9.6E-01
38282	SLC8A2	A_32_P45844	NM_015063	1.4	3.16E-04	1.8	3.16E-04	1.2	2.98E-03	1.4	1.81E-05	1.4	3.38E-02	1.4	4.24E-02	1.4	2.34E-02	1.5	3.38E-02	0.2	1.0E+00	0.2	1.0E+00	0.1	7.2E-01	0.2	1.0E+00	0.0	9.7E-01	0.1	1.0E+00	0.0	9.5E-01	0.0	9.7E-01
12345	GABRE	A_23_P159775	NM_004961	1.4	1.25E-03	1.6	4.08E-03	1.1	1.25E-03	1.4	2.24E-06	0.8	8.29E-02	0.8	1.79E-02	0.4	2.21E-01	0.8	8.29E-02	0.1	1.0E+00	0.0	1.0E+00	0.3	7.1E-01	0.1	1.0E+00	0.3	3.1E-01	0.1	1.0E+00	0.3	3.1E-01	0.4	3.0E-01
28795	PNPLA7	A_23_P43455	NM_152286	1.4	5.45E-04	1.4	5.45E-04	1.5	2.50E-03	0.9	3.48E-04	1.3	4.62E-02	1.1	1.30E-01	1.3	4.62E-02	1.4	1.80E-02	0.2	1.0E+00	0.2	1.0E+00	0.4	7.1E-01	-0.1	1.0E+00	0.1	9.4E-01	0.0	1.0E+00	0.1	9.4E-01	0.1	9.3E-01
15424	MYCT1	A_24_P945059	NM_025107	1.4	3.17E-04	0.8	2.66E-02	1.6	3.17E-04	1.4	3.66E-06	0.4	1.46E-01	0.4	1.46E-01	0.3	2.27E-01	0.7	1.15E-01	-0.2	1.0E+00	-0.2	1.0E+00	-0.2	8.1E-01	-0.2	1.0E+00	-0.1	7.6E-01	0.0	1.0E+00	-0.1	7.6E-01	-0.2	5.9E-01
20023	LOC255374	A_24_P221198	NM_203397	1.4	1.94E-03	1.4	1.94E-03	1.5	1.50E-02	1.2	1.25E-03	1.2	2.25E-02	1.2	2.25E-02	1.1	4.28E-02	1.2	5.63E-03	0.1	1.0E+00	0.1	1.0E+00	0.1	8.1E-01	0.1	1.0E+00	0.0	9.7E-01	0.2	1.0E+00	0.0	9.7E-01	-0.1	9.7E-01
9199	RAMP1	A_23_P50946	NM_005855	1.4	1.05E-02	1.4	1.16E-02	1.7	1.05E-02	1.1	3.12E-03	1.2	5.94E-03	1.2	5.94E-03	1.4	1.07E-02	1.2	1.41E-03	0.1	1.0E+00	0.1	1.0E+00	0.0	9.5E-01	0.1	1.0E+00	-0.1	9.0E-01	0.1	1.0E+00	-0.1	8.6E-01	-0.1	9.0E-01
7015	NKD2	A_23_P41804	NM_033120	1.4	9.79E-04	1.5	9.79E-04	1.2	1.70E-03	1.4	3.25E-06	1.3	1.02E-03	1.2	2.61E-03	1.3	1.02E-03	1.5	4.81E-04	0.2	1.0E+00	0.0	1.0E+00	0.2	7.1E-01	0.2	1.0E+00	-0.1	9.4E-01	0.0	1.0E+00	-0.1	7.3E-01	-0.1	9.4E-01
15263	CRTAC1	A_23_P431971	NM_018058	1.4	1.29E-02	1.4	1.29E-02	1.4	1.75E-02	1.1	1.93E-03	0.9	2.39E-01	0.9	2.39E-01	0.6	3.81E-01	1.1	8.40E-02	0.1	1.0E+00	0.1	1.0E+00	-0.2	7.4E-01	0.1	1.0E+00	-0.1	9.0E-01	0.1	1.0E+00	-0.2	5.3E-01	-0.1	9.0E-01
17273	CALCA	A_23_P301846	NM_001033952	1.4	6.45E-04	1.0	3.47E-03	2.1	6.45E-04	1.4	7.26E-05	0.5	1.52E-01	0.4	4.04E-01	0.8	7.93E-02	0.5	1.52E-01	0.2	1.0E+00	0.2	1.0E+00	0.0	9.9E-01	0.4	1.0E+00	0.1	7.5E-01	0.0	1.0E+00	0.1	7.5E-01	0.2	4.9E-01
36711	TP53INP1	A_23_P168882	NM_033285	1.4	3.41E-03	1.2	3.41E-03	1.7	4.61E-03	1.4	2.27E-04	0.7	1.94E-01	0.7	1.94E-01	0.4	6.28E-01	0.8	1.21E-01	0.2	1.0E+00	0.2	1.0E+00	0.3	7.1E-01	0.2	1.0E+00	-0.3	7.0E-01	-0.2	1.0E+00	-0.3	7.0E-01	-0.6	6.8E-01
16560	TTC21A	A_23_P428248	NM_145755	1.4	4.82E-04	1.4	2.53E-03	2.0	4.82E-04	1.2	5.19E-05	1.4	2.96E-02	1.4	2.27E-02	1.6	3.84E-02	1.2	2.96E-02	0.1	1.0E+00	0.1	1.0E+00	-0.1	9.1E-01	0.1	1.0E+00	-0.1	9.7E-01	-0.1	1.0E+00	-0.2	6.5E-01	-0.1	9.7E-01
9604	CCK	A_23_P425681	NM_000729	1.4	5.11E-03	1.4	5.11E-03	1.0	3.85E-02	1.8	7.66E-05	0.4	7.48E-01	0.4	7.60E-01	0.3	7.48E-01	0.9	2.81E-01	0.1	1.0E+00	0.0	1.0E+00	0.2	7.1E-01	0.1	1.0E+00	0.0	9.9E-01	0.0	1.0E+00	0.1	8.7E-01	0.0	9.9E-01
6036	KRTAP9-2	A_23_P89646	NM_031961	1.4	1.66E-04	1.4	3.19E-03	3.4	1.66E-04	1.3	1.35E-04	1.2	1.55E-01	1.2	2.95E-01	2.1	2.78E-02	1.1	1.55E-01	0.2	9.1E-01	0.2	9.1E-01	0.5	6.2E-01	0.2	1.0E+00	0.2	5.9E-01	-0.1	1.0E+00	0.2	5.4E-01	0.2	5.9E-01
39470	IFI27	A_23_P48513	NM_005532	1.4	9.01E-04	1.1	5.59E-03	2.7	9.01E-04	1.4	2.01E-04	1.3	1.58E-02	1.0	2.15E-02	1.9	1.58E-02	1.3	1.22E-03	0.0	1.0E+00	0.0	1.0E+00	0.0	9.2E-01	0.0	1.0E+00	0.0	9.7E-01	0.2	1.0E+00	-0.1	8.6E-01	0.0	9.7E-01
39459	IFI27	A_23_P48513	NM_005532	1.4	6.70E-04	1.1	5.84E-03	2.8	6.70E-04	1.4	1.43E-04	1.3	1.47E-02	1.0	1.95E-02	1.9	1.47E-02	1.3	9.61E-04	0.0	1.0E+00	-0.1	1.0E+00	0.0	9.3E-01	0.1	1.0E+00	0.0	1.0E+00	0.1	1.0E+00	-0.1	8.2E-01	0.0	1.0E+00
39862	HBB	A_23_P203558	NM_000518	1.4	5.18E-03	1.4	1.05E-02	1.4	5.18E-03	1.6	3.48E-05	0.5	2.94E-01	0.3	6.81E-01	0.5	2.94E-01	0.8	2.14E-01	0.0	1.0E+00	0.0	1.0E+00	0.1	7.4E-01	0.0	1.0E+00	-0.1	8.7E-01	0.1	1.0E+00	-0.2	5.2E-01	-0.1	8.7E-01
34303	HAVCR2	A_23_P18903	NM_032782	1.4	2.94E-03	0.8	1.30E-02	1.6	2.94E-03	1.4	6.83E-05	1.2	4.52E-03	0.6	1.54E-01	1.2	4.52E-03	1.2	1.72E-03	0.1	1.0E+00	0.1	1.0E+00	0.0	9.5E-01	0.2	1.0E+00	-0.3	3.2E-01	-0.2	1.0E+00	-0.3	1.4E-01	-0.3	3.2E-01
7213	THC2678045	A_32_P174285	THC2678045	1.4	6.66E-04	1.4	6.66E-04	2.2	1.12E-03	1.2	2.11E-04	1.6	1.70E-03	1.4	4.00E-03	1.7	1.70E-03	1.6	2.72E-04	-0.1	1.0E+00	-0.1	1.0E+00	-0.1	8.6E-01	0.0	1.0E+00	0.0	1.0E+00	0.1	1.0E+00	0.0	9.4E-01	0.0	1.0E+00
19269	BX101288	A_32_P34696	BX101288	1.4	1.35E-03	1.4	1.35E-03	1.5	1.66E-03	1.2	3.33E-05	1.2	9.98E-03	1.3	9.98E-03	0.8	3.82E-02	1.2	5.45E-03	0.0	1.0E+00	0.0	1.0E+00	-0.1	8.2E-01	0.1	1.0E+00	-0.2	3.9E-01	0.0	1.0E+00	-0.2	3.9E-01	-0.3	3.7E-01
43465	TM4SF5	A_23_P27107	NM_003963	1.4	4.59E-03	1.2	2.47E-02	2.2	4.59E-03	1.4	8.46E-04	0.7	1.73E-01	0.3	6.82E-01	0.7	1.18E-01	0.7	1.73E-01	0.0	1.0E+00	0.0	1.0E+00	0.1	9.0E-01	-0.1	1.0E+00	0.1	9.8E-01	0.3	1.0E+00	0.0	9.8E-01	0.1	9.4E-01
6793	C1orf38	A_24_P85775	NM_001039477	1.4	6.71E-04	1.2	1.26E-03	1.8	6.71E-04	1.4	8.79E-06	1.0	3.95E-02	1.0	3.95E-02	0.7	4.64E-01	1.4	9.33E-04	0.2	1.0E+00	0.2	1.0E+00	0.1	9.1E-01	0.2	1.0E+00	-0.1	9.9E-01	-0.1	1.0E+00	-0.1	8.6E-01	0.0	9.9E-01
36112	IFI27	A_23_P48513	NM_005532	1.4	2.02E-03	1.2	1.27E-02	2.6	2.02E-03	1.4	6.18E-04	1.3	7.39E-03	1.1	3.46E-02	1.9	7.39E-03	1.3	1.87E-03	0.0	1.0E+00	0.0	1.0E+00	0.0	1.0E+00	0.2	1.0E+00	-0.1	6.7E-01	0.1	1.0E+00	-0.1	6.5E-01	-0.2	6.7E-01
2737	DNAH2	A_32_P206899	NM_020877	1.4	1.93E-03	1.2	1.64E-02	2.5	1.93E-03	1.4	6.29E-04	0.8	1.60E-01	0.7	3.58E-01	1.2	1.34E-01	0.8	1.60E-01	0.1	1.0E+00	0.1	1.0E+00	0.4	7.1E-01	0.1	1.0E+00	0.2	7.1E-01	0.0	1.0E+00	0.2	4.7E-01	0.2	7.1E-01
25208	TACSTD2	A_23_P149529	NM_002353	1.4	4.69E-03	1.0	4.69E-03	1.7	6.74E-03	1.4	2.65E-04	0.9	2.28E-02	0.9	2.28E-02	0.8	5.36E-02	0.9	3.04E-03	0.0	1.0E+00	0.1	1.0E+00	-0.1	8.2E-01	0.0	1.0E+00	-0.1	9.8E-01	-0.1	1.0E+00	-0.1	8.9E-01	0.0	9.8E-01
9561	A_24_P477353	A_24_P477353	A_24_P477353	1.4	1.40E-02	1.4	8.51E-03	1.6	2.95E-02	1.0	1.40E-02	0.8	1.70E-01	0.5	3.61E-01	0.9	1.70E-01	0.8	5.38E-02	0.0	1.0E+00	0.0	1.0E+00	0.2	8.7E-01	-0.2	1.0E+00	0.4	7.5E-01	0.1	1.0E+00	0.4	4.7E-01	0.4	7.5E-01
26916	OPTN	A_23_P1461	NM_001008211	1.4	1.00E-02	0.9	1.04E-02	1.7	1.00E-02	1.4	5.02E-04	0.9	1.49E-02	0.9	1.49E-02	1.1	1.59E-02	0.9	2.67E-03	0.1	1.0E+00	0.1	1.0E+00	0.1	9.1E-01	0.1	1.0E+00	-0.2	9.0E-01	-0.2	1.0E+00	-0.2	6.8E-01	-0.1	9.0E-01

Feature	Gene symbol	Probe	Transcript	72 hour siCASP8AP2								48 hours siCASP8AP2								24 hours siCASP8AP2								10 hours siCASP8AP2							
				Median	Median	1	1	3	3	6	6	Median	Median	1	1	3	3	6	6	Median	Median	1	1	3	3	6	6	Median	Median	1	1	3	3	6	6
				M	q	M	q	M	q	M	q	M	q	M	q	M	q	M	q	M	q	M	q	M	q	M	q	M	q	M	q	M	q	M	q
16364	LMO2	A_23_P53126	NM_005574	1.4	3.52E-03	1.4	3.52E-03	1.4	2.97E-02	1.4	1.29E-03	1.0	2.07E-02	0.9	2.07E-02	1.1	8.83E-03	1.0	2.84E-02	-0.1	1.0E+00	-0.1	1.0E+00	-0.1	9.0E-01	-0.1	1.0E+00	-0.3	8.2E-01	-0.2	1.0E+00	-0.3	6.4E-01	-0.3	8.2E-01
35078	TBXA2R	A_23_P90357	NM_001060	1.4	6.52E-05	1.2	1.75E-04	2.4	6.52E-05	1.4	7.34E-06	1.2	8.50E-02	1.2	1.31E-01	1.6	7.81E-02	1.0	8.50E-02	0.3	1.0E+00	0.3	1.0E+00	0.5	7.1E-01	0.3	1.0E+00	-0.2	6.9E-01	-0.1	1.0E+00	-0.2	6.9E-01	-0.3	4.5E-01
9392	CGB	A_24_P395814	NM_000737	1.4	4.09E-04	1.4	4.80E-03	2.0	4.09E-04	1.4	1.53E-05	0.7	3.24E-01	0.6	5.59E-01	0.7	3.24E-01	1.1	9.42E-02	0.0	1.0E+00	0.0	1.0E+00	0.3	7.1E-01	0.0	1.0E+00	0.0	9.8E-01	0.0	1.0E+00	0.2	3.2E-01	0.0	9.8E-01
11288	IFI27	A_23_P48513	NM_005532	1.4	1.01E-03	1.2	5.37E-03	2.8	1.01E-03	1.4	2.77E-04	1.4	1.19E-02	1.2	2.22E-02	2.0	1.19E-02	1.4	1.83E-03	0.0	1.0E+00	0.0	1.0E+00	-0.1	9.2E-01	0.1	1.0E+00	0.0	9.8E-01	0.2	1.0E+00	-0.1	8.1E-01	0.0	9.8E-01
22484	SH3D19	A_24_P366656	NM_001009555	1.4	3.69E-03	1.2	3.69E-03	1.8	4.50E-03	1.4	1.92E-04	0.7	1.69E-01	0.7	7.14E-02	1.2	1.69E-01	0.7	2.03E-01	-0.1	1.0E+00	0.0	1.0E+00	-0.2	7.9E-01	-0.1	1.0E+00	-0.1	8.1E-01	0.0	1.0E+00	-0.1	7.6E-01	-0.2	8.1E-01
12624	CACNA2D2	A_23_P346900	NM_001005505	1.4	5.96E-03	1.7	6.58E-03	1.4	5.96E-03	1.0	1.06E-03	1.6	1.81E-02	1.6	6.40E-03	1.0	1.81E-02	1.6	1.90E-02	0.0	1.0E+00	0.0	1.0E+00	-0.3	7.1E-01	0.0	1.0E+00	-0.2	5.9E-01	-0.1	1.0E+00	-0.3	2.8E-01	-0.2	5.9E-01
28618	PDE2A	A_23_P401106	NM_002599	1.4	3.03E-04	1.1	1.53E-03	2.2	3.03E-04	1.4	1.63E-05	0.8	9.38E-03	0.7	3.17E-02	1.2	7.78E-03	0.8	9.38E-03	0.1	1.0E+00	0.1	1.0E+00	0.1	7.8E-01	0.0	1.0E+00	0.0	9.6E-01	0.0	1.0E+00	0.0	9.4E-01	-0.1	9.6E-01
5930	IFI27	A_23_P48513	NM_005532	1.4	1.09E-03	1.1	5.84E-03	2.8	1.09E-03	1.4	2.86E-04	1.3	1.71E-02	1.1	1.71E-02	2.0	1.77E-02	1.3	2.34E-03	0.0	1.0E+00	0.0	1.0E+00	0.0	9.8E-01	0.1	1.0E+00	0.0	9.9E-01	0.2	1.0E+00	-0.1	8.8E-01	0.0	9.9E-01
31348	P2RY2	A_23_P24903	NM_176072	1.4	5.03E-03	1.3	6.86E-03	1.7	5.03E-03	1.4	2.19E-04	1.3	2.44E-02	1.3	3.17E-02	1.3	2.44E-02	1.4	9.63E-03	0.1	1.0E+00	0.1	1.0E+00	-0.2	7.4E-01	0.1	1.0E+00	-0.1	8.9E-01	0.0	1.0E+00	-0.3	3.2E-01	-0.1	8.9E-01
17800	RASA4	A_23_P168449	NM_006989	1.4	5.33E-03	1.3	5.33E-03	1.9	8.03E-03	1.4	7.45E-04	1.2	4.65E-02	1.1	4.65E-02	1.6	5.27E-02	1.2	6.88E-03	0.1	1.0E+00	0.0	1.0E+00	0.1	7.7E-01	0.1	1.0E+00	0.2	7.4E-01	0.3	1.0E+00	0.2	4.1E-01	0.1	7.4E-01
37843	CHRD	A_23_P502047	NM_003741	1.4	1.13E-03	1.4	1.13E-03	2.0	1.83E-03	1.4	1.33E-04	1.1	6.98E-02	0.9	1.23E-01	1.1	6.98E-02	1.5	1.02E-02	0.2	1.0E+00	0.1	1.0E+00	0.3	7.1E-01	0.2	1.0E+00	0.1	9.8E-01	0.2	1.0E+00	0.0	9.8E-01	0.1	9.6E-01
13797	ATP4A	A_23_P430728	NM_000704	1.4	3.56E-03	1.5	1.09E-02	1.4	3.56E-03	1.4	3.71E-05	1.5	1.21E-02	1.2	1.21E-02	1.5	1.24E-02	1.5	8.77E-04	0.0	1.0E+00	-0.1	1.0E+00	0.0	9.8E-01	0.1	1.0E+00	0.0	9.8E-01	0.0	1.0E+00	-0.2	3.8E-01	0.0	9.8E-01
12795	MAGEC1	A_23_P217178	NM_005462	1.4	6.05E-04	1.5	6.05E-04	1.1	4.23E-03	1.4	9.51E-06	1.4	3.52E-03	1.6	1.94E-03	0.9	1.58E-02	1.4	3.52E-03	0.1	1.0E+00	-0.1	1.0E+00	0.2	7.3E-01	0.1	1.0E+00	0.0	9.7E-01	0.1	1.0E+00	-0.1	7.1E-01	0.0	9.7E-01
38022	ASMTL	A_23_P159539	NM_004192	1.4	1.66E-02	1.4	3.10E-03	1.5	1.71E-02	0.8	1.66E-02	1.6	2.81E-03	1.3	5.95E-03	1.6	2.81E-03	1.6	2.51E-04	0.1	1.0E+00	0.0	1.0E+00	0.1	8.6E-01	0.1	1.0E+00	0.1	9.9E-01	0.2	1.0E+00	0.0	9.9E-01	0.1	7.4E-01
18746	AA514833	A_32_P81623	AA514833	1.4	9.95E-04	1.4	3.88E-03	2.0	9.95E-04	1.4	3.47E-05	1.3	1.86E-02	1.6	1.28E-02	1.0	2.27E-02	1.3	1.86E-02	0.4	9.3E-01	0.5	7.2E-01	0.0	9.3E-01	0.4	1.0E+00	-0.2	8.6E-01	-0.1	1.0E+00	-0.3	3.5E-01	-0.2	8.6E-01
15068	N4BP3	A_23_P58747	NM_015111	1.4	1.02E-02	1.4	1.64E-02	2.4	1.02E-02	1.3	7.88E-03	1.4	4.03E-02	1.2	4.03E-02	1.7	6.10E-02	1.4	4.16E-03	0.1	8.2E-01	0.1	1.0E+00	0.1	8.2E-01	0.4	3.7E-01	0.1	8.3E-01	0.2	1.0E+00	0.1	8.0E-01	0.1	8.3E-01
31202	SOX3	A_23_P85218	NM_005634	1.4	3.03E-04	1.4	3.03E-04	1.3	4.92E-03	2.0	4.47E-05	0.9	4.74E-01	0.1	9.58E-01	1.6	3.47E-01	0.9	4.74E-01	0.0	1.0E+00	0.0	1.0E+00	0.3	8.1E-01	-0.3	1.0E+00	0.9	2.8E-01	-0.2	1.0E+00	0.9	2.8E-01	0.9	6.2E-02
27675	CLEC11A	A_23_P153489	NM_002975	1.4	8.37E-04	1.4	8.37E-04	1.9	1.01E-03	1.3	9.03E-05	1.2	7.27E-02	1.2	7.27E-02	1.1	9.25E-02	1.3	1.91E-02	0.0	1.0E+00	0.1	1.0E+00	0.0	9.8E-01	0.0	1.0E+00	0.0	9.7E-01	0.0	1.0E+00	0.0	9.7E-01	-0.1	9.3E-01
39911	FLJ37078	A_23_P331700	NM_001110199	1.5	7.24E-04	1.5	1.40E-02	2.4	7.24E-04	1.3	1.77E-04	1.3	2.95E-02	1.2	5.13E-02	1.3	2.95E-02	1.2	1.56E-02	0.1	1.0E+00	0.1	1.0E+00	-0.1	8.7E-01	0.1	1.0E+00	-0.2	6.3E-01	-0.1	1.0E+00	-0.3	3.1E-01	-0.2	6.3E-01
32565	GLI1	A_23_P105251	NM_005269	1.5	1.24E-03	1.4	4.46E-03	1.7	1.24E-03	1.5	1.29E-04	1.3	1.64E-02	1.2	2.67E-02	1.9	1.15E-02	1.3	1.64E-02	0.1	1.0E+00	0.1	1.0E+00	0.0	9.6E-01	0.2	1.0E+00	-0.2	8.4E-01	-0.2	1.0E+00	-0.2	5.9E-01	-0.2	8.4E-01
10416	COLEC11	A_23_P120125	NM_199235	1.5	1.66E-02	1.5	1.66E-02	2.6	5.22E-03	1.0	1.74E-02	1.2	3.90E-03	1.2	2.96E-03	1.3	3.90E-03	1.1	1.83E-02	0.2	1.0E+00	0.2	1.0E+00	0.1	8.7E-01	0.2	1.0E+00	-0.2	9.3E-01	-0.1	1.0E+00	-0.3	8.1E-01	-0.2	9.3E-01
32784	TNFAIP2	A_23_P421423	NM_006291	1.5	6.20E-04	1.3	6.20E-04	1.5	5.31E-03	1.5	5.83E-05	1.2	3.20E-03	1.5	3.20E-03	1.1	3.36E-03	1.2	2.37E-03	0.4	7.1E-01	0.5	2.7E-01	0.2	7.1E-01	0.4	1.0E+00	-0.1	9.3E-01	0.0	1.0E+00	-0.1	8.3E-01	-0.1	9.3E-01
20476	DENND3	A_32_P175301	NM_014957	1.5	1.84E-04	1.2	1.84E-04	1.8	2.22E-04	1.5	1.25E-06	1.0	5.62E-03	0.6	1.13E-01	1.3	2.15E-03	1.0	5.62E-03	0.1	1.0E+00	0.1	1.0E+00	0.1	7.4E-01	0.0	1.0E+00	0.1	8.9E-01	0.1	1.0E+00	0.1	6.9E-01	0.1	8.9E-01
24816	C20orf112	A_23_P303548	AK097804	1.5	2.30E-03	1.1	2.30E-03	1.7	3.64E-03	1.5	9.75E-05	1.4	1.01E-02	1.4	1.01E-02	1.0	4.53E-02	1.6	2.26E-03	0.2	8.0E-01	0.3	7.3E-01	0.1	8.0E-01	0.2	1.0E+00	-0.1	8.7E-01	0.0	1.0E+00	-0.1	6.8E-01	-0.1	8.7E-01
34436	BG216262	A_32_P133090	BG216262	1.5	5.47E-04	1.3	2.97E-03	1.9	5.47E-04	1.5	1.28E-05	1.5	2.28E-03	1.3	3.11E-03	1.5	2.28E-03	1.6	1.96E-03	0.1	1.0E+00	0.1	1.0E+00	-0.1	8.5E-01	0.1	1.0E+00	0.0	9.4E-01	0.0	1.0E+00	0.0	9.3E-01	-0.1	9.4E-01
18102	NDRG4	A_23_P140748	NM_022910	1.5	3.18E-03	1.4	3.18E-03	1.5	2.51E-02	1.6	4.50E-04	1.2	5.13E-02	1.0	8.09E-02	1.2	5.13E-02	1.3	1.16E-02	0.1	1.0E+00	0.1	1.0E+00	0.2	7.1E-01	0.1	1.0E+00	-0.1	9.8E-01	-0.1	1.0E+00	-0.1	9.1E-01	0.0	9.8E-01
5260	RNF182	A_23_P399255	NM_152737	1.5	1.25E-03	1.0	1.25E-03	1.5	1.79E-03	1.6	6.79E-06	0.7	1.81E-01	0.7	1.81E-01	0.7	2.76E-01	0.9	3.58E-02	0.1	1.0E+00	0.1	1.0E+00	0.1	8.1E-01	0.1	1.0E+00	-0.2	4.7E-01	0.0	1.0E+00	-0.2	4.1E-01	-0.3	4.7E-01
13294	NLRP7	A_23_P16384	NM_139176	1.5	1.14E-03	1.5	1.24E-03	1.7	1.14E-03	1.3	3.11E-05	1.1	5.38E-03	0.7	9.71E-02	1.4	5.38E-03	1.1	2.06E-03	0.0	1.0E+00	0.0	1.0E+00	0.0	9.8E-01	0.1	1.0E+00	0.0	9.5E-01	0.0	1.0E+00	0.0	9.5E-01	-0.1	9.2E-01
13754	ASGR2	A_23_P130113	NM_080912	1.5	5.69E-03	1.4	5.69E-03	2.0	9.10E-03	1.5	1.65E-03	0.6	6.83E-01	0.4	8.35E-01	0.6	6.83E-01	0.6	6.20E-01	-0.2	1.0E+00	-0.3	1.0E+00	0.4	7.1E-01	-0.2	1.0E+00	0.2	6.7E-01	0.0	1.0E+00	0.5	3.1E-02	0.2	6.7E-01
9211	IFI27	A_23_P48513	NM_005532	1.5	2.18E-03	1.2	1.11E-02	2.6	2.18E-03	1.5	5.87E-04	1.3	1.04E-02	1.1	3.76E-02	1.9	1.04E-02	1.3	2.38E-03	0.0	1.0E+00	0.0	1.0E+00	0.0	9.6E-01	0.2	1.0E+00	-0.1	8.2E-01	0.1	1.0E+00	-0.1	7.4E-01	-0.2	8.2E-01
4278	RAB11FIP1	A_23_P31873	NM_001002233	1.5	4.17E-04	1.5	4.17E-04	1.4	4.35E-03	1.6	9.82E-05	1.1	5.64E-03	1.0	1.98E-02	1.1	5.20E-03	1.2	5.64E-03	0.3	1.0E+00	0.3	1.0E+00	0.2	7.1E-01	0.4	1.0E+00	-0.2	6.8E-01	0.1	1.0E+00	-0.2	6.5E-01	-0.3	6.8E-01
41271	HBB	A_23_P203558	NM_000518	1.5	4.11E-03	1.4	7.97E-03	1.5	4.11E-03	1.5	6.10E-05	0.6	1.15E-01	0.4	2.92E-01	0.6	2.89E-02	0.9	1.15E-01	0.1	1.0E+00	0.1	1.0E+00	0.1	8.0E-01	0.1	1.0E+00	-0.1	8.7E-01	0.0	1.0E+00	-0.2	4.4E-01	-0.1	8.7E-01
41631	ZAP70	A_23_P39682	NM_001079	1.5	2.61E-03	1.1	2.61E-03	1.6	2.78E-03	1.5	9.93E-05	1.2	1.96E-02	0.6	1.62E-01	1.5	6.99E-03	1.2	1.96E-02	0.1	1.0E+00	0.1	1.0E+00	0.1	9.1E-01	0.1	1.0E+00	-0.2	8.8E-01	0.0	1.0E+00	-0.2	7.8E-01	-0.2	8.8E-01
2309	CCR7	A_23_P343398	NM_001838	1.5	5.46E-03	0.9	3.37E-02	2.5	5.46E-03	1.5	2.52E-03	0.9	4.64E-03	0.7	3.51E-02	1.6	3.37E-03	0.9	4.64E-03	0.4	1.0E+00	0.3	1.0E+00	0.4	7.1E-01	0.4	1.0E+00	-0.3	7.5E-01	-0.3	1.0E+00	-0.3	6.3E-01	-0.4	7.5E-01
11646	NCRNA00087	A_32_P104432	NR_024493	1.5	2.25E-03	1.4	2.25E-03	1.5	3.71E-03	1.5	3.67E-05	1.4	2.70E-03	1.4	9.78E-03	1.5	2.70E-03	1.3	1.93E-03	0.4	4.3E-01	0.3	4.3E-01	0.4	3.0E-01	0.4	1.0E+00	0.0	1.0E+00	0.1	1.0E+00	-0.1	6.6E-01	0.0	1.0E+00
9430	EPOR	A_23_P381954	NM_000121	1.5	1.24E-03	1.6	1.82E-03	1.5	1.24E-03	1.1	3.90E-05	1.4	1.76E-03	1.6	6.44E-04	1.1	1.76E-03	1.4	7.41E-03	0.1	1.0E+00	0.1	1.0E+00	0.0	9.2E-01	0.1	1.0E+00	-0.2	7.5E-01	0.0	1.0E+00	-0.3	2.8E-01	-0.2	7.5E-01
29583	SLC30A3	A_23_P302568	NM_003459	1.5	1.79E-04	1.5	1.79E-04	1.7	1.24E-03	1.5	8.59E-05	1.4	4.17E-03	1.4	4.17E-03	1.4	4.56E-03	1.5	5.57E-04	0.2	1.0E+00	0.1	1.0E+00	0.2	7.1E-01	0.2	1.0E+00	0.2	7.0E-01	0.1	1.0E+00	0.2	5.4E-01	0.2	7.0E-01
24263	TRIM15	A_23_P500300	NM_033229	1.5	2.58E-03	1.3	2.58E-03	1.6	5.96E-03	1.5	1.61E-04	1.1	3.04E-02	1.1	3.04E-02	1.2	4.00E-02	1.1	7.97E-03	0.1	1.0E+00	0.0	1.0E+00	0.1	9.1E-01	0.1	1.0E+00	0.2	9.0E-01	0.2	1.0E+00	0.1	8.7E-01	0.2	9.0E-01
28018	DHX58	A_23_P38346	NM_024119	1.5	4.37E-03	1.2	4.37E-03	2.1	5.69E-03	1.5	7.51E-04	1.3	1.20E-02	1.3	3.72E-02	1.7	1.05E-02	1.3	1.20E-02	0.0	1.0E+00	0.0	1.0E+00	-0.2	7.6E-01	0.1	1.0E+00	-0.2	8.6E-01	-0.2	1.0E+00	-0.2	6.8E-01	-0.2	8.6E-01
41401	A_24_P332953	A_24_P332953	A_24_P332953	1.5	9.21E-03	1.5	9.21E-03	2.2	1.09E-02	1.2	8.29E-03	1.0	1.79E-01	0.6	3.36E-01	1.0	1.79E-01	1.1	3.95E-02	0.1	1.0E+00	0.1	1.0E+00	0.2	8.0E-01	0.0	1.0E+00	0.0	1.0E+00	0.3	1.0E+00	-0.1	9.7E-01	0.0	1.0E+00
2226	A_32_P73413	A_32_P73413	A_32_P73413	1.5	1.14E-03	1.5	3.46E-03	1.9	1.14E-03	1.4	4.20E-05	1.1	7.87E-03	1.1	1.46E-02	1.6	7.87E-03	1.1	4.09E-03	0.3	1.0E+00	0.3	1.0E+00	0.1	9.2E-01	0.3	1.0E+00	-0.2	8.3E-01	0.0	1.0E+00	-0.2	7.9E-01	-0.3	8.3E-01
37092	ENST00000342314	A_32_P42236	ENST00000342314	1.5	1.10E-03	1.5	1.10E-03	1.4	8.20E-03	1.6	7.19E-05	1.1	1.54E-02	1.1	1.54E-02	0.9	2.59E-02	1.3	3.65E-03	0.2	1.0E+00	0.2	1.0E+00	0.1	8.2E-01	0.4	1.0E+00	0.1	9.3E-01	0.1	1.0E+00	0.1	8.3E-01	0.1	9.3E-01
41609	ZCCHC12	A_23_P125809	NM_173798	1.5	2.57E-03	1.5	3.30E-03	1.4	2.57E-03	1.9	7.58E-06	1.0	3.87E-02	1.0	3.87E-02	1.0	1.76E-01	1.5	9.51E-03	0.0	1.0E+00	0.0	1.0E+00	-0.2	7.1E-01	0.1	1.0E+00	-0.2	9.9E-01	-0.2	1.0E+00	-0.3	5.1E-01	0.0	9.9E-01
33229	C15orf48	A_23_P26024	NM_032413	1.5	3.45E-04	1.0	4.74E-03	1.7	3.45E-04	1.5	3.88E-06	0.8	4.64E-02	0.6	1.75E-01	0.8	4.64E-02	1.1	1.13E-02	0.0	1.0E+00	0.0	1.0E+00	0.0	9.7E-01	0.0	1.0E+00	-0.1	9.0E-01	0.0	1.0E+00	-0.2	3.0E-01	-0.1	9.0E-01
33581	LOC284422	A_23_P397120	NM_001136503	1.5	1.13E-03	1.3	1.85E-03	2.7	1.13E-03	1.5	2.11E-04	1.4	7.06E-03	1.1	5.17E-02	1.7	7.06E-03	1.4	5.83E-03	0.1	1.0E+00	0.1	1.0E+00	0.0	9.5E-01	0.1	1.0E+00	0.1	9.6E-01	0.2	1.0E+00	-0.2	7.1E-01	0.1	9.6E-01
32648	IFI27	A_23_P48513	NM_005532	1.5	1.24E-03	1.2	7.83E-03	2.8	1.24E-03	1.5	3.30E-04	1.2	1.55E-02	1.1	2.42E-02	2.0	1.55E-02	1.2	2.16E-03	0.0	1.0E+00	-0.1	1.0E+00	0.0	9.6E-01	0.1	1.0E+00	0.0	9.9E-01	0.1	1.0E+00	-0.1	9.0E-01	0.0	9.9E-01
8558	RHOV	A_23_P424561	NM_133639	1.5	3.77E-04	1.5	3.77E-04	1.8	2.48E-03	1.2	2.55E-04	1.5	4.52E-03	1.5	4.97E-03	1.4	4.52E-03	1.6	4.92E-04	0.3	1.0E+00	0.1	1.0E+00	0.3	7.1E-01	0.3	1.0E+00	0.2	7.1E-01	0.2	1.0E+00	0.1	7.1E-01	0.3	3.4E-01
32260	CCDC37	A_23_P426153	NM_182628	1.5	1.25E-03	1.4	6.82E-03	3.1	1.25E-03	1.5	6.11E-04	1.5	1.24E-02	1.4	1.24E-02	2.3	1.68E-02	1.5	8.68E-04	0.1	1.0E+00	0.1	1.0E+00	-0.2	7.4E-01	0.1	1.0E+00	0.1	9.6E-01	0.1	1.0E+00	0.0	9.6E-01	0.1	9.3E-01
31896	GDPD5	A_23_P87401	NM_030792	1.5	3.57E-03	1.5	3.65E-03	2.2	3.57E-03	1.3	9.50E-04	1.4	1.42E-02	1.4	1.42E-02	1.4	1.62E-02	1.4	1.16E-03	0.3	1.0E+00	0.3	1.0E+00	0.2	7.2E-01	0.3	1.0E+00	0.1	9.1E-01	0.2	1.0E+00	0.0	9.1E-01	0.1	8.3E-01
44135	A_23_P21882	A_23_P21882	A_23_P21882	1.5	5.15E-03	1.4	1.01E-02	2.7	5.15E-03	1.5	3.06E-03	1.0	3.24E-01	0.6	4.55E-01	1.1	3.24E-01	1.0	4.70E-02	0.1	1.0E+00	0.2	1.0E+00	0.1	9.0E-01	-0.1	1.0E+00	0.0	9.9E-01	0.3	1.0E+00	0.0	9.9E-01	-0.1	9.7E-01
43511	HCP5	A_32_P85500	NM_006674	1.5	5.70E-03	1.6	8.34E-03	1.4	5.70E-03	1.5	5.39E-05	0.8	8.12E-02	0.8	8.12E-02	0.4	5.98E-01	1.3	6.37E-03	0.1	1.0E+00	0.1	1.0E+00	0.0	9.7E-01	0.2	1.0E+00	-0.2	6.7E-01	-0.1	1.0E+00	-0.2	4.8E-01	-0.3	6.7E-01
39372	TMEM191A	A_23_P166336	AL136879	1.5	9.48E-04	1.5	9.48E-04	1.8	4.91E-03	1.5	3.25E-04	1.4	3.30E-03	1.3	3.72E-03	1.6	3.30E-03	1.4	5.46E-04	0.1	1.0E+00	0.2	1.0E+00	0.1	7.9E-01	0.0	1.0E+00	0.0	9.7E-01	0.2	1.0E+00	0.0	9.4E-01	0.0	9.7E-01
32282	BX101252	A_32_P56463	BX101252	1.5	6.47E-04	1.6	6.47E-04	1.4	2.13E-03	1.5	9.23E-06	1.4	1.78E-03	1.7	1.78E-03	1.4	1.37E-03	1.4	3.67E-03	0.1	1.0E+00	0.1	1.0E+00	0.1	7.2E-01	0.1	1.0E+00	-0.2	3.8E-01	-0.2	1.0E+00	-0.2	2.7E-01	-0.3	3.8E-01
11097	FOXA3	A_23_P208737	NM_004497	1.5	3.16E-04	1.8	3.16E-04	1.4	5.36E-04	1.5	3.90E-06	1.5	1.24E-03	1.5	3.41E-03	1.5	1.24E-03	1.9	2.51E-04	0.3	1.0E+00	0.0	1.0E+00	0.3	7.1E-01	0.3	1.0E+00	-0.1	9.6E-01	-0.1	1.0E+00	-0.2	8.1E-01	-0.1	9.6E-01
15814	GNG7	A_23_P153897	NM_052847	1.5	1.73E-04	1.5	7.74E-03	2.1	1.73E-04	1.0	3.55E-05	1.1	1.75E-02	1.0	2.28E-02	1.7	1.05E-02	1.1	1.75E-02	0.0	1.0E+00	0.0	1.0E+00	0.4	7.1E-01	-0.1	1.0E+00	0.1	9.1E-01	-0.1	1.0E+00	0.1	8.3E-01	0.1	9.1E-01
34494	PRRX2	A_23_P83298	NM_016307	1.5	2.31E-03	1.4	3.03E-03	1.8	2.31E-03	1.5	7.64E-05	1.4	4.59E-03	1.4	4.59E-03	1.6	5.33E-03	1.2	1.77E-03	0.1	1.0E+00	0.1	1.0E+00	0.1	8.4E-01	0.3	1.0E+00	-0.2	4.1E-01	-0.2	1.0E+00	-0.2	4.1E-01	-0.3	4.0E-01
43720	TMOD1	A_23_P112289	NM_003275	1.5	2.40E-04	1.5	2.40E-04	1.3	1.40E-03	1.5	6.60E-06	1.2	1.55E-02	1.2	1.55E-02	1.1	6.26E-02	1.4	2.13E-03	0.1	1.0E+00	0.1	1.0E+00	0.0	9.2E-01	0.2	1.0E+00	-0.1	9.3E-01	0.1	1.0E+00	-0.2	2.1E-01	-0.1	9.3E-01
29316	C1orf54	A_23_P74778	NM_024579	1.5	9.83E-04	1.5	9.83E-04	1.3	9.87E-03	1.6	7.19E-05	1.5	6.70E-03	1.2	2.38E-02	1.5	6.70E-03	1.5	2.21E-03	0.1	1.0E+00	0.1	1.0E+00	0.0	1.0E+00	0.2	1.0E+00	-0.1	8.7E-01	0.1	1.0E+00	-0.2	4.5E-01	-0.1	8.7E-01
5593	TUFT1	A_23_P371824	NM_020127	1.5	4.00E-04	1.4	4.00E-04	1.9	2.12E-03	1.5	7.49E-05	1.3	3.20E-03	1.3	3.20E-03	1.1	3.97E-03	1.3	8.08E-04	0.1	1.0E+00	0.1	1.0E+00	0.0	9.9E-01	0.1	1.0E+00	-0.1	9.0E-01	-0.1	1.0E+00	-0.2	4.2E-01	-0.1	9.0E-01
32912	GRASP	A_23_P105442	NM_181711	1.5	1.81E-03	1.2	5.37E-03	2.1	1.81E-03	1.5	1.05E-04	0.9	4.20E-02	0.5	1.01E-01	1.1	4.20E-02	0.9	8.50E-03	-0.1	1.0E+00	-0.1	1.0E+00	-0.1	8.2E-01	0.1	1.0E+00	-0.1	9.6E-01	0.0	1.0E+00	-0.2	7.8E-01	-0.1	9.6E-01
33499	S100A5	A_23_P115467	NM_002962	1.5	1.64E-03	1.3	3.25E-03	3.0	3.64E-04	1.5	1.64E-03	0.8	5.02E-01	0.3	8.79E-01	2.0	3.73E-01	0.8	5.02E-01	-0.2	1.0E+00	-0.2	1.0E+00	-0.2	9.1E-01	-0.6	1.0E+00	0.5	5.6E-01	0.0	1.0E+00	0.5	5.6E-01	0.7	3.9E-01
32657	MYH2	A_23_P38271	NM_017534	1.5	1.54E-03	1.0	2.10E-03	1.5	1.54E-03	1.5	6.63E-05	0.3	3.05E-01	0.2	7.04E-01	0.3	3.05E-01	0.6	5.76E-02	0.0	1.0E+00	0.0	1.0E+00	0.0	9.7E-01	0.1	1.0E+00	0.0	9.9E-01	0.0	1.0E+00	-0.2	5.0E-01	0.0	9.9E-01
26457	FCGRT	A_23_P55936	NM_004107	1.5	1.36E-03	1.5	1.36E-03	1.7	2.64E-03	1.5	5.21E-05	1.3	5.84E-03	1.2	7.92E-03	1.3	5.84E-03	1.3	2.38E-03	0.2	1.0E+00	0.2	1.0E+00	0.3	7.1E-01	0.1	1.0E+00	0.1	9.2E-01	0.1	1.0E+00	0.1	8.7E-01	0.1	9.2E-01
11263	ZAP70	A_23_P39682	NM_001079	1.5	1.12E-03	1.2	1.12E-03	1.9	1.79E-03	1.5	6.85E-05	1.4	8.46E-03	0.8	3.23E-02	1.7	8.46E-03	1.4	3.64E-03	0.1	1.0E+00	0.0	1.0E+00	0.1	9.1E-01	0.1	1.0E+00	0.0	9.9E-01	0.0	1.0E+00	-0.1	9.2E-01	0.0	9.9E-01
34879	IL10RA	A_24_P107303	NM_001558	1.5	1.73E-03	1.6	1.73E-03	1.3	2.21E-02	1.5	1.13E-03	0.8	6.17E-01	0.3	9.07E-01	0.8	6.17E-01	1.3	2.73E-01	0.2	1.0E+00	0.2	1.0E+00	0.2	9.0E-01	0.0	1.0E+00	0.4	5.9E-01	0.0	1.0E+00	0.4	5.9E-01	0.8	3.1E-01
4505	MAT1A	A_23_P23996	NM_000429	1.5	2.46E-03	1.5	2.46E-03	1.9	6.54E-03	1.2	1.82E-03	0.6	3.73E-01	0.3	7.83E-01	0.6	3.73E-01	0.9	2.66E-01	0.0	1.0E+00	-0.1	1.0E+00	0.3	7.1E-01	0.0	1.0E+00	0.3	7.6E-01	0.1	1.0E+00	0.3	4.6E-01	0.3	7.6E-01
31369	TYMP	A_23_P91802	NM_001953	1.5	2.30E-03	1.5	3.47E-03	2.5	1.64E-03	1.1	2.30E-03	1.3	1.78E-02	1.2	1.78E-02	1.8	3.92E-02	1.3	2.20E-03	0.1	1.0E+00	0.0	1.0E+00	0.1	8.4E-01	0.1	1.0E+00	0.0	1.0E+00	0.2	1.0E+00	-0.1	8.2E-01	0.0	1.0E+00
43762	CCDC136	A_23_P416178	NM_022742	1.5	3.18E-04	1.5	1.88E-04	2.0	3.18E-04	1.3	4.49E-04	1.5	1.97E-02	1.3	1.02E-01	2.3	1.97E-02	1.5	1.74E-02	0.3	1.0E+00	0.3	1.0E+00	0.1	9.4E-01	0.4	1.0E+00	-0.4	6.5E-01	-0.2	1.0E+00	-0.4	4.1E-01	-0.4	6.5E-01
6621	CAPN12	A_23_P16409	NM_144691	1.5	3.50E-04	1.5	3.50E-04	2.6	7.92E-04	1.3	2.20E-04	1.3	2.59E-02	0.9	3.42E-02	1.7	2.59E-02	1.3	2.09E-03	0.1	1.0E+00	0.2	1.0E+00	0.1	9.1E-01	-0.1	1.0E+00	0.2	9.0E-01	0.2	1.0E+00	0.1	9.0E-01	0.2	8.5E-01
38514	MPO	A_23_P141173	NM_000250	1.5	4.23E-05	1.2	6.59E-04	2.3	4.23E-05	1.5	3.93E-07	1.0	1.96E-02	0.6	1.11E-01	1.1	1.96E-02	1.0	1.20E-02	0.0	1.0E+00	0.0	1.0E+00	0.1	8.0E-01	-0.1	1.0E+00	0.0	9.5E-01	0.0	1.0E+00	0.1	6.7E-01	0.0	9.5E-01
43661	HBB	A_23_P203558	NM_000518	1.5	5.10E-03	1.3	1.08E-02	1.5	5.10E-03	1.5	8.76E-05	0.5	1.79E-01	0.3	6.00E-01	0.5	1.50E-01	0.8	1.79E-01	0.0	1.0E+00	-0.1	1.0E+00	0.0	9.6E-01	0.0	1.0E+00	-0.1	7.9E-01	0.1	1.0E+00	-0.2	2.8E-01	-0.1	7.9E-01
34006	IFI27	A_23_P48513	NM_005532	1.5	1.21E-03	1.2	6.84E-03	2.8	1.21E-03	1.5	2.77E-04	1.3	1.32E-02	1.0	1.99E-02	2.0	1.32E-02	1.3	1.09E-03	0.0	1.0E+00	-0.1	1.0E+00	0.0	9.0E-01	0.1	1.0E+00	0.0	9.9E-01	0.2	1.0E+00	0.0	9.4E-01	0.0	9.9E-01
35230	FAM105A	A_23_P133438	NM_019018	1.5	6.59E-04	1.5	6.59E-04	1.4	6.20E-03	1.6	1.49E-04	1.9	3.34E-03	1.8	9.90E-03	1.9	3.34E-03	2.1	8.67E-04	0.3	7.1E-01	0.3	1.0E+00	0.2	7.1E-01	0.5	4.8E-01	-0.3	5.2E-01	0.1	1.0E+00	-0.3	3.1E-01	-0.3	5.2E-01
5081	C11orf20	A_23_P202773	NM_001039496	1.5	1.78E-03	1.9	1.78E-03	1.5	4.20E-03	1.5	6.20E-05	1.5	2.21E-02	1.6	1.92E-03	1.0	8.21E-02	1.5	2.21E-02	-0.2	1.0E+00	0.0	1.0E+00	-0.2	7.2E-01	-0.2	1.0E+00	-0.2	8.9E-01	0.1	1.0E+00	-0.2	8.1E-01	-0.2	8.9E-01
8149	AATK	A_23_P10559	NM_001080395	1.5	4.03E-03	1.4	4.03E-03	2.1	5.51E-03	1.5	1.61E-03	1.4	1.85E-03	1.4	1.85E-03	2.0	2.83E-04	1.4	4.13E-03	0.3	1.0E+00	0.1	1.0E+00	0.3	7.1E-01	0.3	1.0E+00	0.1	9.6E-01	0.2	1.0E+00	0.0	9.4E-01	0.1	9.6E-01
40399	LBX2	A_23_P79458	NM_001009812	1.5	2.11E-03	1.5	2.50E-03	2.3	2.11E-03	1.3	1.34E-03	1.1	1.26E-01	0.8	1.55E-01	1.2	1.26E-01	1.1	4.07E-02	0.1	1.0E+00	0.0	1.0E+00	0.0	9.8E-01	0.1	1.0E+00	-0.1	9.3E-01	-0.1	1.0E+00	-0.1	9.2E-01	0.2	9.3E-01
3758	P704P	A_32_P16323	BC063888	1.5	2.82E-03	1.5	2.82E-03	1.0	4.22E-02	2.1	6.72E-05	0.7	1.68E-01	0.6	2.81E-01	0.7	1.68E-01	1.1	5.59E-02	0.2	1.0E+00	0.2	1.0E+00	0.0	9.7E-01	0.2	1.0E+00	0.1	9.7E-01	0.1	1.0E+00	0.0	9.7E-01	0.2	9.2E-01
4116	SIRPA	A_23_P210708	NM_001040022	1.5	4.28E-04	1.5	1.29E-03	1.6	4.28E-04	1.1	9.59E-06	1.0	1.81E-02	1.0	1.81E-02	1.0	7.94E-03	1.2	6.74E-02	0.1	1.0E+00	0.1	1.0E+00	-0.1	8.4E-01	0.1	1.0E+00	0.0	9.1E-01	0.0	1.0E+00	0.0	9.0E-01	-0.1	9.1E-01
19257	A_32_P25065	A_32_P25065	A_32_P25065	1.5	4.49E-03	1.3	4.49E-03	1.6	1.96E-02	1.5	7.35E-04	1.4	5.45E-03	1.4	6.40E-03	1.3	5.45E-03	1.4	1.29E-03	0.2	1.0E+00	0.1	1.0E+00	0.2	7.3E-01	0.2	1.0E+00	0.0	9.8E-01	0.3	1.0E+00	0.0	9.7E-01	0.0	9.8E-01
22511	ADAD2	A_23_P49136	NM_139174	1.5	3.23E-04	1.5	2.31E-04	2.2	3.23E-04	1.2	4.69E-03	0.1	8.78E-01	-0.3	8.78E-01	0.7	7.00E-01	0.1	9.36E-01	-0.1	1.0E+00	-0.1	1.0E+00	0.5	7.9E-01	-0.4	1.0E+00	0.6	5.5E-01	0.2	1.0E+00	0.9	9.7E-02	0.6	5.5E-01
22234	CAMK2N1	A_24_P117620	NM_018584	1.5	1.48E-02	1.4	1.48E-02	2.0	1.50E-02	1.5	2.18E-03	0.8	1.87E-01	0.7	1.87E-01	1.1	2.13E-01	0.8	9.49E-02	0.0	1.0E+00	0.2	1.0E+00	0.0	1.0E+00	-0.2	1.0E+00	0.1	9.5E-01	0.4	1.0E+00	0.1	8.8E-01	0.1	9.5E-01
22196	C10orf116	A_23_P161439	NM_006829	1.5	1.40E-03	1.4	1.40E-03	1.9	1.73E-03	1.5	1.12E-04	1.1	3.13E-02	0.9	3.51E-02	1.1	3.13E-02	1.1	1.38E-02	0.0	1.0E+00	0.2	1.0E+00	0.0	9.7E-01	-0.1	1.0E+00	0.1	9.8E-01	0.1	1.0E+00	0.1	9.2E-01	0.0	9.8E-01
3785	LOC79999	A_23_P4052	AK026312	1.5	3.24E-03	1.5	4.84E-03	2.2	3.24E-03	1.4	5.69E-04	1.2	7.70E-02	1.2	1.34E-01	1.4	7.70E-02	1.2	5.35E-02	0.0	1.0E+00	0.0	1.0E+00	0.3	7.1E-01	0.0	1.0E+00	0.3	4.8E-01	0.2	1.0E+00	0.3	4.0E-01	0.4	4.8E-01
38537	HTR7	A_23_P500381	NM_019859	1.5	1.80E-04	1.2	1.80E-04	1.6	8.85E-04	1.5	4.47E-06	1.1	6.85E-03	1.1	6.85E-03	0.9	7.36E-03	1.3	1.77E-03	0.0	1.0E+00	0.1	1.0E+00	-0.3	7.1E-01	0.0	1.0E+00	-0.3	4.9E-01	0.0	1.0E+00	-0.4	1.2E-01	-0.3	4.9E-01
21729	HRG	A_23_P321892	NM_000412	1.5	4.67E-04	1.4	4.71E-04	2.9	4.67E-04	1.5	7.40E-05	1.2	1.91E-02	0.9	1.14E-01	2.1	1.58E-02	1.2	1.91E-02	0.1	1.0E+00	0.0	1.0E+00	0.3	7.1E-01	0.1	1.0E+00	-0.1	1.0E+00	-0.1	1.0E+00	0.0	1.0E+00	-0.1	9.2E-01
22453	LRG1	A_23_P50638	NM_052972	1.5	6.14E-04	1.4	6.14E-04	2.1	1.32E-03	1.5	6.32E-05	1.0	3.86E-02	1.0	4.10E-02	1.0	2.12E-02	1.2	3.86E-02	0.0	1.0E+00	0.1	1.0E+00	0.0	9.7E-01	-0.2	1.0E+00	0.3	3.5E-01	0.1	1.0E+00	0.3	2.2E-01	0.3	3.5E-01
44406	CA12	A_23_P372234	NM_001218	1.5	1.68E-03	1.4	9.62E-03	2.1	1.68E-03	1.5	8.83E-05	1.2	6.40E-03	1.3	6.40E-03	1.0	1.42E-02	1.4	2.51E-04	0.0	1.0E+00	0.1	1.0E+00	0.0	9.6E-01	0.0	1.0E+00	-0.1	9.3E-01	0.0	1.0E+00	-0.2	4.4E-01	-0.1	9.3E-01
33792	C6orf141	A_23_P386398	BC036917	1.5	6.82E-04	1.9	6.82E-04	1.2	2.46E-03	1.5	8.17E-06	1.5	3.17E-03	1.5	2.30E-03	1.4	3.17E-03	1.6	3.22E-03	0.1	1.0E+00	0.1	1.0E+00	-0.1	8.0E-01	0.1	1.0E+00	-0.1	7.1E-01	0.1	1.0E+00	-0.1	4.3E-01	-0.1	7.1E-01
29711	AK128103	A_24_P887615	AK128103	1.5	2.52E-04	1.2	1.94E-03	1.5	2.52E-04	1.6	2.31E-07	1.1	1.66E-03	1.0	1.34E-02	1.1	1.66E-03	1.3	1.12E-03	-0.1	1.0E+00	-0.1	1.0E+00	-0.2	8.1E-01	0.0	1.0E+00	-0.1	6.9E-01	0.0	1.0E+00	-0.1	6.9E-01	-0.2	5.9E-01
1757	ENST00000358064	A_24_P261691	ENST00000358064	1.5	1.63E-04	1.4	1.63E-04	2.3	3.03E-04	1.5	6.39E-06	0.8	2.99E-01	0.8	3.05E-01	0.8	2.99E-01	1.0	6.02E-02	0.2	1.0E+00	0.1	1.0E+00	0.3	7.1E-01	0.2	1.0E+00	0.0	9.8E-01	-0.1	1.0E+00	0.0	9.8E-01	0.1	9.3E-01
11997	C19orf59	A_23_P330561	NM_174918	1.5	3.24E-04	1.2	3.53E-03	1.9	3.24E-04	1.5	4.67E-06	1.3	8.75E-03	1.4	1.14E-02	1.2	8.75E-03	1.3	2.48E-03	0.1	1.0E+00	0.0	1.0E+00	0.5	7.1E-01	0.1	1.0E+00	0.2	6.7E-01	0.0	1.0E+00	0.2	6.2E-01	0.3	6.7E-01
41284	AK023391	A_24_P918147	AK023391	1.5	2.83E-03	1.3	9.32E-03	2.0	2.83E-03	1.5	1.39E-04	1.1	1.26E-01	0.5	5.00E-01	1.5	1.26E-01	1.1	6.88E-02	0.1	1.0E+00	0.1	1.0E+00	-0.1	9.3E-01	0.2	1.0E+00	-0.2	8.4E-01	-0.1	1.0E+00	-0.3	5.6E-01	-0.2	8.4E-01
18621	HBB	A_23_P203558	NM_000518	1.5	3.22E-03	1.4	1.49E-02	1.5	3.22E-03	1.7	2.45E-05	0.8	3.66E-01	0.3	7.59E-01	0.8	1.83E-01	0.8	3.66E-01	0.0	1.0E+00	-0.1	1.0E+00	0.1	8.5E-01	0.0	1.0E+00	0.0	9.5E-01	0.1	1.0E+00	0.0	9.2E-01	-0.1	9.5E-01
14884	MIAT	A_24_P595223	NR_003491	1.5	1.38E-03	1.5	2.45E-03	1.6	1.38E-03	1.4	3.57E-05	1.3	6.15E-03	1.3	1.27E-02	1.3	6.15E-03	1.4	3.88E-03	0.0	1.0E+00	0.0	1.0E+00	-0.1	8.3E-01	0.1	1.0E+00	-0.1	8.4E-01	-0.1	1.0E+00	-0.1	6.6E-01	-0.1	8.4E-01
40877	RAC2	A_23_P218770	NM_002872	1.5	2.40E-03	1.2	2.47E-03	1.8	2.40E-03	1.5	5.03E-05	1.0	3.21E-02	0.7	2.00E-01	1.0	3.21E-02	1.1	9.89E-03	0.1	1.0E+00	0.1	1.0E+00	0.1	7.7E-01	0.0	1.0E+00	-0.1	9.1E-01	0.1	1.0E+00	-0.1	8.4E-01	-0.2	9.1E-01
32776	ZAP70	A_23_P39682	NM_001079	1.5	1.56E-03	1.2	1.56E-03	1.9	2.02E-03	1.5	4.48E-05	1.4	7.91E-03	0.7	6.34E-02	1.7	7.91E-03	1.4	3.61E-03	0.1	1.0E+00	0.1	1.0E+00	0.1	7.3E-01	0.1	1.0E+00	0.0	9.9E-01	0.0	1.0E+00	-0.1	9.2E-01	0.0	9.9E-01
45184	CYTH4	A_23_P155057	NM_013385	1.5	2.67E-04	1.5	2.67E-04	2.5	3.41E-04	1.5	1.83E-05	1.5	1.56E-03	1.2	9.24E-03	1.9	9.58E-04	1.5	1.56E-03	0.3	8.0E-01	0.3	8.0E-01	0.2	7.1E-01	0.3	1.0E+00	-0.2	7.6E-01	0.1	1.0E+00	-0.2	3.4E-01	-0.2	7.6E-01
8724	CACNB3	A_23_P204016	NM_000725	1.5	1.32E-03	1.5	1.32E-03	2.2	1.69E-03	1.1	9.78E-04	1.3	6.18E-03	1.4	6.18E-03	1.3	1.38E-02	1.3	2.14E-03	0.2	7.5E-01	0.4	7.5E-01	0.1	7.4E-01	0.2	1.0E+00	0.0	9.9E-01	0.1	1.0E+00	-0.2	5.5E-01	0.0	9.9E-01
1373	TMEM125	A_23_P115022	NM_144626	1.5	2.59E-04	1.5	2.59E-04	2.1	3.76E-04	1.5	6.39E-06	1.0	1.10E-02	1.0	1.39E-02	1.9	1.10E-02	1.0	3.45E-03	0.5	1.0E+00	0.3	1.0E+00	0.5	7.1E-01	0.5	1.0E+00	-0.1	8.1E-01	0.1	1.0E+00	-0.2	5.8E-01	-0.1	8.1E-01
35488	CDKN1A	A_23_P59210	NM_000389	1.5	3.00E-03	1.3	3.00E-03	1.9	6.37E-03	1.5	3.99E-04	1.2	1.95E-03	0.8	2.39E-02	1.2	1.65E-03	1.4	1.95E-03	0.4	1.0E+00	0.3	1.0E+00	0.4	6.7E-01	0.4	1.0E+00	-0.1	9.7E-01	-0.2	1.0E+00	-0.1	8.7E-01	0.1	9.7E-01
21369	H2AFJ	A_24_P236003	NM_177925	1.5	2.10E-04	1.5	2.10E-04	1.6	2.30E-03	1.5	8.11E-05	1.2	1.95E-02	1.2	1.95E-02	1.0	2.77E-02	1.4	7.32E-03	0.1	1.0E+00	0.1	1.0E+00	0.1	7.9E-01	0.1	1.0E+00	-0.1	9.2E-01	0.1	1.0E+00	-0.1	8.5E-01	-0.2	9.2E-01
38034	ENST00000244321	A_23_P50646	ENST00000244321	1.5	2.05E-04	1.5	9.10E-04	1.5	2.05E-04	1.6	1.49E-07	1.1	8.15E-02	0.6	8.43E-02	1.4	8.15E-02	1.1	4.22E-03	0.0	1.0E+00	0.0	1.0E+00	-0.1	8.5E-01	0.1	1.0E+00	0.0	1.0E+00	0.1	1.0E+00	-0.1	8.7E-01	0.0	1.0E+00
45163	SH2D4B	A_24_P417904	NM_207372	1.5	3.53E-04	1.0	3.53E-04	1.6	3.90E-04	1.5	2.36E-06	0.8	4.84E-02	0.3	5.41E-01	0.8	4.84E-02	0.9	1.76E-02	0.0	1.0E+00	0.0	1.0E+00	0.0	9.6E-01	-0.1	1.0E+00	0.0	9.8E-01	0.0	1.0E+00	0.0	9.8E-01	0.0	9.7E-01
21258	ZNF438	A_23_P161156	NM_182755	1.5	4.72E-03	1.4	4.72E-03	1.5	8.60E-03	1.7	7.40E-05	1.2	7.06E-03	0.7	2.24E-02	1.3	2.70E-03	1.2	7.06E-03	0.0	1.0E+00	0.0	1.0E+00	-0.1	8.3E-01	0.1	1.0E+00	-0.2	7.3E-01	-0.1	1.0E+00	-0.2	5.5E-01	-0.2	7.3E-01
33821	ZAP70	A_23_P39682	NM_001079	1.5	1.56E-03	1.1	1.97E-03	1.8	1.56E-03	1.5	2.47E-05	1.2	8.68E-03	0.6	1.02E-01	1.5	8.68E-03	1.2	7.24E-03	0.0	1.0E+00	0.0	1.0E+00	0.0	9.1E-01	0.0	1.0E+00	-0.1	9.2E-01	0.0	1.0E+00	-0.1	8.8E-01	-0.2	9.2E-01
1334	ADC	A_24_P11462	NM_052998	1.5	2.31E-03	1.5	2.31E-03	1.6	2.83E-03	1.3	1.07E-04	1.2	1.17E-02	1.2	1.35E-02	1.0	1.17E-02	1.3	5.62E-03	0.2	1.0E+00	0.1	1.0E+00	0.2	7.1E-01	0.2	1.0E+00	-0.1	9.0E-01	-0.1	1.0E+00	-0.2	4.7E-01	-0.1	9.0E-01
9695	CEBPA	A_24_P224727	NM_004364	1.5	2.61E-03	1.5	2.61E-03	0.9	1.94E-02	1.6	1.82E-05	1.2	6.11E-03	1.2	6.11E-03	1.1	1.03E-01	1.7	4.74E-04	0.2	1.0E+00	0.1	1.0E+00	0.2	7.4E-01	0.3	1.0E+00	-0.1	1.0E+00	-0.1	1.0E+00	-0.1	9.0E-01	0.0	1.0E+00
34623	HIST1H2AJ	A_24_P394510	NM_021066	1.5	8.50E-04	1.5	8.50E-04	1.6	7.98E-03	1.4	4.65E-04	1.1	6.91E-03	0.9	1.96E-02	1.1	6.91E-03	1.5	9.30E-04	-0.3	1.0E+00	-0.1	1.0E+00	-0.3	7.2E-01	-0.4	1.0E+00	0.1	9.8E-01	0.2	1.0E+00	0.0	9.7E-01	0.1	9.8E-01

Feature	Gene symbol	Probe	Transcript	72 hour								48 hours								24 hours								10 hours							
				siCASP8AP2								siCASP8AP2								siCASP8AP2								siCASP8AP2							
				Median	Median	1	1	3	3	6	6	Median	Median	1	1	3	3	6	6	Median	Median	1	1	3	3	6	6	Median	Median	1	1	3	3	6	6
				M	q	M	q	M	q	M	q	M	q	M	q	M	q	M	q	M	q	M	q	M	q	M	q	M	q	M	q	M	q	M	q
11040	CORO1A	A_23_P106761	NM_007074	1.5	3.52E-04	2.0	3.12E-04	1.5	1.34E-02	1.5	3.52E-04	2.0	2.28E-03	2.0	2.28E-03	1.6	1.44E-02	2.0	1.27E-04	0.3	1.0E+00	0.3	1.0E+00	0.3	5.7E-01	0.3	1.0E+00	0.1	9.1E-01	0.1	1.0E+00	0.1	6.7E-01	0.1	9.1E-01
17972	ZAP70	A_24_P169234	NM_001079	1.5	1.35E-03	1.0	1.35E-03	1.5	2.84E-03	1.6	1.82E-05	1.1	3.78E-02	0.8	2.09E-01	1.1	3.78E-02	1.2	3.24E-02	0.2	1.0E+00	0.1	1.0E+00	0.2	7.1E-01	0.2	1.0E+00	0.0	9.8E-01	0.0	1.0E+00	-0.1	8.7E-01	0.0	9.8E-01
18229	CAPG	A_23_P165636	NM_001747	1.5	1.03E-03	1.2	1.94E-03	2.1	1.03E-03	1.5	2.48E-05	1.1	2.95E-02	0.9	6.38E-02	1.2	2.95E-02	1.1	7.32E-03	0.1	1.0E+00	0.1	1.0E+00	0.1	8.8E-01	0.0	1.0E+00	0.0	9.9E-01	0.1	1.0E+00	0.0	9.9E-01	0.0	9.9E-01
22639	PRAP1	A_23_P202275	NM_145202	1.5	2.64E-03	1.5	2.64E-03	2.0	8.20E-03	1.5	2.60E-03	1.1	8.84E-02	0.6	1.88E-01	1.1	6.36E-02	1.1	8.84E-02	0.0	1.0E+00	0.0	1.0E+00	0.0	9.8E-01	-0.3	1.0E+00	0.2	9.4E-01	0.2	1.0E+00	0.3	6.1E-01	0.1	9.4E-01
9856	HBB	A_23_P203558	NM_000518	1.5	2.19E-03	1.4	4.34E-03	1.5	2.19E-03	1.6	1.36E-05	0.6	1.83E-01	0.4	4.70E-01	0.6	1.30E-01	0.9	1.83E-01	0.0	1.0E+00	0.0	1.0E+00	0.1	7.5E-01	-0.1	1.0E+00	0.0	9.8E-01	0.1	1.0E+00	-0.1	7.8E-01	0.0	9.8E-01
35520	SLC30A2	A_24_P156748	NM_001004434	1.5	3.65E-04	1.5	9.43E-04	2.6	3.65E-04	1.5	3.17E-05	0.9	4.54E-02	0.7	4.54E-02	1.2	5.43E-02	0.9	2.73E-03	0.1	1.0E+00	0.2	1.0E+00	0.0	9.7E-01	0.1	1.0E+00	-0.1	8.8E-01	-0.1	1.0E+00	-0.1	8.8E-01	-0.2	8.7E-01
13979	CRIP1	A_23_P44674	NM_001311	1.5	8.10E-03	1.5	8.10E-03	2.3	9.75E-03	1.5	3.51E-03	1.3	1.77E-02	1.2	1.77E-02	1.3	6.04E-02	1.4	5.76E-03	-0.1	1.0E+00	0.1	1.0E+00	-0.2	8.1E-01	-0.1	1.0E+00	-0.1	8.7E-01	0.1	1.0E+00	-0.1	8.2E-01	-0.2	8.7E-01
355	RAB3D	A_24_P236956	BC007960	1.5	1.10E-03	1.2	4.38E-03	1.9	1.10E-03	1.5	3.20E-05	1.6	4.29E-03	1.6	4.29E-03	1.8	4.85E-03	1.3	2.14E-03	0.2	1.0E+00	0.2	1.0E+00	0.0	9.8E-01	0.3	1.0E+00	-0.4	2.3E-01	-0.2	1.0E+00	-0.4	2.3E-01	-0.5	2.1E-01
32696	PAIP2B	A_24_P860797	NM_020459	1.6	9.48E-04	1.2	1.15E-03	1.9	9.48E-04	1.6	2.90E-05	1.2	1.60E-03	0.8	2.87E-02	1.5	7.48E-04	1.2	1.60E-03	0.2	1.0E+00	-0.1	1.0E+00	0.2	7.4E-01	0.3	1.0E+00	-0.1	9.7E-01	-0.1	1.0E+00	-0.2	7.7E-01	-0.1	9.7E-01
38074	LOC643201	A_32_P30891	BC052945	1.6	4.44E-04	1.4	4.44E-04	1.8	2.14E-03	1.6	9.28E-05	1.2	7.28E-02	0.2	8.31E-01	1.8	1.41E-02	1.2	7.28E-02	0.0	1.0E+00	0.0	1.0E+00	0.2	8.2E-01	-0.1	1.0E+00	0.1	9.2E-01	0.1	1.0E+00	0.2	6.5E-01	0.1	9.2E-01
10628	THC2667995	A_32_P73991	THC2667995	1.6	7.41E-04	1.6	2.82E-03	1.2	7.41E-04	1.6	2.21E-06	1.5	3.12E-02	1.4	8.62E-02	1.8	3.12E-02	1.5	1.26E-02	0.2	1.0E+00	0.1	1.0E+00	0.2	7.1E-01	0.2	1.0E+00	-0.2	8.5E-01	-0.1	1.0E+00	-0.4	2.8E-01	-0.2	8.5E-01
29839	PLEKHO2	A_23_P129246	NM_025201	1.6	3.25E-04	1.6	3.25E-04	2.0	1.56E-03	1.5	4.32E-05	1.5	3.93E-03	1.3	6.18E-03	1.5	3.93E-03	1.6	3.65E-04	0.2	1.0E+00	0.2	1.0E+00	0.1	7.1E-01	0.3	1.0E+00	0.0	9.9E-01	0.0	1.0E+00	-0.1	6.8E-01	0.0	9.9E-01
28347	GREM2	A_24_P40626	NM_022469	1.6	3.26E-03	1.6	3.26E-03	1.4	5.96E-03	2.0	1.17E-05	1.3	9.13E-03	1.3	9.13E-03	1.0	1.03E-02	1.4	2.11E-03	0.1	1.0E+00	0.1	1.0E+00	0.0	9.9E-01	0.2	1.0E+00	-0.3	5.3E-01	0.1	1.0E+00	-0.3	2.3E-01	-0.3	5.3E-01
8403	FLJ40125	A_23_P141893	NM_001080401	1.6	1.24E-04	1.5	1.24E-04	1.9	5.06E-04	1.6	6.22E-06	1.4	7.83E-03	1.4	7.83E-03	1.5	1.39E-02	1.3	7.08E-03	-0.1	1.0E+00	0.0	1.0E+00	-0.1	7.4E-01	-0.1	1.0E+00	0.0	1.0E+00	0.0	1.0E+00	0.2	8.1E-01	0.0	1.0E+00
28904	ENST00000272035	A_32_P16315	ENST00000272035	1.6	3.65E-04	1.2	8.30E-03	1.7	3.65E-04	1.6	2.56E-06	1.0	7.55E-02	1.1	7.55E-02	1.0	8.48E-02	1.0	2.44E-02	0.0	1.0E+00	0.0	1.0E+00	0.0	9.9E-01	0.2	1.0E+00	-0.1	8.4E-01	-0.1	1.0E+00	-0.1	6.1E-01	-0.1	8.4E-01
38832	C4orf34	A_23_P112634	NM_174921	1.6	1.51E-03	1.6	2.51E-03	1.6	1.51E-03	1.5	1.52E-05	1.5	1.47E-02	1.5	1.69E-02	1.5	1.47E-02	1.6	4.40E-03	0.2	1.0E+00	0.2	1.0E+00	0.2	7.7E-01	0.3	1.0E+00	-0.3	4.6E-01	-0.2	1.0E+00	-0.3	4.6E-01	-0.4	4.5E-01
29123	ULBP2	A_24_P149314	NM_025217	1.6	1.53E-03	1.6	1.53E-03	1.3	1.07E-02	1.8	4.32E-05	1.3	5.23E-02	1.3	9.44E-02	1.3	5.23E-02	1.6	1.59E-02	0.2	1.0E+00	0.2	1.0E+00	-0.1	8.2E-01	0.4	1.0E+00	0.0	9.8E-01	0.2	1.0E+00	-0.2	6.4E-01	0.0	9.8E-01
40618	CDKN1A	A_23_P59210	NM_000389	1.6	3.78E-03	1.3	3.78E-03	1.9	6.90E-03	1.6	4.01E-04	1.1	1.07E-02	0.7	6.51E-02	1.1	1.07E-02	1.2	6.39E-03	0.4	1.0E+00	0.2	1.0E+00	0.4	4.8E-01	0.4	1.0E+00	-0.2	9.8E-01	-0.3	1.0E+00	-0.2	8.3E-01	-0.1	9.8E-01
30321	HIST1H2AB	A_24_P223384	NM_003513	1.6	9.38E-05	2.3	4.20E-05	1.5	7.54E-03	1.6	9.38E-05	1.9	2.82E-03	1.9	2.82E-03	1.3	5.53E-03	2.1	1.83E-04	0.3	1.0E+00	0.3	1.0E+00	0.2	7.7E-01	0.5	1.0E+00	-0.2	9.3E-01	0.1	1.0E+00	-0.2	8.8E-01	-0.2	9.3E-01
37507	THC2732746	A_32_P220591	THC2732746	1.6	1.12E-03	1.4	1.12E-03	1.7	5.12E-03	1.6	1.43E-04	0.8	5.54E-01	0.6	7.97E-01	0.9	5.54E-01	0.8	5.22E-01	-0.1	1.0E+00	0.1	1.0E+00	-0.1	9.3E-01	-0.2	1.0E+00	-0.1	9.8E-01	-0.2	1.0E+00	0.0	9.8E-01	-0.1	9.7E-01
2985	NUPR1	A_24_P270728	NM_001042483	1.6	3.50E-04	1.6	3.50E-04	2.4	3.97E-04	1.4	3.55E-05	1.3	4.87E-03	1.3	1.40E-02	2.4	4.87E-03	1.3	4.59E-03	0.4	8.4E-01	0.3	1.0E+00	0.4	3.4E-01	0.4	8.4E-01	0.0	9.8E-01	0.0	1.0E+00	0.0	9.7E-01	0.0	9.8E-01
25398	C1orf225	A_24_P693986	NM_001013642	1.6	4.57E-03	1.6	4.57E-03	2.0	7.16E-03	1.4	8.82E-04	1.2	5.32E-02	1.2	3.97E-03	1.4	1.27E-01	1.1	5.32E-02	-0.1	1.0E+00	0.0	1.0E+00	-0.3	7.1E-01	-0.1	1.0E+00	0.0	9.8E-01	0.0	1.0E+00	0.1	9.3E-01	0.0	9.8E-01
15464	ZAP70	A_23_P39682	NM_001079	1.6	2.39E-03	1.1	2.39E-03	1.8	2.45E-03	1.6	6.63E-05	1.3	1.26E-02	0.7	8.92E-02	1.6	1.26E-02	1.3	5.77E-03	0.0	1.0E+00	-0.1	1.0E+00	0.1	8.8E-01	0.0	1.0E+00	0.0	9.8E-01	0.1	1.0E+00	0.0	9.6E-01	0.0	9.8E-01
12742	NOS3	A_23_P70849	NM_000603	1.6	2.00E-03	1.6	2.42E-03	2.9	2.00E-03	1.6	8.21E-04	0.4	7.92E-01	0.2	9.30E-01	0.4	7.92E-01	0.9	3.39E-01	-0.2	1.0E+00	-0.3	1.0E+00	0.5	7.1E-01	-0.2	1.0E+00	0.4	5.8E-01	-0.1	1.0E+00	0.8	6.8E-02	0.4	5.8E-01
21226	LRRC15	A_24_P827037	NM_130830	1.6	5.55E-04	1.6	1.63E-03	1.6	5.55E-04	1.7	7.15E-07	1.0	1.03E-02	1.0	1.03E-02	0.6	8.40E-02	1.5	1.92E-03	0.0	1.0E+00	0.0	1.0E+00	-0.1	8.2E-01	0.5	1.0E+00	0.0	1.0E+00	0.0	1.0E+00	0.0	8.9E-01	0.0	1.0E+00
3842	C11orf67	A_24_P49383	NM_024684	1.6	6.51E-03	1.6	6.51E-03	1.6	7.53E-03	1.6	2.80E-04	1.3	6.41E-03	1.1	4.45E-02	1.4	6.41E-03	1.3	2.29E-03	0.1	1.0E+00	0.2	1.0E+00	-0.1	8.7E-01	0.1	1.0E+00	-0.1	9.2E-01	0.1	1.0E+00	-0.1	7.5E-01	-0.1	9.2E-01
41134	IQCF1	A_32_P121834	NM_152397	1.6	1.09E-03	1.2	1.57E-03	2.3	1.09E-03	1.6	6.14E-05	0.8	3.17E-01	0.2	8.68E-01	0.9	3.17E-01	0.8	2.70E-01	0.0	1.0E+00	0.0	1.0E+00	0.2	7.8E-01	0.0	1.0E+00	0.1	9.4E-01	-0.1	1.0E+00	0.2	5.3E-01	0.1	9.4E-01
16934	CD83	A_23_P70670	NM_004233	1.6	3.19E-03	1.2	3.19E-03	2.0	3.37E-03	1.6	1.61E-04	0.8	9.64E-03	0.8	2.63E-02	0.8	9.64E-03	1.0	1.67E-03	0.1	1.0E+00	0.1	1.0E+00	0.0	9.8E-01	0.2	1.0E+00	0.1	9.6E-01	0.1	1.0E+00	0.1	9.0E-01	0.1	9.6E-01
20360	CD274	A_23_P338479	NM_014143	1.6	9.79E-04	1.2	9.79E-04	1.6	2.84E-03	1.6	2.90E-05	1.0	1.83E-02	0.9	2.11E-02	1.0	1.83E-02	1.1	4.12E-03	0.1	1.0E+00	0.3	1.0E+00	0.1	7.2E-01	0.0	1.0E+00	0.1	9.8E-01	0.1	1.0E+00	0.2	4.9E-01	0.0	9.8E-01
37657	LOC389634	A_32_P221305	NR_024420	1.6	2.13E-03	1.7	2.13E-03	1.6	7.58E-03	1.6	6.48E-04	1.7	3.20E-03	1.7	3.20E-03	1.6	1.59E-03	1.7	9.88E-03	0.1	1.0E+00	0.1	1.0E+00	0.0	9.5E-01	0.1	1.0E+00	0.0	9.7E-01	0.1	1.0E+00	-0.1	8.5E-01	0.0	9.7E-01
42318	PDK4	A_24_P243749	NM_002612	1.6	2.39E-02	1.0	3.91E-02	1.6	2.39E-02	1.6	6.85E-04	0.4	8.41E-01	-0.3	9.04E-01	0.4	8.41E-01	0.5	7.82E-01	0.0	1.0E+00	-0.2	1.0E+00	0.5	7.4E-01	0.0	1.0E+00	-0.1	9.4E-01	-0.3	1.0E+00	0.2	7.2E-01	-0.1	9.4E-01
11346	A_32_P103966	A_32_P103966	A_32_P103966	1.6	2.59E-04	1.5	2.74E-04	1.7	2.59E-04	1.6	2.45E-06	0.9	2.64E-02	0.9	2.64E-02	0.5	1.55E-01	1.4	2.01E-03	0.1	1.0E+00	0.0	1.0E+00	0.4	7.1E-01	0.1	1.0E+00	-0.1	8.9E-01	-0.2	1.0E+00	0.0	8.2E-01	-0.1	8.9E-01
12546	C6orf54	A_23_P93109	AB016899	1.6	3.07E-05	1.4	3.07E-05	1.8	3.07E-04	1.6	2.75E-06	1.7	2.61E-03	1.8	2.61E-03	1.7	3.27E-03	1.6	5.73E-04	0.0	1.0E+00	0.0	1.0E+00	0.0	9.2E-01	0.0	1.0E+00	-0.1	7.3E-01	-0.1	1.0E+00	-0.1	5.3E-01	-0.1	7.3E-01
9214	CEBPA	A_23_P375494	NM_004364	1.6	3.44E-03	1.6	3.44E-03	1.1	2.76E-02	1.6	9.93E-05	1.3	1.87E-03	1.1	4.97E-03	1.3	1.87E-03	1.6	2.51E-04	0.1	1.0E+00	0.1	1.0E+00	0.1	8.0E-01	0.3	1.0E+00	0.0	9.5E-01	-0.1	1.0E+00	0.0	9.5E-01	0.0	9.5E-01
34257	LYG2	A_23_P5703	NM_175735	1.6	1.27E-05	1.3	1.27E-05	2.1	6.79E-05	1.6	1.75E-06	1.3	1.36E-03	1.0	1.24E-02	1.5	1.15E-03	1.3	1.36E-03	0.1	1.0E+00	0.1	1.0E+00	0.1	8.0E-01	0.2	1.0E+00	-0.1	9.2E-01	0.0	1.0E+00	-0.1	8.6E-01	-0.1	9.2E-01
18540	MLC1	A_23_P211680	NM_015166	1.6	3.13E-04	1.6	2.22E-03	1.9	3.13E-04	1.5	9.15E-06	1.5	8.54E-03	1.5	8.54E-03	1.9	4.25E-03	1.4	1.37E-02	-0.1	1.0E+00	0.0	1.0E+00	-0.2	8.2E-01	-0.1	1.0E+00	-0.3	6.0E-01	0.0	1.0E+00	-0.4	1.9E-01	-0.3	6.0E-01
1289	FAM110C	A_32_P47643	ENST00000327669	1.6	2.29E-03	1.5	3.65E-03	1.8	2.29E-03	1.6	2.98E-04	1.5	9.25E-03	1.3	2.36E-02	1.5	9.25E-03	1.5	3.07E-03	0.5	1.0E+00	0.3	1.0E+00	0.5	7.1E-01	0.5	1.0E+00	-0.2	6.6E-01	0.0	1.0E+00	-0.2	6.6E-01	-0.3	5.8E-01
23474	ZAP70	A_23_P39682	NM_001079	1.6	2.07E-03	1.2	2.07E-03	1.8	2.11E-03	1.6	3.73E-05	1.3	1.56E-02	0.7	1.21E-01	1.6	1.56E-02	1.3	1.25E-02	0.1	1.0E+00	0.0	1.0E+00	0.1	8.6E-01	0.1	1.0E+00	-0.1	9.7E-01	0.0	1.0E+00	-0.1	9.4E-01	-0.1	9.7E-01
10759	NXF5	A_23_P114353	NM_032946	1.6	2.19E-03	2.0	4.76E-03	1.3	2.19E-03	1.6	4.48E-06	1.5	6.41E-02	1.6	6.41E-02	1.1	1.51E-01	1.5	3.42E-02	0.3	1.0E+00	0.1	1.0E+00	0.3	7.1E-01	0.3	1.0E+00	-0.1	9.2E-01	0.0	1.0E+00	-0.1	8.7E-01	-0.1	9.2E-01
38234	A_24_P229911	A_24_P229911	A_24_P229911	1.6	1.13E-03	1.6	1.13E-03	1.1	2.38E-02	1.7	6.76E-05	1.1	2.81E-02	1.1	3.46E-02	0.8	2.81E-02	1.6	2.40E-04	0.2	1.0E+00	0.2	1.0E+00	0.2	7.3E-01	0.3	1.0E+00	0.0	9.7E-01	0.0	1.0E+00	0.0	9.7E-01	0.0	9.7E-01
42023	TRPV2	A_23_P207911	NM_016113	1.6	4.79E-03	1.4	4.79E-03	2.1	6.07E-03	1.6	4.82E-04	1.0	1.23E-02	1.0	1.23E-02	1.0	1.52E-02	1.2	3.66E-03	0.1	1.0E+00	0.3	1.0E+00	0.1	7.3E-01	0.0	1.0E+00	-0.1	9.5E-01	0.0	1.0E+00	-0.3	7.7E-01	-0.1	9.5E-01
38121	CTSS	A_23_P46141	NM_004079	1.6	3.25E-04	1.6	3.25E-04	1.6	1.42E-03	2.4	7.89E-07	1.5	5.82E-03	1.3	1.09E-02	1.5	5.82E-03	1.8	4.74E-04	0.0	1.0E+00	0.0	1.0E+00	0.0	9.5E-01	0.1	1.0E+00	-0.3	6.7E-01	-0.2	1.0E+00	-0.4	1.5E-01	-0.3	6.7E-01
25257	W60781	A_23_P8812	W60781	1.6	1.43E-04	1.5	7.25E-03	1.7	1.43E-04	1.6	5.27E-07	1.1	1.50E-02	0.8	1.50E-02	1.1	4.19E-03	1.1	2.57E-02	0.0	1.0E+00	0.0	1.0E+00	0.0	9.9E-01	0.5	1.0E+00	-0.1	8.3E-01	0.1	1.0E+00	-0.1	7.4E-01	-0.1	8.3E-01
29200	BC013171	A_24_P101651	BC013171	1.6	2.59E-04	1.2	2.70E-03	1.6	2.59E-04	1.6	1.91E-06	0.7	9.01E-02	0.4	3.00E-01	0.7	9.01E-02	1.0	1.11E-02	-0.1	1.0E+00	-0.1	1.0E+00	-0.2	8.5E-01	-0.1	1.0E+00	0.0	9.3E-01	-0.1	1.0E+00	0.1	6.8E-01	0.0	9.3E-01
20103	HIST1H2AH	A_23_P81859	NM_080596	1.6	3.26E-04	1.5	3.77E-04	1.9	3.26E-04	1.6	6.85E-06	1.0	1.12E-02	0.8	1.54E-02	1.0	1.12E-02	1.0	6.57E-03	-0.2	1.0E+00	-0.1	1.0E+00	-0.2	8.8E-01	-0.3	1.0E+00	0.0	9.7E-01	0.0	1.0E+00	0.0	9.7E-01	-0.1	9.7E-01
26462	ST8SIA1	A_23_P354705	NM_003034	1.6	9.01E-04	1.1	1.46E-02	1.6	9.01E-04	1.8	1.42E-06	0.8	2.01E-02	0.8	6.33E-02	0.8	2.01E-02	1.0	6.61E-03	0.0	1.0E+00	0.0	1.0E+00	0.0	8.8E-01	0.1	1.0E+00	-0.1	6.4E-01	0.0	1.0E+00	-0.2	1.7E-01	-0.1	6.4E-01
27559	HBB	A_23_P203558	NM_000518	1.6	2.02E-02	1.5	2.02E-02	1.6	2.72E-02	1.8	6.53E-04	0.8	6.91E-02	0.7	6.91E-02	0.8	1.47E-02	1.1	1.05E-01	0.0	1.0E+00	0.1	1.0E+00	0.0	9.7E-01	0.0	1.0E+00	0.0	9.7E-01	0.1	1.0E+00	-0.1	6.9E-01	0.0	9.7E-01
42171	RASIP1	A_23_P119353	NM_017805	1.6	3.39E-03	1.5	3.39E-03	1.7	1.91E-02	1.6	1.32E-03	1.4	6.42E-03	0.8	3.20E-02	1.6	6.42E-03	1.4	2.36E-03	0.1	1.0E+00	0.1	1.0E+00	0.0	9.9E-01	0.2	1.0E+00	-0.2	9.8E-01	-0.2	1.0E+00	-0.2	8.2E-01	0.1	9.8E-01
38765	DNHD2	A_23_P372496	NM_178504	1.6	4.03E-03	1.0	4.74E-03	1.8	4.03E-03	1.6	1.08E-04	1.3	6.06E-03	0.8	5.03E-02	1.3	2.62E-03	1.3	6.06E-03	0.1	1.0E+00	0.1	1.0E+00	0.0	9.8E-01	0.2	1.0E+00	-0.2	8.8E-01	-0.2	1.0E+00	-0.2	6.2E-01	-0.2	8.8E-01
23102	A_32_P213948	A_32_P213948	A_32_P213948	1.6	3.17E-04	1.6	1.40E-03	1.8	3.17E-04	1.2	1.68E-05	1.5	4.91E-03	1.8	2.74E-03	1.5	1.17E-02	1.3	4.91E-03	0.2	1.0E+00	0.2	1.0E+00	-0.1	8.1E-01	0.2	1.0E+00	-0.1	8.3E-01	0.0	1.0E+00	-0.2	5.7E-01	-0.1	8.3E-01
35662	APOE	A_23_P164650	NM_000041	1.6	6.84E-03	1.6	2.99E-02	2.8	6.84E-03	1.6	3.37E-03	1.2	3.45E-02	0.9	4.00E-02	1.2	3.45E-02	1.3	8.02E-03	0.1	1.0E+00	0.1	1.0E+00	0.1	8.2E-01	0.1	1.0E+00	-0.1	9.1E-01	0.1	1.0E+00	-0.1	8.3E-01	-0.1	9.1E-01
15273	KIAA0513	A_23_P206310	NM_014732	1.6	6.57E-04	1.6	6.57E-04	2.1	1.11E-03	1.5	4.09E-05	1.7	3.46E-04	1.7	7.70E-04	1.5	2.83E-04	1.8	3.46E-04	0.3	1.0E+00	0.1	1.0E+00	0.3	7.1E-01	0.3	1.0E+00	-0.1	9.5E-01	-0.1	1.0E+00	-0.1	8.7E-01	-0.1	9.5E-01
13025	TNF	A_23_P376488	NM_000594	1.6	1.27E-05	1.6	1.27E-05	1.8	4.64E-04	1.6	6.21E-06	1.7	5.92E-04	1.7	5.92E-04	1.6	2.48E-04	1.8	6.27E-04	0.2	1.0E+00	0.2	1.0E+00	0.2	7.4E-01	0.1	1.0E+00	0.1	7.9E-01	0.1	1.0E+00	0.1	7.9E-01	0.2	5.5E-01
12402	LOC646241	A_32_P122128	BC028204	1.6	2.90E-03	1.6	3.51E-03	1.5	2.90E-03	1.8	1.33E-05	0.4	7.02E-01	0.4	7.95E-01	0.4	7.02E-01	0.8	4.03E-01	-0.1	1.0E+00	-0.1	1.0E+00	0.3	7.1E-01	-0.1	1.0E+00	0.1	8.5E-01	-0.1	1.0E+00	0.2	2.8E-01	0.1	8.5E-01
20538	C12orf34	A_23_P128375	NM_032829	1.6	4.90E-03	1.6	4.90E-03	1.7	2.68E-02	1.4	3.04E-03	1.4	1.03E-02	1.2	1.81E-02	1.6	8.17E-03	1.4	1.03E-02	0.1	1.0E+00	0.1	1.0E+00	0.3	7.1E-01	0.1	1.0E+00	0.3	9.1E-01	0.3	1.0E+00	0.3	6.0E-01	0.2	9.1E-01
9238	ELL2	A_23_P41645	NM_012081	1.6	2.27E-04	1.5	2.27E-04	1.6	6.45E-04	1.6	1.96E-06	1.3	1.63E-03	1.3	2.31E-03	1.4	1.63E-03	1.3	4.81E-04	0.3	1.0E+00	0.3	1.0E+00	0.3	7.1E-01	0.3	1.0E+00	0.1	9.0E-01	0.0	1.0E+00	0.1	4.4E-01	0.1	9.0E-01
31641	SGSH	A_23_P254254	NM_000199	1.6	1.72E-03	1.6	7.66E-03	2.3	1.72E-03	1.5	1.52E-04	1.1	5.05E-02	1.1	7.57E-02	1.0	5.05E-02	1.6	1.83E-02	0.1	1.0E+00	0.2	1.0E+00	0.0	9.6E-01	0.1	1.0E+00	0.0	7.6E-01	0.0	1.0E+00	-0.2	7.5E-01	0.3	7.6E-01
42907	LCK	A_23_P103361	NM_005356	1.6	1.64E-03	1.4	1.64E-03	1.6	8.49E-03	1.6	4.17E-04	1.6	3.49E-03	1.6	4.59E-03	1.5	3.49E-03	1.6	3.14E-03	0.4	1.0E+00	0.2	1.0E+00	0.4	5.0E-01	0.4	1.0E+00	0.0	1.0E+00	0.0	1.0E+00	0.0	9.2E-01	0.0	1.0E+00
33645	SPOCD1	A_23_P431388	NM_144569	1.6	1.58E-02	1.3	4.75E-02	2.3	1.58E-02	1.6	3.85E-03	1.4	5.33E-03	1.3	5.33E-03	1.8	7.97E-03	1.4	5.07E-04	0.1	1.0E+00	0.1	1.0E+00	0.0	9.2E-01	0.2	1.0E+00	0.1	1.0E+00	0.1	1.0E+00	0.1	9.1E-01	0.0	1.0E+00
22536	FAM57B	A_23_P163711	NM_031478	1.6	4.27E-04	1.7	2.21E-03	1.6	4.27E-04	1.1	8.51E-06	1.4	4.07E-02	1.5	2.13E-02	1.1	9.08E-02	1.4	4.07E-02	-0.1	1.0E+00	0.0	1.0E+00	-0.1	9.3E-01	-0.1	1.0E+00	0.1	8.5E-01	0.0	1.0E+00	0.1	8.5E-01	0.2	7.8E-01
32618	TNNI3	A_23_P67453	NM_000363	1.6	2.58E-03	1.5	2.58E-03	2.4	2.85E-03	1.6	3.25E-04	1.4	3.50E-03	1.3	9.02E-03	1.5	3.50E-03	1.4	8.27E-04	0.1	1.0E+00	0.1	1.0E+00	0.1	7.3E-01	0.1	1.0E+00	0.0	9.8E-01	0.1	1.0E+00	0.0	9.0E-01	0.0	9.8E-01
10755	LGSN	A_23_P93169	NM_016571	1.6	8.27E-04	1.6	1.10E-03	1.3	8.27E-04	2.4	3.27E-06	1.1	3.01E-03	0.8	3.52E-02	1.1	3.01E-03	1.5	1.26E-03	0.1	1.0E+00	0.1	1.0E+00	0.1	7.8E-01	0.1	1.0E+00	0.0	9.4E-01	0.0	1.0E+00	-0.1	5.8E-01	0.0	9.4E-01
27822	TNFSF9	A_24_P5856	NM_003811	1.6	2.36E-03	1.5	2.36E-03	1.6	1.81E-02	1.7	4.33E-04	1.0	1.54E-01	0.8	3.15E-01	1.0	1.54E-01	1.3	2.88E-02	0.1	1.0E+00	0.2	1.0E+00	0.0	9.9E-01	0.1	1.0E+00	0.0	9.9E-01	-0.1	1.0E+00	0.0	9.9E-01	0.0	9.8E-01
37569	AKR1B1	A_23_P258190	NM_001628	1.6	2.73E-03	1.3	2.73E-03	1.6	3.15E-03	1.8	4.59E-05	0.9	1.13E-02	0.9	1.13E-02	0.9	7.46E-02	1.4	2.62E-04	0.1	8.9E-01	0.1	1.0E+00	0.1	8.9E-01	0.3	6.9E-01	-0.2	8.4E-01	-0.2	1.0E+00	-0.3	3.0E-01	-0.1	8.4E-01
10787	ANKRD35	A_23_P325690	NM_144698	1.6	7.23E-04	1.2	2.16E-03	2.3	7.23E-04	1.6	1.84E-05	0.7	2.37E-01	0.4	6.86E-01	0.7	2.37E-01	0.7	1.82E-01	0.1	1.0E+00	-0.1	1.0E+00	0.1	7.6E-01	0.1	1.0E+00	0.1	8.0E-01	0.0	1.0E+00	0.1	5.7E-01	0.1	8.0E-01
24614	PTGS1	A_23_P216966	NM_000962	1.6	9.45E-03	1.4	2.16E-02	2.5	9.45E-03	1.6	2.25E-03	1.4	4.35E-03	1.6	4.35E-03	1.0	2.89E-02	1.4	3.10E-03	-0.1	1.0E+00	-0.1	1.0E+00	-0.2	7.1E-01	0.0	1.0E+00	-0.1	9.8E-01	0.1	1.0E+00	-0.1	9.5E-01	-0.1	9.8E-01
17345	NOS3	A_23_P70849	NM_000603	1.6	2.05E-04	1.6	2.64E-03	2.9	2.05E-04	1.6	9.76E-06	1.2	1.30E-02	0.6	1.28E-01	1.2	1.30E-02	1.8	2.09E-03	-0.1	1.0E+00	-0.1	1.0E+00	0.7	7.1E-01	-0.2	1.0E+00	0.4	6.4E-01	-0.1	1.0E+00	0.5	2.2E-01	0.4	6.4E-01
32770	COL27A1	A_23_P158096	AK021957	1.6	3.40E-04	1.6	3.40E-04	2.3	4.33E-04	1.1	1.25E-04	1.1	5.22E-02	1.1	5.73E-02	1.2	5.22E-02	1.1	4.97E-02	0.1	1.0E+00	0.1	1.0E+00	-0.1	7.7E-01	0.1	1.0E+00	0.1	9.9E-01	0.1	1.0E+00	0.0	9.9E-01	0.1	8.8E-01
35372	JUNB	A_23_P4821	NM_002229	1.6	1.98E-03	1.4	1.98E-03	1.6	8.04E-03	1.8	5.63E-04	1.5	2.93E-03	1.3	2.61E-03	1.8	7.60E-03	1.5	2.93E-03	0.1	1.0E+00	0.0	1.0E+00	0.1	8.5E-01	0.1	1.0E+00	-0.1	9.1E-01	-0.1	1.0E+00	-0.4	5.2E-01	0.1	9.1E-01
12635	PPY	A_23_P207336	NM_002722	1.6	1.17E-04	1.1	5.79E-03	3.0	1.17E-04	1.6	1.27E-05	1.0	2.42E-01	0.5	5.02E-01	1.0	2.42E-01	1.0	6.06E-02	-0.3	1.0E+00	-0.4	1.0E+00	0.5	7.1E-01	-0.3	1.0E+00	0.3	7.3E-01	0.1	1.0E+00	0.7	1.4E-02	0.3	7.3E-01
28990	FLJ45248	A_32_P228348	AK127183	1.6	7.64E-04	1.3	7.64E-04	2.3	1.14E-03	1.6	5.22E-05	1.7	7.58E-04	1.4	1.57E-03	1.9	7.58E-04	1.7	2.30E-04	0.4	3.8E-01	0.3	5.8E-01	0.4	5.0E-02	0.4	3.8E-01	-0.1	9.6E-01	-0.1	1.0E+00	-0.1	7.6E-01	-0.1	9.6E-01
27264	TMEM190	A_23_P107775	NM_139172	1.6	2.98E-03	1.6	4.51E-03	2.8	2.98E-03	1.5	1.62E-03	1.3	1.82E-01	0.7	4.48E-01	1.5	1.82E-01	1.3	7.80E-02	-0.1	1.0E+00	-0.1	1.0E+00	0.1	9.6E-01	-0.2	1.0E+00	0.3	3.9E-01	0.2	1.0E+00	0.4	1.9E-01	0.3	3.9E-01
5203	APOE	A_23_P164650	NM_000041	1.6	5.48E-03	1.6	2.68E-02	2.8	5.48E-03	1.6	2.23E-03	1.1	3.20E-02	0.9	3.20E-02	1.1	3.66E-02	1.2	1.79E-02	0.1	1.0E+00	0.1	1.0E+00	0.2	7.7E-01	0.1	1.0E+00	-0.1	8.5E-01	0.1	1.0E+00	-0.1	7.8E-01	-0.2	8.5E-01
20173	FBXO2	A_23_P45999	NM_012168	1.6	5.21E-03	1.7	5.21E-03	1.5	8.82E-03	1.6	2.08E-04	1.4	2.92E-02	1.4	2.25E-02	1.0	3.00E-02	1.4	2.92E-02	0.1	1.0E+00	0.2	1.0E+00	0.1	8.1E-01	0.1	1.0E+00	0.1	9.1E-01	0.1	1.0E+00	0.1	8.7E-01	0.1	9.1E-01
1838	LCK	A_23_P103361	NM_005356	1.6	2.10E-03	1.5	2.10E-03	1.9	9.28E-03	1.6	6.98E-04	1.7	1.08E-03	1.7	1.50E-03	1.6	1.08E-03	1.7	5.47E-04	0.4	1.0E+00	0.2	1.0E+00	0.4	7.1E-01	0.4	1.0E+00	0.0	1.0E+00	0.0	1.0E+00	0.0	1.0E+00	0.2	6.6E-01
14652	SELENBP1	A_23_P86021	NM_003944	1.6	1.27E-03	1.3	3.99E-03	1.7	1.27E-03	1.6	1.38E-05	1.4	3.37E-03	1.4	6.18E-03	1.2	3.37E-03	1.4	2.94E-03	0.2	1.0E+00	0.2	1.0E+00	0.0	9.2E-01	0.2	1.0E+00	-0.1	9.7E-01	-0.1	1.0E+00	-0.1	8.2E-01	0.0	9.7E-01
35176	ENST00000383006	A_24_P787889	ENST00000383006	1.6	1.56E-03	1.6	1.56E-03	1.9	8.84E-03	1.4	1.04E-03	1.5	1.73E-03	1.4	3.03E-03	1.7	1.73E-03	1.5	7.24E-04	0.1	1.0E+00	0.2	1.0E+00	0.1	9.2E-01	0.1	1.0E+00	0.0	9.8E-01	0.3	1.0E+00	0.0	9.8E-01	-0.1	9.1E-01
40890	NOS3	A_23_P70849	NM_000603	1.6	1.83E-04	1.5	1.83E-04	2.7	2.42E-04	1.6	2.50E-05	0.9	1.98E-01	0.1	9.00E-01	0.9	1.98E-01	1.2	6.79E-02	0.0	1.0E+00	0.0	1.0E+00	0.7	7.1E-01	-0.1	1.0E+00	0.1	9.3E-01	0.1	1.0E+00	0.5	4.1E-01	0.1	9.3E-01
27708	ATPGD1	A_23_P344884	NM_020811	1.6	5.44E-04	1.7	5.44E-04	1.6	4.20E-03	1.6	4.80E-05	1.2	1.62E-01	1.2	1.62E-01	0.8	3.27E-01	1.5	2.87E-02	0.2	8.6E-01	0.2	8.6E-01	0.2	7.1E-01	0.1	1.0E+00	0.1	8.2E-01	0.1	1.0E+00	0.1	8.2E-01	0.2	7.5E-01
23242	NCR3	A_23_P251881	NM_147130	1.6	1.41E-03	1.3	2.46E-03	2.3	1.41E-03	1.6	1.14E-04	1.2	6.46E-03	0.9	3.95E-02	1.4	2.94E-03	1.2	6.46E-03	-0.1	1.0E+00	0.0	1.0E+00	-0.1	7.7E-01	-0.1	1.0E+00	0.0	9.2E-01	0.1	1.0E+00	0.0	9.2E-01	-0.2	7.7E-01
39122	TTC9B	A_23_P332392	AK054935	1.6	1.53E-04	1.4	1.53E-04	1.7	1.44E-03	1.6	1.62E-05	0.8	4.65E-01	0.2	9.04E-01	0.8	4.65E-01	0.9	2.02E-01	0.0	1.0E+00	0.0	1.0E+00	0.2	7.3E-01	0.0	1.0E+00	0.1	7.9E-01	0.0	1.0E+00	0.1	7.9E-01	0.3	7.5E-01
38833	NOS3	A_23_P70849	NM_000603	1.6	2.22E-04	1.6	3.57E-04	2.6	2.22E-04	1.6	1.25E-05	0.6	6.32E-01	-0.1	9.74E-01	0.6	6.32E-01	0.8	4.26E-01	-0.1	1.0E+00	-0.2	1.0E+00	0.3	8.0E-01	-0.1	1.0E+00	0.2	9.2E-01	-0.1	1.0E+00	0.5	3.5E-01	0.2	9.2E-01
25644	FLJ00290	A_23_P303417	AK090404	1.6	8.27E-04	1.6	9.34E-04	1.2	8.27E-04	1.8	1.94E-07	1.2	7.33E-03	1.1	7.33E-03	1.2	1.77E-02	1.6	2.89E-04	0.1	1.0E+00	0.0	1.0E+00	0.1	7.4E-01	0.1	1.0E+00	-0.1	9.9E-01	-0.1	1.0E+00	-0.1	8.5E-01	0.0	9.9E-01
36070	DMBT1	A_23_P86599	NM_007329	1.6	1.75E-04	1.5	1.75E-04	1.6	3.52E-04	1.7	6.44E-07	1.5	3.01E-03	1.0	2.83E-02	1.8	3.01E-03	1.5	9.17E-04	0.0	1.0E+00	0.0	1.0E+00	0.0	9.5E-01	0.0	1.0E+00	-0.1	9.2E-01	-0.1	1.0E+00	-0.1	6.6E-01	0.0	9.2E-01
33974	LCK	A_23_P103361	NM_005356	1.6	1.11E-03	1.6	1.11E-03	1.8	7.27E-03	1.6	7.01E-04	1.9	1.65E-03	1.9	2.51E-03	1.8	1.65E-03	1.9	1.24E-03	0.4	1.0E+00	0.3	1.0E+00	0.4	3.7E-01	0.5	1.0E+00	0.0	9.6E-01	0.0	1.0E+00	0.0	9.6E-01	0.1	9.2E-01
29875	ATP8B3	A_23_P79108	NM_138813	1.6	1.31E-03	1.6	1.31E-03	1.6	4.89E-03	2.0	3.06E-05	1.6	2.72E-03	1.6	2.72E-03	1.5	8.50E-03	1.9	7.47E-04	0.2	1.0E+00	0.3	1.0E+00	-0.1	7.9E-01	0.2	1.0E+00	0.0	9.9E-01	0.0	1.0E+00	0.0	9.3E-01	0.0	9.9E-01
10929	CRABP2	A_23_P115064	NM_001878	1.6	1.08E-03	1.7	9.18E-04	1.6	1.45E-02	1.6	1.08E-03	1.1	4.83E-03	1.1	4.83E-03	1.0	7.87E-03	1.8	1.68E-04	0.1	1.0E+00	0.2	1.0E+00	0.1	8.6E-01	-0.1	1.0E+00	0.1	9.3E-01	0.1	1.0E+00	0.2	6.9E-01	0.1	9.3E-01
37091	CDKN1A	A_23_P59210	NM_000389	1.7	4.93E-03	1.2	4.93E-03	2.1	6.35E-03	1.7	4.50E-04	1.3	3.27E-03	0.8	3.70E-02	1.3	3.27E-03	1.4	1.69E-03	0.2	1.0E+00	0.2	1.0E+00	0.4	7.1E-01	0.2	1.0E+00	-0.1	9.2E-01	-0.2	1.0E+00	-0.1	8.4E-01	0.2	9.2E-01
35742	LOC645722	A_24_P896205	XM_944447	1.7	8.76E-04	1.4	9.51E-03	2.4	8.76E-04	1.7	3.14E-05	1.2	6.62E-03	1.2	8.31E-03	1.6	6.62E-03	1.2	1.37E-03	0.2	1.0E+00	0.2	1.0E+00	0.1	9.3E-01	0.4	1.0E+00	-0.1	9.5E-01	-0.1	1.0E+00	-0.2	7.6E-01	-0.1	9.5E-01
3233	KRT37	A_23_P118854	NM_003770	1.7	5.31E-05	1.7	5.31E-05	2.0	2.27E-04	1.5	2.36E-06	2.0	9.92E-04	2.0	9.92E-04	2.2	2.48E-04	1.6	1.71E-03	0.4	8.8E-02	0.4	8.8E-02	0.3	5.3E-01	0.5	6.2E-02	-0.1	8.8E-01	0.0	1.0E+00	-0.1	4.4E-01	-0.1	8.8E-01
31255	MX1	A_23_P17663	NM_002462	1.7	1.37E-03	1.5	1.37E-03	2.0	4.78E-03	1.7	1.88E-04	1.3	8.30E-04	1.3	3.45E-03	1.3	8.30E-04	1.5	9.99E-05	0.3	6.9E-01	0.3	6.9E-01	0.3	6.0E-01	0.4	1.0E+00	-0.2	9.5E-01	-0.2	1.0E+00	-0.2	8.6E-01	-0.1	9.5E-01
24589	A_24_P110601	A_24_P110601	A_24_P110601	1.7	4.26E-03	1.5	4.37E-03	2.6	4.26E-03	1.7	7.18E-04	1.0	1.18E-01	0.9	1.18E-01	1.4	1.66E-01	1.0	4.71E-02	0.0	1.0E+00	0.2	1.0E+00	0.0	9.8E-01	-0.2	1.0E+00	0.2	9.7E-01	0.2	1.0E+00	0.2	7.7E-01	0.1	9.7E-01
30643	GRRP1	A_23_P46131	NM_024869	1.7	3.56E-03	1.7	3.73E-03	1.8	3.56E-03	1.2	9.22E-04	1.6	4.94E-02	1.6	4.94E-02	1.7	2.34E-02	1.3	8.73E-02	0.0	1.0E+00	0.0	1.0E+00	-0.2	7.1E-01	0.1	1.0E+00	-0.1	9.4E-01	0.0	1.0E+00	-0.2	4.7E-01	-0.1	9.4E-01
12730	RAC2	A_23_P218774	NM_002872	1.7	4.00E-04	1.2	4.00E-04	1.9	6.59E-04	1.7	5.48E-06	1.3	1.24E-03	1.1	3.12E-03	1.3	1.24E-03	1.4	3.40E-04	0.2	1.0E+00	0.3	1.0E+00	0.2	7.1E-01	0.1	1.0E+00	0.0	9.8E-01	0.0	1.0E+00	-0.1	8.1E-01	0.0	9.8E-01
21044	CA11	A_23_P78782	NM_001217	1.7	5.21E-03	1.6	5.21E-03	1.9	1.18E-02	1.7	6.13E-04	1.7	1.55E-02	1.7	1.55E-02	1.3	2.15E-01	2.0	2.55E-03	0.2	1.0E+00	0.2	1.0E+00	0.2	7.4E-01	0.2	1.0E+00	-0.1	9.5E-01	0.0	1.0E+00	-0.1	9.4E-01	-0.1	9.5E-01
31402	PGA3	A_23_P150549	NM_001079807	1.7	3.50E-04	1.7	5.58E-04	1.6	3.50E-04	1.8	2.21E-07	0.6	3.81E-01	0.5	5.14E-01	0.6	3.81E-01	1.4	2.62E-02	-0.2	1.0E+00	-0.1	1.0E+00	-0.2	7.1E-01	-0.2	1.0E+00	-0.1	8.7E-01	-0.1	1.0E+00	-0.1	8.7E-01	0.4	3.9E-01
43394	SH2D4B	A_24_P273489	NM_207372	1.7	1.47E-04	1.3	1.47E-04	1.7	3.73E-04	1.7	1.82E-06	1.1	7.78E-02	0.5	4.40E-01	1.1	7.78E-02	1.1	2.17E-02	0.0	1.0E+00	0.0	1.0E+00	0.2	7.2E-01	0.0	1.0E+00	0.0	9.9E-01	0.0	1.0E+00	0.0	9.9E-01	0.1	9.3E-01
37354	NPAS1	A_23_P27734	NM_002517	1.7	1.23E-02	1.7	1.85E-02	2.9	1.23E-02	1.5	9.85E-03	1.7	2.95E-02	1.2	2.98E-02	1.9	2.95E-02	1.7	2.81E-03	0.0	1.0E+00	0.1	1.0E+00	0.0	9.7E-01	0.0	1.0E+00	0.0	1.0E+00	0.3	1.0E+00	-0.1	9.4E-01	0.0	1.0E+00
14316	ZAP70	A_23_P39682	NM_001079	1.7	1.48E-03	1.2	1.48E-03	1.9	2.00E-03	1.7	4.11E-05	1.4	8.99E-03	0.8	5.66E-02	1.7	8.99E-03	1.4	4.94E-03	0.0	1.0E+00	0.0	1.0E+00	0.1	8.9E-01	0.0	1.0E+00	-0.1	9.7E-01	0.0	1.0E+00	-0.1	9.4E-01	-0.1	9.7E-01
33009	APOE	A_23_P164650	NM_000041	1.7	4.56E-03	1.7	1.58E-02	3.1	4.56E-03	1.6	2.37E-03	1.3	3.76E-02	1.0	3.76E-02	1.3	5.84E-02	1.4	1.51E-02	0.1	1.0E+00	0.1	1.0E+00	0.1	8.6E-01	0.0	1.0E+00	0.1	9.5E-01	0.2	1.0E+00	-0.1	9.5E-01	0.1	9.5E-01
17497	PHLDA2	A_23_P47614	NM_003311	1.7	8.78E-03	1.2	8.78E-03	1.7	1.41E-02	1.7	4.79E-04	1.1	1.84E-02	1.1	2.39E-02	1.1	1.84E-02	1.0	1.47E-02	0.1	1.0E+00	0.1	1.0E+00	0.2	7.3E-01	0.1	1.0E+00	0.2	8.1E-01	0.1	1.0E+00	0.2	7.2E-01	0.2	8.1E-01
35398	ENST00000332750	A_23_P349147	ENST00000332750	1.7	4.71E-04	1.7	4.71E-04	1.9	5.33E-03	1.5	2.93E-04	1.8	1.72E-02	1.5	6.00E-02	2.0	1.72E-02	1.8	6.32E-03	0.1	1.0E+00	0.2	1.0E+00	-0.1	8.3E-01	0.1	1.0E+00	0.2	7.9E-01	0.2	1.0E+00	-0.1	7.9E-01	0.2	7.5E-01
23951	RHOV	A_23_P117912	ENST00000220507	1.7	1.25E-04	1.7	1.25E-04	2.0	1.28E-03	1.4	5.19E-05	1.6	1.28E-03	1.6	2.61E-03	1.5	1.28E-03	1.6	2.46E-04	0.4	1.0E+00	0.2	1.0E+00	0.4	7.1E-01	0.4	1.0E+00	0.2	5.4E-01	0.1	1.0E+00	0.2	5.4E-01	0.3	4.2E-01
34715	DES	A_23_P90710	NM_001927	1.7	5.90E-04	1.7	6.79E-03	3.2	5.90E-04	1.5	2.77E-04	1.6	3.04E-02	1.3	9.78E-02	2.2	3.04E-02	1.6	1.26E-02	0.0	1.0E+00	0.1	1.0E+00	-0.1	8.4E-01	0.0	1.0E+00	-0.1	8.5E-01	0.1	1.0E+00	-0.2	4.7E-01	-0.1	8.5E-01
40162	C8G	A_23_P20713	NM_000606	1.7	4.46E-04	1.7	4.46E-04	2.1	1.26E-03	1.6	3.71E-05	1.7	1.12E-03	1.6	4.54E-03	1.7	1.12E-03	1.8	5.86E-04	0.1	1.0E+00	0.1	1.0E+00	0.0	9.9E-01	0.3	1.0E+00	-0.2	7.6E-01	0.1	1.0E+00	-0.2	3.7E-01	-0.2	7.6E-01
30249	SMPD3	A_23_P163567	NM_018667	1.7	1.73E-03	2.7	1.73E-03	1.7	1.53E-02	1.7	4.97E-04	2.3	2.50E-03	3.0	1.58E-03	2.1	2.50E-03	2.3	1.67E-02	0.1	1.0E+00	0.1	1.0E+00	0.0	9.3E-01	0.2	1.0E+00	-0.1	9.6E-01	0.0	1.0E+00	-0.1	9.3E-01	-0.1	9.6E-01
14026	HSPA2	A_23_P88303	NM_021979	1.7	4.01E-03	1.5	4.01E-03	1.8	6.53E-03	1.7	6.54E-04	1.2	1.85E-02	1.0	1.16E-01	1.2	1.60E-02	1.4	1.85E-02	0.0	1.0E+00	0.1	1.0E+00	-0.1	8.7E-01	0.0	1.0E+00	-0.1	9.7E-01	-0.1	1.0E+00	-0.1	8.8E-01	0.1	9.7E-01
14377	APOE	A_23_P164650	NM_000041	1.7	3.32E-03	1.6	1.58E-02	3.0	3.32E-03	1.7	1.07E-03	1.3	2.08E-02	1.0	2.08E-02	1.3	3.35E-02	1.3	1.39E-02	0.1	1.0E+00	0.1	1.0E+00	0.1	8.0E-01	0.0	1.0E+00	-0.1	9.6E-01	0.2	1.0E+00	-0.1	9.3E-01	-0.1	9.6E-01

Feature	Gene symbol	Probe	Transcript	72 hour siCASP8AP2								48 hours siCASP8AP2								24 hours siCASP8AP2								10 hours siCASP8AP2							
				Median M	Median q	1 M	1 q	3 M	3 q	6 M	6 q	Median M	Median q	1 M	1 q	3 M	3 q	6 M	6 q	Median M	Median q	1 M	1 q	3 M	3 q	6 M	6 q	Median M	Median q	1 M	1 q	3 M	3 q	6 M	6 q
36026	CDKN1A	A_23_P59210	NM_000389	1.7	2.46E-03	1.4	2.46E-03	2.0	5.66E-03	1.7	2.60E-04	1.2	2.32E-03	0.7	2.82E-02	1.2	2.32E-03	1.3	1.94E-03	0.4	1.0E+00	0.3	1.0E+00	0.4	5.0E-01	0.4	1.0E+00	-0.1	1.0E+00	-0.3	1.0E+00	-0.1	8.8E-01	0.0	1.0E+00
30072	PAEP	A_23_P257129	NM_002571	1.7	9.38E-04	1.4	4.01E-03	3.1	9.38E-04	1.7	1.99E-04	1.6	7.69E-04	1.0	2.32E-02	1.6	7.69E-04	1.6	4.59E-04	-0.1	1.0E+00	0.0	1.0E+00	-0.1	7.1E-01	-0.1	1.0E+00	0.0	9.8E-01	0.1	1.0E+00	-0.2	2.8E-01	0.0	9.8E-01
23413	AK123333	A_32_P53234	AK123333	1.7	9.36E-04	1.7	2.76E-03	1.7	9.36E-04	1.5	8.50E-06	1.0	1.34E-01	0.6	3.28E-01	1.0	1.34E-01	1.1	3.68E-02	0.0	1.0E+00	0.1	1.0E+00	0.0	9.5E-01	0.0	1.0E+00	0.1	9.5E-01	0.1	1.0E+00	0.2	6.3E-01	-0.1	9.5E-01
33180	NOS3	A_23_P70849	NM_000603	1.7	3.03E-04	1.7	3.25E-04	2.9	3.03E-04	1.7	3.11E-05	1.2	1.10E-01	0.5	6.84E-01	1.2	1.10E-01	1.5	3.59E-02	0.1	1.0E+00	0.1	1.0E+00	0.1	8.9E-01	0.0	1.0E+00	0.1	8.6E-01	0.0	1.0E+00	0.1	8.6E-01	0.3	6.3E-01
26574	ZAP70	A_23_P39682	NM_001079	1.7	1.81E-03	1.2	1.94E-03	2.0	1.81E-03	1.7	3.29E-05	1.3	1.86E-02	0.7	1.37E-01	1.6	1.86E-02	1.3	9.68E-03	0.0	1.0E+00	0.0	1.0E+00	0.1	8.5E-01	0.0	1.0E+00	0.0	9.7E-01	0.0	1.0E+00	0.0	9.7E-01	-0.1	9.7E-01
14968	APOE	A_23_P164650	NM_000041	1.7	4.30E-03	1.7	1.64E-02	2.9	4.30E-03	1.7	1.36E-03	1.3	2.10E-02	1.0	2.10E-02	1.3	3.65E-02	1.3	1.50E-02	0.0	1.0E+00	0.0	1.0E+00	0.1	8.0E-01	0.0	1.0E+00	0.0	9.7E-01	0.2	1.0E+00	0.0	9.4E-01	-0.1	9.7E-01
43207	HIST1H2AJ	A_23_P168014	NM_021066	1.7	8.50E-04	1.7	8.50E-04	1.7	6.53E-03	1.5	4.44E-04	1.2	3.60E-02	1.0	4.55E-02	1.2	3.60E-02	1.5	3.22E-03	-0.3	1.0E+00	0.0	1.0E+00	-0.3	7.6E-01	-0.5	1.0E+00	0.0	1.0E+00	0.1	1.0E+00	-0.1	9.6E-01	0.0	1.0E+00
23598	WBSCR28	A_23_P418274	NM_182504	1.7	8.06E-05	1.5	5.48E-04	2.3	8.06E-05	1.7	8.19E-07	1.2	1.24E-02	1.2	3.48E-02	1.1	1.24E-02	1.3	2.16E-03	0.1	1.0E+00	0.1	1.0E+00	0.0	9.8E-01	0.1	1.0E+00	-0.2	8.9E-01	-0.1	1.0E+00	-0.2	6.8E-01	-0.2	8.9E-01
29055	LCK	A_23_P103361	NM_005356	1.7	6.09E-04	1.6	6.09E-04	1.9	3.63E-03	1.7	1.63E-04	1.8	1.28E-03	1.8	1.66E-03	1.7	1.28E-03	1.9	9.30E-04	0.2	1.0E+00	0.1	1.0E+00	0.3	7.1E-01	0.2	1.0E+00	0.0	9.9E-01	0.0	1.0E+00	0.0	9.9E-01	0.3	6.5E-01
41625	CR608961	A_32_P163392	CR608961	1.7	7.85E-04	1.7	9.25E-04	2.3	7.85E-04	1.5	5.05E-05	1.3	5.84E-03	1.2	2.98E-02	1.5	3.88E-03	1.3	5.84E-03	0.1	1.0E+00	0.1	1.0E+00	0.2	7.1E-01	0.1	1.0E+00	-0.3	7.8E-01	0.1	1.0E+00	-0.4	3.3E-01	-0.3	7.8E-01
14346	A_24_P845631	A_24_P845631	A_24_P845631	1.7	1.27E-03	1.3	6.50E-03	2.5	1.27E-03	1.7	7.26E-05	1.2	1.45E-02	1.2	5.54E-03	1.5	2.02E-02	1.0	1.45E-02	0.2	1.0E+00	0.1	1.0E+00	0.2	7.2E-01	0.4	1.0E+00	-0.2	9.2E-01	-0.1	1.0E+00	-0.2	6.5E-01	-0.2	9.2E-01
39833	CST6	A_23_P146946	NM_001323	1.7	1.74E-02	1.1	4.20E-02	2.3	1.74E-02	1.7	2.84E-03	0.7	8.70E-02	0.6	8.70E-02	1.0	1.51E-01	0.7	1.20E-02	0.0	1.0E+00	0.2	1.0E+00	0.0	1.0E+00	0.0	1.0E+00	0.0	9.9E-01	0.2	1.0E+00	0.0	9.9E-01	0.0	9.8E-01
13340	THC2668815	A_24_P478940	THC2668815	1.7	3.04E-04	1.3	4.00E-04	2.6	3.04E-04	1.7	1.12E-05	1.2	3.72E-03	1.2	4.41E-03	1.9	3.68E-03	1.2	3.72E-03	0.0	1.0E+00	0.1	1.0E+00	0.0	9.9E-01	-0.1	1.0E+00	-0.1	8.4E-01	0.0	1.0E+00	-0.1	7.2E-01	-0.2	8.4E-01
19209	NOS3	A_23_P70849	NM_000603	1.7	3.34E-03	1.6	3.93E-03	2.9	3.34E-03	1.7	1.19E-03	0.6	6.44E-01	0.2	9.26E-01	0.6	6.44E-01	0.9	3.54E-01	-0.2	1.0E+00	-0.2	1.0E+00	0.5	7.1E-01	-0.2	1.0E+00	0.4	3.9E-01	-0.1	1.0E+00	0.7	6.5E-02	0.4	3.9E-01
13069	OR7E91P	A_23_P433218	NR_002185	1.7	5.69E-04	1.5	5.69E-04	2.0	1.99E-03	1.7	7.38E-05	0.4	8.27E-01	0.3	9.28E-01	0.4	8.27E-01	0.9	5.49E-01	-0.1	1.0E+00	-0.1	1.0E+00	0.7	7.1E-01	-0.1	1.0E+00	0.5	1.2E-01	0.1	1.0E+00	0.6	1.4E-02	0.5	1.2E-01
18776	LCK	A_23_P103361	NM_005356	1.7	9.25E-04	1.6	9.25E-04	1.9	4.78E-03	1.7	2.52E-04	1.8	6.46E-04	1.8	6.46E-04	1.7	8.59E-04	1.8	4.16E-04	0.3	1.0E+00	0.2	1.0E+00	0.3	7.1E-01	0.4	1.0E+00	0.1	7.8E-01	0.1	1.0E+00	0.1	7.8E-01	0.2	5.5E-01
15126	LCK	A_23_P103361	NM_005356	1.7	1.32E-03	1.5	1.32E-03	1.9	5.40E-03	1.7	2.53E-04	1.8	8.74E-04	1.8	8.74E-04	1.6	1.30E-03	1.8	4.87E-04	0.3	1.0E+00	0.2	1.0E+00	0.4	4.7E-01	0.3	1.0E+00	0.1	7.2E-01	0.1	1.0E+00	0.1	7.2E-01	0.2	6.4E-01
24907	HIST1H2AK	A_24_P217848	NM_003510	1.7	4.54E-04	1.7	4.54E-04	2.0	1.32E-03	1.6	3.72E-05	1.2	2.72E-03	1.1	2.72E-03	1.2	2.73E-03	1.3	1.25E-03	0.0	1.0E+00	0.1	1.0E+00	0.0	9.7E-01	0.0	1.0E+00	-0.1	9.6E-01	0.0	1.0E+00	-0.1	8.8E-01	-0.1	9.6E-01
19749	ZNF554	A_23_P343250	NM_001102651	1.7	5.89E-04	1.6	8.42E-04	2.7	5.89E-04	1.7	3.37E-05	1.5	1.17E-03	1.3	2.72E-03	1.7	6.82E-04	1.5	1.17E-03	0.2	1.0E+00	0.2	1.0E+00	0.2	7.1E-01	0.2	1.0E+00	0.0	1.0E+00	0.1	1.0E+00	0.0	9.8E-01	0.0	1.0E+00
3817	APOE	A_23_P164650	NM_000041	1.7	4.20E-03	1.6	1.53E-02	3.0	4.20E-03	1.7	1.49E-03	1.2	5.05E-02	0.9	5.05E-02	1.2	9.90E-02	1.3	2.34E-02	0.1	1.0E+00	0.1	1.0E+00	0.1	8.5E-01	-0.1	1.0E+00	0.1	9.8E-01	0.2	1.0E+00	0.0	9.8E-01	0.1	9.6E-01
9760	SCN4B	A_23_P303833	NM_174934	1.7	2.91E-04	1.7	1.43E-03	2.2	2.91E-04	1.7	3.04E-06	1.2	5.26E-02	1.3	5.26E-02	0.9	1.10E-01	1.2	3.92E-02	0.2	1.0E+00	0.1	1.0E+00	0.2	7.1E-01	0.2	1.0E+00	-0.2	7.7E-01	-0.1	1.0E+00	-0.2	5.2E-01	-0.2	7.7E-01
17649	HBB	A_23_P203558	NM_000518	1.7	1.08E-03	1.7	1.08E-03	1.6	6.08E-03	1.9	4.26E-05	0.6	6.78E-01	0.2	9.41E-01	0.6	6.78E-01	0.7	6.37E-01	0.1	1.0E+00	0.1	1.0E+00	0.1	7.4E-01	0.0	1.0E+00	0.0	9.7E-01	0.1	1.0E+00	-0.1	6.7E-01	0.0	9.7E-01
21780	HBB	A_23_P203558	NM_000518	1.7	3.51E-03	1.6	5.07E-03	1.7	3.51E-03	1.8	2.97E-05	1.0	1.07E-01	0.7	1.16E-01	1.0	1.39E-02	1.1	1.07E-01	0.0	1.0E+00	0.1	1.0E+00	-0.1	7.6E-01	0.0	1.0E+00	-0.1	9.5E-01	0.1	1.0E+00	-0.1	6.7E-01	-0.1	9.5E-01
32540	LCK	A_23_P103361	NM_005356	1.7	5.67E-04	1.6	5.67E-04	1.8	4.26E-03	1.7	2.64E-04	1.8	1.25E-03	1.8	1.56E-03	1.8	1.25E-03	1.8	1.16E-03	0.3	1.0E+00	0.2	1.0E+00	0.3	6.6E-01	0.4	1.0E+00	0.0	9.8E-01	0.0	1.0E+00	0.0	9.8E-01	0.1	8.7E-01
185	CCNA1	A_23_P48414	NM_003914	1.7	1.90E-03	1.7	4.29E-03	1.0	1.90E-03	2.5	4.35E-07	0.6	1.84E-02	0.6	1.84E-02	0.5	1.81E-01	1.2	3.56E-03	0.0	1.0E+00	0.1	1.0E+00	0.0	1.0E+00	0.0	1.0E+00	0.0	9.4E-01	0.0	1.0E+00	-0.1	6.8E-01	0.0	9.4E-01
41106	TDRD7	A_23_P123672	NM_014290	1.7	1.55E-05	1.6	1.55E-05	1.9	3.12E-04	1.7	2.78E-06	1.4	3.91E-03	1.4	3.91E-03	1.3	4.86E-03	1.4	1.16E-03	0.6	3.4E-01	0.6	3.4E-01	0.6	3.4E-01	0.6	9.7E-01	-0.1	7.7E-01	0.2	1.0E+00	-0.1	7.7E-01	-0.2	6.3E-01
42924	THC2577566	A_32_P44932	THC2577566	1.7	2.43E-02	1.7	3.74E-02	2.4	2.43E-02	1.7	5.08E-03	1.4	7.16E-03	1.0	9.19E-03	1.7	7.16E-03	1.4	2.32E-03	0.3	1.0E+00	0.0	1.0E+00	0.3	7.1E-01	0.4	1.0E+00	-0.5	8.3E-01	-0.2	1.0E+00	-0.5	6.6E-01	-0.6	8.3E-01
32819	CDKN1A	A_23_P59210	NM_000389	1.7	6.73E-03	1.3	6.73E-03	2.2	1.02E-02	1.7	7.82E-04	1.3	2.27E-03	0.8	2.63E-02	1.3	2.27E-03	1.4	1.40E-03	0.3	1.0E+00	0.2	1.0E+00	0.4	6.6E-01	0.3	1.0E+00	0.0	9.7E-01	-0.1	1.0E+00	0.0	9.7E-01	0.2	8.8E-01
30575	APOE	A_23_P164650	NM_000041	1.7	4.09E-03	1.7	1.57E-02	3.0	4.09E-03	1.7	1.34E-03	1.3	2.79E-02	1.0	2.79E-02	1.3	3.84E-02	1.4	1.25E-02	0.2	1.0E+00	0.2	1.0E+00	0.2	7.7E-01	0.1	1.0E+00	0.0	1.0E+00	0.1	1.0E+00	-0.1	9.4E-01	0.0	1.0E+00
9685	SERPINB9	A_24_P295010	NM_004155	1.7	3.92E-03	1.1	6.85E-03	1.9	3.92E-03	1.7	7.67E-05	1.1	1.26E-02	1.1	5.36E-02	1.3	1.26E-02	1.1	8.04E-03	0.4	1.0E+00	0.2	1.0E+00	0.5	7.1E-01	0.4	1.0E+00	-0.2	7.8E-01	-0.1	1.0E+00	-0.2	7.5E-01	-0.2	7.8E-01
16051	APOE	A_23_P164650	NM_000041	1.7	4.33E-03	1.7	1.42E-02	3.0	4.33E-03	1.7	1.47E-03	1.2	3.10E-02	1.0	3.10E-02	1.2	5.55E-02	1.3	1.92E-02	0.1	1.0E+00	0.1	1.0E+00	0.1	8.4E-01	-0.1	1.0E+00	0.0	1.0E+00	0.2	1.0E+00	0.0	9.9E-01	0.0	1.0E+00
10089	KLK11	A_23_P101505	NM_144947	1.7	2.00E-03	1.4	7.68E-03	2.2	2.00E-03	1.7	7.26E-05	1.1	2.71E-03	1.1	9.36E-03	1.0	2.71E-03	1.4	8.36E-04	0.1	1.0E+00	0.1	1.0E+00	0.0	9.8E-01	0.1	1.0E+00	-0.1	9.5E-01	0.1	1.0E+00	-0.2	7.0E-01	-0.1	9.5E-01
17652	C1QTNF2	A_23_P92899	NM_031908	1.7	3.58E-05	1.5	3.58E-05	1.7	5.57E-04	1.8	2.52E-06	1.6	9.16E-04	1.5	1.94E-03	1.6	9.16E-04	1.7	1.27E-04	0.2	1.0E+00	0.1	1.0E+00	0.2	7.1E-01	0.2	1.0E+00	0.1	9.6E-01	0.1	1.0E+00	0.1	8.6E-01	0.0	9.6E-01
2202	THC2682291	A_32_P90346	THC2682291	1.7	5.20E-04	1.6	5.20E-04	2.5	1.69E-03	1.7	1.08E-04	1.6	6.69E-04	1.6	2.00E-03	1.9	5.31E-04	1.6	6.69E-04	0.5	7.1E-01	0.5	5.1E-01	0.3	7.1E-01	0.5	1.0E+00	-0.4	7.6E-01	-0.2	1.0E+00	-0.4	5.1E-01	-0.4	7.6E-01
35850	CTSL2	A_23_P146456	NM_001333	1.7	3.15E-04	1.7	3.15E-04	1.4	3.10E-03	1.8	5.49E-06	1.2	8.40E-03	1.2	3.92E-03	1.0	8.40E-03	1.4	1.46E-02	0.1	1.0E+00	0.1	1.0E+00	-0.1	8.5E-01	0.1	1.0E+00	-0.2	6.8E-01	-0.1	1.0E+00	-0.2	6.6E-01	-0.3	6.8E-01
8933	VAV1	A_23_P38959	NM_005428	1.7	2.29E-04	1.7	8.23E-04	2.7	2.29E-04	1.7	1.14E-05	1.7	3.55E-03	1.4	2.52E-02	1.7	3.55E-03	1.7	1.70E-03	0.1	1.0E+00	0.1	1.0E+00	0.0	9.2E-01	0.4	1.0E+00	-0.1	7.9E-01	0.0	1.0E+00	-0.2	5.8E-01	-0.1	7.9E-01
28177	NR4A1	A_23_P128230	NM_002135	1.7	7.23E-03	1.2	7.23E-03	1.7	1.16E-02	1.9	1.74E-04	1.0	1.65E-02	1.0	1.65E-02	1.1	2.95E-02	1.0	3.73E-03	0.1	1.0E+00	0.1	1.0E+00	0.1	8.4E-01	0.3	1.0E+00	0.1	9.4E-01	-0.1	1.0E+00	0.1	8.2E-01	0.1	9.4E-01
19318	WDR45	A_23_P251717	NM_007075	1.7	4.17E-04	1.7	4.17E-04	1.4	3.03E-03	1.9	5.69E-06	1.3	7.64E-03	1.3	7.64E-03	0.9	1.94E-02	1.7	2.72E-04	0.3	8.5E-01	0.3	8.5E-01	0.1	7.1E-01	0.3	1.0E+00	0.1	9.7E-01	0.1	1.0E+00	0.0	9.7E-01	0.1	9.3E-01
6639	THC2651085	A_23_P256487	THC2651085	1.7	7.18E-04	1.7	7.18E-04	1.7	5.96E-03	2.0	4.52E-05	1.1	3.80E-01	1.1	3.80E-01	0.7	5.71E-01	1.5	6.74E-02	0.4	1.0E+00	0.1	1.0E+00	0.6	7.1E-01	0.4	1.0E+00	0.2	5.6E-01	0.0	1.0E+00	0.2	5.6E-01	0.3	4.9E-01
35234	NOS3	A_23_P70849	NM_000603	1.7	3.07E-04	1.7	3.71E-04	2.8	3.07E-04	1.7	2.33E-05	1.2	1.03E-01	0.5	6.22E-01	1.2	1.03E-01	1.5	3.51E-02	0.1	1.0E+00	0.1	1.0E+00	0.2	7.9E-01	0.1	1.0E+00	0.2	8.7E-01	-0.2	1.0E+00	0.2	8.4E-01	0.2	8.7E-01
9997	SILV	A_23_P312851	NM_006928	1.7	1.12E-03	1.7	1.48E-03	1.9	1.12E-03	1.7	8.50E-06	1.5	4.63E-03	1.5	5.75E-03	1.2	4.63E-03	1.9	1.17E-03	0.1	1.0E+00	0.2	1.0E+00	0.0	9.2E-01	0.1	1.0E+00	0.1	9.2E-01	0.1	1.0E+00	0.0	8.4E-01	0.1	9.2E-01
36020	CRYM	A_23_P77731	NM_001888	1.7	2.11E-03	1.7	2.11E-03	1.9	3.37E-03	1.7	9.05E-05	1.1	2.65E-02	1.0	8.62E-02	1.1	2.65E-02	1.3	1.84E-02	0.0	1.0E+00	0.0	1.0E+00	0.3	7.6E-01	0.0	1.0E+00	-0.2	9.3E-01	-0.2	1.0E+00	-0.1	9.3E-01	-0.3	8.3E-01
15901	IL2RB	A_24_P203000	NM_000878	1.7	2.27E-04	1.3	1.19E-03	2.0	2.27E-04	1.7	1.43E-06	1.0	1.72E-03	0.9	5.01E-03	1.1	1.72E-03	1.0	4.78E-04	0.3	1.0E+00	0.0	1.0E+00	0.4	7.1E-01	0.3	1.0E+00	0.0	9.8E-01	-0.1	1.0E+00	0.0	9.8E-01	0.0	9.8E-01
34273	HRK	A_23_P25194	NM_003806	1.7	7.56E-04	1.6	5.47E-03	3.0	7.56E-04	1.7	6.55E-05	1.2	3.83E-02	0.9	3.70E-02	1.8	4.58E-02	1.2	3.83E-02	0.1	1.0E+00	0.0	1.0E+00	0.1	8.7E-01	0.1	1.0E+00	-0.3	8.6E-01	-0.1	1.0E+00	-0.4	5.6E-01	-0.3	8.6E-01
3131	PTPRH	A_23_P101642	NM_002842	1.7	4.78E-03	1.7	4.78E-03	2.3	1.09E-02	1.7	1.66E-03	1.4	8.17E-03	1.2	9.51E-03	1.4	8.17E-03	1.4	1.70E-03	0.3	1.0E+00	0.3	1.0E+00	0.3	7.1E-01	0.2	1.0E+00	-0.2	1.0E+00	-0.2	1.0E+00	-0.2	8.2E-01	0.0	1.0E+00
41182	PDCD4	A_23_P258862	NM_145341	1.7	7.22E-04	1.6	1.77E-03	3.0	7.22E-04	1.7	8.84E-05	1.4	2.61E-02	1.4	3.59E-02	1.8	2.61E-02	1.3	1.16E-02	0.1	1.0E+00	-0.1	1.0E+00	0.1	9.1E-01	0.1	1.0E+00	-0.3	6.9E-01	-0.3	1.0E+00	-0.3	6.5E-01	-0.4	6.9E-01
26050	AL832451	A_24_P36898	AL832451	1.7	6.21E-04	1.1	9.83E-04	1.7	6.21E-04	2.5	2.21E-07	1.2	3.58E-02	0.6	3.58E-02	1.2	3.79E-02	1.3	5.46E-04	0.1	1.0E+00	0.1	1.0E+00	0.0	9.3E-01	0.1	1.0E+00	0.0	9.7E-01	0.4	1.0E+00	0.0	9.7E-01	-0.1	8.9E-01
20547	VGF	A_24_P129326	NM_003378	1.7	5.84E-03	2.2	5.84E-03	1.7	4.23E-02	1.5	5.70E-03	1.4	7.54E-02	1.3	4.91E-02	1.4	2.07E-01	1.6	7.54E-02	0.1	1.0E+00	0.1	1.0E+00	0.2	8.2E-01	-0.2	1.0E+00	0.2	8.8E-01	0.2	1.0E+00	0.3	6.2E-01	0.2	8.8E-01
41995	BST2	A_23_P39465	NM_004335	1.7	2.22E-04	1.6	3.51E-04	2.6	2.22E-04	1.7	3.25E-05	1.7	3.61E-03	1.6	3.45E-03	1.7	4.61E-03	1.7	3.61E-03	0.1	1.0E+00	0.2	1.0E+00	0.1	8.9E-01	0.1	1.0E+00	-0.2	8.5E-01	-0.1	1.0E+00	-0.2	7.0E-01	-0.2	8.5E-01
10827	CA13	A_23_P381714	NM_198584	1.7	6.95E-04	1.3	6.95E-04	1.8	2.56E-03	1.7	3.75E-05	1.7	1.94E-03	1.7	1.94E-03	1.7	2.37E-03	1.7	1.33E-03	0.3	1.0E+00	0.1	1.0E+00	0.3	7.1E-01	0.4	1.0E+00	-0.3	3.6E-01	-0.2	1.0E+00	-0.3	3.0E-01	-0.3	3.6E-01
36490	LILRP2	A_23_P27781	NR_003061	1.8	8.72E-05	1.8	8.72E-05	1.7	1.20E-03	1.8	3.28E-06	0.9	8.30E-02	0.4	6.34E-01	0.9	8.30E-02	1.2	2.69E-02	0.1	1.0E+00	0.1	1.0E+00	0.0	9.6E-01	0.1	1.0E+00	0.1	9.9E-01	0.3	1.0E+00	0.1	9.2E-01	0.0	9.9E-01
4693	TCEB3C	A_23_P315910	NM_145653	1.8	2.22E-04	1.1	1.11E-02	1.8	2.22E-04	2.6	8.92E-07	0.5	5.36E-02	0.1	7.11E-01	0.5	5.36E-02	0.9	1.55E-02	0.0	1.0E+00	0.0	1.0E+00	-0.1	8.3E-01	0.0	1.0E+00	0.0	9.7E-01	0.0	1.0E+00	0.0	9.7E-01	-0.1	7.9E-01
24115	HBB	A_23_P203558	NM_000518	1.8	3.63E-03	1.6	6.44E-03	1.8	3.63E-03	1.8	3.78E-05	1.0	7.88E-02	0.6	1.08E-01	1.0	4.33E-03	1.2	7.88E-02	0.0	1.0E+00	-0.1	1.0E+00	0.1	9.2E-01	0.0	1.0E+00	0.0	9.7E-01	0.1	1.0E+00	-0.1	6.8E-01	0.0	9.7E-01
37272	CXorf27	A_32_P90859	NM_012274	1.8	2.22E-04	1.5	1.28E-03	1.8	2.22E-04	2.4	7.22E-08	1.4	1.36E-03	0.9	2.15E-02	1.4	1.36E-03	1.4	1.56E-04	0.0	1.0E+00	0.0	1.0E+00	0.0	9.2E-01	0.1	1.0E+00	0.0	9.4E-01	0.1	1.0E+00	0.0	8.8E-01	0.0	9.4E-01
2780	ENST00000375672	A_24_P101800	ENST00000375672	1.8	7.48E-04	0.9	2.76E-03	1.8	7.48E-04	1.8	2.20E-06	1.3	5.71E-03	1.3	8.20E-03	1.4	5.71E-03	1.3	2.36E-03	0.2	1.0E+00	0.3	1.0E+00	0.0	9.5E-01	0.2	1.0E+00	-0.1	7.8E-01	0.0	1.0E+00	-0.2	3.2E-01	-0.1	7.8E-01
10053	RGS2	A_23_P114947	NM_002923	1.8	2.87E-03	1.8	2.87E-03	1.3	1.23E-02	1.9	2.51E-05	1.5	1.56E-02	1.5	1.56E-02	1.4	4.28E-02	1.8	1.56E-03	0.3	1.0E+00	0.0	1.0E+00	0.3	7.1E-01	0.3	1.0E+00	-0.1	8.4E-01	0.0	1.0E+00	-0.1	7.5E-01	-0.1	8.4E-01
32406	SNIP	A_24_P57977	NM_025248	1.8	1.01E-03	1.8	4.10E-03	2.9	1.01E-03	1.5	2.92E-04	1.7	2.01E-02	1.7	1.20E-02	2.2	6.06E-02	1.4	2.01E-02	0.2	1.0E+00	0.2	1.0E+00	-0.1	8.2E-01	0.2	1.0E+00	-0.1	9.1E-01	-0.1	1.0E+00	-0.3	3.7E-01	-0.1	9.1E-01
34942	HCST	A_23_P39386	NM_014266	1.8	1.15E-03	1.5	1.15E-03	2.7	1.15E-03	1.8	8.68E-05	1.2	1.41E-01	0.6	5.85E-01	1.6	1.41E-01	1.2	8.15E-02	-0.1	1.0E+00	-0.1	1.0E+00	0.2	7.6E-01	-0.1	1.0E+00	0.2	8.9E-01	0.2	1.0E+00	0.2	6.6E-01	0.2	8.9E-01
31162	ZNF404	A_23_P90333	NM_001033719	1.8	4.82E-04	0.9	6.14E-04	1.8	4.82E-04	2.1	5.32E-07	0.9	1.83E-02	0.9	1.83E-02	0.7	1.29E-01	1.3	2.08E-03	0.3	8.0E-01	0.3	1.0E+00	0.1	8.0E-01	0.5	6.0E-01	-0.1	8.5E-01	-0.1	1.0E+00	-0.1	6.9E-01	-0.2	8.5E-01
3236	PODNL1	A_23_P433798	NM_024825	1.8	2.31E-04	2.2	1.52E-03	1.7	2.31E-04	1.8	3.09E-06	2.0	2.00E-03	2.3	3.12E-03	1.6	2.00E-03	2.0	1.68E-04	0.2	1.0E+00	0.2	1.0E+00	0.0	9.9E-01	0.2	1.0E+00	-0.2	7.1E-01	-0.1	1.0E+00	-0.2	3.1E-01	-0.2	7.1E-01
23782	CDKN1A	A_23_P59210	NM_000389	1.8	1.67E-03	1.4	1.67E-03	2.1	4.25E-03	1.8	1.48E-04	1.3	1.28E-03	0.9	1.12E-02	1.3	1.28E-03	1.4	6.27E-04	0.3	1.0E+00	0.3	1.0E+00	0.4	5.2E-01	0.2	1.0E+00	0.0	9.9E-01	-0.1	1.0E+00	0.0	9.9E-01	0.1	9.5E-01
25284	ZAP70	A_23_P39682	NM_001079	1.8	1.84E-03	1.2	1.84E-03	2.0	1.93E-03	1.8	2.99E-05	1.3	1.99E-02	0.7	2.04E-01	1.6	1.99E-02	1.3	1.33E-02	0.0	1.0E+00	0.0	1.0E+00	0.0	9.2E-01	0.0	1.0E+00	0.0	9.9E-01	0.1	1.0E+00	0.0	9.9E-01	0.0	9.9E-01
32230	PDCD1	A_23_P136405	NM_005018	1.8	2.33E-03	1.4	2.33E-03	2.3	3.10E-03	1.8	1.74E-04	1.2	1.92E-02	0.6	3.04E-01	1.4	1.92E-02	1.2	1.74E-02	0.1	1.0E+00	0.1	1.0E+00	0.4	7.0E-01	0.1	1.0E+00	0.2	7.8E-01	0.1	1.0E+00	0.2	6.0E-01	0.2	7.8E-01
34328	LOC650293	A_32_P352358	NM_001040071	1.8	1.26E-03	1.4	1.26E-03	2.0	1.63E-03	1.8	3.35E-05	1.5	7.97E-03	1.3	8.24E-03	1.5	7.97E-03	1.8	2.62E-04	0.0	1.0E+00	0.0	1.0E+00	0.0	9.4E-01	0.3	1.0E+00	-0.2	6.9E-01	0.0	1.0E+00	-0.2	3.8E-01	-0.2	6.9E-01
20112	TNNT1	A_23_P56050	NM_001126132	1.8	5.73E-05	1.7	5.73E-05	2.4	3.14E-04	1.8	5.68E-06	1.4	6.42E-03	1.4	1.62E-02	1.6	6.42E-03	1.4	4.81E-03	0.1	1.0E+00	0.1	1.0E+00	0.2	7.8E-01	0.1	1.0E+00	0.2	8.4E-01	0.2	1.0E+00	0.2	7.6E-01	0.2	8.4E-01
1164	NXF2	A_23_P148568	NM_017809	1.8	2.83E-03	1.9	3.52E-03	1.2	2.83E-03	1.8	9.15E-06	1.5	1.16E-02	1.5	9.12E-03	1.2	2.09E-02	1.5	1.16E-02	0.2	1.0E+00	0.1	1.0E+00	0.2	7.1E-01	0.2	1.0E+00	-0.1	9.3E-01	0.0	1.0E+00	-0.1	6.3E-01	-0.1	9.3E-01
27312	KRTAP19-1	A_24_P367247	NM_181607	1.8	3.53E-03	1.8	3.53E-03	1.5	4.91E-03	1.8	2.47E-05	1.1	8.40E-02	0.7	3.19E-01	1.1	3.37E-02	1.2	8.40E-02	-0.1	1.0E+00	-0.1	1.0E+00	-0.2	7.1E-01	0.0	1.0E+00	0.1	9.3E-01	0.1	1.0E+00	0.2	6.3E-01	-0.1	9.3E-01
23040	NOS3	A_23_P70849	NM_000603	1.8	3.14E-04	1.8	3.14E-04	3.0	3.18E-04	1.7	2.46E-05	1.3	3.63E-02	0.8	2.14E-01	1.3	3.63E-02	1.5	1.45E-02	-0.2	1.0E+00	-0.2	1.0E+00	0.3	7.1E-01	-0.2	1.0E+00	0.4	5.0E-01	-0.1	1.0E+00	0.4	2.1E-01	0.4	5.0E-01
38503	DPYSL4	A_23_P331049	NM_006426	1.8	1.80E-04	1.7	1.80E-04	2.1	8.43E-04	1.8	1.24E-05	1.7	6.06E-04	1.6	2.38E-03	1.8	6.06E-04	1.7	1.83E-04	0.3	7.1E-01	0.3	4.8E-01	0.1	7.1E-01	0.3	1.0E+00	-0.2	3.8E-01	0.0	1.0E+00	-0.2	3.8E-01	-0.3	3.3E-01
22689	DAPL1	A_23_P165598	NM_001017920	1.8	8.45E-03	1.3	2.44E-02	1.8	8.45E-03	1.8	1.84E-04	1.4	3.50E-03	1.2	2.36E-02	1.4	3.50E-03	1.7	2.30E-03	0.0	1.0E+00	0.0	1.0E+00	-0.2	7.3E-01	0.1	1.0E+00	-0.2	4.3E-01	0.1	1.0E+00	-0.3	6.0E-02	-0.2	4.3E-01
19757	ZAP70	A_23_P39682	NM_001079	1.8	1.38E-03	1.3	1.38E-03	2.0	1.95E-03	1.8	2.92E-05	1.2	1.86E-02	0.7	1.58E-01	1.6	1.86E-02	1.2	1.22E-02	0.0	1.0E+00	0.0	1.0E+00	0.1	8.1E-01	0.0	1.0E+00	0.0	9.6E-01	0.1	1.0E+00	0.0	9.6E-01	-0.1	9.5E-01
25546	CCDC153	A_24_P450285	NM_001033658	1.8	9.02E-03	1.8	9.20E-03	2.3	9.02E-03	1.8	7.68E-04	1.5	3.56E-02	1.5	2.69E-02	1.4	4.76E-02	1.5	3.56E-02	0.0	1.0E+00	0.1	1.0E+00	-0.2	8.0E-01	0.0	1.0E+00	0.2	8.8E-01	0.3	1.0E+00	0.2	7.6E-01	0.2	8.8E-01
27972	TMEM158	A_23_P369899	NM_015444	1.8	1.06E-02	1.6	1.06E-02	2.2	2.18E-02	1.8	5.93E-03	1.9	5.01E-04	2.0	6.35E-04	1.8	5.01E-04	1.9	3.14E-04	0.0	1.0E+00	0.2	1.0E+00	-0.2	8.3E-01	0.0	1.0E+00	0.1	9.7E-01	0.1	1.0E+00	0.1	9.4E-01	-0.1	9.7E-01
11951	hCG_1818231	A_32_P52153	XR_042007	1.8	7.04E-04	1.8	5.33E-03	3.4	7.04E-04	1.8	2.02E-04	1.4	1.63E-02	1.4	3.48E-03	2.1	2.12E-02	1.3	1.63E-02	0.1	1.0E+00	0.0	1.0E+00	0.1	9.2E-01	0.3	1.0E+00	-0.4	8.7E-01	-0.2	1.0E+00	-0.5	5.8E-01	-0.4	8.7E-01
28423	AV698803	A_32_P98776	AV698803	1.8	2.29E-04	1.5	3.37E-04	2.4	2.29E-04	1.8	3.77E-06	1.1	2.73E-01	0.6	6.16E-01	1.1	2.73E-01	1.1	1.55E-01	0.0	1.0E+00	0.2	1.0E+00	-0.2	7.4E-01	0.0	1.0E+00	0.1	9.9E-01	0.2	1.0E+00	0.1	9.0E-01	0.0	9.9E-01
26881	APOE	A_23_P164650	NM_000041	1.8	3.64E-03	1.7	1.40E-02	3.1	3.64E-03	1.8	9.92E-04	1.3	4.12E-02	1.0	4.12E-02	1.3	5.69E-02	1.3	2.12E-02	0.1	1.0E+00	0.1	1.0E+00	0.1	8.4E-01	0.0	1.0E+00	0.0	9.9E-01	0.2	1.0E+00	0.0	9.9E-01	0.0	9.8E-01
41916	AK055306	A_24_P117368	AK055306	1.8	3.37E-03	1.3	1.90E-02	3.0	3.37E-03	1.8	7.10E-04	0.5	1.92E-01	-0.1	8.75E-01	0.5	1.92E-01	1.1	1.06E-02	-0.1	1.0E+00	-0.1	1.0E+00	0.4	7.1E-01	-0.1	1.0E+00	0.1	9.5E-01	-0.1	1.0E+00	0.1	8.8E-01	0.1	9.5E-01
15125	RNF122	A_23_P134744	NM_024787	1.8	5.00E-04	1.8	5.00E-04	2.5	7.77E-04	1.6	1.24E-04	1.3	1.10E-01	1.3	9.07E-02	1.6	1.10E-01	1.2	1.17E-01	-0.2	1.0E+00	-0.2	1.0E+00	0.1	9.0E-01	-0.2	1.0E+00	0.0	9.8E-01	0.0	1.0E+00	0.1	9.0E-01	0.0	9.8E-01
29934	PMCH	A_23_P321223	NM_002674	1.8	2.22E-03	2.1	2.22E-03	1.0	4.79E-03	1.8	6.25E-06	1.6	4.04E-03	1.5	1.00E-02	1.8	3.81E-03	1.6	4.04E-03	0.0	1.0E+00	0.0	1.0E+00	-0.1	7.4E-01	0.1	1.0E+00	-0.2	5.6E-01	0.0	1.0E+00	-0.2	2.6E-01	-0.2	5.6E-01
22238	RAB31	A_24_P236799	NM_006868	1.8	7.64E-04	1.5	1.26E-03	1.9	7.64E-04	1.8	1.36E-05	1.1	2.55E-02	1.1	2.16E-02	0.6	1.41E-01	1.2	2.55E-02	0.1	1.0E+00	0.2	1.0E+00	0.1	9.1E-01	0.1	1.0E+00	-0.1	9.1E-01	0.0	1.0E+00	-0.1	9.1E-01	-0.2	8.7E-01
8425	ADRB2	A_23_P145024	NM_000024	1.8	6.62E-03	1.6	1.05E-02	1.8	6.62E-03	2.0	1.07E-04	1.1	3.50E-02	0.8	1.92E-01	1.1	3.50E-02	1.4	1.74E-02	0.2	1.0E+00	0.2	1.0E+00	-0.1	9.0E-01	0.2	1.0E+00	-0.1	9.0E-01	0.1	1.0E+00	-0.1	7.1E-01	-0.1	9.0E-01
18699	TCL1B	A_23_P48495	NM_199206	1.8	3.60E-05	1.7	2.23E-03	2.4	3.60E-05	1.8	9.56E-08	0.7	3.20E-01	0.2	8.61E-01	0.7	3.20E-01	0.7	2.81E-01	0.1	1.0E+00	0.0	1.0E+00	0.1	8.9E-01	0.2	1.0E+00	0.2	7.4E-01	-0.1	1.0E+00	0.2	2.7E-01	0.2	7.4E-01
9791	AQP1	A_23_P372834	NM_198098	1.8	3.17E-04	1.3	6.78E-03	2.1	3.17E-04	1.8	3.77E-06	1.4	1.08E-02	1.3	2.35E-02	1.9	1.08E-02	1.4	1.30E-03	0.2	1.0E+00	0.2	1.0E+00	0.1	9.3E-01	0.4	1.0E+00	-0.2	6.4E-01	-0.1	1.0E+00	-0.3	2.8E-01	-0.2	6.4E-01
26633	PARP16	A_23_P163278	NM_017851	1.8	4.03E-04	1.6	4.03E-04	1.9	1.09E-03	1.8	7.61E-06	1.2	7.15E-03	1.2	7.15E-03	1.2	2.44E-02	1.6	6.22E-04	0.0	1.0E+00	0.0	1.0E+00	0.0	9.8E-01	0.0	1.0E+00	0.0	9.5E-01	0.0	1.0E+00	0.1	7.9E-01	-0.1	9.5E-01
43485	FER1L4	A_23_P80048	NR_024377	1.8	1.43E-04	1.8	2.78E-04	2.5	1.43E-04	1.7	1.16E-06	1.2	6.87E-02	1.2	1.08E-01	1.2	6.87E-02	1.3	3.98E-02	0.2	1.0E+00	0.2	1.0E+00	0.4	7.1E-01	0.2	1.0E+00	0.1	9.1E-01	0.0	1.0E+00	0.1	9.1E-01	0.2	6.7E-01
14234	MAN1C1	A_23_P103601	NM_020379	1.8	7.41E-04	1.9	7.41E-04	1.6	1.71E-02	1.8	2.15E-04	1.8	1.42E-03	1.8	1.63E-03	1.6	3.05E-04	1.9	1.42E-03	0.4	1.0E+00	0.4	1.0E+00	0.5	5.2E-01	0.4	1.0E+00	0.1	9.8E-01	0.2	1.0E+00	0.1	8.7E-01	0.0	9.8E-01
22587	ABCA13	A_23_P364324	NM_152701	1.8	2.52E-04	1.3	2.59E-02	2.0	2.52E-04	1.8	4.10E-06	0.7	2.10E-02	0.7	5.00E-02	0.7	2.10E-02	1.1	1.93E-02	0.0	1.0E+00	0.0	1.0E+00	0.0	9.7E-01	0.1	1.0E+00	-0.2	8.1E-01	0.2	1.0E+00	-0.2	6.8E-01	-0.2	8.1E-01
26805	RAB31	A_23_P141688	NM_006868	1.8	1.14E-03	1.3	2.50E-03	1.8	1.14E-03	1.8	7.98E-06	1.0	1.78E-02	1.0	1.94E-02	1.0	1.78E-02	1.3	1.25E-02	0.1	9.8E-01	0.3	4.1E-01	0.0	9.8E-01	0.1	1.0E+00	0.0	9.8E-01	0.0	1.0E+00	0.0	9.8E-01	0.1	9.5E-01
12085	CDH15	A_32_P25357	NM_004933	1.8	2.22E-04	1.8	2.89E-03	2.7	2.22E-04	1.6	1.21E-05	1.5	1.03E-02	1.5	1.03E-02	2.0	2.95E-02	1.4	6.52E-03	0.2	1.0E+00	0.2	1.0E+00	0.2	7.1E-01	0.3	1.0E+00	-0.2	9.1E-01	-0.2	1.0E+00	-0.2	4.8E-01	-0.1	9.1E-01
10656	ASNS	A_32_P232747	BC030024	1.8	7.92E-04	1.6	2.16E-03	2.2	7.92E-04	1.8	1.34E-05	1.4	6.70E-03	1.4	1.31E-02	1.4	6.70E-03	1.8	1.82E-03	0.1	1.0E+00	0.1	1.0E+00	0.1	9.0E-01	0.1	1.0E+00	-0.1	8.4E-01	0.1	1.0E+00	-0.1	7.9E-01	-0.1	8.4E-01
756	IL10RA	A_23_P203173	NM_001558	1.8	3.58E-05	1.8	3.58E-05	1.7	8.50E-04	2.1	2.98E-06	1.2	9.90E-03	1.2	9.90E-03	0.9	2.07E-02	1.8	1.50E-03	0.2	1.0E+00	0.2	1.0E+00	0.2	7.1E-01	0.4	1.0E+00	-0.1	9.4E-01	-0.1	1.0E+00	-0.2	6.6E-01	-0.1	9.4E-01
25584	CACNA1G	A_23_P107247	NM_018896	1.8	3.17E-04	1.8	3.17E-04	2.4	4.36E-04	1.8	8.70E-06	1.3	5.23E-02	1.1	1.53E-01	1.3	5.23E-02	1.3	3.25E-02	0.1	1.0E+00	0.0	1.0E+00	0.1	7.9E-01	0.1	1.0E+00	0.1	9.8E-01	0.1	1.0E+00	0.0	9.8E-01	0.1	9.0E-01
1929	RPL3L	A_23_P118095	NM_005061	1.8	2.23E-02	1.5	4.08E-02	2.7	2.23E-02	1.8	7.94E-03	0.4	8.57E-01	0.2	9.50E-01	0.4	8.57E-01	0.4	8.41E-01	-0.3	1.0E+00	-0.4	1.0E+00	0.7	7.1E-01	-0.3	1.0E+00	1.0	1.7E-01	0.4	1.0E+00	1.0	6.2E-02	1.1	1.7E-01
9183	AK026750	A_24_P589028	AK026750	1.8	1.10E-03	1.8	1.52E-03	1.8	1.10E-03	1.9	2.78E-06	1.7	7.42E-04	1.8	2.61E-03	1.5	7.42E-04	1.7	3.14E-04	0.1	1.0E+00	0.1	1.0E+00	0.0	9.6E-01	0.2	1.0E+00	-0.3	2.4E-01	-0.1	1.0E+00	-0.3	9.3E-02	-0.3	2.4E-01
5358	ADRB2	A_23_P145024	NM_000024	1.8	8.62E-03	1.6	1.36E-02	1.8	8.62E-03	2.0	1.34E-04	1.2	2.15E-02	0.8	1.77E-01	1.2	2.15E-02	1.4	1.29E-02	0.1	1.0E+00	0.1	1.0E+00	0.0	9.4E-01	0.3	1.0E+00	-0.1	8.8E-01	0.0	1.0E+00	-0.2	5.9E-01	-0.1	8.8E-01
16712	MC1R	A_23_P329271	NM_002386	1.9	2.78E-04	1.9	2.78E-04	2.1	6.65E-04	1.6	1.22E-05	1.8	6.53E-03	1.7	6.53E-03	1.8	7.48E-03	1.9	9.56E-04	0.1	1.0E+00	0.1	1.0E+00	0.1	7.5E-01	0.2	1.0E+00	0.1	1.0E+00	0.1	1.0E+00	0.1	8.3E-01	0.0	1.0E+00
4039	ULBP2	A_23_P145485	NM_025217	1.9	3.65E-04	1.9	3.65E-04	1.8	5.22E-03	2.0	3.60E-05	1.5	1.53E-02	1.5	2.32E-02	1.3	1.53E-02	1.9	3.71E-03	0.2	1.0E+00	0.2	1.0E+00	-0.1	8.6E-01	0.3	1.0E+00	0.0	9.5E-01	0.0	1.0E+00	-0.2	5.0E-01	0.0	9.5E-01
15181	CDKN1A	A_23_P59210	NM_000389	1.9	2.93E-03	1.4	2.93E-03	2.1	6.52E-03	1.9	2.34E-04	1.2	3.42E-03	0.9	2.28E-02	1.2	3.42E-03	1.4	1.35E-03	0.3	1.0E+00	0.2	1.0E+00	0.4	5.0E-01	0.3	1.0E+00	0.0	1.0E+00	-0.1	1.0E+00	0.0	1.0E+00	0.1	9.5E-01
23830	LOC90835	A_24_P842002	NM_001014979	1.9	2.01E-04	1.8	2.01E-04	1.9	8.58E-04	1.9	4.67E-06	1.3	1.02E-02	1.3	1.02E-02	1.5	1.16E-02	1.3	8.50E-03	0.0	1.0E+00	0.1	1.0E+00	0.0	9.7E-01	0.0	1.0E+00	0.1	9.5E-01	0.1	1.0E+00	0.1	7.6E-01	0.1	9.5E-01
19055	FAM110C	A_24_P50248	NM_001077710	1.9	4.68E-05	1.6	4.68E-05	2.6	9.04E-05	1.9	1.42E-06	1.4	2.69E-03	1.0	2.86E-02	1.4	2.69E-03	1.6	2.07E-03	0.1	1.0E+00	0.1	1.0E+00	-0.1	7.5E-01	0.1	1.0E+00	-0.1	8.6E-01	0.1	1.0E+00	-0.1	4.3E-01	-0.1	8.6E-01
30377	SALL4	A_23_P109072	NM_020436	1.9	4.00E-04	1.5	4.00E-04	2.6	7.92E-04	1.9	2.09E-05	1.5	3.28E-03	1.1	4.33E-02	1.7	3.28E-03	1.5	2.43E-03	0.3	9.4E-01	0.3	1.0E+00	0.0	9.2E-01	0.5	9.4E-01	-0.2	9.6E-01	-0.2	1.0E+00	-0.2	5.3E-01	-0.1	9.6E-01
37760	DLX4	A_23_P164196	NM_138281	1.9	9.08E-04	1.8	9.08E-04	2.2	4.13E-03	1.9	2.14E-04	2.0	4.04E-03	1.6	2.44E-02	2.3	1.72E-03	2.0	4.04E-03	0.2	1.0E+00	0.2	1.0E+00	0.2	8.0E-01	0.2	1.0E+00	-0.1	9.7E-01	-0.1	1.0E+00	-0.4	5.7E-01	-0.1	9.7E-01
36561	CYP24A1	A_24_P356930	NM_000782	1.9	2.19E-03	1.9	2.19E-03	0.6	4.11E-02	1.9	2.81E-06	2.0	2.57E-02	2.0	2.57E-02	1.0	1.34E-01	2.0	1.53E-03	0.6	7.1E-01	0.7	5.2E-01	0.4	7.1E-01	0.6	1.0E+00	0.0	9.9E-01	0.2	1.0E+00	0.0	9.9E-01	0.0	9.9E-01
27910	PGM2L1	A_23_P396765	NM_173582	1.9	3.01E-03	0.9	2.90E-02	2.3	3.01E-03	1.9	2.43E-04	0.7	6.88E-03	0.0	9.44E-01	1.2	8.51E-04	0.7	6.88E-03	0.1	1.0E+00	0.1	1.0E+00	0.0	9.9E-01	0.1	1.0E+00	0.0	9.8E-01	0.0	1.0E+00	0.0	9.8E-01	0.2	9.1E-01
27626	ENST00000390541	A_24_P109921	ENST00000390541	1.9	5.38E-03	1.9	5.38E-03	2.7	5.63E-03	1.5	2.20E-03	1.3	4.79E-02	1.1	4.79E-02	1.3	1.95E-01	1.3	1.06E-02	0.5	7.1E-01	0.5	6.8E-01	0.2	7.1E-01	0.6	1.0E+00	-0.2	9.7E-01	-0.3	1.0E+00	-0.2	8.9E-01	-0.1	9.7E-01
41539	FBP1	A_23_P257111	NM_000507	1.9	5.35E-04	1.0	4.69E-03	1.9	5.35E-04	1.9	1.75E-06	1.0	2.35E-02	0.8	1.58E-01	1.1	2.35E-02	1.0	2.12E-02	0.0	1.0E+00	0.1	1.0E+00	-0.1	7.6E-01	0.0	1.0E+00	-0.3	5.3E-01	-0.1	1.0E+00	-0.3	3.1E-01	-0.4	5.3E-01
31479	HCP5	A_24_P17870	NM_006674	1.9	1.50E-03	1.9	1.50E-03	1.6	1.82E-03	1.9	1.33E-05	0.9	3.94E-01	0.9	3.94E-01	0.6	5.61E-01	1.4	1.02E-01	0.1	1.0E+00	0.2	1.0E+00	0.0	9.9E-01	0.1	1.0E+00	0.0	9.6E-01	0.0	1.0E+00	0.0	9.6E-01	0.4	3.1E-01
9039	ADRB2	A_23_P145024	NM_000024	1.9	9.30E-03	1.7	1.02E-02	1.9	9.30E-03	1.9	1.62E-04	1.3	2.51E-02	0.8	1.59E-01	1.3	2.51E-02	1.5	1.26E-02	0.2	1.0E+00	0.2	1.0E+00	-0.1	9.3E-01	0.2	1.0E+00	-0.2	7.3E-01	0.0	1.0E+00	-0.2	6.6E-01	-0.2	7.3E-01

Feature	Gene symbol	Probe	Transcript	72 hour								48 hours								24 hours								10 hours							
				siCASP8AP2								siCASP8AP2								siCASP8AP2								siCASP8AP2							
				Median M	Median q	1 M	1 q	3 M	3 q	6 M	6 q	Median M	Median q	1 M	1 q	3 M	3 q	6 M	6 q	Median M	Median q	1 M	1 q	3 M	3 q	6 M	6 q	Median M	Median q	1 M	1 q	3 M	3 q	6 M	6 q
14273	HIST2H2BE	A_23_P149545	NM_003528	1.9	5.02E-04	2.0	5.02E-04	1.3	1.29E-03	1.9	9.33E-07	1.9	3.39E-03	1.9	3.39E-03	1.3	2.91E-01	2.0	1.45E-04	1.0	2.3E-02	0.8	4.6E-02	1.0	1.2E-03	1.0	2.3E-02	0.0	9.6E-01	0.0	1.0E+00	-0.1	7.9E-01	0.0	9.6E-01
6497	A_32_P117908	A_32_P117908	A_32_P117908	1.9	5.01E-04	1.9	5.01E-04	2.1	8.35E-04	1.7	1.12E-05	1.9	8.67E-03	1.9	8.67E-03	2.0	1.10E-02	1.7	2.30E-03	0.1	1.0E+00	0.1	1.0E+00	0.2	7.1E-01	0.1	1.0E+00	-0.2	5.1E-01	-0.1	1.0E+00	-0.2	3.8E-01	-0.2	5.1E-01
15753	GNGT2	A_23_P26994	NM_031498	1.9	6.79E-05	1.3	2.19E-03	2.6	6.79E-05	1.9	4.13E-07	0.9	2.66E-02	0.8	2.66E-02	1.1	5.36E-02	0.9	5.09E-03	0.0	1.0E+00	0.0	1.0E+00	0.0	9.7E-01	0.1	1.0E+00	-0.1	7.7E-01	-0.1	1.0E+00	-0.1	4.6E-01	-0.1	7.7E-01
26043	CDKN1A	A_23_P59210	NM_000389	1.9	2.66E-03	1.4	2.66E-03	2.1	6.45E-03	1.9	2.09E-04	1.3	7.83E-03	0.9	5.46E-02	1.3	7.83E-03	1.4	2.20E-03	0.3	1.0E+00	0.3	1.0E+00	0.4	6.8E-01	0.3	1.0E+00	0.0	1.0E+00	-0.1	1.0E+00	0.0	1.0E+00	0.1	9.4E-01
15513	CDKN1A	A_23_P59210	NM_000389	1.9	5.52E-03	1.3	5.52E-03	2.2	5.60E-03	1.9	1.86E-04	1.3	3.44E-03	0.9	2.84E-02	1.3	3.44E-03	1.4	1.70E-03	0.3	1.0E+00	0.2	1.0E+00	0.4	6.0E-01	0.3	1.0E+00	0.0	1.0E+00	-0.1	1.0E+00	0.0	1.0E+00	0.1	9.5E-01
31031	PRPH	A_23_P13713	NM_006262	1.9	1.35E-03	1.7	1.35E-03	2.3	5.46E-03	1.9	2.79E-04	1.4	4.94E-02	1.1	7.14E-02	1.4	4.94E-02	1.4	1.35E-02	0.1	1.0E+00	0.1	1.0E+00	0.1	8.7E-01	0.2	1.0E+00	-0.1	9.6E-01	-0.1	1.0E+00	-0.1	8.7E-01	-0.1	9.6E-01
23055	EPHB3	A_23_P95060	NM_004443	1.9	9.77E-04	1.9	9.77E-04	2.2	2.42E-03	1.4	2.86E-04	1.6	7.02E-03	1.5	7.02E-03	1.6	1.50E-02	1.8	1.08E-03	0.3	1.0E+00	0.4	1.0E+00	0.1	8.2E-01	0.3	1.0E+00	0.1	9.7E-01	0.2	1.0E+00	0.0	9.7E-01	0.1	9.7E-01
43464	MGC29506	A_23_P84596	NM_016459	1.9	3.65E-04	1.9	3.65E-04	2.1	1.47E-03	1.9	1.73E-05	1.8	6.18E-03	1.6	6.18E-03	2.3	7.44E-03	1.8	4.08E-04	0.5	9.3E-01	0.3	1.0E+00	0.5	4.1E-01	0.5	9.3E-01	-0.2	8.7E-01	-0.1	1.0E+00	-0.3	6.5E-01	-0.2	8.7E-01
17187	SILV	A_23_P2233	NM_006928	1.9	1.26E-03	1.8	1.26E-03	2.0	3.12E-03	1.9	4.10E-05	1.4	3.34E-02	1.0	3.34E-02	1.4	3.47E-02	1.6	3.35E-03	0.0	1.0E+00	0.0	1.0E+00	0.1	8.6E-01	-0.2	1.0E+00	0.2	5.7E-01	0.2	1.0E+00	0.2	4.2E-01	0.3	5.7E-01
5863	WNT11	A_24_P253003	NM_004626	1.9	4.32E-03	1.9	4.32E-03	1.9	2.54E-02	2.1	6.07E-04	1.6	8.69E-04	1.6	8.69E-04	1.6	4.36E-04	1.9	3.19E-03	-0.2	1.0E+00	-0.2	1.0E+00	-0.2	7.4E-01	-0.1	1.0E+00	0.2	9.3E-01	0.2	1.0E+00	0.1	9.3E-01	0.4	8.6E-01
25365	BEX4	A_24_P40551	NM_001127688	1.9	1.49E-03	1.6	1.49E-03	1.9	9.80E-03	1.9	4.08E-04	1.2	4.59E-02	1.2	1.29E-01	1.1	4.59E-02	1.4	1.90E-02	0.2	1.0E+00	0.2	1.0E+00	0.1	7.9E-01	0.4	1.0E+00	-0.3	7.1E-01	-0.2	1.0E+00	-0.3	5.9E-01	-0.4	7.1E-01
1678	MMP9	A_23_P40174	NM_004994	1.9	2.22E-02	1.9	2.22E-02	2.7	3.50E-02	1.8	1.05E-02	1.5	6.81E-02	1.3	7.07E-02	1.6	6.81E-02	1.5	3.01E-02	0.0	1.0E+00	0.0	1.0E+00	0.2	7.1E-01	0.0	1.0E+00	0.1	9.8E-01	0.3	1.0E+00	0.0	9.8E-01	0.1	9.5E-01
1570	MMP9	A_23_P40174	NM_004994	1.9	3.74E-02	1.9	3.74E-02	2.6	4.67E-02	1.8	1.68E-02	1.4	3.31E-02	1.3	5.46E-02	1.7	3.31E-02	1.4	1.91E-02	0.1	1.0E+00	0.0	1.0E+00	0.3	4.4E-01	0.1	1.0E+00	0.0	1.0E+00	0.3	1.0E+00	0.0	9.8E-01	0.0	1.0E+00
37853	ROM1	A_23_P105002	NM_000327	1.9	2.16E-04	1.9	2.16E-04	2.6	4.09E-04	1.8	5.70E-05	1.7	1.26E-03	1.6	1.26E-03	2.4	5.88E-03	1.7	6.69E-04	0.0	1.0E+00	0.0	1.0E+00	-0.1	8.0E-01	0.0	1.0E+00	-0.1	9.5E-01	0.0	1.0E+00	-0.2	8.2E-01	-0.1	9.5E-01
7841	CPT1C	A_23_P16283	NM_152359	1.9	7.13E-03	2.1	7.13E-03	1.7	1.86E-02	1.9	2.75E-04	1.5	2.15E-02	1.5	3.18E-02	1.4	2.15E-02	1.6	1.44E-02	0.2	1.0E+00	0.1	1.0E+00	0.2	7.1E-01	0.2	1.0E+00	0.1	7.7E-01	0.1	1.0E+00	0.1	7.4E-01	0.2	7.7E-01
15834	ENST00000398572	A_24_P848707	ENST00000398572	1.9	3.84E-04	1.9	5.73E-03	1.9	3.84E-04	1.8	5.35E-06	1.2	8.50E-03	1.2	8.50E-03	1.0	1.84E-03	1.6	1.56E-02	0.0	1.0E+00	0.0	1.0E+00	0.1	7.1E-01	-0.1	1.0E+00	-0.1	8.9E-01	-0.1	1.0E+00	0.0	8.9E-01	-0.1	7.6E-01
25127	CDKN1A	A_23_P59210	NM_000389	1.9	2.16E-03	1.4	2.16E-03	2.2	4.84E-03	1.9	1.34E-04	1.3	8.96E-03	0.9	6.96E-02	1.3	8.96E-03	1.4	3.08E-03	0.3	1.0E+00	0.3	1.0E+00	0.4	6.6E-01	0.3	1.0E+00	0.0	1.0E+00	-0.1	1.0E+00	0.0	1.0E+00	0.0	9.9E-01
33315	BU532663	A_32_P134634	BU532663	1.9	2.75E-04	1.6	2.75E-04	2.6	7.29E-04	1.9	2.97E-05	1.3	1.79E-02	0.7	3.33E-01	1.6	1.73E-02	1.3	1.79E-02	0.1	1.0E+00	0.1	1.0E+00	0.2	7.4E-01	0.1	1.0E+00	0.0	9.8E-01	0.0	1.0E+00	0.1	8.2E-01	0.0	9.8E-01
35986	ADRB2	A_23_P145024	NM_000024	1.9	2.90E-03	1.6	2.90E-03	2.0	3.40E-03	1.9	4.55E-05	1.3	2.89E-02	0.9	1.86E-01	1.3	2.89E-02	1.6	1.63E-02	0.1	1.0E+00	0.1	1.0E+00	-0.2	7.3E-01	0.1	1.0E+00	0.1	8.8E-01	0.2	1.0E+00	-0.1	8.0E-01	0.1	8.8E-01
26812	CCRL2	A_23_P69310	NM_003965	1.9	1.83E-04	1.5	1.83E-04	3.2	2.01E-04	1.9	9.48E-06	1.6	1.73E-03	1.2	1.73E-02	1.7	1.73E-03	1.6	5.07E-04	0.2	1.0E+00	0.2	1.0E+00	0.0	1.0E+00	0.2	1.0E+00	-0.1	9.0E-01	0.0	1.0E+00	-0.2	3.4E-01	-0.1	9.0E-01
21006	LYPD3	A_23_P39265	NM_014400	1.9	2.54E-04	1.7	2.54E-04	2.3	1.68E-03	1.9	3.03E-05	1.7	3.36E-03	1.1	3.31E-02	1.7	3.36E-03	1.7	9.03E-04	0.2	1.0E+00	0.2	1.0E+00	0.2	7.1E-01	0.2	1.0E+00	-0.1	9.8E-01	0.1	1.0E+00	-0.1	8.6E-01	-0.1	9.8E-01
18664	APOE	A_23_P164650	NM_000041	1.9	3.38E-03	1.7	1.28E-02	3.2	3.38E-03	1.9	7.26E-04	1.2	9.08E-02	1.0	1.01E-01	1.2	9.08E-02	1.3	3.90E-02	0.1	1.0E+00	0.1	1.0E+00	0.1	8.6E-01	0.0	1.0E+00	0.0	9.8E-01	0.2	1.0E+00	0.0	9.7E-01	0.0	9.8E-01
34126	ADRB2	A_23_P145024	NM_000024	1.9	6.26E-03	1.7	6.26E-03	2.0	8.06E-03	1.9	1.90E-04	1.4	1.86E-02	0.9	1.49E-01	1.4	1.86E-02	1.7	1.15E-02	0.1	1.0E+00	0.1	1.0E+00	-0.1	8.7E-01	0.2	1.0E+00	0.1	9.2E-01	0.2	1.0E+00	-0.1	8.1E-01	0.1	9.2E-01
37531	RAI2	A_23_P254165	NM_021785	1.9	4.76E-03	2.3	4.76E-03	1.7	4.71E-02	1.9	2.32E-03	1.7	5.17E-03	1.7	2.05E-02	1.5	5.17E-03	2.1	4.41E-03	-0.1	1.0E+00	0.0	1.0E+00	-0.4	7.2E-01	-0.1	1.0E+00	-0.3	5.6E-01	-0.1	1.0E+00	-0.3	2.0E-01	-0.3	5.6E-01
25496	AW972815	A_32_P104334	AW972815	1.9	3.65E-04	1.9	3.65E-04	1.9	2.72E-03	1.8	3.95E-05	2.0	1.24E-03	2.0	2.15E-03	2.1	1.24E-03	1.8	1.24E-03	0.3	9.3E-01	0.2	1.0E+00	0.3	5.7E-01	0.3	9.3E-01	0.0	9.8E-01	0.1	1.0E+00	0.0	9.7E-01	0.0	9.8E-01
30097	RP13-36C9.6	A_32_P170547	NM_001007551	1.9	2.47E-03	1.9	2.47E-03	1.1	3.37E-02	2.0	5.75E-05	1.7	3.03E-03	1.7	1.50E-03	1.2	3.03E-03	2.0	3.77E-03	0.0	1.0E+00	0.0	1.0E+00	-0.1	8.8E-01	0.2	1.0E+00	-0.2	5.1E-01	-0.2	1.0E+00	-0.3	1.5E-01	-0.2	5.1E-01
21809	C10orf10	A_24_P329795	NM_007021	1.9	9.72E-05	1.8	9.72E-05	1.9	2.52E-04	2.4	4.17E-07	1.6	5.94E-03	1.2	5.94E-03	1.7	3.27E-03	1.6	6.89E-03	0.1	1.0E+00	0.1	1.0E+00	0.0	8.8E-01	0.2	1.0E+00	-0.1	9.3E-01	-0.1	1.0E+00	-0.1	6.3E-01	-0.1	9.3E-01
5294	THC2724353	A_32_P116957	THC2724353	1.9	3.60E-04	1.3	4.40E-03	2.4	3.60E-04	1.9	2.72E-06	0.9	8.57E-02	0.7	2.53E-01	0.9	8.57E-02	1.1	1.95E-02	0.0	1.0E+00	0.0	1.0E+00	0.1	7.1E-01	0.0	1.0E+00	0.0	1.0E+00	-0.1	1.0E+00	0.1	8.2E-01	0.0	1.0E+00
13774	TMEM151A	A_23_P354217	NM_153266	1.9	2.96E-03	1.9	2.96E-03	1.9	1.32E-02	1.5	1.27E-03	1.7	6.11E-03	1.7	6.11E-03	1.6	1.59E-02	1.7	8.49E-04	0.2	1.0E+00	0.2	1.0E+00	0.4	7.1E-01	0.1	1.0E+00	0.3	6.1E-01	0.3	1.0E+00	0.2	6.1E-01	0.4	5.3E-01
24861	NEURL	A_23_P138492	NM_004210	1.9	3.19E-04	1.8	8.31E-04	2.0	3.19E-04	1.9	8.36E-07	1.8	1.73E-03	1.6	6.97E-03	1.8	1.73E-03	1.8	9.03E-04	0.0	1.0E+00	0.0	1.0E+00	0.0	9.6E-01	0.2	1.0E+00	-0.1	8.7E-01	0.0	1.0E+00	-0.1	7.5E-01	-0.1	8.7E-01
43617	CRYBA2	A_23_P165457	NM_005209	1.9	1.70E-03	1.9	2.83E-03	2.4	1.70E-03	1.8	7.19E-05	1.3	1.40E-02	1.2	1.40E-02	1.3	4.17E-02	1.6	5.37E-03	-0.2	1.0E+00	0.1	1.0E+00	-0.2	7.2E-01	-0.2	1.0E+00	0.0	1.0E+00	0.3	1.0E+00	0.0	9.9E-01	0.0	1.0E+00
24830	ROPN1L	A_23_P121885	NM_031916	1.9	6.79E-05	1.9	7.77E-04	2.4	6.79E-05	1.7	6.42E-07	1.7	1.63E-02	1.7	2.07E-02	1.8	1.63E-02	1.7	8.80E-03	0.1	1.0E+00	0.1	1.0E+00	-0.2	7.2E-01	0.1	1.0E+00	-0.2	8.2E-01	0.0	1.0E+00	-0.3	4.4E-01	-0.2	8.2E-01
8138	IFITM1	A_23_P72737	NM_003641	1.9	2.25E-02	1.7	2.57E-02	2.5	2.25E-02	1.9	3.27E-03	1.9	2.61E-03	1.7	2.61E-03	1.9	1.84E-03	2.0	5.22E-03	0.0	1.0E+00	0.2	1.0E+00	0.0	9.5E-01	0.0	1.0E+00	0.1	9.6E-01	0.2	1.0E+00	0.1	9.6E-01	0.1	9.5E-01
154	ADRB2	A_23_P145024	NM_000024	1.9	2.84E-03	1.5	3.53E-03	2.1	2.84E-03	1.9	4.45E-05	1.2	1.30E-02	0.7	1.11E-01	1.2	1.26E-02	1.4	1.30E-02	0.1	1.0E+00	0.1	1.0E+00	-0.1	8.8E-01	0.1	1.0E+00	0.1	9.6E-01	0.1	1.0E+00	-0.1	7.5E-01	0.1	9.6E-01
1390	SNAI1	A_23_P131846	NM_005985	2.0	7.57E-04	2.0	7.57E-04	1.8	1.64E-03	2.3	2.59E-06	1.6	1.02E-03	1.1	2.45E-02	1.6	1.02E-03	1.9	4.47E-04	0.2	1.0E+00	0.1	1.0E+00	0.2	7.1E-01	0.3	1.0E+00	0.1	8.7E-01	0.2	1.0E+00	0.1	8.6E-01	0.1	8.7E-01
27969	TCN2	A_23_P40611	NM_000355	2.0	1.32E-03	1.7	1.32E-03	2.0	1.44E-03	2.5	2.10E-06	2.3	4.63E-03	1.8	4.63E-03	2.3	1.70E-02	2.8	1.37E-03	0.5	3.3E-01	0.5	3.3E-01	0.5	2.4E-01	0.5	9.8E-01	-0.2	8.5E-01	-0.1	1.0E+00	-0.2	6.2E-01	-0.2	8.5E-01
44101	CSH1	A_23_P207170	NM_022640	2.0	8.22E-03	1.9	8.22E-03	2.0	3.22E-02	2.0	1.47E-03	0.7	6.12E-01	0.0	9.93E-01	0.7	6.12E-01	0.9	4.91E-01	-0.2	1.0E+00	-0.2	1.0E+00	0.2	8.9E-01	-0.2	1.0E+00	0.3	8.4E-01	0.1	1.0E+00	0.5	2.7E-01	0.3	8.4E-01
43631	CFTR	A_23_P215720	NM_000492	2.0	3.11E-05	2.1	3.11E-05	0.9	2.09E-02	2.0	4.76E-06	2.0	5.71E-04	2.0	5.71E-04	1.5	9.16E-04	2.0	7.52E-05	0.2	1.0E+00	0.2	1.0E+00	0.1	7.2E-01	0.2	1.0E+00	0.0	9.9E-01	0.1	1.0E+00	-0.1	7.5E-01	0.0	9.9E-01
31525	ZMYND15	A_23_P89570	NM_032265	2.0	1.66E-03	1.9	1.92E-03	2.6	1.66E-03	2.0	5.92E-05	1.6	1.48E-02	1.2	1.96E-02	1.8	1.48E-02	1.6	2.33E-03	0.1	1.0E+00	0.1	1.0E+00	0.1	7.8E-01	0.0	1.0E+00	0.1	8.2E-01	0.2	1.0E+00	0.1	7.7E-01	0.1	8.2E-01
26311	HBB	A_23_P203558	NM_000518	2.0	1.51E-03	1.6	5.28E-03	2.0	1.51E-03	2.0	9.51E-06	0.8	1.38E-01	0.5	1.62E-01	0.8	2.04E-02	1.1	1.38E-01	0.0	1.0E+00	0.0	1.0E+00	-0.1	7.8E-01	0.1	1.0E+00	0.1	9.5E-01	0.1	1.0E+00	-0.1	8.9E-01	0.1	9.5E-01
40398	MMP9	A_23_P40174	NM_004994	2.0	2.16E-02	2.0	2.16E-02	2.7	3.73E-02	1.9	9.50E-03	1.5	3.01E-02	1.2	4.62E-02	1.8	3.01E-02	1.5	1.40E-02	0.0	1.0E+00	0.0	1.0E+00	0.1	8.2E-01	0.0	1.0E+00	0.2	9.8E-01	0.3	1.0E+00	0.0	9.8E-01	0.2	9.4E-01
31474	SIGLEC15	A_23_P50146	NM_213602	2.0	1.05E-03	1.2	1.35E-03	2.2	1.05E-03	2.0	3.17E-05	0.8	4.48E-01	0.1	9.54E-01	1.1	3.74E-01	0.8	4.48E-01	0.0	1.0E+00	0.0	1.0E+00	0.1	9.6E-01	-0.2	1.0E+00	0.1	9.2E-01	-0.1	1.0E+00	0.1	9.2E-01	0.7	1.7E-01
33824	NRXN2	A_24_P261470	NM_138732	2.0	3.69E-04	2.0	2.87E-03	2.2	3.69E-04	1.6	5.69E-06	1.6	9.16E-03	1.7	3.33E-03	1.6	9.16E-03	1.6	9.77E-03	0.1	1.0E+00	0.1	1.0E+00	0.1	8.2E-01	0.2	1.0E+00	-0.1	9.3E-01	0.0	1.0E+00	-0.1	6.4E-01	-0.1	9.3E-01
22000	FAM148C	A_32_P82895	NM_001136263	2.0	2.12E-04	1.7	2.12E-04	2.4	8.95E-04	2.0	1.26E-05	1.5	3.01E-03	1.5	6.18E-03	1.9	3.01E-03	1.5	1.70E-03	0.3	1.0E+00	0.3	1.0E+00	0.4	7.1E-01	0.3	1.0E+00	0.0	9.5E-01	0.0	1.0E+00	0.0	9.5E-01	-0.2	9.2E-01
32149	PTGIR	A_23_P340848	NM_000960	2.0	6.52E-05	1.8	1.07E-03	3.0	6.52E-05	2.0	6.75E-07	1.5	9.76E-03	1.2	3.04E-02	1.6	9.76E-03	1.5	2.05E-03	0.1	1.0E+00	0.1	1.0E+00	0.0	9.8E-01	0.2	1.0E+00	-0.2	5.2E-01	0.0	1.0E+00	-0.2	3.2E-01	-0.2	5.2E-01
44857	A_32_P99804	A_32_P99804	A_32_P99804	2.0	7.11E-05	2.0	1.54E-03	2.4	7.11E-05	1.7	8.92E-07	1.7	2.91E-03	1.7	3.72E-03	2.1	2.10E-03	1.6	2.91E-03	-0.1	1.0E+00	-0.1	1.0E+00	0.1	7.7E-01	-0.1	1.0E+00	-0.3	5.0E-01	-0.1	1.0E+00	-0.3	4.1E-01	-0.4	5.0E-01
28432	THC2533854	A_32_P184268	THC2533854	2.0	2.93E-04	1.3	6.84E-04	2.3	2.93E-04	2.0	2.66E-06	1.4	1.52E-02	1.2	2.37E-02	1.5	1.52E-02	1.4	3.66E-03	0.2	1.0E+00	0.2	1.0E+00	0.0	9.4E-01	0.2	1.0E+00	-0.2	5.6E-01	-0.1	1.0E+00	-0.2	5.0E-01	-0.3	5.6E-01
4652	CFTR	A_23_P215720	NM_000492	2.0	3.54E-05	2.1	3.54E-05	1.0	7.23E-03	2.0	1.57E-06	2.0	5.63E-04	2.0	8.74E-04	1.6	5.63E-04	2.0	1.12E-04	0.1	1.0E+00	0.1	1.0E+00	0.1	7.9E-01	0.1	1.0E+00	-0.1	9.3E-01	0.1	1.0E+00	-0.1	8.3E-01	-0.1	9.3E-01
39018	CFTR	A_23_P215720	NM_000492	2.0	1.27E-05	2.1	1.27E-05	1.0	3.21E-03	2.0	5.53E-07	2.0	1.22E-03	2.0	2.40E-03	1.6	1.22E-03	2.0	2.72E-04	0.1	1.0E+00	0.1	1.0E+00	0.1	8.4E-01	0.2	1.0E+00	0.0	9.8E-01	0.1	1.0E+00	-0.1	8.2E-01	0.0	9.8E-01
1988	ITGA1	A_23_P256334	NM_181501	2.0	6.45E-04	1.5	9.47E-04	2.0	6.45E-04	2.0	2.90E-06	1.5	2.20E-02	1.0	5.72E-02	1.8	2.20E-02	1.5	7.39E-03	-0.1	1.0E+00	0.0	1.0E+00	-0.4	7.1E-01	-0.1	1.0E+00	-0.2	8.2E-01	-0.1	1.0E+00	-0.2	5.2E-01	-0.2	8.2E-01
44378	ISG15	A_23_P819	NM_005101	2.0	2.00E-04	1.8	2.00E-04	2.7	7.10E-04	2.0	3.51E-05	1.5	6.25E-03	1.4	8.51E-03	1.5	6.25E-03	1.7	2.73E-03	0.0	1.0E+00	0.2	1.0E+00	0.0	9.6E-01	-0.1	1.0E+00	0.0	9.9E-01	0.2	1.0E+00	0.0	9.9E-01	0.0	9.9E-01
28844	ULBP2	A_23_P168259	NM_025217	2.0	3.77E-04	2.0	3.77E-04	1.7	5.22E-03	2.2	1.62E-05	1.6	1.55E-02	1.6	1.61E-02	1.3	1.55E-02	2.0	1.99E-03	0.1	1.0E+00	0.1	1.0E+00	-0.1	8.3E-01	0.3	1.0E+00	0.0	9.9E-01	0.0	1.0E+00	-0.1	7.9E-01	0.0	9.9E-01
34180	ADRB2	A_23_P145024	NM_000024	2.0	3.11E-03	1.6	3.37E-03	2.1	3.11E-03	2.0	3.78E-05	1.3	1.39E-02	0.8	1.11E-01	1.3	1.39E-02	1.6	1.16E-02	0.0	1.0E+00	0.0	1.0E+00	-0.2	7.1E-01	0.1	1.0E+00	0.1	9.6E-01	0.1	1.0E+00	-0.1	8.3E-01	0.1	9.6E-01
25413	MMP9	A_23_P40174	NM_004994	2.0	2.50E-02	2.0	2.50E-02	2.7	4.20E-02	1.9	1.22E-02	1.5	1.99E-02	1.3	2.32E-02	1.8	1.99E-02	1.5	1.25E-02	0.0	1.0E+00	0.0	1.0E+00	0.2	7.1E-01	0.0	1.0E+00	0.1	9.9E-01	0.3	1.0E+00	0.0	9.9E-01	0.1	9.4E-01
36000	LMTK3	A_23_P164918	NM_001080434	2.0	4.27E-04	1.8	4.27E-04	2.7	7.22E-04	2.0	4.74E-05	1.9	1.04E-02	1.3	6.41E-02	2.3	1.04E-02	1.9	3.16E-03	0.1	1.0E+00	0.3	1.0E+00	0.1	8.6E-01	0.1	1.0E+00	0.1	9.7E-01	0.1	1.0E+00	0.0	9.7E-01	0.1	9.2E-01
3929	DPY19L2P2	A_24_P186608	NR_003561	2.0	2.31E-04	1.2	4.09E-04	2.0	2.31E-04	2.2	8.55E-07	1.5	1.56E-02	1.0	3.08E-02	1.6	1.56E-02	1.5	4.68E-03	0.0	1.0E+00	0.0	1.0E+00	-0.1	7.9E-01	0.1	1.0E+00	-0.2	4.2E-01	-0.1	1.0E+00	-0.2	2.6E-01	-0.2	4.2E-01
38591	KIF25	A_23_P59410	NM_030615	2.0	3.01E-05	1.6	3.01E-05	2.6	5.71E-05	2.0	3.18E-07	1.8	2.73E-03	1.7	2.73E-03	2.2	3.36E-03	1.8	4.47E-04	0.0	1.0E+00	0.0	1.0E+00	0.0	9.9E-01	0.0	1.0E+00	0.0	9.8E-01	-0.1	1.0E+00	0.0	9.7E-01	0.0	9.8E-01
25985	FLJ20433	A_24_P49183	NM_017820	2.0	1.30E-04	1.7	1.02E-02	4.2	1.30E-04	2.0	1.81E-05	1.3	4.68E-02	0.6	1.46E-01	1.3	3.78E-03	1.4	4.68E-02	0.0	1.0E+00	-0.1	1.0E+00	0.0	9.3E-01	0.1	1.0E+00	-0.1	8.1E-01	0.1	1.0E+00	-0.1	7.6E-01	-0.1	8.1E-01
26418	LILRA5	A_23_P107847	NM_181985	2.0	1.82E-04	2.0	3.25E-04	1.8	1.82E-04	2.1	1.94E-07	1.4	1.02E-03	1.1	4.28E-03	1.4	1.02E-03	1.8	3.36E-04	0.0	1.0E+00	0.0	1.0E+00	0.0	1.0E+00	-0.1	1.0E+00	0.0	9.1E-01	0.0	1.0E+00	0.0	8.9E-01	0.1	9.1E-01
3738	NPPC	A_23_P301942	NM_024409	2.0	1.27E-05	2.0	1.27E-05	1.3	2.60E-03	2.2	1.95E-06	1.5	4.34E-02	1.5	4.34E-02	0.7	2.42E-01	2.1	6.16E-03	0.2	1.0E+00	0.2	1.0E+00	-0.2	7.3E-01	0.3	1.0E+00	-0.1	9.4E-01	0.2	1.0E+00	-0.4	2.5E-01	-0.1	9.4E-01
3853	ENST00000357303	A_23_P364504	ENST00000357303	2.0	3.80E-04	2.0	3.80E-04	1.5	1.13E-02	2.1	1.05E-04	2.1	9.10E-04	2.2	9.10E-04	1.6	2.36E-03	2.1	1.23E-04	0.4	9.4E-01	0.4	9.8E-01	0.3	7.1E-01	0.6	9.4E-01	-0.1	9.1E-01	0.1	1.0E+00	-0.1	9.1E-01	-0.2	8.6E-01
4473	PRAME	A_23_P166360	NM_206956	2.0	1.00E-03	2.1	1.00E-03	1.1	3.21E-03	2.0	1.85E-05	1.6	5.64E-03	1.5	8.84E-03	1.6	3.01E-03	1.8	5.64E-03	0.1	1.0E+00	0.1	1.0E+00	-0.1	8.5E-01	0.1	1.0E+00	-0.1	7.0E-01	-0.1	1.0E+00	-0.2	4.1E-01	-0.1	7.0E-01
28361	WISP2	A_23_P102611	NM_003881	2.0	2.96E-03	2.5	2.96E-03	1.3	1.27E-02	2.0	3.90E-05	1.9	4.27E-03	2.0	4.27E-03	1.6	3.50E-03	1.9	3.38E-02	0.1	1.0E+00	0.3	1.0E+00	-0.2	7.2E-01	0.1	1.0E+00	-0.1	8.1E-01	-0.1	1.0E+00	-0.2	4.9E-01	-0.1	8.1E-01
41065	GD136470	A_32_P184937	GD136470	2.0	2.69E-03	1.4	3.10E-03	2.5	2.69E-03	2.0	8.55E-05	1.1	2.40E-02	0.7	1.19E-01	1.1	2.40E-02	1.2	5.67E-03	0.2	1.0E+00	0.2	1.0E+00	0.2	7.1E-01	0.2	1.0E+00	-0.1	9.5E-01	0.0	1.0E+00	-0.1	9.2E-01	-0.1	9.5E-01
33261	MAFB	A_23_P17345	NM_005461	2.0	4.07E-03	2.0	4.07E-03	1.9	1.00E-02	2.2	1.30E-04	1.7	3.67E-03	1.2	1.71E-02	1.7	1.22E-03	2.0	3.67E-03	-0.2	1.0E+00	-0.2	1.0E+00	-0.2	7.1E-01	-0.2	1.0E+00	0.2	9.0E-01	0.2	1.0E+00	0.1	9.0E-01	0.2	7.9E-01
15762	DIO3	A_23_P105923	NM_001362	2.0	7.17E-04	2.0	6.64E-03	3.1	7.17E-04	2.0	3.30E-05	1.4	2.85E-02	0.9	2.85E-02	1.7	5.36E-03	1.4	9.43E-02	-0.2	1.0E+00	-0.2	1.0E+00	-0.2	7.3E-01	-0.3	1.0E+00	-0.1	9.4E-01	-0.1	1.0E+00	-0.1	9.1E-01	-0.1	9.4E-01
16641	NRIP3	A_23_P47682	NM_020645	2.0	6.84E-04	2.0	6.84E-04	2.0	1.42E-03	2.4	1.76E-05	1.1	2.96E-02	1.1	2.96E-02	1.1	1.44E-01	2.0	2.88E-04	0.3	1.0E+00	0.3	1.0E+00	0.4	7.1E-01	0.1	1.0E+00	0.2	8.8E-01	0.3	1.0E+00	0.2	7.4E-01	0.2	8.8E-01
15842	GADD45G	A_24_P120934	NM_006705	2.0	6.91E-03	1.8	7.74E-03	2.7	6.91E-03	2.0	6.61E-04	1.6	1.03E-02	0.9	4.09E-02	1.6	3.50E-03	1.6	1.03E-02	0.2	1.0E+00	-0.2	1.0E+00	0.4	4.3E-01	0.2	1.0E+00	-0.1	9.6E-01	-0.1	1.0E+00	-0.2	8.9E-01	-0.1	9.6E-01
24885	LPXN	A_23_P87150	NM_004811	2.0	5.92E-05	1.9	5.92E-05	2.0	6.19E-04	2.2	2.55E-06	1.9	3.99E-03	1.6	2.50E-02	2.0	3.99E-03	1.9	2.21E-03	0.0	1.0E+00	-0.1	1.0E+00	0.0	9.6E-01	0.2	1.0E+00	-0.2	8.0E-01	-0.1	1.0E+00	-0.3	3.3E-01	-0.2	8.0E-01
22351	FOSB	A_23_P429998	NM_006732	2.0	2.72E-04	1.9	2.72E-04	2.0	3.84E-03	2.2	2.96E-05	0.9	2.99E-01	0.9	2.99E-01	0.6	6.52E-01	1.7	1.33E-02	0.1	1.0E+00	0.0	1.0E+00	0.3	7.1E-01	0.1	1.0E+00	0.4	3.8E-01	-0.1	1.0E+00	0.4	3.5E-01	0.4	3.8E-01
34245	PLAC1	A_23_P148609	NM_021796	2.1	2.20E-04	2.1	2.20E-04	2.3	6.15E-04	1.7	4.02E-05	1.9	2.10E-03	2.1	3.20E-03	1.9	2.10E-03	1.9	9.54E-04	0.3	7.1E-01	0.3	7.3E-01	0.3	7.1E-01	0.5	6.3E-01	-0.1	8.5E-01	0.0	1.0E+00	-0.2	5.0E-01	-0.1	8.5E-01
37055	ACVRL1	A_24_P945113	NM_000020	2.1	3.99E-05	2.1	3.77E-04	3.0	3.99E-05	2.0	4.07E-07	1.8	3.27E-03	1.5	5.65E-03	2.0	3.27E-03	1.8	3.38E-04	0.0	1.0E+00	0.0	1.0E+00	0.1	7.1E-01	0.0	1.0E+00	-0.1	9.0E-01	0.0	1.0E+00	-0.1	7.5E-01	-0.1	9.0E-01
14202	CR603982	A_32_P146635	CR603982	2.1	1.52E-03	2.2	1.52E-03	1.8	2.86E-02	2.1	5.27E-04	1.9	1.92E-03	1.9	1.85E-03	1.4	1.92E-03	1.9	3.60E-03	0.3	1.0E+00	0.3	1.0E+00	0.2	7.1E-01	0.5	1.0E+00	-0.3	7.4E-01	-0.2	1.0E+00	-0.3	6.4E-01	-0.4	7.4E-01
10478	CFTR	A_23_P215720	NM_000492	2.1	1.74E-04	2.3	1.74E-04	0.9	2.58E-02	2.1	8.84E-06	2.1	1.63E-03	2.2	1.63E-03	1.7	1.84E-03	2.1	3.19E-05	0.2	1.0E+00	0.2	1.0E+00	0.0	9.0E-01	0.3	1.0E+00	-0.1	9.2E-01	0.0	1.0E+00	-0.1	7.9E-01	-0.1	9.2E-01
12888	PDE8B	A_24_P197537	NM_003719	2.1	2.93E-04	2.1	2.93E-04	1.8	7.16E-03	2.9	8.18E-06	2.2	3.36E-03	1.3	5.92E-02	2.4	3.36E-03	2.2	1.03E-03	0.2	1.0E+00	-0.1	1.0E+00	0.2	7.1E-01	0.4	1.0E+00	0.0	9.7E-01	0.1	1.0E+00	-0.2	6.7E-01	0.0	9.7E-01
18112	HIST1H3F	A_24_P97914	BC062305	2.1	2.60E-06	2.1	2.60E-06	1.9	1.35E-04	2.3	5.22E-08	2.1	8.48E-04	2.1	1.01E-03	2.5	2.48E-04	2.0	8.48E-04	0.6	3.7E-01	0.6	5.5E-01	0.4	3.7E-01	0.6	8.1E-02	-0.3	5.1E-01	0.0	1.0E+00	-0.3	2.3E-01	-0.3	5.1E-01
30711	HCP5	A_23_P111126	L06175	2.1	4.61E-04	2.1	1.40E-03	2.2	4.61E-04	2.0	3.30E-06	1.4	2.30E-02	1.4	2.30E-02	1.2	2.30E-02	2.0	5.87E-03	0.2	1.0E+00	0.2	1.0E+00	0.0	9.7E-01	0.2	1.0E+00	0.1	1.0E+00	0.1	1.0E+00	0.0	1.0E+00	0.1	9.3E-01
27172	THC2726026	A_32_P49035	THC2726026	2.1	1.77E-04	2.0	1.77E-04	3.6	2.21E-04	2.1	8.60E-06	2.0	1.78E-03	1.7	1.78E-03	2.8	2.89E-03	2.0	9.12E-05	0.3	1.0E+00	0.2	1.0E+00	0.4	5.7E-01	0.3	1.0E+00	-0.1	9.6E-01	0.1	1.0E+00	-0.1	9.3E-01	-0.1	9.6E-01
23705	XX-FW88277B6.1	A_32_P780817	NM_001017417	2.1	1.28E-03	2.1	1.28E-03	1.2	2.78E-03	2.2	3.66E-06	1.7	1.92E-03	1.7	1.85E-03	1.0	9.13E-03	1.8	1.92E-03	0.1	1.0E+00	0.1	1.0E+00	0.0	9.7E-01	0.1	1.0E+00	-0.1	7.5E-01	0.0	1.0E+00	-0.2	2.8E-01	-0.1	7.5E-01
11395	CFTR	A_23_P215720	NM_000492	2.1	7.25E-05	2.3	7.25E-05	1.0	4.13E-03	2.1	2.66E-06	2.2	2.72E-03	2.2	2.72E-03	1.6	3.49E-03	2.2	7.52E-05	0.1	1.0E+00	0.1	1.0E+00	0.1	7.7E-01	0.2	1.0E+00	-0.1	8.3E-01	0.1	1.0E+00	-0.2	5.0E-01	-0.1	8.3E-01
42665	FBXO32	A_23_P82814	NM_058229	2.1	2.22E-03	1.8	2.92E-03	2.1	2.22E-03	2.1	2.30E-05	0.9	4.10E-02	0.8	7.76E-02	0.9	4.10E-02	1.4	1.36E-02	0.2	1.0E+00	0.2	1.0E+00	0.0	1.0E+00	0.2	1.0E+00	-0.2	8.2E-01	-0.1	1.0E+00	-0.2	6.1E-01	-0.2	8.2E-01
27784	CFTR	A_23_P215720	NM_000492	2.1	4.69E-05	2.3	4.69E-05	1.0	1.19E-02	2.1	3.06E-06	2.2	5.01E-04	2.3	6.44E-04	1.8	5.01E-04	2.2	1.58E-05	0.1	1.0E+00	0.0	1.0E+00	0.1	9.2E-01	0.2	1.0E+00	-0.2	6.2E-01	-0.1	1.0E+00	-0.3	2.3E-01	-0.2	6.2E-01
17855	TRIM43	A_23_P17173	NM_138800	2.1	1.82E-04	1.7	3.21E-04	2.1	1.82E-04	2.3	3.93E-07	1.3	1.87E-03	0.8	3.53E-02	1.3	1.87E-03	1.7	9.66E-04	0.1	1.0E+00	0.1	1.0E+00	0.1	7.7E-01	0.2	1.0E+00	-0.2	6.9E-01	-0.2	1.0E+00	-0.2	6.9E-01	-0.3	6.9E-01
35800	CD33	A_23_P331748	NM_001772	2.1	5.65E-04	1.8	5.65E-04	2.1	6.24E-04	2.1	1.95E-06	1.5	1.16E-03	1.3	6.74E-03	1.5	9.13E-04	1.7	1.16E-03	0.1	1.0E+00	0.1	1.0E+00	-0.1	7.8E-01	0.1	1.0E+00	-0.1	8.8E-01	0.1	1.0E+00	-0.2	4.0E-01	-0.1	8.8E-01
30565	HSD17B1	A_23_P15542	NM_000413	2.1	2.22E-04	2.0	1.25E-03	2.9	2.22E-04	2.1	3.26E-06	1.8	1.25E-03	1.5	4.29E-03	2.0	8.51E-04	1.8	1.25E-03	0.1	1.0E+00	0.0	1.0E+00	0.1	7.7E-01	0.1	1.0E+00	0.1	1.0E+00	0.1	1.0E+00	0.1	9.2E-01	0.0	1.0E+00
32982	C1orf38	A_23_P873	BC031655	2.1	3.01E-05	1.8	3.01E-05	2.3	3.84E-04	2.1	3.18E-06	1.7	3.80E-04	1.4	1.94E-03	1.7	3.80E-04	1.8	2.96E-04	0.2	1.0E+00	0.2	1.0E+00	0.1	8.9E-01	0.2	1.0E+00	0.0	9.5E-01	0.0	1.0E+00	0.0	9.5E-01	0.1	9.0E-01
39195	RUNDC3B	A_23_P111724	NM_138290	2.1	2.64E-04	1.8	3.25E-04	2.4	2.64E-04	2.1	1.27E-06	1.6	3.17E-03	1.6	4.74E-03	1.6	3.17E-03	2.2	2.30E-03	0.0	1.0E+00	0.0	1.0E+00	-0.3	7.1E-01	0.1	1.0E+00	-0.1	9.2E-01	0.0	1.0E+00	-0.1	8.4E-01	-0.1	9.2E-01
8836	CD68	A_23_P15394	NM_001251	2.1	5.24E-05	2.0	3.77E-04	2.9	5.24E-05	2.1	1.23E-06	1.7	6.83E-04	1.4	1.78E-03	1.7	5.12E-04	2.0	6.83E-04	0.2	1.0E+00	0.2	1.0E+00	0.1	8.1E-01	0.2	1.0E+00	0.0	1.0E+00	0.0	1.0E+00	-0.1	7.5E-01	0.0	1.0E+00
28802	GPR20	A_23_P159237	NM_005293	2.1	9.72E-05	2.1	9.72E-05	2.4	3.66E-04	2.0	3.06E-06	1.7	7.67E-03	1.7	7.67E-03	1.7	2.04E-02	1.9	3.64E-03	0.0	1.0E+00	0.0	1.0E+00	0.1	8.3E-01	-0.1	1.0E+00	0.1	8.9E-01	0.1	1.0E+00	0.1	8.9E-01	0.3	5.7E-01
33195	METRNL	A_23_P10591	NM_001004431	2.1	9.51E-04	2.1	9.51E-04	2.3	2.27E-03	1.9	1.05E-04	1.5	8.83E-03	1.5	8.83E-03	1.5	1.37E-02	2.0	1.30E-03	0.2	1.0E+00	0.2	1.0E+00	0.1	8.3E-01	0.4	1.0E+00	0.0	9.8E-01	0.0	1.0E+00	-0.2	6.8E-01	0.0	9.8E-01
32265	ROBO4	A_23_P104881	NM_019055	2.1	3.54E-04	1.3	2.90E-03	2.7	3.54E-04	2.1	8.70E-06	1.4	5.80E-02	0.8	2.93E-01	1.4	5.80E-02	1.7	1.73E-02	0.0	1.0E+00	0.2	1.0E+00	0.0	9.9E-01	0.0	1.0E+00	-0.1	6.8E-01	-0.1	1.0E+00	-0.1	6.8E-01	0.2	6.8E-01
21517	THC2660636	A_32_P184888	THC2660636	2.1	1.46E-04	2.1	1.46E-04	2.3	1.11E-03	1.9	3.11E-05	2.1	2.70E-03	2.1	5.95E-03	2.2	1.70E-03	2.1	2.70E-03	0.4	7.1E-01	0.4	6.9E-01	0.2	7.1E-01	0.4	1.0E+00	-0.2	7.0E-01	0.1	1.0E+00	-0.2	5.0E-01	-0.2	7.0E-01
18236	PFKFB3	A_23_P95755	NM_004566	2.1	5.25E-03	1.7	1.05E-02	2.6	5.25E-03	2.1	2.74E-04	1.6	1.80E-02	1.2	2.33E-02	1.6	1.80E-02	1.7	1.07E-02	0.0	1.0E+00	0.0	1.0E+00	0.2	8.0E-01	-0.1	1.0E+00	0.0	9.5E-01	0.0	1.0E+00	0.2	5.9E-01	0.0	9.5E-01
33999	LOC388242	A_32_P135336	NR_002556	2.1	2.16E-04	2.0	2.16E-04	2.8	2.28E-04	2.1	3.96E-06	1.5	1.36E-01	1.5	2.61E-01	1.8	1.36E-01	1.5	1.21E-01	0.1	1.0E+00	0.1	1.0E+00	0.5	7.1E-01	0.0	1.0E+00	-0.1	9.8E-01	-0.3	1.0E+00	-0.1	8.6E-01	0.0	9.8E-01
29280	CSPG4	A_23_P21976	NM_001897	2.1	1.79E-05	2.1	4.31E-04	3.3	1.79E-05	2.0	1.63E-07	1.8	6.42E-03	1.8	6.42E-03	2.1	6.70E-03	1.7	1.50E-03	0.2	8.6E-01	0.2	7.7E-01	0.1	8.6E-01	0.3	1.0E+00	-0.1	8.4E-01	-0.1	1.0E+00	-0.2	3.8E-01	-0.1	8.4E-01
36677	CLIC3	A_23_P254654	NM_004669	2.1	4.42E-03	2.1	4.42E-03	2.2	9.77E-03	1.6	2.18E-03	2.0	1.25E-02	1.9	2.10E-02	2.0	1.25E-02	2.3	1.65E-03	0.0	1.0E+00	0.0	1.0E+00	-0.2	7.9E-01	0.0	1.0E+00	-0.1	9.1E-01	0.3	1.0E+00	-0.1	9.1E-01	-0.2	8.8E-01
40588	LRRC32	A_24_P389916	NM_005512	2.1	1.28E-04	1.7	1.28E-04	2.1	2.22E-04	2.1	4.26E-07	1.1	6.23E-02	0.6	4.12E-01	1.1	6.23E-02	1.4	2.60E-02	0.0	1.0E+00	0.0	1.0E+00	0.4	7.2E-01	0.0	1.0E+00	0.1	9.9E-01	0.1	1.0E+00	0.1	7.4E-01	0.0	9.9E-01
44814	PTPN7	A_23_P201778	NM_080588	2.1	1.47E-04	1.4	5.76E-04	2.6	1.47E-04	2.1	5.27E-07	1.6	1.28E-03	1.3	1.64E-02	1.6	1.28E-03	1.8	2.88E-04	0.2	1.0E+00	0.2	1.0E+00	0.1	8.3E-01	0.2	1.0E+00	-0.2	8.2E-01	0.0	1.0E+00	-0.2	5.4E-01	-0.2	8.2E-01
7826	AEBP1	A_23_P157299	NM_001129	2.1	1.02E-03	2.1	1.02E-03	2.1	1.28E-03	2.0	9.07E-06	1.9	3.71E-04	1.9	4.96E-03	1.7	3.71E-04	1.9	1.31E-04	0.2	1.0E+00	0.2	1.0E+00	0.0	9.4E-01	0.2	1.0E+00	-0.2	3.8E-01	0.0	1.0E+00	-0.2	2.5E-01	-0.3	3.8E-01
22942	IRF7	A_24_P378019	NM_004031	2.1	1.80E-04	2.0	1.80E-04	2.5	9.75E-04	2.1	2.45E-05	1.9	6.06E-04	1.7	2.28E-03	1.9	6.06E-04	2.0	9.19E-05	0.2	1.0E+00	0.3	1.0E+00	0.2	7.3E-01	0.2	1.0E+00	0.1	1.0E+00	0.1	1.0E+00	0.1	9.1E-01	0.0	1.0E+00
43333	NRGN	A_23_P116264	NM_006176	2.1	6.13E-04	1.8	1.26E-03	2.7	6.13E-04	2.1	8.70E-06	1.7	1.28E-03	1.4	2.12E-02	1.8	1.28E-03	1.7	1.83E-04	0.2	1.0E+00	0.2	1.0E+00	0.1	9.0E-01	0.2	1.0E+00	-0.2	8.8E-01	0.0	1.0E+00	-0.2	7.2E-01	-0.2	8.8E-01
42249	ZC3H7B	A_24_P797678	BC004857	2.1	1.34E-04	2.1	1.82E-04	2.3	1.34E-04	2.1	2.27E-05	2.5	1.83E-04	2.3	2.30E-03	2.7	1.31E-04	2.5	1.83E-04	0.4	7.3E-01	0.4	8.3E-01	0.2	7.3E-01	0.5	6.6E-01	-0.1	8.7E-01	-0.1	1.0E+00	-0.1	7.0E-01	-0.1	8.7E-01
7470	RPRML	A_32_P3476	NM_203400	2.1	5.59E-03	2.1	7.35E-03	2.3	5.46E-03	1.1	5.59E-03	2.4	6.34E-03	2.4	2.40E-03	2.4	6.34E-03	1.9	2.93E-02	0.3	1.0E+00	0.3	1.0E+00	0.0	1.0E+00	0.3	1.0E+00	-0.1	8.0E-01	-0.1	1.0E+00	-0.1	8.0E-01	-0.4	6.6E-01
40940	CCDC88B	A_23_P24384	NM_032251	2.1	1.01E-03	2.0	2.34E-03	2.8	1.01E-03	2.1	2.04E-05	1.6	1.70E-03	1.6	1.70E-03	2.0	6.42E-03	1.6	1.17E-03	0.1	1.0E+00	0.1	1.0E+00	0.1	8.1E-01	0.2	1.0E+00	-0.3	8.2E-01	-0.1	1.0E+00	-0.3	6.8E-01	-0.4	8.2E-01
16886	DNALI1	A_23_P160377	NM_003462	2.1	2.22E-04	2.0	2.84E-03	2.4	2.22E-04	2.1	1.12E-06	1.8	4.19E-03	1.6	2.43E-03	1.9	4.19E-03	1.8	6.29E-03	-0.1	1.0E+00	-0.2	1.0E+00	-0.1	9.3E-01	0.0	1.0E+00	-0.1	6.9E-01	0.0	1.0E+00	-0.1	4.9E-01	-0.2	6.9E-01
35797	ASGR1	A_23_P118722	NM_001671	2.1	7.69E-05	1.6	2.20E-04	3.7	7.69E-05	2.1	2.24E-06	1.7	7.15E-03	1.2	7.25E-03	2.3	7.15E-03	1.7	6.12E-04	0.1	1.0E+00	0.0	1.0E+00	0.1	8.2E-01	0.2	1.0E+00	-0.2	8.2E-01	0.0	1.0E+00	-0.3	5.6E-01	-0.2	8.2E-01
21619	COL11A2	A_23_P42322	NM_080680	2.1	4.88E-05	2.1	4.88E-05	2.4	8.23E-05	2.0	2.02E-07	1.7	4.87E-03	1.6	7.87E-03	1.8	4.87E-03	1.7	1.96E-03	0.0	1.0E+00	0.0	1.0E+00	-0.1	8.2E-01	0.1	1.0E+00	0.0	9.7E-01	0.0	1.0E+00	0.0	9.0E-01	0.0	9.7E-01
5078	CSH1	A_24_P211044	NM_022640	2.1	5.21E-03	1.9	5.21E-03	2.4	1.25E-02	2.1	6.72E-04	0.7	6.34E-01	0.3	8.99E-01	0.7	6.34E-01	1.0	3.98E-01	-0.2	1.0E+00	-0.4	1.0E+00	0.3	7.6E-01	-0.2	1.0E+00	0.7	1.7E-01	0.2	1.0E+00	0.7	1.6E-02	0.7	1.7E-01

Feature	Gene symbol	Probe	Transcript	72 hour								48 hours								24 hours								10 hours							
				siCASP8AP2								siCASP8AP2								siCASP8AP2								siCASP8AP2							
				Median	Median	1	1	3	3	6	6	Median	Median	1	1	3	3	6	6	Median	Median	1	1	3	3	6	6	Median	Median	1	1	3	3	6	6
				M	q	M	q	M	q	M	q	M	q	M	q	M	q	M	q	M	q	M	q	M	q	M	q	M	q	M	q	M	q	M	q
14852	MMP9	A_23_P40174	NM_004994	2.2	2.15E-02	2.2	2.15E-02	2.9	4.11E-02	2.1	1.13E-02	1.7	8.71E-03	1.5	8.71E-03	1.9	1.70E-02	1.7	5.86E-03	0.0	1.0E+00	0.0	1.0E+00	0.2	7.1E-01	0.0	1.0E+00	0.1	9.7E-01	0.3	1.0E+00	0.0	9.7E-01	0.1	9.5E-01
14430	MMP9	A_23_P40174	NM_004994	2.2	2.02E-02	2.2	2.02E-02	2.8	3.97E-02	2.0	1.07E-02	1.7	7.83E-03	1.5	7.83E-03	1.8	1.63E-02	1.7	5.87E-03	0.0	1.0E+00	0.0	1.0E+00	0.2	7.1E-01	0.0	1.0E+00	0.1	9.5E-01	0.3	1.0E+00	0.1	9.5E-01	0.1	9.5E-01
32961	HIST1H4I	A_24_P20873	NM_003495	2.2	4.71E-05	2.3	4.71E-05	1.5	4.26E-03	2.2	4.67E-06	2.0	1.33E-03	1.9	1.33E-03	2.0	1.02E-02	2.5	6.67E-05	0.3	1.0E+00	0.3	1.0E+00	0.3	7.1E-01	0.2	1.0E+00	-0.1	9.6E-01	0.0	1.0E+00	-0.1	8.6E-01	-0.1	9.6E-01
30664	LOC643201	A_24_P526190	BC034407	2.2	1.10E-03	1.6	1.29E-03	2.6	1.10E-03	2.2	2.71E-05	1.8	1.17E-03	1.2	2.43E-02	2.3	8.03E-04	1.8	1.17E-03	0.1	1.0E+00	0.0	1.0E+00	0.1	8.5E-01	0.2	1.0E+00	-0.2	6.4E-01	-0.1	1.0E+00	-0.2	4.0E-01	-0.2	6.4E-01
10564	RAB37	A_23_P414654	NM_175738	2.2	5.69E-05	2.2	1.16E-02	3.7	5.69E-05	2.1	1.33E-06	2.0	9.62E-03	1.9	4.63E-03	2.3	1.53E-02	2.0	9.62E-03	0.1	1.0E+00	0.1	1.0E+00	-0.1	7.9E-01	0.1	1.0E+00	-0.4	6.1E-01	-0.2	1.0E+00	-0.4	3.6E-01	-0.4	6.1E-01
22458	PGF	A_23_P76992	NM_002632	2.2	3.10E-05	2.1	3.10E-05	2.2	5.36E-04	2.2	1.76E-06	1.6	5.35E-02	1.6	5.35E-02	1.5	7.47E-02	2.2	3.50E-03	0.1	1.0E+00	0.1	1.0E+00	0.3	7.3E-01	0.1	1.0E+00	0.3	5.2E-01	0.2	1.0E+00	0.3	5.2E-01	0.3	5.2E-01
17716	ENST00000381655	A_24_P944714	ENST00000381655	2.2	1.71E-04	2.2	1.71E-04	2.4	1.24E-03	2.1	1.33E-05	1.8	4.09E-03	1.8	1.29E-02	1.8	4.09E-03	2.2	2.16E-03	0.1	1.0E+00	0.1	1.0E+00	0.1	8.7E-01	0.2	1.0E+00	-0.2	8.4E-01	-0.1	1.0E+00	-0.2	7.2E-01	-0.3	8.4E-01
14981	CFTR	A_23_P215720	NM_000492	2.2	5.73E-05	2.4	5.73E-05	1.0	1.16E-02	2.2	2.20E-06	2.3	7.42E-04	2.3	8.10E-04	1.8	7.42E-04	2.3	2.92E-05	0.2	1.0E+00	0.2	1.0E+00	0.0	9.9E-01	0.2	1.0E+00	-0.1	8.8E-01	0.0	1.0E+00	-0.2	5.9E-01	-0.1	8.8E-01
30528	ECM1	A_23_P160559	NM_004425	2.2	4.15E-04	1.4	6.55E-03	2.3	4.15E-04	2.2	1.92E-06	1.2	1.26E-02	0.8	3.63E-02	1.2	1.26E-02	1.7	1.95E-03	0.0	1.0E+00	0.1	1.0E+00	-0.2	7.1E-01	0.0	1.0E+00	-0.1	9.9E-01	-0.1	1.0E+00	-0.2	4.9E-01	0.0	9.9E-01
43513	HOXD1	A_23_P120243	NM_024501	2.2	5.02E-04	2.2	5.11E-04	1.8	5.02E-04	2.4	1.49E-07	1.2	1.69E-01	1.2	1.69E-01	1.1	1.95E-01	1.7	2.67E-02	0.1	1.0E+00	0.0	1.0E+00	0.2	7.1E-01	0.1	1.0E+00	-0.1	7.5E-01	-0.1	1.0E+00	-0.1	7.2E-01	-0.2	7.5E-01
38808	HIST1H3B	A_23_P93258	NM_003537	2.2	1.70E-04	2.3	1.70E-04	2.1	4.01E-03	2.2	3.43E-05	1.9	1.54E-04	1.5	4.22E-03	1.9	1.54E-04	2.1	6.67E-05	-0.1	1.0E+00	-0.1	1.0E+00	0.0	9.9E-01	-0.1	1.0E+00	-0.1	9.9E-01	-0.1	1.0E+00	0.0	9.9E-01	-0.1	9.8E-01
7672	HIST1H3D	A_24_P217834	NM_003530	2.2	1.46E-04	2.2	1.46E-04	2.0	4.16E-03	2.2	3.25E-05	2.1	5.09E-04	1.7	2.84E-03	2.1	2.66E-04	2.2	5.09E-04	0.6	1.0E+00	0.6	1.0E+00	0.6	7.1E-01	0.8	1.0E+00	-0.1	9.7E-01	-0.1	1.0E+00	0.1	9.6E-01	-0.1	9.7E-01
37581	HIST1H4F	A_23_P359540	NM_003540	2.2	2.52E-05	2.4	2.52E-05	1.7	9.11E-04	2.2	6.63E-07	2.0	1.17E-03	1.7	1.17E-03	2.0	4.21E-03	2.4	1.84E-04	0.2	1.0E+00	0.2	1.0E+00	0.2	7.1E-01	0.1	1.0E+00	0.1	9.8E-01	0.1	1.0E+00	0.1	9.4E-01	0.0	9.8E-01
21363	MMP9	A_23_P40174	NM_004994	2.2	2.06E-02	2.2	2.06E-02	3.0	3.37E-02	2.2	7.41E-03	1.8	6.08E-03	1.7	6.08E-03	2.2	9.71E-03	1.8	4.93E-03	0.1	1.0E+00	0.1	1.0E+00	0.1	8.8E-01	0.0	1.0E+00	0.1	9.6E-01	0.4	1.0E+00	0.1	9.6E-01	0.1	9.6E-01
11761	GMFG	A_23_P208866	NM_004877	2.2	2.49E-03	1.5	5.06E-03	2.9	2.49E-03	2.2	1.30E-04	1.6	2.61E-03	1.2	2.61E-03	1.9	6.55E-03	1.6	1.16E-04	0.2	1.0E+00	0.2	1.0E+00	0.2	7.4E-01	0.1	1.0E+00	0.0	9.7E-01	0.1	1.0E+00	0.0	9.5E-01	0.0	9.7E-01
28675	MMP9	A_23_P40174	NM_004994	2.2	1.77E-02	2.2	1.77E-02	2.9	3.67E-02	2.2	7.84E-03	1.8	1.41E-02	1.6	1.86E-02	2.2	1.41E-02	1.8	7.86E-03	0.1	1.0E+00	0.1	1.0E+00	0.1	7.4E-01	0.1	1.0E+00	0.1	9.7E-01	0.3	1.0E+00	0.0	9.7E-01	0.1	9.4E-01
5137	IRX4	A_23_P110837	NM_016358	2.2	4.29E-05	2.0	4.29E-05	2.2	3.06E-04	2.3	7.20E-07	1.7	1.52E-03	1.5	5.94E-03	1.7	1.52E-03	1.9	3.36E-04	0.3	4.5E-01	0.3	3.9E-01	0.2	7.1E-01	0.4	4.5E-01	-0.3	2.1E-01	0.0	1.0E+00	-0.3	6.3E-02	-0.3	2.1E-01
32294	ENST00000293201	A_24_P369898	ENST00000293201	2.2	2.90E-03	2.2	2.90E-03	2.9	6.45E-03	1.8	2.21E-03	1.7	2.73E-03	1.6	1.09E-02	1.8	2.73E-03	1.7	4.81E-04	0.1	1.0E+00	0.1	1.0E+00	0.1	7.8E-01	0.2	1.0E+00	0.2	9.2E-01	0.3	1.0E+00	0.0	9.2E-01	0.2	7.5E-01
19280	GPR162	A_23_P99141	NM_019858	2.2	5.81E-04	2.2	9.43E-04	3.0	5.81E-04	2.1	1.67E-05	2.0	6.33E-03	1.8	8.46E-03	2.0	6.33E-03	2.1	1.70E-03	0.2	1.0E+00	0.3	1.0E+00	0.1	8.5E-01	0.2	1.0E+00	-0.4	8.5E-01	-0.2	1.0E+00	-0.4	6.6E-01	-0.4	8.5E-01
12799	ANTXR2	A_23_P170733	NM_058172	2.2	1.26E-03	2.2	1.26E-03	1.9	4.48E-03	2.2	2.51E-05	2.0	6.34E-03	2.0	5.08E-03	1.5	6.34E-03	2.0	9.56E-03	0.1	1.0E+00	0.1	1.0E+00	0.1	8.5E-01	0.3	1.0E+00	-0.1	8.4E-01	0.1	1.0E+00	-0.2	4.7E-01	-0.1	8.4E-01
28592	MMP9	A_23_P40174	NM_004994	2.2	1.88E-02	2.2	1.88E-02	2.9	3.85E-02	2.2	8.57E-03	1.8	1.72E-02	1.7	2.57E-02	2.2	1.72E-02	1.8	1.13E-02	0.1	1.0E+00	0.1	1.0E+00	0.1	8.3E-01	0.1	1.0E+00	0.1	9.4E-01	0.3	1.0E+00	0.1	9.3E-01	0.1	9.4E-01
39244	FAM25C	A_32_P34138	NM_001137548	2.2	4.90E-05	1.6	7.05E-03	3.3	4.90E-05	2.2	3.41E-07	1.2	2.28E-02	0.6	1.33E-01	1.4	2.28E-02	1.2	9.03E-03	0.1	1.0E+00	0.1	1.0E+00	0.0	9.6E-01	0.1	1.0E+00	-0.3	7.5E-01	-0.1	1.0E+00	-0.3	3.6E-01	-0.3	7.5E-01
9339	FCGBP	A_23_P21495	NM_003890	2.2	3.25E-04	1.6	3.25E-04	2.4	1.04E-03	2.2	7.21E-06	1.4	1.04E-02	0.9	2.24E-02	1.4	1.04E-02	1.7	1.53E-04	0.1	1.0E+00	-0.1	1.0E+00	0.2	7.3E-01	0.1	1.0E+00	0.2	6.7E-01	0.2	1.0E+00	0.2	3.5E-01	0.2	6.7E-01
33910	HIST1H3F	A_23_P30799	NM_021018	2.2	2.90E-04	2.2	2.90E-04	2.0	7.39E-03	2.2	6.87E-05	2.1	5.12E-04	1.7	6.27E-03	2.1	5.12E-04	2.4	2.15E-04	0.4	1.0E+00	0.4	1.0E+00	0.4	7.1E-01	0.5	1.0E+00	0.1	9.6E-01	0.1	1.0E+00	0.2	8.1E-01	0.1	9.6E-01
23427	NPPC	A_24_P174353	ENST00000409852	2.2	1.04E-03	2.2	1.04E-03	1.7	4.84E-03	2.8	3.54E-06	2.4	8.28E-04	2.5	8.28E-04	1.8	7.16E-03	2.4	9.12E-05	0.2	1.0E+00	0.2	1.0E+00	-0.3	7.1E-01	0.5	1.0E+00	-0.3	4.9E-01	-0.1	1.0E+00	-0.5	4.6E-02	-0.3	4.9E-01
9933	HIC1	A_23_P129856	NM_006497	2.2	5.31E-05	2.2	5.31E-05	2.3	7.85E-04	2.0	7.84E-06	2.0	1.64E-03	2.0	1.64E-03	1.7	3.54E-03	2.2	2.51E-04	0.2	1.0E+00	0.2	1.0E+00	0.3	7.1E-01	0.2	1.0E+00	0.1	8.9E-01	0.0	1.0E+00	0.1	6.3E-01	0.1	8.9E-01
43123	ENST00000285206	A_23_P421843	ENST00000285206	2.2	6.52E-05	2.2	1.69E-04	3.0	6.52E-05	2.2	3.41E-07	2.1	8.74E-04	1.7	8.74E-04	2.1	3.37E-03	2.1	5.07E-04	0.1	1.0E+00	0.1	1.0E+00	0.1	7.1E-01	0.1	1.0E+00	0.0	1.0E+00	0.0	1.0E+00	-0.1	7.3E-01	0.0	1.0E+00
29729	HIST1H1E	A_23_P7976	NM_005321	2.3	8.52E-05	2.3	8.52E-05	2.3	1.82E-03	2.2	1.09E-05	2.2	1.24E-03	1.9	1.74E-02	2.2	1.24E-03	2.3	3.61E-04	0.1	1.0E+00	0.2	1.0E+00	0.1	9.0E-01	0.1	1.0E+00	-0.1	9.9E-01	-0.1	1.0E+00	0.0	9.9E-01	-0.1	9.8E-01
34166	FAM148B	A_24_P788878	NM_001007595	2.3	1.16E-04	2.3	4.18E-04	2.3	1.16E-04	2.3	1.49E-07	1.7	4.96E-03	1.3	4.96E-03	1.7	1.72E-03	1.7	1.51E-02	0.1	1.0E+00	0.0	1.0E+00	0.1	8.2E-01	0.1	1.0E+00	-0.1	9.2E-01	0.0	1.0E+00	-0.1	5.9E-01	-0.1	9.2E-01
33067	THC2683228	A_32_P53603	THC2683228	2.3	2.37E-04	2.3	2.37E-04	2.3	2.45E-03	2.1	9.42E-05	1.4	2.63E-01	0.8	6.90E-01	1.6	2.63E-01	1.4	2.14E-01	0.0	1.0E+00	0.0	1.0E+00	0.4	7.6E-01	-0.2	1.0E+00	0.5	5.7E-01	0.1	1.0E+00	0.5	5.7E-01	1.1	1.1E-01
38078	KRT20	A_23_P66854	NM_019010	2.3	1.83E-04	2.6	1.83E-04	1.2	4.71E-02	2.3	7.77E-05	2.6	1.47E-03	2.8	1.47E-03	1.8	1.88E-02	2.6	4.45E-04	0.5	3.7E-01	0.5	1.2E-01	0.3	7.1E-01	0.5	3.7E-01	-0.2	8.8E-01	0.1	1.0E+00	-0.2	5.6E-01	-0.2	8.8E-01
22115	A_24_P152345	A_24_P152345	A_24_P152345	2.3	1.35E-04	2.3	1.35E-04	1.4	4.53E-03	2.3	3.33E-06	2.5	6.23E-03	2.5	6.23E-03	2.2	3.19E-02	2.8	1.56E-03	1.3	2.4E-02	1.1	2.4E-02	1.3	5.8E-03	1.5	3.7E-02	-0.1	9.5E-01	0.0	1.0E+00	-0.2	7.6E-01	-0.1	9.5E-01
43041	B3GNT3	A_23_P78980	NM_014256	2.3	9.25E-04	2.2	9.25E-04	2.8	4.68E-03	2.3	2.13E-04	2.0	5.77E-04	1.9	5.77E-04	2.3	2.28E-03	2.0	4.81E-04	0.2	1.0E+00	0.2	1.0E+00	0.2	7.1E-01	0.3	1.0E+00	0.1	9.7E-01	0.2	1.0E+00	0.1	9.5E-01	0.1	9.7E-01
29825	CFTR	A_23_P215720	NM_000492	2.3	4.41E-05	2.4	4.41E-05	1.0	1.06E-02	2.3	1.43E-06	2.3	5.38E-04	2.4	5.38E-04	1.8	9.13E-04	2.3	1.20E-05	0.1	1.0E+00	0.1	1.0E+00	0.1	8.2E-01	0.1	1.0E+00	0.0	9.6E-01	0.0	1.0E+00	-0.1	6.7E-01	0.0	9.6E-01
32255	SOX8	A_23_P66137	NM_014587	2.3	3.57E-03	2.3	3.79E-03	3.6	3.57E-03	2.1	9.79E-04	2.0	7.19E-02	1.2	2.14E-01	2.0	7.19E-02	2.2	4.02E-02	0.1	1.0E+00	0.1	1.0E+00	0.2	7.7E-01	0.1	1.0E+00	-0.1	9.9E-01	-0.2	1.0E+00	-0.1	9.6E-01	0.0	9.9E-01
17843	CRYGC	A_23_P142606	NM_020989	2.3	1.38E-04	2.1	7.06E-04	2.3	1.38E-04	2.5	1.94E-07	1.8	3.07E-03	1.0	4.21E-02	1.8	3.07E-03	1.8	9.82E-04	0.0	1.0E+00	0.0	1.0E+00	0.0	8.9E-01	0.1	1.0E+00	0.0	9.6E-01	0.0	1.0E+00	0.0	9.6E-01	0.0	9.6E-01
15950	CFTR	A_23_P215720	NM_000492	2.3	2.52E-05	2.5	2.52E-05	1.1	1.20E-02	2.3	1.87E-06	2.4	5.12E-04	2.4	6.70E-04	1.9	5.12E-04	2.4	1.19E-05	0.1	1.0E+00	0.1	1.0E+00	0.1	7.4E-01	0.2	1.0E+00	-0.1	7.9E-01	0.1	1.0E+00	-0.2	3.2E-01	-0.1	7.9E-01
43677	HIST1H2AM	A_24_P86389	NM_003514	2.3	1.48E-04	2.4	1.48E-04	2.3	1.13E-03	2.1	2.33E-05	1.9	1.24E-03	1.9	4.36E-03	1.9	1.24E-03	2.3	3.49E-04	0.2	1.0E+00	0.2	1.0E+00	0.2	7.6E-01	0.2	1.0E+00	-0.2	9.0E-01	0.1	1.0E+00	-0.2	8.0E-01	-0.2	9.0E-01
22249	NTNG2	A_23_P423331	NM_032536	2.3	2.77E-03	2.3	3.10E-03	4.2	6.45E-04	1.6	2.77E-03	2.6	5.95E-03	2.6	5.95E-03	3.1	1.43E-02	2.3	3.35E-03	0.2	1.0E+00	0.2	1.0E+00	0.2	8.1E-01	0.4	1.0E+00	-0.3	8.7E-01	-0.2	1.0E+00	-0.3	6.2E-01	-0.3	8.7E-01
42637	FXYD2	A_23_P161769	NM_021603	2.3	3.60E-05	2.0	2.78E-04	3.2	3.60E-05	2.3	1.48E-07	1.4	1.63E-01	0.6	6.54E-01	1.4	1.63E-01	1.4	6.39E-02	0.0	1.0E+00	0.0	1.0E+00	0.3	7.1E-01	0.0	1.0E+00	0.0	1.0E+00	0.0	1.0E+00	0.0	9.7E-01	0.0	1.0E+00
17372	CFTR	A_23_P215720	NM_000492	2.3	5.92E-05	2.5	5.92E-05	1.1	1.72E-02	2.3	5.92E-06	2.2	1.63E-03	2.3	2.29E-03	1.9	1.63E-03	2.2	2.47E-04	0.2	1.0E+00	0.2	1.0E+00	0.1	8.5E-01	0.2	1.0E+00	-0.1	8.8E-01	0.1	1.0E+00	-0.1	5.5E-01	-0.1	8.8E-01
3984	THC2496620	A_24_P683829	THC2496620	2.3	1.24E-04	2.1	1.24E-04	2.3	2.27E-04	2.5	1.69E-06	2.2	3.05E-04	2.2	5.38E-04	1.9	3.05E-04	2.2	4.33E-05	0.6	2.5E-01	0.6	2.0E-01	0.5	4.7E-01	0.7	2.5E-01	-0.2	8.2E-01	0.0	1.0E+00	-0.2	6.9E-01	-0.2	8.2E-01
25451	FAM46C	A_23_P137751	NM_017709	2.3	2.19E-05	1.6	3.19E-04	2.9	2.19E-05	2.3	1.38E-07	1.4	5.24E-03	1.3	1.02E-02	1.4	5.24E-03	1.7	2.40E-03	0.0	1.0E+00	0.1	1.0E+00	-0.2	7.1E-01	0.0	1.0E+00	-0.2	6.3E-01	-0.1	1.0E+00	-0.3	2.1E-01	-0.2	6.3E-01
34994	ATP8A2	A_23_P258612	NM_016529	2.3	2.08E-04	1.9	6.40E-03	2.9	2.08E-04	2.3	2.30E-06	1.5	5.10E-03	1.4	2.10E-02	1.5	5.10E-03	1.9	2.88E-03	0.0	1.0E+00	0.0	1.0E+00	-0.1	8.5E-01	0.3	1.0E+00	-0.4	4.2E-01	-0.1	1.0E+00	-0.4	1.8E-01	-0.4	4.2E-01
23547	KRT34	A_23_P101054	NM_021013	2.3	3.06E-04	1.7	7.35E-04	3.2	3.06E-04	2.3	4.67E-06	2.0	1.14E-03	1.8	5.68E-03	2.0	1.14E-03	2.0	9.77E-04	0.4	7.8E-01	0.4	7.8E-01	0.2	8.0E-01	0.6	6.0E-01	0.0	9.8E-01	0.0	1.0E+00	-0.1	8.0E-01	0.0	9.8E-01
28948	SRPX2	A_23_P136978	NM_014467	2.3	2.70E-05	1.7	1.27E-03	2.8	2.70E-05	2.3	3.73E-08	1.2	2.48E-02	0.8	1.73E-01	1.4	1.94E-02	1.2	2.48E-02	0.1	1.0E+00	0.1	1.0E+00	-0.1	8.0E-01	0.3	1.0E+00	-0.2	7.8E-01	-0.2	1.0E+00	-0.2	4.1E-01	-0.2	7.8E-01
39351	MGC42105	A_23_P254863	NM_153361	2.3	1.79E-05	1.7	1.07E-03	3.3	1.79E-05	2.3	4.70E-08	0.9	7.15E-03	0.5	2.07E-01	0.9	7.15E-03	1.3	4.42E-03	0.0	1.0E+00	0.0	1.0E+00	0.0	9.4E-01	-0.1	1.0E+00	0.0	9.9E-01	0.0	1.0E+00	0.0	9.9E-01	0.0	9.8E-01
31032	AK124097	A_32_P202798	AK124097	2.3	2.28E-04	1.5	4.32E-03	2.3	2.28E-04	2.3	3.84E-07	0.7	1.44E-01	0.7	1.44E-01	0.5	1.53E-01	1.5	5.25E-03	0.1	1.0E+00	0.1	1.0E+00	-0.4	7.1E-01	0.1	1.0E+00	-0.2	4.5E-01	0.0	1.0E+00	-0.2	3.6E-01	-0.3	4.5E-01
43399	CABYR	A_23_P314712	NM_012189	2.3	1.26E-03	2.1	1.26E-03	2.3	1.80E-03	2.5	1.13E-05	1.7	2.52E-03	1.1	1.33E-02	1.7	2.52E-03	1.7	7.72E-04	0.2	1.0E+00	0.2	1.0E+00	0.1	8.3E-01	0.2	1.0E+00	-0.3	7.2E-01	-0.2	1.0E+00	-0.3	5.0E-01	-0.3	7.2E-01
38848	LAMA2	A_23_P70719	NM_000426	2.3	3.66E-06	2.8	3.09E-06	1.3	4.35E-03	2.3	3.66E-06	2.4	1.39E-03	2.4	1.39E-03	1.5	2.97E-02	2.6	9.99E-05	0.0	1.0E+00	0.0	1.0E+00	0.0	9.3E-01	0.2	1.0E+00	-0.1	9.3E-01	0.1	1.0E+00	-0.1	9.2E-01	-0.1	9.3E-01
5384	LOC729085	A_23_P69154	NM_001129908	2.3	1.26E-03	2.7	2.73E-03	1.9	1.26E-03	2.3	2.10E-06	1.7	4.36E-03	1.7	4.36E-03	0.9	1.77E-02	2.4	3.73E-03	0.2	1.0E+00	0.2	1.0E+00	-0.2	7.1E-01	0.2	1.0E+00	-0.2	5.2E-01	-0.1	1.0E+00	-0.2	2.7E-01	-0.2	5.2E-01
39320	MMP1	A_23_P1691	NM_002421	2.4	3.97E-04	2.4	5.94E-03	3.2	3.97E-04	2.1	1.10E-05	1.4	2.21E-02	1.4	2.21E-02	1.4	6.41E-03	1.8	3.91E-02	-0.1	1.0E+00	0.0	1.0E+00	-0.1	7.8E-01	-0.3	1.0E+00	-0.3	7.3E-01	-0.2	1.0E+00	-0.3	5.0E-01	-0.3	7.3E-01
452	KIRREL2	A_23_P315451	NM_199180	2.4	4.12E-05	2.4	5.73E-05	2.4	4.12E-05	2.3	2.29E-07	1.9	3.90E-03	1.8	8.32E-03	1.9	3.90E-03	2.0	2.04E-03	0.1	1.0E+00	0.1	1.0E+00	-0.1	8.3E-01	0.2	1.0E+00	-0.3	3.6E-01	-0.1	1.0E+00	-0.3	2.0E-01	-0.3	3.6E-01
40070	BC042589	A_32_P94685	BC042589	2.4	7.88E-05	2.4	7.88E-05	2.9	8.77E-04	2.3	2.58E-05	2.2	1.66E-03	2.1	1.71E-02	2.3	1.66E-03	2.2	9.37E-04	1.7	9.4E-04	1.7	9.4E-04	1.7	9.2E-05	1.4	4.4E-02	0.1	9.9E-01	0.3	1.0E+00	0.0	9.9E-01	0.1	9.5E-01
11864	SGK1	A_23_P19673	NM_005627	2.4	8.15E-04	2.4	8.15E-04	2.3	5.23E-03	2.7	3.08E-05	1.7	2.85E-03	1.7	2.85E-03	1.5	3.82E-02	2.4	2.40E-04	0.2	1.0E+00	0.0	1.0E+00	0.2	7.1E-01	0.2	1.0E+00	-0.1	8.6E-01	0.1	1.0E+00	-0.1	8.6E-01	-0.1	7.8E-01
21280	TNXB	A_23_P156708	NM_032470	2.4	1.50E-04	2.4	1.74E-03	3.0	1.50E-04	2.2	8.71E-07	2.1	7.34E-03	1.8	7.34E-03	2.2	1.10E-02	2.1	4.22E-03	0.0	1.0E+00	0.0	1.0E+00	0.0	9.7E-01	0.2	1.0E+00	-0.1	6.4E-01	0.1	1.0E+00	-0.1	5.1E-01	-0.2	6.4E-01
15065	A_24_P913576	A_24_P913576	A_24_P913576	2.4	5.24E-05	1.3	4.66E-02	3.1	5.24E-05	2.4	1.63E-07	1.0	4.94E-02	0.7	4.94E-02	1.3	3.37E-02	1.0	1.91E-01	0.0	1.0E+00	-0.1	1.0E+00	0.0	9.9E-01	0.1	1.0E+00	-0.1	9.3E-01	0.0	1.0E+00	-0.1	8.2E-01	-0.1	9.3E-01
30343	MYH7	A_24_P282383	NM_000257	2.4	4.23E-05	2.3	9.98E-04	2.8	4.23E-05	2.4	7.18E-08	1.8	4.38E-03	1.2	2.52E-02	2.1	4.38E-03	1.8	2.90E-03	0.1	1.0E+00	0.0	1.0E+00	0.1	8.6E-01	0.1	1.0E+00	-0.2	9.3E-01	-0.2	1.0E+00	-0.2	7.9E-01	-0.2	9.3E-01
33107	C18orf19	A_23_P380978	NM_152352	2.4	2.71E-05	1.9	2.71E-05	2.4	3.52E-04	2.5	6.78E-07	1.3	9.42E-03	1.3	9.42E-03	1.0	8.81E-02	2.3	9.01E-05	0.1	1.0E+00	0.1	1.0E+00	-0.1	7.3E-01	0.2	1.0E+00	0.1	9.2E-01	0.1	1.0E+00	-0.1	8.3E-01	0.1	9.2E-01
27725	DPY19L2P2	A_32_P78385	BC094802	2.4	2.52E-05	1.5	2.52E-05	3.0	3.08E-05	2.4	1.51E-07	1.3	1.89E-01	0.6	7.19E-01	1.4	1.89E-01	1.3	1.40E-01	-0.1	1.0E+00	-0.1	1.0E+00	-0.2	7.3E-01	0.0	1.0E+00	-0.1	6.5E-01	0.0	1.0E+00	-0.2	2.7E-01	-0.1	6.5E-01
3475	MMP1	A_23_P1691	NM_002421	2.4	5.03E-04	2.4	5.78E-03	3.2	5.03E-04	2.1	2.04E-05	1.3	4.00E-02	1.2	3.18E-02	1.3	4.00E-02	1.7	6.16E-02	-0.1	1.0E+00	0.0	1.0E+00	-0.1	8.0E-01	-0.3	1.0E+00	-0.1	9.1E-01	-0.1	1.0E+00	-0.1	8.2E-01	-0.2	9.1E-01
40097	AQP3	A_23_P112482	NM_004925	2.4	2.37E-04	2.6	2.37E-04	2.3	2.97E-03	2.4	2.31E-05	2.1	1.18E-02	2.1	1.18E-02	2.0	1.63E-02	2.6	5.73E-04	0.2	1.0E+00	0.2	1.0E+00	0.1	7.4E-01	0.3	1.0E+00	-0.1	9.8E-01	0.1	1.0E+00	-0.3	7.5E-01	-0.1	9.8E-01
37039	SLPI	A_24_P190472	NM_003064	2.4	1.49E-02	2.2	1.49E-02	2.7	2.02E-02	2.4	1.20E-03	1.6	2.12E-03	1.4	3.26E-02	1.6	1.84E-03	2.0	2.12E-03	0.1	1.0E+00	0.1	1.0E+00	0.1	7.3E-01	0.1	1.0E+00	0.0	1.0E+00	0.3	1.0E+00	-0.1	9.3E-01	0.0	1.0E+00
19605	EEF1A2	A_23_P256033	NM_001958	2.4	5.20E-04	2.4	5.20E-04	2.3	3.82E-03	2.7	1.69E-05	1.9	3.27E-03	1.8	8.24E-03	1.9	3.27E-03	2.4	7.97E-04	0.1	1.0E+00	0.3	1.0E+00	0.1	8.2E-01	0.1	1.0E+00	0.2	6.6E-01	0.2	1.0E+00	0.2	6.1E-01	0.3	6.6E-01
40767	MMP1	A_23_P1691	NM_002421	2.4	4.75E-04	2.4	7.23E-03	3.1	4.75E-04	2.0	1.86E-05	1.4	2.64E-02	1.3	2.64E-02	1.4	1.09E-02	1.7	4.88E-02	-0.2	1.0E+00	0.0	1.0E+00	-0.2	7.1E-01	-0.3	1.0E+00	-0.3	7.4E-01	-0.2	1.0E+00	-0.3	6.5E-01	-0.3	7.4E-01
6423	NXF3	A_23_P171336	NM_022052	2.4	1.11E-02	1.7	1.37E-02	2.7	1.11E-02	2.4	4.66E-04	1.1	3.58E-01	0.6	7.74E-01	1.1	3.58E-01	1.2	2.34E-01	-0.2	1.0E+00	-0.3	1.0E+00	0.6	7.1E-01	-0.2	1.0E+00	0.6	1.1E-01	0.2	1.0E+00	0.7	2.0E-02	0.6	1.1E-01
19676	FBP2	A_23_P94472	NM_003837	2.4	2.27E-05	2.0	2.27E-05	2.7	2.70E-05	2.4	1.53E-07	1.9	2.16E-04	1.5	4.00E-03	2.2	8.06E-05	1.9	2.16E-04	0.1	1.0E+00	0.1	1.0E+00	0.1	7.1E-01	0.3	1.0E+00	0.0	1.0E+00	0.0	1.0E+00	-0.1	5.8E-01	0.0	1.0E+00
6055	THC2633920	A_24_P746314	THC2633920	2.4	8.96E-05	1.6	4.55E-04	2.6	8.96E-05	2.4	6.63E-07	1.5	1.26E-01	1.1	3.03E-01	1.5	1.26E-01	1.5	6.24E-02	0.2	1.0E+00	0.1	1.0E+00	0.4	7.1E-01	0.2	1.0E+00	-0.1	8.7E-01	-0.1	1.0E+00	-0.1	8.1E-01	-0.2	8.7E-01
1866	CD52	A_23_P85800	NM_001803	2.4	1.24E-03	2.2	5.23E-03	3.9	1.24E-03	2.4	1.17E-04	2.0	7.97E-03	1.5	7.97E-03	2.4	3.68E-03	2.0	2.01E-02	-0.1	1.0E+00	0.0	1.0E+00	-0.2	7.5E-01	-0.1	1.0E+00	0.1	9.3E-01	0.2	1.0E+00	-0.1	9.3E-01	0.1	9.2E-01
39863	HIST1H3D	A_23_P219045	NM_003530	2.4	8.72E-05	2.4	8.72E-05	2.4	2.87E-03	2.6	1.56E-05	2.5	4.06E-04	2.1	2.63E-03	2.5	1.07E-04	2.6	4.06E-04	0.7	1.0E+00	0.7	1.0E+00	0.7	7.1E-01	1.0	1.0E+00	0.6	9.3E-01	0.6	1.0E+00	0.8	7.6E-01	0.5	9.3E-01
12634	LY96	A_23_P94230	NM_015364	2.4	2.89E-04	2.0	4.06E-03	3.3	2.89E-04	2.4	1.74E-05	2.1	8.17E-03	1.5	4.02E-02	2.1	8.17E-03	2.1	1.68E-03	0.0	1.0E+00	0.0	1.0E+00	0.0	9.9E-01	0.1	1.0E+00	-0.3	7.9E-01	-0.2	1.0E+00	-0.3	6.5E-01	-0.4	7.9E-01
22571	DNAH3	A_23_P329340	NM_017539	2.4	1.93E-05	2.4	1.93E-05	2.4	1.47E-04	3.1	4.31E-08	2.6	5.08E-03	1.9	5.08E-03	2.6	8.99E-03	2.7	1.19E-04	0.1	1.0E+00	0.1	1.0E+00	0.1	9.2E-01	0.3	1.0E+00	-0.3	7.0E-01	-0.1	1.0E+00	-0.3	3.9E-01	-0.3	7.0E-01
43824	SLC26A11	A_23_P125078	NM_173626	2.4	1.65E-03	2.1	1.65E-03	2.4	2.10E-03	2.5	1.22E-05	1.9	1.02E-03	1.9	7.50E-04	1.4	1.10E-02	2.1	1.02E-03	0.1	1.0E+00	0.1	1.0E+00	-0.1	9.3E-01	0.2	1.0E+00	-0.3	7.3E-01	-0.2	1.0E+00	-0.4	4.2E-01	-0.3	7.3E-01
38296	DLL1	A_23_P167920	NM_005618	2.4	8.34E-04	2.2	1.07E-03	2.8	8.34E-04	2.4	7.27E-06	1.8	6.70E-03	1.8	6.70E-03	1.8	1.73E-03	2.0	8.13E-03	0.0	1.0E+00	0.1	1.0E+00	-0.2	8.0E-01	0.0	1.0E+00	-0.3	4.9E-01	-0.1	1.0E+00	-0.3	4.2E-01	-0.4	4.9E-01
33784	CHGA	A_32_P27046	NM_001275	2.5	3.19E-04	2.5	3.19E-04	2.9	9.71E-04	2.0	4.32E-05	1.3	2.56E-01	0.8	5.73E-01	1.3	2.56E-01	1.9	6.09E-02	0.1	1.0E+00	0.1	1.0E+00	0.1	8.9E-01	-0.1	1.0E+00	0.3	5.2E-01	0.2	1.0E+00	0.3	5.2E-01	0.5	4.9E-01
38540	MMP1	A_23_P1691	NM_002421	2.5	4.27E-04	2.5	3.83E-03	3.2	4.27E-04	2.2	9.96E-06	1.4	1.86E-02	1.3	1.86E-02	1.4	7.57E-03	1.9	3.49E-02	-0.4	1.0E+00	0.0	1.0E+00	-0.4	4.9E-01	-0.4	1.0E+00	-0.3	8.0E-01	-0.1	1.0E+00	-0.3	6.0E-01	-0.3	8.0E-01
24357	CX3CL1	A_23_P37727	NM_002996	2.5	5.35E-04	2.1	6.92E-04	3.2	5.35E-04	2.5	6.97E-06	1.9	3.52E-03	1.7	5.95E-03	2.2	2.00E-03	1.9	3.52E-03	0.2	1.0E+00	0.1	1.0E+00	0.2	7.3E-01	0.3	1.0E+00	-0.1	9.5E-01	-0.1	1.0E+00	0.0	9.5E-01	-0.1	9.2E-01
27596	PROCR	A_23_P80040	NM_006404	2.5	1.50E-04	1.7	1.50E-04	2.9	3.96E-04	2.5	2.78E-06	1.4	2.09E-03	1.0	6.75E-03	1.4	2.09E-03	1.6	1.98E-03	0.1	1.0E+00	0.3	1.0E+00	0.1	8.5E-01	0.0	1.0E+00	-0.2	9.4E-01	-0.1	1.0E+00	-0.2	8.0E-01	-0.2	9.4E-01
40394	EPPK1	A_24_P357169	NM_031308	2.5	1.30E-04	2.4	1.30E-04	3.0	1.25E-03	2.5	2.90E-05	2.4	1.37E-03	2.5	9.10E-04	2.1	3.27E-03	2.4	1.37E-03	0.2	1.0E+00	0.4	7.0E-01	0.0	1.0E+00	0.2	1.0E+00	0.1	9.9E-01	0.1	1.0E+00	0.0	9.9E-01	0.1	9.7E-01
34154	XAGE1D	A_24_P271696	NM_133431	2.5	6.67E-05	2.5	6.67E-05	1.6	2.47E-03	2.6	7.09E-07	2.6	1.72E-03	2.7	4.63E-03	2.1	1.72E-03	2.6	2.69E-04	0.1	1.0E+00	0.1	1.0E+00	-0.1	9.2E-01	0.3	1.0E+00	-0.4	2.7E-01	0.0	1.0E+00	-0.4	9.5E-02	-0.4	2.7E-01
1054	VWF	A_23_P105562	NM_000552	2.5	3.65E-04	2.3	4.80E-03	3.7	3.65E-04	2.5	1.11E-05	2.0	5.01E-03	1.5	2.00E-02	2.2	5.01E-03	2.0	3.47E-03	0.3	1.0E+00	0.1	1.0E+00	0.4	6.7E-01	0.3	1.0E+00	0.0	9.6E-01	0.0	1.0E+00	0.0	9.4E-01	0.0	9.6E-01
816	VWF	A_23_P105562	NM_000552	2.5	3.65E-04	2.2	3.64E-03	3.8	3.65E-04	2.5	1.12E-05	1.9	6.41E-03	1.4	1.42E-02	2.1	6.41E-03	1.9	3.96E-03	0.1	1.0E+00	0.0	1.0E+00	0.3	7.1E-01	0.1	1.0E+00	0.1	8.3E-01	0.0	1.0E+00	0.1	8.3E-01	0.2	7.4E-01
8105	VWF	A_23_P105562	NM_000552	2.5	3.19E-04	2.3	5.21E-03	3.6	3.19E-04	2.5	7.15E-06	2.0	4.94E-03	1.5	1.96E-02	2.2	4.94E-03	2.0	3.32E-03	0.3	1.0E+00	0.2	1.0E+00	0.3	7.1E-01	0.3	1.0E+00	0.0	9.8E-01	0.0	1.0E+00	0.0	9.8E-01	0.1	9.5E-01
8474	THC2534012	A_24_P653054	THC2534012	2.5	3.99E-05	2.0	4.92E-04	3.1	3.99E-05	2.5	8.84E-08	1.5	2.03E-02	1.2	8.33E-02	1.5	2.03E-02	2.0	2.65E-03	0.2	1.0E+00	0.0	1.0E+00	0.2	7.1E-01	0.2	1.0E+00	-0.1	9.4E-01	0.0	1.0E+00	-0.1	8.6E-01	-0.1	9.4E-01
10000	BC068044	A_24_P544661	BC068044	2.5	1.34E-04	2.6	1.34E-04	2.1	5.94E-03	2.5	3.14E-05	2.4	3.53E-04	2.4	3.53E-04	2.3	1.89E-03	2.9	1.58E-05	1.2	1.5E-02	0.9	1.5E-02	1.2	7.0E-04	1.2	6.3E-02	0.1	9.6E-01	0.1	1.0E+00	0.0	9.6E-01	0.2	8.5E-01
4123	RP6-166C19.11	A_24_P332857	NM_173571	2.5	3.99E-05	2.5	1.12E-03	2.3	3.99E-05	2.8	1.31E-08	1.4	7.89E-02	1.0	9.60E-02	1.4	7.89E-02	1.9	1.05E-02	0.1	1.0E+00	0.1	1.0E+00	0.0	9.9E-01	0.1	1.0E+00	-0.2	5.0E-01	0.0	1.0E+00	-0.2	2.4E-01	-0.2	5.0E-01
1707	VWF	A_23_P105562	NM_000552	2.5	4.61E-04	2.3	4.82E-03	3.6	4.61E-04	2.5	1.69E-05	1.9	5.56E-03	1.5	3.39E-02	2.2	5.56E-03	1.9	4.95E-03	0.3	1.0E+00	0.1	1.0E+00	0.3	7.1E-01	0.3	1.0E+00	0.0	9.8E-01	0.0	1.0E+00	0.0	9.8E-01	0.1	9.3E-01
3308	LOC100130938	A_32_P196142	XM_001722558	2.5	2.52E-02	1.6	2.98E-02	2.9	2.52E-02	2.5	2.34E-03	0.5	8.40E-01	0.5	8.40E-01	0.3	8.53E-01	1.1	3.93E-01	-0.1	1.0E+00	-0.2	1.0E+00	0.5	7.2E-01	-0.1	1.0E+00	0.8	1.8E-01	0.0	1.0E+00	0.8	8.7E-02	1.1	1.8E-01
27589	IQCD	A_24_P390060	NM_138451	2.5	1.35E-04	2.5	2.91E-04	2.9	1.35E-04	2.1	2.70E-06	2.2	6.53E-03	2.1	6.53E-03	2.2	1.26E-02	2.3	4.37E-03	0.2	1.0E+00	0.2	1.0E+00	0.0	9.5E-01	0.3	1.0E+00	-0.3	7.8E-01	0.0	1.0E+00	-0.3	6.3E-01	-0.4	7.8E-01
3696	DIRAS1	A_23_P386942	NM_145173	2.5	5.88E-05	2.5	2.79E-03	3.6	5.88E-05	1.8	5.46E-06	2.0	8.84E-03	2.0	8.84E-03	2.6	6.90E-03	2.0	9.04E-03	0.6	4.4E-01	0.5	4.4E-01	0.6	2.9E-01	0.6	8.8E-01	-0.1	9.2E-01	-0.1	1.0E+00	-0.2	6.3E-01	-0.1	9.2E-01
15354	IL11	A_23_P67169	NM_000641	2.5	3.77E-04	2.2	3.77E-04	3.0	7.01E-04	2.5	2.54E-05	1.6	7.71E-03	1.4	4.63E-03	1.9	7.71E-03	1.6	1.62E-02	0.2	1.0E+00	0.1	1.0E+00	0.2	7.3E-01	0.2	1.0E+00	0.1	9.9E-01	0.1	1.0E+00	0.2	8.0E-01	0.0	9.9E-01
11425	SOST	A_23_P118571	NM_025237	2.5	1.80E-05	2.2	3.26E-03	3.1	1.80E-05	2.5	1.94E-07	1.9	8.66E-03	1.3	8.66E-03	2.2	2.02E-02	1.9	2.55E-03	0.0	1.0E+00	0.0	1.0E+00	0.2	7.1E-01	0.0	1.0E+00	0.0	9.3E-01	0.0	1.0E+00	0.1	8.6E-01	-0.1	9.3E-01
8024	TKTL1	A_23_P259901	NM_012253	2.5	1.14E-05	2.5	1.14E-05	2.5	4.78E-04	3.6	2.17E-07	1.8	3.17E-03	1.3	2.21E-02	1.8	3.17E-03	2.3	1.23E-03	0.7	2.7E-01	0.5	4.6E-01	0.7	1.6E-01	0.8	2.7E-01	-0.3	7.8E-01	-0.2	1.0E+00	-0.3	6.1E-01	-0.3	7.8E-01
27286	HIST1H2BC	A_23_P93180	NM_003526	2.5	1.77E-04	2.5	1.77E-04	1.9	3.03E-03	2.6	4.09E-06	2.5	8.88E-04	2.5	8.88E-04	2.3	8.31E-03	2.8	1.16E-04	1.3	1.6E-02	0.9	1.6E-02	1.3	5.4E-04	1.3	2.2E-02	0.0	1.0E+00	0.1	1.0E+00	-0.1	9.3E-01	0.0	1.0E+00
11540	RP6-166C19.11	A_24_P302070	NM_173571	2.6	4.23E-05	2.6	6.27E-04	2.5	4.23E-05	2.9	3.98E-08	1.5	7.46E-02	1.1	1.21E-01	1.5	7.46E-02	2.0	1.48E-02	0.1	1.0E+00	0.2	1.0E+00	-0.2	7.1E-01	0.1	1.0E+00	-0.2	5.9E-01	0.0	1.0E+00	-0.3	1.2E-01	-0.2	5.9E-01
7845	C19orf30	A_23_P353667	AB058893	2.6	2.36E-03	2.8	4.48E-03	2.5	2.36E-03	2.6	1.41E-05	1.5	1.90E-01	1.5	1.90E-01	0.9	4.09E-01	2.1	4.68E-02	0.2	1.0E+00	0.0	1.0E+00	0.5	7.1E-01	0.2	1.0E+00	0.2	6.4E-01	0.0	1.0E+00	0.2	6.4E-01	0.3	6.2E-01
36503	CRLF1	A_23_P56197	NM_004750	2.6	4.26E-03	2.6	4.26E-03	3.2	8.50E-03	2.5	7.57E-04	2.6	1.63E-03	2.3	4.32E-03	2.6	1.63E-03	2.7	9.66E-04	0.0	1.0E+00	0.0	1.0E+00	-0.1	9.1E-01	0.0	1.0E+00	0.1	9.7E-01	0.2	1.0E+00	0.0	9.7E-01	0.1	9.3E-01
8131	ZFP90	A_24_P176404	NM_133458	2.6	6.23E-05	1.9	6.23E-05	2.6	2.22E-04	2.6	5.70E-07	2.1	2.83E-04	1.5	2.94E-03	2.3	2.83E-04	2.1	1.31E-04	0.0	1.0E+00	0.1	1.0E+00	0.0	9.7E-01	0.0	1.0E+00	0.0	9.7E-01	0.0	1.0E+00	-0.3	5.7E-01	0.0	9.7E-01
27859	PCP4L1	A_32_P214665	NM_001102566	2.6	1.46E-04	2.4	1.46E-04	3.1	2.29E-04	2.6	1.33E-06	2.0	2.27E-02	1.6	8.62E-02	2.1	2.27E-02	2.0	1.74E-02	0.1	1.0E+00	0.1	1.0E+00	0.4	7.1E-01	0.0	1.0E+00	0.0	1.0E+00	-0.1	1.0E+00	0.1	8.4E-01	0.0	1.0E+00
9643	MMP1	A_23_P1691	NM_002421	2.6	9.95E-04	2.6	1.35E-02	3.2	9.95E-04	2.2	3.86E-05	1.5	3.61E-02	1.4	5.14E-02	1.5	2.95E-02	1.9	3.61E-02	-0.1	1.0E+00	0.1	1.0E+00	-0.1	7.9E-01	-0.3	1.0E+00	-0.2	7.8E-01	-0.1	1.0E+00	-0.2	7.8E-01	-0.3	7.5E-01
39222	VWF	A_23_P105562	NM_000552	2.6	1.35E-04	2.4	1.37E-03	3.7	1.35E-04	2.6	1.43E-06	2.0	3.01E-03	1.4	3.26E-03	2.2	3.01E-03	2.0	2.11E-03	0.2	1.0E+00	0.2	1.0E+00	0.1	8.7E-01	0.3	1.0E+00	0.0	9.2E-01	0.0	1.0E+00	-0.2	4.6E-01	0.1	9.2E-01
9048	MMP1	A_23_P1691	NM_002421	2.6	1.16E-03	2.6	1.13E-02	3.3	1.16E-03	2.2	6.34E-05	1.5	3.59E-02	1.5	4.33E-02	1.4	3.05E-02	1.9	3.59E-02	-0.2	1.0E+00	0.1	1.0E+00	-0.2	7.6E-01	-0.3	1.0E+00	-0.2	7.9E-01	-0.1	1.0E+00	-0.2	7.7E-01	-0.3	7.9E-01
6316	NEFM	A_24_P264832	NM_005382	2.6	3.17E-04	1.6	1.22E-02	2.8	3.17E-04	2.6	1.14E-06	1.0	1.62E-01	0.1	9.68E-01	1.6	1.48E-01	1.0	1.62E-01	0.1	1.0E+00	0.0	1.0E+00	0.4	7.1E-01	0.1	1.0E+00	0.2	4.3E-01	0.0	1.0E+00	0.2	3.7E-01	0.2	4.3E-01
16631	SELM	A_23_P6413	NM_080430	2.6	7.92E-04	2.2	1.40E-03	3.3	7.92E-04	2.6	1.85E-05	2.4	5.70E-03	1.8	5.70E-03	2.4	6.28E-03	2.4	6.99E-04	0.0	1.0E+00	0.0	1.0E+00	-0.1	8.5E-01	0.1	1.0E+00	0.0	9.7E-01	0.2	1.0E+00	-0.1	7.8E-01	0.0	9.7E-01
38094	LAT2	A_23_P259621	NM_032464	2.6	1.27E-05	2.0	1.27E-05	2.7	1.33E-04	2.6	2.19E-07	2.1	1.32E-04	1.9	6.91E-04	2.3	1.32E-04	2.1	1.16E-04	0.3	1.0E+00	0.1	1.0E+00	0.3	7.1E-01	0.3	1.0E+00	-0.1	8.4E-01	-0.1	1.0E+00	-0.1	8.3E-01	0.1	8.4E-01
31651	TBC1D10C	A_24_P392201	NM_198517	2.6	3.23E-04	2.3	8.92E-04	4.4	3.23E-04	2.6	1.62E-05	2.1	1.05E-02	1.6	8.65E-02	2.9	1.05E-02	2.1	8.64E-03	0.1	1.0E+00	0.1	1.0E+00	0.0	9.8E-01	0.3	1.0E+00	0.0	9.8E-01	0.1	1.0E+00	-0.2	7.0E-01	0.0	9.8E-01
4834	PGA3	A_23_P150547	NM_001079807	2.6	2.59E-04	2.4	5.56E-04	2.9	2.59E-04	2.6	7.01E-07	1.5	6.53E-03	1.4	1.79E-02	1.5	3.50E-03	2.0	6.53E-03	-0.1	1.0E+00	-0.1	1.0E+00	0.1	7.6E-01	-0.1	1.0E+00	0.2	5.1E-01	0.0	1.0E+00	0.2	2.0E-01	0.2	5.1E-01
15638	MMP1	A_23_P1691	NM_002421	2.6	5.35E-04	2.6	3.73E-03	3.5	5.35E-04	2.3	1.74E-05	1.4	5.14E-02	1.4	5.14E-02	1.4	3.07E-02	1.9	5.97E-02	-0.2	1.0E+00	-0.1	1.0E+00	-0.2	7.1E-01	-0.4	1.0E+00	-0.1	8.3E-01	-0.1	1.0E+00	-0.1	8.3E-01	-0.3	7.8E-01

Feature	Gene symbol	Probe	Transcript	72 hour siCASP8AP2								48 hours siCASP8AP2								24 hours siCASP8AP2								10 hours siCASP8AP2							
				Median M	Median q	1 M	1 q	3 M	3 q	6 M	6 q	Median M	Median q	1 M	1 q	3 M	3 q	6 M	6 q	Median M	Median q	1 M	1 q	3 M	3 q	6 M	6 q	Median M	Median q	1 M	1 q	3 M	3 q	6 M	6 q
16539	HIST2H4B	A_23_P436281	NM_001034077	2.6	1.30E-05	2.8	1.30E-05	2.1	5.33E-04	2.6	4.81E-07	2.3	8.74E-04	2.3	8.74E-04	2.2	7.63E-03	2.7	1.65E-04	0.5	1.0E+00	0.5	1.0E+00	0.5	7.1E-01	0.5	1.0E+00	0.0	9.9E-01	0.2	1.0E+00	0.0	9.9E-01	0.0	9.8E-01
36755	C3orf32	A_23_P144005	NM_015931	2.6	1.50E-04	2.1	1.50E-04	3.1	7.22E-04	2.6	6.25E-06	2.5	1.28E-03	1.9	8.83E-03	2.5	1.28E-03	2.7	3.63E-04	0.2	1.0E+00	0.2	1.0E+00	0.1	9.0E-01	0.2	1.0E+00	0.0	9.8E-01	0.1	1.0E+00	-0.1	8.7E-01	0.0	9.8E-01
31623	CDKN1C	A_23_P428129	NM_000076	2.6	9.99E-04	2.3	9.99E-04	2.6	3.36E-03	2.7	3.17E-05	2.1	5.07E-04	1.7	2.28E-03	2.1	1.95E-04	2.2	5.07E-04	0.1	1.0E+00	0.0	1.0E+00	0.2	7.1E-01	0.1	1.0E+00	-0.1	9.5E-01	-0.1	1.0E+00	-0.2	8.0E-01	-0.1	9.5E-01
3752	FOXJ1	A_23_P348636	NM_001454	2.6	3.99E-05	2.6	1.75E-04	3.4	3.99E-05	2.5	2.04E-07	2.1	1.72E-03	2.1	4.29E-03	2.5	1.23E-03	2.1	1.72E-03	0.8	4.4E-02	0.8	8.7E-03	0.6	4.1E-01	0.9	4.4E-02	-0.3	7.2E-01	-0.1	1.0E+00	-0.3	4.6E-01	-0.3	7.2E-01
9013	SERPINB2	A_23_P153185	NM_002575	2.6	3.52E-03	2.1	3.52E-03	2.6	1.30E-02	2.8	1.98E-04	1.6	3.17E-01	0.9	7.21E-01	1.6	3.17E-01	1.9	1.54E-01	0.1	1.0E+00	0.1	1.0E+00	0.7	7.1E-01	0.1	1.0E+00	0.3	7.5E-01	0.2	1.0E+00	0.3	7.4E-01	0.4	7.5E-01
9288	CKM	A_23_P50250	NM_001824	2.6	5.89E-03	2.6	5.89E-03	2.4	2.07E-02	2.8	2.92E-04	2.3	1.97E-03	2.0	1.23E-02	2.3	1.28E-03	2.4	1.97E-03	-0.1	1.0E+00	-0.1	1.0E+00	-0.2	8.3E-01	0.2	1.0E+00	-0.1	9.0E-01	0.1	1.0E+00	-0.2	7.4E-01	-0.1	9.0E-01
25977	LOC387763	A_32_P74409	ENST00000339446	2.6	1.50E-04	2.6	1.50E-04	2.2	6.57E-04	2.8	3.93E-07	2.2	2.65E-03	2.2	1.85E-03	1.6	4.54E-03	2.3	2.65E-03	0.2	1.0E+00	0.2	1.0E+00	0.0	9.8E-01	0.2	1.0E+00	0.0	1.0E+00	0.1	1.0E+00	-0.1	9.3E-01	0.0	1.0E+00
39393	COMP	A_23_P90436	NM_000095	2.6	5.05E-03	2.8	8.18E-03	2.6	5.05E-03	2.3	1.73E-04	1.7	1.04E-02	1.7	1.04E-02	1.5	3.50E-03	2.1	1.74E-02	0.1	1.0E+00	0.1	1.0E+00	-0.1	9.0E-01	0.2	1.0E+00	0.0	1.0E+00	0.3	1.0E+00	-0.1	8.5E-01	0.0	1.0E+00
27380	DNHD2	A_32_P14721	NM_178504	2.7	5.23E-03	2.0	5.23E-03	2.7	1.58E-02	2.8	3.01E-04	1.9	1.79E-03	1.1	3.30E-02	2.2	1.18E-03	1.9	1.79E-03	0.2	1.0E+00	0.2	1.0E+00	0.1	8.9E-01	0.5	1.0E+00	-0.2	9.5E-01	-0.1	1.0E+00	-0.3	7.8E-01	-0.2	9.5E-01
12024	LILRA2	A_23_P142205	NM_006866	2.7	1.89E-04	2.7	1.89E-04	1.7	9.38E-04	2.8	1.59E-07	1.7	7.65E-03	1.7	7.65E-03	1.4	1.33E-01	2.4	2.39E-04	0.0	1.0E+00	0.0	1.0E+00	0.1	7.1E-01	0.0	1.0E+00	0.0	1.0E+00	0.0	1.0E+00	0.0	8.8E-01	0.0	1.0E+00
20565	VWF	A_23_P105562	NM_000552	2.7	2.05E-04	2.5	1.37E-03	3.9	2.05E-04	2.7	3.90E-06	1.8	1.15E-01	1.2	4.31E-01	1.9	1.15E-01	1.8	8.79E-02	0.2	1.0E+00	0.1	1.0E+00	0.2	7.1E-01	0.2	1.0E+00	0.1	8.4E-01	0.1	1.0E+00	0.1	6.4E-01	0.1	8.4E-01
31447	BX118026	A_32_P175125	BX118026	2.7	8.67E-05	2.6	5.26E-04	2.7	8.67E-05	2.7	2.79E-07	2.4	7.29E-04	2.0	8.74E-04	2.4	8.98E-05	2.4	7.29E-04	0.2	1.0E+00	0.2	1.0E+00	0.1	7.1E-01	0.2	1.0E+00	-0.1	9.2E-01	-0.1	1.0E+00	-0.1	7.4E-01	-0.1	9.2E-01
14083	LSDP5	A_23_P39251	NM_001013706	2.7	4.55E-05	2.7	6.94E-04	3.3	4.55E-05	2.4	1.63E-07	2.2	7.29E-04	2.2	1.15E-03	2.2	1.32E-04	2.5	7.29E-04	0.1	1.0E+00	0.0	1.0E+00	0.3	7.1E-01	0.1	1.0E+00	0.1	7.7E-01	0.0	1.0E+00	0.1	4.4E-01	0.1	7.7E-01
33013	MMP1	A_23_P1691	NM_002421	2.7	8.04E-04	2.7	3.77E-03	3.5	8.04E-04	2.2	5.13E-05	1.8	1.40E-02	1.6	1.40E-02	1.8	6.17E-03	2.2	2.25E-02	-0.4	1.0E+00	0.0	1.0E+00	-0.4	7.1E-01	-0.4	1.0E+00	-0.2	9.0E-01	0.0	1.0E+00	-0.2	7.6E-01	-0.2	9.0E-01
19908	MMP1	A_23_P1691	NM_002421	2.7	8.27E-04	2.7	5.55E-03	3.5	8.27E-04	2.5	2.99E-05	1.8	6.91E-03	1.7	6.91E-03	1.8	6.55E-03	2.2	2.54E-02	-0.4	1.0E+00	-0.1	1.0E+00	-0.4	7.1E-01	-0.4	1.0E+00	-0.2	8.8E-01	-0.1	1.0E+00	-0.2	7.7E-01	-0.2	8.8E-01
5826	SRPK3	A_23_P125772	NM_014370	2.7	3.09E-03	2.7	3.09E-03	2.5	8.73E-03	2.8	8.68E-05	1.4	2.20E-01	1.4	2.23E-01	1.2	2.20E-01	2.0	4.63E-02	0.1	1.0E+00	-0.1	1.0E+00	0.5	7.1E-01	0.1	1.0E+00	0.4	6.2E-01	0.2	1.0E+00	0.4	3.4E-01	0.4	6.2E-01
43561	IL4I1	A_23_P502520	NM_172374	2.7	1.58E-04	2.5	1.79E-04	3.7	1.58E-04	2.7	1.22E-06	2.3	5.71E-04	2.0	5.71E-04	2.3	1.08E-03	2.4	1.06E-04	-0.1	1.0E+00	-0.1	1.0E+00	-0.1	8.3E-01	0.1	1.0E+00	-0.1	9.2E-01	0.0	1.0E+00	-0.2	6.5E-01	-0.1	9.2E-01
34737	HIST1H2BN	A_23_P402081	NM_003520	2.7	1.24E-04	2.8	1.24E-04	2.0	6.21E-03	2.7	1.53E-05	2.5	1.37E-03	2.5	1.37E-03	2.4	7.63E-03	3.0	1.83E-04	1.4	3.8E-03	1.2	3.8E-03	1.4	7.2E-04	1.6	8.5E-03	-0.1	9.4E-01	0.0	1.0E+00	-0.1	8.5E-01	-0.1	9.4E-01
19665	EPPK1	A_23_P83388	NM_031308	2.7	5.15E-04	2.6	5.15E-04	3.1	4.99E-03	2.7	1.32E-04	2.5	2.91E-03	3.1	4.29E-03	2.4	2.91E-03	2.5	2.66E-03	0.4	1.0E+00	0.4	1.0E+00	0.1	8.7E-01	0.4	1.0E+00	0.2	9.4E-01	0.2	1.0E+00	0.2	8.8E-01	-0.2	9.4E-01
24308	AK098637	A_24_P358063	AK098637	2.7	2.27E-05	2.4	2.27E-05	3.3	1.38E-04	2.7	4.80E-07	2.5	6.63E-05	2.2	4.08E-04	2.7	6.63E-05	2.5	3.42E-05	0.5	4.7E-01	0.3	4.7E-01	0.5	7.1E-01	0.8	4.4E-02	-0.2	8.2E-01	-0.1	1.0E+00	-0.3	4.3E-01	-0.2	8.2E-01
39646	HIST2H2AC	A_24_P8721	NM_003517	2.7	3.11E-05	2.7	3.11E-05	2.8	4.03E-04	2.7	9.33E-07	2.4	1.32E-04	2.2	5.24E-04	2.4	1.32E-04	2.4	8.23E-05	0.2	1.0E+00	0.2	1.0E+00	0.2	7.9E-01	0.1	1.0E+00	-0.1	9.7E-01	0.0	1.0E+00	-0.1	9.0E-01	-0.1	9.7E-01
9433	NLRP3	A_23_P9883	NM_001079821	2.8	3.10E-05	1.4	3.10E-05	2.8	3.56E-05	2.8	1.07E-07	0.8	1.25E-01	0.6	4.09E-01	0.8	1.25E-01	1.3	1.74E-02	0.2	1.0E+00	-0.1	1.0E+00	0.2	7.3E-01	0.2	1.0E+00	0.0	9.8E-01	0.0	1.0E+00	0.1	6.9E-01	0.0	9.8E-01
22126	OVOL1	A_23_P202810	NM_004561	2.8	2.30E-04	2.8	2.30E-04	2.5	2.52E-04	2.8	1.94E-07	2.4	2.10E-03	2.4	2.65E-03	1.9	2.10E-03	2.6	1.37E-03	0.3	1.0E+00	0.3	1.0E+00	0.0	9.9E-01	0.3	1.0E+00	-0.1	8.3E-01	0.0	1.0E+00	-0.2	4.5E-01	-0.1	8.3E-01
19414	TMEM173	A_23_P61371	NM_198282	2.8	9.67E-05	2.5	9.67E-05	2.8	1.66E-03	3.0	5.68E-06	2.1	1.46E-03	2.1	2.30E-03	2.1	1.46E-03	2.3	6.22E-04	0.2	1.0E+00	0.4	1.0E+00	0.2	7.8E-01	0.2	1.0E+00	0.1	9.1E-01	0.3	1.0E+00	0.1	7.4E-01	0.1	9.1E-01
41648	MYH6	A_23_P37164	NM_002471	2.8	4.97E-05	2.7	3.83E-04	2.9	4.97E-05	2.8	7.60E-08	2.1	2.69E-03	1.6	1.87E-02	2.1	2.69E-03	2.1	1.05E-03	0.0	1.0E+00	0.0	1.0E+00	0.0	9.4E-01	0.0	1.0E+00	0.1	9.3E-01	0.1	1.0E+00	0.1	6.1E-01	0.1	9.3E-01
36161	C3	A_23_P101407	NM_000064	2.8	9.38E-04	2.3	2.42E-03	3.4	9.38E-04	2.8	1.57E-05	1.8	2.71E-03	1.5	2.30E-02	1.8	2.71E-03	2.4	1.11E-03	0.1	1.0E+00	0.2	1.0E+00	-0.1	8.0E-01	0.1	1.0E+00	0.0	9.7E-01	0.1	1.0E+00	-0.1	8.4E-01	0.0	9.7E-01
37026	LYPD5	A_24_P323148	NM_182573	2.8	1.47E-04	1.8	2.34E-04	3.1	1.47E-04	2.8	3.11E-07	2.3	2.32E-03	1.6	7.13E-03	2.5	2.32E-03	2.3	4.55E-04	0.4	1.0E+00	0.4	1.0E+00	0.3	7.1E-01	0.5	1.0E+00	-0.2	6.7E-01	-0.1	1.0E+00	-0.2	6.7E-01	-0.4	6.4E-01
26731	RASD1	A_24_P348006	NM_016084	2.8	1.23E-04	2.8	1.23E-04	2.3	7.41E-04	3.3	3.93E-07	2.1	4.31E-03	2.1	4.31E-03	1.9	2.17E-02	3.2	2.51E-04	0.3	1.0E+00	0.3	1.0E+00	0.0	1.0E+00	0.4	9.7E-01	0.0	9.7E-01	0.2	1.0E+00	0.0	9.7E-01	-0.1	8.2E-01
36262	HIST2H2BE	A_24_P148321	NM_003528	2.8	2.71E-05	3.4	2.71E-05	2.6	9.36E-04	2.8	4.76E-06	3.1	1.37E-03	3.1	1.37E-03	2.8	6.41E-03	3.1	1.83E-04	1.9	2.1E-02	1.7	2.1E-02	1.9	4.0E-03	2.0	6.2E-02	-0.1	9.6E-01	-0.1	1.0E+00	-0.3	7.4E-01	-0.1	9.6E-01
5745	LIM2	A_23_P130435	NM_030657	2.8	6.67E-05	2.5	4.26E-04	4.3	6.67E-05	2.8	7.86E-07	1.9	2.03E-02	1.3	5.14E-02	2.4	2.03E-02	1.9	3.74E-03	-0.1	1.0E+00	-0.1	1.0E+00	0.2	7.1E-01	-0.1	1.0E+00	0.1	9.0E-01	0.1	1.0E+00	0.2	6.5E-01	0.1	9.0E-01
7742	HIST3H2A	A_23_P149301	NM_033445	2.8	3.96E-05	2.9	3.96E-05	2.8	8.23E-04	2.7	3.50E-06	2.4	1.95E-04	2.3	1.55E-03	2.4	1.95E-04	2.7	8.64E-05	0.4	1.0E+00	0.4	1.0E+00	0.4	7.1E-01	0.5	1.0E+00	-0.1	9.5E-01	0.1	1.0E+00	-0.1	9.0E-01	-0.1	9.5E-01
42203	BC012185	A_32_P794894	BC012185	2.8	2.52E-05	3.2	2.52E-05	2.8	3.56E-05	2.8	2.46E-08	3.0	1.69E-04	3.3	1.69E-04	2.9	2.21E-04	3.0	1.32E-04	1.1	2.8E-03	1.0	9.4E-04	1.1	2.8E-03	1.3	3.2E-03	-0.2	9.2E-01	0.0	1.0E+00	-0.2	7.7E-01	-0.2	9.2E-01
20886	MMP1	A_23_P1691	NM_002421	2.9	7.24E-04	2.9	3.44E-03	3.7	7.24E-04	2.7	1.78E-05	1.7	8.66E-03	1.7	8.66E-03	1.7	7.48E-03	2.1	3.57E-02	-0.3	1.0E+00	0.0	1.0E+00	-0.3	7.1E-01	-0.4	1.0E+00	-0.1	8.9E-01	-0.1	1.0E+00	-0.1	8.9E-01	-0.3	7.6E-01
24795	VWF	A_23_P105562	NM_000552	2.9	1.35E-04	2.6	1.02E-03	4.0	1.35E-04	2.9	1.15E-06	2.4	3.75E-03	1.8	1.40E-02	2.5	3.75E-03	2.4	3.22E-03	0.1	1.0E+00	0.1	1.0E+00	0.2	7.1E-01	0.1	1.0E+00	0.1	9.2E-01	0.1	1.0E+00	0.0	9.2E-01	0.1	8.8E-01
24421	VWF	A_23_P105562	NM_000552	2.9	1.35E-04	2.6	9.03E-04	4.1	1.35E-04	2.9	1.56E-06	2.4	2.07E-03	1.9	5.95E-03	2.6	2.07E-03	2.4	1.88E-03	0.2	1.0E+00	0.2	1.0E+00	0.1	9.0E-01	0.2	1.0E+00	0.1	9.0E-01	0.1	1.0E+00	0.0	9.0E-01	0.1	8.9E-01
20008	ATP1B2	A_24_P31275	NM_001678	2.9	3.77E-04	2.7	3.77E-04	2.9	1.10E-03	3.2	3.01E-06	2.7	3.83E-04	1.8	9.23E-03	2.7	3.83E-04	2.9	1.83E-04	0.0	1.0E+00	0.0	1.0E+00	0.1	8.7E-01	-0.1	1.0E+00	0.3	9.5E-01	0.3	1.0E+00	0.3	4.9E-01	0.1	9.5E-01
29705	FAM71F1	A_23_P134204	NM_032599	2.9	3.10E-05	2.8	3.10E-05	3.4	1.72E-04	2.9	7.15E-07	2.2	2.06E-02	1.7	4.96E-02	2.2	2.06E-02	2.2	1.47E-02	0.2	1.0E+00	0.3	1.0E+00	-0.2	7.6E-01	0.2	1.0E+00	0.1	9.7E-01	0.1	1.0E+00	0.0	9.7E-01	0.1	9.3E-01
33526	UCA1	A_32_P200238	NR_015379	2.9	2.77E-05	2.0	2.77E-05	3.1	2.22E-04	2.9	8.07E-07	2.1	1.13E-03	1.4	1.89E-02	2.1	1.13E-03	2.5	4.17E-04	0.1	1.0E+00	0.1	1.0E+00	-0.2	7.7E-01	0.2	1.0E+00	-0.2	9.1E-01	-0.1	1.0E+00	-0.3	7.0E-01	-0.2	9.1E-01
26788	ARC	A_23_P365738	NM_015193	2.9	3.16E-04	2.9	3.16E-04	2.8	4.87E-03	3.1	3.51E-05	2.6	1.37E-03	2.3	1.37E-03	2.6	1.40E-03	3.3	7.96E-05	0.4	1.0E+00	0.3	1.0E+00	0.4	3.3E-01	0.4	1.0E+00	0.1	8.8E-01	0.1	1.0E+00	0.1	8.1E-01	0.2	8.8E-01
43716	FTCD	A_23_P91552	NM_206965	2.9	1.27E-05	2.5	1.27E-05	3.1	1.80E-05	2.9	5.77E-09	2.2	8.00E-04	1.8	1.68E-03	2.2	8.00E-04	2.5	7.31E-05	0.3	5.3E-01	0.3	1.6E-01	0.2	7.1E-01	0.3	5.3E-01	-0.1	8.6E-01	0.2	1.0E+00	-0.1	7.8E-01	-0.2	8.6E-01
25530	SPINK1	A_23_P214079	NM_003122	2.9	4.80E-04	3.0	5.13E-04	2.6	4.80E-04	2.9	1.12E-06	2.3	1.38E-02	1.9	1.38E-02	2.3	2.41E-02	2.3	7.53E-03	0.3	7.1E-01	0.3	8.3E-01	0.3	7.1E-01	0.5	5.3E-01	-0.1	9.7E-01	-0.2	1.0E+00	0.0	9.7E-01	-0.1	9.5E-01
39784	KRTAP2-4	A_32_P24382	BC063625	2.9	6.31E-04	2.3	6.31E-04	3.2	2.84E-03	2.9	4.32E-05	2.4	8.74E-04	1.6	8.74E-04	2.4	9.29E-04	2.4	4.33E-05	0.1	1.0E+00	0.3	1.0E+00	0.1	8.2E-01	0.0	1.0E+00	0.5	8.1E-01	0.5	1.0E+00	0.2	8.1E-01	0.5	7.2E-01
17918	DEFA3	A_23_P31816	NM_005217	2.9	6.60E-03	2.9	6.60E-03	4.3	8.06E-03	2.9	1.38E-03	2.7	4.31E-03	2.4	4.31E-03	2.8	7.82E-03	2.7	7.75E-04	0.0	1.0E+00	-0.1	1.0E+00	0.0	9.8E-01	0.2	1.0E+00	0.3	9.0E-01	0.6	1.0E+00	0.2	8.6E-01	0.3	9.0E-01
36921	ATP10A	A_24_P215765	NM_024490	2.9	1.55E-06	2.9	1.55E-06	3.7	3.56E-05	2.8	6.66E-08	3.4	1.07E-04	3.4	1.44E-04	3.4	1.07E-04	3.2	9.29E-05	0.6	2.5E-01	0.6	2.5E-01	0.4	7.1E-01	0.8	1.1E-01	-0.2	5.6E-01	0.0	1.0E+00	-0.3	1.5E-01	-0.2	5.6E-01
25192	VWF	A_23_P105562	NM_000552	2.9	1.76E-04	2.7	8.92E-04	4.1	1.76E-04	2.9	1.78E-06	2.3	4.37E-03	1.7	1.62E-02	2.5	3.88E-03	2.3	4.37E-03	0.1	1.0E+00	0.1	1.0E+00	0.0	9.9E-01	0.1	1.0E+00	0.0	9.9E-01	0.0	1.0E+00	0.0	9.9E-01	0.0	9.9E-01
18241	VWF	A_23_P105562	NM_000552	2.9	1.38E-04	2.6	1.46E-03	4.1	1.38E-04	2.9	1.25E-06	2.5	3.19E-03	1.9	9.90E-03	2.6	3.19E-03	2.5	2.31E-03	0.2	1.0E+00	0.1	1.0E+00	0.3	3.8E-01	0.2	1.0E+00	0.1	9.0E-01	0.1	1.0E+00	0.1	7.2E-01	0.1	9.0E-01
7596	LGALS7B	A_23_P108062	NM_001042507	3.0	7.83E-03	2.8	7.83E-03	3.4	1.87E-02	3.0	1.29E-03	2.4	1.70E-03	1.7	8.04E-03	2.4	1.70E-03	2.5	4.74E-04	0.0	1.0E+00	0.0	1.0E+00	0.0	8.9E-01	0.2	1.0E+00	0.1	9.9E-01	0.4	1.0E+00	0.1	9.5E-01	0.0	9.9E-01
21862	CLDN5	A_23_P6321	NM_003277	3.0	6.58E-05	3.0	9.83E-04	3.0	6.58E-05	2.8	3.93E-07	1.9	4.30E-02	1.7	1.25E-01	1.9	4.00E-02	2.0	4.30E-02	0.2	1.0E+00	0.2	1.0E+00	0.0	9.0E-01	0.3	1.0E+00	-0.1	7.9E-01	0.5	1.0E+00	-0.1	5.9E-01	-0.1	7.9E-01
21741	TEX14	A_23_P54996	NM_198393	3.0	1.27E-05	3.0	1.27E-05	2.4	3.70E-04	3.4	3.18E-07	2.7	4.26E-04	2.1	2.84E-03	2.7	4.26E-04	3.1	9.03E-05	0.0	1.0E+00	0.0	1.0E+00	-0.1	8.6E-01	0.2	1.0E+00	-0.2	7.4E-01	-0.1	1.0E+00	-0.3	4.5E-01	-0.2	7.4E-01
37697	HIST1H2BD	A_24_P146211	NM_021063	3.1	2.71E-05	3.1	2.71E-05	2.8	7.27E-04	3.1	1.07E-06	2.9	9.20E-04	2.8	9.20E-04	2.9	1.68E-03	3.3	5.40E-05	1.6	2.3E-03	1.3	2.3E-03	1.6	7.0E-04	1.7	8.5E-03	-0.1	9.5E-01	0.0	1.0E+00	-0.3	5.0E-01	-0.1	9.5E-01
13597	KRTAP2-4	A_32_P24376	BC063625	3.1	7.13E-04	2.7	7.13E-04	3.6	3.56E-03	3.1	1.04E-04	2.7	2.77E-04	2.3	2.77E-04	2.7	7.59E-04	2.7	3.03E-05	0.3	1.0E+00	0.3	1.0E+00	0.1	9.2E-01	0.3	1.0E+00	0.2	8.5E-01	0.4	1.0E+00	0.2	7.2E-01	0.2	8.5E-01
41239	LILRB5	A_23_P4773	NM_006840	3.1	3.56E-05	3.1	2.55E-04	3.1	3.56E-05	3.5	8.60E-09	2.8	1.27E-04	2.4	4.08E-04	2.8	1.07E-04	2.9	1.27E-04	0.1	1.0E+00	0.0	1.0E+00	0.1	8.0E-01	0.1	1.0E+00	0.0	9.9E-01	0.1	1.0E+00	0.0	9.8E-01	0.0	9.9E-01
25067	HIST1H2AG	A_24_P414658	NM_021064	3.1	2.00E-05	2.5	4.88E-05	3.1	2.00E-05	3.2	5.29E-09	2.7	5.54E-04	2.7	5.54E-04	2.7	1.84E-02	3.0	1.14E-05	1.4	9.4E-04	1.1	9.4E-04	1.4	6.4E-04	1.7	3.2E-03	-0.1	8.3E-01	-0.1	1.0E+00	-0.1	6.4E-01	-0.2	8.3E-01
41059	ACR	A_23_P40734	NM_001097	3.1	2.39E-06	2.8	2.39E-06	3.5	8.23E-05	3.1	1.44E-06	3.1	4.96E-03	2.4	4.96E-03	3.1	8.33E-03	3.2	6.69E-04	0.2	1.0E+00	0.2	1.0E+00	0.1	9.1E-01	0.2	1.0E+00	-0.1	9.5E-01	0.0	1.0E+00	-0.2	7.3E-01	-0.1	9.5E-01
24140	IL3RA	A_32_P217750	NM_002183	3.1	1.35E-03	2.9	1.35E-03	4.1	2.69E-03	3.1	1.11E-04	2.8	6.35E-04	2.5	6.35E-04	3.1	4.62E-03	2.8	4.78E-04	0.2	1.0E+00	0.2	1.0E+00	0.1	8.9E-01	0.3	1.0E+00	-0.1	9.5E-01	0.2	1.0E+00	-0.1	8.4E-01	-0.1	9.5E-01
14168	GBP5	A_23_P74290	NM_052942	3.2	1.84E-04	2.6	1.84E-04	3.2	1.93E-04	3.2	2.00E-07	2.6	4.40E-04	1.8	8.58E-04	2.6	4.40E-04	2.7	3.67E-04	0.3	1.0E+00	0.2	1.0E+00	0.3	7.1E-01	0.4	1.0E+00	-0.3	7.1E-01	-0.2	1.0E+00	-0.3	5.6E-01	-0.3	7.1E-01
644	LILRA4	A_23_P90497	NM_012276	3.2	2.37E-04	3.2	2.37E-04	2.7	3.31E-03	3.3	7.94E-06	1.6	9.04E-02	1.6	1.12E-01	1.6	9.04E-02	2.4	7.05E-03	0.0	1.0E+00	-0.1	1.0E+00	0.3	7.4E-01	0.0	1.0E+00	0.5	6.6E-02	0.0	1.0E+00	0.5	2.6E-02	0.7	6.6E-02
4031	RRAD	A_23_P88849	NM_004165	3.2	1.49E-03	3.0	1.49E-03	3.6	1.87E-03	3.2	2.92E-05	2.2	7.87E-03	1.6	2.08E-02	2.2	7.87E-03	2.3	3.38E-03	0.3	1.0E+00	0.2	1.0E+00	0.3	7.1E-01	0.3	1.0E+00	0.0	1.0E+00	-0.1	1.0E+00	0.0	9.9E-01	0.0	1.0E+00
41440	TBX21	A_23_P141555	NM_013351	3.2	3.83E-05	3.2	2.81E-04	3.2	3.83E-05	2.9	1.08E-07	2.7	1.87E-03	2.5	2.77E-04	2.9	1.87E-03	2.7	3.29E-03	0.1	1.0E+00	0.1	1.0E+00	0.1	7.3E-01	0.3	1.0E+00	-0.3	5.7E-01	0.0	1.0E+00	-0.3	2.9E-01	-0.3	5.7E-01
13404	NPTX2	A_23_P82651	NM_002523	3.2	1.41E-03	2.7	1.41E-03	3.6	5.17E-03	3.2	1.43E-04	2.3	1.52E-03	2.1	2.72E-03	2.3	2.23E-04	2.6	1.52E-03	-0.1	1.0E+00	-0.1	1.0E+00	-0.3	7.1E-01	-0.1	1.0E+00	0.0	9.7E-01	0.0	1.0E+00	-0.1	9.5E-01	0.1	9.7E-01
26504	THC2754547	A_32_P97780	THC2754547	3.2	2.25E-07	3.2	2.25E-07	3.2	1.79E-05	3.2	1.18E-08	3.2	1.11E-05	3.1	9.98E-05	3.4	1.11E-05	3.2	5.34E-06	0.9	3.2E-03	0.9	9.4E-04	0.9	6.8E-03	1.0	3.2E-03	-0.1	8.8E-01	0.0	1.0E+00	-0.1	6.9E-01	-0.1	8.8E-01
18611	HIST1H2AM	A_32_P221799	NM_003514	3.2	3.60E-05	3.2	1.03E-04	3.5	3.60E-05	3.1	7.60E-08	2.9	2.77E-04	2.8	2.77E-04	2.9	6.55E-05	2.9	1.19E-03	0.0	1.0E+00	0.1	1.0E+00	-0.2	8.8E-01	0.0	1.0E+00	-0.1	9.5E-01	0.0	1.0E+00	-0.1	9.0E-01	-0.1	9.5E-01
21639	GZMH	A_23_P128993	NM_033423	3.2	1.35E-04	3.0	1.61E-04	4.3	1.35E-04	3.2	7.09E-07	2.4	1.42E-02	2.1	3.08E-02	2.8	1.42E-02	2.4	6.43E-03	0.2	1.0E+00	0.2	1.0E+00	0.2	7.1E-01	0.3	1.0E+00	0.1	8.6E-01	0.2	1.0E+00	0.1	8.1E-01	-0.1	8.6E-01
4054	CSF1R	A_23_P110791	NM_005211	3.2	1.40E-03	2.9	1.40E-03	3.4	6.97E-03	3.2	1.42E-04	2.7	6.63E-04	2.3	3.97E-03	2.7	6.63E-04	2.9	3.14E-04	0.3	1.0E+00	0.0	1.0E+00	0.3	7.1E-01	0.4	1.0E+00	0.3	8.9E-01	0.3	1.0E+00	0.1	8.9E-01	0.3	8.9E-01
13756	MC5R	A_23_P50039	NM_005913	3.2	8.63E-04	3.5	8.63E-04	2.1	2.61E-03	3.2	2.09E-06	2.1	2.16E-01	2.1	2.16E-01	1.2	6.15E-01	3.0	2.45E-02	0.5	1.0E+00	0.1	1.0E+00	0.8	7.1E-01	0.5	1.0E+00	0.3	8.1E-01	0.0	1.0E+00	0.5	2.5E-01	0.3	8.1E-01
21119	ART5	A_23_P427122	NM_053017	3.2	4.41E-05	3.0	4.41E-05	3.8	3.18E-04	3.2	2.21E-06	2.6	6.38E-04	2.2	6.38E-04	2.6	7.42E-04	2.8	1.02E-04	0.1	1.0E+00	0.1	1.0E+00	0.2	7.1E-01	0.1	1.0E+00	-0.1	9.3E-01	0.1	1.0E+00	-0.2	7.4E-01	-0.1	9.3E-01
15697	THC2635964	A_24_P732106	THC2635964	3.2	2.58E-06	3.6	2.58E-06	2.7	2.93E-04	3.2	1.51E-07	3.2	6.46E-05	3.2	1.98E-04	2.8	6.46E-05	3.4	2.92E-05	0.1	1.0E+00	0.1	1.0E+00	0.1	7.1E-01	0.3	1.0E+00	0.0	9.8E-01	0.1	1.0E+00	0.0	9.4E-01	0.0	9.8E-01
34784	VCX2	A_24_P230282	NM_016378	3.3	6.04E-05	3.3	6.04E-05	2.9	5.52E-04	3.5	3.84E-07	1.8	3.11E-02	1.2	1.56E-01	1.8	2.85E-02	2.3	3.11E-02	0.0	1.0E+00	0.0	1.0E+00	-0.1	8.8E-01	0.1	1.0E+00	0.0	9.7E-01	0.2	1.0E+00	-0.1	9.0E-01	0.0	9.7E-01
36856	HIST2H2AB	A_24_P68631	NM_175065	3.3	5.73E-05	3.5	5.73E-05	3.3	6.54E-04	3.3	1.82E-06	3.1	2.42E-04	2.9	1.77E-03	3.1	2.42E-04	3.4	1.02E-04	0.6	1.0E+00	0.4	1.0E+00	0.6	7.1E-01	0.7	1.0E+00	-0.2	9.4E-01	0.0	1.0E+00	-0.2	8.7E-01	-0.2	9.4E-01
37197	LGALS7B	A_24_P348118	NM_001042507	3.4	9.54E-03	3.1	9.54E-03	3.9	2.33E-02	3.4	1.91E-03	2.9	9.16E-04	2.1	8.01E-03	2.9	9.16E-04	3.0	3.66E-04	0.1	1.0E+00	-0.1	1.0E+00	0.1	8.0E-01	0.2	1.0E+00	0.1	9.8E-01	0.4	1.0E+00	0.1	9.2E-01	0.1	9.8E-01
4184	COMP	A_24_P264943	NM_000095	3.4	6.23E-03	3.4	8.84E-03	3.4	6.23E-03	2.9	2.49E-04	2.3	2.31E-03	2.3	2.31E-03	2.3	1.40E-03	2.7	7.92E-03	0.1	1.0E+00	0.0	1.0E+00	0.1	8.8E-01	0.2	1.0E+00	0.1	9.9E-01	0.4	1.0E+00	0.0	9.9E-01	0.1	9.7E-01
35559	ACRC	A_23_P171237	NM_052957	3.4	9.44E-06	2.7	9.44E-06	4.0	5.24E-05	3.4	3.90E-07	2.6	4.84E-03	1.5	8.44E-03	2.6	4.84E-03	2.9	4.88E-05	0.0	1.0E+00	0.0	1.0E+00	-0.1	9.4E-01	0.2	1.0E+00	0.0	9.9E-01	0.1	1.0E+00	0.0	9.7E-01	0.0	9.9E-01
35067	HIST1H3H	A_23_P333484	NM_003536	3.4	7.18E-06	3.4	7.18E-06	2.9	4.40E-04	3.4	3.72E-07	3.1	1.44E-04	2.9	1.44E-04	3.1	3.40E-03	3.6	7.37E-06	0.7	7.1E-01	0.6	7.1E-01	0.7	4.5E-01	0.9	9.7E-01	0.0	1.0E+00	0.1	1.0E+00	0.0	9.9E-01	0.0	1.0E+00
4673	DEFB1	A_23_P71480	NM_005218	3.4	3.79E-05	3.4	7.26E-05	4.8	3.79E-05	3.1	2.45E-07	2.3	5.17E-02	1.8	1.56E-01	2.7	4.82E-02	2.3	5.17E-02	0.0	1.0E+00	-0.1	1.0E+00	0.3	7.8E-01	0.0	1.0E+00	0.4	3.6E-01	0.2	1.0E+00	0.4	2.5E-01	0.5	3.6E-01
22722	ACSBG1	A_23_P54488	NM_015162	3.4	7.35E-06	3.4	7.35E-06	3.3	5.07E-05	3.6	5.28E-08	3.6	1.32E-04	3.0	8.74E-04	3.9	1.32E-04	3.6	5.65E-05	0.3	4.2E-01	0.3	4.2E-01	0.2	7.1E-01	0.5	3.4E-01	-0.1	8.8E-01	-0.1	1.0E+00	-0.3	2.9E-01	-0.1	8.8E-01
34337	FRG2	A_24_P623782	NM_001005217	3.5	3.28E-07	3.5	3.28E-07	2.6	1.78E-04	4.1	1.11E-08	2.3	9.02E-03	2.3	9.02E-03	1.9	1.38E-02	3.4	4.74E-04	0.2	1.0E+00	0.1	1.0E+00	0.2	7.4E-01	0.3	1.0E+00	-0.1	9.7E-01	-0.1	1.0E+00	-0.3	3.5E-01	0.0	9.7E-01
33329	FGFR3	A_23_P500501	NM_000142	3.5	5.73E-05	3.5	5.73E-05	3.5	1.83E-03	3.3	1.74E-05	3.5	2.18E-04	3.5	5.38E-04	3.3	2.18E-04	3.7	4.88E-05	0.5	7.3E-01	0.5	4.0E-01	0.2	7.3E-01	0.5	1.0E+00	0.1	9.6E-01	0.1	1.0E+00	0.0	9.6E-01	0.3	7.3E-01
33255	AQP3	A_23_P112481	NM_004925	3.5	7.31E-05	3.5	7.31E-05	3.5	2.34E-03	3.5	1.83E-05	2.9	1.43E-03	2.9	1.43E-03	2.9	2.55E-03	3.4	7.69E-05	0.4	7.9E-01	0.4	5.2E-01	0.1	7.9E-01	0.4	1.0E+00	0.1	9.2E-01	0.1	1.0E+00	-0.1	9.1E-01	0.2	9.2E-01
29301	OSCAR	A_23_P50368	NM_206818	3.5	4.13E-06	2.9	4.13E-06	3.5	1.79E-05	4.0	6.29E-09	2.9	5.55E-04	1.9	2.77E-03	2.9	5.55E-04	2.9	3.95E-04	0.1	1.0E+00	0.1	1.0E+00	0.1	8.2E-01	0.3	1.0E+00	-0.1	9.3E-01	0.0	1.0E+00	-0.2	3.3E-01	-0.1	9.3E-01
42082	ASMT	A_23_P255535	NM_004043	3.5	8.05E-08	3.7	8.05E-08	3.5	1.79E-05	3.4	5.29E-09	3.8	1.19E-05	3.7	9.66E-05	4.2	2.07E-06	3.8	1.19E-05	0.5	1.2E-01	0.4	8.5E-02	0.5	1.2E-01	0.6	1.2E-01	-0.1	9.2E-01	0.1	1.0E+00	-0.1	7.2E-01	-0.1	9.2E-01
36193	OASL	A_23_P139786	NM_003733	3.5	1.80E-05	3.4	1.80E-04	3.8	1.80E-05	3.5	6.17E-08	2.8	2.48E-04	2.3	3.72E-03	2.8	2.48E-04	3.2	1.53E-04	0.1	1.0E+00	0.1	1.0E+00	0.4	6.7E-01	0.1	1.0E+00	-0.1	7.8E-01	-0.1	1.0E+00	-0.1	7.8E-01	-0.2	6.9E-01
38290	A_23_P156609	A_23_P156609	A_23_P156609	3.5	1.43E-05	3.5	2.71E-05	4.4	1.43E-05	3.5	1.31E-08	3.6	1.72E-05	3.3	3.33E-04	3.7	1.72E-05	3.6	1.19E-05	1.2	3.2E-03	1.1	9.4E-04	1.2	4.0E-03	1.5	3.2E-03	-0.3	6.6E-01	0.0	1.0E+00	-0.4	2.3E-01	-0.3	6.6E-01
20510	SLPI	A_23_P91230	NM_003064	3.6	3.43E-03	3.1	3.43E-03	3.9	6.45E-03	3.6	1.79E-04	2.5	2.49E-03	1.9	2.49E-03	2.5	1.72E-03	2.5	2.59E-03	0.1	1.0E+00	0.1	1.0E+00	0.2	7.1E-01	0.1	1.0E+00	0.3	7.3E-01	0.5	1.0E+00	0.2	6.9E-01	0.3	7.3E-01
3477	HIST1H4H	A_23_P323685	NM_003543	3.6	2.52E-05	3.6	2.52E-05	2.5	5.60E-04	3.8	1.51E-07	3.1	4.08E-04	3.0	4.08E-04	3.1	1.71E-02	3.8	1.14E-05	0.8	6.7E-01	0.7	6.7E-01	0.8	4.1E-01	0.8	1.0E+00	0.1	9.5E-01	0.1	1.0E+00	0.1	9.4E-01	0.1	9.5E-01
20688	SLC22A1	A_23_P145569	NM_153187	3.6	6.51E-06	3.1	6.51E-06	4.2	1.80E-05	3.6	1.18E-08	2.6	6.16E-04	2.6	1.43E-03	2.4	6.16E-04	3.2	3.67E-05	0.0	1.0E+00	0.0	1.0E+00	-0.1	7.7E-01	0.0	1.0E+00	-0.1	9.5E-01	-0.2	1.0E+00	0.0	9.1E-01	-0.1	9.5E-01
27057	YPEL4	A_23_P300220	NM_145008	3.6	1.55E-04	3.3	1.55E-04	4.4	4.65E-04	3.6	3.23E-06	3.3	1.58E-03	2.6	2.61E-03	3.3	1.58E-03	3.4	1.37E-04	0.3	7.1E-01	0.3	1.0E+00	0.3	7.1E-01	0.6	6.8E-01	-0.4	8.8E-01	-0.2	1.0E+00	-0.4	6.3E-01	-0.4	8.8E-01
40135	VCY	A_23_P11390	NM_004679	3.7	4.24E-04	3.7	4.24E-04	3.4	8.64E-04	4.0	1.06E-06	2.6	2.24E-03	2.0	4.95E-03	2.6	6.60E-04	3.3	2.24E-03	0.0	1.0E+00	0.0	1.0E+00	-0.1	8.6E-01	0.1	1.0E+00	0.0	1.0E+00	0.2	1.0E+00	-0.1	7.8E-01	0.0	1.0E+00
39115	LGALS7	A_24_P238250	NM_002307	3.7	4.68E-03	3.3	4.68E-03	4.2	1.38E-02	3.7	8.01E-04	2.9	6.40E-04	2.1	6.40E-04	3.0	9.16E-04	2.9	1.83E-04	0.0	1.0E+00	-0.1	1.0E+00	0.0	9.7E-01	0.0	1.0E+00	0.3	9.0E-01	0.5	1.0E+00	0.2	8.4E-01	0.3	9.0E-01
7601	KLK12	A_23_P500010	NM_145894	3.7	1.43E-05	3.5	5.70E-04	4.7	1.43E-05	3.7	7.10E-09	3.0	1.64E-03	2.6	2.61E-03	3.0	1.46E-03	3.0	1.64E-03	0.3	7.2E-01	0.3	1.0E+00	0.2	7.2E-01	0.5	5.2E-01	-0.3	4.8E-01	0.0	1.0E+00	-0.4	1.8E-01	-0.3	4.8E-01
28209	ACR	A_32_P191262	NM_001097	3.8	4.65E-05	3.4	4.65E-05	4.3	4.90E-05	3.8	1.60E-07	3.3	1.47E-02	2.6	1.51E-02	3.4	1.47E-02	3.3	9.30E-04	0.0	1.0E+00	0.0	1.0E+00	0.2	8.7E-01	-0.1	1.0E+00	0.1	8.8E-01	0.0	1.0E+00	0.1	8.7E-01	0.2	8.8E-01
6340	C1S	A_23_P2492	NM_001734	3.8	1.51E-07	2.8	1.51E-07	3.8	1.79E-05	3.8	5.29E-09	3.2	1.04E-03	2.2	3.41E-03	3.3	1.04E-03	3.2	6.67E-05	0.0	1.0E+00	0.0	1.0E+00	-0.1	9.1E-01	0.2	1.0E+00	-0.1	8.8E-01	-0.1	1.0E+00	-0.1	6.2E-01	-0.1	8.8E-01
2876	HIST1H2AD	A_23_P428184	NM_021065	4.0	5.79E-06	3.9	5.79E-06	4.1	2.22E-04	4.0	2.84E-07	3.5	2.64E-05	3.4	1.44E-04	3.5	2.64E-05	3.6	1.95E-05	1.2	8.8E-03	1.0	8.8E-03	1.2	7.1E-03	1.2	1.8E-02	0.1	9.6E-01	0.1	1.0E+00	-0.1	9.0E-01	0.1	9.6E-01
42881	NR1H4	A_23_P25396	NM_005123	4.0	1.51E-07	2.8	1.51E-07	4.0	1.43E-05	4.1	3.71E-09	3.0	8.28E-04	1.7	8.28E-04	3.3	3.82E-03	3.0	7.37E-06	0.1	1.0E+00	0.0	1.0E+00	0.2	7.1E-01	0.1	1.0E+00	-0.1	9.9E-01	-0.1	1.0E+00	-0.1	5.6E-01	0.0	9.9E-01
15038	CTSG	A_23_P140384	NM_001911	4.0	1.86E-05	3.6	1.86E-05	4.9	1.78E-04	4.0	6.46E-07	3.0	6.70E-03	2.4	1.84E-02	3.0	6.70E-03	3.0	1.43E-03	0.4	1.0E+00	0.2	1.0E+00	0.5	6.7E-01	0.4	1.0E+00	0.0	9.6E-01	0.0	1.0E+00	0.1	6.8E-01	0.0	9.6E-01
7963	LILRB3	A_32_P70158	NM_006864	4.0	3.11E-06	3.9	3.11E-06	4.8	1.43E-05	4.0	5.29E-09	3.7	2.23E-04	3.2	5.54E-04	4.3	2.23E-04	3.7	3.03E-05	0.3	9.5E-01	0.4	9.5E-01	0.3	7.1E-01	0.3	1.0E+00	-0.1	9.0E-01	0.0	1.0E+00	-0.1	7.7E-01	-0.1	9.0E-01
17207	DACT3	A_23_P360964	NM_145056	4.0	5.50E-06	3.9	5.50E-06	4.5	6.58E-05	4.0	1.19E-07	3.8	2.83E-04	3.7	5.54E-04	4.1	2.83E-04	3.8	7.88E-05	0.6	1.5E-01	0.5	3.2E-01	0.6	1.5E-01	0.7	1.4E-01	-0.2	8.6E-01	0.0	1.0E+00	-0.2	7.3E-01	-0.2	8.6E-01
29198	HIST2H2AA4	A_23_P309381	NM_001040874	4.1	5.14E-06	4.1	5.14E-06	3.9	2.44E-04	4.1	3.90E-07	3.5	1.44E-04	3.5	1.44E-04	3.5	2.23E-04	3.7	9.60E-06	1.4	1.2E-02	1.2	1.2E-02	1.5	4.0E-03	1.4	2.5E-02	0.3	9.2E-01	0.3	1.0E+00	0.3	8.0E-01	0.2	9.2E-01
29964	GAST	A_23_P159191	NM_000805	4.1	7.57E-04	3.0	7.57E-04	4.9	7.90E-04	4.1	8.34E-06	2.9	7.60E-03	1.9	9.87E-03	3.0	7.60E-03	2.9	7.33E-04	-0.1	1.0E+00	-0.1	1.0E+00	-0.2	7.1E-01	0.0	1.0E+00	0.0	9.8E-01	0.2	1.0E+00	-0.1	9.2E-01	0.0	9.8E-01
14498	RASD1	A_23_P118392	NM_016084	4.1	2.91E-06	4.1	2.91E-06	4.0	7.71E-05	4.9	1.58E-08	3.7	6.24E-05	3.5	9.66E-05	3.7	2.64E-05	4.0	6.24E-05	0.5	3.9E-01	0.4	7.5E-01	0.5	3.9E-01	0.7	3.2E-01	-0.1	8.8E-01	0.0	1.0E+00	-0.1	7.5E-01	-0.1	8.8E-01
16102	GZMB	A_23_P117602	NM_004131	4.2	3.65E-04	3.5	3.65E-04	4.5	2.73E-03	4.2	3.52E-05	3.7	7.17E-05	3.2	3.83E-04	3.7	7.17E-05	3.7	3.03E-05	0.3	1.0E+00	0.1	1.0E+00	0.3	7.1E-01	0.5	1.0E+00	0.3	9.2E-01	0.3	1.0E+00	0.4	5.7E-01	0.2	9.2E-01
23210	TPO	A_24_P257224	NM_000547	4.3	1.79E-05	3.8	5.78E-05	4.9	1.79E-05	4.3	5.49E-09	3.1	3.67E-04	2.5	8.69E-04	3.1	1.32E-04	3.6	3.67E-04	0.1	1.0E+00	0.1	1.0E+00	-0.1	7.7E-01	0.3	1.0E+00	-0.3	3.9E-01	0.0	1.0E+00	-0.3	3.2E-01	-0.4	3.9E-01
38916	VCX2	A_32_P33083	NM_016378	4.4	4.74E-04	4.4	4.74E-04	4.1	1.29E-03	4.9	2.21E-06	3.4	1.24E-03	2.8	2.49E-03	3.4	3.05E-04	3.9	1.24E-03	0.1	1.0E+00	0.1	1.0E+00	-0.1	9.2E-01	0.2	1.0E+00	-0.1	9.6E-01	0.2	1.0E+00	-0.3	6.1E-01	-0.1	9.6E-01
29903	VCX	A_24_P245976	NM_013452	4.4	2.20E-04	4.4	2.20E-04	4.3	9.95E-04	4.9	1.36E-06	3.5	1.02E-03	2.9	1.02E-03	3.5	2.23E-04	4.1	1.12E-03	0.1	1.0E+00	0.1	1.0E+00	-0.2	7.1E-01	0.1	1.0E+00	0.1	9.1E-01	0.3	1.0E+00	-0.2	7.7E-01	0.1	9.1E-01
39382	P2RY5	A_23_P2705	NM_005767	4.5	1.51E-07	3.8	1.51E-07	5.4	1.41E-05	4.5	3.71E-09	4.0	1.72E-05	3.1	7.00E-04	4.0	1.72E-05	4.4	1.19E-05	0.2	7.1E-01	0.2	9.4E-01	0.1	7.1E-01	0.4	6.1E-01	-0.1	9.6E-01	0.1	1.0E+00	-0.1	9.0E-01	-0.1	9.6E-01
7740	FAM90A7	A_24_P515319	NM_001136572	4.5	3.56E-05	4.5	9.07E-05	4.5	3.56E-05	4.9	4.93E-08	3.8	8.06E-05	3.4	7.54E-04	3.8	8.06E-05	4.3	5.45E-05	0.2	1.0E+00	0.1	1.0E+00	0.2	7.1E-01	0.4	1.0E+00	0.0	9.9E-01	0.1	1.0E+00	-0.1	8.8E-01	0.0	9.9E-01
15159	MYOM1	A_23_P96271	NM_003803	4.5	3.14E-07	4.9	3.14E-07	3.9	4.90E-05	4.5	1.11E-08	4.6	6.91E-05	4.6	2.77E-04	4.7	6.91E-05	4.6	2.20E-05	0.7	1.5E-02	0.4	2.3E-01	0.7	1.5E-02	0.8	1.5E-02	-0.3	8.5E-01	-0.2	1.0E+00	-0.3	6.7E-01	-0.3	8.5E-01
16039	FAM90A1	A_24_P643028	NM_018088	4.5	1.79E-05	4.3	2.77E-05	4.5	1.79E-05	4.9	5.29E-09	4.0	1.82E-04	3.4	2.77E-04	4.0	1.82E-04	4.2	5.87E-05	0.1	1.0E+00	0.0	1.0E+00	0.1	8.2E-01	0.3	1.0E+00	0.1	7.8E-01	0.1	1.0E+00	0.1	6.8E-01	0.2	7.8E-01
20117	VCX3A	A_24_P23979	NM_016379	4.6	1.74E-04	4.6	1.74E-04	4.4	1.04E-03	5.2	1.40E-06	3.6	8.74E-04	2.9	8.74E-04	3.6	1.32E-04	4.1	1.34E-03	0.1	1.0E+00	0.1	1.0E+00	-0.1	7.1E-01	0.1	1.0E+00	0.2	9.1E-01	0.4	1.0E+00	-0.1	9.1E-01	0.2	9.0E-01

72 hour — siCASP8AP2

Feature	Gene symbol	Probe	Transcript	Median M	Median q	1 M	1 q	3 M	3 q	6 M	6 q
21112	KRTAP3-1	A_23_P107454	NM_031958	4.6	1.40E-03	3.9	1.40E-03	5.0	5.97E-03	4.6	1.77E-04
43441	FAM90A1	A_23_P151059	NM_018088	4.6	1.79E-05	4.3	2.27E-05	4.6	1.79E-05	4.8	5.29E-09
19659	CSAG1	A_24_P11061	NM_153478	4.7	2.70E-05	4.1	7.91E-05	4.9	2.70E-05	4.7	1.18E-08
8565	HIST1H3F	A_23_P8004	BC062305	4.7	1.51E-07	4.7	1.51E-07	4.7	1.80E-05	4.9	5.29E-09
20839	LILRA3	A_23_P79094	NM_006865	4.8	1.22E-05	4.8	1.22E-05	4.2	1.58E-04	4.9	7.60E-08
11848	DHRS2	A_23_P321501	NM_182908	4.8	1.51E-07	4.1	1.51E-07	4.8	1.79E-05	4.8	5.29E-09
18338	GBP2	A_23_P85693	NM_004120	4.8	9.48E-06	3.5	9.48E-06	5.3	9.04E-05	4.8	1.94E-07
26060	FAM90A7	A_24_P903680	NM_001136572	4.9	9.48E-06	4.9	9.48E-06	4.9	6.79E-05	5.6	1.94E-08
17728	C5orf27	A_32_P128209	AK096140	4.9	1.51E-07	4.0	1.51E-07	5.0	1.79E-05	4.9	5.29E-09
3054	DHRS2	A_23_P48570	NM_182908	4.9	1.05E-06	4.0	1.05E-06	5.3	1.80E-05	4.9	7.10E-09
37038	A_23_P251002	A_23_P251002	A_23_P251002	5.3	2.68E-07	5.3	2.68E-07	4.5	2.80E-05	5.4	5.29E-09
33119	NEFH	A_23_P300600	NM_021076	5.8	2.52E-05	5.8	2.52E-05	4.9	2.22E-04	5.9	8.09E-08
17415	X15675	A_32_P32406	X15675	6.4	4.19E-07	5.7	4.19E-07	6.8	1.43E-05	6.4	5.29E-09

48 hours — siCASP8AP2

Feature	Median M	Median q	1 M	1 q	3 M	3 q	6 M	6 q
21112	3.1	1.58E-03	3.1	1.82E-03	3.1	7.69E-04	3.6	1.58E-03
43441	3.8	7.14E-05	3.3	1.45E-04	3.8	2.75E-05	4.3	7.14E-05
19659	3.6	3.65E-04	2.8	5.71E-04	3.6	1.31E-04	3.9	3.65E-04
8565	4.6	1.02E-04	4.4	1.02E-04	4.6	1.83E-04	4.7	5.34E-06
20839	4.0	3.57E-04	3.7	4.08E-04	4.0	3.57E-04	4.7	3.21E-05
11848	4.0	2.38E-05	3.3	3.70E-04	4.3	2.38E-05	4.0	1.20E-05
18338	3.3	4.21E-03	1.5	9.26E-02	3.7	4.21E-03	3.3	6.68E-04
26060	4.2	7.52E-05	3.7	2.77E-04	4.2	2.32E-05	4.7	7.52E-05
17728	4.2	1.07E-04	3.2	8.88E-04	5.0	1.07E-04	4.2	2.16E-05
3054	3.9	1.72E-05	3.2	5.38E-04	4.3	1.72E-05	3.9	1.14E-05
37038	5.1	3.44E-04	5.1	3.44E-04	5.1	5.36E-03	5.8	1.19E-05
33119	4.8	4.88E-05	4.8	1.44E-04	4.6	1.72E-05	5.8	4.88E-05
17415	5.7	5.34E-06	4.8	9.98E-05	6.1	2.07E-06	5.7	5.34E-06

24 hours — siCASP8AP2

Feature	Median M	Median q	1 M	1 q	3 M	3 q	6 M	6 q
21112	0.1	1.0E+00	0.1	1.0E+00	-0.3	7.9E-01	0.1	1.0E+00
43441	0.1	1.0E+00	0.0	1.0E+00	0.1	7.1E-01	0.3	1.0E+00
19659	0.1	1.0E+00	0.1	1.0E+00	-0.1	7.7E-01	0.1	1.0E+00
8565	1.8	9.4E-04	1.5	9.4E-04	1.8	1.9E-04	1.8	7.7E-03
20839	0.2	9.9E-01	0.5	2.3E-01	0.0	9.9E-01	0.2	1.0E+00
11848	0.3	1.0E+00	0.3	1.0E+00	0.3	7.1E-01	0.4	1.0E+00
18338	0.1	1.0E+00	0.0	1.0E+00	0.2	7.1E-01	0.1	1.0E+00
26060	0.3	1.0E+00	0.1	1.0E+00	0.3	7.1E-01	0.4	1.0E+00
17728	0.1	1.0E+00	-0.1	1.0E+00	0.7	7.1E-01	0.1	1.0E+00
3054	0.3	1.0E+00	0.3	1.0E+00	0.3	7.1E-01	0.3	1.0E+00
37038	2.6	9.4E-04	2.2	9.4E-04	2.7	9.2E-05	2.6	3.2E-03
33119	0.4	4.8E-01	0.4	4.8E-01	0.1	8.4E-01	0.6	4.5E-01
17415	0.7	6.3E-02	0.5	1.0E-01	0.7	1.4E-02	0.8	6.3E-02

10 hours — siCASP8AP2

Feature	Median M	Median q	1 M	1 q	3 M	3 q	6 M	6 q
21112	0.3	8.9E-01	0.4	1.0E+00	0.3	6.4E-01	0.2	8.9E-01
43441	0.0	9.6E-01	0.1	1.0E+00	-0.1	5.8E-01	0.0	9.6E-01
19659	-0.2	7.3E-01	0.0	1.0E+00	-0.3	3.2E-01	-0.2	7.3E-01
8565	0.0	9.9E-01	0.1	1.0E+00	-0.1	9.3E-01	0.0	9.9E-01
20839	0.0	9.9E-01	0.1	1.0E+00	-0.2	3.3E-01	0.0	9.9E-01
11848	0.0	8.8E-01	0.0	1.0E+00	-0.3	4.1E-01	0.1	8.8E-01
18338	0.0	9.6E-01	0.1	1.0E+00	0.0	8.8E-01	0.0	9.6E-01
26060	0.0	9.7E-01	0.1	1.0E+00	0.0	9.7E-01	0.0	9.6E-01
17728	0.3	8.4E-01	0.1	1.0E+00	0.5	1.9E-01	0.3	8.4E-01
3054	0.1	7.6E-01	0.1	1.0E+00	-0.1	7.6E-01	0.3	6.6E-01
37038	-0.3	9.1E-01	0.1	1.0E+00	-0.4	7.1E-01	-0.3	9.1E-01
33119	-0.2	6.0E-01	0.1	1.0E+00	-0.3	3.4E-01	-0.2	6.0E-01
17415	-0.1	1.0E+00	-0.1	1.0E+00	-0.3	5.5E-01	0.0	1.0E+00
