# Supplementary material for: Systems-wide RNAi analysis of CASP8AP2/FLASH shows transcriptional deregulation of the replication-dependent histone genes and extensive effects on the transcriptome of colorectal cancer cells
Source: Mol Cancer. 2012 Jan 4;11:1. doi: 10.1186/1476-4598-11-1 (PMC3281783; doi:10.1186/1476-4598-11-1)
Supplement: Additional file 12 — Table S8. Changes in histone transcript levels at multiple time points following silencing of CASP8AP2/FLASH. [file 1476-4598-11-1-S12.PDF]

Additional file 12, Table S8

				CASP8AP2/FLASH SILENCED SW480 cells								Published data: Hela cells	
				72 hours		48 hours		24 hours		10 hours			
Ref Seq	Gene Symbol	Chromosomal position	Agilent Probe Name	Median Fold change (Log2)	q value	Median Fold change (Log2)	q value	Median Fold change (Log2)	q value	Median Fold change (Log2)	q value	Narita et al	Shepard et al PAS-Seq[#]
NM_001034077	HIST2H4B,<br>HIST2H4A	chr1:148071020-148071079	A_23_P436281	2.63	1.30E-05	2.27	8.74E-04	0.48	n.s	-0.01	n.s		chr1 - 148098952-1
NM_001040874	HIST2H2AA3<br>HIST2H2AA4	chr1:148089252-148089311	A_23_P309381	4.09	5.14E-06	3.54	1.44E-04	1.44	1.17E-02	0.26	n.s	YES[*]	
NM_001040874	HIST2H2AA3<br>HIST2H2AA4[1]	chr1:148089729-148089785	A_23_P103981	3.79	1.51E-07	3.34	3.65E-05	1.37	1.81E-03	-0.02	n.s		
NM_003528	HIST2H2BE	chr1:148123097-148123038	A_24_P148321	2.82	2.71E-05	3.10	1.37E-03	1.90	2.13E-02	-0.13	n.s		chr1-148122634-6
NM_003528	HIST2H2BE	chr1:148123747-148123688	A_23_P149545	1.88	5.02E-04	1.86	3.39E-03	1.01	2.28E-02	-0.05	n.s		chr-148124269-1
NM_003528	HIST2H2BE[2]	chr1:148124713-148124654	A_24_P156911	2.75	1.79E-04	2.47	5.38E-04	1.38	1.41E-02	0.14	n.s		chr1-148124357-1
NM_003517	HIST2H2AC	chr1:148125462-148125521	A_24_P8721	2.75	3.11E-05	2.39	1.32E-04	0.21	n.s	-0.08	n.s		chr1-148125568-4
NM_003517	HIST2H2AC	chr1:148125487-148125546	A_23_P301247	-1.92	5.29E-04	-1.75	1.22E-02	-0.89	n.s	-0.23	n.s		
NM_175065	HIST2H2AB	chr1:148125836-148125777	A_23_P343927	-1.21	1.37E-03	-1.17	4.81E-02	-0.41	n.s	-0.26	n.s		
NM_175065	HIST2H2AB	chr1:148125868-148125809	A_24_P68631	3.32	5.73E-05	3.12	2.42E-04	0.56	n.s	-0.16	n.s		
NM_033445	HIST3H2A	chr1:226711932-226711873	A_23_P149301	2.85	3.96E-05	2.41	1.95E-04	0.42	n.s	-0.10	n.s		
NM_175055	HIST3H2BB	chr1:226712648-226712707	A_23_P332992	3.07	3.58E-05	2.78	5.71E-04	1.56	2.01E-03	0.10	n.s		
NM_003544	HIST1H4B	chr6:26135187-26135128	A_24_P166407	-1.98	3.65E-04	-1.73	n.s	-0.31	n.s	-0.17	n.s		
NM_003537	HIST1H3B	chr6:26139947-26139888	A_24_P174924	1.03	3.08E-03	0.90	8.17E-03	0.17	n.s	0.01	n.s		
NM_003537	HIST1H3B	chr6:26139960-26139901	A_23_P93258	2.18	1.70E-04	1.93	1.54E-04	-0.07	n.s	-0.06	n.s		
NM_003513	HIST1H2AB	chr6:26141499-26141440	A_24_P223384	1.56	9.38E-05	1.92	2.82E-03	0.32	n.s	-0.15	n.s		
NM_021062	HIST1H2BB	chr6:26151544-26151485	A_23_P111054	2.48	2.27E-05	2.41	3.17E-04	1.20	6.08E-03	0.02	n.s		
NM_005319	HIST1H1C	chr6:26164088-26164029	A_23_P122443	3.03	2.05E-06	2.84	1.65E-04	1.26	1.64E-02	-0.17	n.s	YES	
NM_003542	HIST1H4C	chr6:26212447-26212506	A_23_P214487	-1.90	2.71E-05	-1.76	6.11E-03	-0.28	n.s	-0.23	n.s		chr6-26212552-1
NM_003526	HIST1H2BC	chr6:26232021-26231962	A_23_P93180	2.55	1.77E-04	2.50	8.88E-04	1.28	1.62E-02	-0.01	n.s		
BC017379[3]	HIST1H2AC	chr6:26246801-26246860	A_23_P167983	2.75	5.27E-07	2.48	2.77E-04	1.70	9.37E-04	-0.16	n.s.		chr6-26246429-2,<br>ch6-26246577-4<br>ch6-26247162-18<br>chr6-26247313-3<br>ch6-26232895-1
NM_005321	HIST1H1E	chr6:26264907-26264966	A_23_P7976	2.25	8.52E-05	2.22	1.24E-03	0.14	n.s	-0.09	n.s		chr6-26265322-1
NM_021063[4]	HIST1H2BD	chr6:26266453-26266512	A_24_P146211	3.06	2.71E-05	2.87	9.20E-04	1.65	2.29E-03	-0.07	n.s	YES	ch6-26279217-1<br>ch6-26279551-20<br>chr6-26266834-7
NM_003523	HIST1H2BK[5]	chr6:26292068-26292127	A_23_P40470	2.79	1.20E-04	2.76	6.35E-04	1.52	6.50E-03	0.02	n.s	YES	
NM_003523	HIST1H2BE	chr6:26292319-26292378	A_23_P30776	2.90	3.83E-06	2.84	8.06E-05	1.39	9.37E-04	0.01	n.s	YES	
NM_003530	HIST1H3D	chr6:26305256-26305197	A_23_P219045	2.44	8.72E-05	2.47	4.06E-04	0.68	n.s	0.61	n.s		
NM_003530	HIST1H3D	chr6:26305296-26305237	A_24_P217834	2.19	1.46E-04	2.06	5.09E-04	0.60	n.s	-0.08	n.s		
NM_021065	HIST1H2AD[6]	chr6:26307121-26307062	A_23_P428184	3.95	5.79E-06	3.55	2.64E-05	1.18	8.80E-03	0.09	n.s		
NM_003522	HIST1H2BF	chr6:26308082-26308141	A_23_P42178	2.85	3.83E-06	2.83	1.07E-04	1.46	9.37E-04	-0.02	n.s	YES	
NM_003518	HIST1H2BG	chr6:26324755-26324696	A_23_P167997	3.16	7.22E-06	2.84	3.72E-04	1.27	1.42E-03	0.01	n.s		chr6-26324393-2
NM_021052	HIST1H2AE	chr6:26325533-26325592	A_23_P59045	3.98	3.11E-06	3.29	1.76E-04	0.65	n.s	0.01	n.s	YES	chr6-26325683-13
NM_005320	HIST1H1D	chr6:26342737-26342678	A_24_P260639	2.59	1.74E-04	2.36	3.60E-04	0.07	n.s	-0.44	n.s		
NM_003540	HIST1H4F	chr6:26348797-26348856	A_23_P359540	2.19	2.52E-05	2.04	1.17E-03	0.23	n.s	0.06	n.s		
NM_021018	HIST1H3F	chr6:26358626-26358567	A_23_P30799	2.23	2.90E-04	2.14	5.12E-04	0.39	9.99E-01	0.13	n.s		chr6-26358379-1
BC062305[7]	HIST1H3F		A_24_P97914	2.06	2.60E-06	2.06	8.48E-04	0.58	n.s	-0.28	n.s		
BC062305[7]	HIST1H3F	chr6:27886335-27886393	A_23_P8004	4.73	1.51E-07	4.59	1.02E-04	1.78	9.37E-04	-0.02	n.s		
NM_003524	HIST1H2BH	chr6:26360201-26360260	A_23_P366216	2.96	2.39E-06	2.82	3.65E-05	1.56	1.32E-03	0.02	n.s	YES	chr6-26360284-3
NM_003534	HIST1H3G	chr6:26379448-26379389	A_23_P42198	1.27	1.28E-03	1.33	n.s	0.30	n.s	-0.12	n.s		
NM_003525	HIST1H2BI	chr6:26381354-26381413	A_23_P111041	2.95	1.61E-04	2.63	5.38E-04	1.75	2.94E-03	-0.02	n.s	YES	chr6-26381629-2
NM_003543	HIST1H4H	chr6:26393535-26393476	A_23_P323685	3.58	2.52E-05	3.06	4.08E-04	0.82	n.s	0.11	n.s		
BC016677[8]	HIST1H2AG	chr6:27210269-27210328	A_24_P414658	3.11	2.00E-05	2.71	5.54E-04	1.43	9.37E-04	-0.15	n.s		chr6-27209294-1<br>ch6-27210796-2<br>chr6-272111049-7
NM_021064	HIST1H2AG	chr6:27209136-27209195	A_24_P303354	1.38	1.03E-03	1.00	1.34E-02	-0.07	n.s	-0.14	n.s		
NM_003495	HIST1H4I	chr6:27215204-27215263	A_24_P20873	2.17	4.71E-05	1.99	1.33E-03	0.27	n.s	-0.08	n.s		
NM_080596	HIST1H2AH	chr6:27223225-27223284	A_23_P81859	1.59	3.26E-04	0.97	1.12E-02	-0.17	n.s	-0.04	n.s		
NM_003536	HIST1H3H	chr6:27886003-27886062	A_23_P333484	3.40	7.18E-06	3.13	1.44E-04	0.73	n.s	0.02	n.s	YES	
NM_021066	HIST1H2AJ/<br>HIST1H2AI[9]	chr6:27890225-27890166	A_23_P168014	1.70	8.50E-04	1.22	3.60E-02	-0.32	n.s	-0.01	n.s		chr6-27890034-2
NM_021066	HIST1H2AJ/<br>HIST1H2AI	chr6:27890432-27890373	A_24_P394510	1.54	8.50E-04	1.14	6.91E-03	-0.29	n.s	0.08	n.s		
NM_003521	HIST1H2BM	chr6:27890982-27891041	A_24_P3783	2.75	2.78E-04	2.67	2.77E-04	1.61	9.37E-04	-0.01	n.s		
NM_003510	HIST1H2AK	chr6:27913767-27913708	A_24_P217848	1.71	4.54E-04	1.23	2.72E-03	0.04	n.s	-0.10	n.s		
NM_003520	HIST1H2BN[10]	chr6:27914524-27914583	A_23_P402081	2.72	1.24E-04	2.53	1.37E-03	1.43	3.85E-03	-0.11	n.s		chr6-27927954-1
NM_003519	HIST1H2BL	chr6:27883324-27883265	A_23_P8013	3.00	2.47E-06	2.84	3.51E-04	1.52	9.37E-04	0.09	n.s		chr6-27883235-1
NM_003511	HIST1H2AL	chr6:27941481-27941540	A_23_P363174	1.39	3.52E-04	1.22	6.32E-03	0.29	n.s	-0.07	n.s		chr6-27941554-1
NM_005322	HIST1H1B	chr6:27942667-27942608	A_23_P250385	-2.58	4.67E-05	-2.71	3.62E-03	-1.07	n.s	-0.09	n.s		
NM_003533	HIST1H3I	chr6:27947895-27947836	A_24_P9321	-0.44	n.s	-0.62	n.s	0.00	n.s	-0.04	n.s		
NM_003546	HIST1H4L	chr6:27948987-27948928	A_23_P70480	-2.11	2.52E-05	-1.94	7.02E-03	-0.33	n.s	-0.09	n.s		
NM_003514	HIST1H2AM	chr6:27968545-27968486	A_32_P221799	3.19	3.60E-05	2.91	2.77E-04	-0.03	n.s	-0.12	n.s		
NM_003514	HIST1H2AM	chr6:27968615-27968556	A_24_P86389	2.31	1.48E-04	1.92	1.24E-03	0.19	n.s	-0.20	n.s		
NM_003527	HIST1H2BO	chr6:27969567-27969626	A_23_P59069	3.09	1.57E-06	2.90	2.38E-05	1.55	3.12E-03	0.02	n.s		
NM_006026	H1FX	chr3:130517215-130517156	A_23_P96087	0.50	2.64E-02	0.62	n.s	0.13	n.s	-0.22	n.s		
NM_153833	H1FOO	chr3:130752732-130752791	A_23_P372994	0.63	1.95E-03	0.10	n.s	0.03	n.s	0.14	n.s		
NM_138635	H2AFV	chr7:44834057-44833998	A_23_P316487	-0.85	1.54E-02	-0.48	n.s	-0.06	n.s	-0.15	n.s		
NM_002105	H2AFX	chr11:118469860-118469801	A_24_P38895	0.57	2.86E-02	0.35	n.s	0.16	n.s	-0.07	n.s		
NM_177925	H2AFJ	chr12:14818894-14818953	A_24_P236003	1.53	2.10E-04	1.18	1.95E-02	0.12	n.s	-0.13	n.s		
NM_177925	H2AFJ	chr12:14819082-14819141	A_23_P204277	1.13	1.09E-03	0.91	1.99E-02	0.06	n.s	-0.15	n.s		

[1]Both scores of 106 bits, [2]HIST2H2BC - score of 100 bits, [3]BC017379/HIST1H2AC cDNA clone IMAGE:2988620 - includes polyA, [4]Also aligns to NM_138720: HIST1H2BD), transcript variant 2, mRNA includes poly A, [5]HIST1H2BK, HIST1H2BE, HIST2H2BF, score of 106 to 100 bits, [6]HIST1H3D - score of 104 bits, [7]BC062305/HIST1H3F cDNA clone IMAGE:3162462 - includes poly A, [8]BC016677/HIST1H2AG cDNA clone IMAGE 4342191 - includes poly A, [9]Both scores of 110 bits, [10]HIST1H2BL, HIST1H2BK, HIST2H2BF, HIST2H2BF, HIST1H2BI, score of 106 to 100 bits, [*]Annotated as HIST2H2AA, [#]Genome location-read count
