# Supplementary material for: Systems-wide RNAi analysis of CASP8AP2/FLASH shows transcriptional deregulation of the replication-dependent histone genes and extensive effects on the transcriptome of colorectal cancer cells
Source: Mol Cancer. 2012 Jan 4;11:1. doi: 10.1186/1476-4598-11-1 (PMC3281783; doi:10.1186/1476-4598-11-1)
Supplement: Additional file 13 — Figure S5. TNF focused knowledge-based gene networks of genes deregulated as following silencing of CASP8AP2/FLASH. TNF network at (A) 48-hours, and (B) 72-hours generated using Ingenuity pathway analysis tools. A maximum of 70 molecules was used for the generation of networks. Solid edges between gene nodes represent direct interactions and dashed lines represent indirect interactions. Red symbols indicate upregulated genes; Green symbols indicate downregulated genes respectively. The number of molecules in each network is stated, as is a score generated by IPA as an estimate of connectivity. [file 1476-4598-11-1-S13.PDF]

CASP8AP2/FLASH
TNF Network 48 hrs

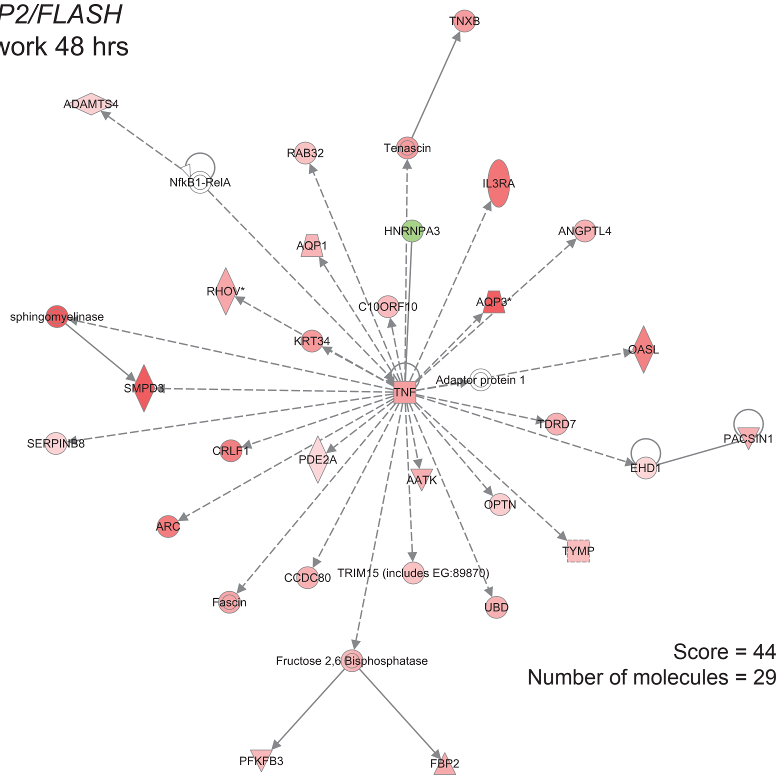

Score = 44
Number of molecules = 29

CASP8AP2/FLASH
TNF Network 72 hrs

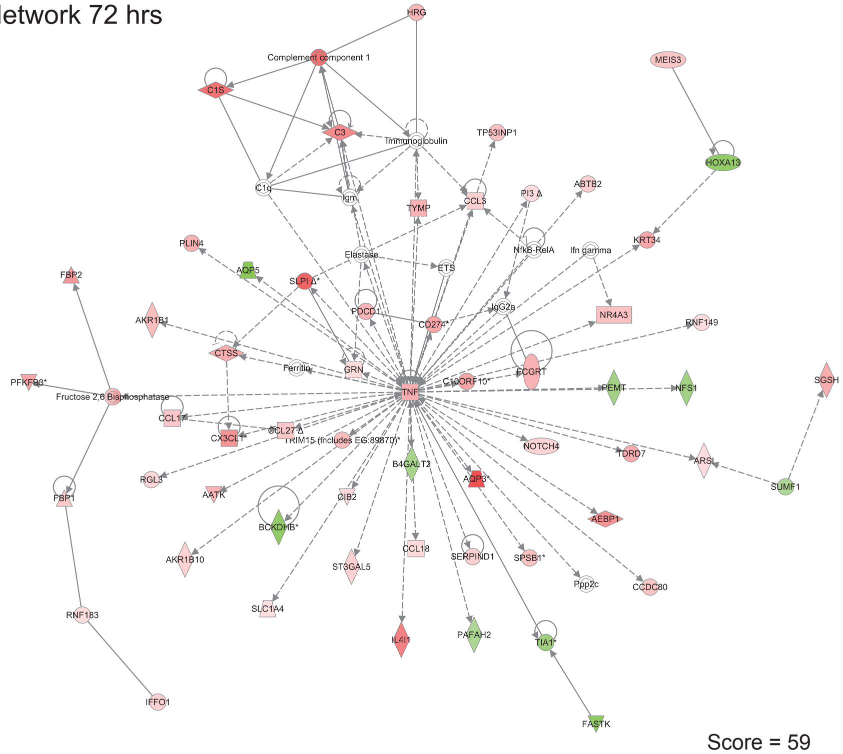

Score = 59
Number of molecules = 58
