# Supplementary material for: Systems-wide RNAi analysis of CASP8AP2/FLASH shows transcriptional deregulation of the replication-dependent histone genes and extensive effects on the transcriptome of colorectal cancer cells
Source: Mol Cancer. 2012 Jan 4;11:1. doi: 10.1186/1476-4598-11-1 (PMC3281783; doi:10.1186/1476-4598-11-1)
Supplement: Additional file 9 — Figure S3. The top three knowledge-based gene networks of genes deregulated as a consequence of silencing CASP8AP2/FLASH were generated using Ingenuity pathway analysis tools. A maximum of 70 molecules was used for the generation of networks. Solid edges between gene nodes represent direct interactions and dashed lines represent indirect interactions. Red symbols indicate upregulated genes; Green symbols indicate downregulated genes respectively. The number of molecules in each network is stated, as is a score generated by IPA as an estimate of connectivity. [file 1476-4598-11-1-S9.PDF]

CASP8AP2/FLASH
Network 1

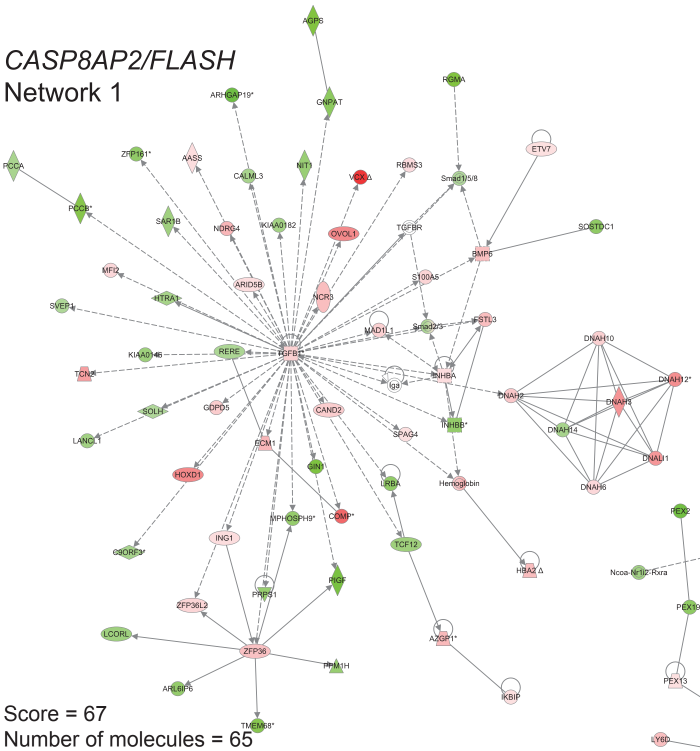

Score = 67
Number of molecules = 65

CASP8AP2/FLASH
Network 2

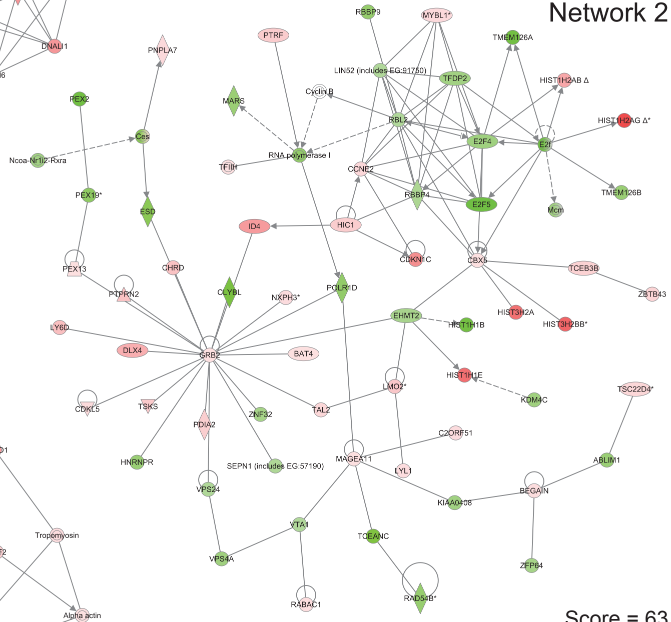

Score = 63
Number of molecules = 63

CASP8AP2/FLASH
Network 3

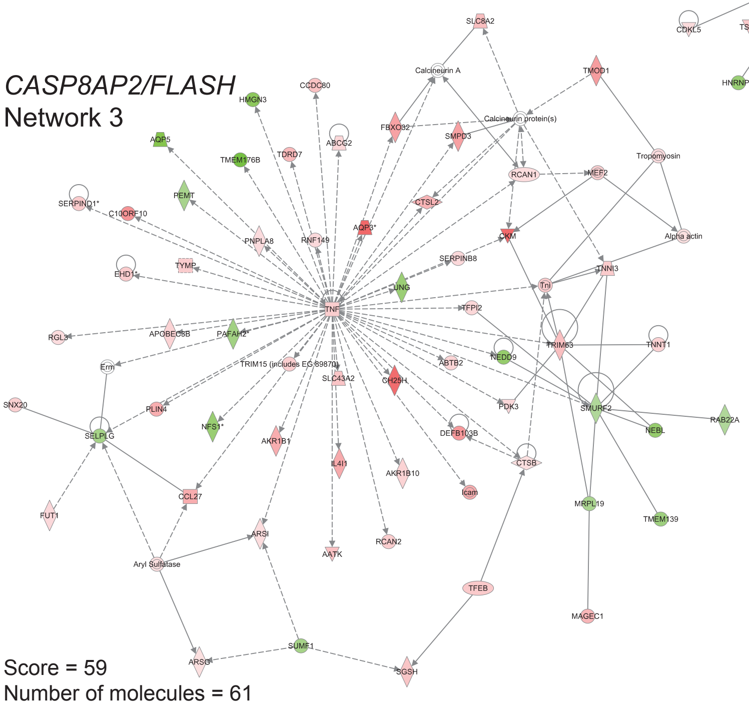

Score = 59
Number of molecules = 61
